# Supplementary figures and images for: The differentiation and integration of the hippocampal dorsoventral axis are controlled by two nuclear receptor genes (part 1 of 6)
Source: eLife. 2023 Sep 26;12:RP86940. doi: 10.7554/eLife.86940 (PMC10522401; doi:10.7554/eLife.86940)

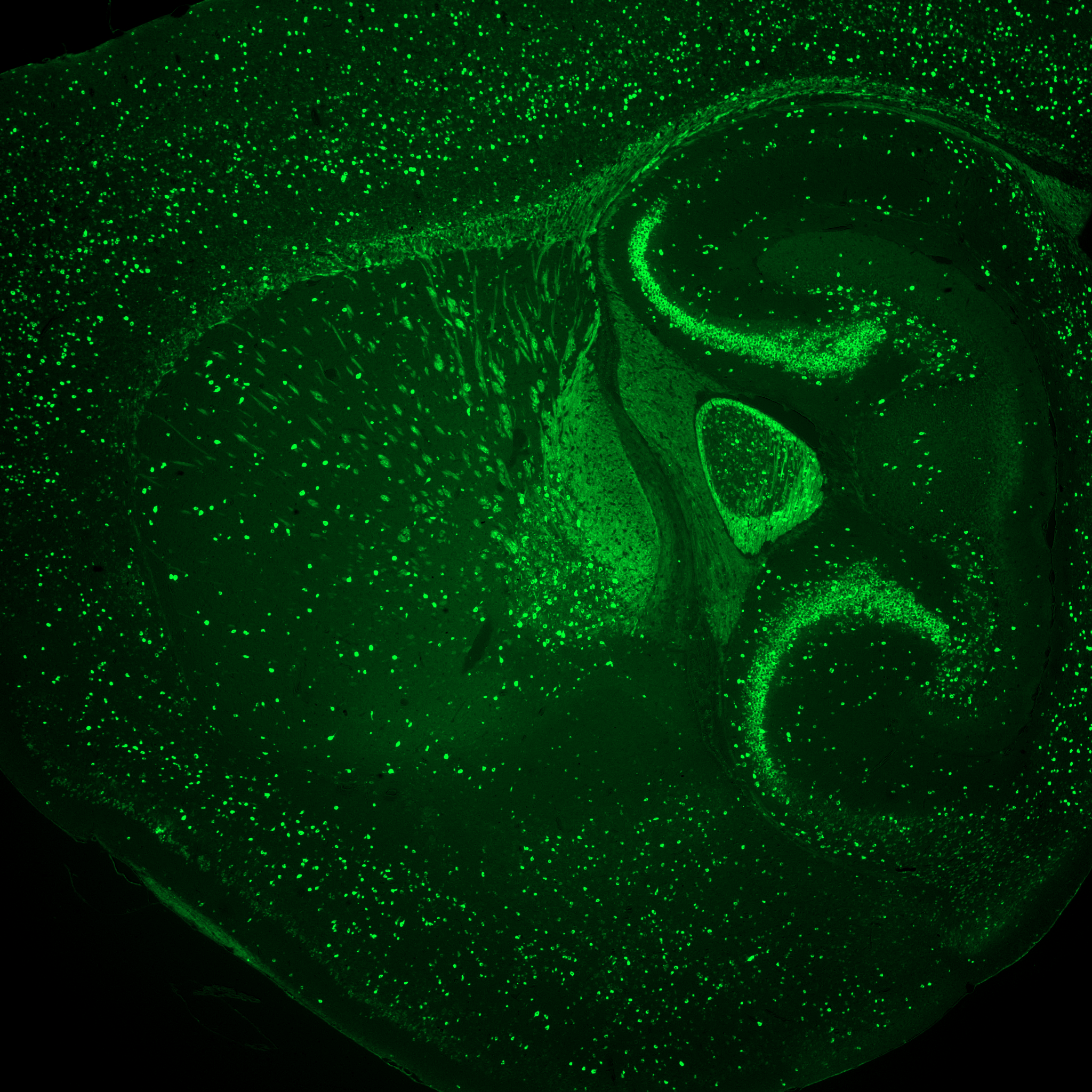

Supplement: Figure 1—source data 1. [file elife-86940-fig1-data1.zip › Figure 1-source data 1/35-CON-CII F+-1M-SAGITAL-HUB-CTIP2-61#-2-2.5X-HPC-Image Export-06_AF488.tif]

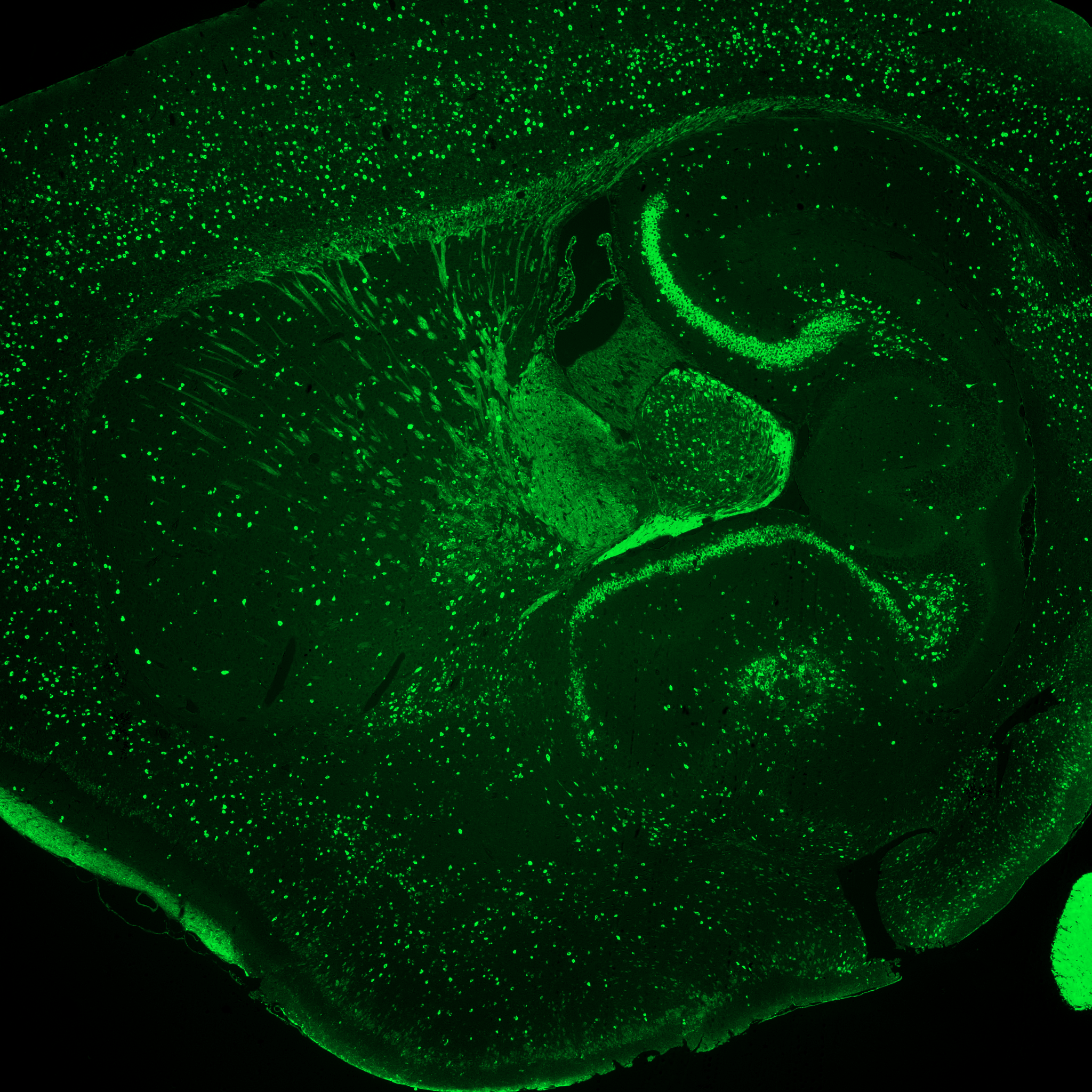

Supplement: Figure 1—source data 1. [file elife-86940-fig1-data1.zip › Figure 1-source data 1/36-CKO-RX CII FF-1M-SAGITAL-HUB-CTIP2-55#-2-2.5X-HPC-Image Export-22_AF488.tif]

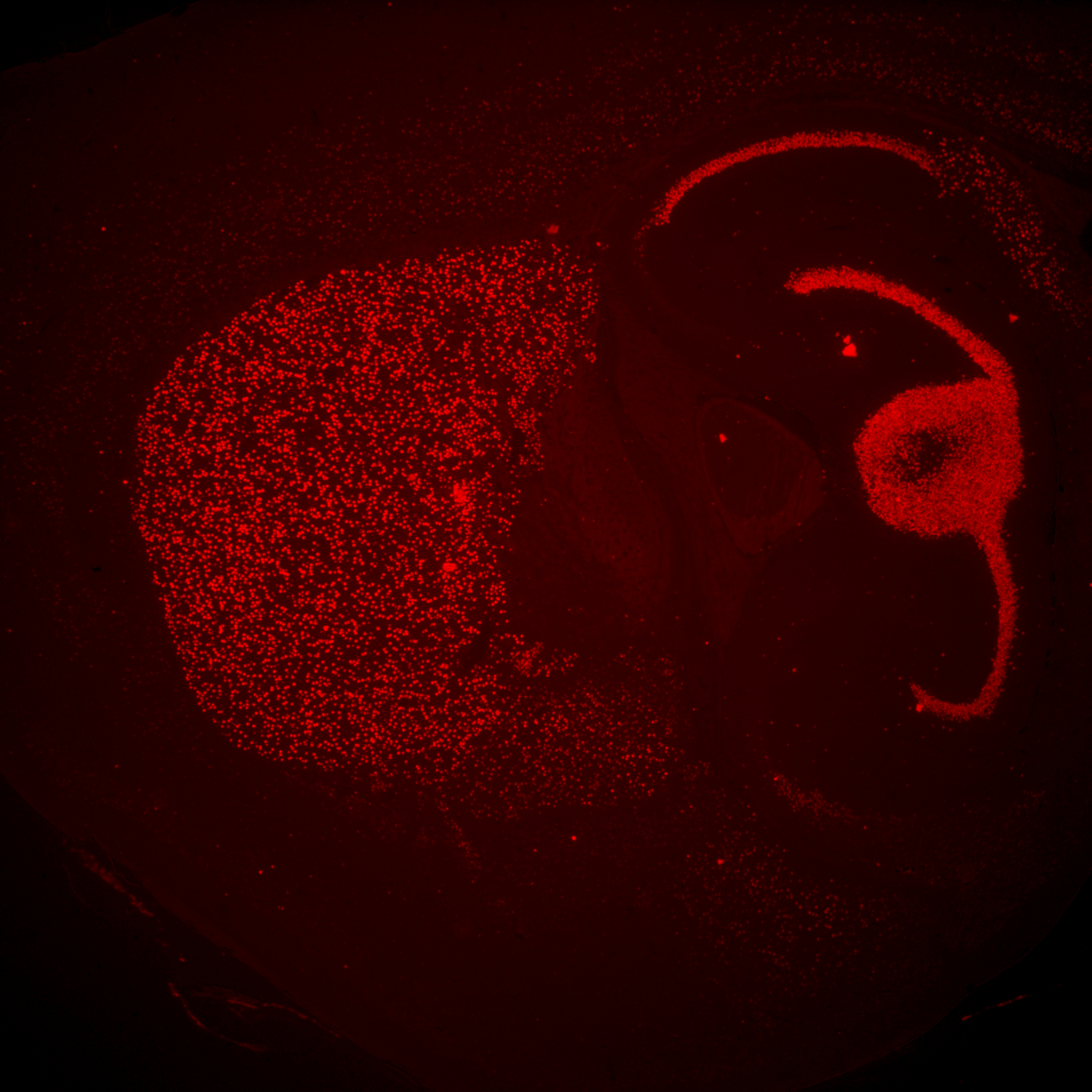

Supplement: Figure 1—source data 1. [file elife-86940-fig1-data1.zip › Figure 1-source data 1/35-CON-CII F+-1M-SAGITAL-HUB-CTIP2-61#-2-2.5X-HPC-Image Export-06_AF594.tif]

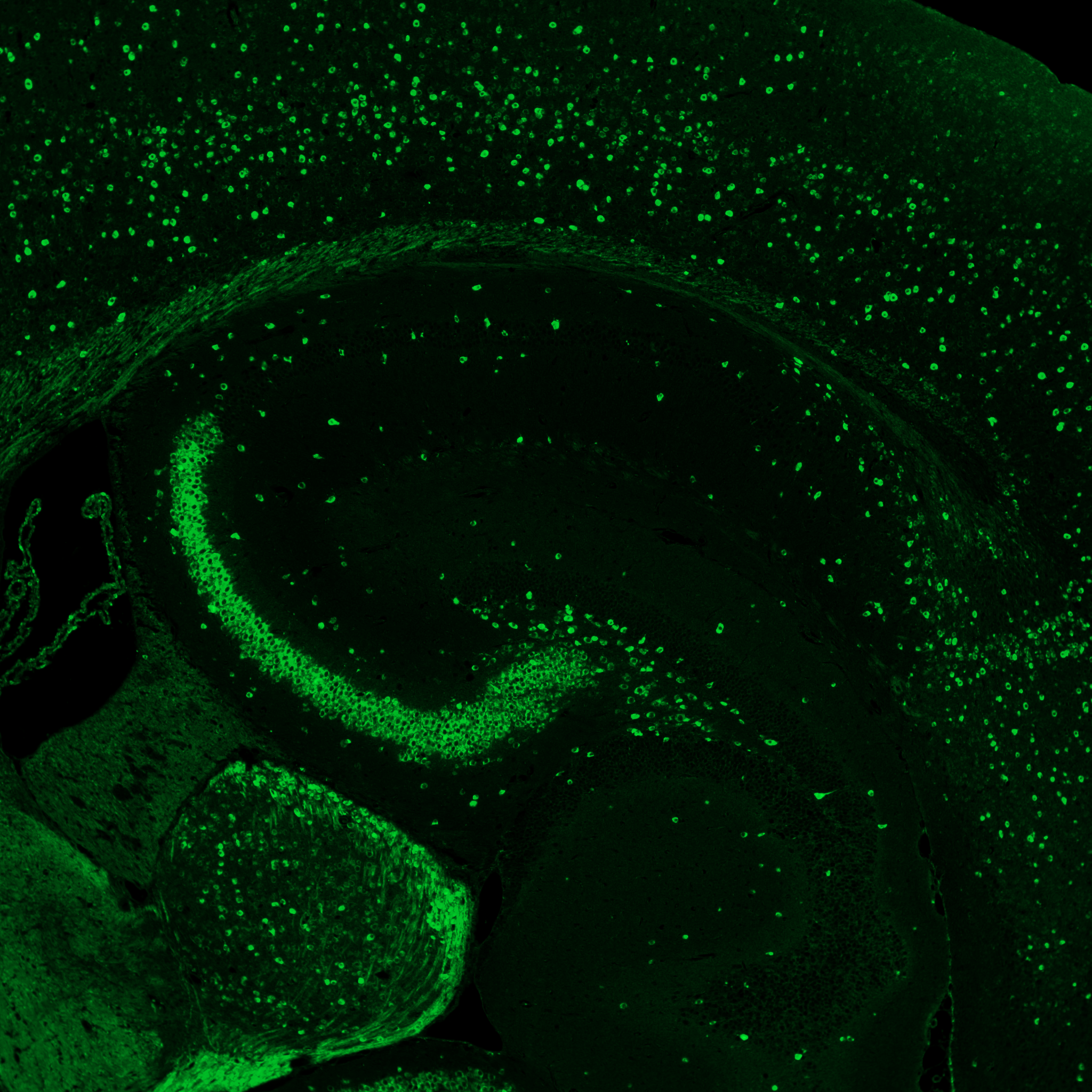

Supplement: Figure 1—source data 1. [file elife-86940-fig1-data1.zip › Figure 1-source data 1/36-CKO-RX CII FF-1M-SAGITAL-HUB-CTIP2-55#-2-5X-dHPC-Image Export-23_AF488.tif]

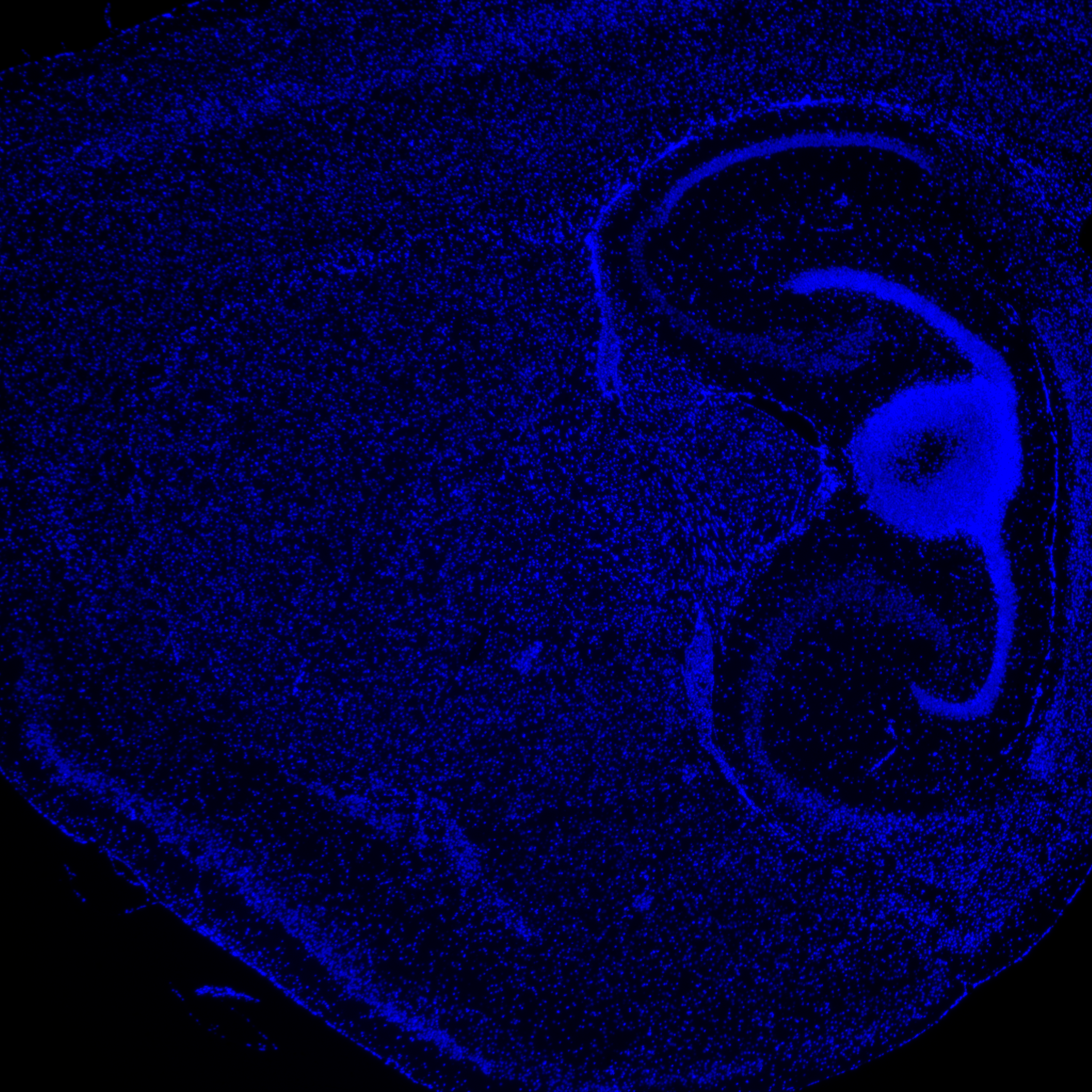

Supplement: Figure 1—source data 1. [file elife-86940-fig1-data1.zip › Figure 1-source data 1/35-CON-CII F+-1M-SAGITAL-HUB-CTIP2-61#-2-2.5X-HPC-Image Export-06_DAPI.tif]

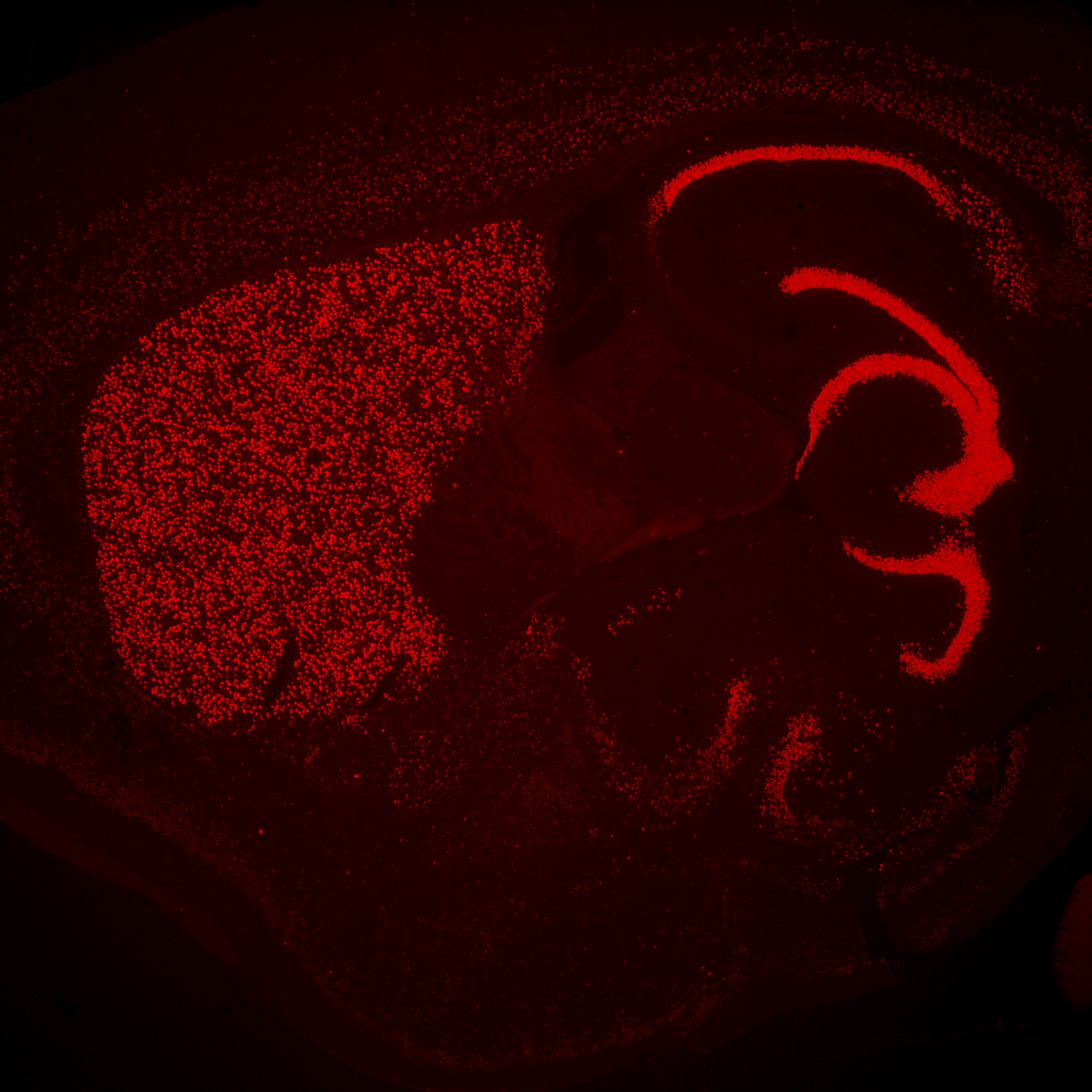

Supplement: Figure 1—source data 1. [file elife-86940-fig1-data1.zip › Figure 1-source data 1/36-CKO-RX CII FF-1M-SAGITAL-HUB-CTIP2-55#-2-2.5X-HPC-Image Export-22_AF594.tif]

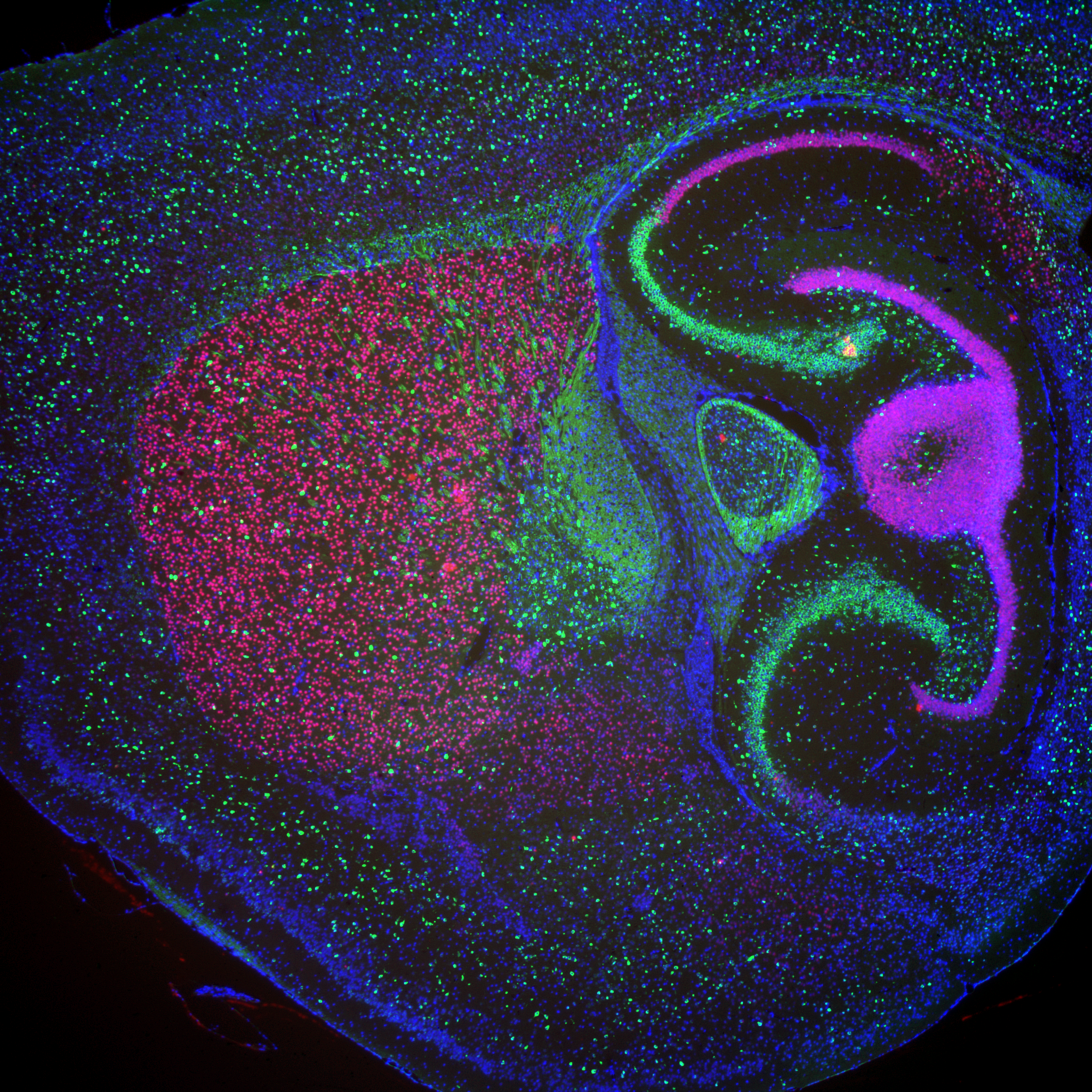

Supplement: Figure 1—source data 1. [file elife-86940-fig1-data1.zip › Figure 1-source data 1/35-CON-CII F+-1M-SAGITAL-HUB-CTIP2-61#-2-2.5X-HPC-Image Export-06.tif]

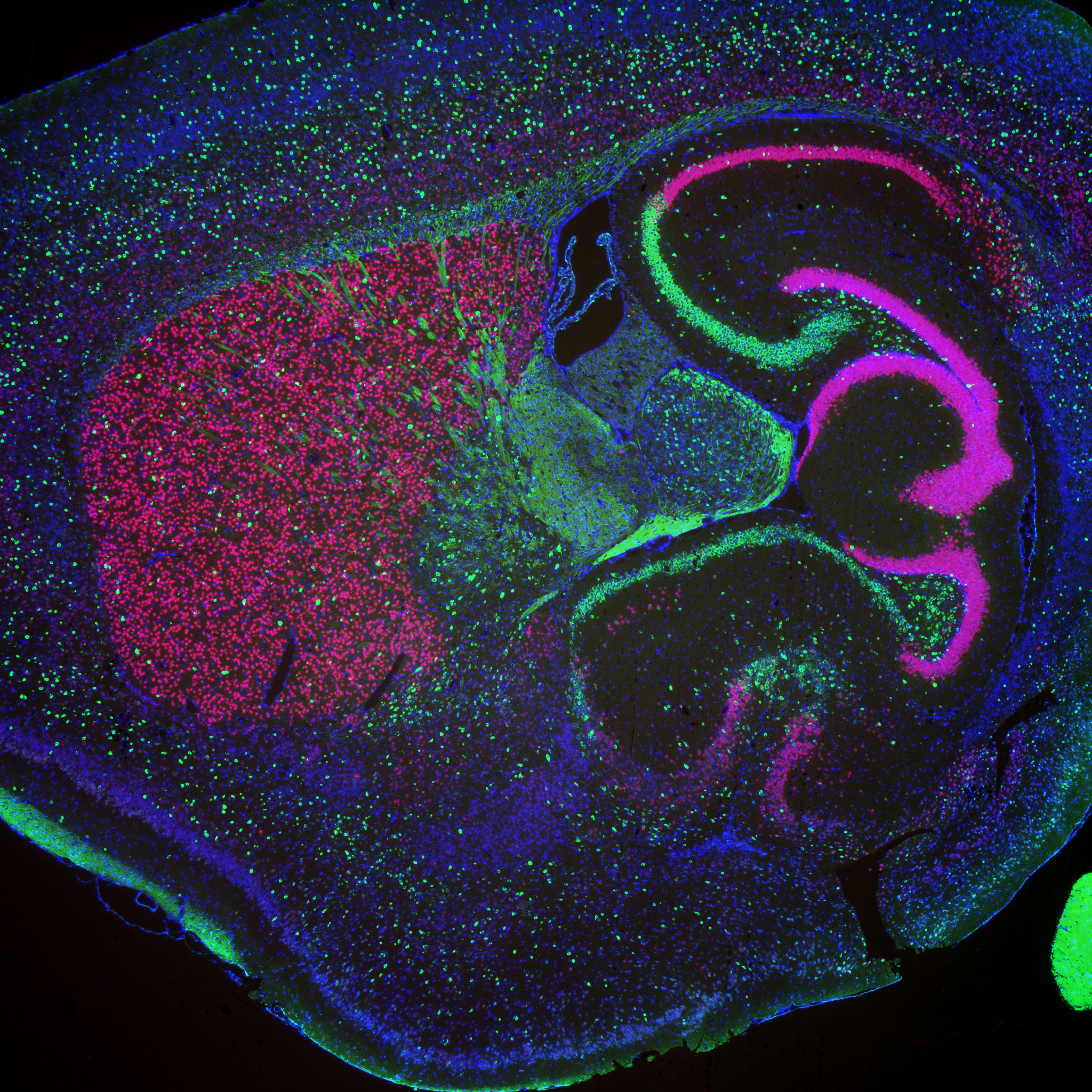

Supplement: Figure 1—source data 1. [file elife-86940-fig1-data1.zip › Figure 1-source data 1/36-CKO-RX CII FF-1M-SAGITAL-HUB-CTIP2-55#-2-2.5X-HPC-Image Export-22.tif]

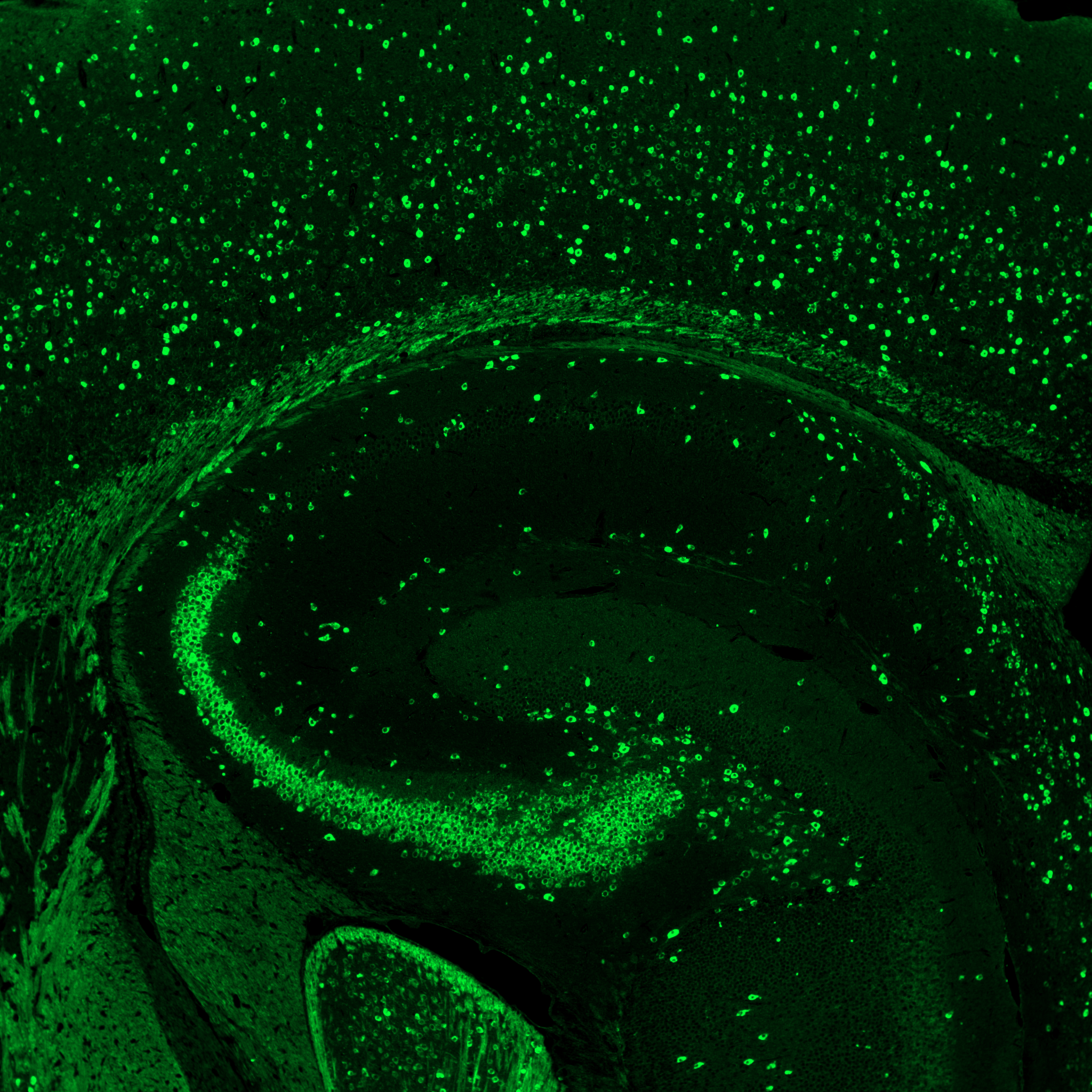

Supplement: Figure 1—source data 1. [file elife-86940-fig1-data1.zip › Figure 1-source data 1/35-CON-CII F+-1M-SAGITAL-HUB-CTIP2-61#-2-5X-dHPC-Image Export-07_AF488.tif]

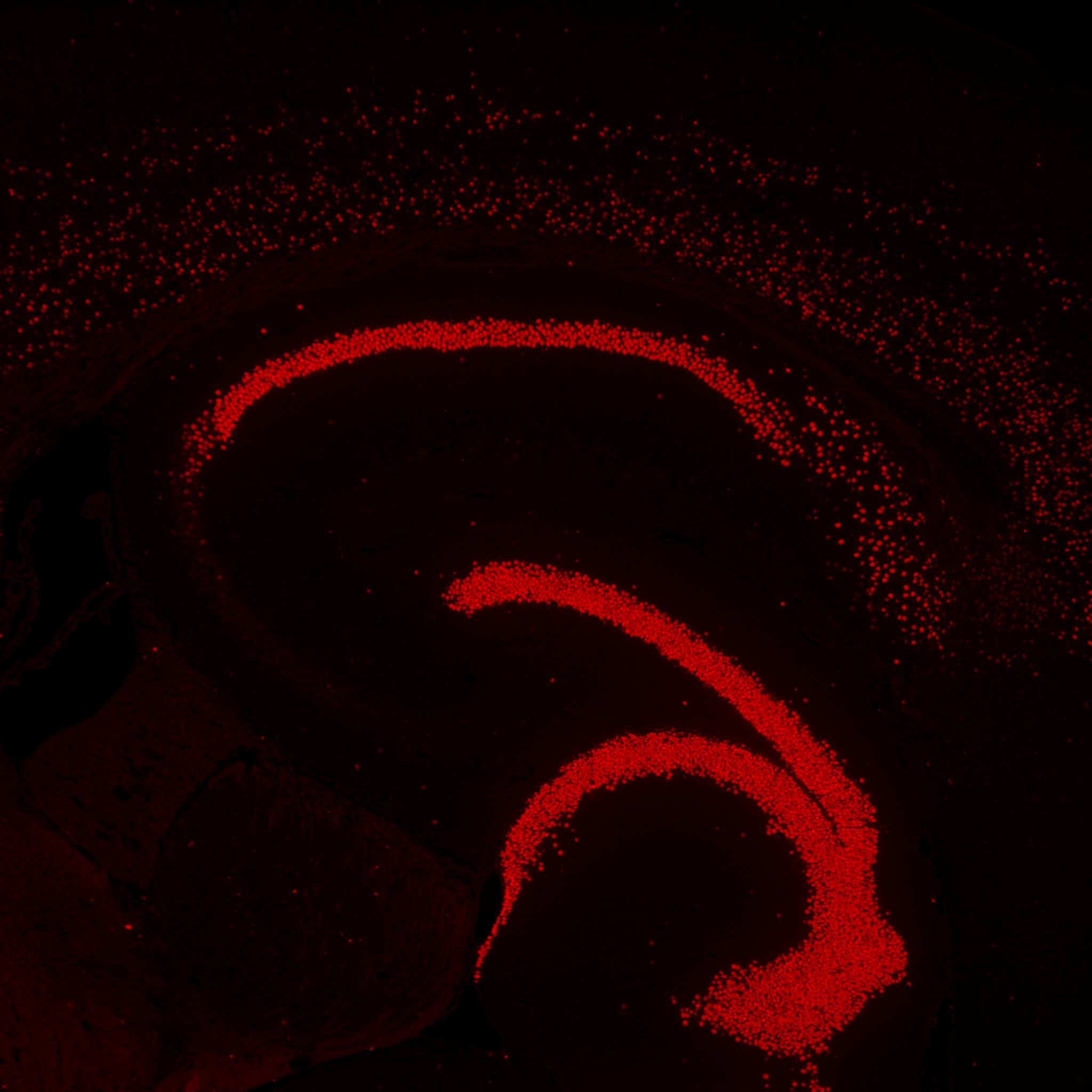

Supplement: Figure 1—source data 1. [file elife-86940-fig1-data1.zip › Figure 1-source data 1/36-CKO-RX CII FF-1M-SAGITAL-HUB-CTIP2-55#-2-5X-dHPC-Image Export-23_AF594.tif]

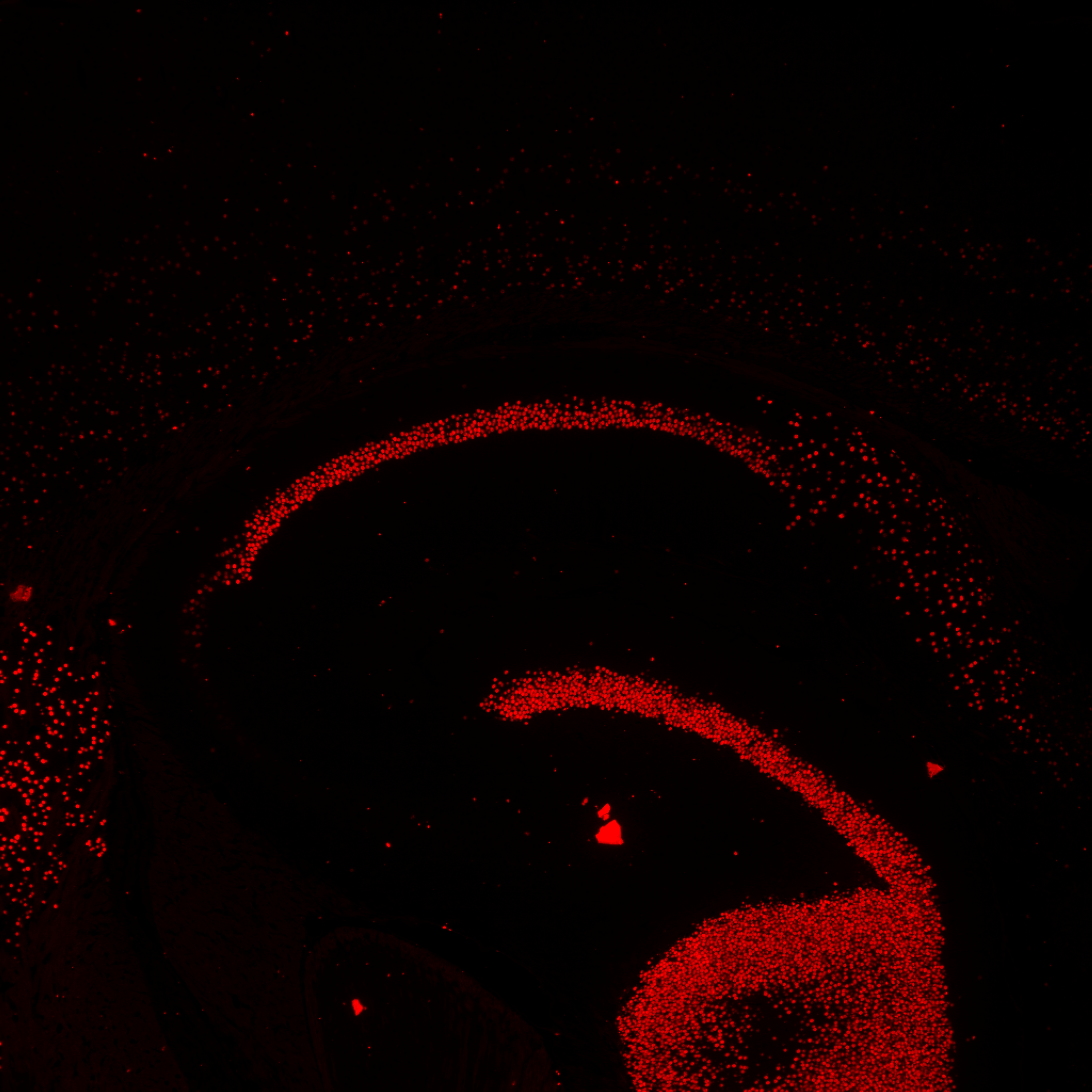

Supplement: Figure 1—source data 1. [file elife-86940-fig1-data1.zip › Figure 1-source data 1/35-CON-CII F+-1M-SAGITAL-HUB-CTIP2-61#-2-5X-dHPC-Image Export-07_AF594.tif]

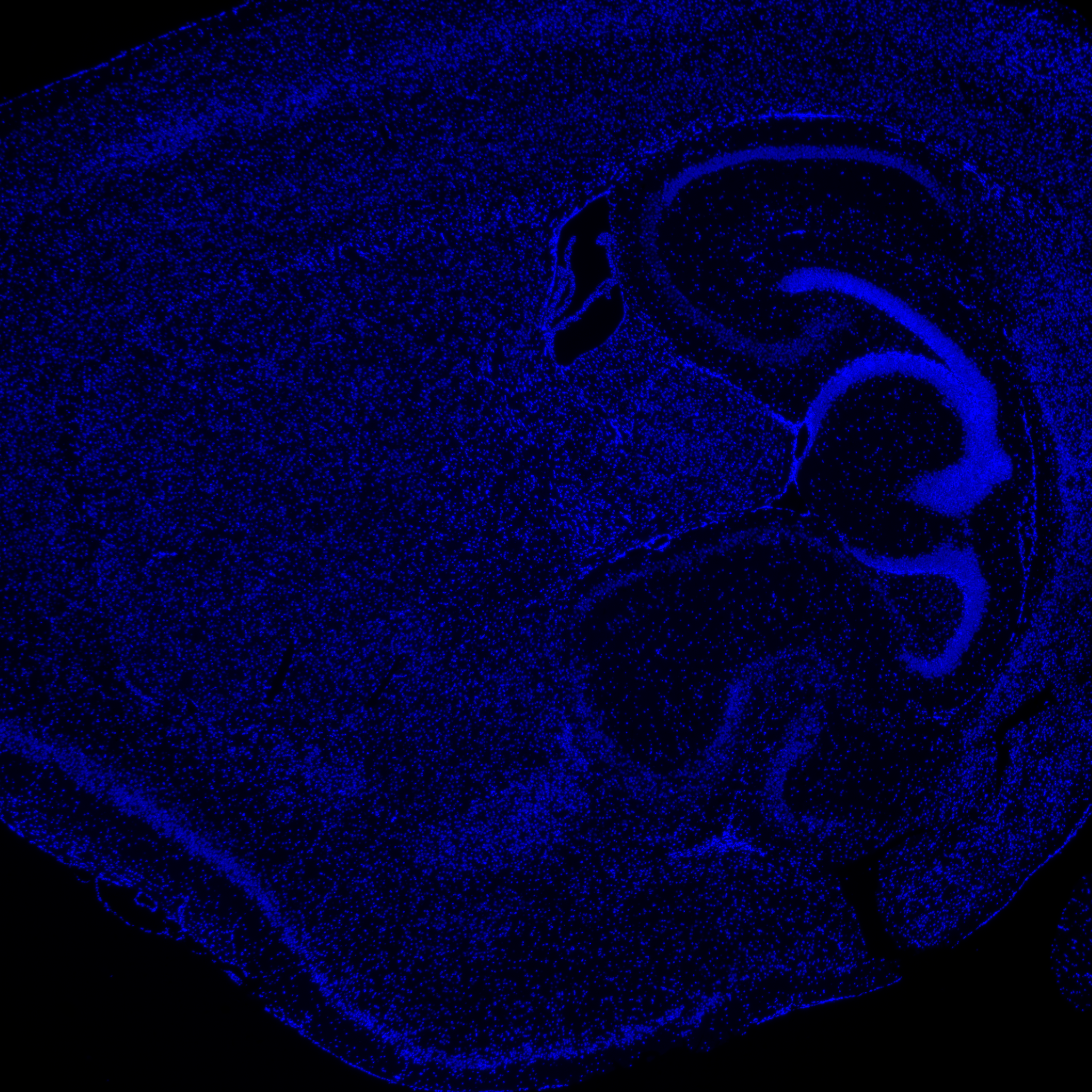

Supplement: Figure 1—source data 1. [file elife-86940-fig1-data1.zip › Figure 1-source data 1/36-CKO-RX CII FF-1M-SAGITAL-HUB-CTIP2-55#-2-2.5X-HPC-Image Export-22_DAPI.tif]

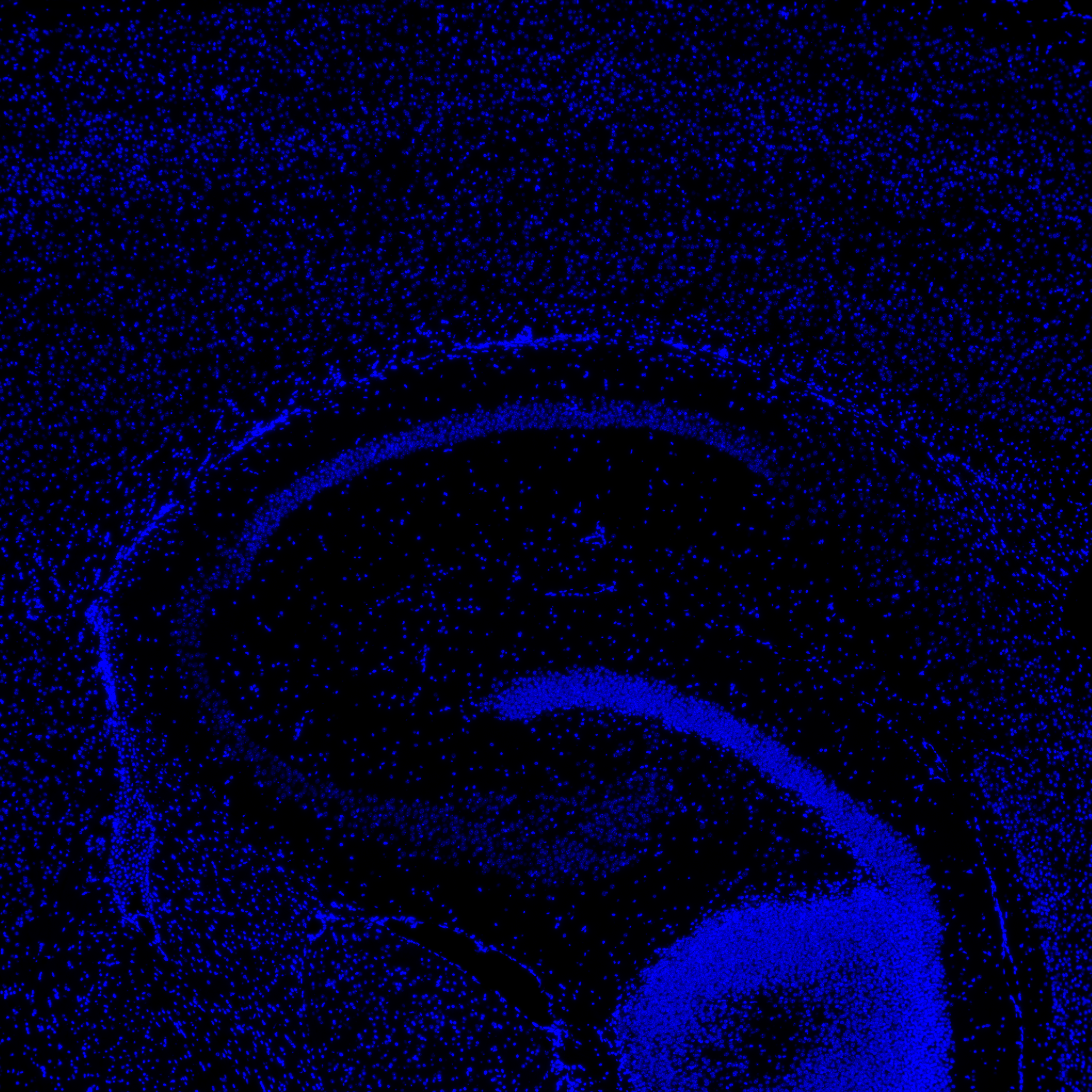

Supplement: Figure 1—source data 1. [file elife-86940-fig1-data1.zip › Figure 1-source data 1/35-CON-CII F+-1M-SAGITAL-HUB-CTIP2-61#-2-5X-dHPC-Image Export-07_DAPI.tif]

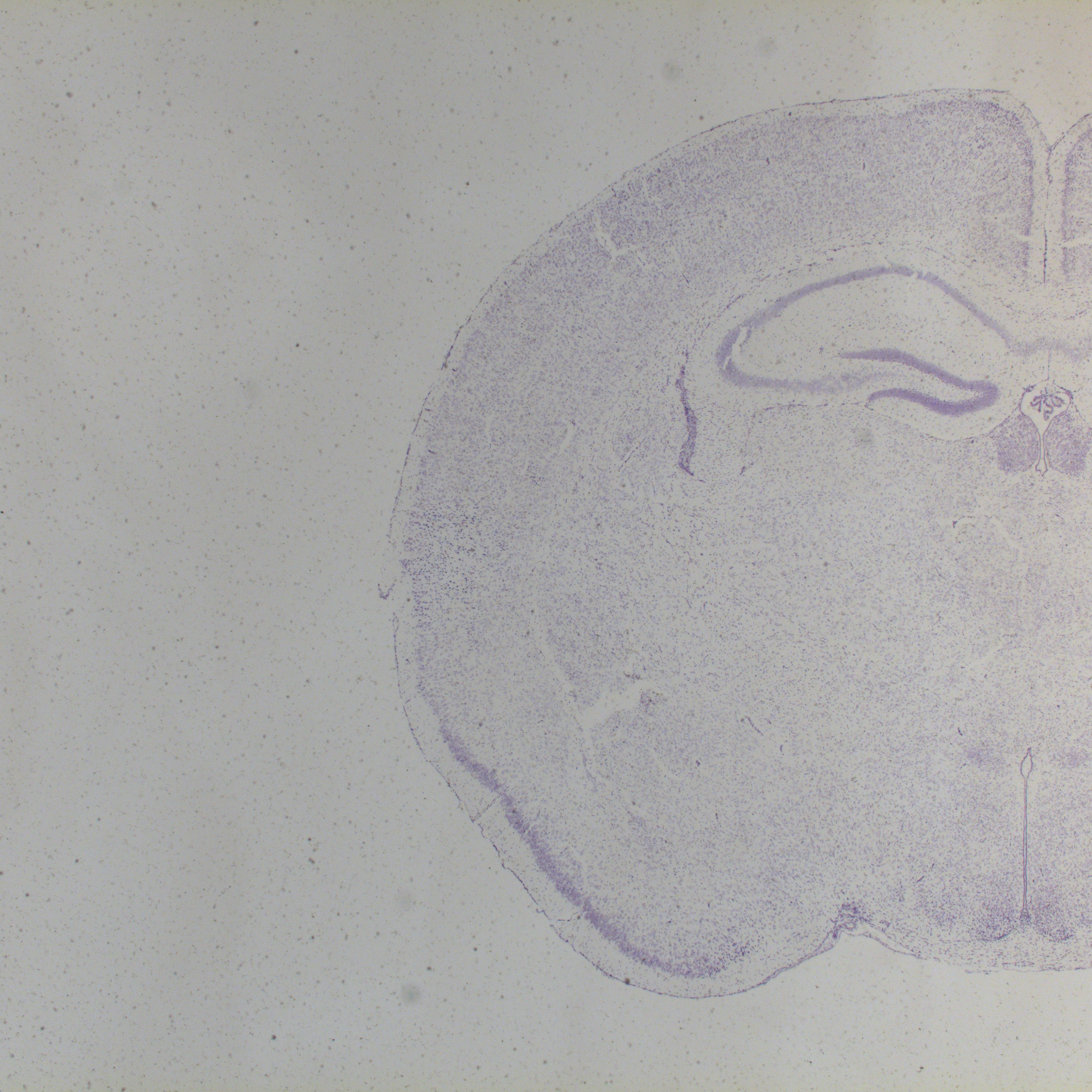

Supplement: Figure 1—source data 1. [file elife-86940-fig1-data1.zip › Figure 1-source data 1/3775-CII CKO-2.5X-RX CII FF-1M-#51-1-Image Export-10.tif]

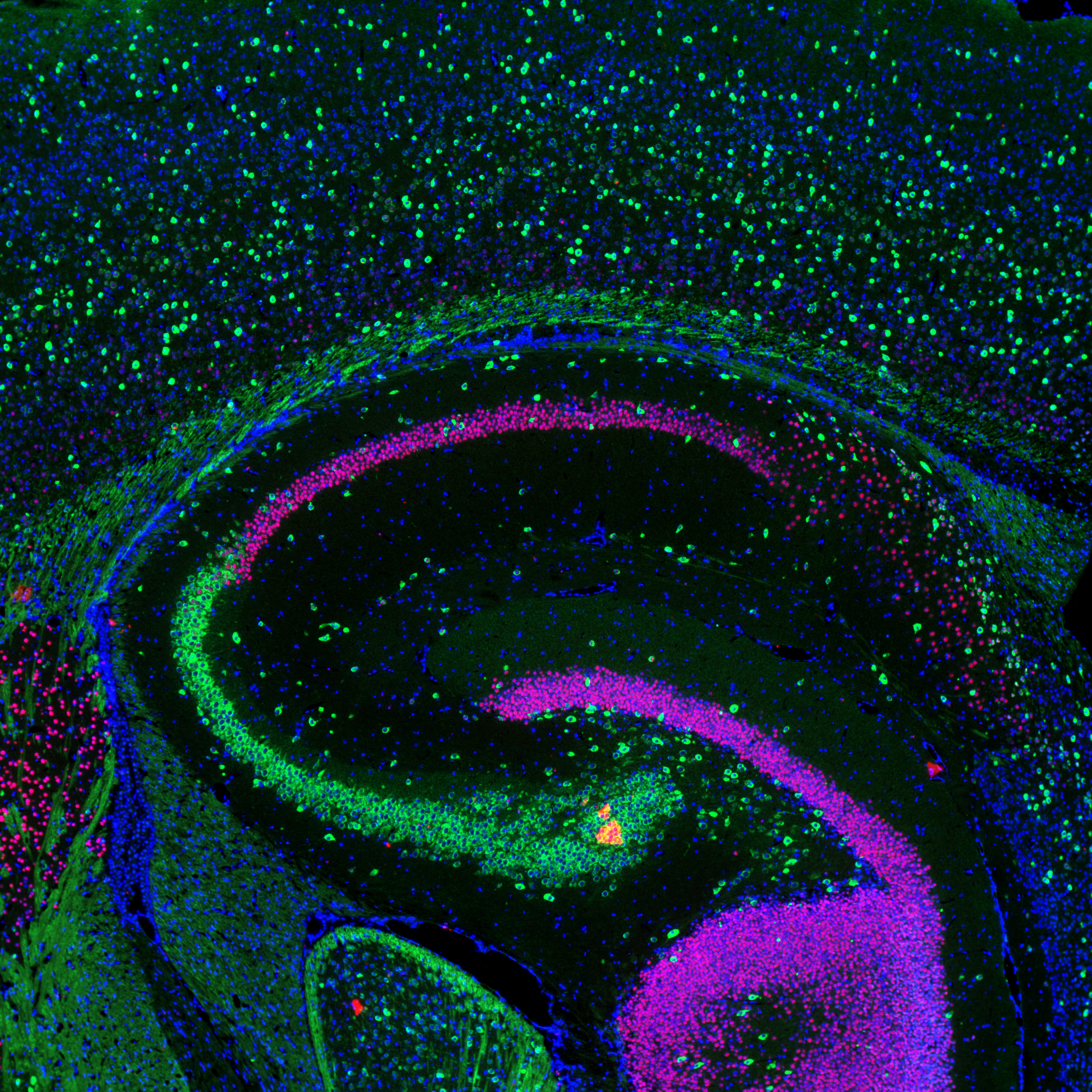

Supplement: Figure 1—source data 1. [file elife-86940-fig1-data1.zip › Figure 1-source data 1/35-CON-CII F+-1M-SAGITAL-HUB-CTIP2-61#-2-5X-dHPC-Image Export-07.tif]

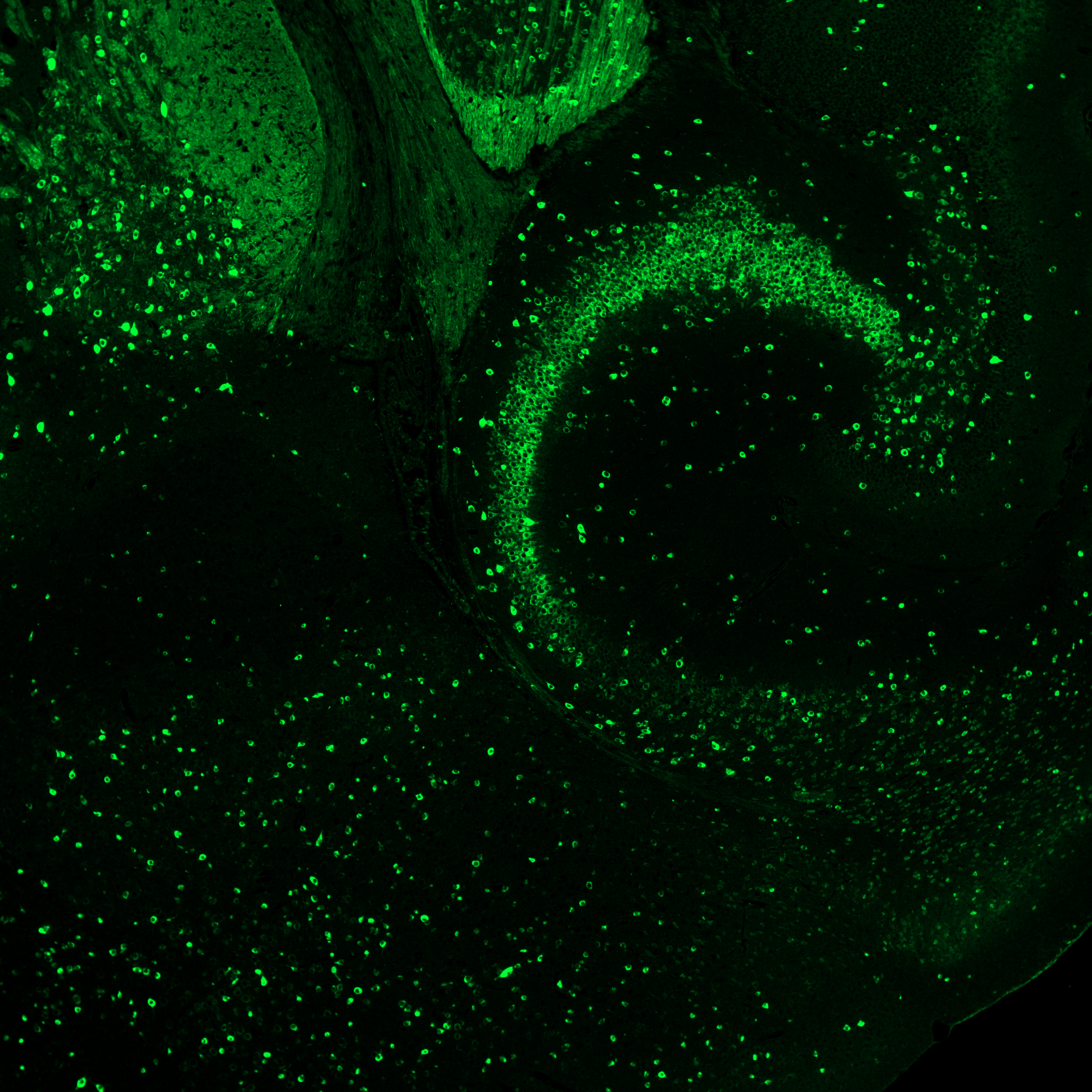

Supplement: Figure 1—source data 1. [file elife-86940-fig1-data1.zip › Figure 1-source data 1/35-CON-CII F+-1M-SAGITAL-HUB-CTIP2-61#-2-5X-vHPC-Image Export-08_AF488.tif]

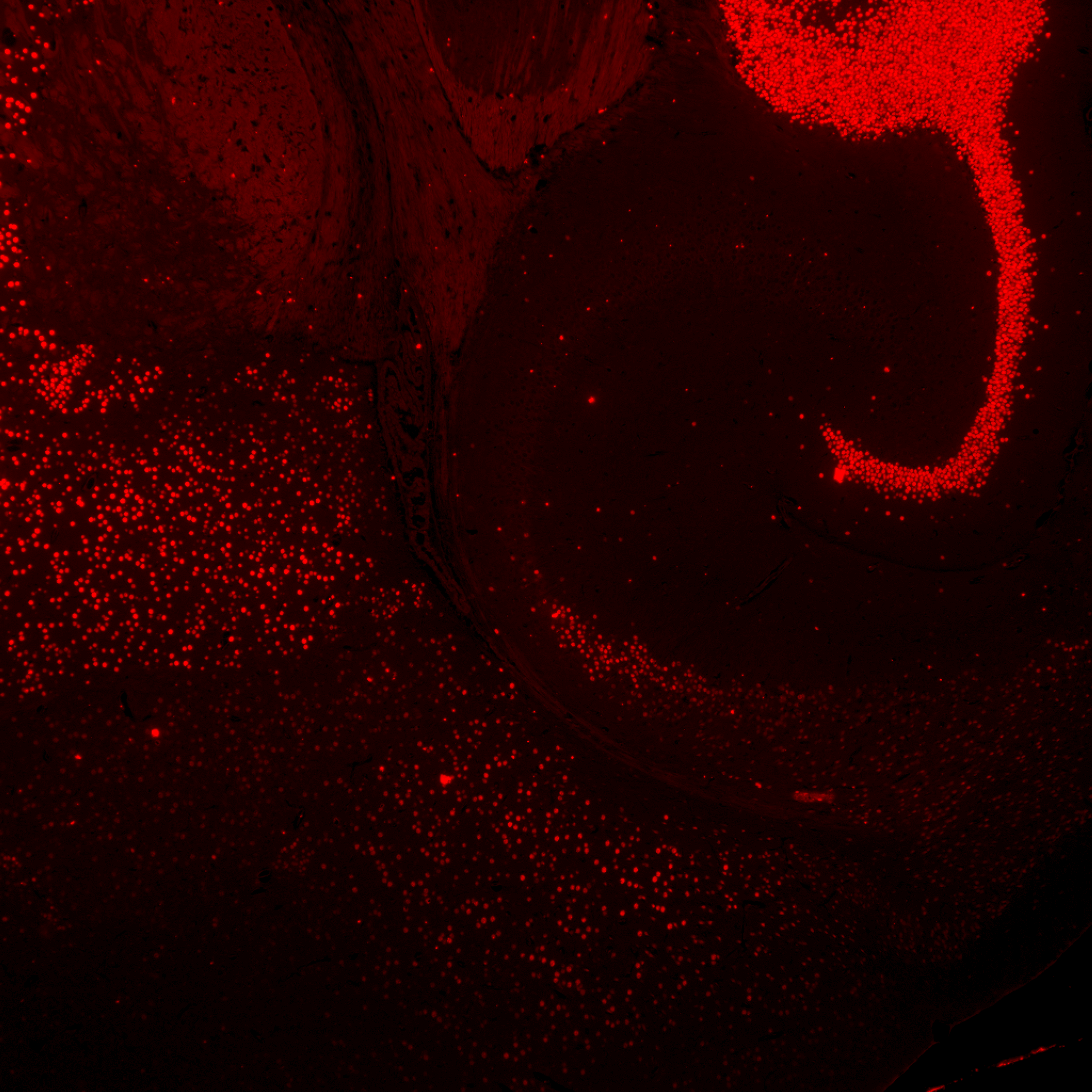

Supplement: Figure 1—source data 1. [file elife-86940-fig1-data1.zip › Figure 1-source data 1/35-CON-CII F+-1M-SAGITAL-HUB-CTIP2-61#-2-5X-vHPC-Image Export-08_AF594.tif]

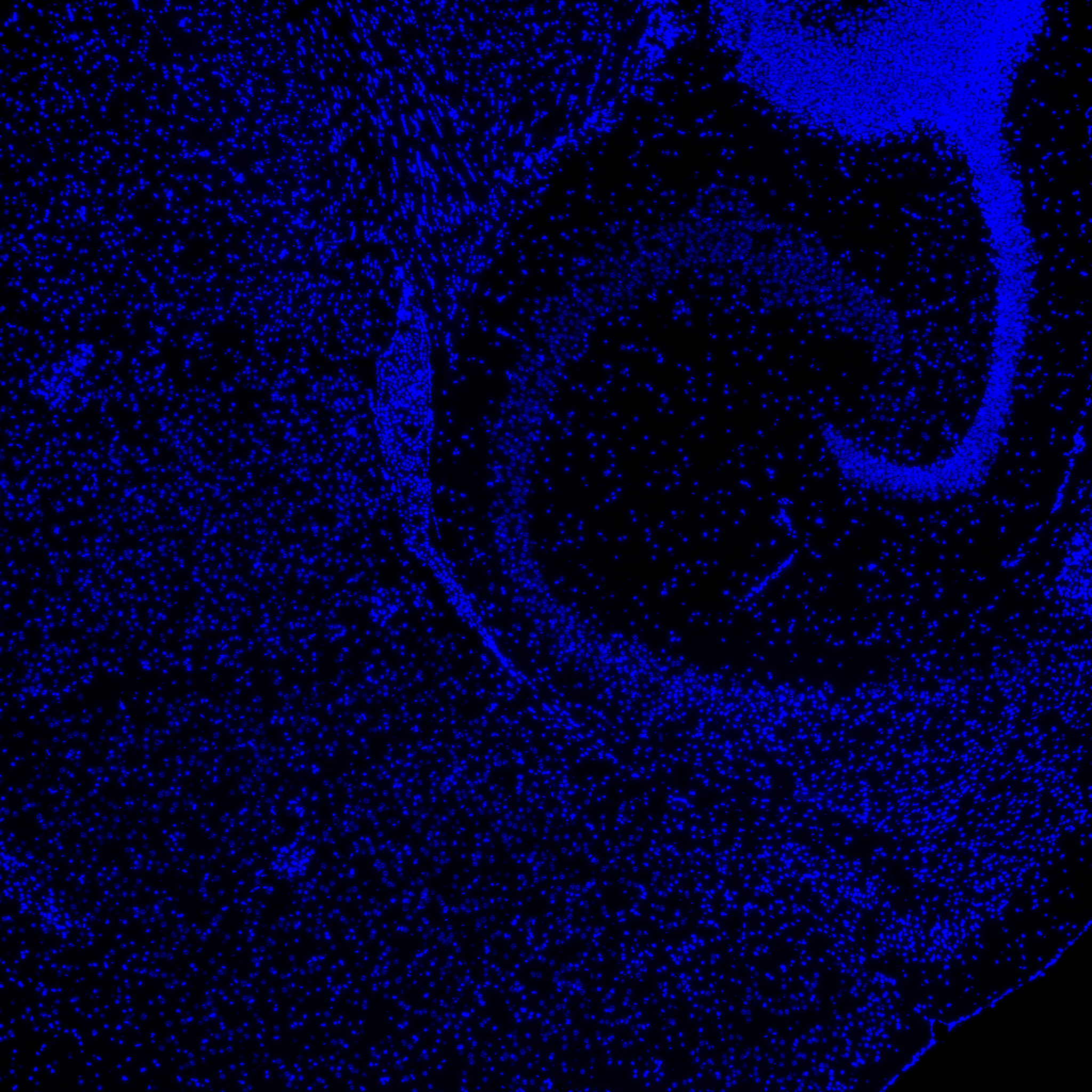

Supplement: Figure 1—source data 1. [file elife-86940-fig1-data1.zip › Figure 1-source data 1/35-CON-CII F+-1M-SAGITAL-HUB-CTIP2-61#-2-5X-vHPC-Image Export-08_DAPI.tif]

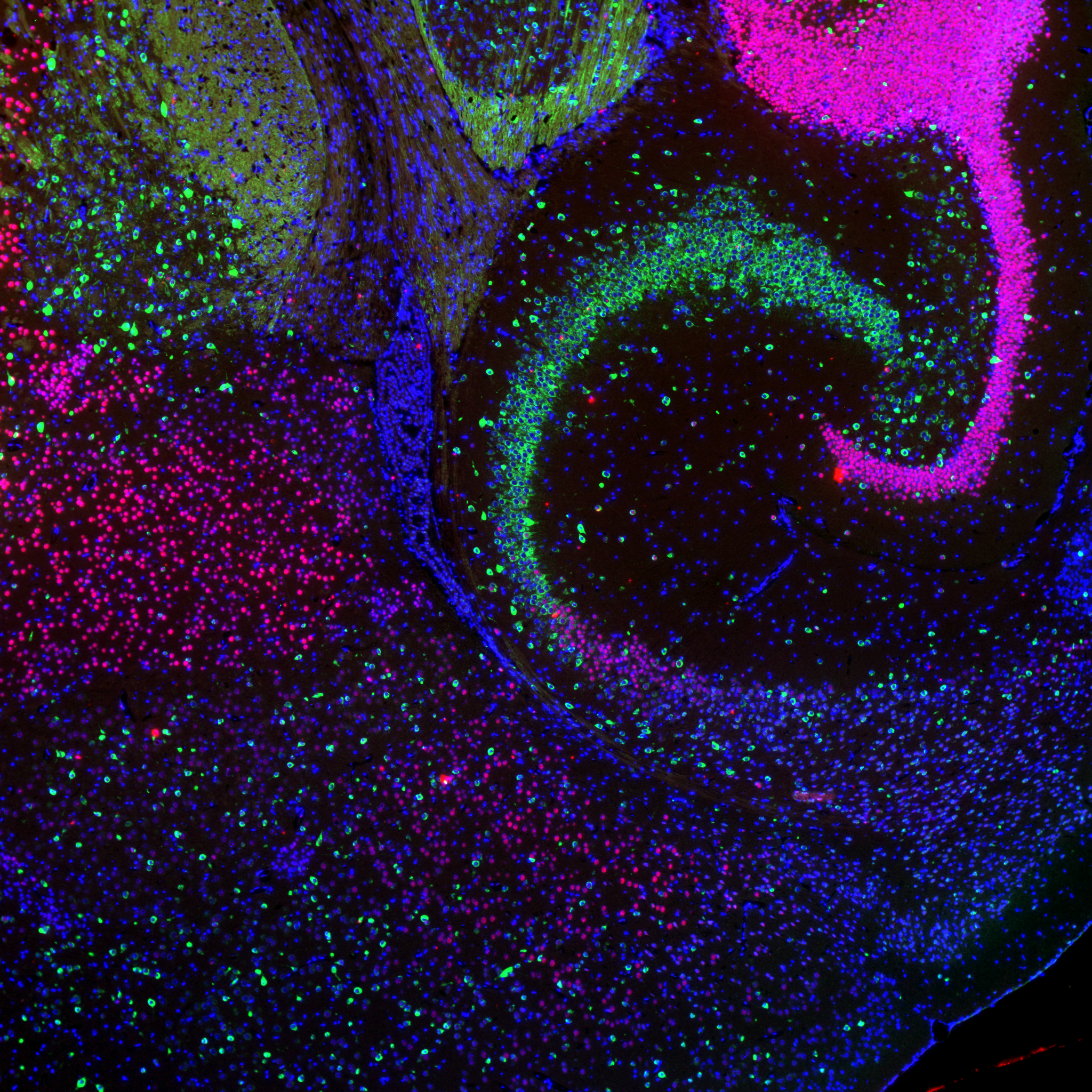

Supplement: Figure 1—source data 1. [file elife-86940-fig1-data1.zip › Figure 1-source data 1/35-CON-CII F+-1M-SAGITAL-HUB-CTIP2-61#-2-5X-vHPC-Image Export-08.tif]

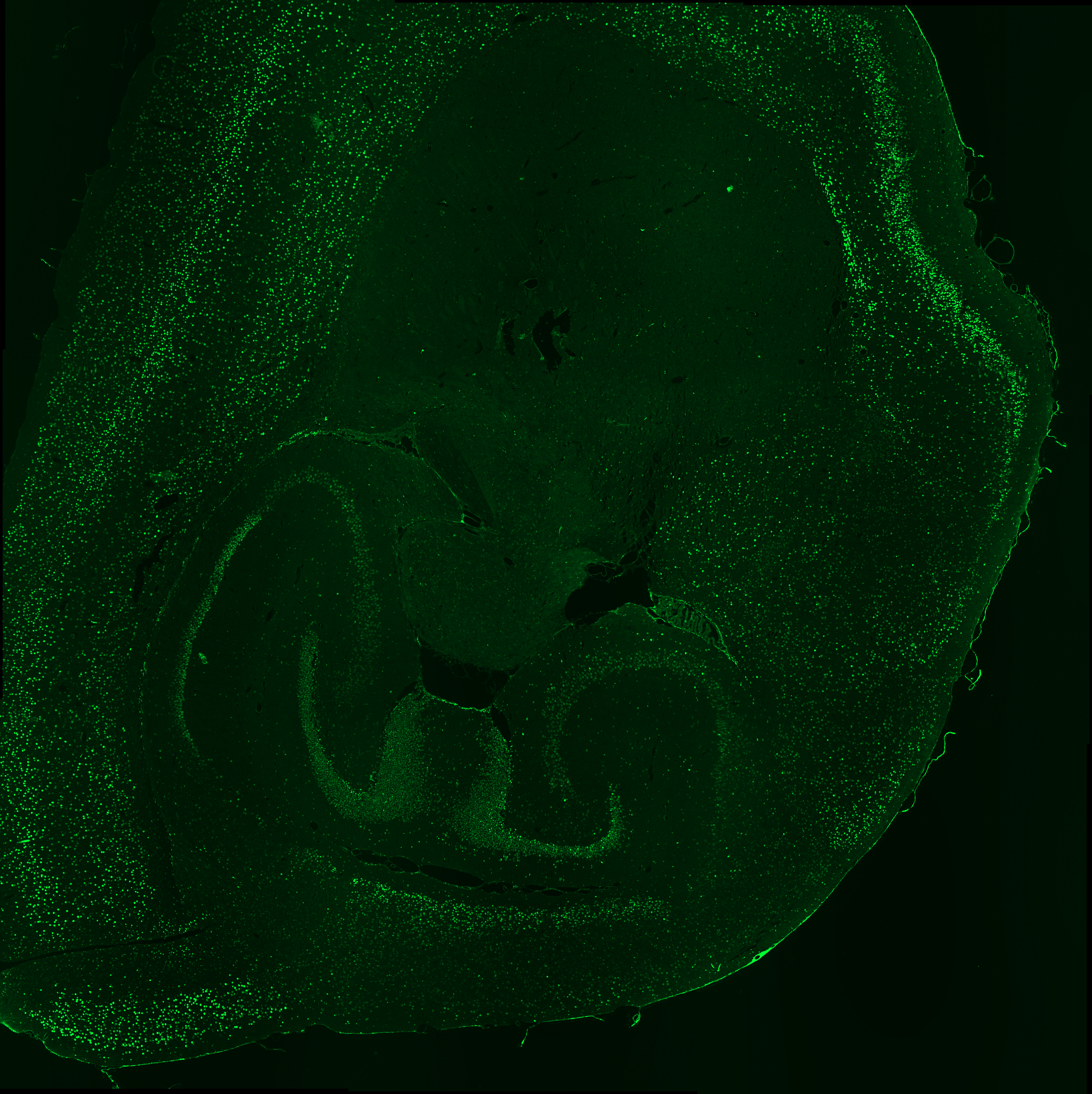

Supplement: Figure 1—source data 2. [file elife-86940-fig1-data2.zip › Figure 1-source data 2/3361-CON-f+-1M-SAGITAL-5X-CI-CII-1-HPC-Image Export-03_AF488-T2.tif]

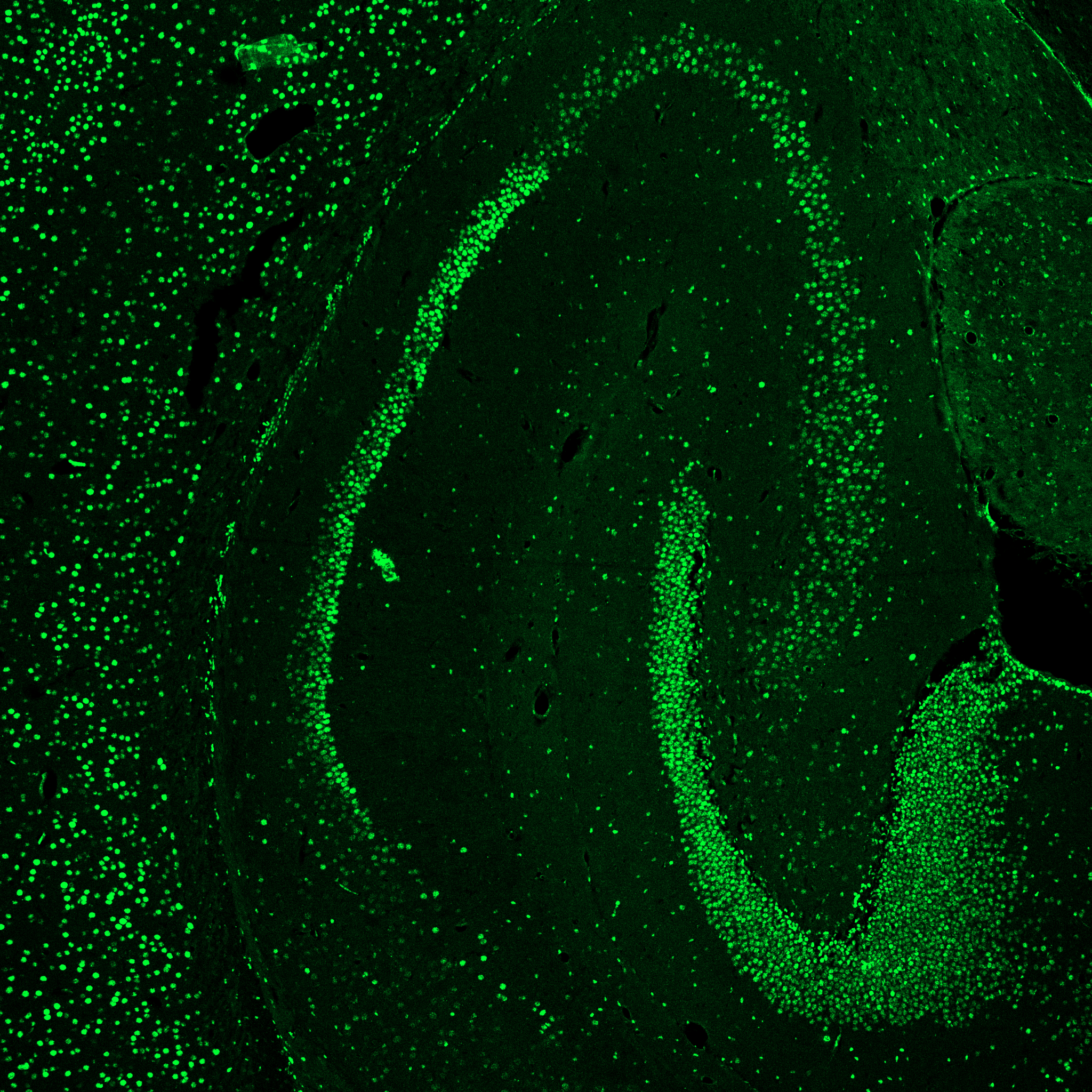

Supplement: Figure 1—source data 2. [file elife-86940-fig1-data2.zip › Figure 1-source data 2/3361-CON-f+-1M-SAGITAL-5X-CI-CII-1-dHPC-Image Export-04_AF488-T2.tif]

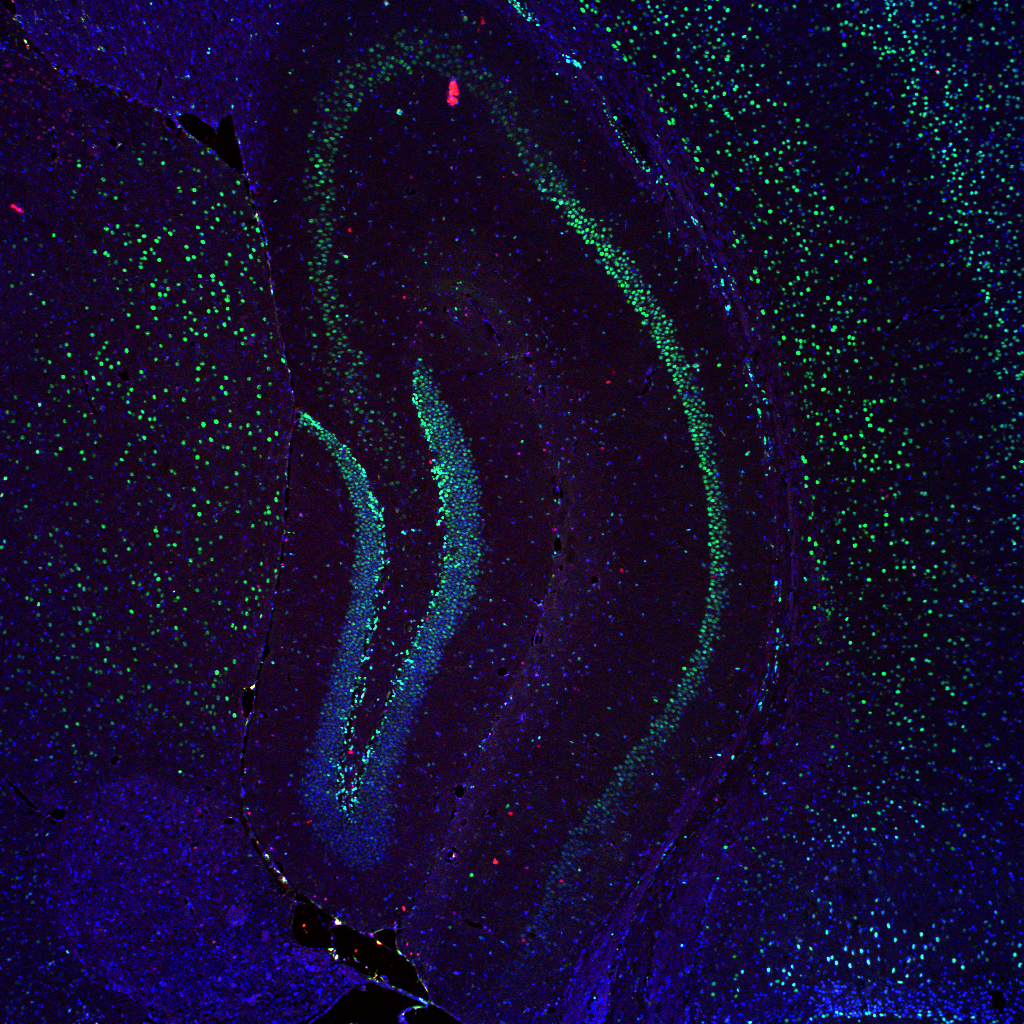

Supplement: Figure 1—source data 2. [file elife-86940-fig1-data2.zip › Figure 1-source data 2/WT-1M-5X-dHPC-CI-CII-2-L-Image Export-04.tif]

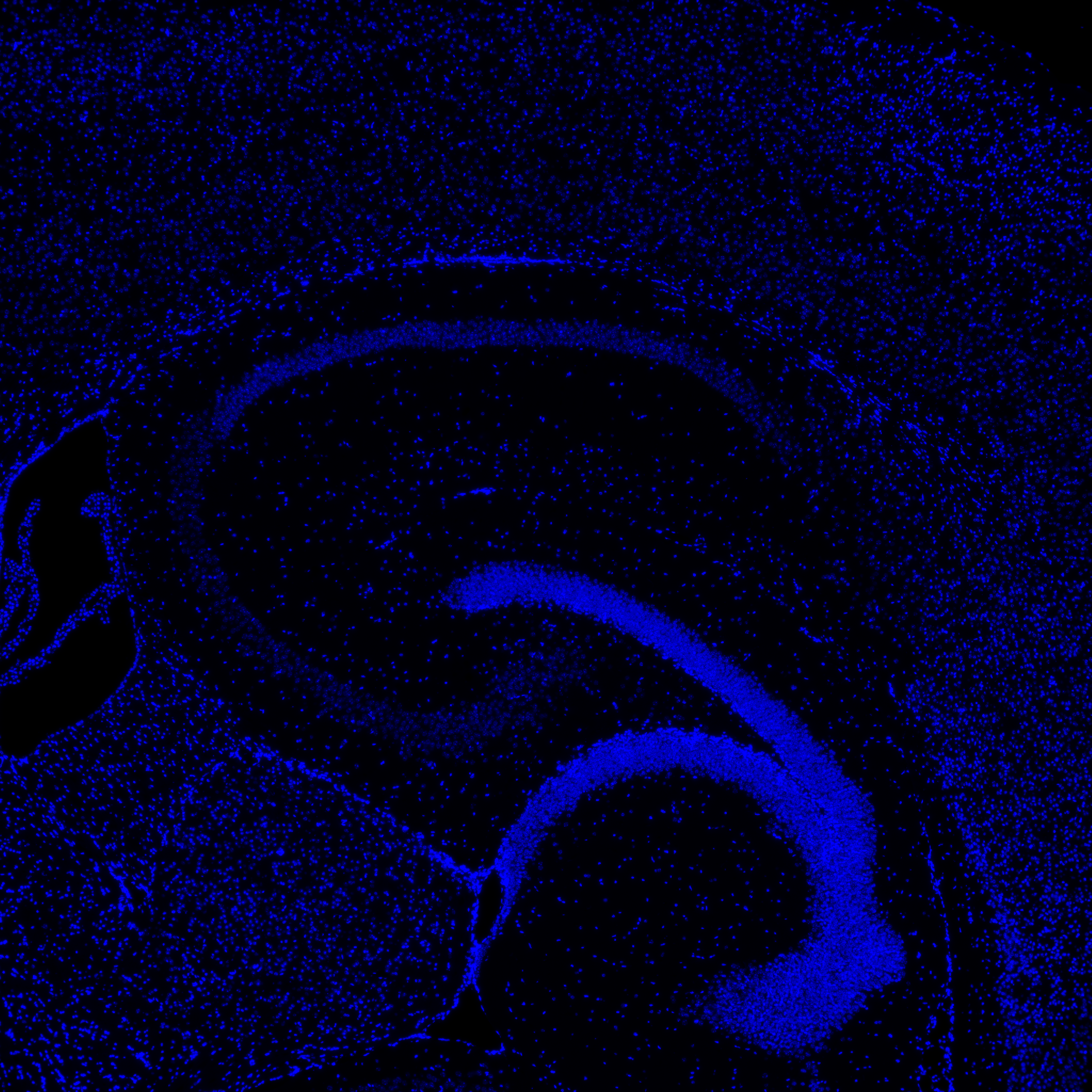

Supplement: Figure 1—source data 2. [file elife-86940-fig1-data2.zip › Figure 1-source data 2/36-CKO-RX CII FF-1M-SAGITAL-HUB-CTIP2-55#-2-5X-dHPC-Image Export-23_DAPI.tif]

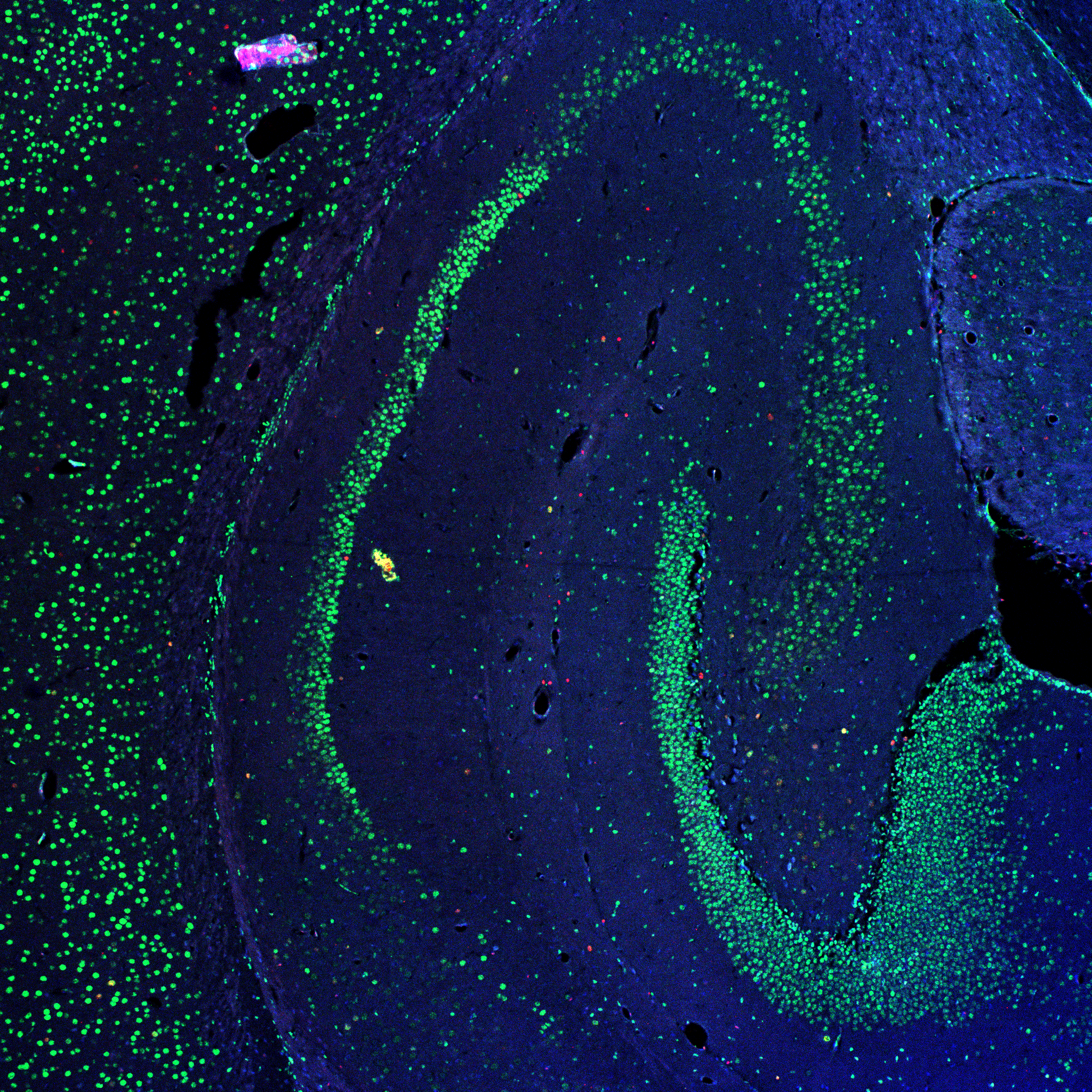

Supplement: Figure 1—source data 2. [file elife-86940-fig1-data2.zip › Figure 1-source data 2/3361-CON-f+-1M-SAGITAL-5X-CI-CII-1-dHPC-Image Export-04.tif]

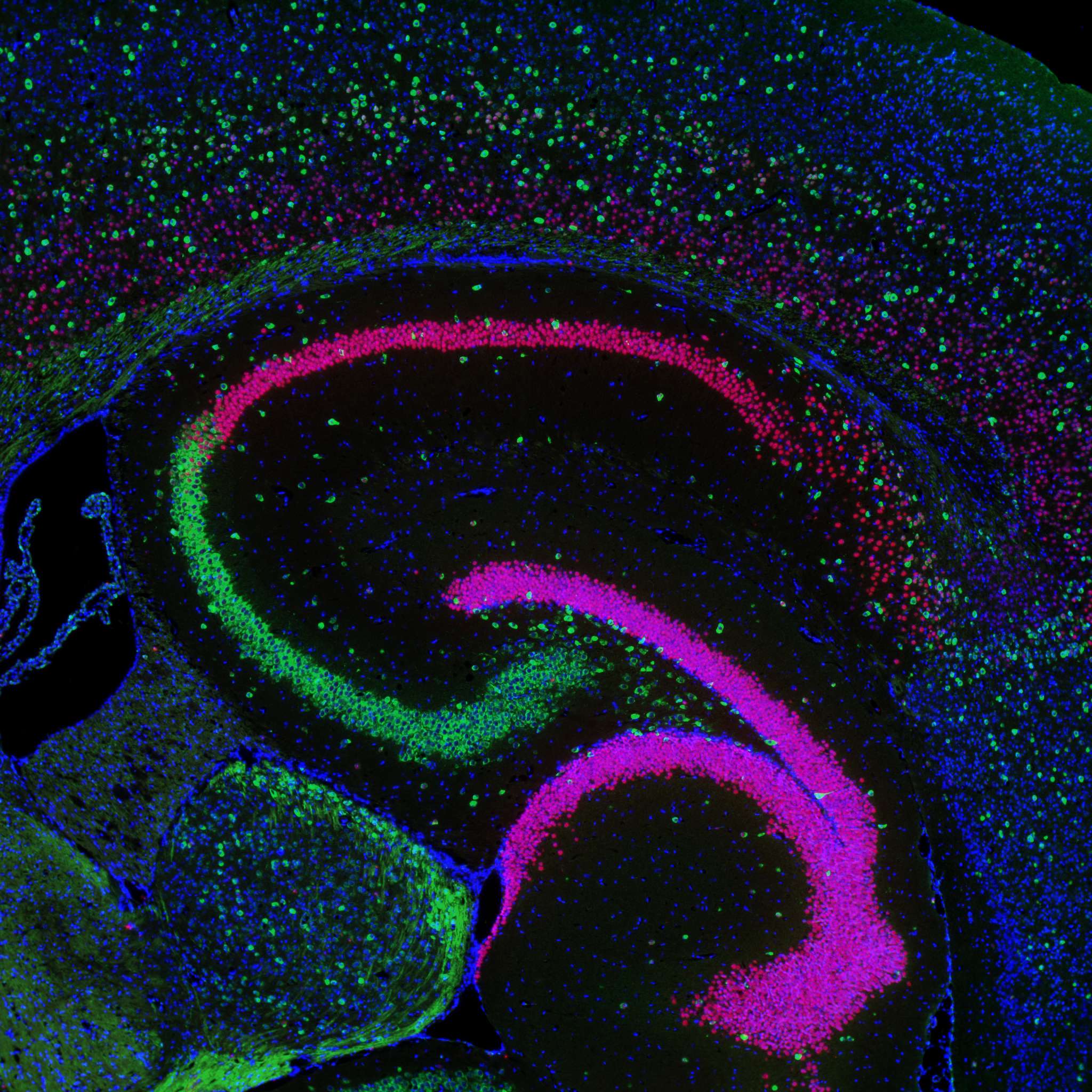

Supplement: Figure 1—source data 2. [file elife-86940-fig1-data2.zip › Figure 1-source data 2/36-CKO-RX CII FF-1M-SAGITAL-HUB-CTIP2-55#-2-5X-dHPC-Image Export-23.tif]

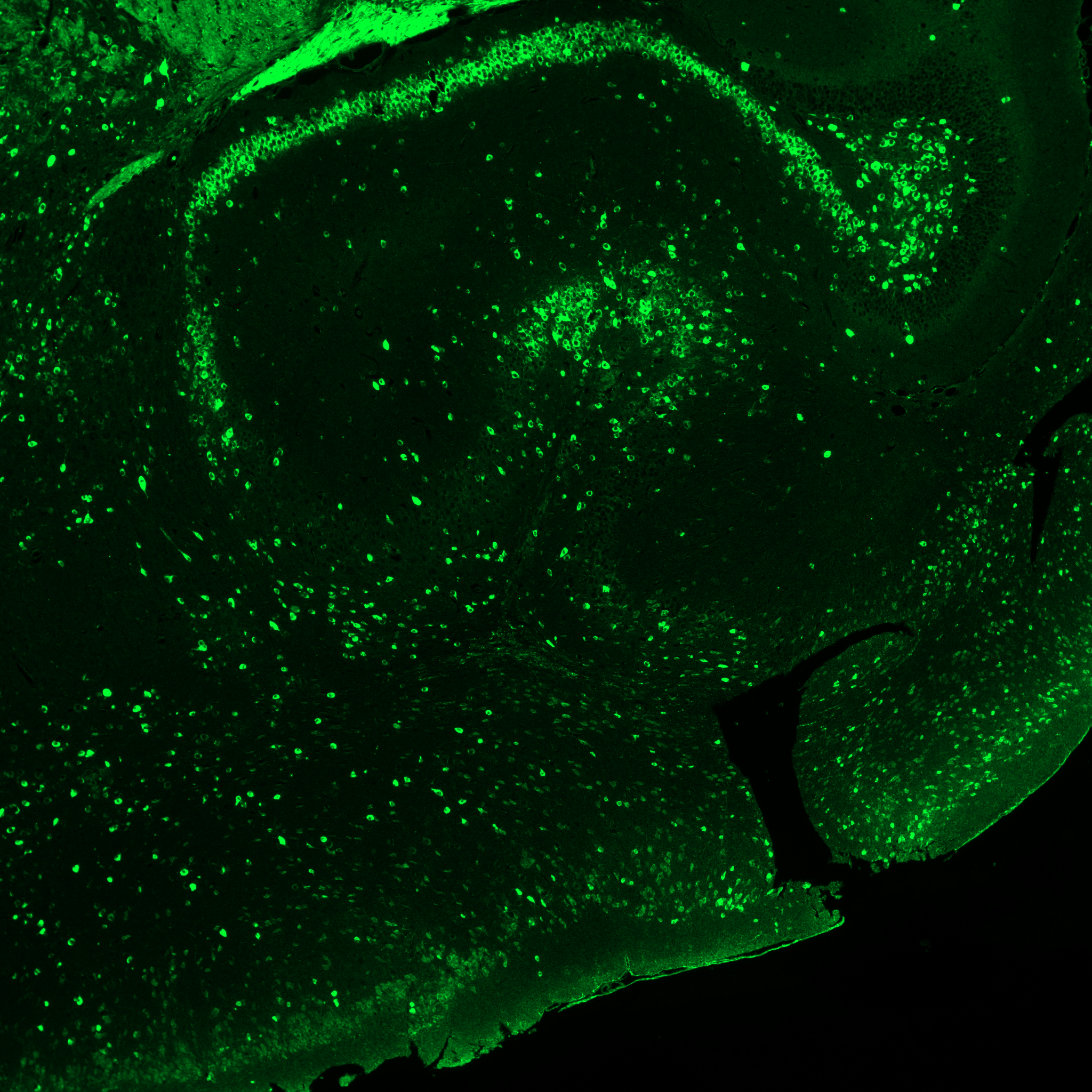

Supplement: Figure 1—source data 2. [file elife-86940-fig1-data2.zip › Figure 1-source data 2/36-CKO-RX CII FF-1M-SAGITAL-HUB-CTIP2-55#-2-5X-vHPC-Image Export-24_AF488.tif]

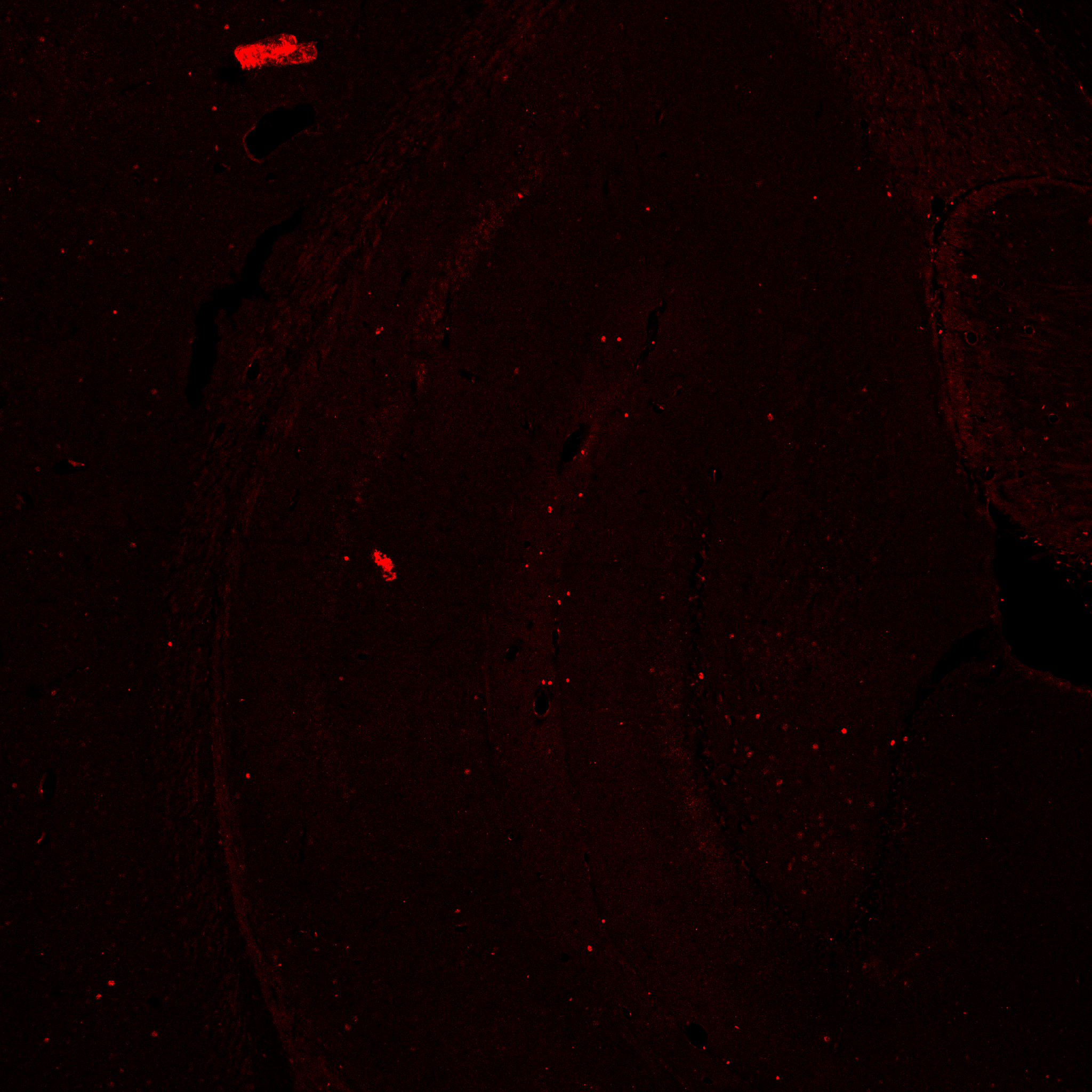

Supplement: Figure 1—source data 2. [file elife-86940-fig1-data2.zip › Figure 1-source data 2/3361-CON-f+-1M-SAGITAL-5X-CI-CII-1-dHPC-Image Export-04_AF594-T1.tif]

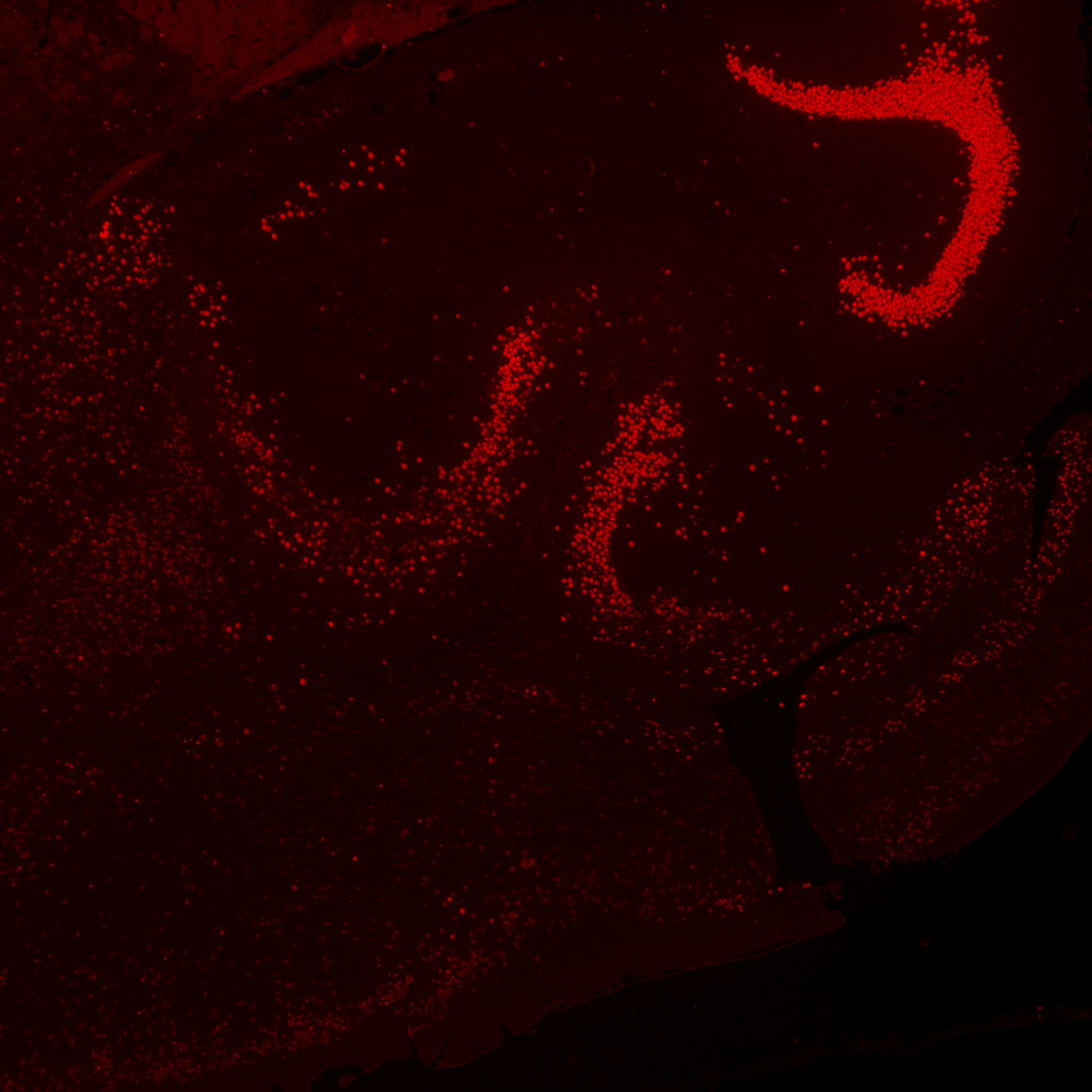

Supplement: Figure 1—source data 2. [file elife-86940-fig1-data2.zip › Figure 1-source data 2/36-CKO-RX CII FF-1M-SAGITAL-HUB-CTIP2-55#-2-5X-vHPC-Image Export-24_AF594.tif]

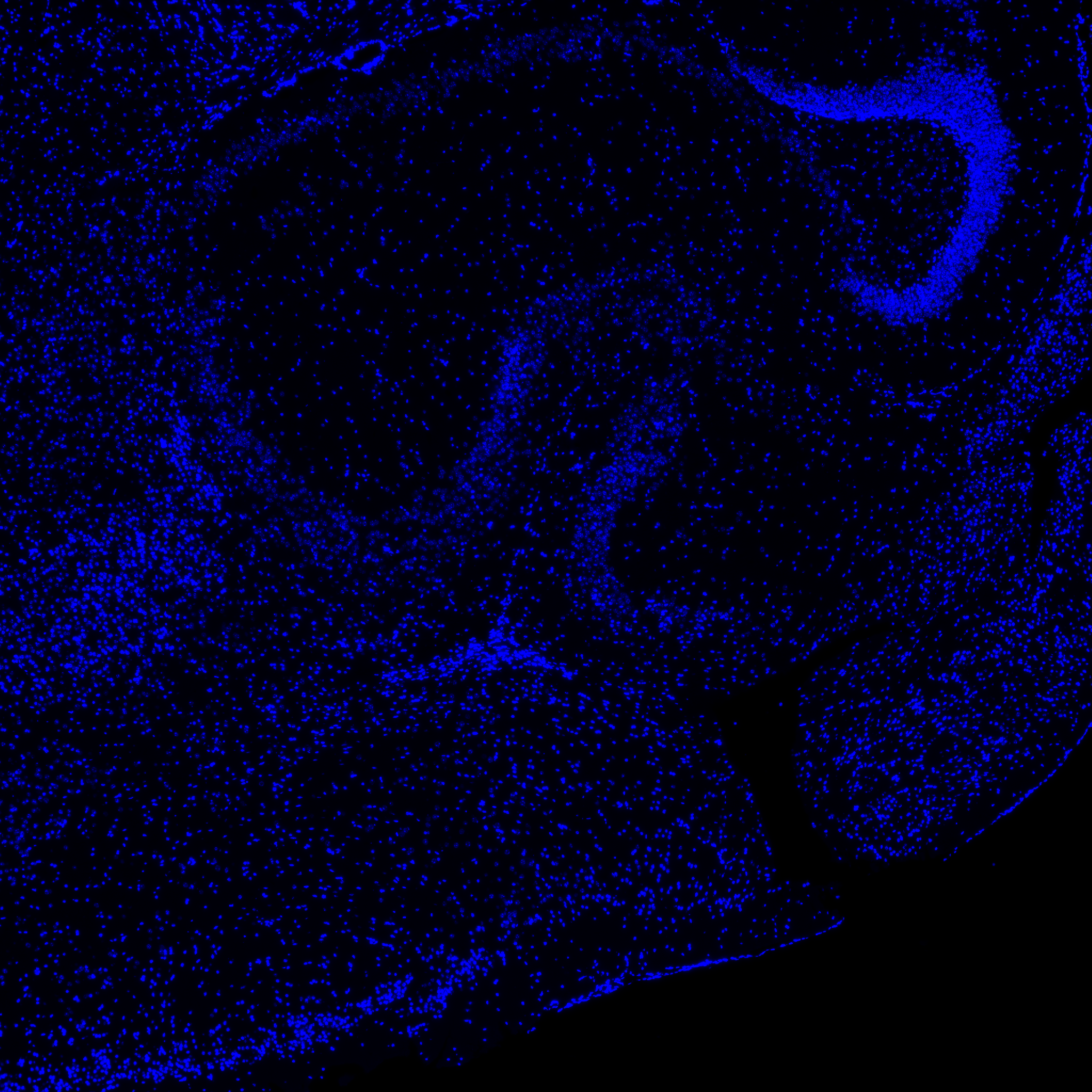

Supplement: Figure 1—source data 2. [file elife-86940-fig1-data2.zip › Figure 1-source data 2/36-CKO-RX CII FF-1M-SAGITAL-HUB-CTIP2-55#-2-5X-vHPC-Image Export-24_DAPI.tif]

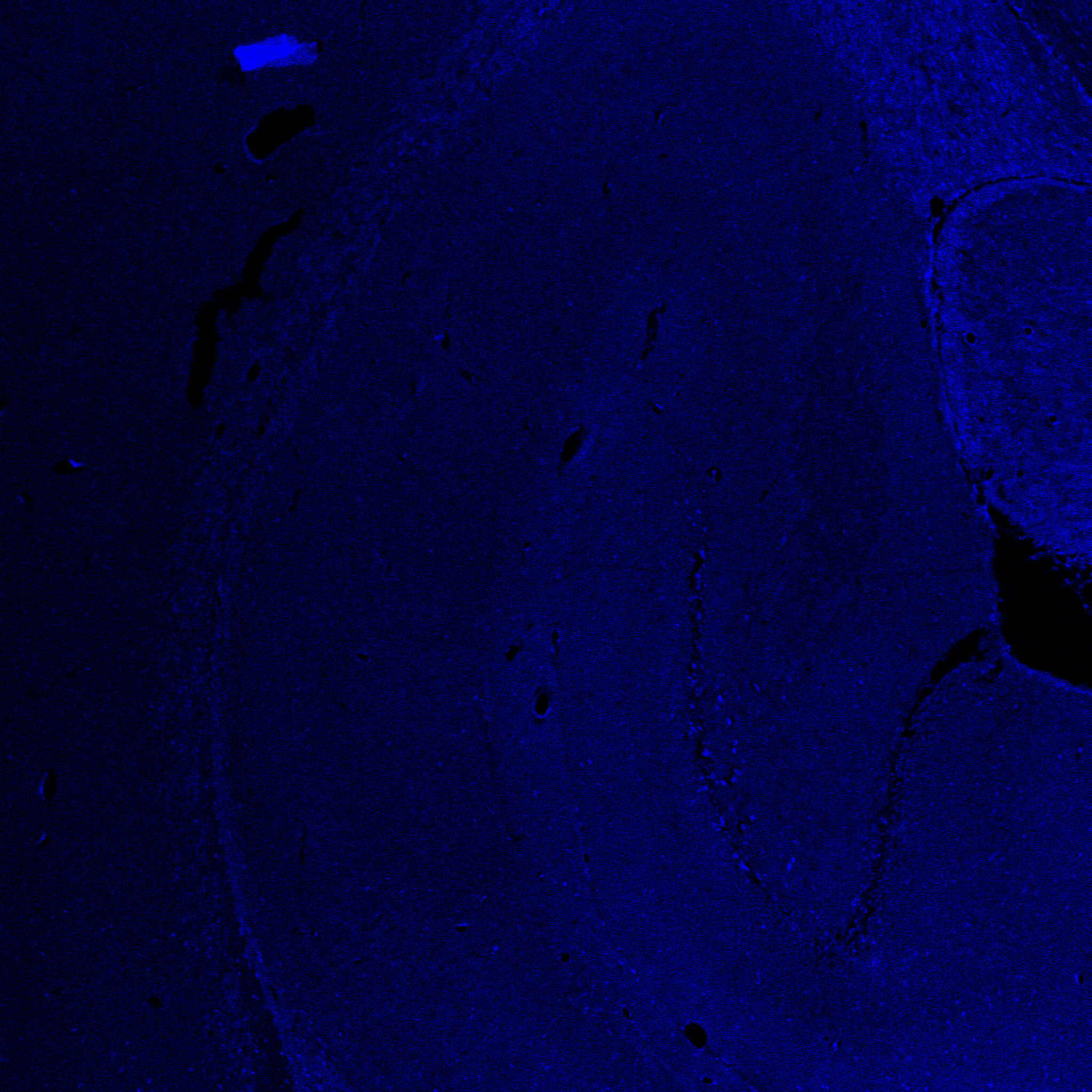

Supplement: Figure 1—source data 2. [file elife-86940-fig1-data2.zip › Figure 1-source data 2/3361-CON-f+-1M-SAGITAL-5X-CI-CII-1-dHPC-Image Export-04_DAPI-T3.tif]

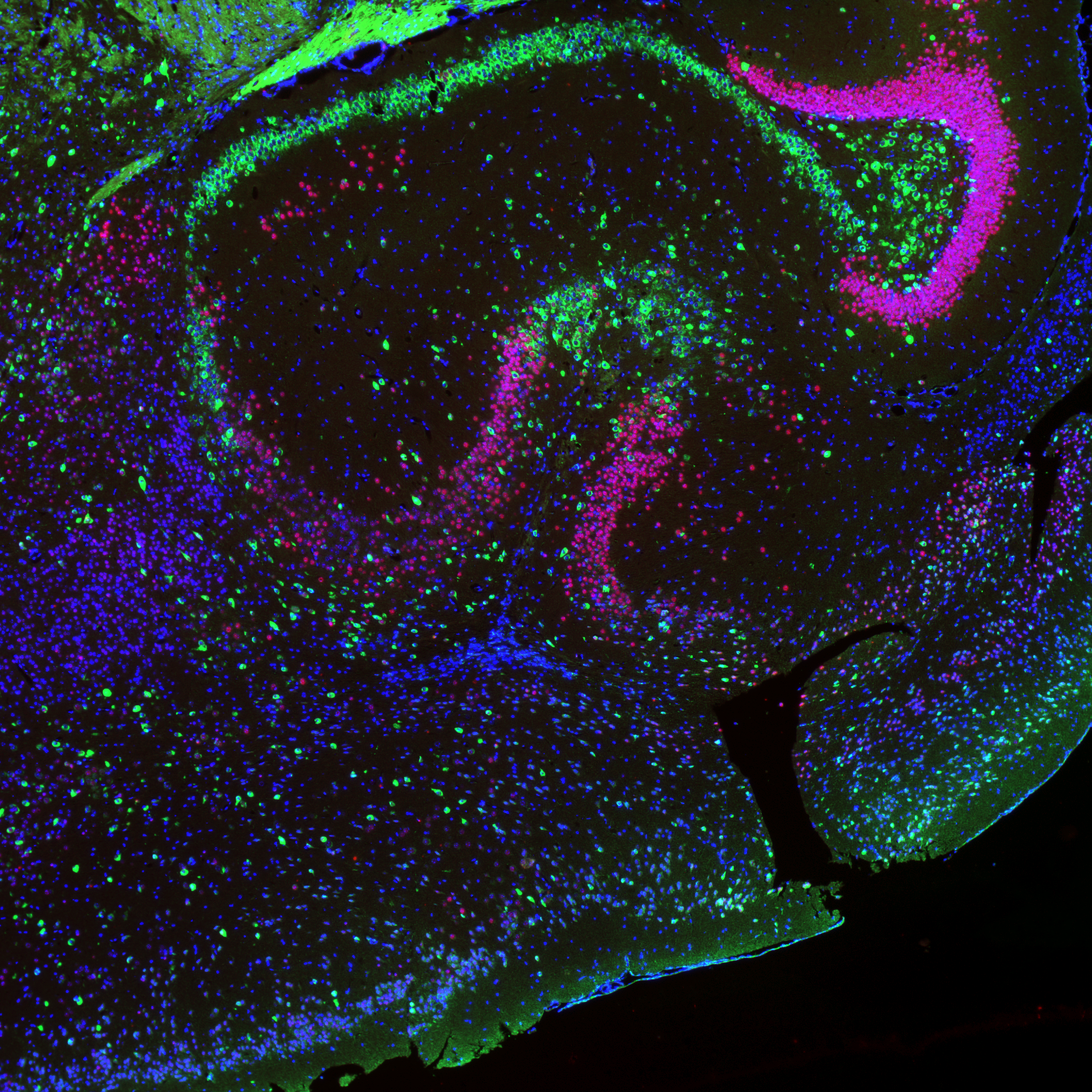

Supplement: Figure 1—source data 2. [file elife-86940-fig1-data2.zip › Figure 1-source data 2/36-CKO-RX CII FF-1M-SAGITAL-HUB-CTIP2-55#-2-5X-vHPC-Image Export-24.tif]

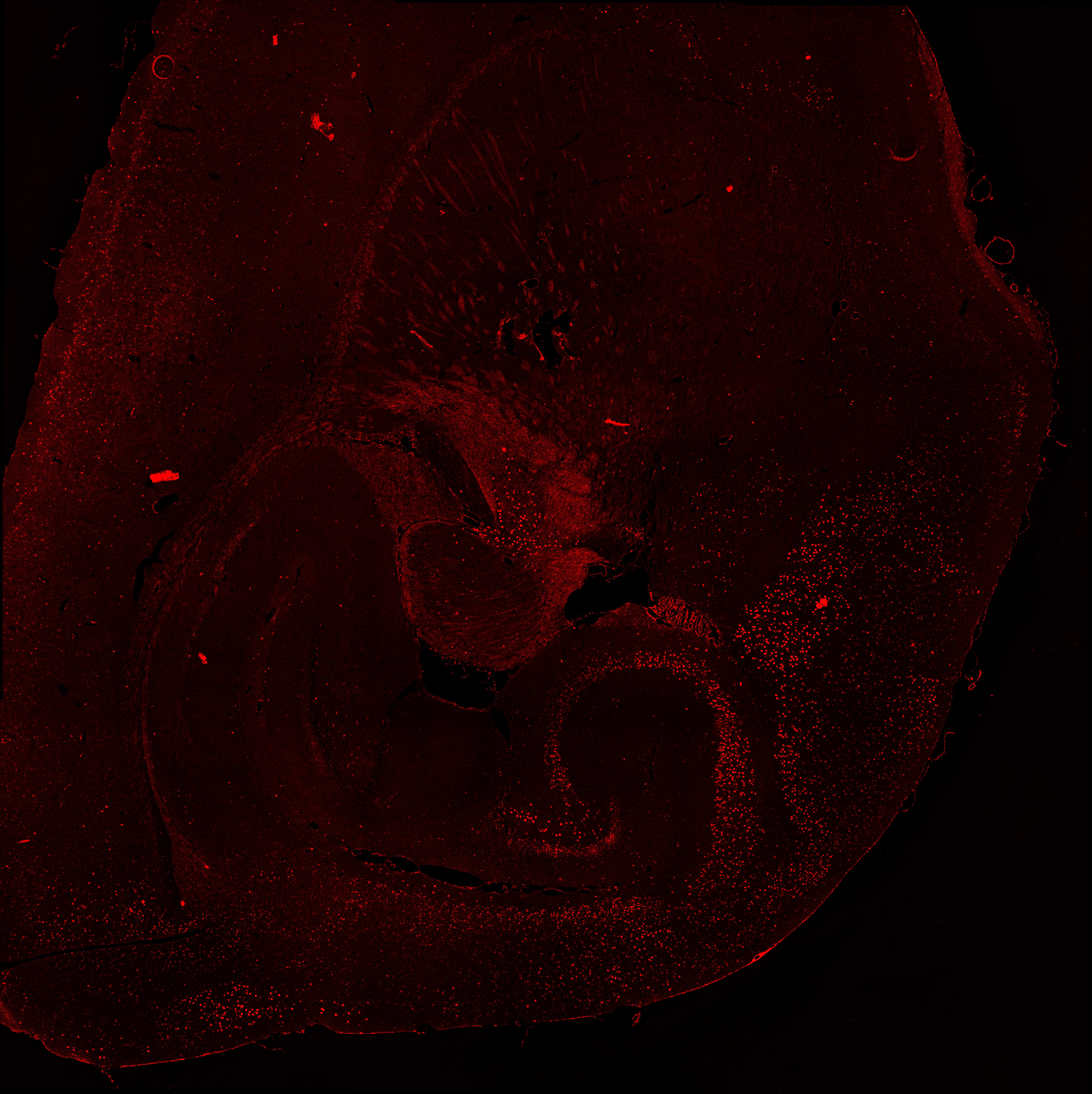

Supplement: Figure 1—source data 3. [file elife-86940-fig1-data3.zip › Figure 1-source data 3/3361-CON-f+-1M-SAGITAL-5X-CI-CII-1-HPC-Image Export-03_AF594-T1.tif]

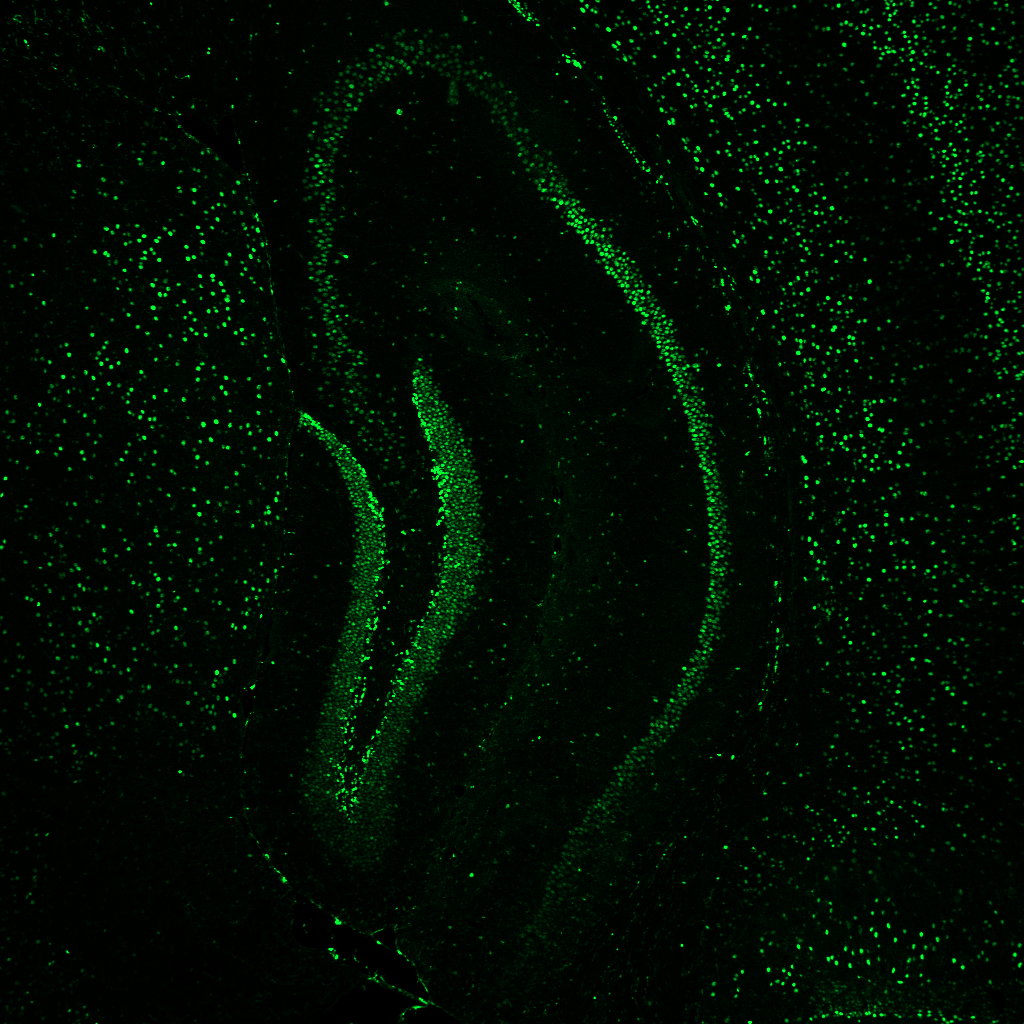

Supplement: Figure 1—source data 3. [file elife-86940-fig1-data3.zip › Figure 1-source data 3/WT-1M-5X-dHPC-CI-CII-2-L-Image Export-04_AF488-T2.tif]

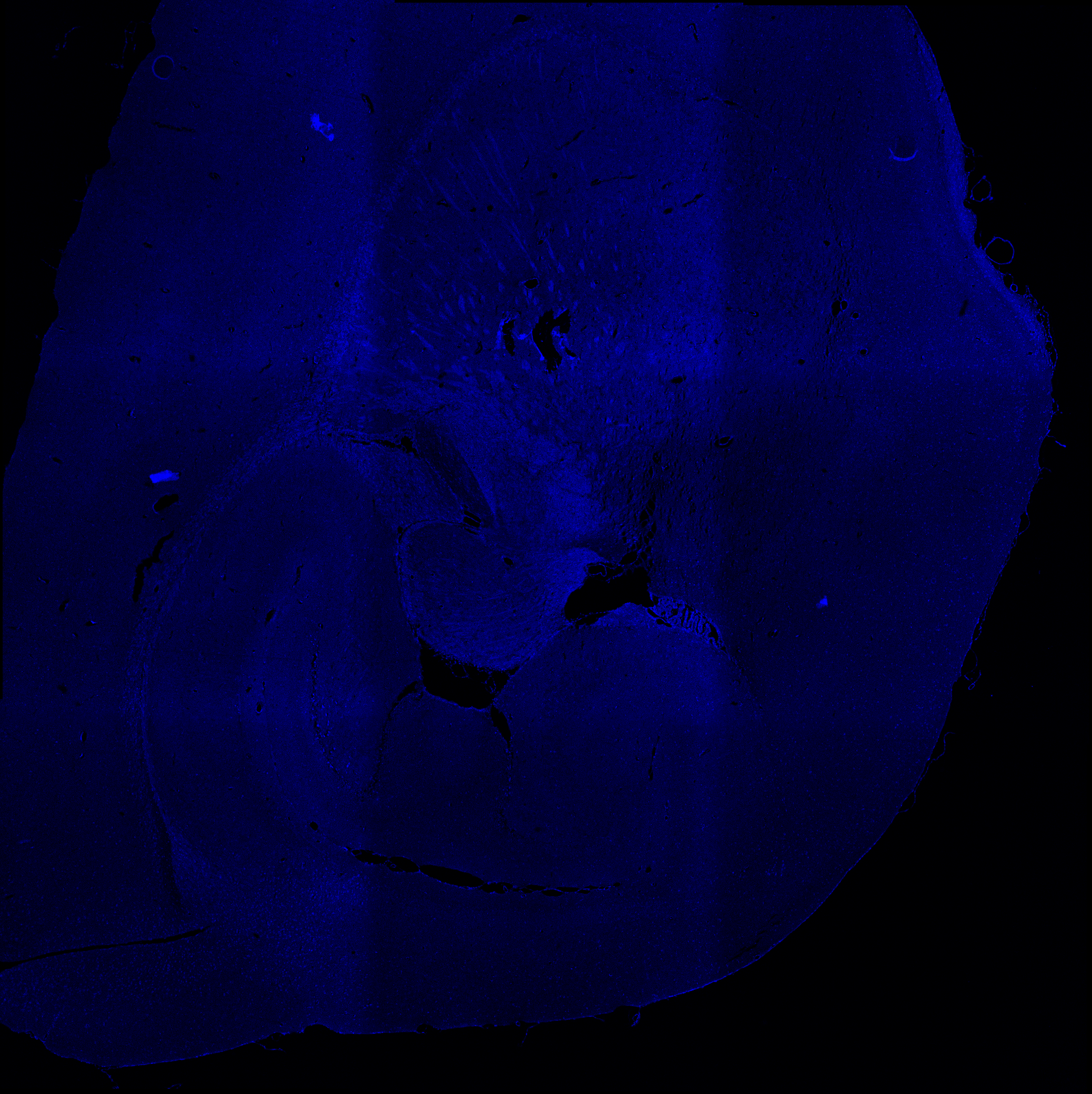

Supplement: Figure 1—source data 3. [file elife-86940-fig1-data3.zip › Figure 1-source data 3/3361-CON-f+-1M-SAGITAL-5X-CI-CII-1-HPC-Image Export-03_DAPI-T3.tif]

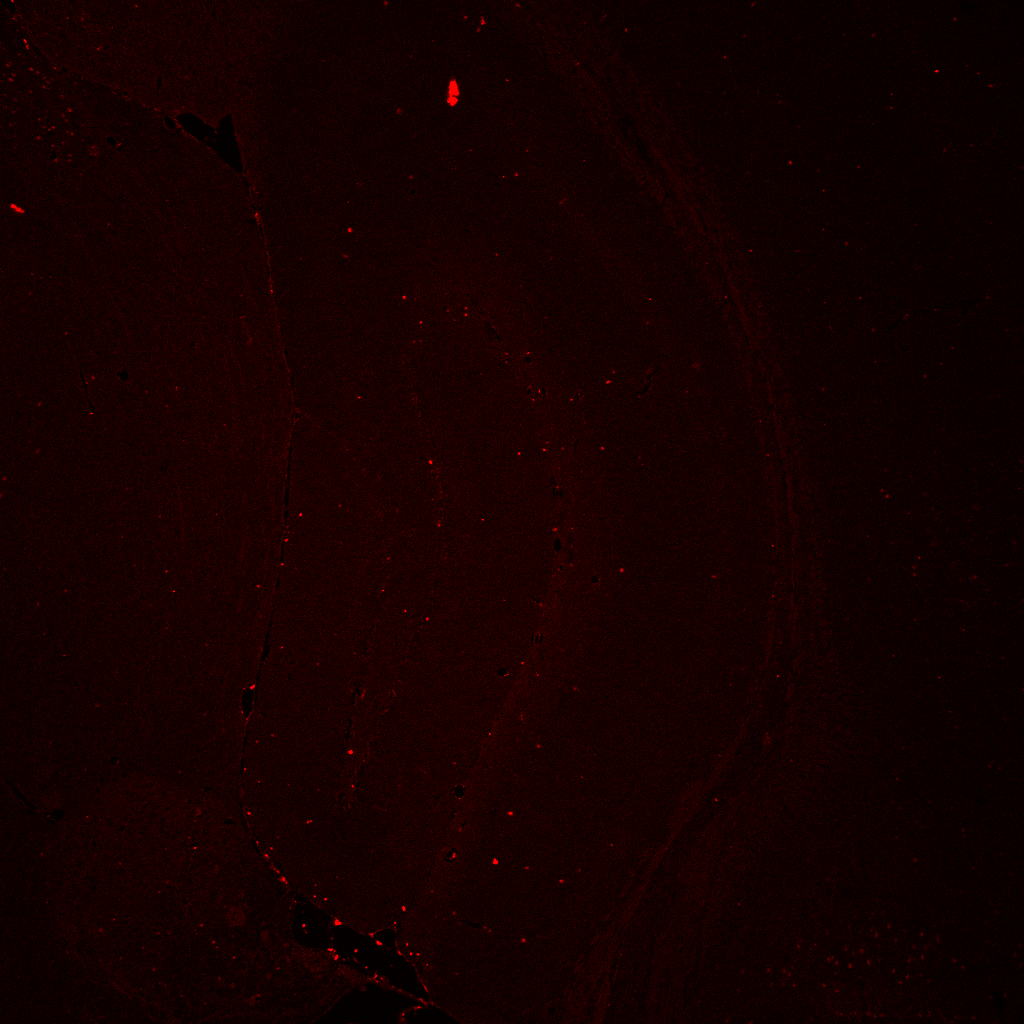

Supplement: Figure 1—source data 3. [file elife-86940-fig1-data3.zip › Figure 1-source data 3/WT-1M-5X-dHPC-CI-CII-2-L-Image Export-04_AF594-T1.tif]

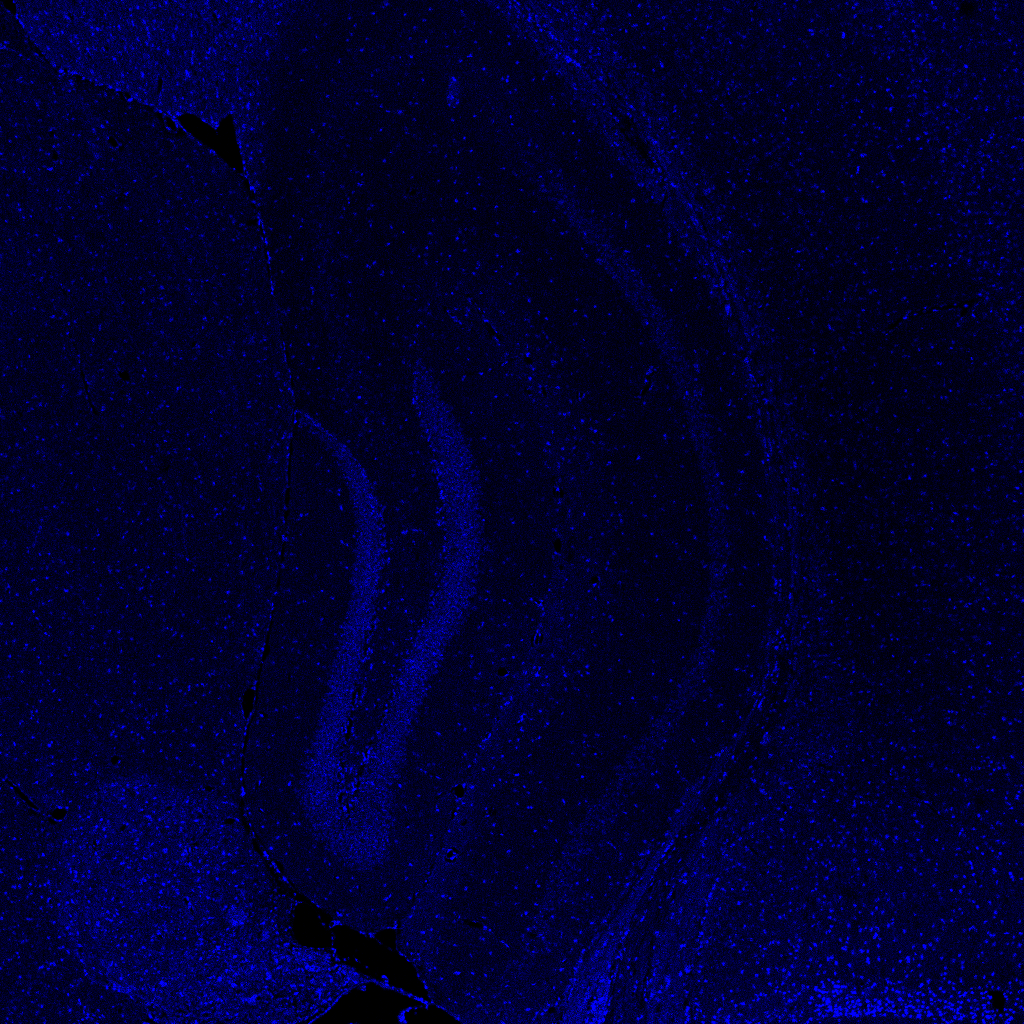

Supplement: Figure 1—source data 3. [file elife-86940-fig1-data3.zip › Figure 1-source data 3/WT-1M-5X-dHPC-CI-CII-2-L-Image Export-04_DAPI-T3.tif]

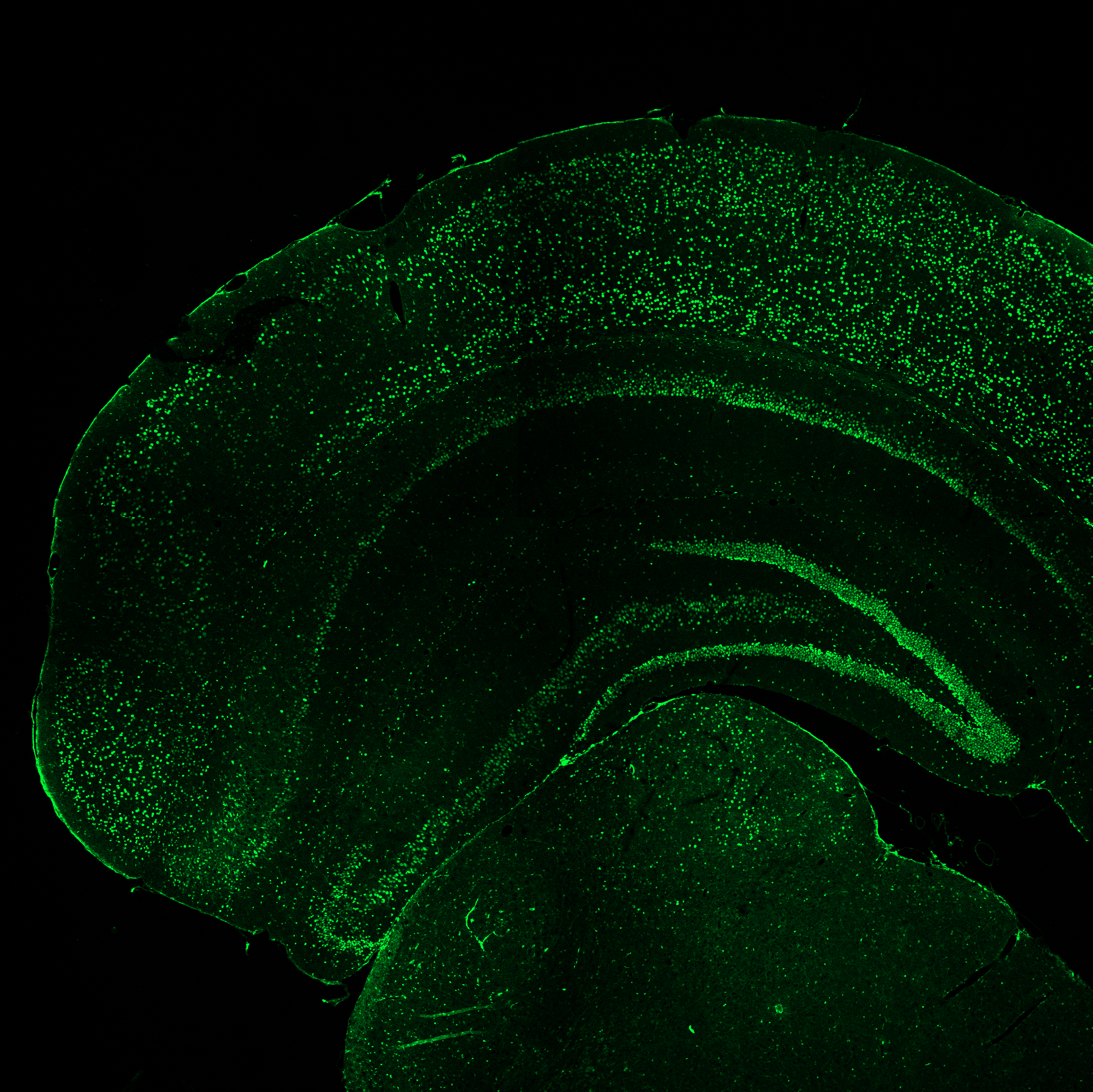

Supplement: Figure 1—source data 3. [file elife-86940-fig1-data3.zip › Figure 1-source data 3/WT-1M-136#-5X-HPC-CI-CII-3-L-Image Export-12_AF488-T2.tif]

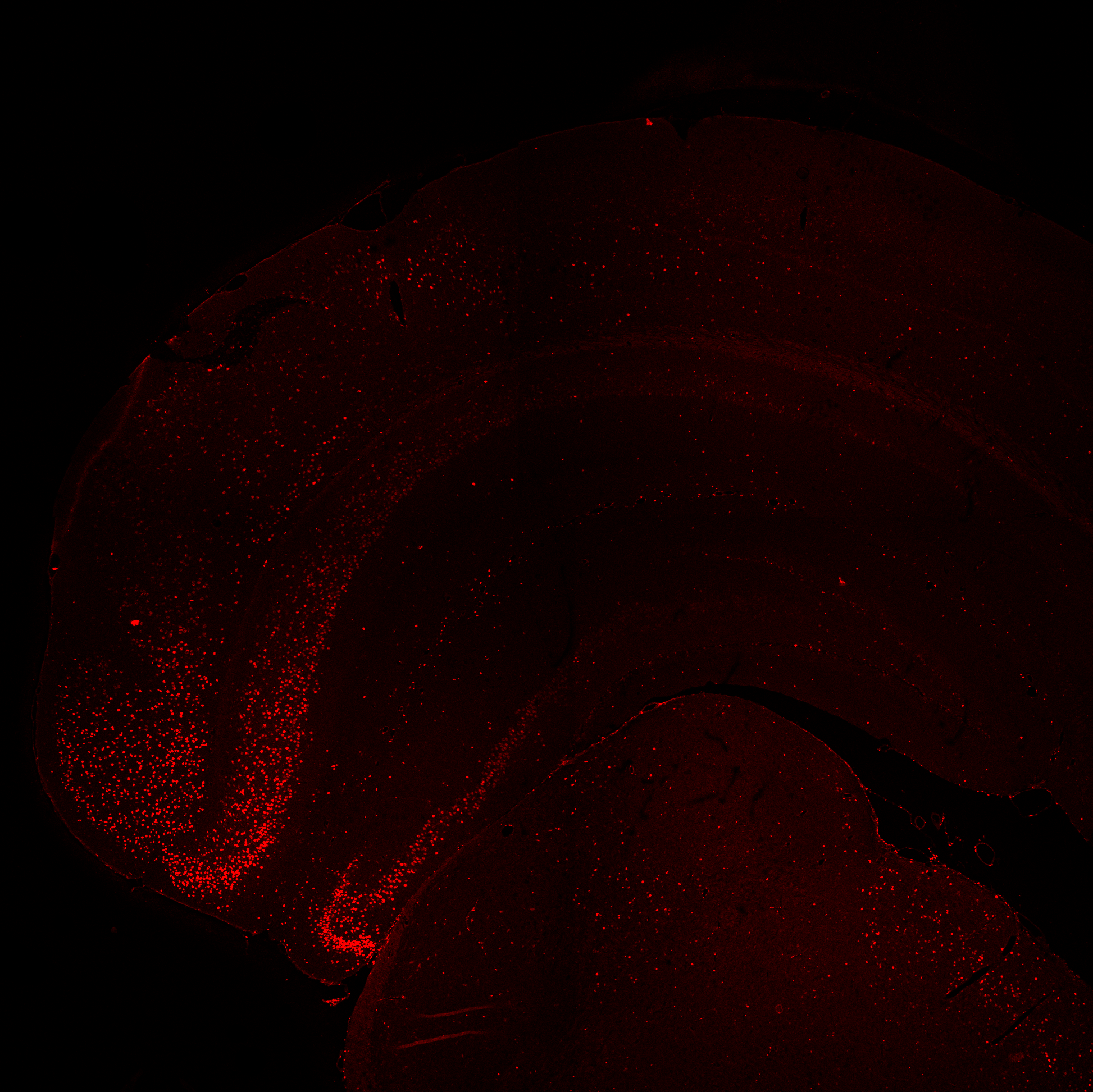

Supplement: Figure 1—source data 3. [file elife-86940-fig1-data3.zip › Figure 1-source data 3/WT-1M-136#-5X-HPC-CI-CII-3-L-Image Export-12_AF594-T1.tif]

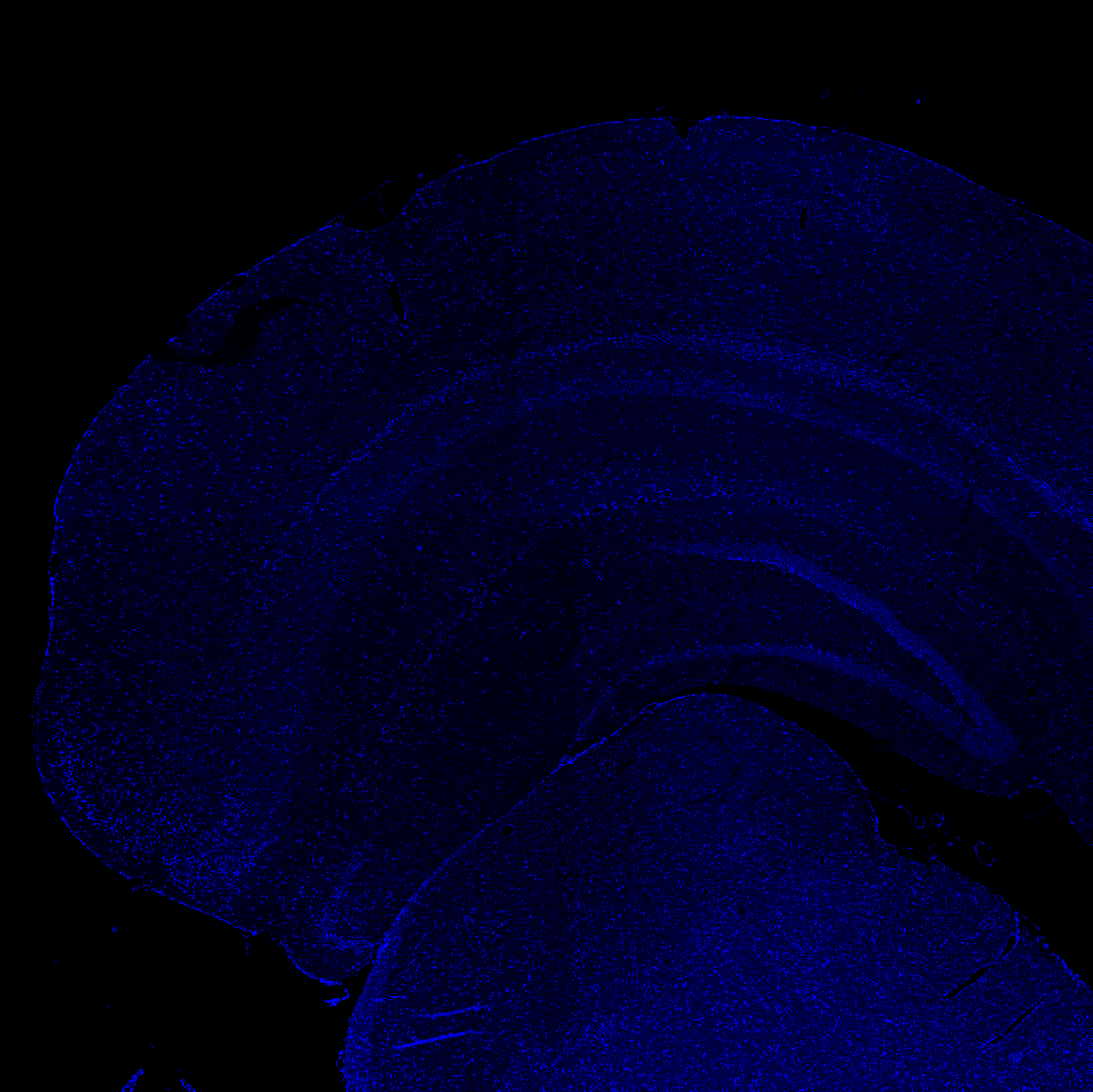

Supplement: Figure 1—source data 3. [file elife-86940-fig1-data3.zip › Figure 1-source data 3/WT-1M-136#-5X-HPC-CI-CII-3-L-Image Export-12_DAPI-T3.tif]

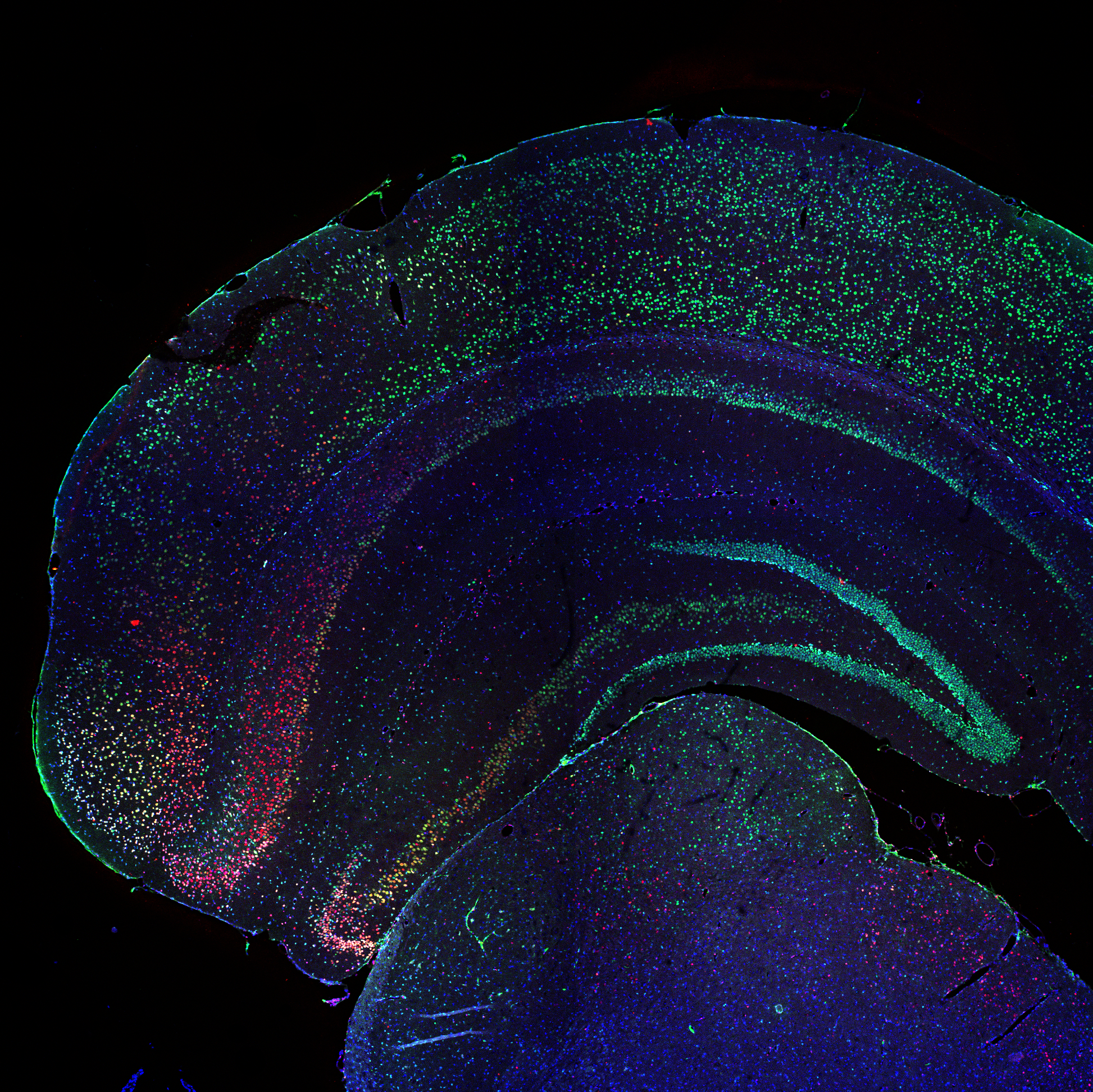

Supplement: Figure 1—source data 3. [file elife-86940-fig1-data3.zip › Figure 1-source data 3/WT-1M-136#-5X-HPC-CI-CII-3-L-Image Export-12.tif]

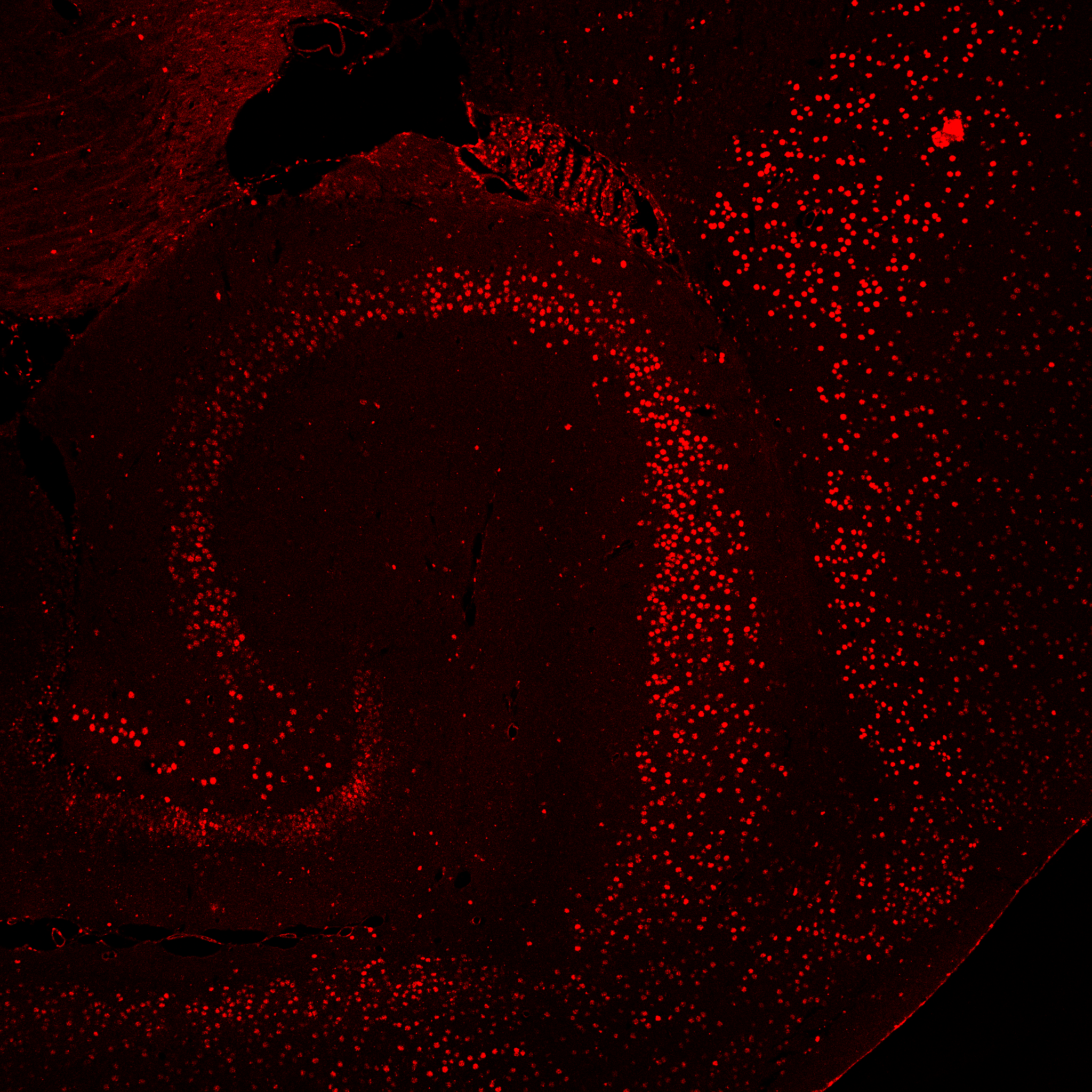

Supplement: Figure 1—source data 5. [file elife-86940-fig1-data5.zip › Figure 1-source data 5/3361-CON-f+-1M-SAGITAL-5X-CI-CII-1-vHPC-Image Export-05_AF594-T1.tif]

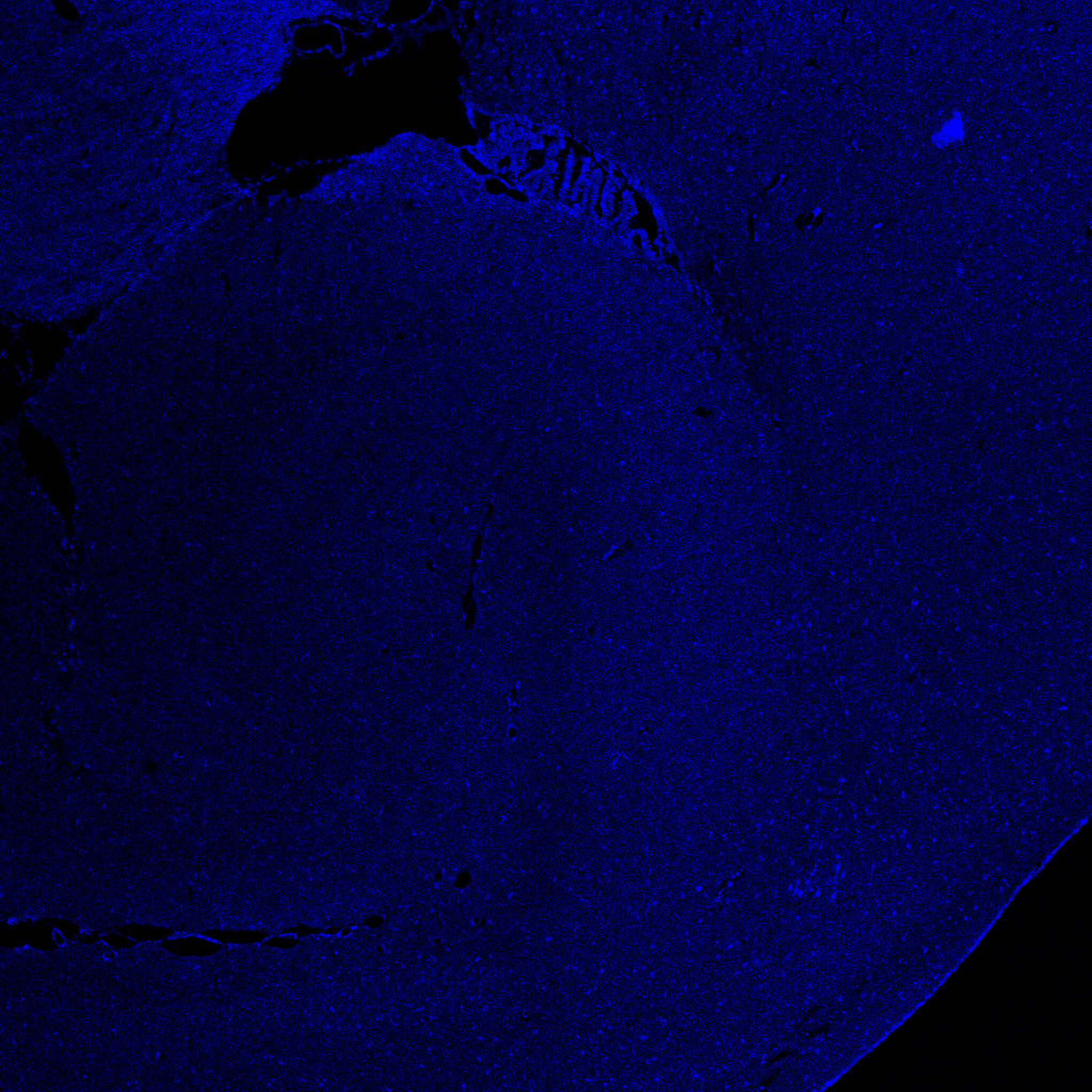

Supplement: Figure 1—source data 5. [file elife-86940-fig1-data5.zip › Figure 1-source data 5/3361-CON-f+-1M-SAGITAL-5X-CI-CII-1-vHPC-Image Export-05_DAPI-T3.tif]

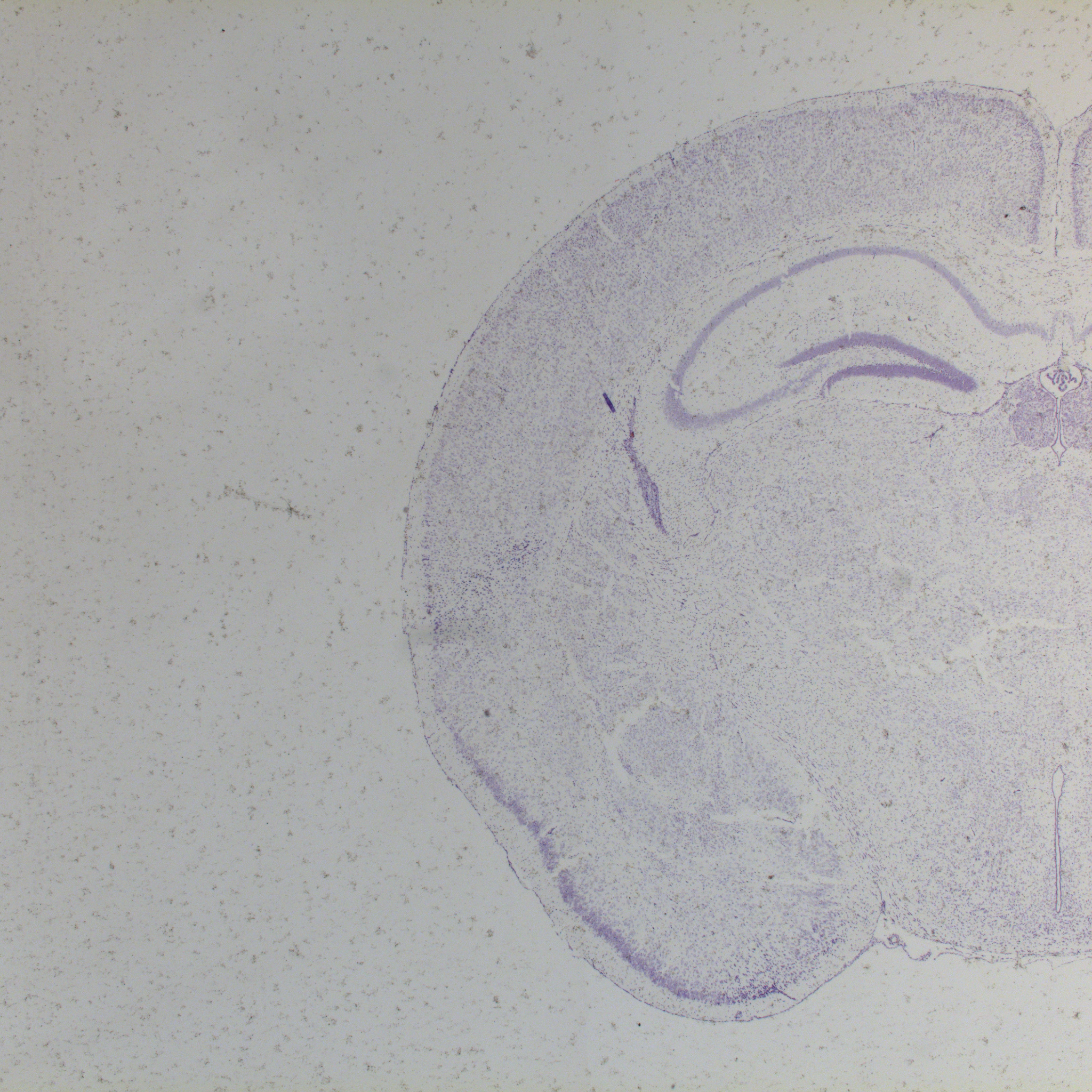

Supplement: Figure 1—source data 5. [file elife-86940-fig1-data5.zip › Figure 1-source data 5/3775-CII CKO-2.5X-RX CII FF-1M-#61-1-Image Export-11.tif]

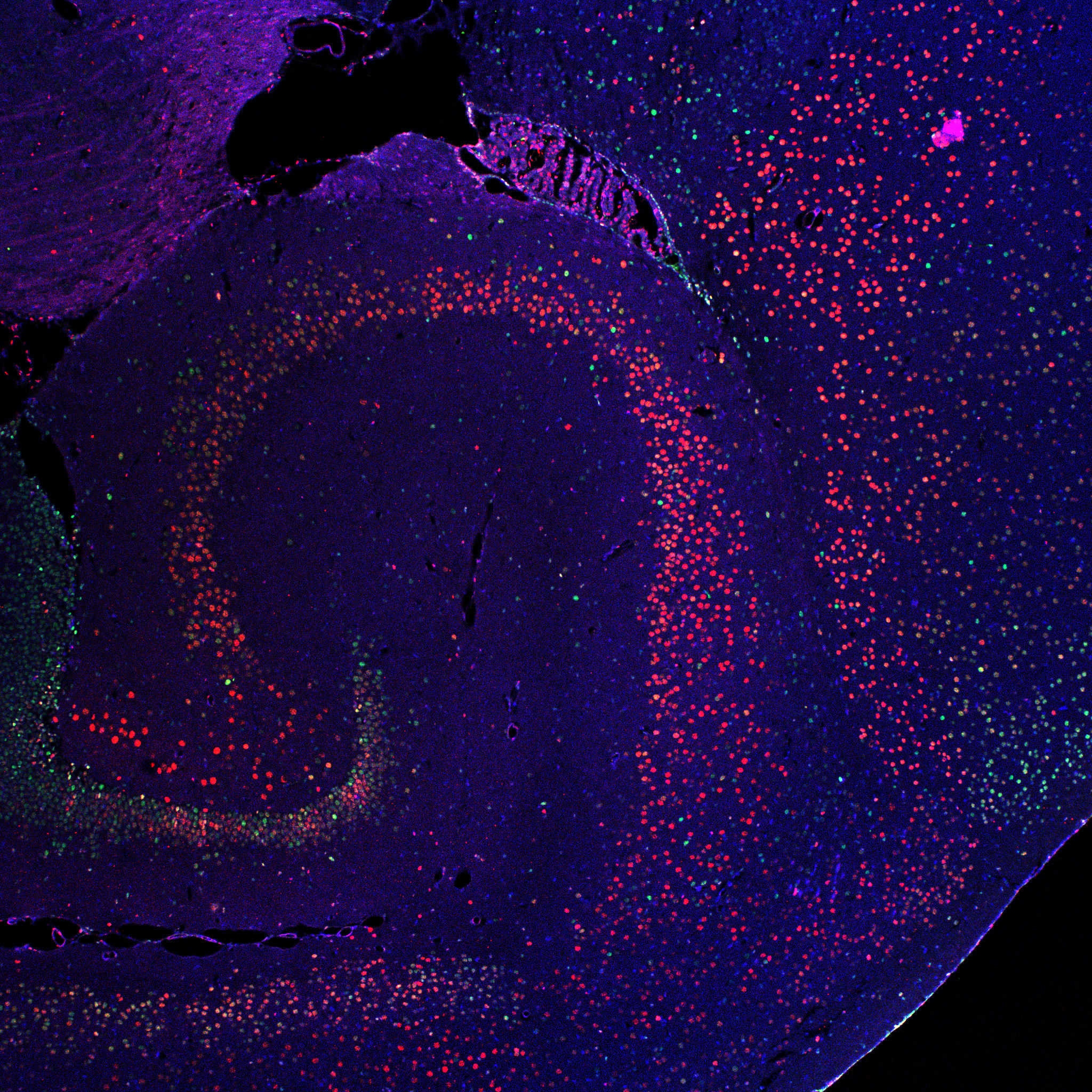

Supplement: Figure 1—source data 5. [file elife-86940-fig1-data5.zip › Figure 1-source data 5/3361-CON-f+-1M-SAGITAL-5X-CI-CII-1-vHPC-Image Export-05.tif]

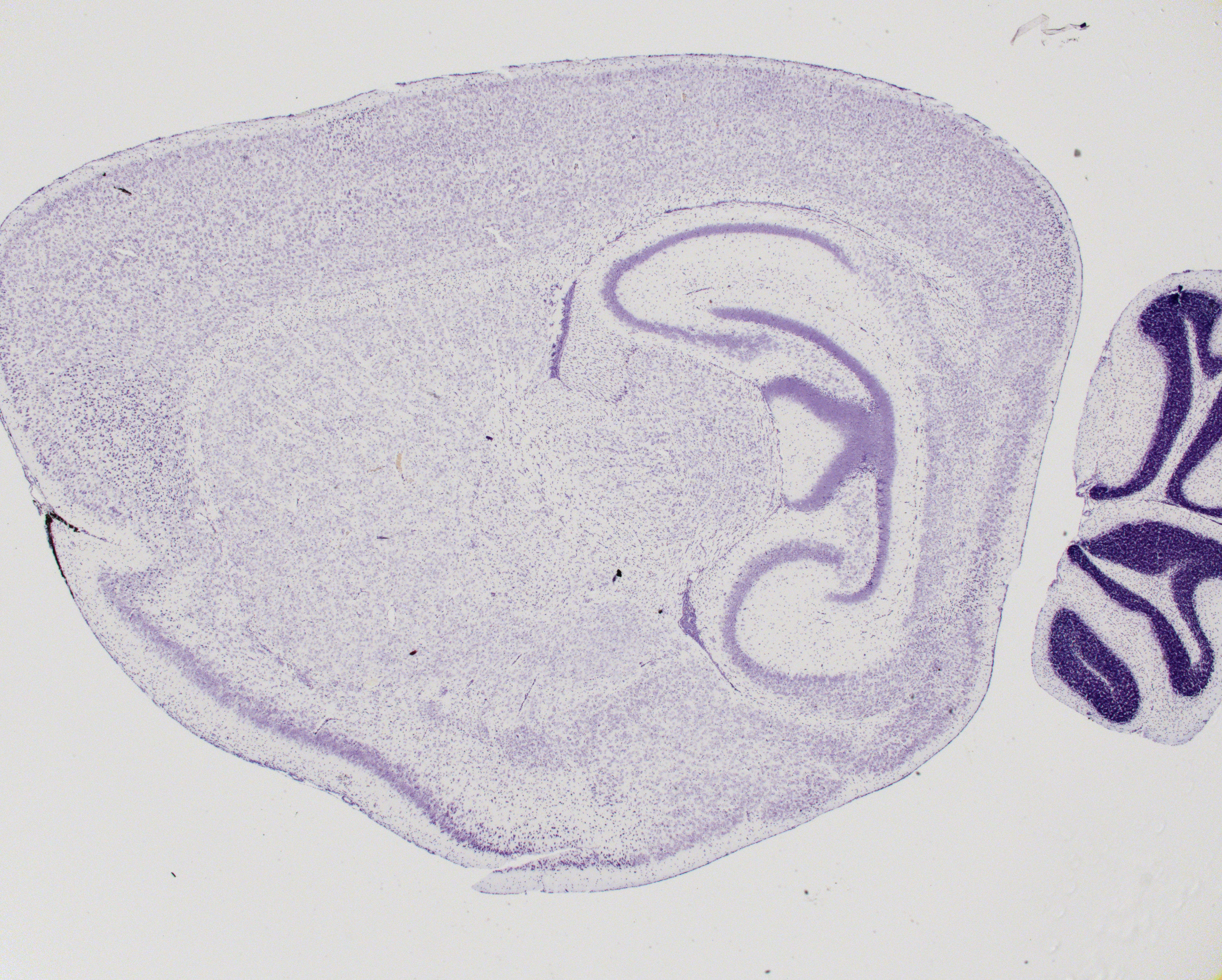

Supplement: Figure 1—source data 5. [file elife-86940-fig1-data5.zip › Figure 1-source data 5/3736-CII F+ CON-2.5X-1M-SAGITAL-26-2-Image Export-02.tif]

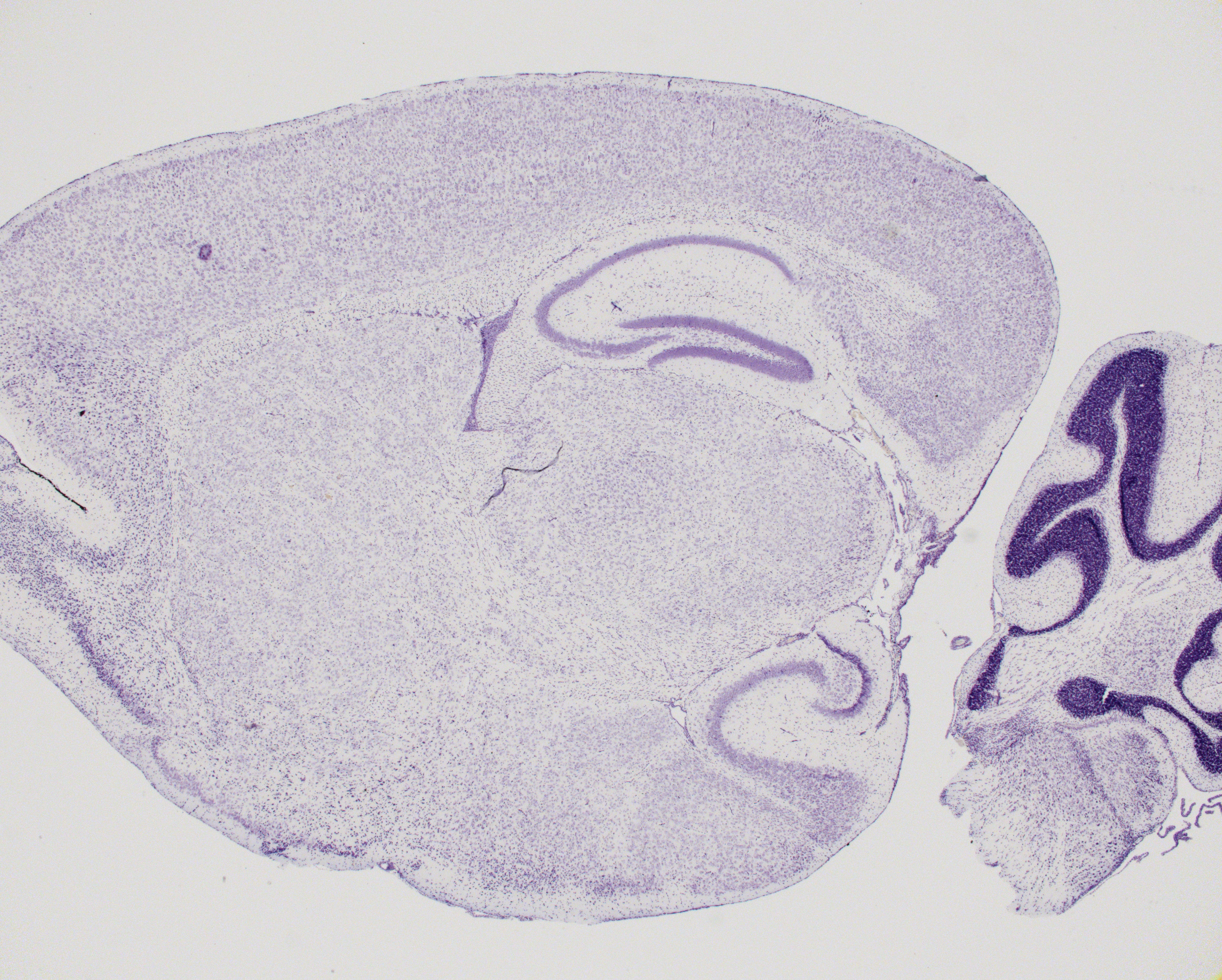

Supplement: Figure 1—source data 5. [file elife-86940-fig1-data5.zip › Figure 1-source data 5/3736-CII F+ CON-2.5X-1M-SAGITAL-34-2-Image Export-03.tif]

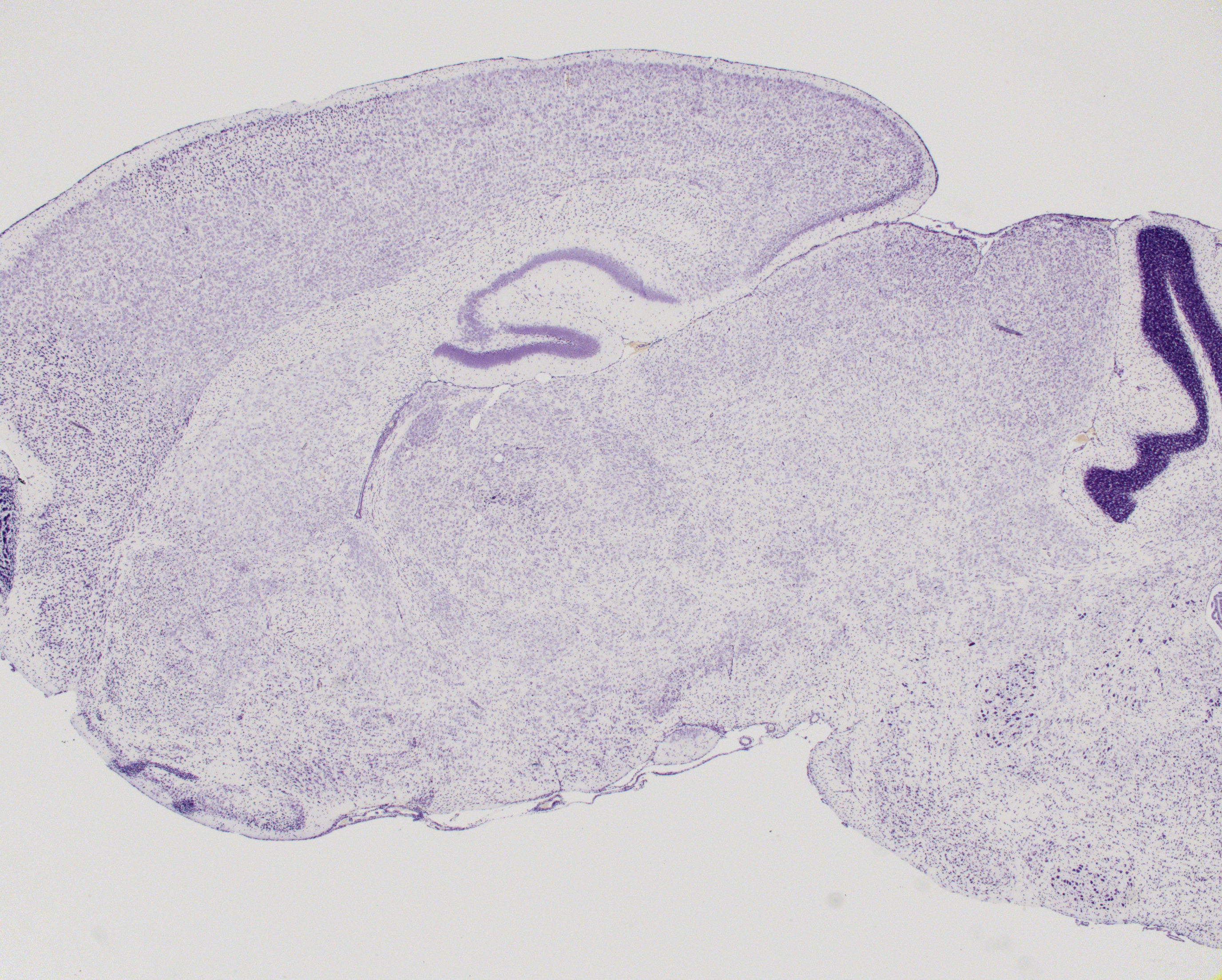

Supplement: Figure 1—source data 5. [file elife-86940-fig1-data5.zip › Figure 1-source data 5/3736-CII F+ CON-2.5X-1M-SAGITAL-50-1-Image Export-05.tif]

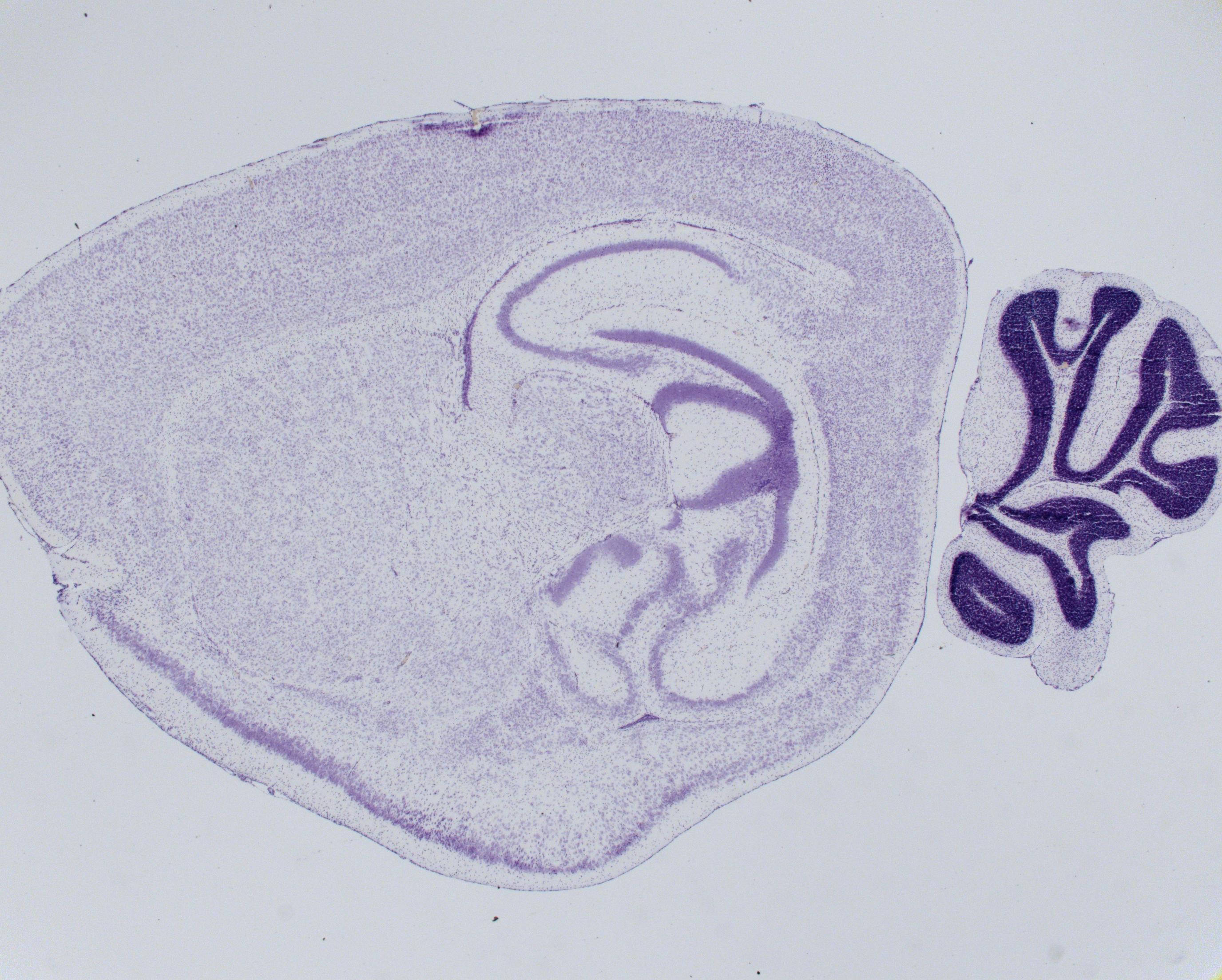

Supplement: Figure 1—source data 5. [file elife-86940-fig1-data5.zip › Figure 1-source data 5/3737-RX CII CKO-2.5X-1M-SAGITAL-26-2-Image Export-02.tif]

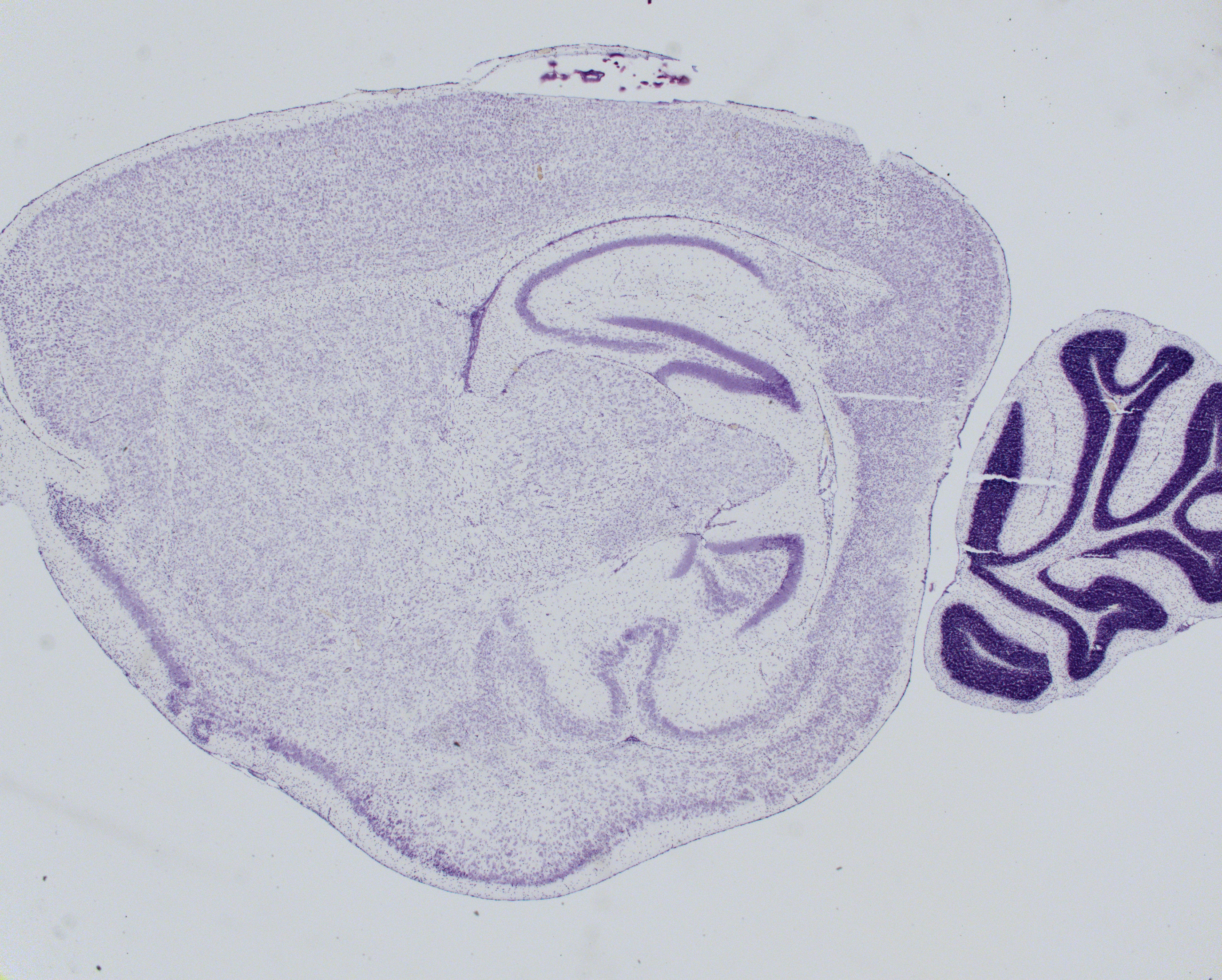

Supplement: Figure 1—source data 6. [file elife-86940-fig1-data6.zip › Figure 1-source data 6/3737-RX CII CKO-2.5X-1M-SAGITAL-34-4-Image Export-03.tif]

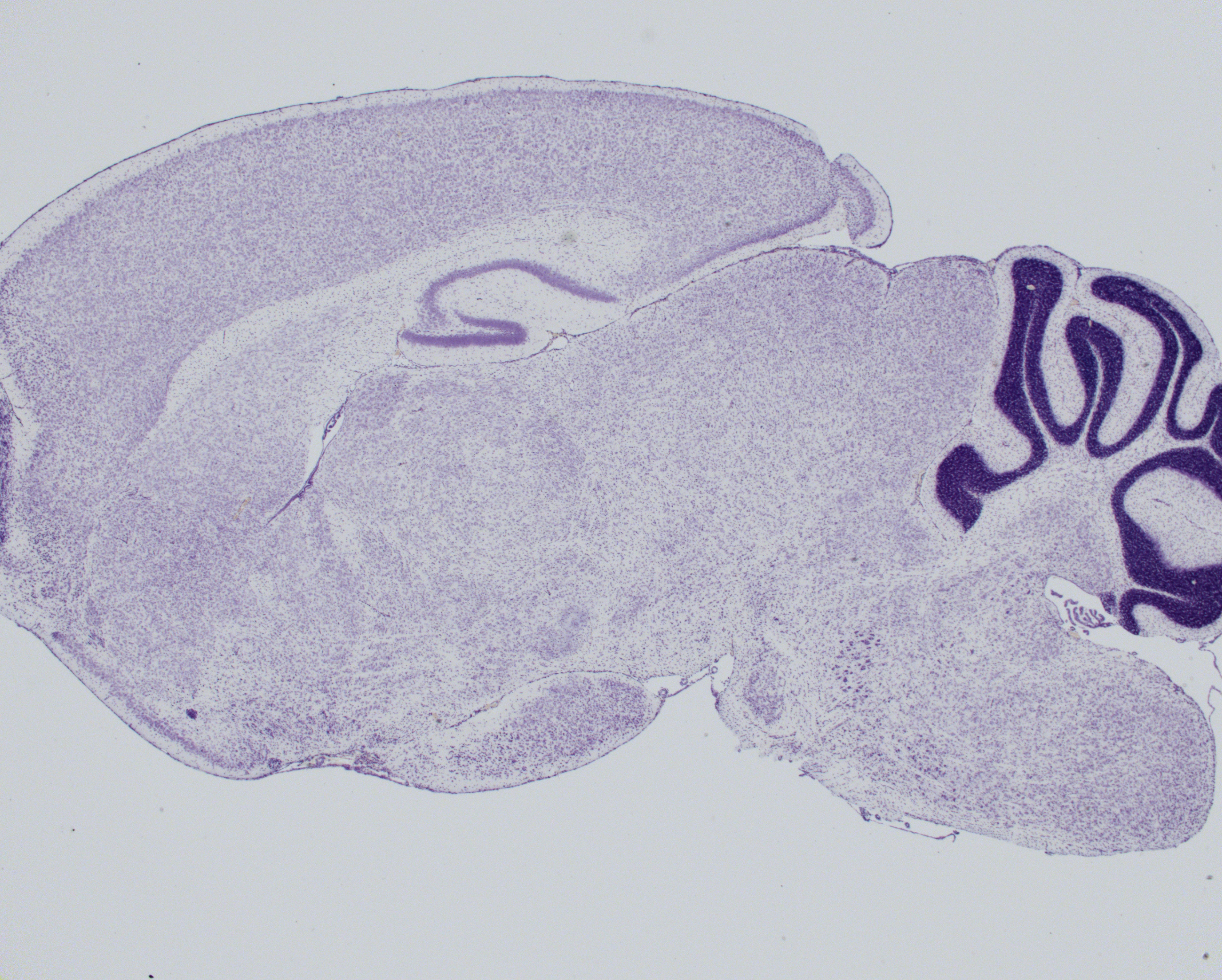

Supplement: Figure 1—source data 6. [file elife-86940-fig1-data6.zip › Figure 1-source data 6/3737-RX CII CKO-2.5X-1M-SAGITAL-50-2-Image Export-05.tif]

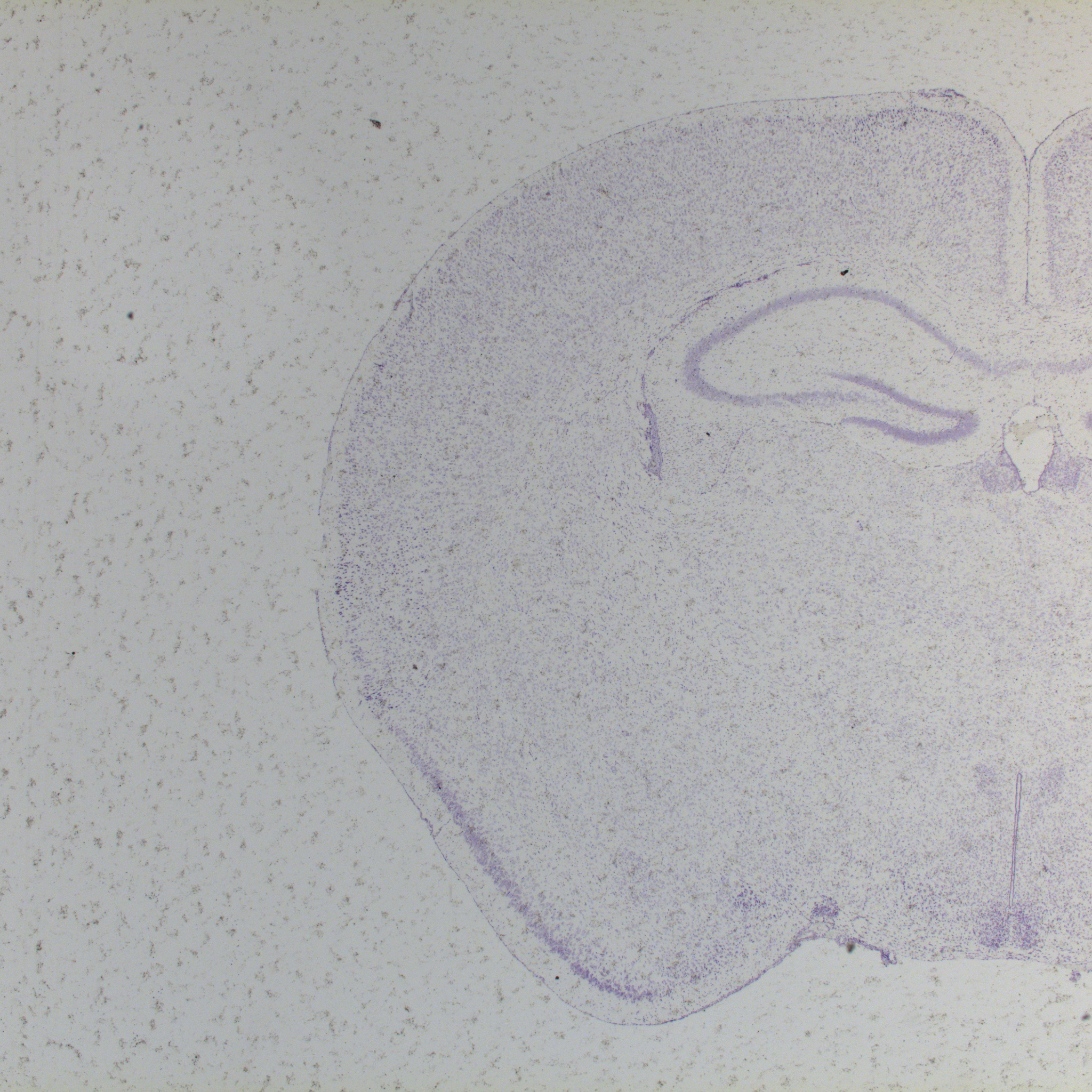

Supplement: Figure 1—source data 6. [file elife-86940-fig1-data6.zip › Figure 1-source data 6/3773-CON-2.5X-RX CII F+-1M-#59-1-Image Export-02.tif]

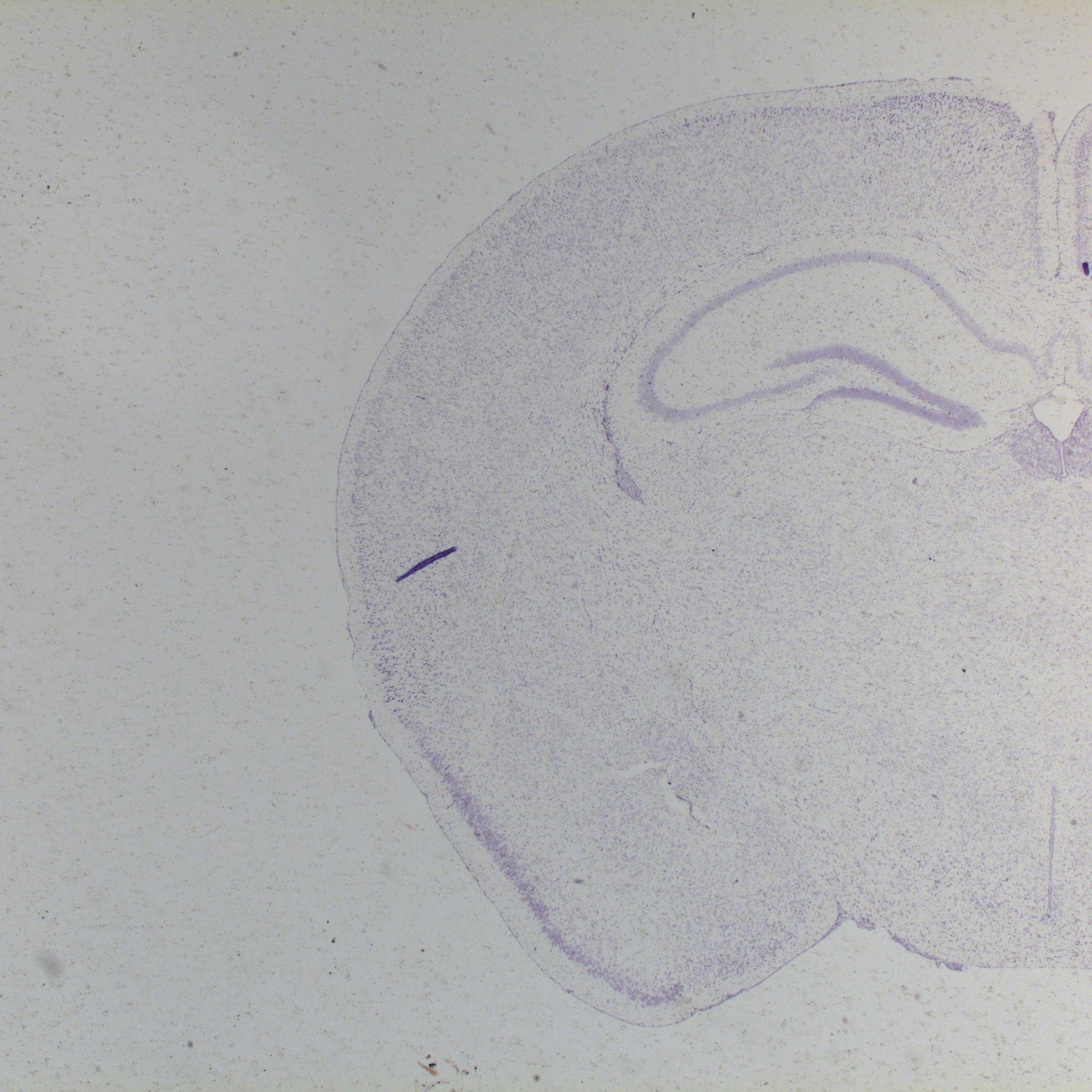

Supplement: Figure 1—source data 6. [file elife-86940-fig1-data6.zip › Figure 1-source data 6/3773-CON-2.5X-RX CII F+-1M-#69-2-Image Export-03.tif]

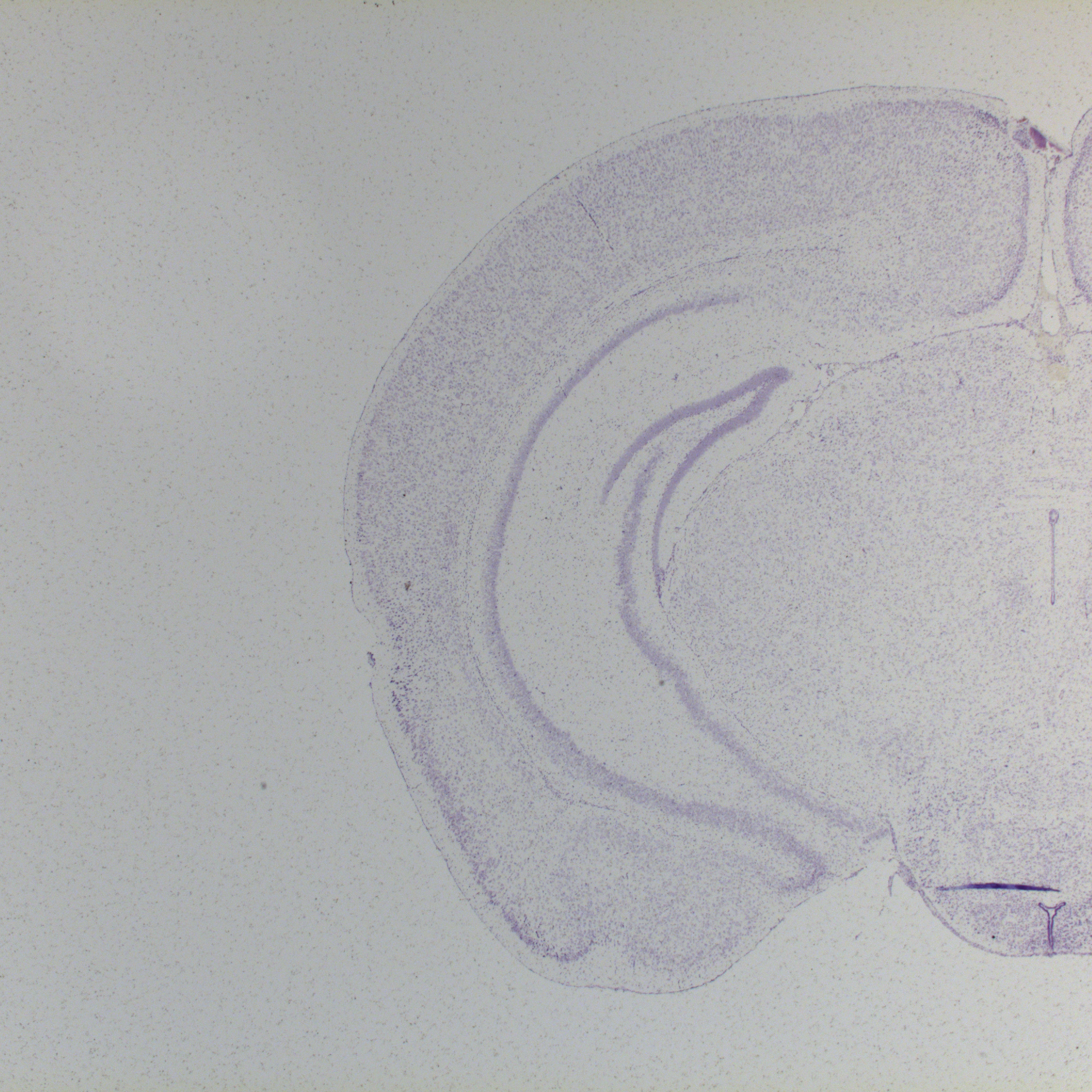

Supplement: Figure 1—source data 6. [file elife-86940-fig1-data6.zip › Figure 1-source data 6/3773-CON-2.5X-RX CII F+-1M-#99-2-Image Export-06.tif]

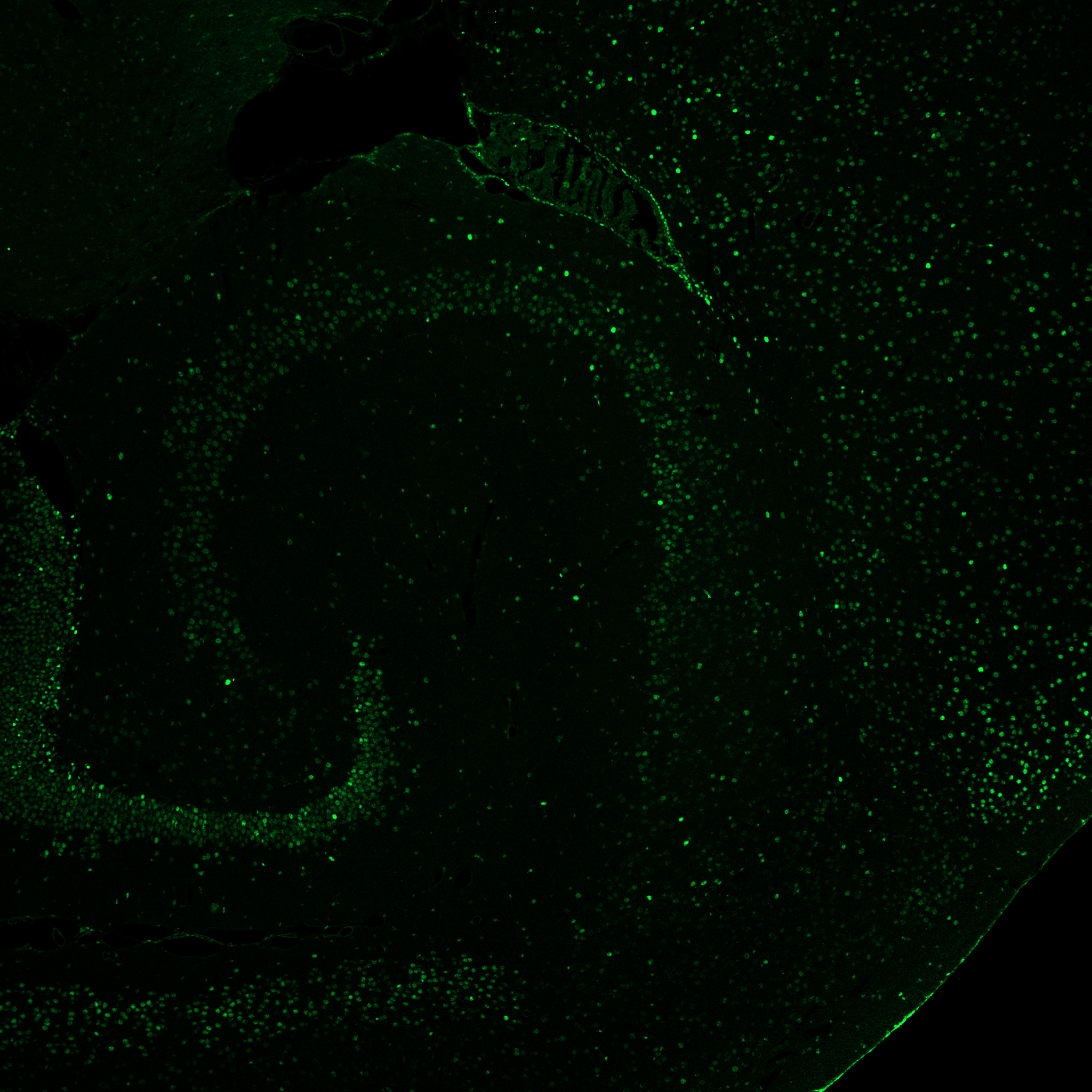

Supplement: Figure 1—source data 6. [file elife-86940-fig1-data6.zip › Figure 1-source data 6/3361-CON-f+-1M-SAGITAL-5X-CI-CII-1-vHPC-Image Export-05_AF488-T2.tif]

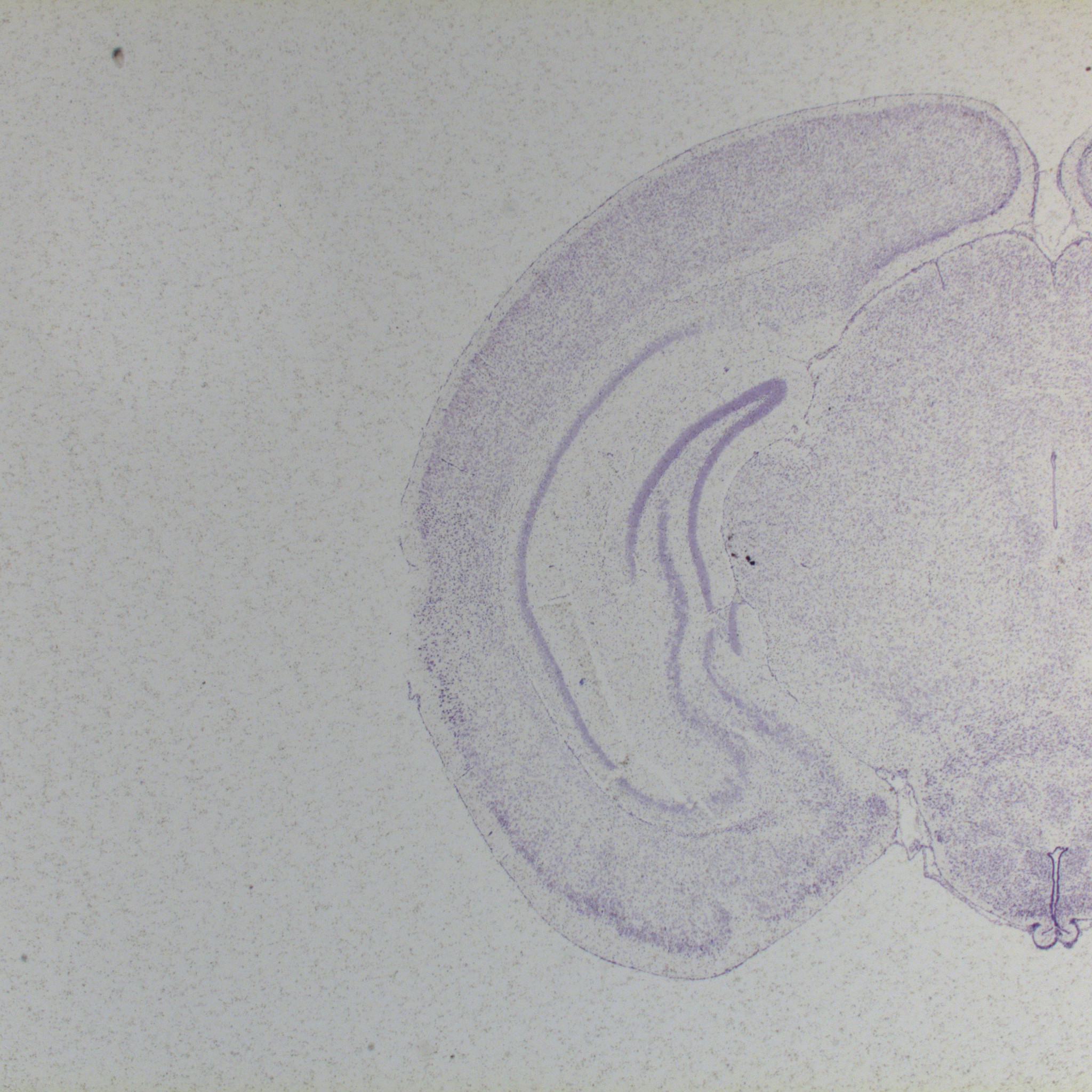

Supplement: Figure 1—source data 6. [file elife-86940-fig1-data6.zip › Figure 1-source data 6/3775-CII CKO-2.5X-RX CII FF-1M-#91-2-Image Export-14.tif]

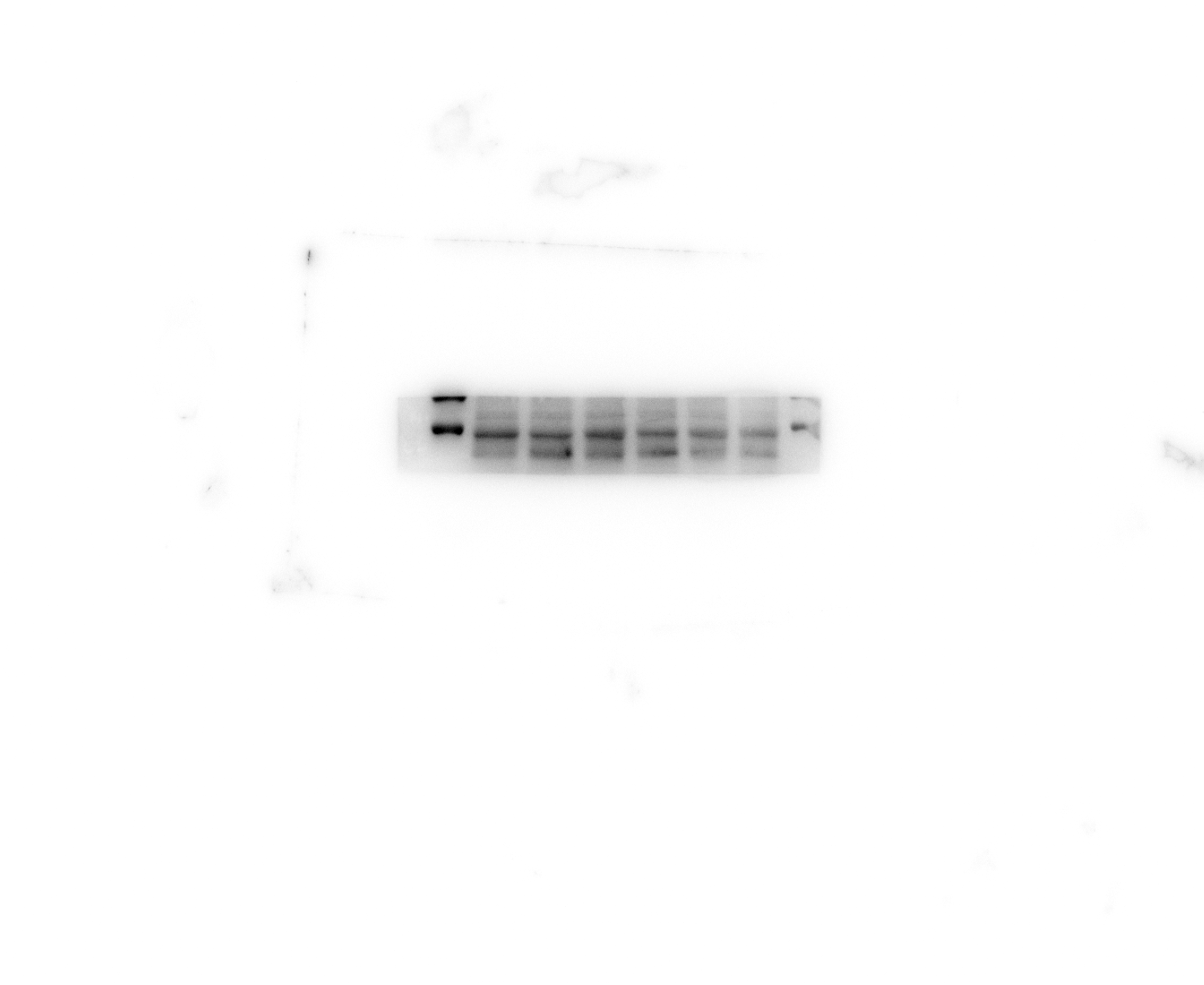

Supplement: Figure 1—source data 6. [file elife-86940-fig1-data6.zip › Figure 1-source data 6/COUP-TFI.Tif]

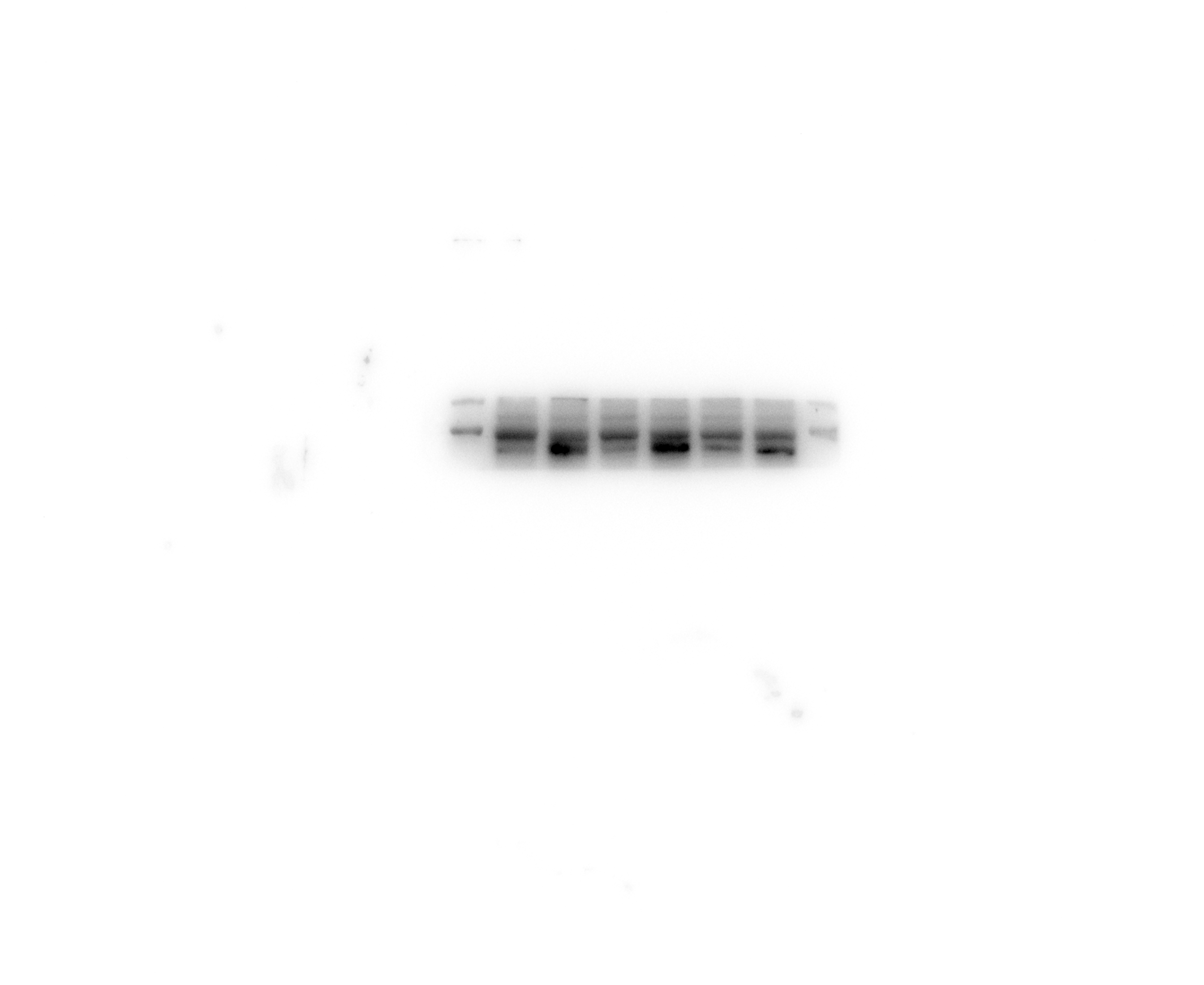

Supplement: Figure 1—source data 6. [file elife-86940-fig1-data6.zip › Figure 1-source data 6/COUP-TFII.Tif]

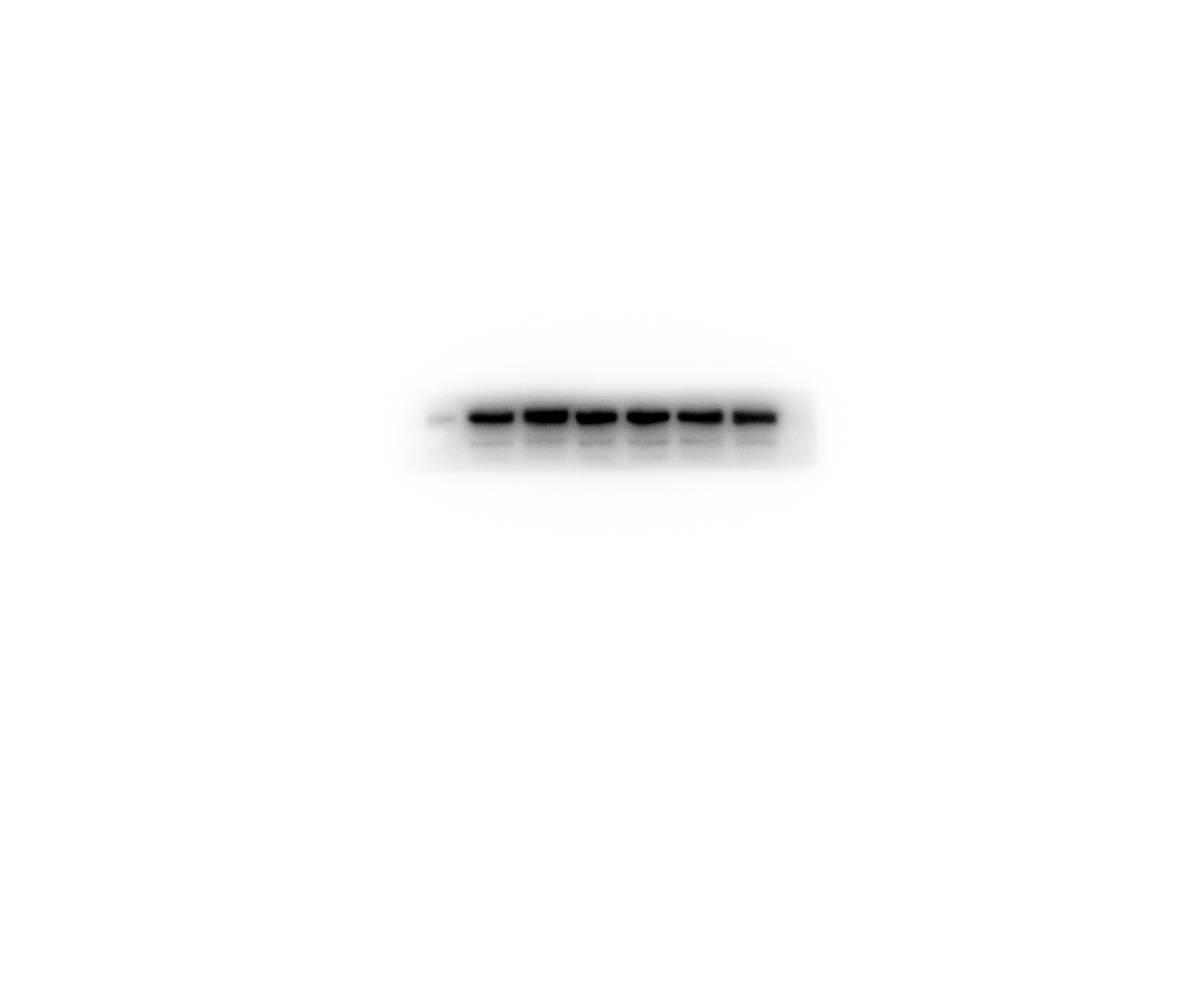

Supplement: Figure 1—source data 6. [file elife-86940-fig1-data6.zip › Figure 1-source data 6/GAPDH.Tif]

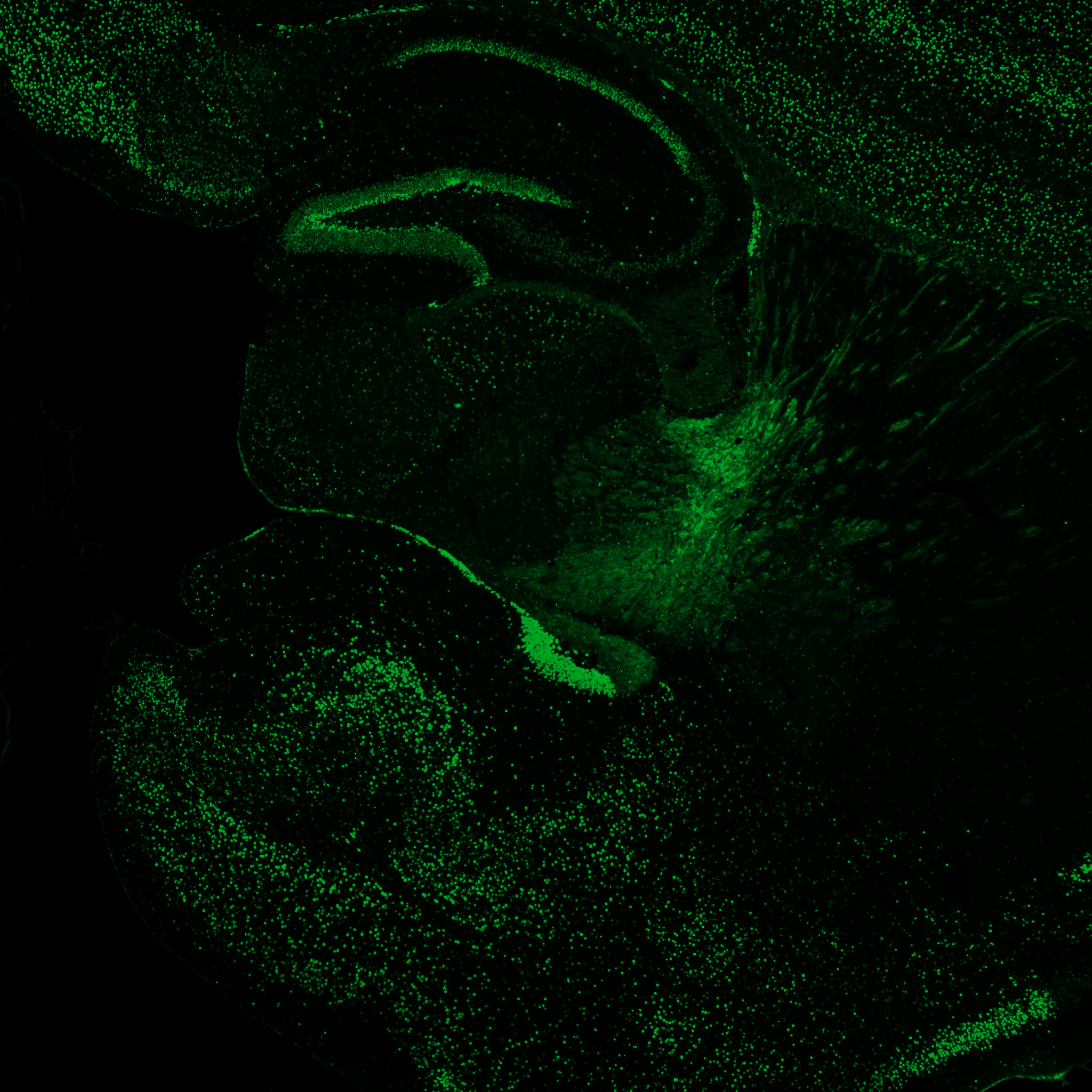

Supplement: Figure 1—figure supplement 1—source data 1. [file elife-86940-fig1-figsupp1-data1.zip › Figure 1-figure supplement 1-source data 1/2879-CKO-1M-RX CII FF-2.5X-SAGITAL-CI-CII-2-Image Export-01_AF488.tif]

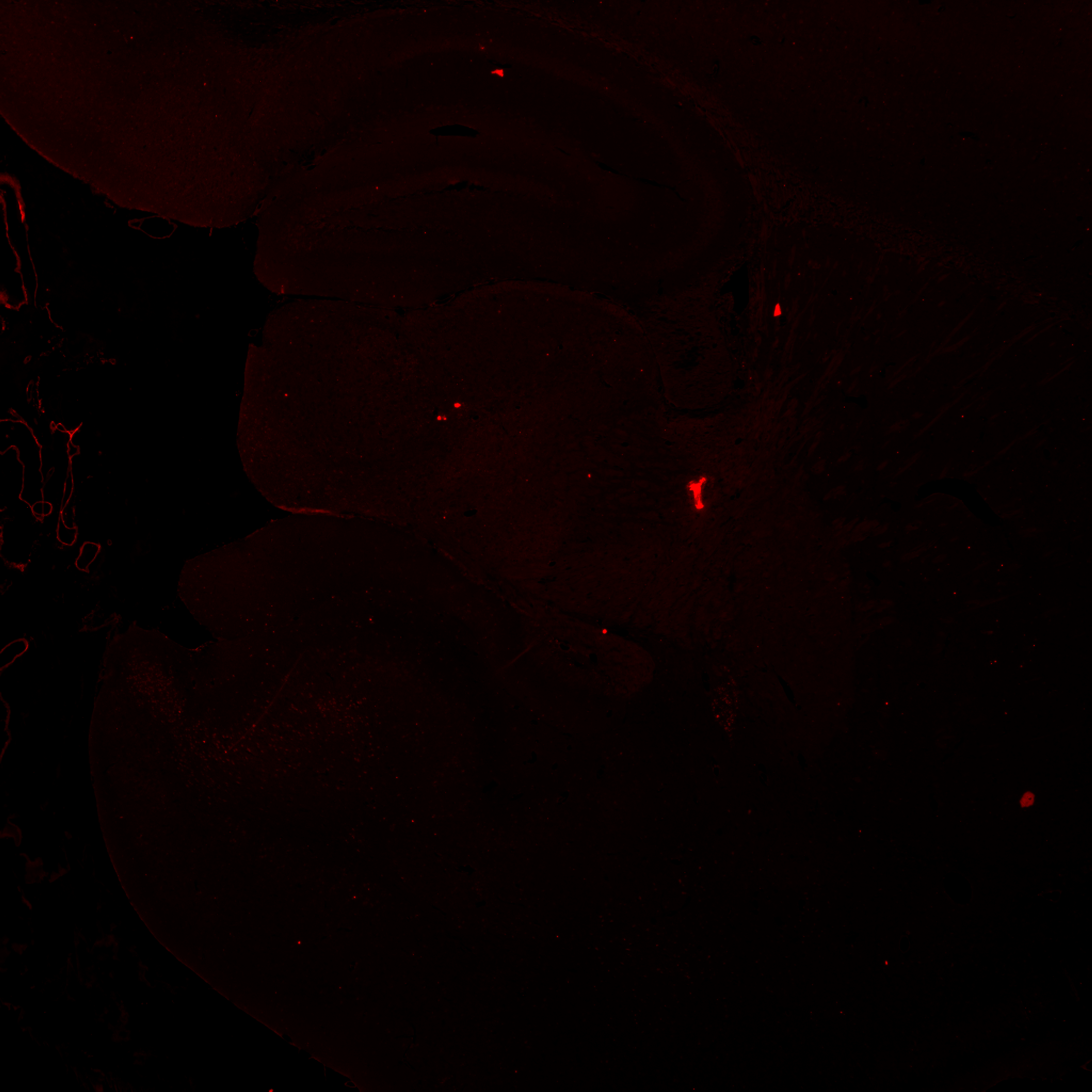

Supplement: Figure 1—figure supplement 1—source data 1. [file elife-86940-fig1-figsupp1-data1.zip › Figure 1-figure supplement 1-source data 1/2879-CKO-1M-RX CII FF-2.5X-SAGITAL-CI-CII-2-Image Export-01_AF594.tif]

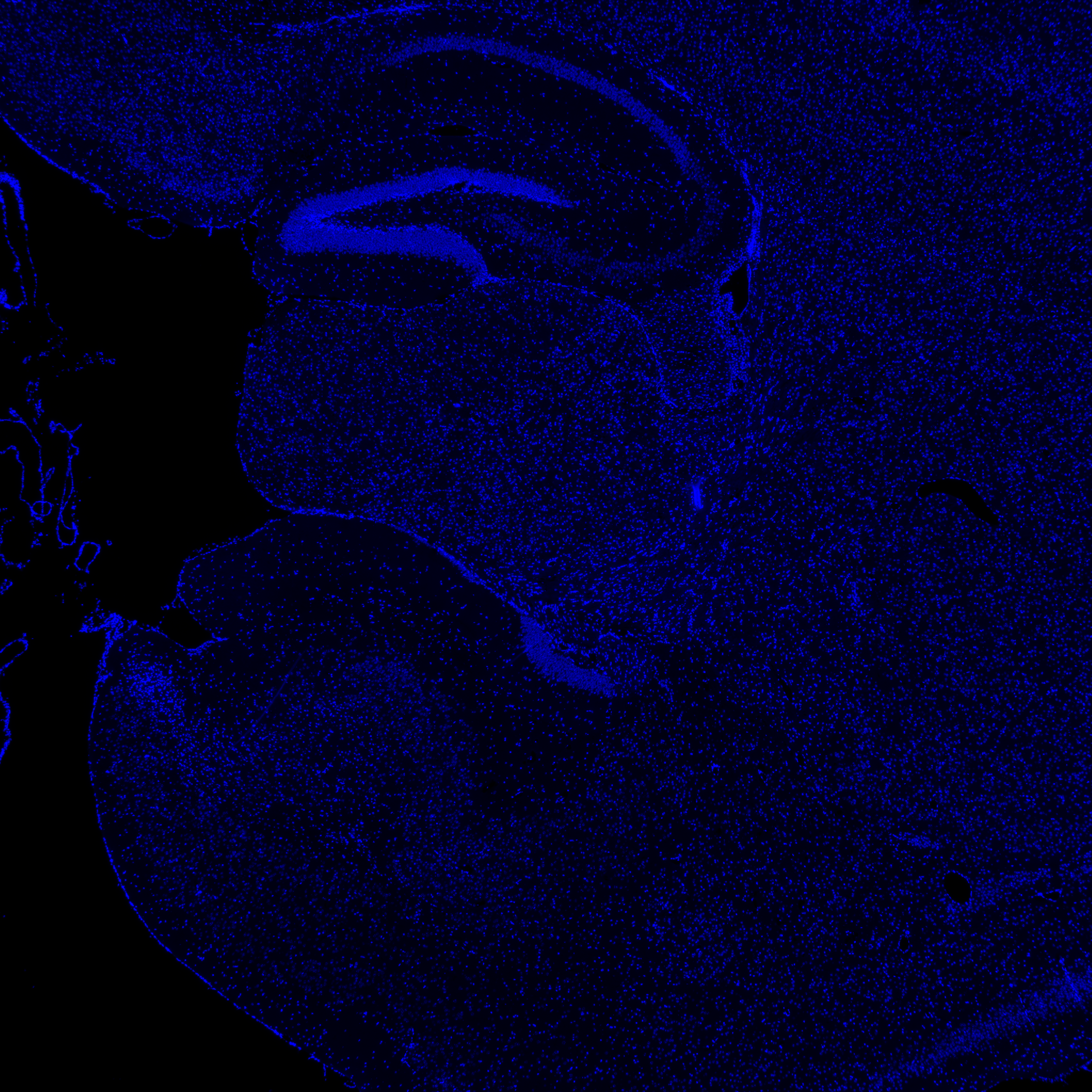

Supplement: Figure 1—figure supplement 1—source data 1. [file elife-86940-fig1-figsupp1-data1.zip › Figure 1-figure supplement 1-source data 1/2879-CKO-1M-RX CII FF-2.5X-SAGITAL-CI-CII-2-Image Export-01_DAPI.tif]

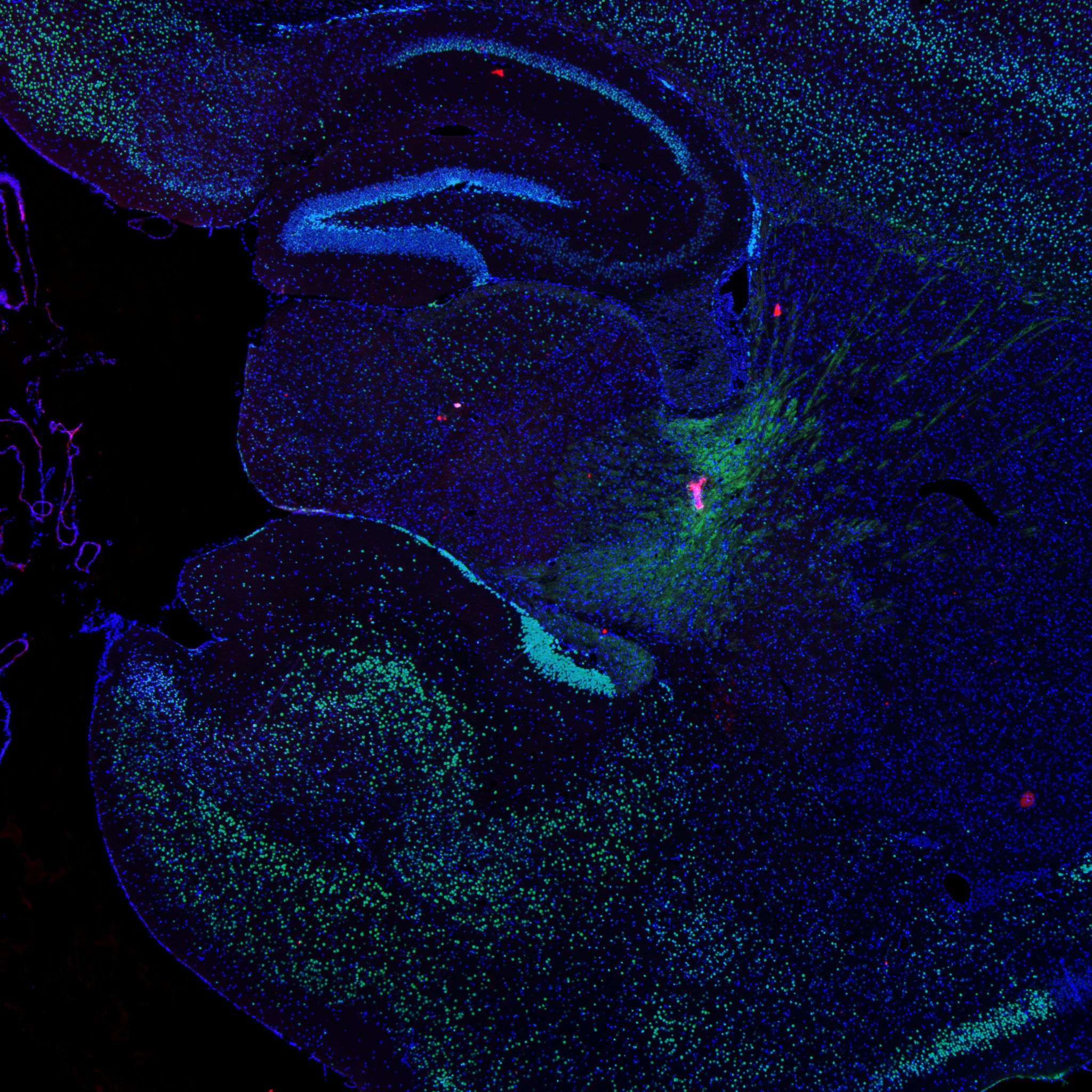

Supplement: Figure 1—figure supplement 1—source data 1. [file elife-86940-fig1-figsupp1-data1.zip › Figure 1-figure supplement 1-source data 1/2879-CKO-1M-RX CII FF-2.5X-SAGITAL-CI-CII-2-Image Export-01.tif]

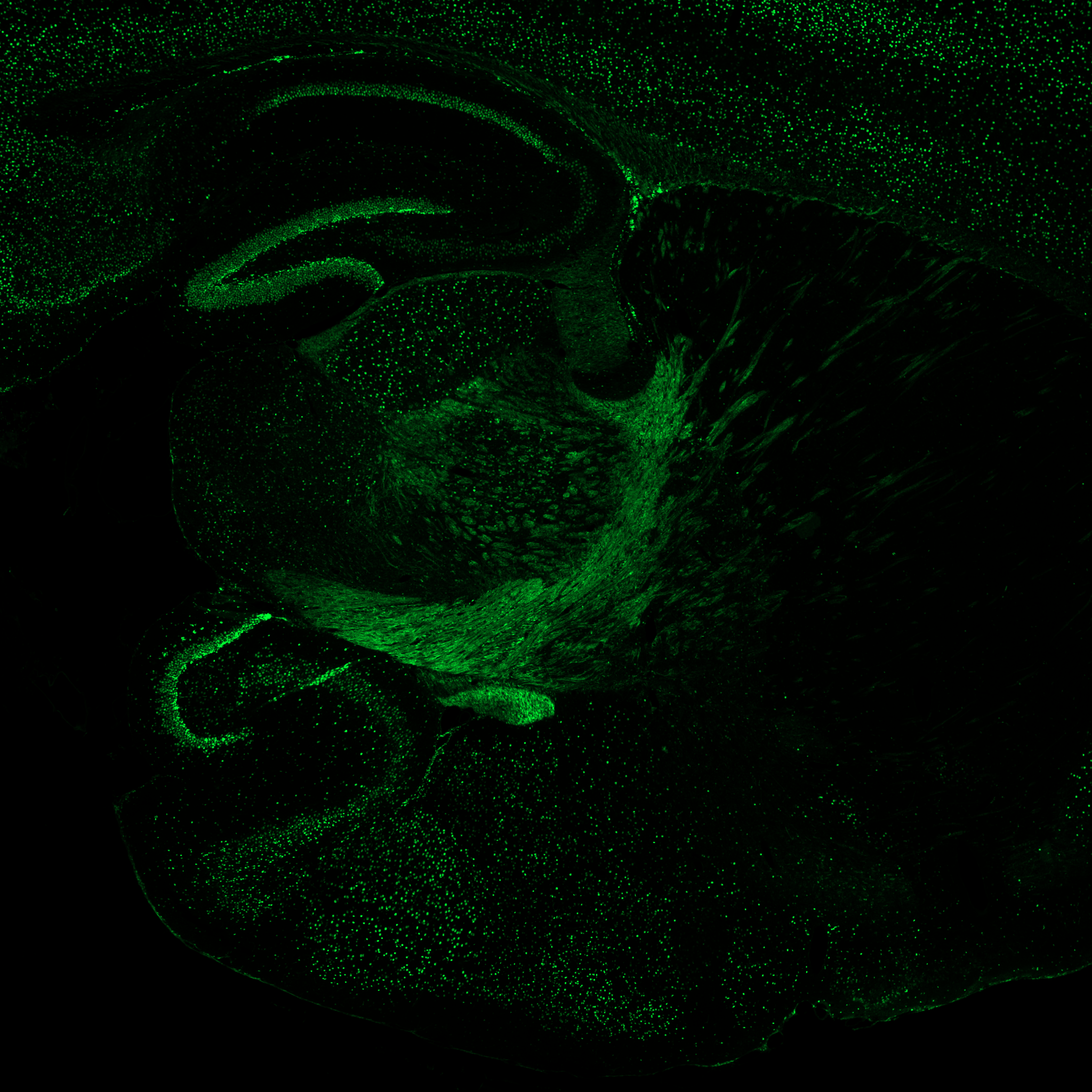

Supplement: Figure 1—figure supplement 1—source data 1. [file elife-86940-fig1-figsupp1-data1.zip › Figure 1-figure supplement 1-source data 1/2879-CON-1M-CII FF-2.5X-SAGITAL-CI-CII-2-Image Export-11_AF488.tif]

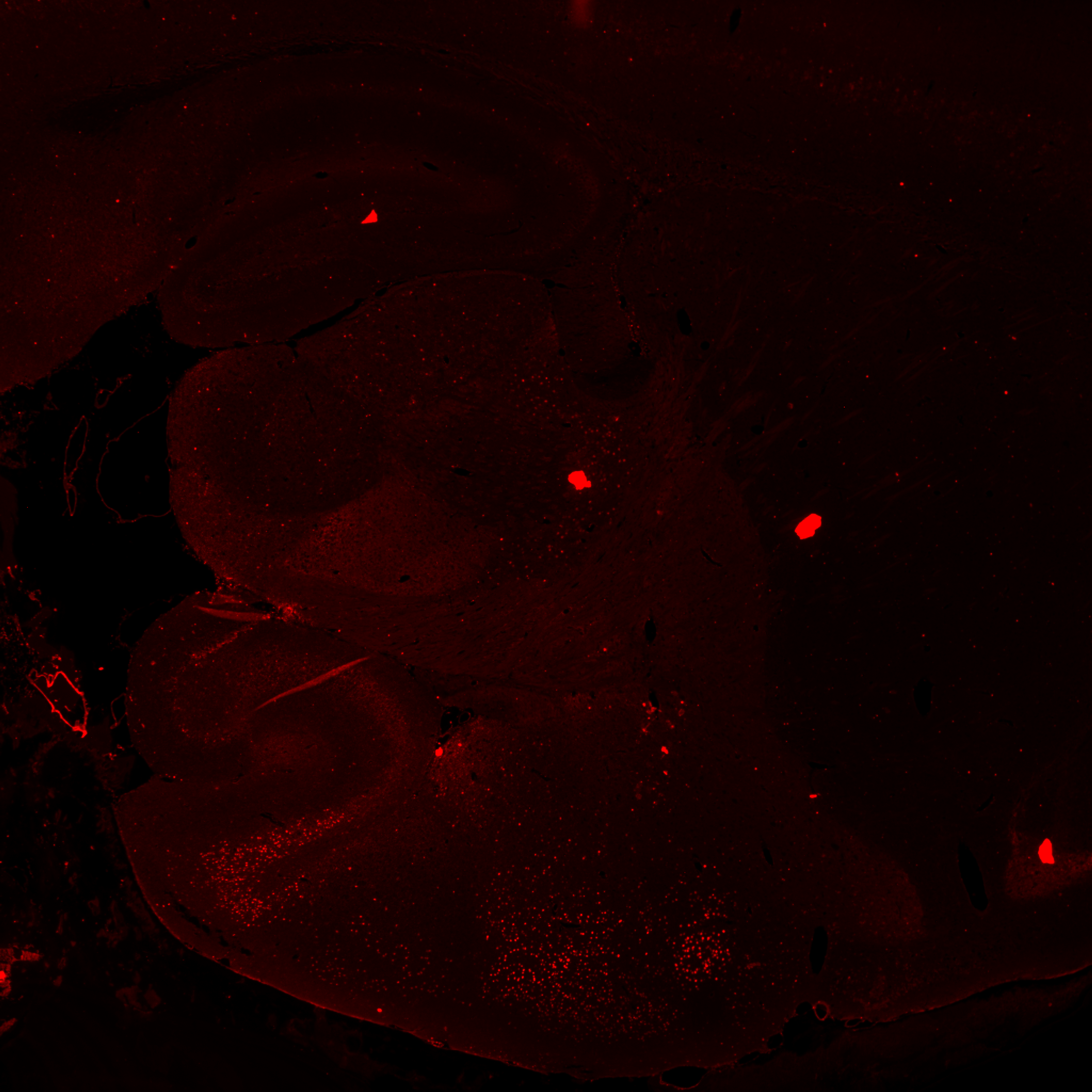

Supplement: Figure 1—figure supplement 1—source data 1. [file elife-86940-fig1-figsupp1-data1.zip › Figure 1-figure supplement 1-source data 1/2879-CON-1M-CII FF-2.5X-SAGITAL-CI-CII-2-Image Export-11_AF594.tif]

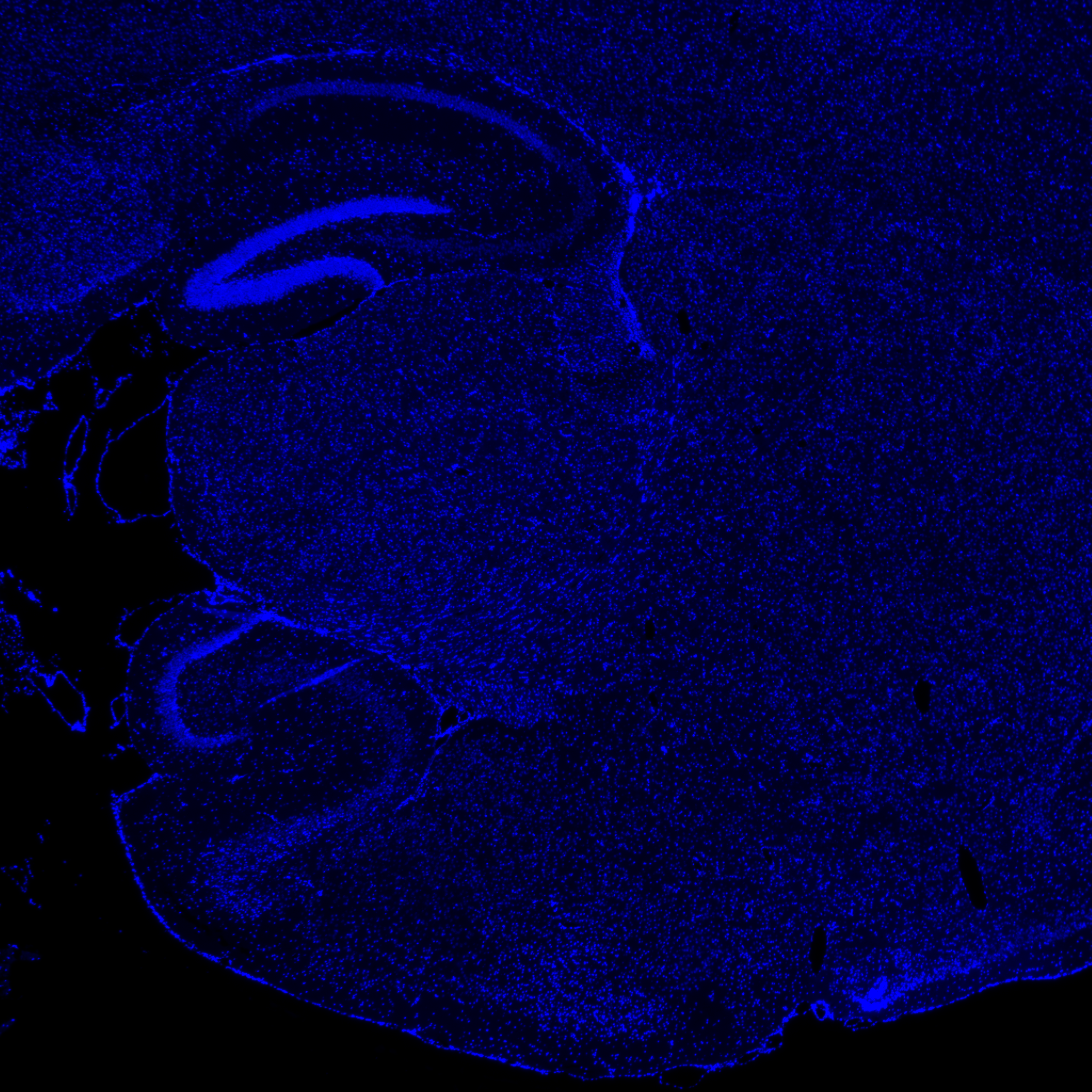

Supplement: Figure 1—figure supplement 1—source data 1. [file elife-86940-fig1-figsupp1-data1.zip › Figure 1-figure supplement 1-source data 1/2879-CON-1M-CII FF-2.5X-SAGITAL-CI-CII-2-Image Export-11_DAPI.tif]

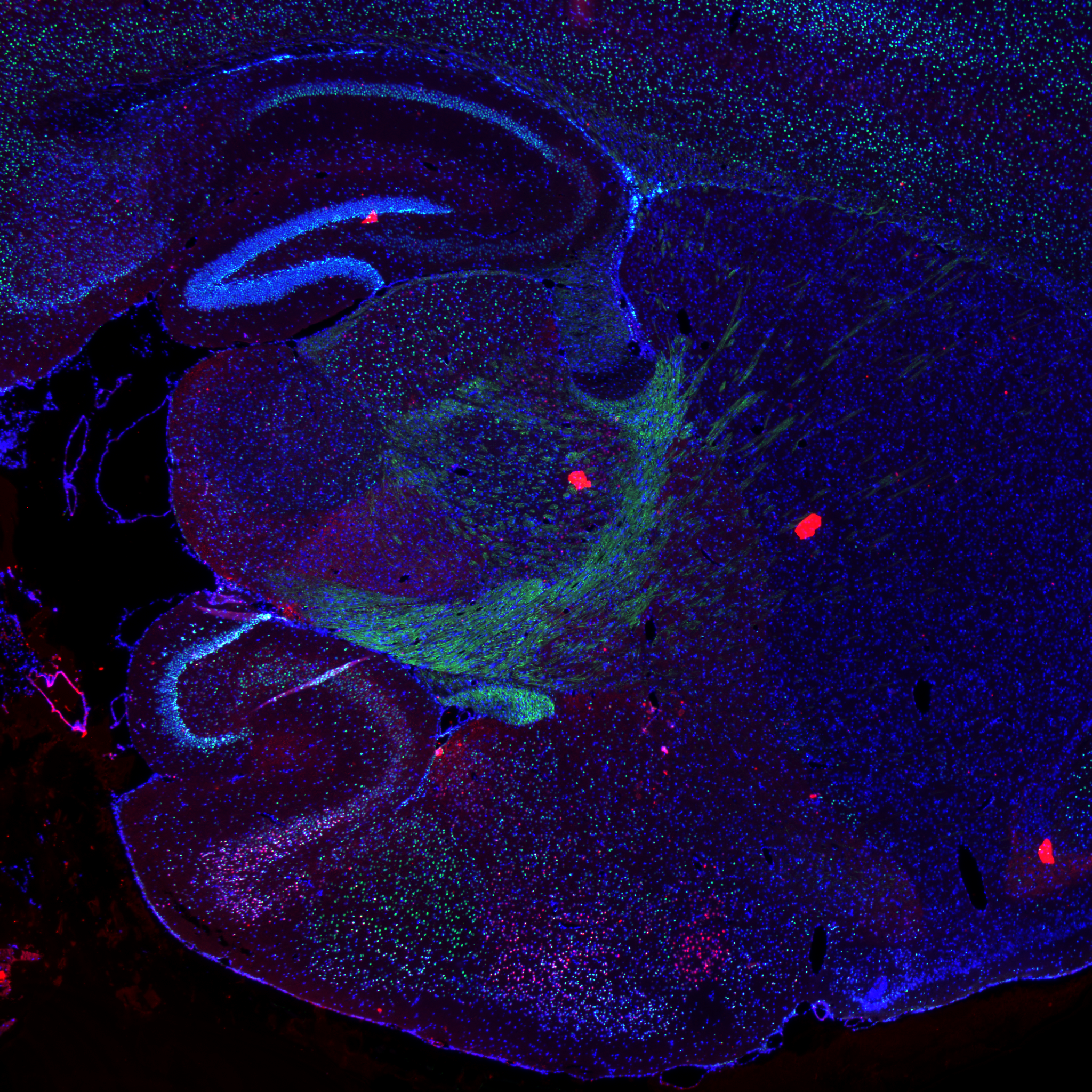

Supplement: Figure 1—figure supplement 1—source data 1. [file elife-86940-fig1-figsupp1-data1.zip › Figure 1-figure supplement 1-source data 1/2879-CON-1M-CII FF-2.5X-SAGITAL-CI-CII-2-Image Export-11.tif]

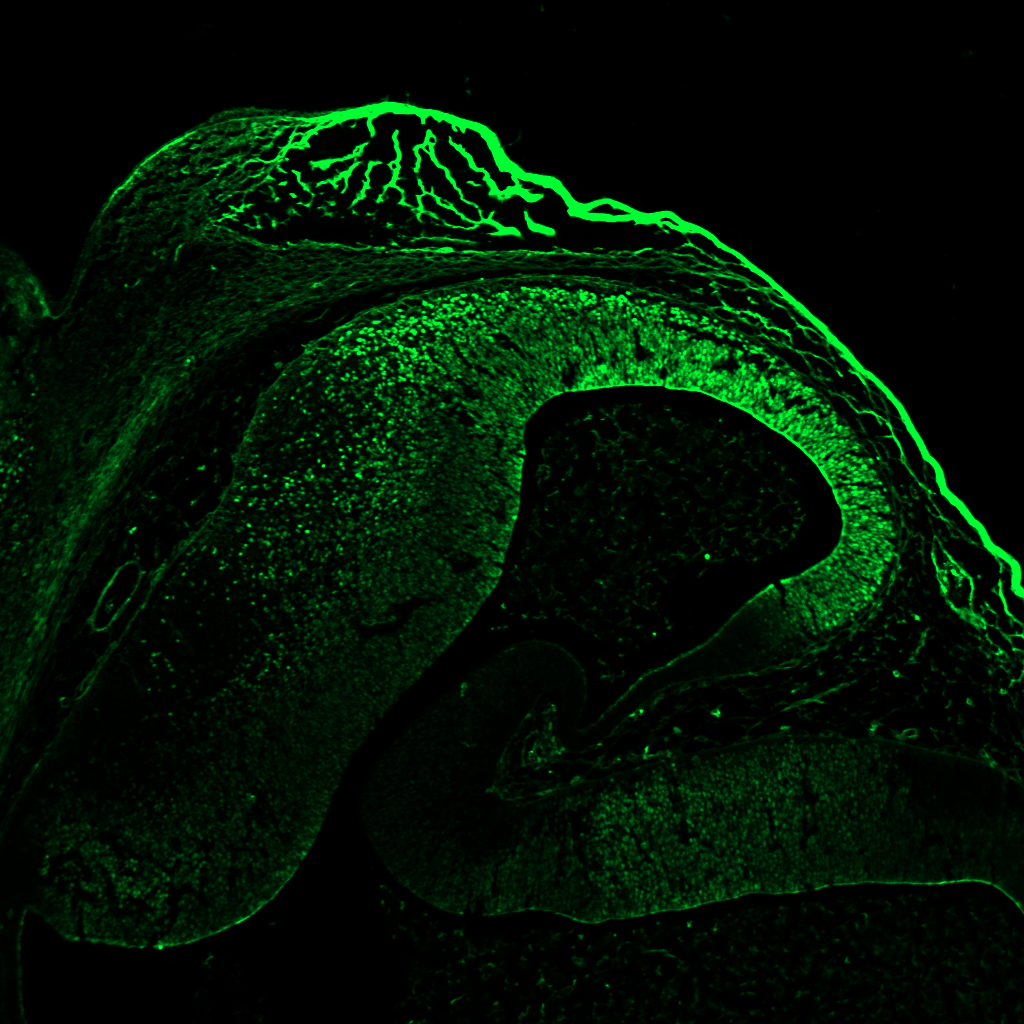

Supplement: Figure 1—figure supplement 1—source data 1. [file elife-86940-fig1-figsupp1-data1.zip › Figure 1-figure supplement 1-source data 1/C57 WT-COOL-2-E12.5-#30-4-5-CI-CII-left brain-Image Export-27_AF488-T2.tif]

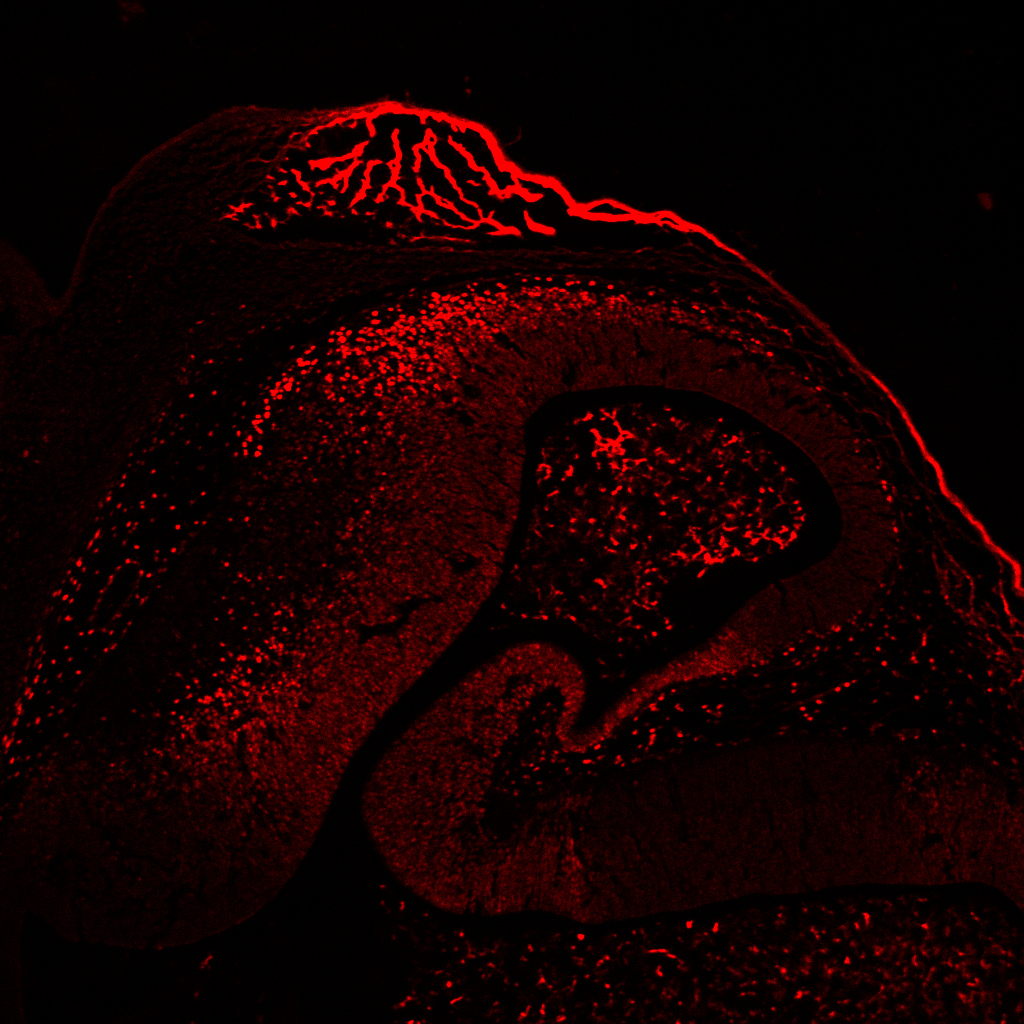

Supplement: Figure 1—figure supplement 1—source data 1. [file elife-86940-fig1-figsupp1-data1.zip › Figure 1-figure supplement 1-source data 1/C57 WT-COOL-2-E12.5-#30-4-5X-CI-CII-left brain-Image Export-27_AF594-T1.tif]

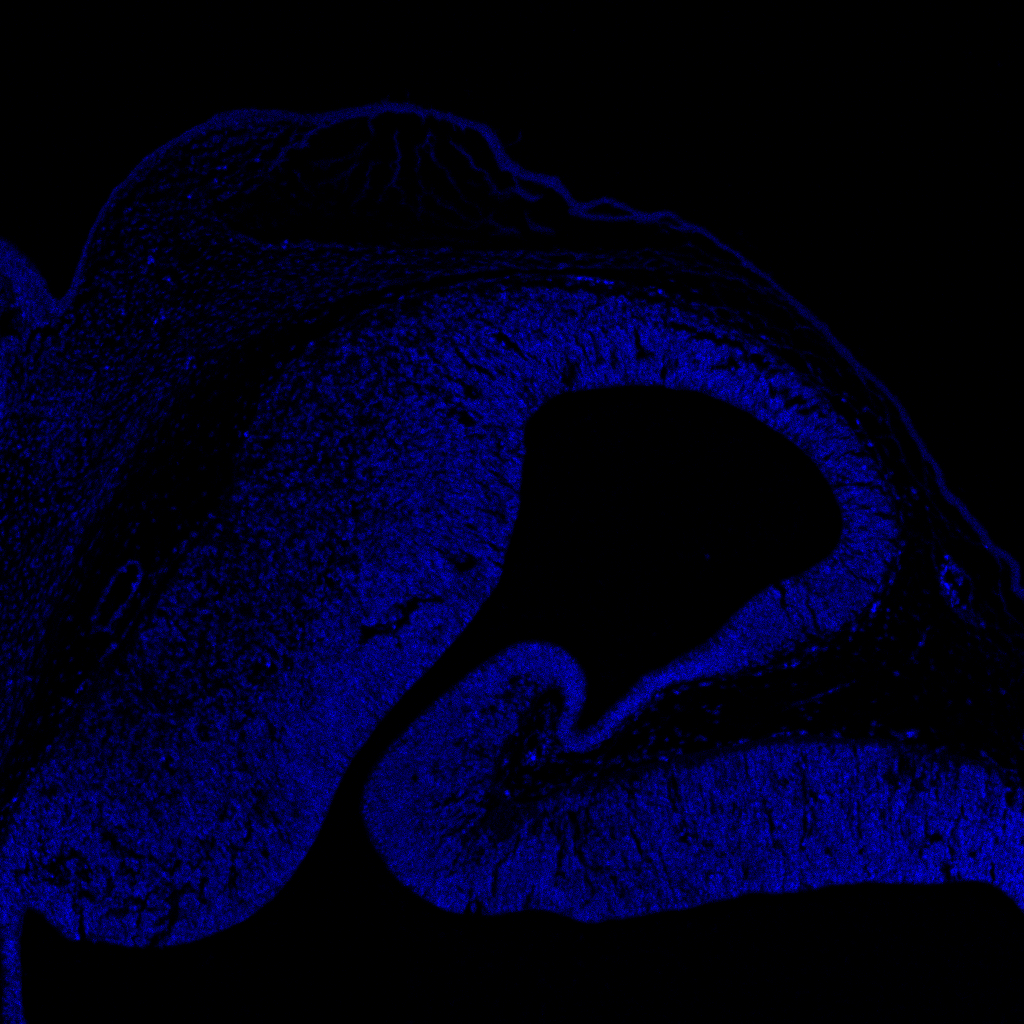

Supplement: Figure 1—figure supplement 1—source data 1. [file elife-86940-fig1-figsupp1-data1.zip › Figure 1-figure supplement 1-source data 1/C57 WT-COOL-2-E12.5-#30-4-5X-CI-CII-left brain-Image Export-27_DAPI-T3.tif]

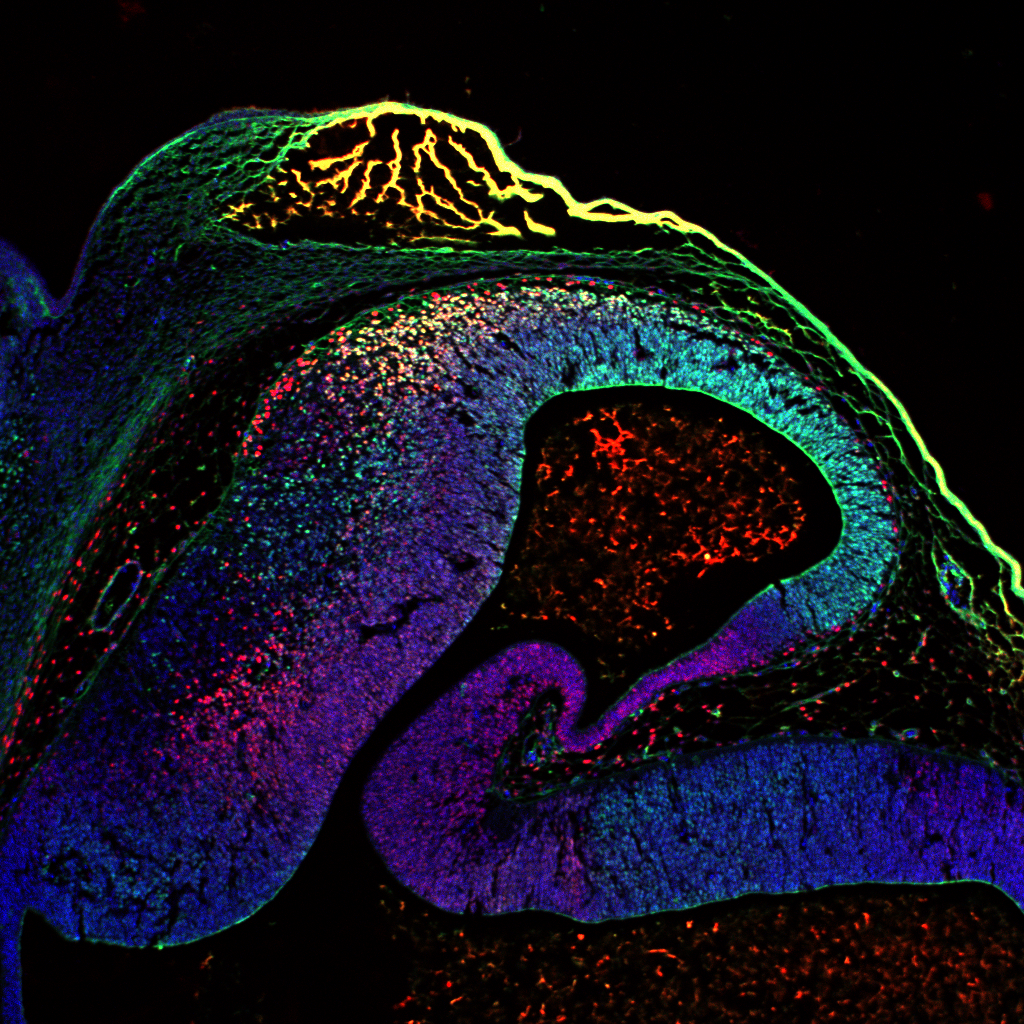

Supplement: Figure 1—figure supplement 1—source data 1. [file elife-86940-fig1-figsupp1-data1.zip › Figure 1-figure supplement 1-source data 1/C57 WT-COOL-2-E12.5-#30-4-5X-CI-CII-left brain-Image Export-27.tif]

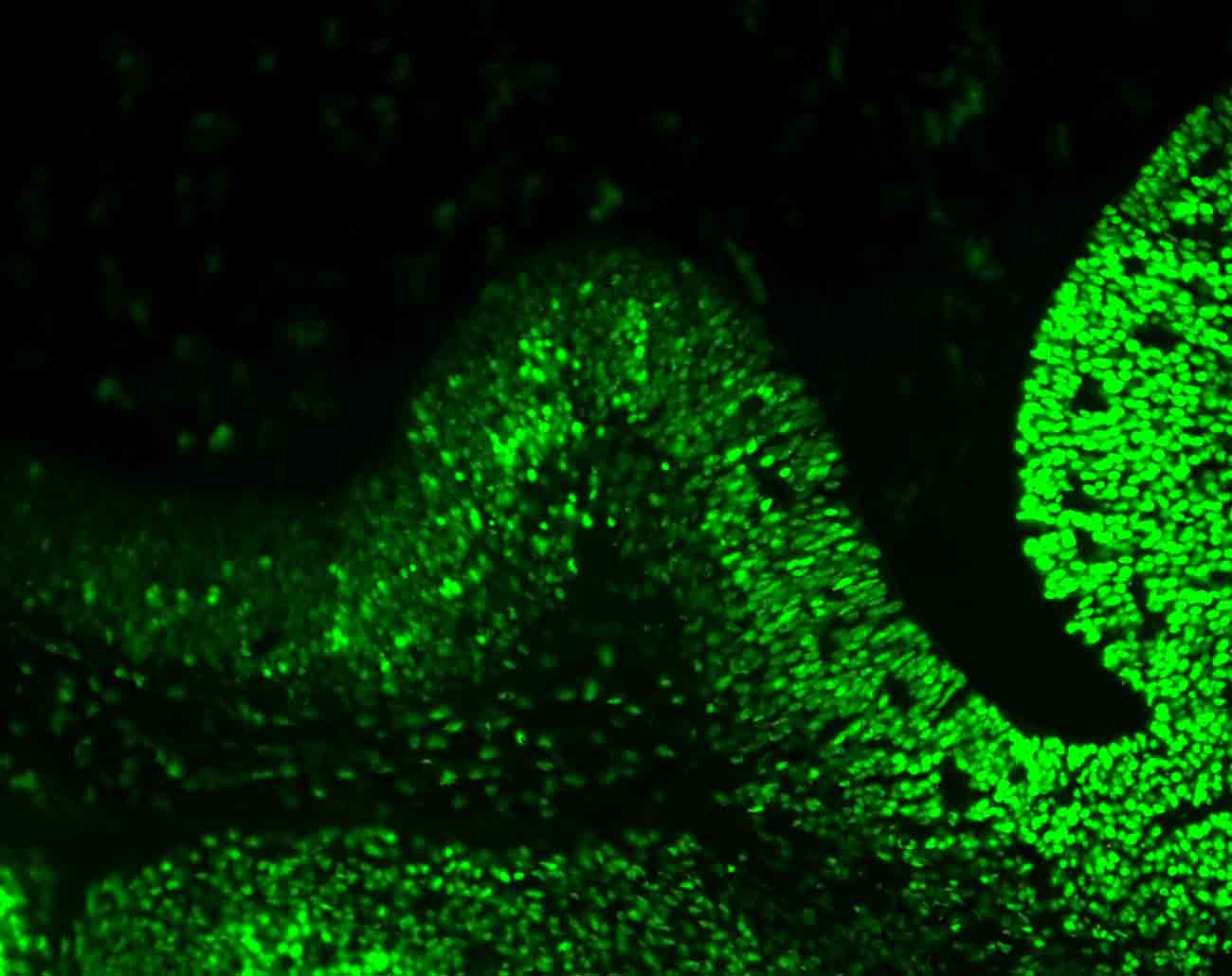

Supplement: Figure 1—figure supplement 1—source data 1. [file elife-86940-fig1-figsupp1-data1.zip › Figure 1-figure supplement 1-source data 1/F1098-1-E11-MUT-CI-LACZ-063X-20X-G10-G.jpg]

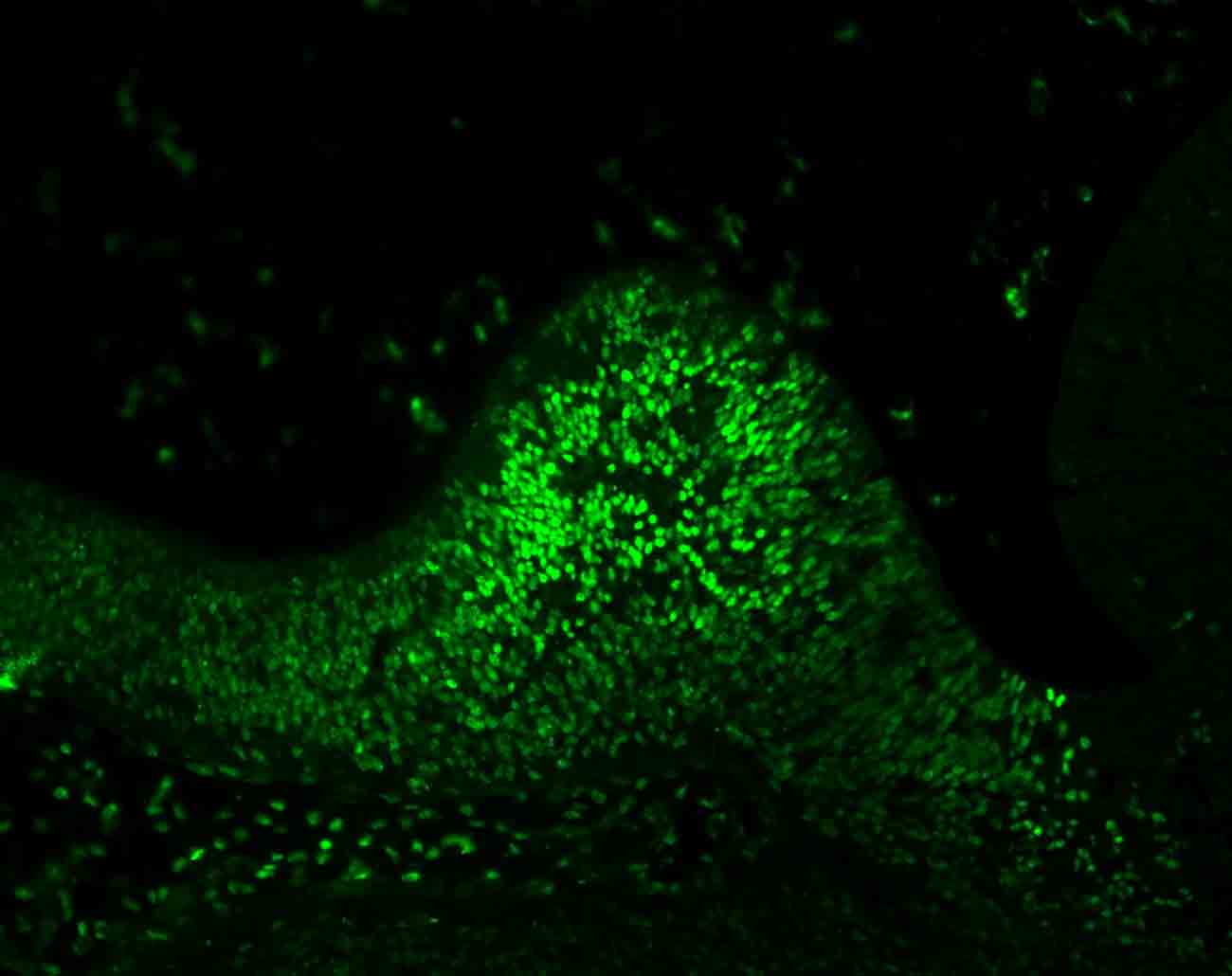

Supplement: Figure 1—figure supplement 1—source data 1. [file elife-86940-fig1-figsupp1-data1.zip › Figure 1-figure supplement 1-source data 1/F1098-1-E11-MUT-CII-LACZ-063X-20X-G15-R150-G.jpg]

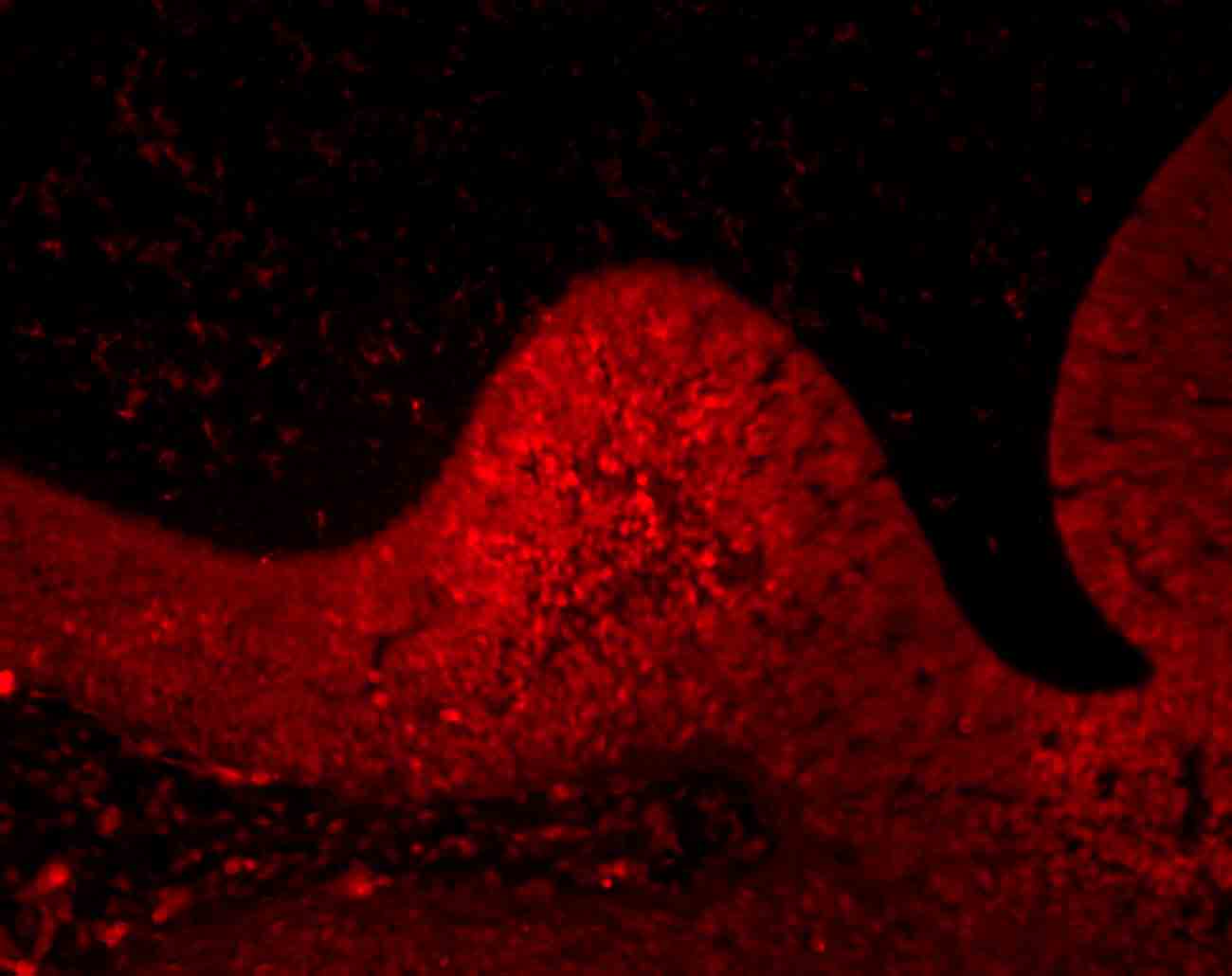

Supplement: Figure 1—figure supplement 1—source data 1. [file elife-86940-fig1-figsupp1-data1.zip › Figure 1-figure supplement 1-source data 1/F1098-1-E11-MUT-CII-LACZ-063X-20X-G15-R150-R.jpg]

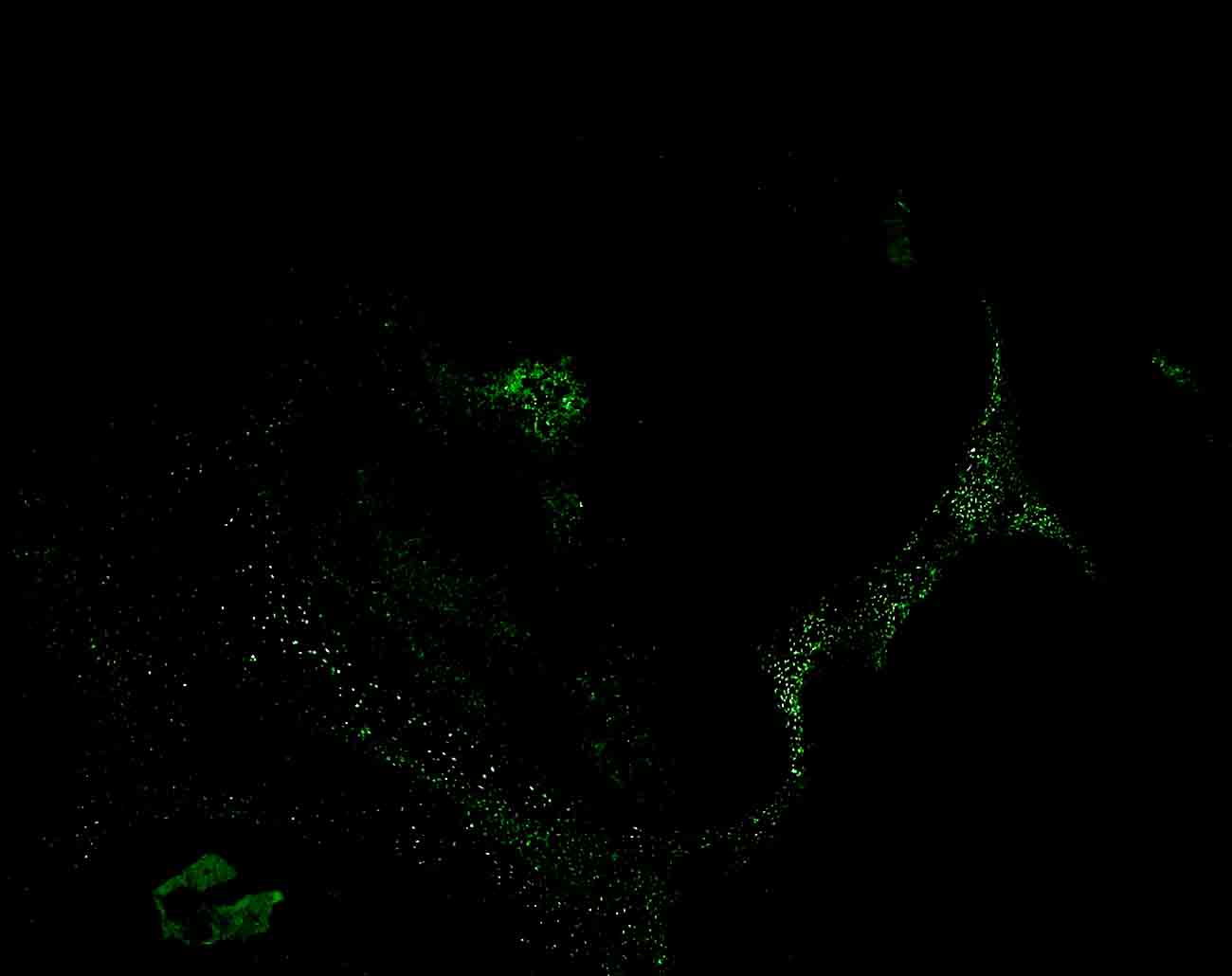

Supplement: Figure 1—figure supplement 1—source data 1. [file elife-86940-fig1-figsupp1-data1.zip › Figure 1-figure supplement 1-source data 1/F1098-1-MUT-CII-LACZ-063X-5X-G80-R600-G.jpg]

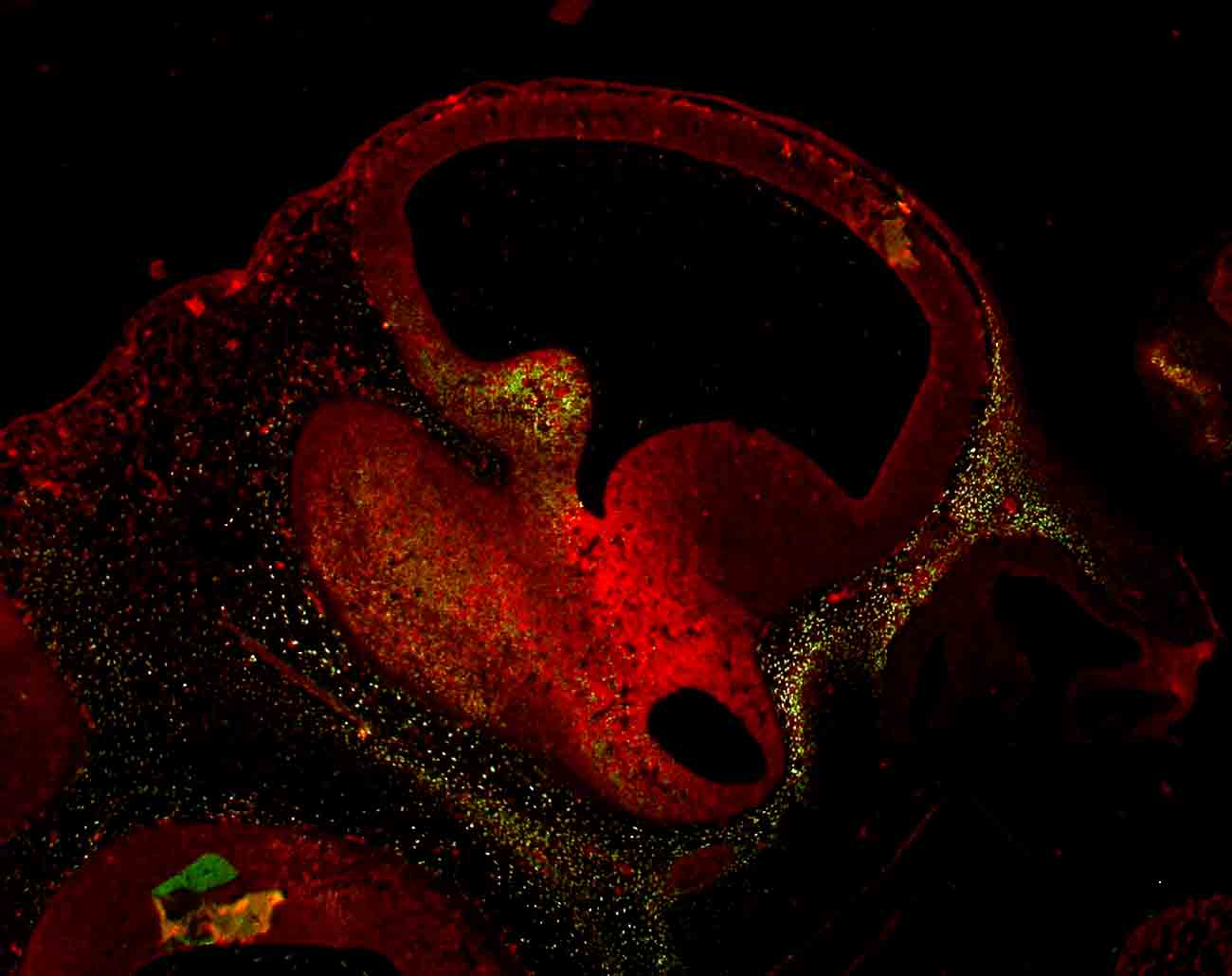

Supplement: Figure 1—figure supplement 1—source data 1. [file elife-86940-fig1-figsupp1-data1.zip › Figure 1-figure supplement 1-source data 1/F1098-1-MUT-CII-LACZ-063X-5X-G80-R600-GR.jpg]

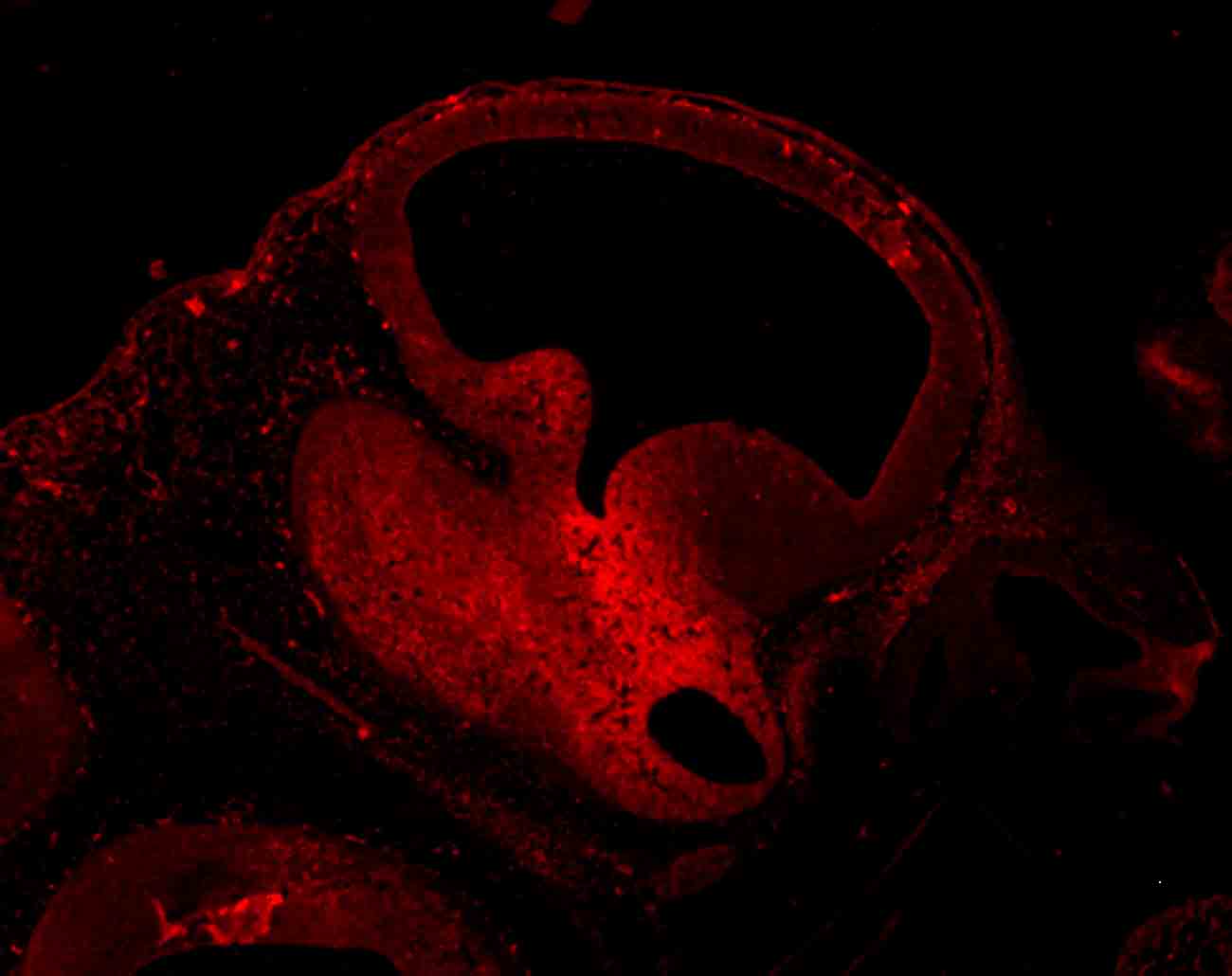

Supplement: Figure 1—figure supplement 1—source data 1. [file elife-86940-fig1-figsupp1-data1.zip › Figure 1-figure supplement 1-source data 1/F1098-1-MUT-CII-LACZ-063X-5X-G80-R600-R.jpg]

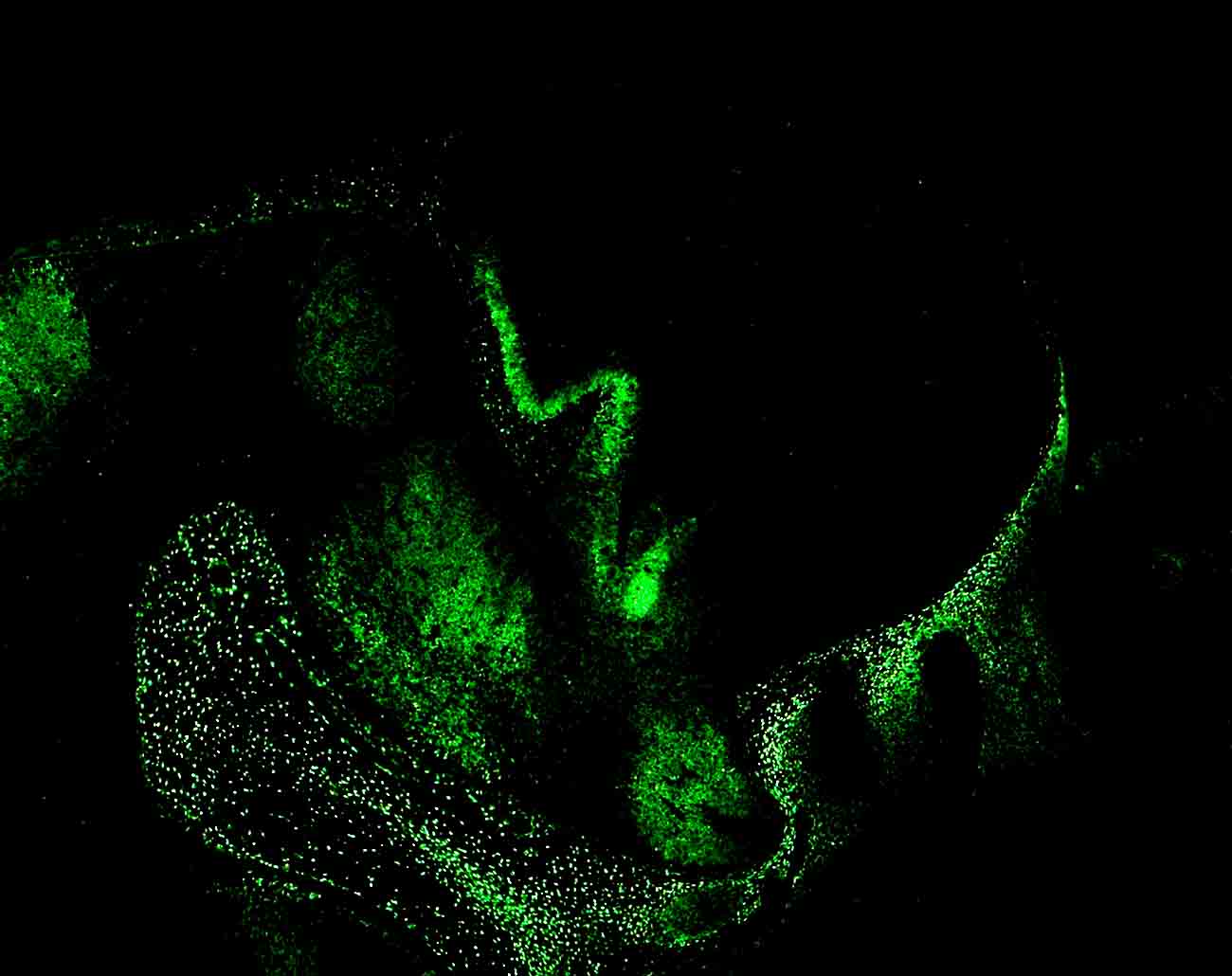

Supplement: Figure 1—figure supplement 1—source data 1. [file elife-86940-fig1-figsupp1-data1.zip › Figure 1-figure supplement 1-source data 1/F1098-2-CON-CII-LACZ-063X-5X-G80-R600-G.jpg]

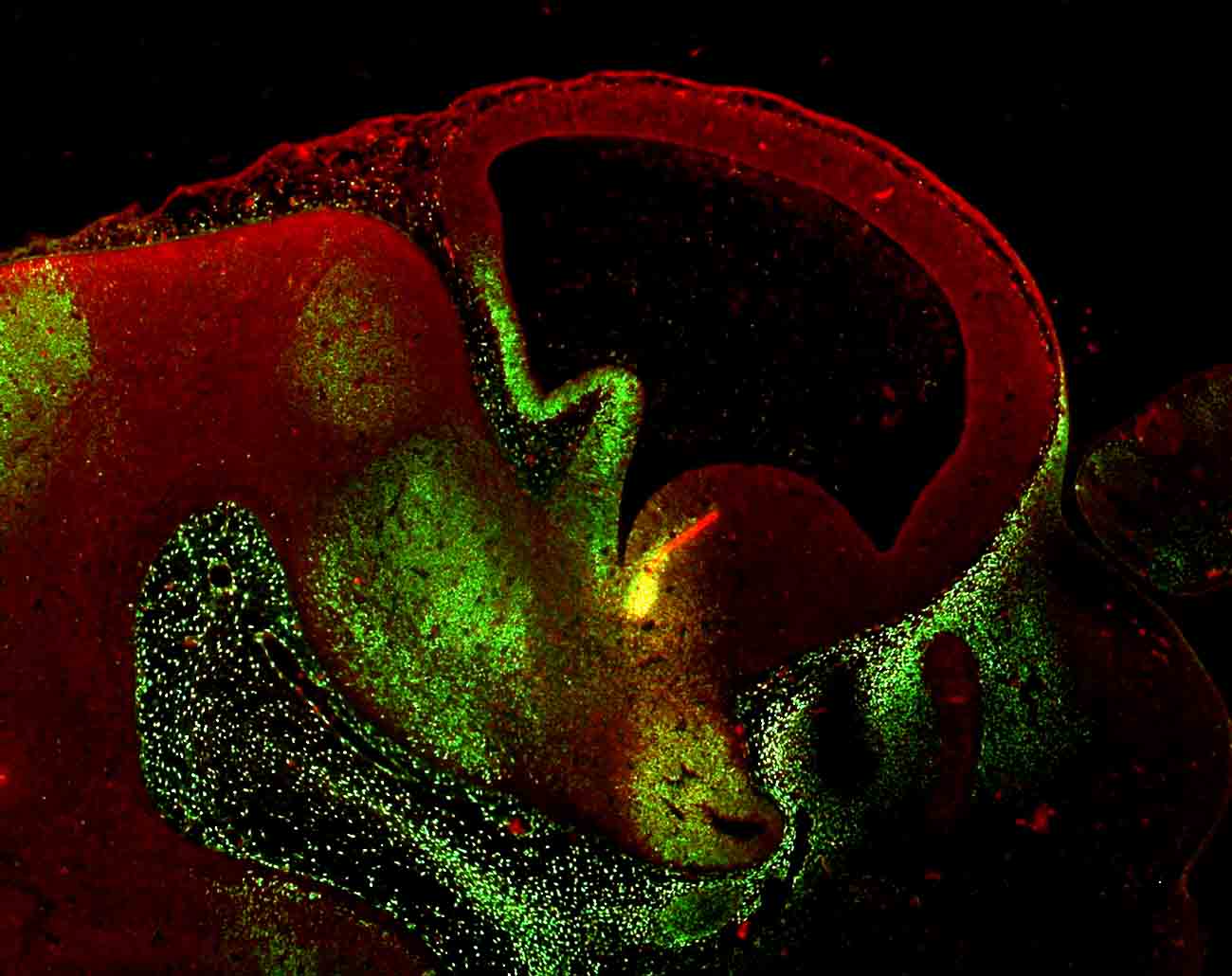

Supplement: Figure 1—figure supplement 1—source data 1. [file elife-86940-fig1-figsupp1-data1.zip › Figure 1-figure supplement 1-source data 1/F1098-2-CON-CII-LACZ-063X-5X-G80-R600-GR.jpg]

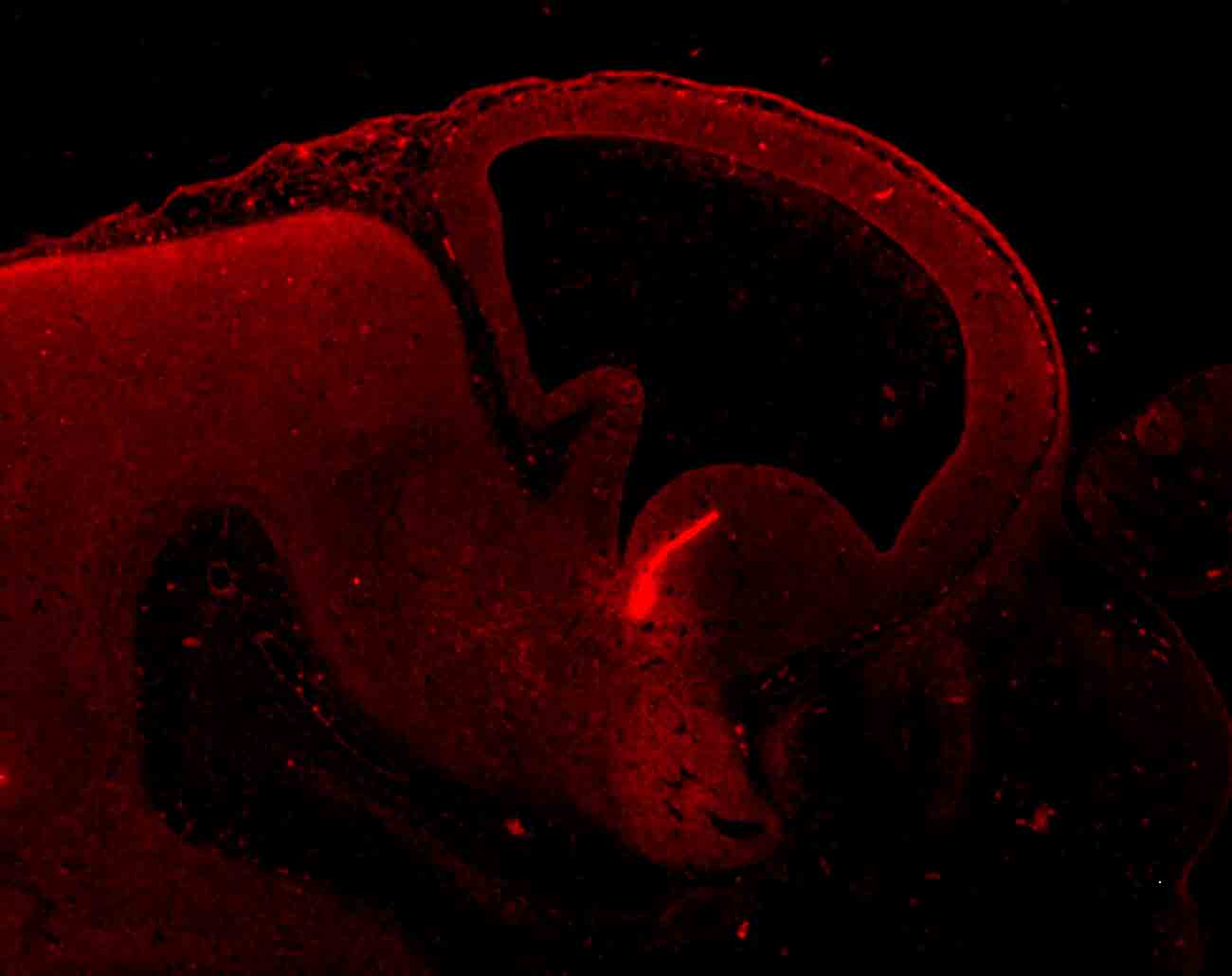

Supplement: Figure 1—figure supplement 1—source data 1. [file elife-86940-fig1-figsupp1-data1.zip › Figure 1-figure supplement 1-source data 1/F1098-2-CON-CII-LACZ-063X-5X-G80-R600-R.jpg]

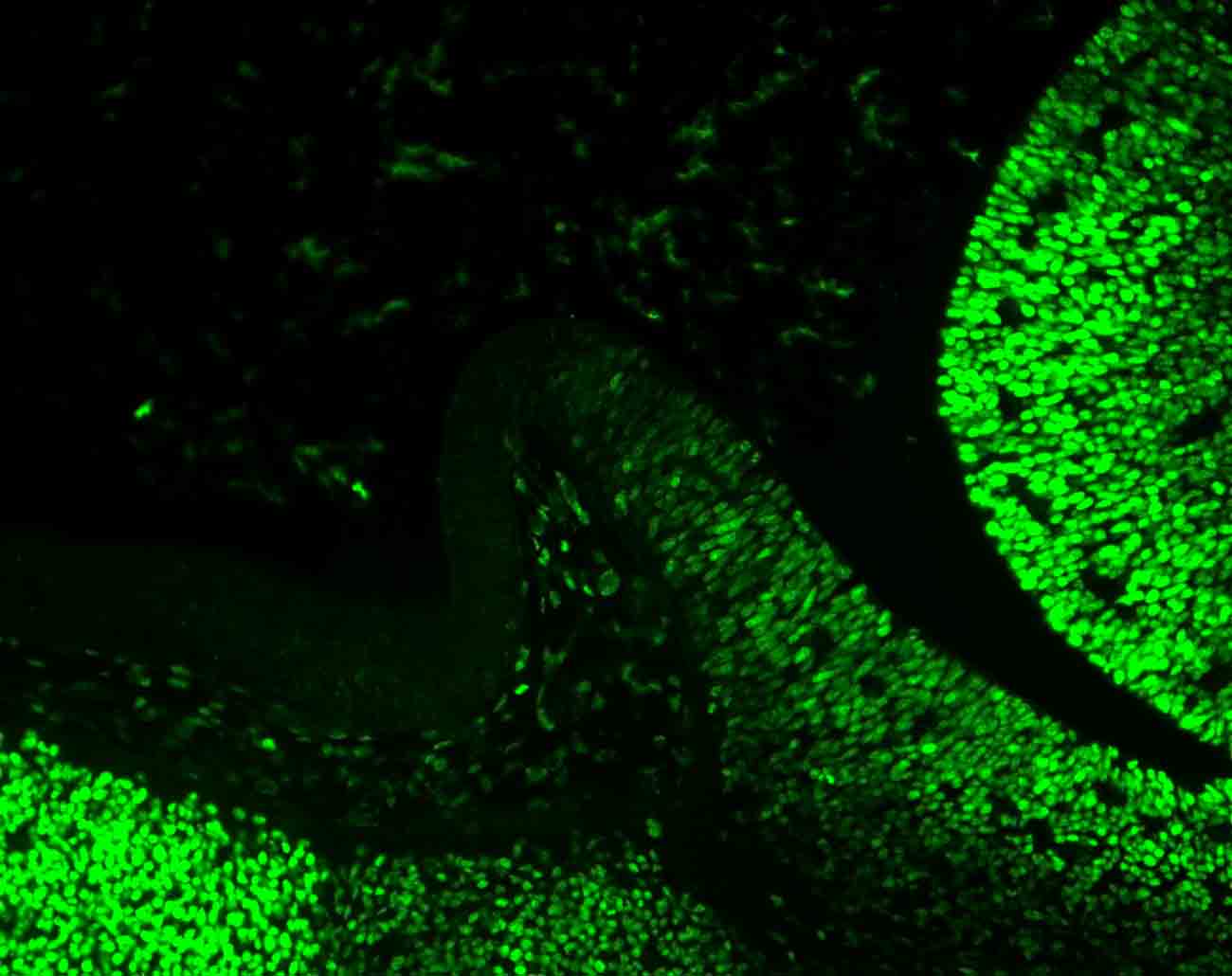

Supplement: Figure 1—figure supplement 1—source data 1. [file elife-86940-fig1-figsupp1-data1.zip › Figure 1-figure supplement 1-source data 1/F1098-2-E11-HET-CI-LACZ-063X-20X-G10-G.jpg]

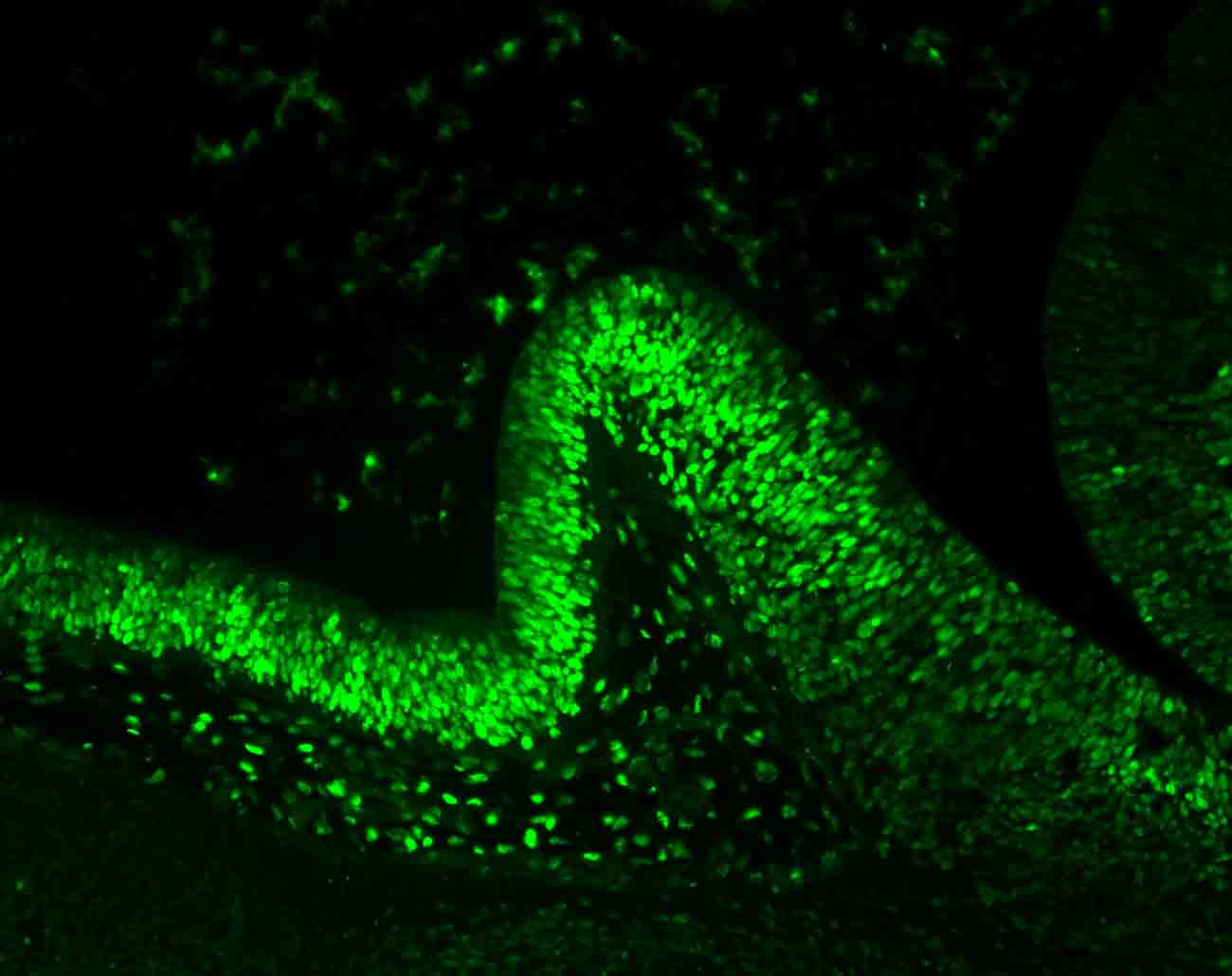

Supplement: Figure 1—figure supplement 1—source data 1. [file elife-86940-fig1-figsupp1-data1.zip › Figure 1-figure supplement 1-source data 1/F1098-2-E11-HET-CII-LACZ-063X-20X-G15-R150-G.jpg]

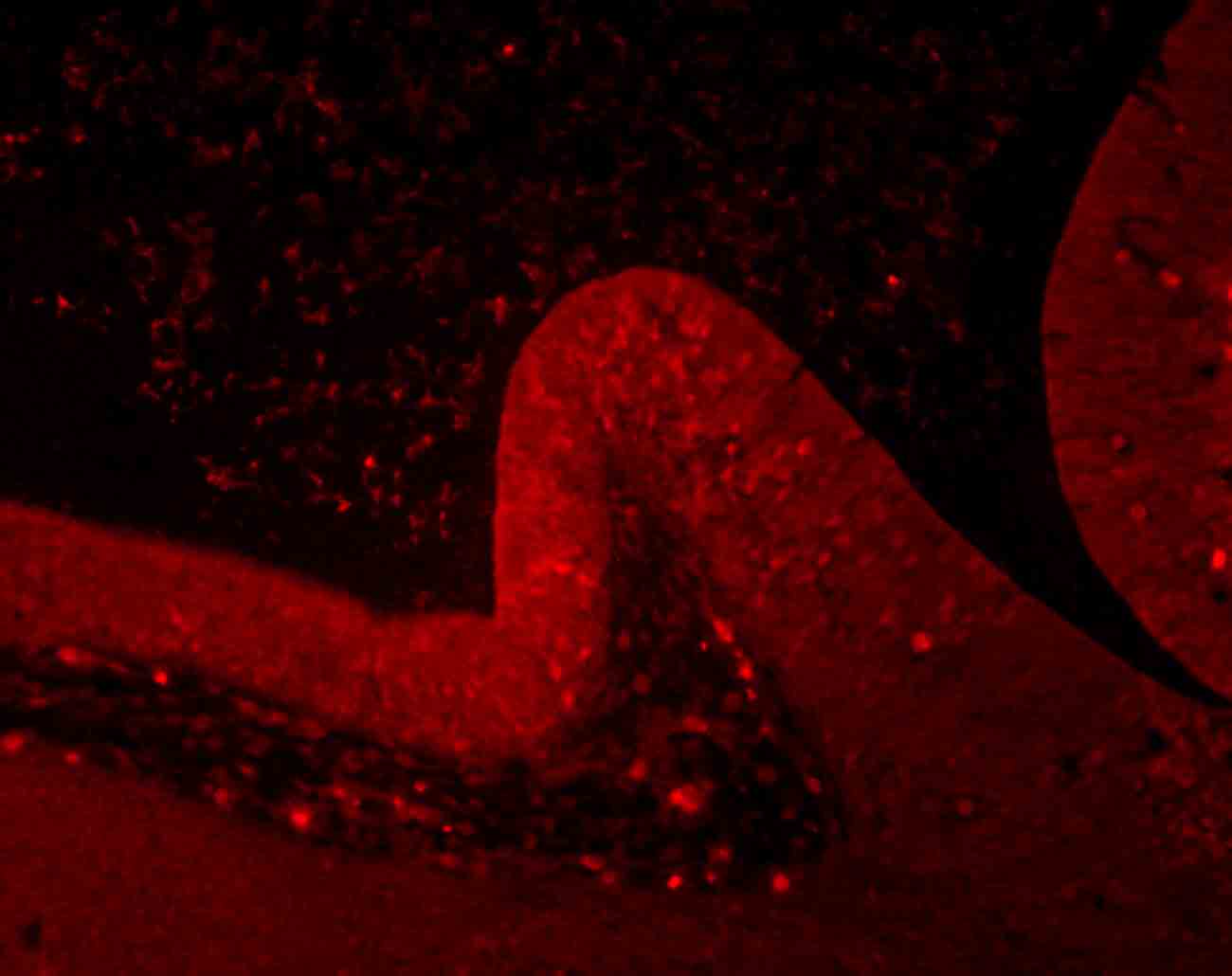

Supplement: Figure 1—figure supplement 1—source data 1. [file elife-86940-fig1-figsupp1-data1.zip › Figure 1-figure supplement 1-source data 1/F1098-2-E11-HET-CII-LACZ-063X-20X-G15-R150-R.jpg]

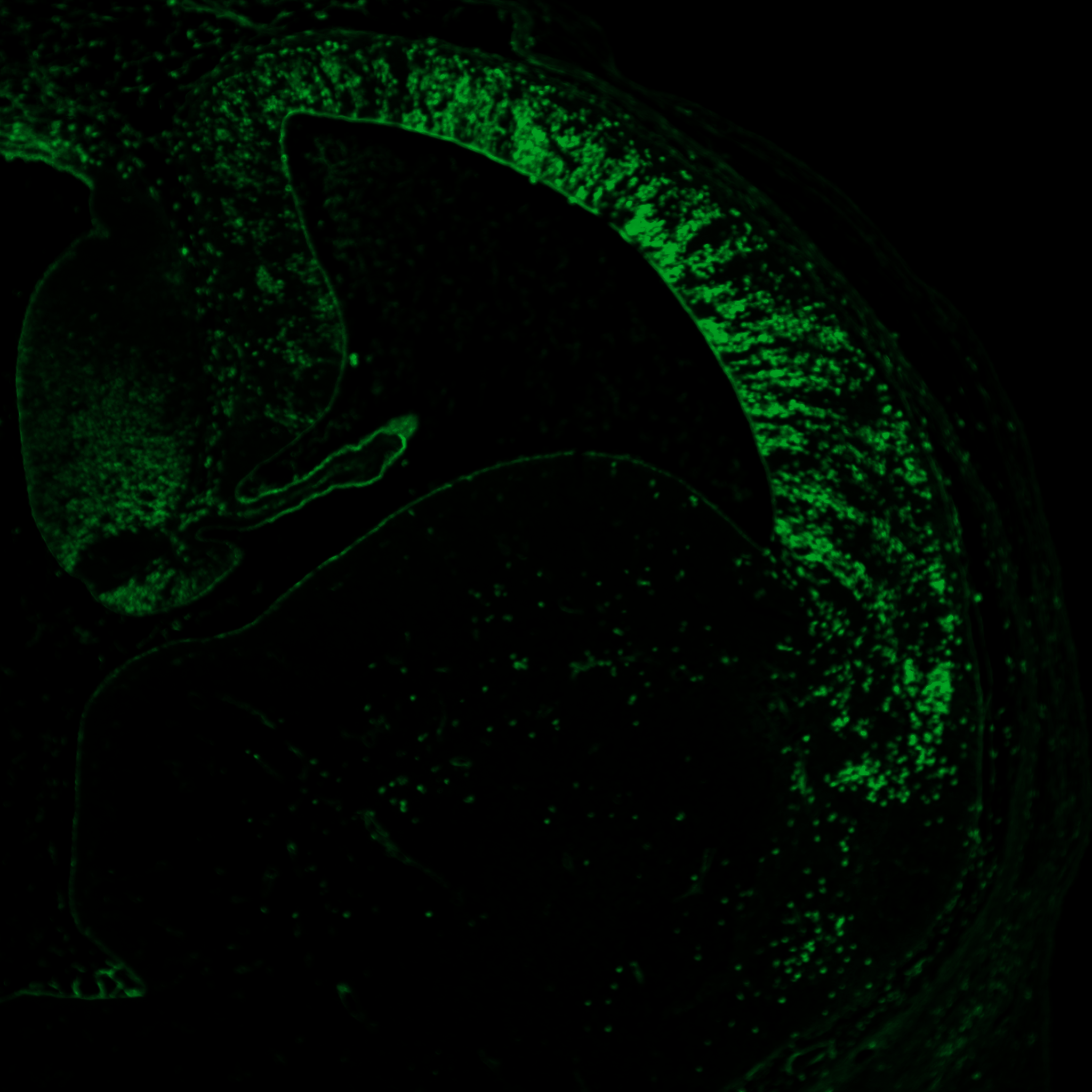

Supplement: Figure 1—figure supplement 1—source data 1. [file elife-86940-fig1-figsupp1-data1.zip › Figure 1-figure supplement 1-source data 1/F1189-7-E14.5-DKO-10X-RX FF ff-#25-CI-CII-4-R-Image Export-24_AF488.tif]

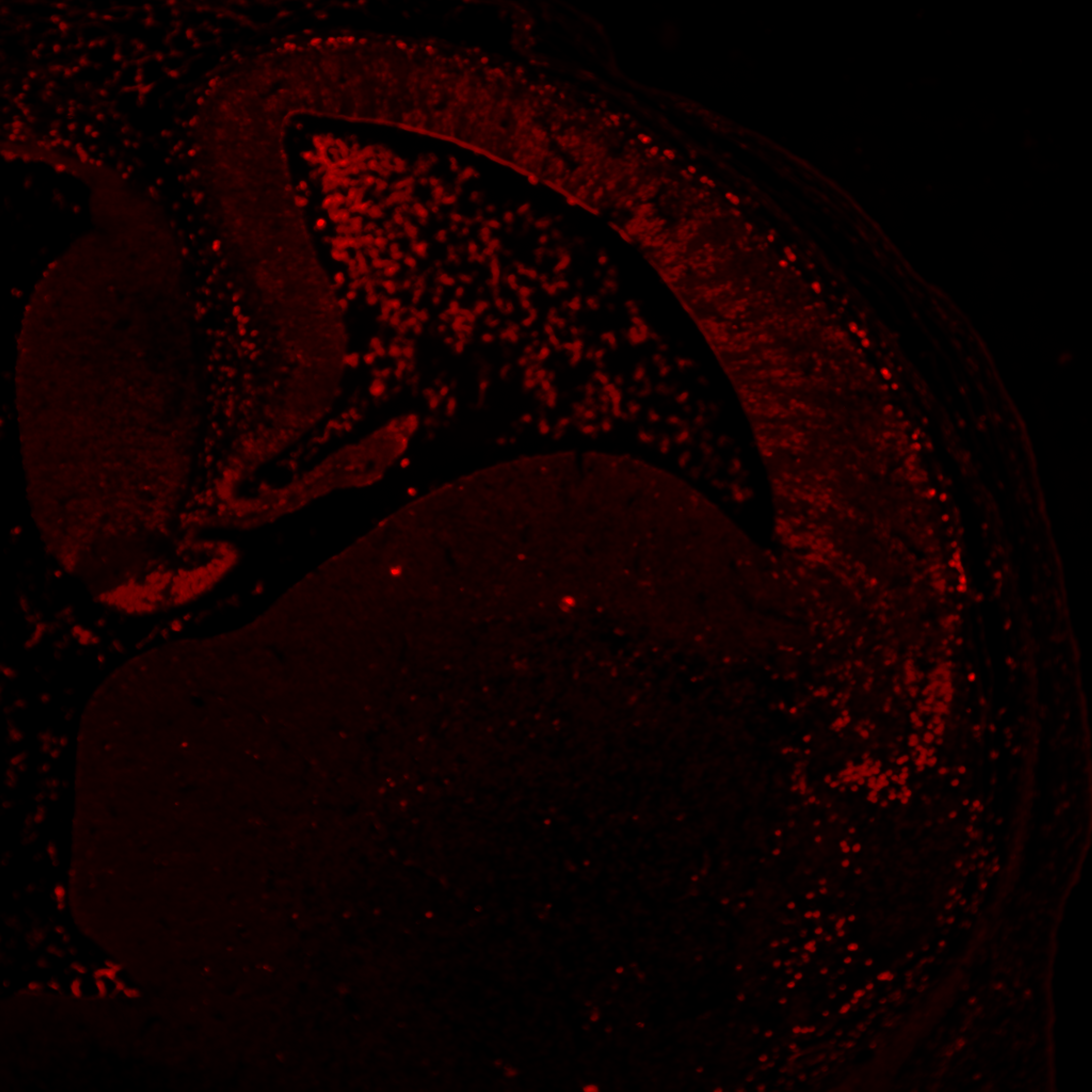

Supplement: Figure 1—figure supplement 1—source data 1. [file elife-86940-fig1-figsupp1-data1.zip › Figure 1-figure supplement 1-source data 1/F1189-7-E14.5-DKO-10X-RX FF ff-#25-CI-CII-4-R-Image Export-24_AF594.tif]

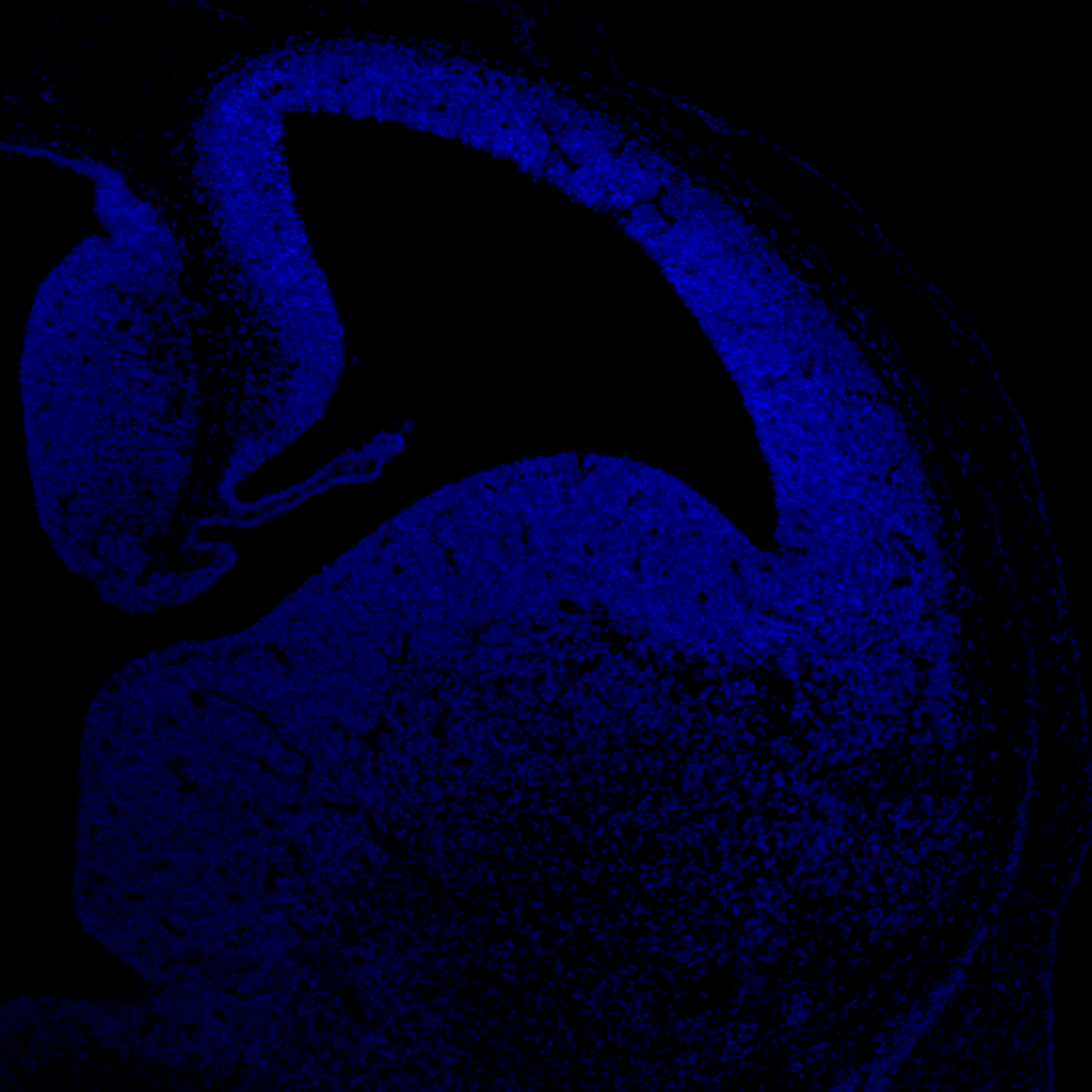

Supplement: Figure 1—figure supplement 1—source data 1. [file elife-86940-fig1-figsupp1-data1.zip › Figure 1-figure supplement 1-source data 1/F1189-7-E14.5-DKO-10X-RX FF ff-#25-CI-CII-4-R-Image Export-24_DAPI.tif]

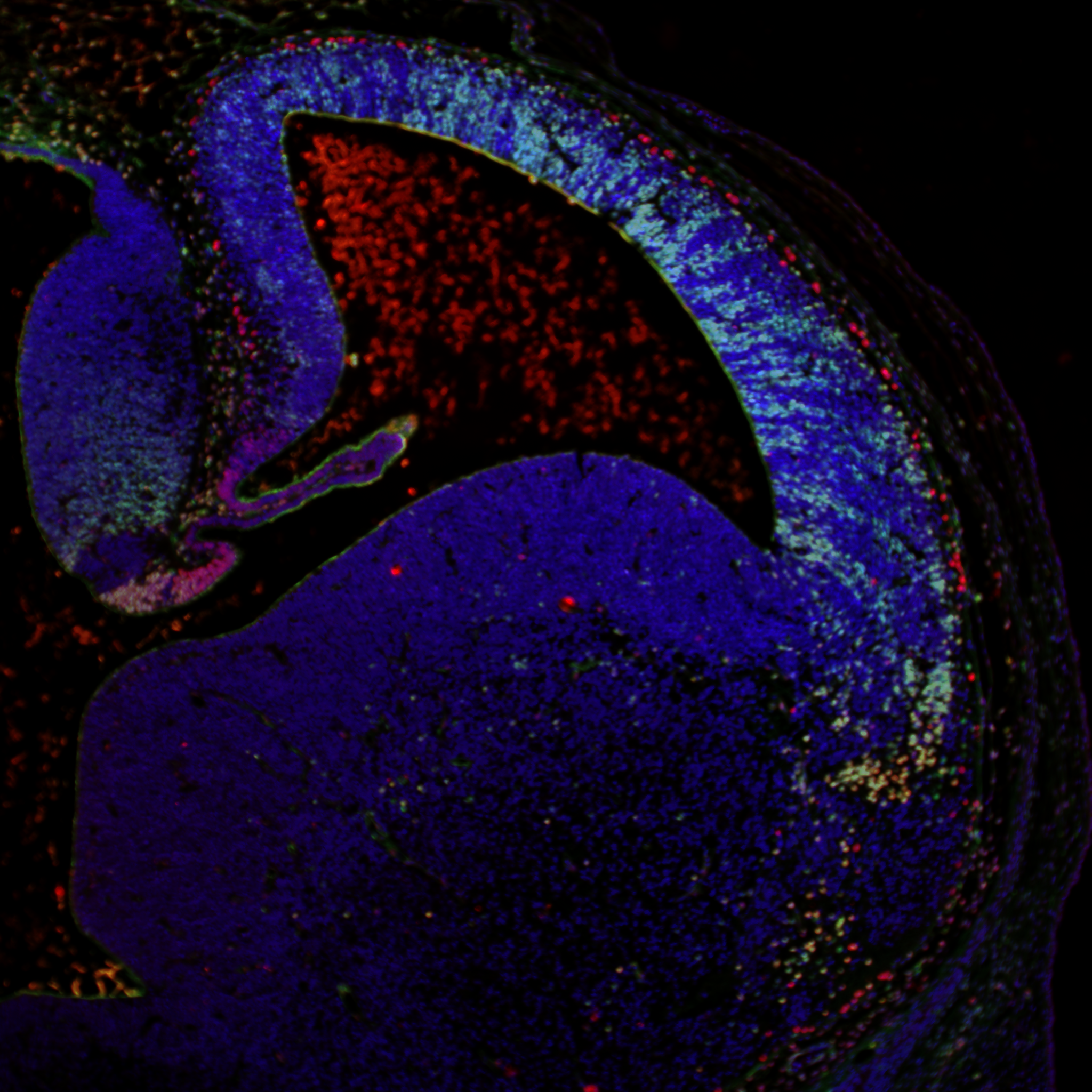

Supplement: Figure 1—figure supplement 1—source data 1. [file elife-86940-fig1-figsupp1-data1.zip › Figure 1-figure supplement 1-source data 1/F1189-7-E14.5-DKO-10X-RX FF ff-#25-CI-CII-4-R-Image Export-24.tif]

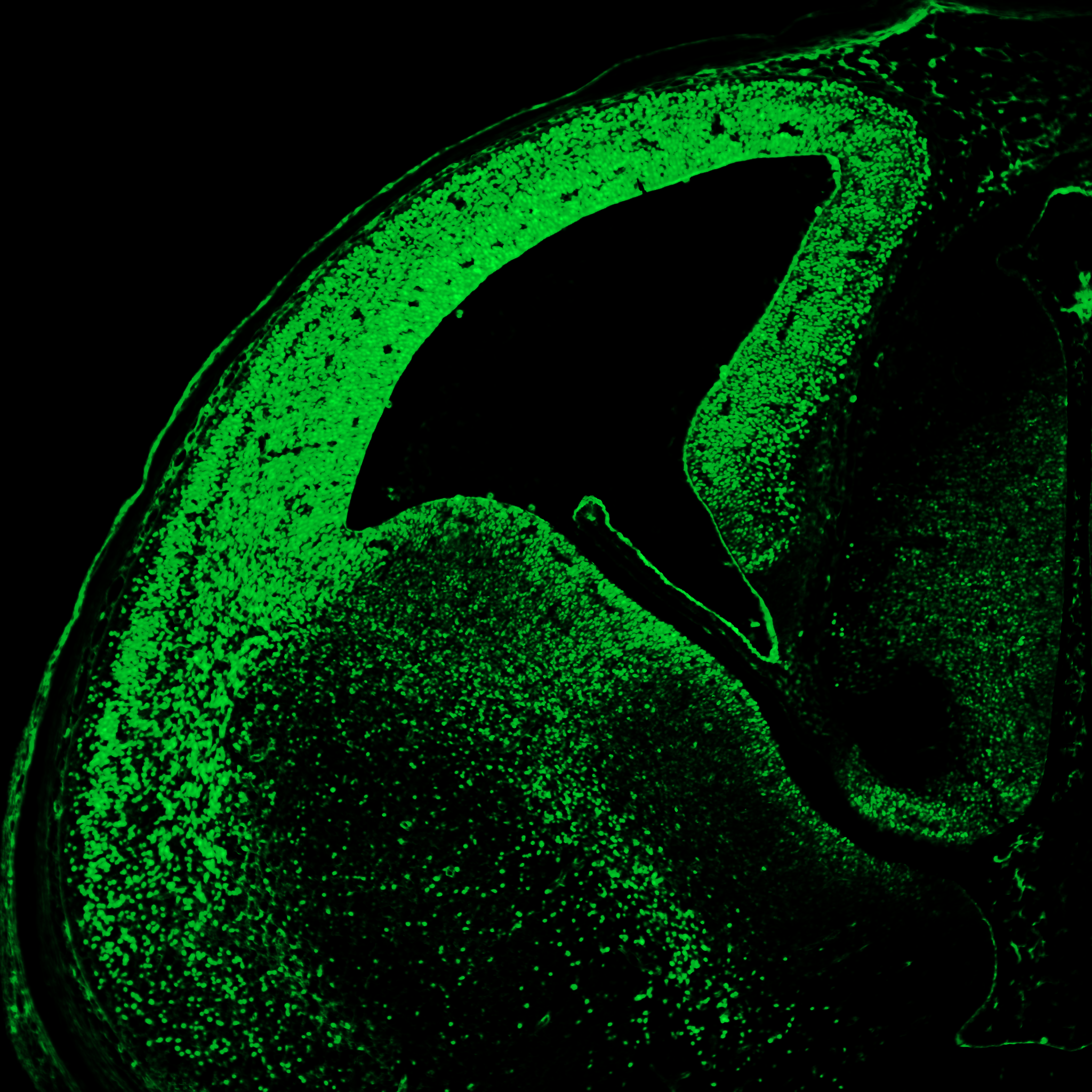

Supplement: Figure 1—figure supplement 1—source data 2. [file elife-86940-fig1-figsupp1-data2.zip › Figure 1-figure supplement 1-source data 2/F1189-8-E14.5-CON-10X-F+ ff-#25-CI-CII-4-L-Image Export-22_AF488.tif]

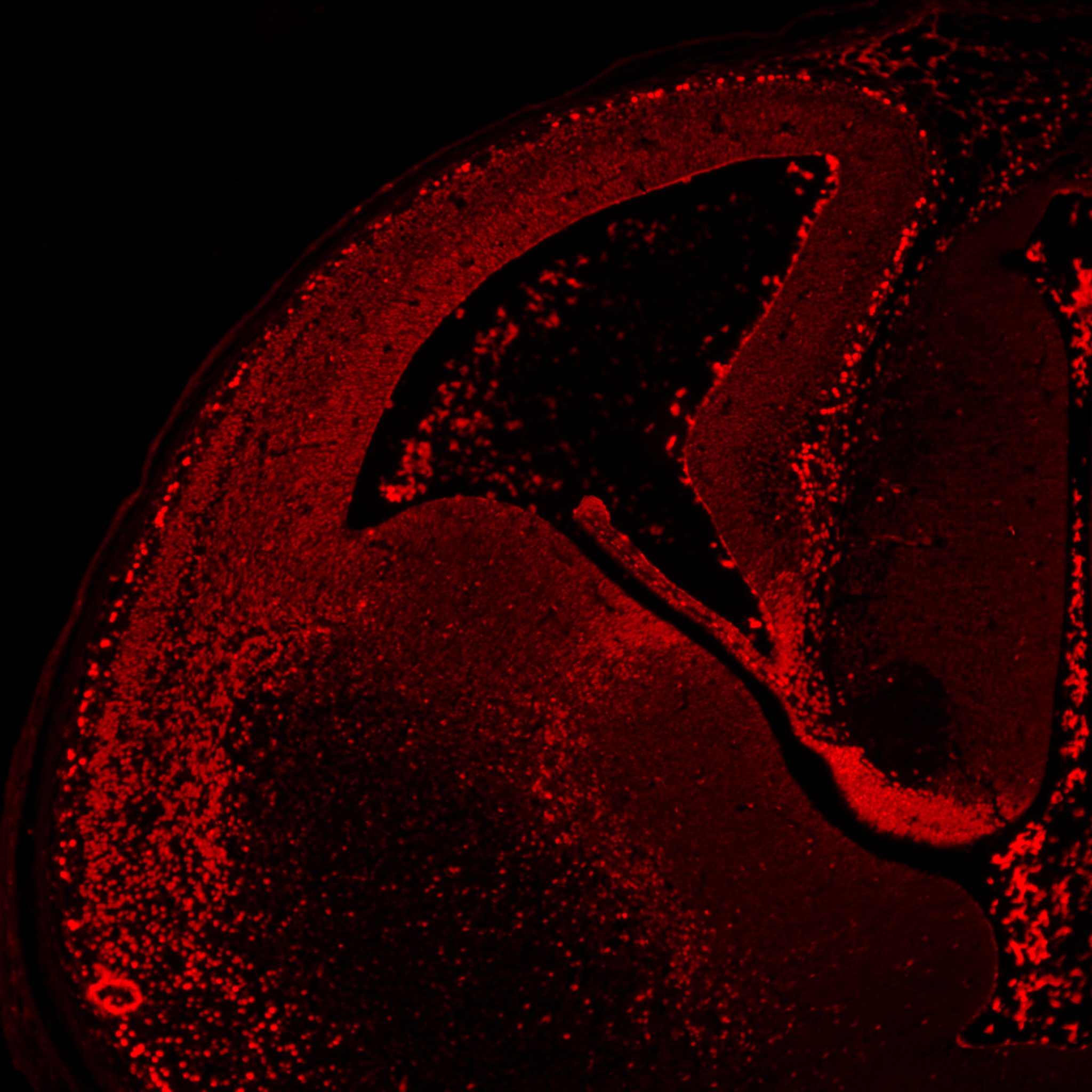

Supplement: Figure 1—figure supplement 1—source data 2. [file elife-86940-fig1-figsupp1-data2.zip › Figure 1-figure supplement 1-source data 2/F1189-8-E14.5-CON-10X-F+ ff-#25-CI-CII-4-L-Image Export-22_AF594.tif]

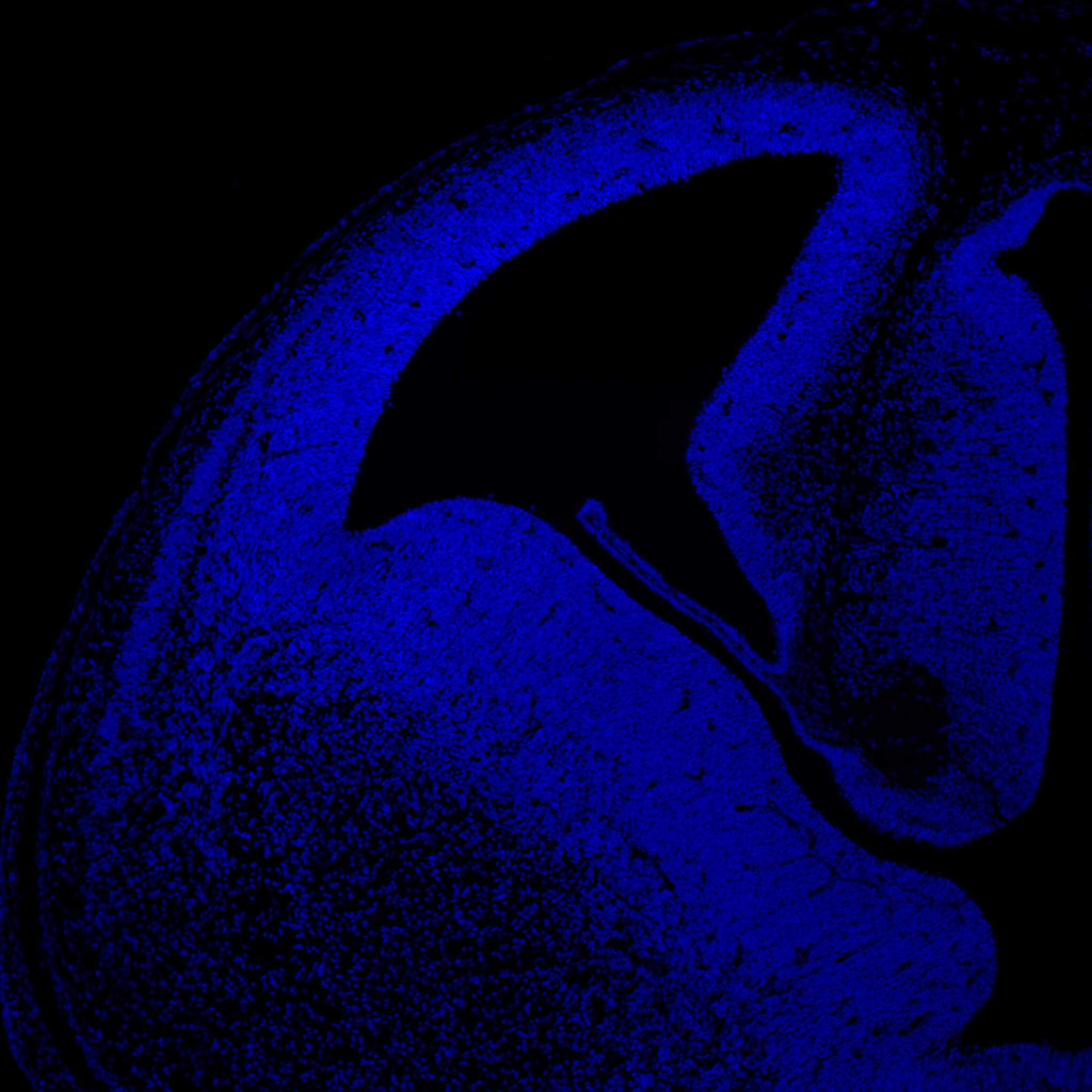

Supplement: Figure 1—figure supplement 1—source data 2. [file elife-86940-fig1-figsupp1-data2.zip › Figure 1-figure supplement 1-source data 2/F1189-8-E14.5-CON-10X-F+ ff-#25-CI-CII-4-L-Image Export-22_DAPI.tif]

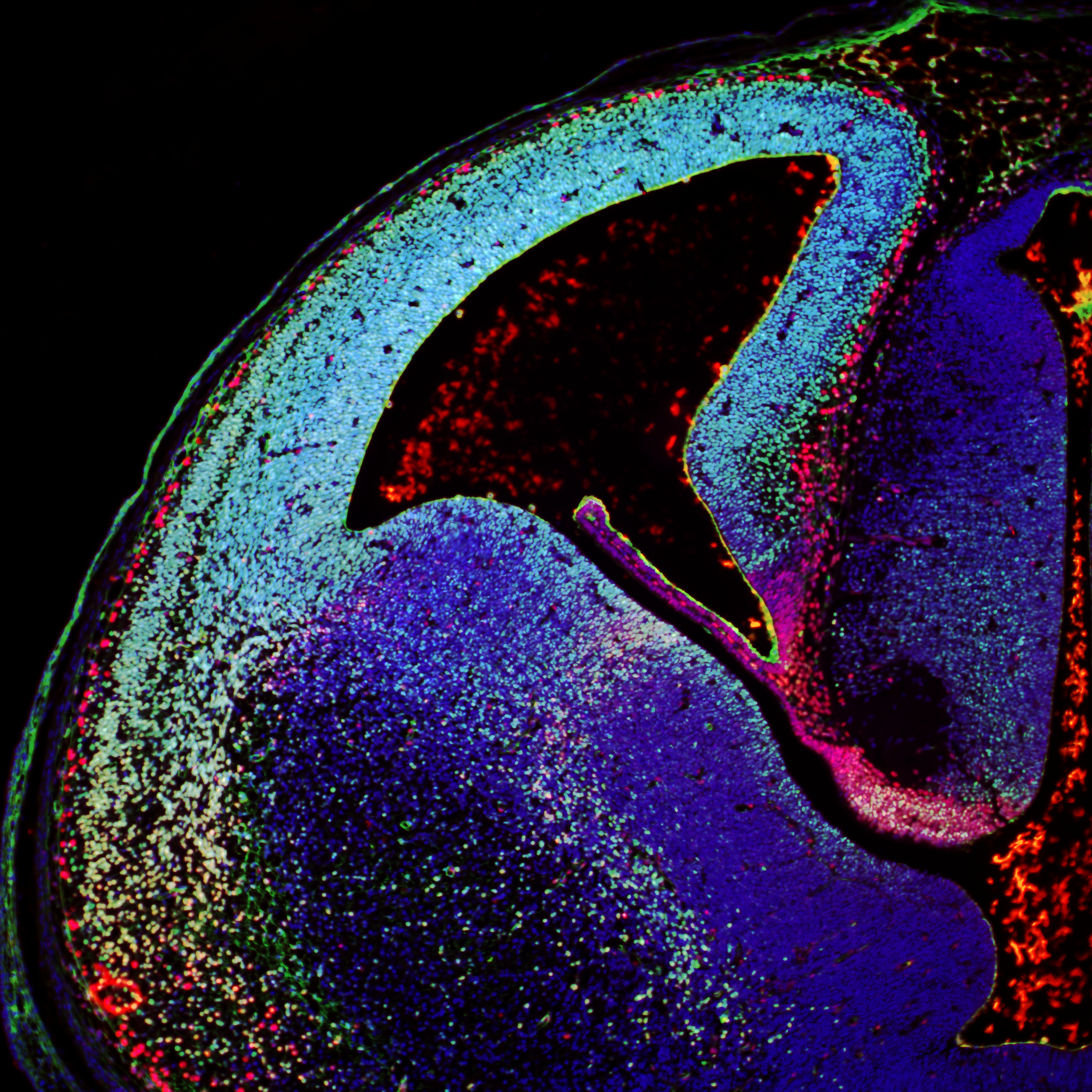

Supplement: Figure 1—figure supplement 1—source data 2. [file elife-86940-fig1-figsupp1-data2.zip › Figure 1-figure supplement 1-source data 2/F1189-8-E14.5-CON-10X-F+ ff-#25-CI-CII-4-L-Image Export-22.tif]

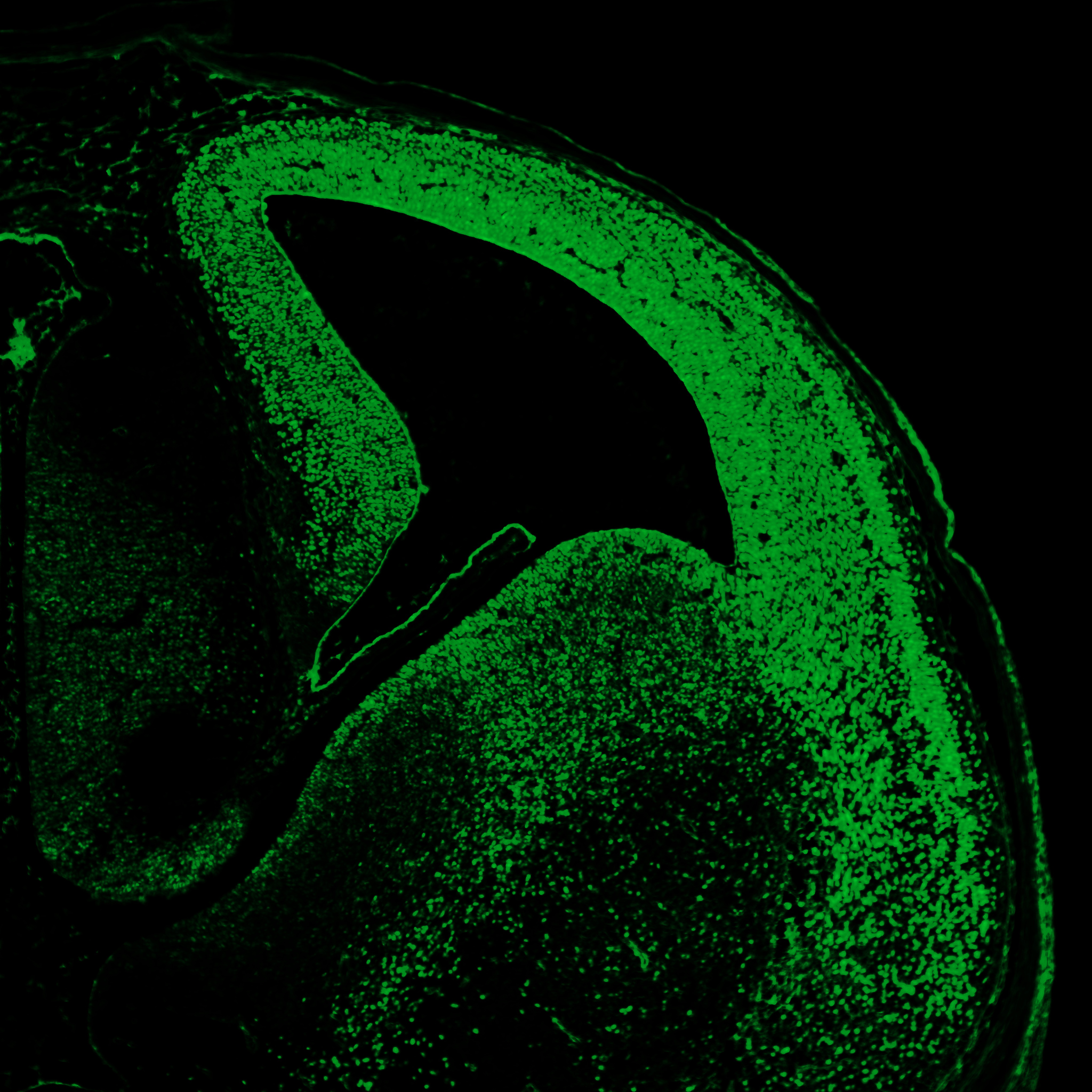

Supplement: Figure 1—figure supplement 1—source data 2. [file elife-86940-fig1-figsupp1-data2.zip › Figure 1-figure supplement 1-source data 2/F1189-8-E14.5-CON-10X-F+ ff-#25-CI-CII-4-R-Image Export-24_AF488.tif]

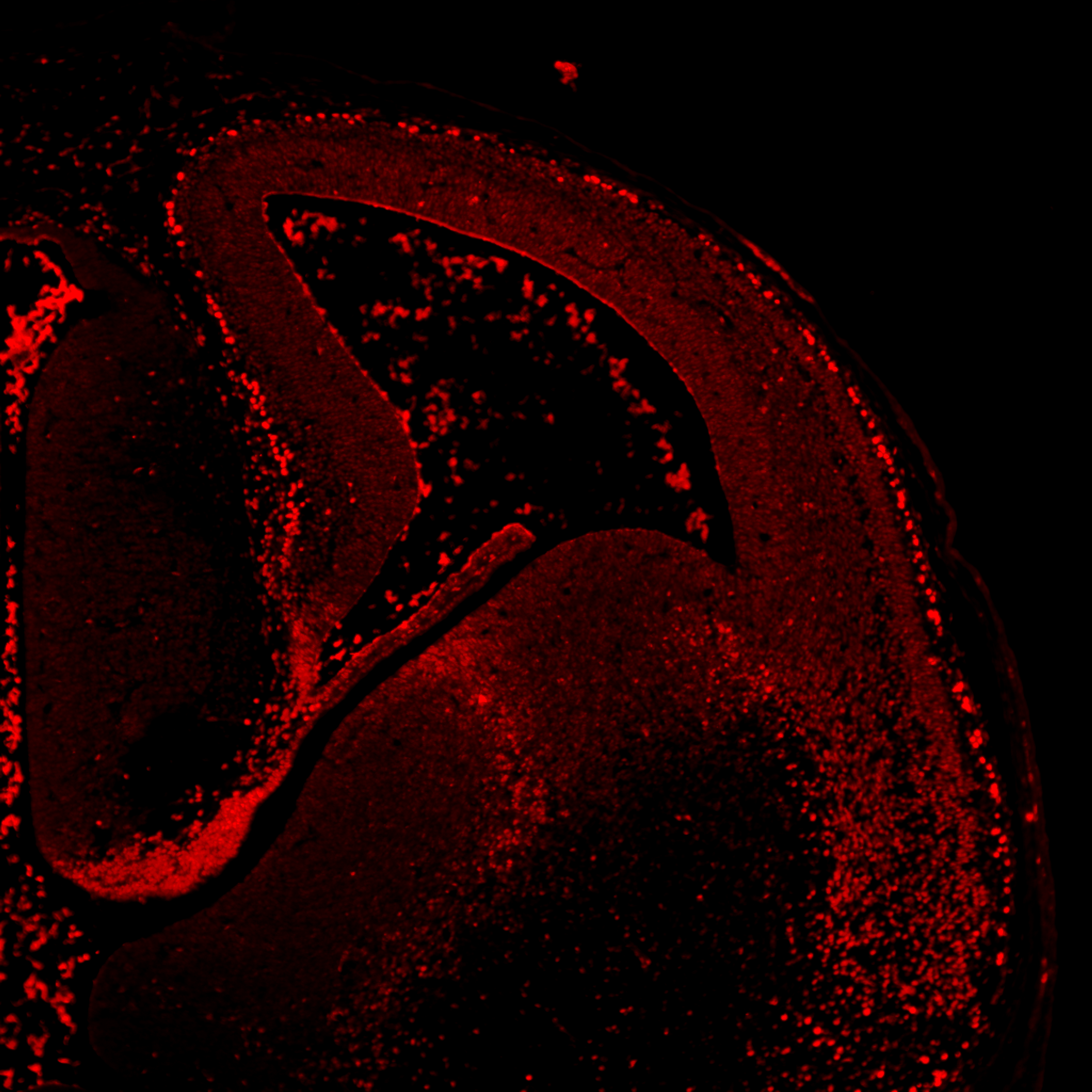

Supplement: Figure 1—figure supplement 1—source data 2. [file elife-86940-fig1-figsupp1-data2.zip › Figure 1-figure supplement 1-source data 2/F1189-8-E14.5-CON-10X-F+ ff-#25-CI-CII-4-R-Image Export-24_AF594.tif]

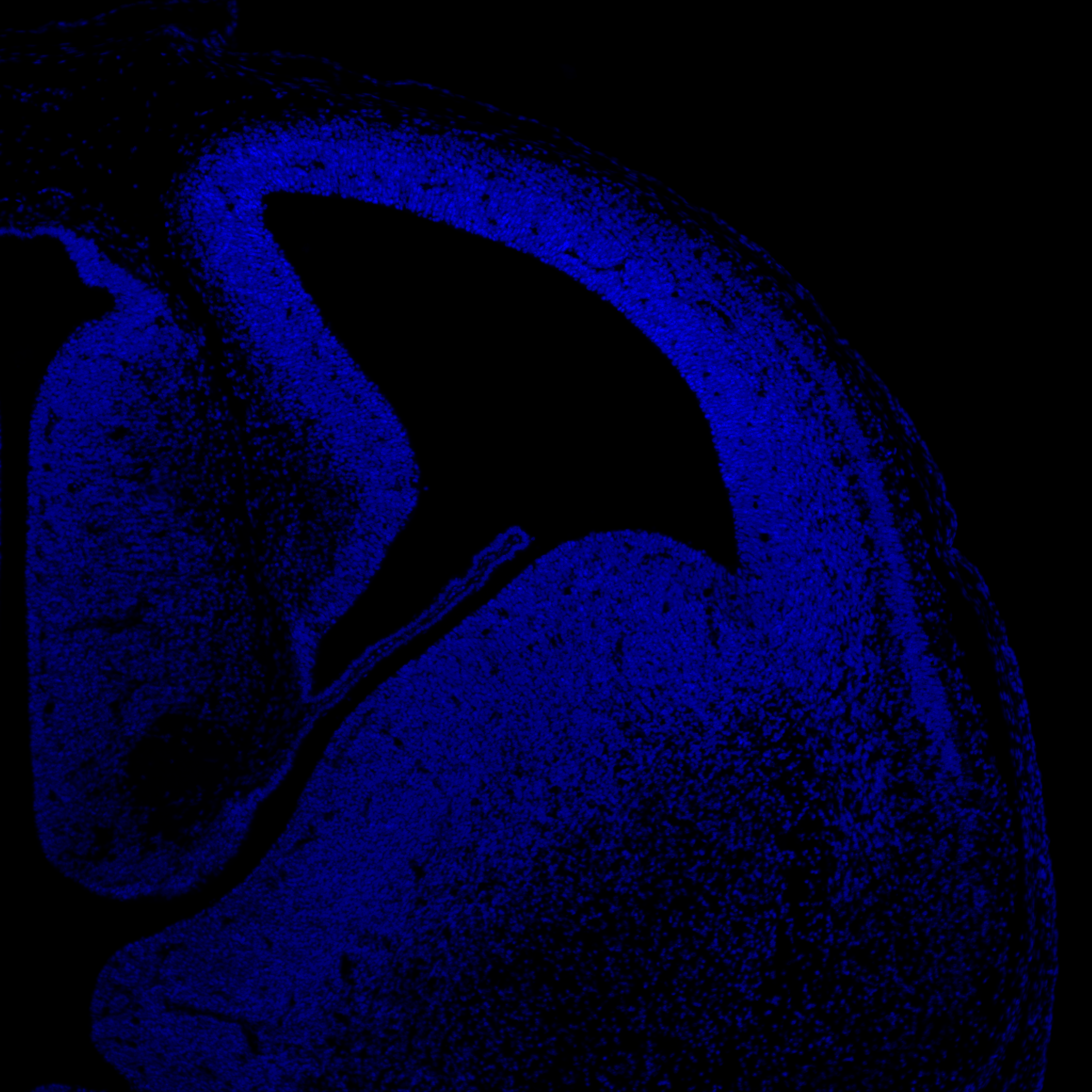

Supplement: Figure 1—figure supplement 1—source data 2. [file elife-86940-fig1-figsupp1-data2.zip › Figure 1-figure supplement 1-source data 2/F1189-8-E14.5-CON-10X-F+ ff-#25-CI-CII-4-R-Image Export-24_DAPI.tif]

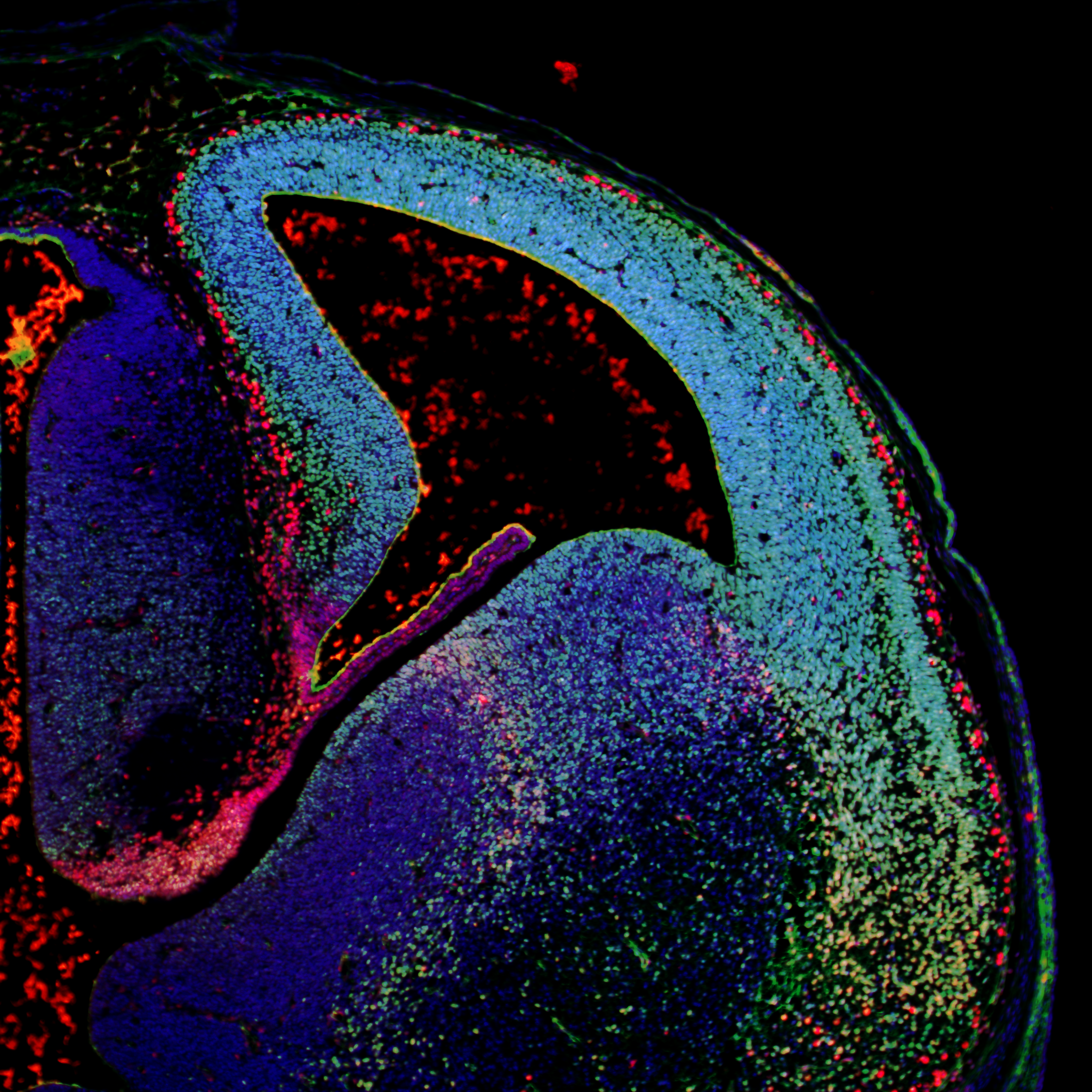

Supplement: Figure 1—figure supplement 1—source data 2. [file elife-86940-fig1-figsupp1-data2.zip › Figure 1-figure supplement 1-source data 2/F1189-8-E14.5-CON-10X-F+ ff-#25-CI-CII-4-R-Image Export-24.tif]

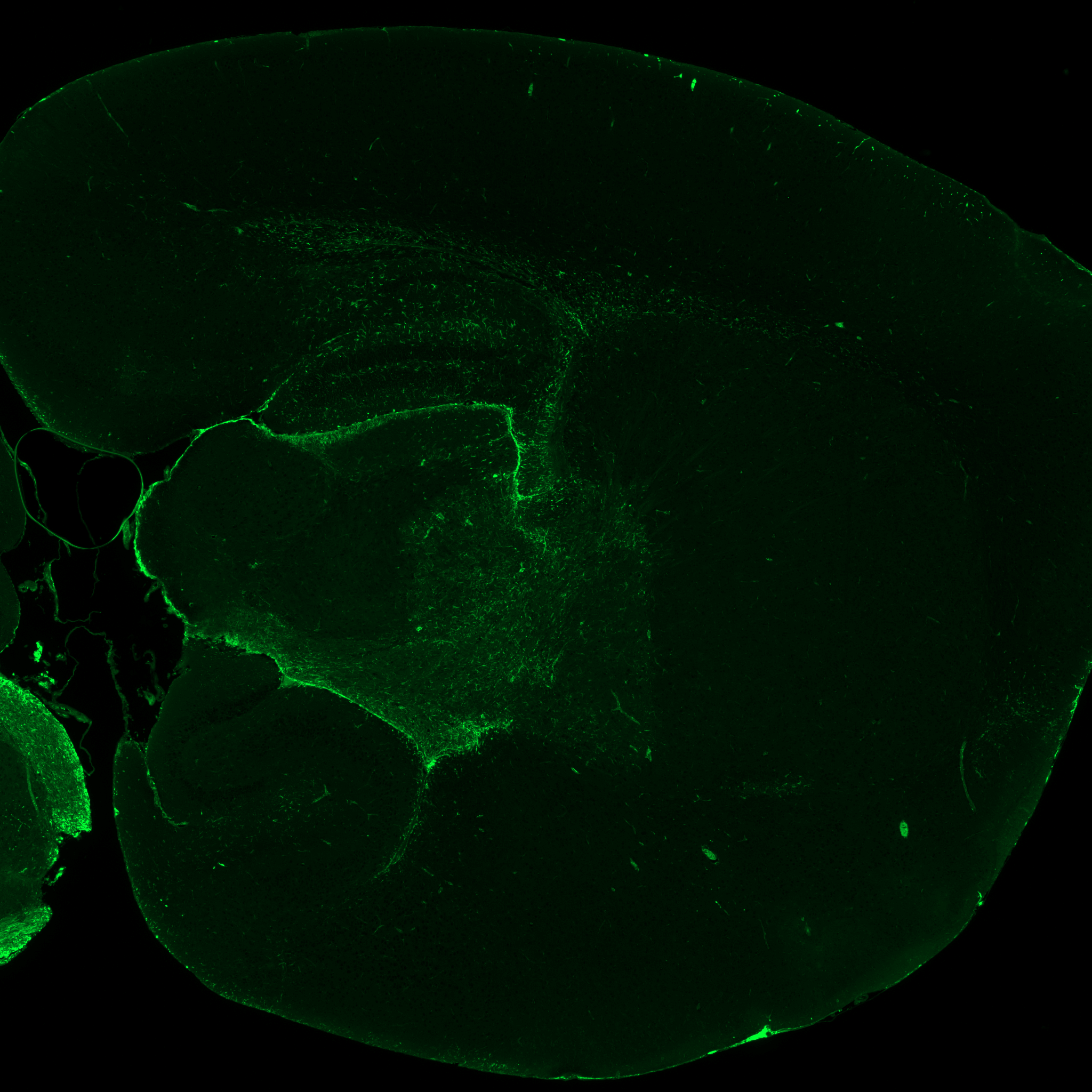

Supplement: Figure 1—figure supplement 1—source data 2. [file elife-86940-fig1-figsupp1-data2.zip › Figure 1-figure supplement 1-source data 2/F3094-3-CI CKO-RX CI ff-1M-2.5X-CI-CII-2-Image Export-24_AF488.tif]

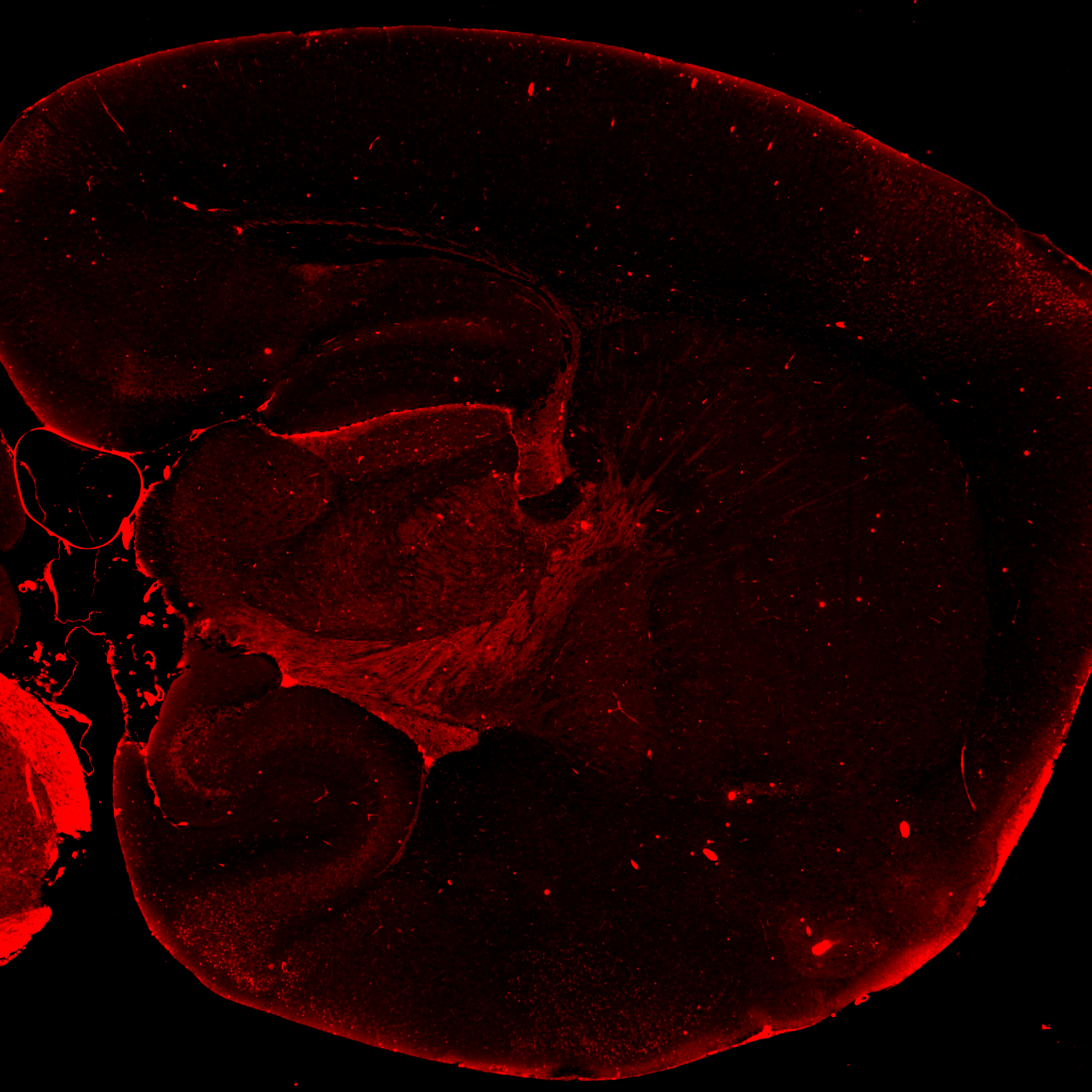

Supplement: Figure 1—figure supplement 1—source data 2. [file elife-86940-fig1-figsupp1-data2.zip › Figure 1-figure supplement 1-source data 2/F3094-3-CI CKO-RX CI ff-1M-2.5X-CI-CII-2-Image Export-24_AF594.tif]

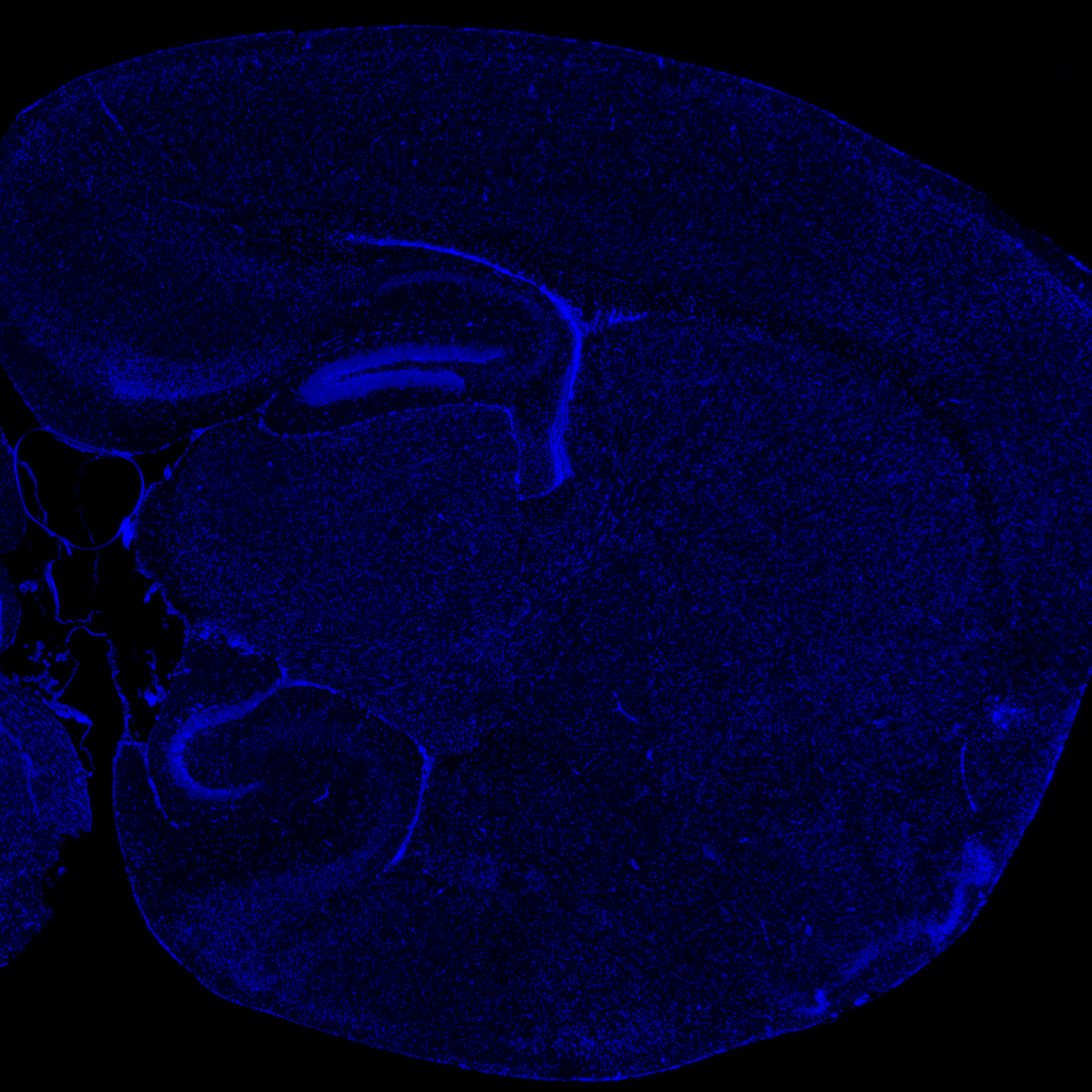

Supplement: Figure 1—figure supplement 1—source data 2. [file elife-86940-fig1-figsupp1-data2.zip › Figure 1-figure supplement 1-source data 2/F3094-3-CI CKO-RX CI ff-1M-2.5X-CI-CII-2-Image Export-24_DAPI.tif]

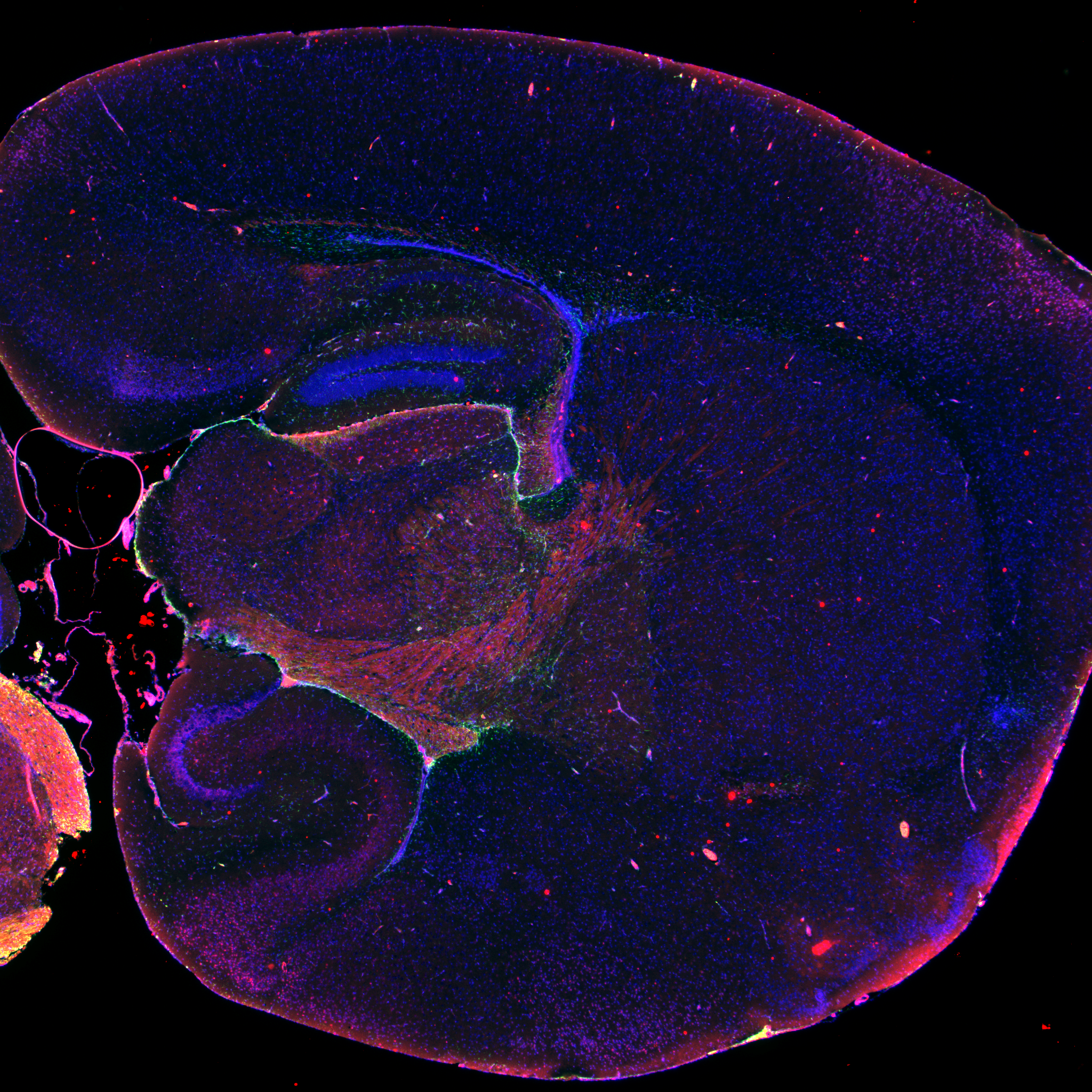

Supplement: Figure 1—figure supplement 1—source data 2. [file elife-86940-fig1-figsupp1-data2.zip › Figure 1-figure supplement 1-source data 2/F3094-3-CI CKO-RX CI ff-1M-2.5X-CI-CII-2-Image Export-24.tif]

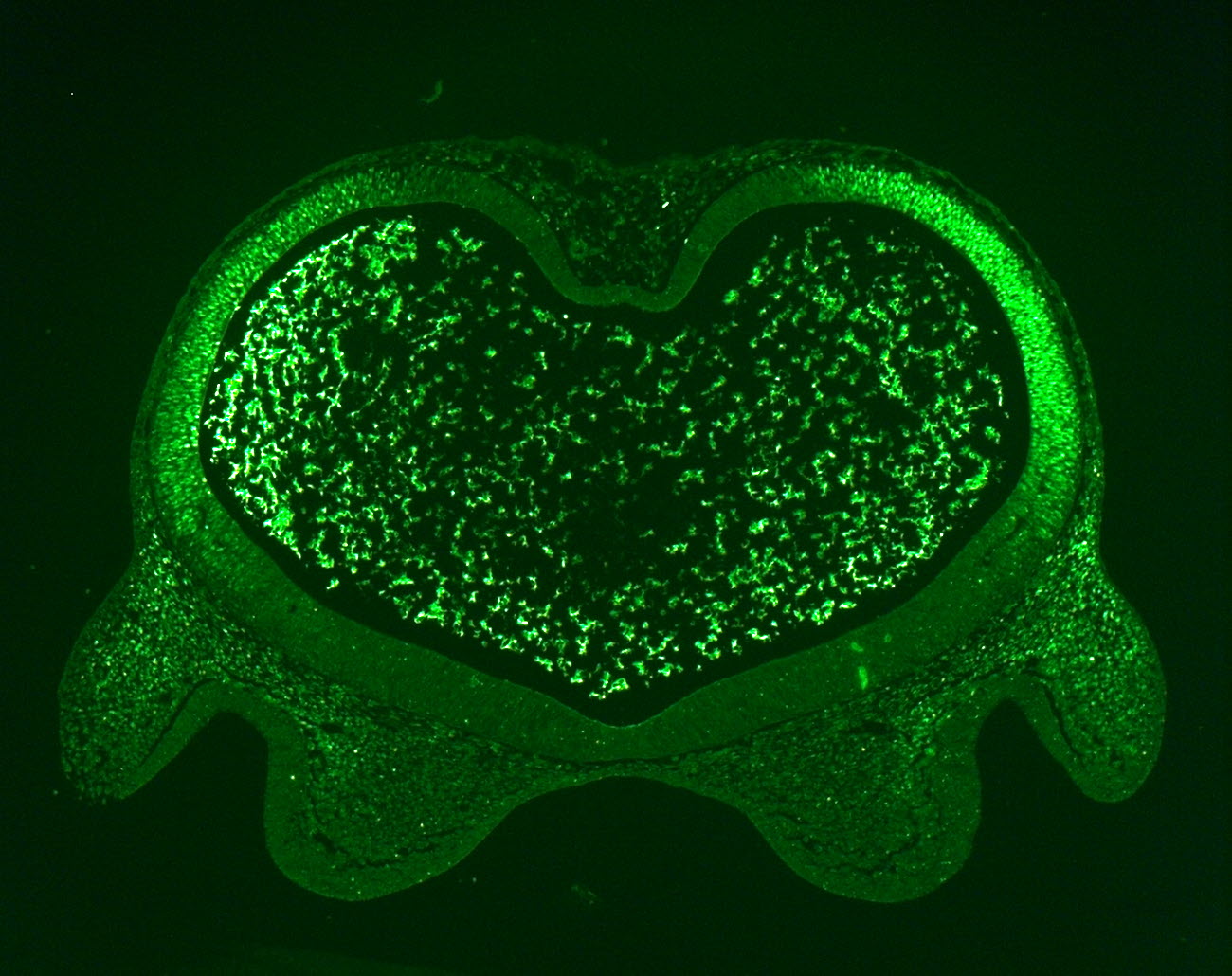

Supplement: Figure 1—figure supplement 1—source data 2. [file elife-86940-fig1-figsupp1-data2.zip › Figure 1-figure supplement 1-source data 2/F5391-3-E10-CI-100X-5X-35-G300.JPG]

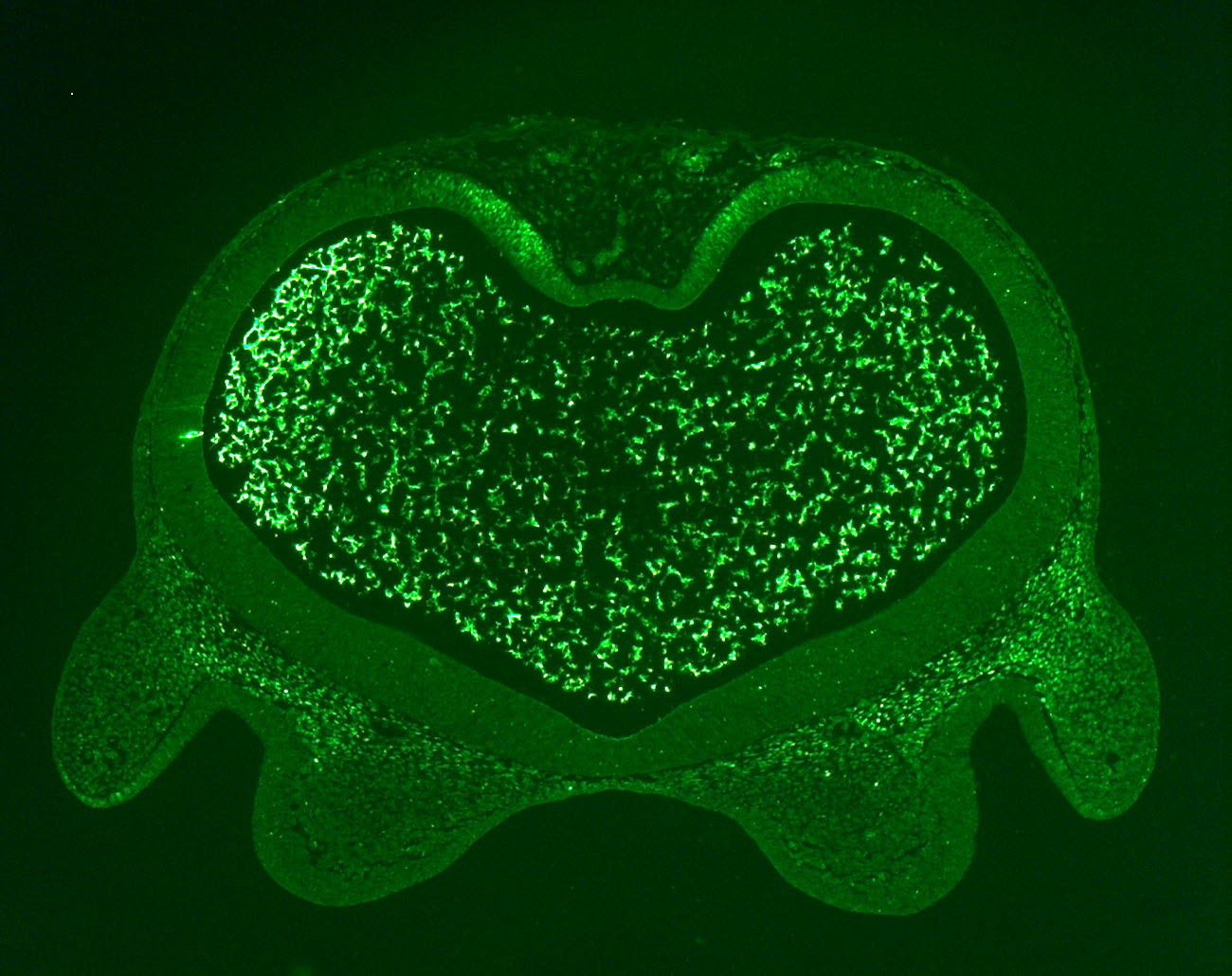

Supplement: Figure 1—figure supplement 1—source data 2. [file elife-86940-fig1-figsupp1-data2.zip › Figure 1-figure supplement 1-source data 2/F5391-3-E10-CII-100X-5X-35-G400.JPG]
