# Supplementary figures and images for: The differentiation and integration of the hippocampal dorsoventral axis are controlled by two nuclear receptor genes (part 2 of 6)
Source: eLife. 2023 Sep 26;12:RP86940. doi: 10.7554/eLife.86940 (PMC10522401; doi:10.7554/eLife.86940)

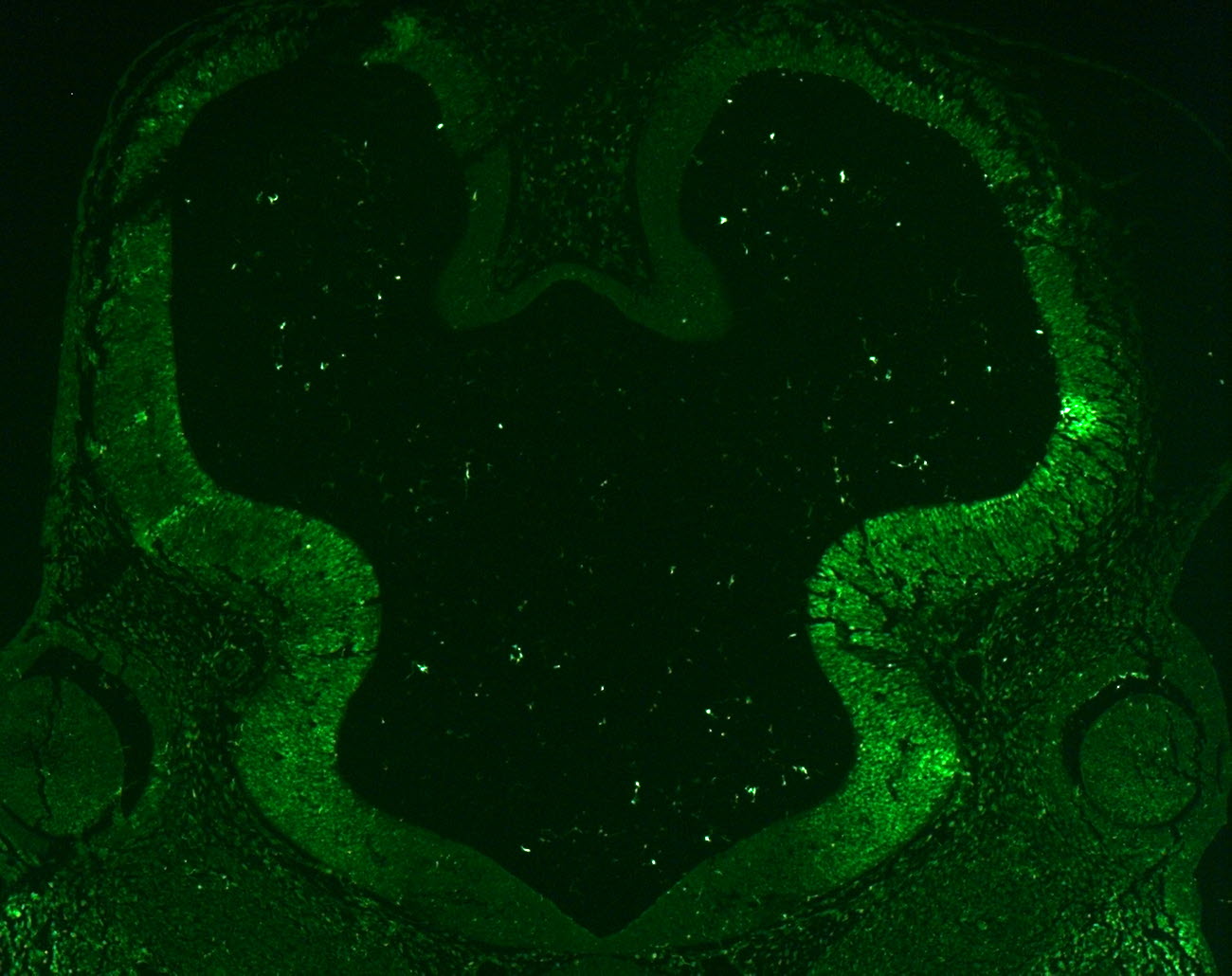

Supplement: Figure 1—figure supplement 1—source data 2. [file elife-86940-fig1-figsupp1-data2.zip › Figure 1-figure supplement 1-source data 2/F8714-2-E11-CI-100X-5X-30-G200.JPG]

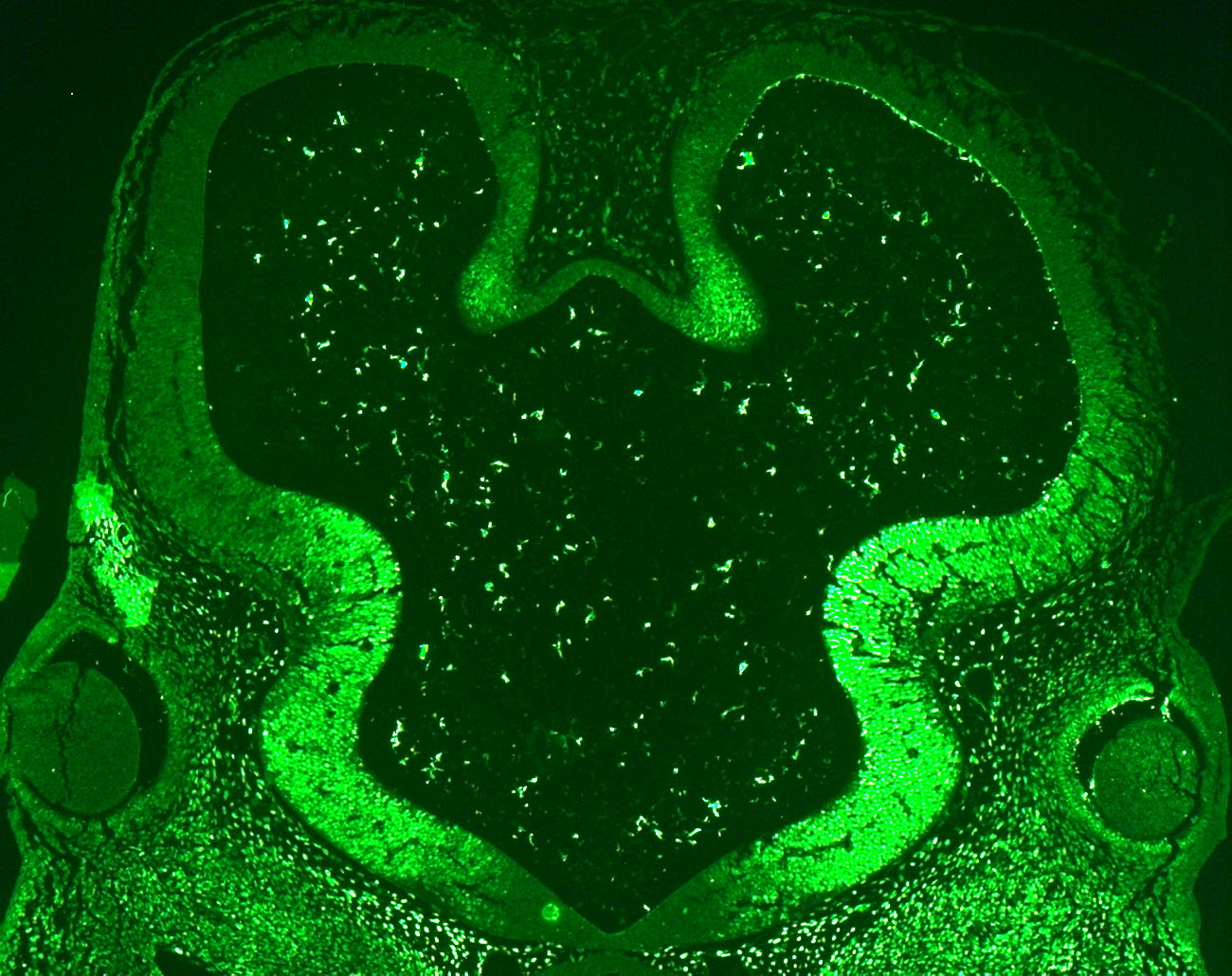

Supplement: Figure 1—figure supplement 1—source data 2. [file elife-86940-fig1-figsupp1-data2.zip › Figure 1-figure supplement 1-source data 2/F8714-2-E11-CII-100X-5X-30-G400.JPG]

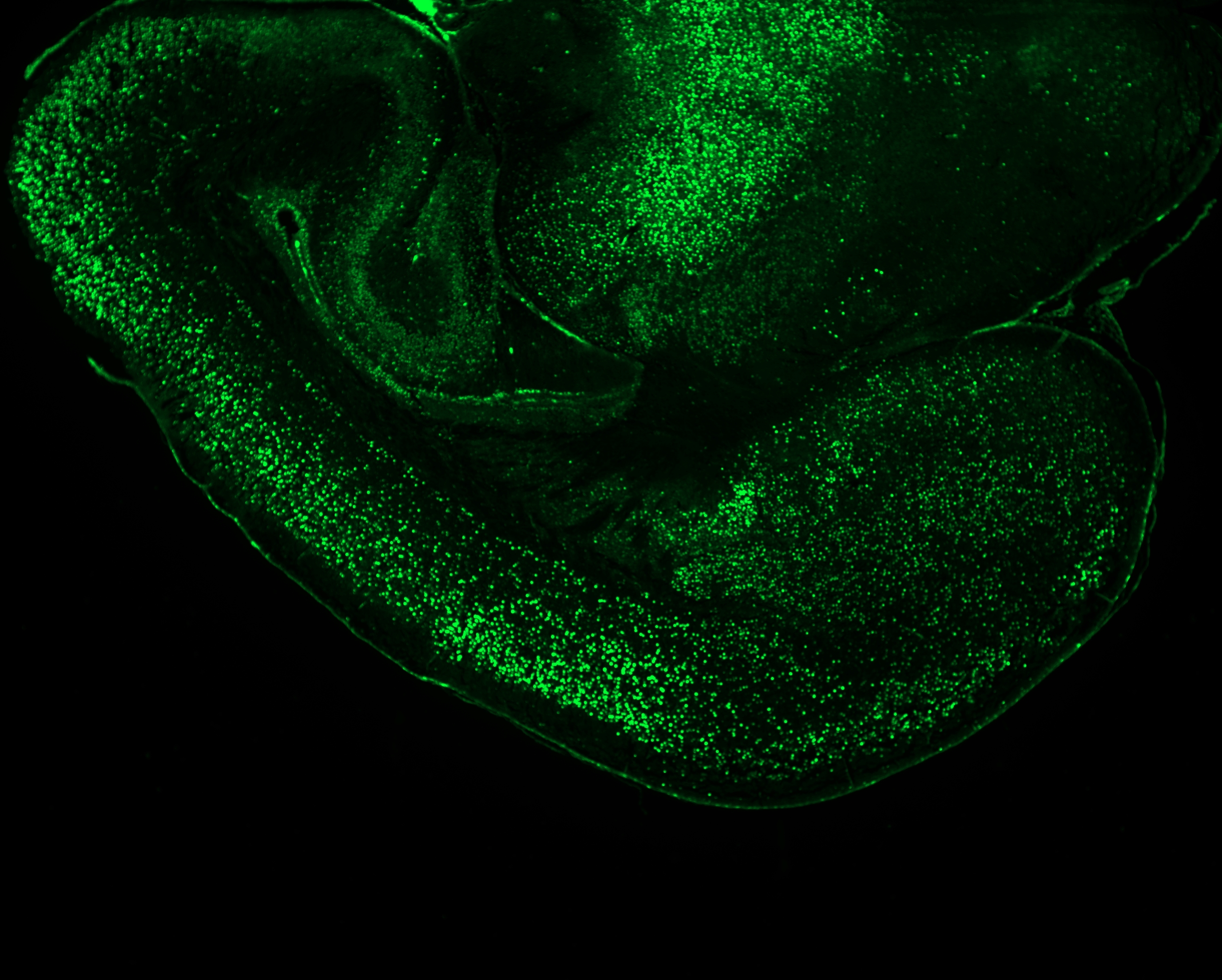

Supplement: Figure 1—figure supplement 1—source data 2. [file elife-86940-fig1-figsupp1-data2.zip › Figure 1-figure supplement 1-source data 2/WT-P0-5X-CI-CII-60-1-L-Image Export-02_AF488.jpg]

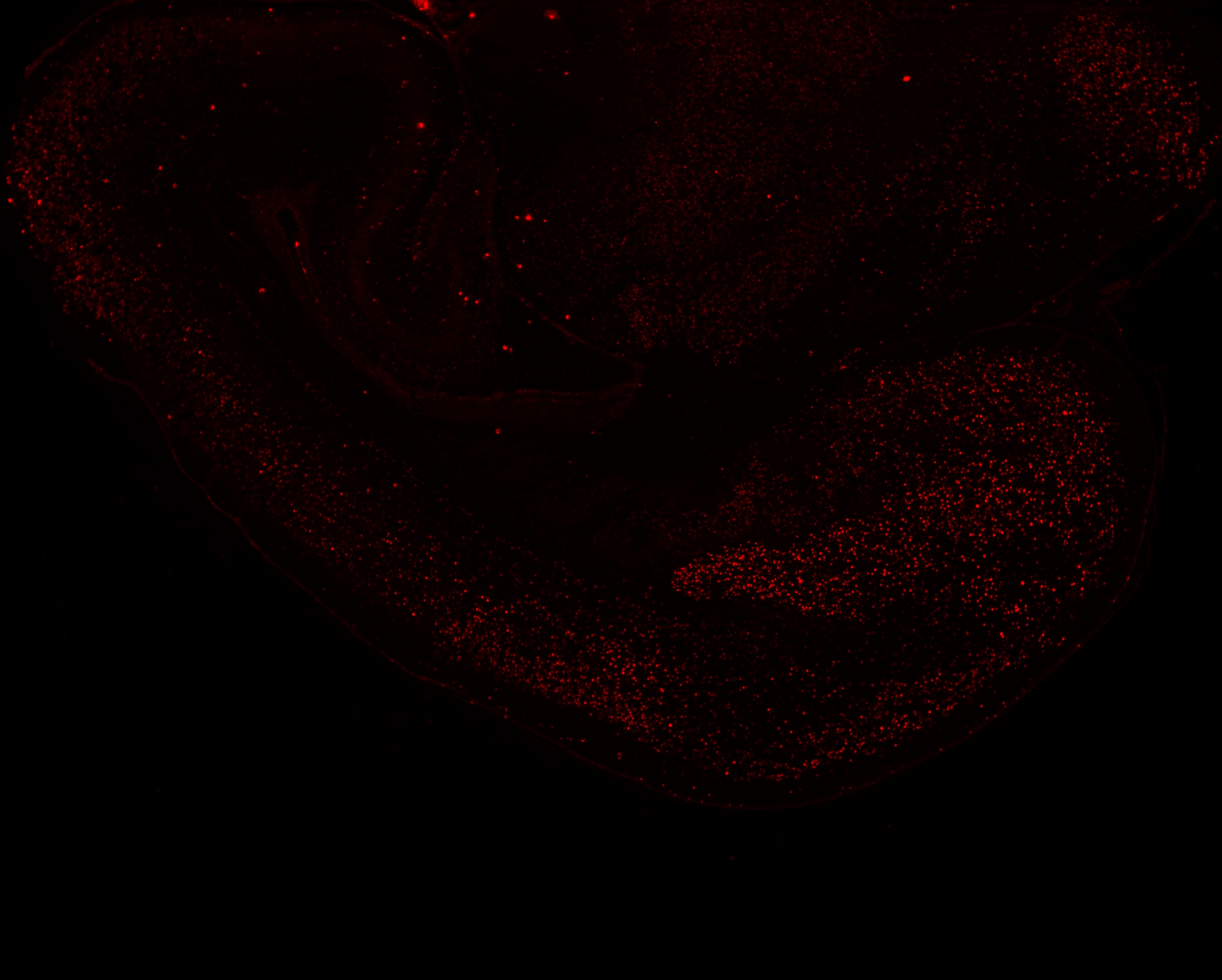

Supplement: Figure 1—figure supplement 1—source data 2. [file elife-86940-fig1-figsupp1-data2.zip › Figure 1-figure supplement 1-source data 2/WT-P0-5X-CI-CII-60-1-L-Image Export-02_AF594.jpg]

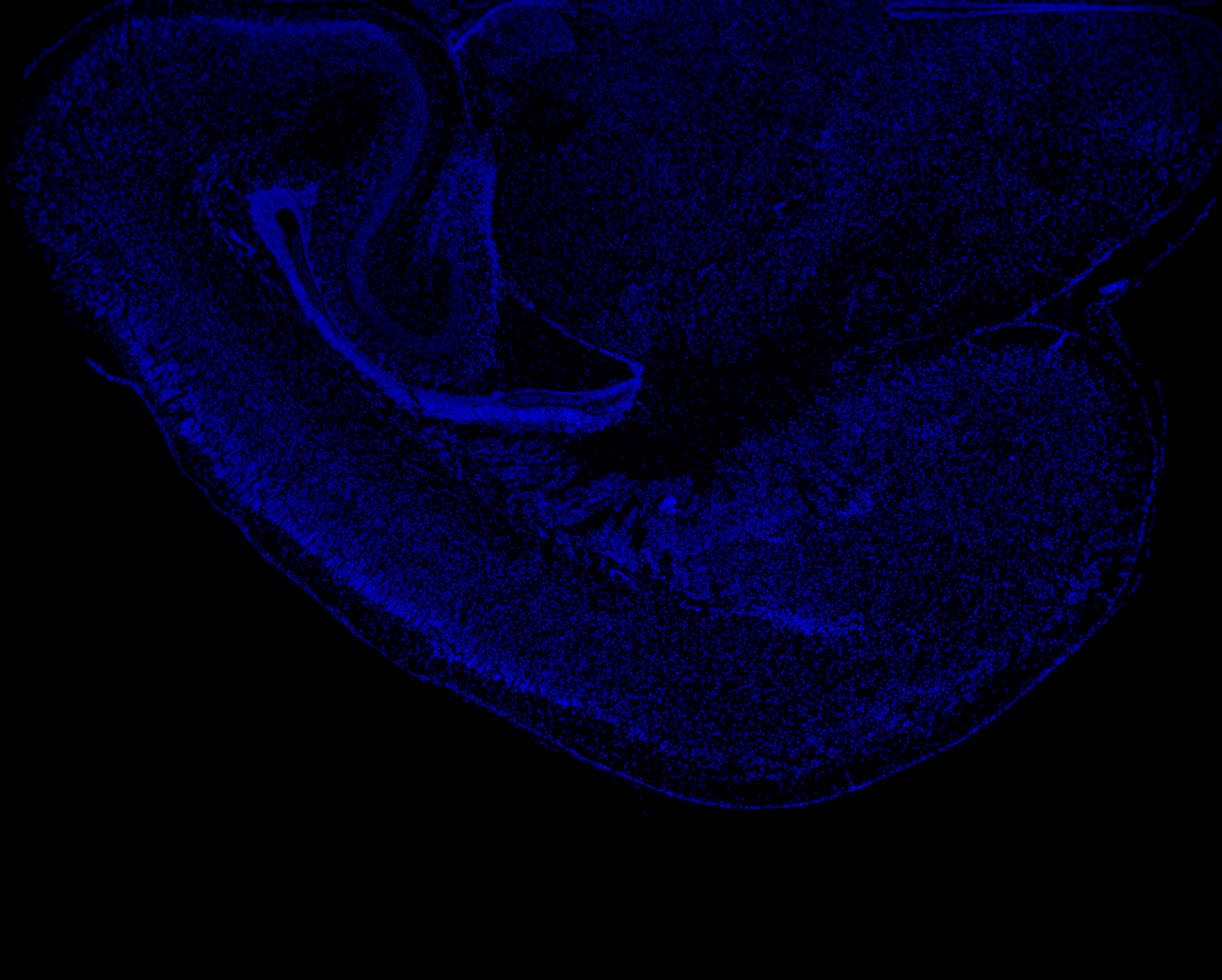

Supplement: Figure 1—figure supplement 1—source data 2. [file elife-86940-fig1-figsupp1-data2.zip › Figure 1-figure supplement 1-source data 2/WT-P0-5X-CI-CII-60-1-L-Image Export-02_DAPI.jpg]

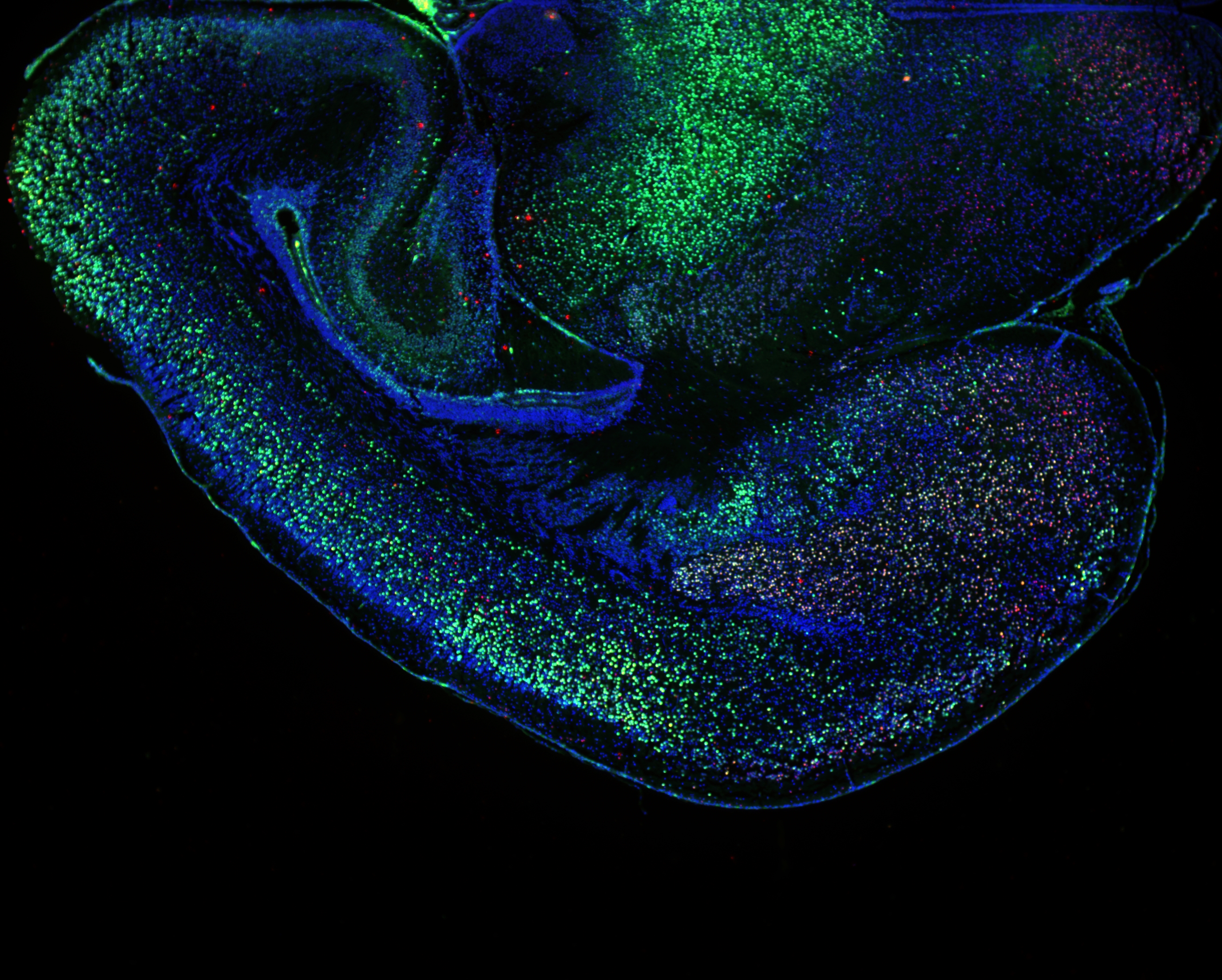

Supplement: Figure 1—figure supplement 1—source data 2. [file elife-86940-fig1-figsupp1-data2.zip › Figure 1-figure supplement 1-source data 2/WT-P0-5X-CI-CII-60-1-L-Image Export-02.jpg]

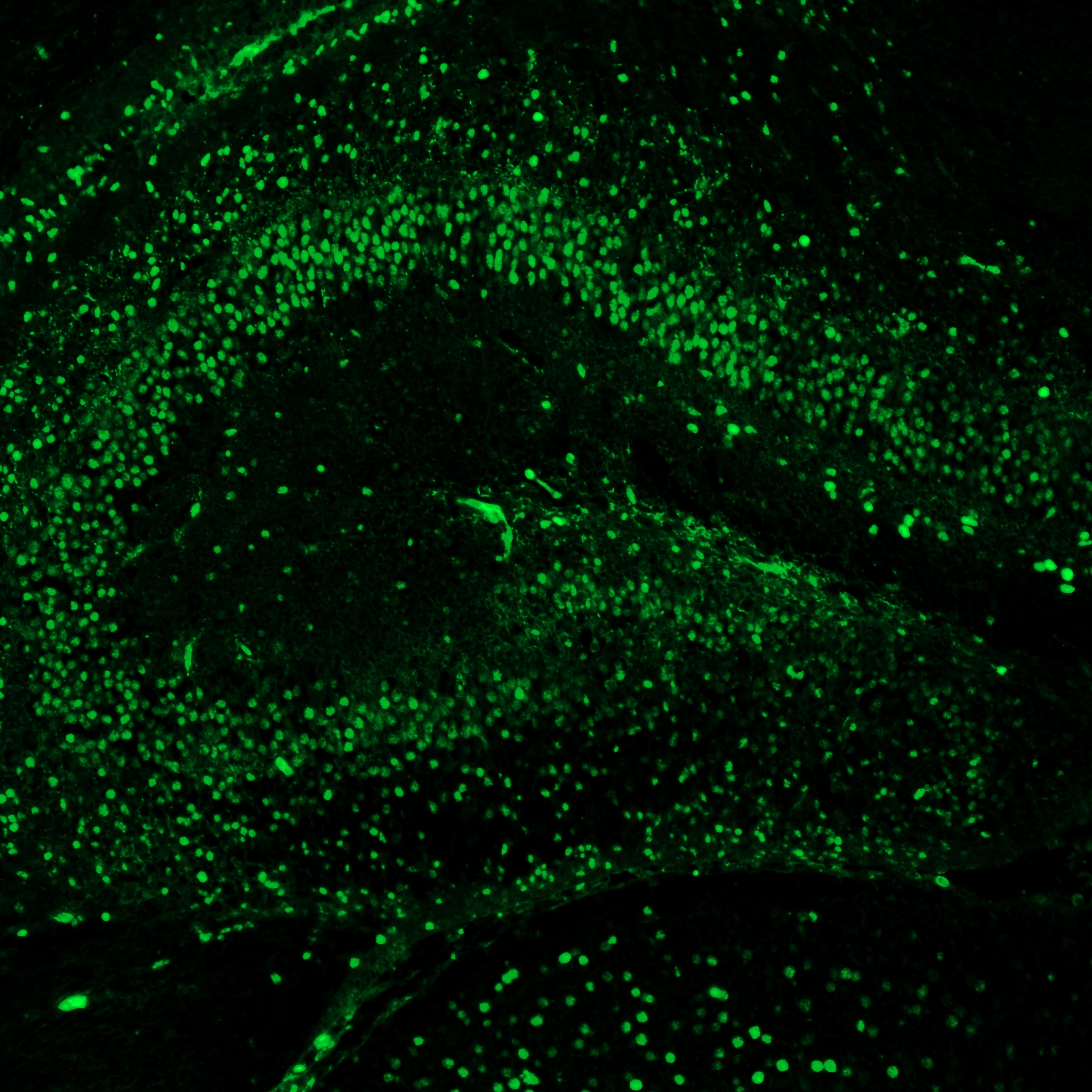

Supplement: Figure 1—figure supplement 1—source data 2. [file elife-86940-fig1-figsupp1-data2.zip › Figure 1-figure supplement 1-source data 2/WT-P0-5X-CI-CII-60-1-LHPC-Image Export-03_AF488.jpg]

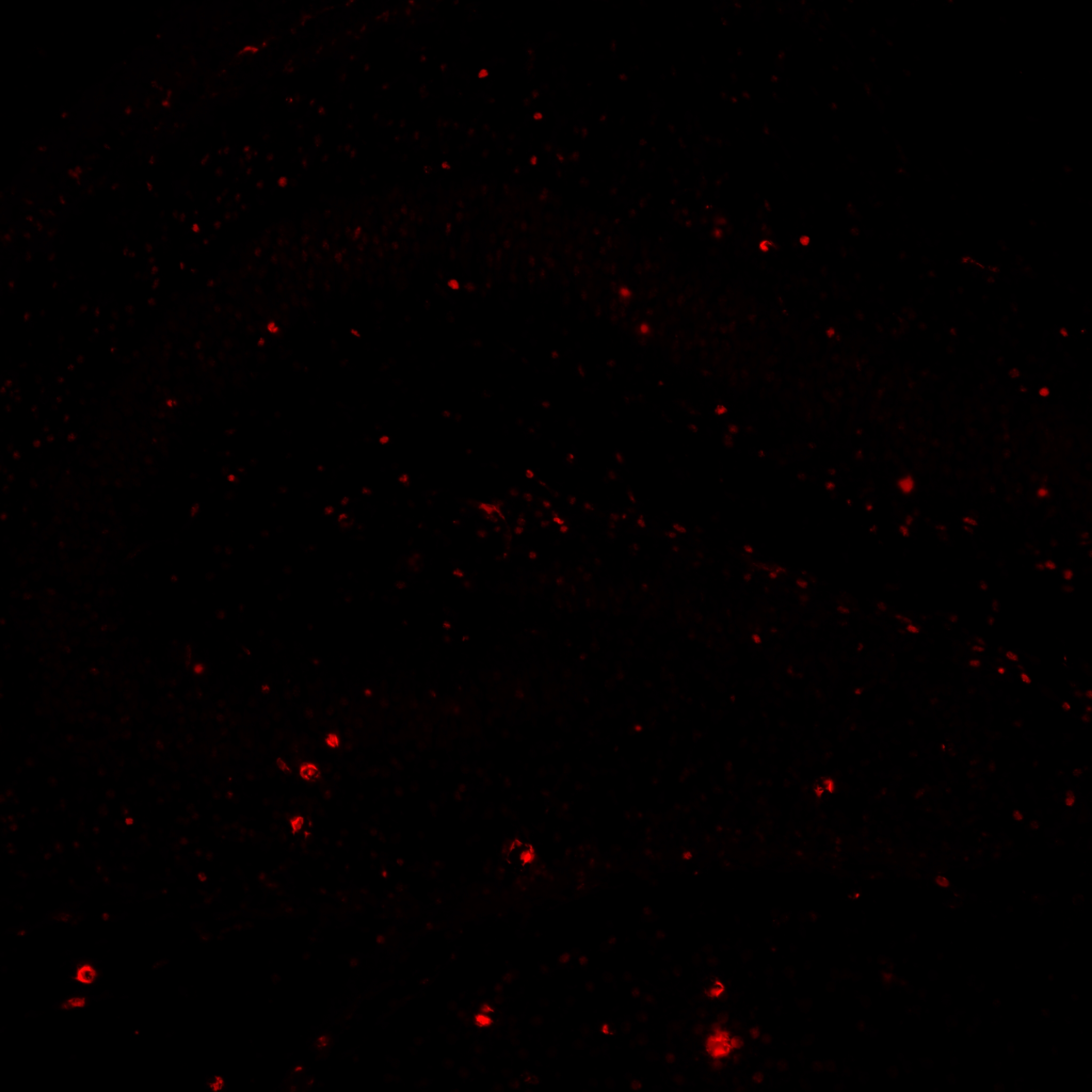

Supplement: Figure 1—figure supplement 1—source data 2. [file elife-86940-fig1-figsupp1-data2.zip › Figure 1-figure supplement 1-source data 2/WT-P0-5X-CI-CII-60-1-LHPC-Image Export-03_AF594.jpg]

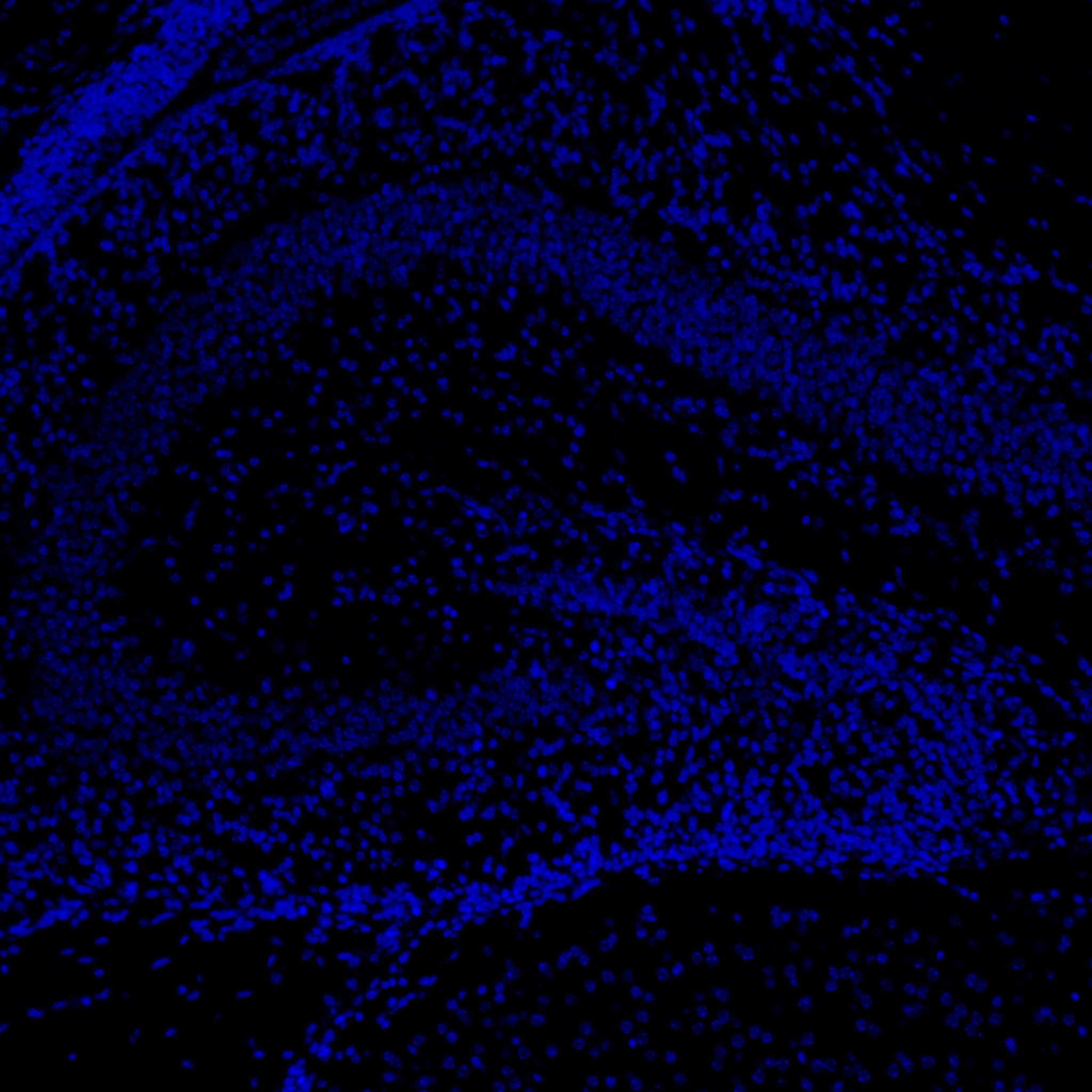

Supplement: Figure 1—figure supplement 1—source data 2. [file elife-86940-fig1-figsupp1-data2.zip › Figure 1-figure supplement 1-source data 2/WT-P0-5X-CI-CII-60-1-LHPC-Image Export-03_DAPI.jpg]

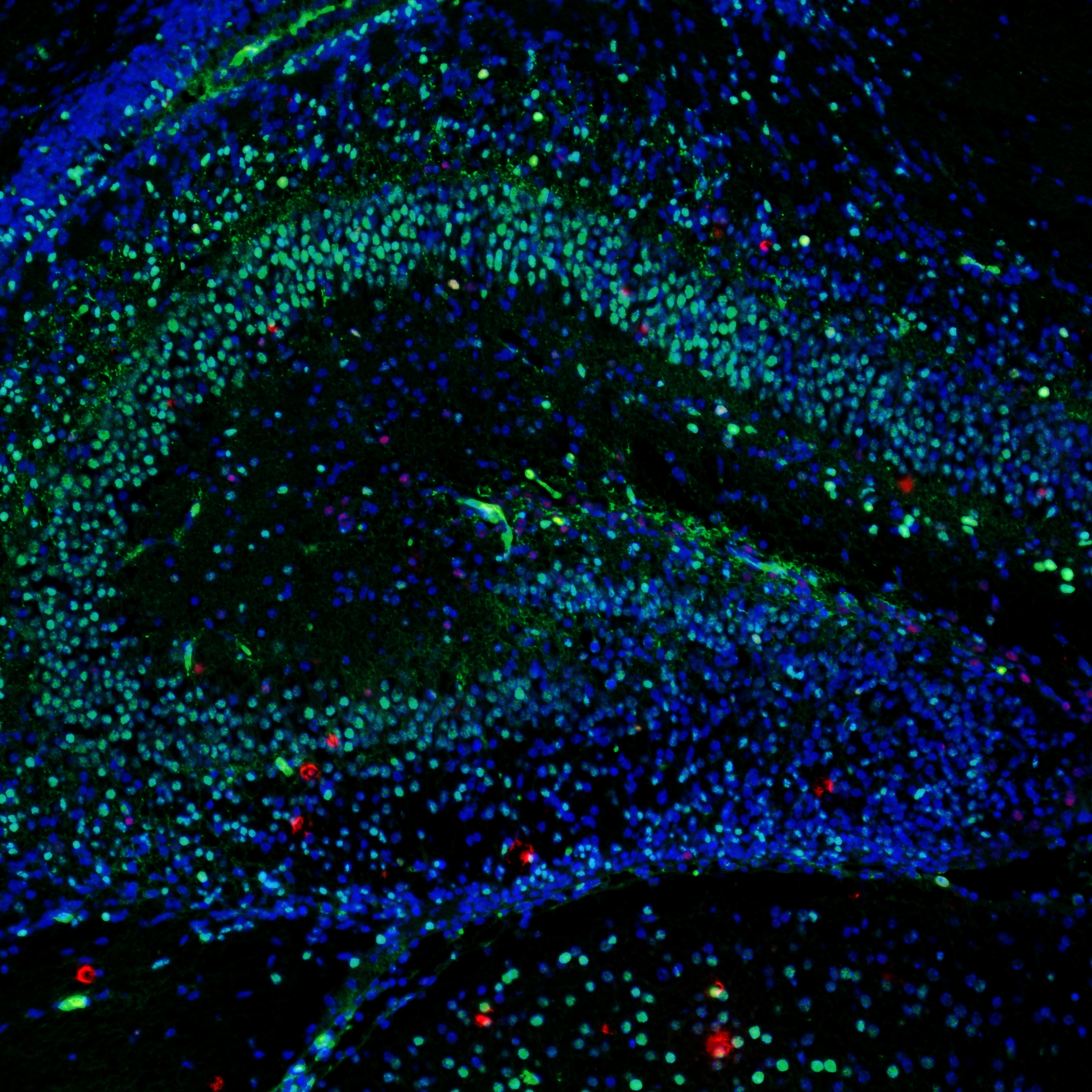

Supplement: Figure 1—figure supplement 1—source data 2. [file elife-86940-fig1-figsupp1-data2.zip › Figure 1-figure supplement 1-source data 2/WT-P0-5X-CI-CII-60-1-LHPC-Image Export-03.jpg]

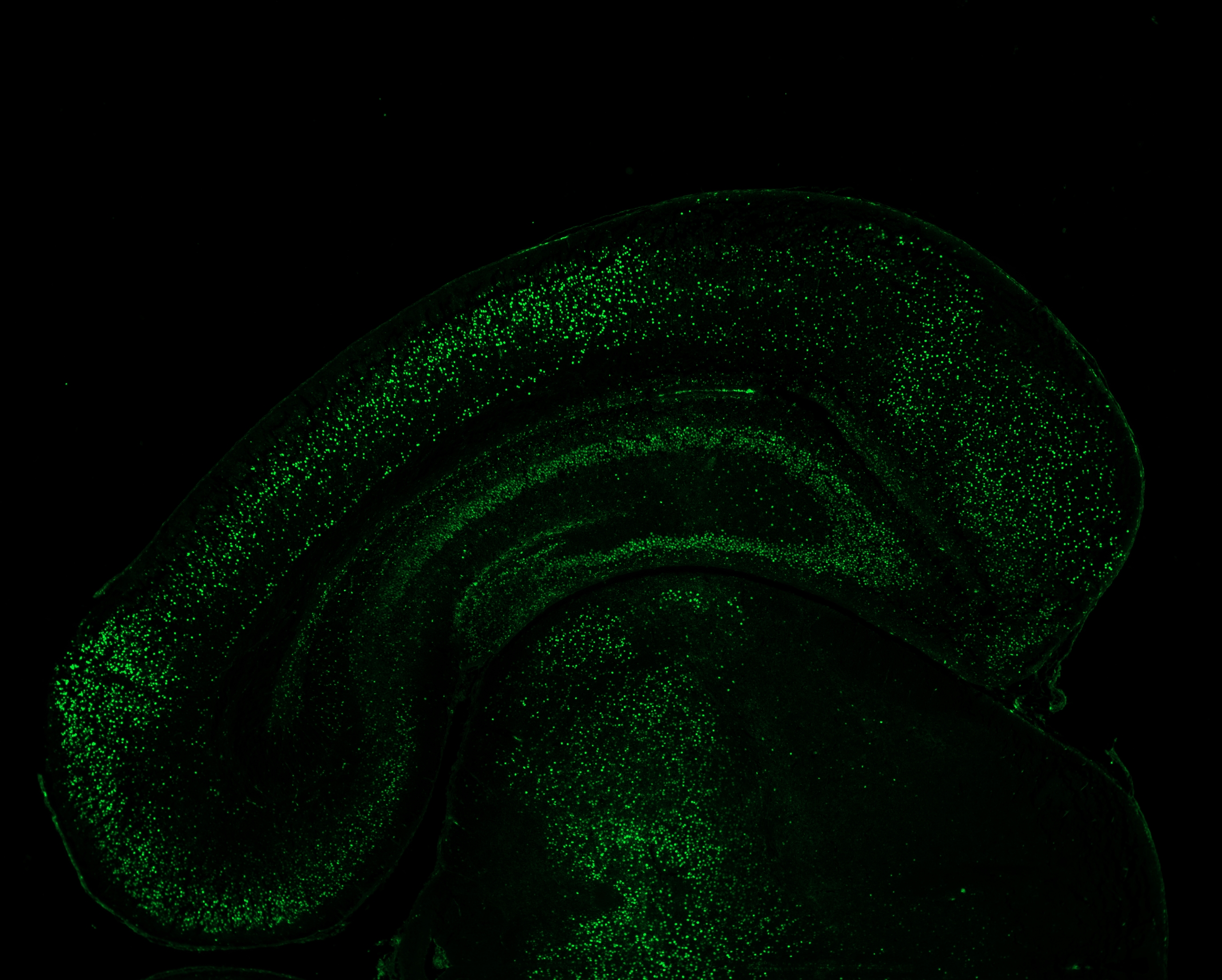

Supplement: Figure 1—figure supplement 1—source data 2. [file elife-86940-fig1-figsupp1-data2.zip › Figure 1-figure supplement 1-source data 2/WT-P0-5X-CI-CII-78-1-R-Image Export-14_AF488.jpg]

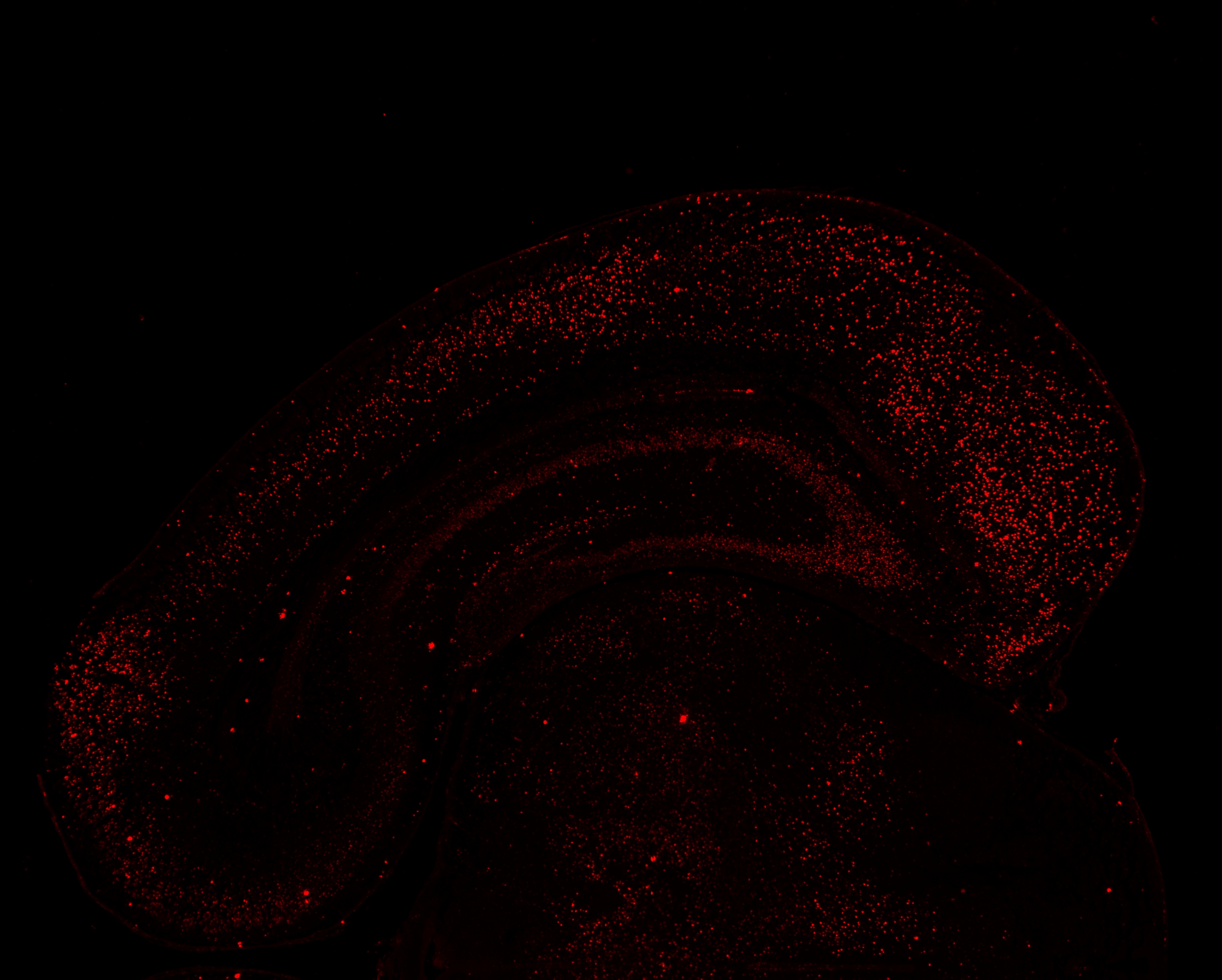

Supplement: Figure 1—figure supplement 1—source data 2. [file elife-86940-fig1-figsupp1-data2.zip › Figure 1-figure supplement 1-source data 2/WT-P0-5X-CI-CII-78-1-R-Image Export-14_AF594.jpg]

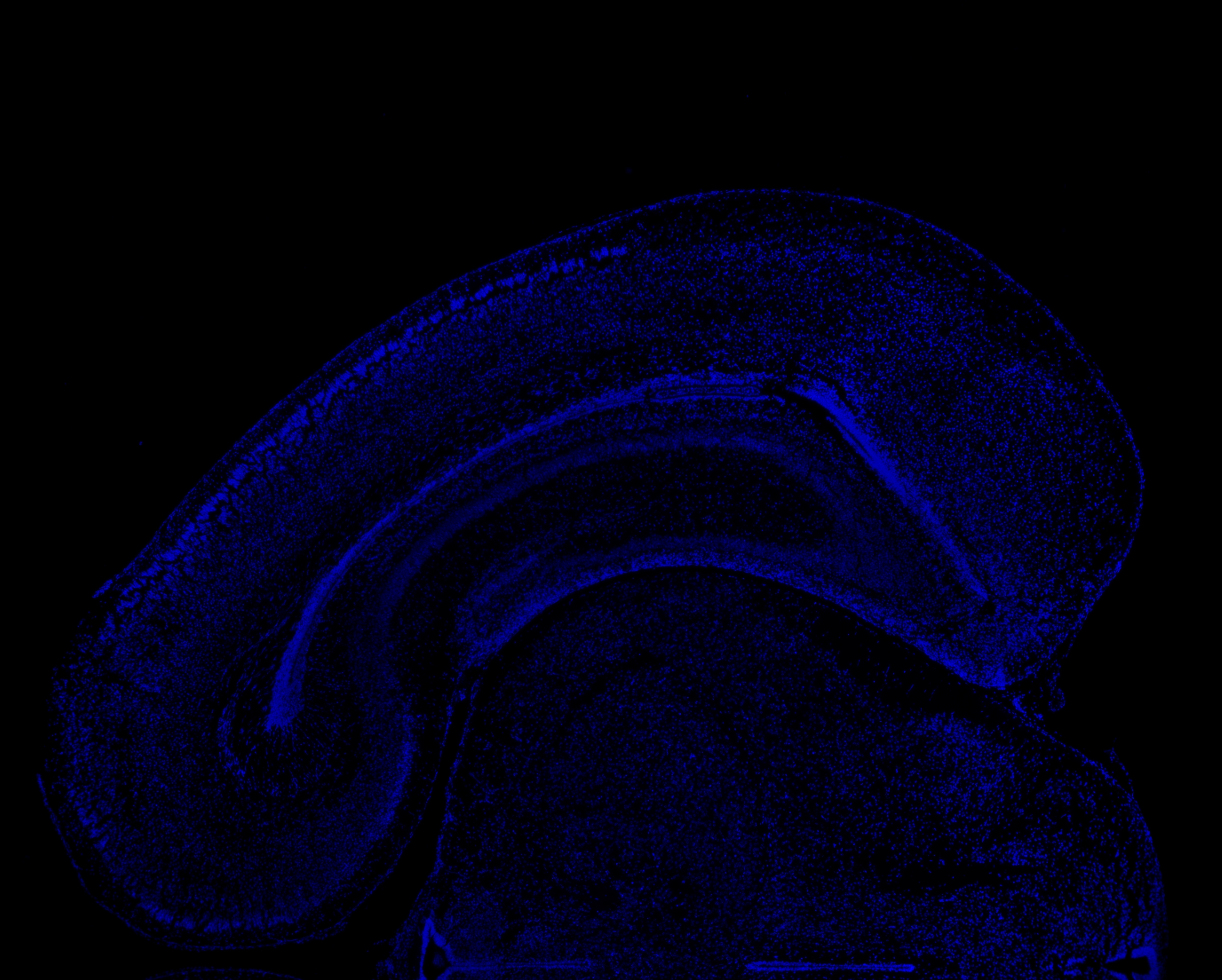

Supplement: Figure 1—figure supplement 1—source data 2. [file elife-86940-fig1-figsupp1-data2.zip › Figure 1-figure supplement 1-source data 2/WT-P0-5X-CI-CII-78-1-R-Image Export-14_DAPI.jpg]

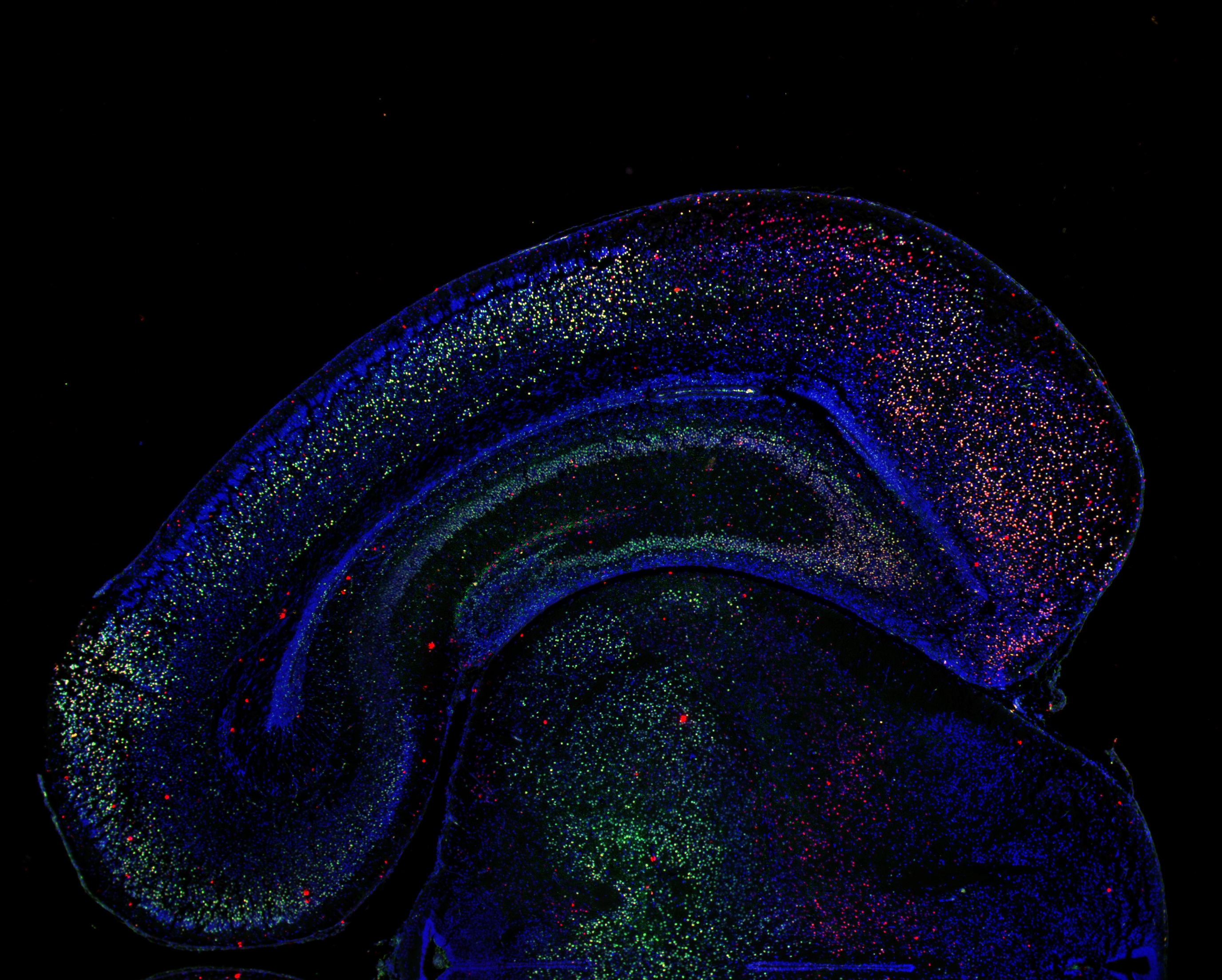

Supplement: Figure 1—figure supplement 1—source data 2. [file elife-86940-fig1-figsupp1-data2.zip › Figure 1-figure supplement 1-source data 2/WT-P0-CI-CII-78-1-R-Image Export-14.jpg]

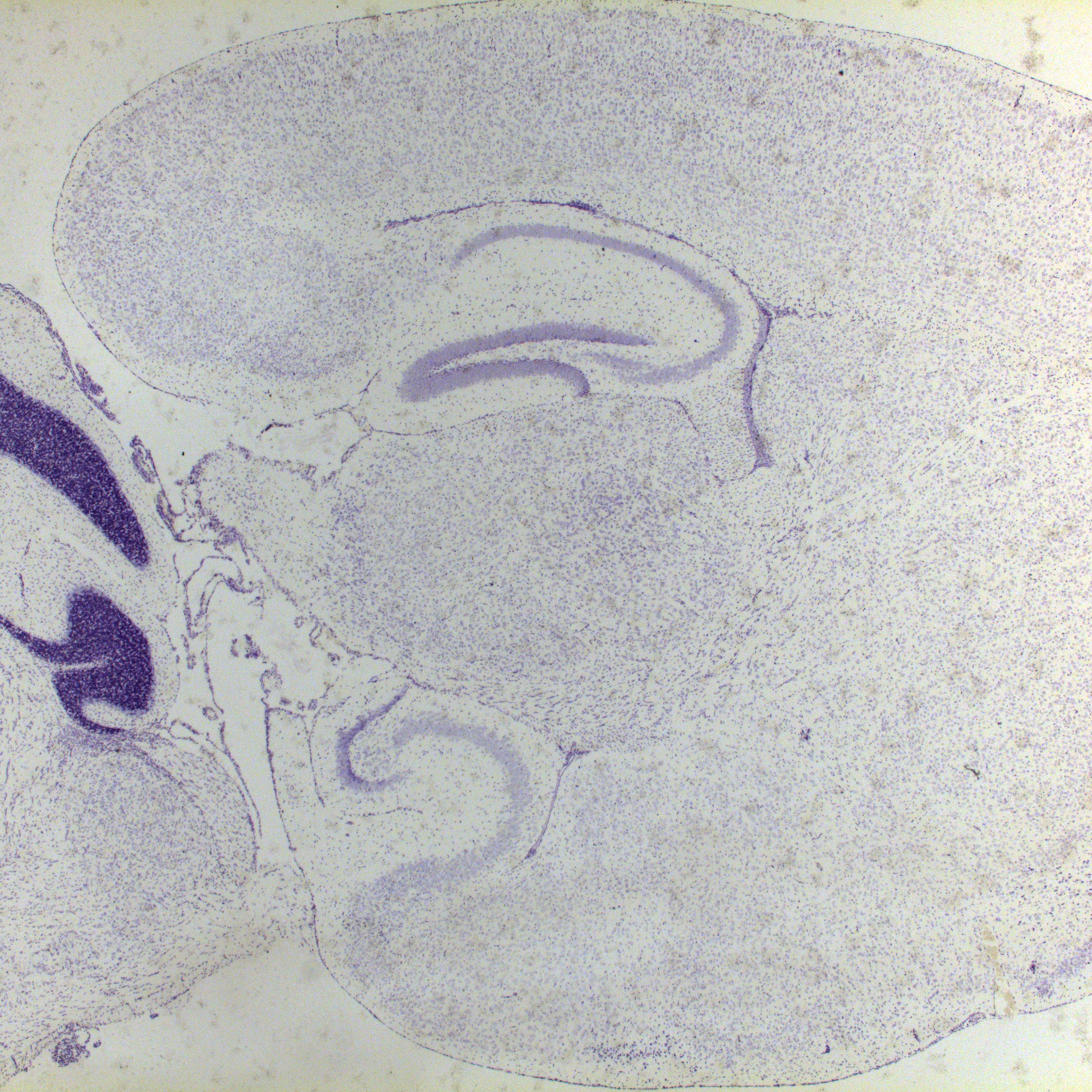

Supplement: Figure 2—source data 1. [file elife-86940-fig2-data1.zip › Figure 2-source data 1/F3092-2-CON-2.5X-RX CI f+-1M-#36-2-Image Export-03.tif]

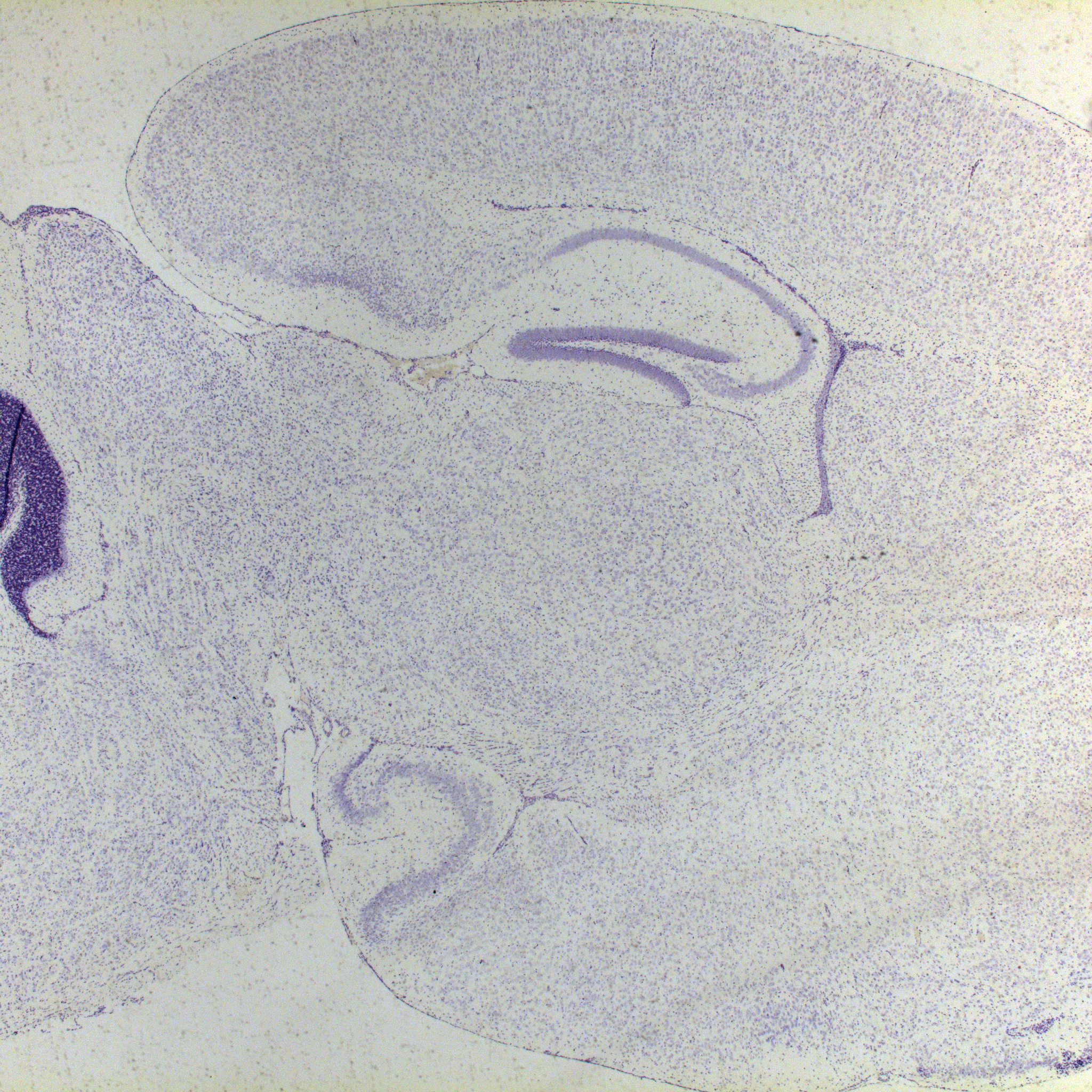

Supplement: Figure 2—source data 1. [file elife-86940-fig2-data1.zip › Figure 2-source data 1/F3092-2-CON-2.5X-RX CI f+-1M-#44-2-Image Export-04.tif]

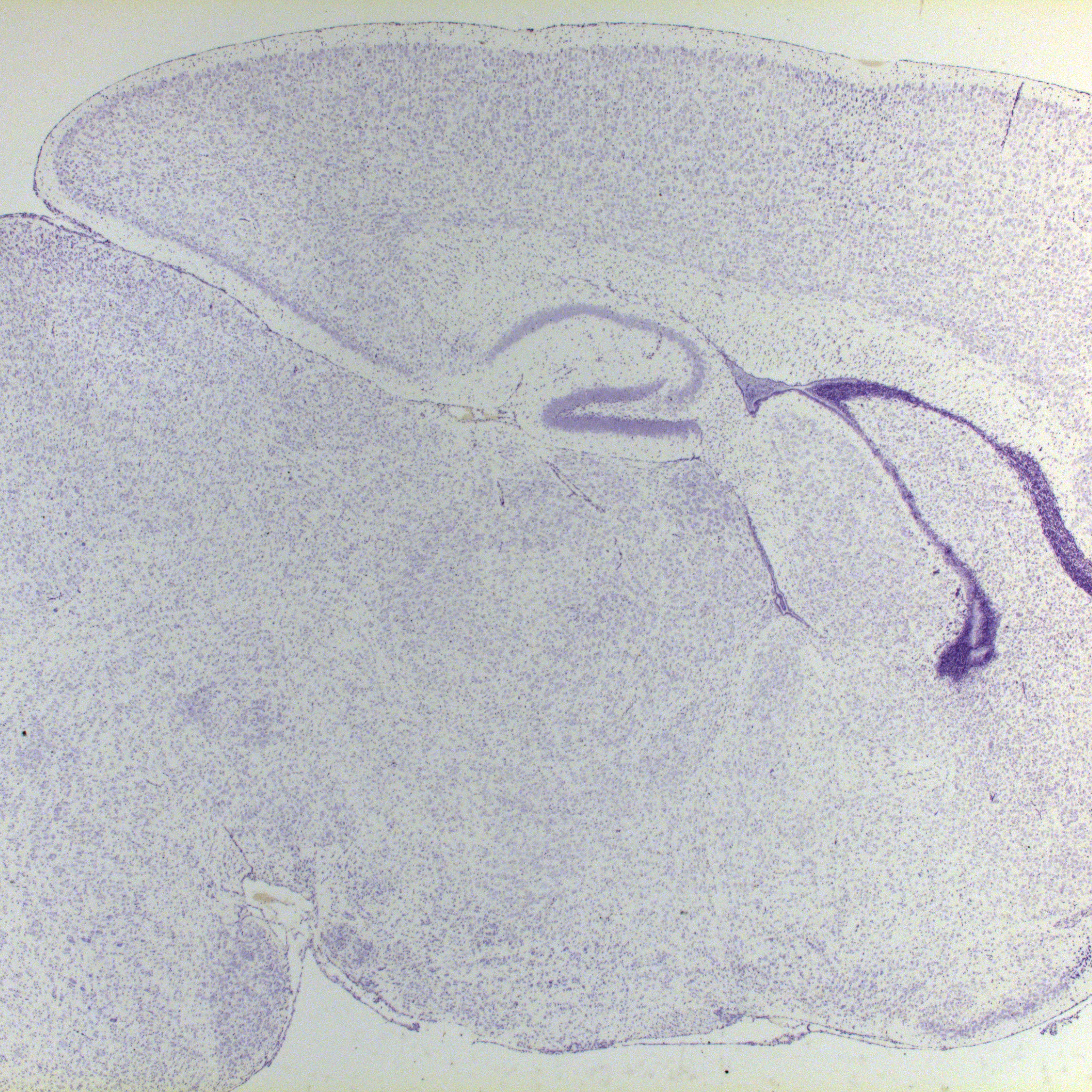

Supplement: Figure 2—source data 1. [file elife-86940-fig2-data1.zip › Figure 2-source data 1/F3092-2-CON-2.5X-RX CI f+-1M-#69-2-Image Export-07.tif]

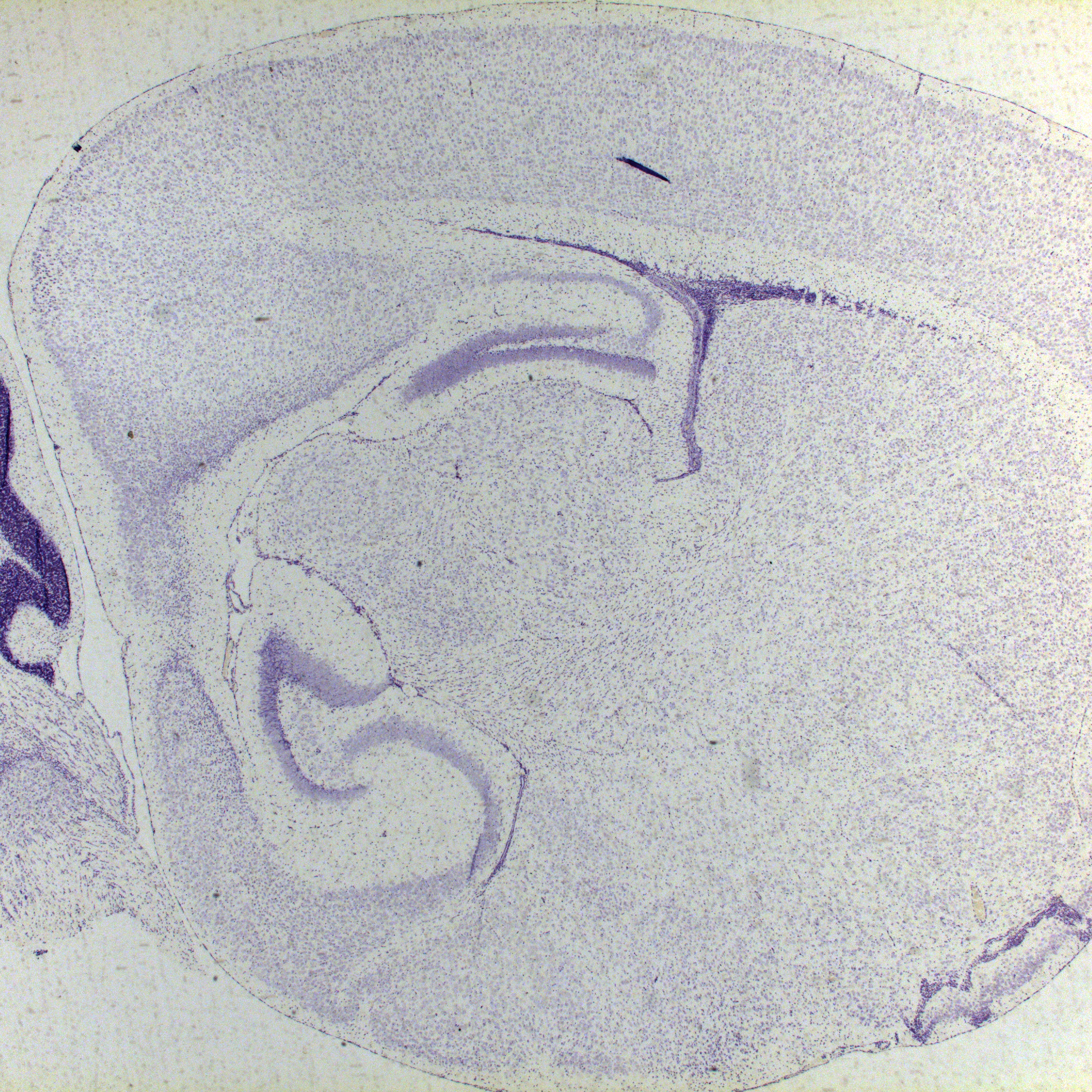

Supplement: Figure 2—source data 1. [file elife-86940-fig2-data1.zip › Figure 2-source data 1/F3094-3-CI CKO-2.5X-RX CI ff-1M-#29-2-Image Export-12.tif]

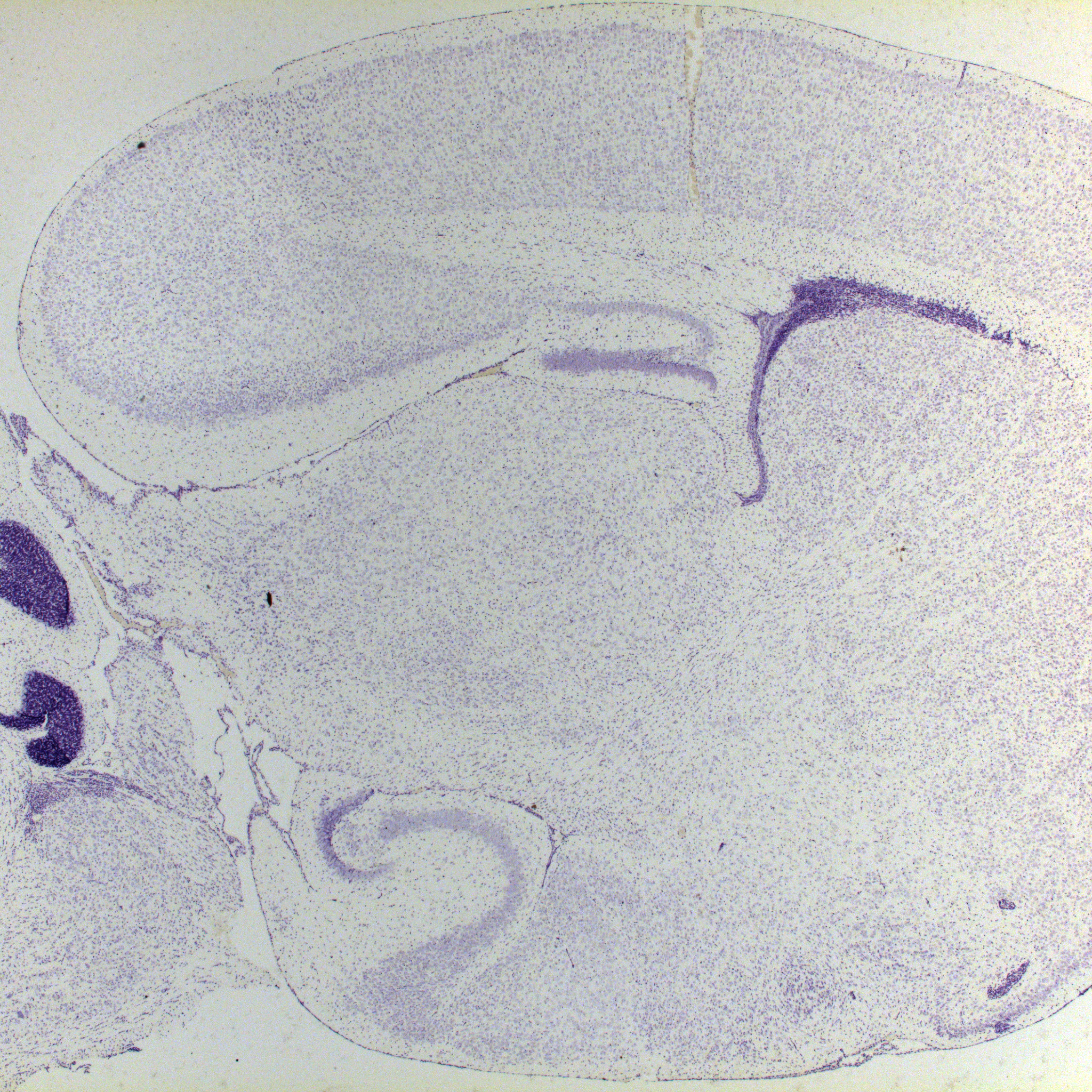

Supplement: Figure 2—source data 1. [file elife-86940-fig2-data1.zip › Figure 2-source data 1/F3094-3-CI CKO-2.5X-RX CI ff-1M-#34-2-Image Export-13.tif]

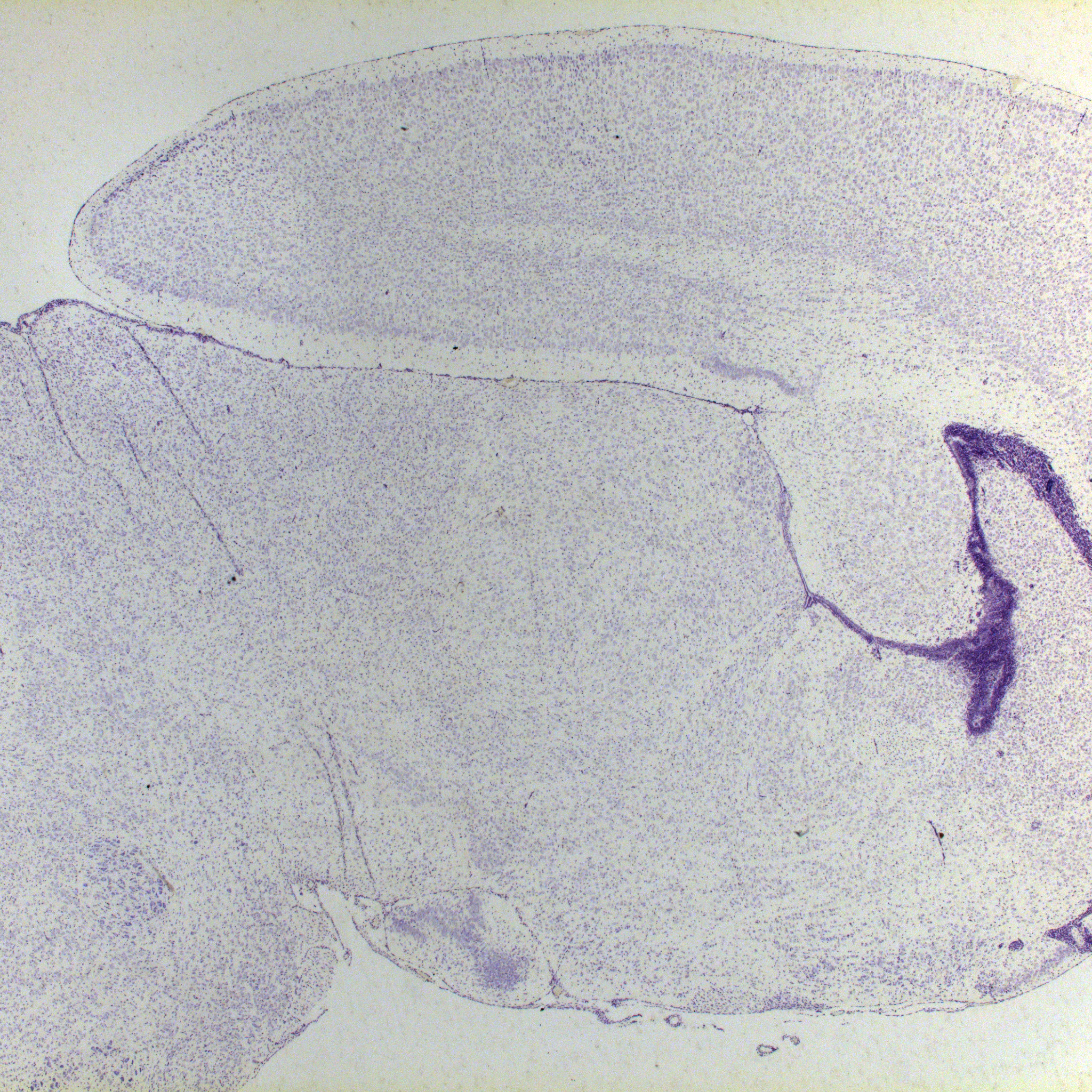

Supplement: Figure 2—source data 1. [file elife-86940-fig2-data1.zip › Figure 2-source data 1/F3094-3-CI CKO-2.5X-RX CI ff-1M-#48-2-Image Export-15.tif]

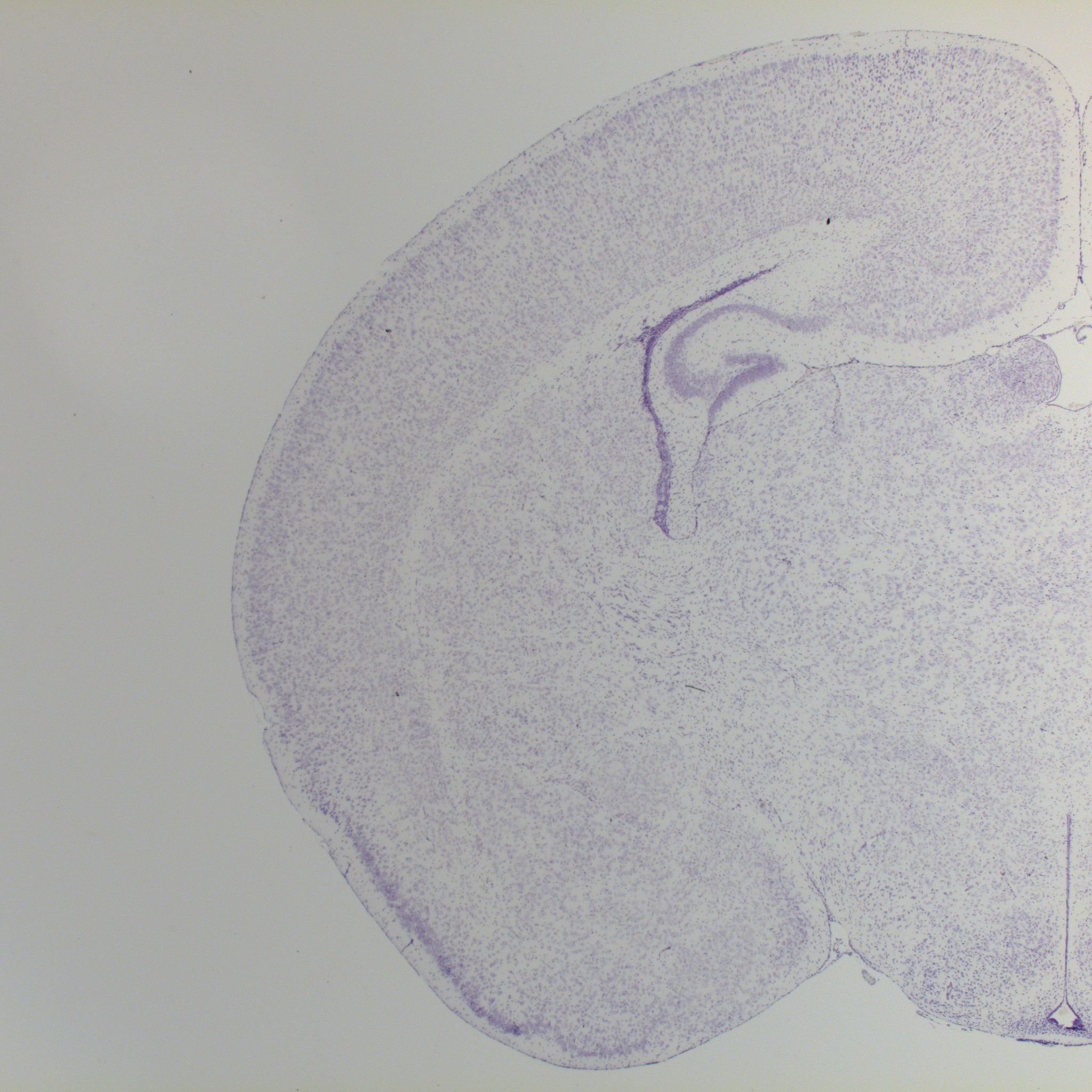

Supplement: Figure 2—source data 1. [file elife-86940-fig2-data1.zip › Figure 2-source data 1/F3094-4-CI CKO-2.5X-RX CI ff-1M-#116-2-Image Export-14.tif]

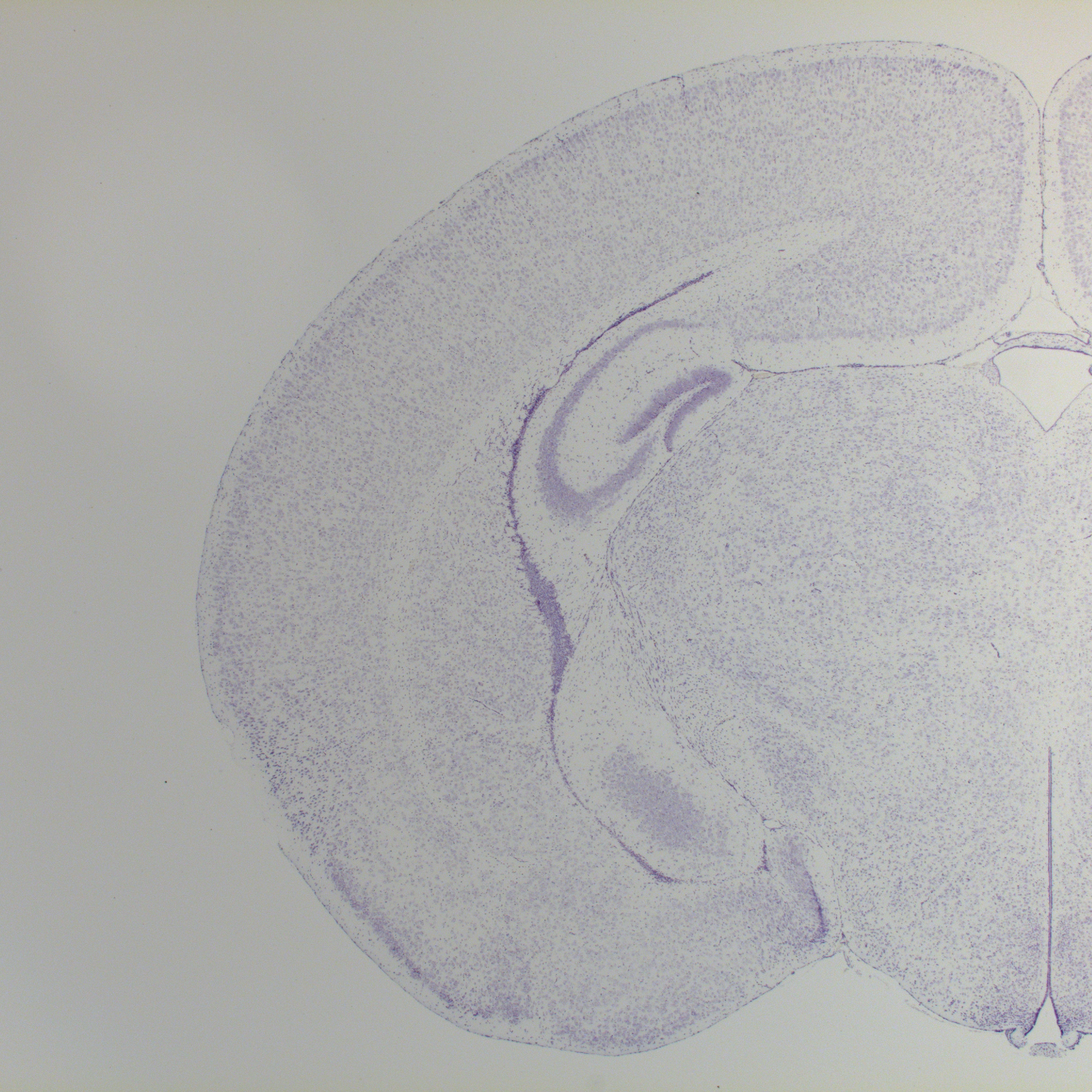

Supplement: Figure 2—source data 1. [file elife-86940-fig2-data1.zip › Figure 2-source data 1/F3094-4-CI CKO-2.5X-RX CI ff-1M-#128-1-Image Export-17.tif]

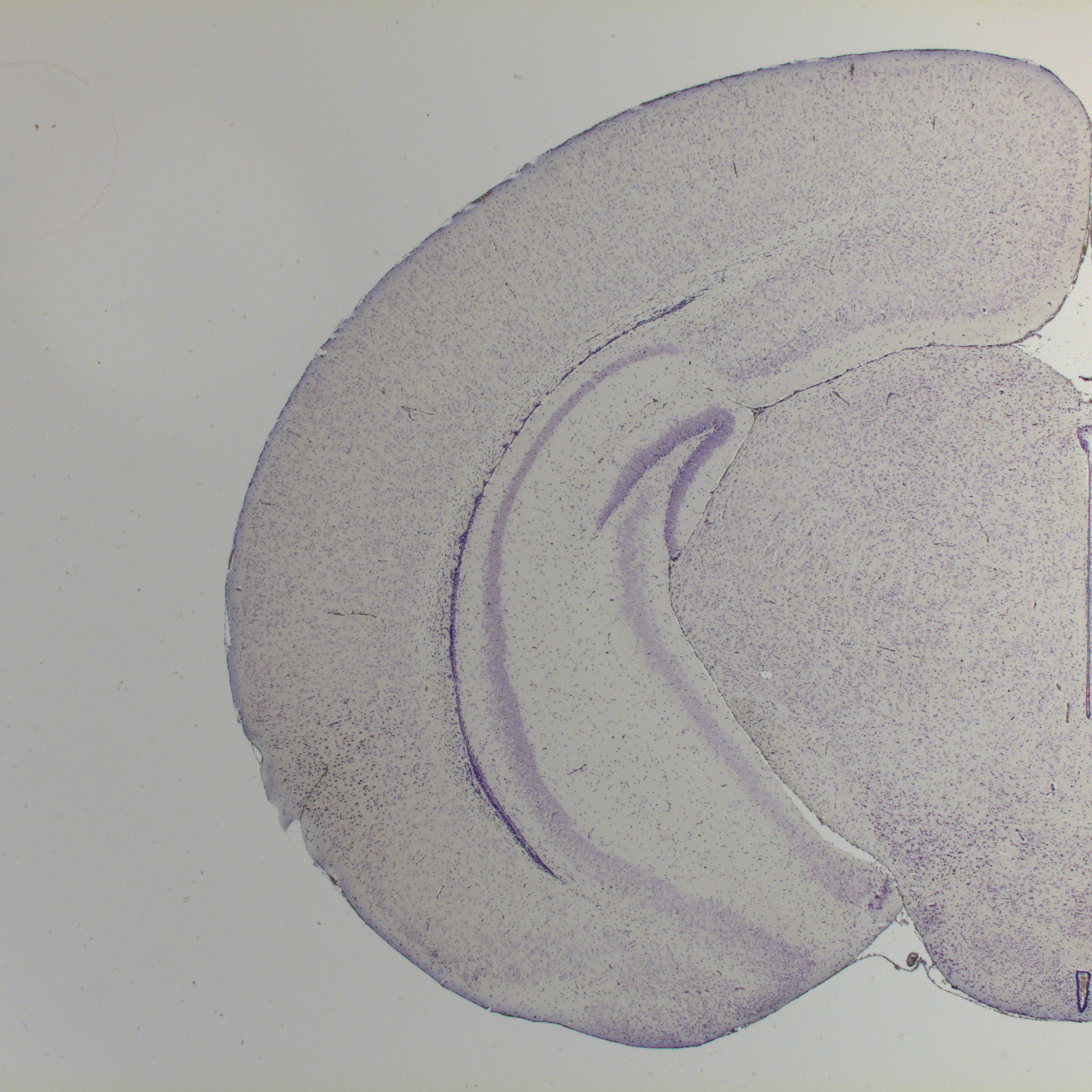

Supplement: Figure 2—source data 1. [file elife-86940-fig2-data1.zip › Figure 2-source data 1/F3094-4-CI CKO-2.5X-RX CI ff-1M-#139-3-Image Export-22.tif]

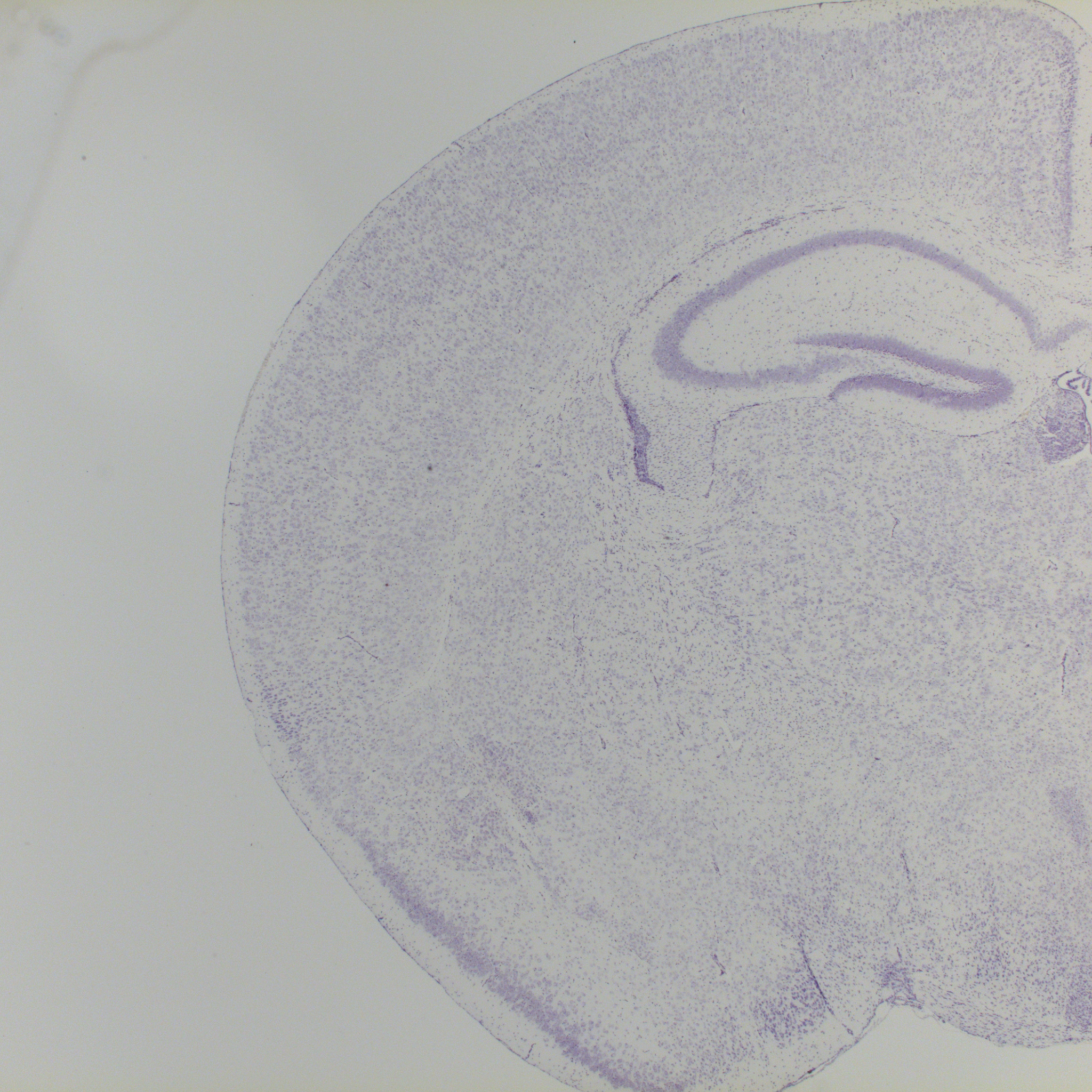

Supplement: Figure 2—source data 2. [file elife-86940-fig2-data2.zip › Figure 2-source data 2/F3094-5-CON-2.5X-CI f+-1M-#126-1-Image Export-04.tif]

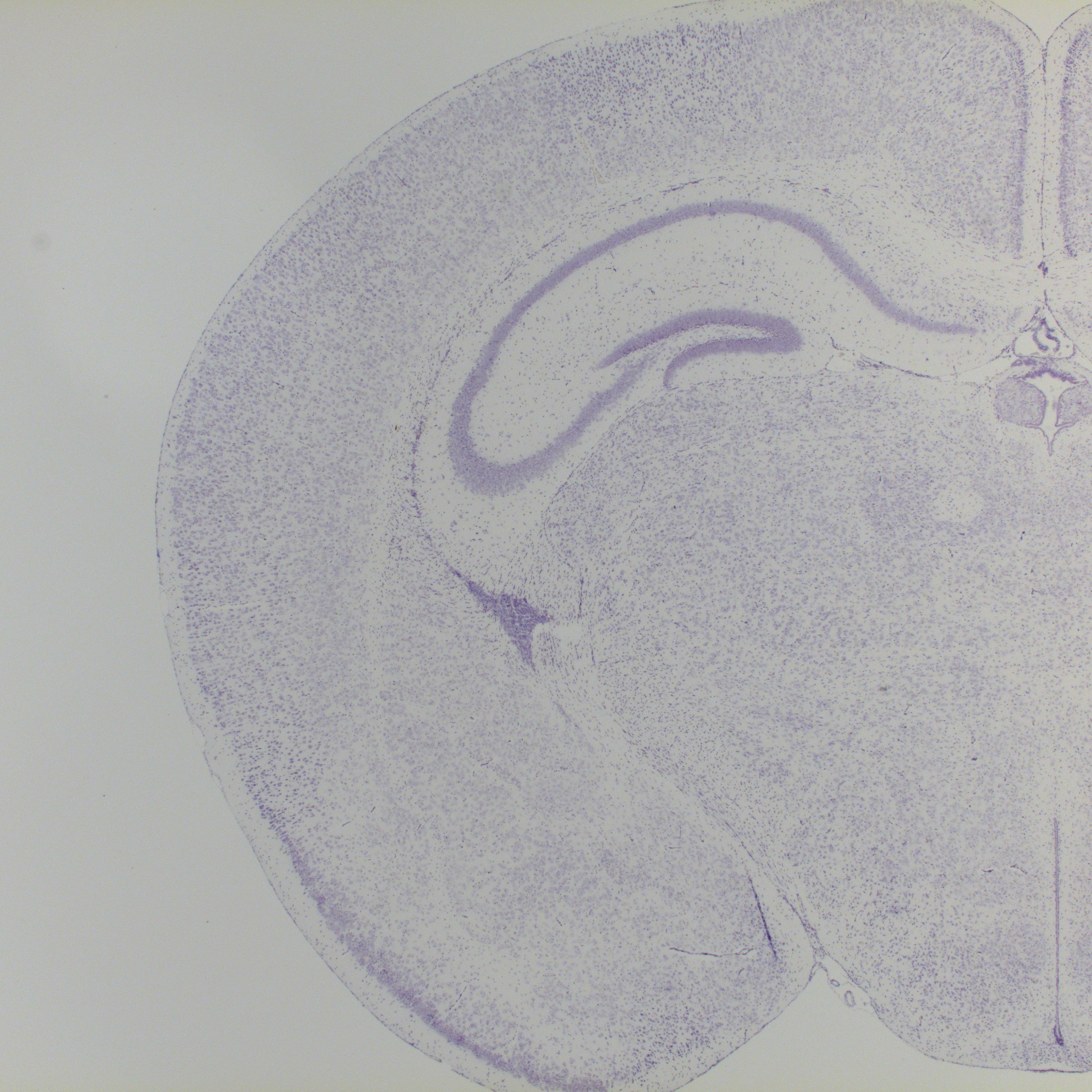

Supplement: Figure 2—source data 2. [file elife-86940-fig2-data2.zip › Figure 2-source data 2/F3094-5-CON-2.5X-CI f+-1M-#146-1-Image Export-07.tif]

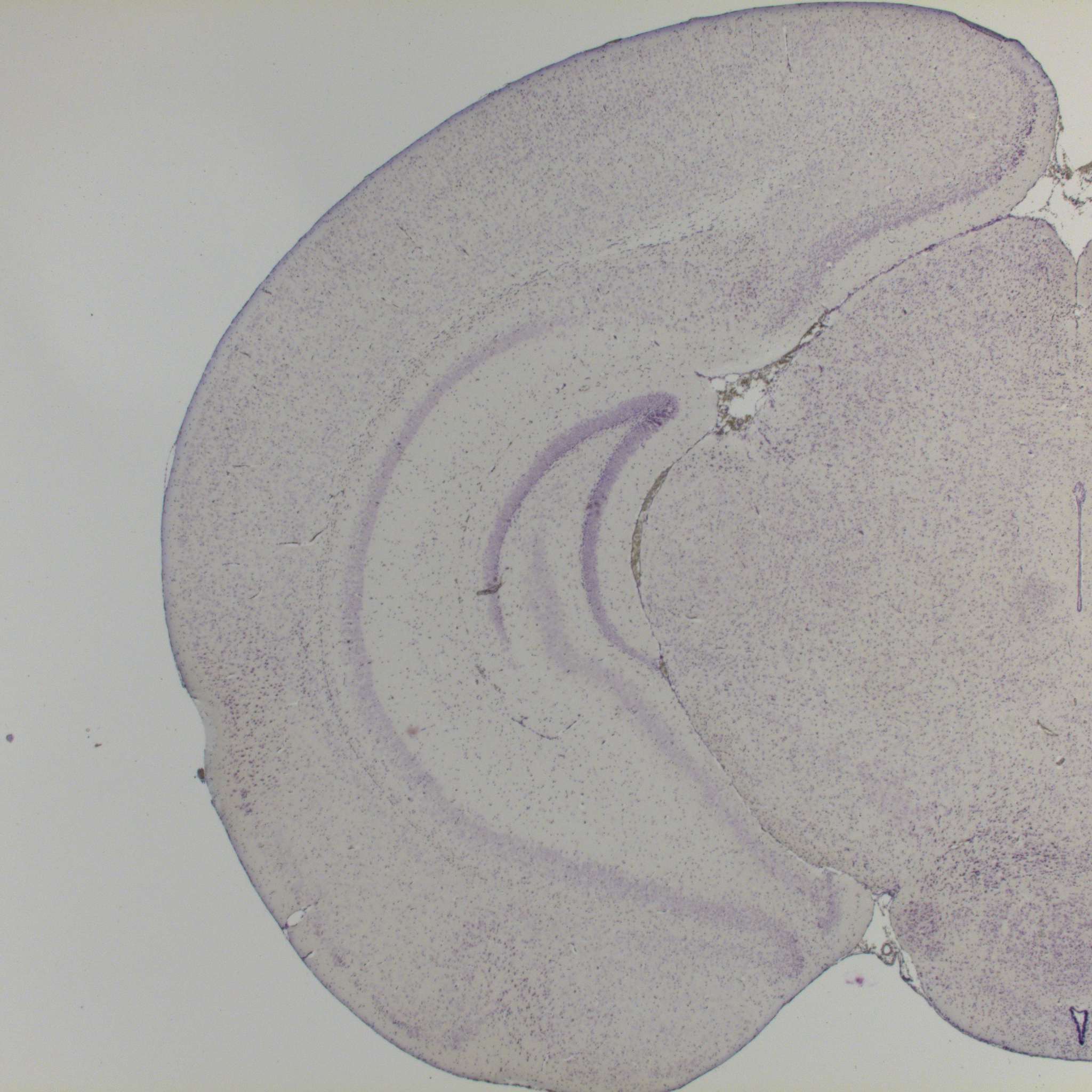

Supplement: Figure 2—source data 2. [file elife-86940-fig2-data2.zip › Figure 2-source data 2/F3094-5-CON-2.5X-CI f+-1M-#171-1-Image Export-19.tif]

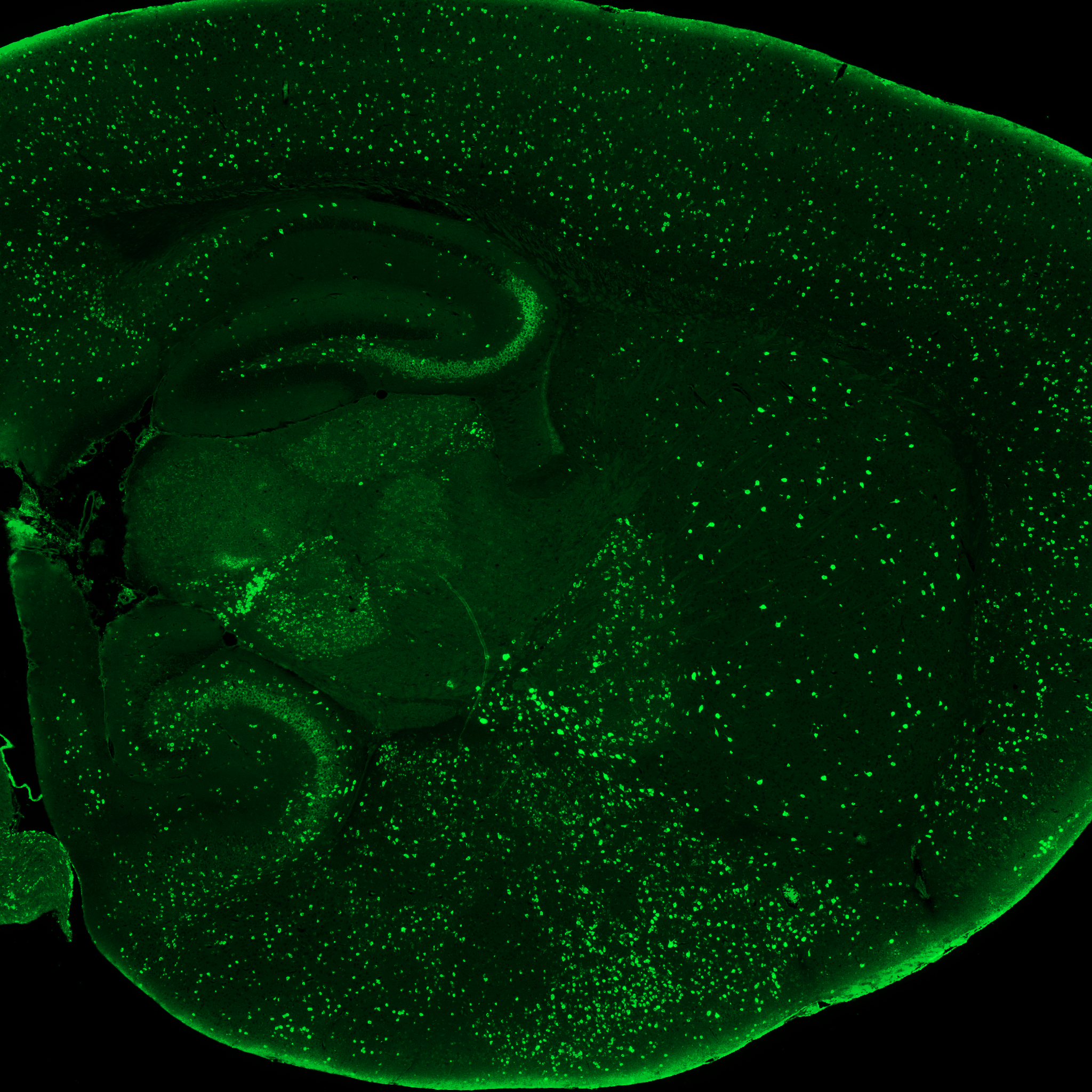

Supplement: Figure 2—source data 2. [file elife-86940-fig2-data2.zip › Figure 2-source data 2/F3094-2-CON-2.5X-RX CI F+-1M-SAGITAL-HUB-WFS1-33#-2-Image Export-05_AF488.tif]

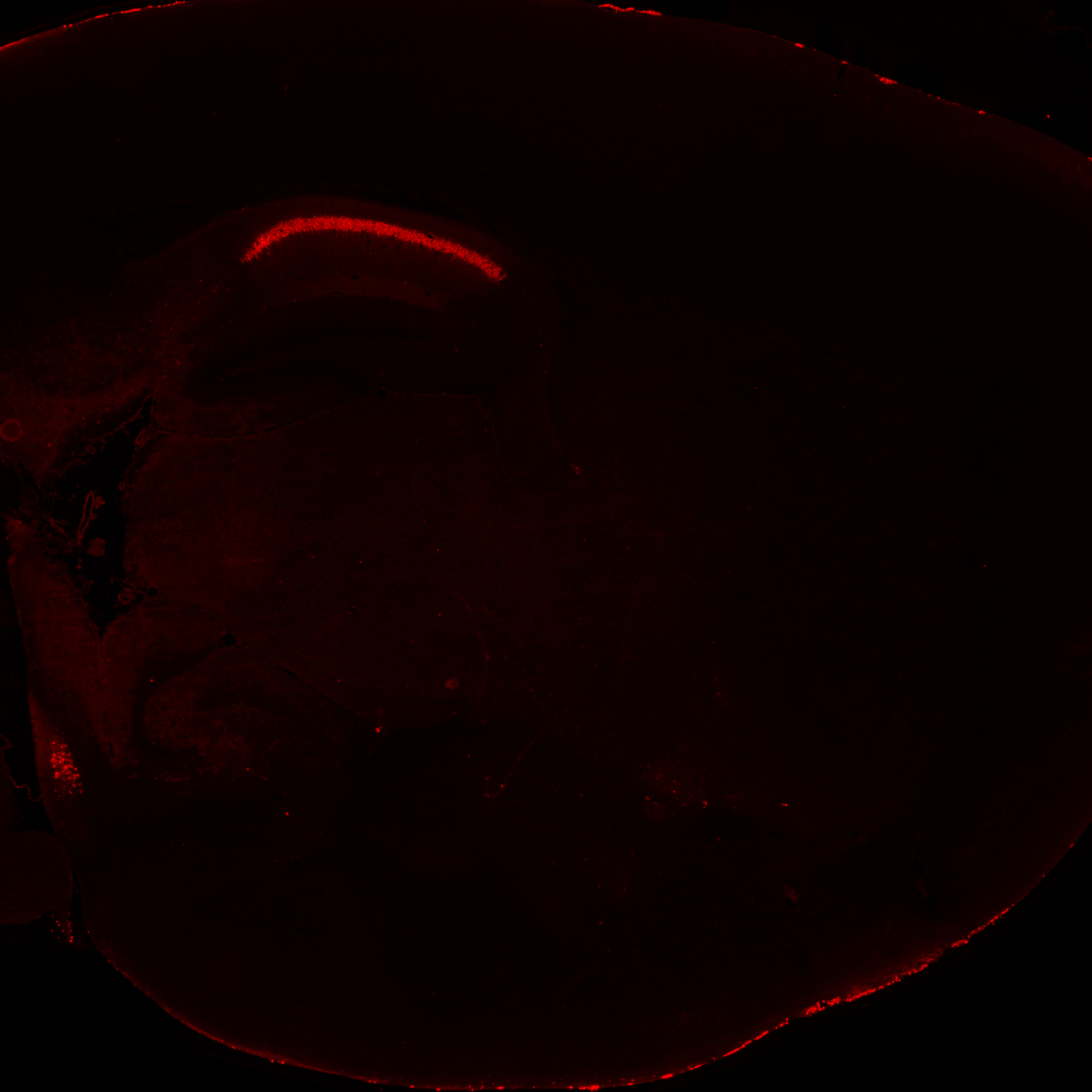

Supplement: Figure 2—source data 2. [file elife-86940-fig2-data2.zip › Figure 2-source data 2/F3094-2-CON-2.5X-RX CI F+-1M-SAGITAL-HUB-WFS1-33#-2-Image Export-05_AF594.tif]

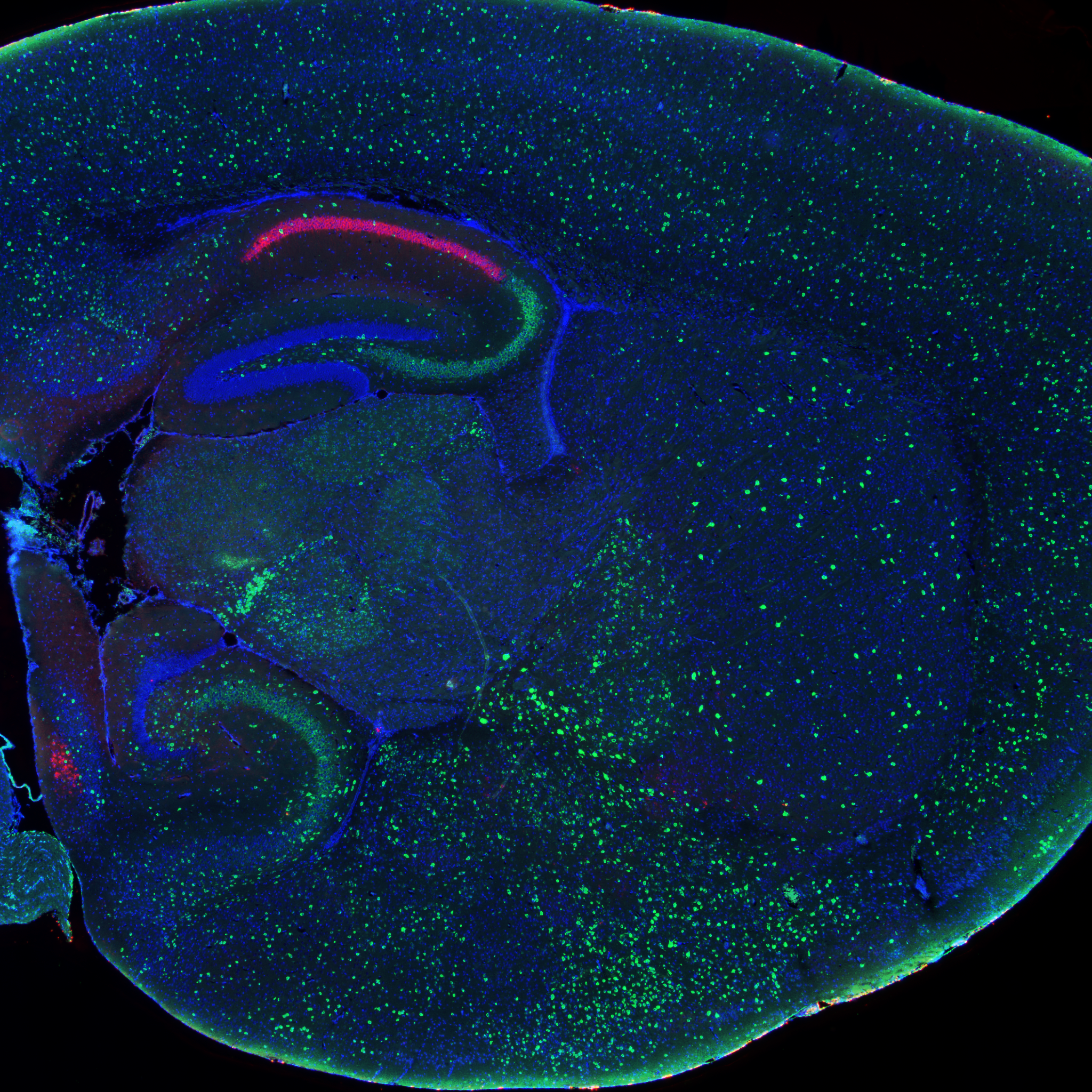

Supplement: Figure 2—source data 2. [file elife-86940-fig2-data2.zip › Figure 2-source data 2/F3094-2-CON-2.5X-RX CI F+-1M-SAGITAL-HUB-WFS1-33#-2-Image Export-05.tif]

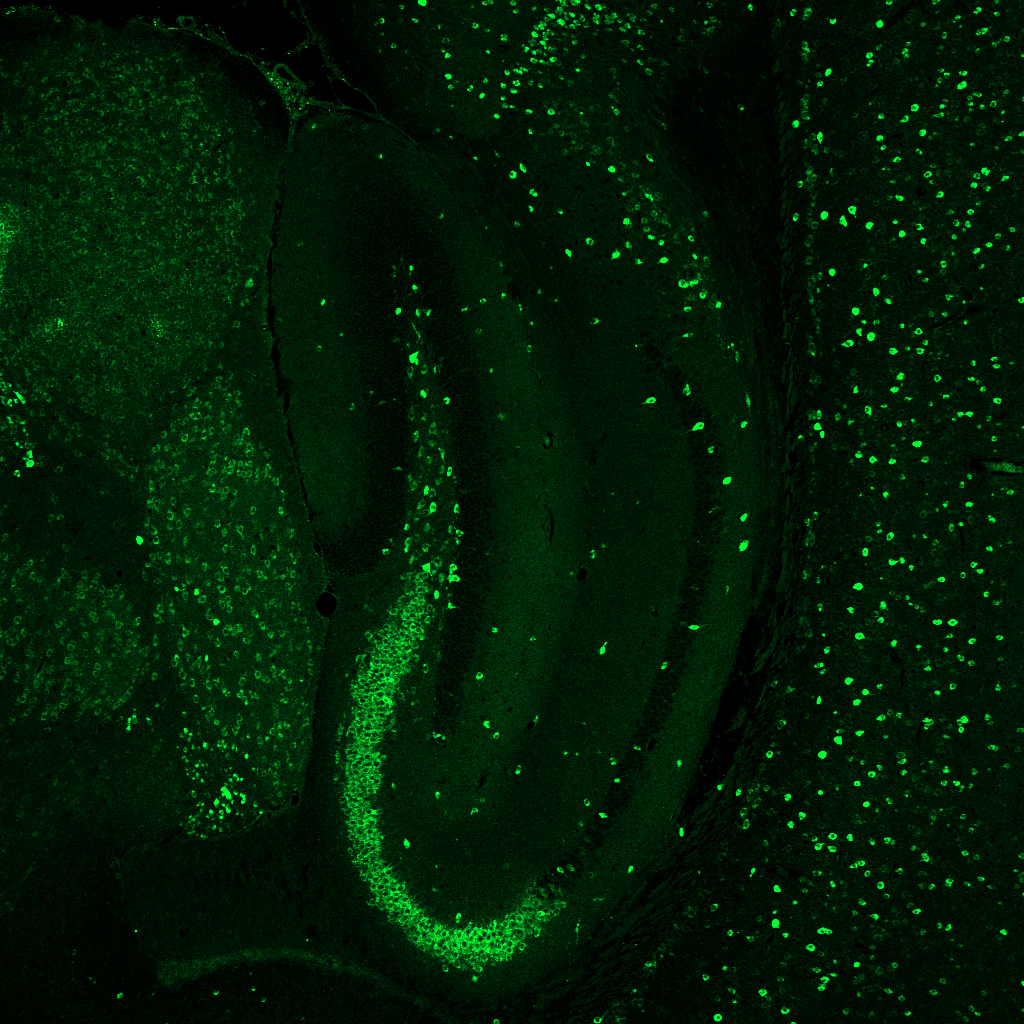

Supplement: Figure 2—source data 2. [file elife-86940-fig2-data2.zip › Figure 2-source data 2/F3094-2-CON-RX CI F+-1M-SAG-HUB-WSF1-#33-2-5X-dHPC-Image Export-04_AF488-T2.tif]

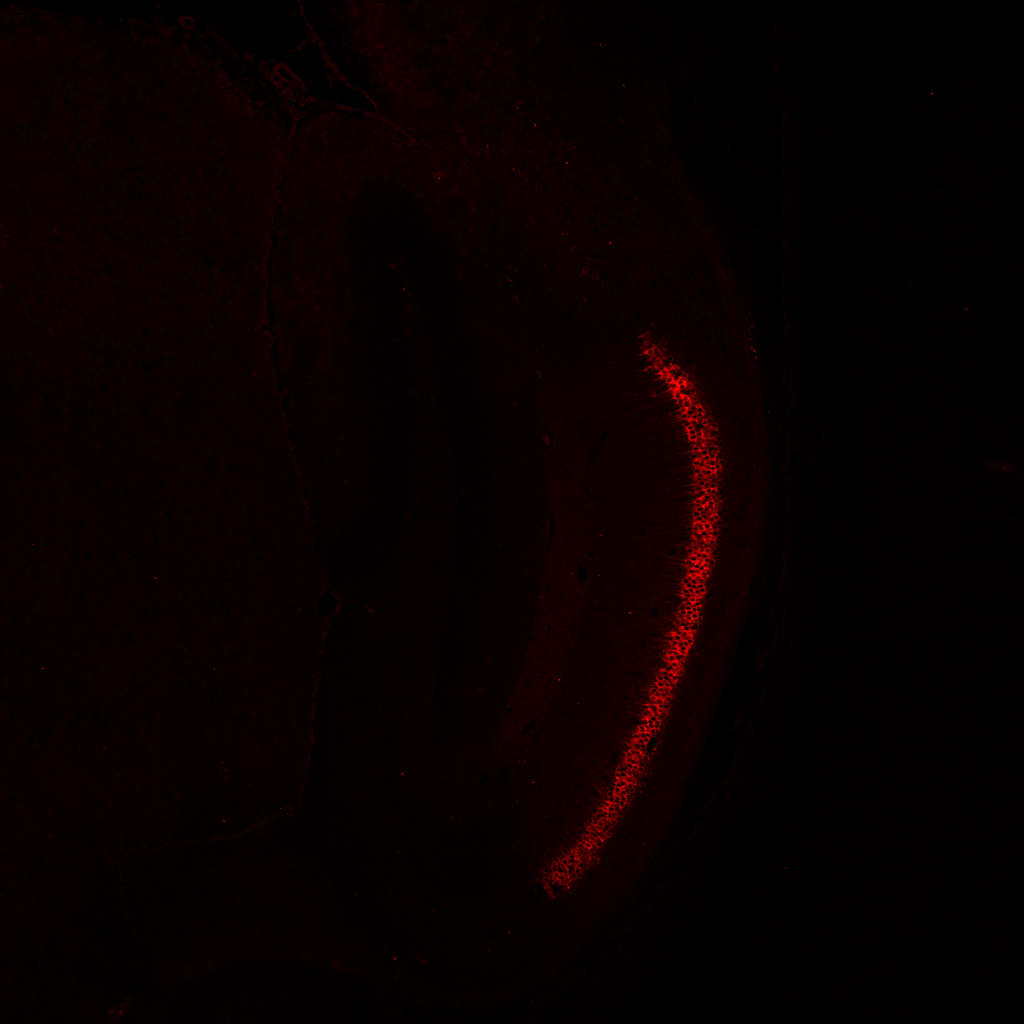

Supplement: Figure 2—source data 2. [file elife-86940-fig2-data2.zip › Figure 2-source data 2/F3094-2-CON-RX CI F+-1M-SAG-HUB-WSF1-#33-2-5X-dHPC-Image Export-04_AF594-T1.tif]

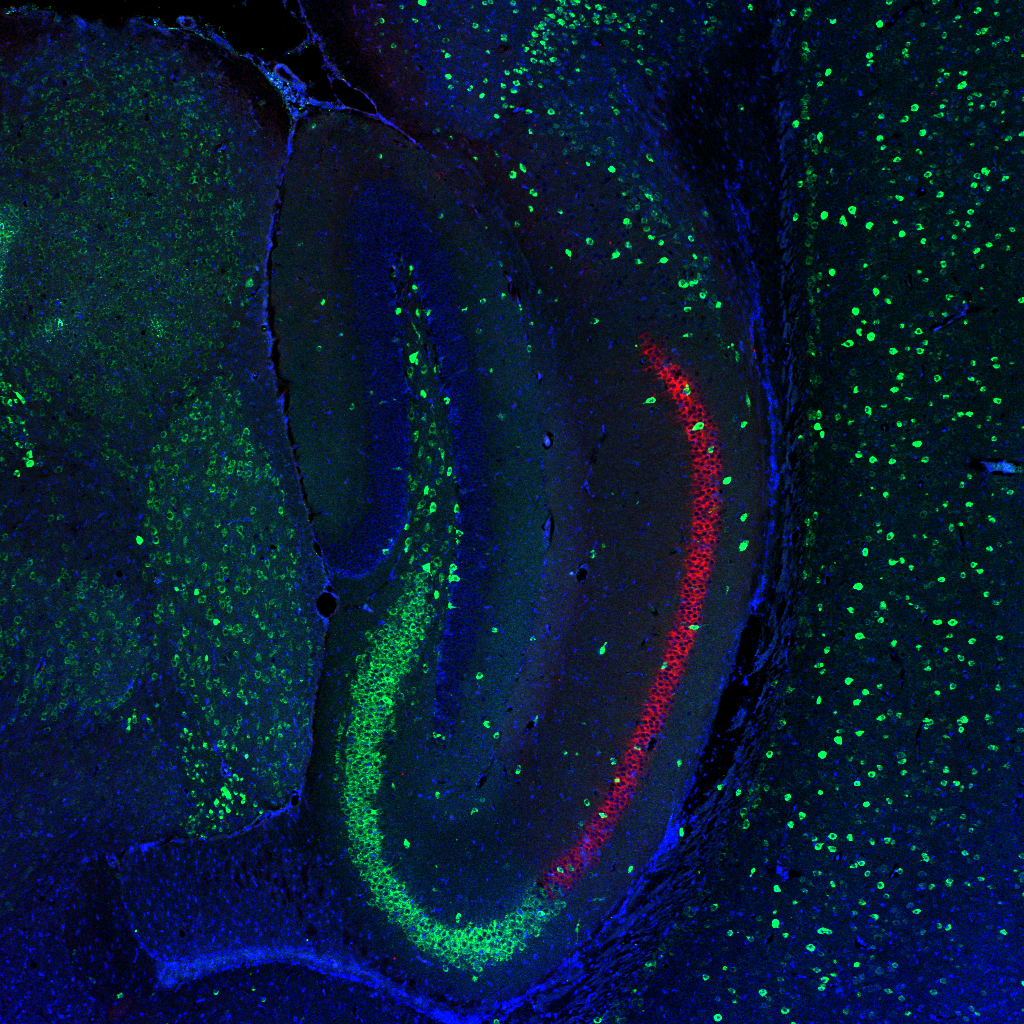

Supplement: Figure 2—source data 2. [file elife-86940-fig2-data2.zip › Figure 2-source data 2/F3094-2-CON-RX CI F+-1M-SAG-HUB-WSF1-#33-2-5X-dHPC-Image Export-04.tif]

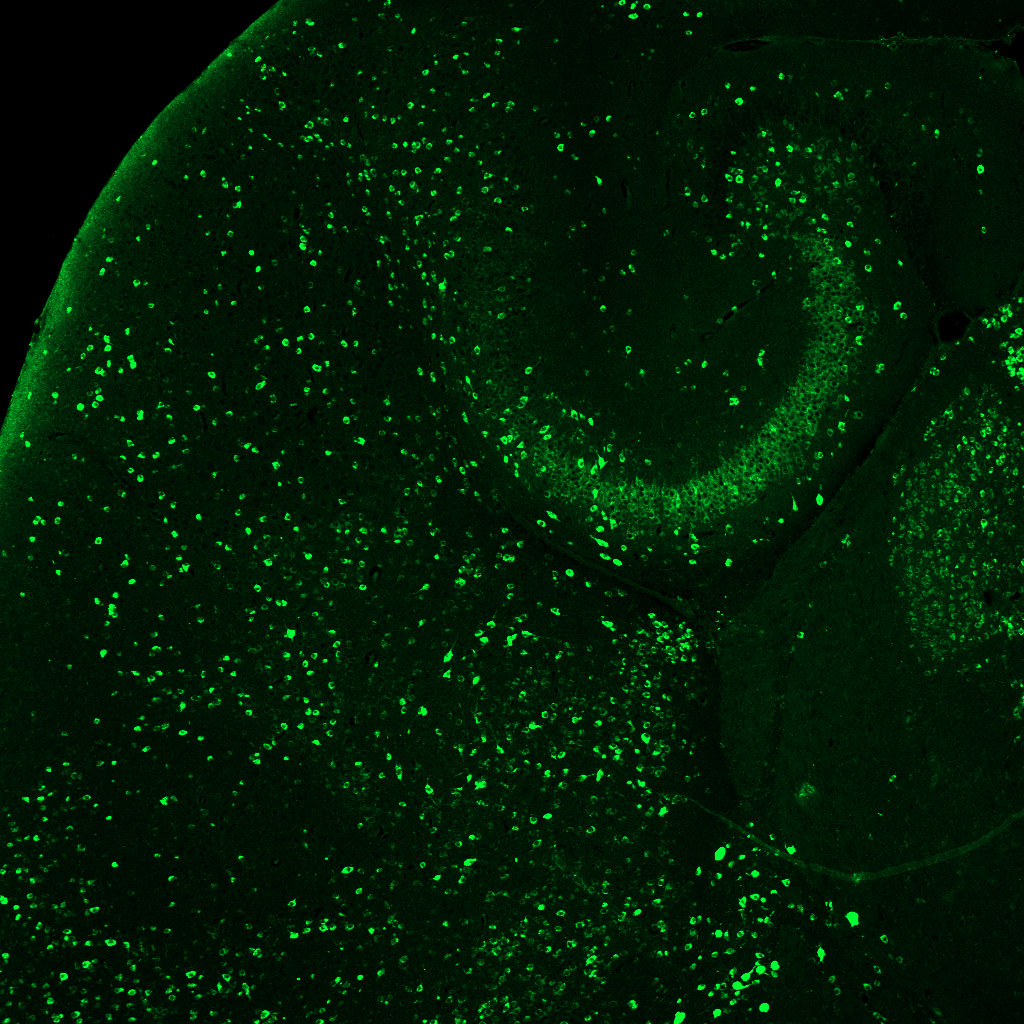

Supplement: Figure 2—source data 2. [file elife-86940-fig2-data2.zip › Figure 2-source data 2/F3094-2-CON-RX CI F+-1M-SAG-HUB-WSF1-#33-2-5X-vHPC-Image Export-05_AF488-T2.tif]

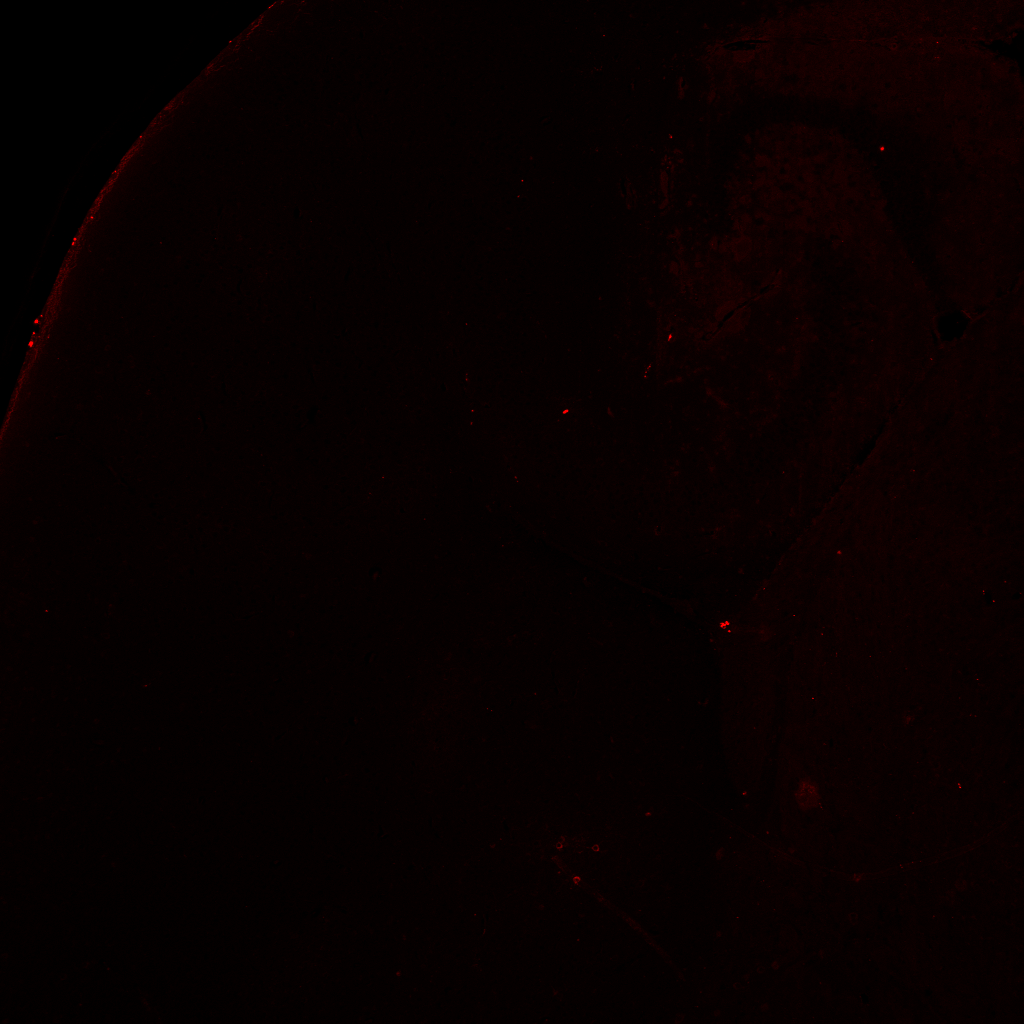

Supplement: Figure 2—source data 2. [file elife-86940-fig2-data2.zip › Figure 2-source data 2/F3094-2-CON-RX CI F+-1M-SAG-HUB-WSF1-#33-2-5X-vHPC-Image Export-05_AF594-T1.tif]

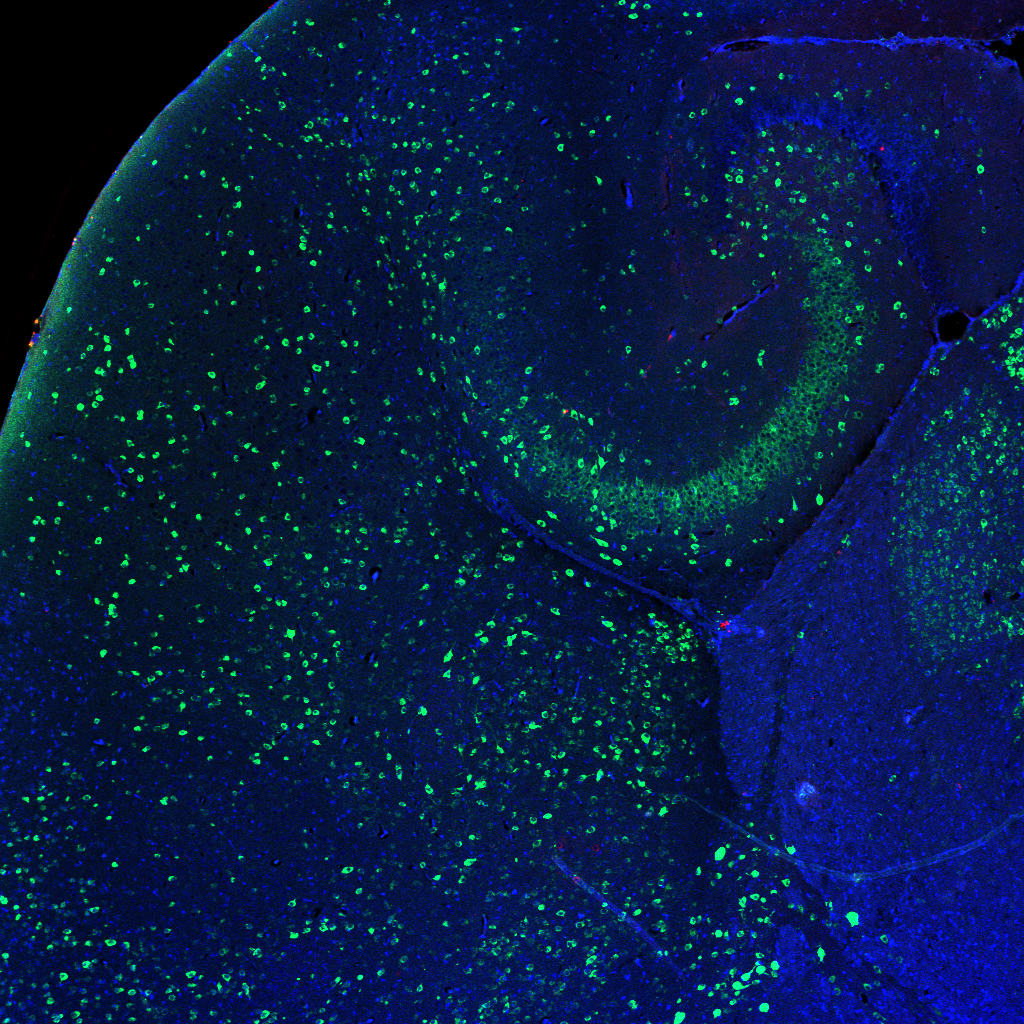

Supplement: Figure 2—source data 2. [file elife-86940-fig2-data2.zip › Figure 2-source data 2/F3094-2-CON-RX CI F+-1M-SAG-HUB-WSF1-#33-2-5X-vHPC-Image Export-05.tif]

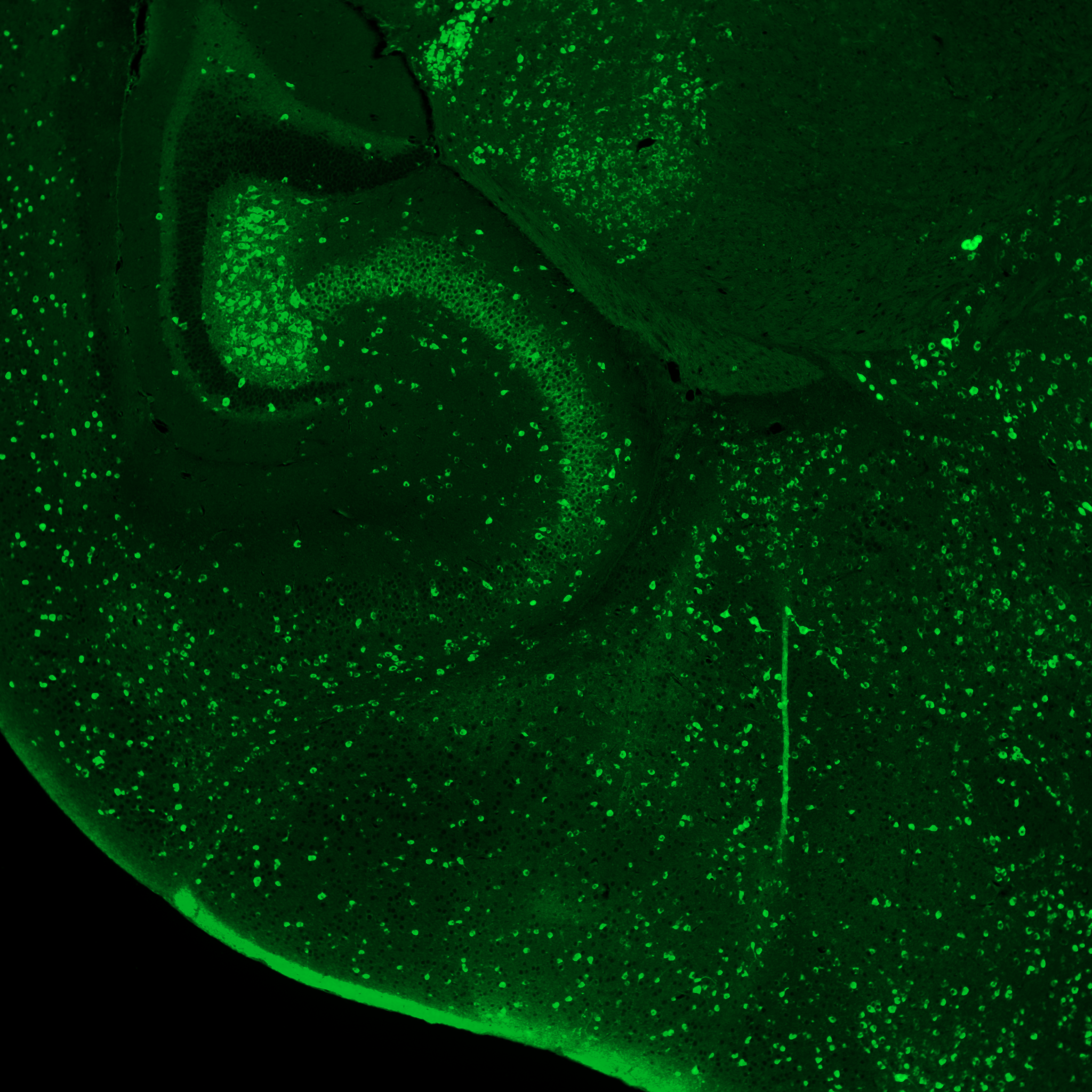

Supplement: Figure 2—source data 3. [file elife-86940-fig2-data3.zip › Figure 2-source data 3/F3094-2-CON-RX CI F+-1M-SAGITAL-HUB-CTIP2-152#-2-5X-vHPC-Image Export-10_AF488.tif]

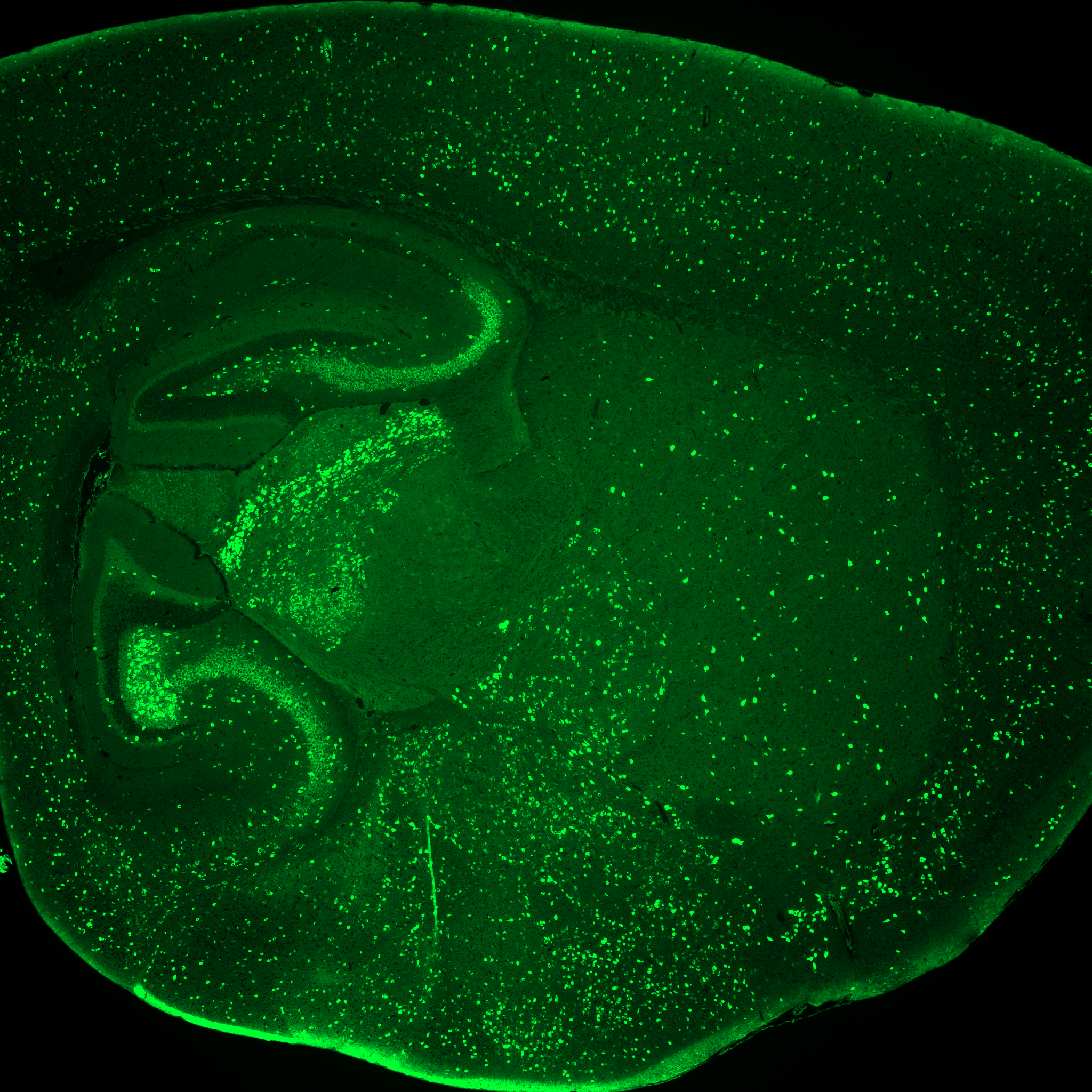

Supplement: Figure 2—source data 3. [file elife-86940-fig2-data3.zip › Figure 2-source data 3/F3094-2-CON-RX CI F+-1M-SAGITAL-HUB-CTIP2-152#-2-2.5X-HPC-Image Export-07_AF488.tif]

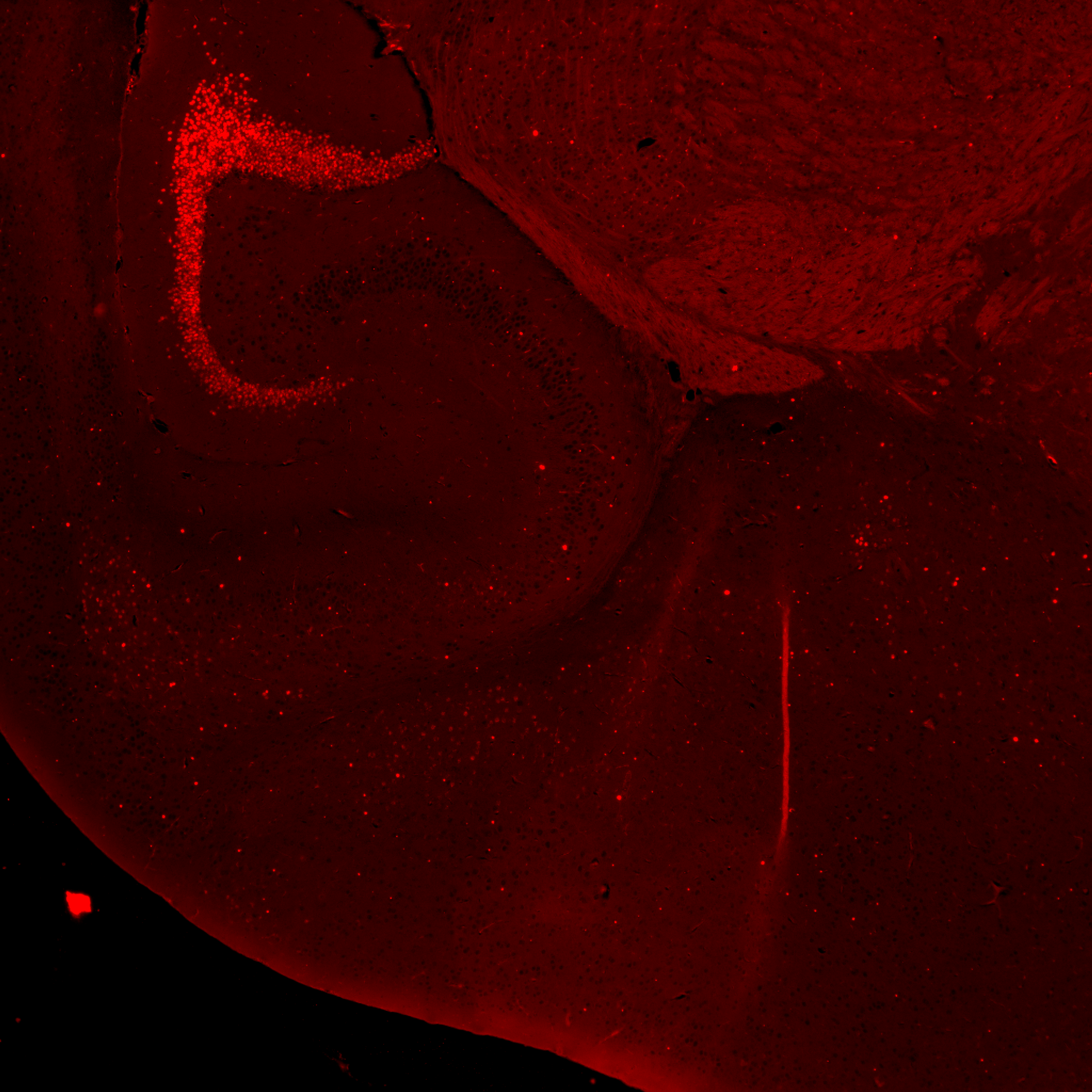

Supplement: Figure 2—source data 3. [file elife-86940-fig2-data3.zip › Figure 2-source data 3/F3094-2-CON-RX CI F+-1M-SAGITAL-HUB-CTIP2-152#-2-5X-vHPC-Image Export-10_AF594.tif]

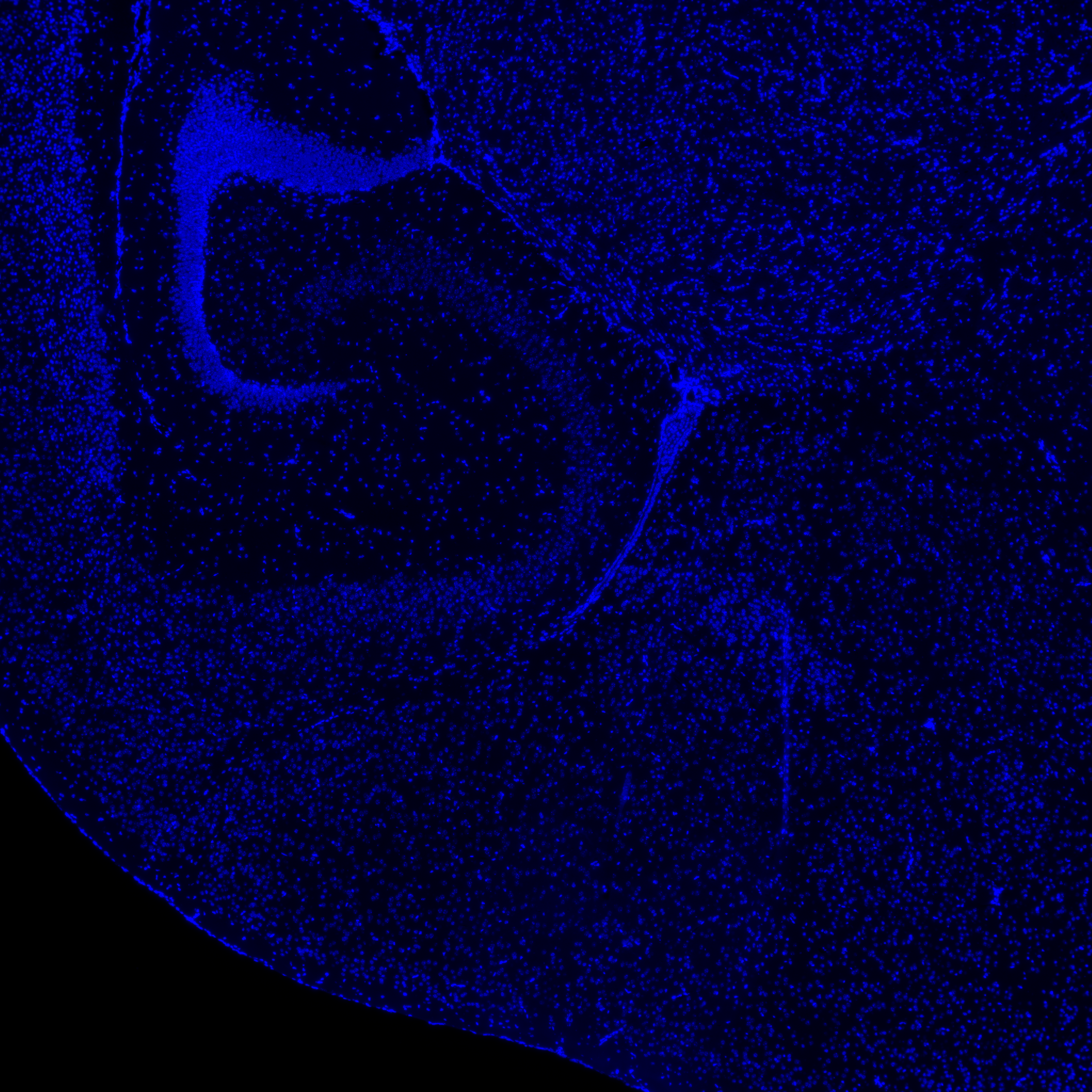

Supplement: Figure 2—source data 3. [file elife-86940-fig2-data3.zip › Figure 2-source data 3/F3094-2-CON-RX CI F+-1M-SAGITAL-HUB-CTIP2-152#-2-5X-vHPC-Image Export-10_DAPI.tif]

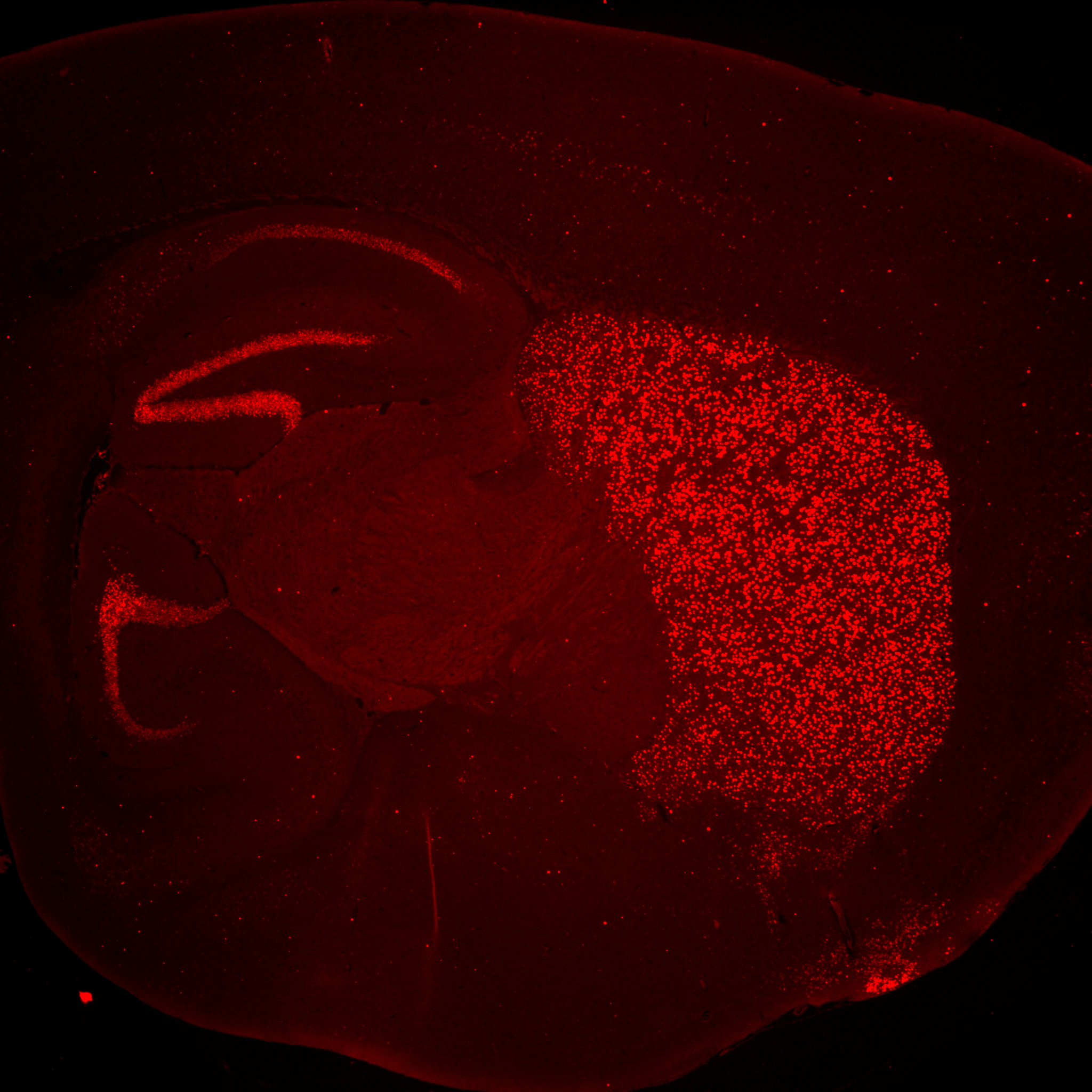

Supplement: Figure 2—source data 3. [file elife-86940-fig2-data3.zip › Figure 2-source data 3/F3094-2-CON-RX CI F+-1M-SAGITAL-HUB-CTIP2-152#-2-2.5X-HPC-Image Export-07_AF594.tif]

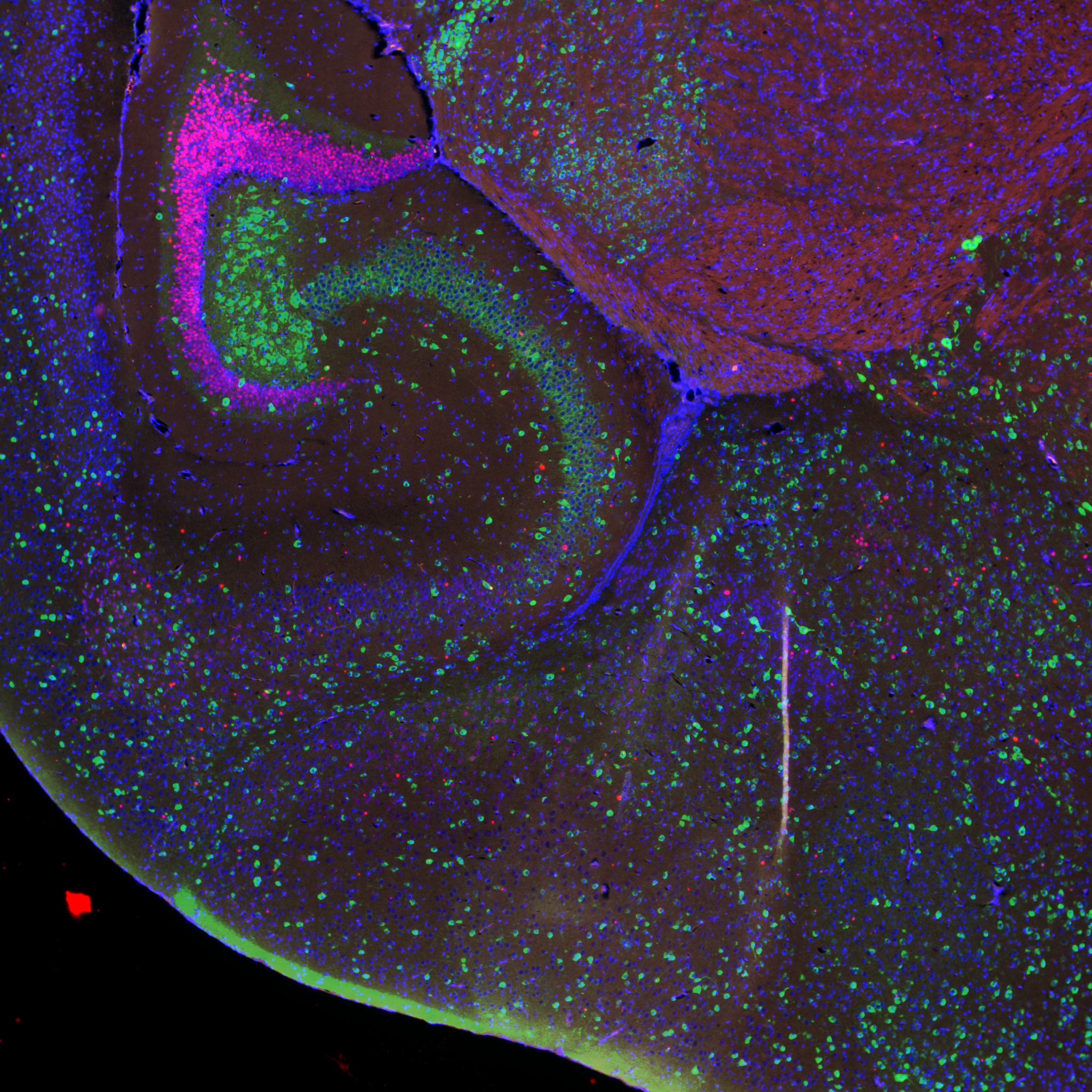

Supplement: Figure 2—source data 3. [file elife-86940-fig2-data3.zip › Figure 2-source data 3/F3094-2-CON-RX CI F+-1M-SAGITAL-HUB-CTIP2-152#-2-5X-vHPC-Image Export-10.tif]

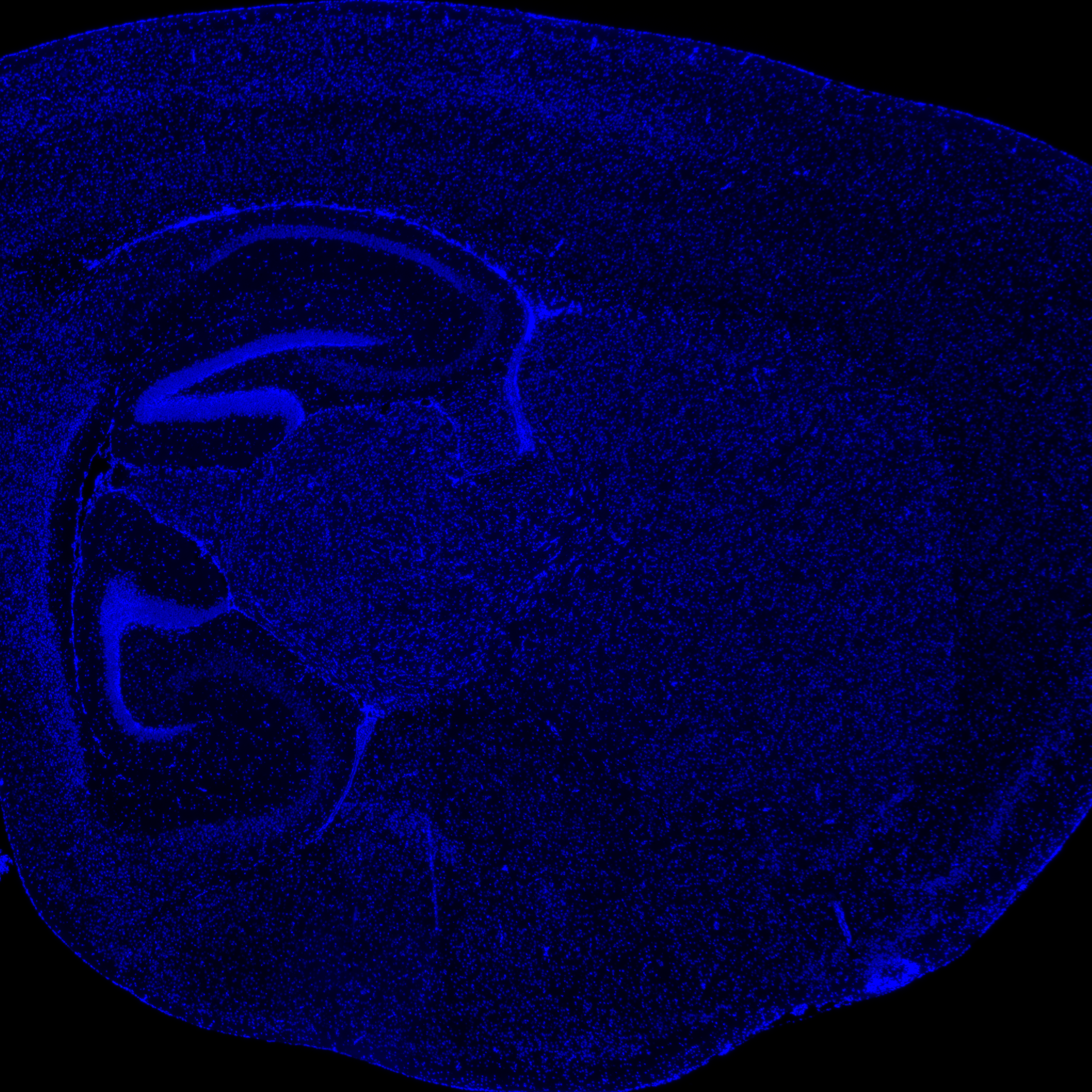

Supplement: Figure 2—source data 3. [file elife-86940-fig2-data3.zip › Figure 2-source data 3/F3094-2-CON-RX CI F+-1M-SAGITAL-HUB-CTIP2-152#-2-2.5X-HPC-Image Export-07_DAPI.tif]

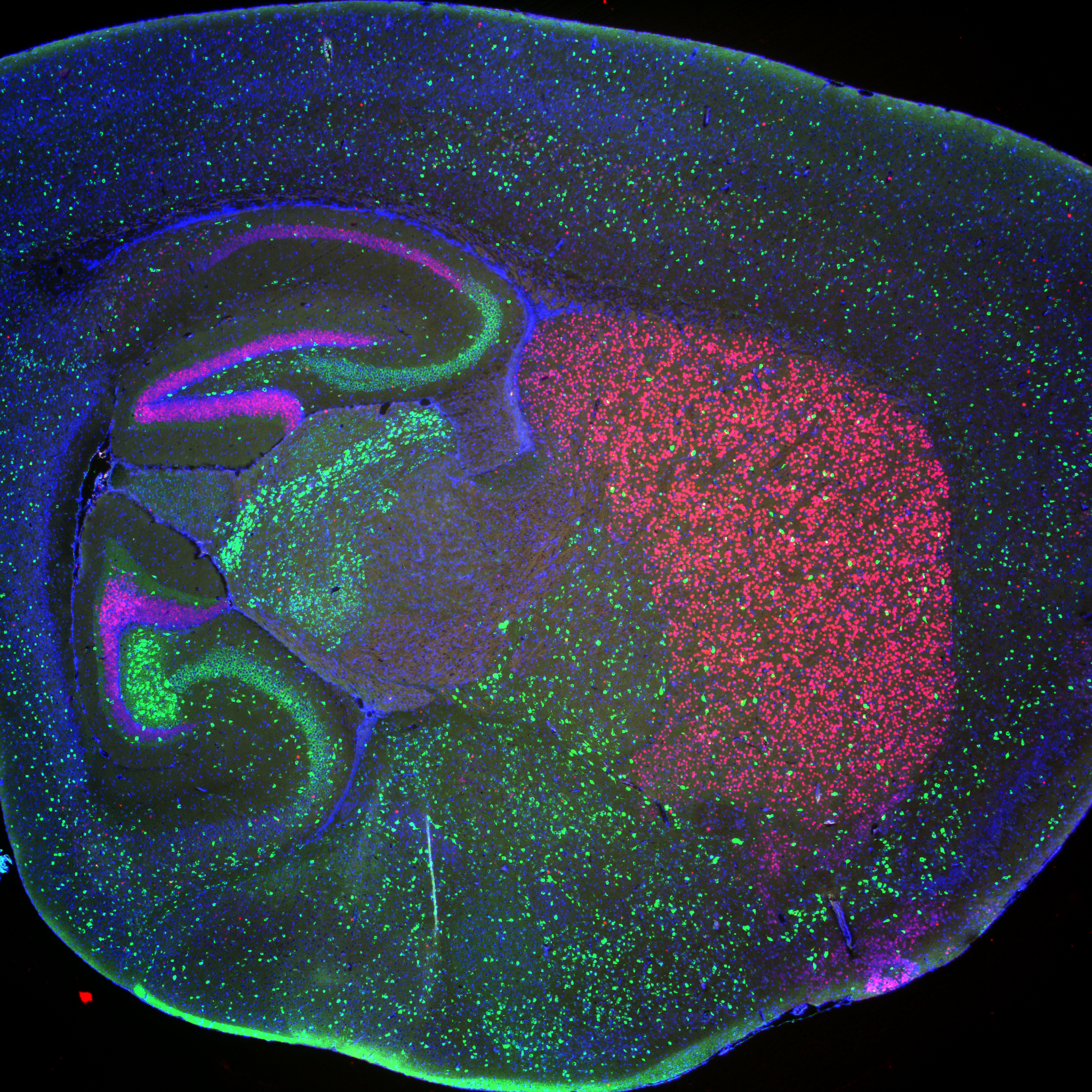

Supplement: Figure 2—source data 3. [file elife-86940-fig2-data3.zip › Figure 2-source data 3/F3094-2-CON-RX CI F+-1M-SAGITAL-HUB-CTIP2-152#-2-2.5X-HPC-Image Export-07.tif]

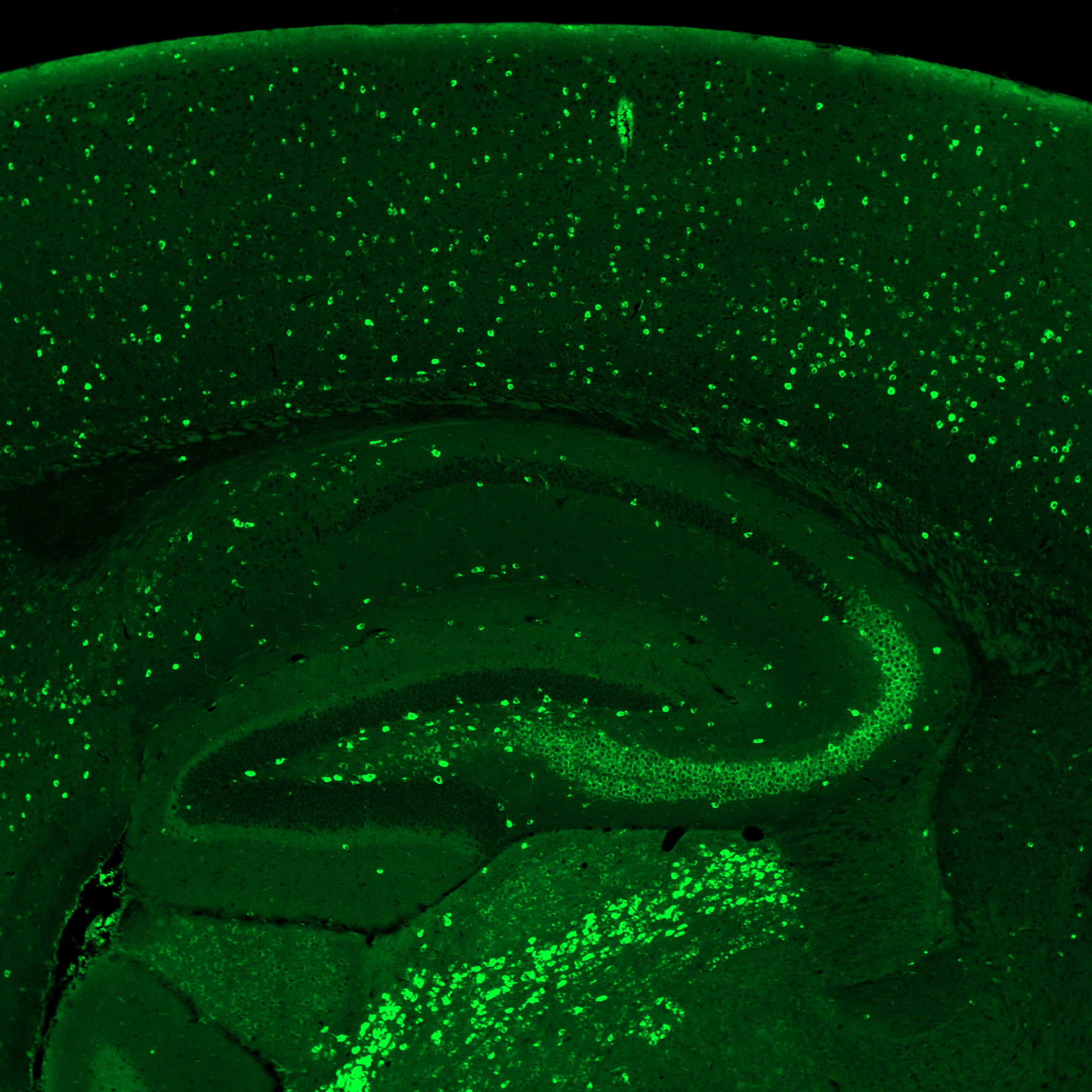

Supplement: Figure 2—source data 3. [file elife-86940-fig2-data3.zip › Figure 2-source data 3/F3094-2-CON-RX CI F+-1M-SAGITAL-HUB-CTIP2-152#-2-5X-dHPC-Image Export-08_AF488.tif]

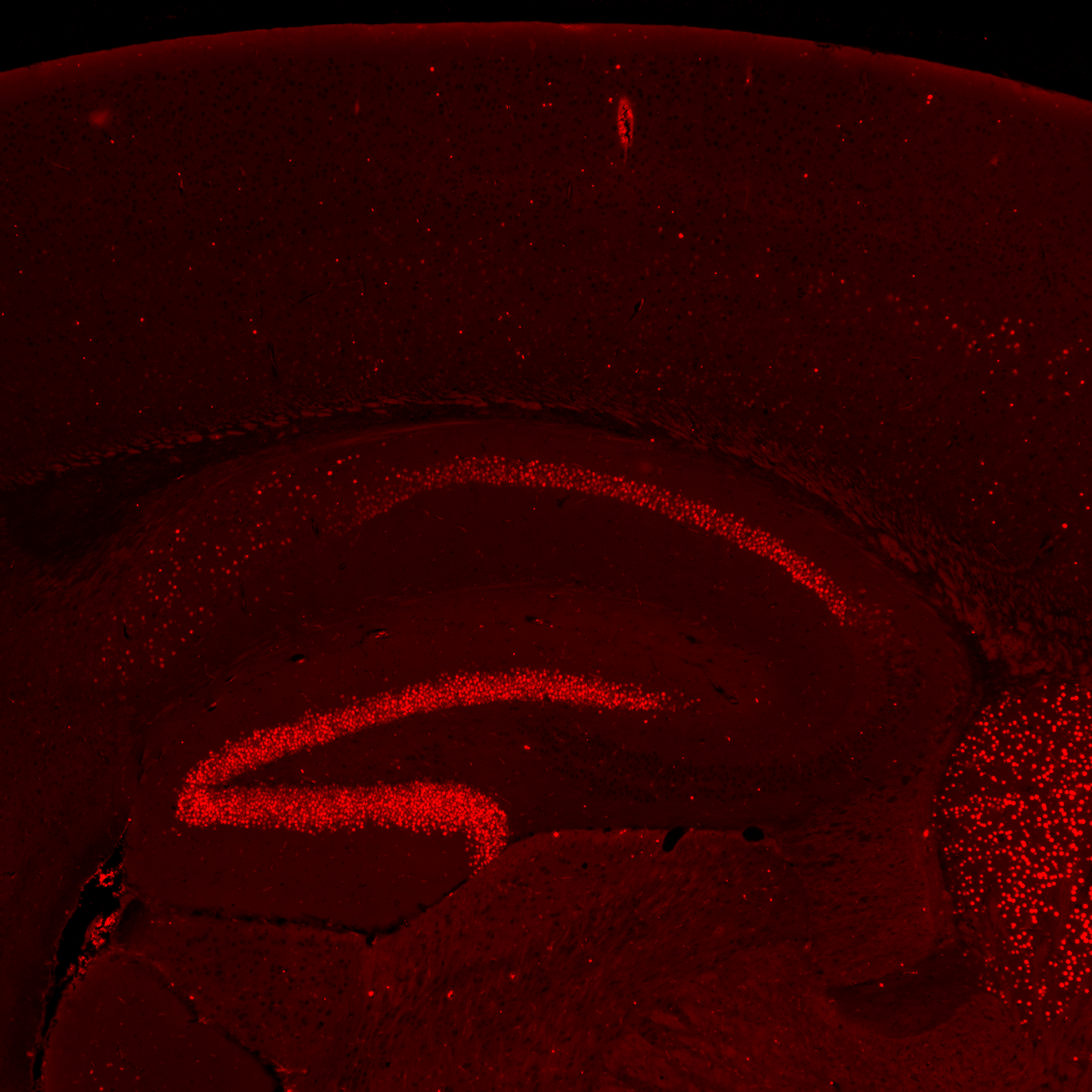

Supplement: Figure 2—source data 3. [file elife-86940-fig2-data3.zip › Figure 2-source data 3/F3094-2-CON-RX CI F+-1M-SAGITAL-HUB-CTIP2-152#-2-5X-dHPC-Image Export-08_AF594.tif]

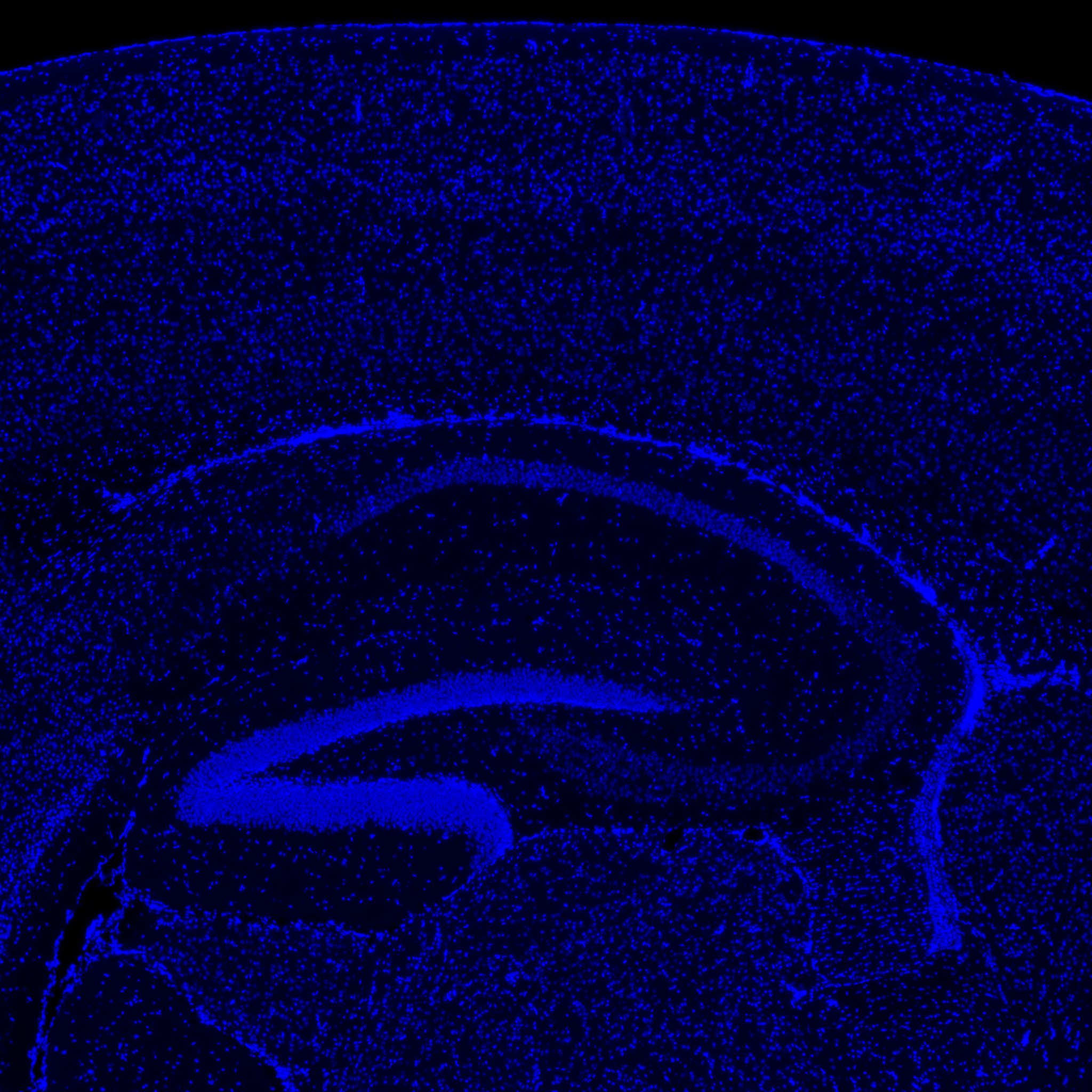

Supplement: Figure 2—source data 3. [file elife-86940-fig2-data3.zip › Figure 2-source data 3/F3094-2-CON-RX CI F+-1M-SAGITAL-HUB-CTIP2-152#-2-5X-dHPC-Image Export-08_DAPI.tif]

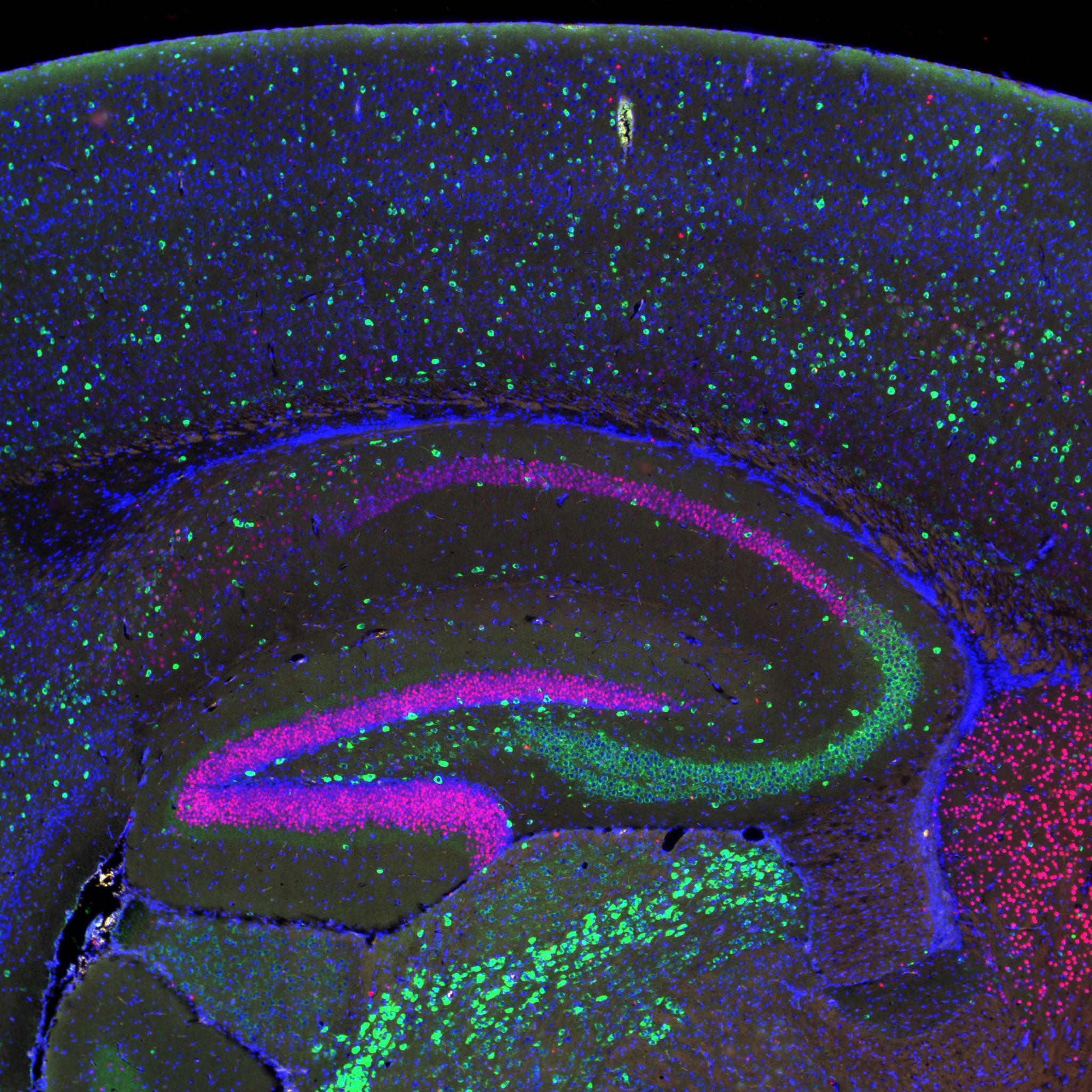

Supplement: Figure 2—source data 3. [file elife-86940-fig2-data3.zip › Figure 2-source data 3/F3094-2-CON-RX CI F+-1M-SAGITAL-HUB-CTIP2-152#-2-5X-dHPC-Image Export-08.tif]

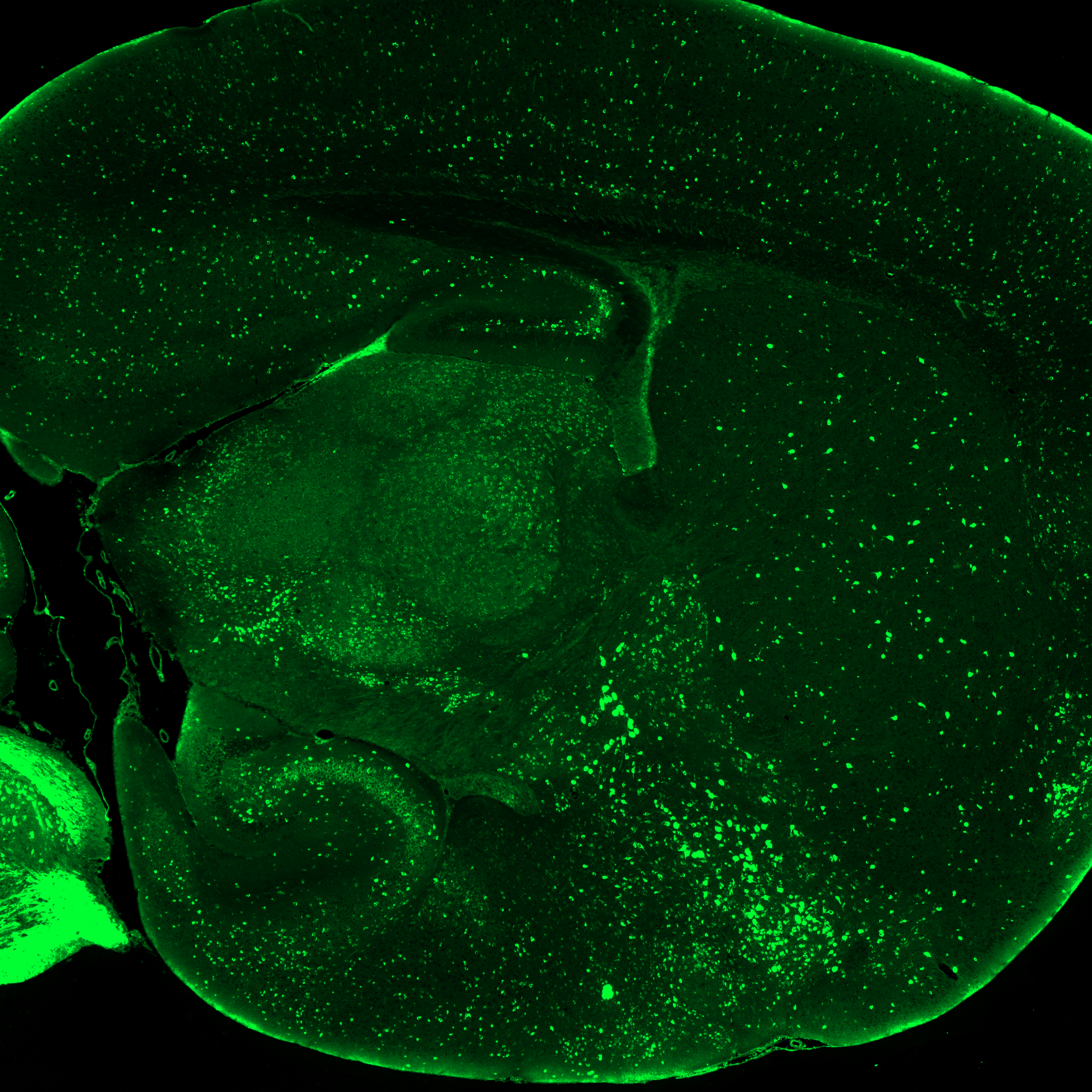

Supplement: Figure 2—source data 4. [file elife-86940-fig2-data4.zip › Figure 2-source data 4/F3094-3-CKO-2.5X-RX CI FF-1M-SAGITAL-HUB-WFS1-32#-2-Image Export-13_AF488.tif]

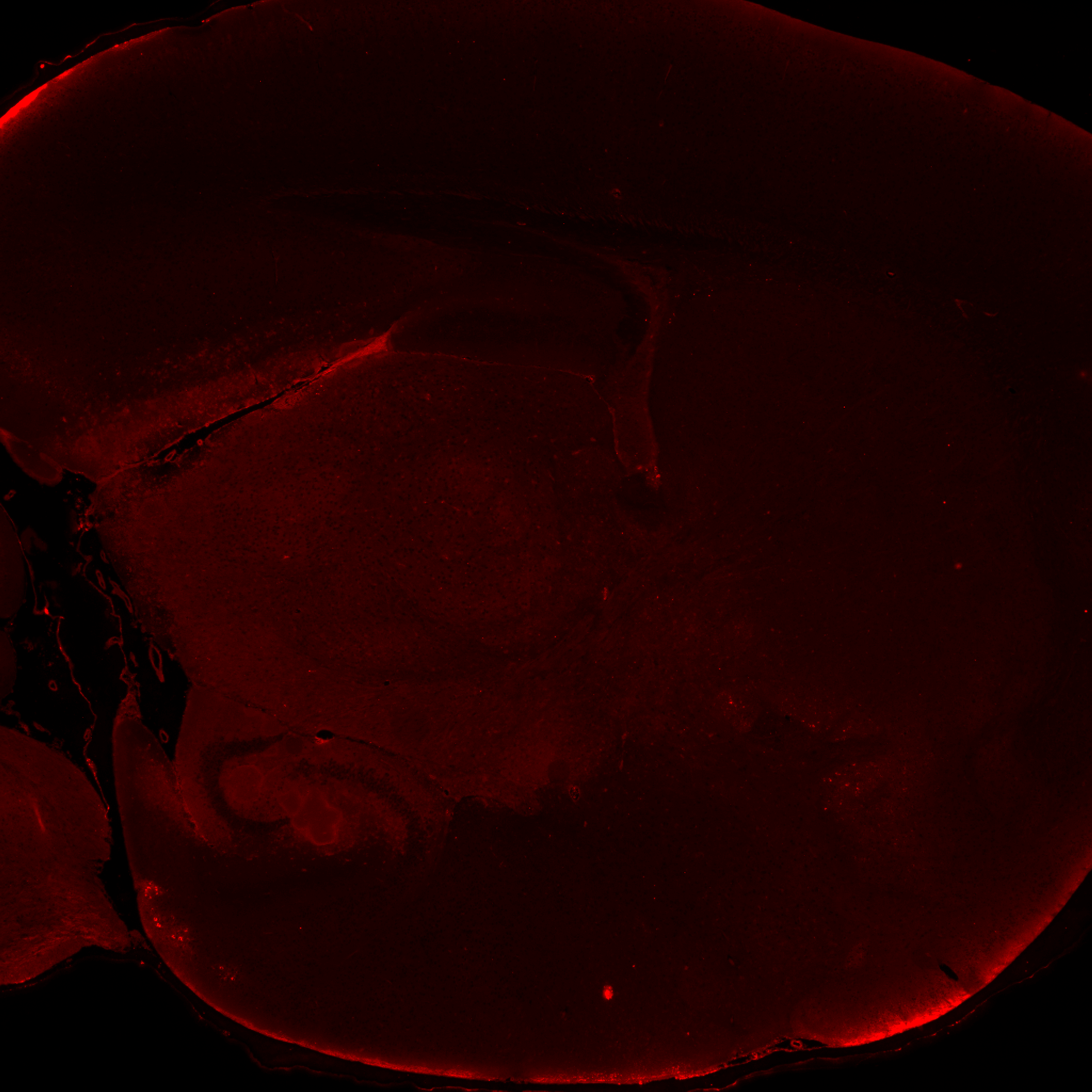

Supplement: Figure 2—source data 4. [file elife-86940-fig2-data4.zip › Figure 2-source data 4/F3094-3-CKO-2.5X-RX CI FF-1M-SAGITAL-HUB-WFS1-32#-2-Image Export-13_AF594.tif]

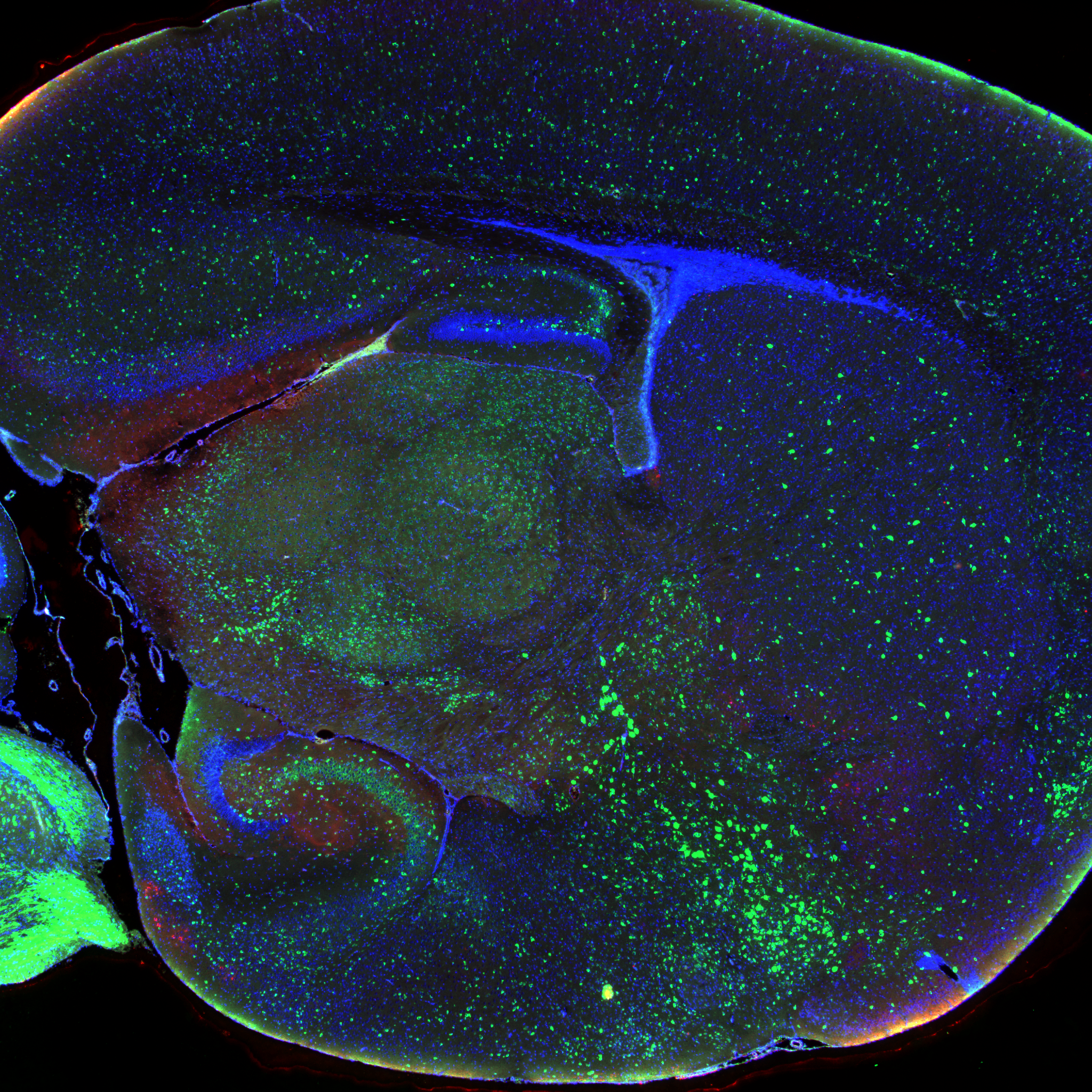

Supplement: Figure 2—source data 4. [file elife-86940-fig2-data4.zip › Figure 2-source data 4/F3094-3-CKO-2.5X-RX CI FF-1M-SAGITAL-HUB-WFS1-32#-2-Image Export-13.tif]

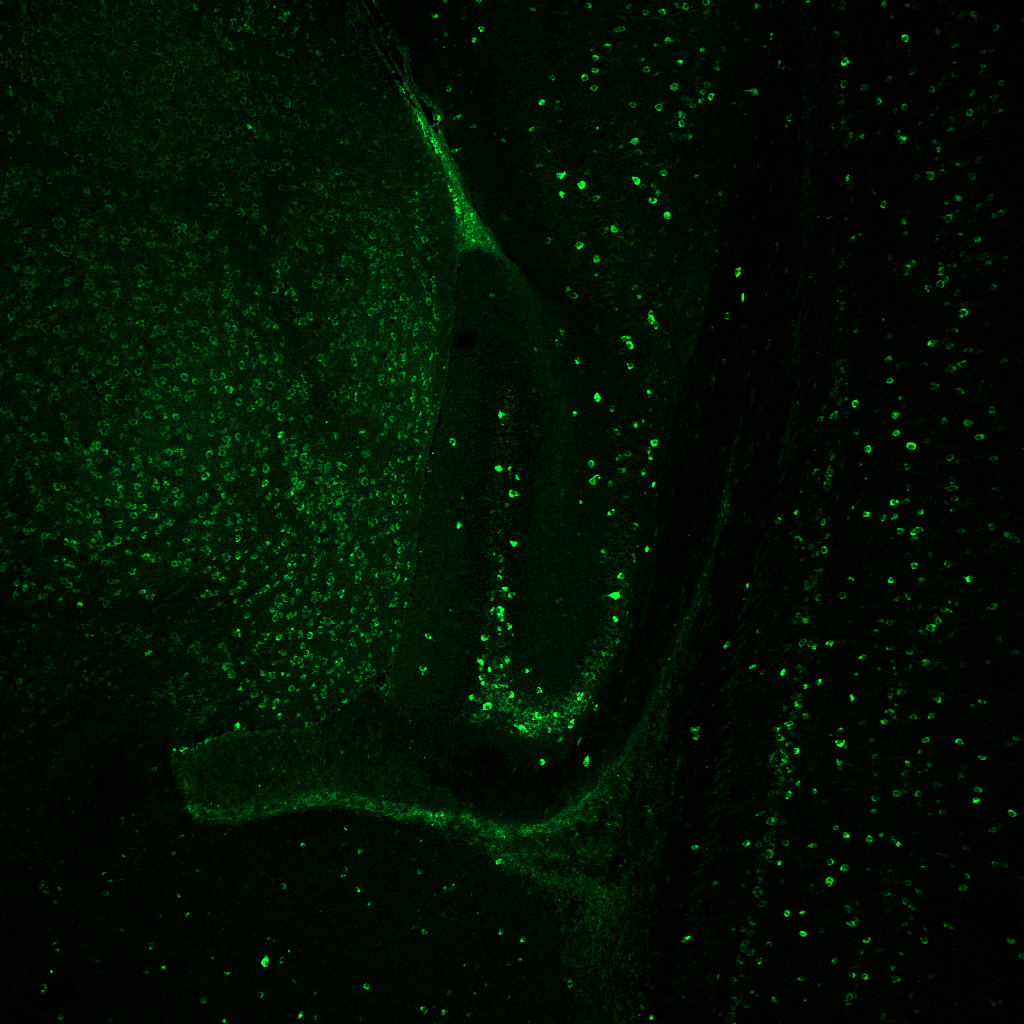

Supplement: Figure 2—source data 4. [file elife-86940-fig2-data4.zip › Figure 2-source data 4/F3094-3-CKO-RX CI FF-1M-SAG-HUB-WFS1-#33-2-5X-dHPC-Image Export-13_AF488-T2.tif]

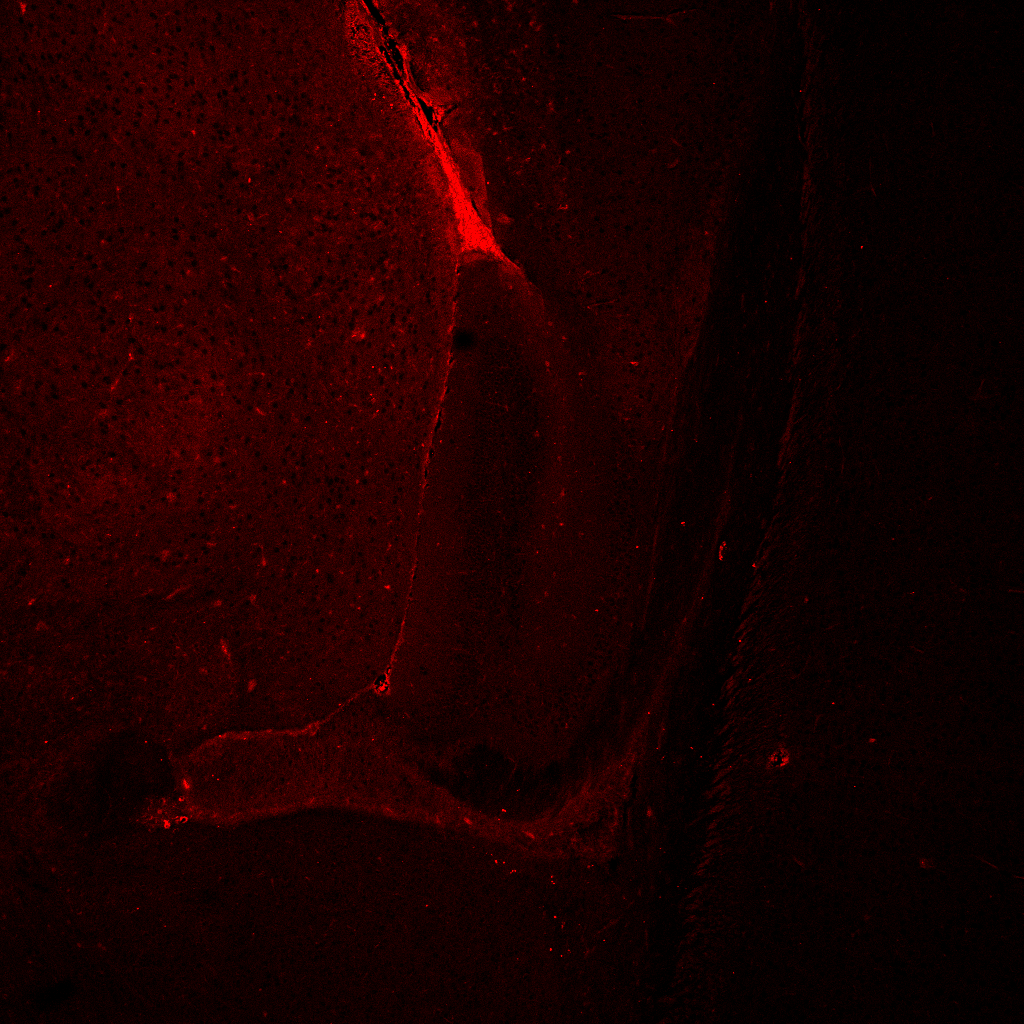

Supplement: Figure 2—source data 4. [file elife-86940-fig2-data4.zip › Figure 2-source data 4/F3094-3-CKO-RX CI FF-1M-SAG-HUB-WFS1-#33-2-5X-dHPC-Image Export-13_AF594-T1.tif]

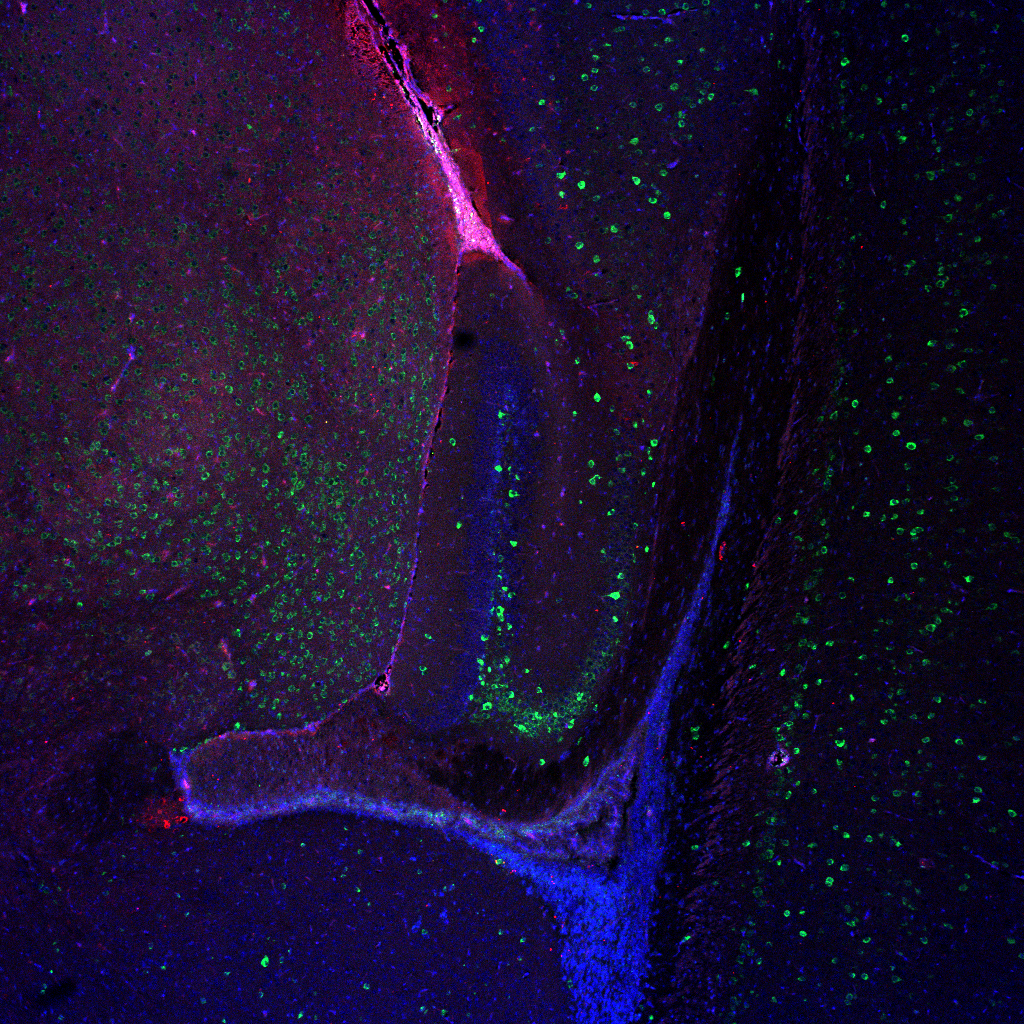

Supplement: Figure 2—source data 4. [file elife-86940-fig2-data4.zip › Figure 2-source data 4/F3094-3-CKO-RX CI FF-1M-SAG-HUB-WFS1-#33-2-5X-dHPC-Image Export-13.tif]

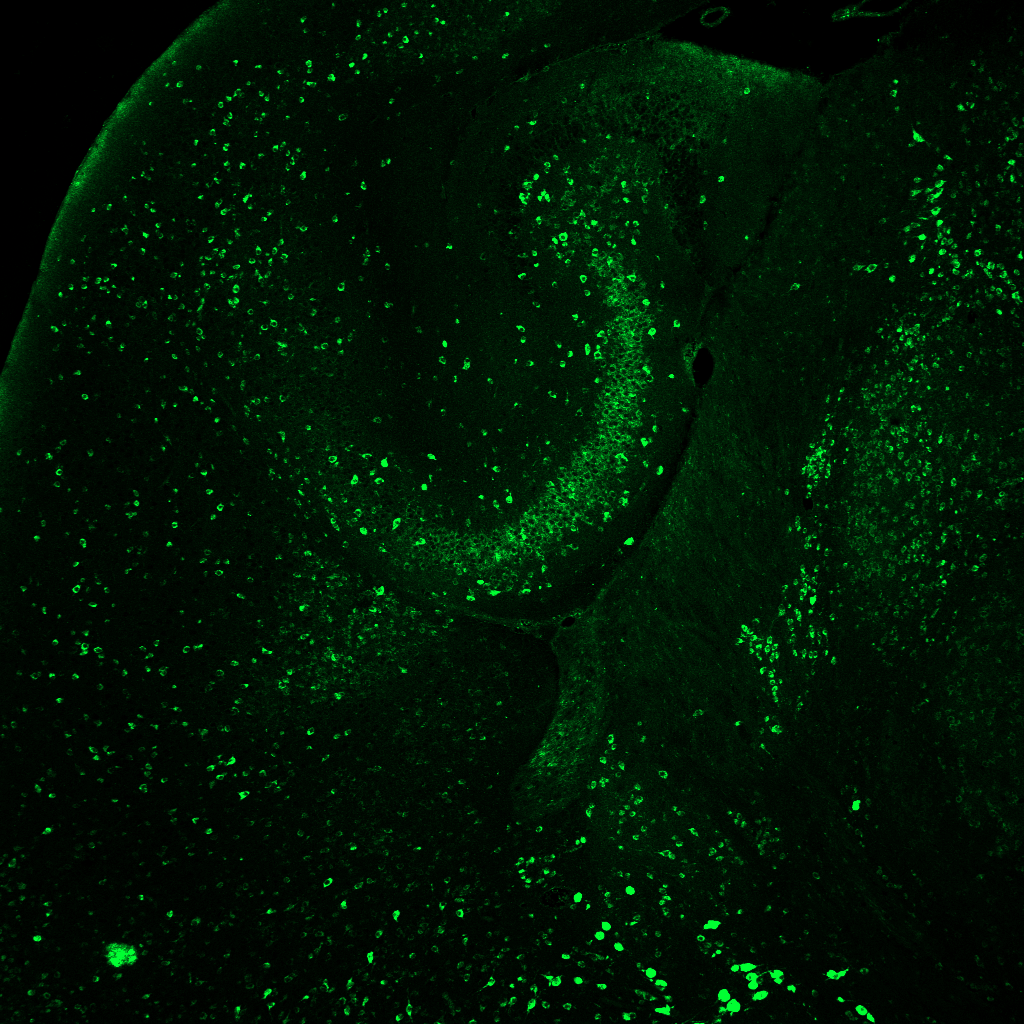

Supplement: Figure 2—source data 4. [file elife-86940-fig2-data4.zip › Figure 2-source data 4/F3094-3-CKO-RX CI FF-1M-SAG-HUB-WFS1-#33-2-5X-vHPC-Image Export-14_AF488-T2.tif]

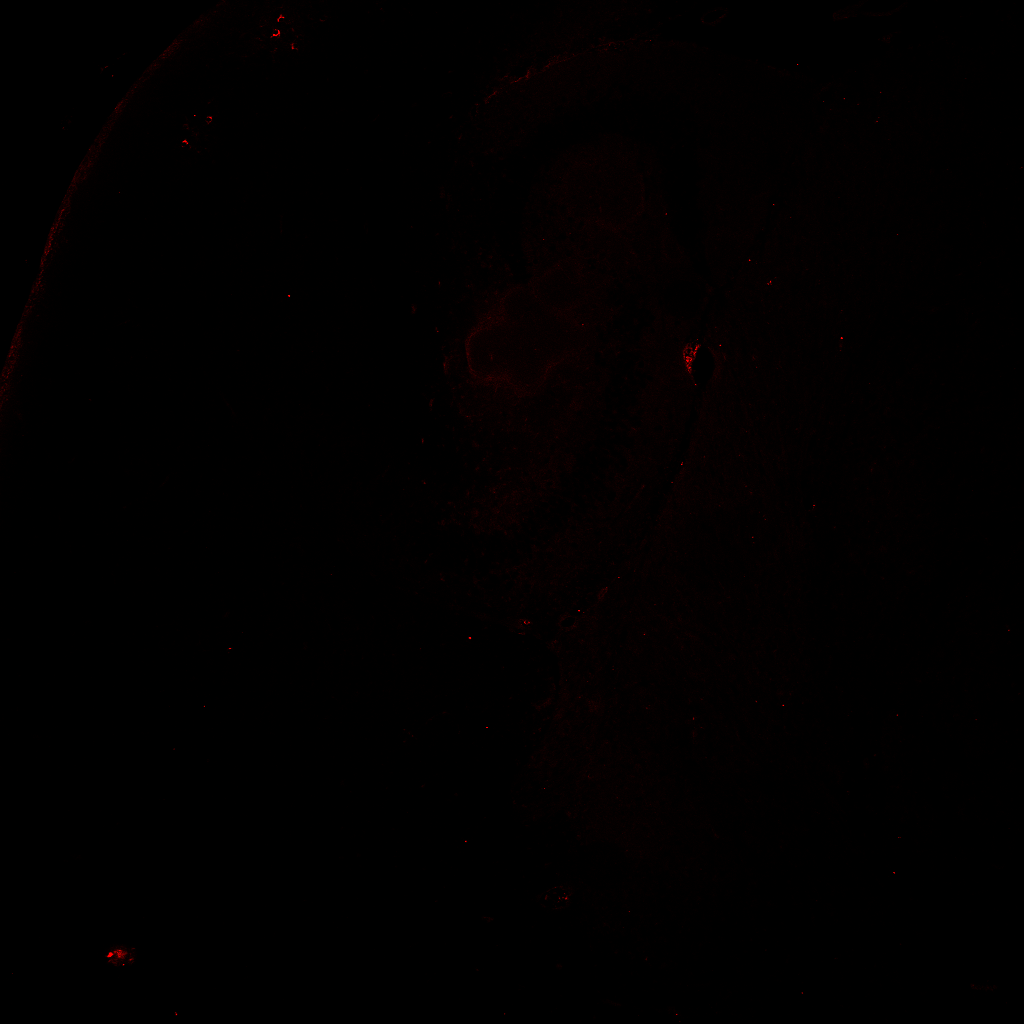

Supplement: Figure 2—source data 4. [file elife-86940-fig2-data4.zip › Figure 2-source data 4/F3094-3-CKO-RX CI FF-1M-SAG-HUB-WFS1-#33-2-5X-vHPC-Image Export-14_AF594-T1.tif]

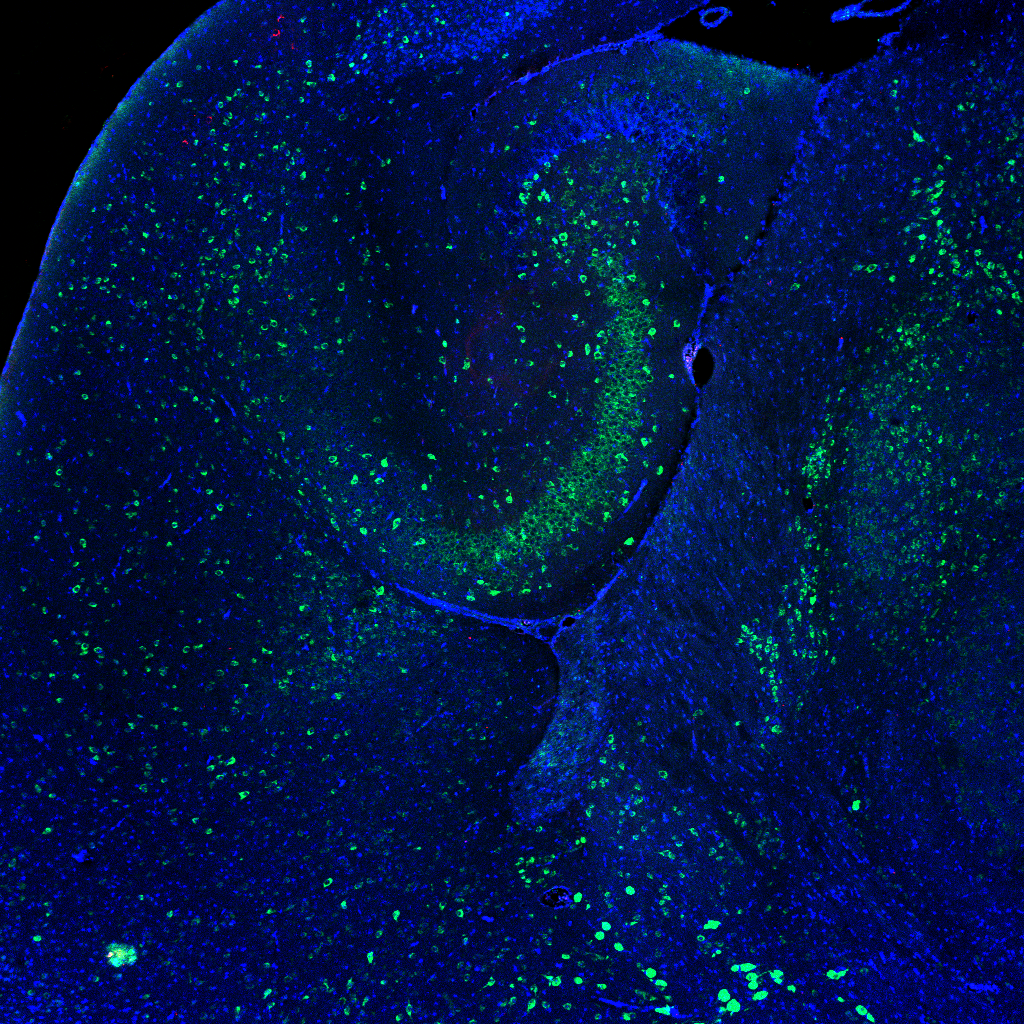

Supplement: Figure 2—source data 4. [file elife-86940-fig2-data4.zip › Figure 2-source data 4/F3094-3-CKO-RX CI FF-1M-SAG-HUB-WFS1-#33-2-5X-vHPC-Image Export-14.tif]

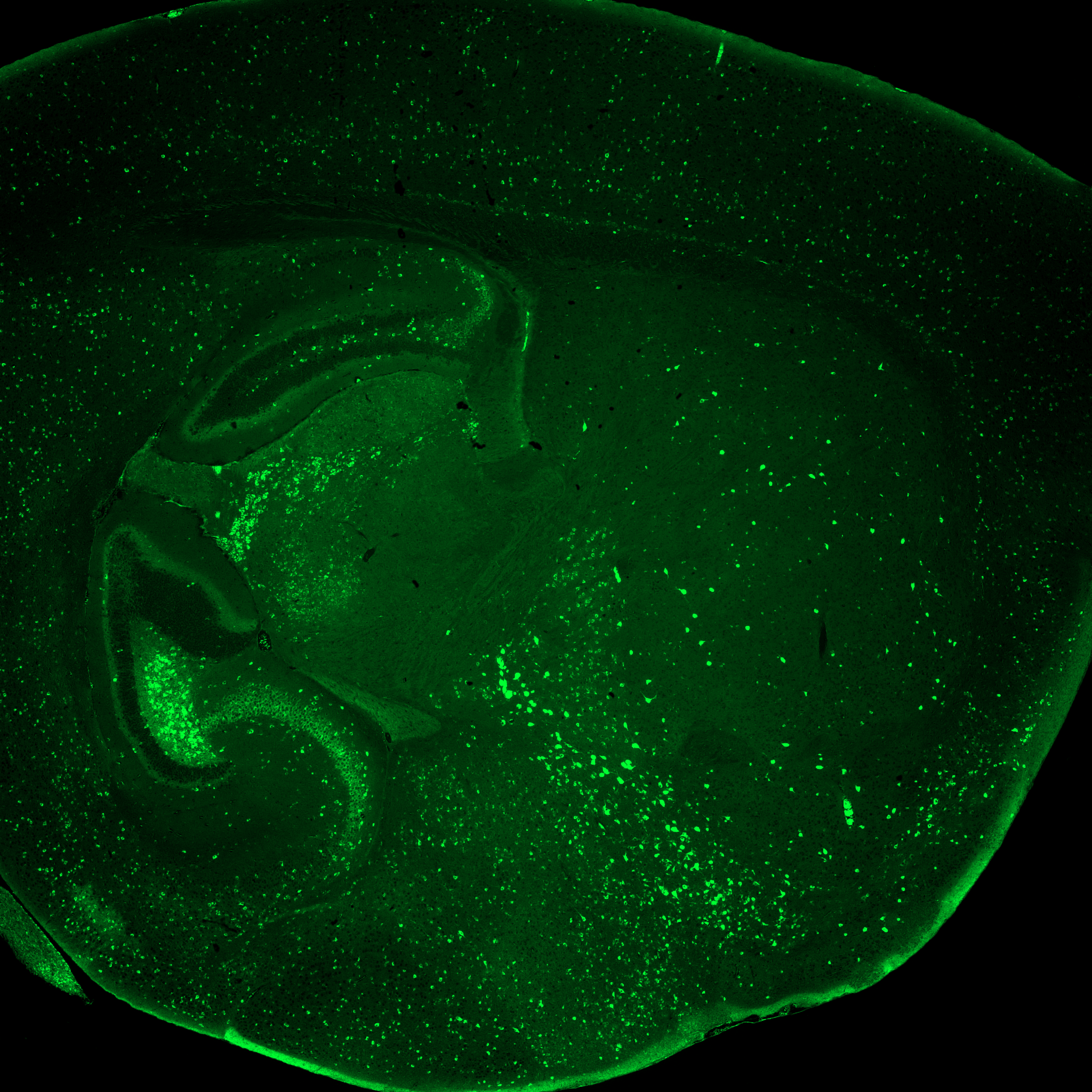

Supplement: Figure 2—source data 4. [file elife-86940-fig2-data4.zip › Figure 2-source data 4/F3094-3-CKO-RX CI FF-1M-SAGITAL-HUB-CTIP2-24#-2-2.5X-HPC-Image Export-06_AF488.tif]

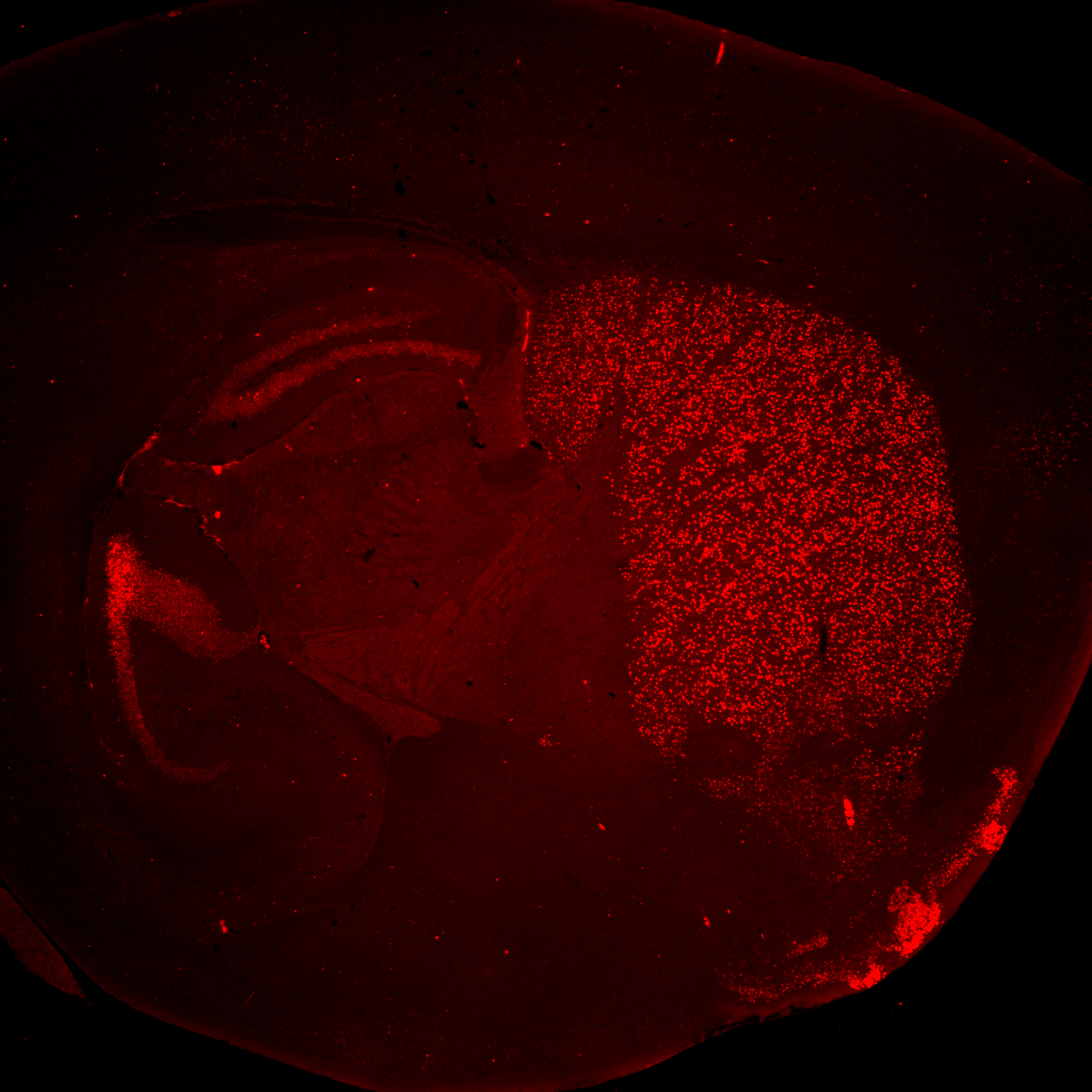

Supplement: Figure 2—source data 4. [file elife-86940-fig2-data4.zip › Figure 2-source data 4/F3094-3-CKO-RX CI FF-1M-SAGITAL-HUB-CTIP2-24#-2-2.5X-HPC-Image Export-06_AF594.tif]

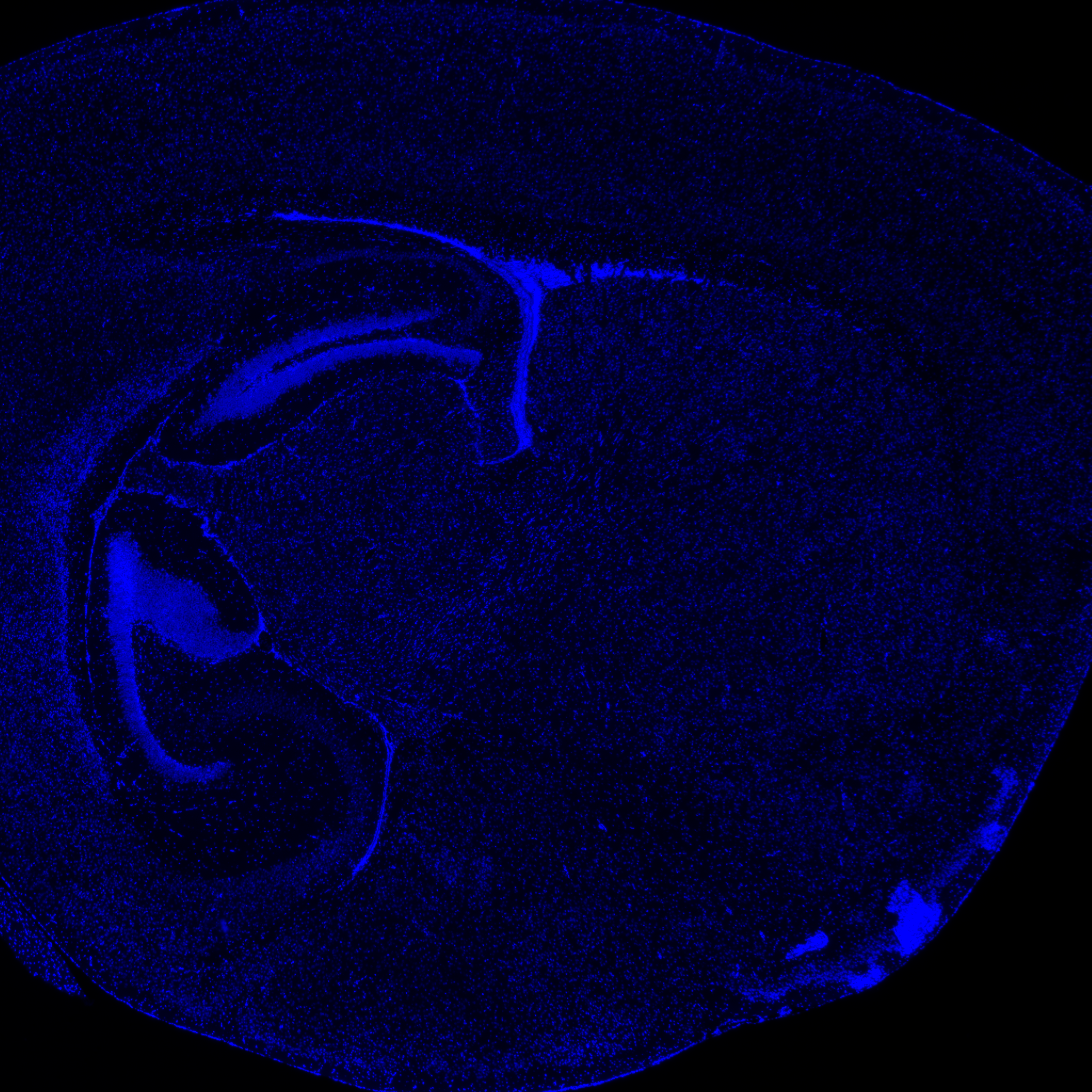

Supplement: Figure 2—source data 4. [file elife-86940-fig2-data4.zip › Figure 2-source data 4/F3094-3-CKO-RX CI FF-1M-SAGITAL-HUB-CTIP2-24#-2-2.5X-HPC-Image Export-06_DAPI.tif]

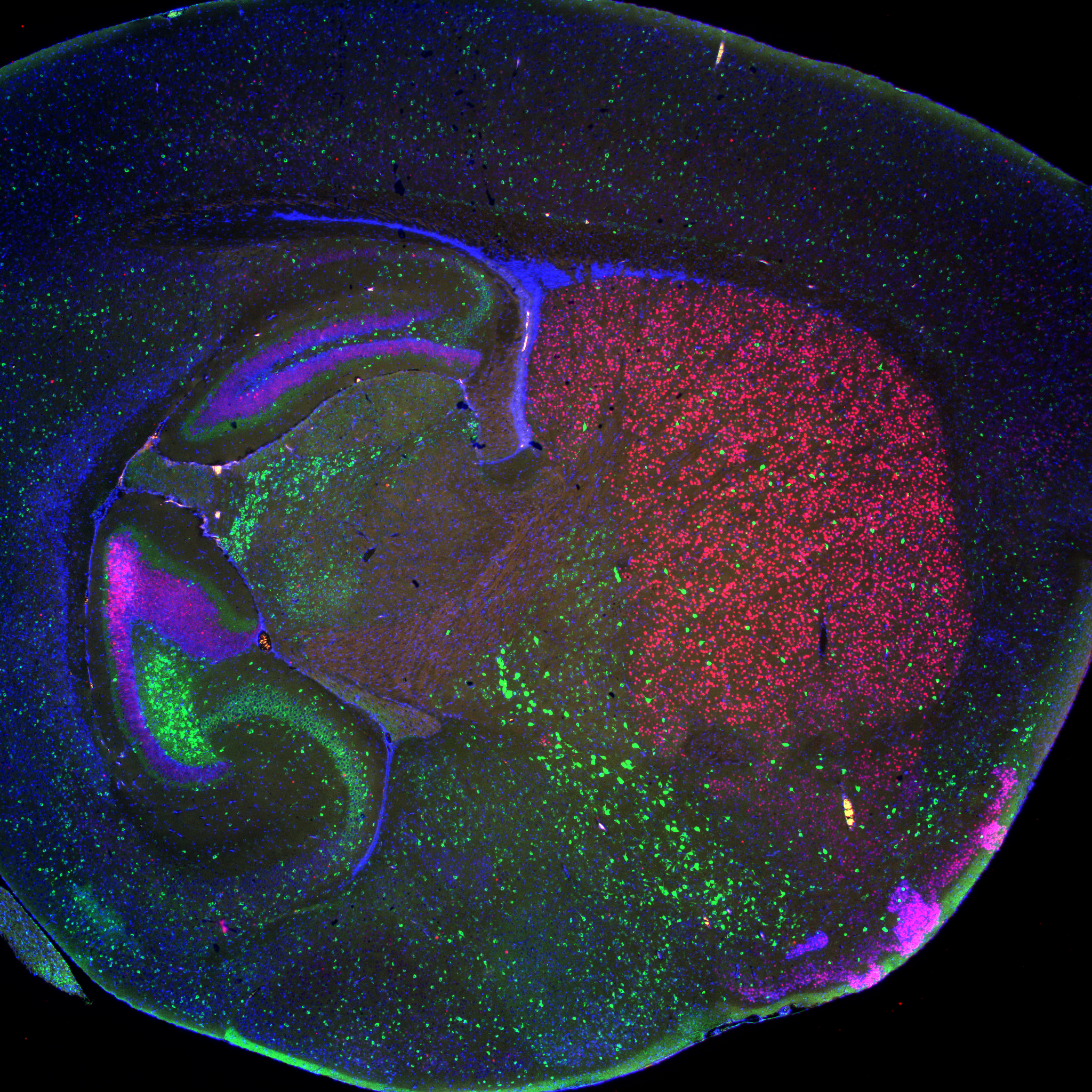

Supplement: Figure 2—source data 4. [file elife-86940-fig2-data4.zip › Figure 2-source data 4/F3094-3-CKO-RX CI FF-1M-SAGITAL-HUB-CTIP2-24#-2-2.5X-HPC-Image Export-06.tif]

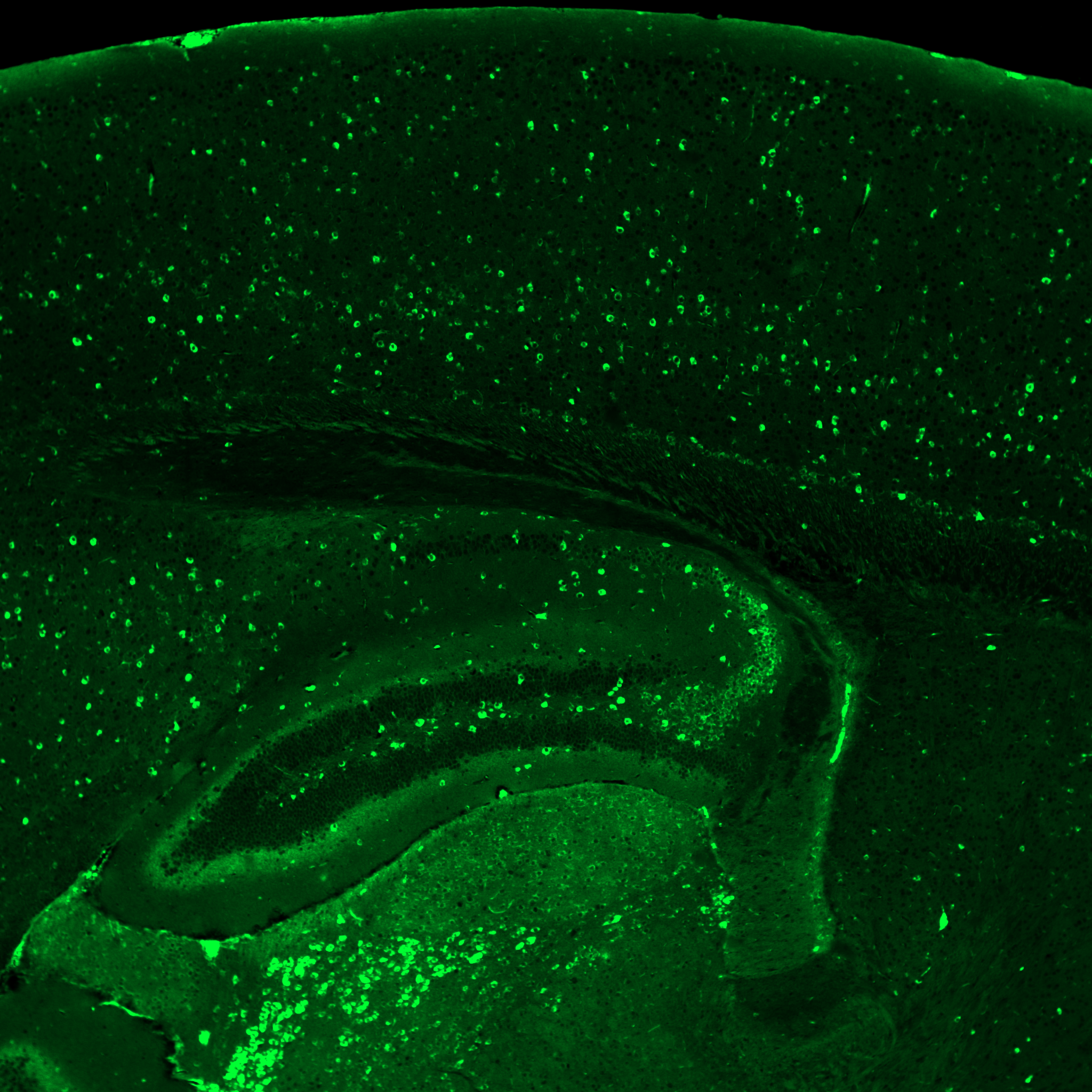

Supplement: Figure 2—source data 4. [file elife-86940-fig2-data4.zip › Figure 2-source data 4/F3094-3-CKO-RX CI FF-1M-SAGITAL-HUB-CTIP2-24#-2-5X-dHPC-Image Export-07_AF488.tif]

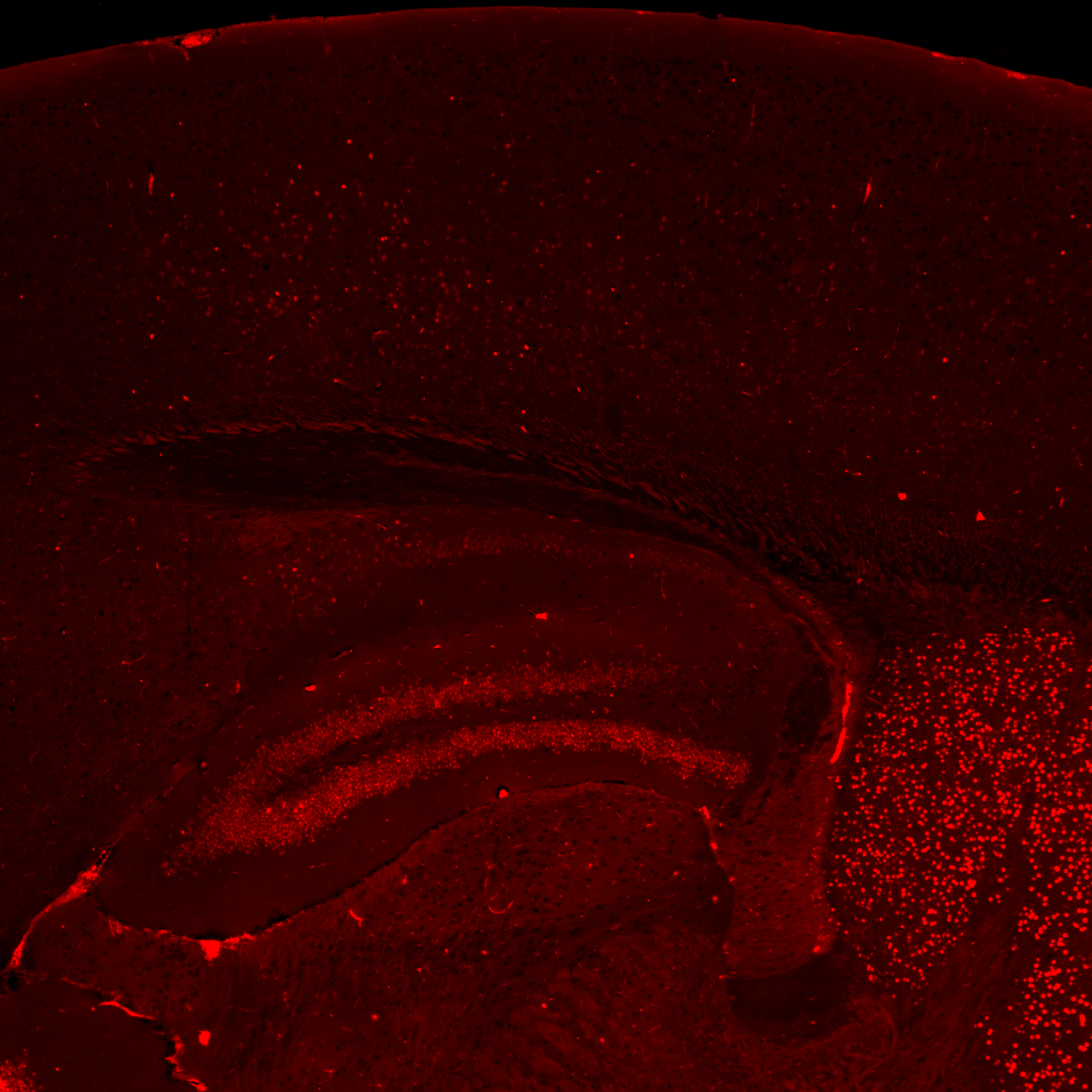

Supplement: Figure 2—source data 4. [file elife-86940-fig2-data4.zip › Figure 2-source data 4/F3094-3-CKO-RX CI FF-1M-SAGITAL-HUB-CTIP2-24#-2-5X-dHPC-Image Export-07_AF594.tif]

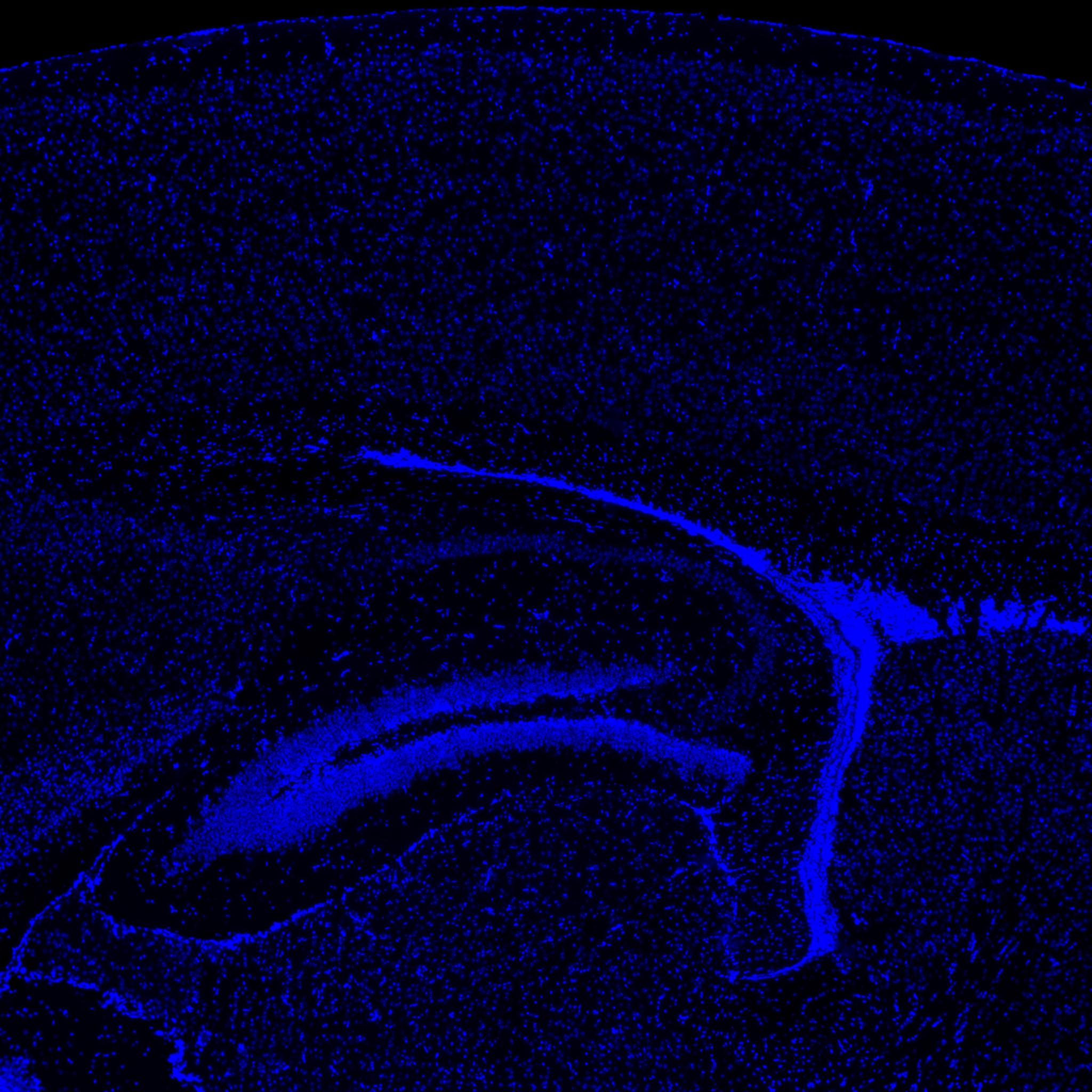

Supplement: Figure 2—source data 4. [file elife-86940-fig2-data4.zip › Figure 2-source data 4/F3094-3-CKO-RX CI FF-1M-SAGITAL-HUB-CTIP2-24#-2-5X-dHPC-Image Export-07_DAPI.tif]

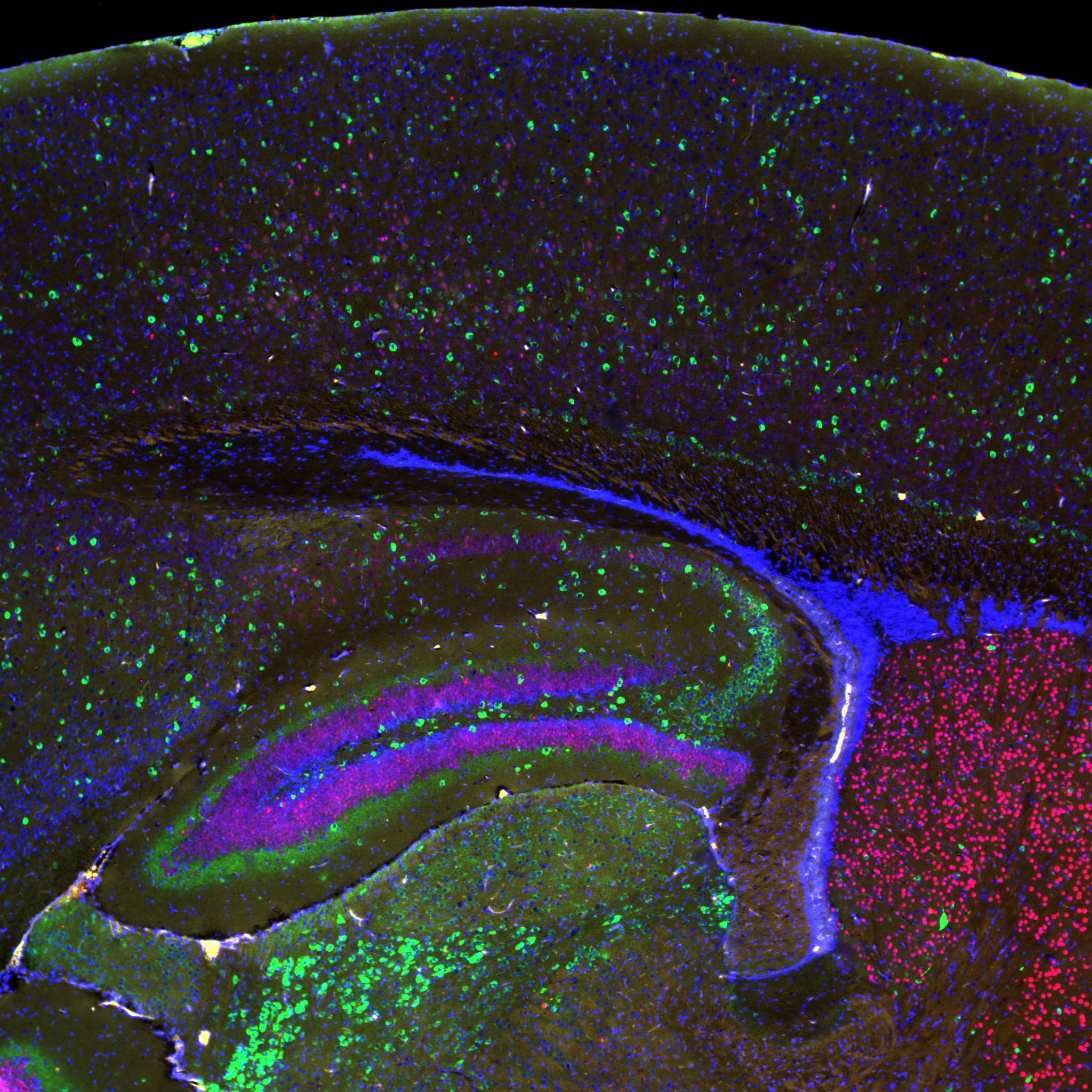

Supplement: Figure 2—source data 4. [file elife-86940-fig2-data4.zip › Figure 2-source data 4/F3094-3-CKO-RX CI FF-1M-SAGITAL-HUB-CTIP2-24#-2-5X-dHPC-Image Export-07.tif]

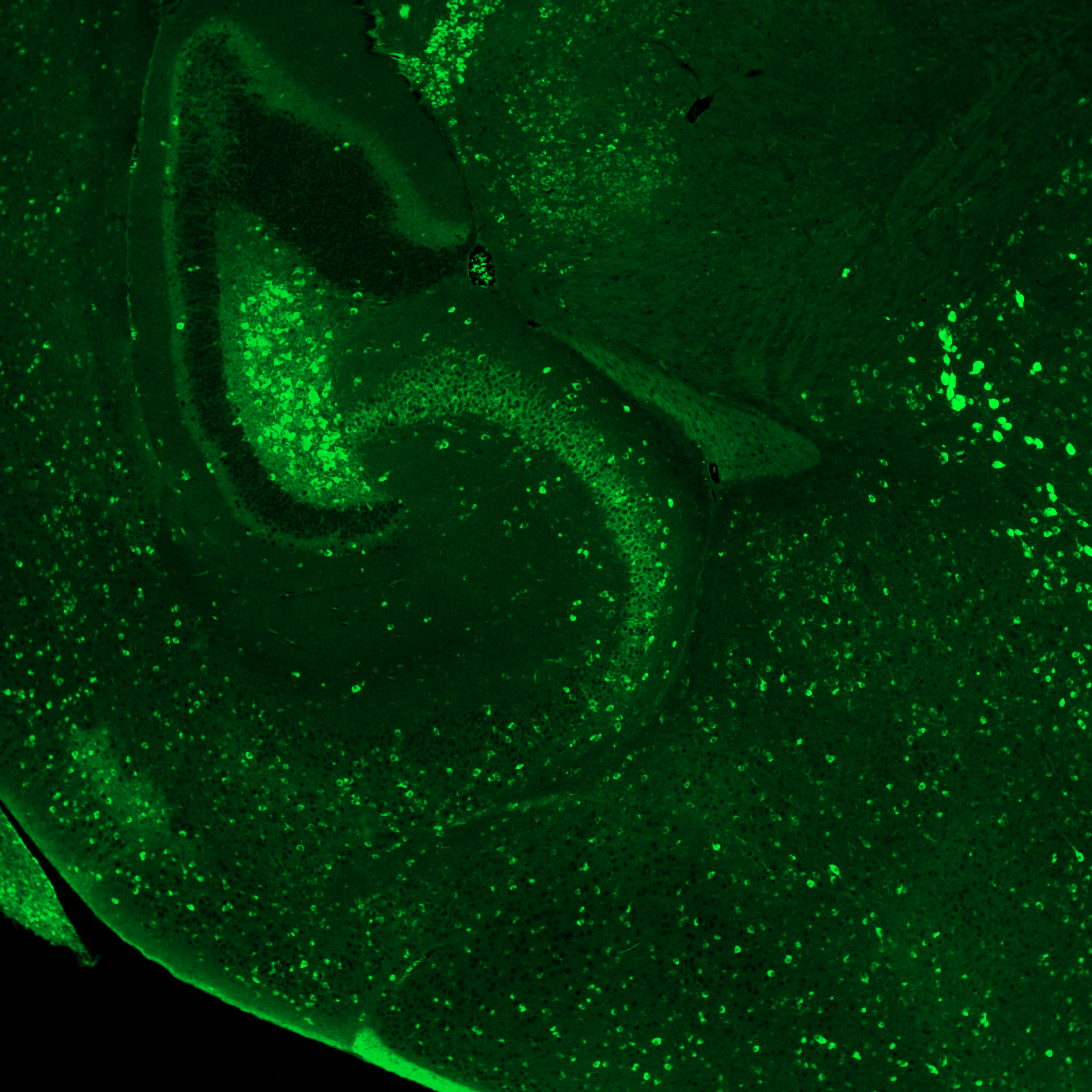

Supplement: Figure 2—source data 4. [file elife-86940-fig2-data4.zip › Figure 2-source data 4/F3094-3-CKO-RX CI FF-1M-SAGITAL-HUB-CTIP2-24#-2-5X-vHPC-Image Export-08_AF488.tif]

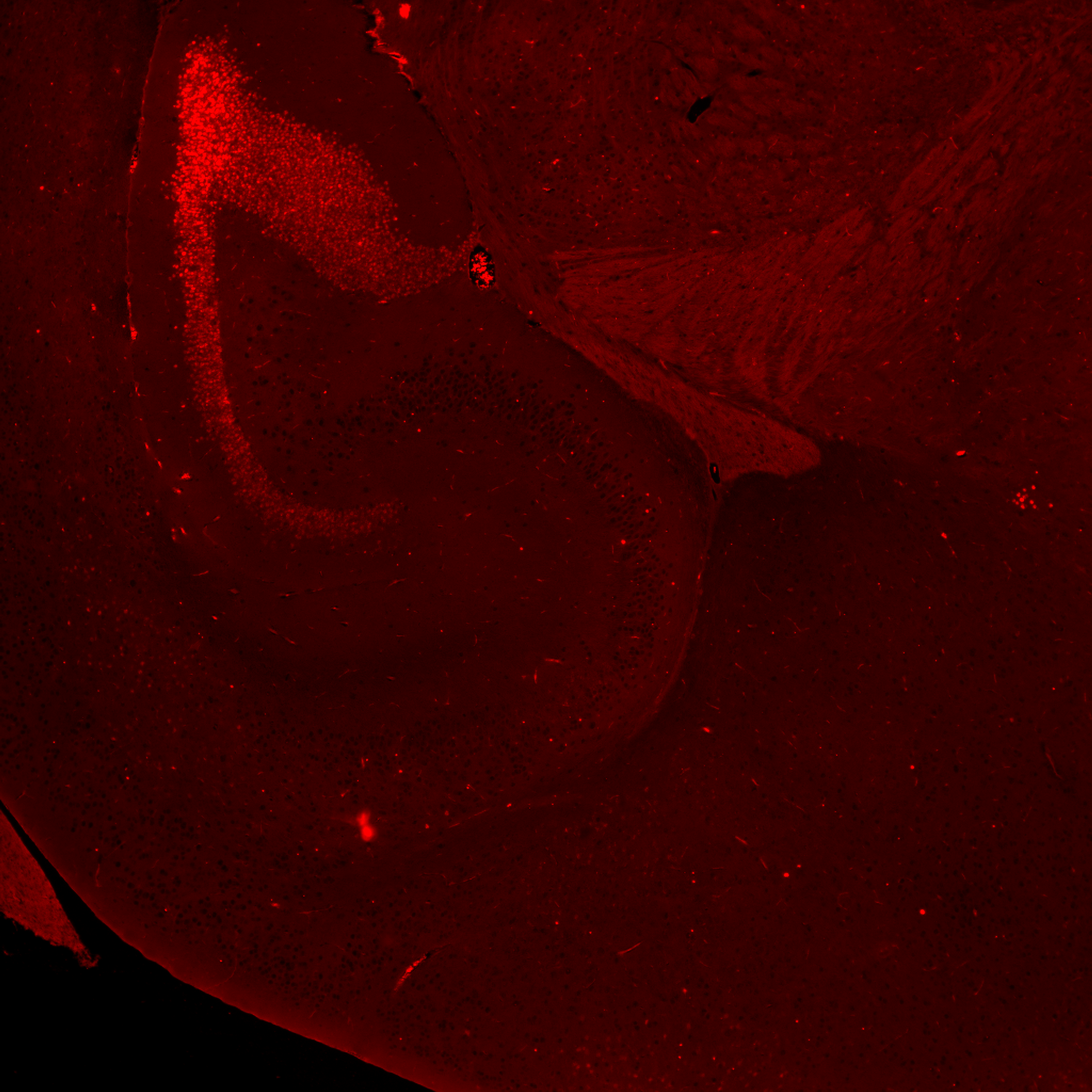

Supplement: Figure 2—source data 4. [file elife-86940-fig2-data4.zip › Figure 2-source data 4/F3094-3-CKO-RX CI FF-1M-SAGITAL-HUB-CTIP2-24#-2-5X-vHPC-Image Export-08_AF594.tif]

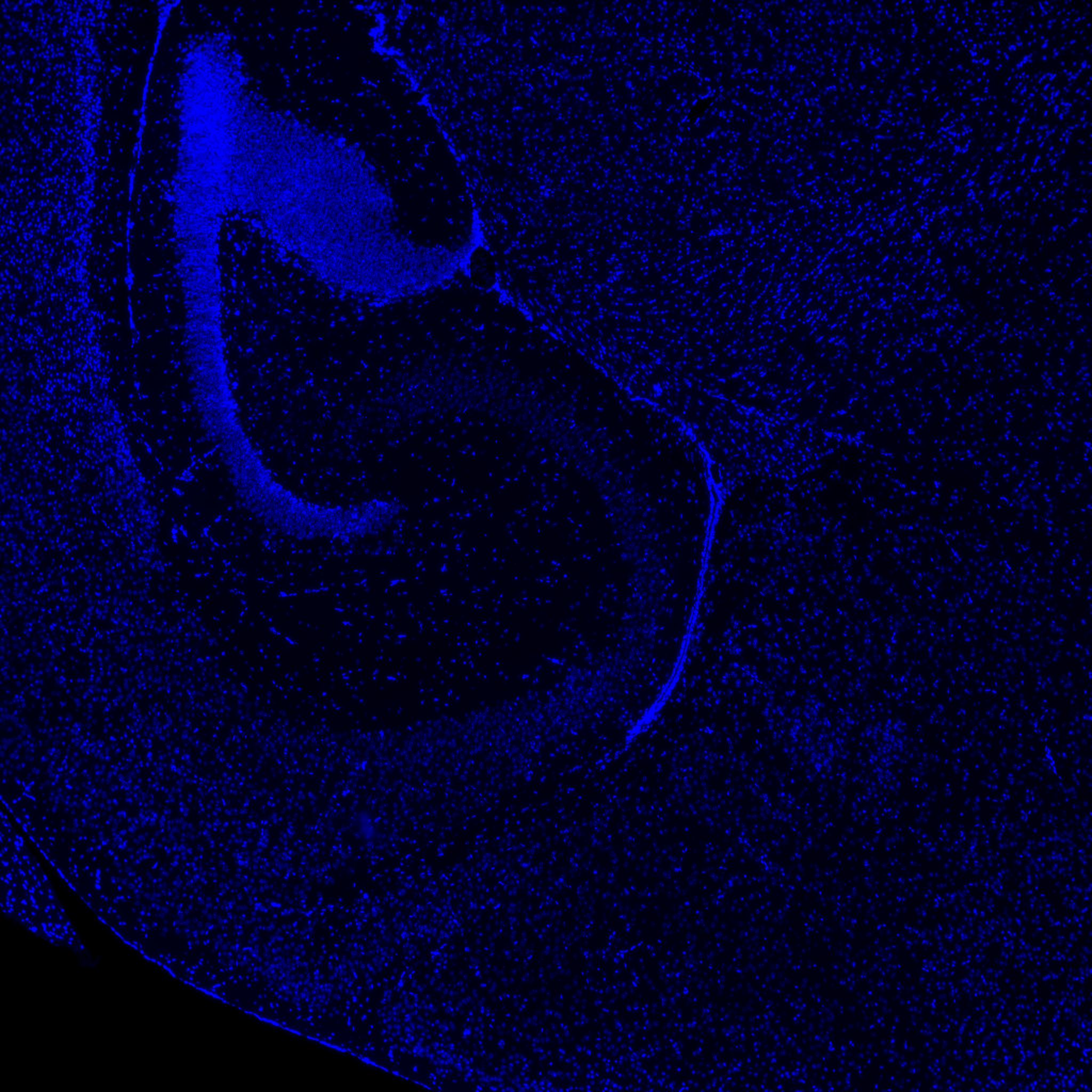

Supplement: Figure 2—source data 4. [file elife-86940-fig2-data4.zip › Figure 2-source data 4/F3094-3-CKO-RX CI FF-1M-SAGITAL-HUB-CTIP2-24#-2-5X-vHPC-Image Export-08_DAPI.tif]

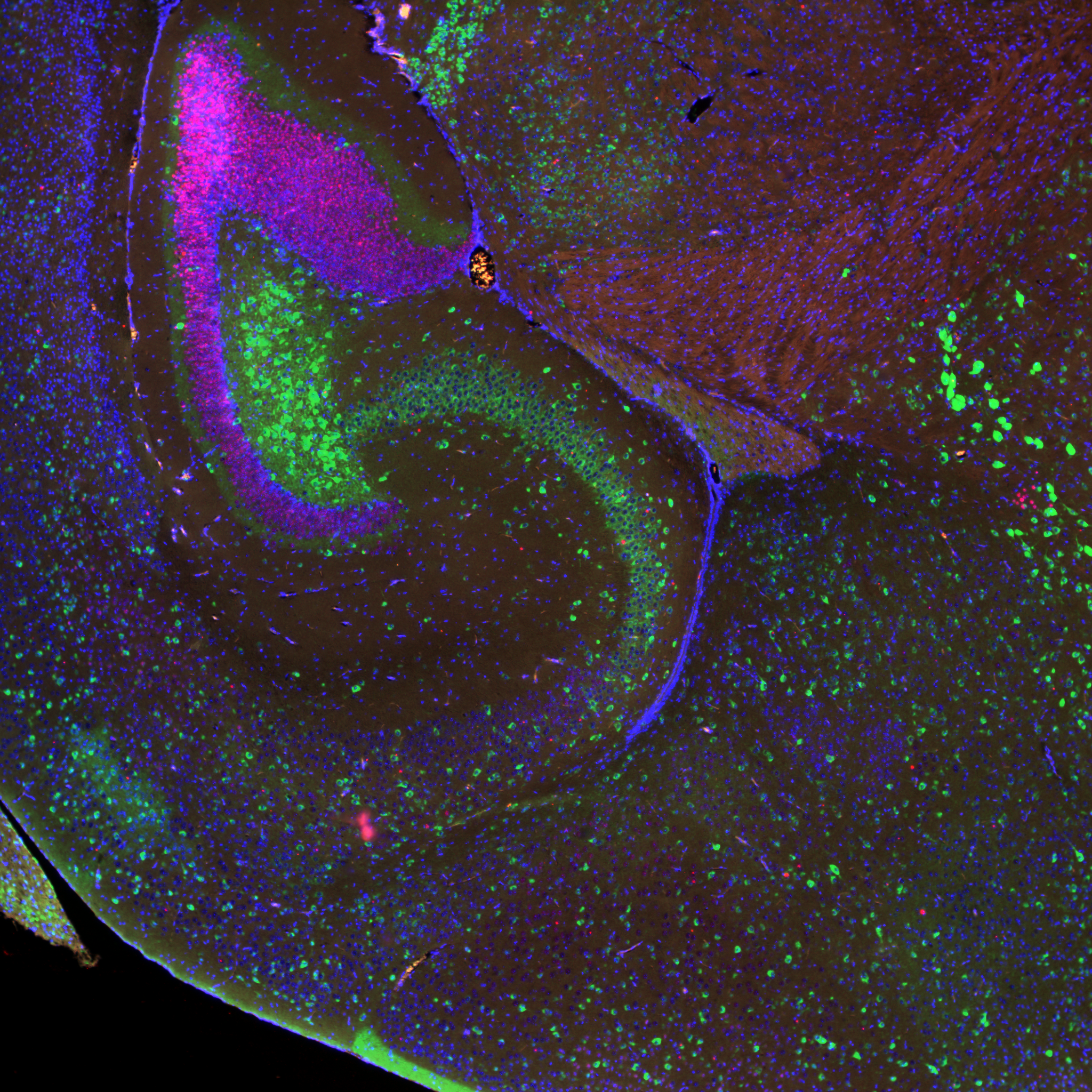

Supplement: Figure 2—source data 4. [file elife-86940-fig2-data4.zip › Figure 2-source data 4/F3094-3-CKO-RX CI FF-1M-SAGITAL-HUB-CTIP2-24#-2-5X-vHPC-Image Export-08.tif]

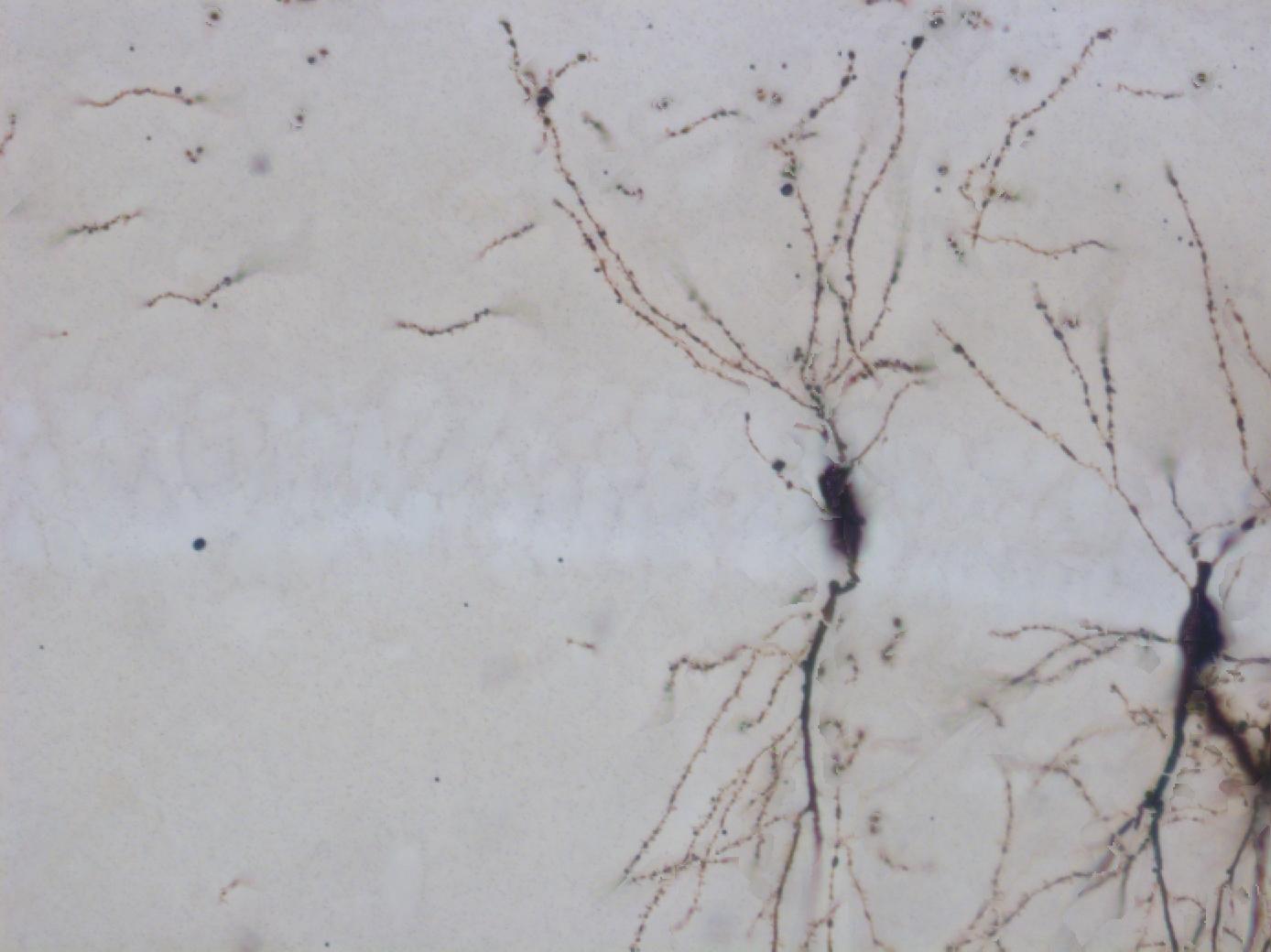

Supplement: Figure 2—figure supplement 1—source data 1. [file elife-86940-fig2-figsupp1-data1.zip › Figure 2-figure supplement 1-source data 1/2064-MUT-1-5-40X-1-2.jpg]

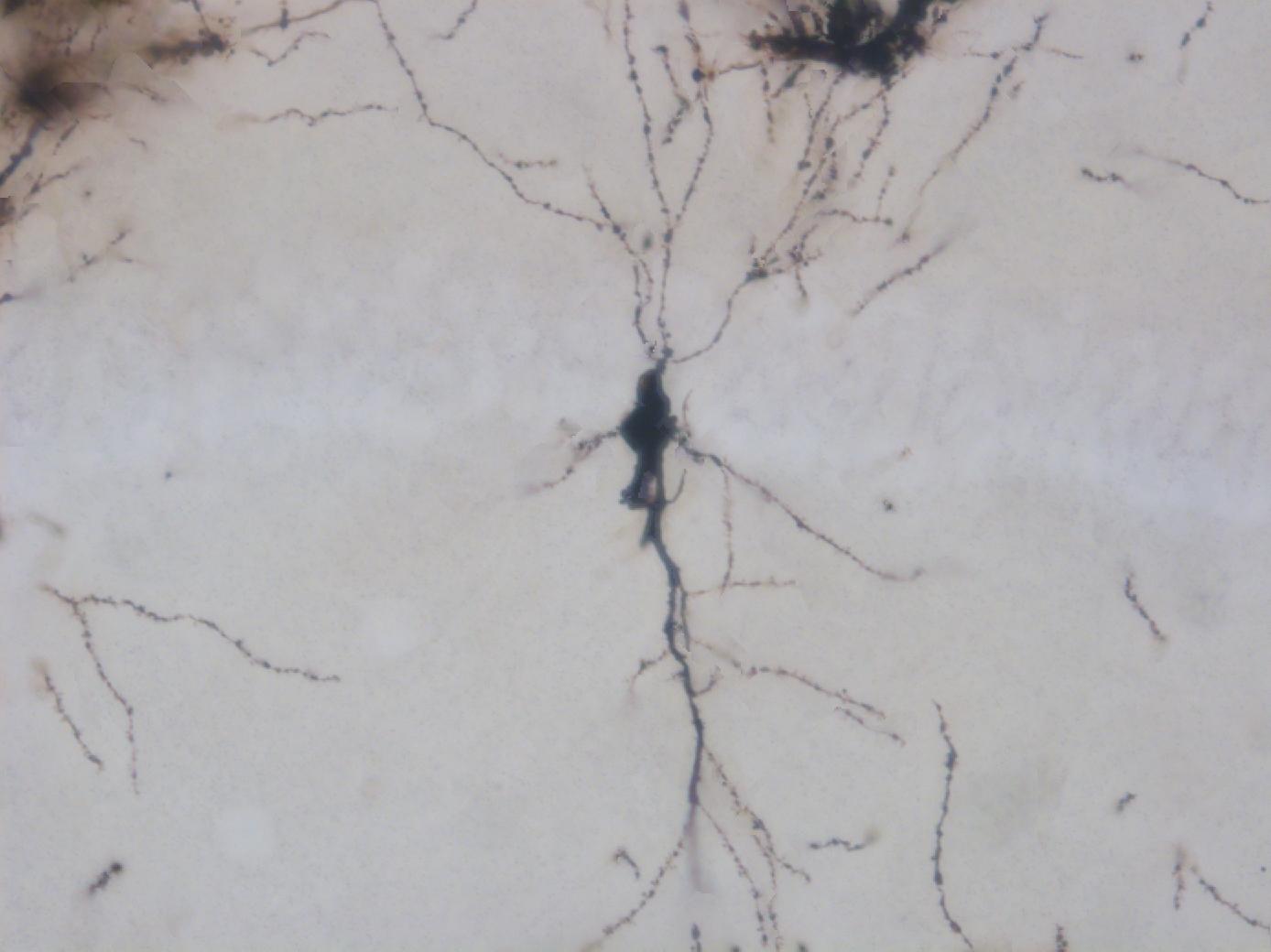

Supplement: Figure 2—figure supplement 1—source data 1. [file elife-86940-fig2-figsupp1-data1.zip › Figure 2-figure supplement 1-source data 1/2064-MUT-1-5-40X-1.jpg]

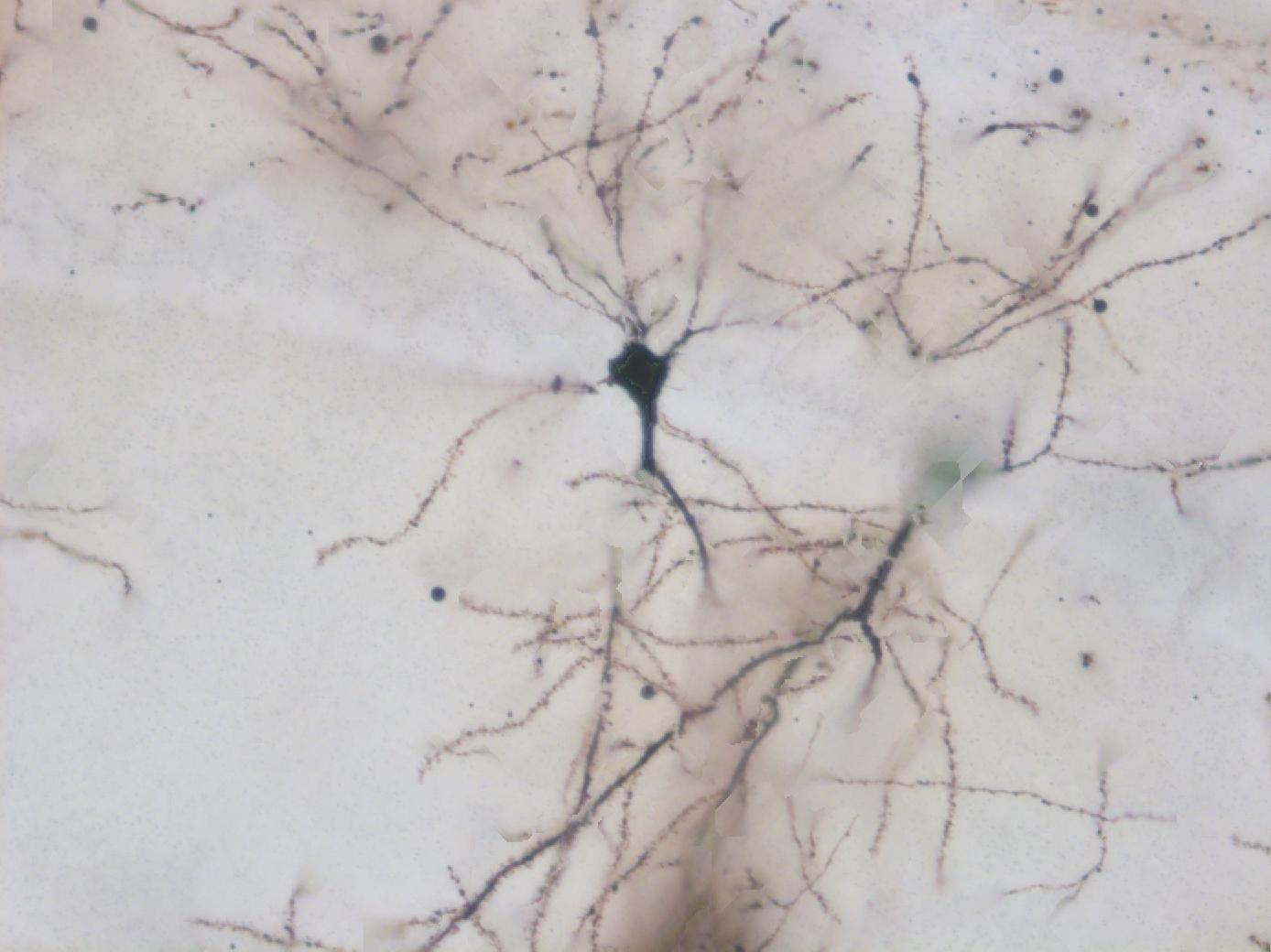

Supplement: Figure 2—figure supplement 1—source data 1. [file elife-86940-fig2-figsupp1-data1.zip › Figure 2-figure supplement 1-source data 1/2065-CON-3-2-40X.jpg]

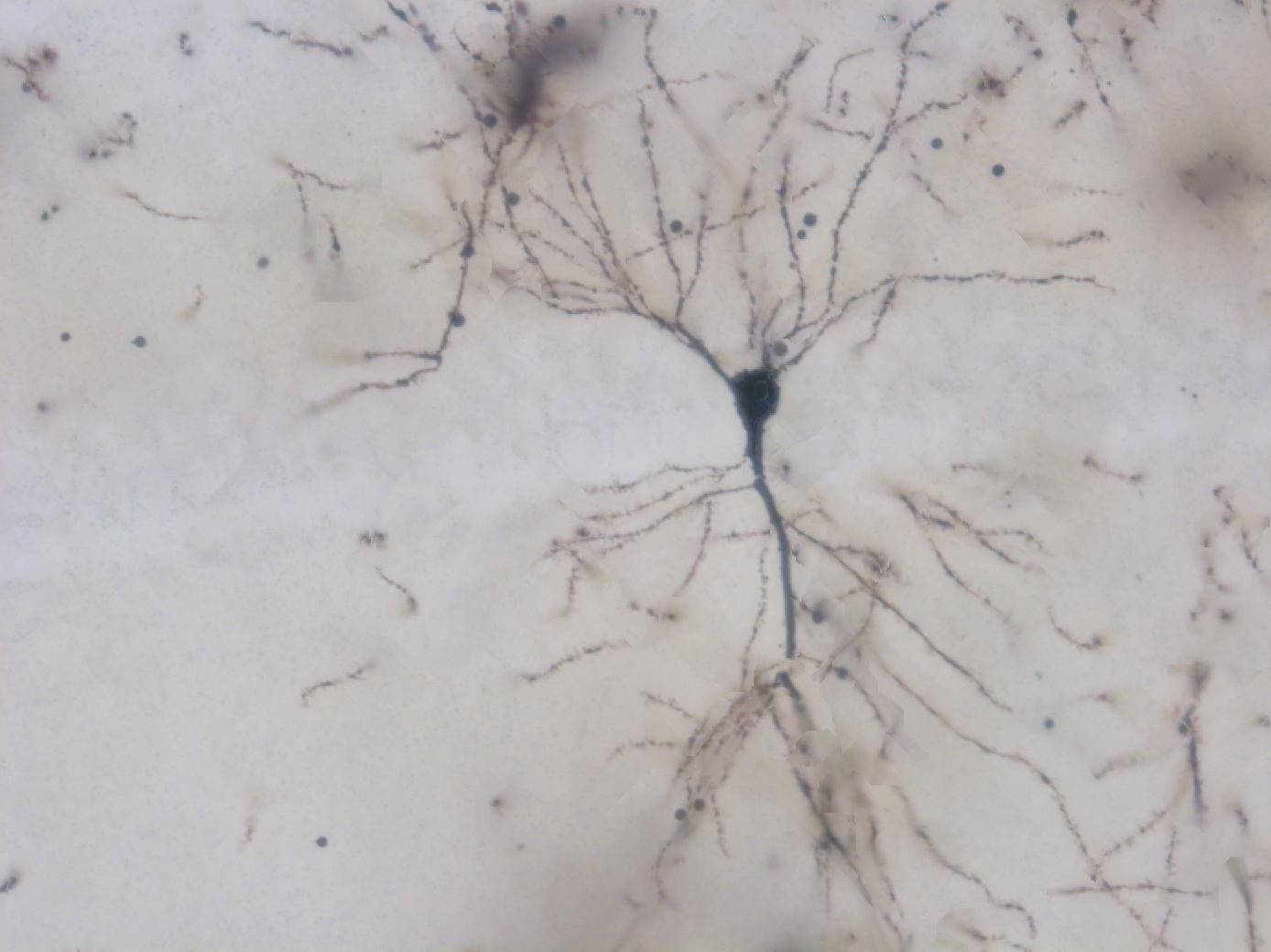

Supplement: Figure 2—figure supplement 1—source data 1. [file elife-86940-fig2-figsupp1-data1.zip › Figure 2-figure supplement 1-source data 1/2065-CON-4-1-40X-1.jpg]

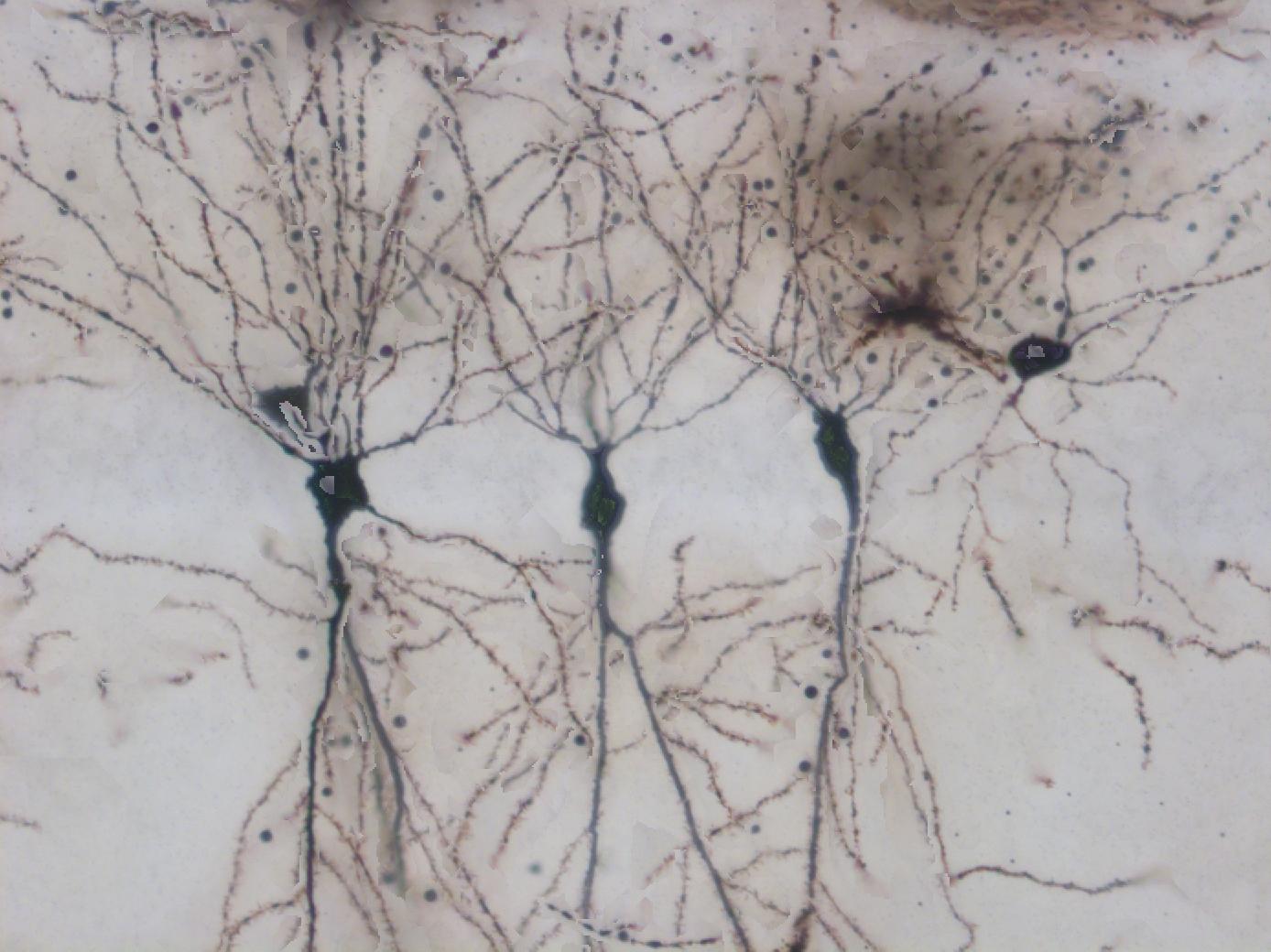

Supplement: Figure 2—figure supplement 1—source data 1. [file elife-86940-fig2-figsupp1-data1.zip › Figure 2-figure supplement 1-source data 1/2065-CON-4-1-40X-2.jpg]

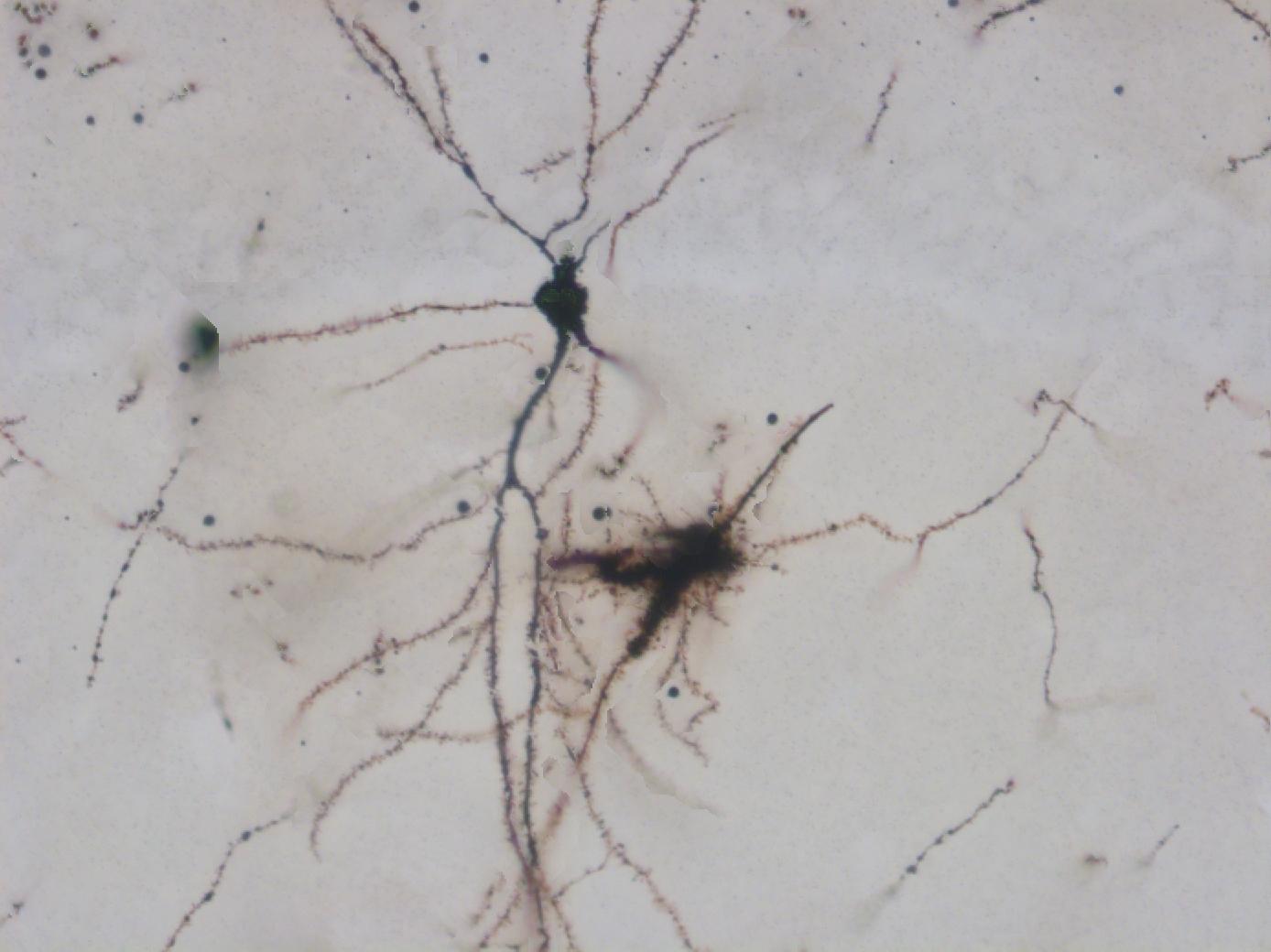

Supplement: Figure 2—figure supplement 1—source data 1. [file elife-86940-fig2-figsupp1-data1.zip › Figure 2-figure supplement 1-source data 1/2065-CON-4-1-40X-3.jpg]

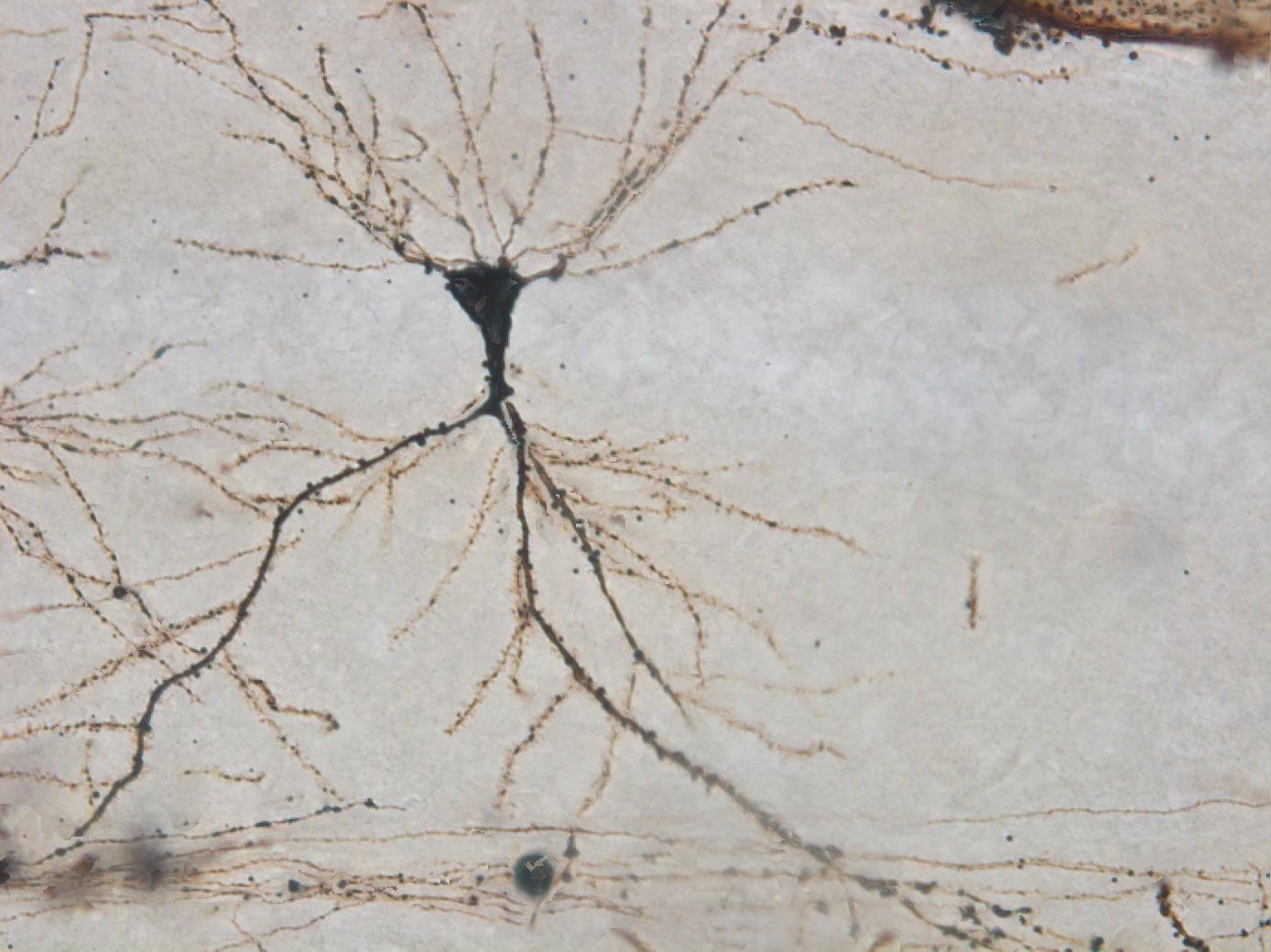

Supplement: Figure 2—figure supplement 1—source data 1. [file elife-86940-fig2-figsupp1-data1.zip › Figure 2-figure supplement 1-source data 1/2065-CON-4-3-40X-1.jpg]

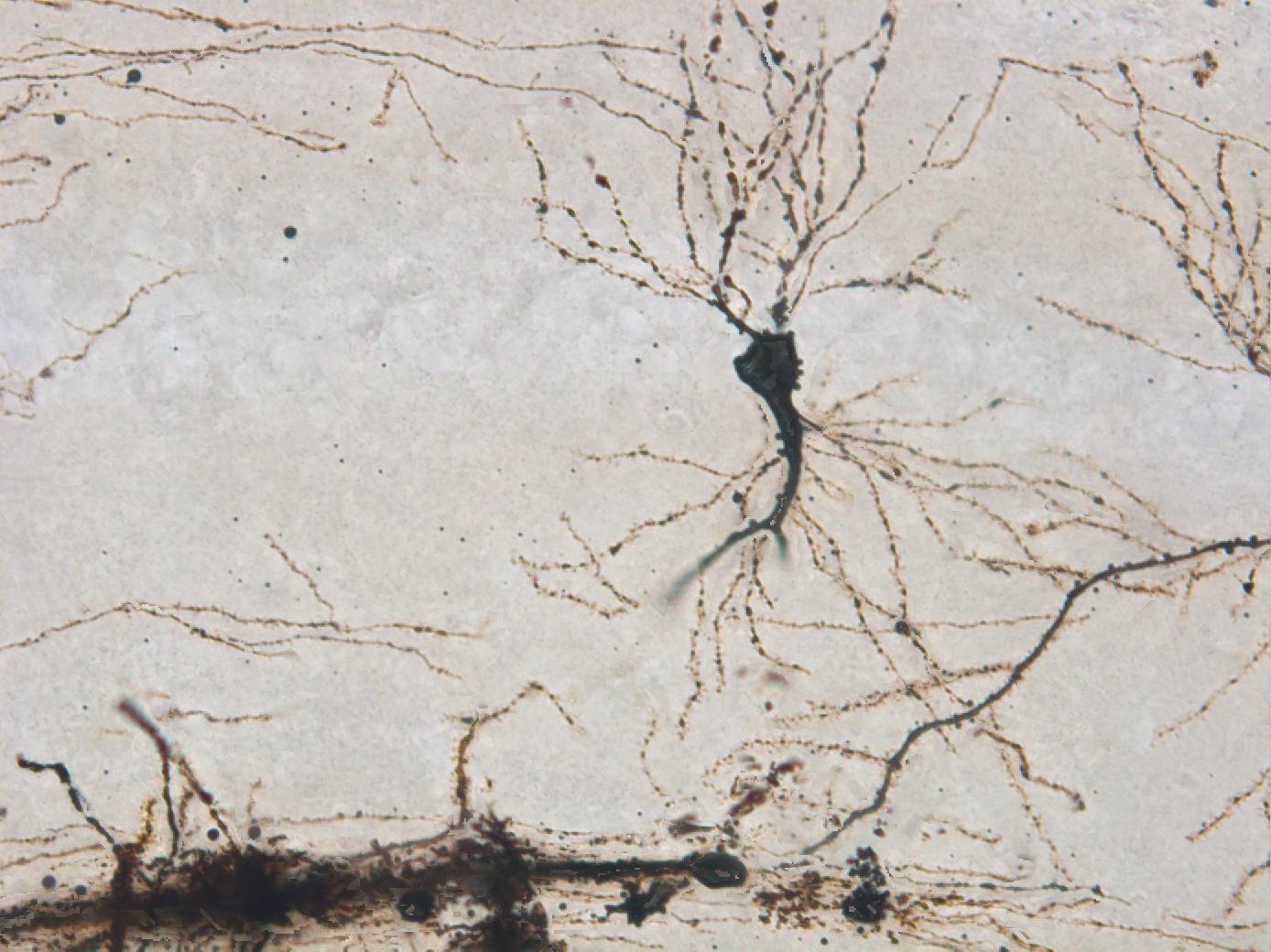

Supplement: Figure 2—figure supplement 1—source data 1. [file elife-86940-fig2-figsupp1-data1.zip › Figure 2-figure supplement 1-source data 1/2065-CON-4-3-40X-2.jpg]

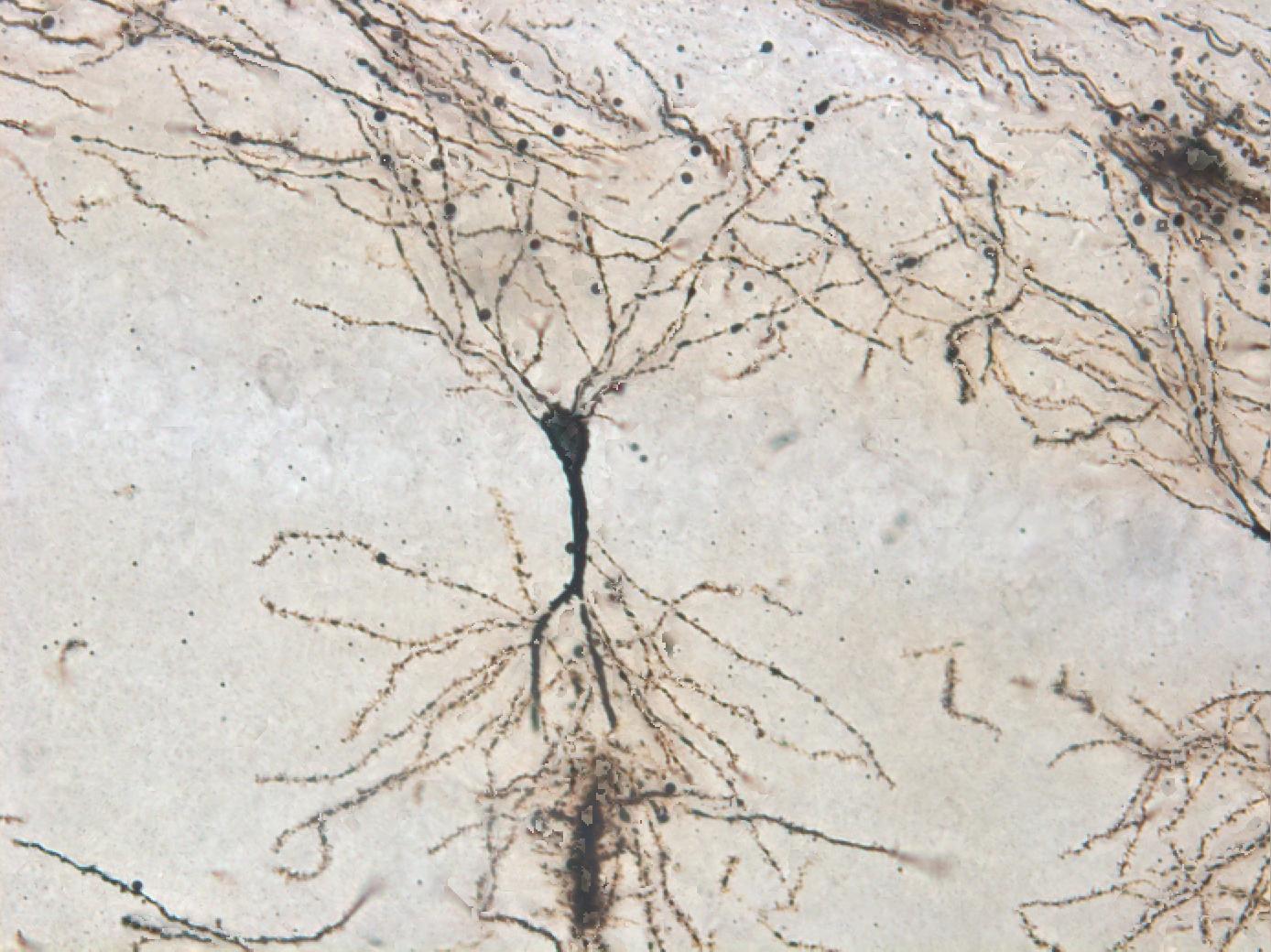

Supplement: Figure 2—figure supplement 1—source data 1. [file elife-86940-fig2-figsupp1-data1.zip › Figure 2-figure supplement 1-source data 1/2065-CON-5-5-40X-2.jpg]

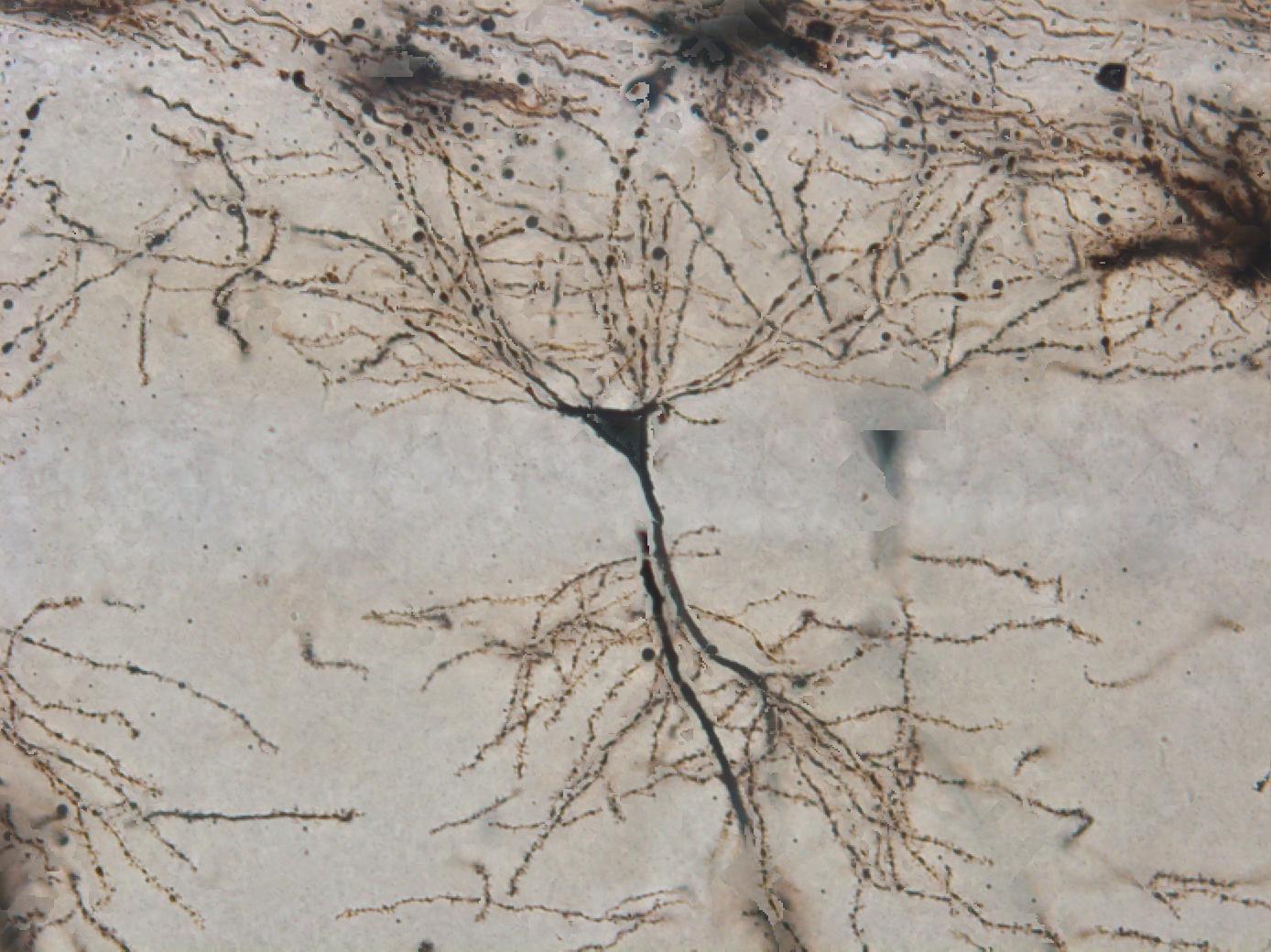

Supplement: Figure 2—figure supplement 1—source data 1. [file elife-86940-fig2-figsupp1-data1.zip › Figure 2-figure supplement 1-source data 1/2065-CON-5-5-40X-3.jpg]

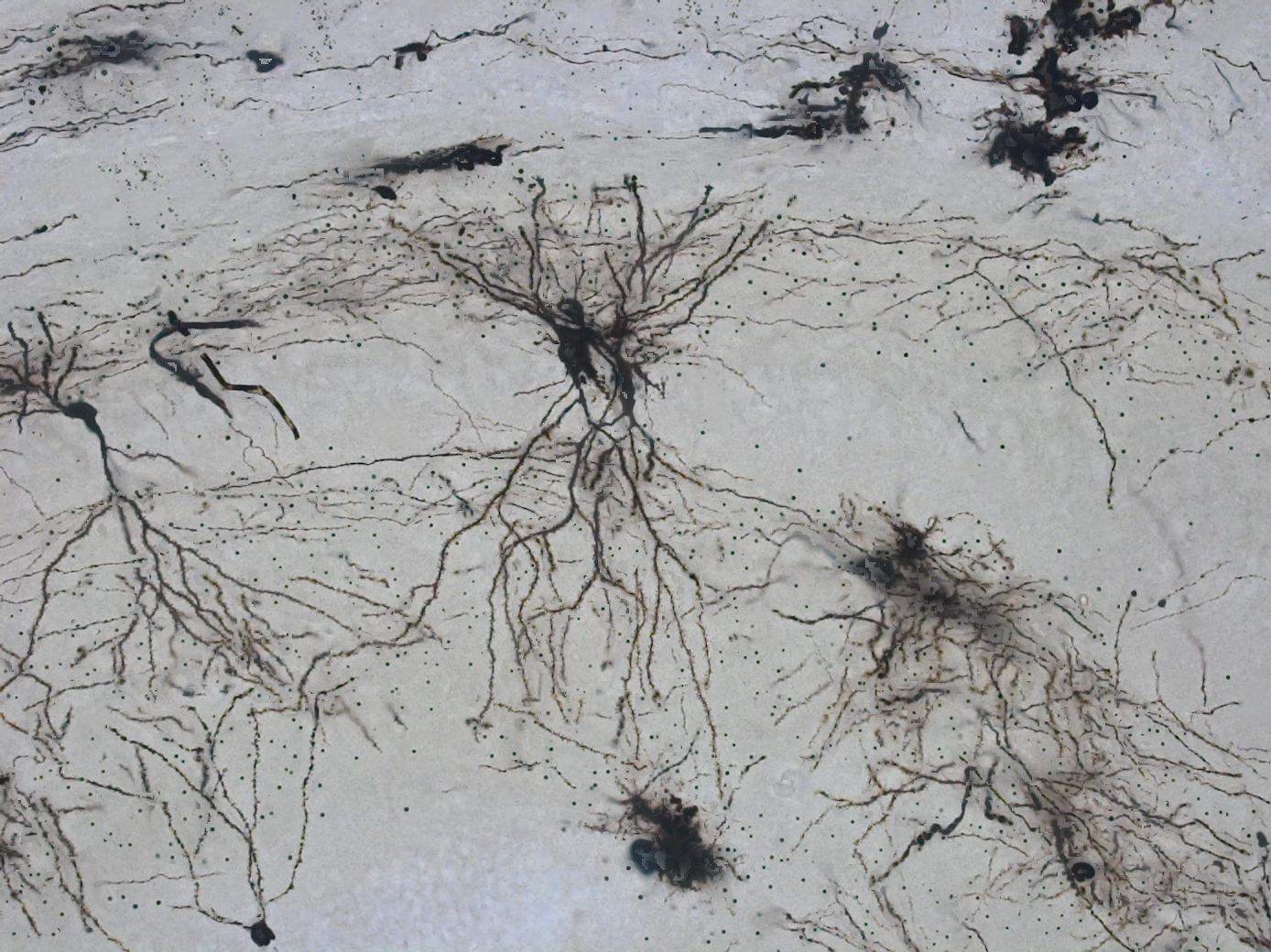

Supplement: Figure 2—figure supplement 1—source data 1. [file elife-86940-fig2-figsupp1-data1.zip › Figure 2-figure supplement 1-source data 1/2130-MUT-20X-2-1.jpg]

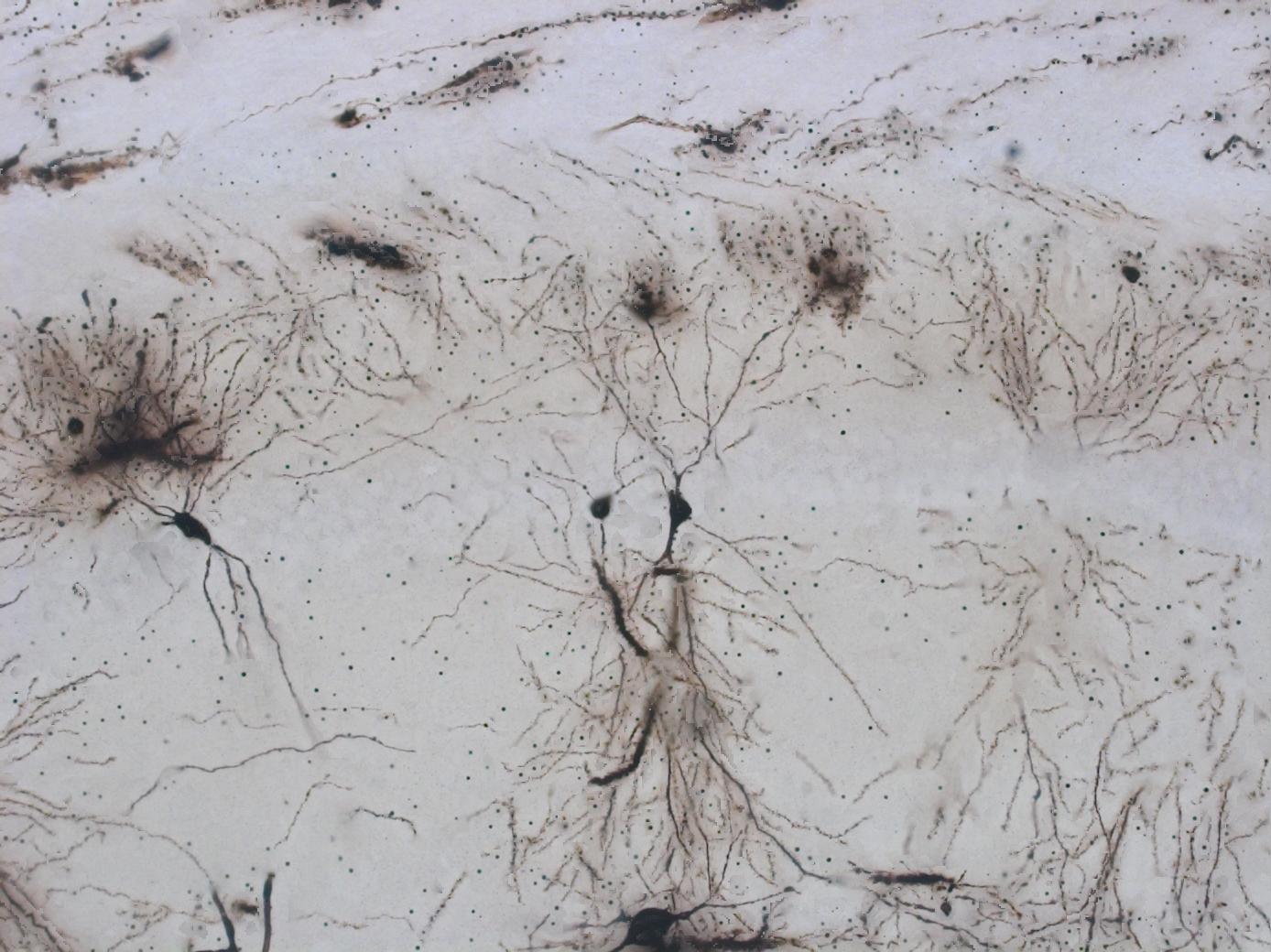

Supplement: Figure 2—figure supplement 1—source data 1. [file elife-86940-fig2-figsupp1-data1.zip › Figure 2-figure supplement 1-source data 1/2130-MUT-20X-7-2.jpg]

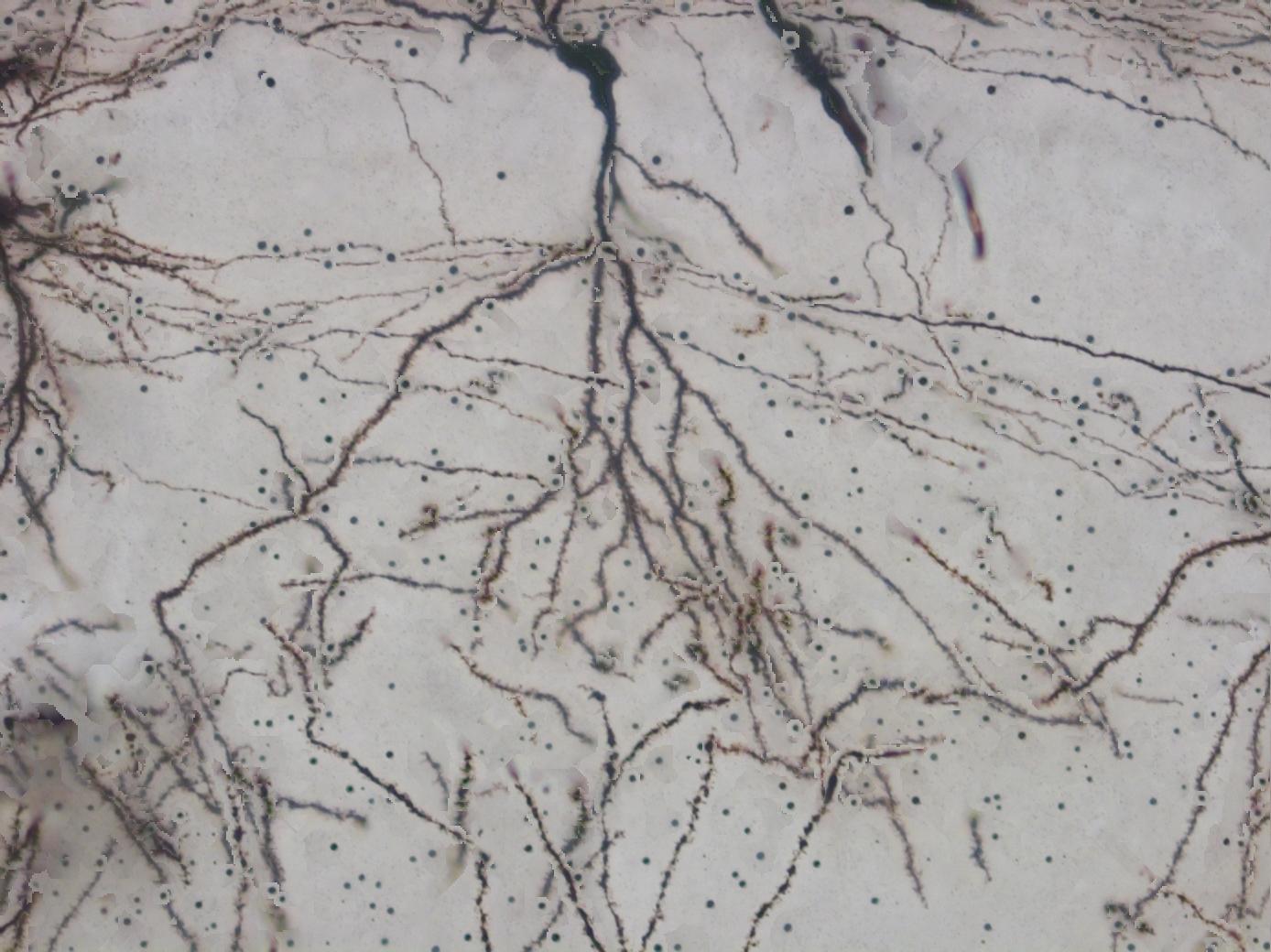

Supplement: Figure 2—figure supplement 1—source data 1. [file elife-86940-fig2-figsupp1-data1.zip › Figure 2-figure supplement 1-source data 1/2130-MUT-40X-2-1a.jpg]

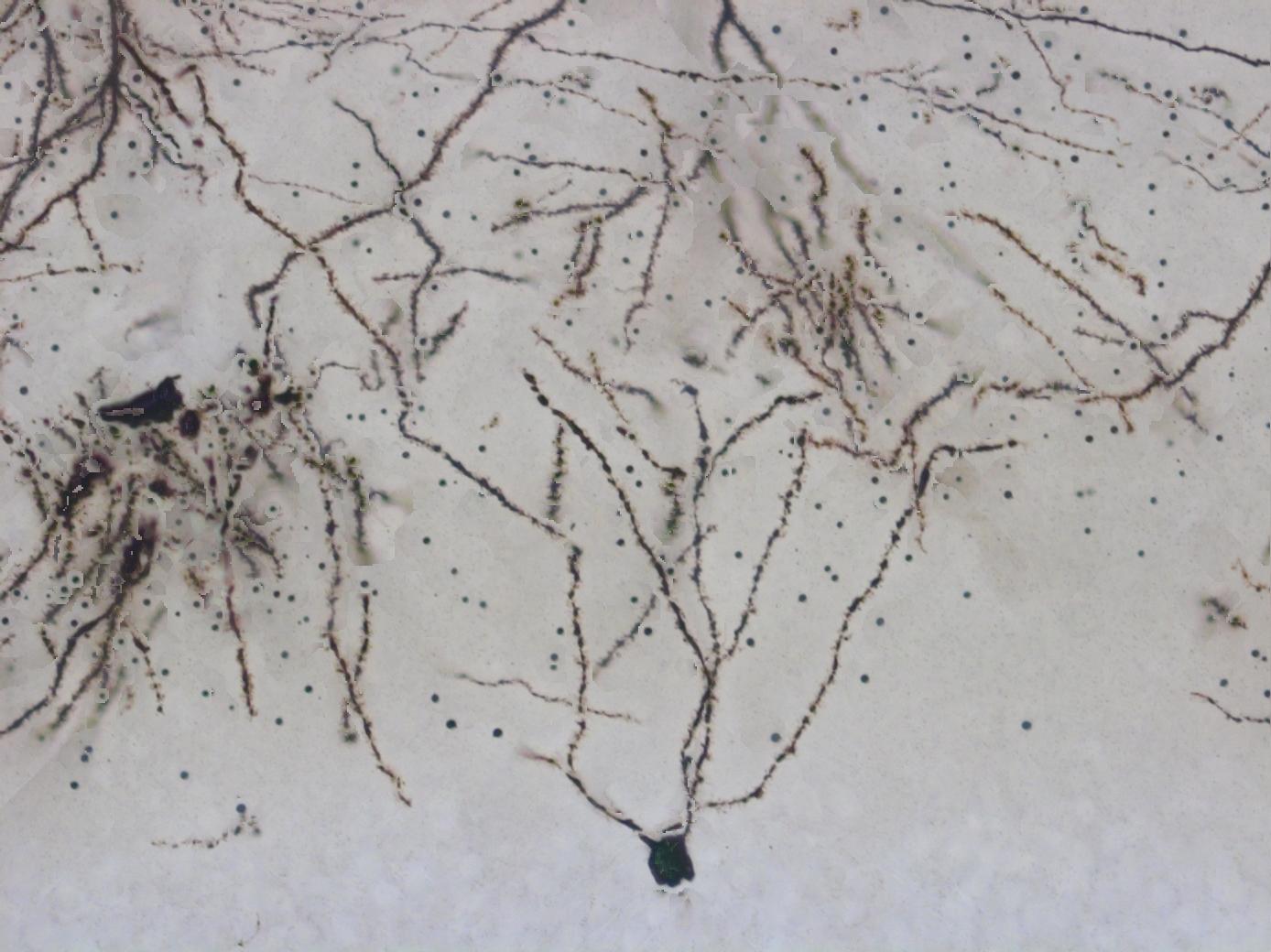

Supplement: Figure 2—figure supplement 1—source data 1. [file elife-86940-fig2-figsupp1-data1.zip › Figure 2-figure supplement 1-source data 1/2130-MUT-40X-2-1b.jpg]

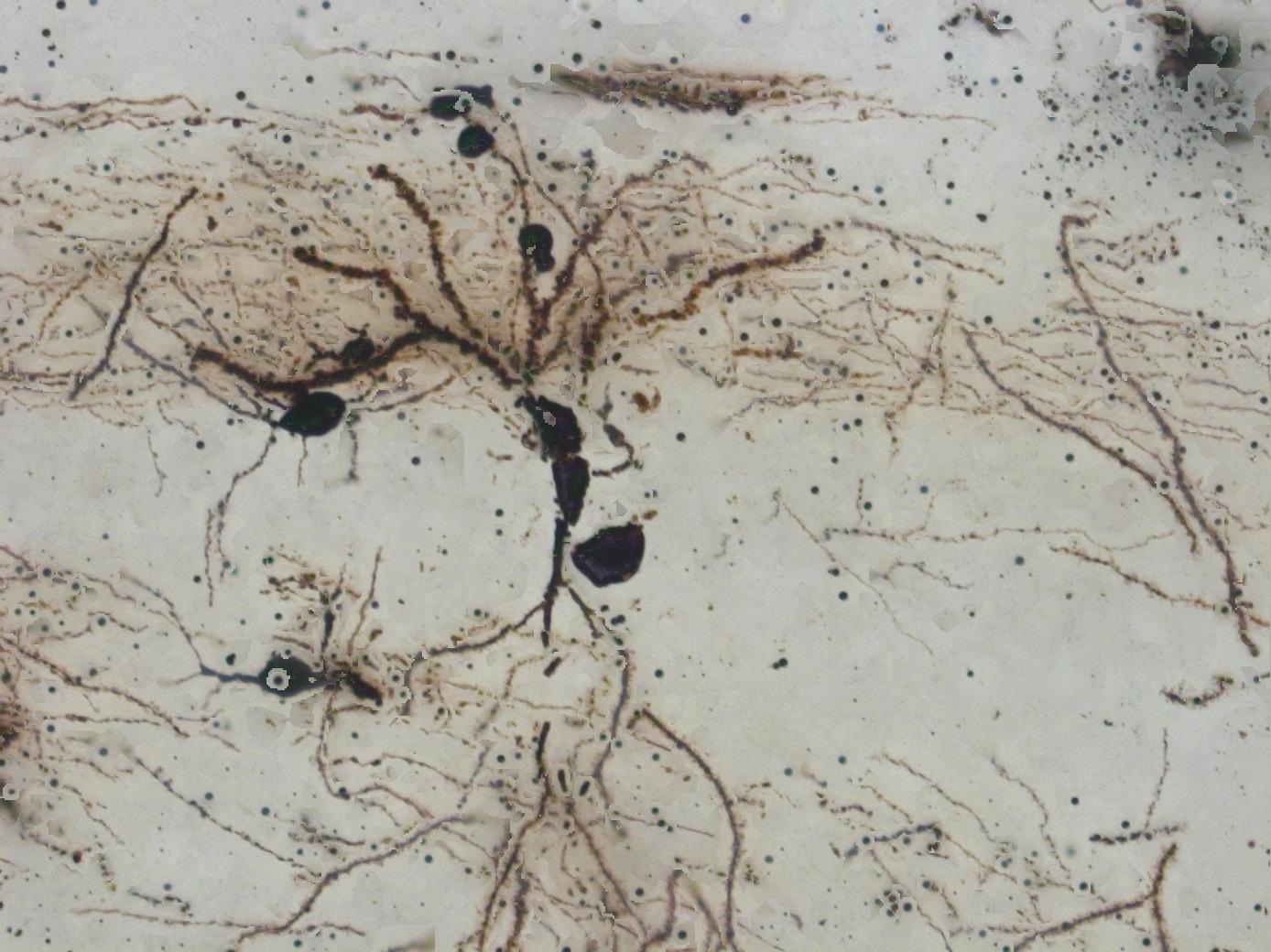

Supplement: Figure 2—figure supplement 1—source data 1. [file elife-86940-fig2-figsupp1-data1.zip › Figure 2-figure supplement 1-source data 1/2130-MUT-40X-2-6.jpg]

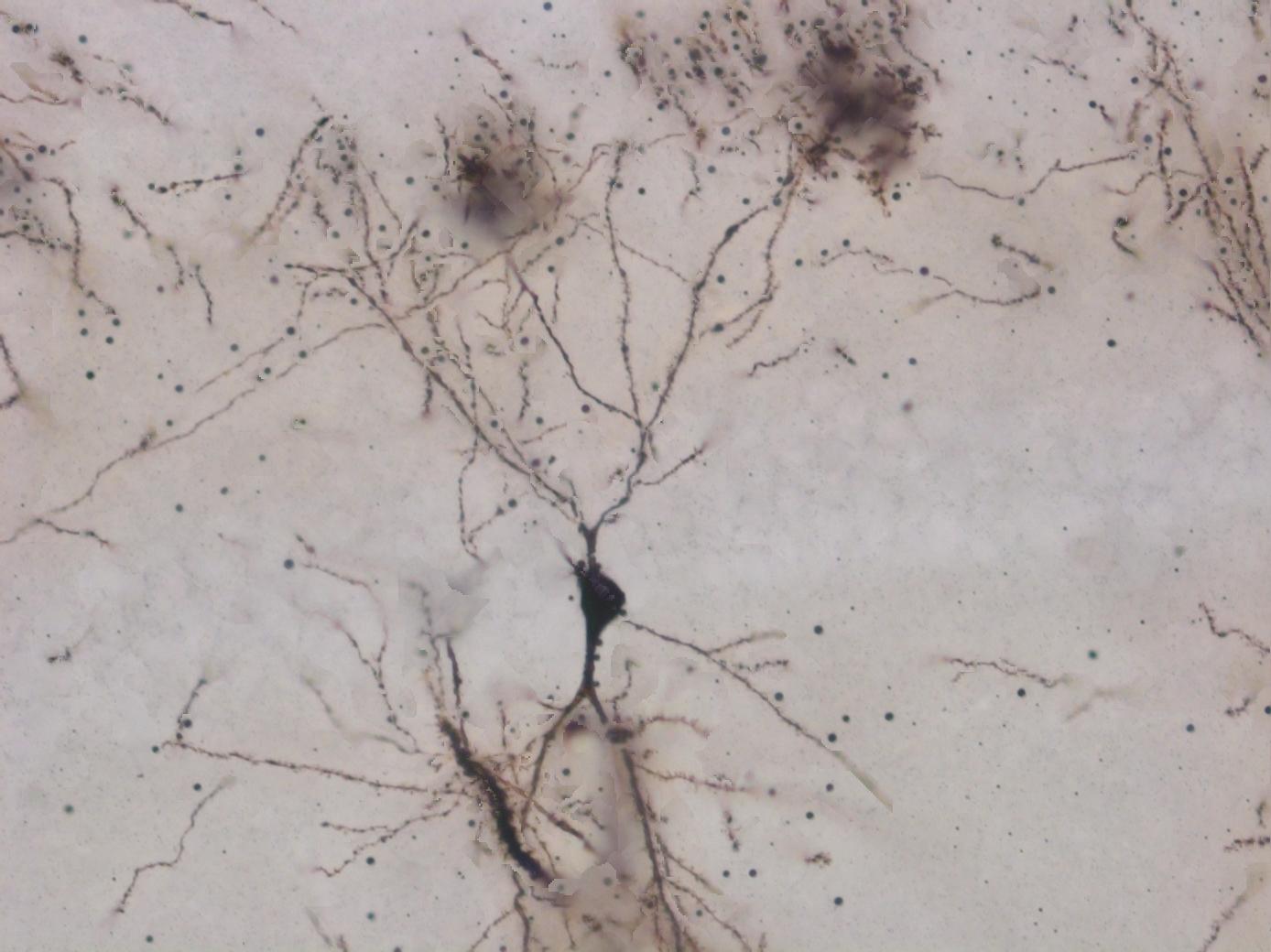

Supplement: Figure 2—figure supplement 1—source data 1. [file elife-86940-fig2-figsupp1-data1.zip › Figure 2-figure supplement 1-source data 1/2130-MUT-40X-7-2a.jpg]

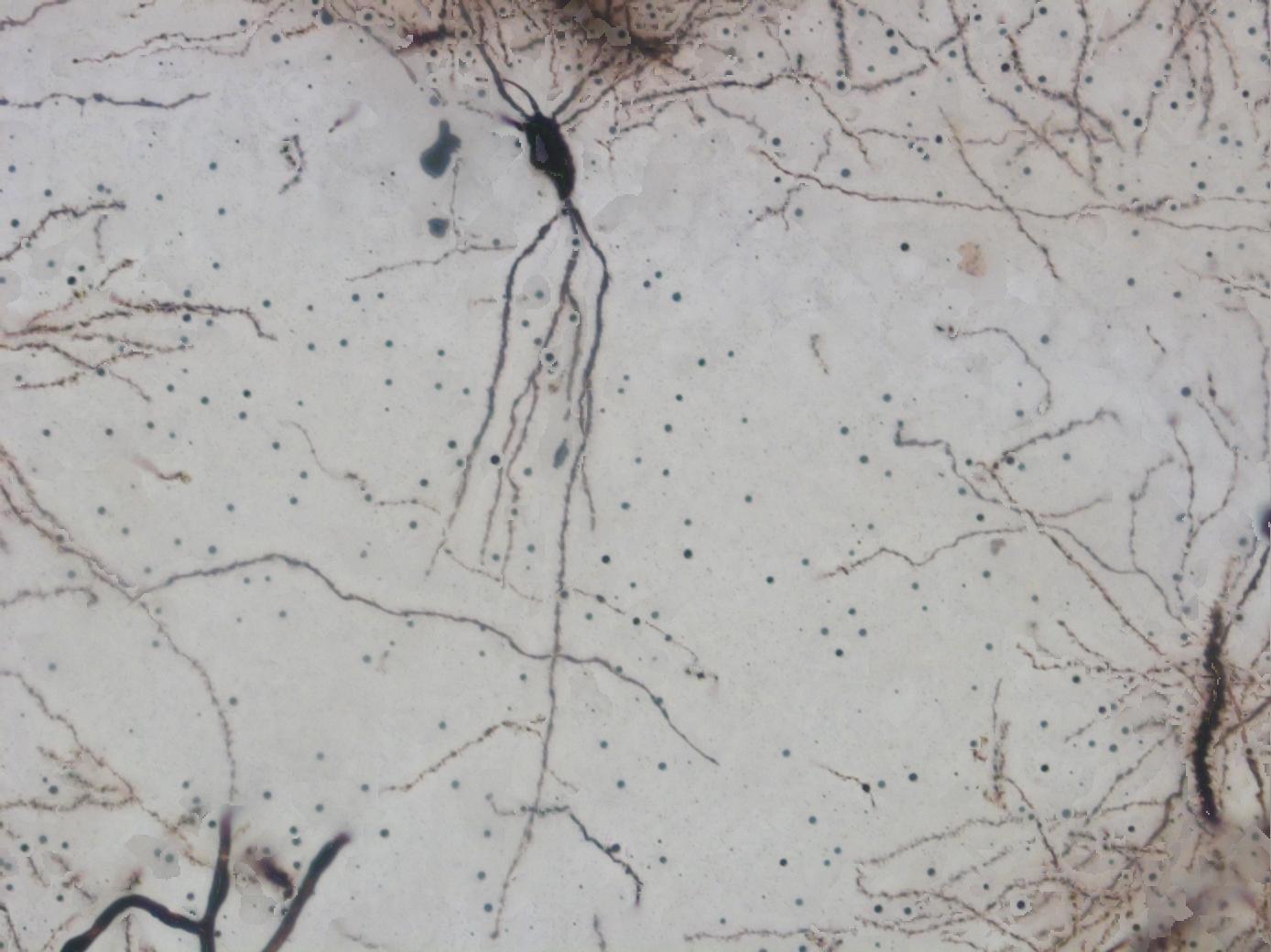

Supplement: Figure 2—figure supplement 1—source data 1. [file elife-86940-fig2-figsupp1-data1.zip › Figure 2-figure supplement 1-source data 1/2130-MUT-40X-7-2b.jpg]

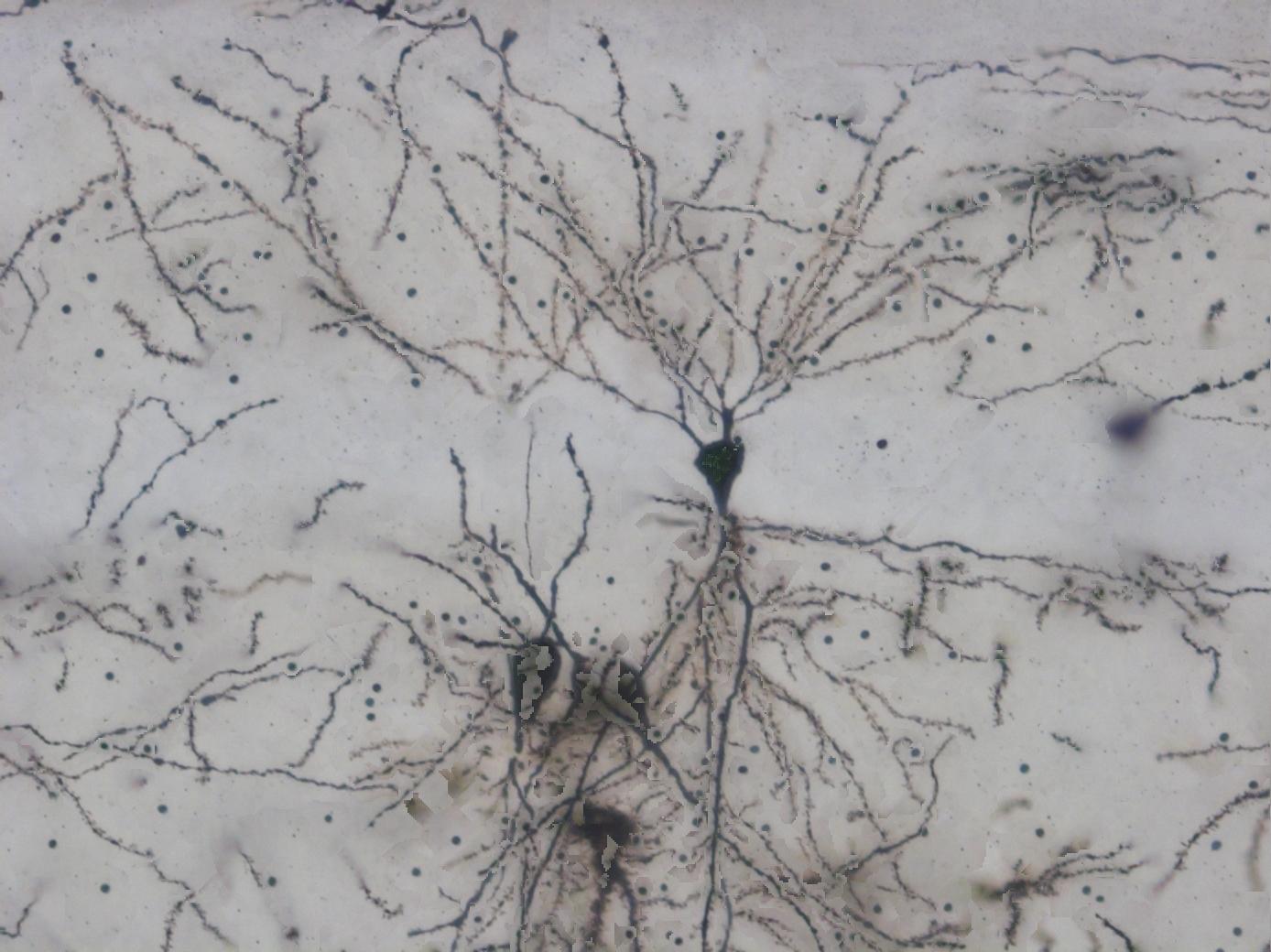

Supplement: Figure 2—figure supplement 1—source data 1. [file elife-86940-fig2-figsupp1-data1.zip › Figure 2-figure supplement 1-source data 1/2134-CON-40X-1-1.jpg]

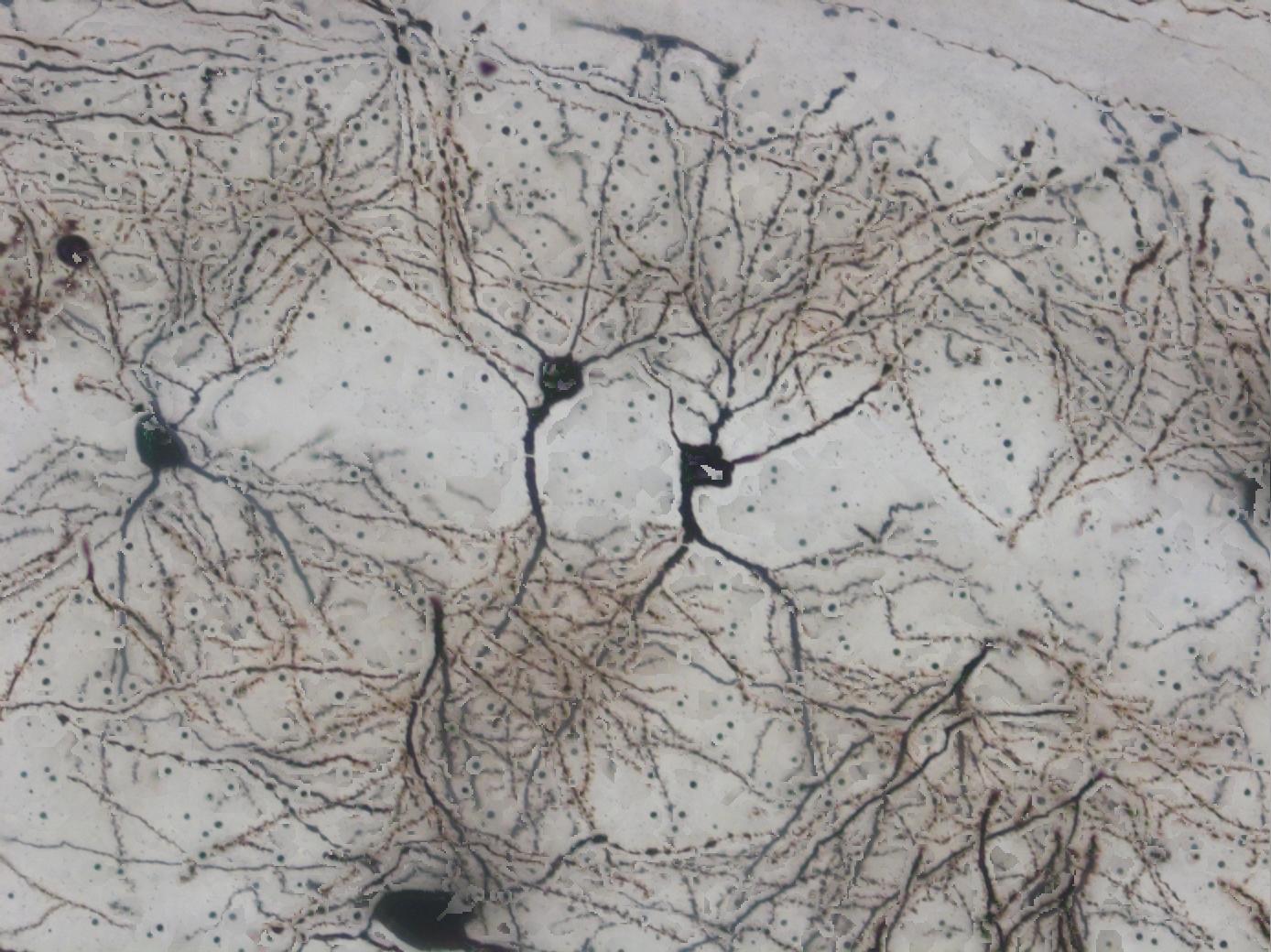

Supplement: Figure 2—figure supplement 1—source data 1. [file elife-86940-fig2-figsupp1-data1.zip › Figure 2-figure supplement 1-source data 1/2134-CON-40X-1-5a.jpg]

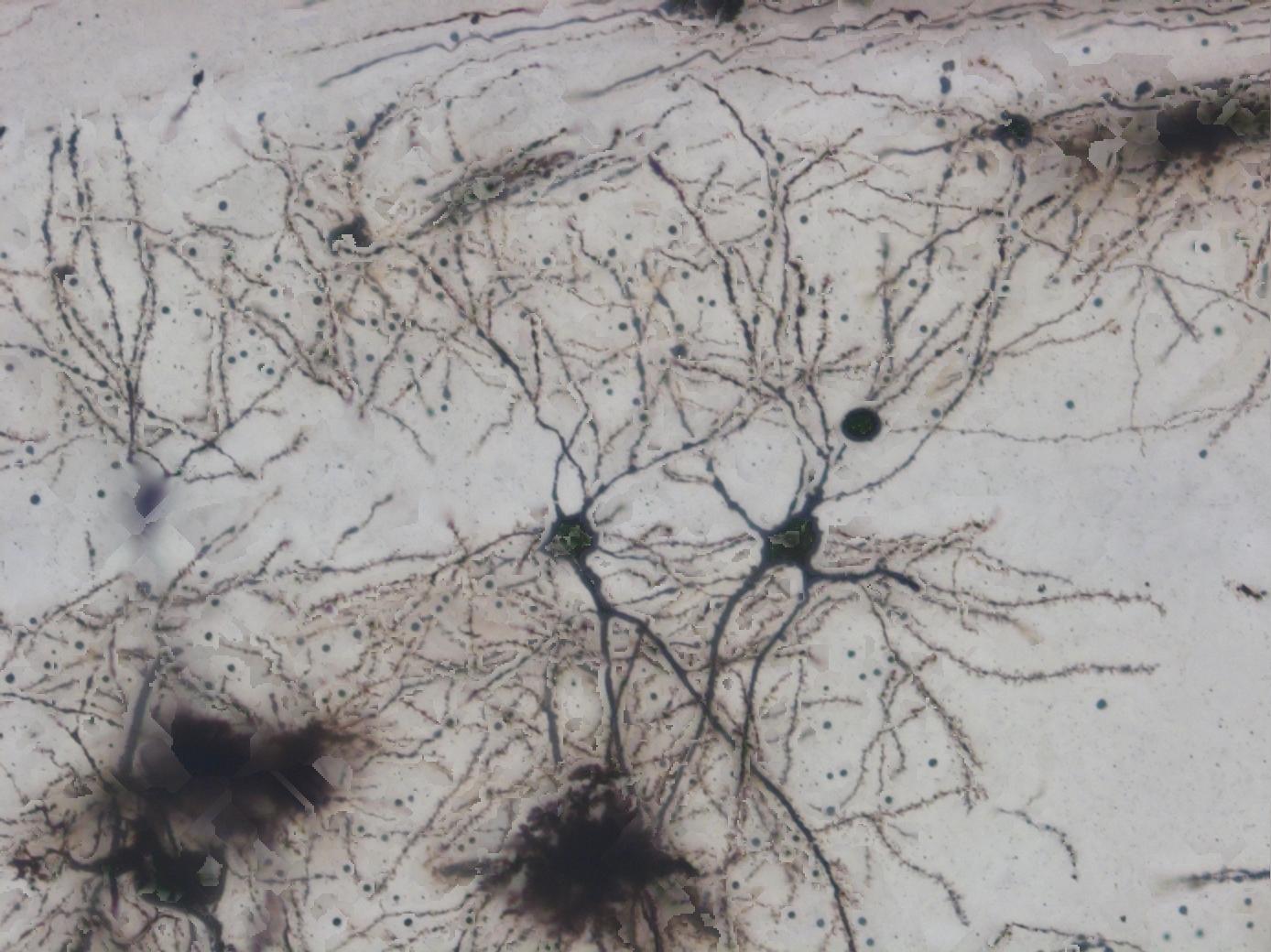

Supplement: Figure 2—figure supplement 1—source data 1. [file elife-86940-fig2-figsupp1-data1.zip › Figure 2-figure supplement 1-source data 1/2134-CON-40X-2-4.jpg]

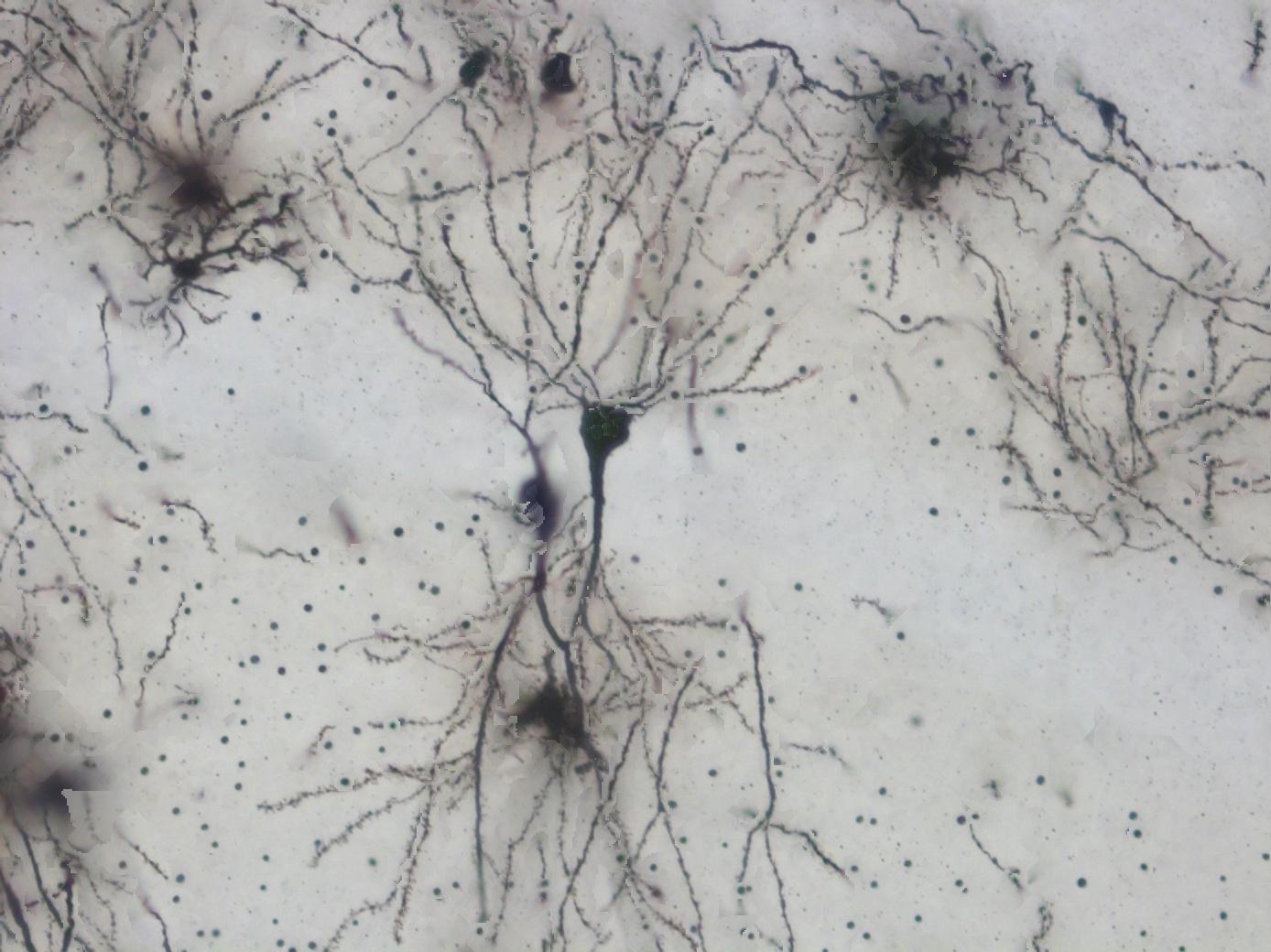

Supplement: Figure 2—figure supplement 1—source data 1. [file elife-86940-fig2-figsupp1-data1.zip › Figure 2-figure supplement 1-source data 1/2134-CON-40X-4-2a.jpg]

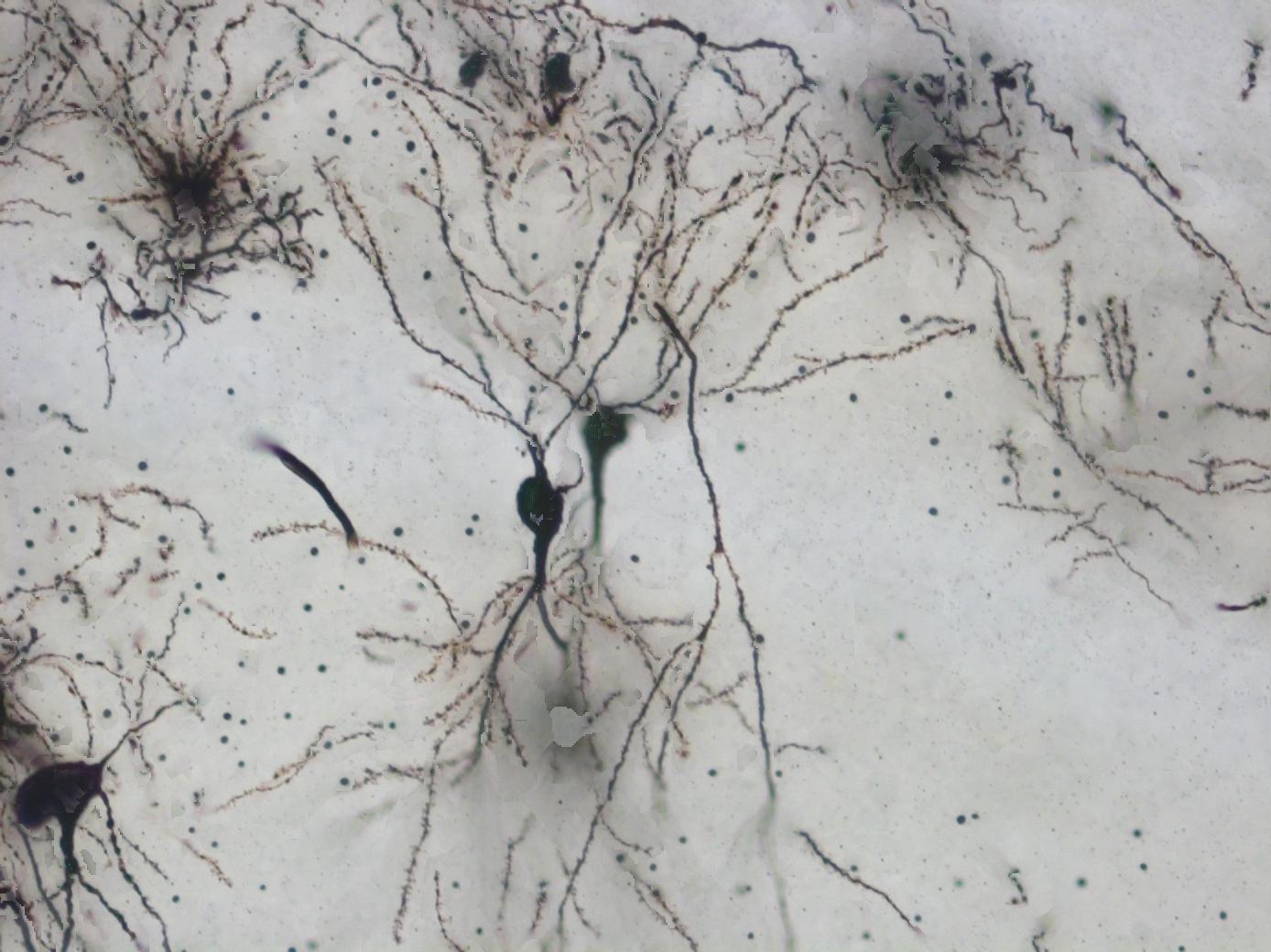

Supplement: Figure 2—figure supplement 1—source data 1. [file elife-86940-fig2-figsupp1-data1.zip › Figure 2-figure supplement 1-source data 1/2134-CON-40X-4-2b.jpg]

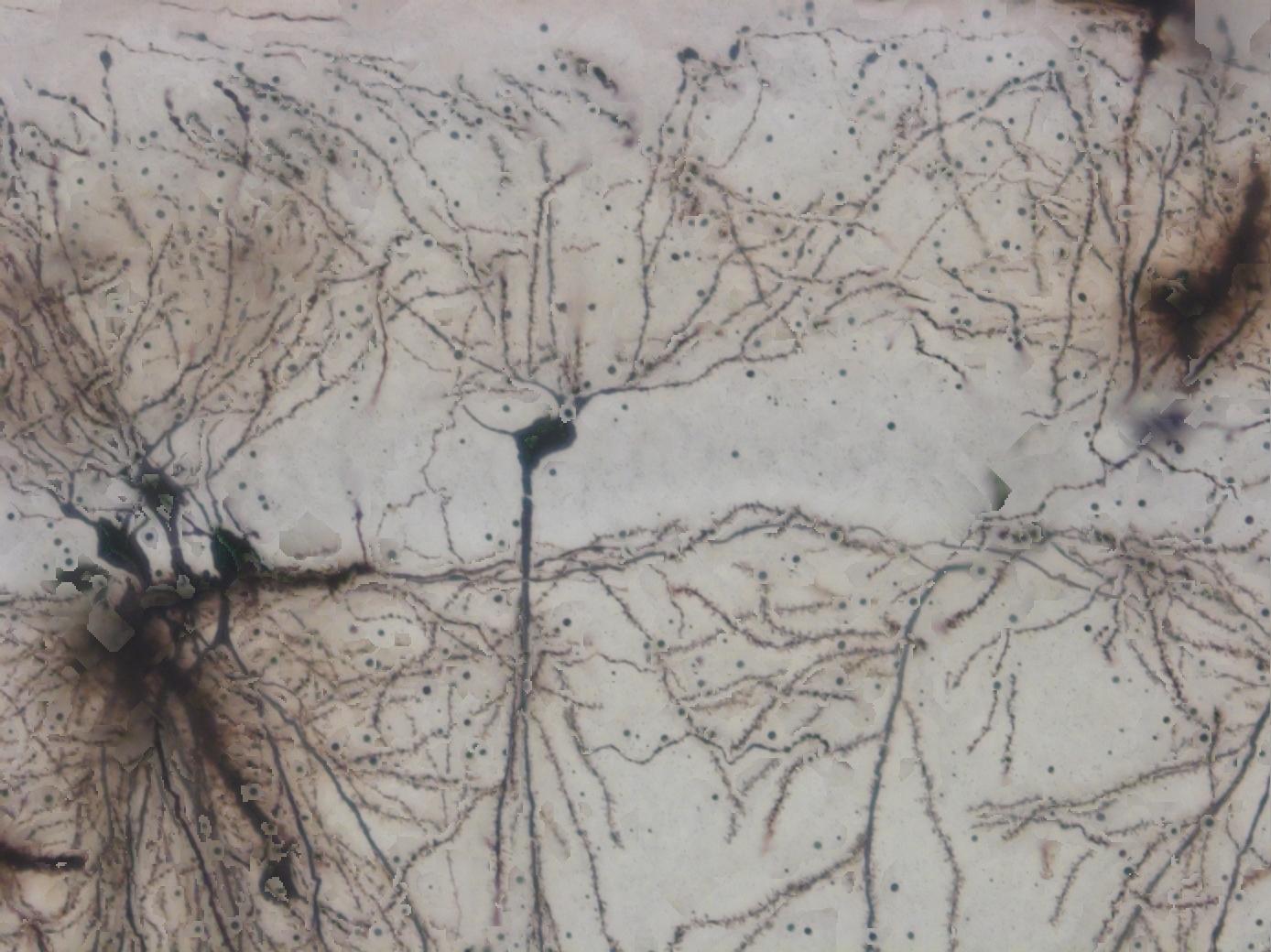

Supplement: Figure 2—figure supplement 1—source data 1. [file elife-86940-fig2-figsupp1-data1.zip › Figure 2-figure supplement 1-source data 1/2134-CON-40X-5-6.jpg]

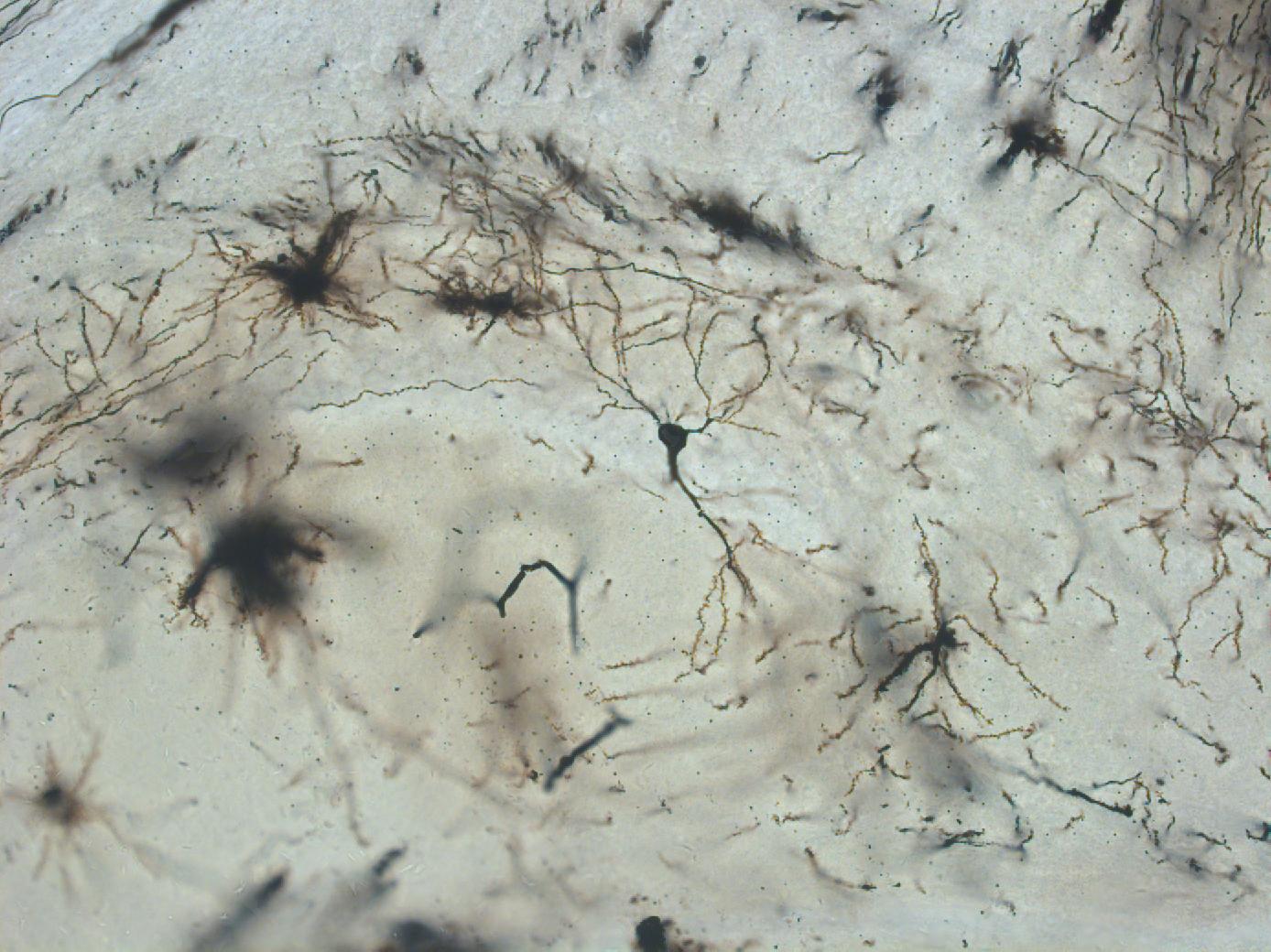

Supplement: Figure 2—figure supplement 1—source data 1. [file elife-86940-fig2-figsupp1-data1.zip › Figure 2-figure supplement 1-source data 1/2366-CON-20X-1-1.jpg]

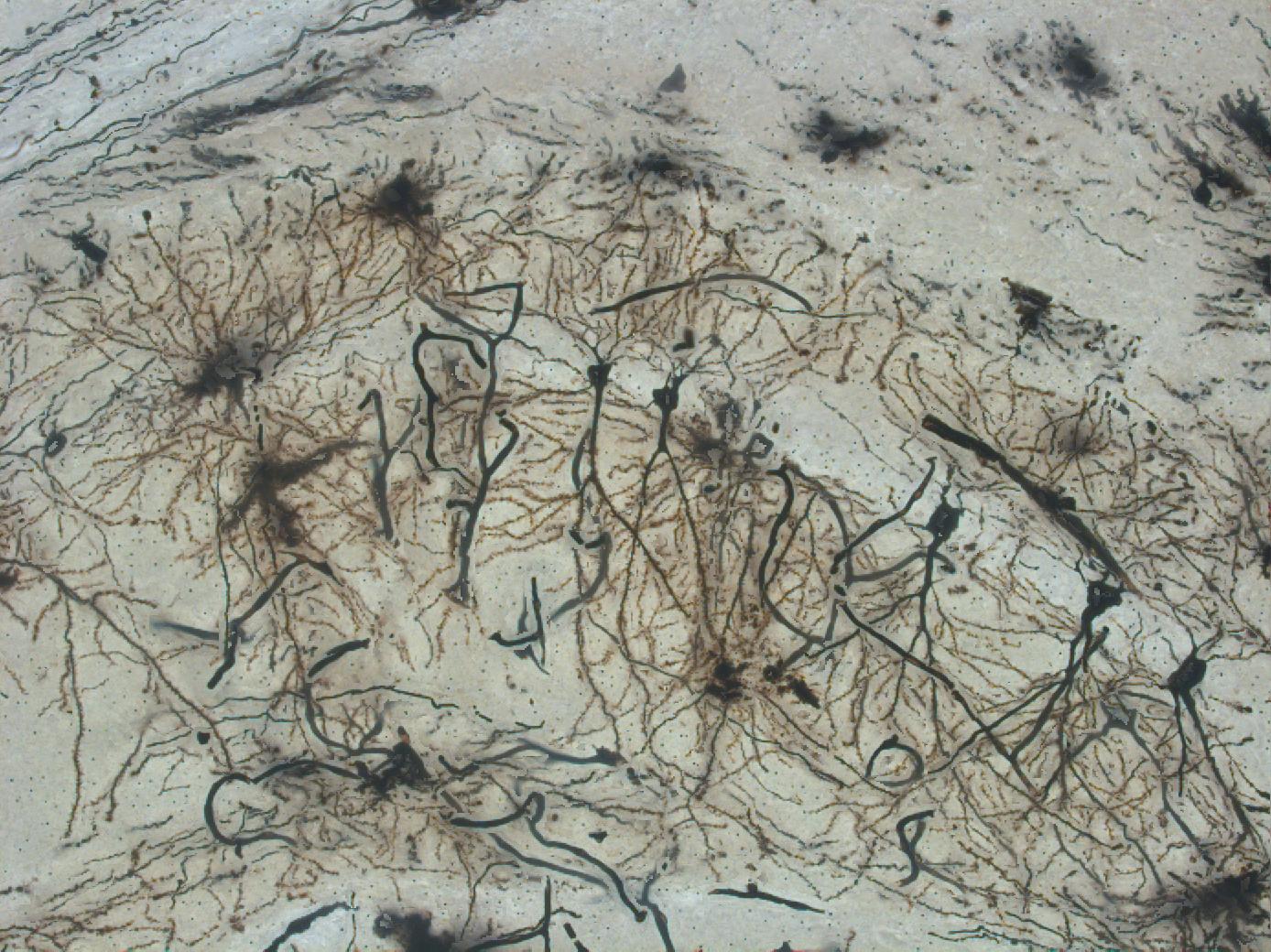

Supplement: Figure 2—figure supplement 1—source data 1. [file elife-86940-fig2-figsupp1-data1.zip › Figure 2-figure supplement 1-source data 1/2366-CON-20X-1-5.jpg]

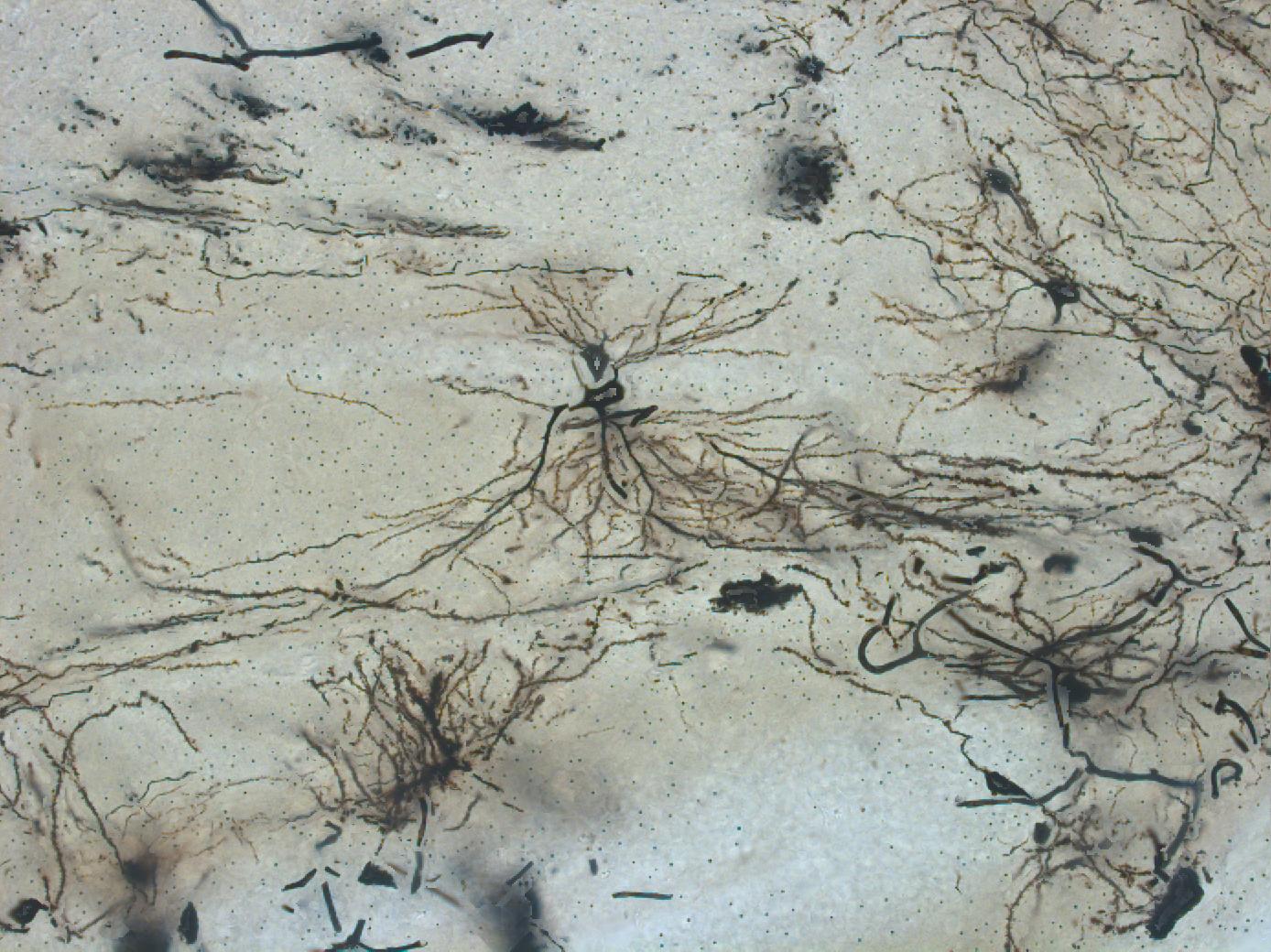

Supplement: Figure 2—figure supplement 1—source data 1. [file elife-86940-fig2-figsupp1-data1.zip › Figure 2-figure supplement 1-source data 1/2366-CON-20X-2-2.jpg]

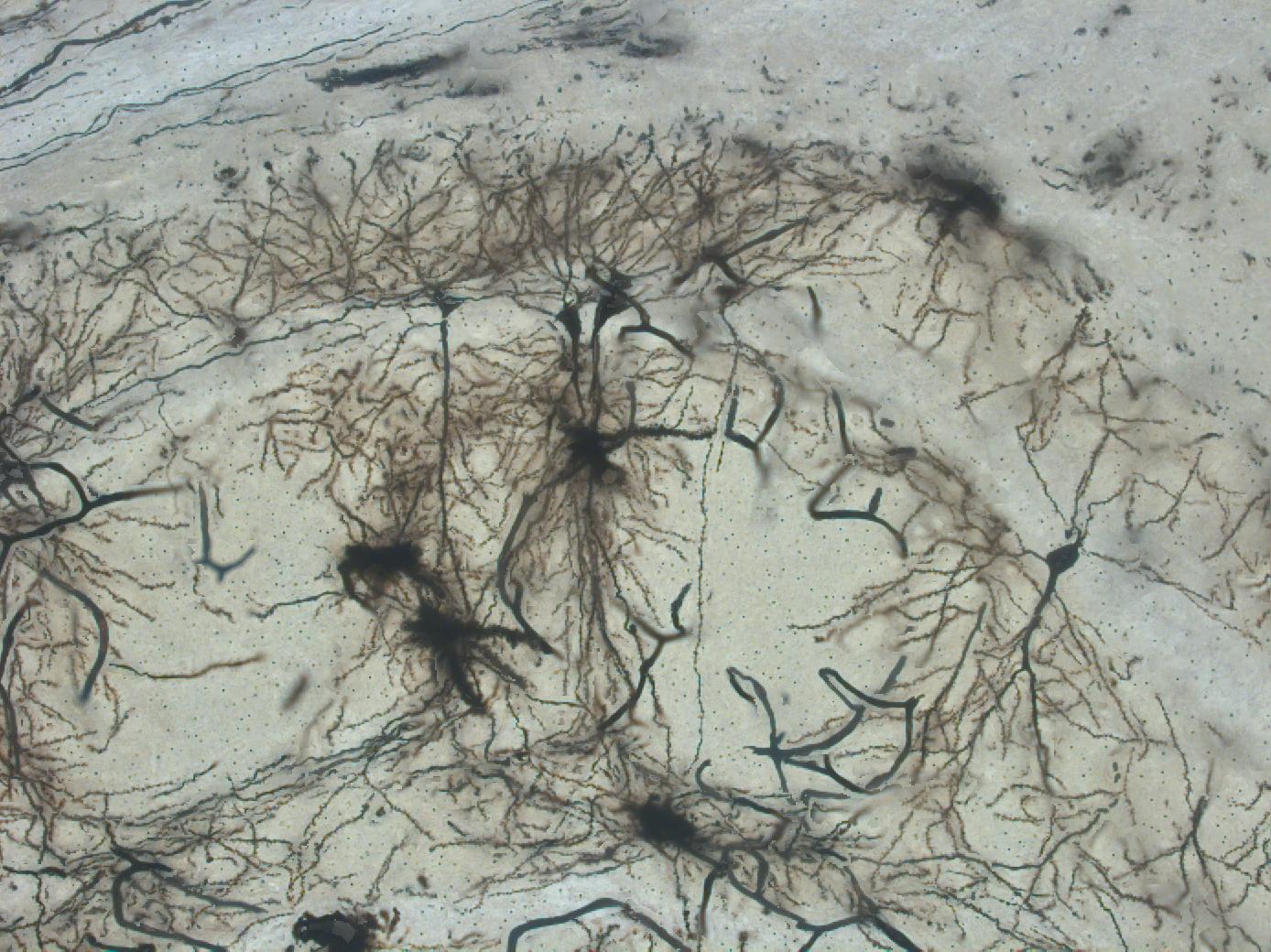

Supplement: Figure 2—figure supplement 1—source data 1. [file elife-86940-fig2-figsupp1-data1.zip › Figure 2-figure supplement 1-source data 1/2366-CON-20X-2-5-02.jpg]

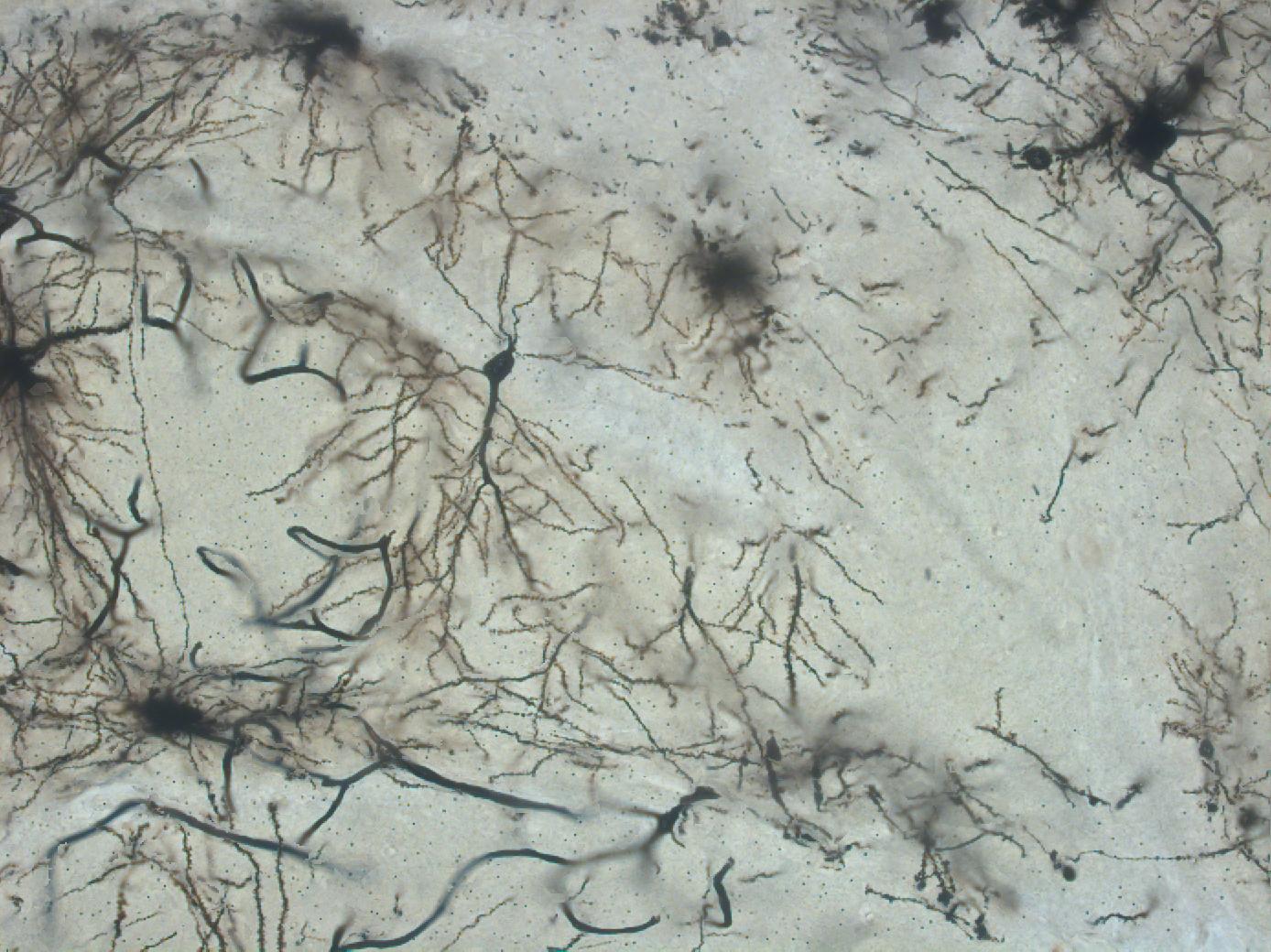

Supplement: Figure 2—figure supplement 1—source data 1. [file elife-86940-fig2-figsupp1-data1.zip › Figure 2-figure supplement 1-source data 1/2366-CON-20X-2-5.jpg]

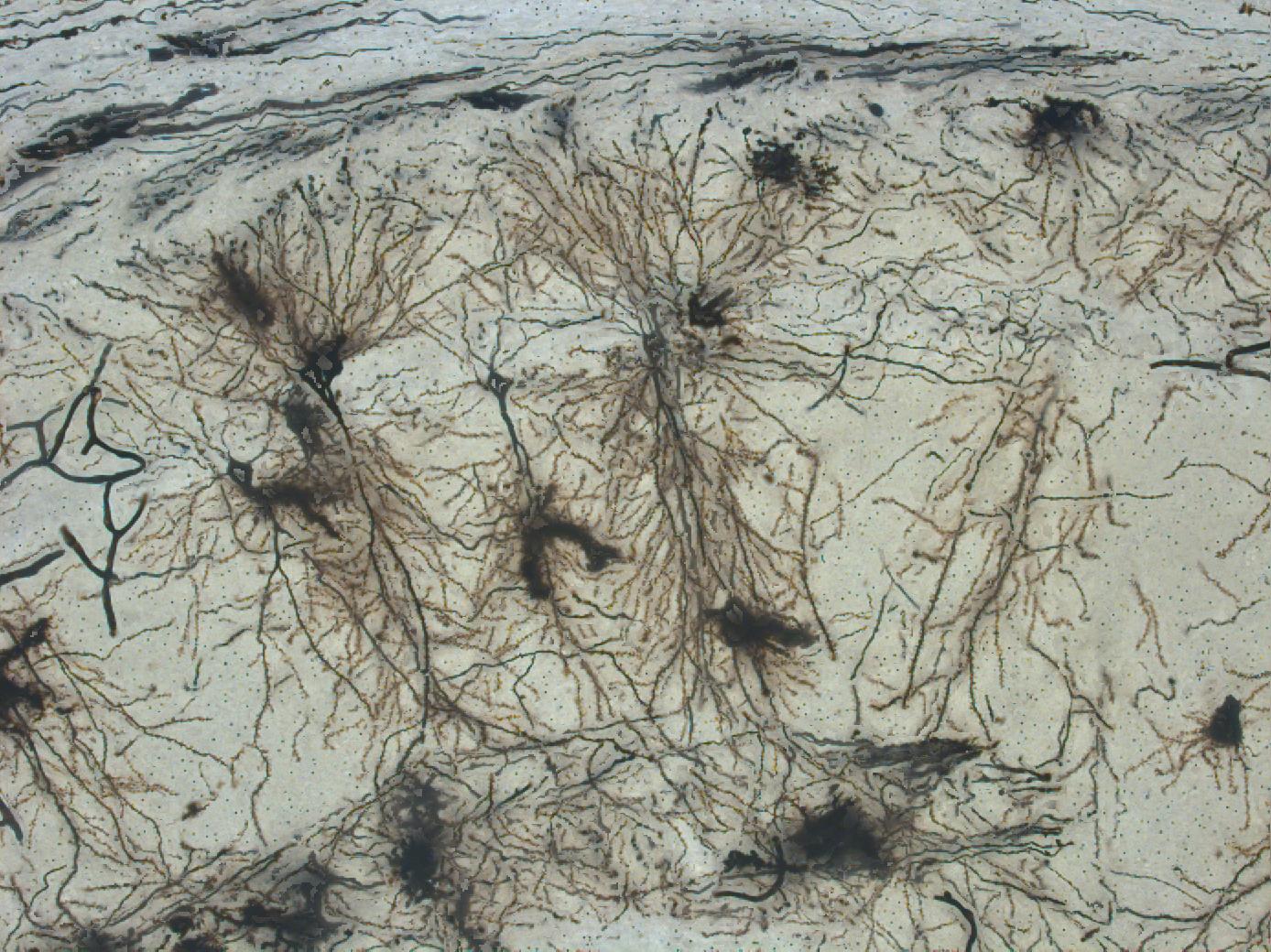

Supplement: Figure 2—figure supplement 1—source data 1. [file elife-86940-fig2-figsupp1-data1.zip › Figure 2-figure supplement 1-source data 1/2366-CON-20X-3-1-02.jpg]

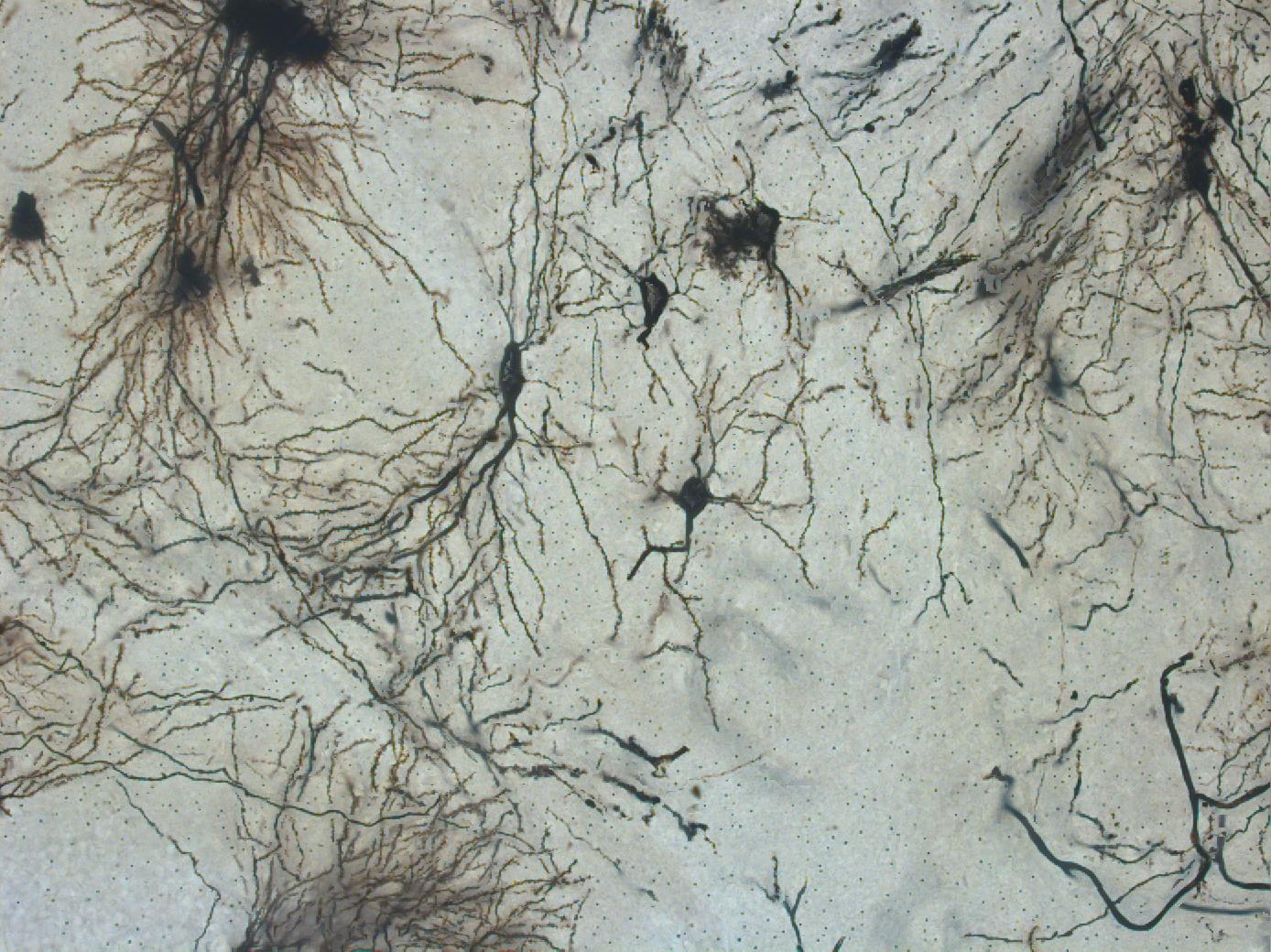

Supplement: Figure 2—figure supplement 1—source data 1. [file elife-86940-fig2-figsupp1-data1.zip › Figure 2-figure supplement 1-source data 1/2366-CON-20X-3-1.jpg]

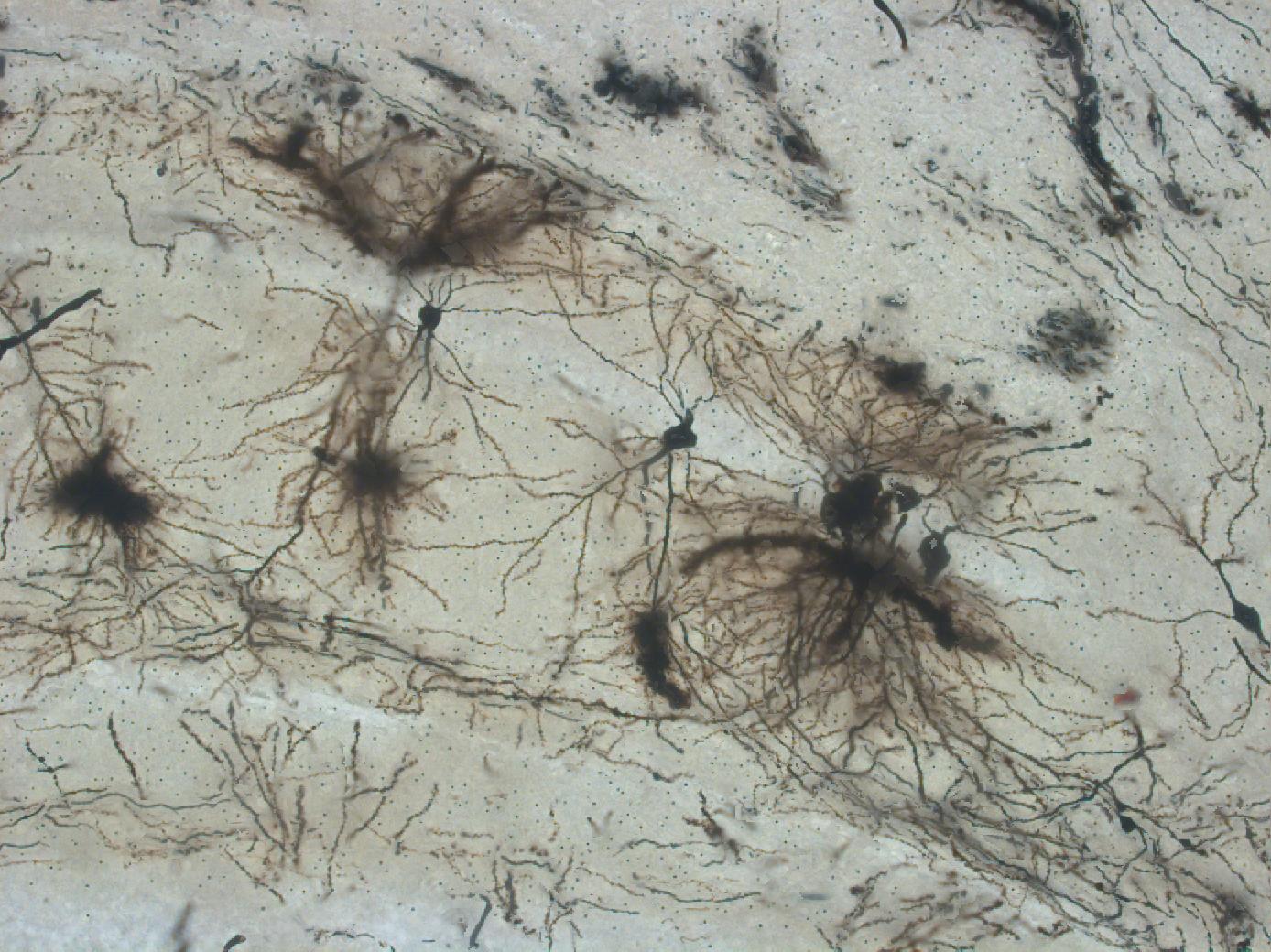

Supplement: Figure 2—figure supplement 1—source data 1. [file elife-86940-fig2-figsupp1-data1.zip › Figure 2-figure supplement 1-source data 1/2366-CON-20X-3-4.jpg]

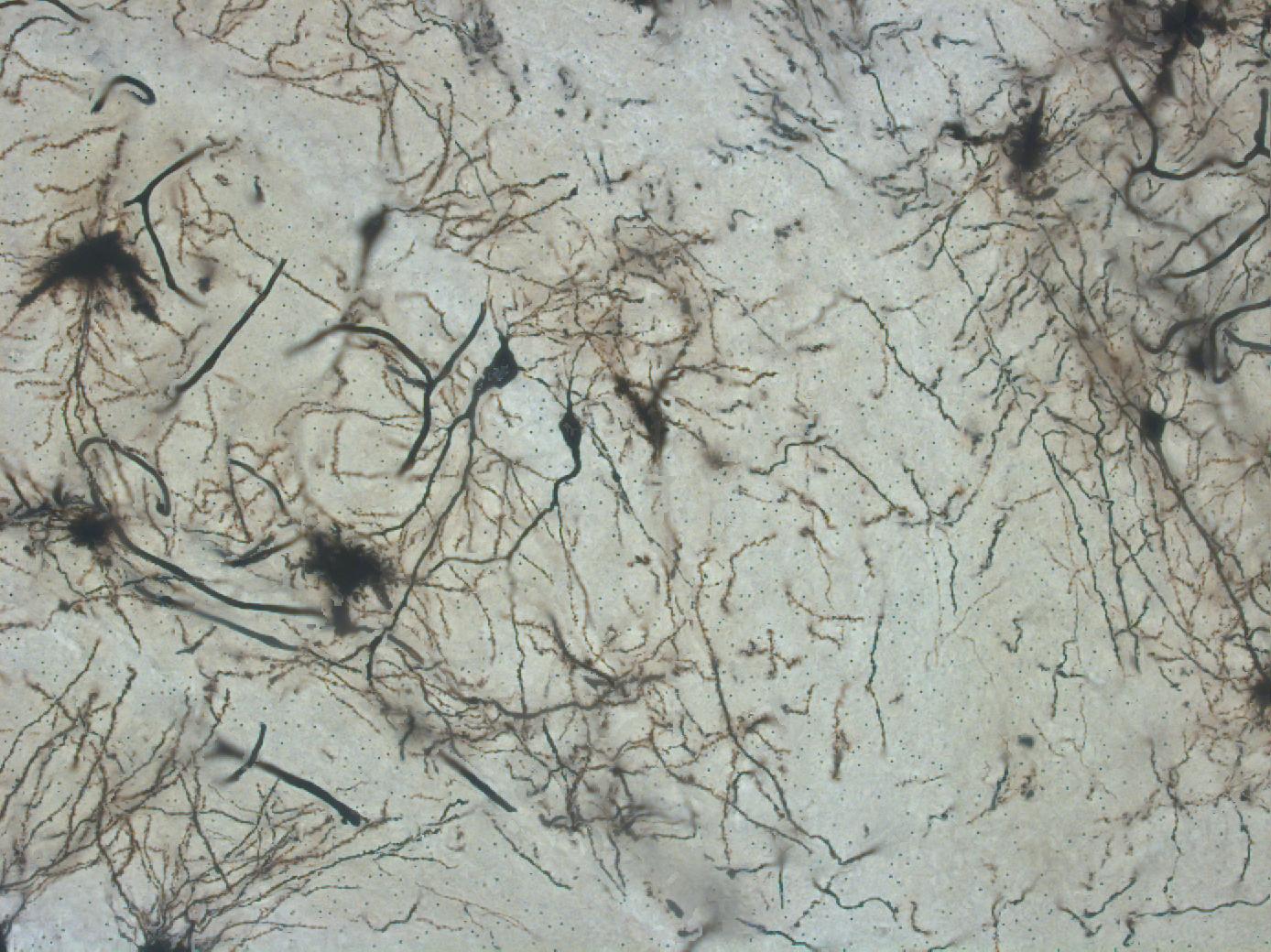

Supplement: Figure 2—figure supplement 1—source data 1. [file elife-86940-fig2-figsupp1-data1.zip › Figure 2-figure supplement 1-source data 1/2366-CON-20X-3-5-02.jpg]
