# Supplementary figures and images for: The differentiation and integration of the hippocampal dorsoventral axis are controlled by two nuclear receptor genes (part 3 of 6)
Source: eLife. 2023 Sep 26;12:RP86940. doi: 10.7554/eLife.86940 (PMC10522401; doi:10.7554/eLife.86940)

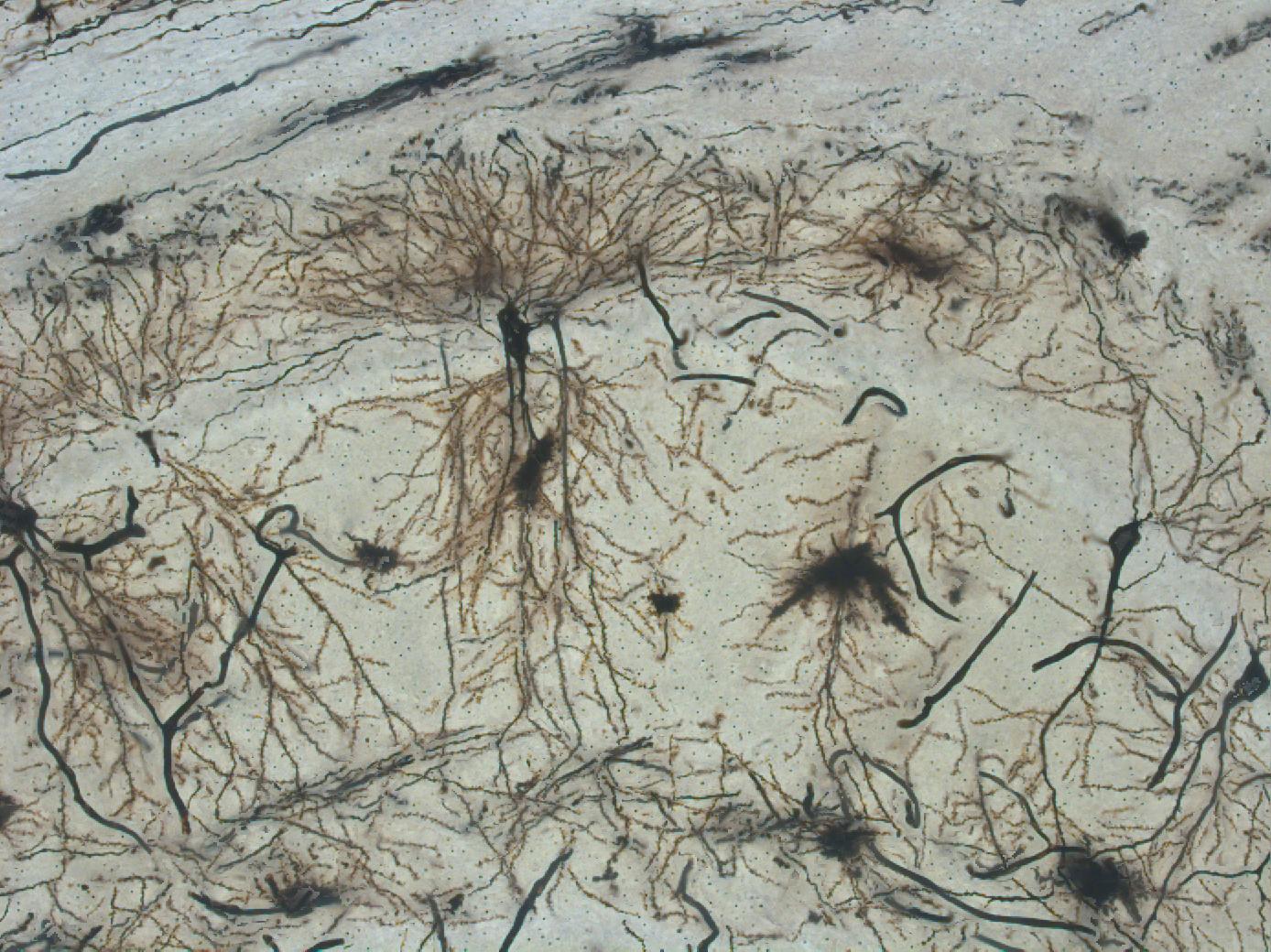

Supplement: Figure 2—figure supplement 1—source data 1. [file elife-86940-fig2-figsupp1-data1.zip › Figure 2-figure supplement 1-source data 1/2366-CON-20X-3-5-03.jpg]

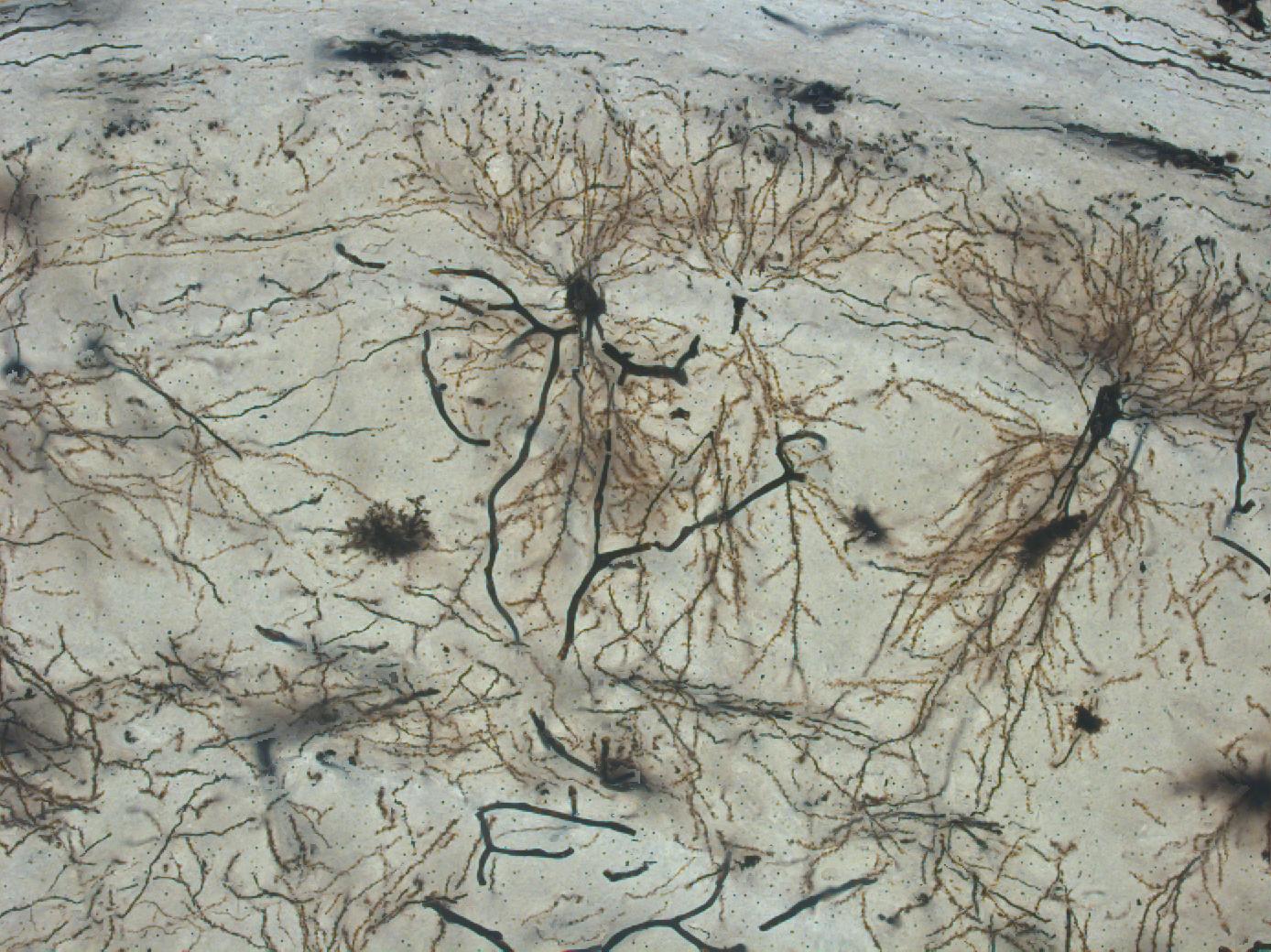

Supplement: Figure 2—figure supplement 1—source data 1. [file elife-86940-fig2-figsupp1-data1.zip › Figure 2-figure supplement 1-source data 1/2366-CON-20X-3-5-04.jpg]

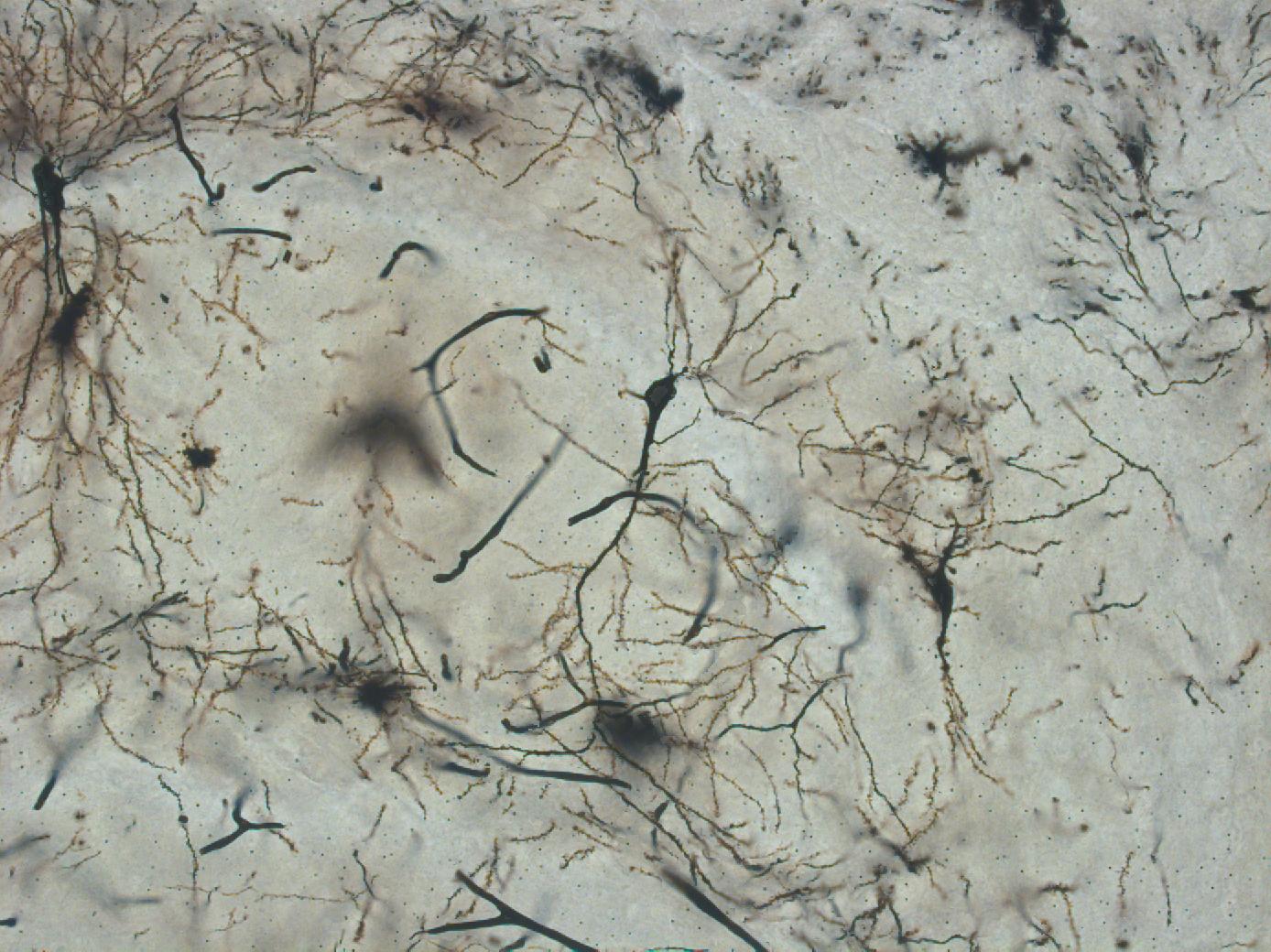

Supplement: Figure 2—figure supplement 1—source data 1. [file elife-86940-fig2-figsupp1-data1.zip › Figure 2-figure supplement 1-source data 1/2366-CON-20X-3-5.jpg]

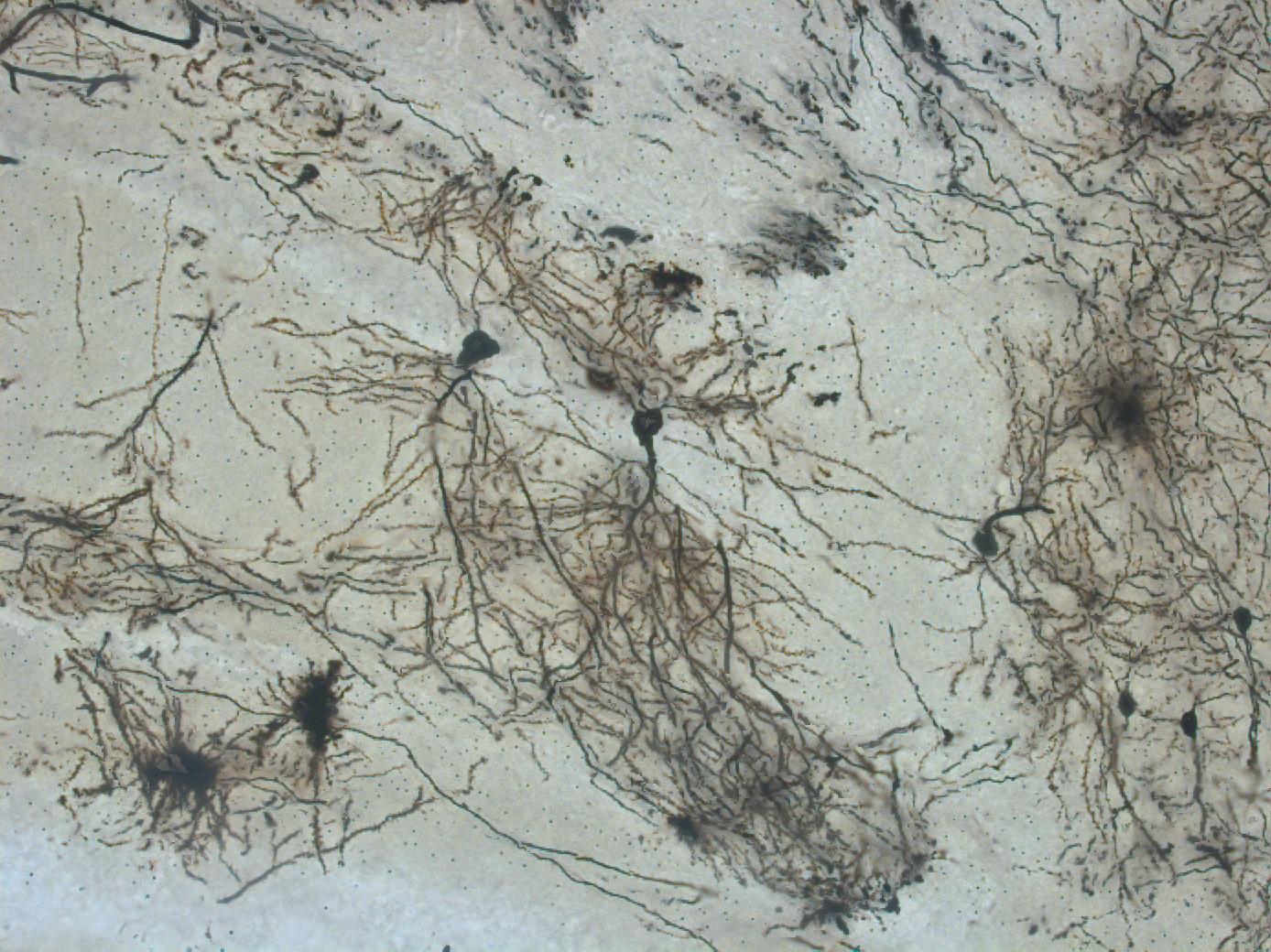

Supplement: Figure 2—figure supplement 1—source data 1. [file elife-86940-fig2-figsupp1-data1.zip › Figure 2-figure supplement 1-source data 1/2366-CON-20X-4-3.jpg]

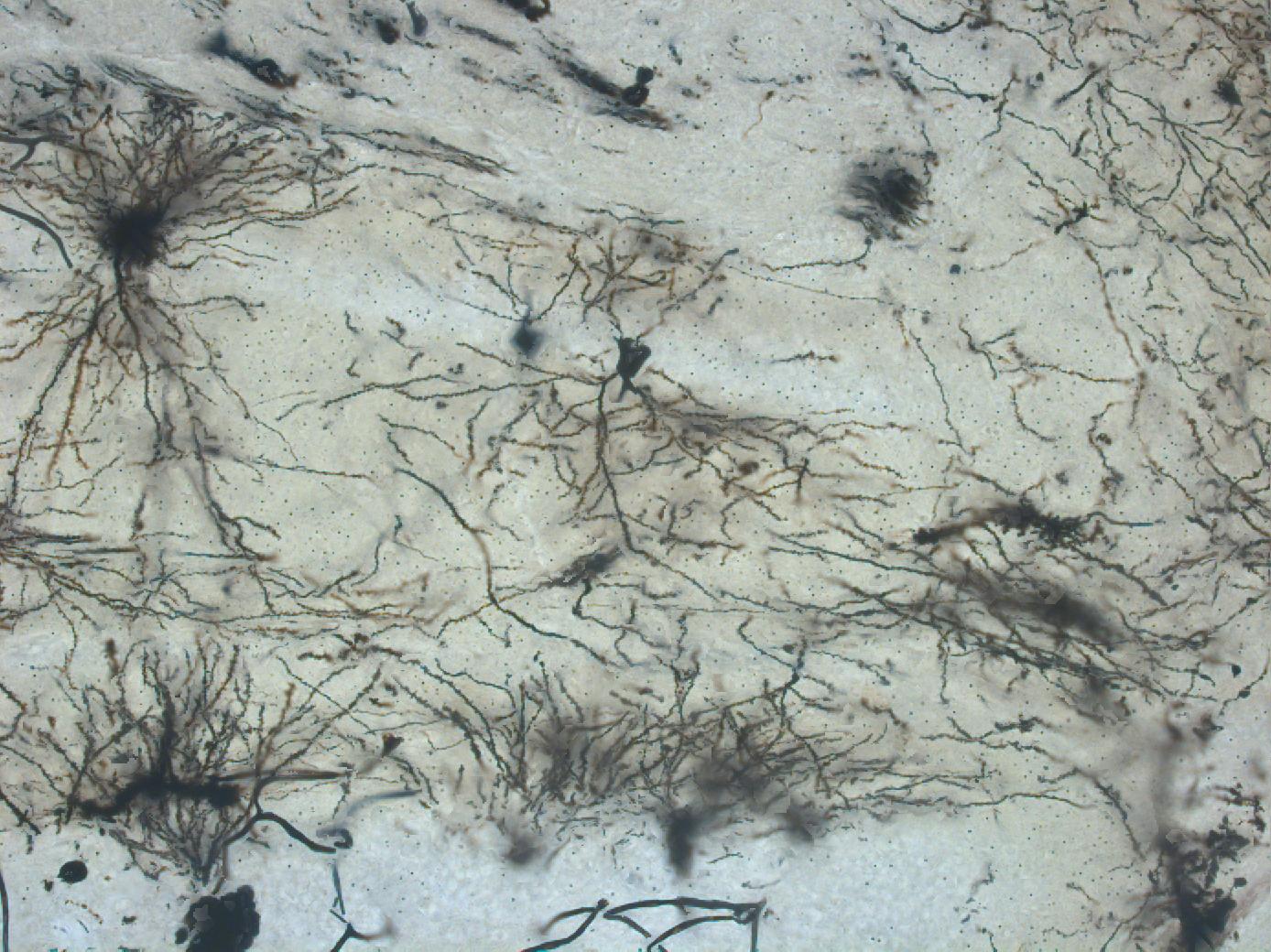

Supplement: Figure 2—figure supplement 1—source data 1. [file elife-86940-fig2-figsupp1-data1.zip › Figure 2-figure supplement 1-source data 1/2366-CON-20X-4-5-02.jpg]

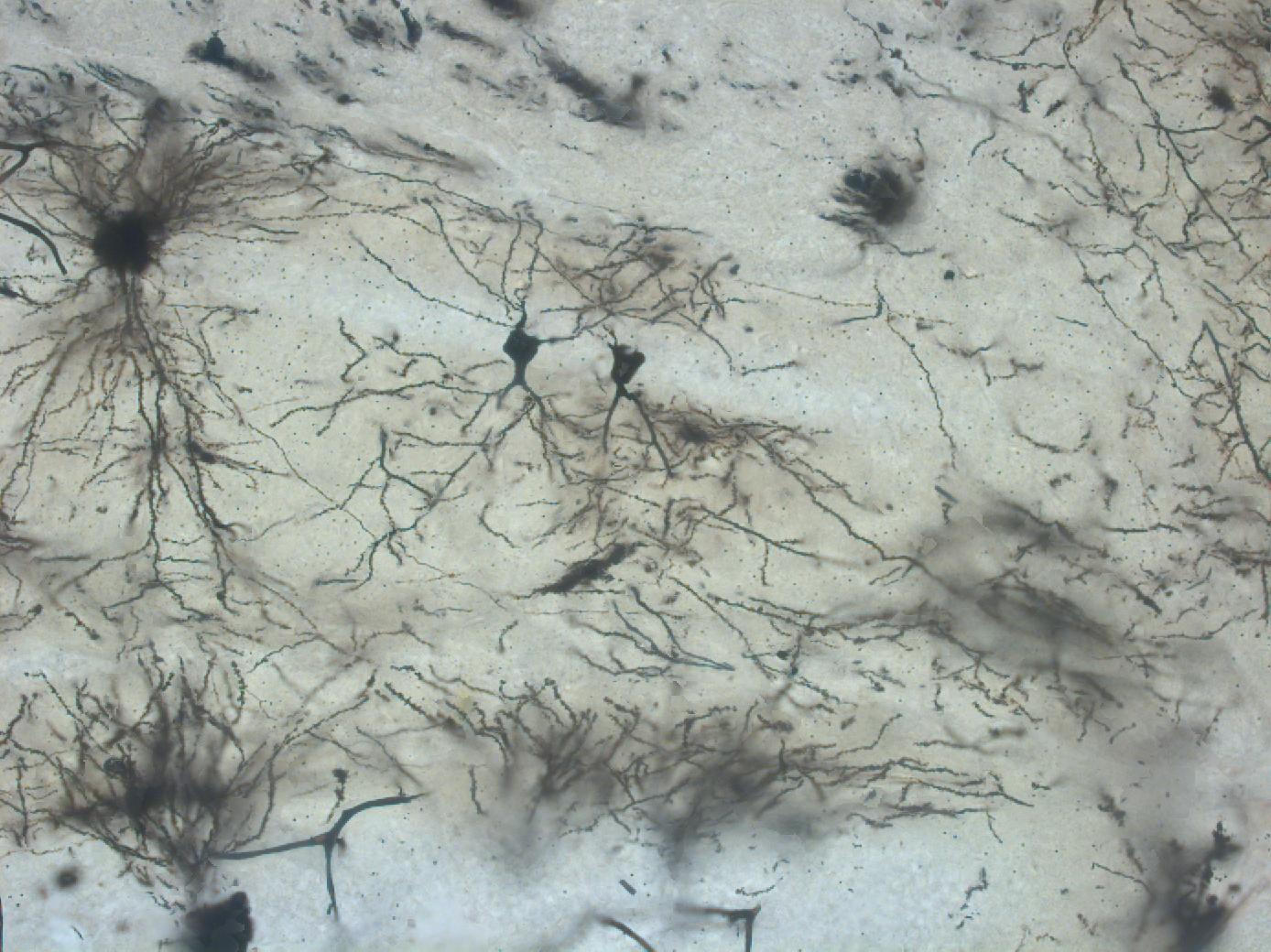

Supplement: Figure 2—figure supplement 1—source data 1. [file elife-86940-fig2-figsupp1-data1.zip › Figure 2-figure supplement 1-source data 1/2366-CON-20X-4-5.jpg]

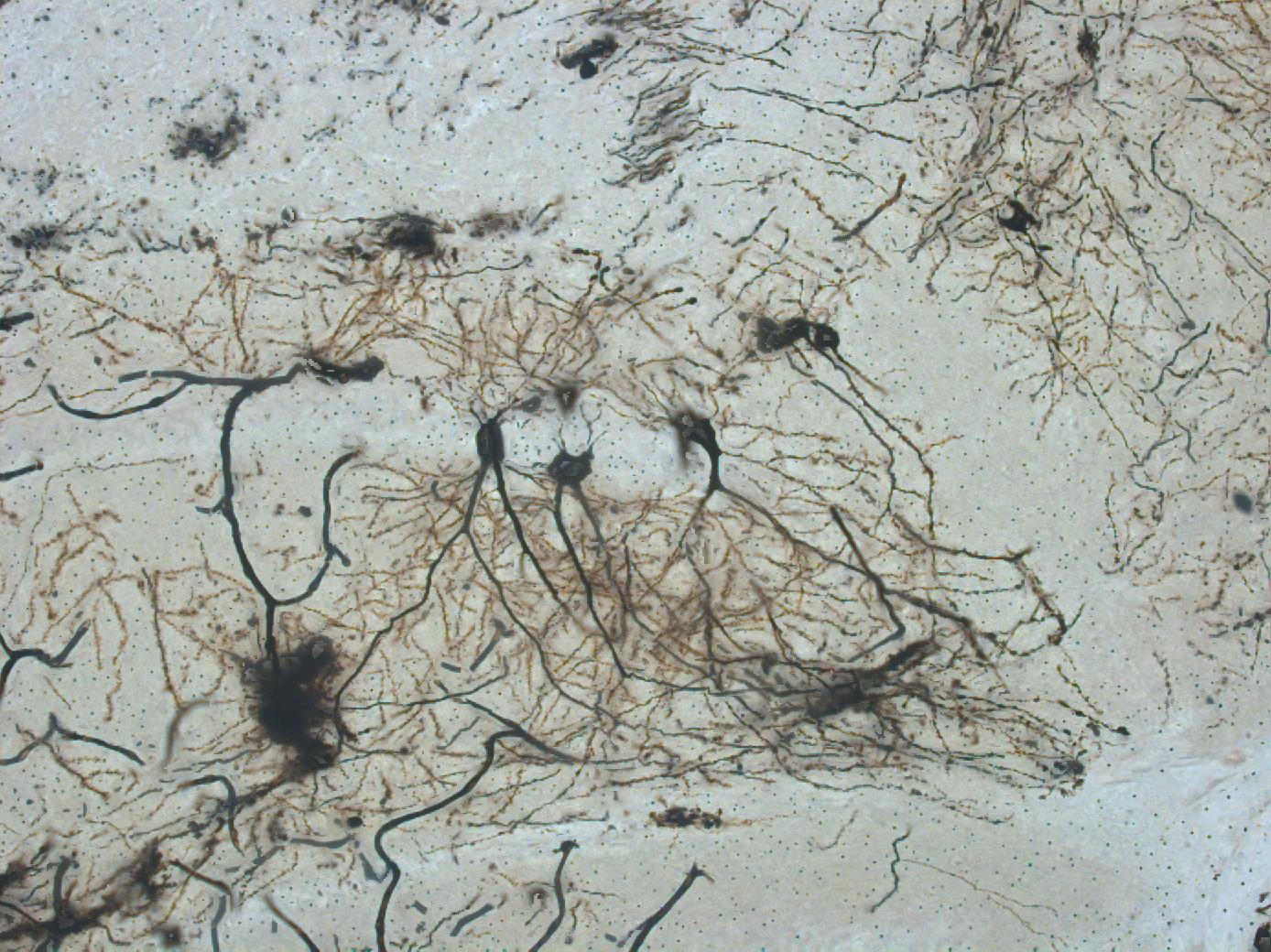

Supplement: Figure 2—figure supplement 1—source data 1. [file elife-86940-fig2-figsupp1-data1.zip › Figure 2-figure supplement 1-source data 1/2366-CON-20X-5-4.jpg]

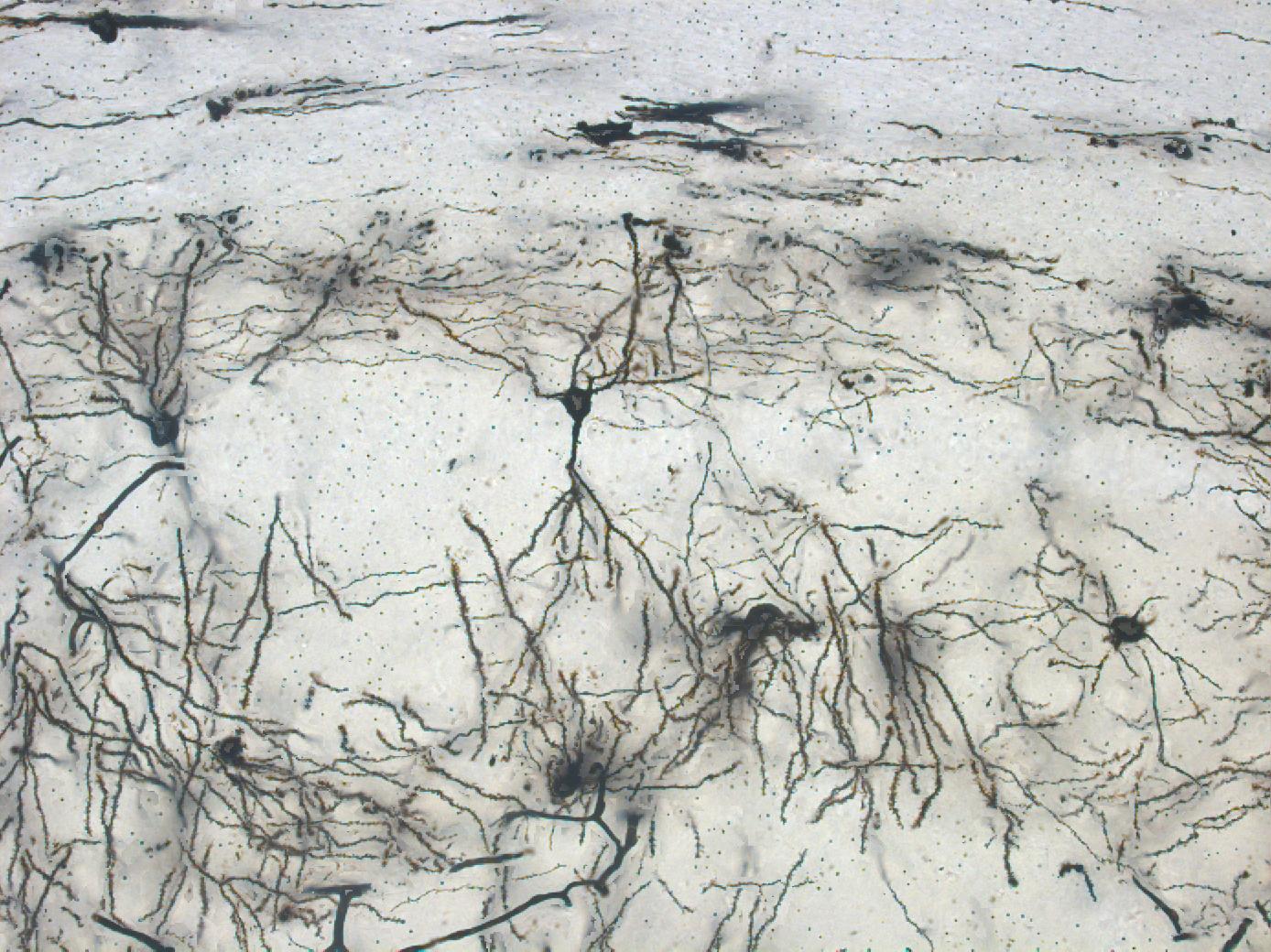

Supplement: Figure 2—figure supplement 1—source data 1. [file elife-86940-fig2-figsupp1-data1.zip › Figure 2-figure supplement 1-source data 1/2369-MUT-20X-1-1-02.jpg]

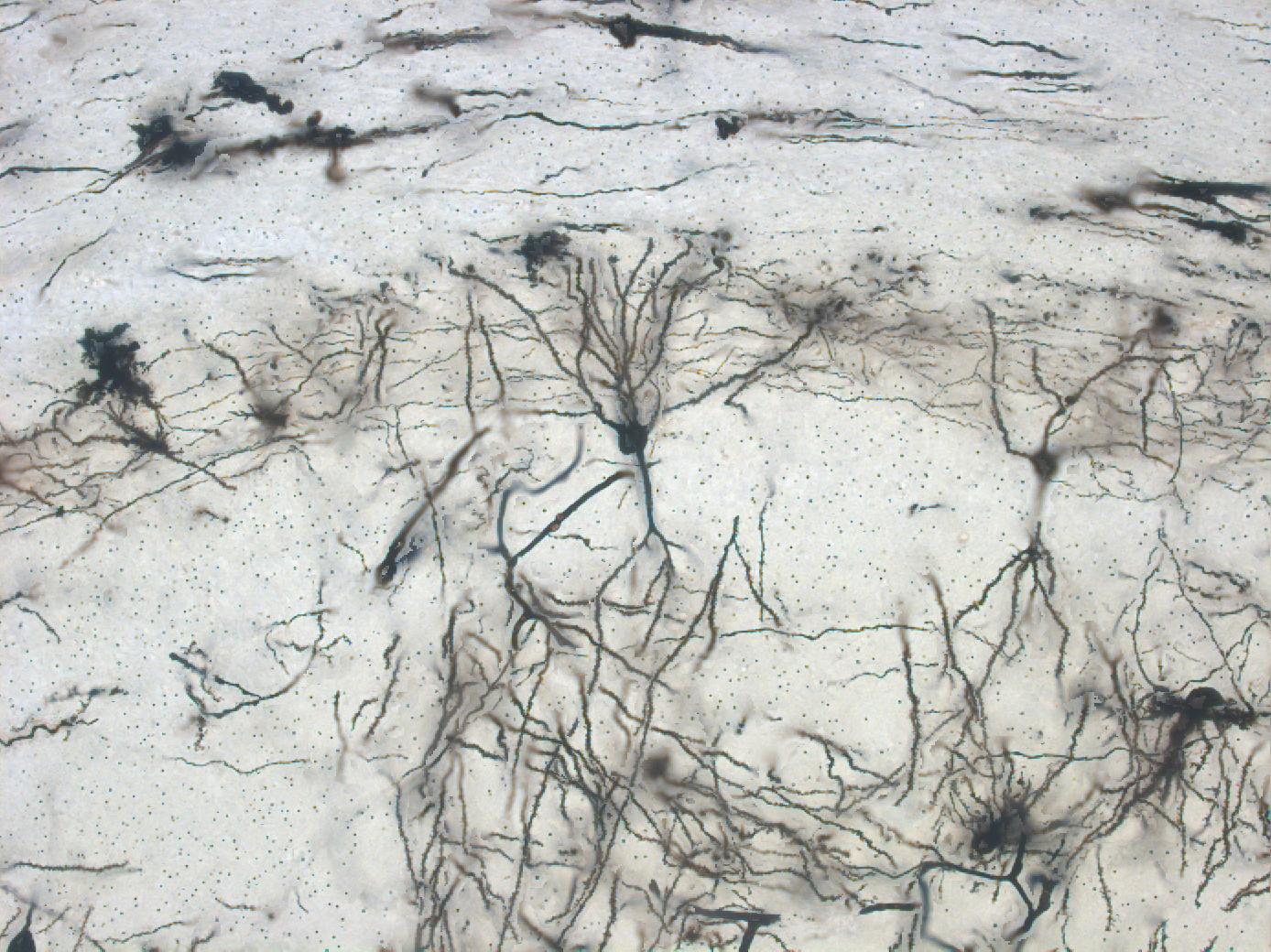

Supplement: Figure 2—figure supplement 1—source data 1. [file elife-86940-fig2-figsupp1-data1.zip › Figure 2-figure supplement 1-source data 1/2369-MUT-20X-1-1-03.jpg]

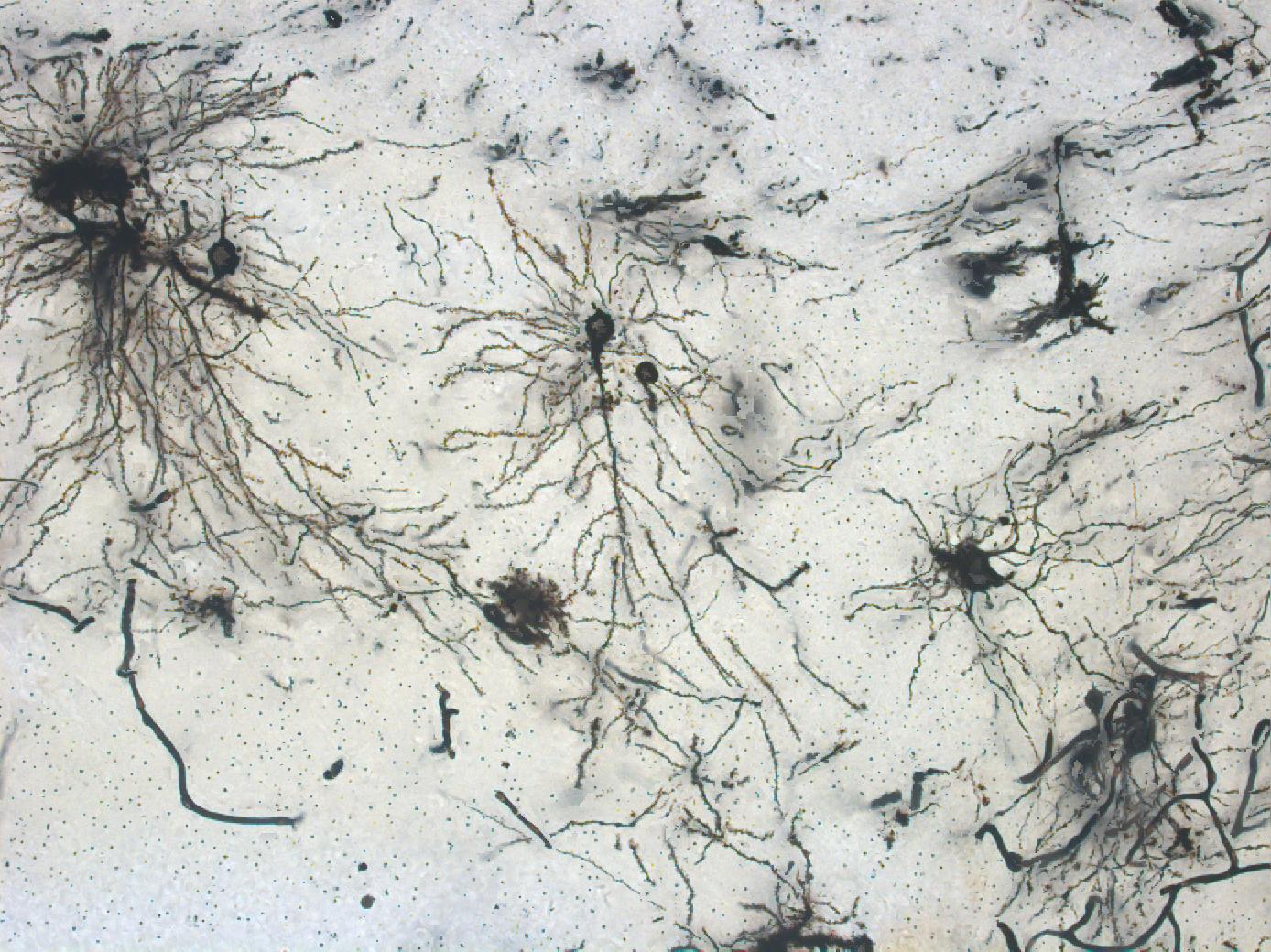

Supplement: Figure 2—figure supplement 1—source data 1. [file elife-86940-fig2-figsupp1-data1.zip › Figure 2-figure supplement 1-source data 1/2369-MUT-20X-1-1-04.jpg]

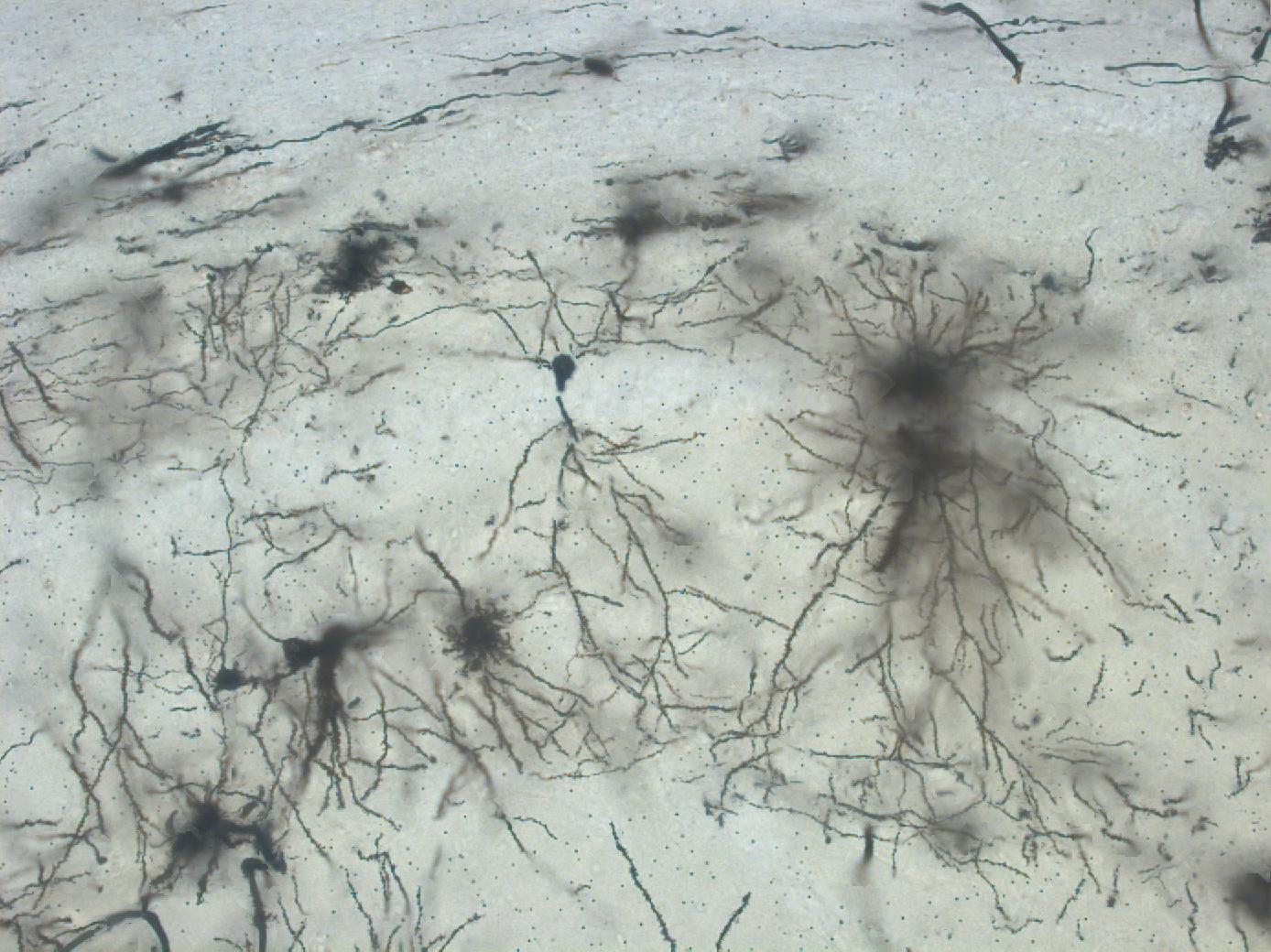

Supplement: Figure 2—figure supplement 1—source data 1. [file elife-86940-fig2-figsupp1-data1.zip › Figure 2-figure supplement 1-source data 1/2369-MUT-20X-1-1.jpg]

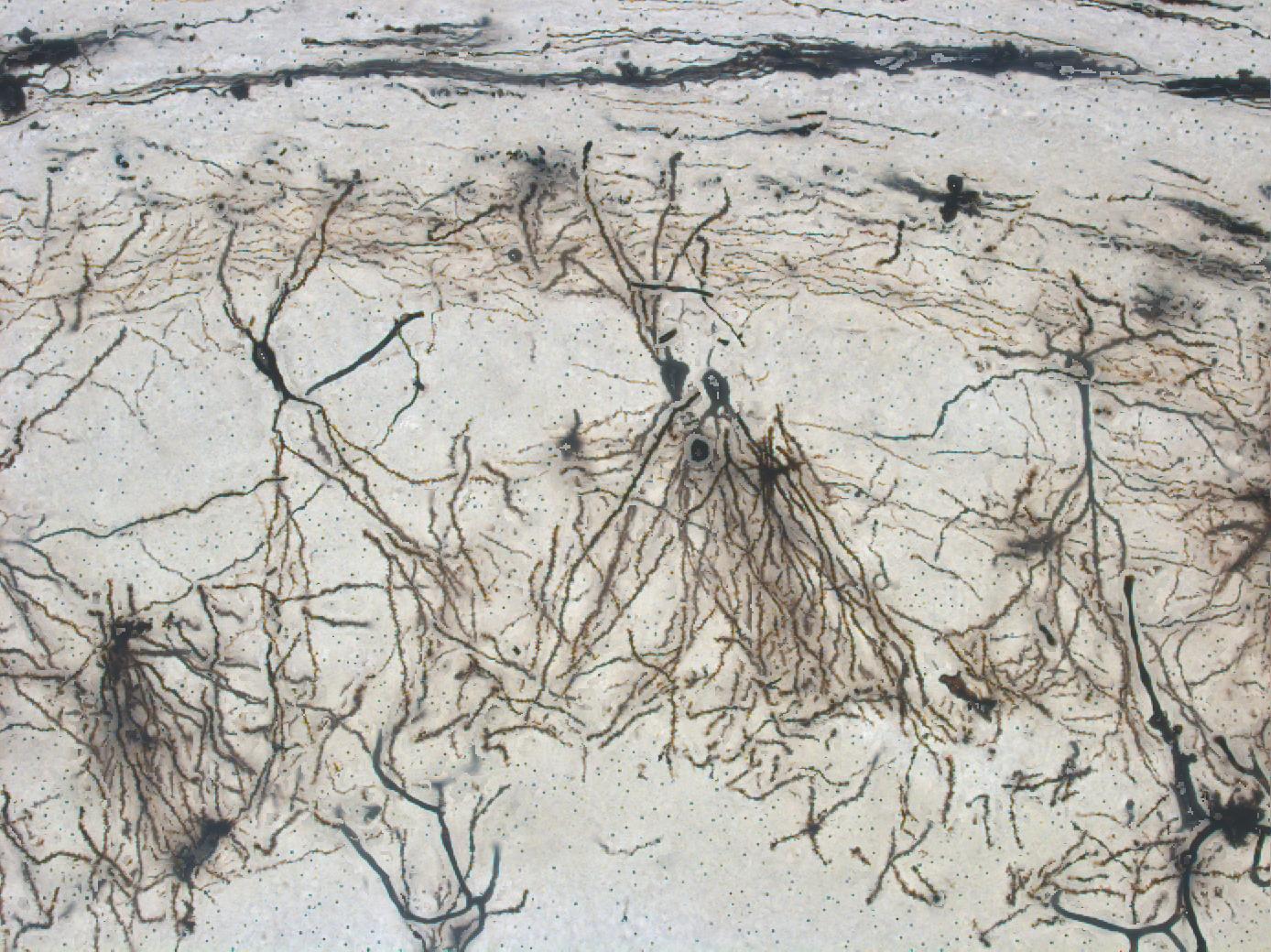

Supplement: Figure 2—figure supplement 1—source data 1. [file elife-86940-fig2-figsupp1-data1.zip › Figure 2-figure supplement 1-source data 1/2369-MUT-20X-1-6-02.jpg]

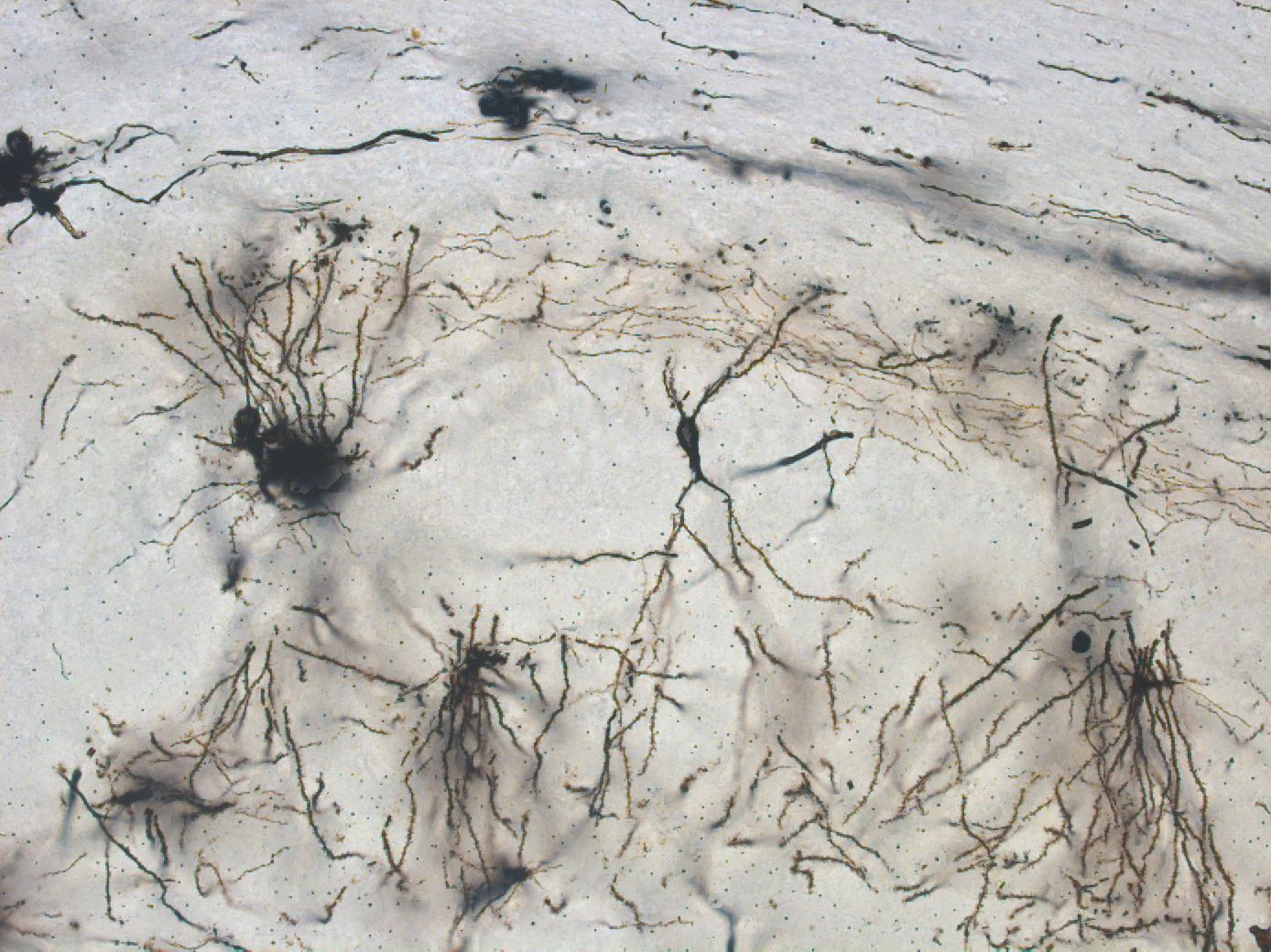

Supplement: Figure 2—figure supplement 1—source data 1. [file elife-86940-fig2-figsupp1-data1.zip › Figure 2-figure supplement 1-source data 1/2369-MUT-20X-1-6-03.jpg]

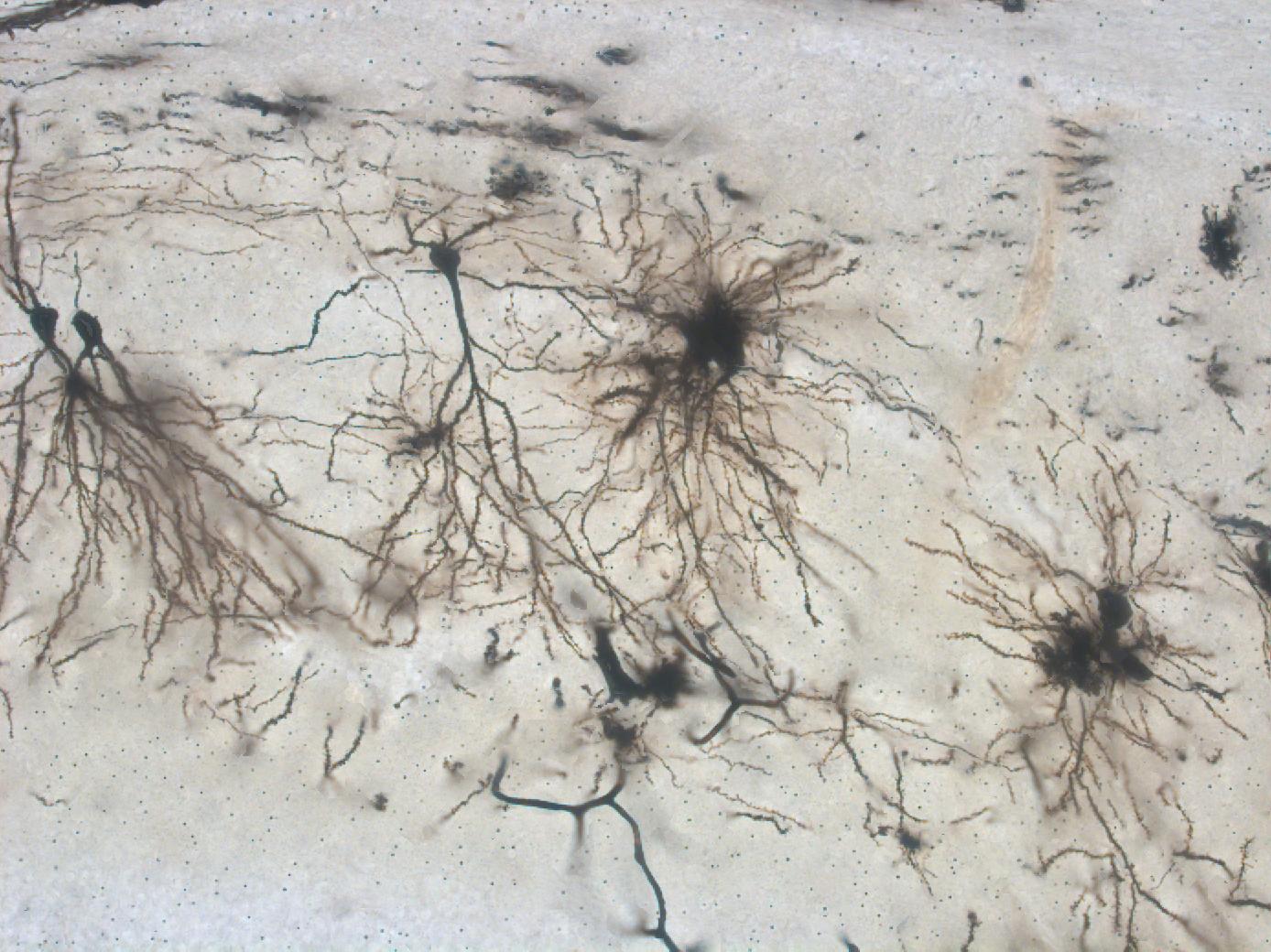

Supplement: Figure 2—figure supplement 1—source data 1. [file elife-86940-fig2-figsupp1-data1.zip › Figure 2-figure supplement 1-source data 1/2369-MUT-20X-1-6.jpg]

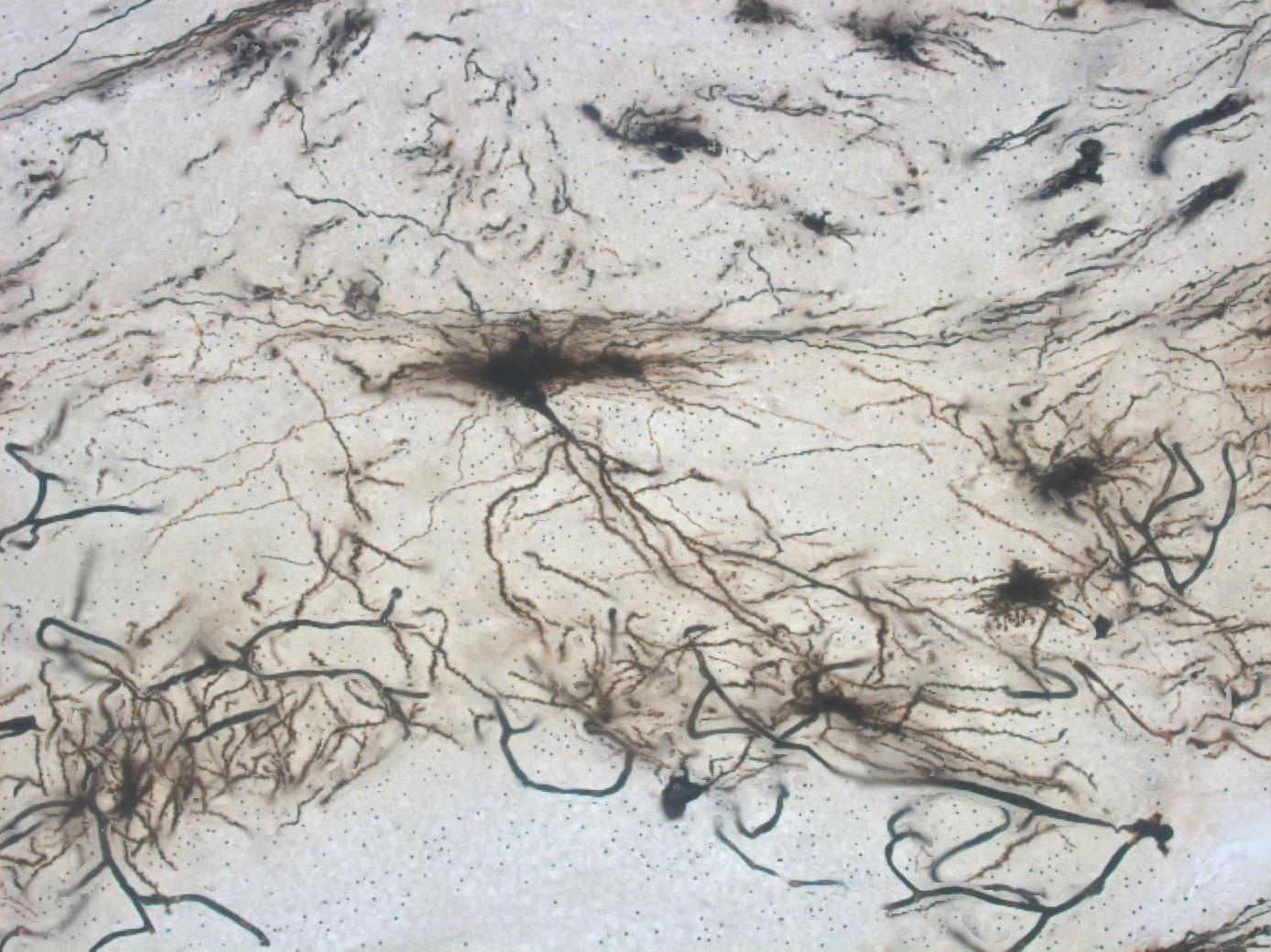

Supplement: Figure 2—figure supplement 1—source data 1. [file elife-86940-fig2-figsupp1-data1.zip › Figure 2-figure supplement 1-source data 1/2369-MUT-20X-2-1.jpg]

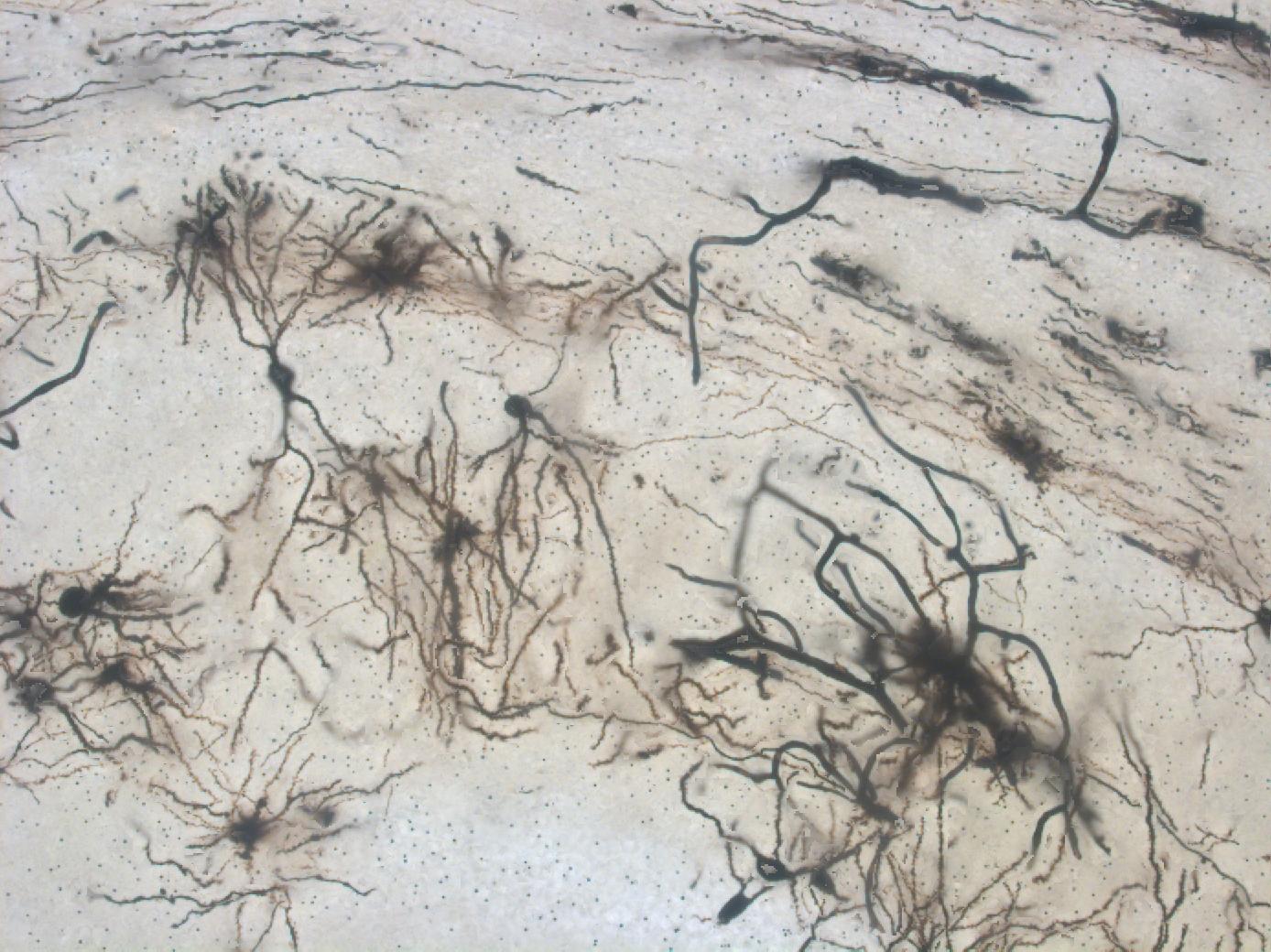

Supplement: Figure 2—figure supplement 1—source data 1. [file elife-86940-fig2-figsupp1-data1.zip › Figure 2-figure supplement 1-source data 1/2369-MUT-20X-2-2-02.jpg]

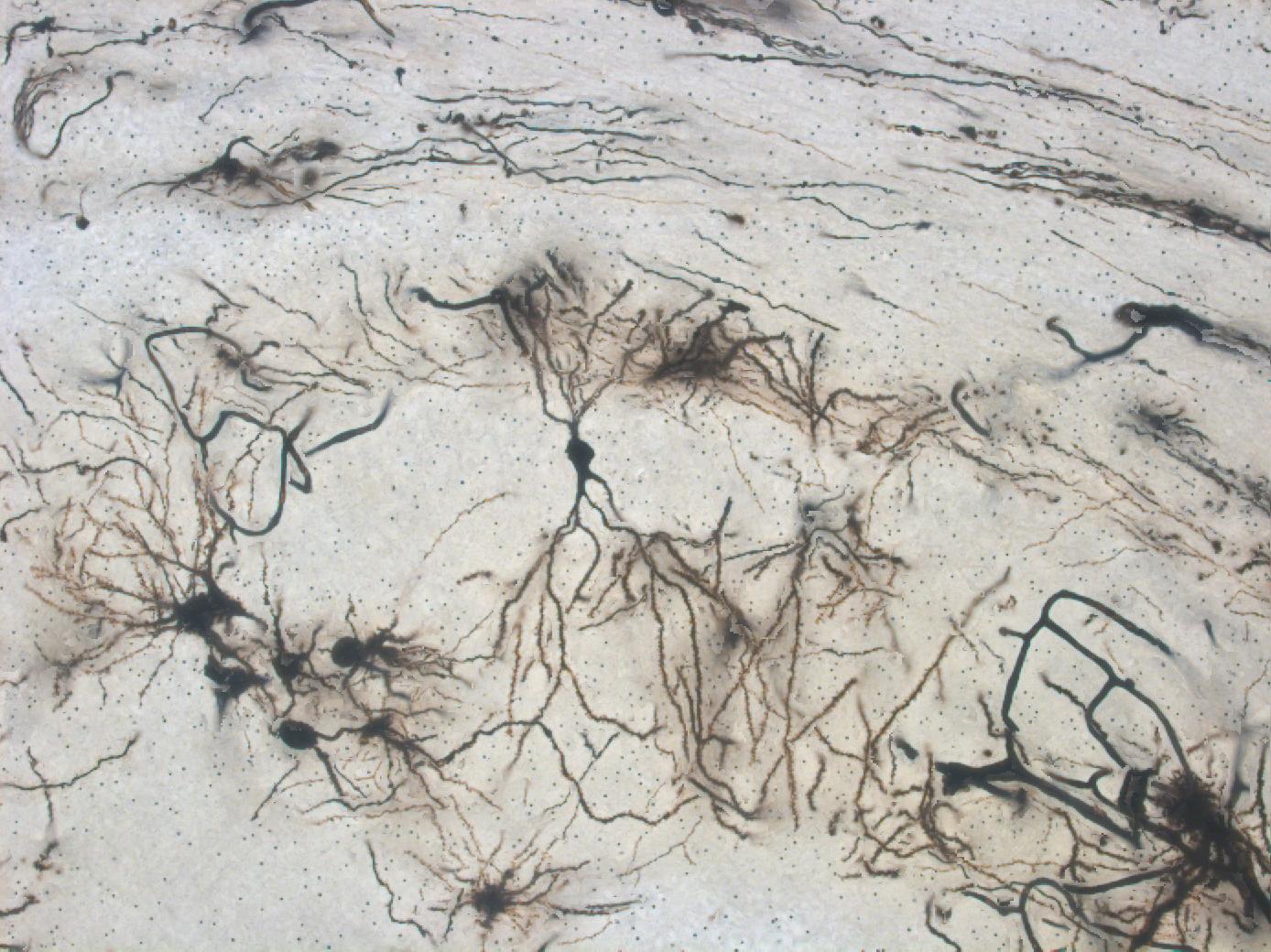

Supplement: Figure 2—figure supplement 1—source data 1. [file elife-86940-fig2-figsupp1-data1.zip › Figure 2-figure supplement 1-source data 1/2369-MUT-20X-2-2.jpg]

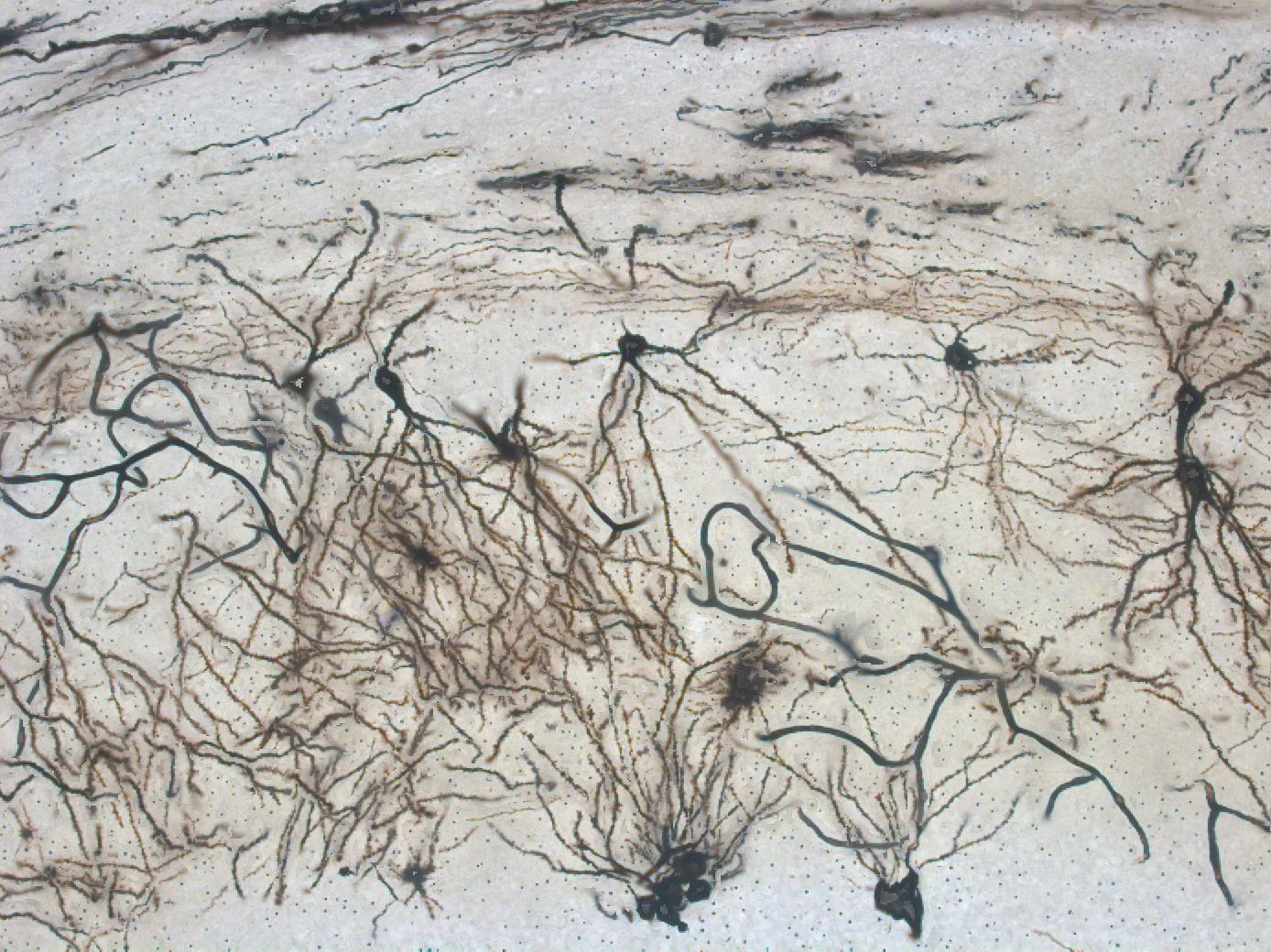

Supplement: Figure 2—figure supplement 1—source data 1. [file elife-86940-fig2-figsupp1-data1.zip › Figure 2-figure supplement 1-source data 1/2369-MUT-20X-6-6-02.jpg]

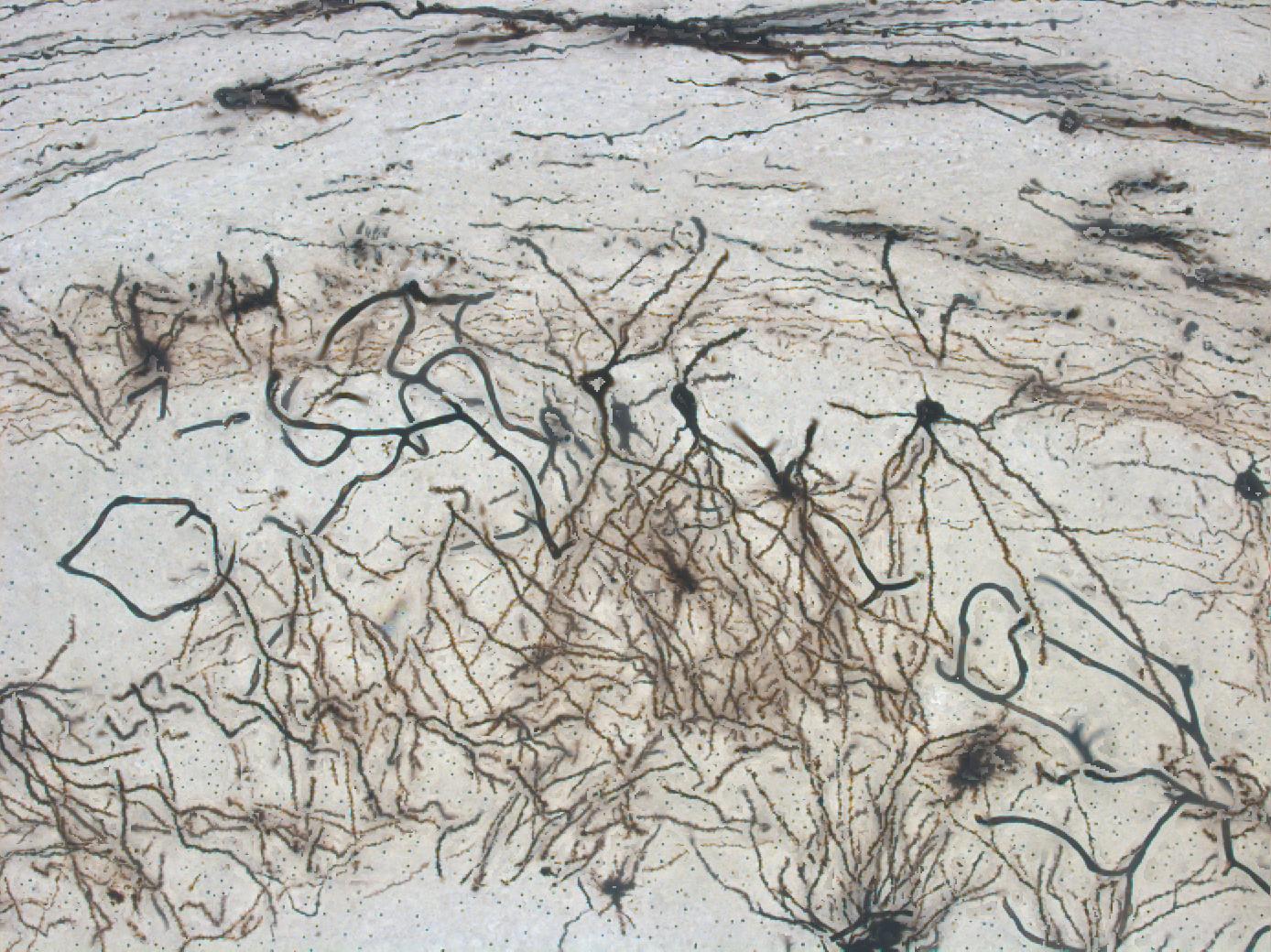

Supplement: Figure 2—figure supplement 1—source data 1. [file elife-86940-fig2-figsupp1-data1.zip › Figure 2-figure supplement 1-source data 1/2369-MUT-20X-6-6-03.jpg]

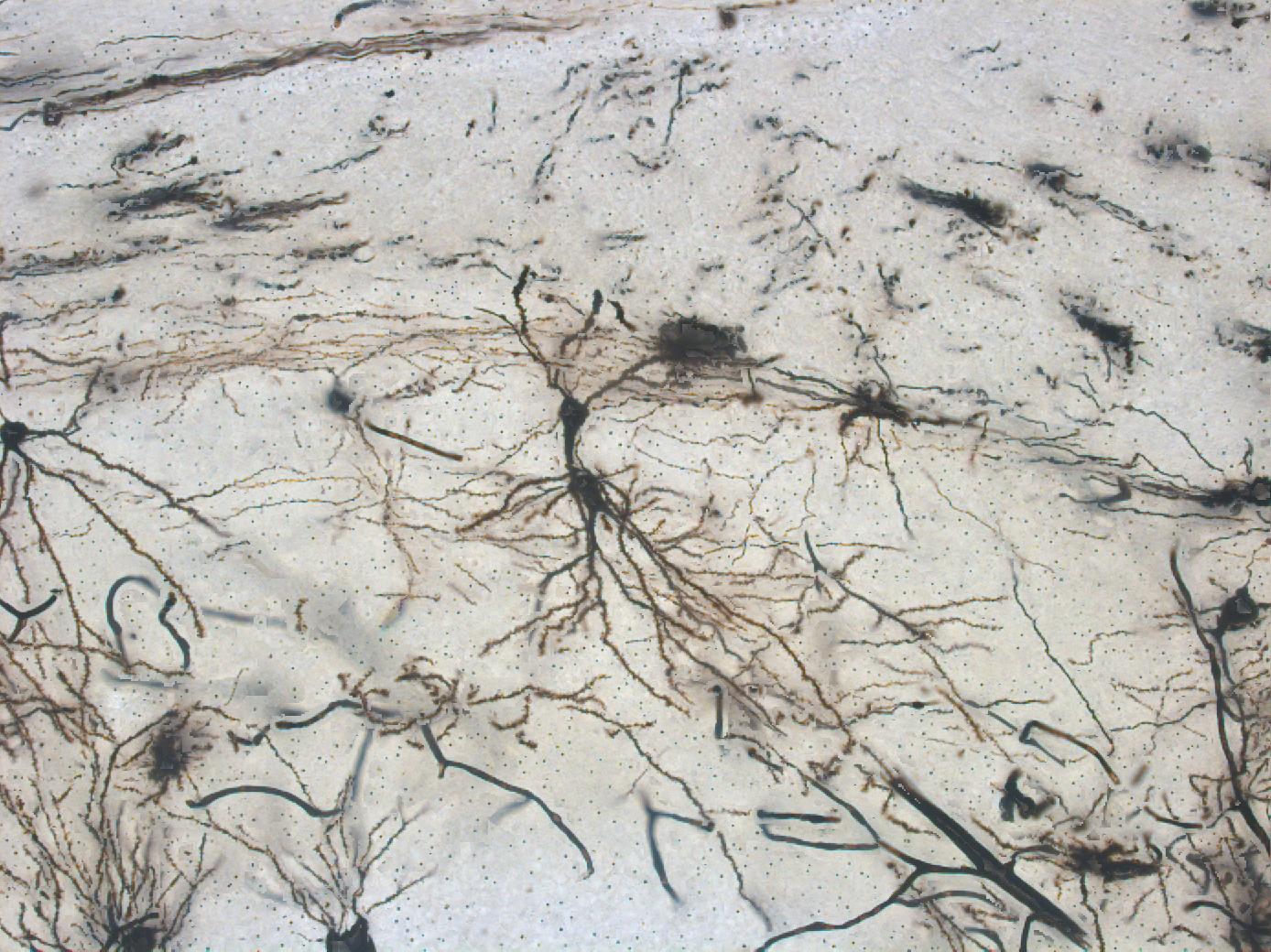

Supplement: Figure 2—figure supplement 1—source data 1. [file elife-86940-fig2-figsupp1-data1.zip › Figure 2-figure supplement 1-source data 1/2369-MUT-20X-6-6.jpg]

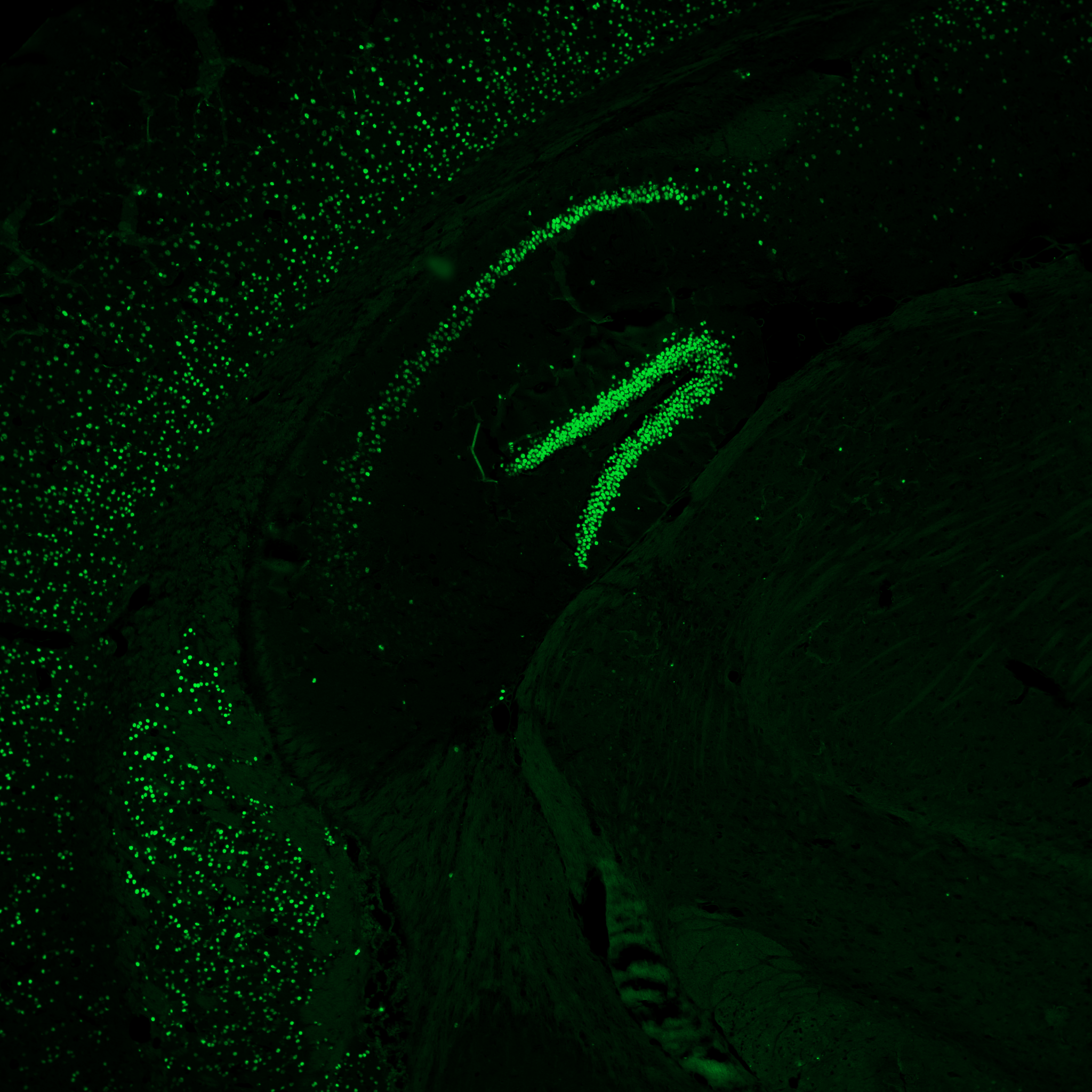

Supplement: Figure 2—figure supplement 1—source data 1. [file elife-86940-fig2-figsupp1-data1.zip › Figure 2-figure supplement 1-source data 1/3442-CKO-EMX1 CI ff-3M-5X-CTIP2-132-3-LHPC-Image Export-42_AF488.tif]

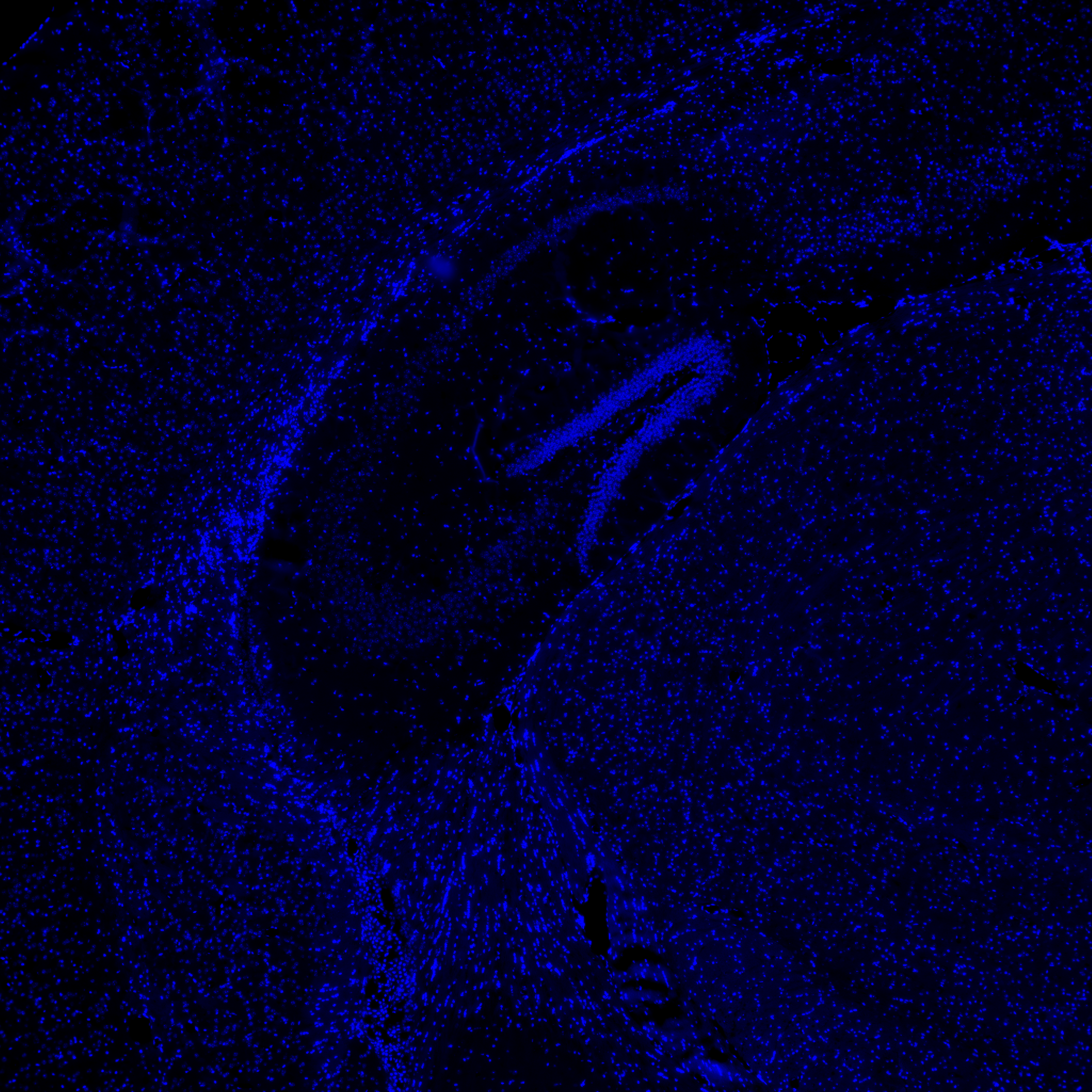

Supplement: Figure 2—figure supplement 1—source data 1. [file elife-86940-fig2-figsupp1-data1.zip › Figure 2-figure supplement 1-source data 1/3442-CKO-EMX1 CI ff-3M-5X-CTIP2-132-3-LHPC-Image Export-42_DAPI.tif]

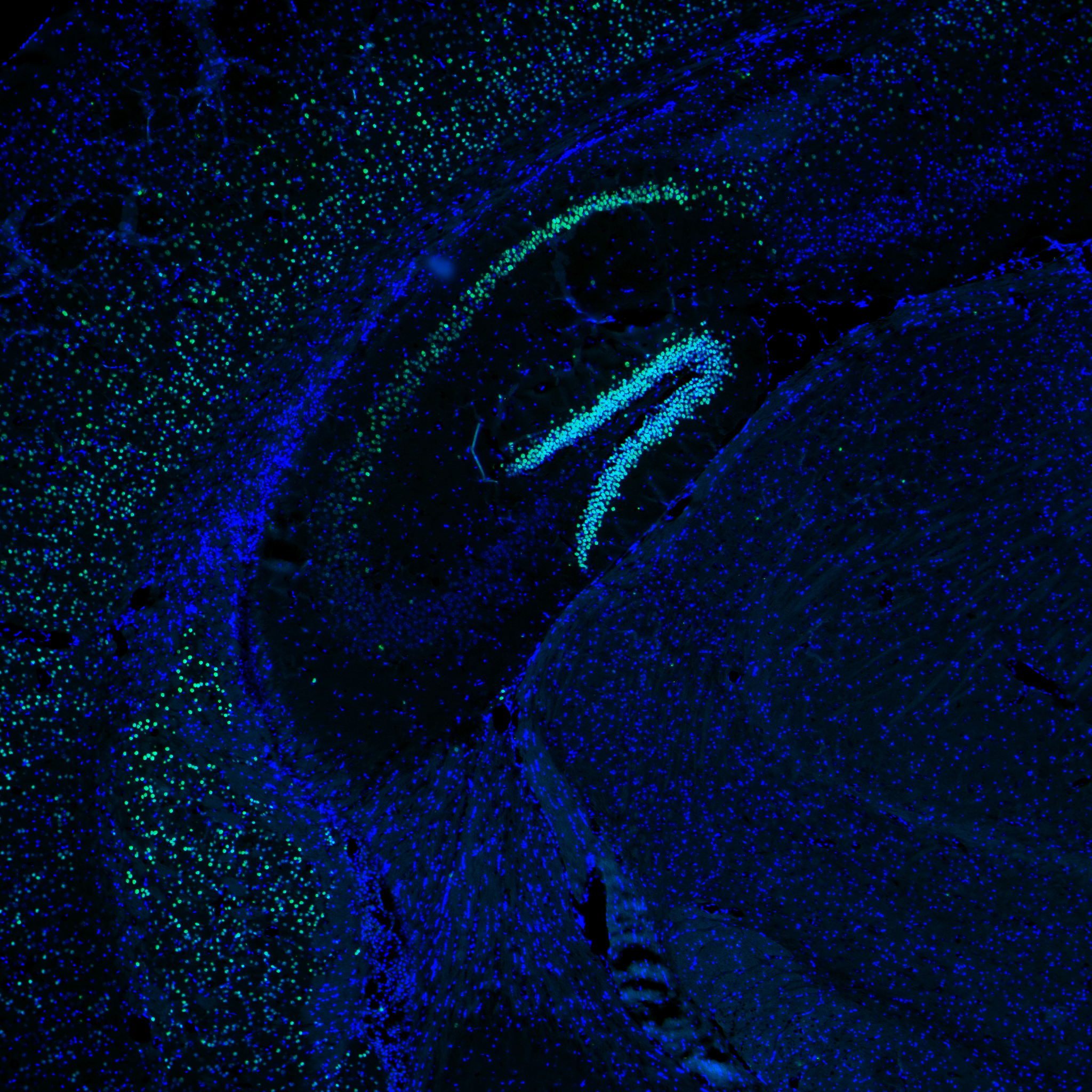

Supplement: Figure 2—figure supplement 1—source data 1. [file elife-86940-fig2-figsupp1-data1.zip › Figure 2-figure supplement 1-source data 1/3442-CKO-EMX1 CI ff-3M-5X-CTIP2-132-3-LHPC-Image Export-42.tif]

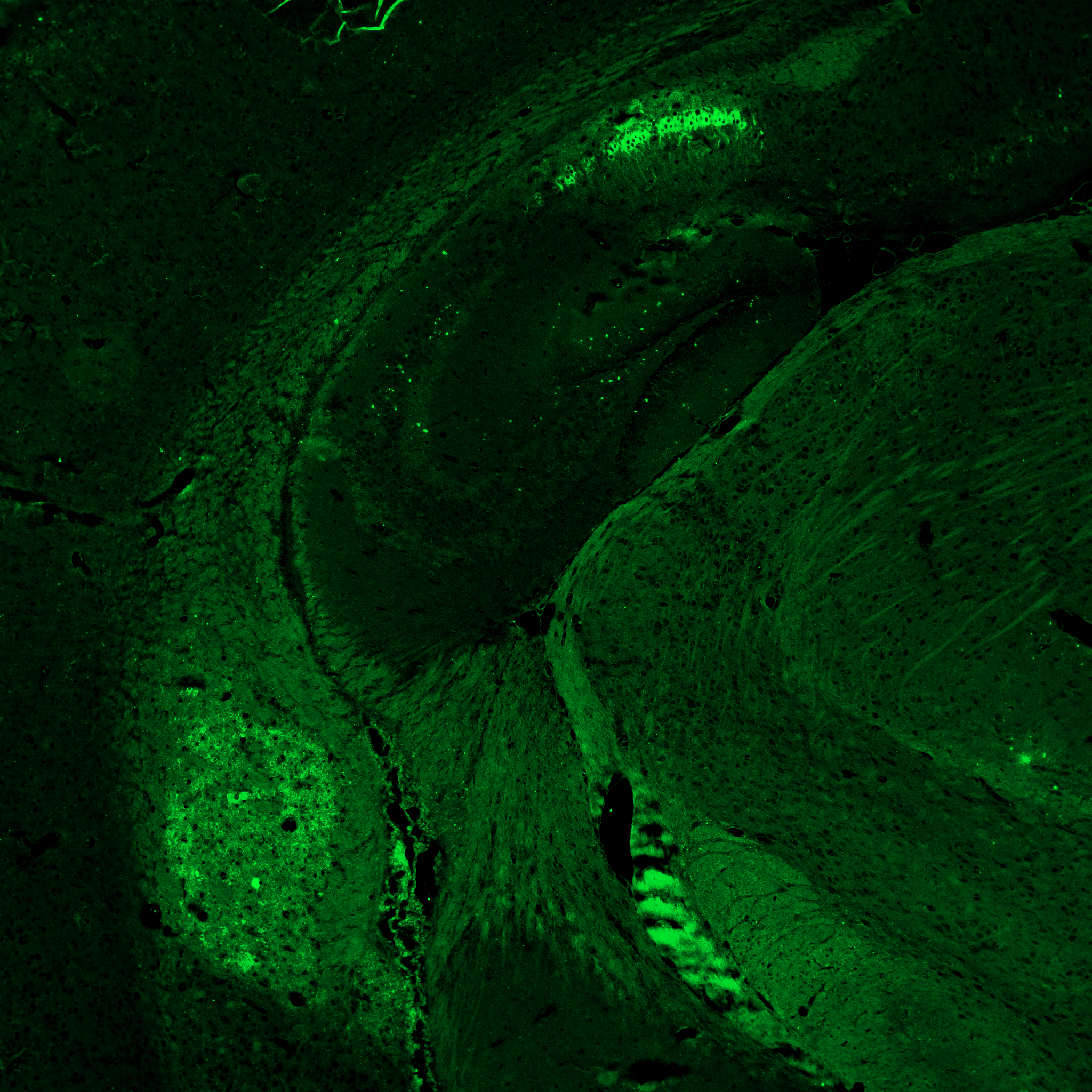

Supplement: Figure 2—figure supplement 1—source data 1. [file elife-86940-fig2-figsupp1-data1.zip › Figure 2-figure supplement 1-source data 1/3442-CKO-EMX1 CI ff-3M-5X-WFS1-132-2-LHPC-Image Export-38_AF488.tif]

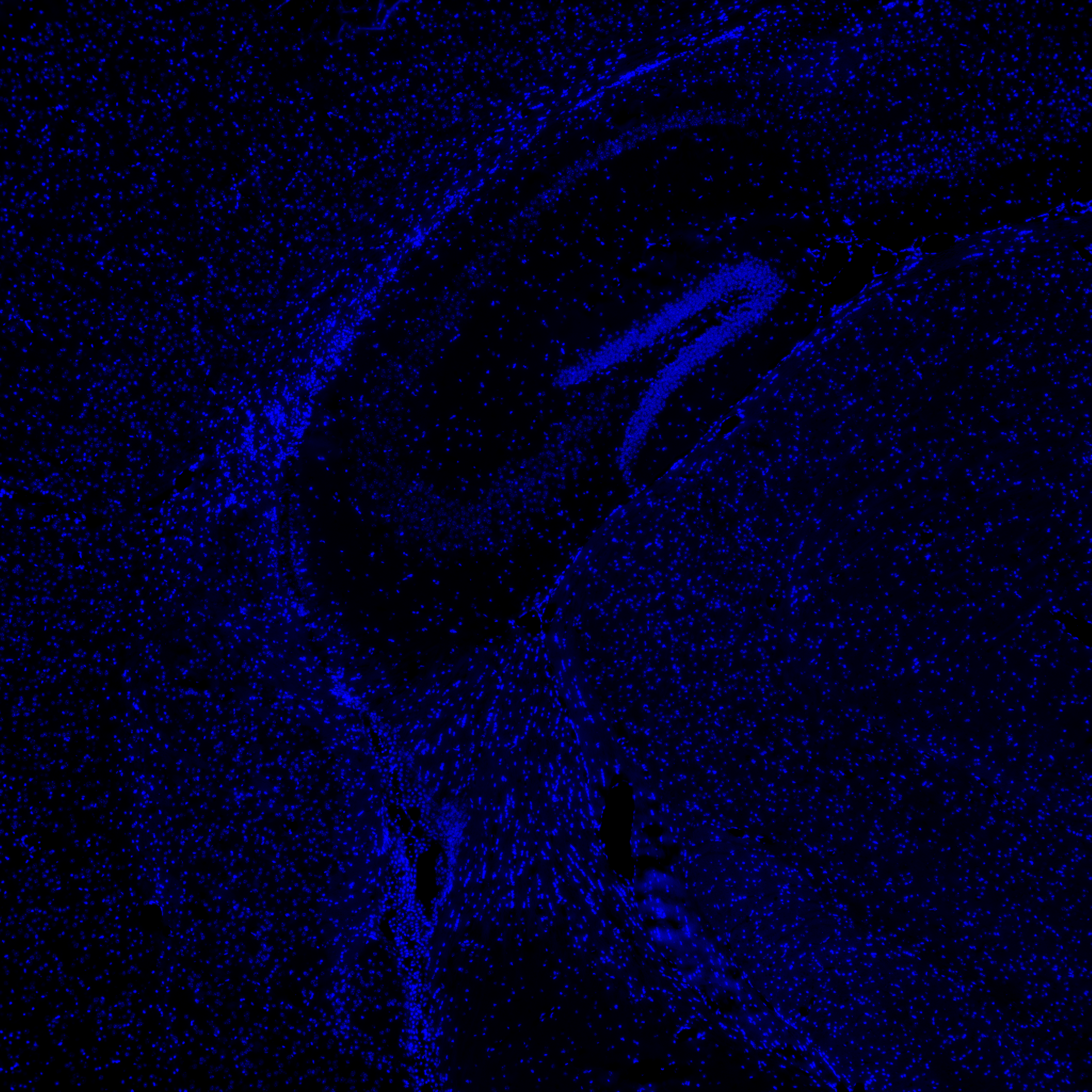

Supplement: Figure 2—figure supplement 1—source data 1. [file elife-86940-fig2-figsupp1-data1.zip › Figure 2-figure supplement 1-source data 1/3442-CKO-EMX1 CI ff-3M-5X-WFS1-132-2-LHPC-Image Export-38_DAPI.tif]

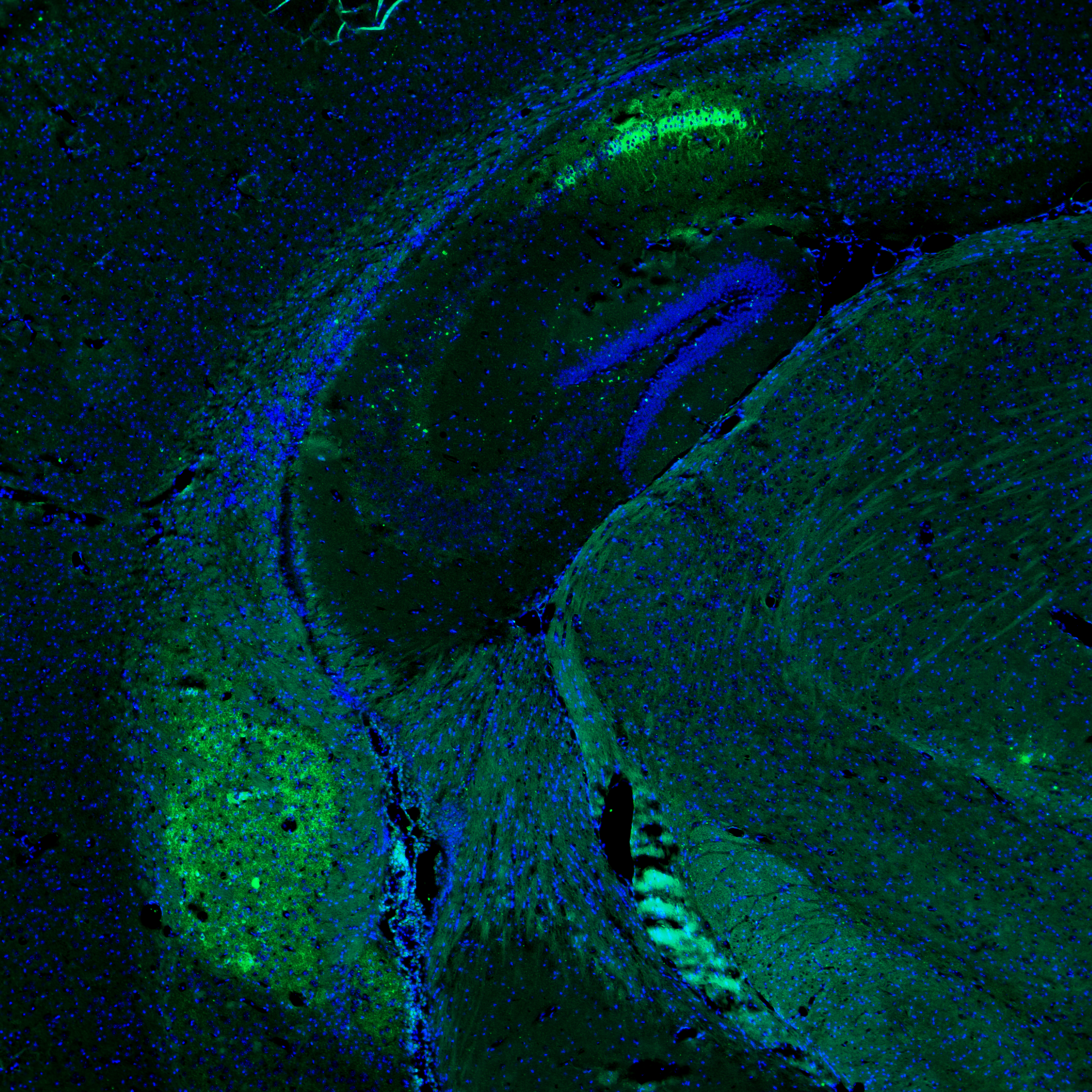

Supplement: Figure 2—figure supplement 1—source data 1. [file elife-86940-fig2-figsupp1-data1.zip › Figure 2-figure supplement 1-source data 1/3442-CKO-EMX1 CI ff-3M-5X-WFS1-132-2-LHPC-Image Export-38.tif]

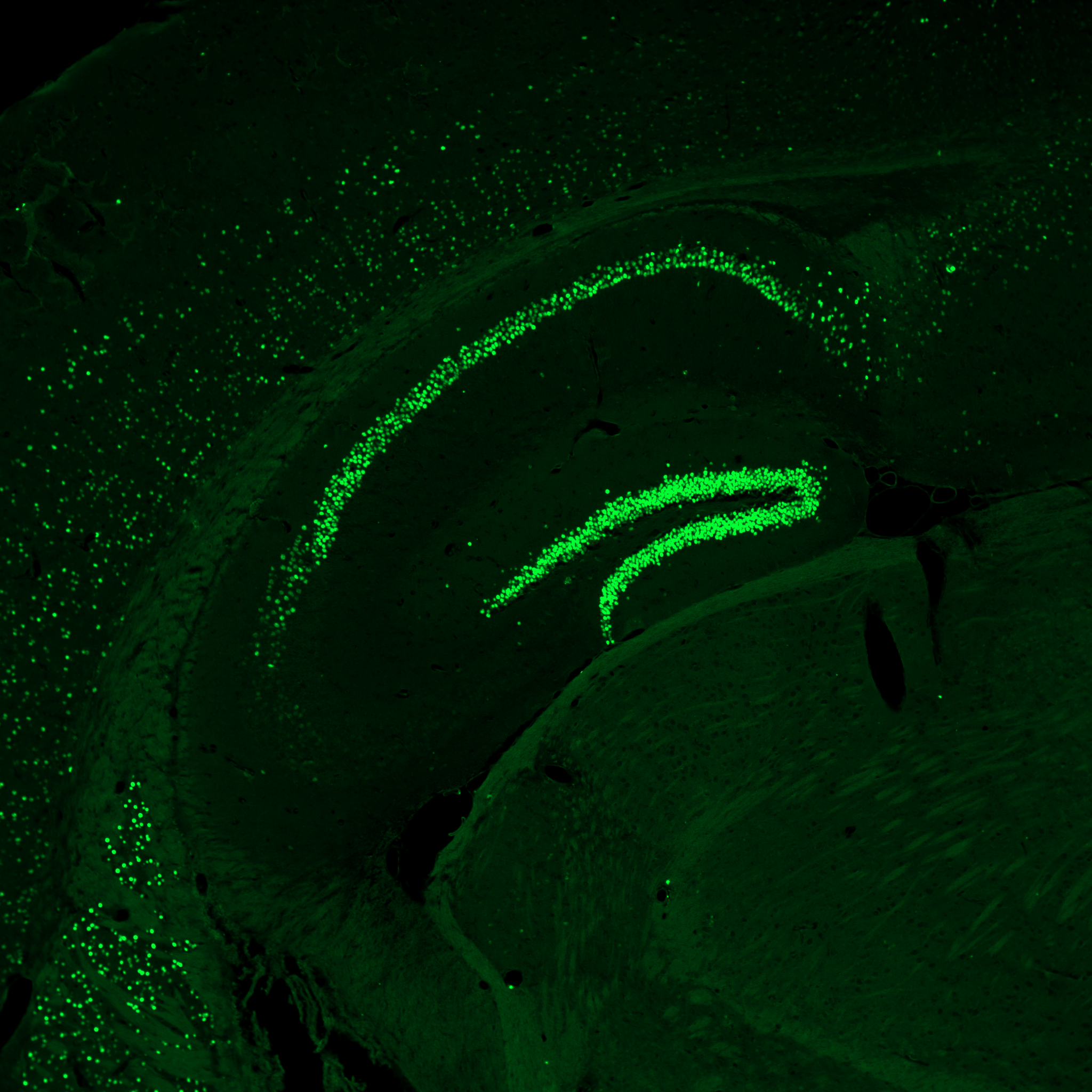

Supplement: Figure 2—figure supplement 1—source data 1. [file elife-86940-fig2-figsupp1-data1.zip › Figure 2-figure supplement 1-source data 1/3442-CON-CI ff-3M-5X-CTIP2-160-3-LHPC-Image Export-10_AF488.tif]

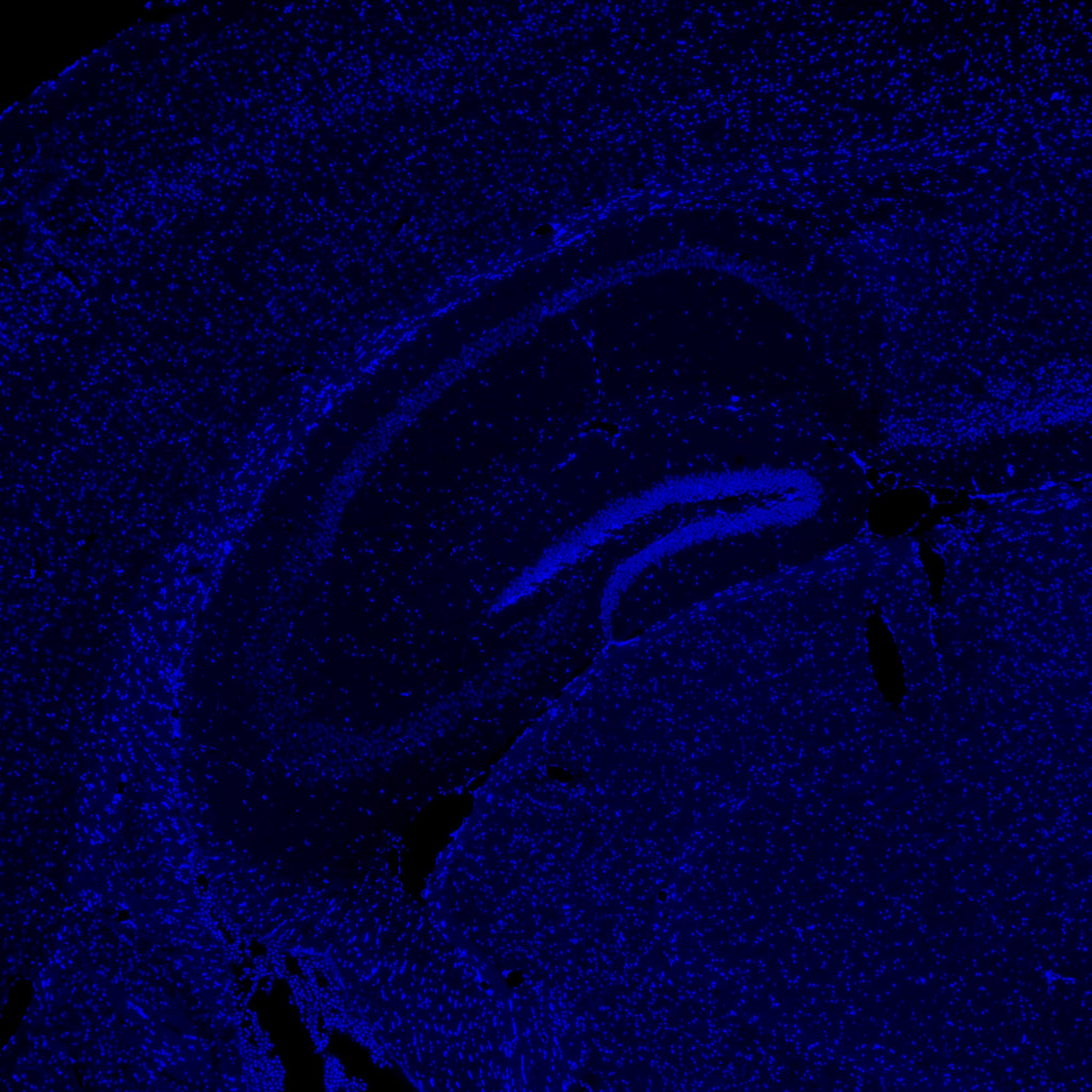

Supplement: Figure 2—figure supplement 1—source data 1. [file elife-86940-fig2-figsupp1-data1.zip › Figure 2-figure supplement 1-source data 1/3442-CON-CI ff-3M-5X-CTIP2-160-3-LHPC-Image Export-10_DAPI.tif]

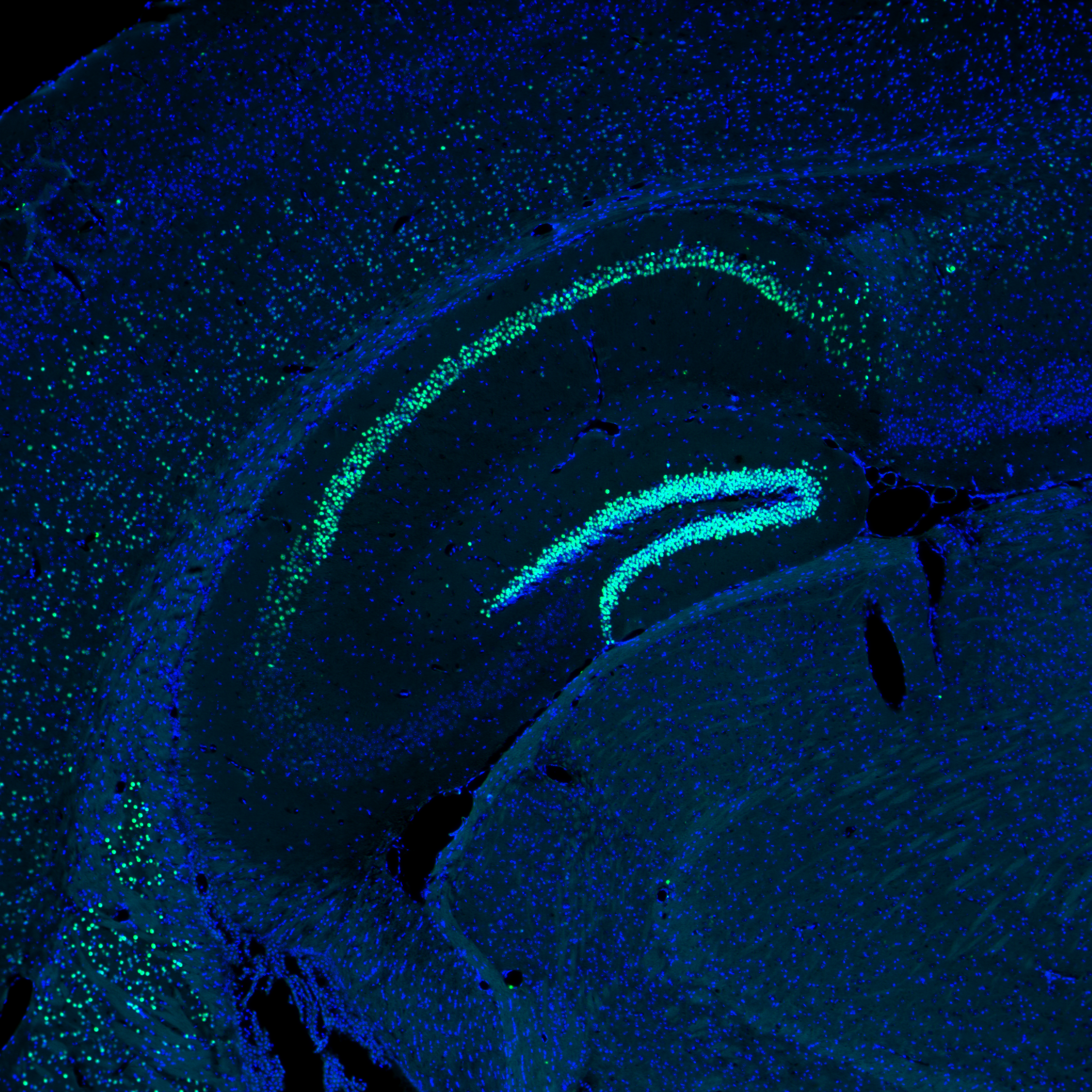

Supplement: Figure 2—figure supplement 1—source data 1. [file elife-86940-fig2-figsupp1-data1.zip › Figure 2-figure supplement 1-source data 1/3442-CON-CI ff-3M-5X-CTIP2-160-3-LHPC-Image Export-10.tif]

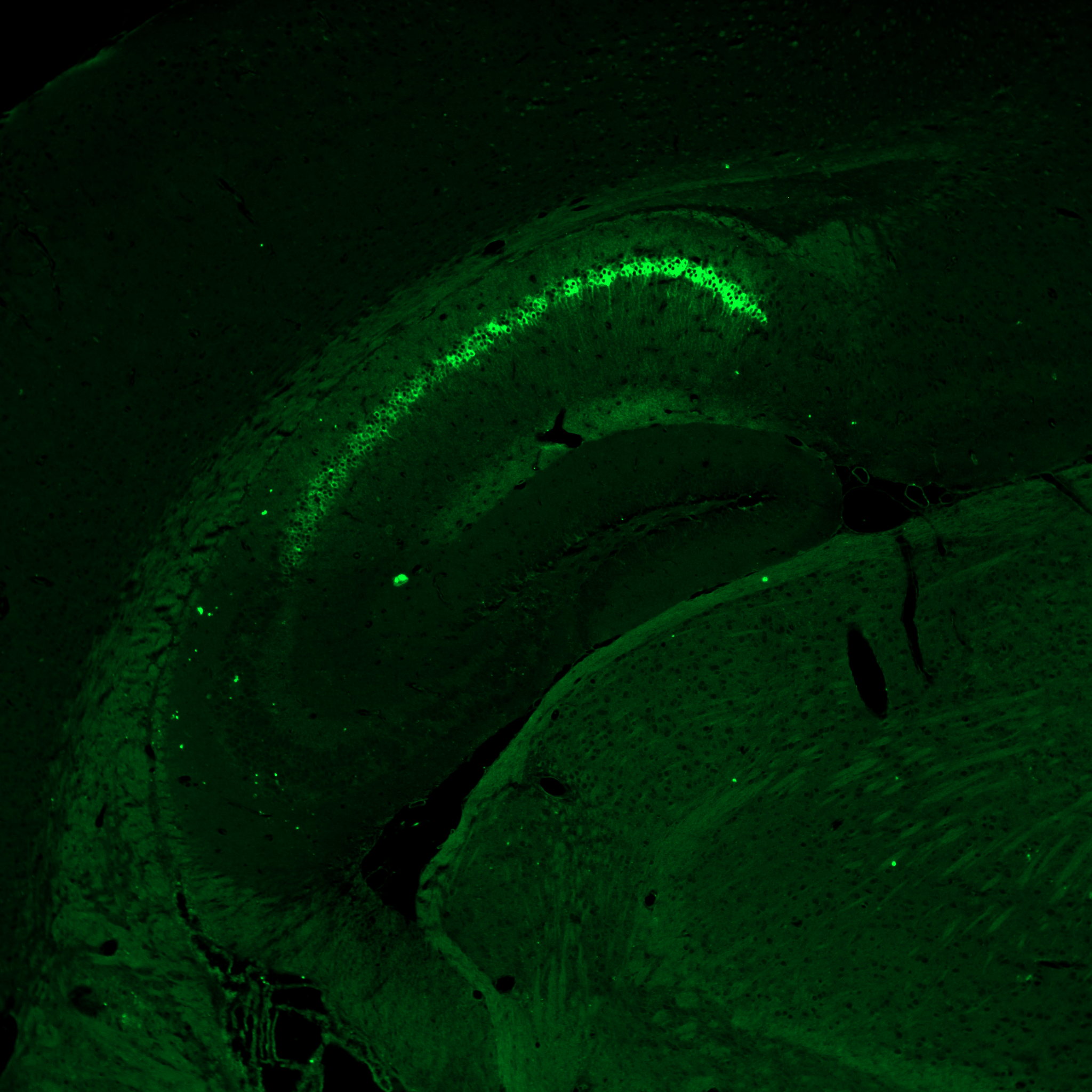

Supplement: Figure 2—figure supplement 1—source data 1. [file elife-86940-fig2-figsupp1-data1.zip › Figure 2-figure supplement 1-source data 1/3442-CON-CI ff-3M-5X-WFS1-160-2-LHPC-Image Export-06_AF488.tif]

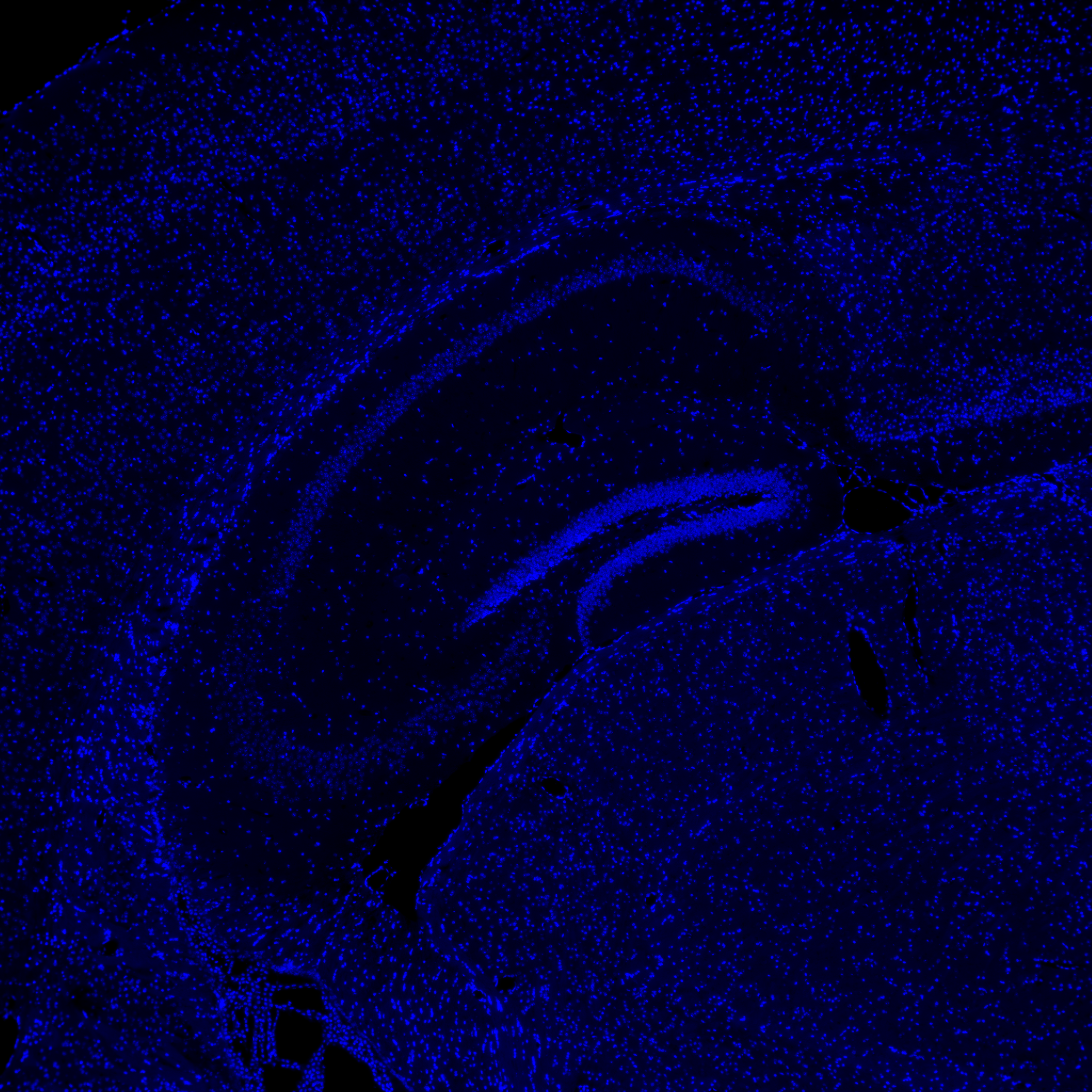

Supplement: Figure 2—figure supplement 1—source data 1. [file elife-86940-fig2-figsupp1-data1.zip › Figure 2-figure supplement 1-source data 1/3442-CON-CI ff-3M-5X-WFS1-160-2-LHPC-Image Export-06_DAPI.tif]

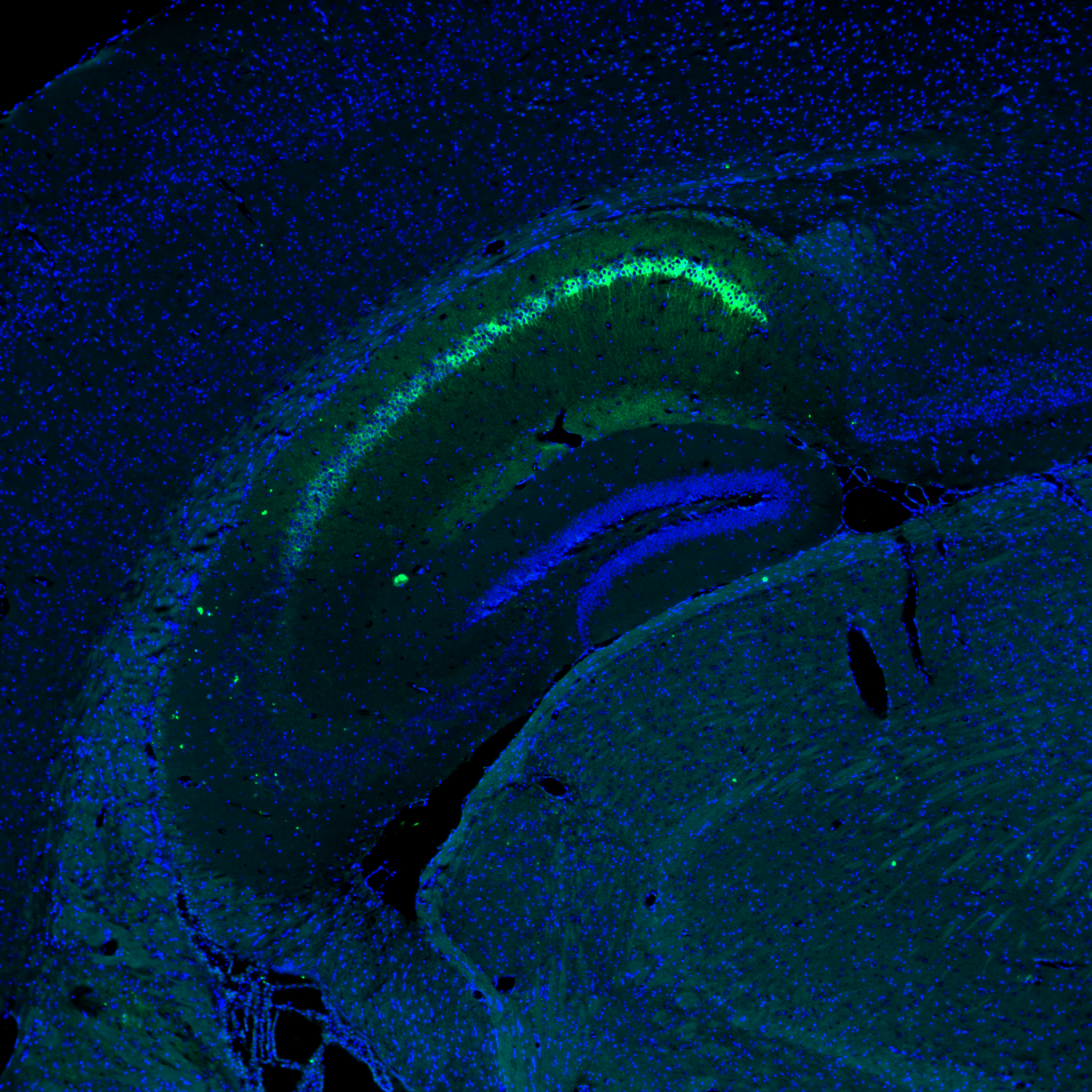

Supplement: Figure 2—figure supplement 1—source data 1. [file elife-86940-fig2-figsupp1-data1.zip › Figure 2-figure supplement 1-source data 1/3442-CON-CI ff-3M-5X-WFS1-160-2-LHPC-Image Export-06.tif]

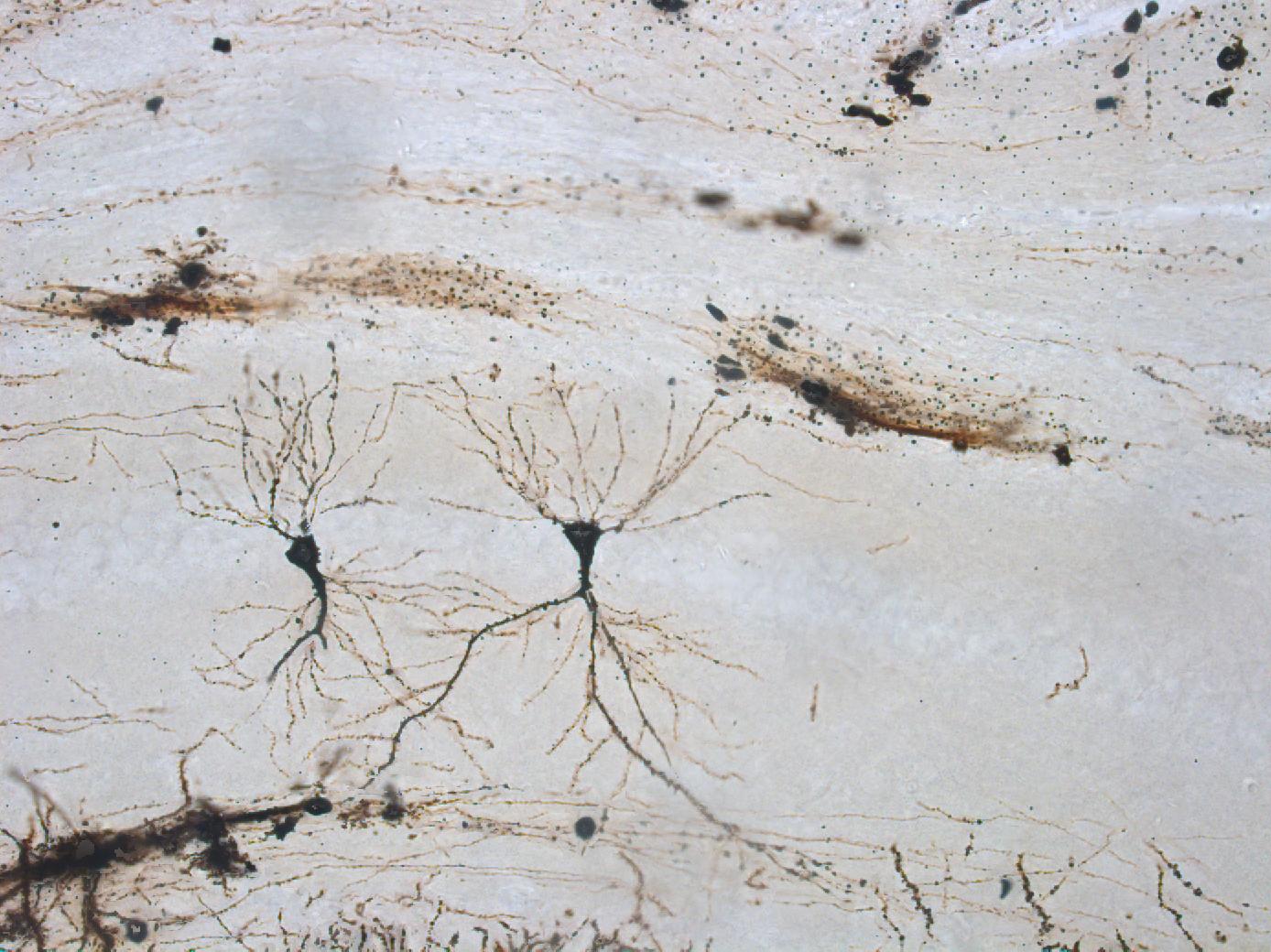

Supplement: Figure 2—figure supplement 1—source data 1. [file elife-86940-fig2-figsupp1-data1.zip › Figure 2-figure supplement 1-source data 1/Golgi figures/2065-CON-4-3-20X-1.jpg]

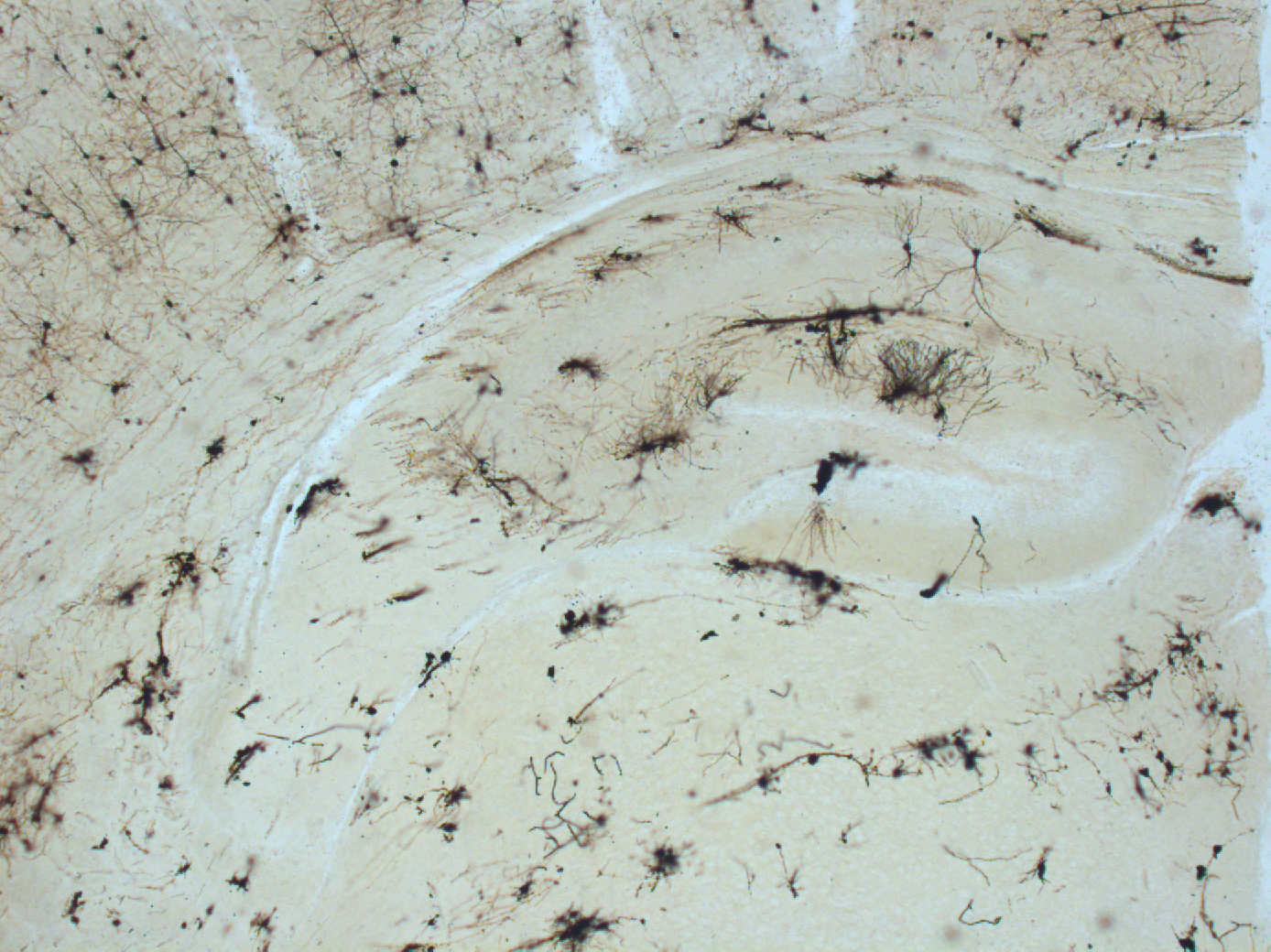

Supplement: Figure 2—figure supplement 1—source data 1. [file elife-86940-fig2-figsupp1-data1.zip › Figure 2-figure supplement 1-source data 1/Golgi figures/2065-CON-4-3-5X.jpg]

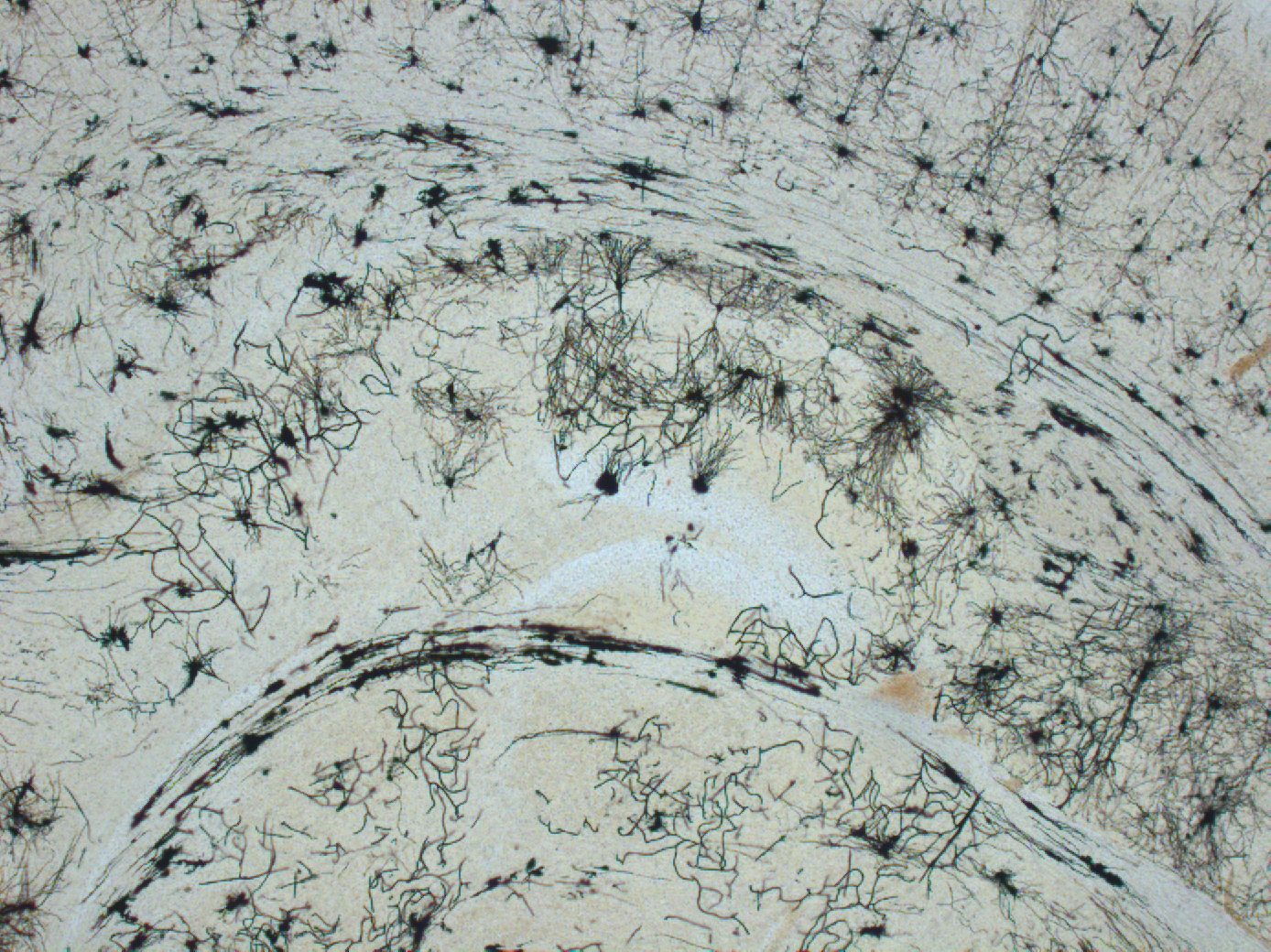

Supplement: Figure 2—figure supplement 1—source data 1. [file elife-86940-fig2-figsupp1-data1.zip › Figure 2-figure supplement 1-source data 1/Golgi figures/2369-MUT-5X-1-01.jpg]

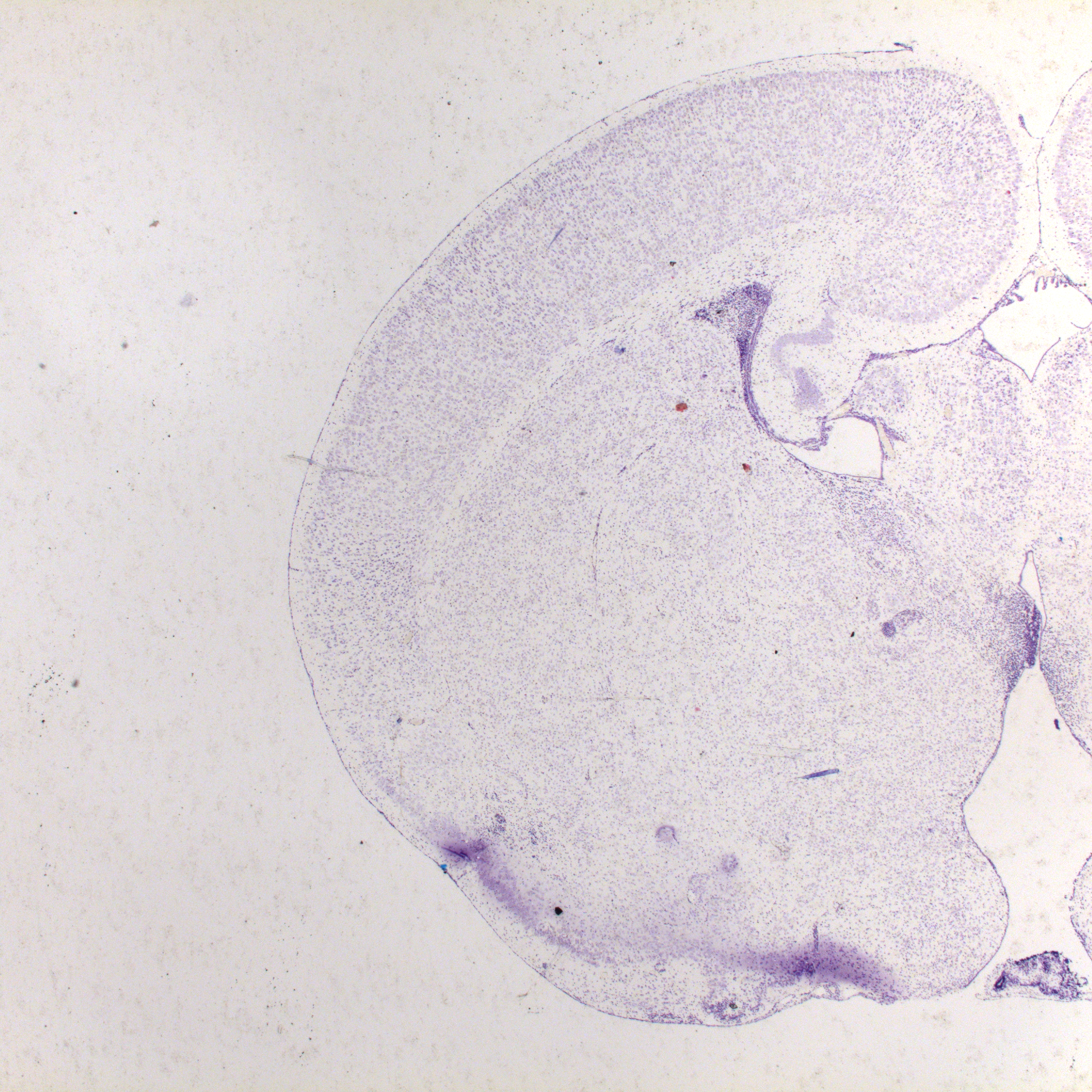

Supplement: Figure 3—source data 1. [file elife-86940-fig3-data1.zip › Figure 3-source data 1/F8099-1-DKO-RX FF ff-P20-2.5X-63-3-L-Image Export-21_Bright.tif]

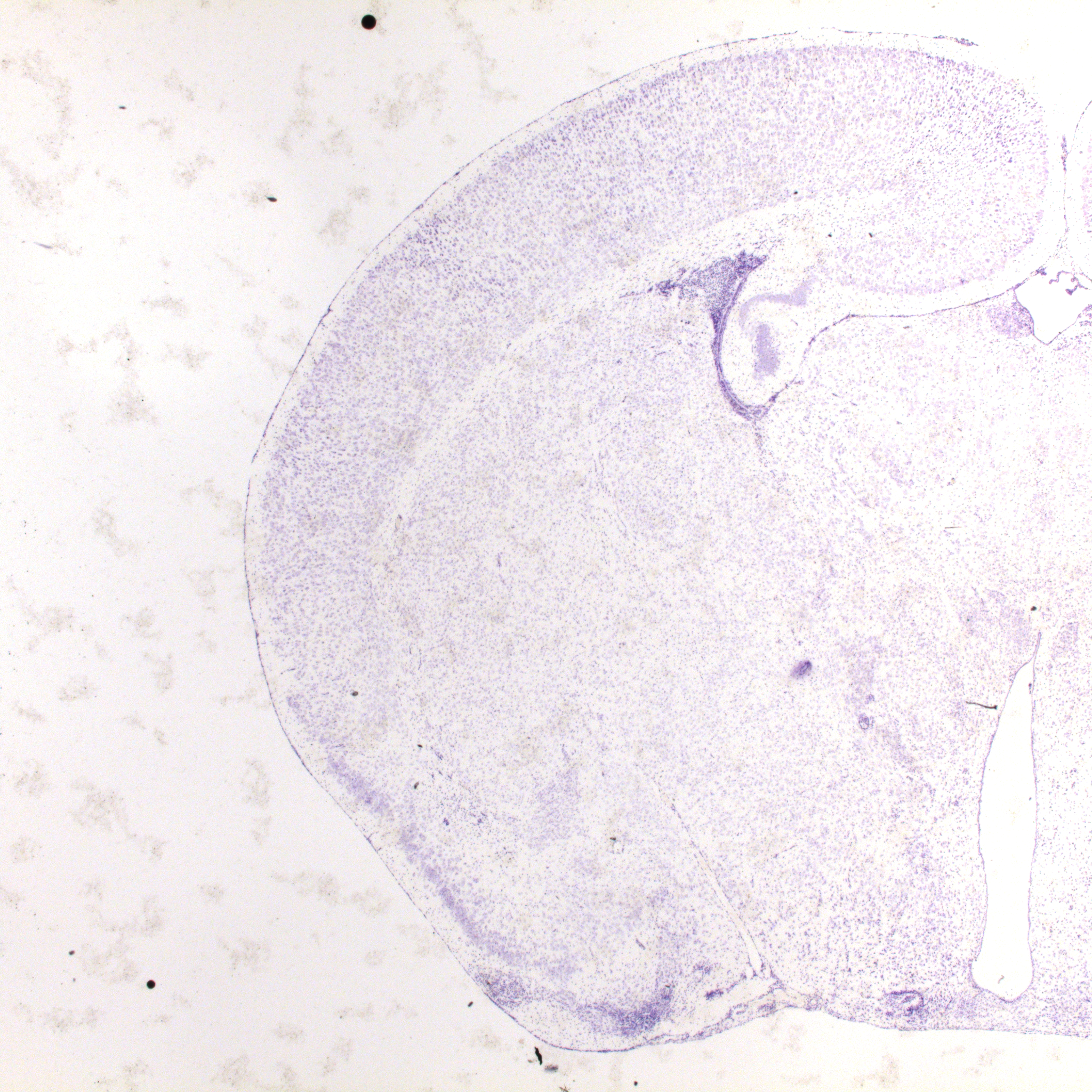

Supplement: Figure 3—source data 1. [file elife-86940-fig3-data1.zip › Figure 3-source data 1/F8099-1-DKO-RX FF ff-P20-2.5X-70-1-L-Image Export-23_Bright.tif]

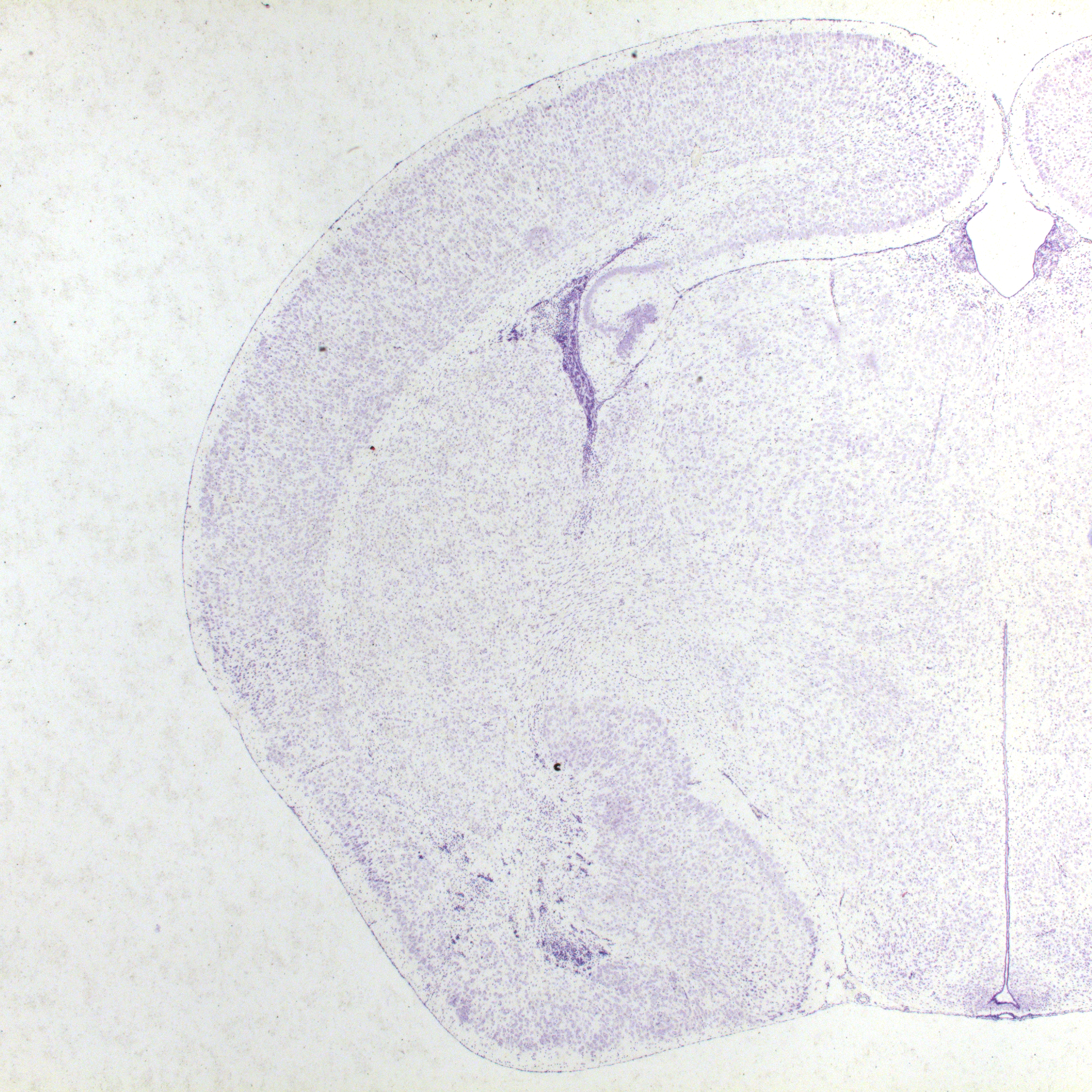

Supplement: Figure 3—source data 1. [file elife-86940-fig3-data1.zip › Figure 3-source data 1/F8099-1-DKO-RX FF ff-P20-2.5X-89-3-L-Image Export-03_Bright.tif]

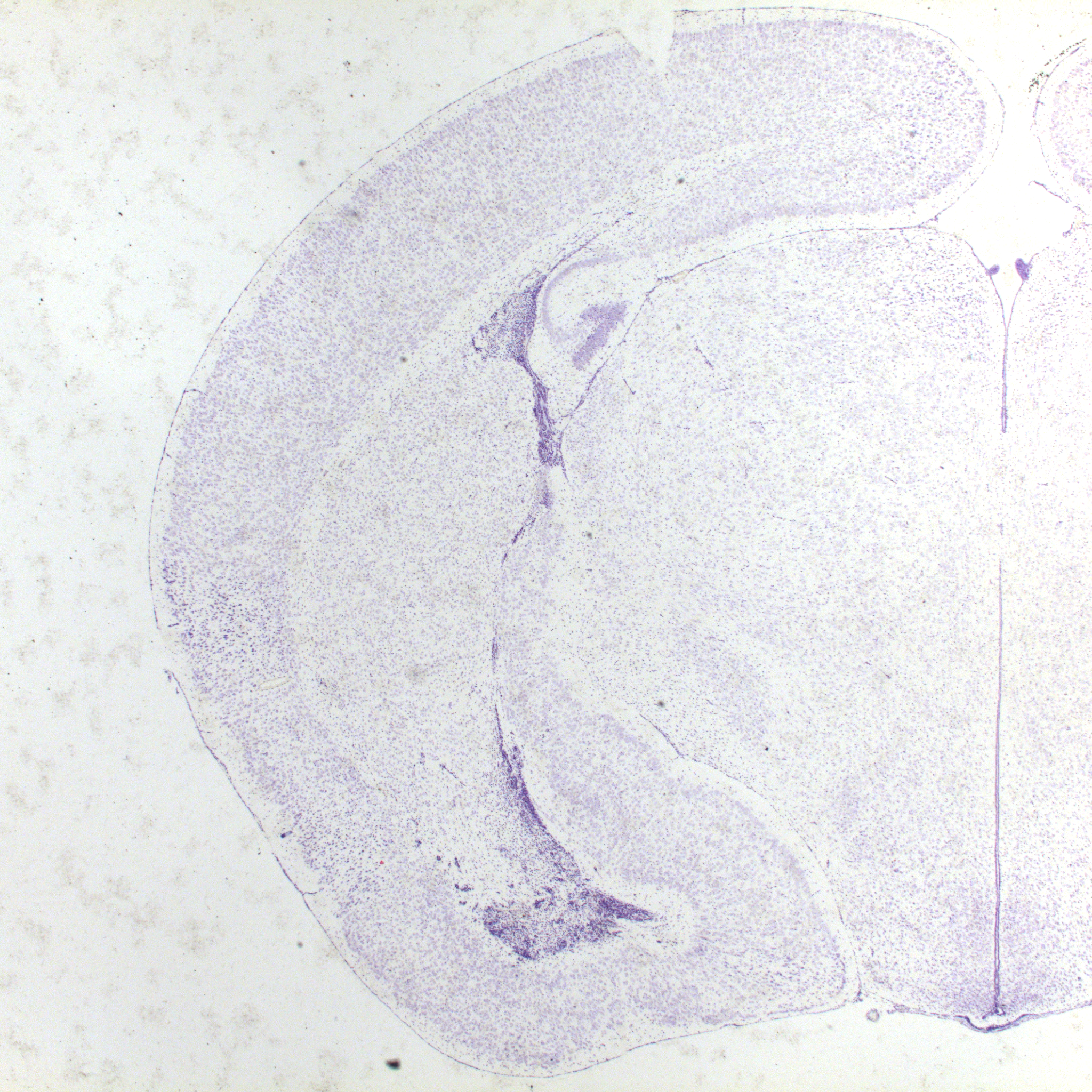

Supplement: Figure 3—source data 1. [file elife-86940-fig3-data1.zip › Figure 3-source data 1/F8099-1-DKO-RX FF ff-P20-2.5X-98-3-L-Image Export-05_Bright.tif]

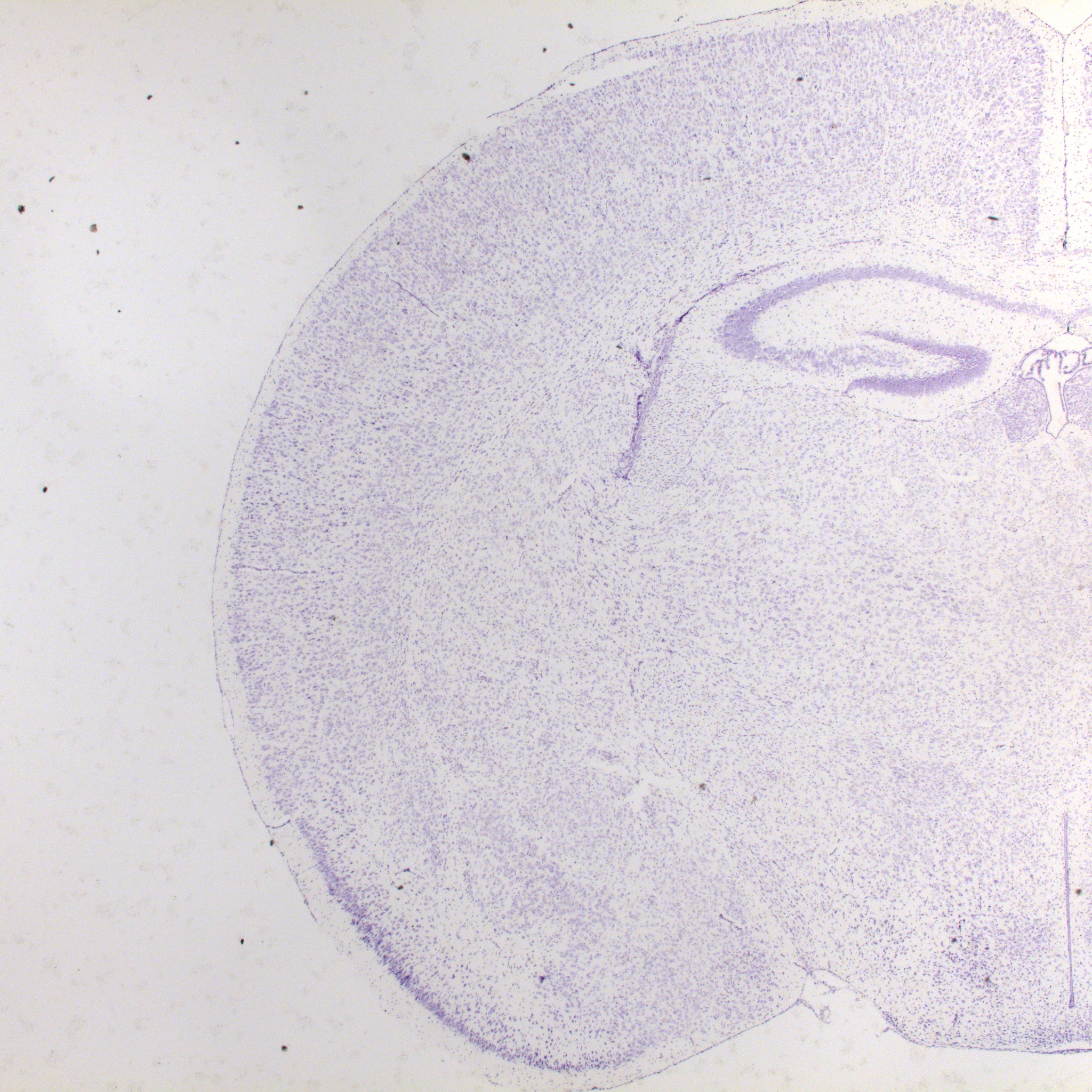

Supplement: Figure 3—source data 1. [file elife-86940-fig3-data1.zip › Figure 3-source data 1/F8099-3-CON-FF ff-P20-2.5X-100-1-L-Image Export-04_Bright.tif]

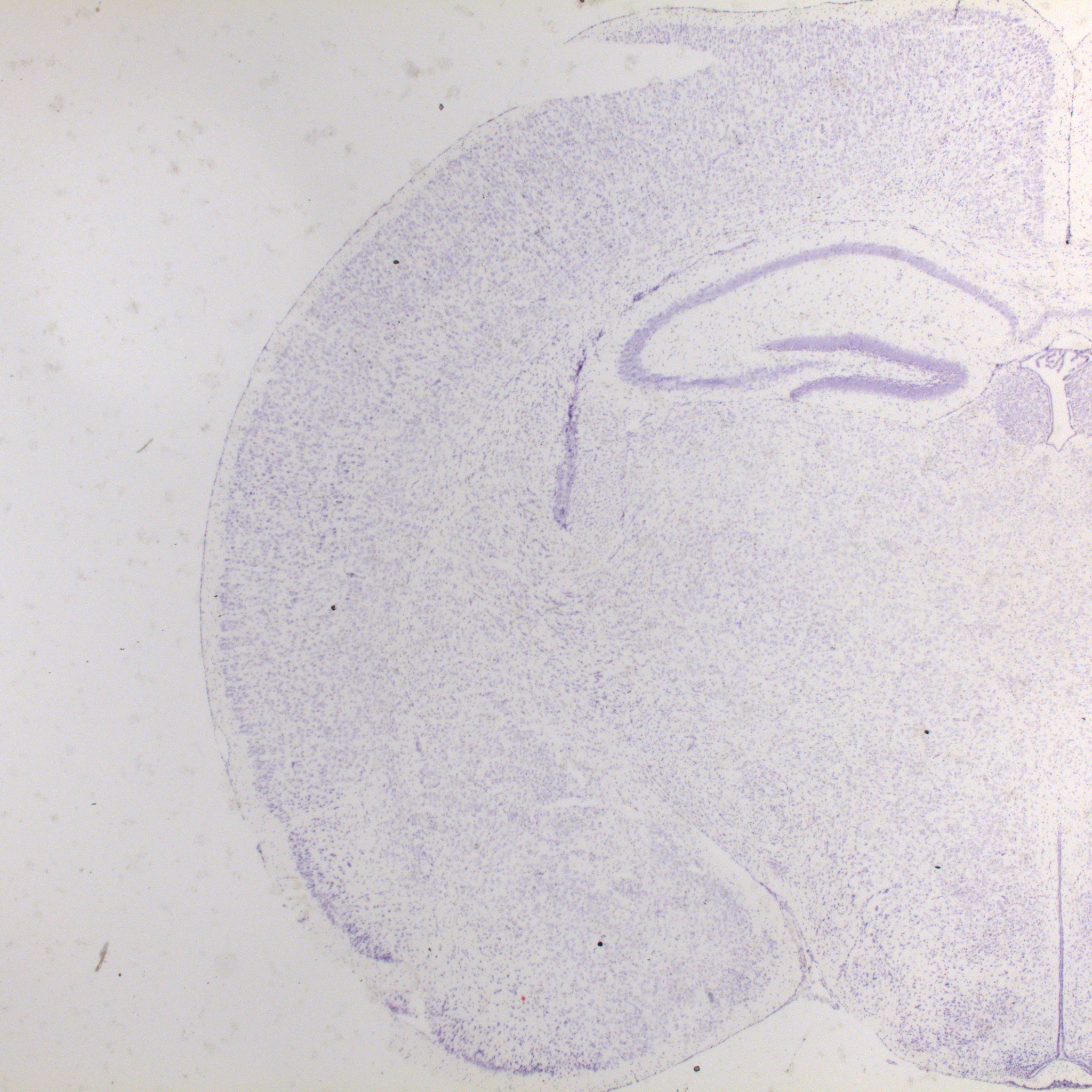

Supplement: Figure 3—source data 1. [file elife-86940-fig3-data1.zip › Figure 3-source data 1/F8099-3-CON-FF ff-P20-2.5X-108-2-L-Image Export-06_Bright.tif]

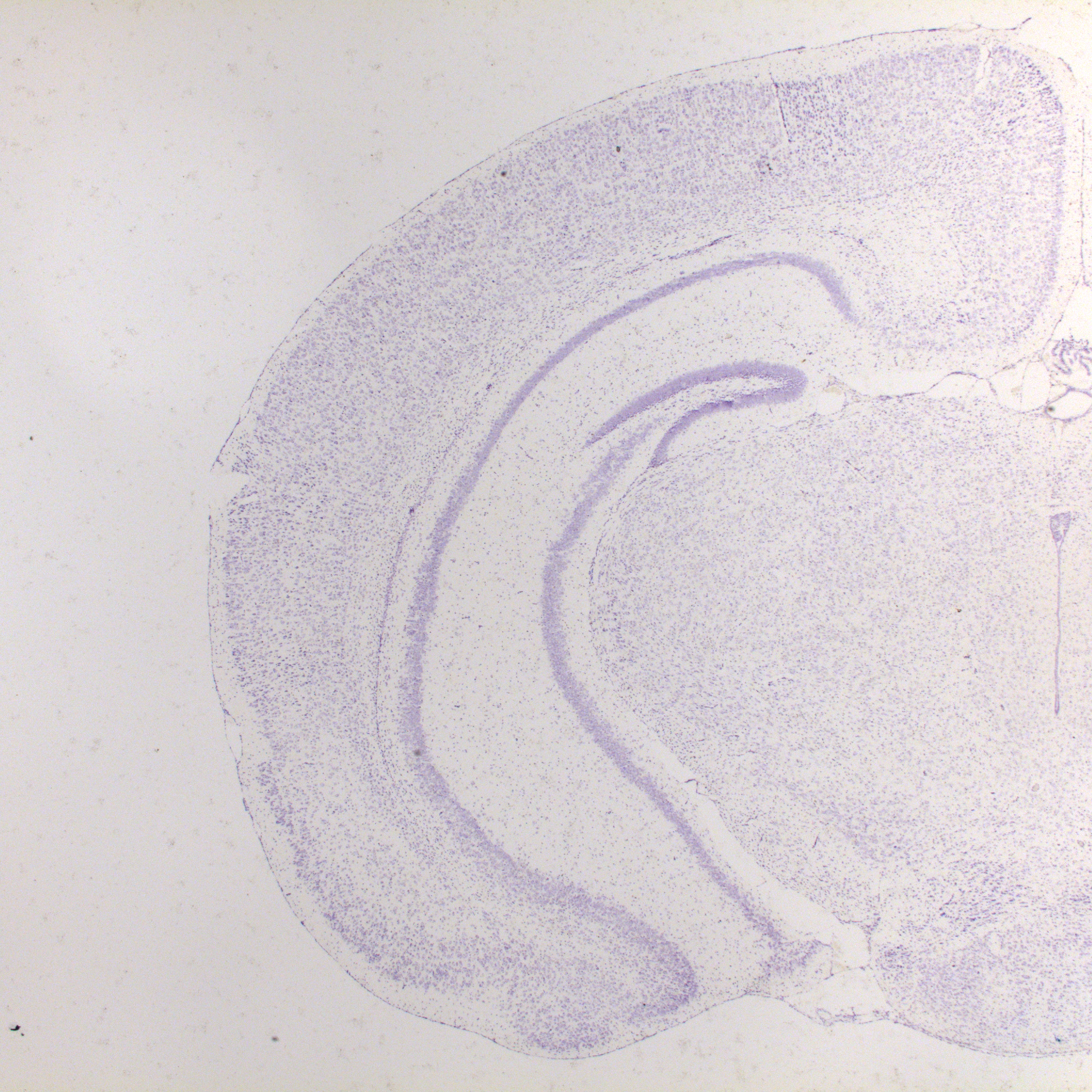

Supplement: Figure 3—source data 1. [file elife-86940-fig3-data1.zip › Figure 3-source data 1/F8099-3-CON-FF ff-P20-2.5X-132-2-L-Image Export-12_Bright.tif]

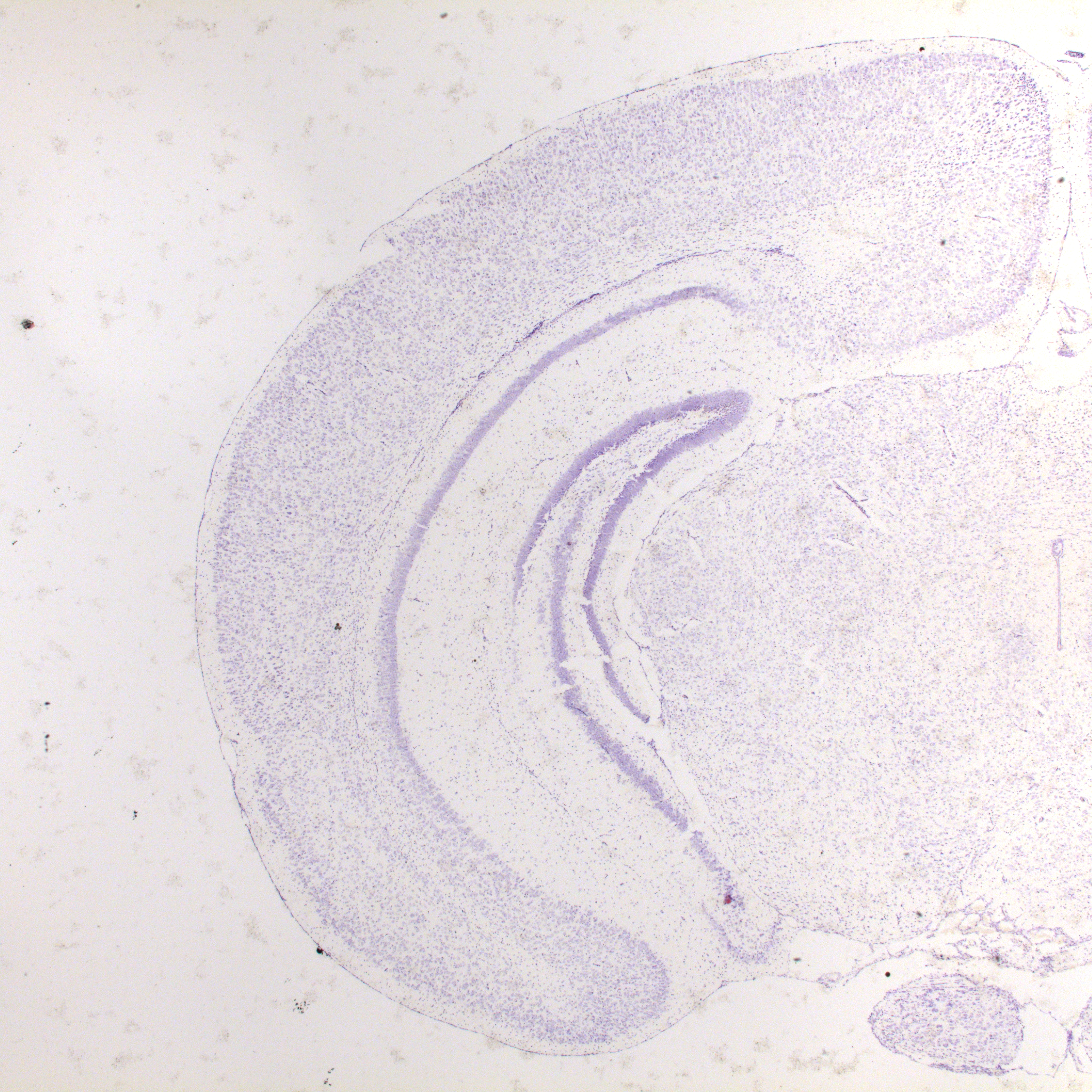

Supplement: Figure 3—source data 1. [file elife-86940-fig3-data1.zip › Figure 3-source data 1/F8099-3-CON-FF ff-P20-2.5X-140-3-L-Image Export-14_Bright.tif]

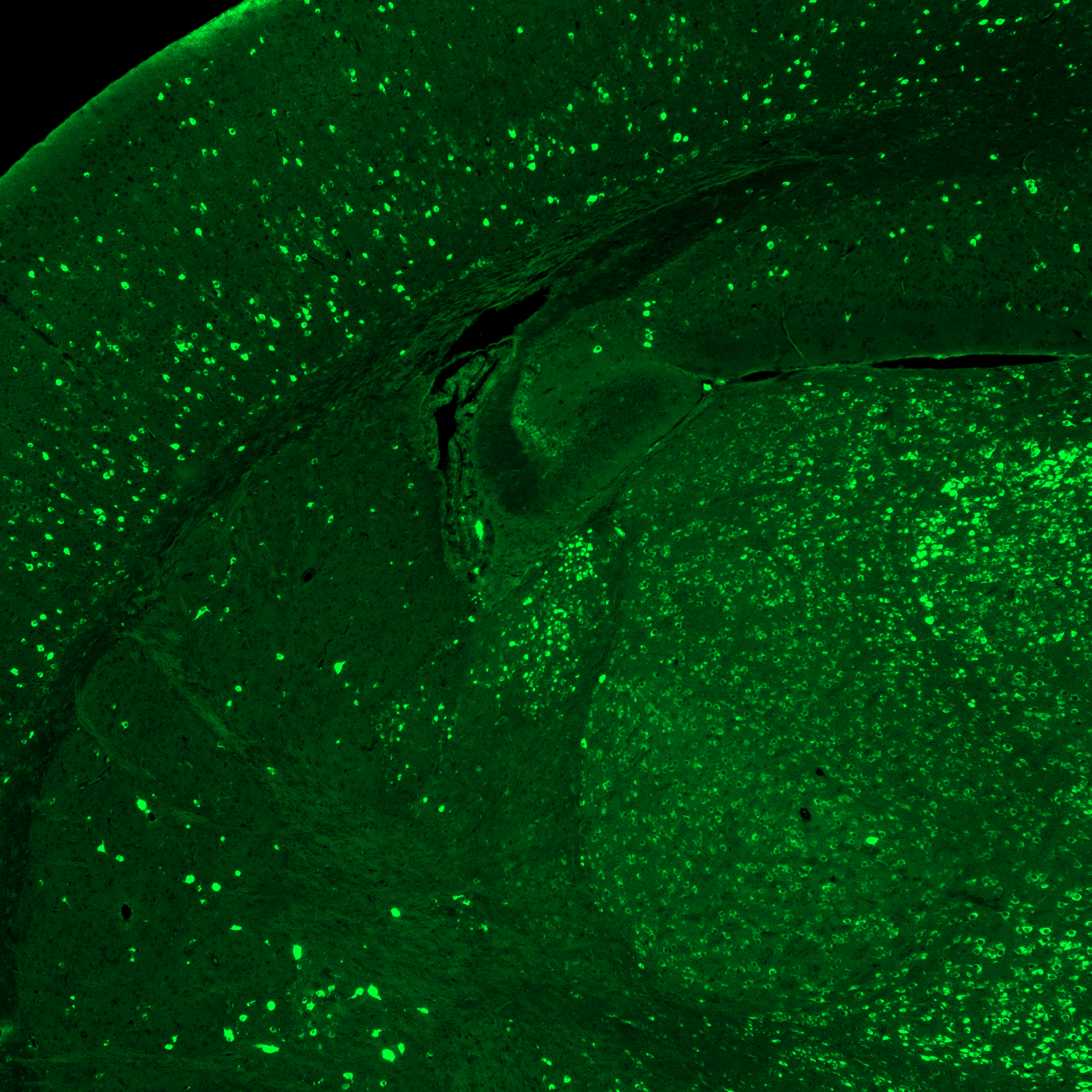

Supplement: Figure 3—source data 2. [file elife-86940-fig3-data2.zip › Figure 3-source data 2/F449-1-DKO-RX FF ff-P18-HUB-CTIP2-115#-1-5X-left dHPC-Image Export-25_AF488.tif]

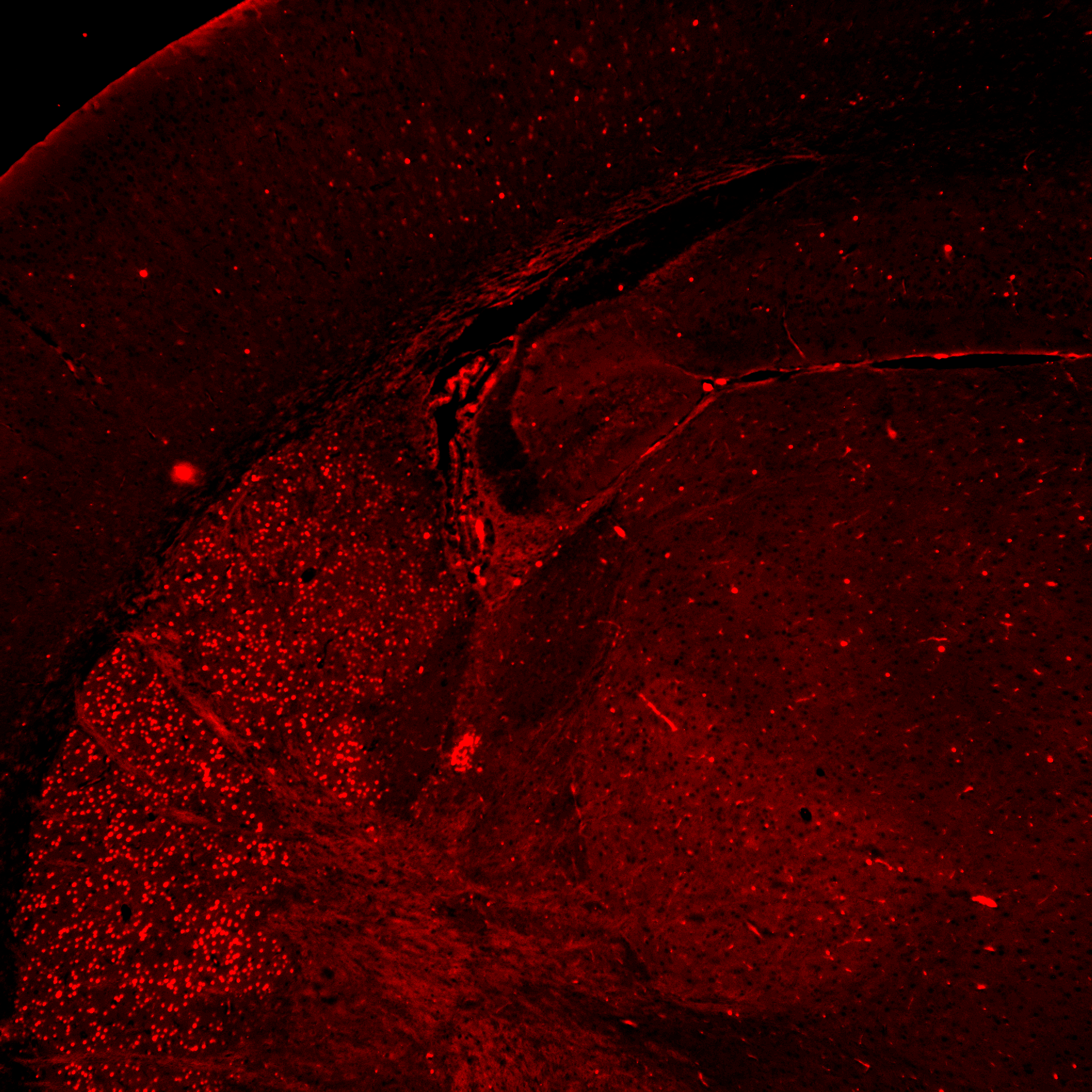

Supplement: Figure 3—source data 2. [file elife-86940-fig3-data2.zip › Figure 3-source data 2/F449-1-DKO-RX FF ff-P18-HUB-CTIP2-115#-1-5X-left dHPC-Image Export-25_AF594.tif]

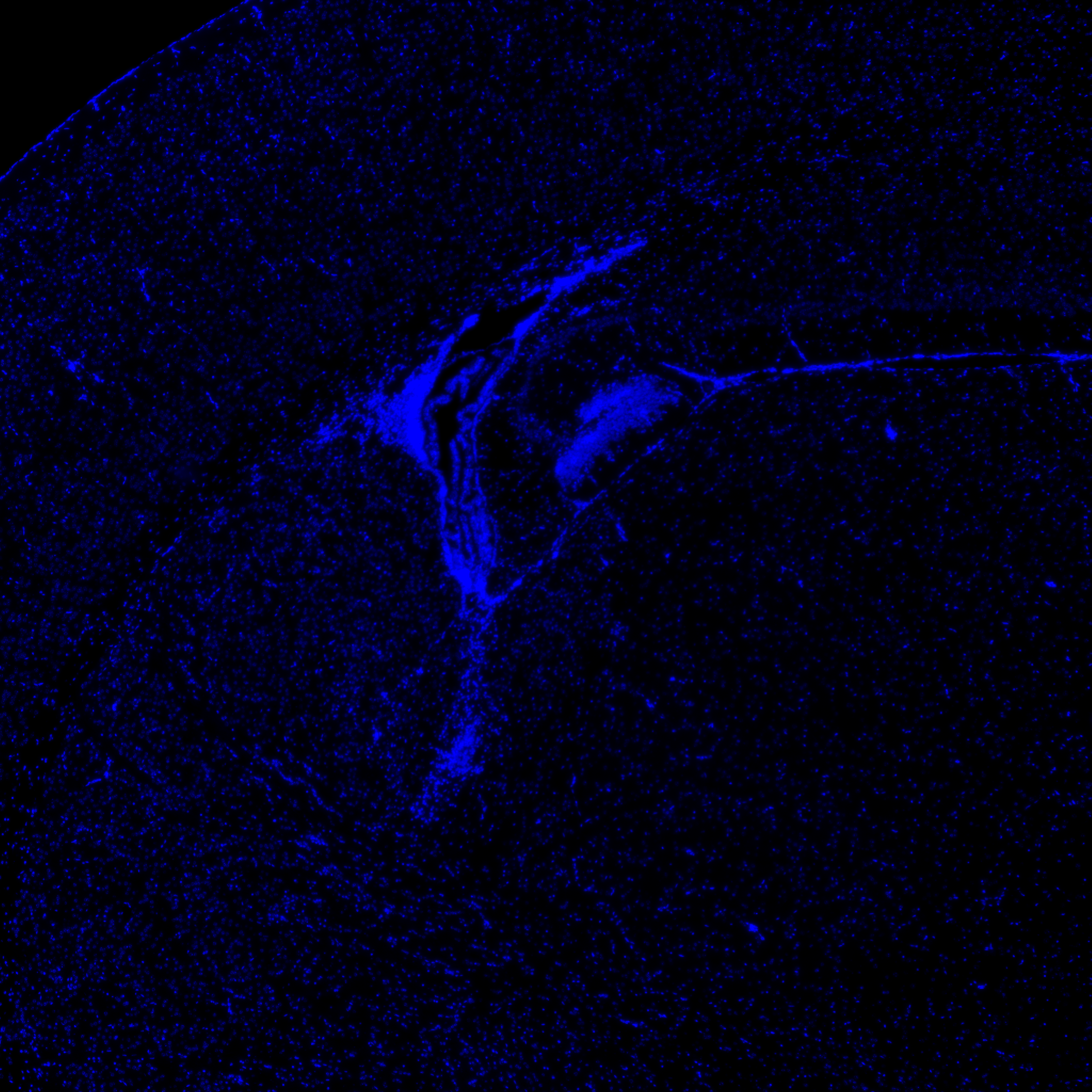

Supplement: Figure 3—source data 2. [file elife-86940-fig3-data2.zip › Figure 3-source data 2/F449-1-DKO-RX FF ff-P18-HUB-CTIP2-115#-1-5X-left dHPC-Image Export-25_DAPI.tif]

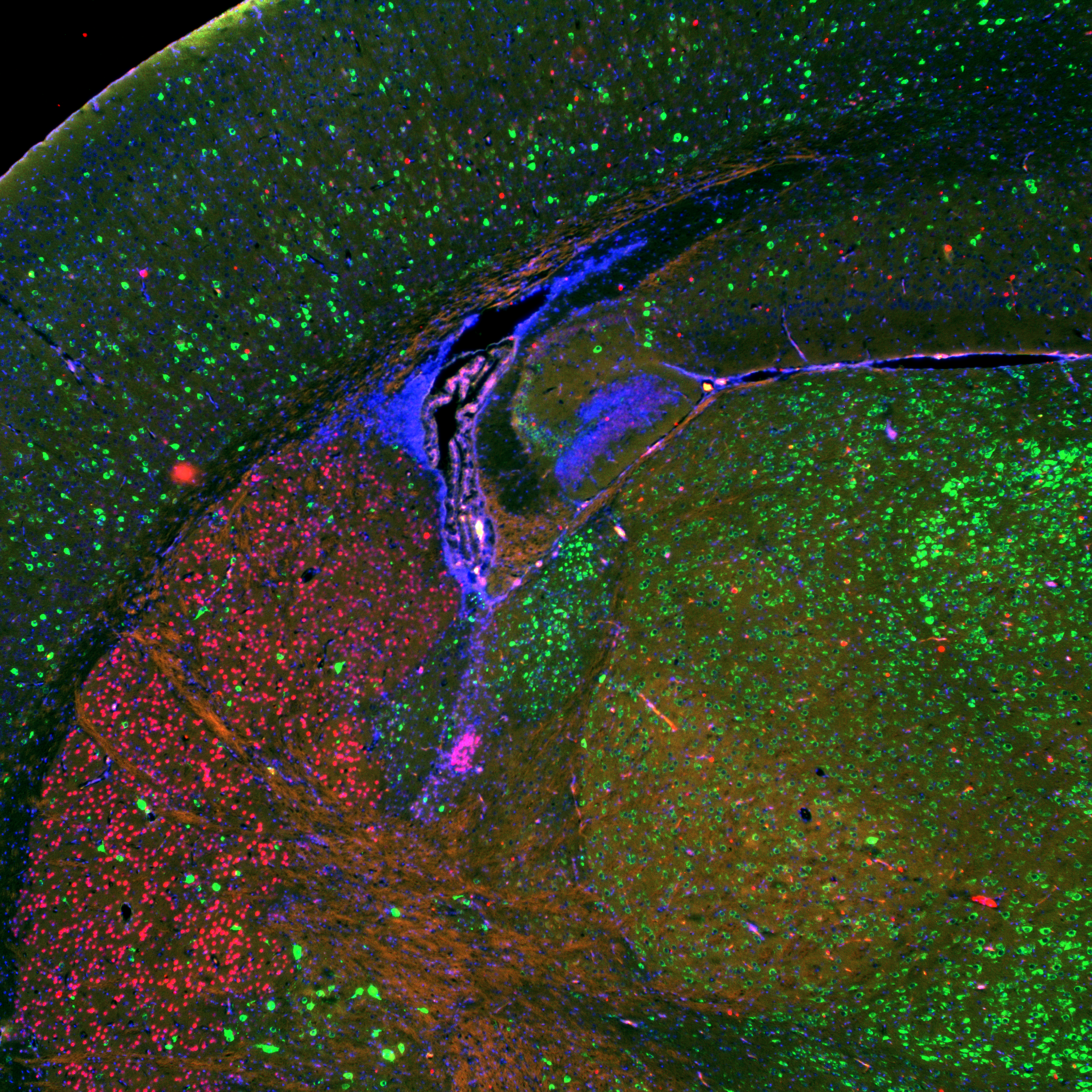

Supplement: Figure 3—source data 2. [file elife-86940-fig3-data2.zip › Figure 3-source data 2/F449-1-DKO-RX FF ff-P18-HUB-CTIP2-115#-1-5X-left dHPC-Image Export-25.tif]

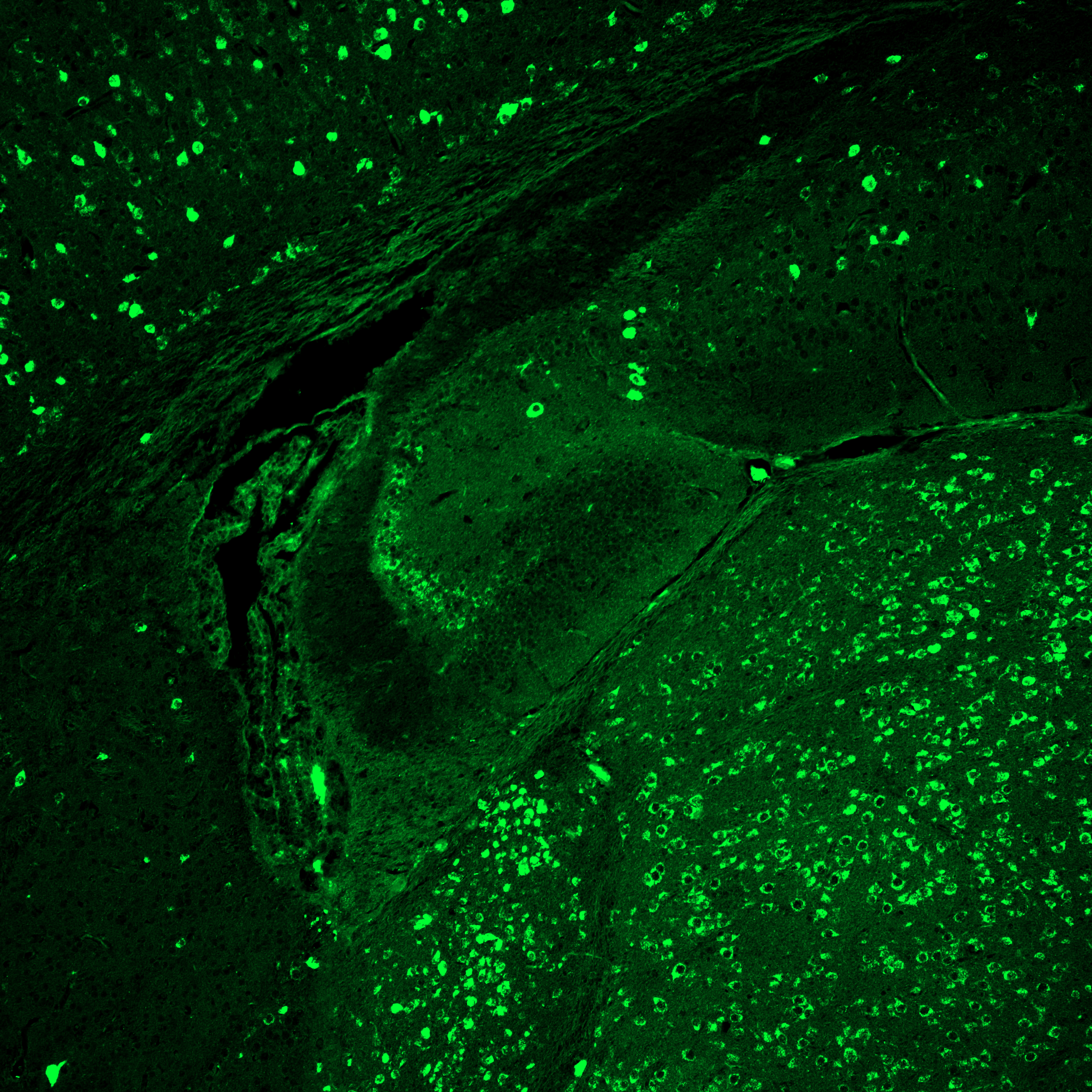

Supplement: Figure 3—source data 2. [file elife-86940-fig3-data2.zip › Figure 3-source data 2/F449-1-DKO-RX FF ff-P18-HUB-CTIP2-115#-1-10X-left dHPC-Image Export-27_AF488.tif]

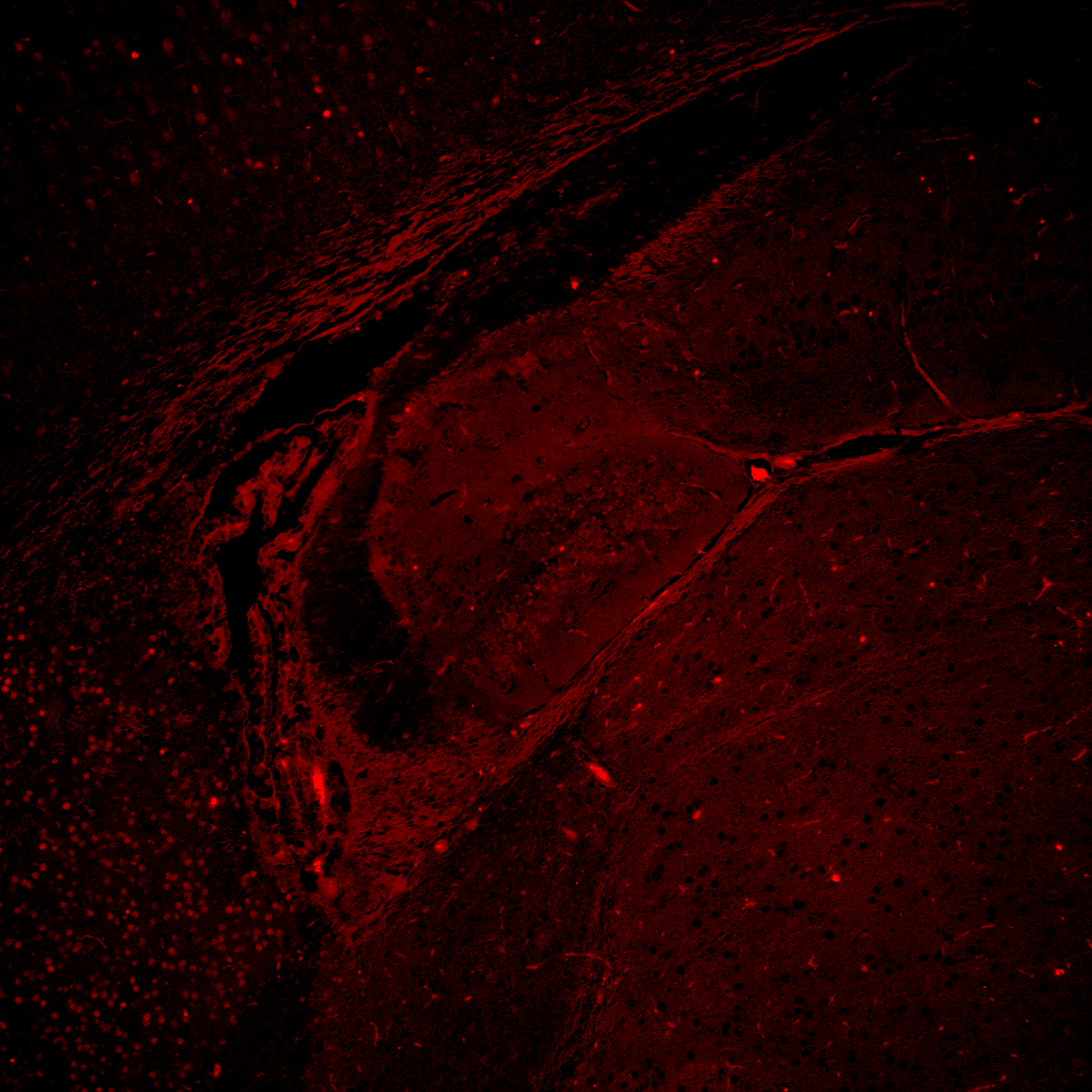

Supplement: Figure 3—source data 2. [file elife-86940-fig3-data2.zip › Figure 3-source data 2/F449-1-DKO-RX FF ff-P18-HUB-CTIP2-115#-1-10X-left dHPC-Image Export-27_AF594.tif]

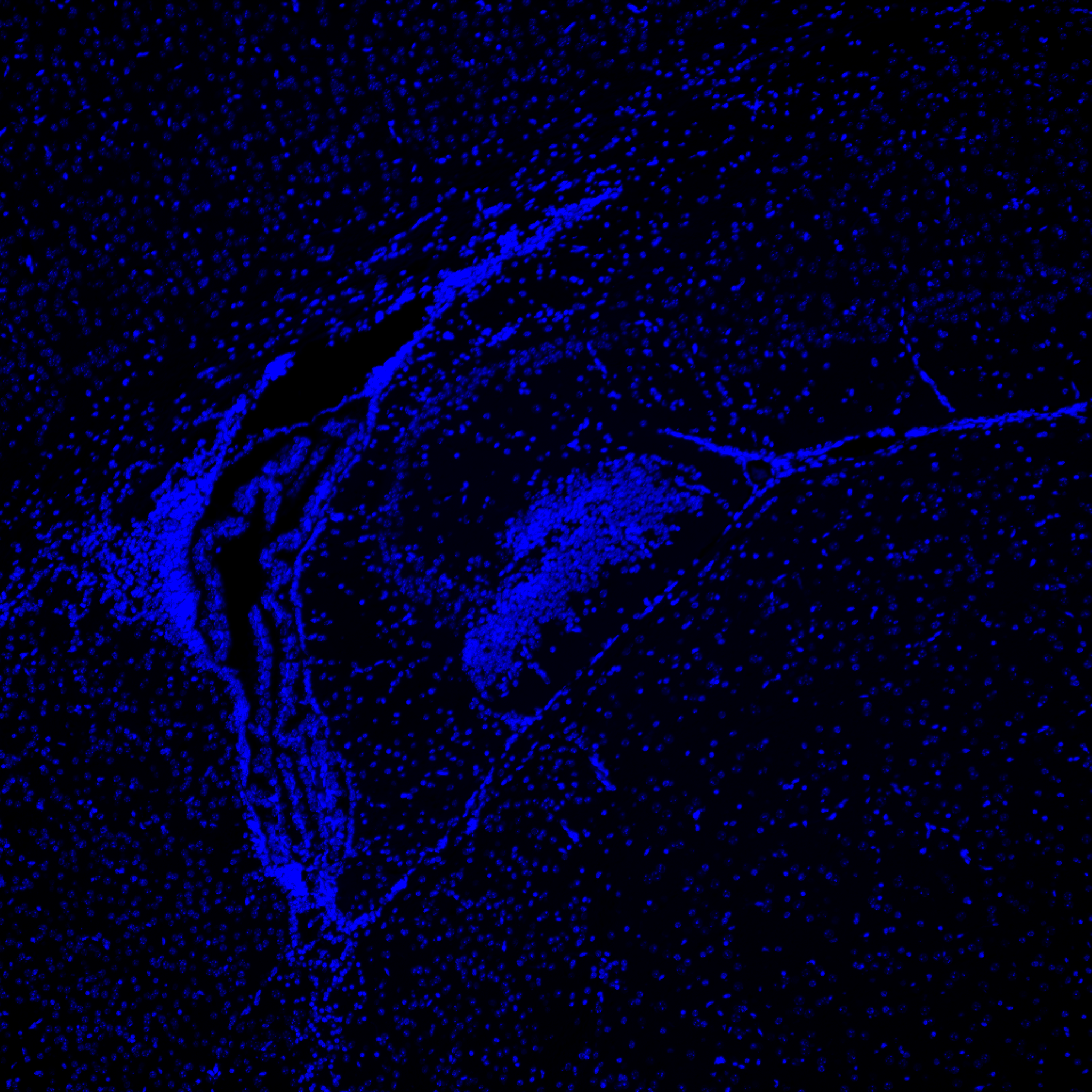

Supplement: Figure 3—source data 2. [file elife-86940-fig3-data2.zip › Figure 3-source data 2/F449-1-DKO-RX FF ff-P18-HUB-CTIP2-115#-1-10X-left dHPC-Image Export-27_DAPI.tif]

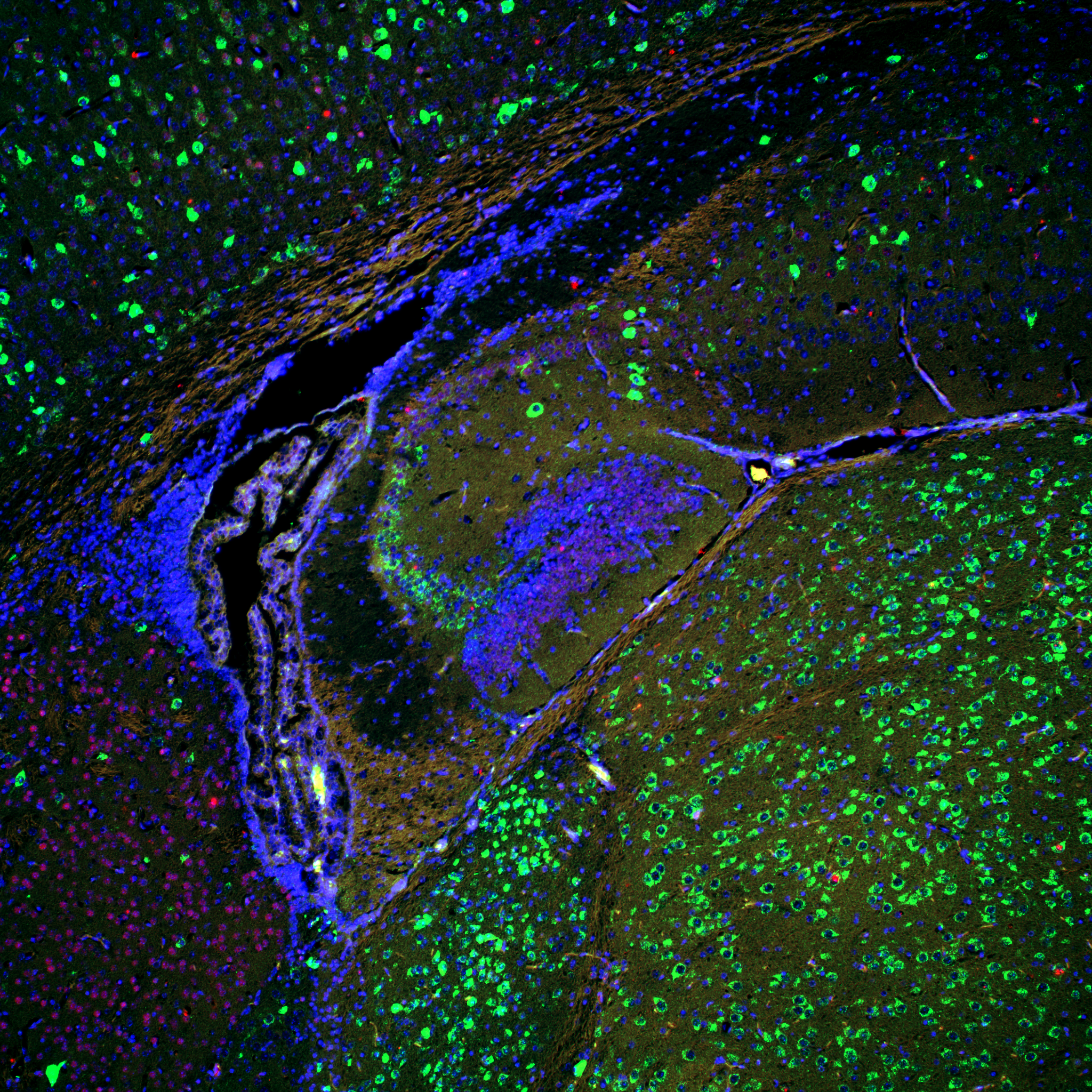

Supplement: Figure 3—source data 2. [file elife-86940-fig3-data2.zip › Figure 3-source data 2/F449-1-DKO-RX FF ff-P18-HUB-CTIP2-115#-1-10X-left dHPC-Image Export-27.tif]

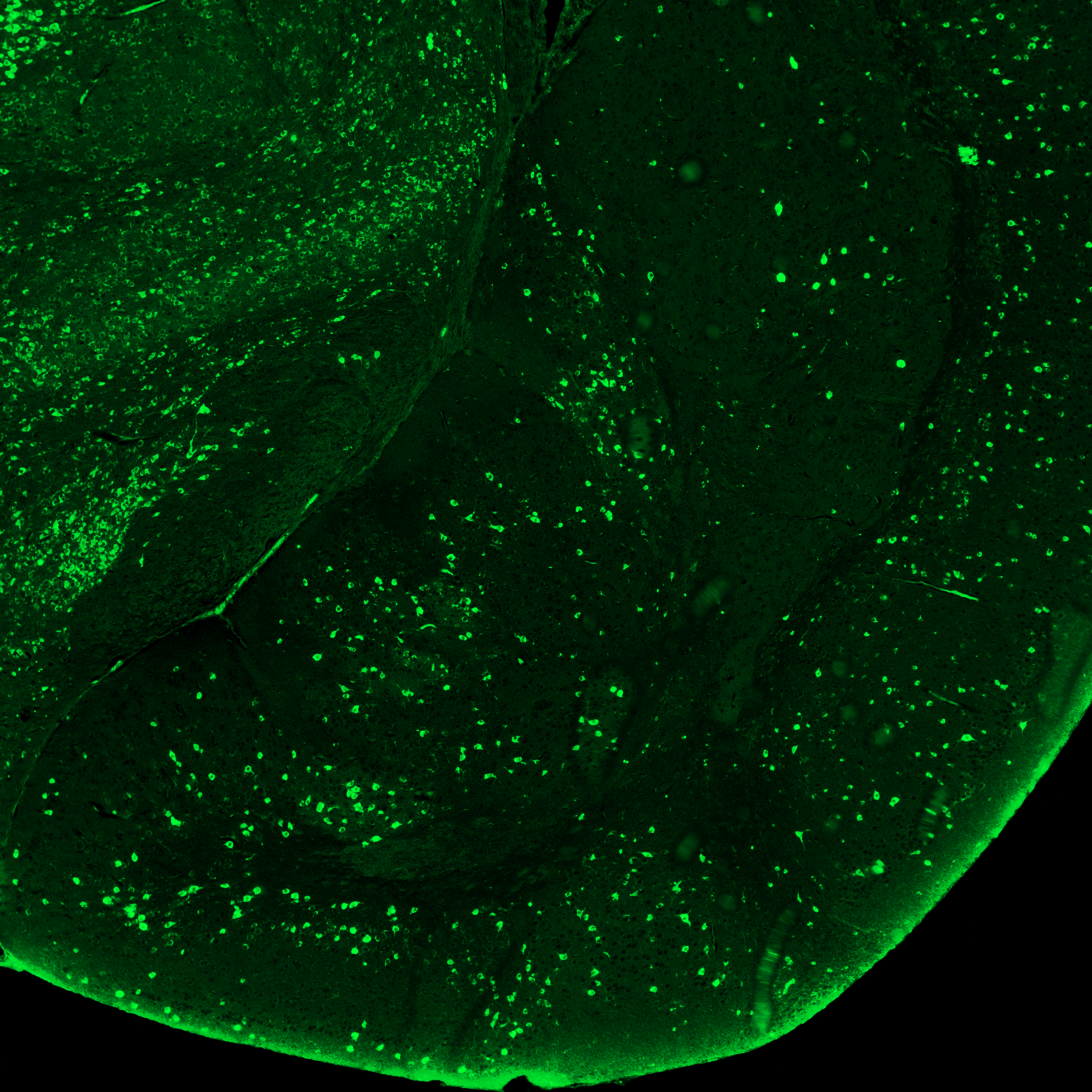

Supplement: Figure 3—source data 2. [file elife-86940-fig3-data2.zip › Figure 3-source data 2/F449-1-DKO-RX FF ff-P18-HUB-CTIP2-125#-1-5X-right vHPC-Image Export-31_AF488.tif]

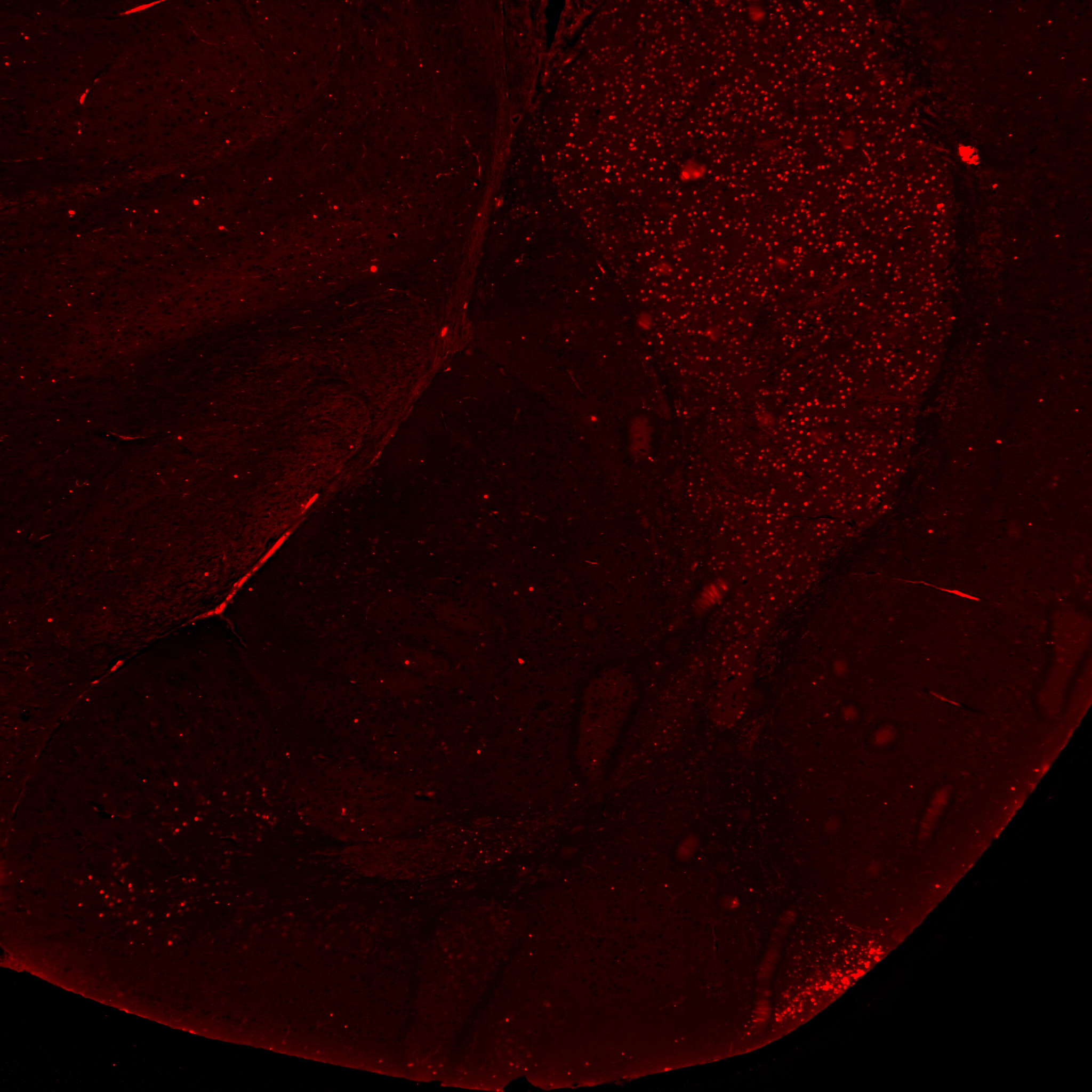

Supplement: Figure 3—source data 2. [file elife-86940-fig3-data2.zip › Figure 3-source data 2/F449-1-DKO-RX FF ff-P18-HUB-CTIP2-125#-1-5X-right vHPC-Image Export-31_AF594.tif]

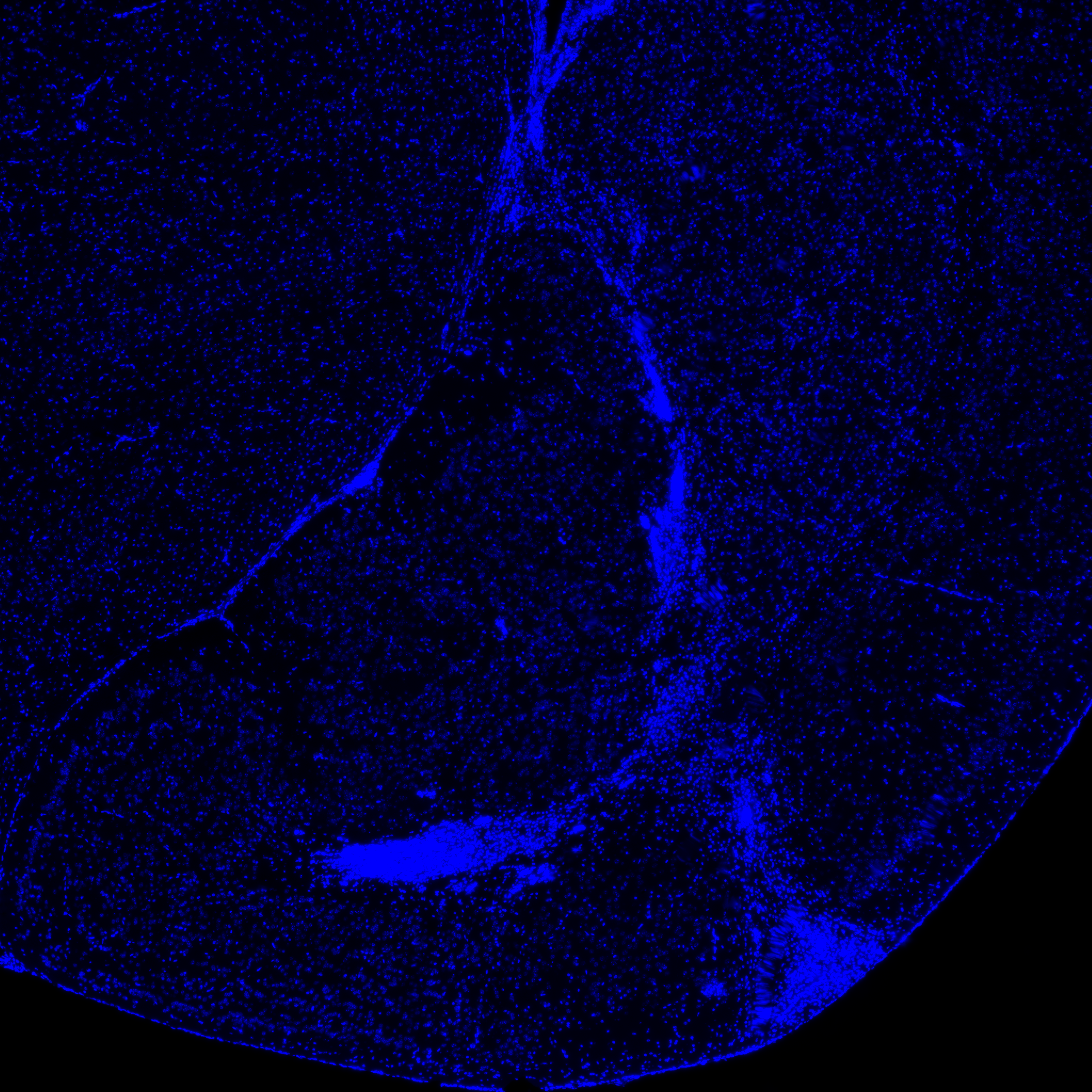

Supplement: Figure 3—source data 2. [file elife-86940-fig3-data2.zip › Figure 3-source data 2/F449-1-DKO-RX FF ff-P18-HUB-CTIP2-125#-1-5X-right vHPC-Image Export-31_DAPI.tif]

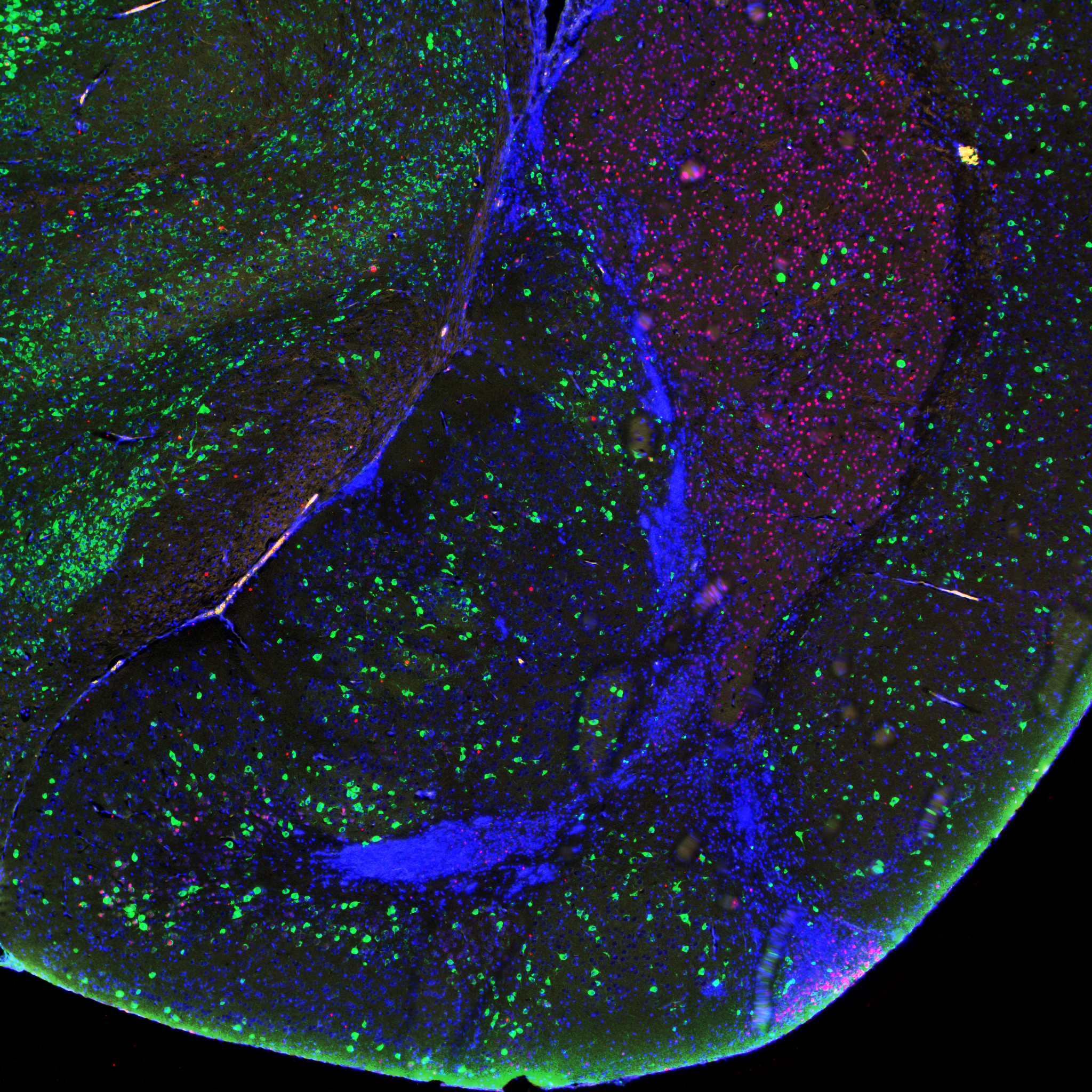

Supplement: Figure 3—source data 2. [file elife-86940-fig3-data2.zip › Figure 3-source data 2/F449-1-DKO-RX FF ff-P18-HUB-CTIP2-125#-1-5X-right vHPC-Image Export-31.tif]

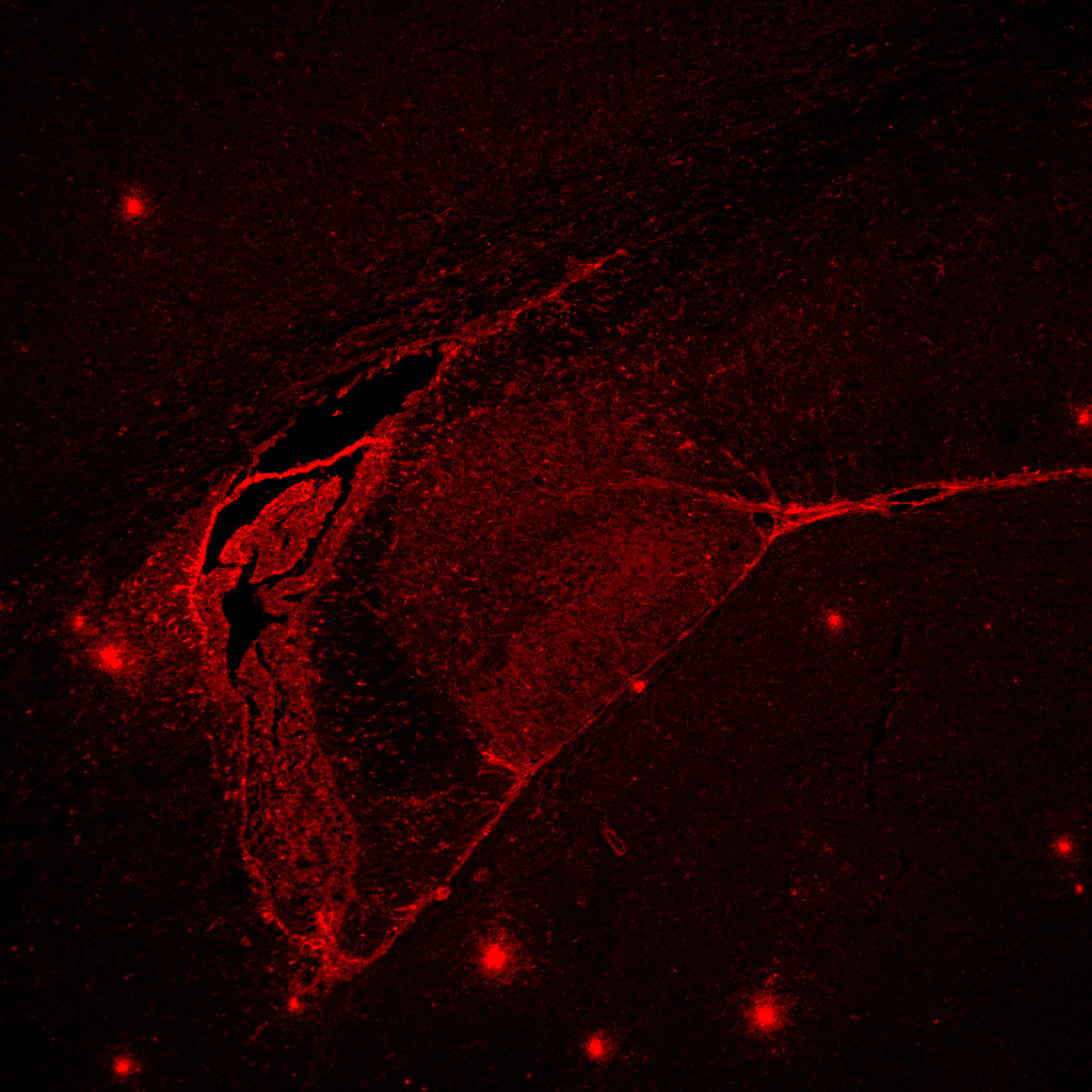

Supplement: Figure 3—source data 3. [file elife-86940-fig3-data3.zip › Figure 3-source data 3/F449-1-DKO-RX FF ff-P18-HUB-PROX1-115#-3-10X-left dHPC-Image Export-14_AF594.tif]

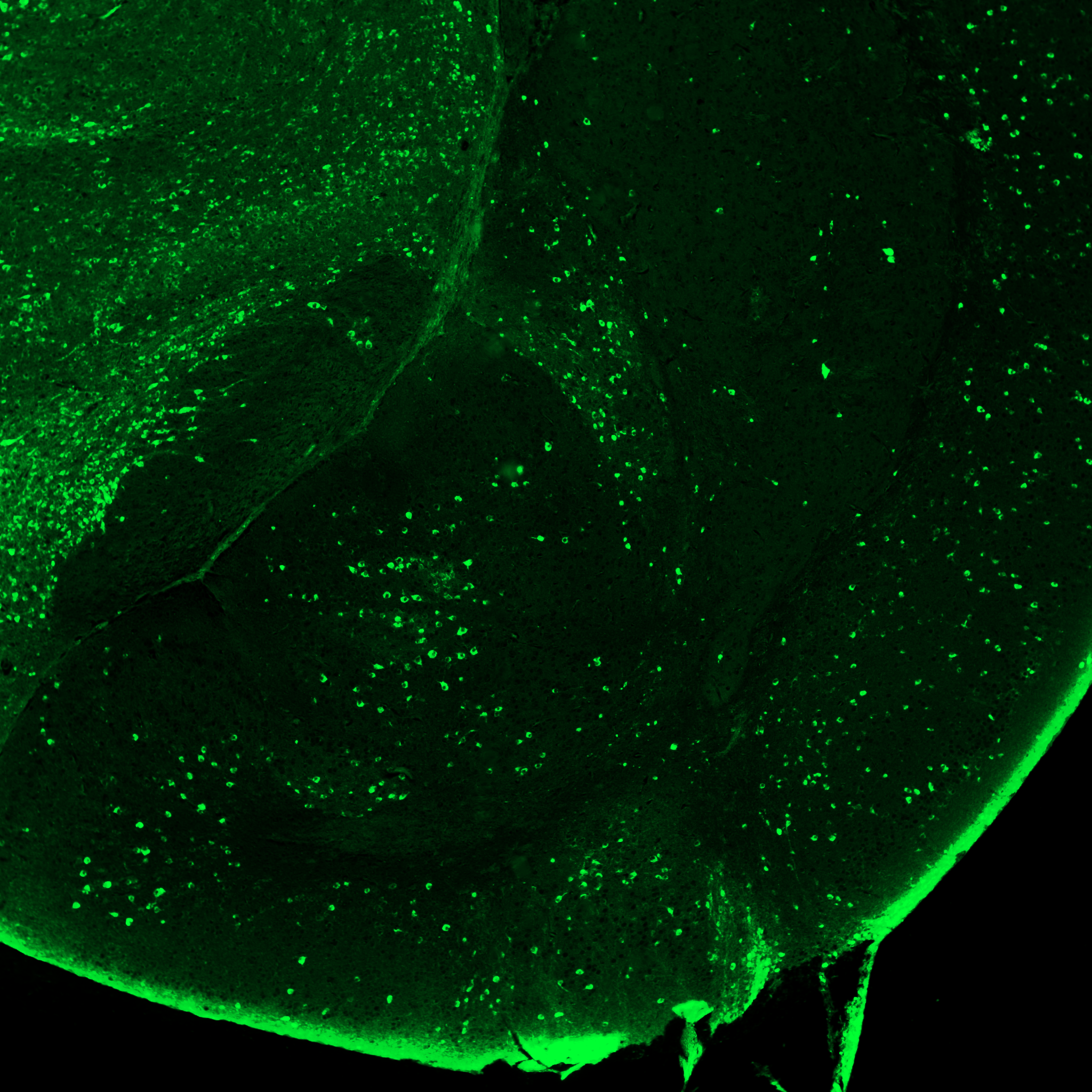

Supplement: Figure 3—source data 3. [file elife-86940-fig3-data3.zip › Figure 3-source data 3/F449-1-DKO-RX FF ff-P18-HUB-PROX1-125#-3-5X-right vHPC-Image Export-18_AF488.tif]

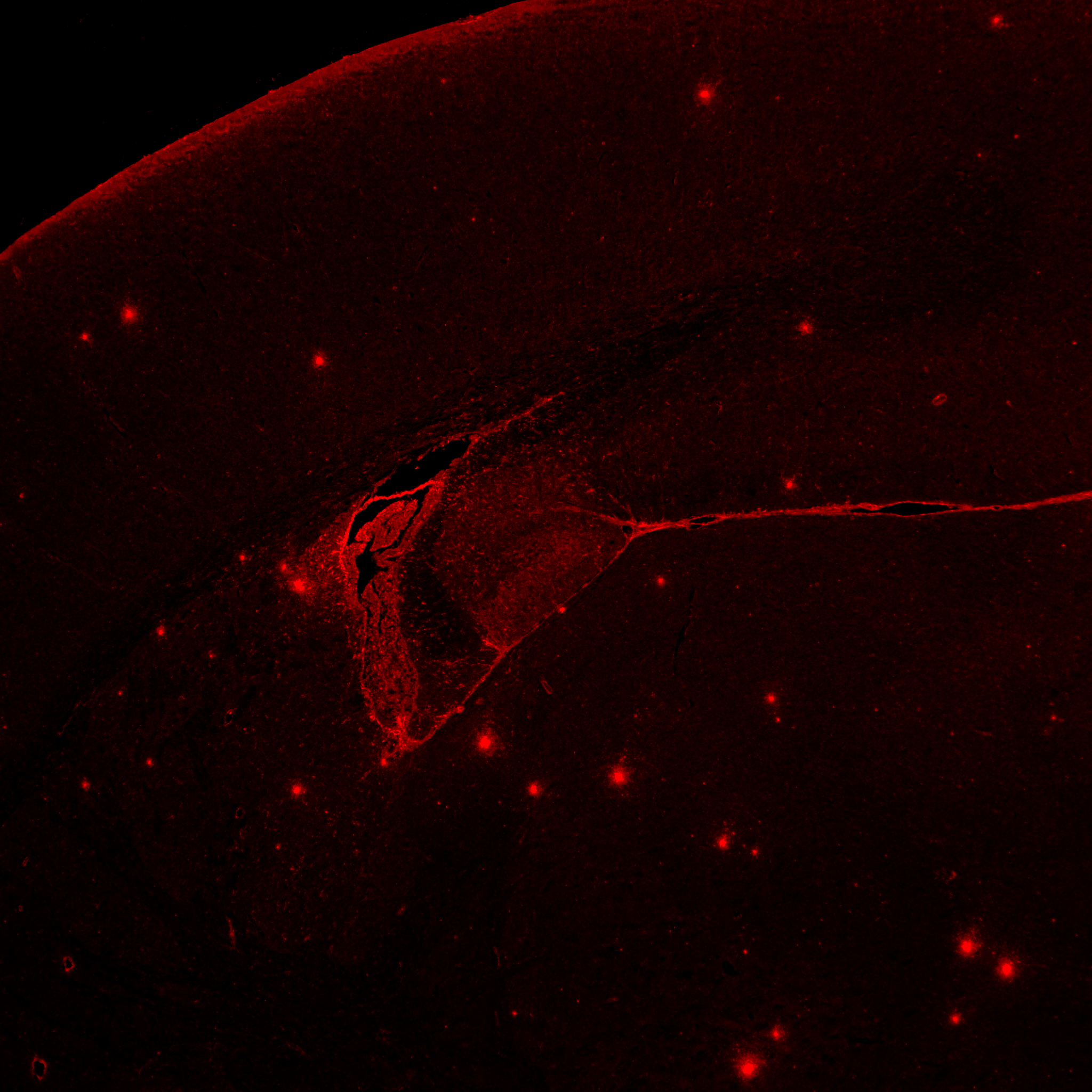

Supplement: Figure 3—source data 3. [file elife-86940-fig3-data3.zip › Figure 3-source data 3/F449-1-DKO-RX FF ff-P18-HUB-PROX1-115#-3-5X-left dHPC-Image Export-12_AF594.tif]

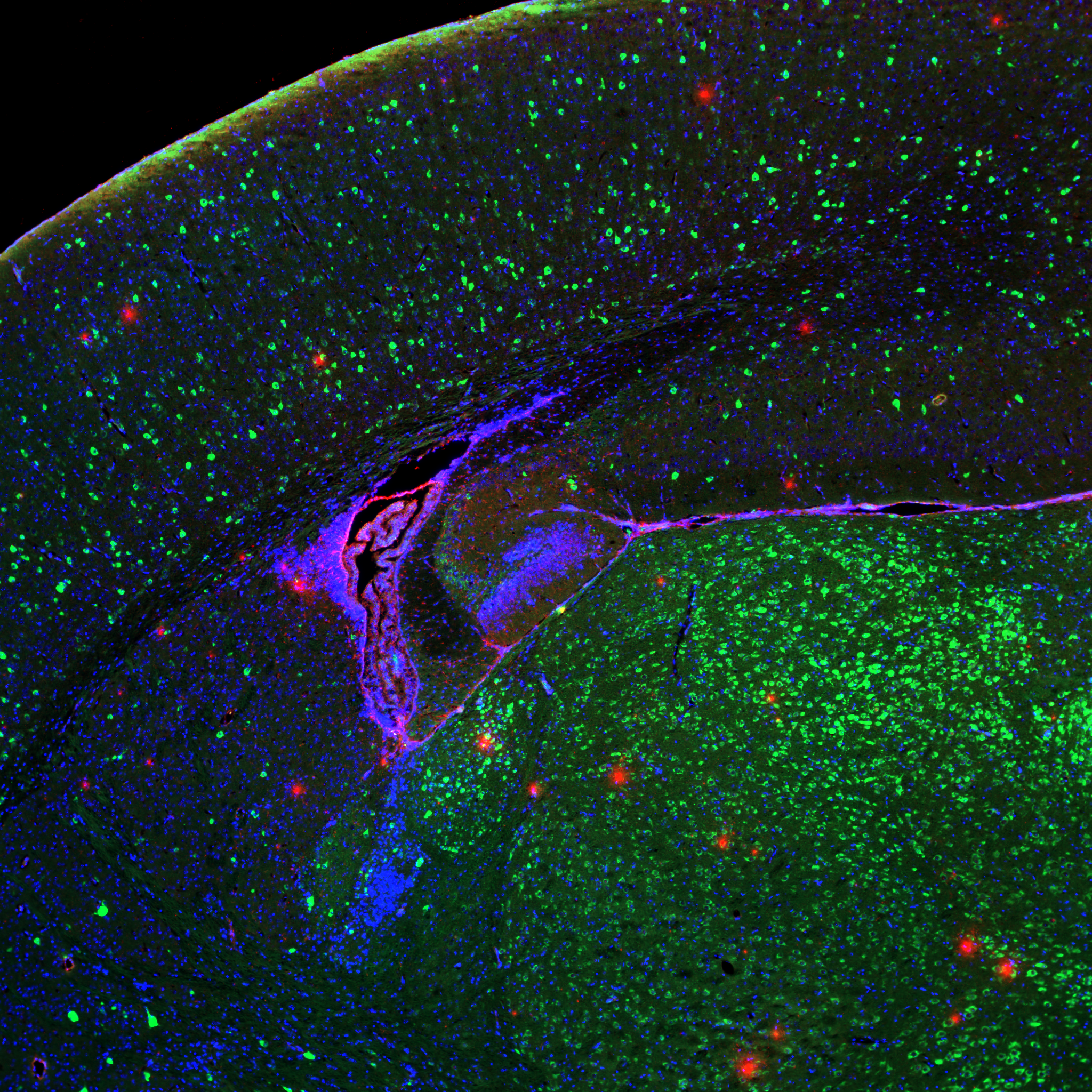

Supplement: Figure 3—source data 3. [file elife-86940-fig3-data3.zip › Figure 3-source data 3/F449-1-DKO-RX FF ff-P18-HUB-PROX1-115#-3-5X-left dHPC-Image Export-12.tif]

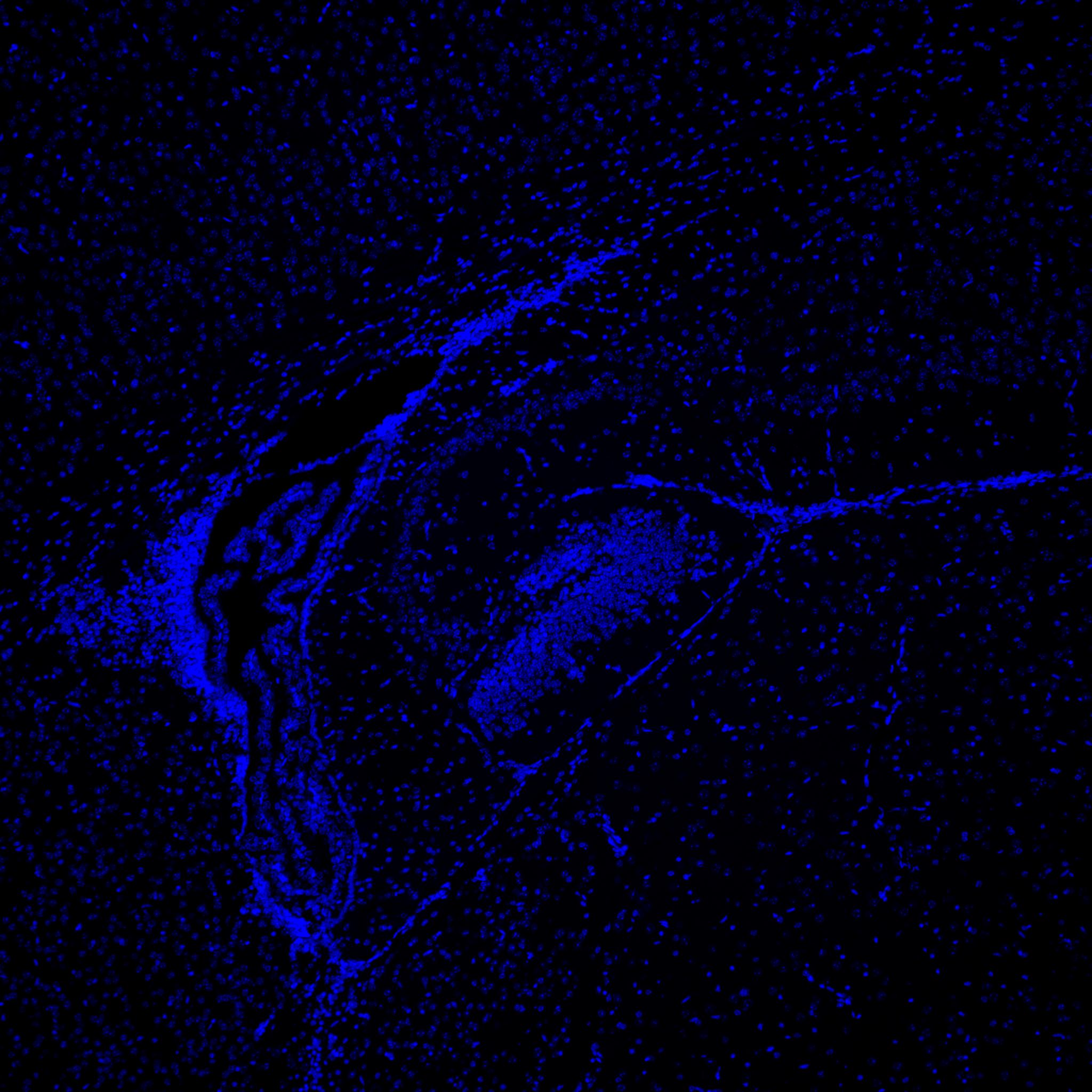

Supplement: Figure 3—source data 3. [file elife-86940-fig3-data3.zip › Figure 3-source data 3/F449-1-DKO-RX FF ff-P18-HUB-PROX1-115#-3-10X-left dHPC-Image Export-14_DAPI.tif]

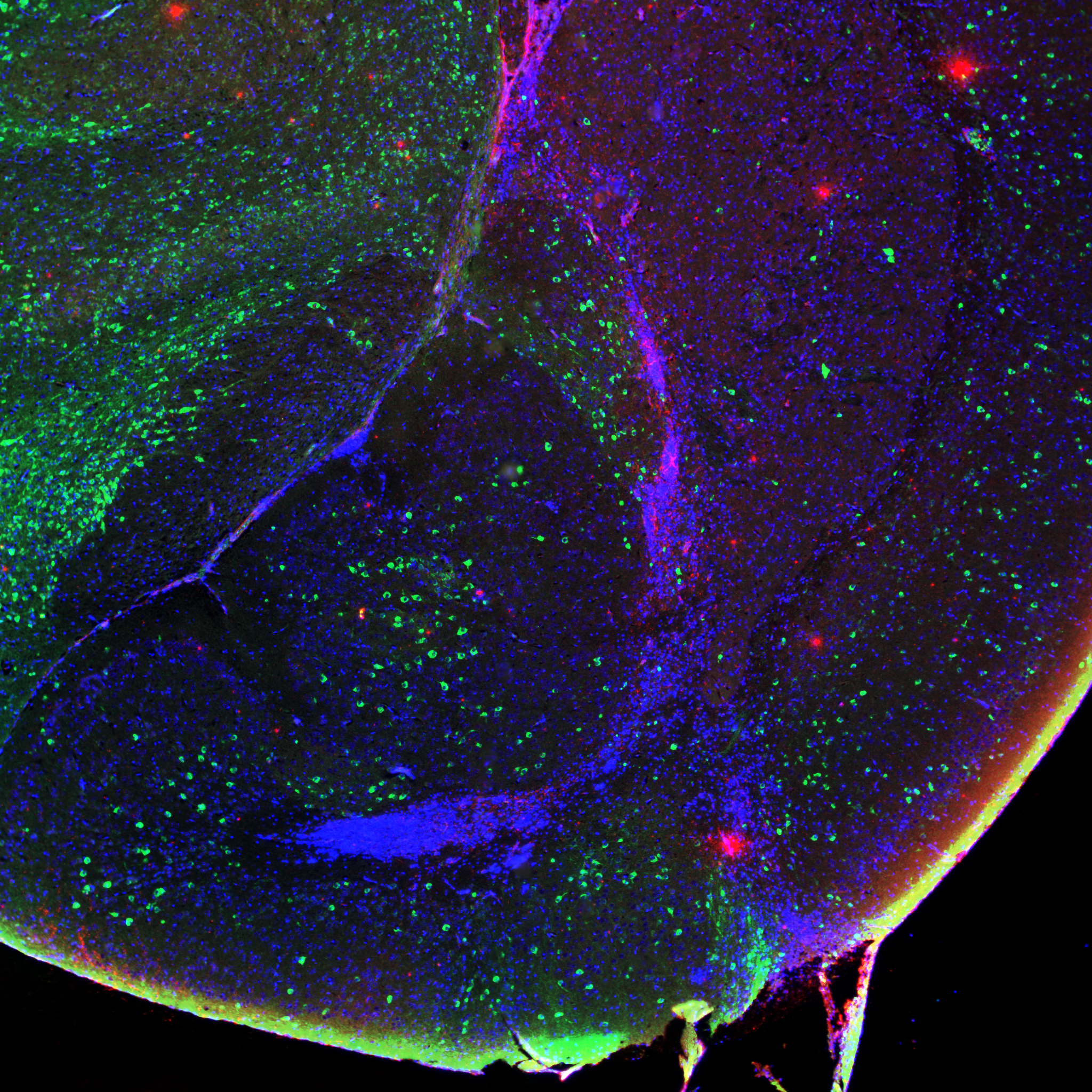

Supplement: Figure 3—source data 3. [file elife-86940-fig3-data3.zip › Figure 3-source data 3/F449-1-DKO-RX FF ff-P18-HUB-PROX1-125#-3-5X-right vHPC-Image Export-18.tif]

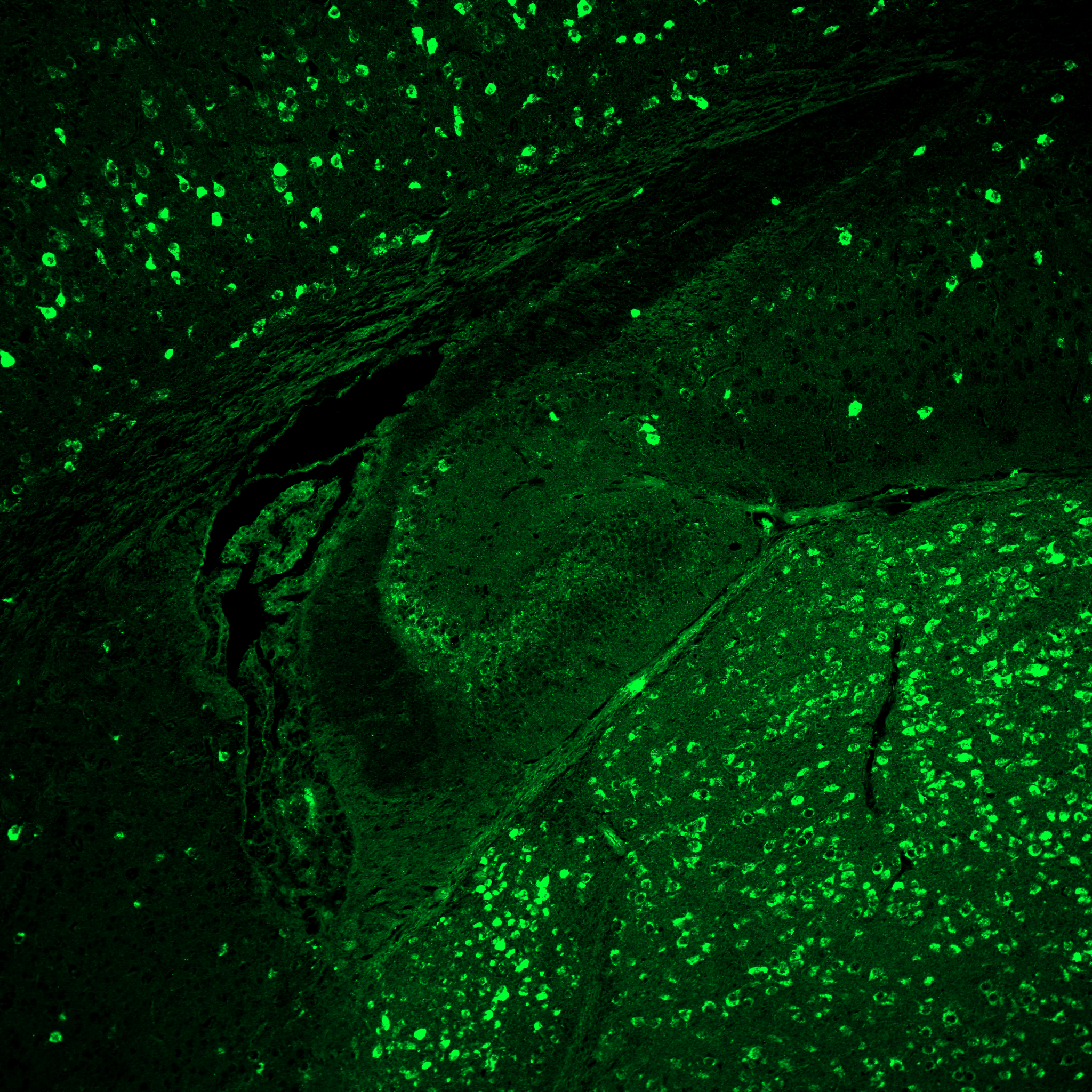

Supplement: Figure 3—source data 3. [file elife-86940-fig3-data3.zip › Figure 3-source data 3/F449-1-DKO-RX FF ff-P18-HUB-PROX1-115#-3-10X-left dHPC-Image Export-14_AF488.tif]

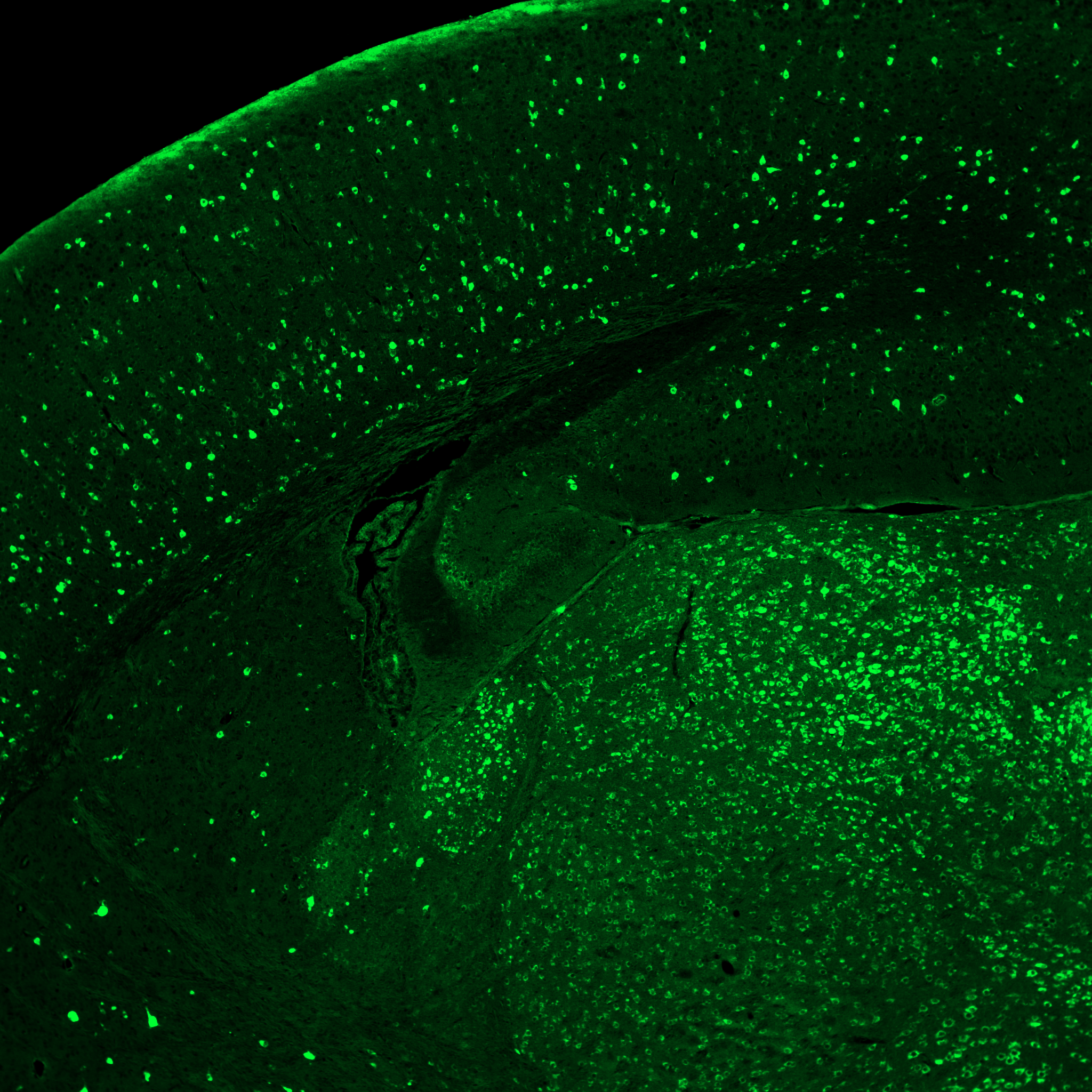

Supplement: Figure 3—source data 3. [file elife-86940-fig3-data3.zip › Figure 3-source data 3/F449-1-DKO-RX FF ff-P18-HUB-PROX1-115#-3-5X-left dHPC-Image Export-12_AF488.tif]

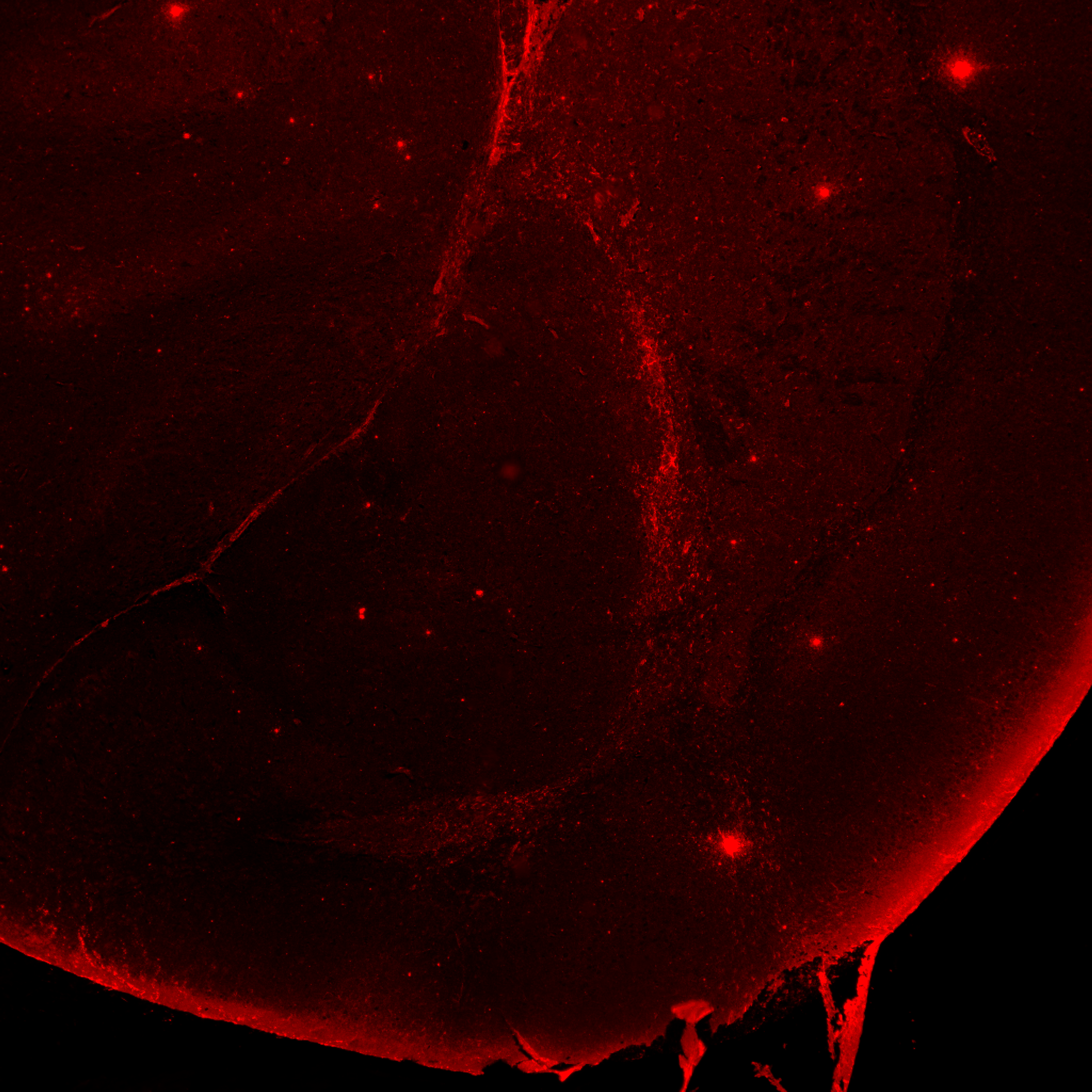

Supplement: Figure 3—source data 3. [file elife-86940-fig3-data3.zip › Figure 3-source data 3/F449-1-DKO-RX FF ff-P18-HUB-PROX1-125#-3-5X-right vHPC-Image Export-18_AF594.tif]

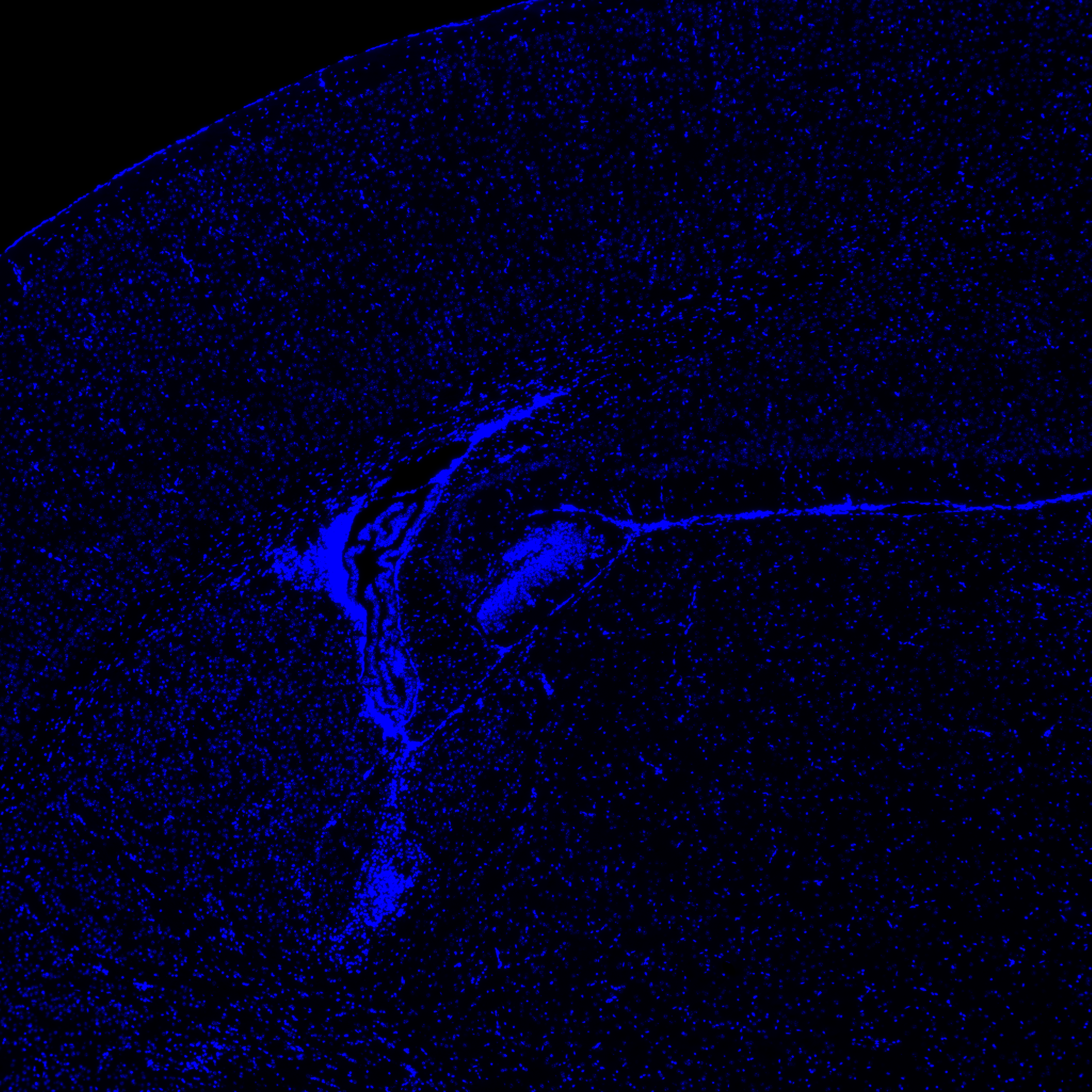

Supplement: Figure 3—source data 3. [file elife-86940-fig3-data3.zip › Figure 3-source data 3/F449-1-DKO-RX FF ff-P18-HUB-PROX1-115#-3-5X-left dHPC-Image Export-12_DAPI.tif]

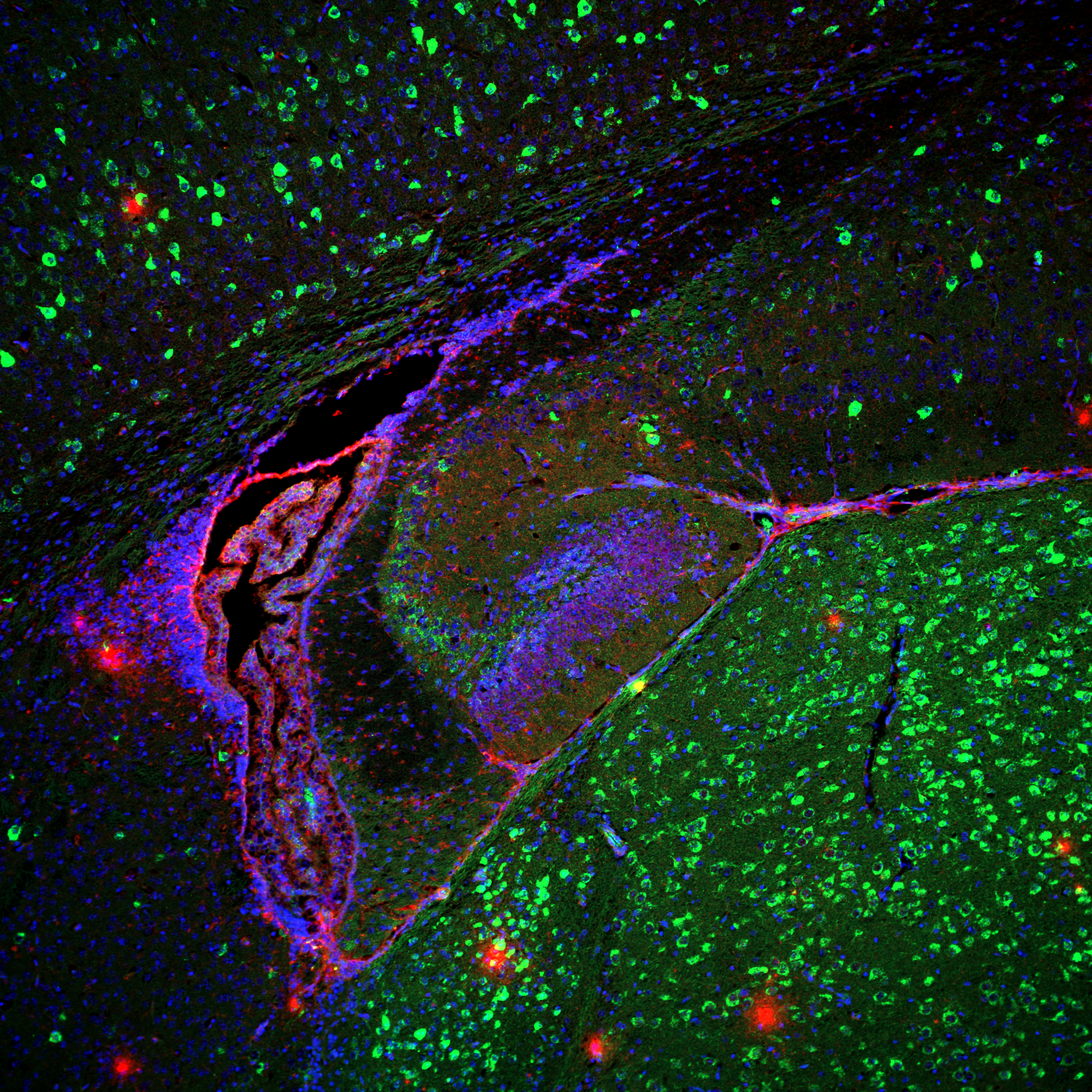

Supplement: Figure 3—source data 3. [file elife-86940-fig3-data3.zip › Figure 3-source data 3/F449-1-DKO-RX FF ff-P18-HUB-PROX1-115#-3-10X-left dHPC-Image Export-14.tif]

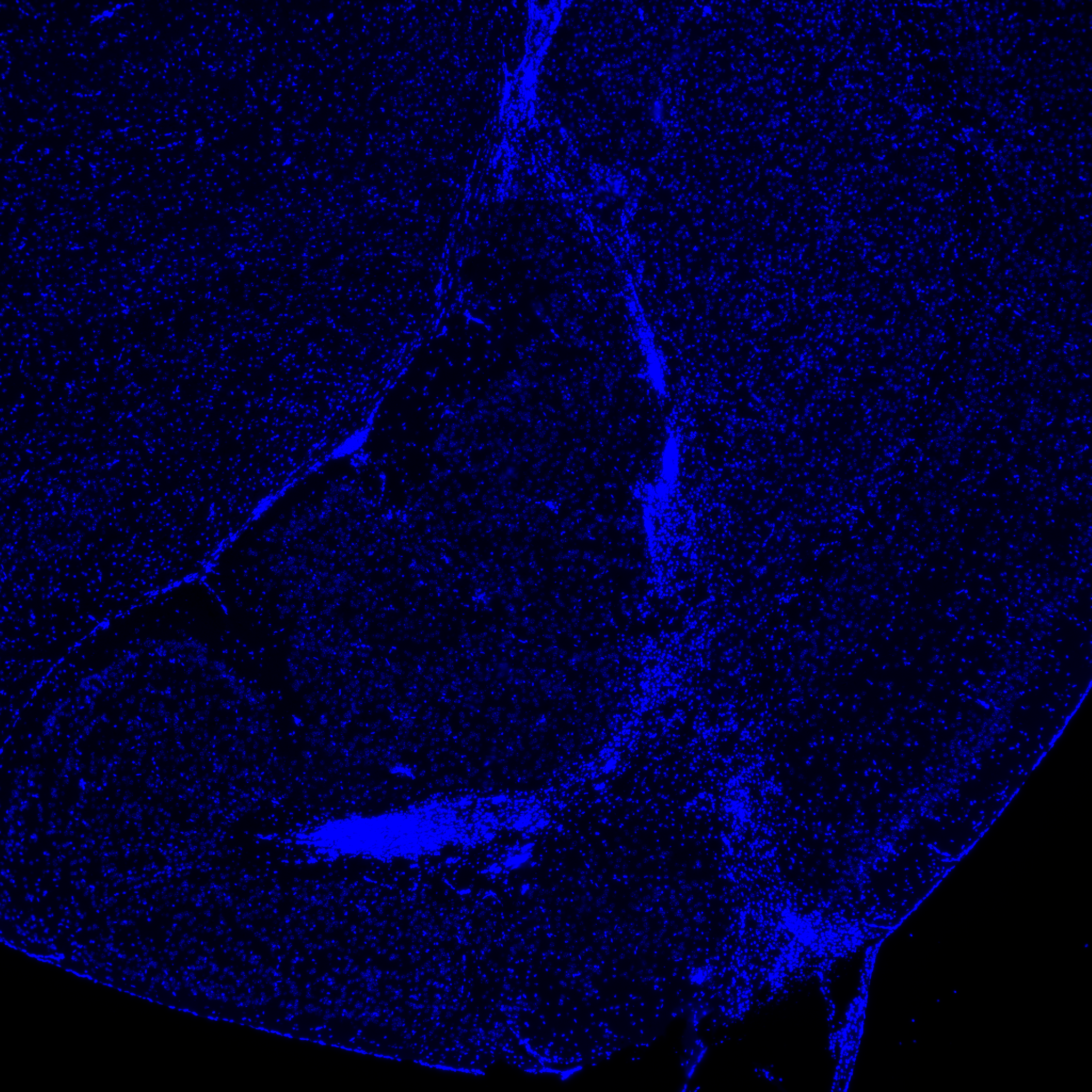

Supplement: Figure 3—source data 3. [file elife-86940-fig3-data3.zip › Figure 3-source data 3/F449-1-DKO-RX FF ff-P18-HUB-PROX1-125#-3-5X-right vHPC-Image Export-18_DAPI.tif]

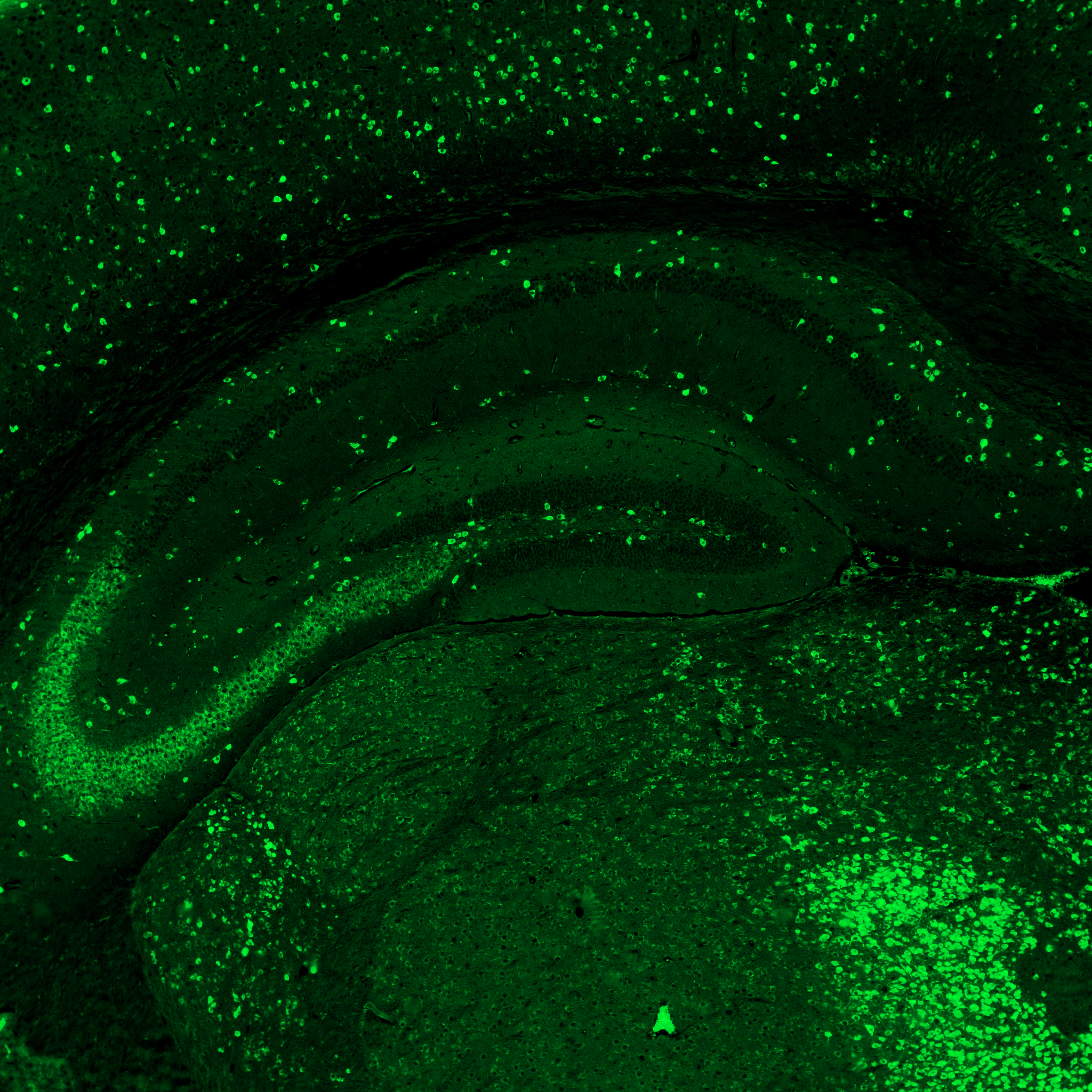

Supplement: Figure 3—source data 4. [file elife-86940-fig3-data4.zip › Figure 3-source data 4/F449-3-CON-F+ ff-P18-HUB-CTIP2-133#-1-5X-left dHPC-Image Export-04_AF488.tif]

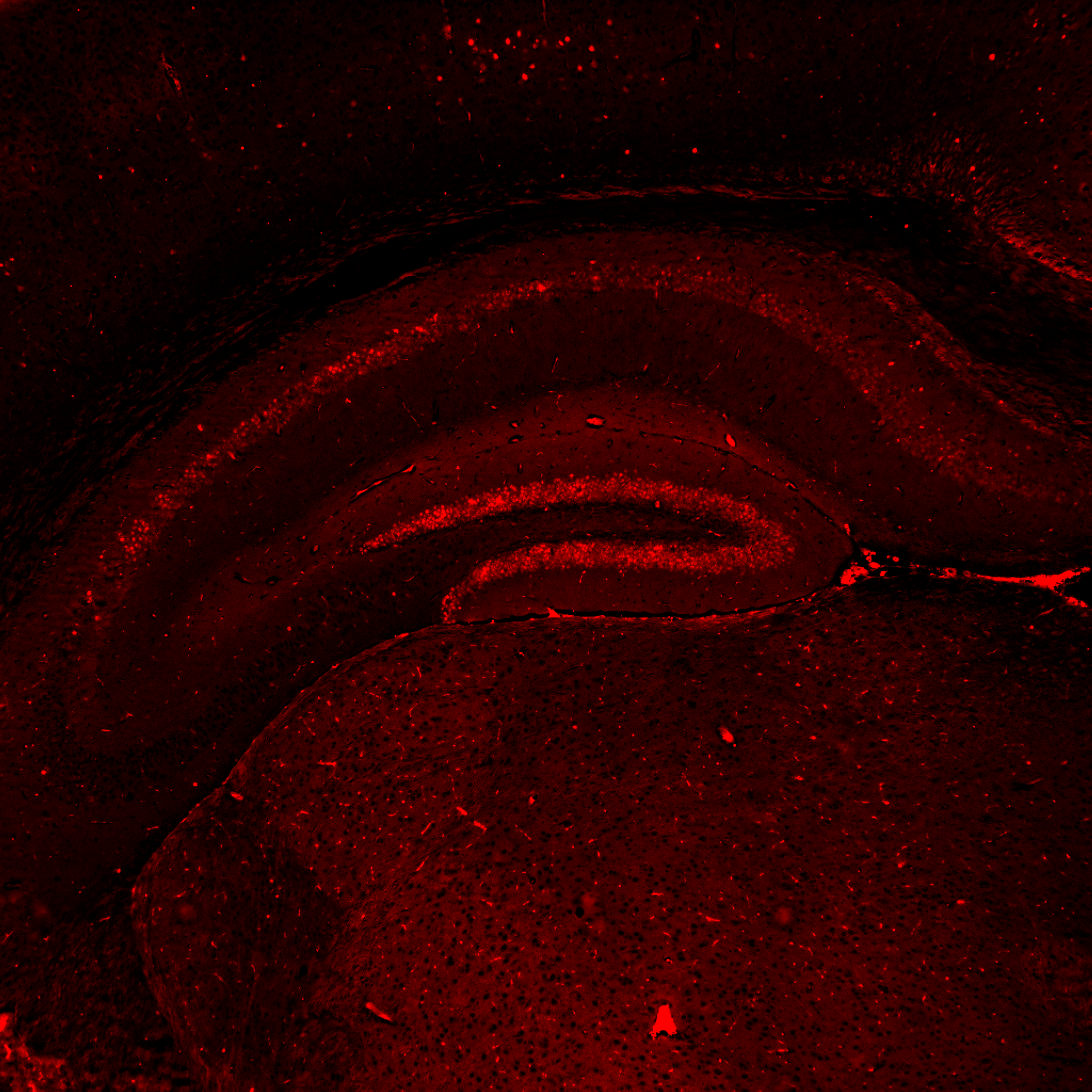

Supplement: Figure 3—source data 4. [file elife-86940-fig3-data4.zip › Figure 3-source data 4/F449-3-CON-F+ ff-P18-HUB-CTIP2-133#-1-5X-left dHPC-Image Export-04_AF594.tif]

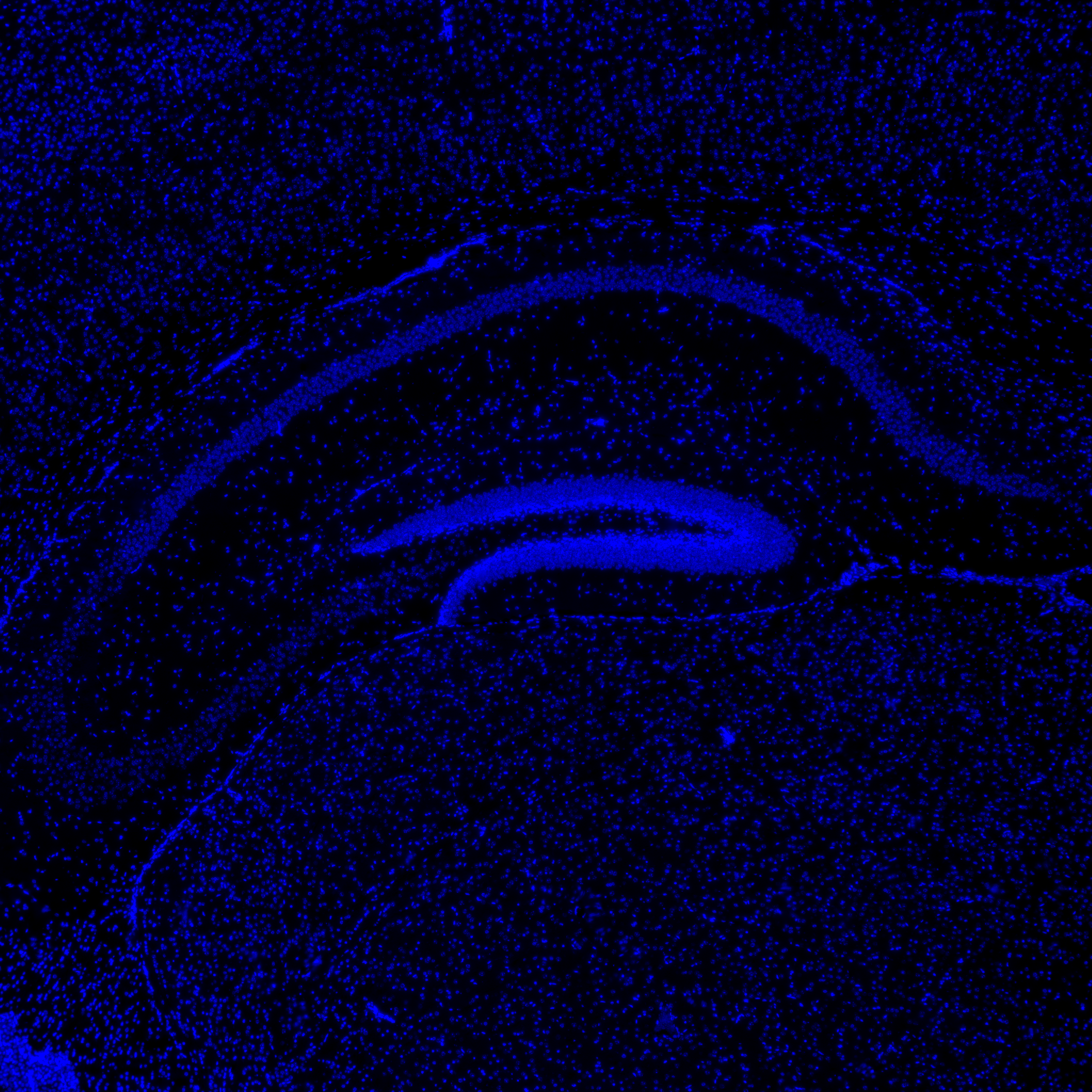

Supplement: Figure 3—source data 4. [file elife-86940-fig3-data4.zip › Figure 3-source data 4/F449-3-CON-F+ ff-P18-HUB-CTIP2-133#-1-5X-left dHPC-Image Export-04_DAPI.tif]

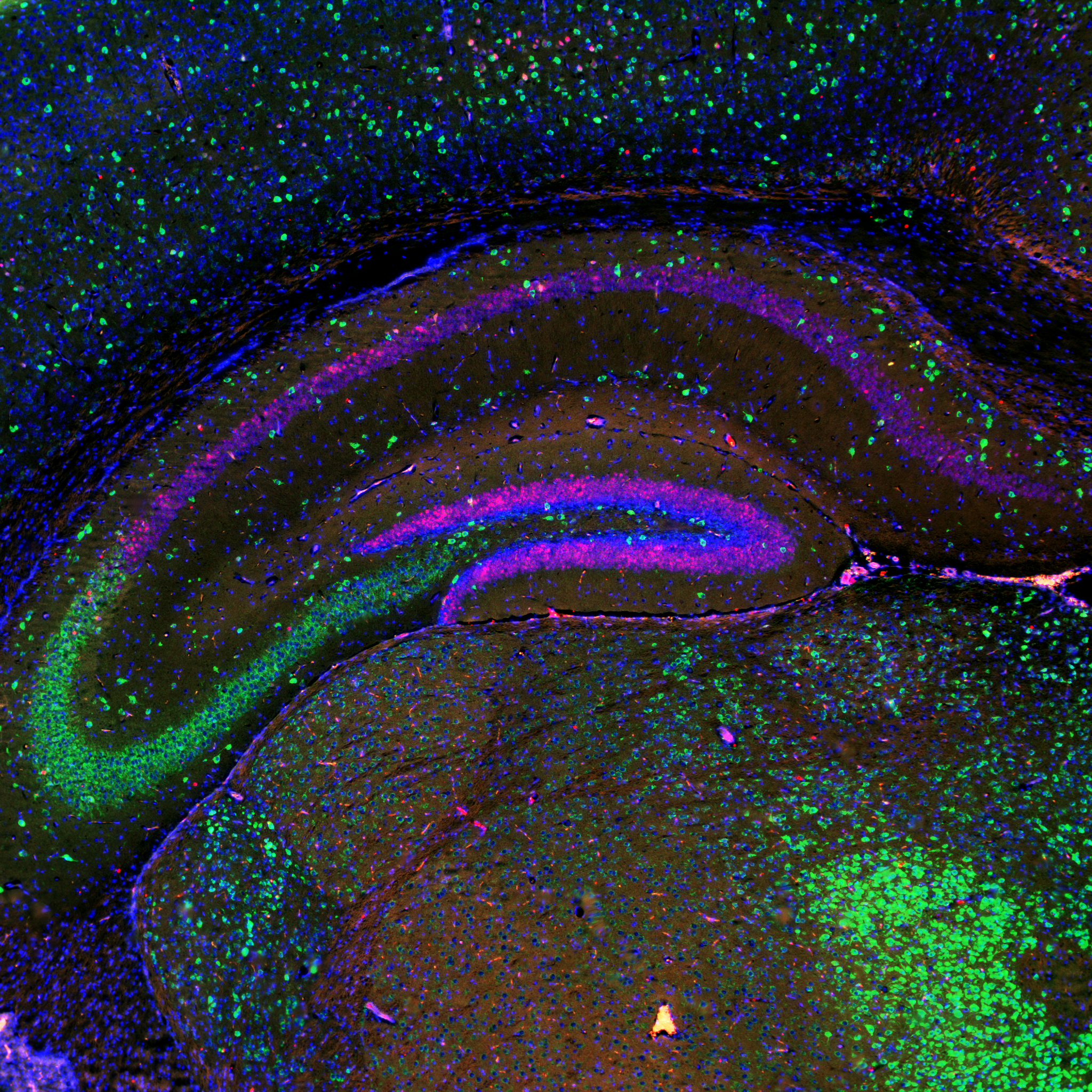

Supplement: Figure 3—source data 4. [file elife-86940-fig3-data4.zip › Figure 3-source data 4/F449-3-CON-F+ ff-P18-HUB-CTIP2-133#-1-5X-left dHPC-Image Export-04.tif]

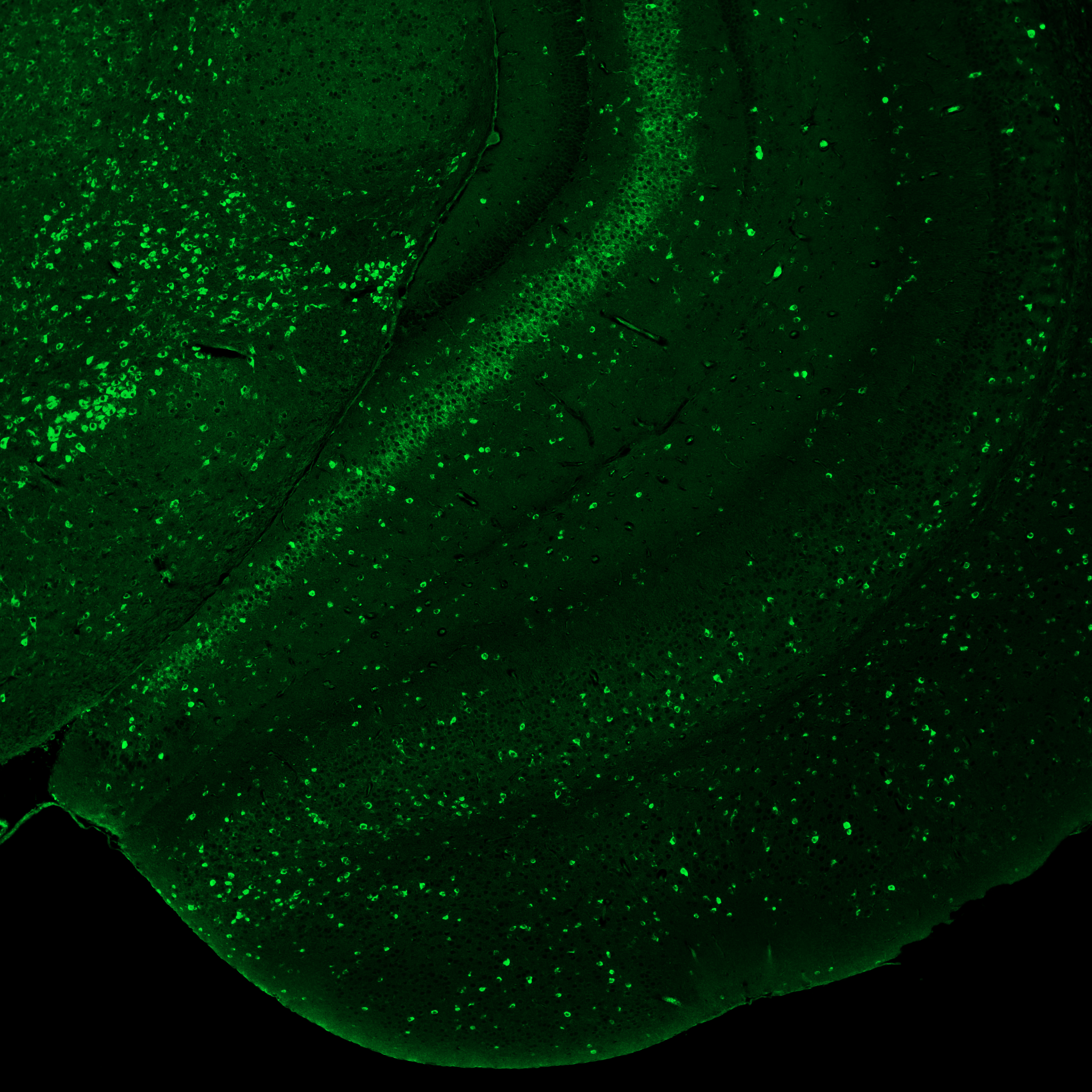

Supplement: Figure 3—source data 4. [file elife-86940-fig3-data4.zip › Figure 3-source data 4/F449-3-CON-F+ ff-P18-HUB-CTIP2-151#-1-5X-right vHPC-Image Export-20_AF488.tif]

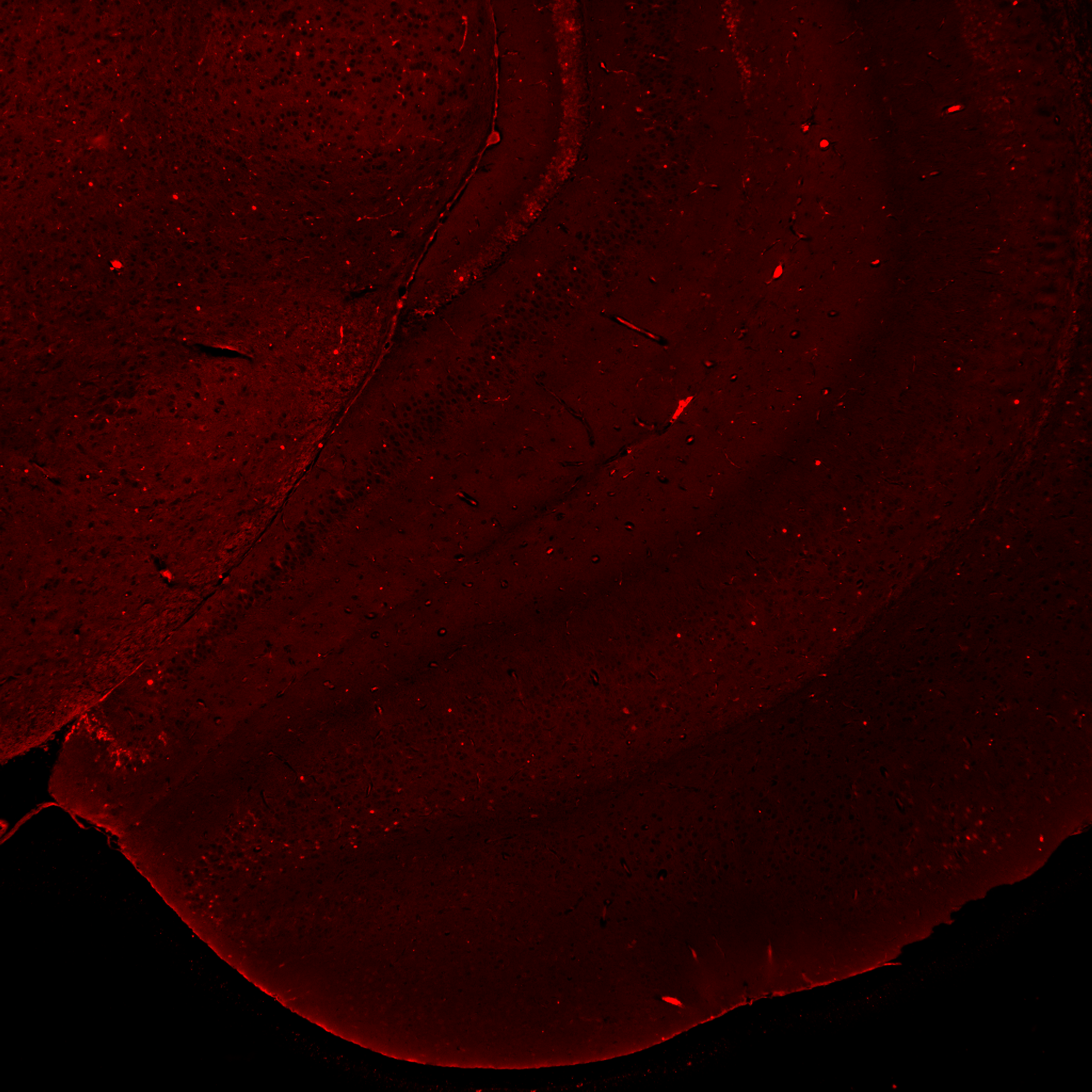

Supplement: Figure 3—source data 4. [file elife-86940-fig3-data4.zip › Figure 3-source data 4/F449-3-CON-F+ ff-P18-HUB-CTIP2-151#-1-5X-right vHPC-Image Export-20_AF594.tif]

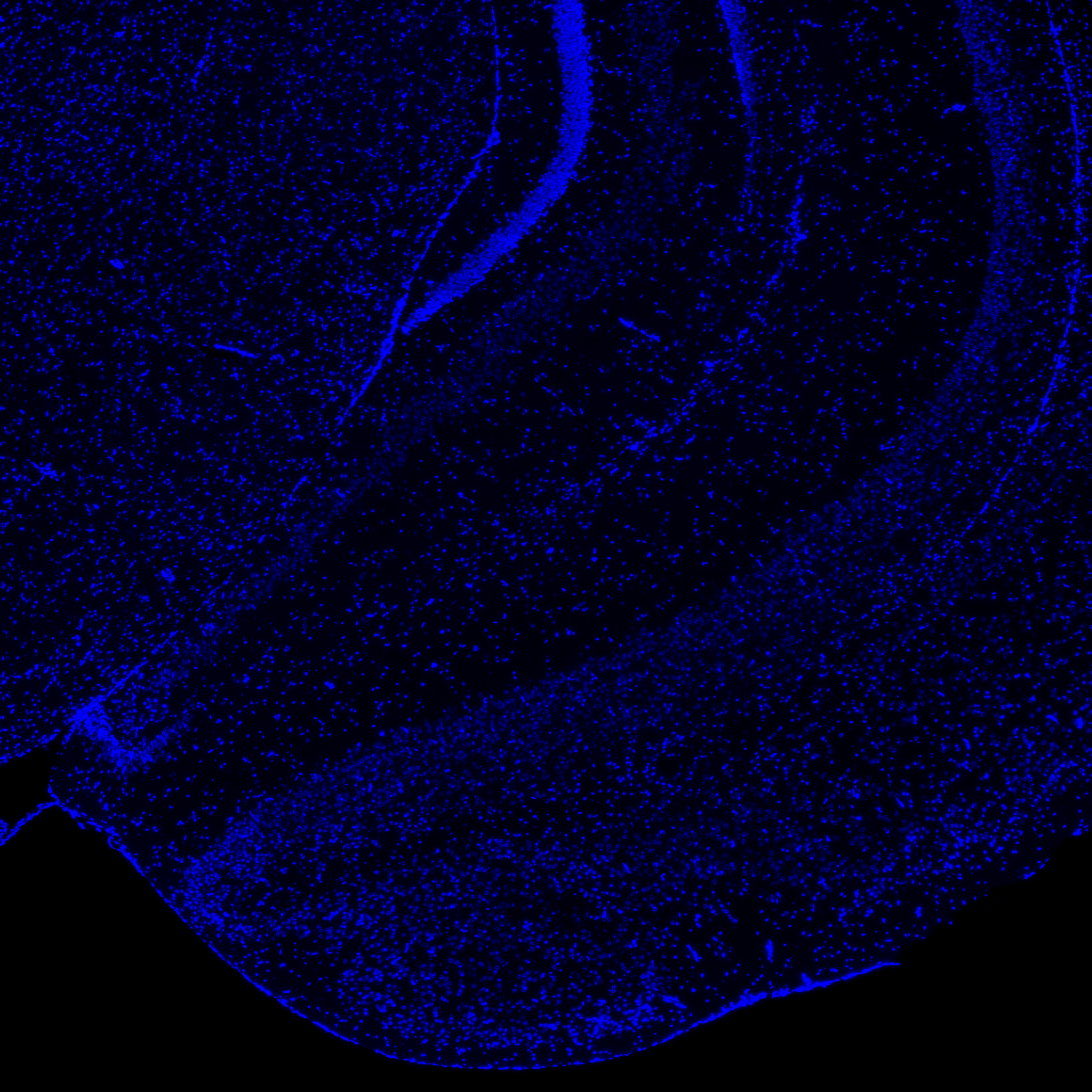

Supplement: Figure 3—source data 4. [file elife-86940-fig3-data4.zip › Figure 3-source data 4/F449-3-CON-F+ ff-P18-HUB-CTIP2-151#-1-5X-right vHPC-Image Export-20_DAPI.tif]

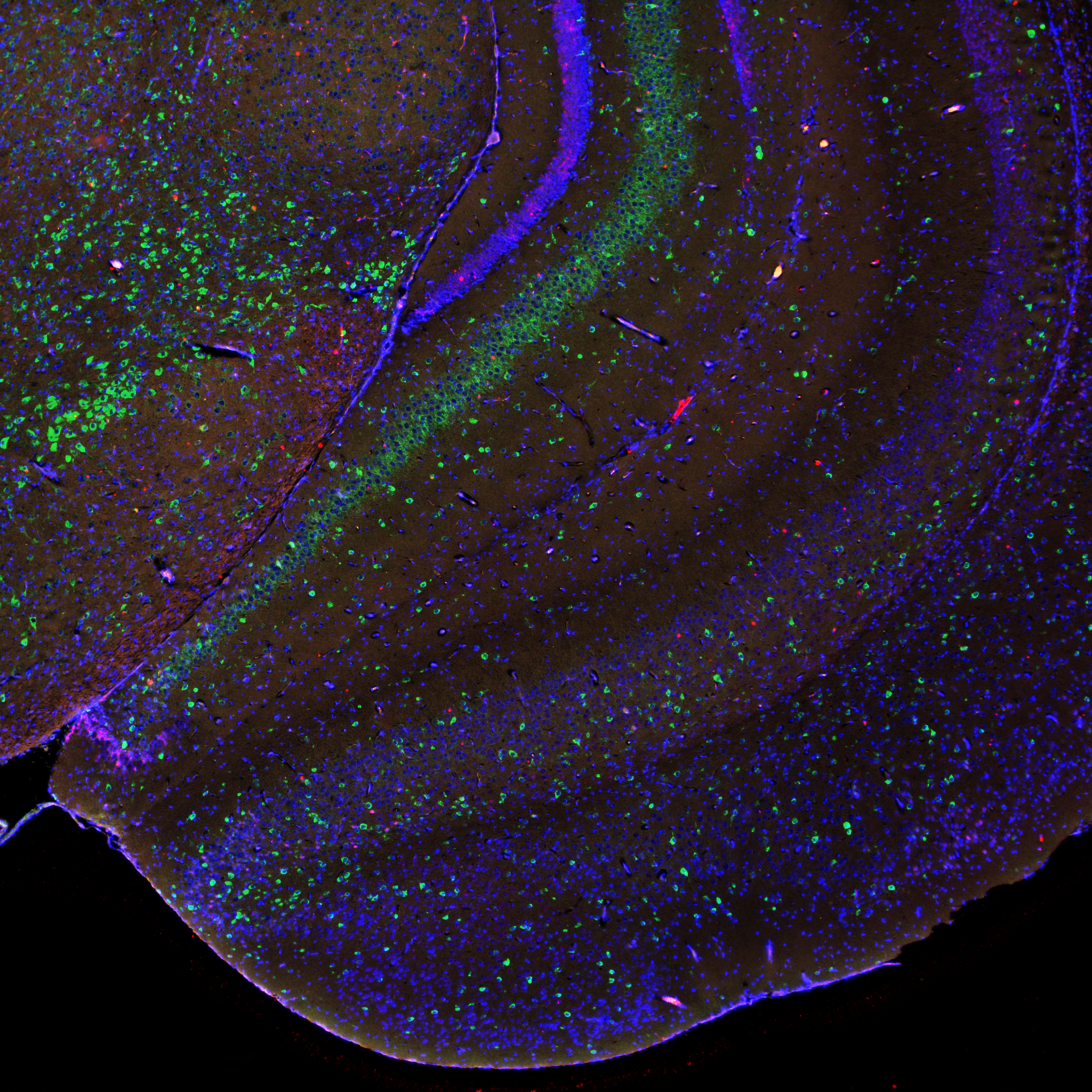

Supplement: Figure 3—source data 4. [file elife-86940-fig3-data4.zip › Figure 3-source data 4/F449-3-CON-F+ ff-P18-HUB-CTIP2-151#-1-5X-right vHPC-Image Export-20.tif]

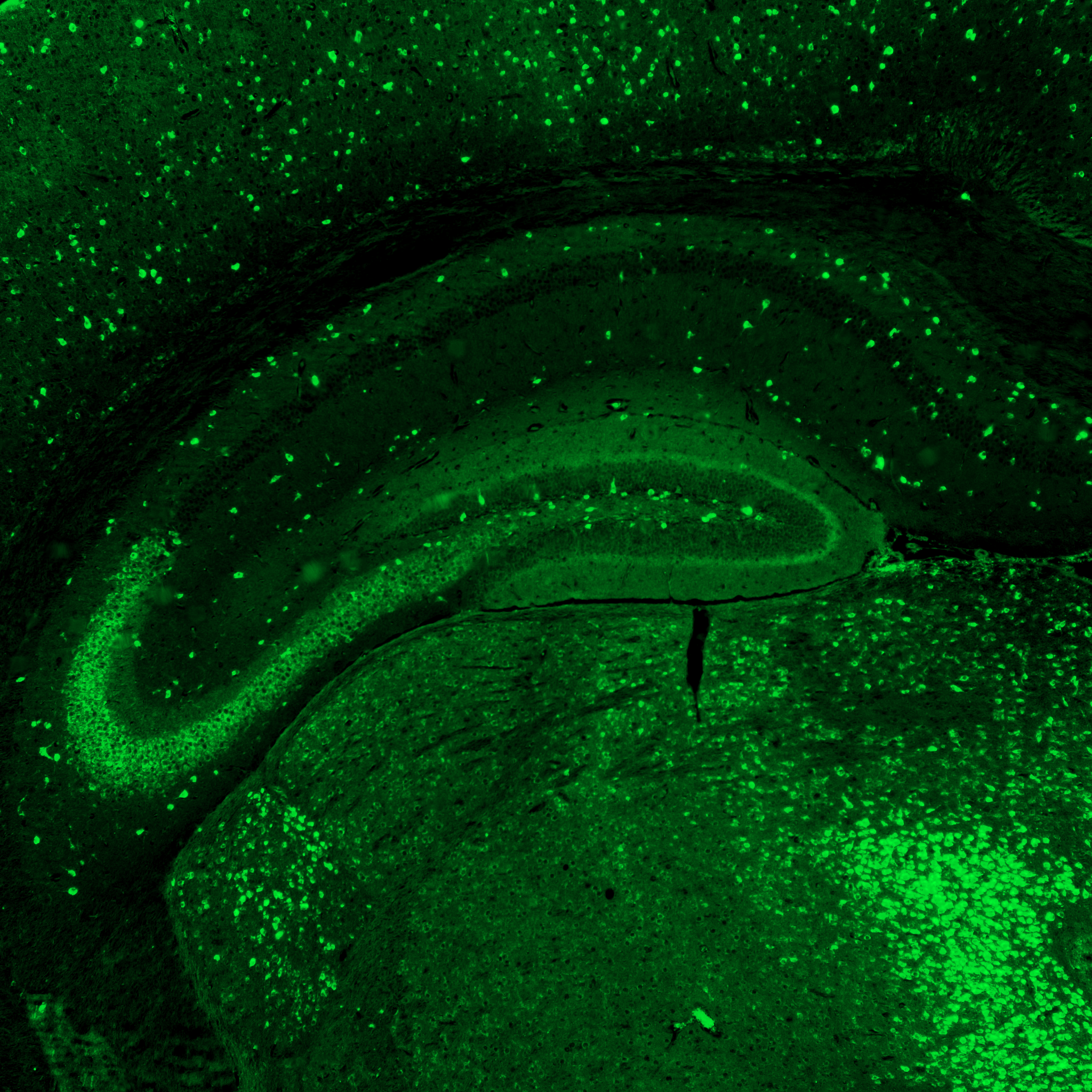

Supplement: Figure 3—source data 4. [file elife-86940-fig3-data4.zip › Figure 3-source data 4/F449-3-CON-F+ ff-P18-HUB-PROX1-133#-3-5X-left dHPC-Image Export-14_AF488.tif]

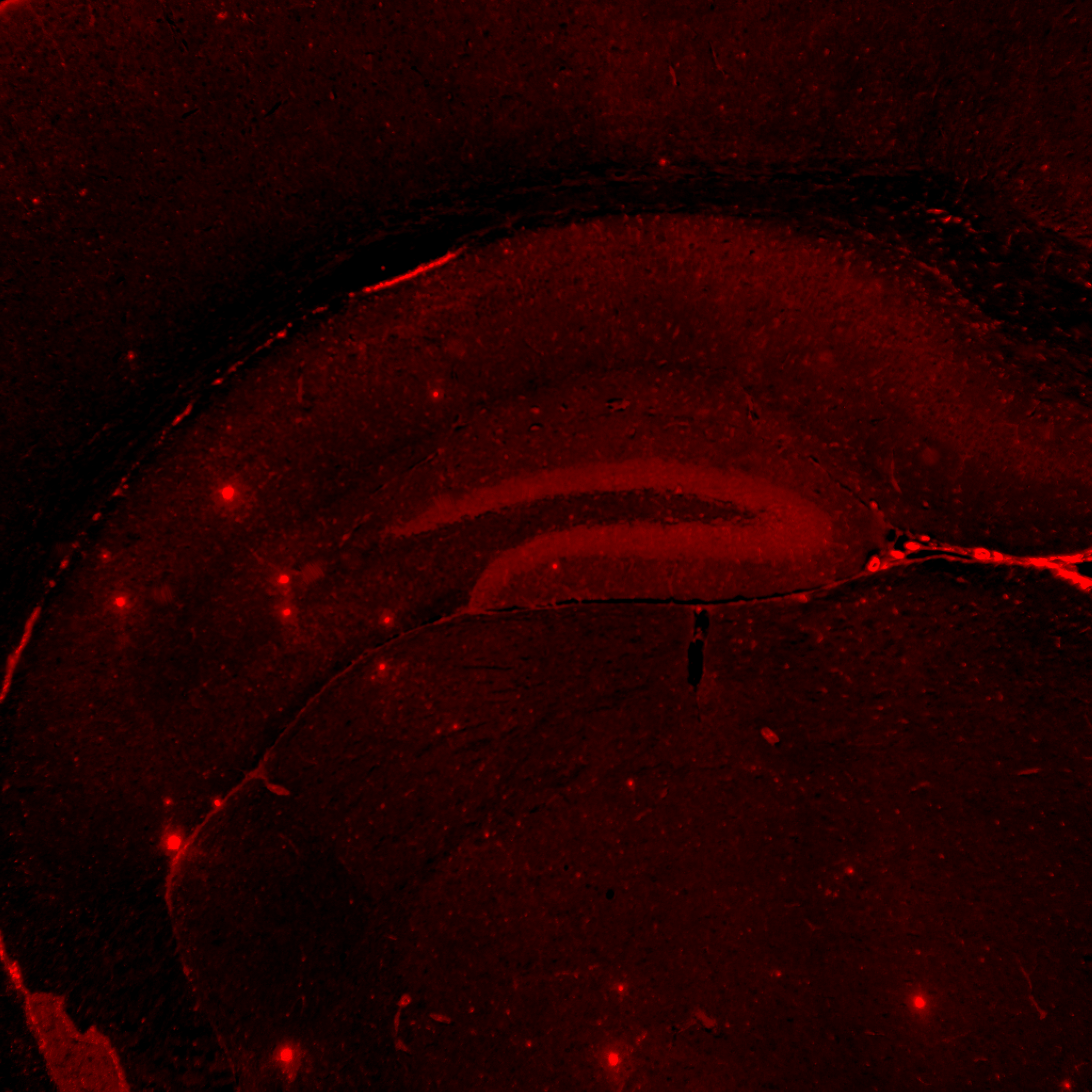

Supplement: Figure 3—source data 4. [file elife-86940-fig3-data4.zip › Figure 3-source data 4/F449-3-CON-F+ ff-P18-HUB-PROX1-133#-3-5X-left dHPC-Image Export-14_AF594.tif]

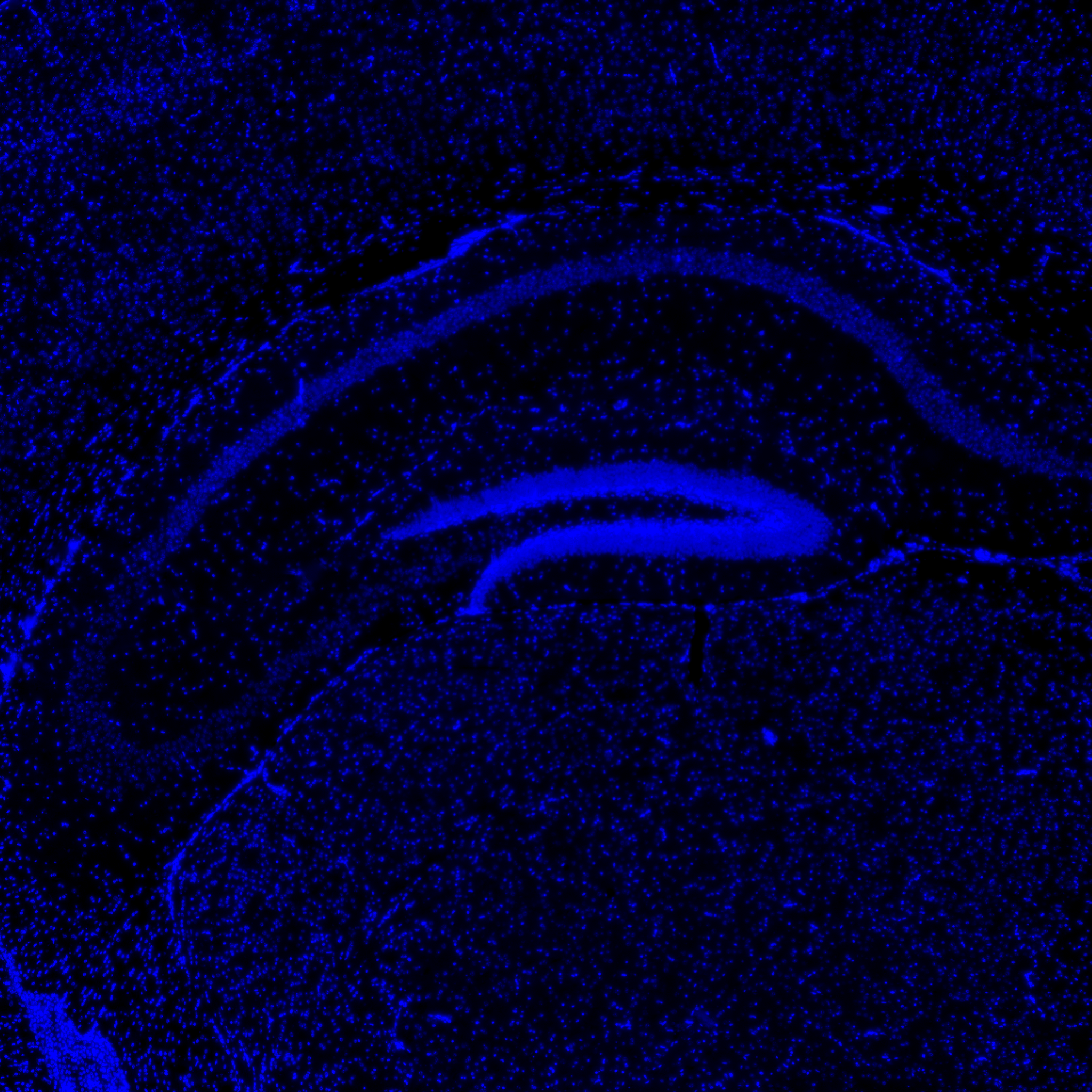

Supplement: Figure 3—source data 4. [file elife-86940-fig3-data4.zip › Figure 3-source data 4/F449-3-CON-F+ ff-P18-HUB-PROX1-133#-3-5X-left dHPC-Image Export-14_DAPI.tif]

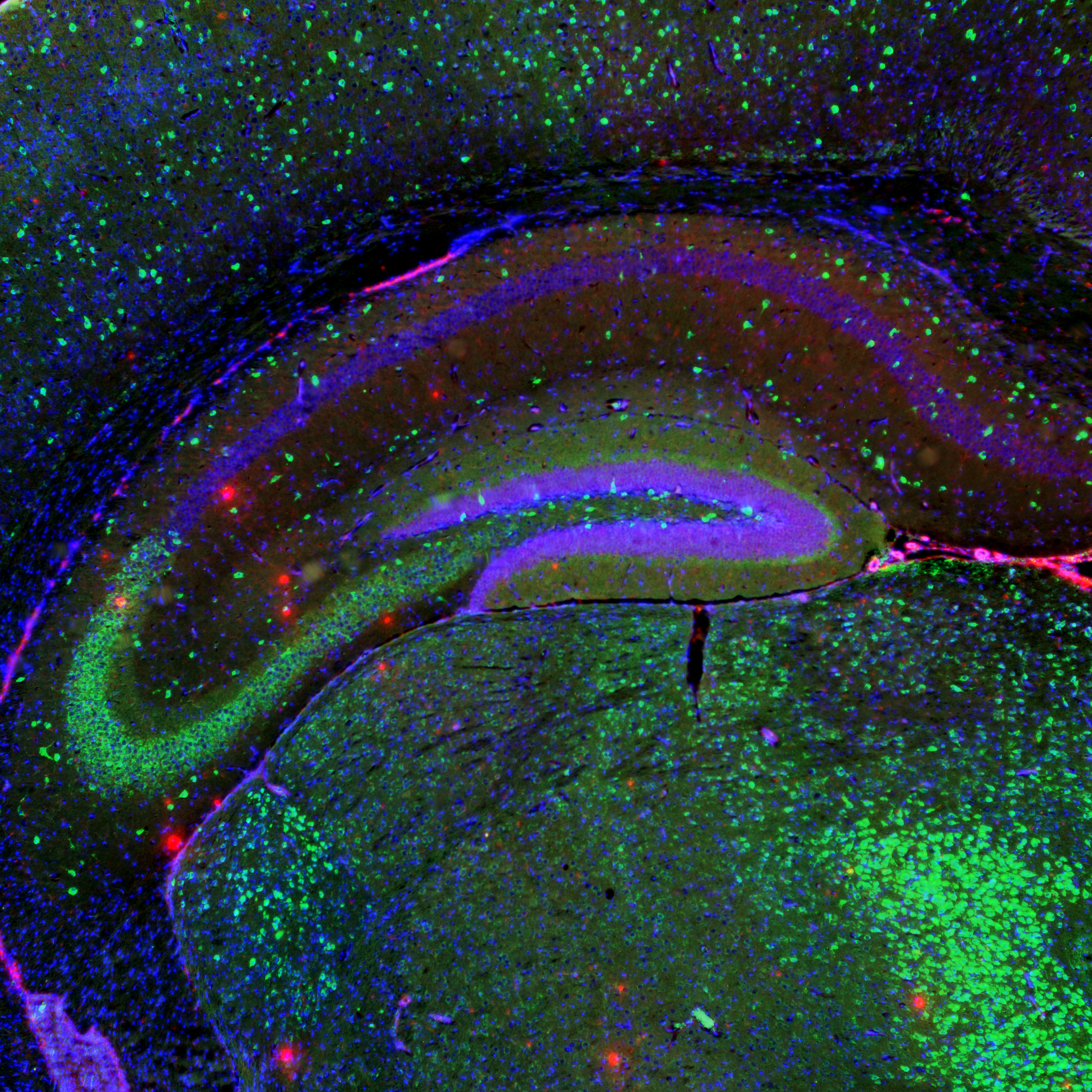

Supplement: Figure 3—source data 4. [file elife-86940-fig3-data4.zip › Figure 3-source data 4/F449-3-CON-F+ ff-P18-HUB-PROX1-133#-3-5X-left dHPC-Image Export-14.tif]

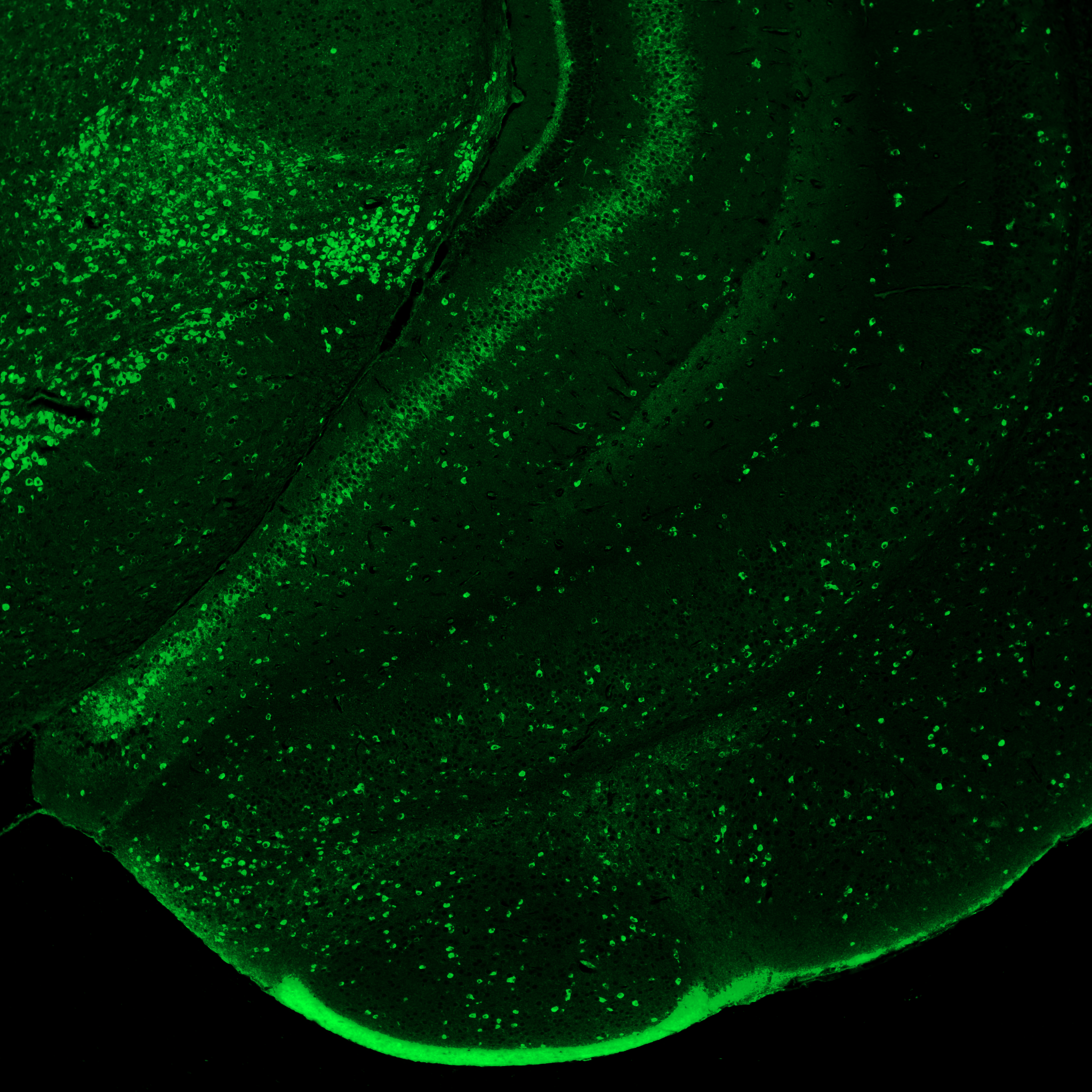

Supplement: Figure 3—source data 4. [file elife-86940-fig3-data4.zip › Figure 3-source data 4/F449-3-CON-F+ ff-P18-HUB-PROX1-151#-3-5X-right vHPC-Image Export-15_AF488.tif]

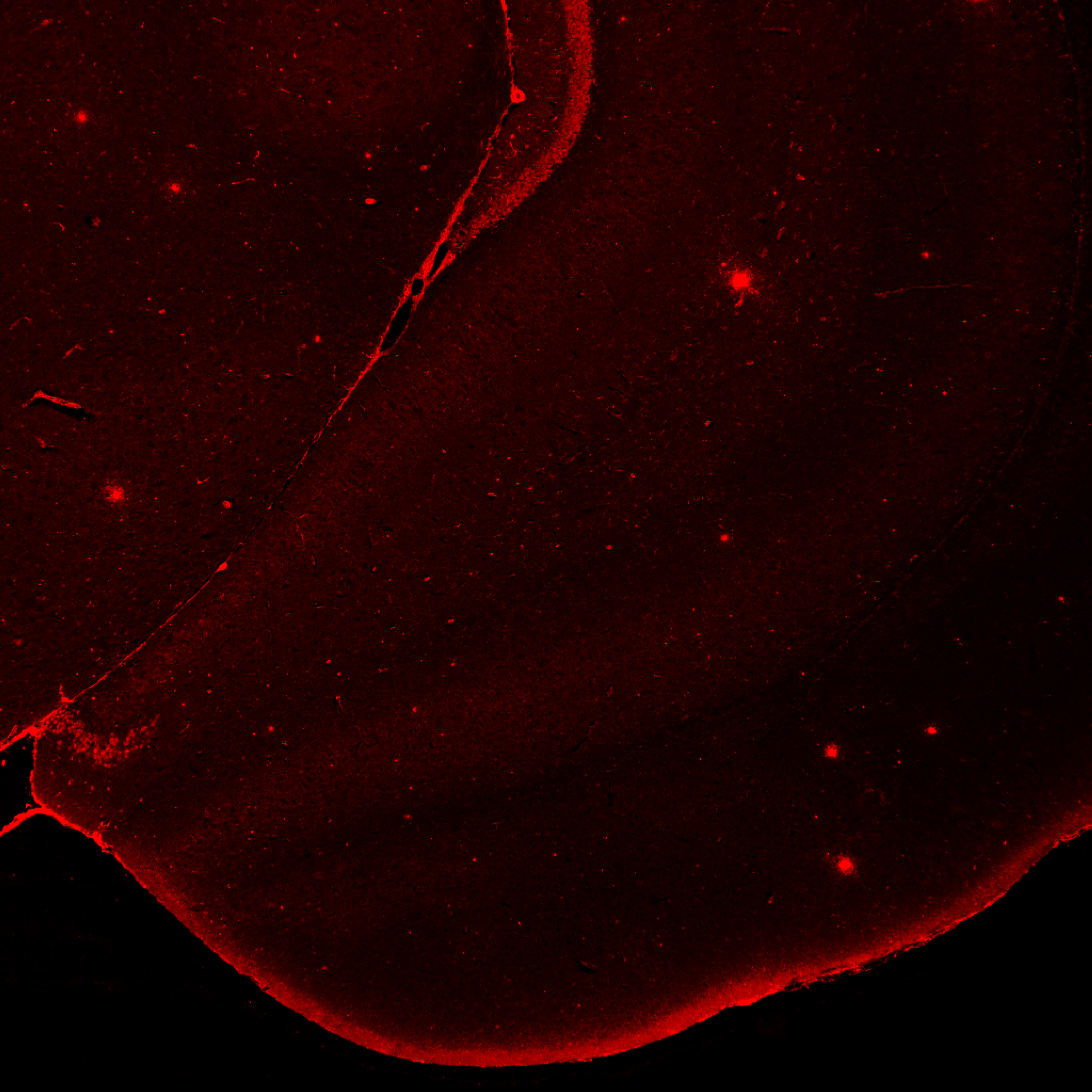

Supplement: Figure 3—source data 4. [file elife-86940-fig3-data4.zip › Figure 3-source data 4/F449-3-CON-F+ ff-P18-HUB-PROX1-151#-3-5X-right vHPC-Image Export-15_AF594.tif]

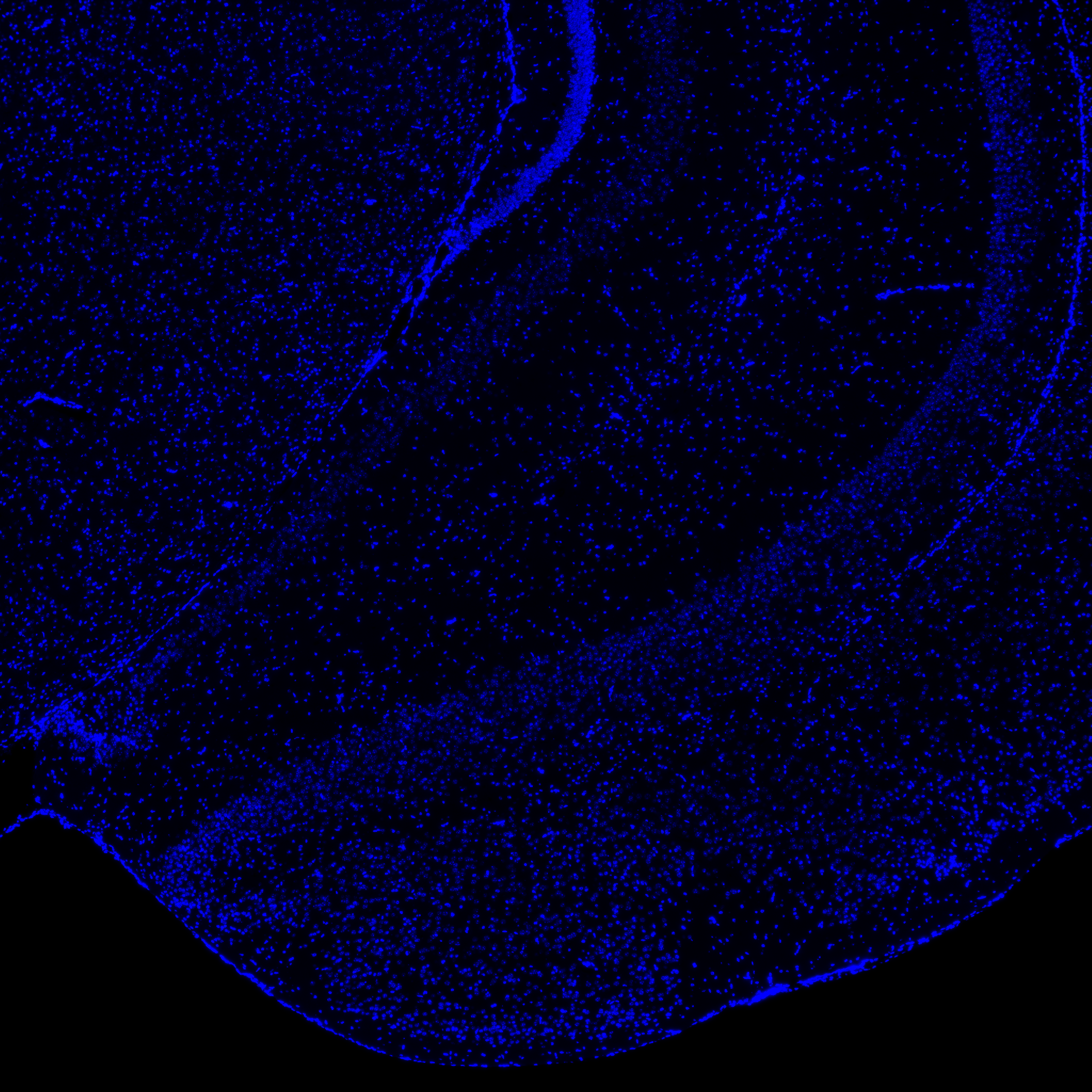

Supplement: Figure 3—source data 4. [file elife-86940-fig3-data4.zip › Figure 3-source data 4/F449-3-CON-F+ ff-P18-HUB-PROX1-151#-3-5X-right vHPC-Image Export-15_DAPI.tif]

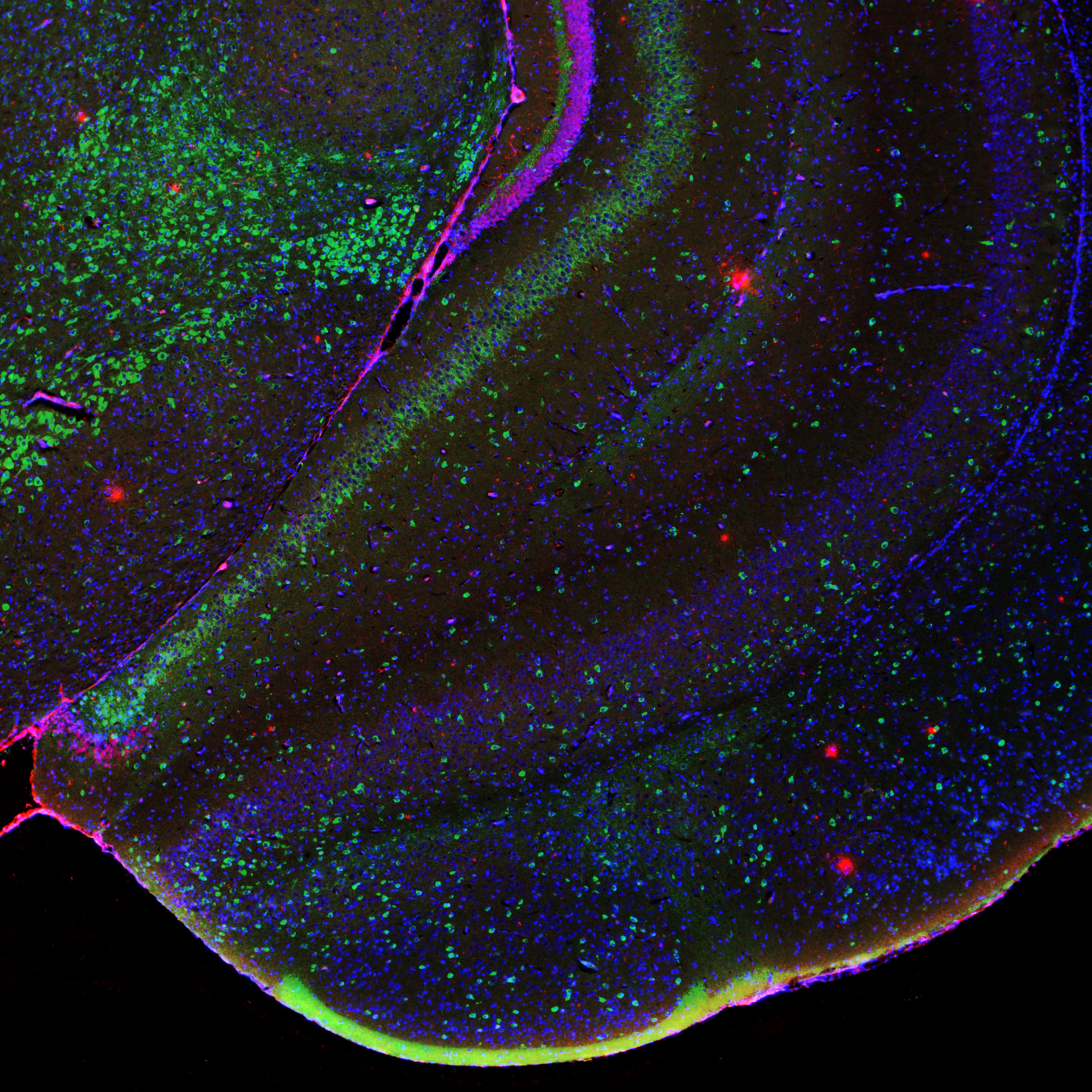

Supplement: Figure 3—source data 4. [file elife-86940-fig3-data4.zip › Figure 3-source data 4/F449-3-CON-F+ ff-P18-HUB-PROX1-151#-3-5X-right vHPC-Image Export-15.tif]

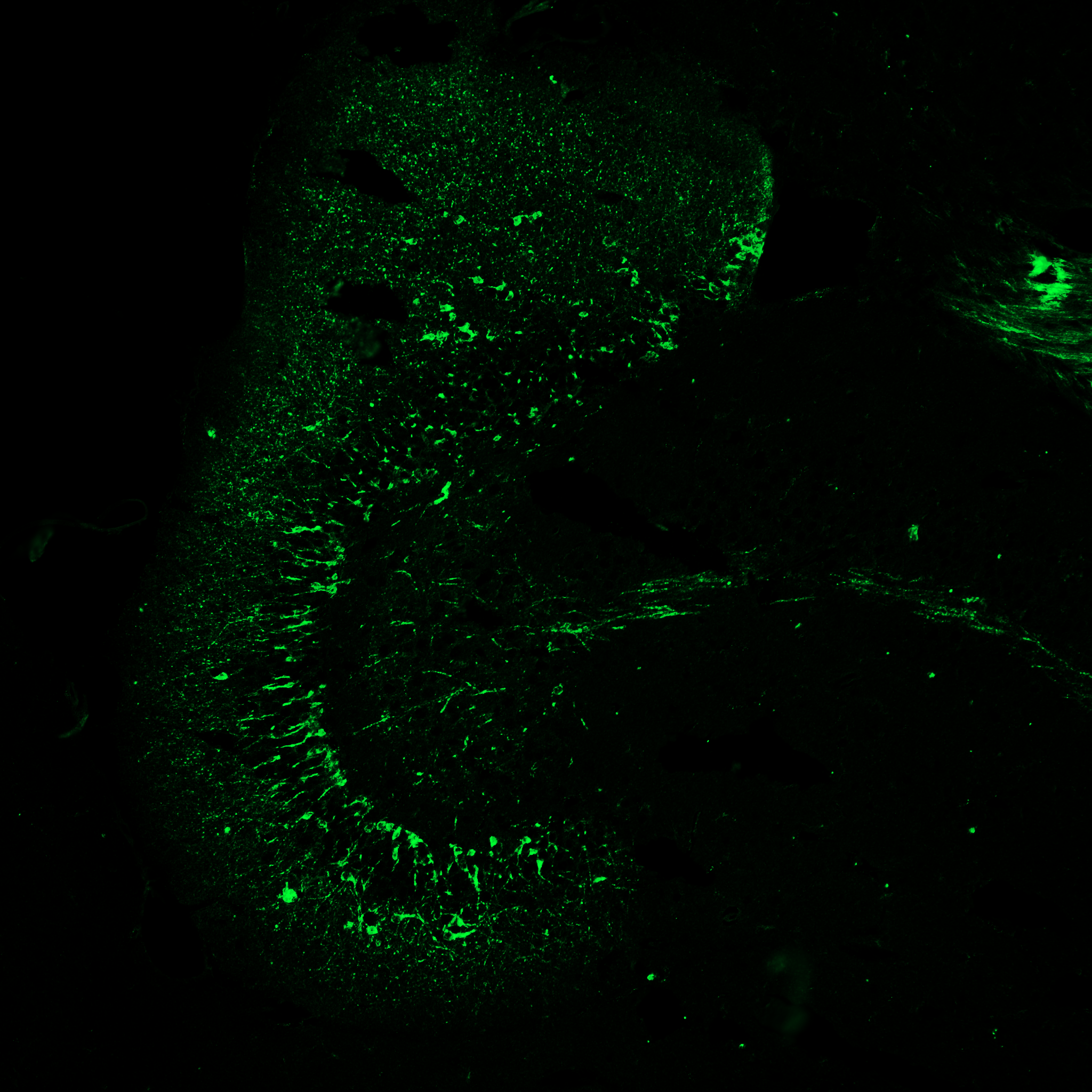

Supplement: Figure 3—figure supplement 1—source data 1. [file elife-86940-fig3-figsupp1-data1.zip › Figure 3-figure supplement 1-source data 1/2879-CII CON-CII FF-1M-10X-DCX-63-2-vDG-Image Export-11_AF488.tif]

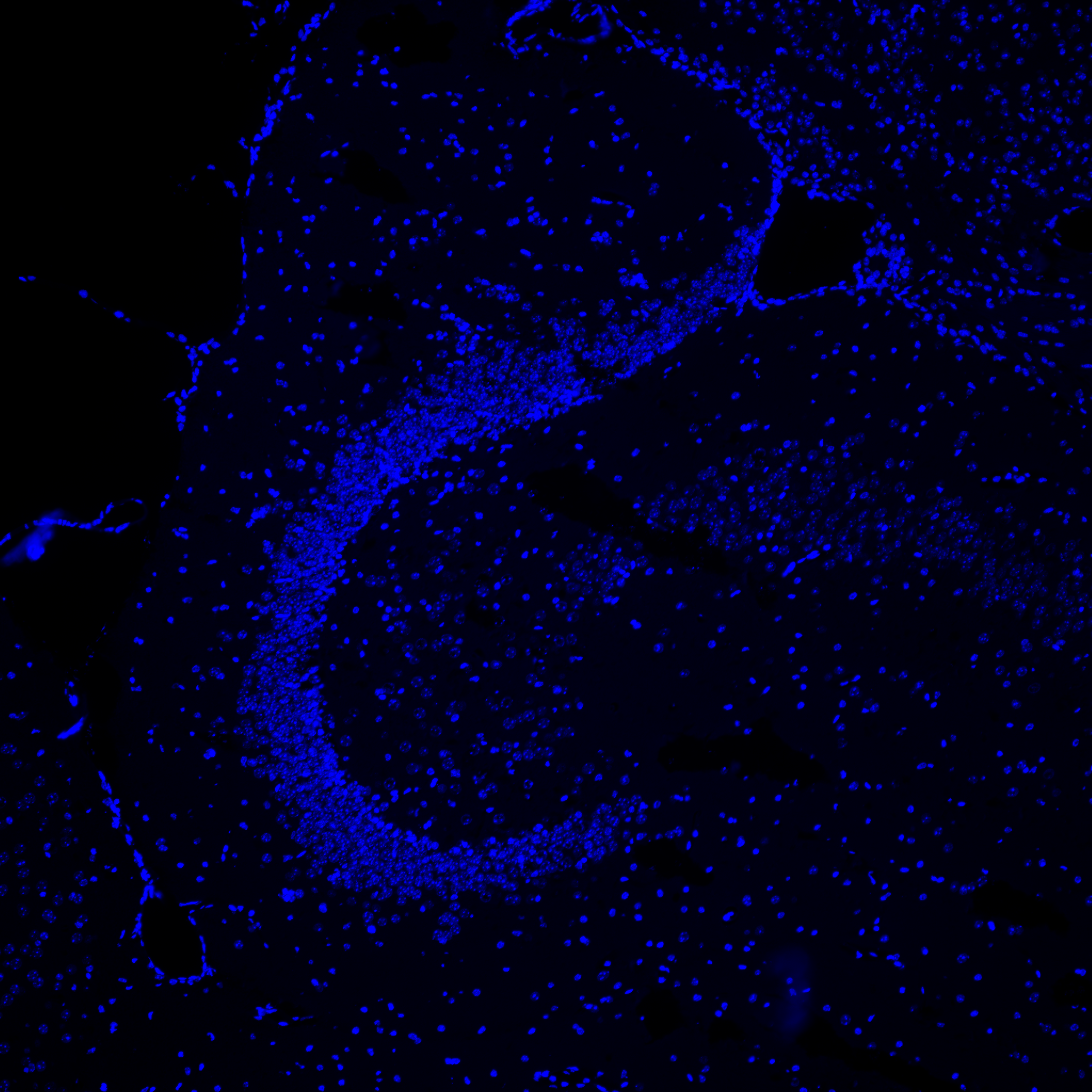

Supplement: Figure 3—figure supplement 1—source data 1. [file elife-86940-fig3-figsupp1-data1.zip › Figure 3-figure supplement 1-source data 1/2879-CII CON-CII FF-1M-10X-DCX-63-2-vDG-Image Export-11_DAPI.tif]

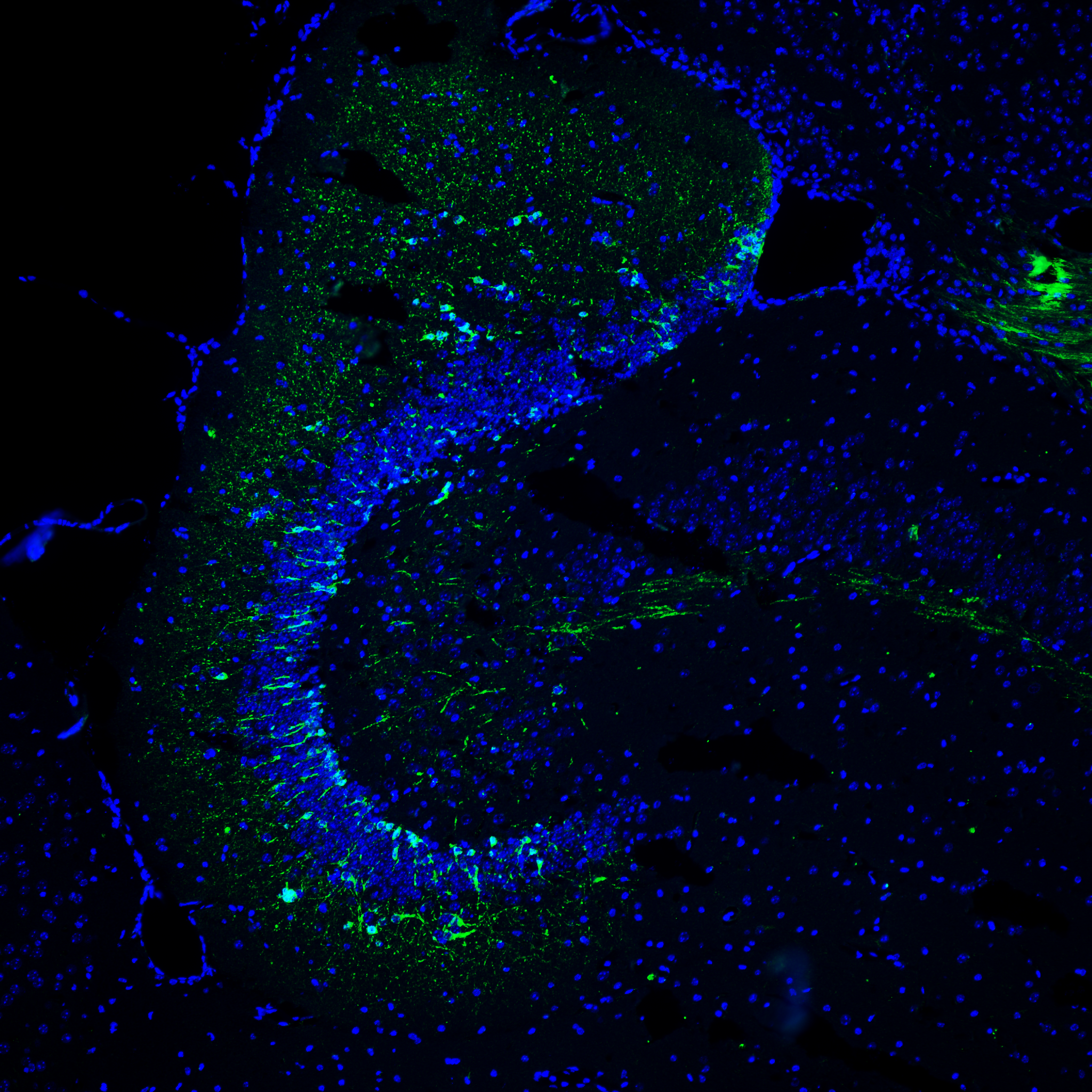

Supplement: Figure 3—figure supplement 1—source data 1. [file elife-86940-fig3-figsupp1-data1.zip › Figure 3-figure supplement 1-source data 1/2879-CII CON-CII FF-1M-10X-DCX-63-2-vDG-Image Export-11_G+D.tif]

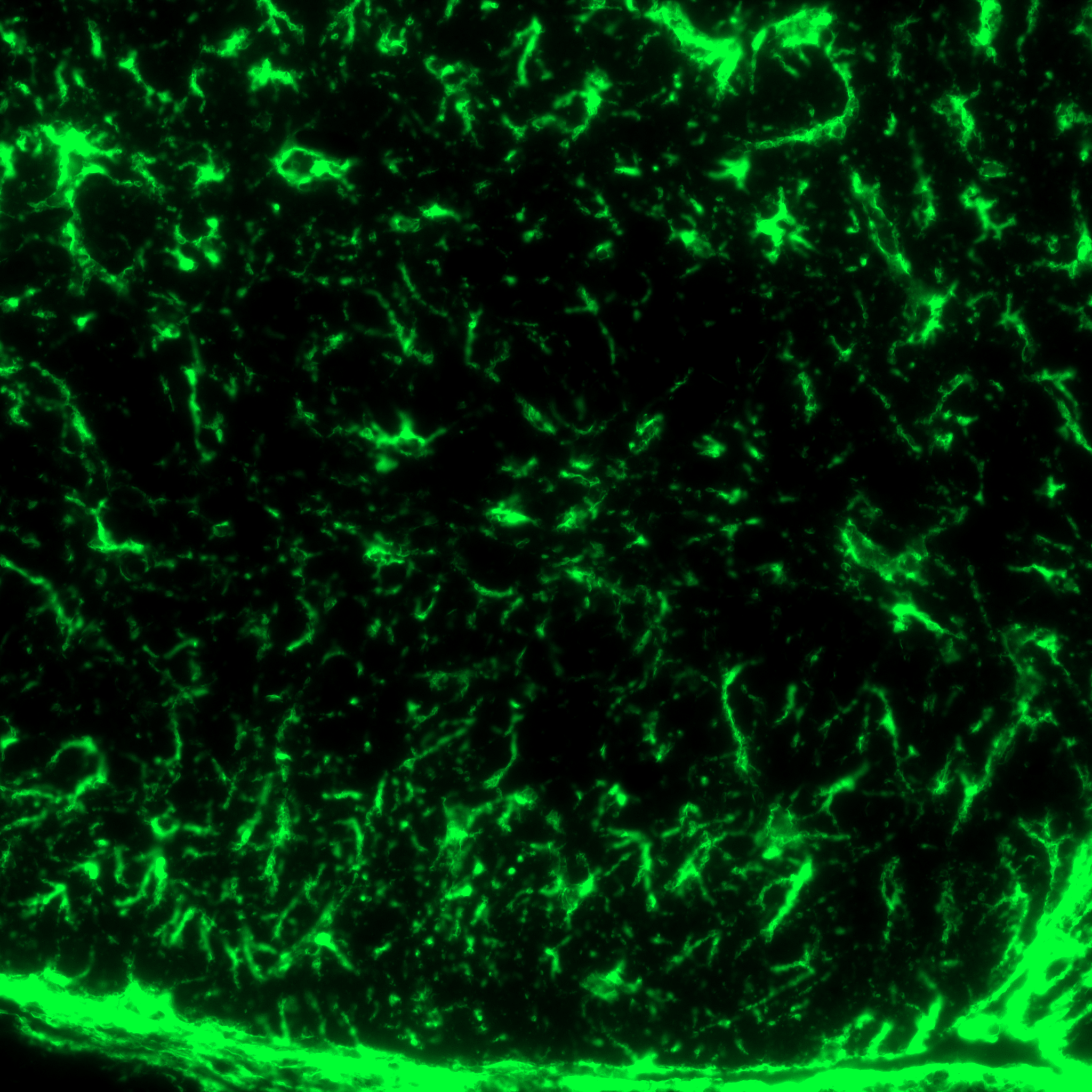

Supplement: Figure 3—figure supplement 1—source data 1. [file elife-86940-fig3-figsupp1-data1.zip › Figure 3-figure supplement 1-source data 1/F449-1-DKO-RX CI CII ff FF-P18-40X-GFAP-NESTIN-#89-1-HPC-R-Image Export-7_AF488.tif]

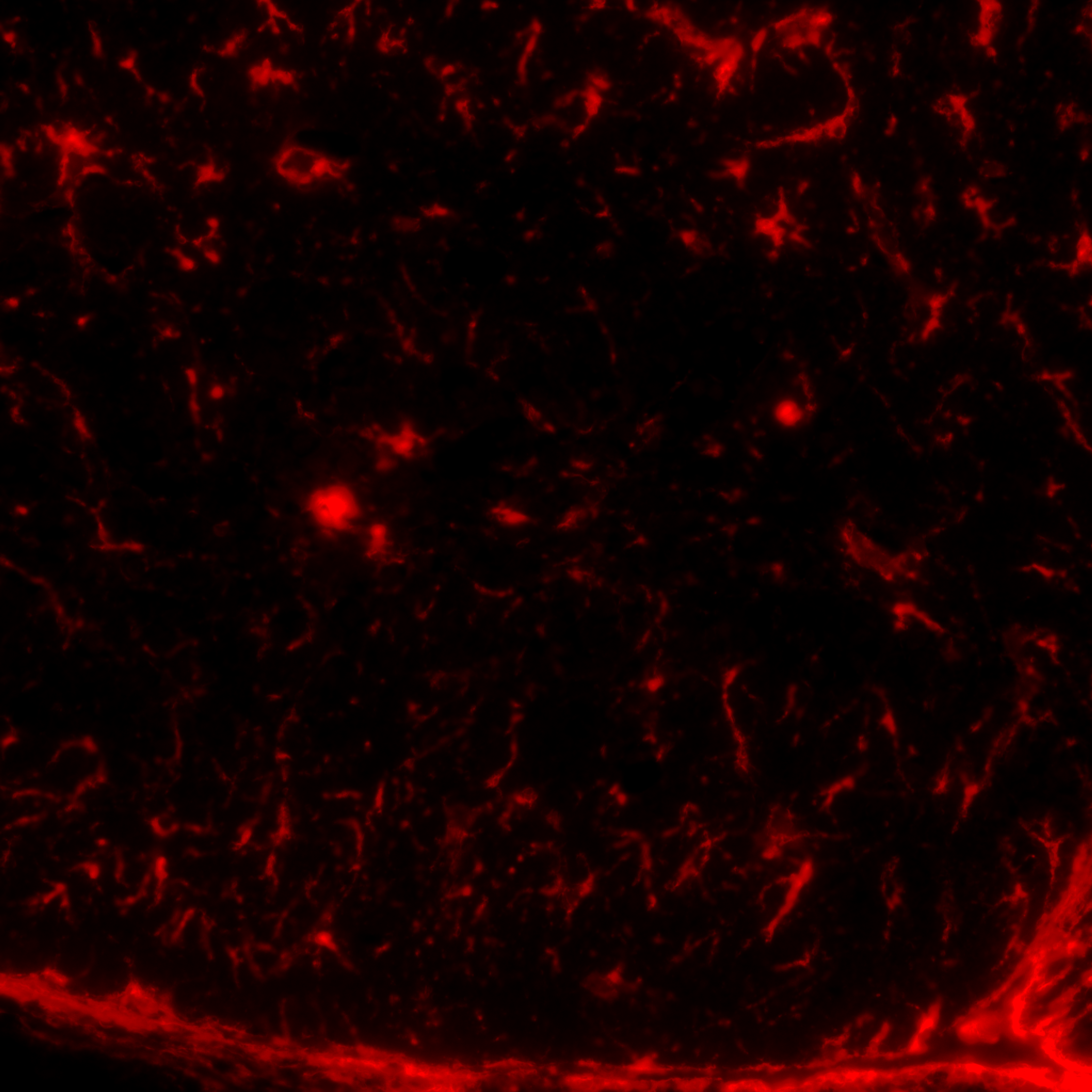

Supplement: Figure 3—figure supplement 1—source data 1. [file elife-86940-fig3-figsupp1-data1.zip › Figure 3-figure supplement 1-source data 1/F449-1-DKO-RX CI CII ff FF-P18-40X-GFAP-NESTIN-#89-1-HPC-R-Image Export-7_AF594.tif]

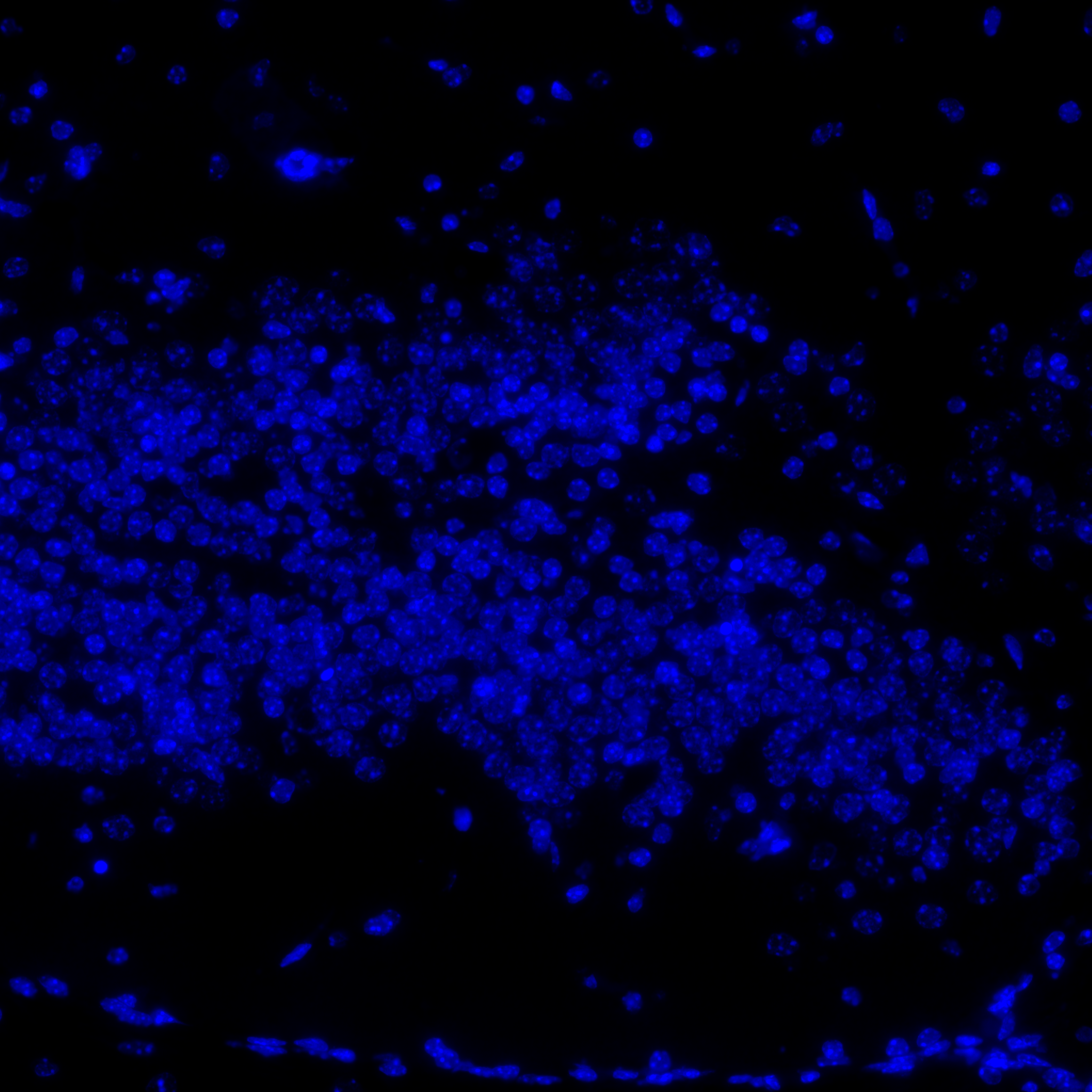

Supplement: Figure 3—figure supplement 1—source data 1. [file elife-86940-fig3-figsupp1-data1.zip › Figure 3-figure supplement 1-source data 1/F449-1-DKO-RX CI CII ff FF-P18-40X-GFAP-NESTIN-#89-1-HPC-R-Image Export-7_DAPI.tif]

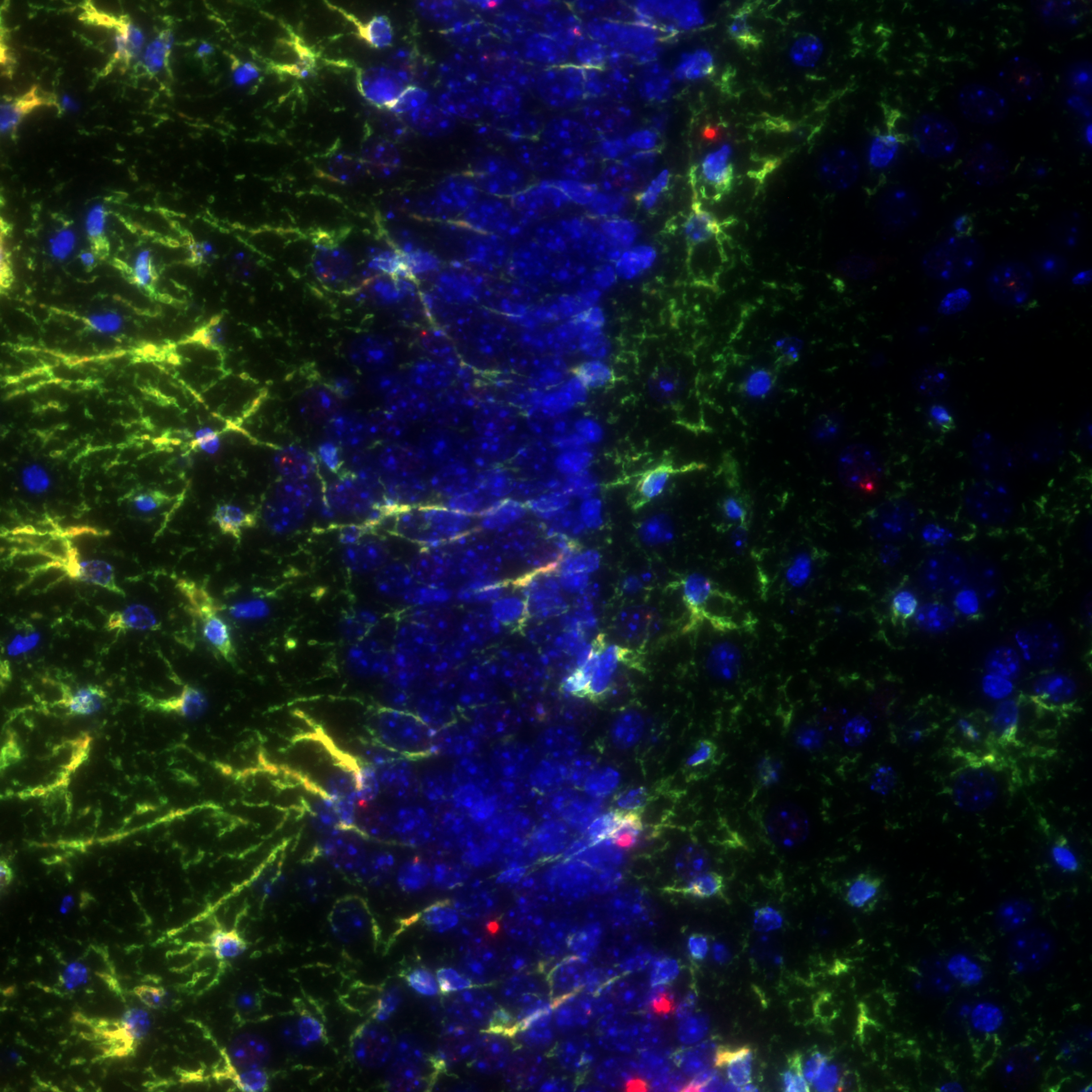

Supplement: Figure 3—figure supplement 1—source data 1. [file elife-86940-fig3-figsupp1-data1.zip › Figure 3-figure supplement 1-source data 1/2879-CON-CII FF-1M-40X-GFAP-NESTIN-#62-1-vHPC-Image Export-08.tif]

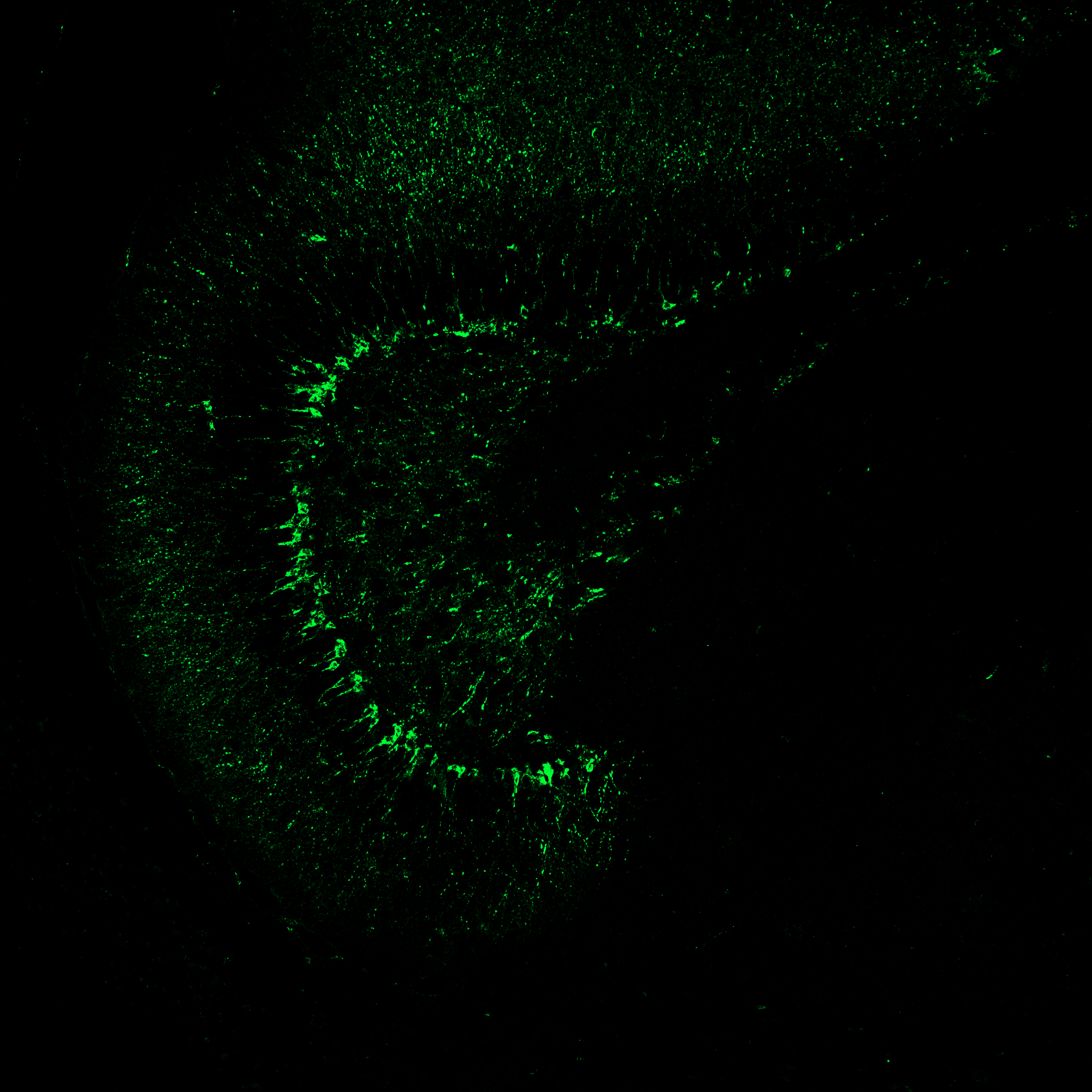

Supplement: Figure 3—figure supplement 1—source data 1. [file elife-86940-fig3-figsupp1-data1.zip › Figure 3-figure supplement 1-source data 1/2881-CII CKO-RX CII FF-1M-10X-DCX-56-2-vDG-Image Export-15_AF488.tif]

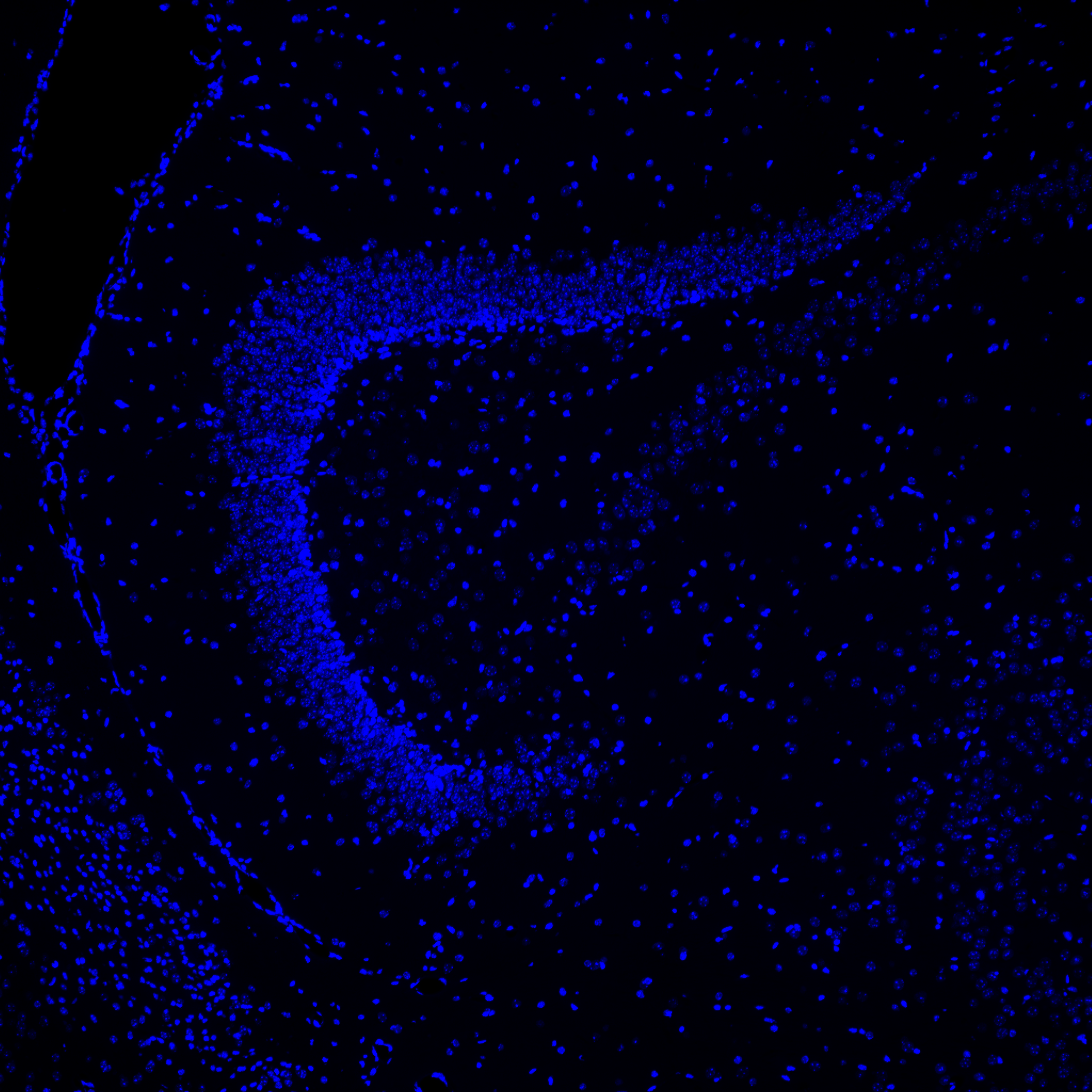

Supplement: Figure 3—figure supplement 1—source data 1. [file elife-86940-fig3-figsupp1-data1.zip › Figure 3-figure supplement 1-source data 1/2881-CII CKO-RX CII FF-1M-10X-DCX-56-2-vDG-Image Export-15_DAPI.tif]

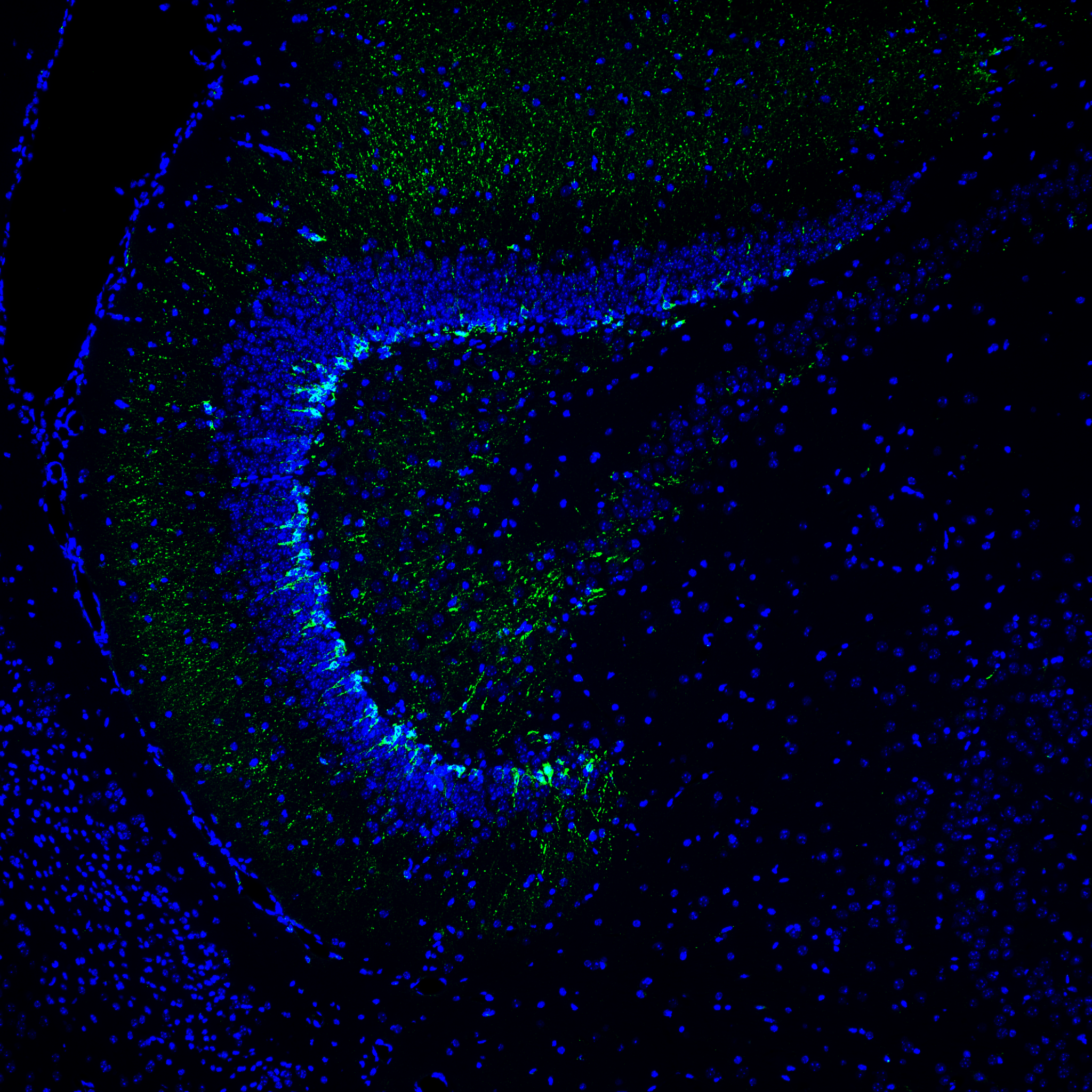

Supplement: Figure 3—figure supplement 1—source data 1. [file elife-86940-fig3-figsupp1-data1.zip › Figure 3-figure supplement 1-source data 1/2881-CII CKO-RX CII FF-1M-10X-DCX-56-2-vDG-Image Export-15_G+D.tif]

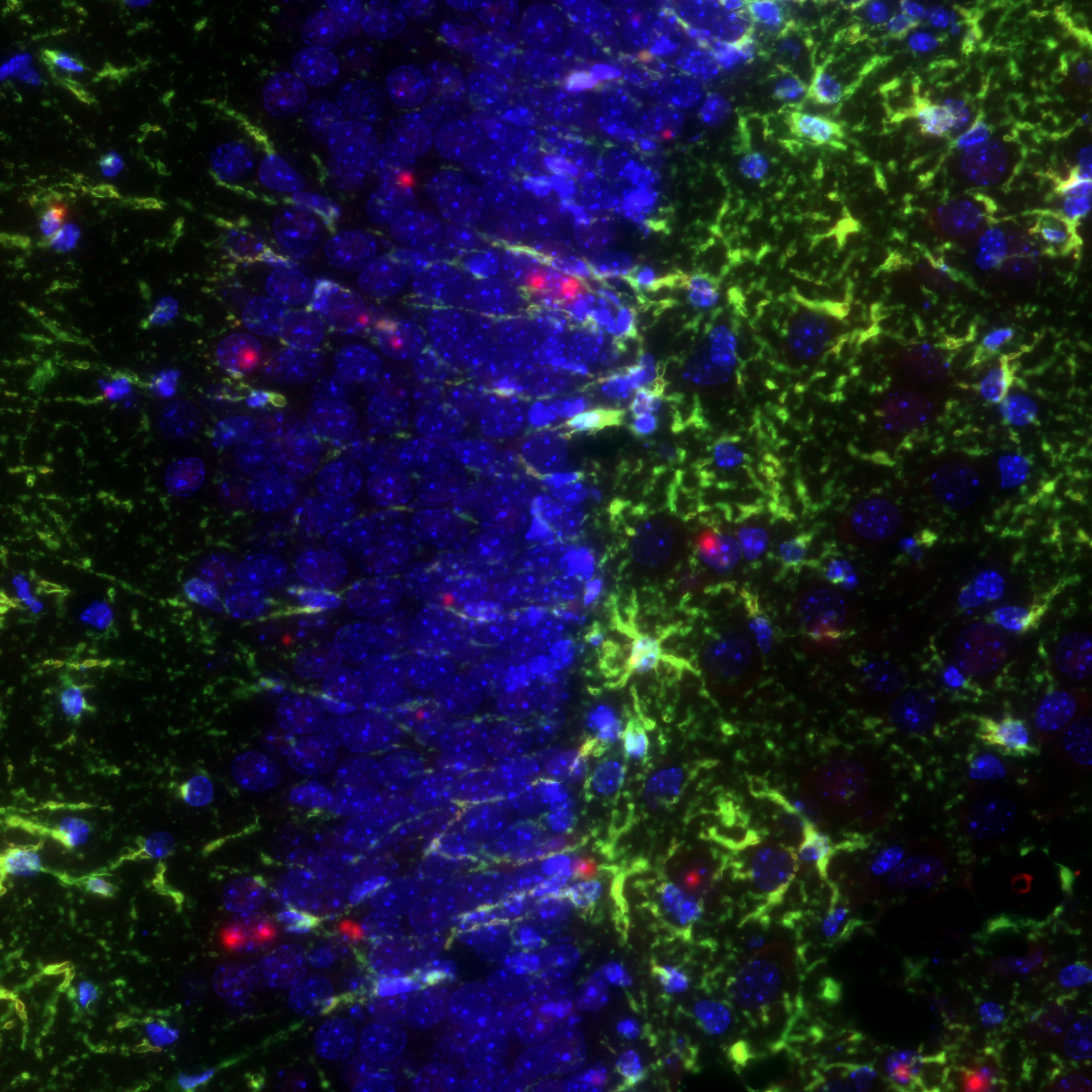

Supplement: Figure 3—figure supplement 1—source data 1. [file elife-86940-fig3-figsupp1-data1.zip › Figure 3-figure supplement 1-source data 1/2881-CKO-RX CII FF-1M-40X-GFAP-NESTIN-#59-1-vHPC-G+R-Image Export-14.tif]

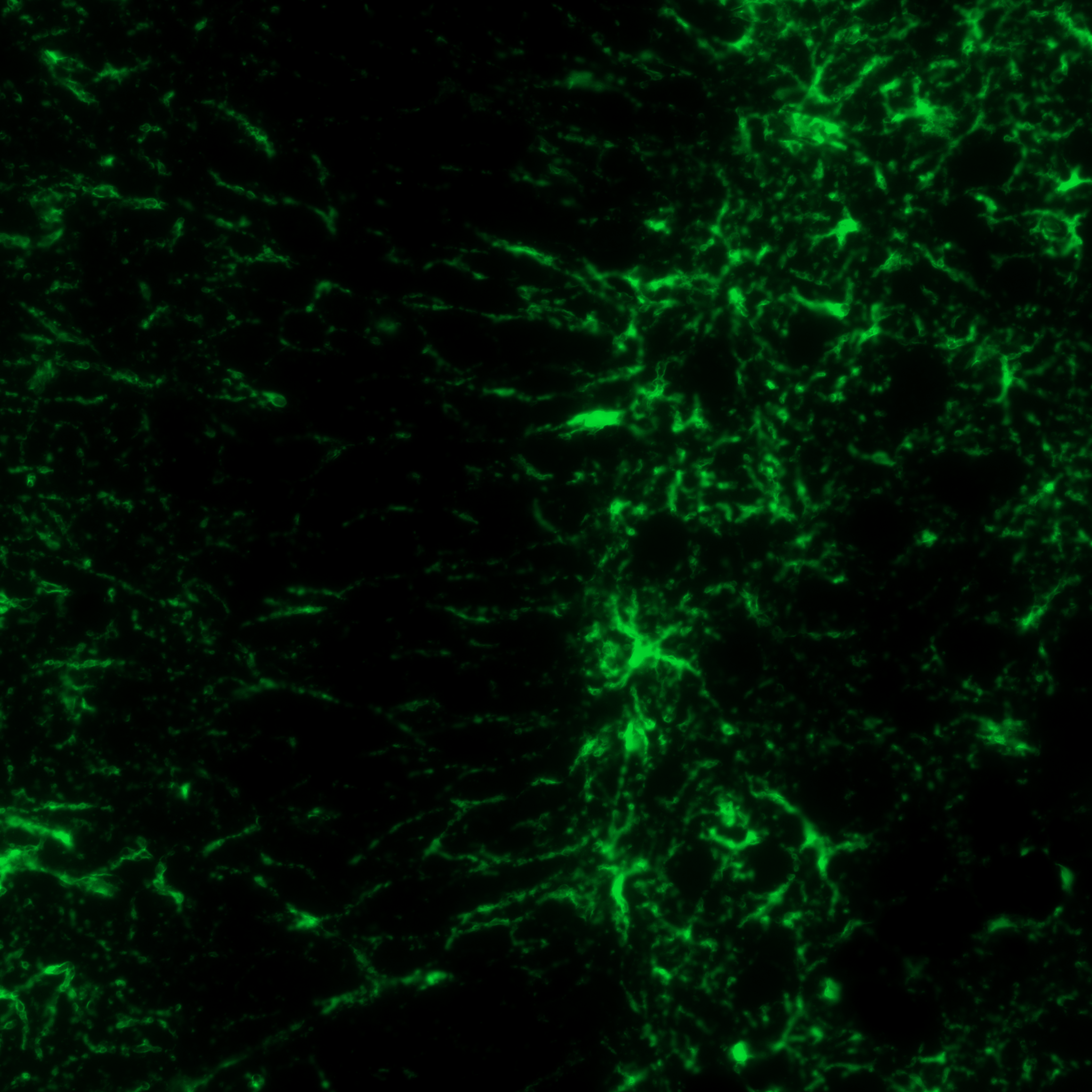

Supplement: Figure 3—figure supplement 1—source data 1. [file elife-86940-fig3-figsupp1-data1.zip › Figure 3-figure supplement 1-source data 1/2881-CKO-RX CII FF-1M-40X-GFAP-NESTIN-#59-1-vHPC-Image Export-13_AF488.tif]

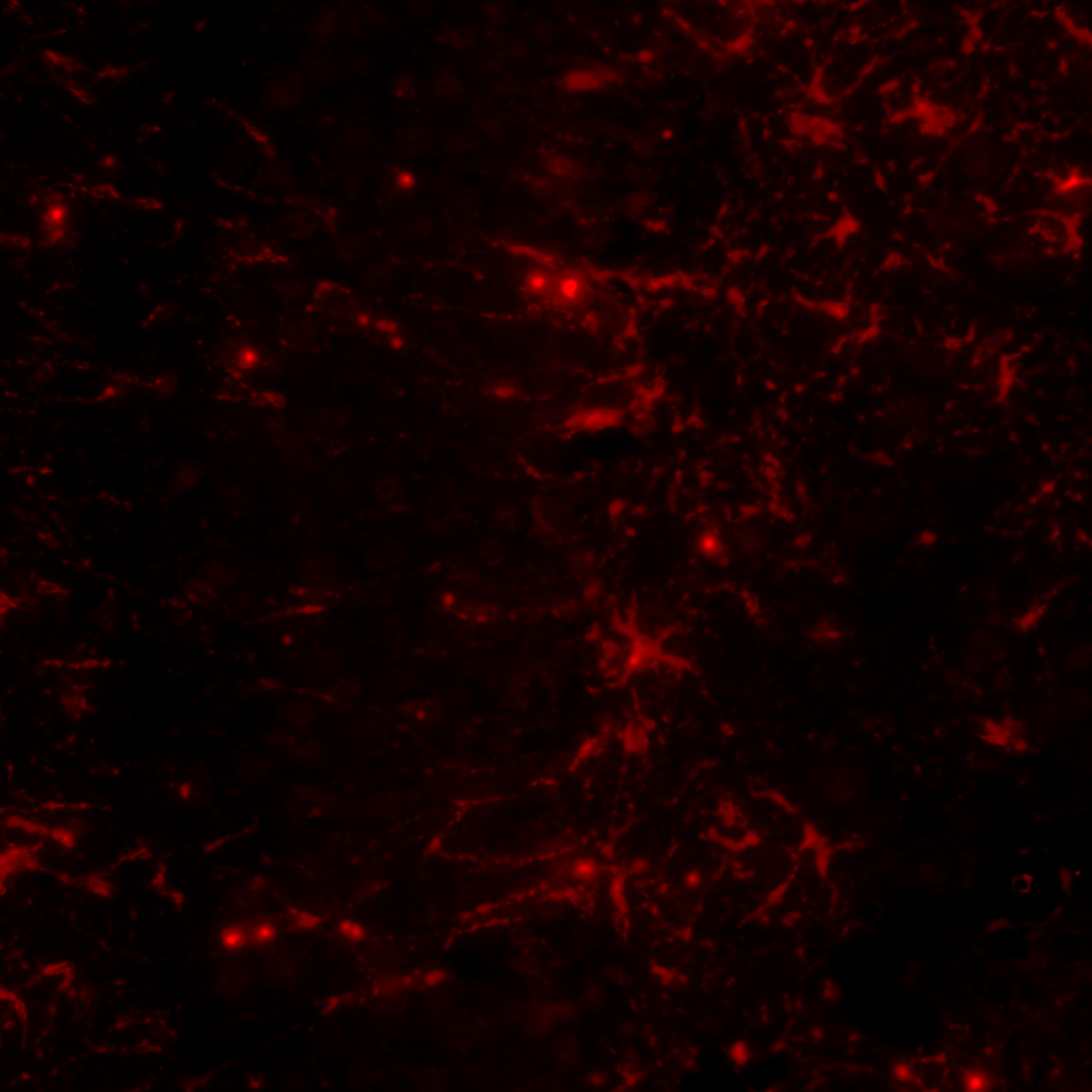

Supplement: Figure 3—figure supplement 1—source data 1. [file elife-86940-fig3-figsupp1-data1.zip › Figure 3-figure supplement 1-source data 1/2881-CKO-RX CII FF-1M-40X-GFAP-NESTIN-#59-1-vHPC-Image Export-13_AF594.tif]

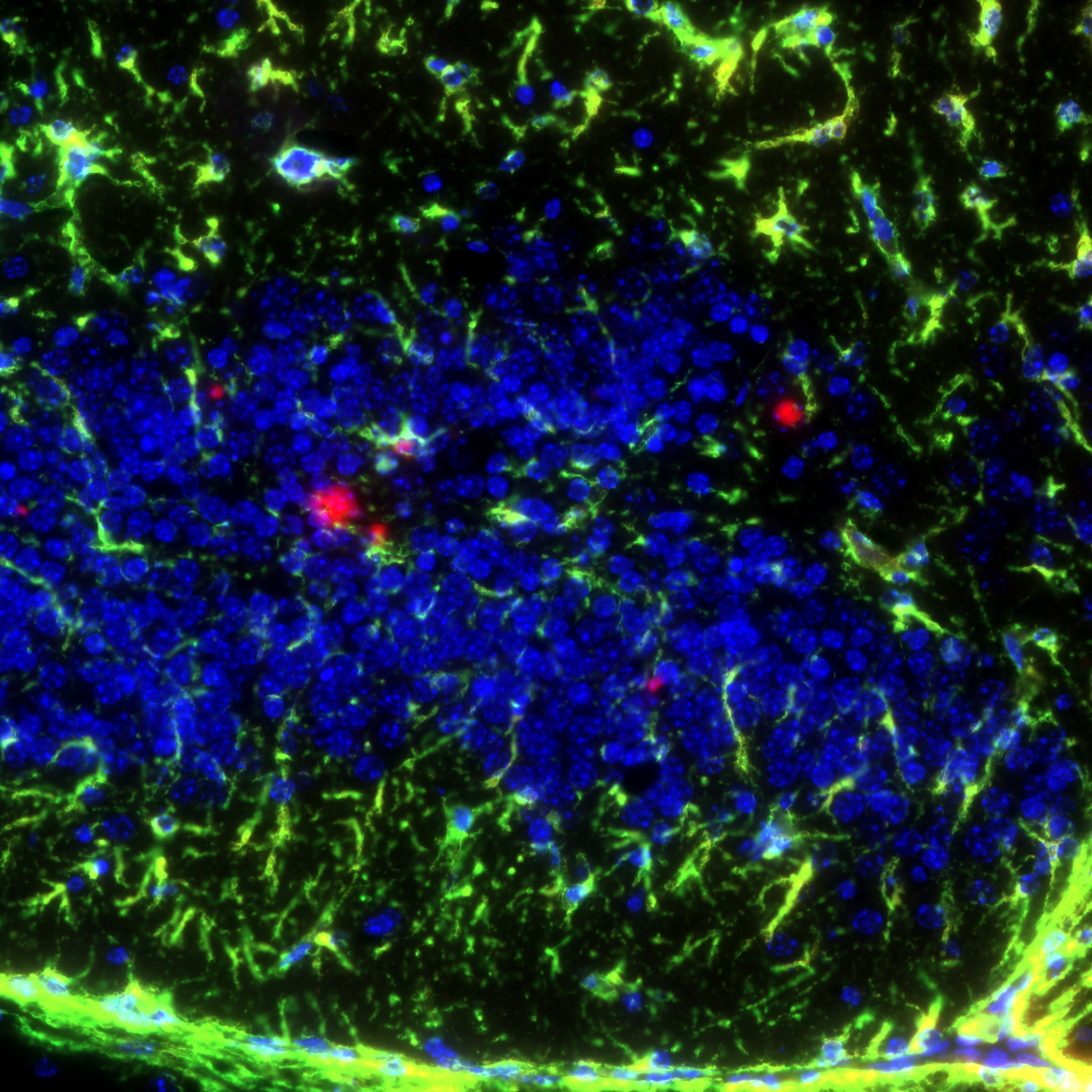

Supplement: Figure 3—figure supplement 1—source data 1. [file elife-86940-fig3-figsupp1-data1.zip › Figure 3-figure supplement 1-source data 1/F449-1-DKO-RX CI CII ff FF-P18-40X-GFAP-NESTIN-#89-1-HPC-R-Image Export-7.tif]

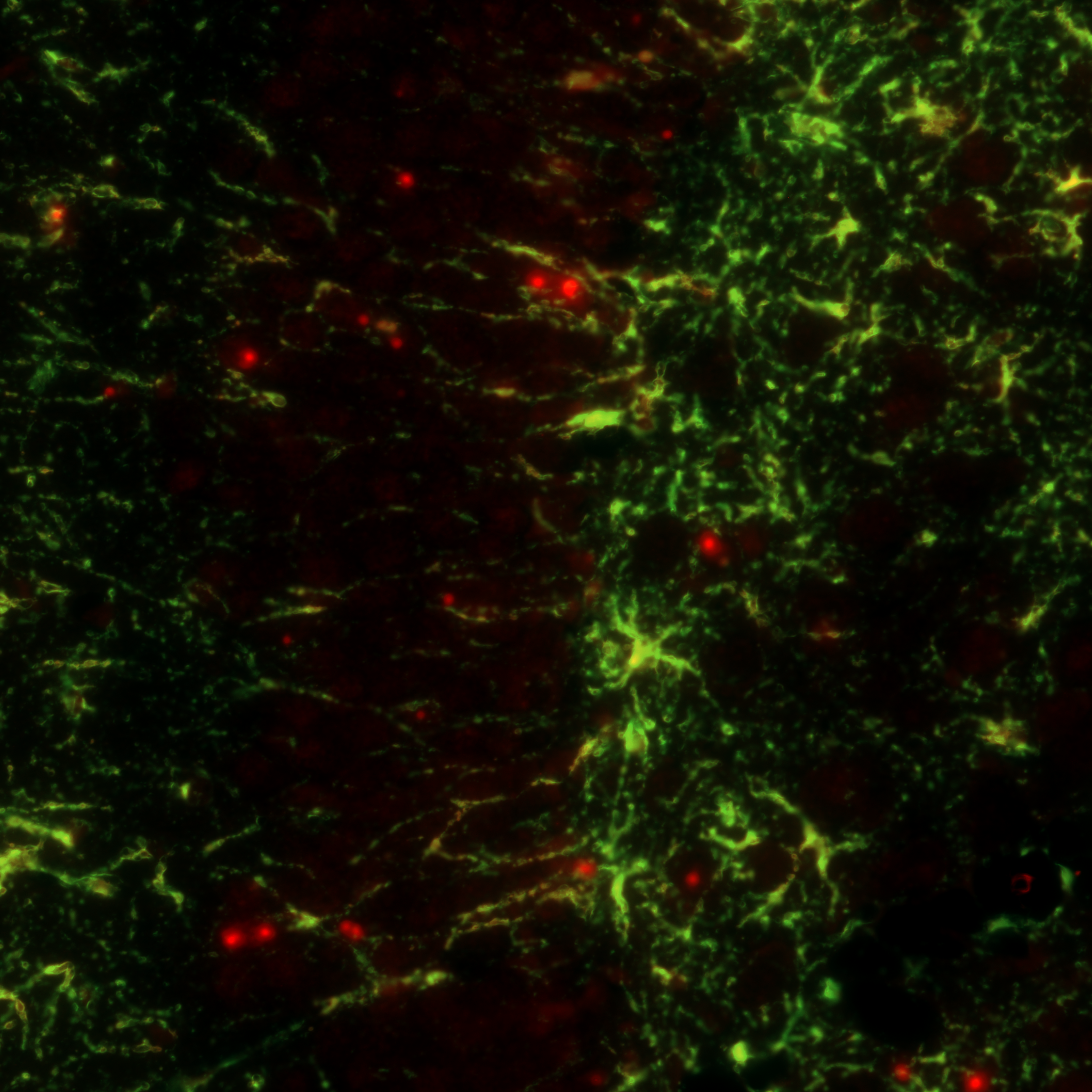

Supplement: Figure 3—figure supplement 1—source data 1. [file elife-86940-fig3-figsupp1-data1.zip › Figure 3-figure supplement 1-source data 1/2881-CKO-RX CII FF-1M-40X-GFAP-NESTIN-#59-1-vHPC-Image Export-13.tif]

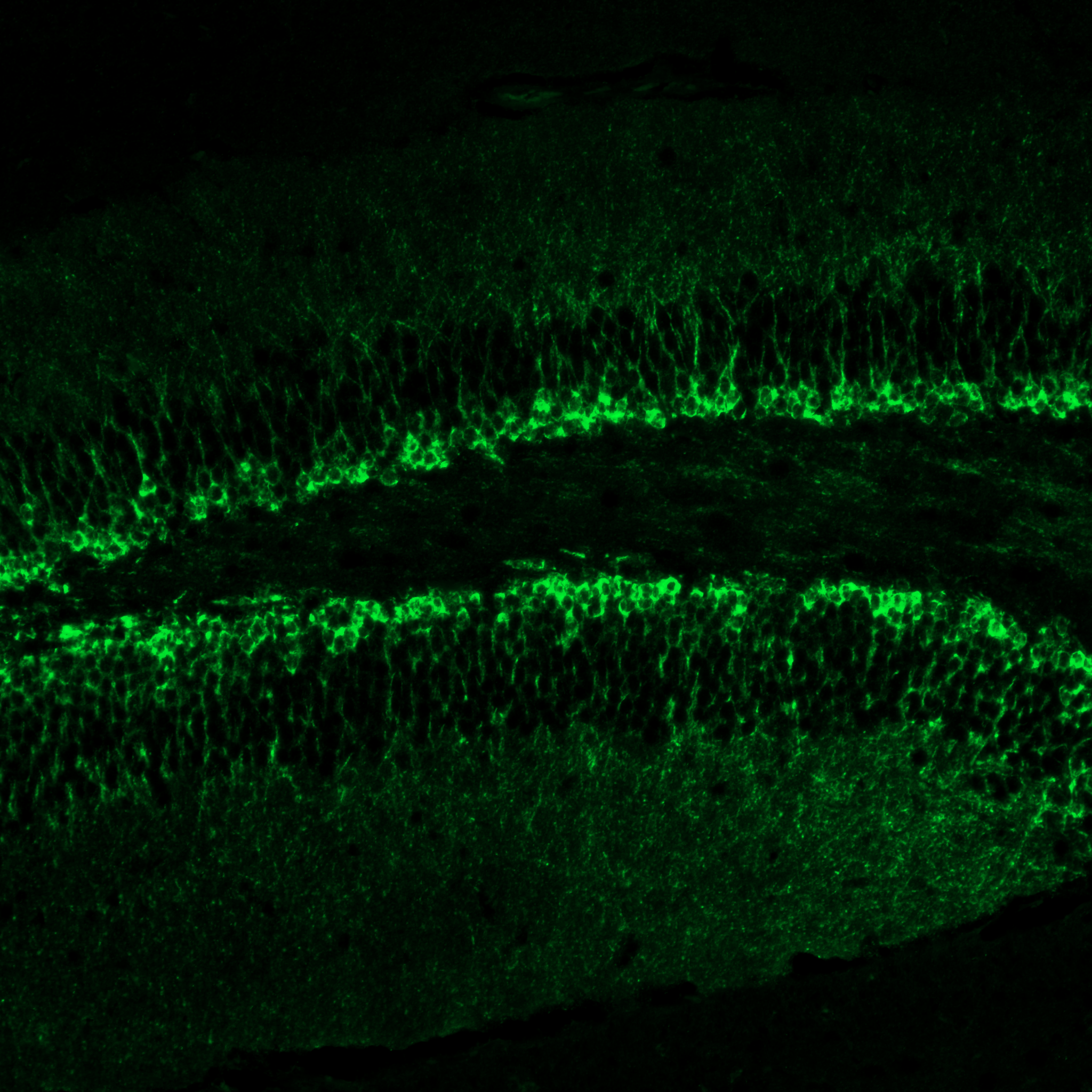

Supplement: Figure 3—figure supplement 1—source data 2. [file elife-86940-fig3-figsupp1-data2.zip › Figure 3-figure supplement 1-source data 2/F3094-2-CI CON-RX CI f+-1M-20X-DCX-34-2-dDG-Image Export-14_AF488.tif]

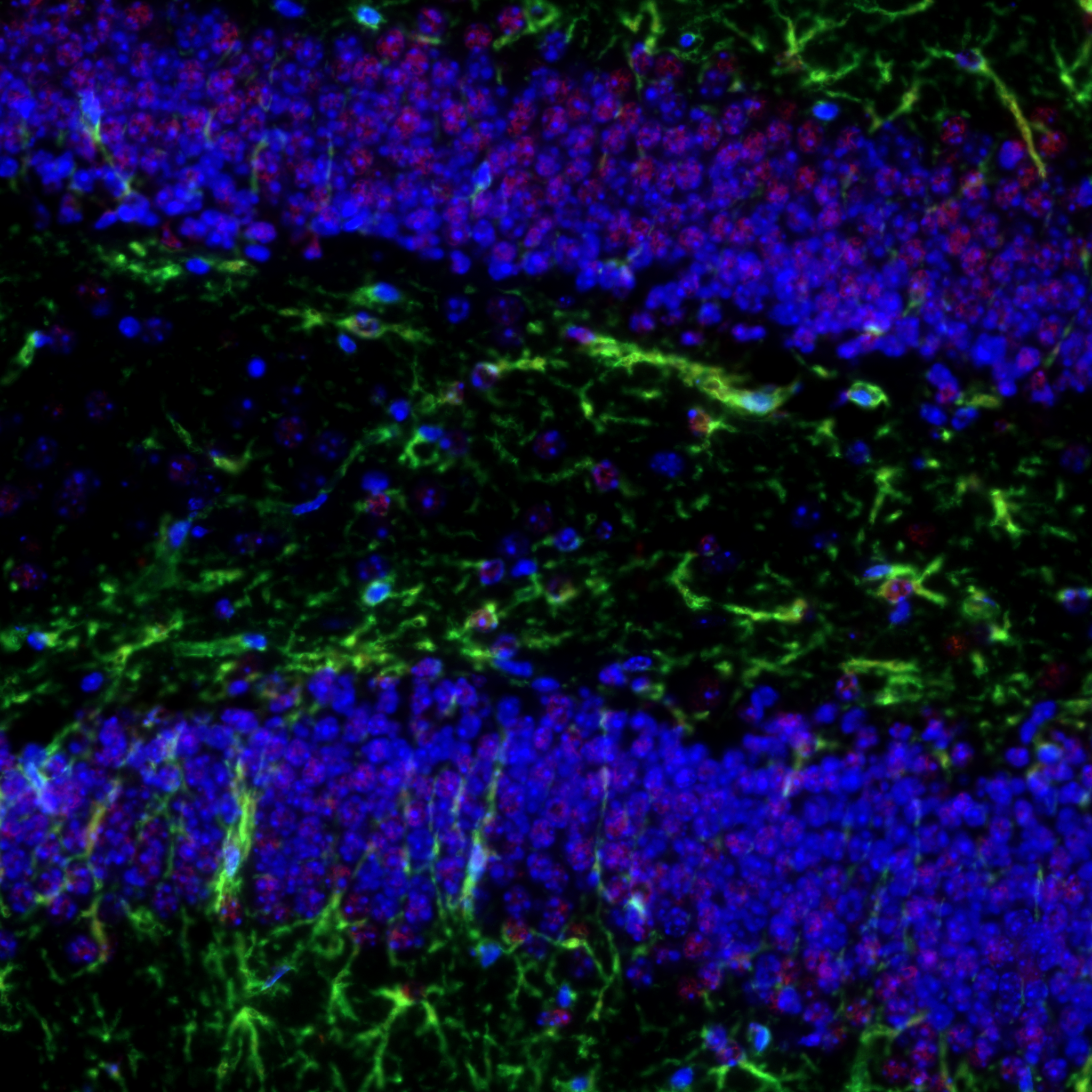

Supplement: Figure 3—figure supplement 1—source data 2. [file elife-86940-fig3-figsupp1-data2.zip › Figure 3-figure supplement 1-source data 2/F448-2-CON-CI CII f+ F+-P18-40X-GFAP-NESTIN-97-3-HPC-L-Image Export-31.tif]
