# Supplementary figures and images for: The differentiation and integration of the hippocampal dorsoventral axis are controlled by two nuclear receptor genes (part 4 of 6)
Source: eLife. 2023 Sep 26;12:RP86940. doi: 10.7554/eLife.86940 (PMC10522401; doi:10.7554/eLife.86940)

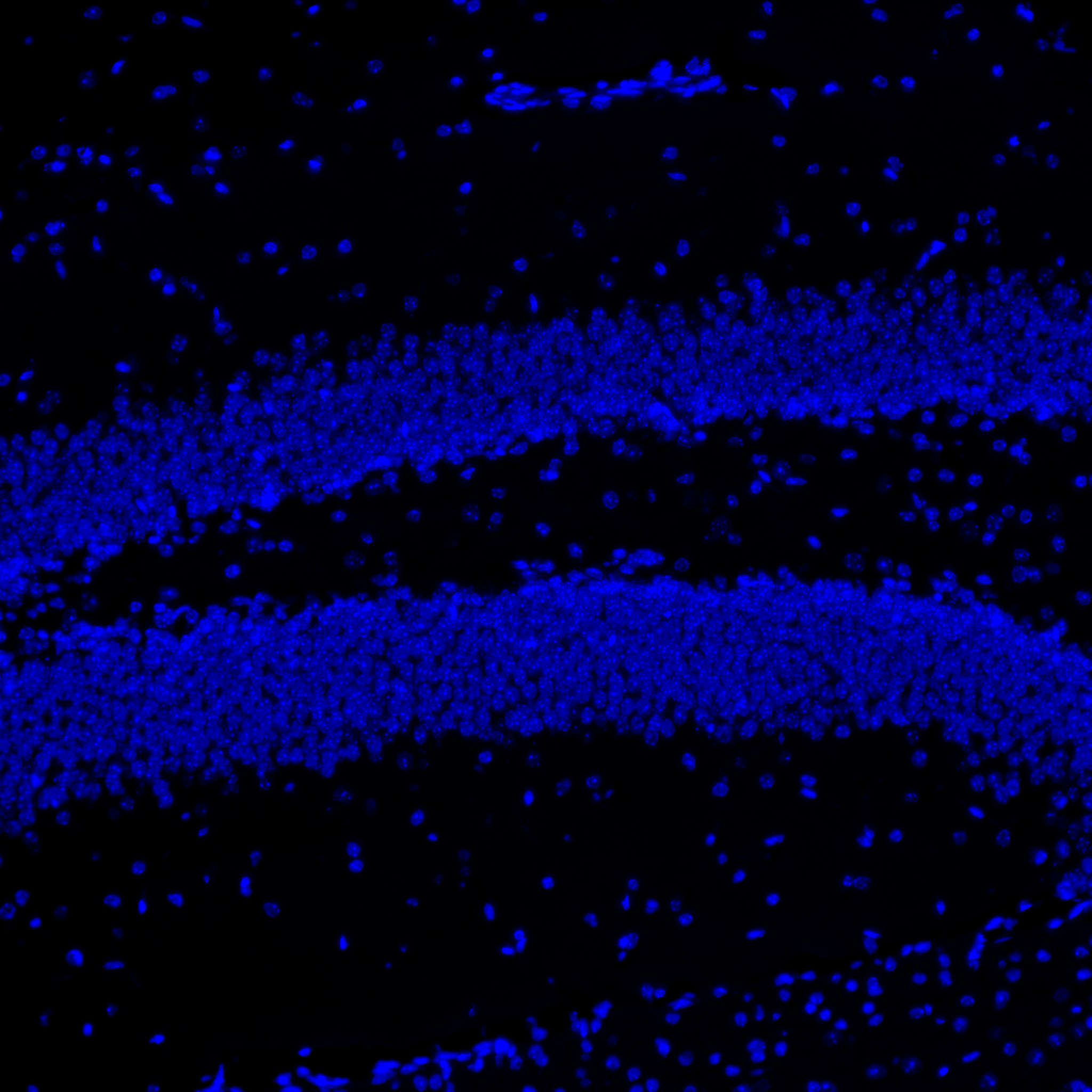

Supplement: Figure 3—figure supplement 1—source data 2. [file elife-86940-fig3-figsupp1-data2.zip › Figure 3-figure supplement 1-source data 2/F3094-2-CI CON-RX CI f+-1M-20X-DCX-34-2-dDG-Image Export-14_DAPI.tif]

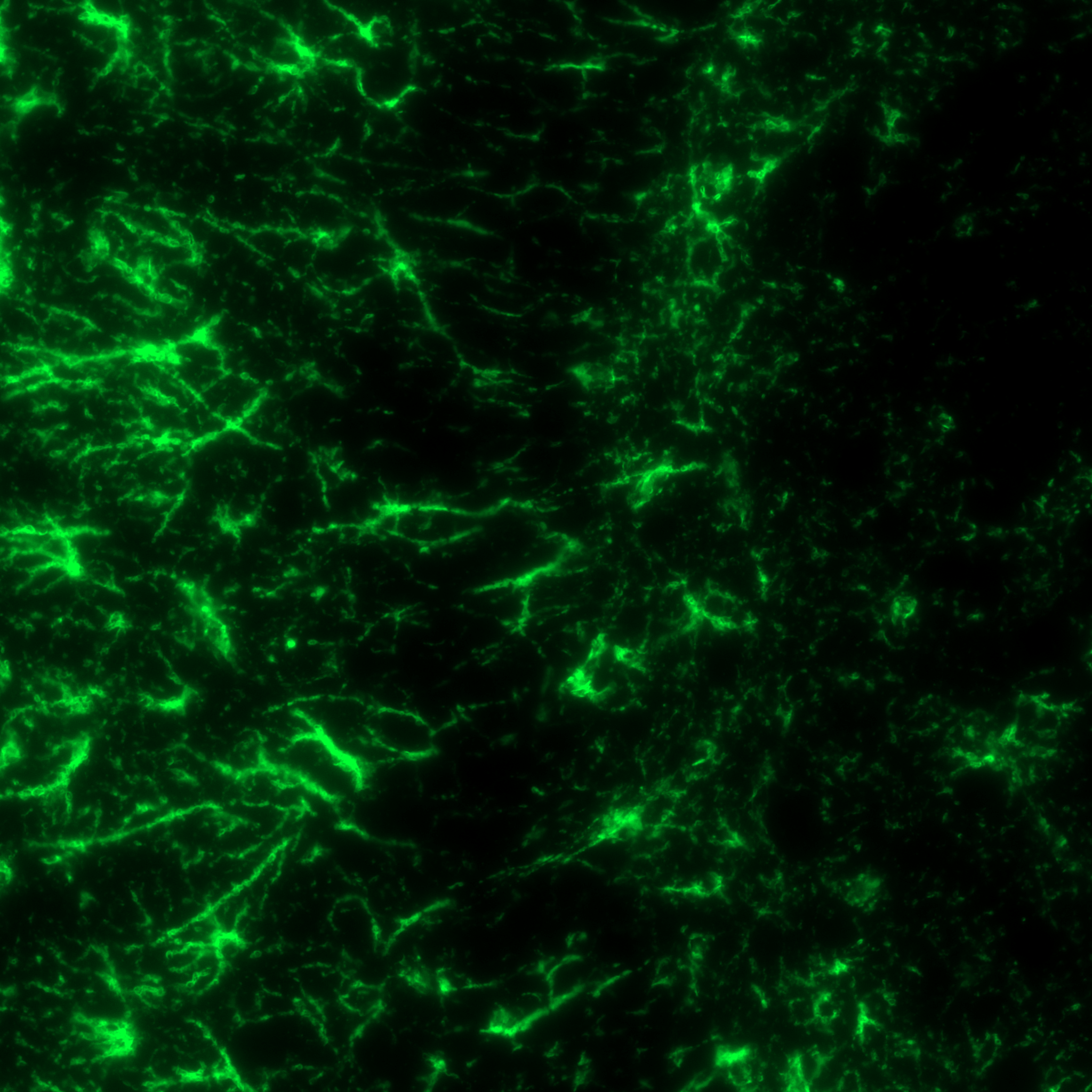

Supplement: Figure 3—figure supplement 1—source data 2. [file elife-86940-fig3-figsupp1-data2.zip › Figure 3-figure supplement 1-source data 2/2879-CON-CII FF-1M-40X-GFAP-NESTIN-#62-1-vHPC-G+R-Image Export-10_AF488.tif]

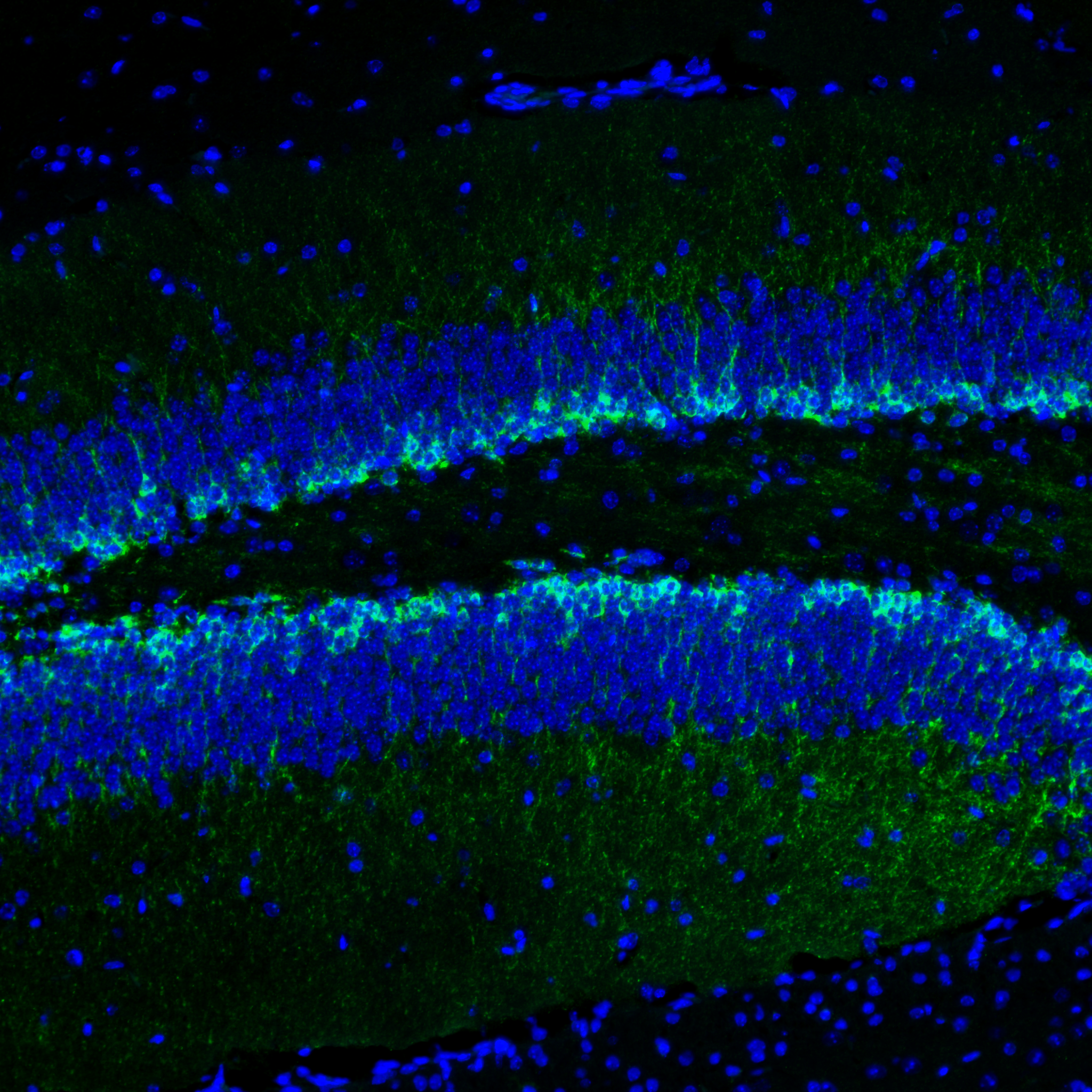

Supplement: Figure 3—figure supplement 1—source data 2. [file elife-86940-fig3-figsupp1-data2.zip › Figure 3-figure supplement 1-source data 2/F3094-2-CI CON-RX CI f+-1M-20X-DCX-34-2-dDG-Image Export-14_G+D.tif]

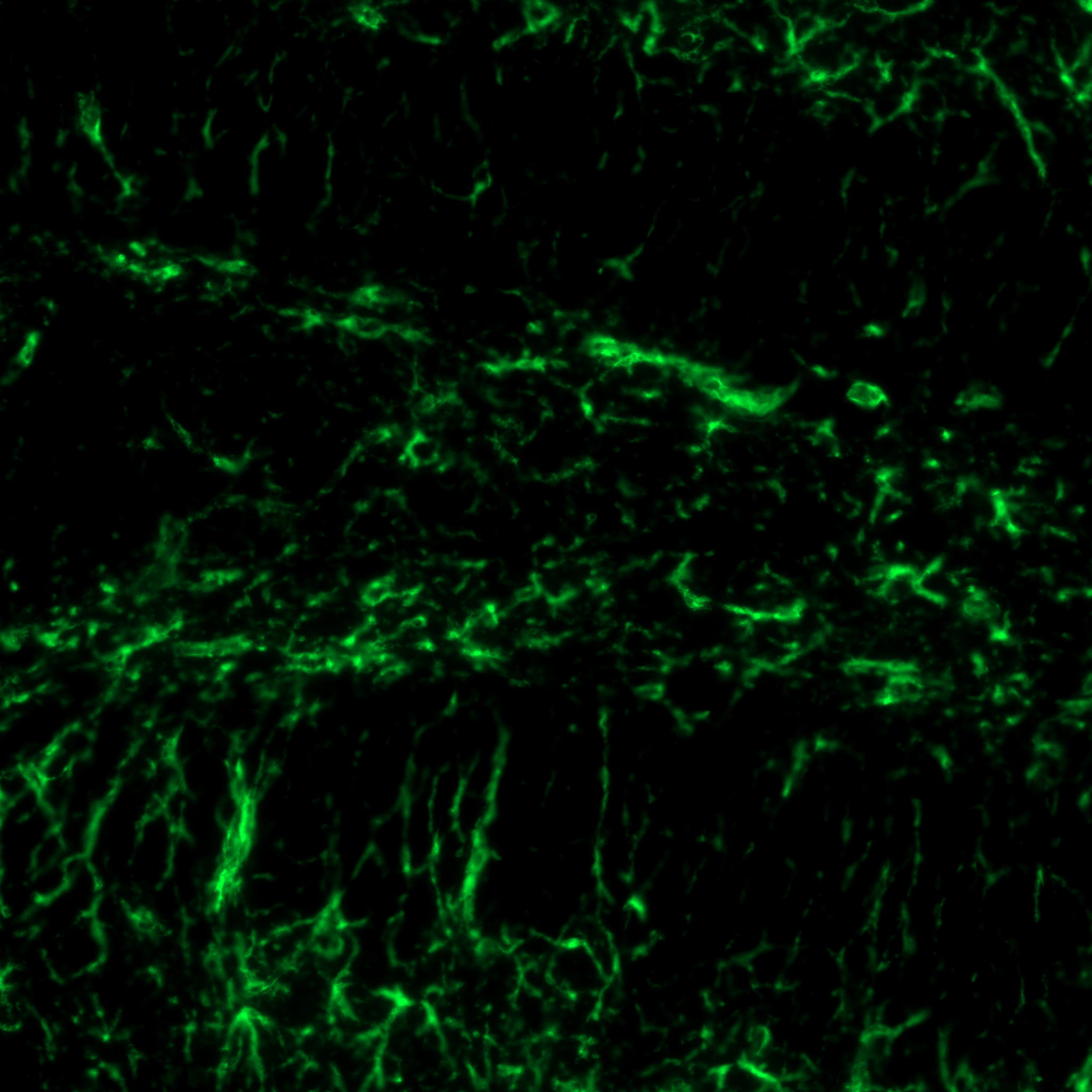

Supplement: Figure 3—figure supplement 1—source data 2. [file elife-86940-fig3-figsupp1-data2.zip › Figure 3-figure supplement 1-source data 2/F448-2-CON-CI CII f+ F+-P18-40X-GFAP-NESTIN-97-3-HPC-L-Image Export-31_AF488.tif]

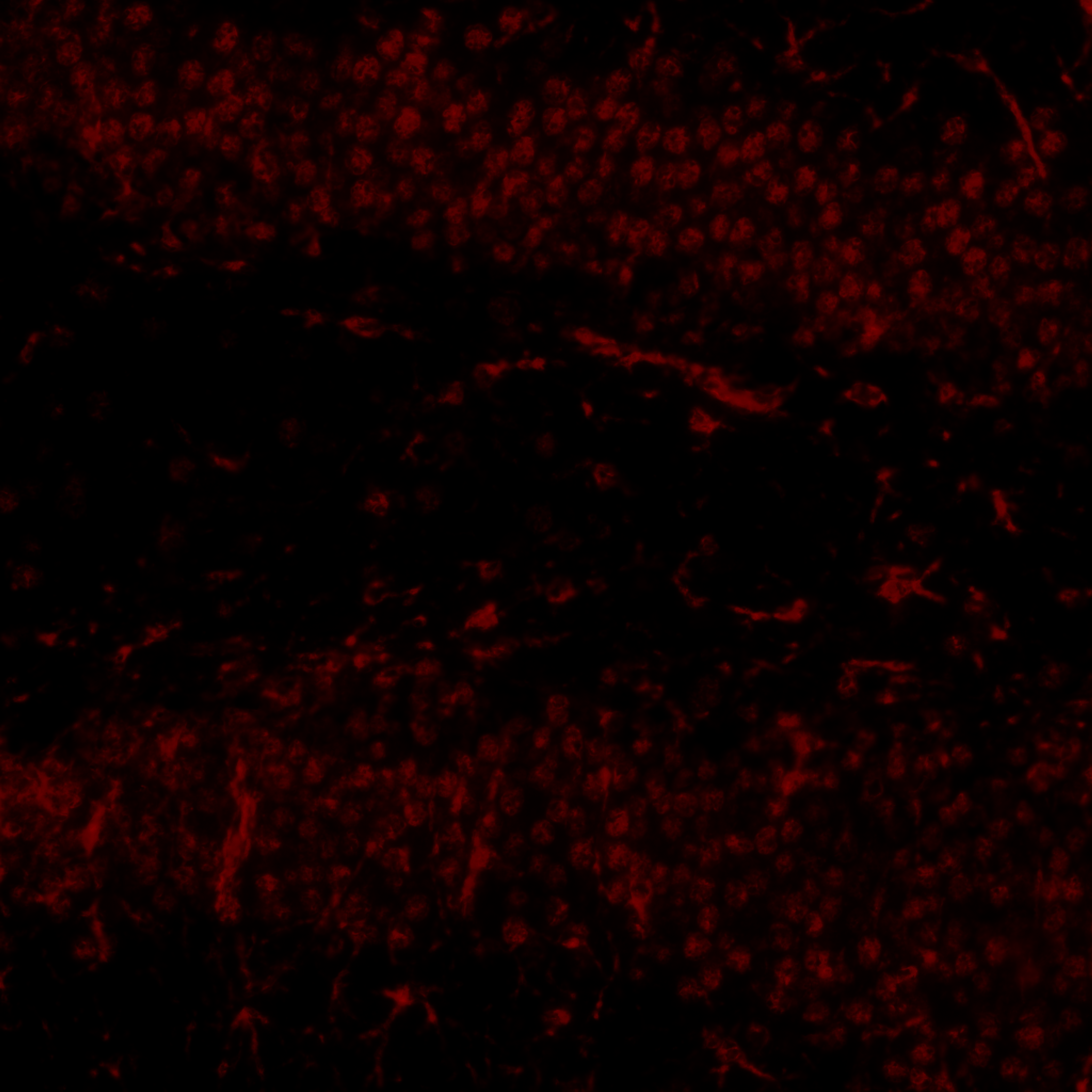

Supplement: Figure 3—figure supplement 1—source data 2. [file elife-86940-fig3-figsupp1-data2.zip › Figure 3-figure supplement 1-source data 2/F448-2-CON-CI CII f+ F+-P18-40X-GFAP-NESTIN-97-3-HPC-L-Image Export-31_AF594.tif]

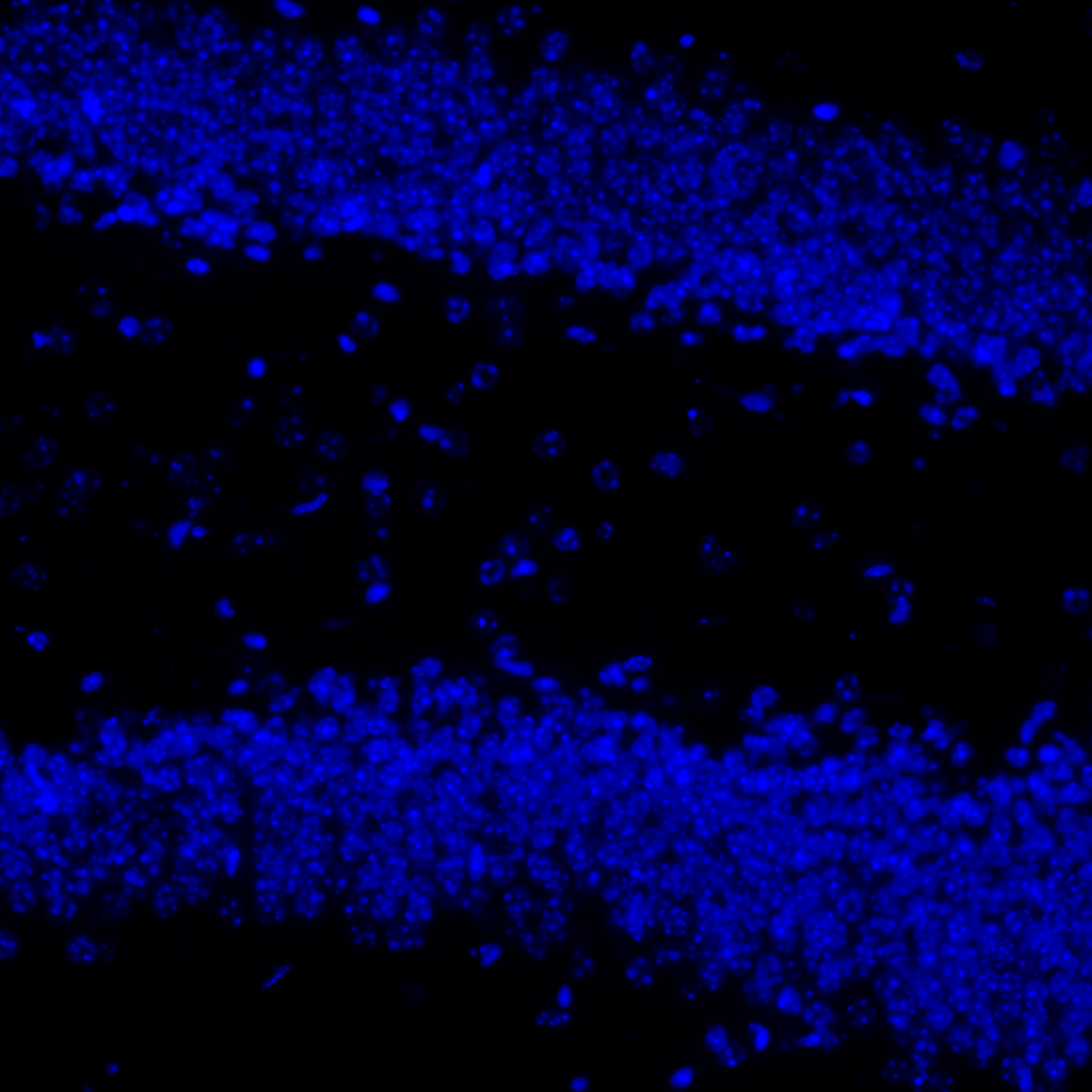

Supplement: Figure 3—figure supplement 1—source data 2. [file elife-86940-fig3-figsupp1-data2.zip › Figure 3-figure supplement 1-source data 2/F448-2-CON-CI CII f+ F+-P18-40X-GFAP-NESTIN-97-3-HPC-L-Image Export-31_DAPI.tif]

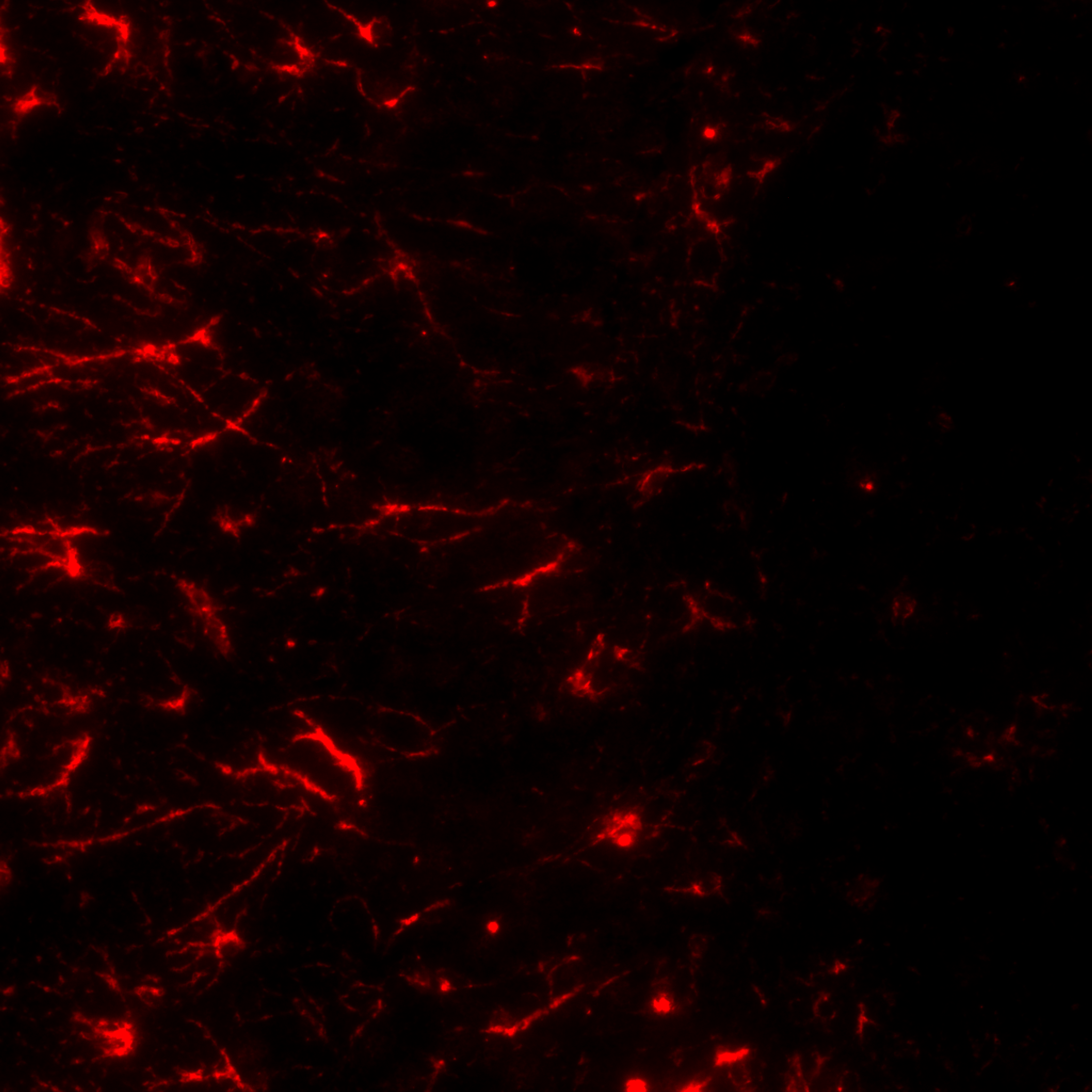

Supplement: Figure 3—figure supplement 1—source data 2. [file elife-86940-fig3-figsupp1-data2.zip › Figure 3-figure supplement 1-source data 2/2879-CON-CII FF-1M-40X-GFAP-NESTIN-#62-1-vHPC-G+R-Image Export-10_AF594.tif]

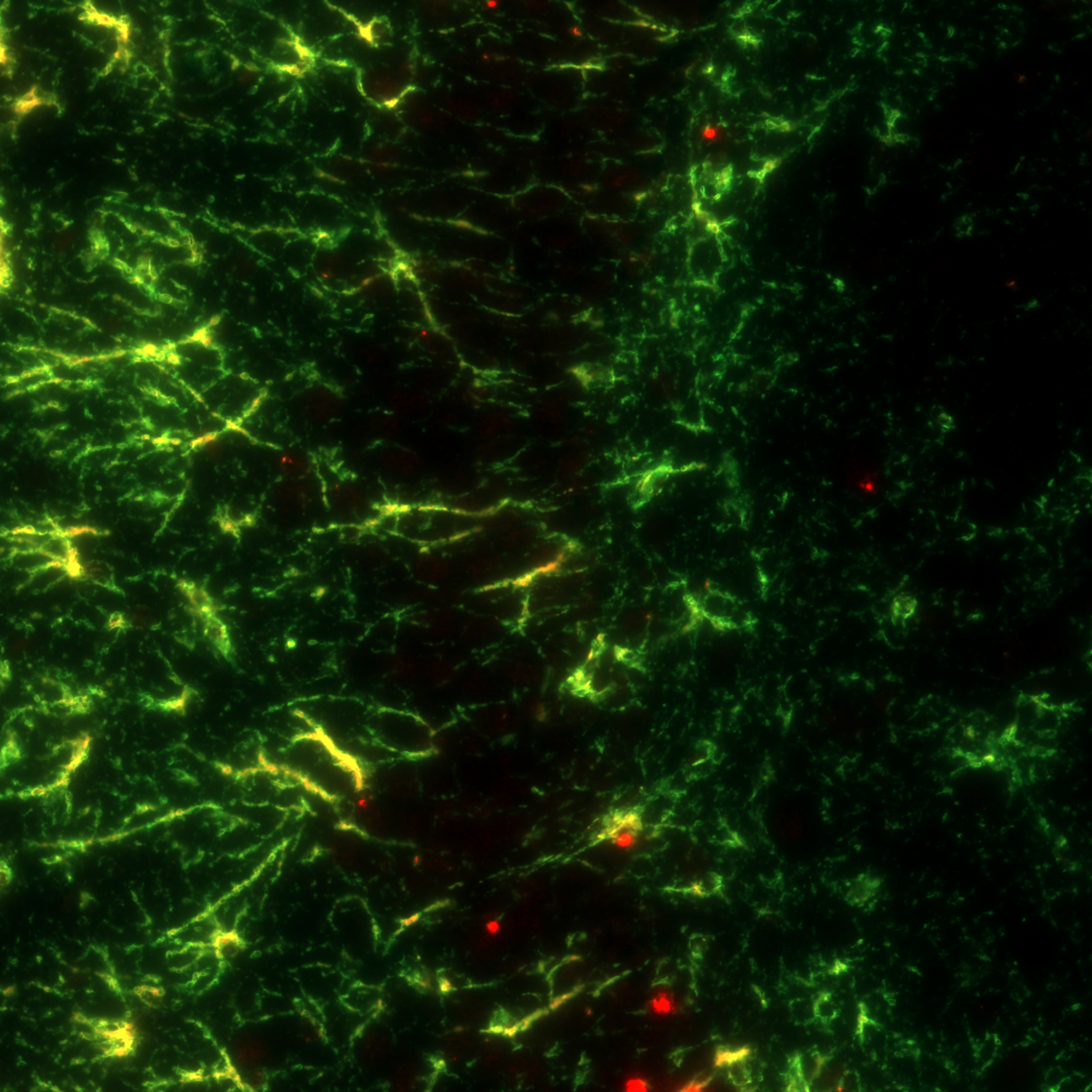

Supplement: Figure 3—figure supplement 1—source data 2. [file elife-86940-fig3-figsupp1-data2.zip › Figure 3-figure supplement 1-source data 2/2879-CON-CII FF-1M-40X-GFAP-NESTIN-#62-1-vHPC-G+R-Image Export-10.tif]

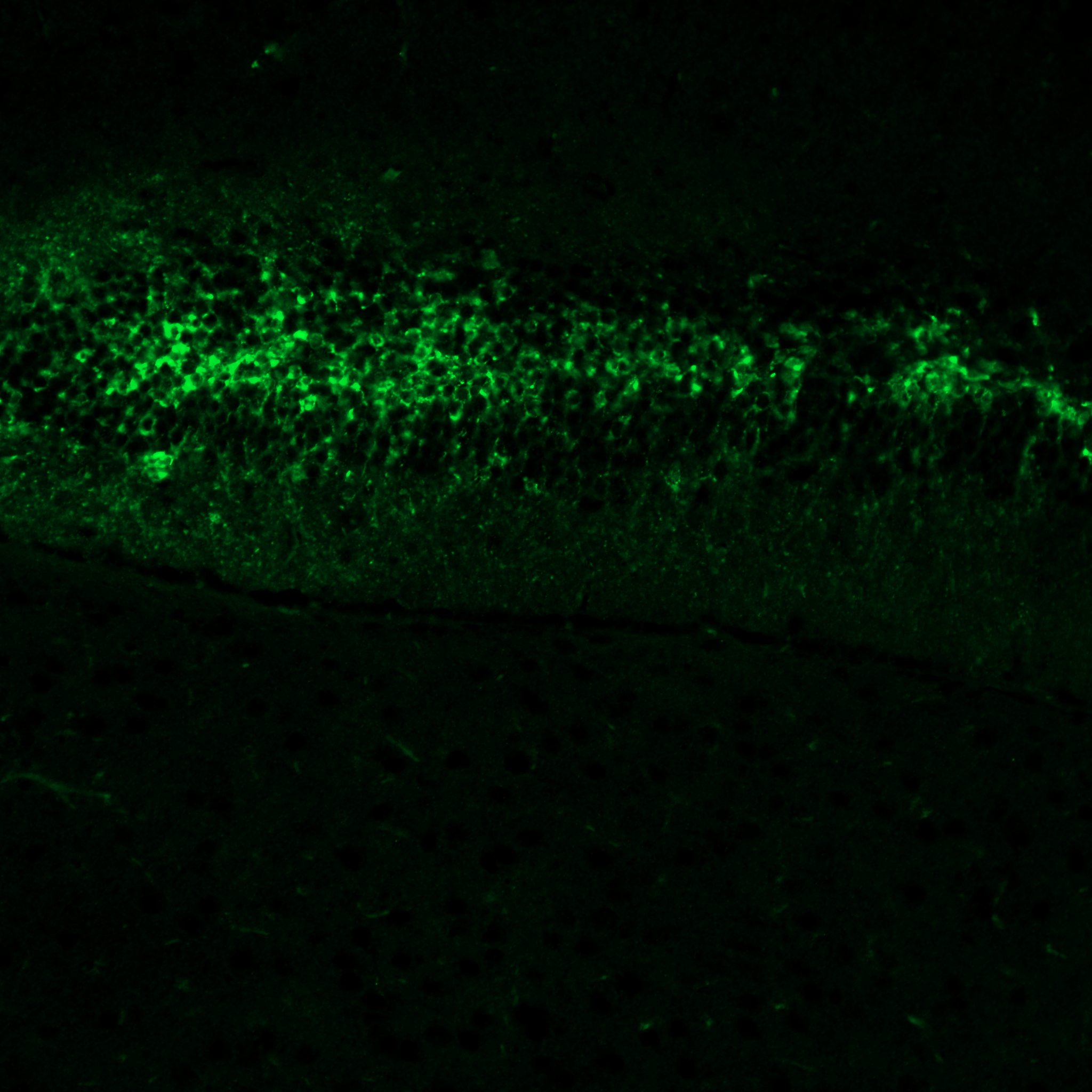

Supplement: Figure 3—figure supplement 1—source data 2. [file elife-86940-fig3-figsupp1-data2.zip › Figure 3-figure supplement 1-source data 2/F3094-3-CI CKO-RX CI ff-1M-20X-DCX-33-2-dDG-Image Export-41_AF488.tif]

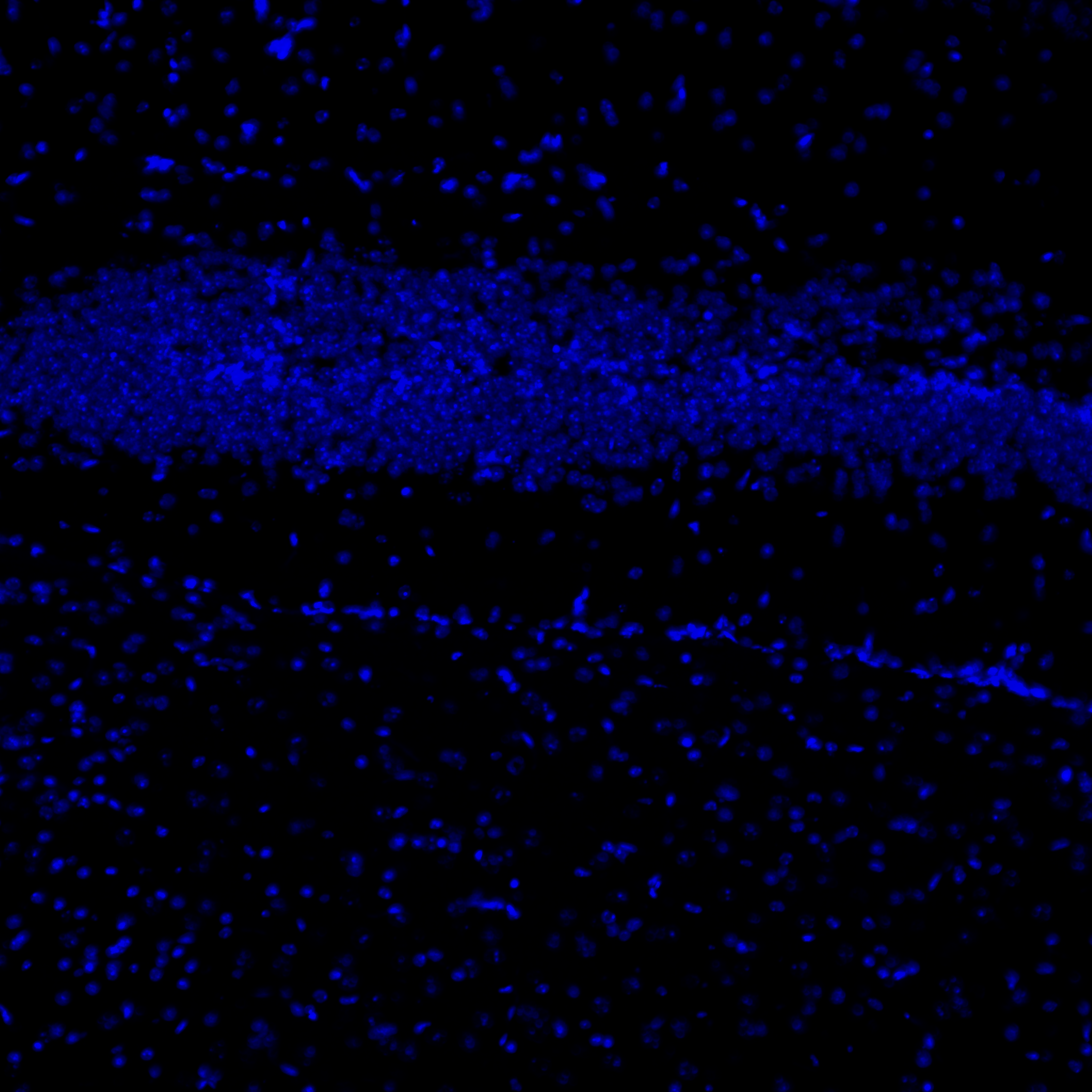

Supplement: Figure 3—figure supplement 1—source data 2. [file elife-86940-fig3-figsupp1-data2.zip › Figure 3-figure supplement 1-source data 2/F3094-3-CI CKO-RX CI ff-1M-20X-DCX-33-2-dDG-Image Export-41_DAPI.tif]

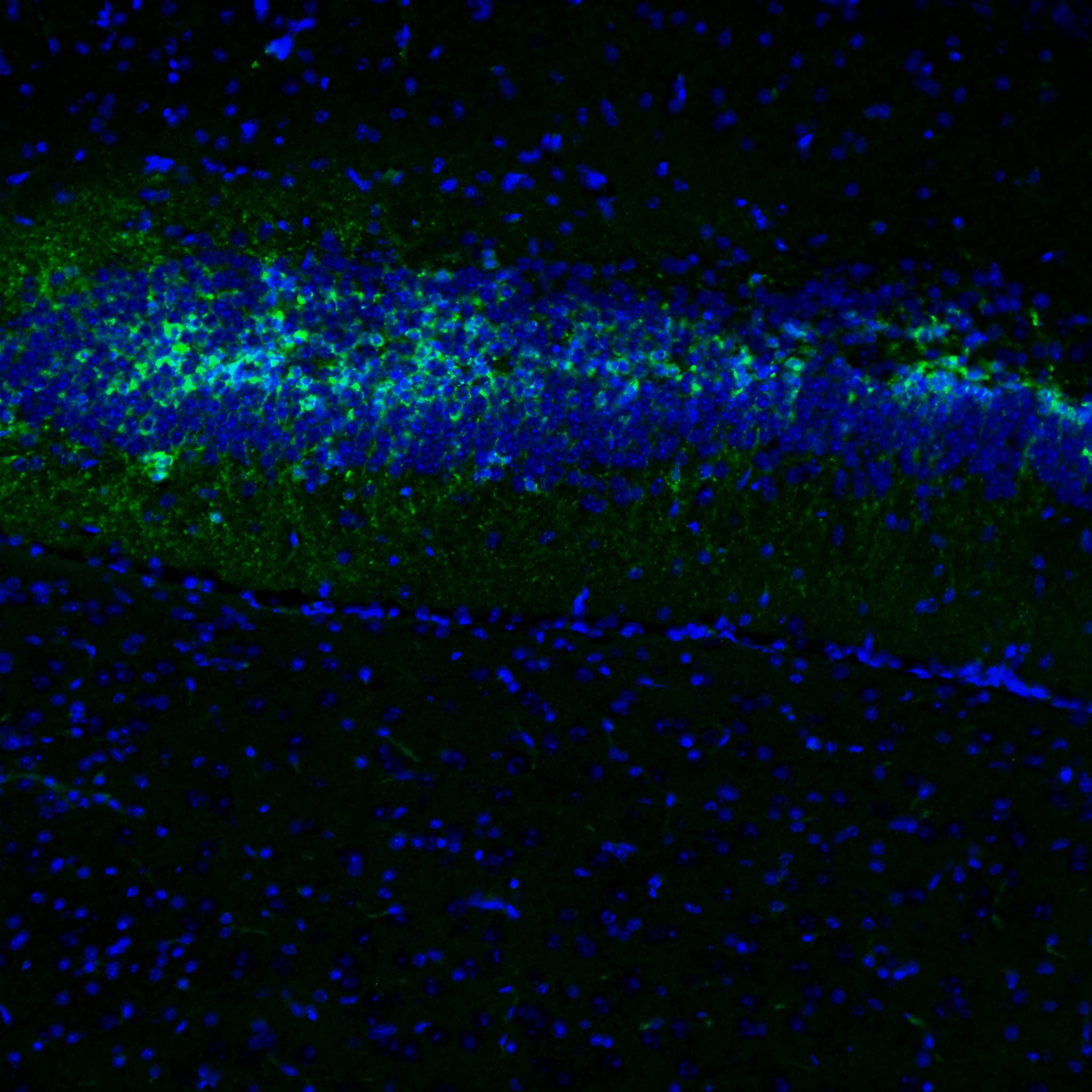

Supplement: Figure 3—figure supplement 1—source data 2. [file elife-86940-fig3-figsupp1-data2.zip › Figure 3-figure supplement 1-source data 2/F3094-3-CI CKO-RX CI ff-1M-20X-DCX-33-2-dDG-Image Export-41_G+D.tif]

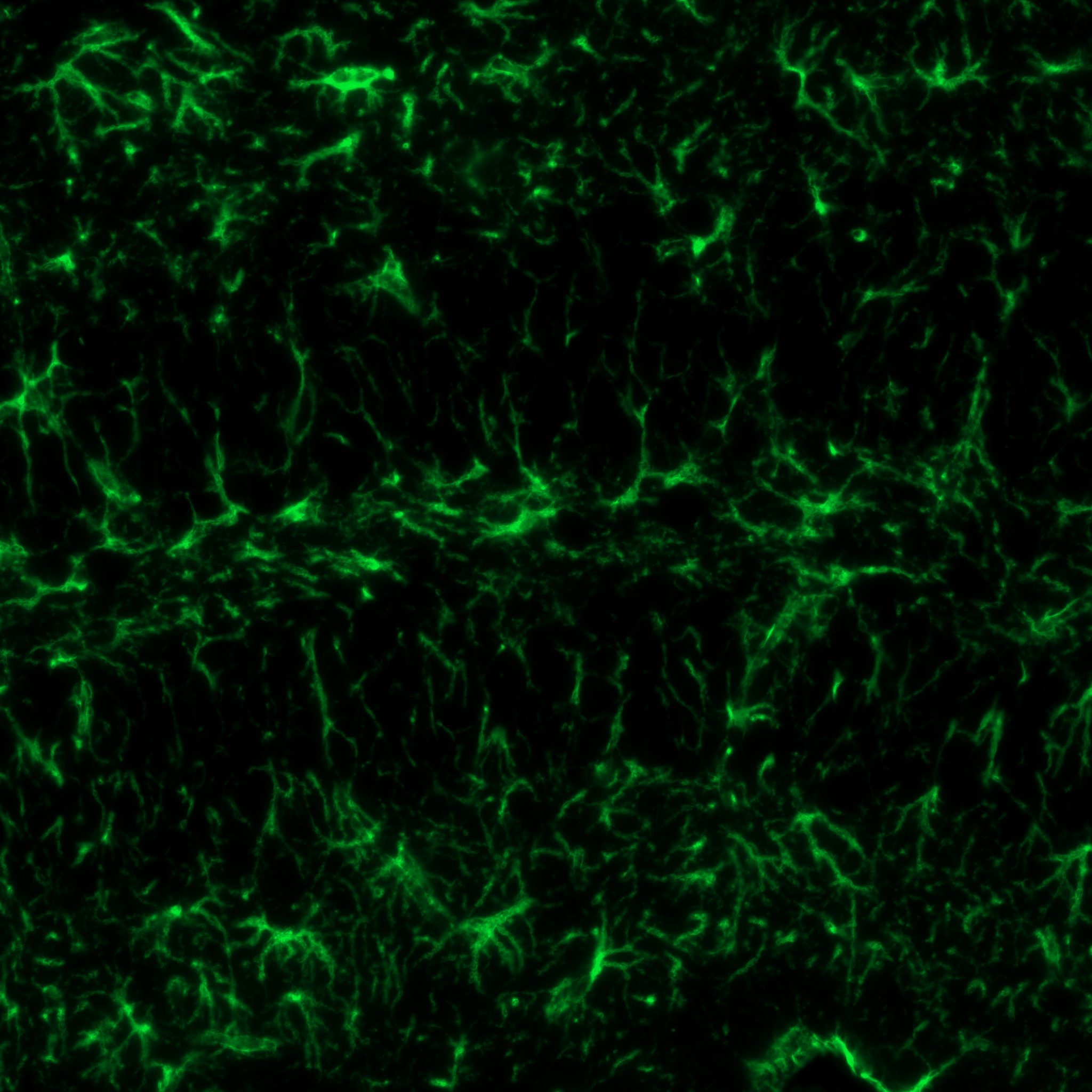

Supplement: Figure 3—figure supplement 1—source data 2. [file elife-86940-fig3-figsupp1-data2.zip › Figure 3-figure supplement 1-source data 2/F3094-3-CI CKO-RX CI ff-1M-40X-GFAP-NESTIN-#128-1-dHPC-G+R-Image Export-20_AF488.tif]

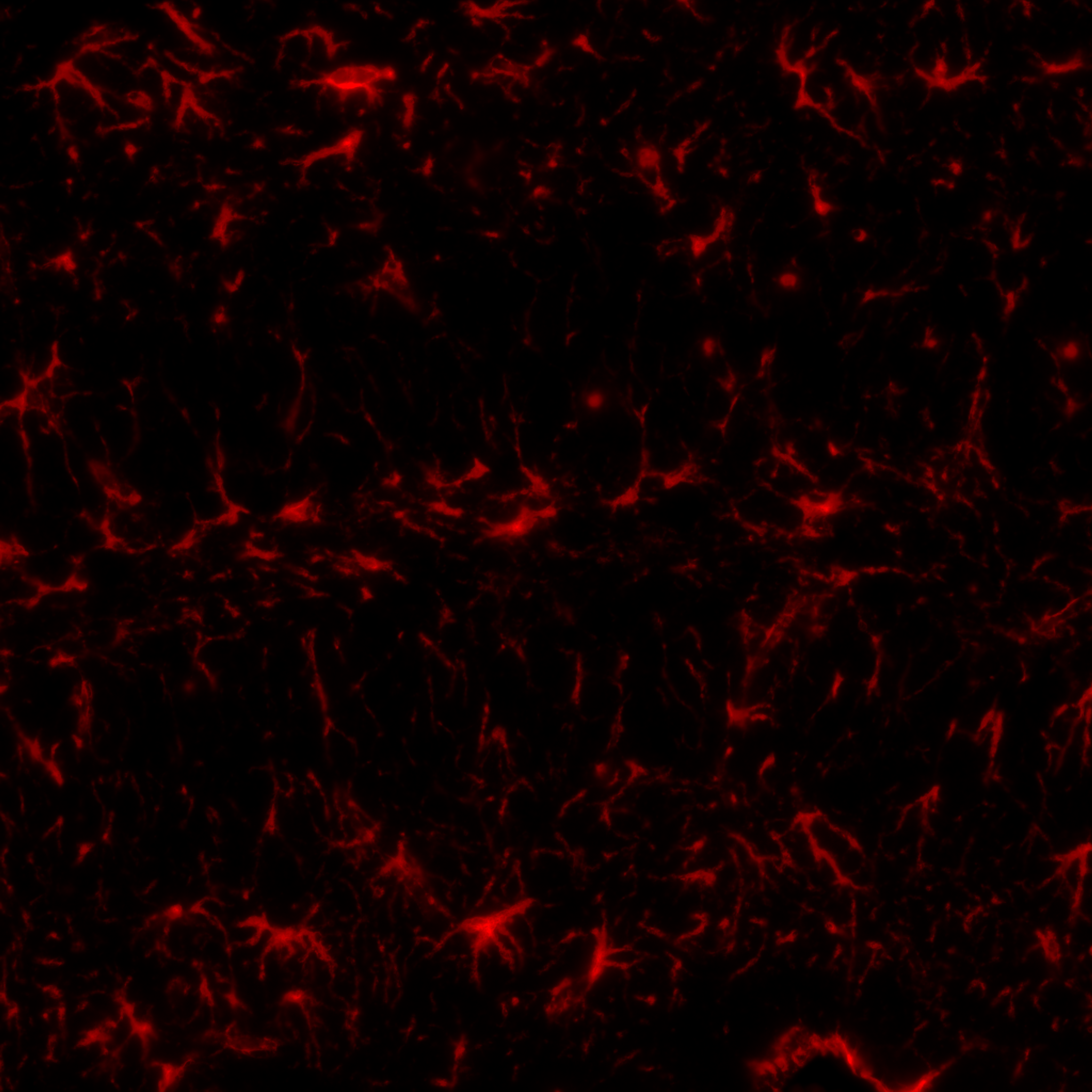

Supplement: Figure 3—figure supplement 1—source data 2. [file elife-86940-fig3-figsupp1-data2.zip › Figure 3-figure supplement 1-source data 2/F3094-3-CI CKO-RX CI ff-1M-40X-GFAP-NESTIN-#128-1-dHPC-G+R-Image Export-20_AF594.tif]

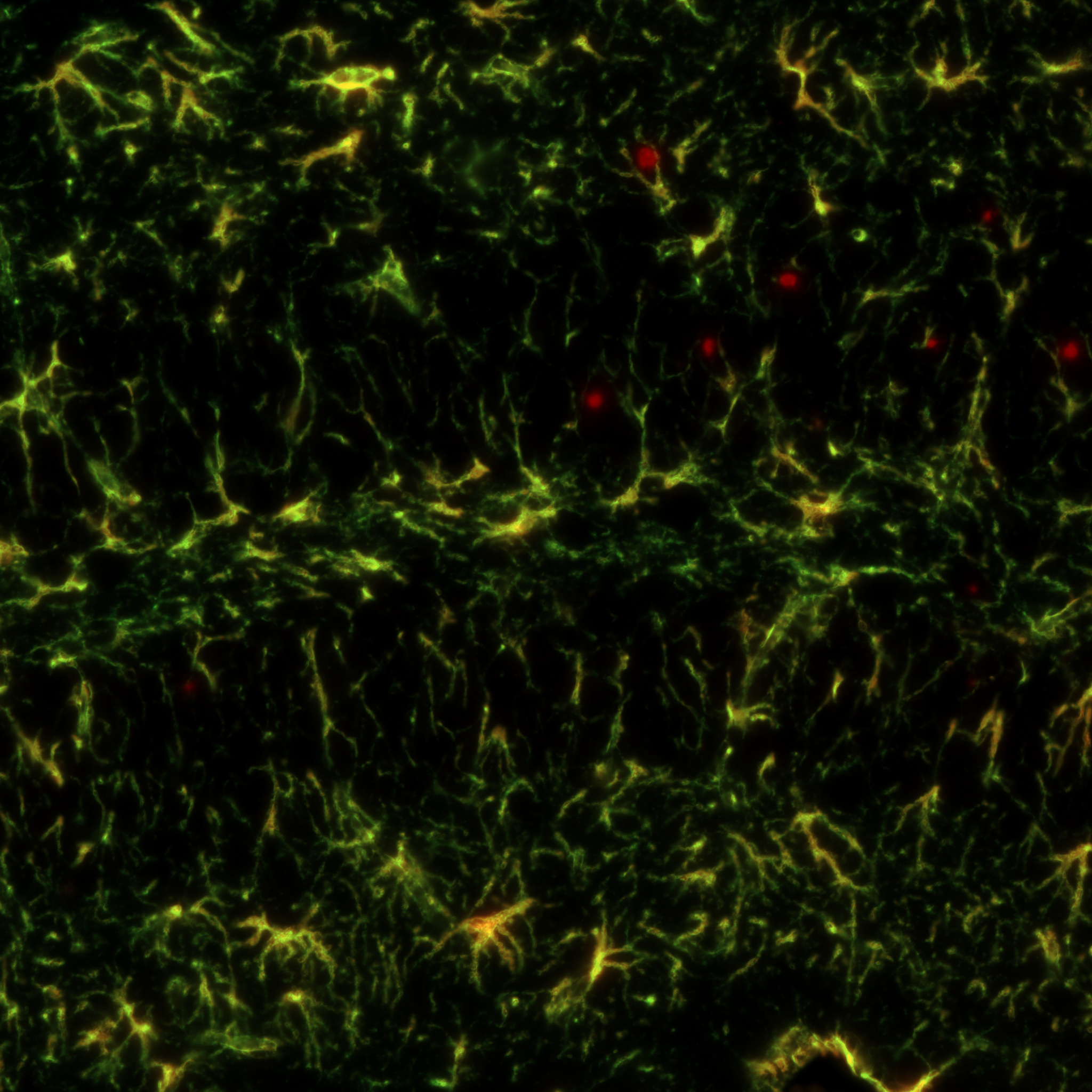

Supplement: Figure 3—figure supplement 1—source data 2. [file elife-86940-fig3-figsupp1-data2.zip › Figure 3-figure supplement 1-source data 2/F3094-3-CI CKO-RX CI ff-1M-40X-GFAP-NESTIN-#128-1-dHPC-G+R-Image Export-20.tif]

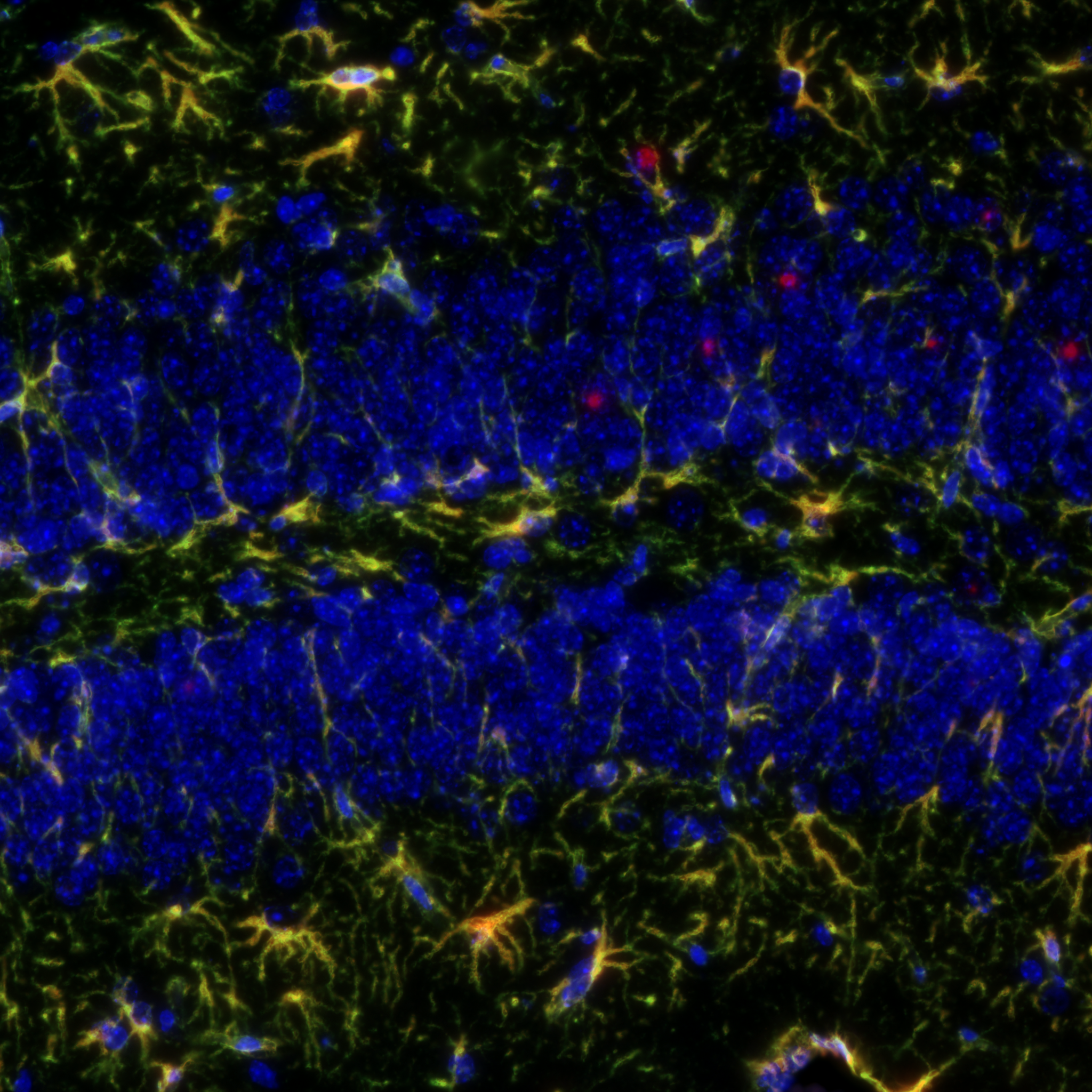

Supplement: Figure 3—figure supplement 1—source data 2. [file elife-86940-fig3-figsupp1-data2.zip › Figure 3-figure supplement 1-source data 2/F3094-3-CI CKO-RX CI ff-1M-40X-GFAP-NESTIN-#128-1-dHPC-Image Export-20.tif]

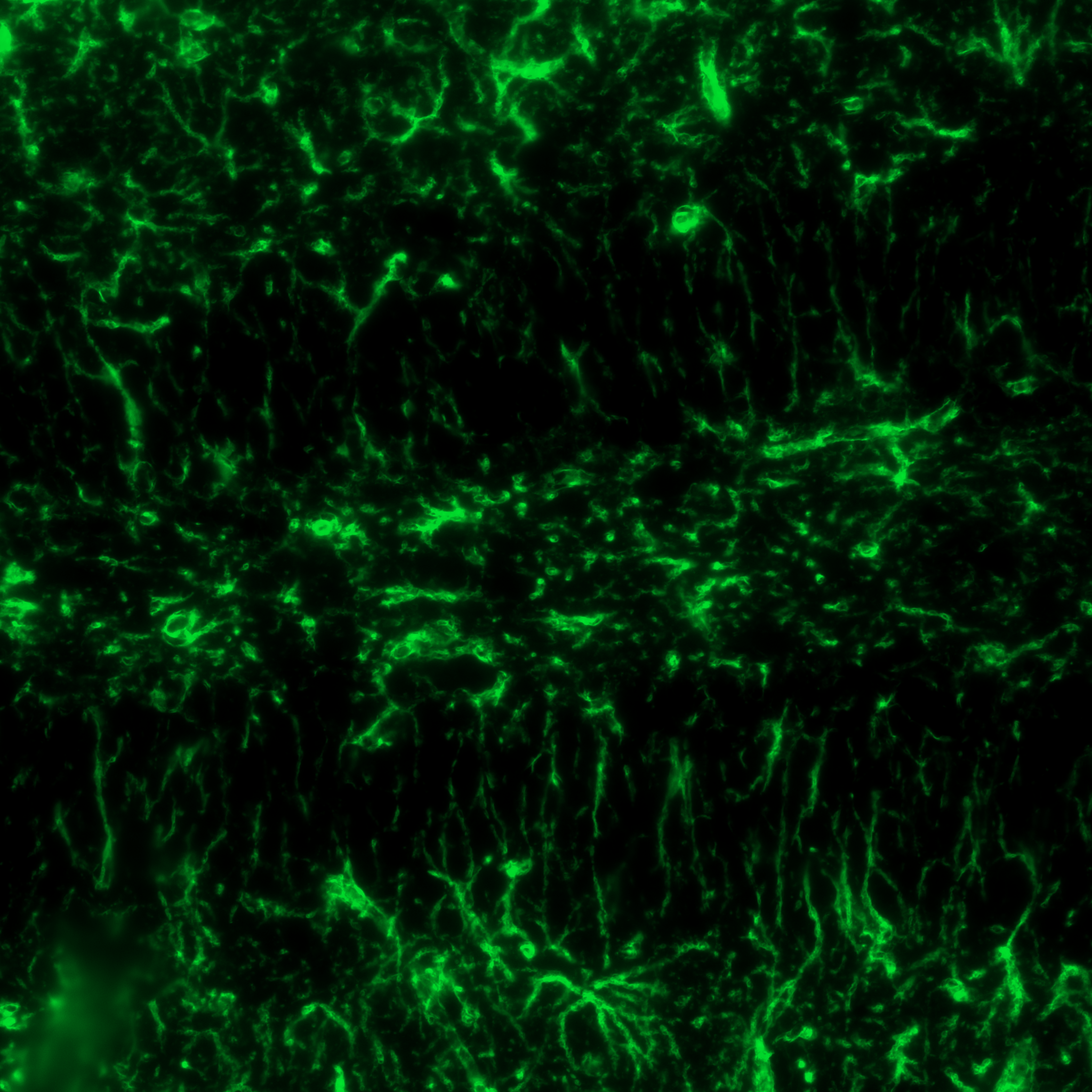

Supplement: Figure 3—figure supplement 1—source data 3. [file elife-86940-fig3-figsupp1-data3.zip › Figure 3-figure supplement 1-source data 3/F449-3-CON-CI CII f+ FF-P18-40X-GFAP-NESTIN-#83-2-HPC-R-G+R-Image Export-5_AF488.tif]

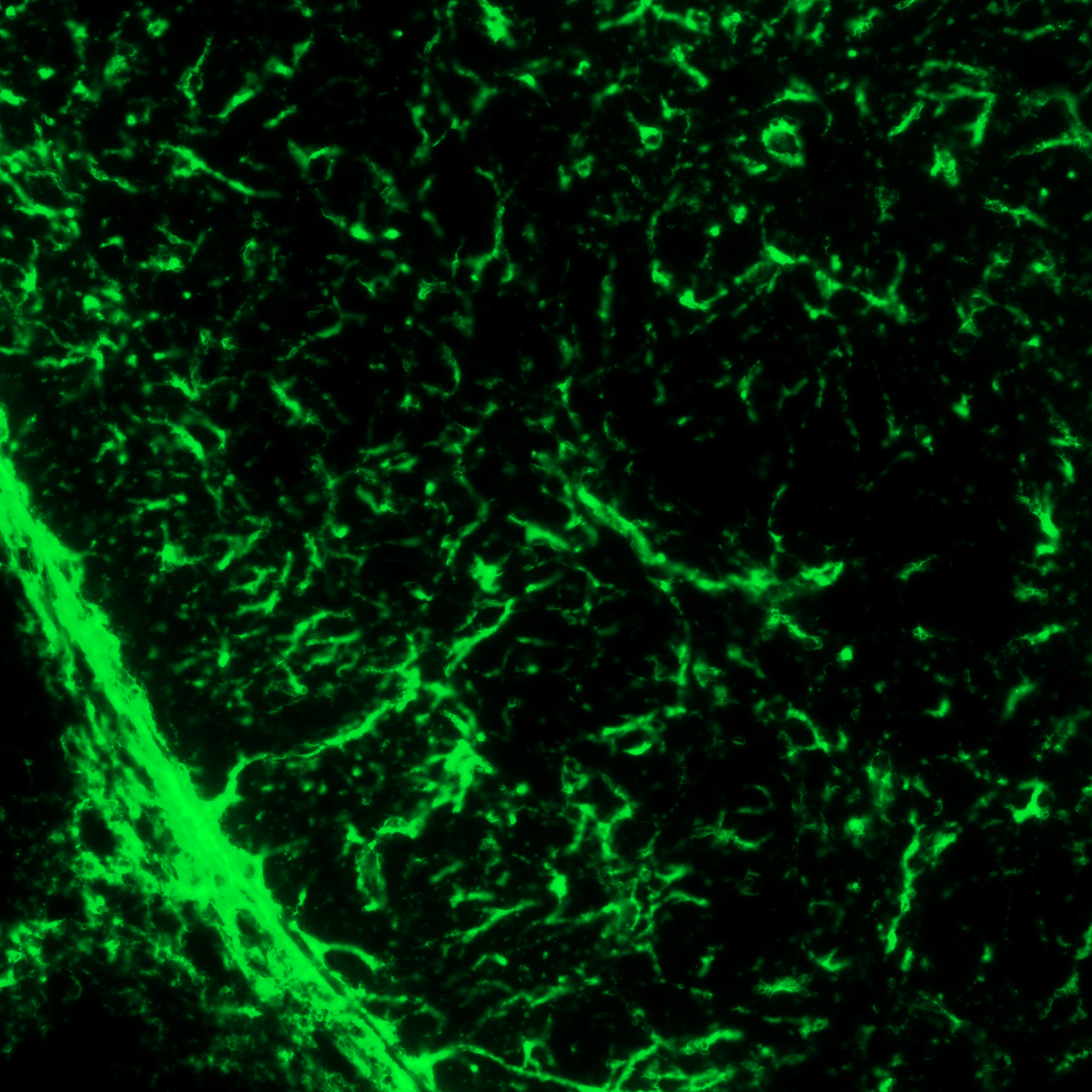

Supplement: Figure 3—figure supplement 1—source data 3. [file elife-86940-fig3-figsupp1-data3.zip › Figure 3-figure supplement 1-source data 3/F448-4-DKO-RX CI CII ff FF-P18-40X-GFAP-NESTIN-88-2-DG-R-Image Export-34_AF488.tif]

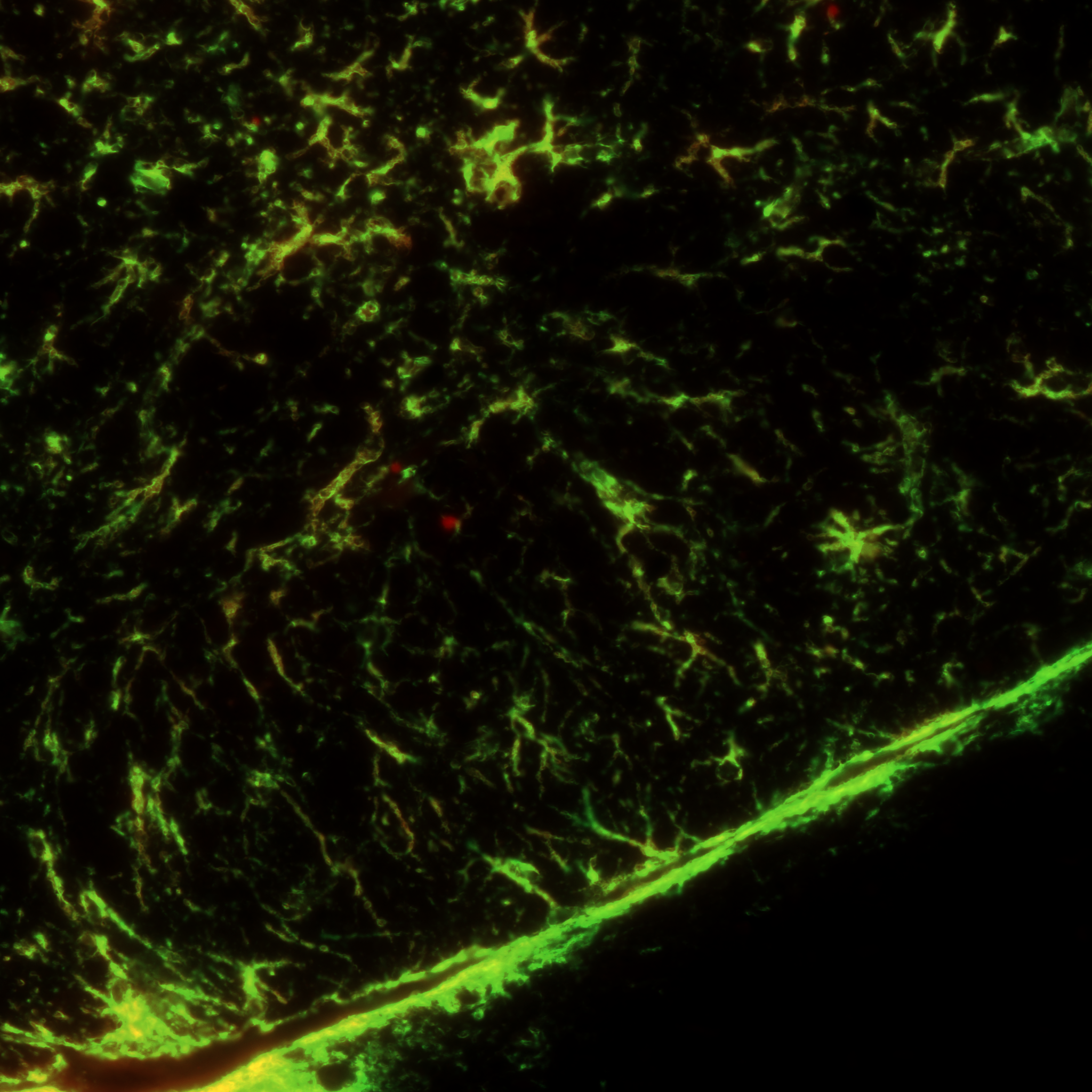

Supplement: Figure 3—figure supplement 1—source data 3. [file elife-86940-fig3-figsupp1-data3.zip › Figure 3-figure supplement 1-source data 3/F8099-1-DKO-RX CI CII ff FF-P20-40X-GFAP-NESTIN-#91-1-HPC-L-G+R-Image Export-28.tif]

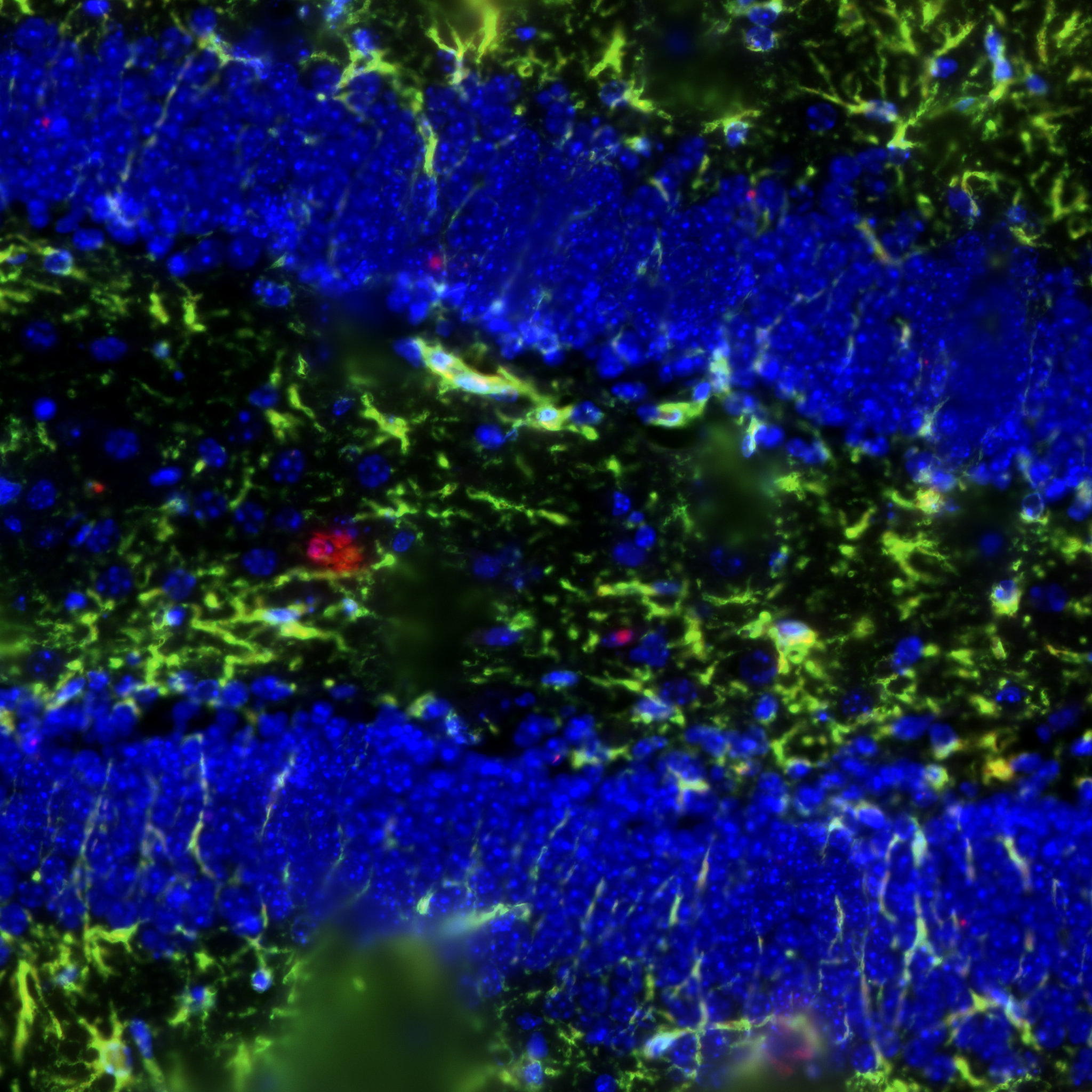

Supplement: Figure 3—figure supplement 1—source data 3. [file elife-86940-fig3-figsupp1-data3.zip › Figure 3-figure supplement 1-source data 3/F8099-3-CON-CI CII ff FF-P20-40X-GFAP-NESTIN-#105-1-HPC-L-Image Export-23.tif]

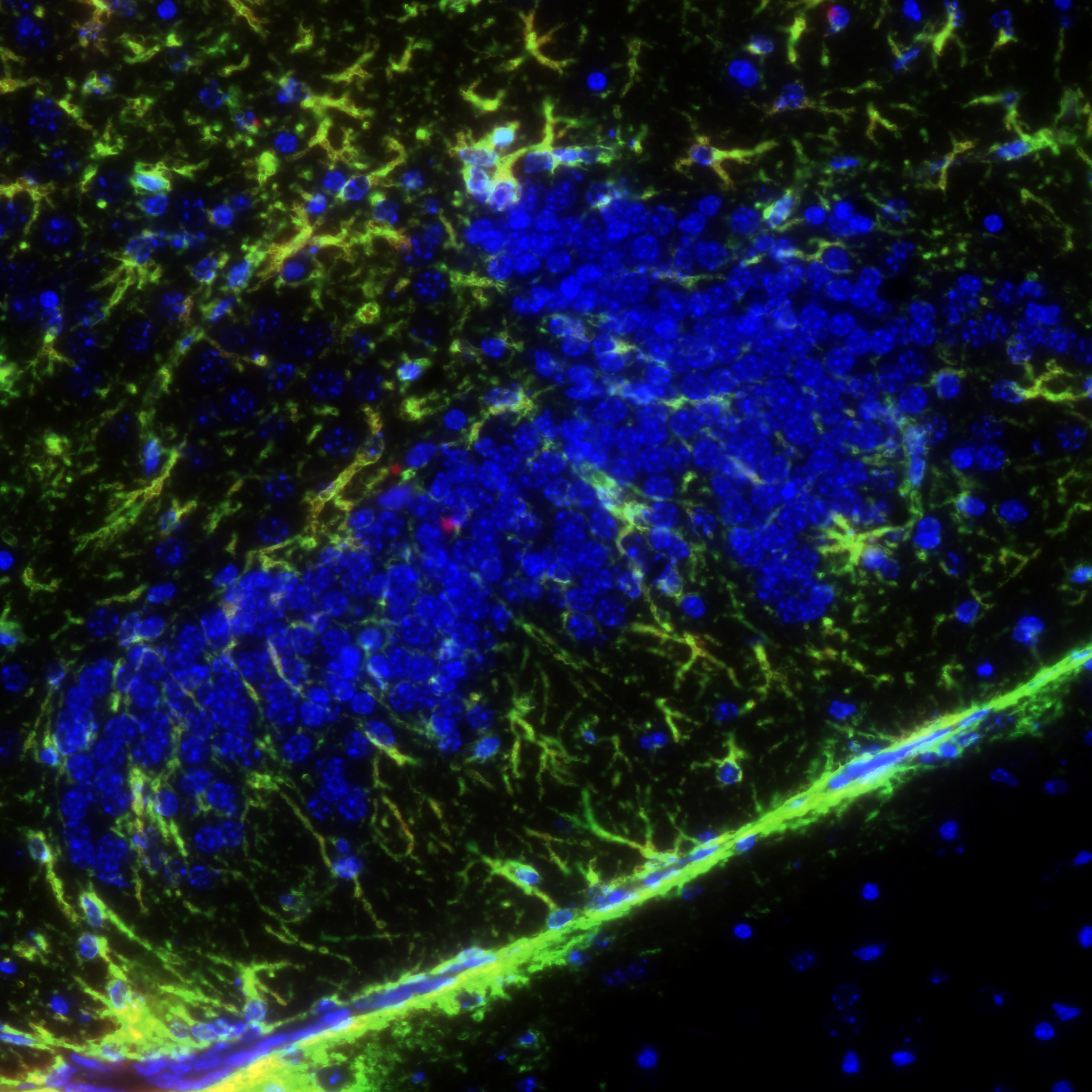

Supplement: Figure 3—figure supplement 1—source data 3. [file elife-86940-fig3-figsupp1-data3.zip › Figure 3-figure supplement 1-source data 3/F8099-1-DKO-RX CI CII ff FF-P20-40X-GFAP-NESTIN-#91-1-HPC-L-Image Export-27.tif]

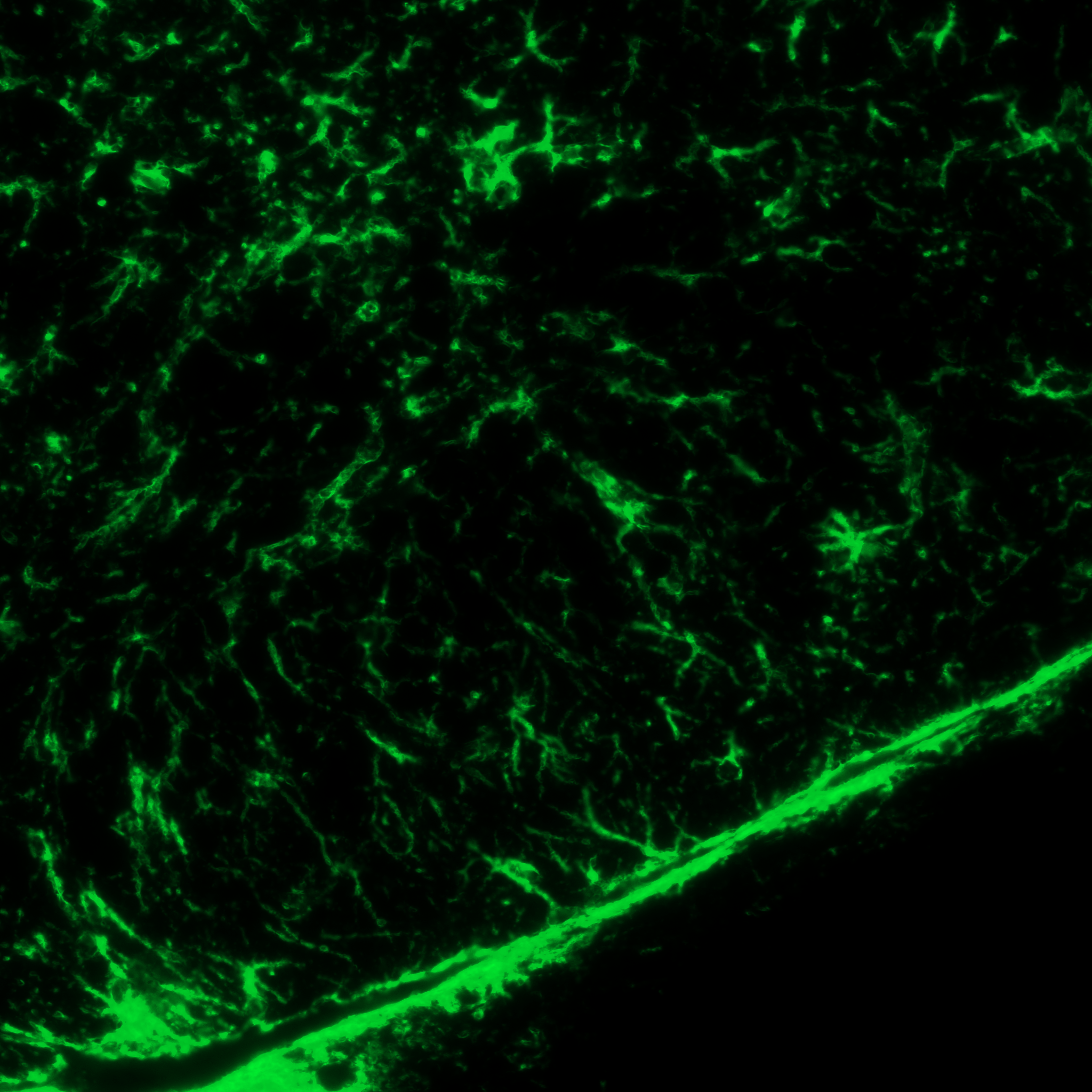

Supplement: Figure 3—figure supplement 1—source data 3. [file elife-86940-fig3-figsupp1-data3.zip › Figure 3-figure supplement 1-source data 3/F8099-1-DKO-RX CI CII ff FF-P20-40X-GFAP-NESTIN-#91-1-HPC-L-G+R-Image Export-28_AF488.tif]

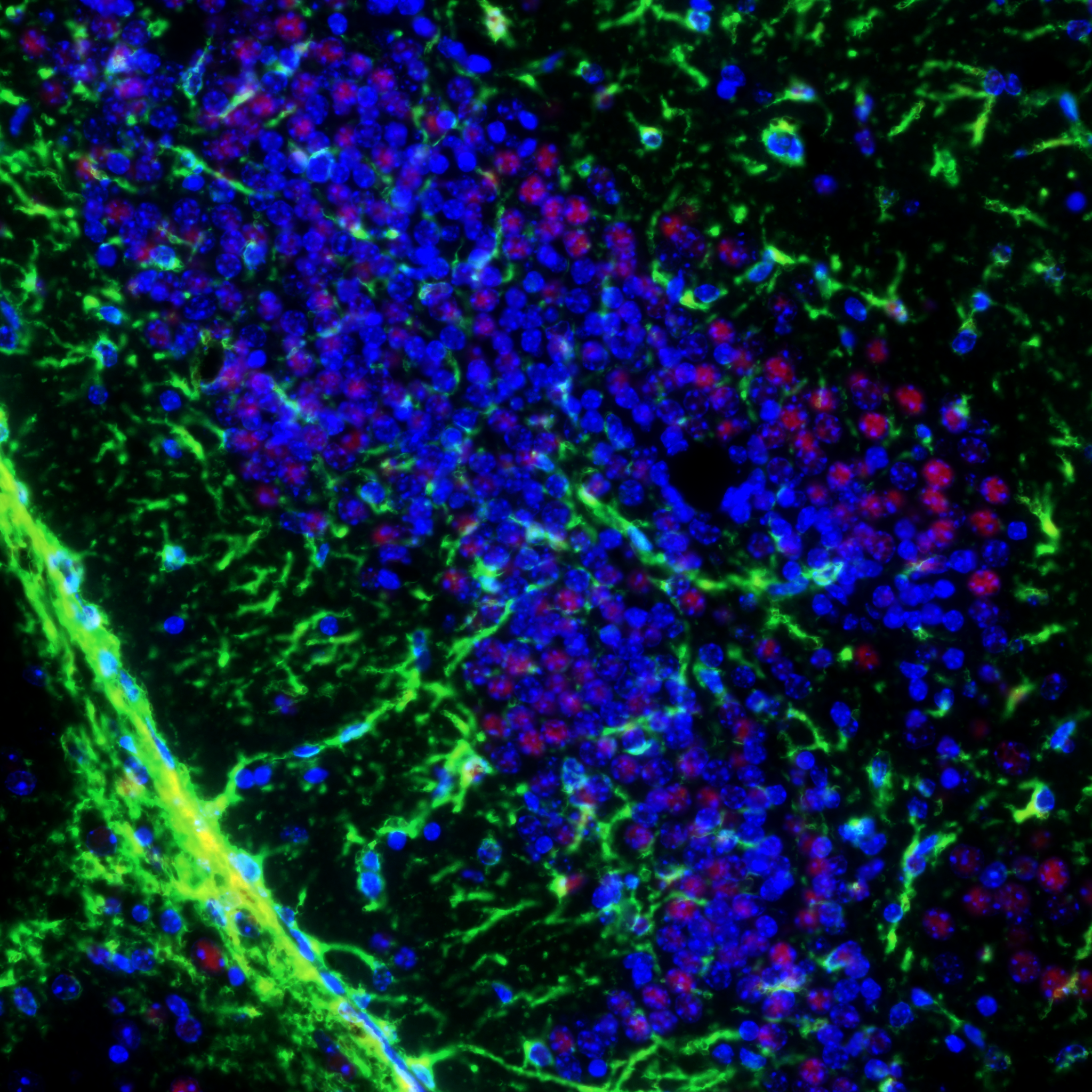

Supplement: Figure 3—figure supplement 1—source data 3. [file elife-86940-fig3-figsupp1-data3.zip › Figure 3-figure supplement 1-source data 3/F448-4-DKO-RX CI CII ff FF-P18-40X-GFAP-NESTIN-88-2-DG-R-Image Export-34.tif]

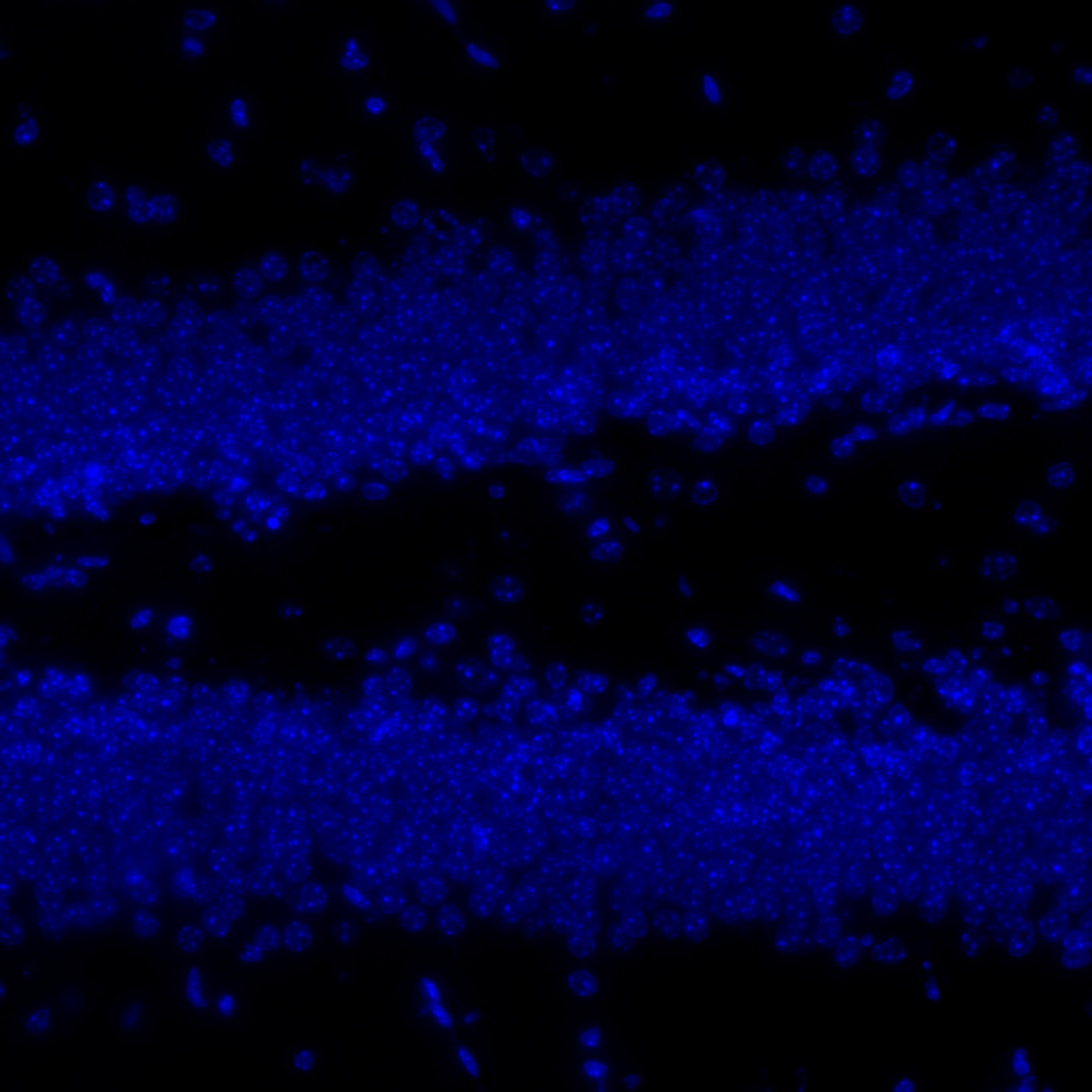

Supplement: Figure 3—figure supplement 1—source data 3. [file elife-86940-fig3-figsupp1-data3.zip › Figure 3-figure supplement 1-source data 3/F449-3-CON-CI CII f+ FF-P18-40X-GFAP-NESTIN-#83-2-HPC-R-G+R-Image Export-5_DAPI.tif]

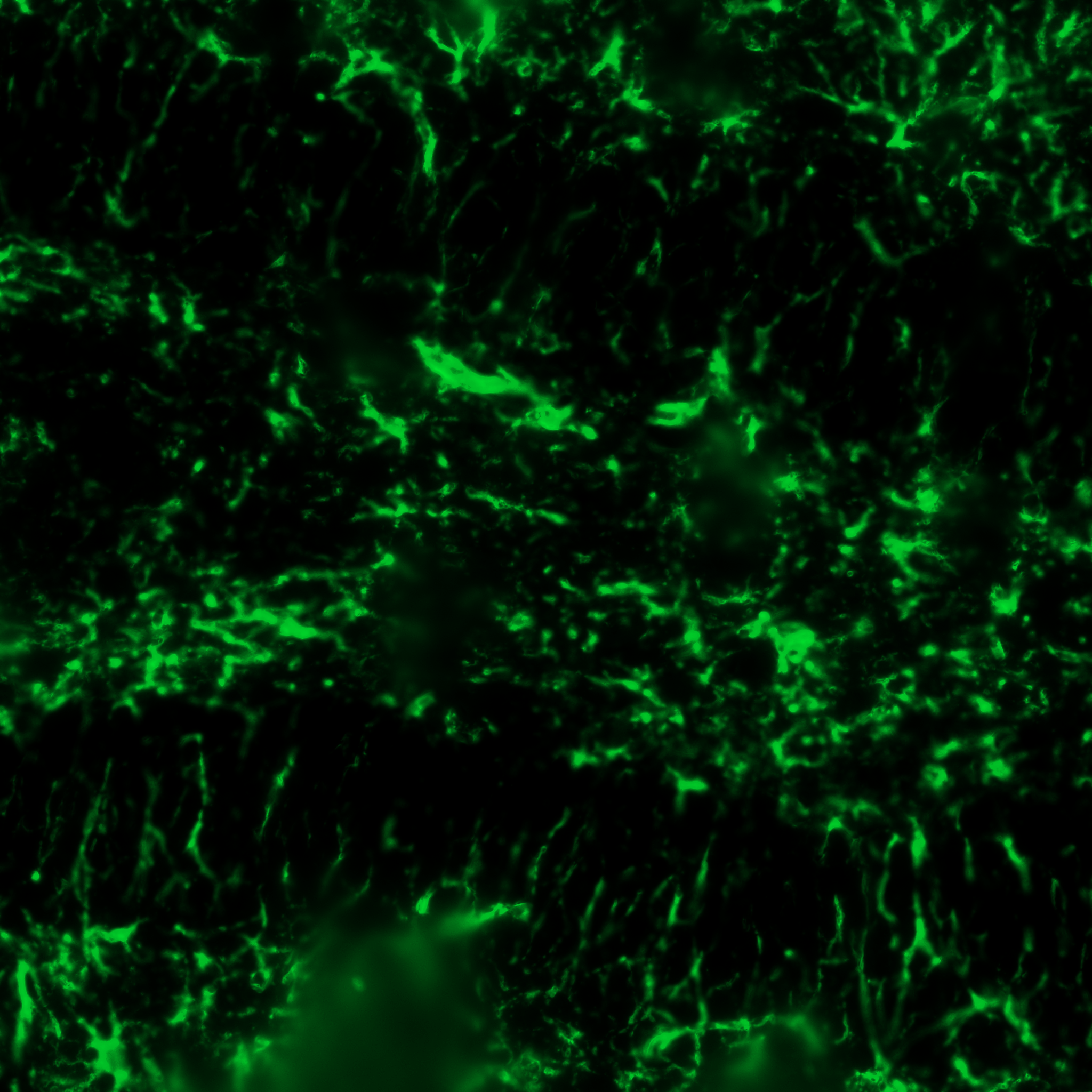

Supplement: Figure 3—figure supplement 1—source data 3. [file elife-86940-fig3-figsupp1-data3.zip › Figure 3-figure supplement 1-source data 3/F8099-3-CON-CI CII ff FF-P20-40X-GFAP-NESTIN-#105-1-HPC-L-G+R-Image Export-24_AF488.tif]

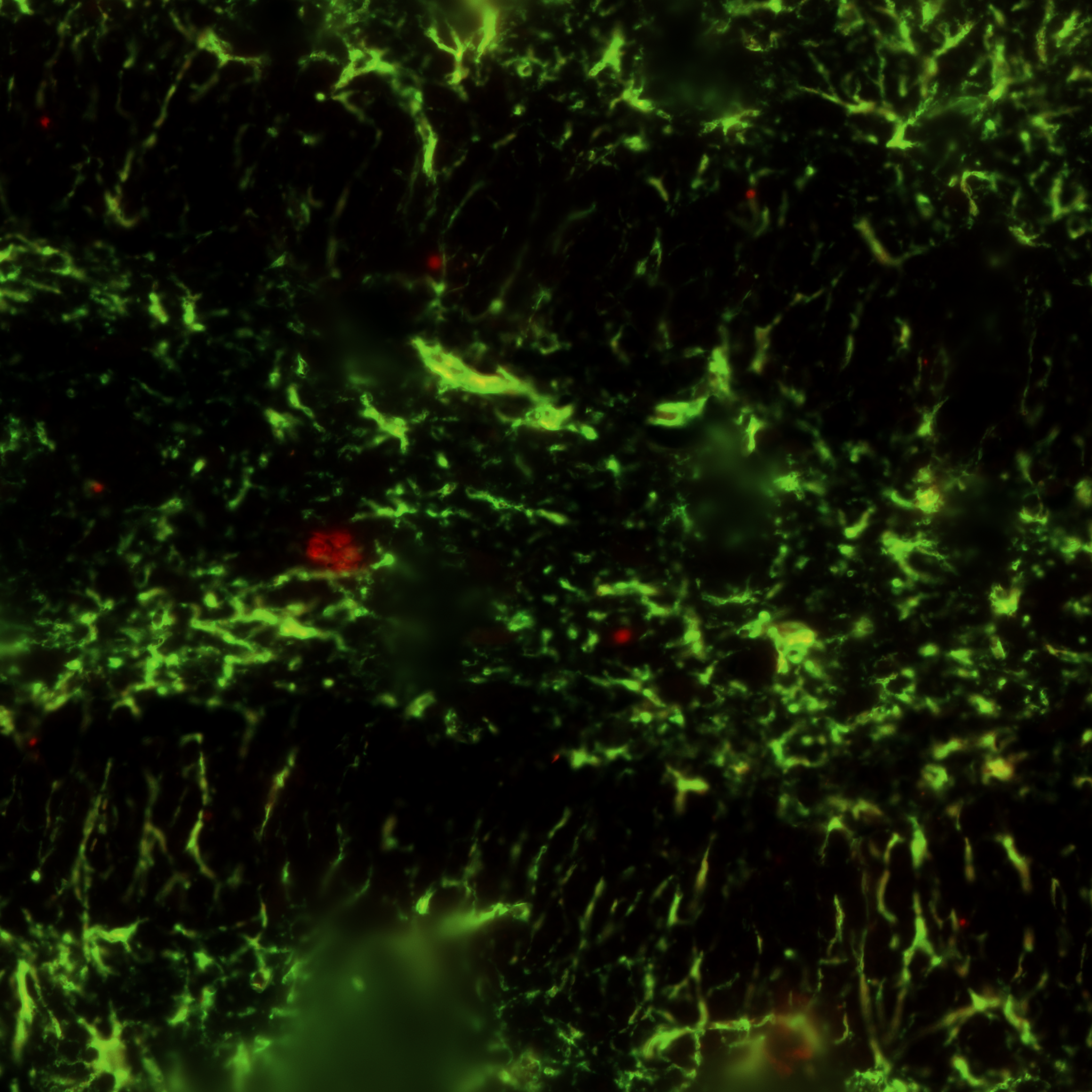

Supplement: Figure 3—figure supplement 1—source data 3. [file elife-86940-fig3-figsupp1-data3.zip › Figure 3-figure supplement 1-source data 3/F8099-3-CON-CI CII ff FF-P20-40X-GFAP-NESTIN-#105-1-HPC-L-G+R-Image Export-24.tif]

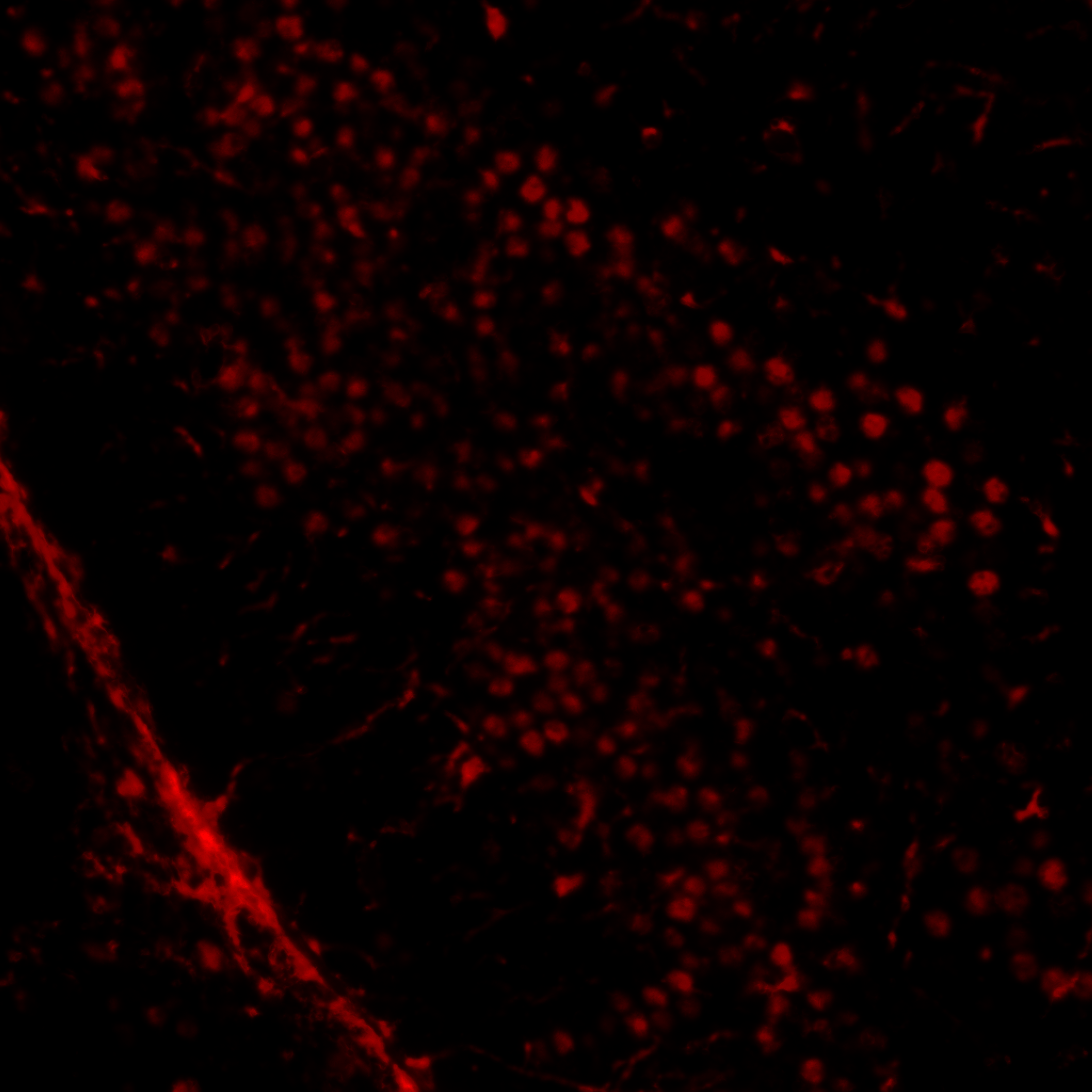

Supplement: Figure 3—figure supplement 1—source data 3. [file elife-86940-fig3-figsupp1-data3.zip › Figure 3-figure supplement 1-source data 3/F448-4-DKO-RX CI CII ff FF-P18-40X-GFAP-NESTIN-88-2-DG-R-Image Export-34_AF594.tif]

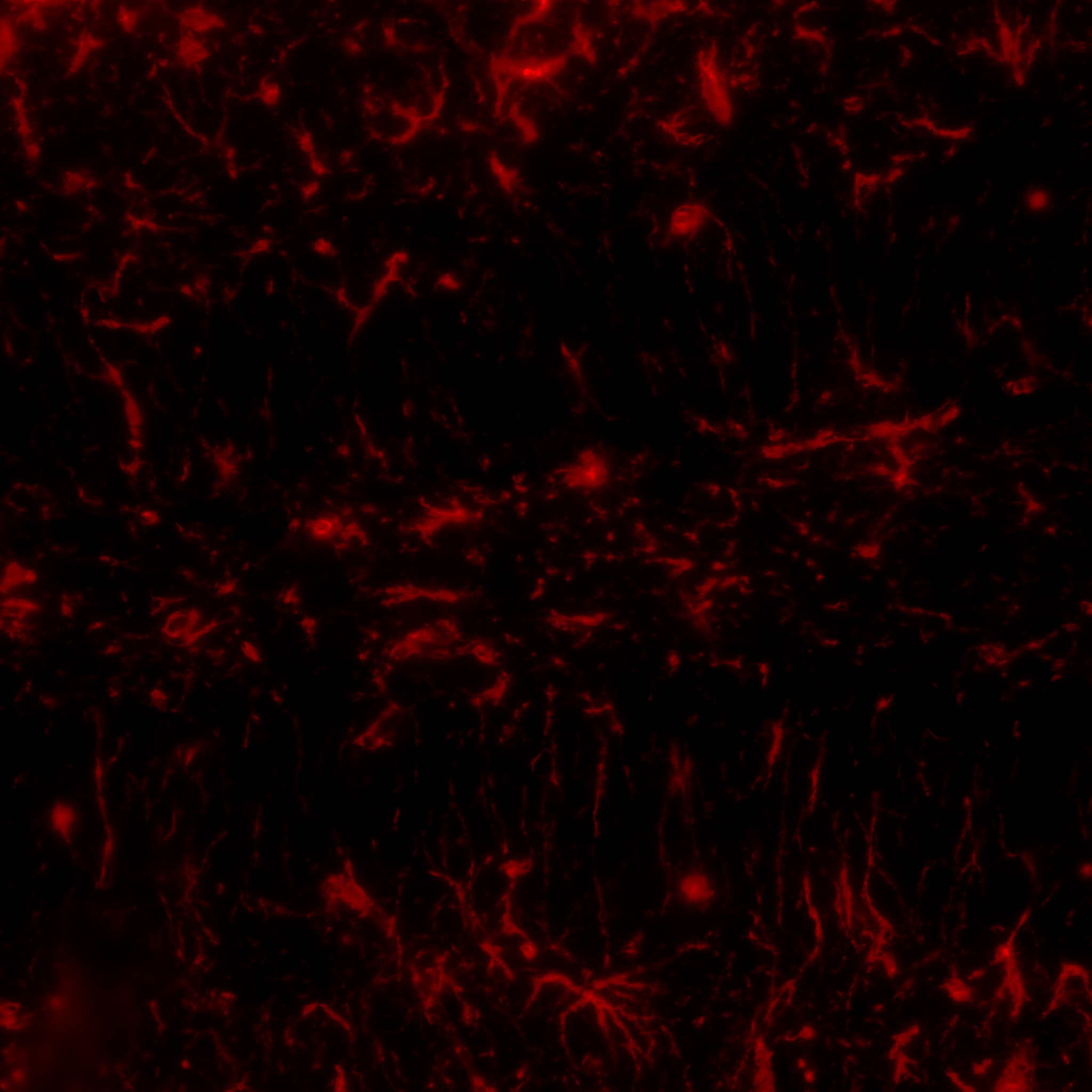

Supplement: Figure 3—figure supplement 1—source data 3. [file elife-86940-fig3-figsupp1-data3.zip › Figure 3-figure supplement 1-source data 3/F449-3-CON-CI CII f+ FF-P18-40X-GFAP-NESTIN-#83-2-HPC-R-G+R-Image Export-5_AF594.tif]

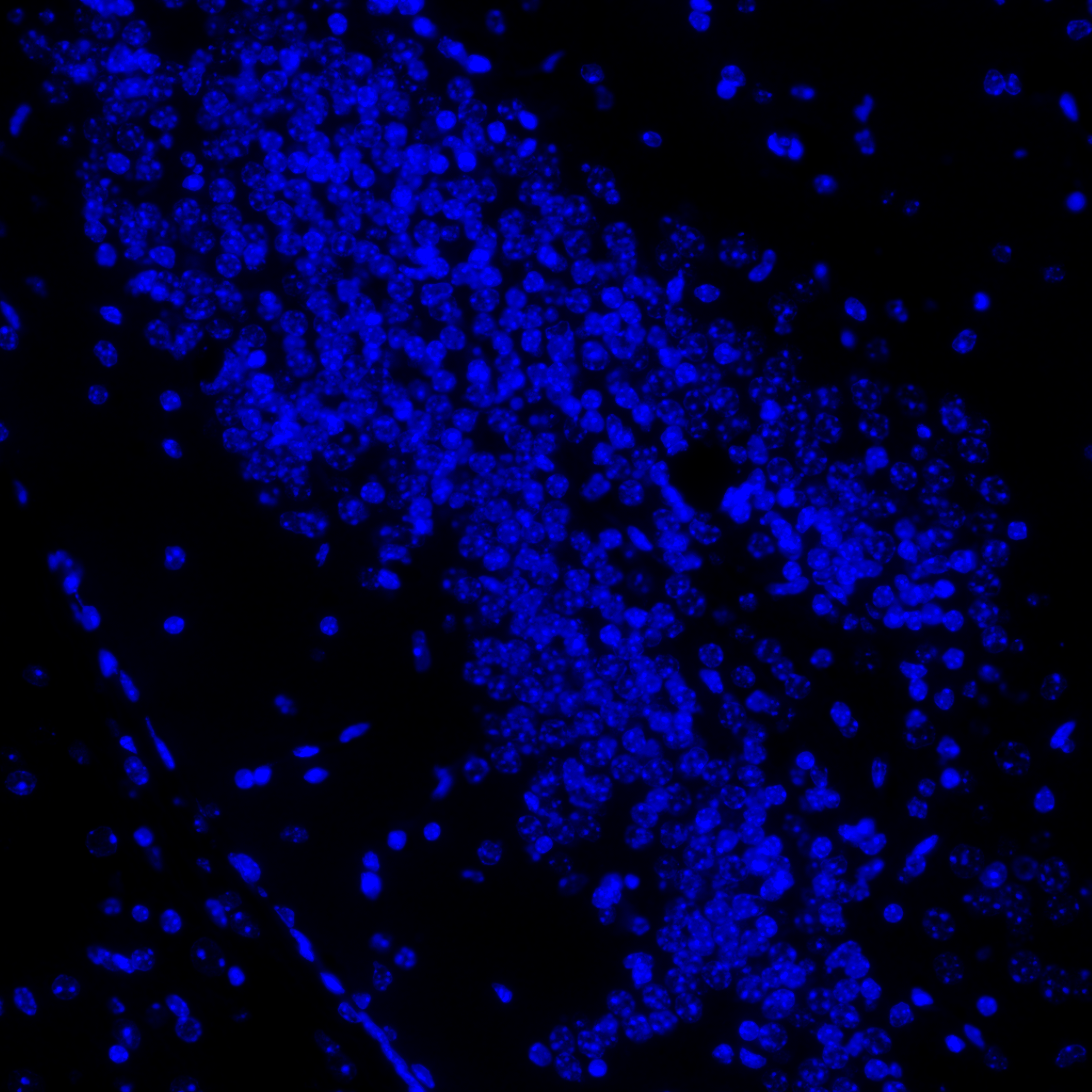

Supplement: Figure 3—figure supplement 1—source data 3. [file elife-86940-fig3-figsupp1-data3.zip › Figure 3-figure supplement 1-source data 3/F448-4-DKO-RX CI CII ff FF-P18-40X-GFAP-NESTIN-88-2-DG-R-Image Export-34_DAPI.tif]

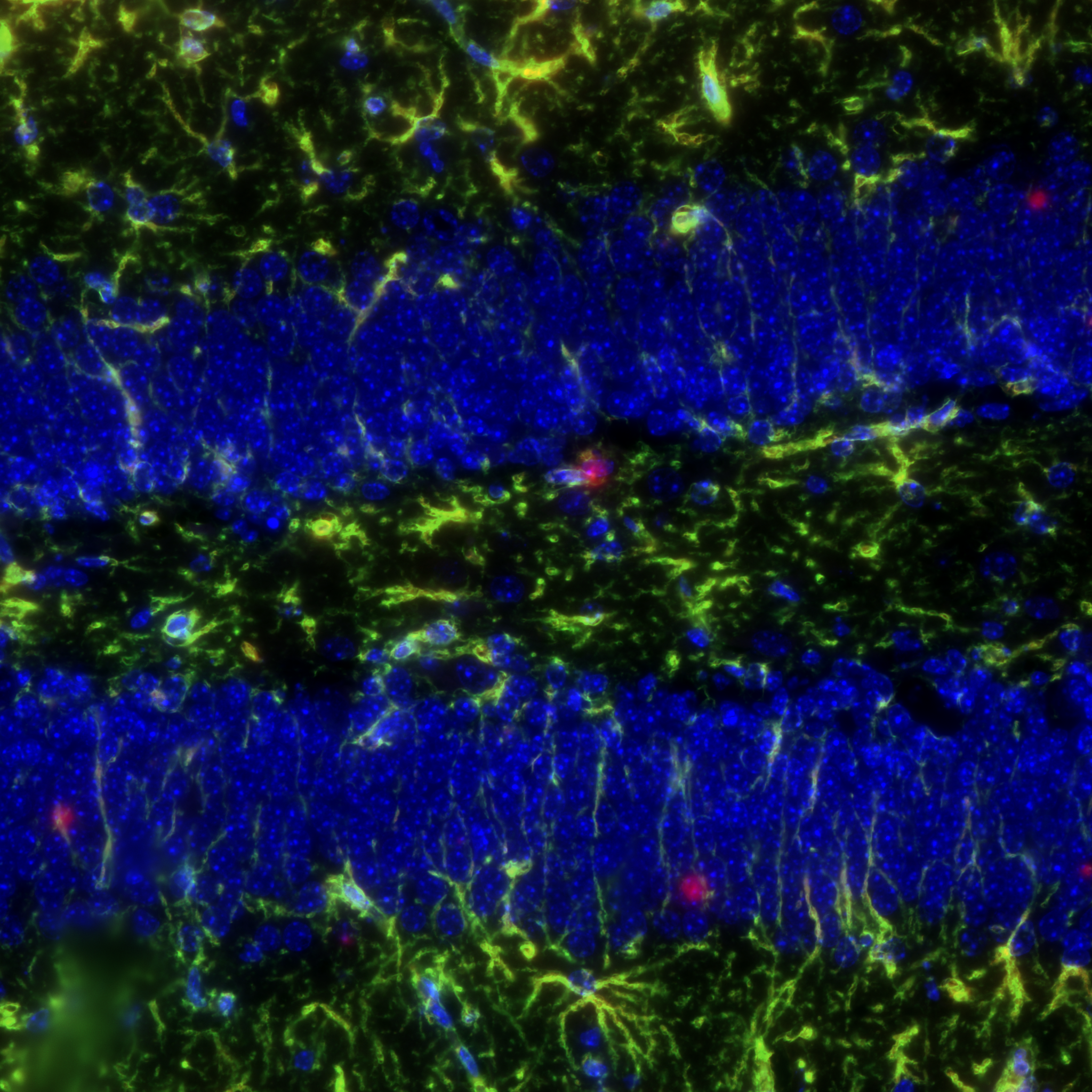

Supplement: Figure 3—figure supplement 1—source data 3. [file elife-86940-fig3-figsupp1-data3.zip › Figure 3-figure supplement 1-source data 3/F449-3-CON-CI CII f+ FF-P18-40X-GFAP-NESTIN-#83-2-HPC-R-G+R-Image Export-5.tif]

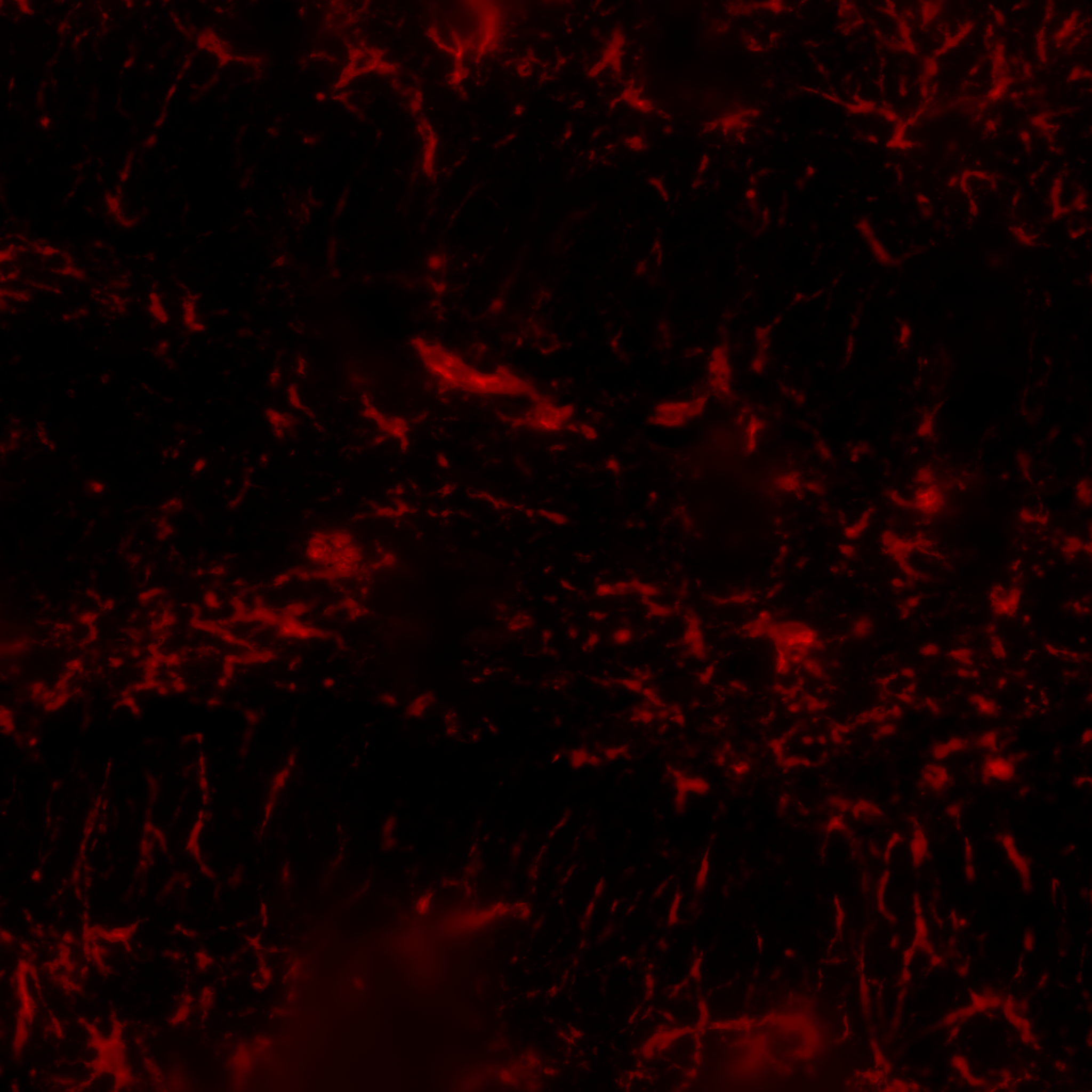

Supplement: Figure 3—figure supplement 1—source data 3. [file elife-86940-fig3-figsupp1-data3.zip › Figure 3-figure supplement 1-source data 3/F8099-3-CON-CI CII ff FF-P20-40X-GFAP-NESTIN-#105-1-HPC-L-G+R-Image Export-24_AF594.tif]

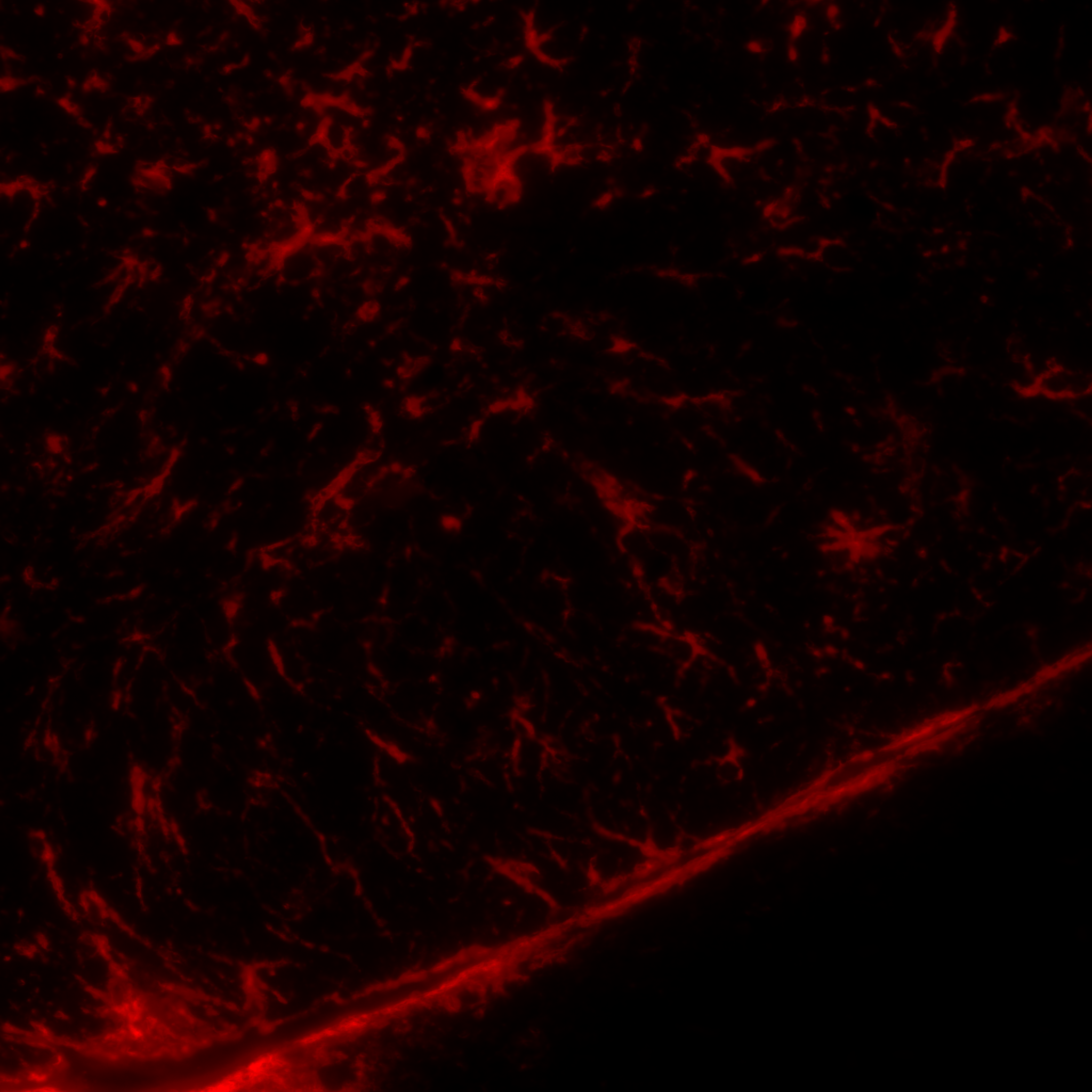

Supplement: Figure 3—figure supplement 1—source data 3. [file elife-86940-fig3-figsupp1-data3.zip › Figure 3-figure supplement 1-source data 3/F8099-1-DKO-RX CI CII ff FF-P20-40X-GFAP-NESTIN-#91-1-HPC-L-G+R-Image Export-28_AF594.tif]

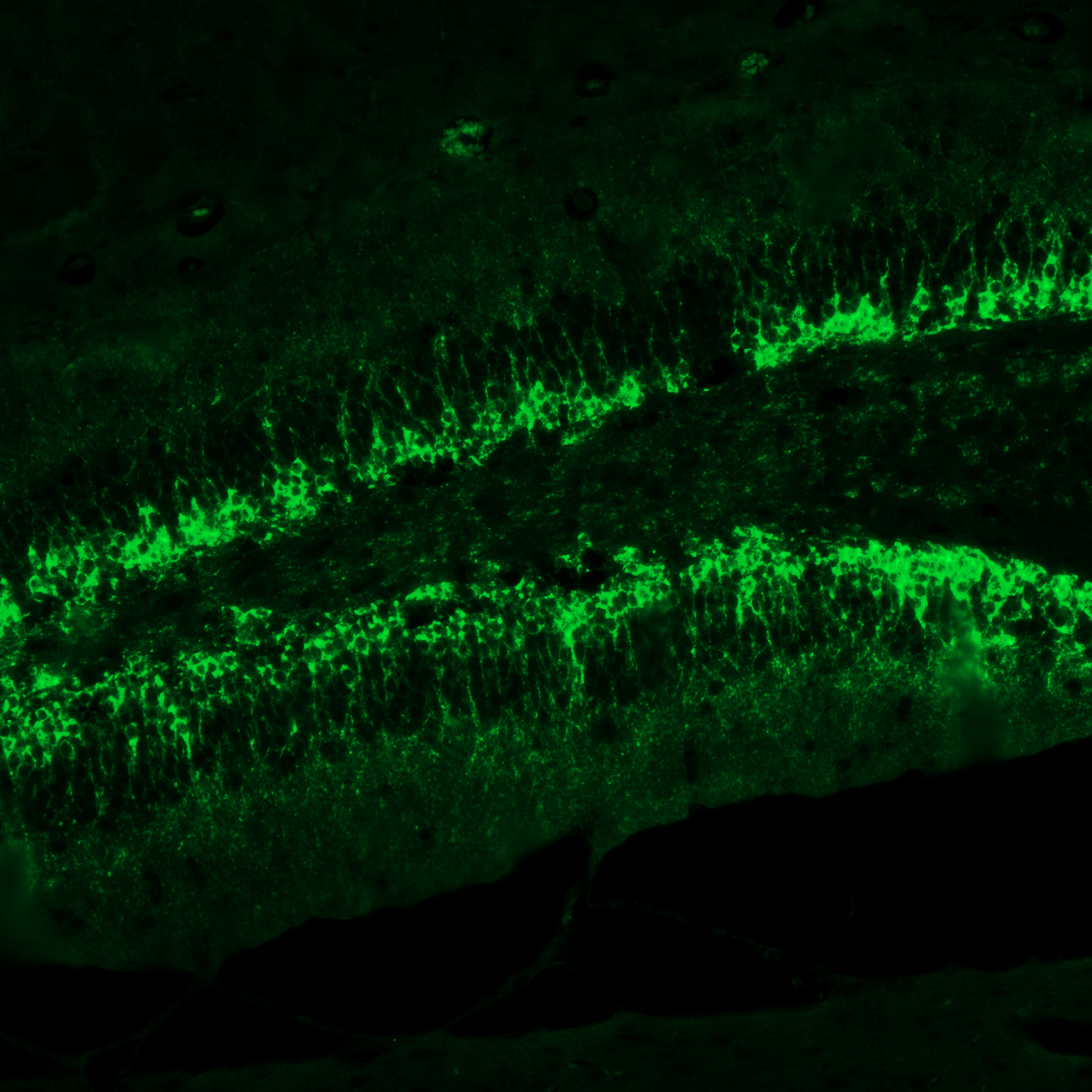

Supplement: Figure 3—figure supplement 1—source data 4. [file elife-86940-fig3-figsupp1-data4.zip › Figure 3-figure supplement 1-source data 4/F448-2-CON-CI CII f+ F+-P18-20X-DCX-102-2-DG-R-Image Export-10_AF488.tif]

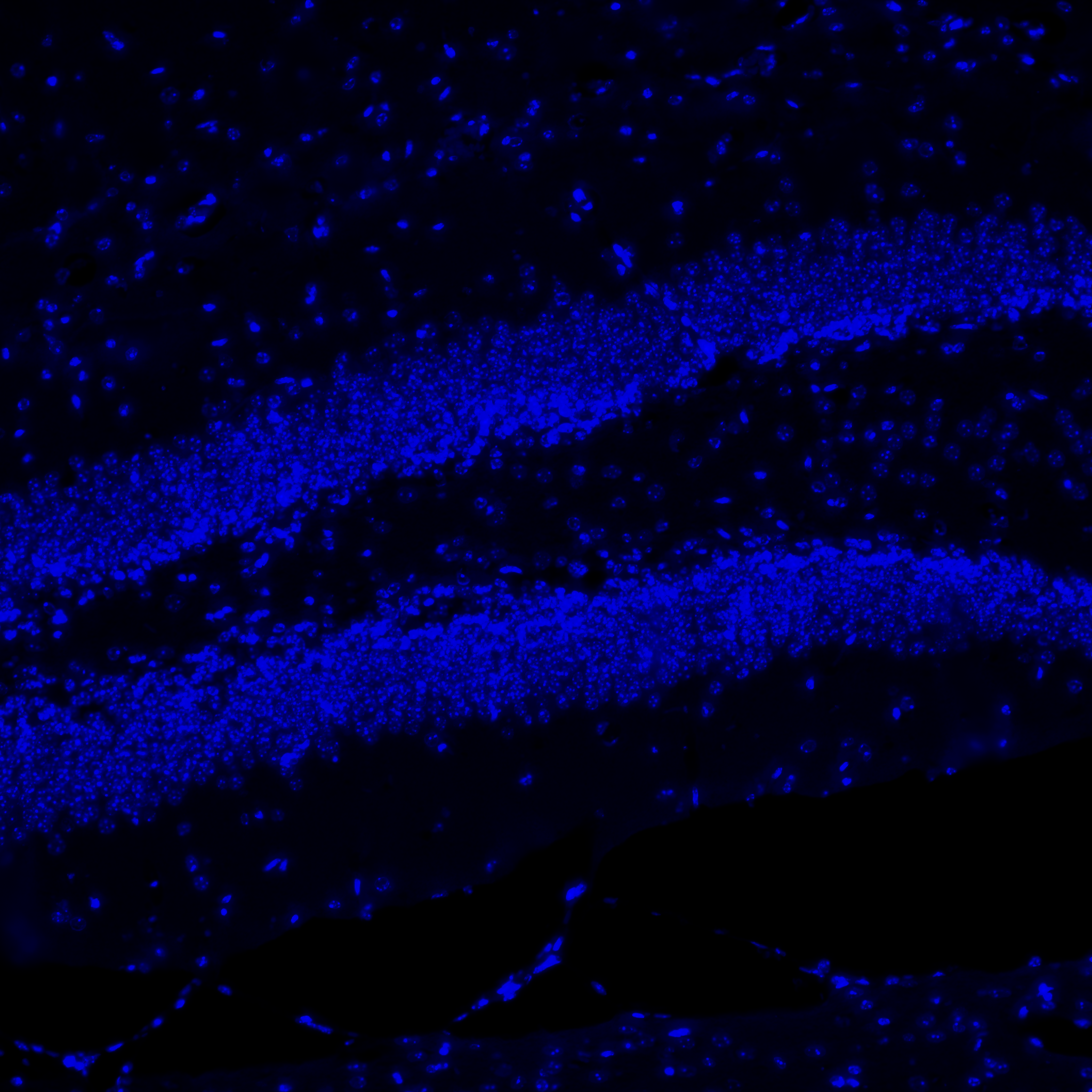

Supplement: Figure 3—figure supplement 1—source data 4. [file elife-86940-fig3-figsupp1-data4.zip › Figure 3-figure supplement 1-source data 4/F448-2-CON-CI CII f+ F+-P18-20X-DCX-102-2-DG-R-Image Export-10_DAPI.tif]

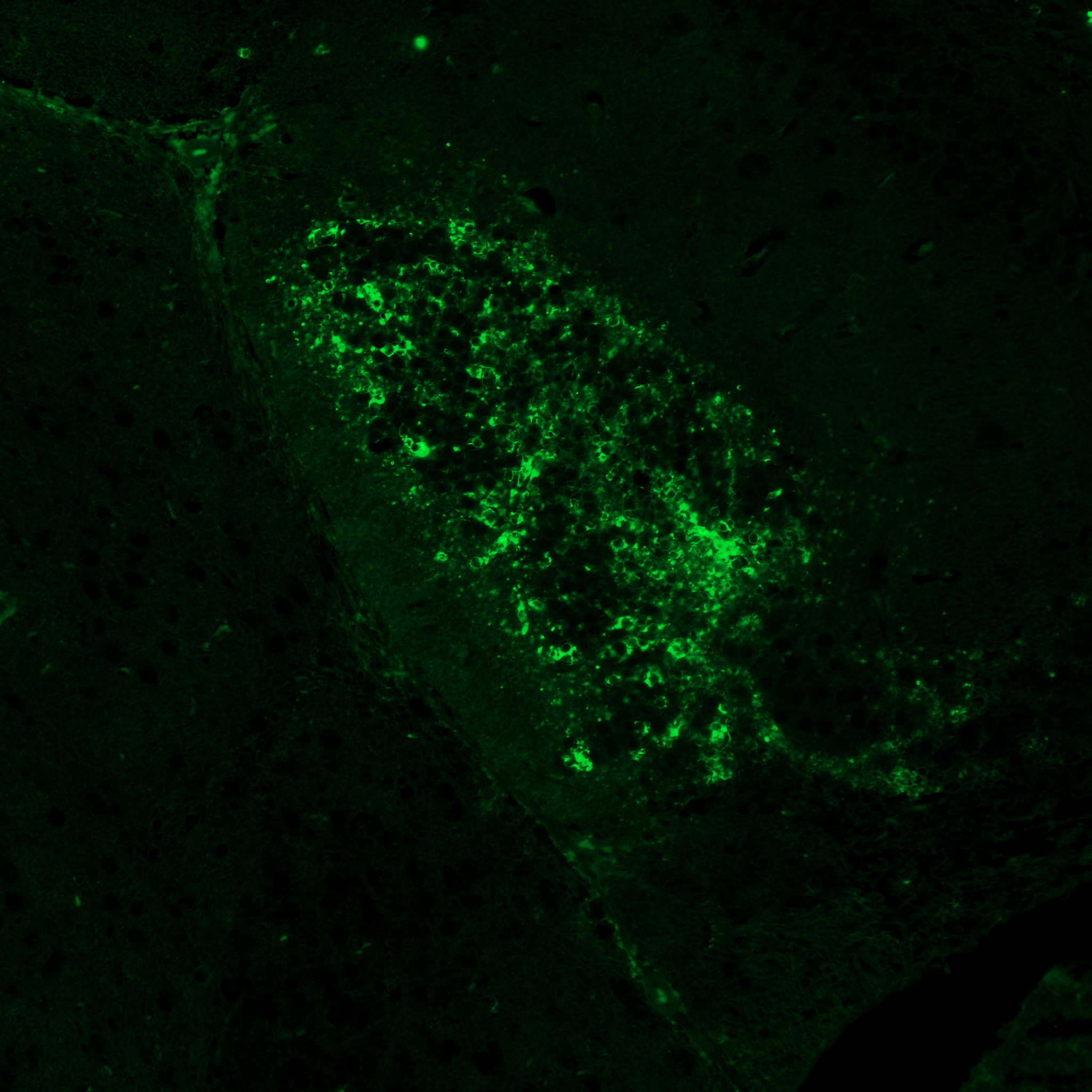

Supplement: Figure 3—figure supplement 1—source data 4. [file elife-86940-fig3-figsupp1-data4.zip › Figure 3-figure supplement 1-source data 4/F448-4-DKO-RX CI CII ff FF-P18-20X-DCX-95-1-DG-R-Image Export-20_AF488.tif]

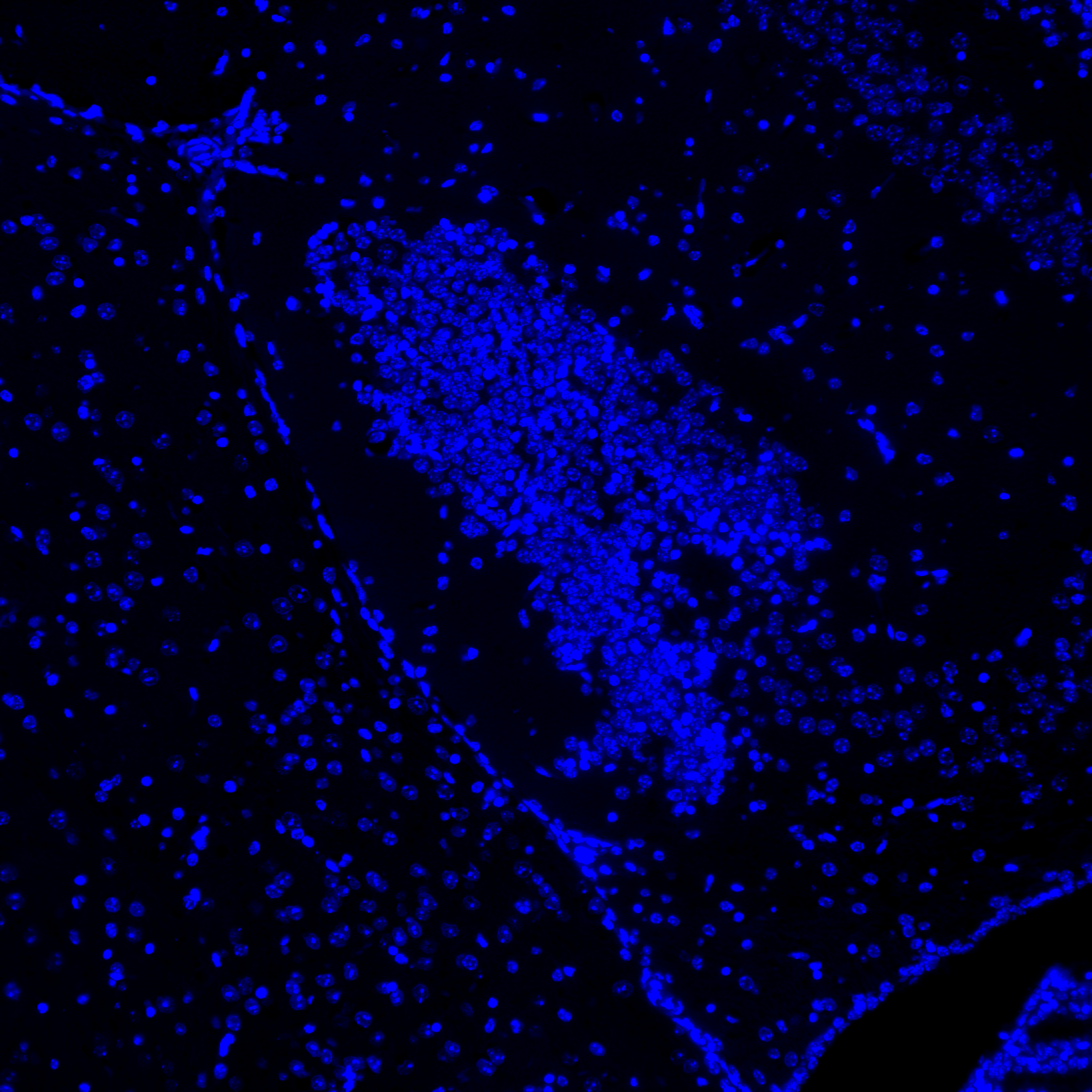

Supplement: Figure 3—figure supplement 1—source data 4. [file elife-86940-fig3-figsupp1-data4.zip › Figure 3-figure supplement 1-source data 4/F448-4-DKO-RX CI CII ff FF-P18-20X-DCX-95-1-DG-R-Image Export-20_DAPI.tif]

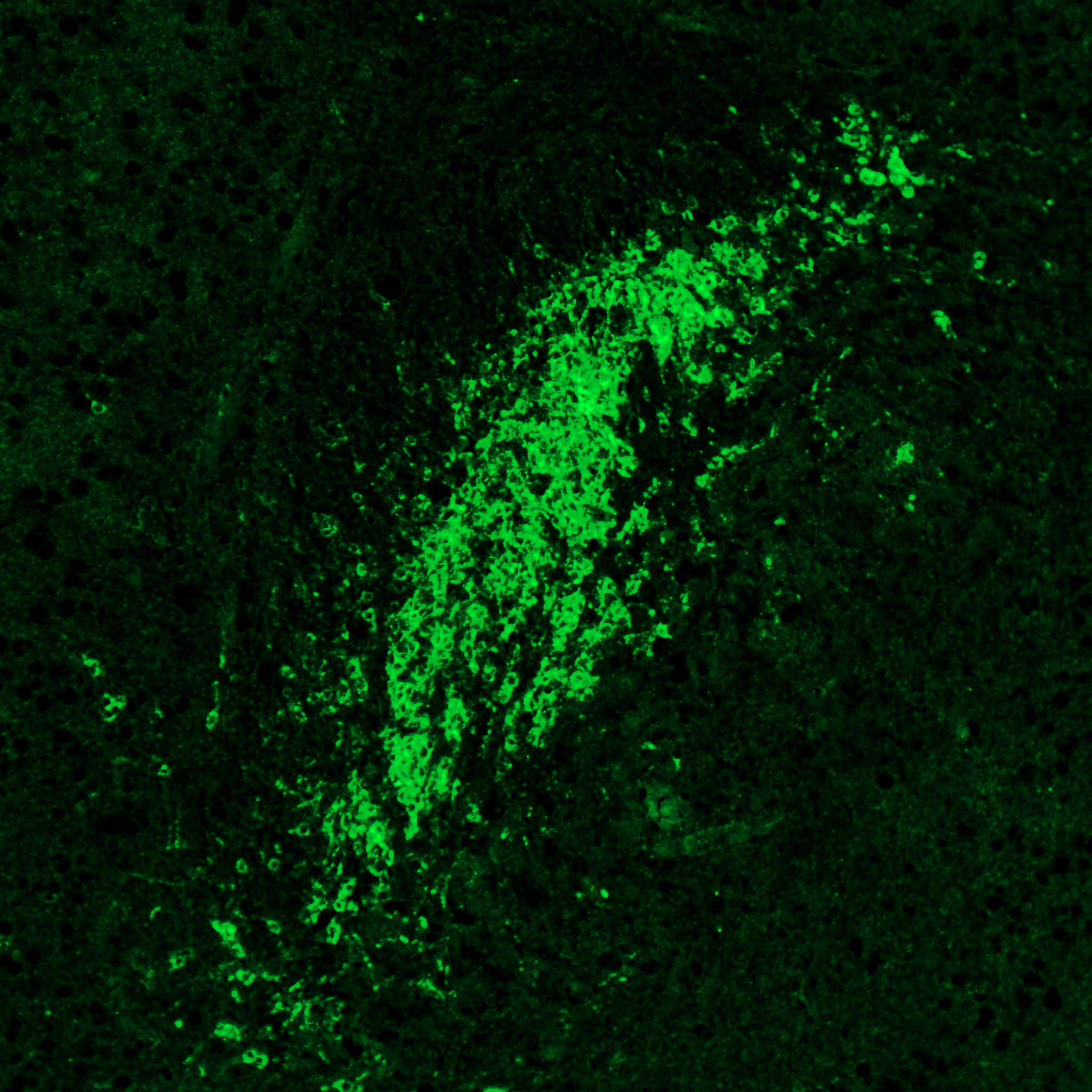

Supplement: Figure 3—figure supplement 1—source data 4. [file elife-86940-fig3-figsupp1-data4.zip › Figure 3-figure supplement 1-source data 4/F449-1-DKO-RX CI CII ff FF-P18-20X-DCX-121-1-R-DG-Image Export-15_AF488.tif]

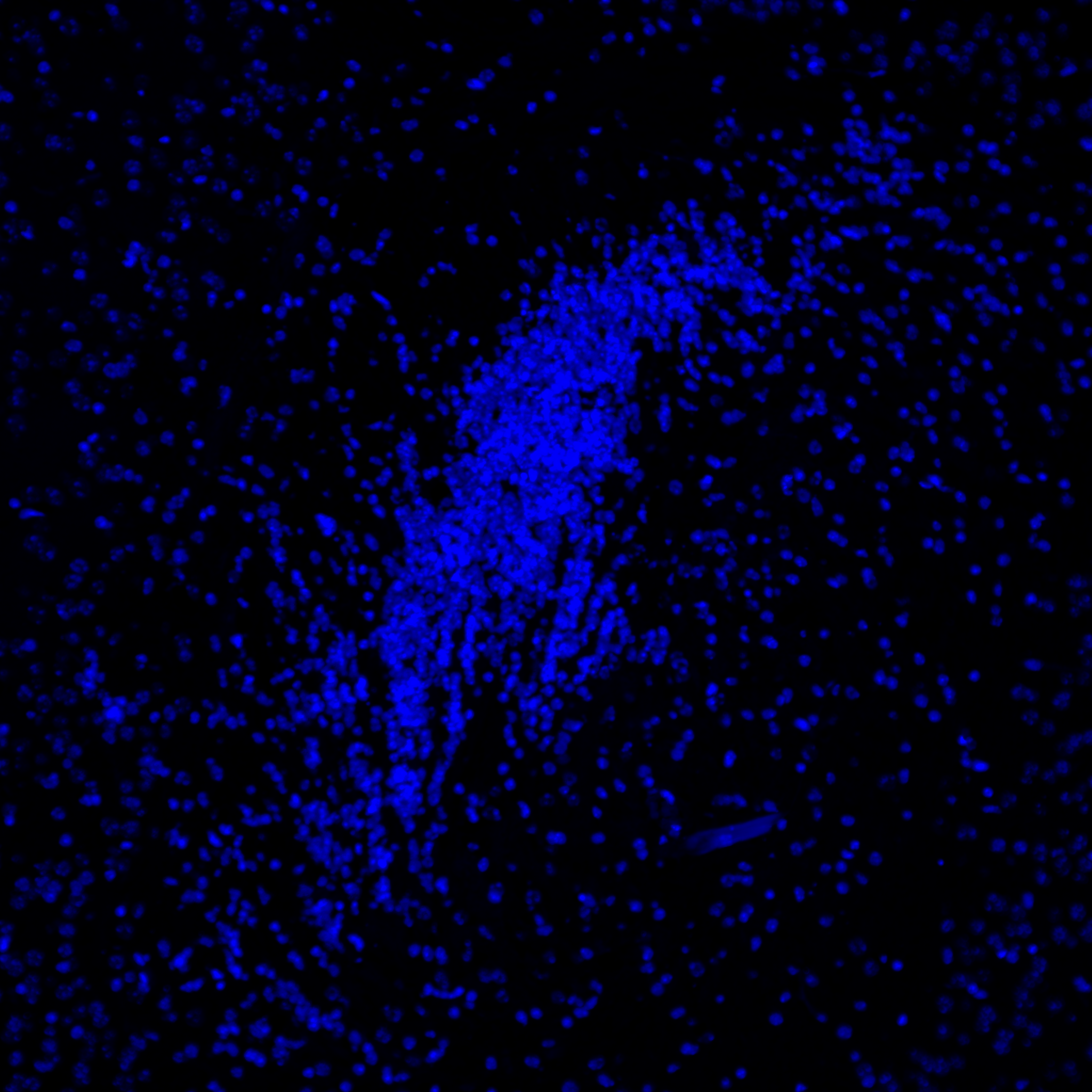

Supplement: Figure 3—figure supplement 1—source data 4. [file elife-86940-fig3-figsupp1-data4.zip › Figure 3-figure supplement 1-source data 4/F449-1-DKO-RX CI CII ff FF-P18-20X-DCX-121-1-R-DG-Image Export-15_DAPI.tif]

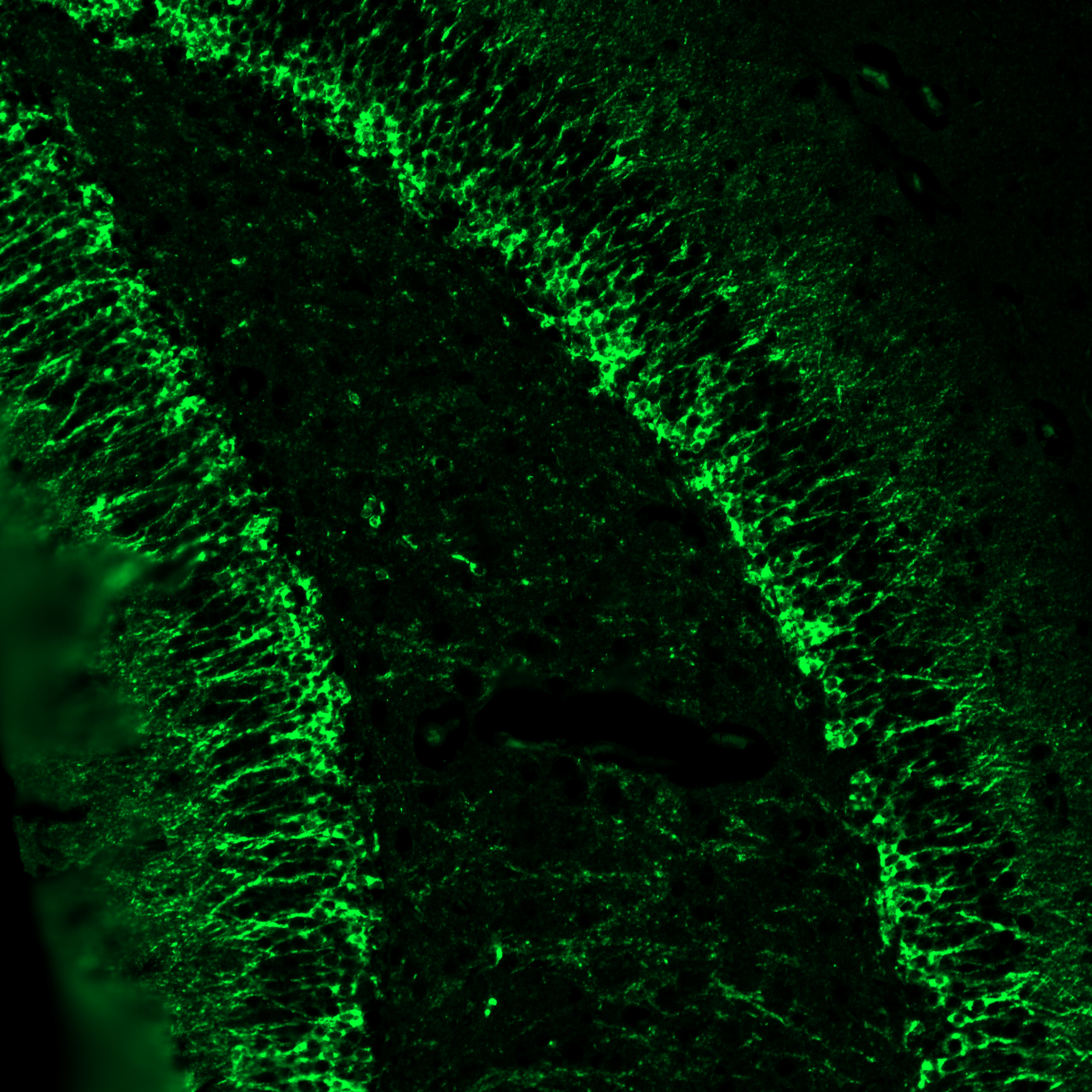

Supplement: Figure 3—figure supplement 1—source data 4. [file elife-86940-fig3-figsupp1-data4.zip › Figure 3-figure supplement 1-source data 4/F449-3-CON-CI CII f+ FF-P18-20X-DCX-139-3-R-DG-Image Export-7_AF488.tif]

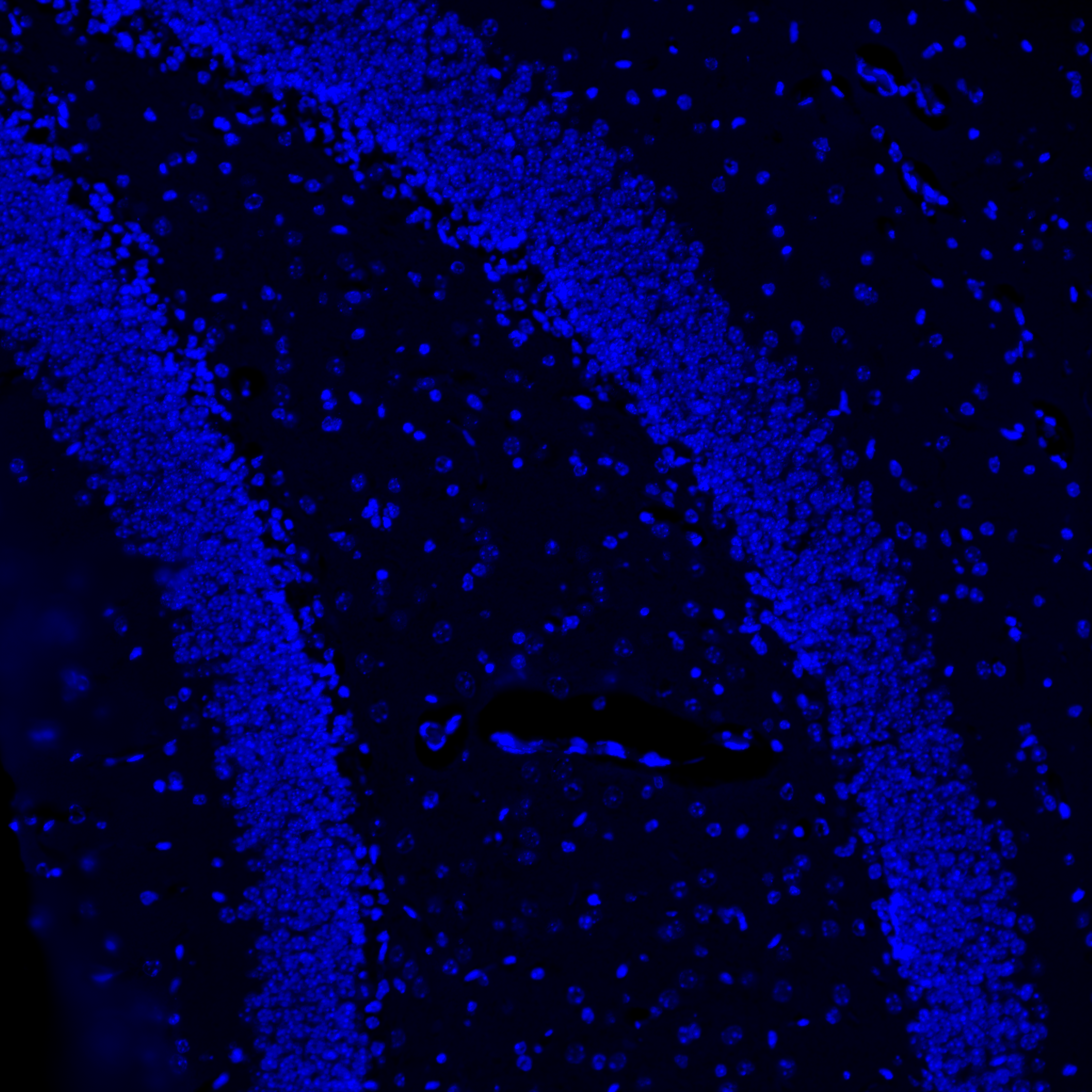

Supplement: Figure 3—figure supplement 1—source data 4. [file elife-86940-fig3-figsupp1-data4.zip › Figure 3-figure supplement 1-source data 4/F449-3-CON-CI CII f+ FF-P18-20X-DCX-139-3-R-DG-Image Export-7_DAPI.tif]

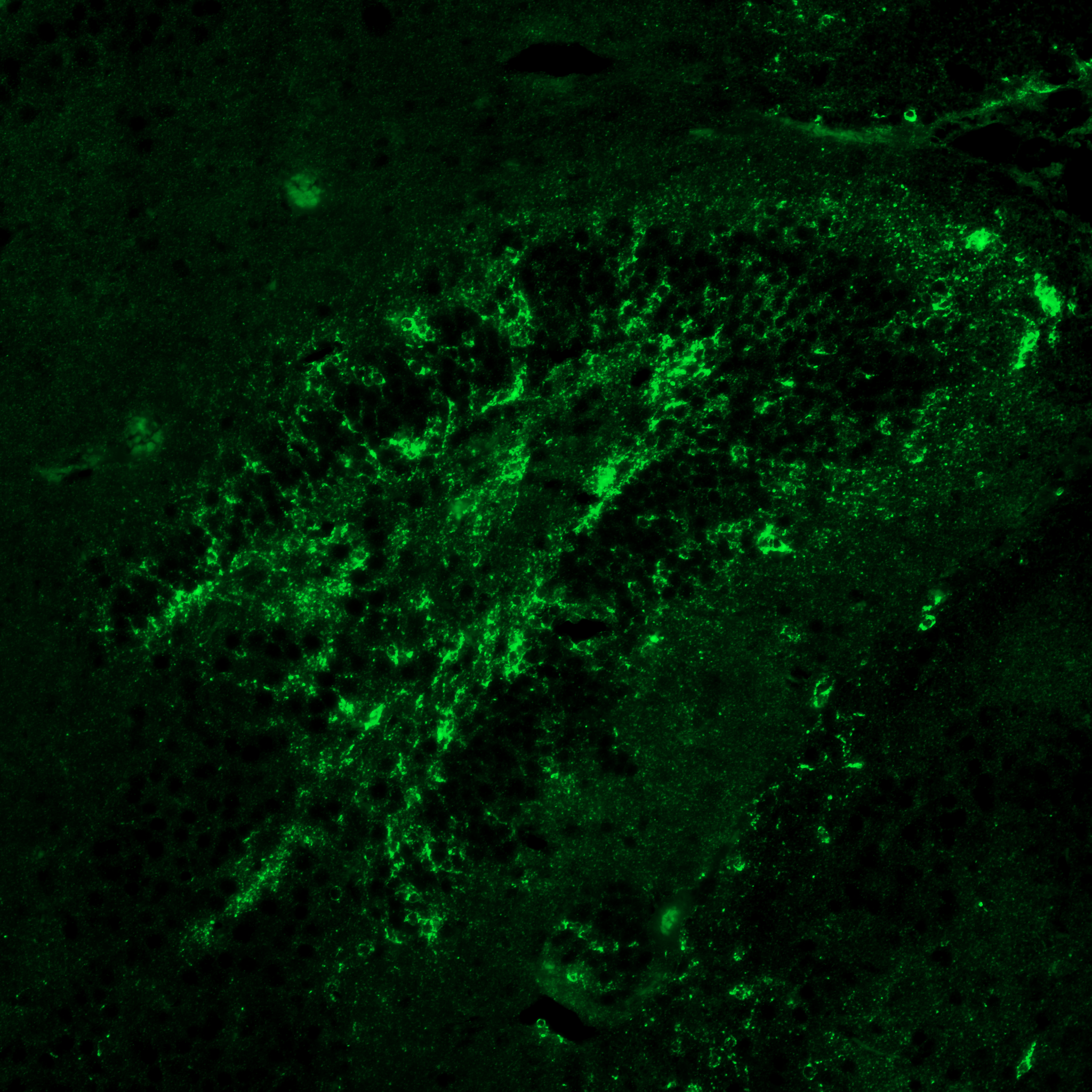

Supplement: Figure 3—figure supplement 1—source data 4. [file elife-86940-fig3-figsupp1-data4.zip › Figure 3-figure supplement 1-source data 4/F8099-1-DKO-RX CI CII ff FF-P20-20X-DCX-128-2-L-dDG-Image Export-25_AF488.tif]

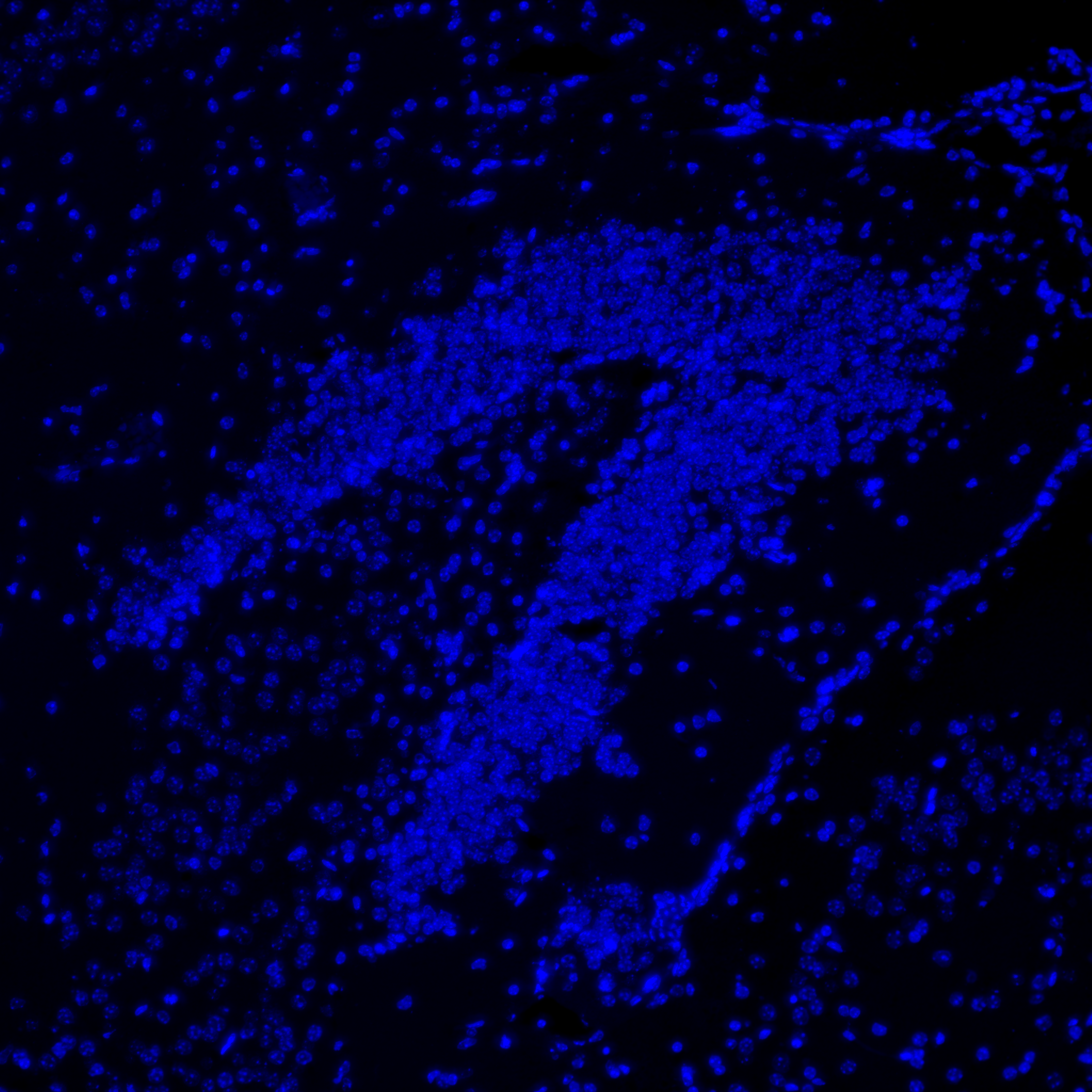

Supplement: Figure 3—figure supplement 1—source data 4. [file elife-86940-fig3-figsupp1-data4.zip › Figure 3-figure supplement 1-source data 4/F8099-1-DKO-RX CI CII ff FF-P20-20X-DCX-128-2-L-dDG-Image Export-25_DAPI.tif]

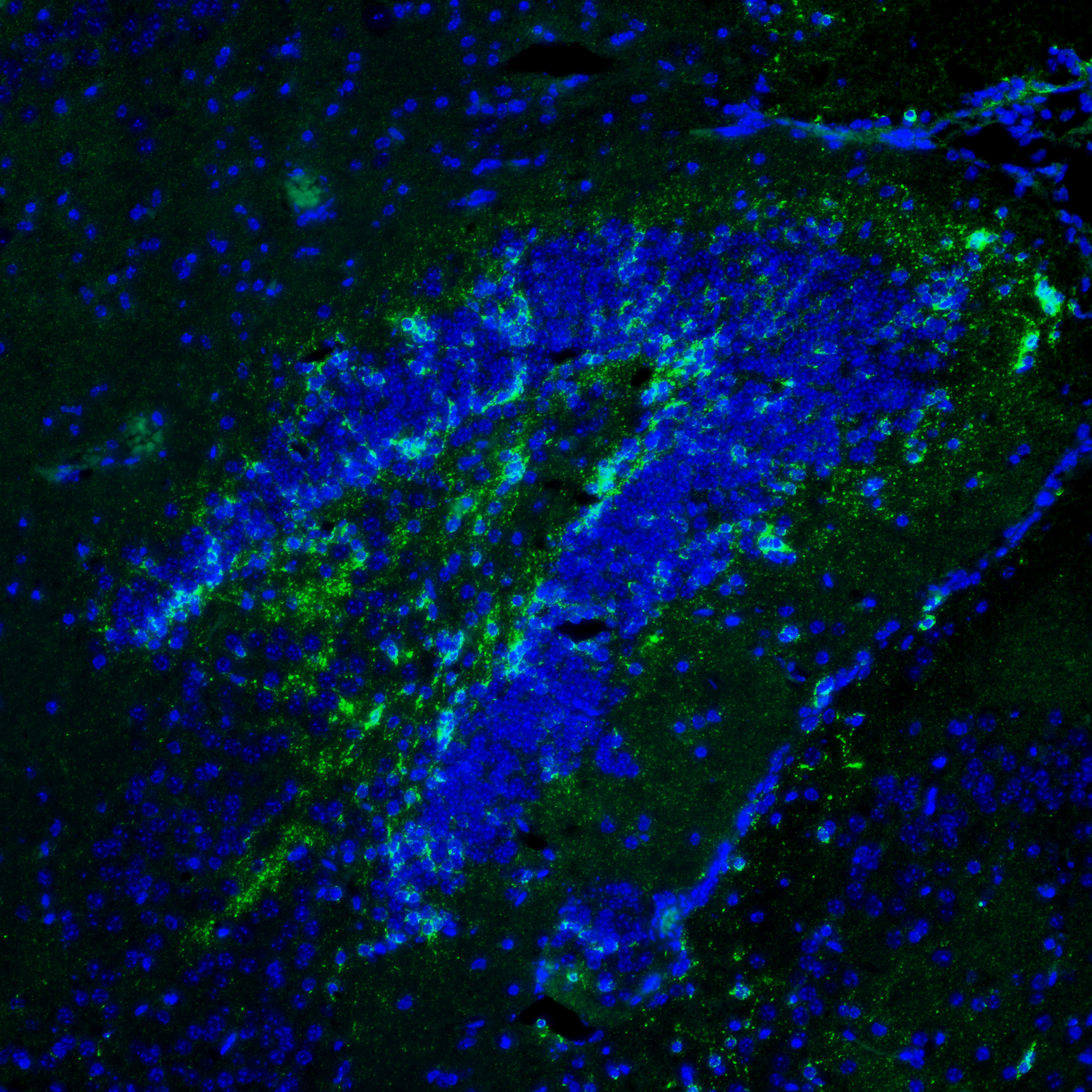

Supplement: Figure 3—figure supplement 1—source data 4. [file elife-86940-fig3-figsupp1-data4.zip › Figure 3-figure supplement 1-source data 4/F8099-1-DKO-RX CI CII ff FF-P20-20X-DCX-128-2-L-dDG-Image Export-25_G+D.tif]

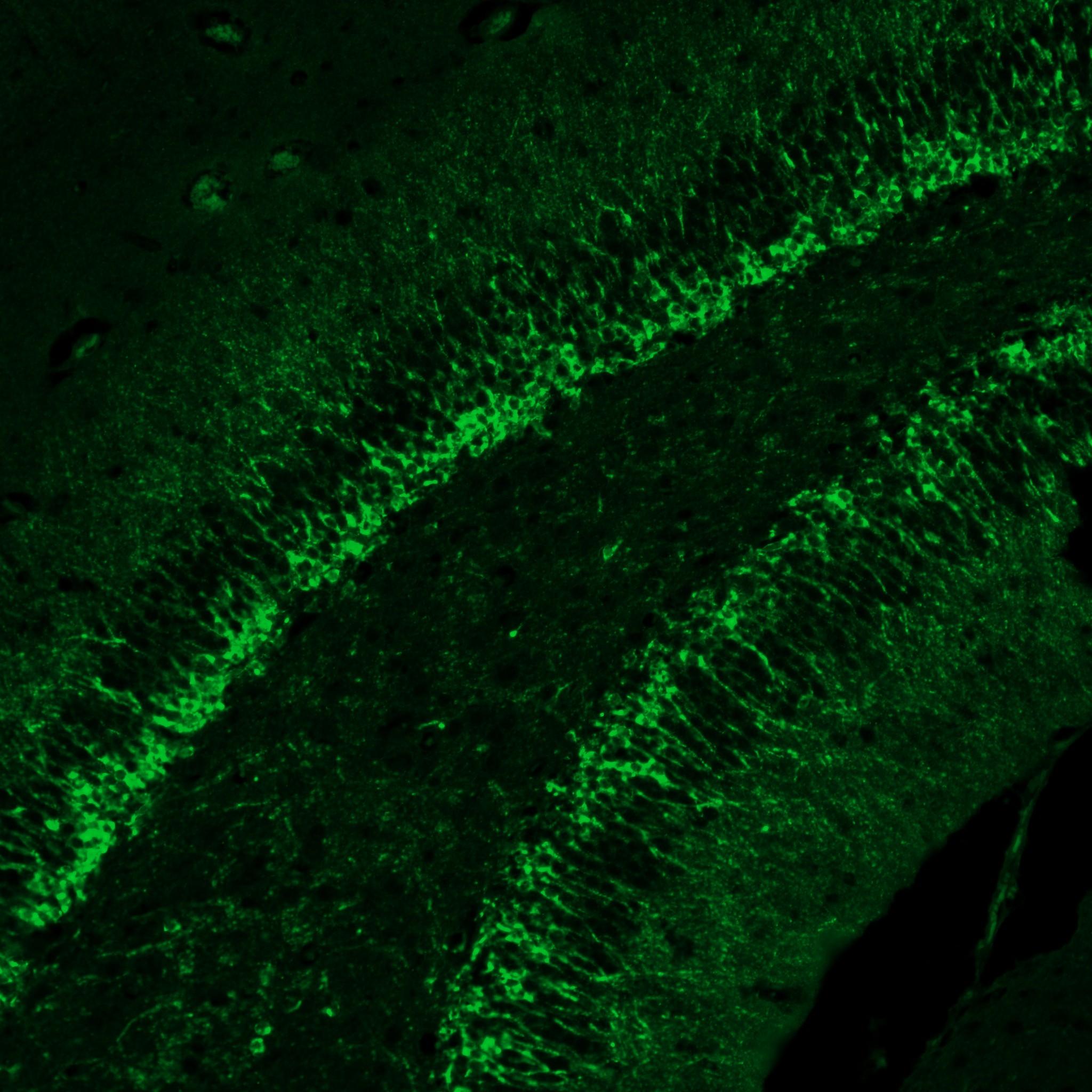

Supplement: Figure 3—figure supplement 1—source data 4. [file elife-86940-fig3-figsupp1-data4.zip › Figure 3-figure supplement 1-source data 4/F8099-3-CON-CI CII ff FF-P20-20X-DCX-144-2-L-dDG-Image Export-26_AF488.tif]

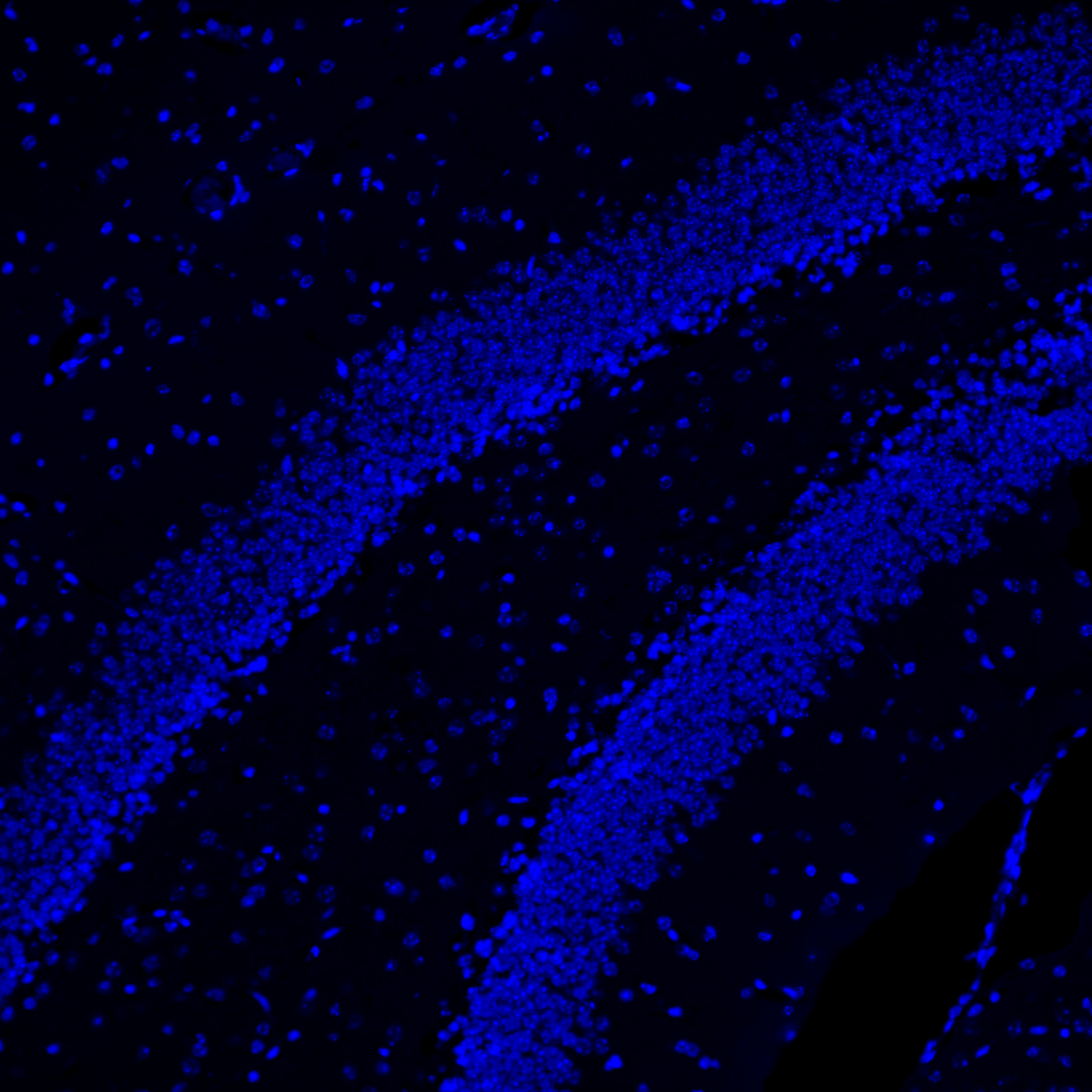

Supplement: Figure 3—figure supplement 1—source data 4. [file elife-86940-fig3-figsupp1-data4.zip › Figure 3-figure supplement 1-source data 4/F8099-3-CON-CI CII ff FF-P20-20X-DCX-144-2-L-dDG-Image Export-26_DAPI.tif]

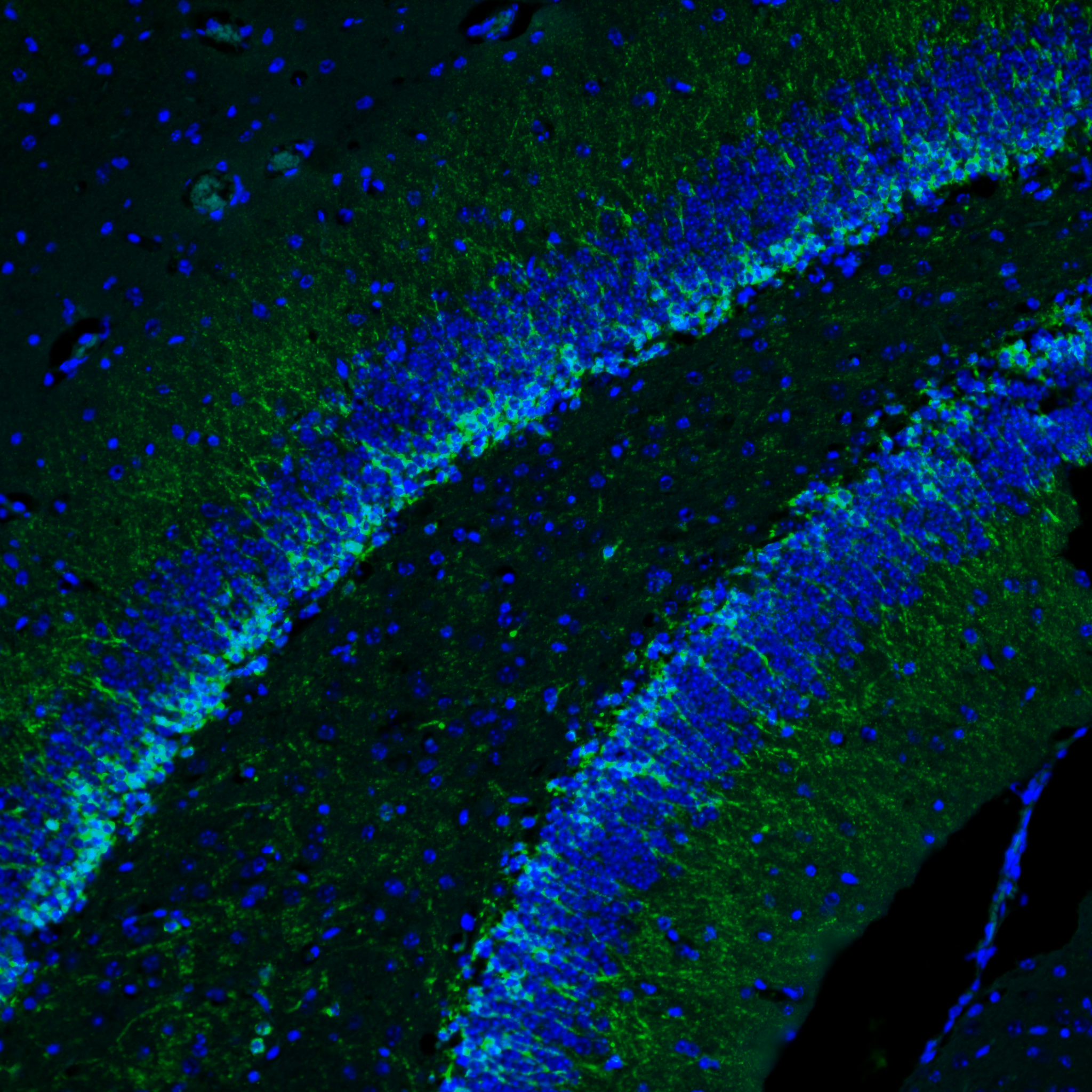

Supplement: Figure 3—figure supplement 1—source data 4. [file elife-86940-fig3-figsupp1-data4.zip › Figure 3-figure supplement 1-source data 4/F8099-3-CON-CI CII ff FF-P20-20X-DCX-144-2-L-dDG-Image Export-26_G+D.tif]

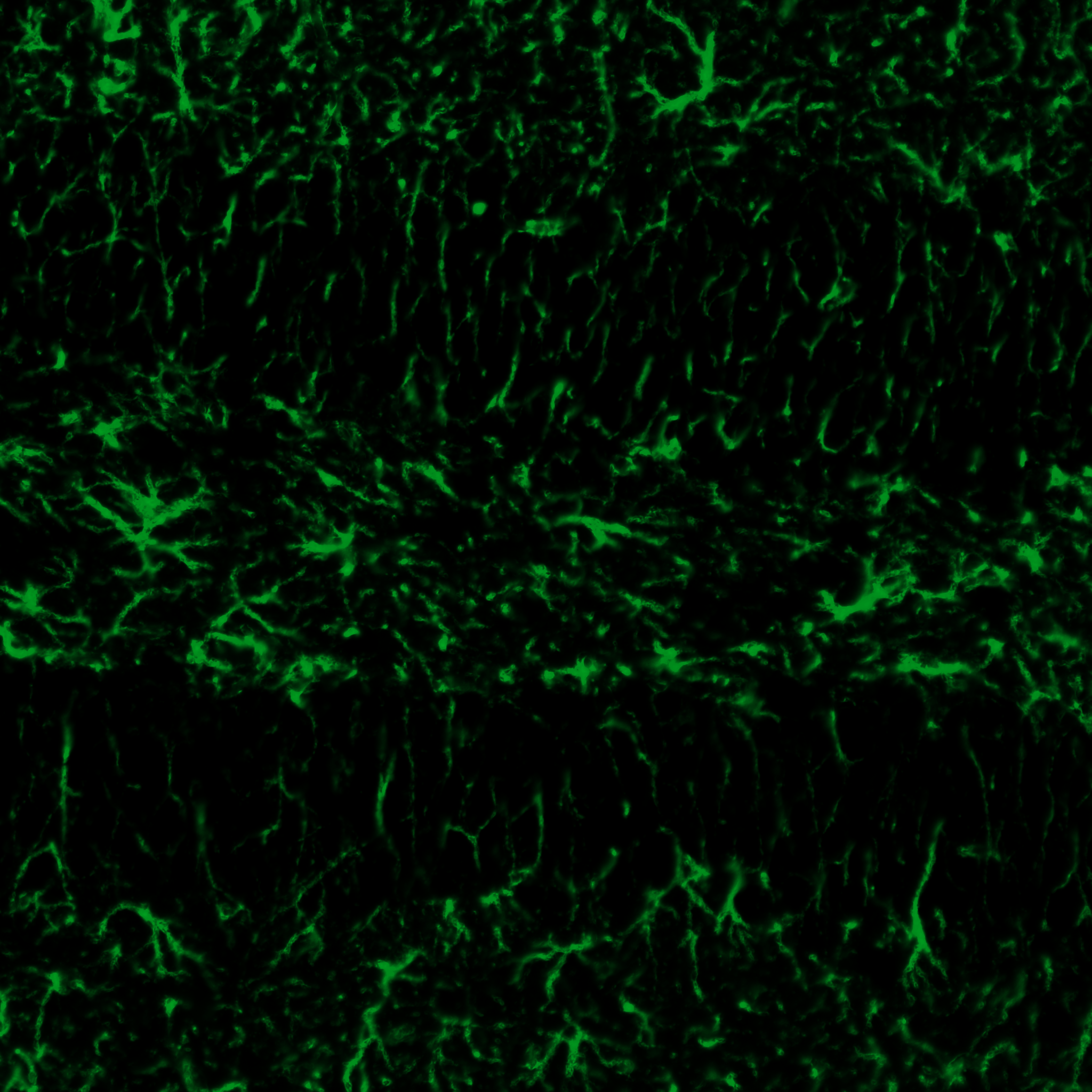

Supplement: Figure 3—figure supplement 1—source data 4. [file elife-86940-fig3-figsupp1-data4.zip › Figure 3-figure supplement 1-source data 4/F3094-2-CI CON-RX CI f+-1M-40X-GFAP-NESTIN-#146-1-dHPC-G+R-Image Export-16_AF488.tif]

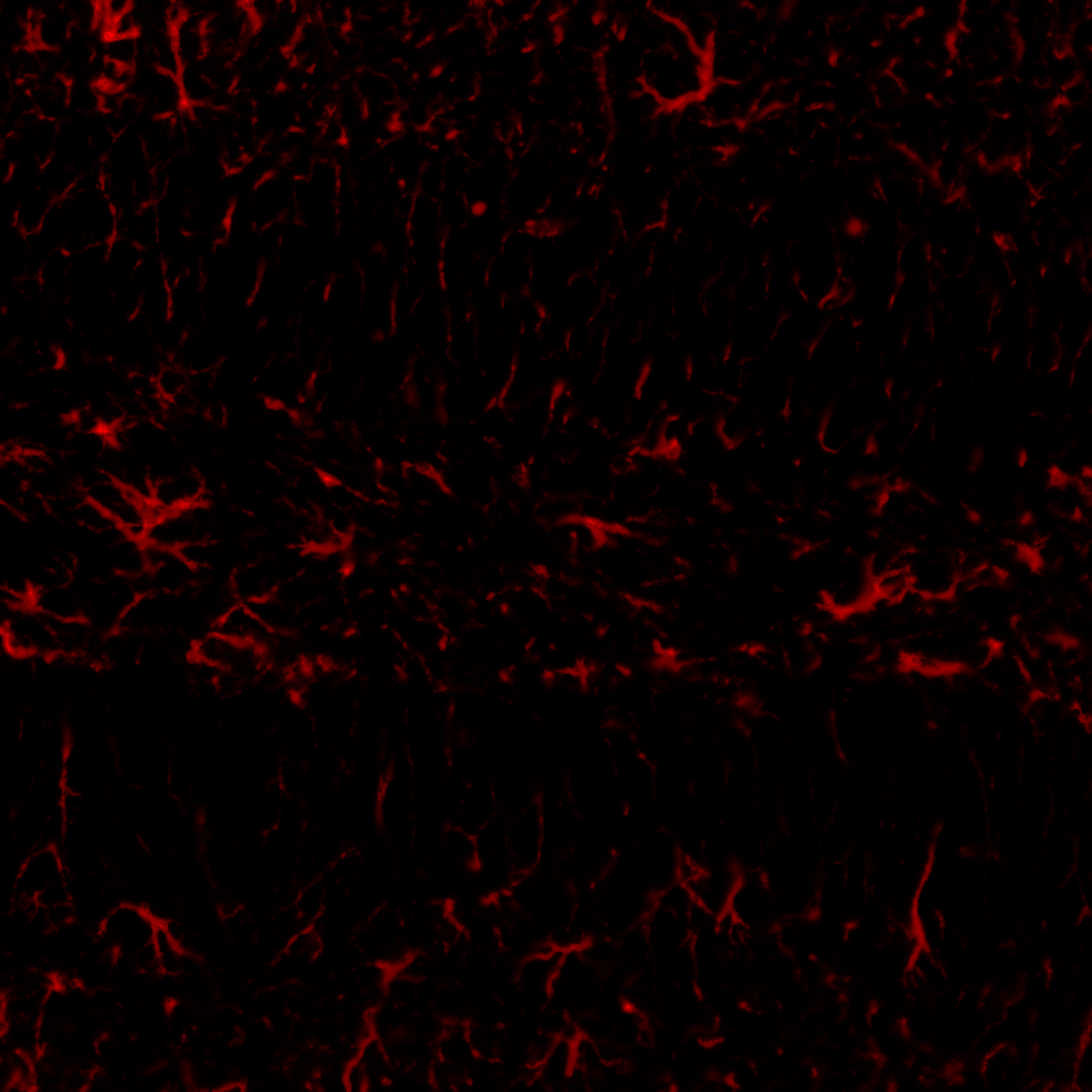

Supplement: Figure 3—figure supplement 1—source data 4. [file elife-86940-fig3-figsupp1-data4.zip › Figure 3-figure supplement 1-source data 4/F3094-2-CI CON-RX CI f+-1M-40X-GFAP-NESTIN-#146-1-dHPC-G+R-Image Export-16_AF594.tif]

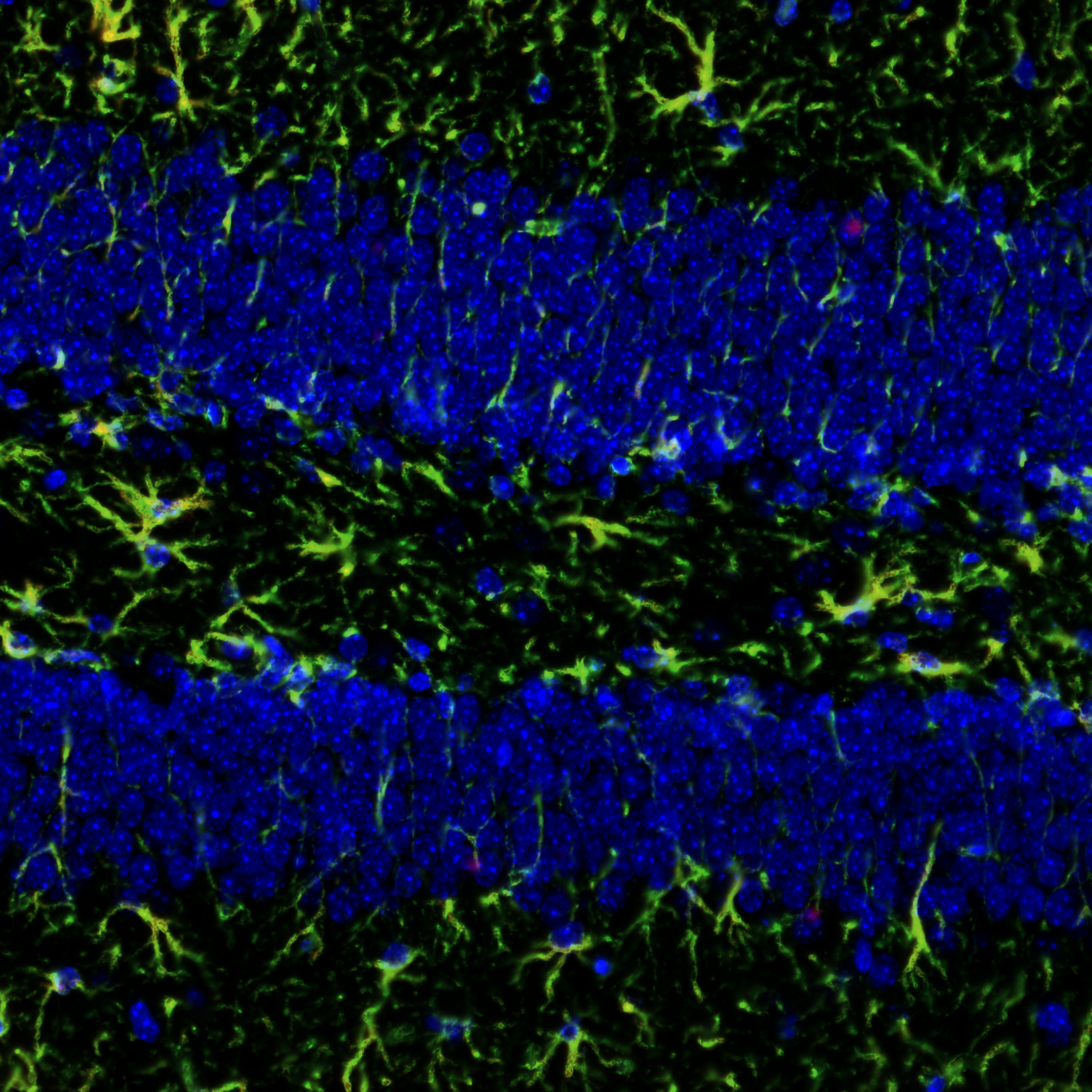

Supplement: Figure 3—figure supplement 1—source data 4. [file elife-86940-fig3-figsupp1-data4.zip › Figure 3-figure supplement 1-source data 4/F3094-2-CI CON-RX CI f+-1M-40X-GFAP-NESTIN-#146-1-dHPC-Image Export-16.tif]

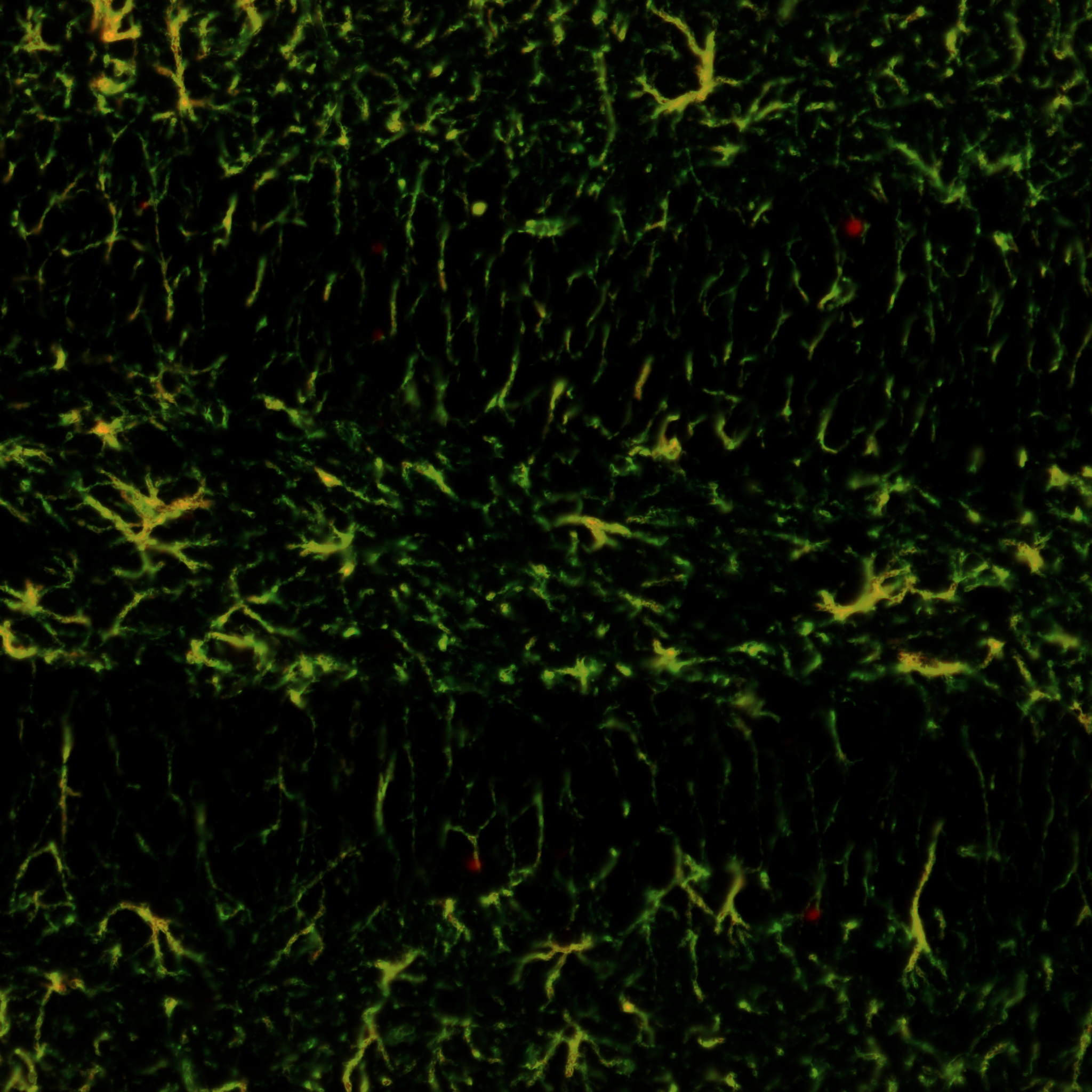

Supplement: Figure 3—figure supplement 1—source data 4. [file elife-86940-fig3-figsupp1-data4.zip › Figure 3-figure supplement 1-source data 4/F3094-2-CI CON-RX CI f+-1M-40X-GFAP-NESTIN-#146-1-dHPC-G+R-Image Export-16.tif]

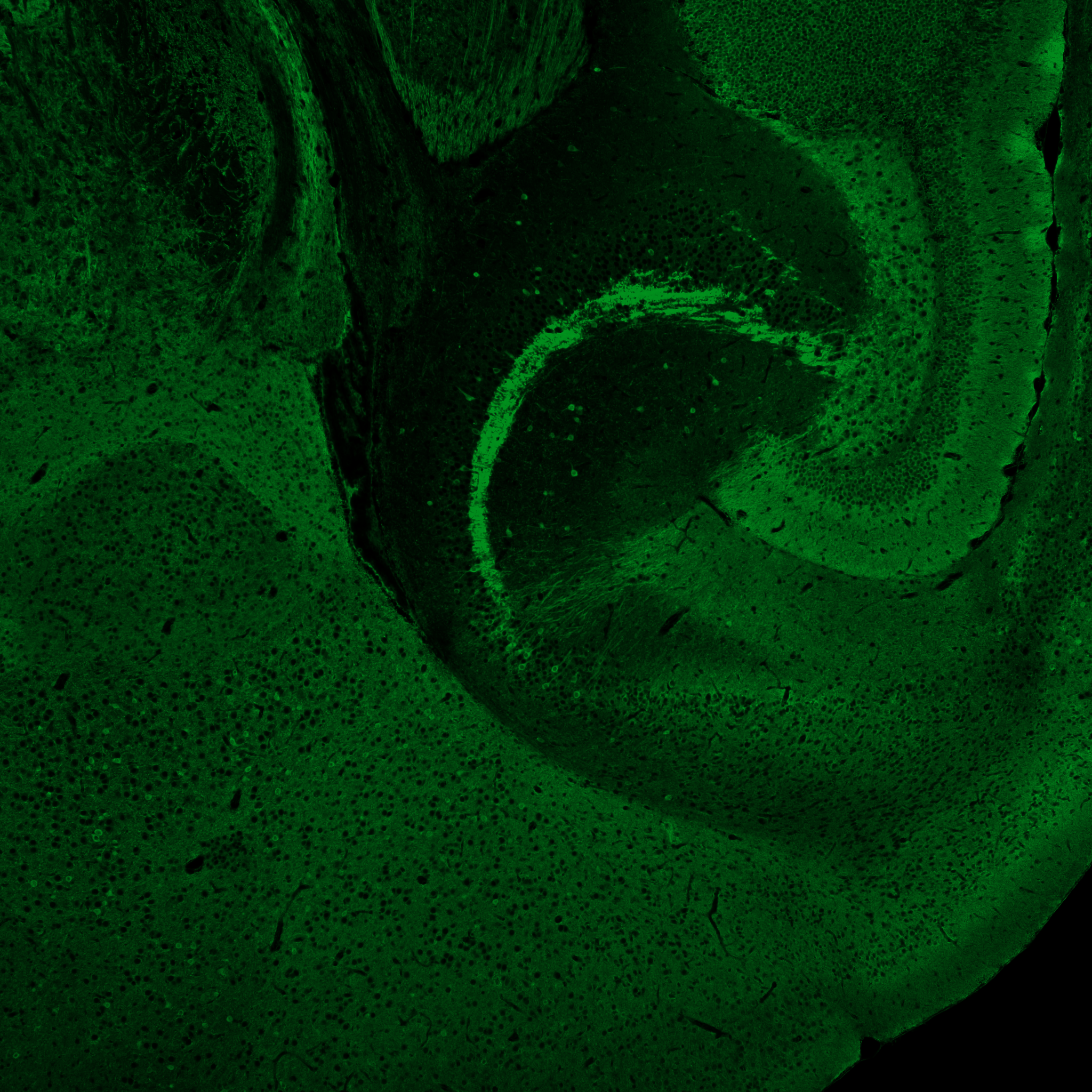

Supplement: Figure 4—source data 1. [file elife-86940-fig4-data1.zip › Figure 4-source data 1/35-CON-CII F+-1M-SAGITAL-CB-61#-4-5X-vHPC-Image Export-16_AF488.tif]

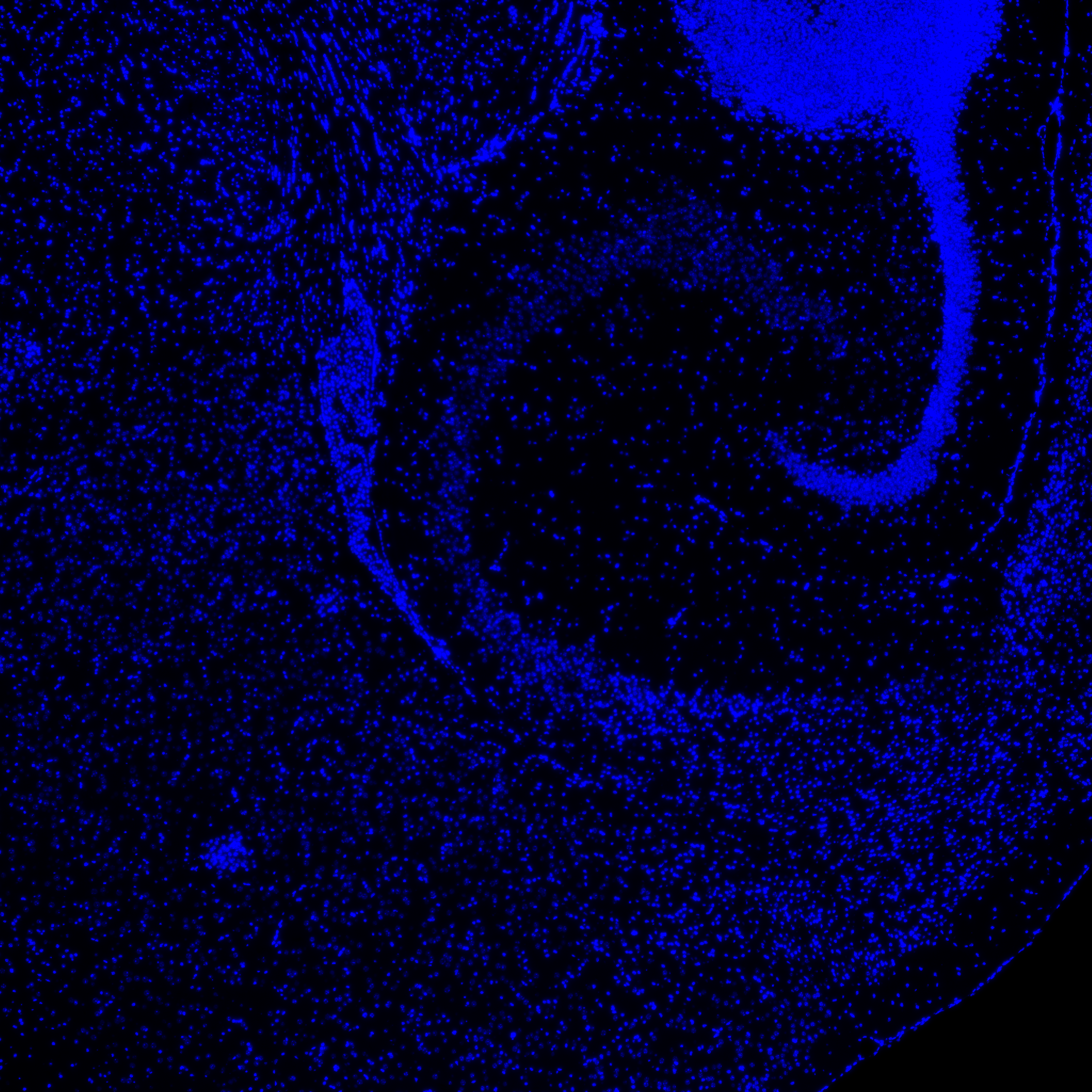

Supplement: Figure 4—source data 1. [file elife-86940-fig4-data1.zip › Figure 4-source data 1/35-CON-CII F+-1M-SAGITAL-CB-61#-4-5X-vHPC-Image Export-16_DAPI.tif]

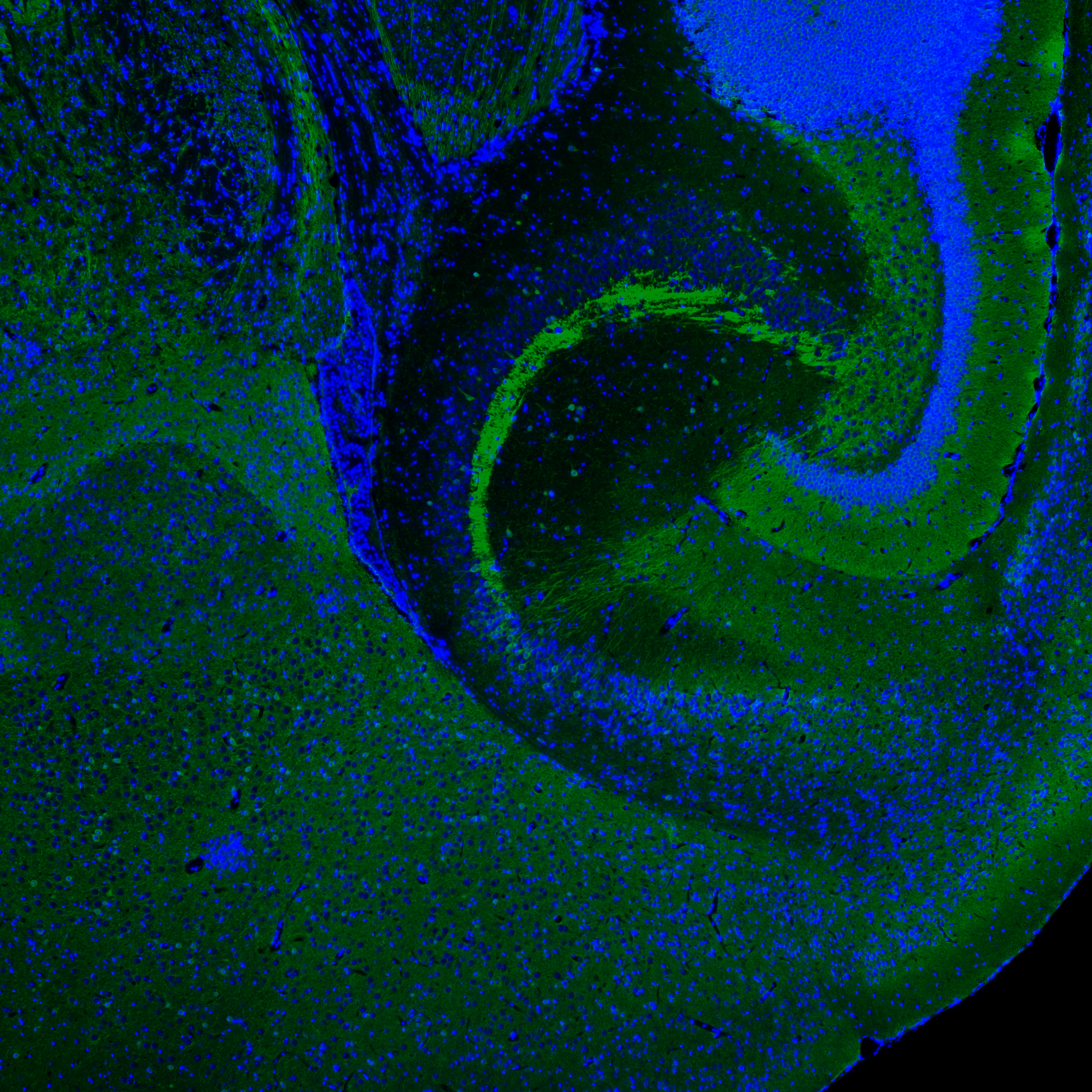

Supplement: Figure 4—source data 1. [file elife-86940-fig4-data1.zip › Figure 4-source data 1/35-CON-CII F+-1M-SAGITAL-CB-61#-4-5X-vHPC-Image Export-16_G+D.tif]

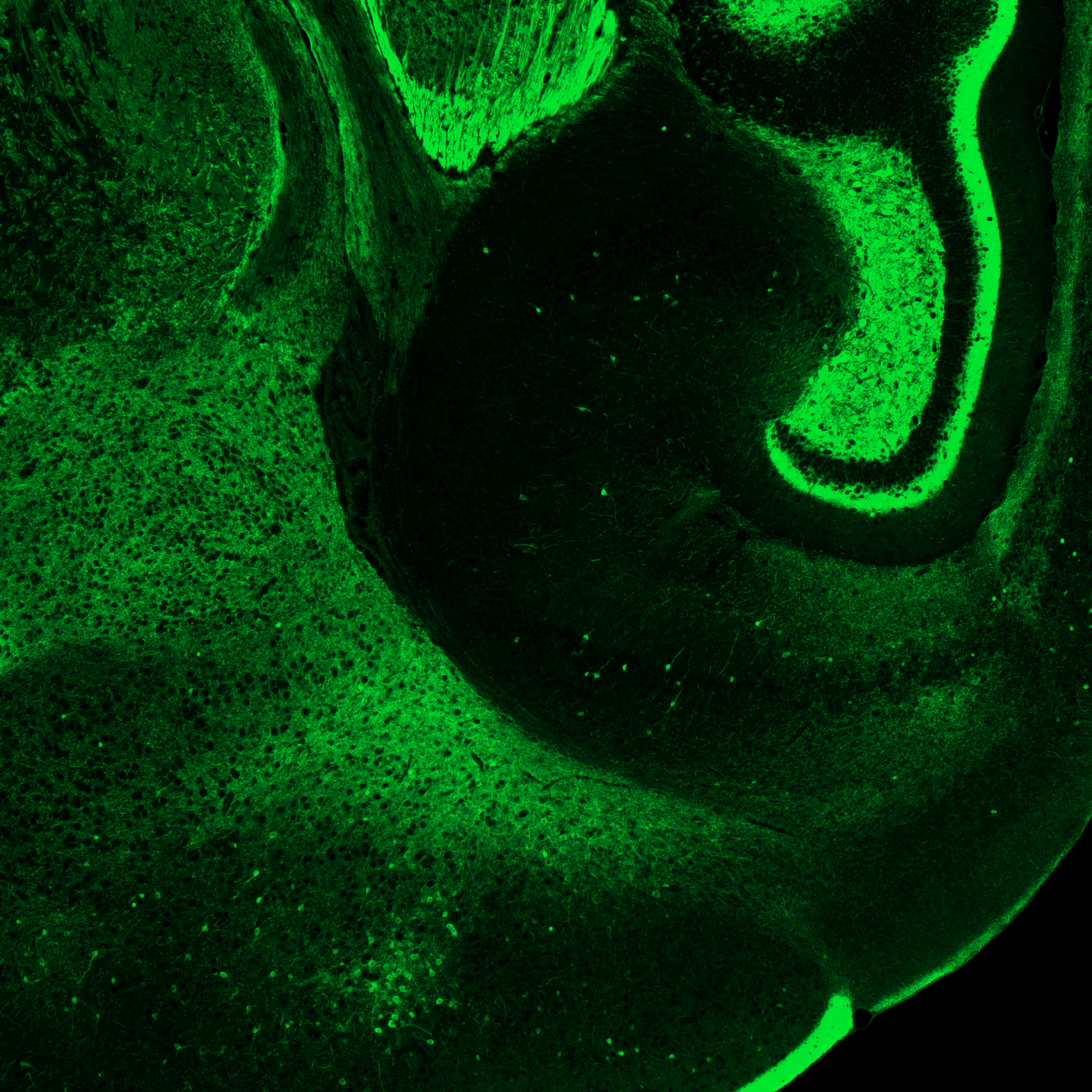

Supplement: Figure 4—source data 1. [file elife-86940-fig4-data1.zip › Figure 4-source data 1/35-CON-CII F+-1M-SAGITAL-CR-61#-1-5X-vHPC-Image Export-04_AF488.tif]

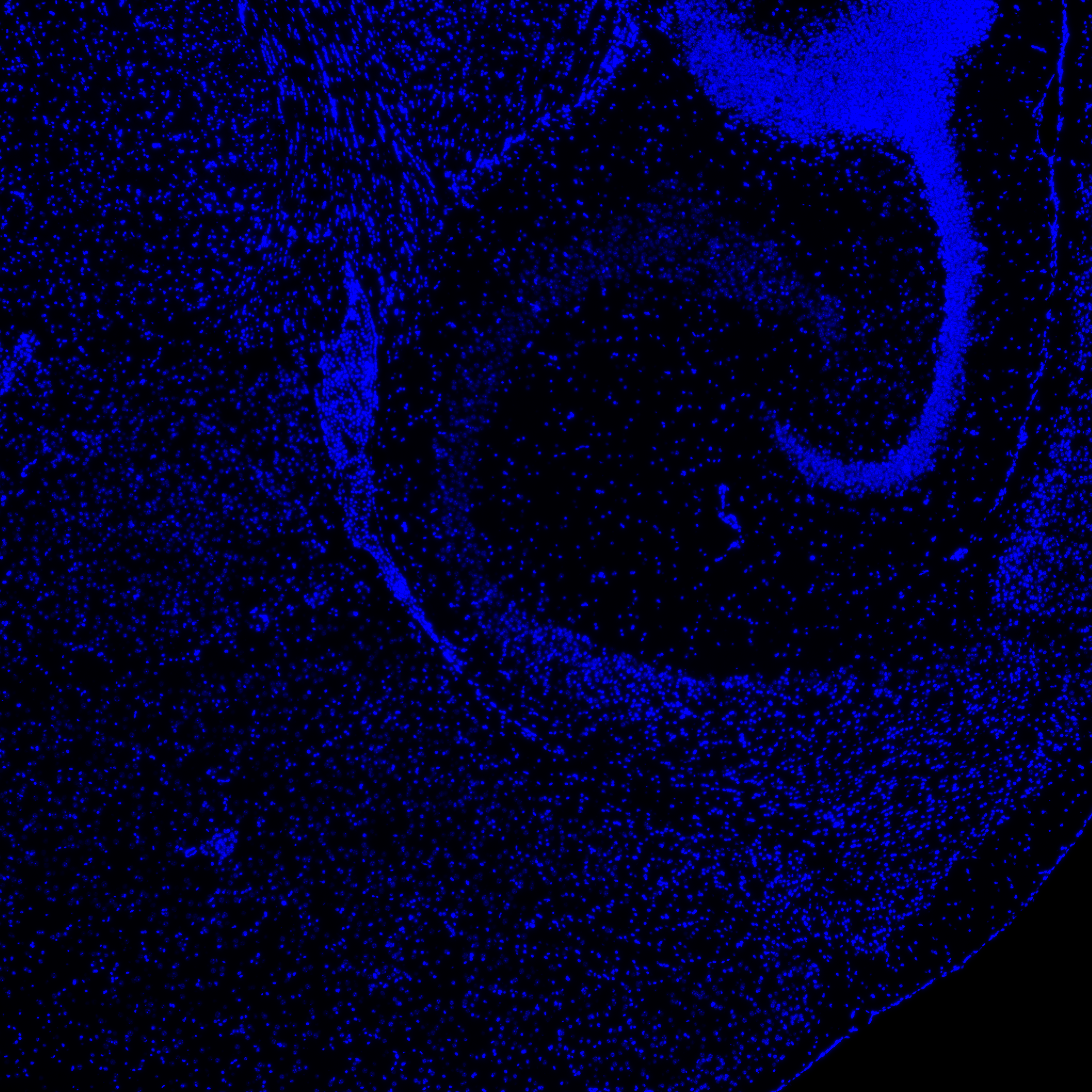

Supplement: Figure 4—source data 1. [file elife-86940-fig4-data1.zip › Figure 4-source data 1/35-CON-CII F+-1M-SAGITAL-CR-61#-1-5X-vHPC-Image Export-04_DAPI.tif]

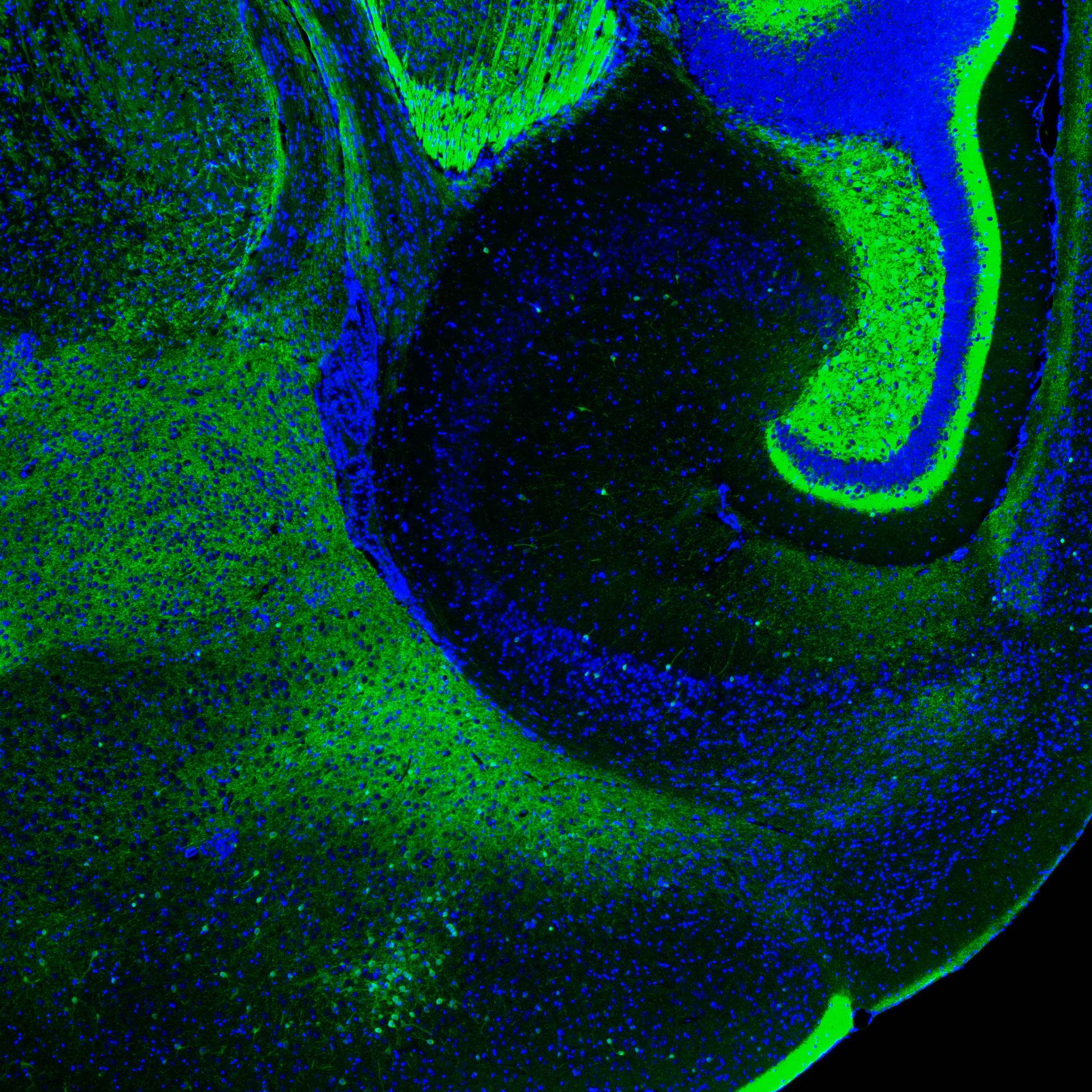

Supplement: Figure 4—source data 1. [file elife-86940-fig4-data1.zip › Figure 4-source data 1/35-CON-CII F+-1M-SAGITAL-CR-61#-1-5X-vHPC-Image Export-04_G+D.tif]

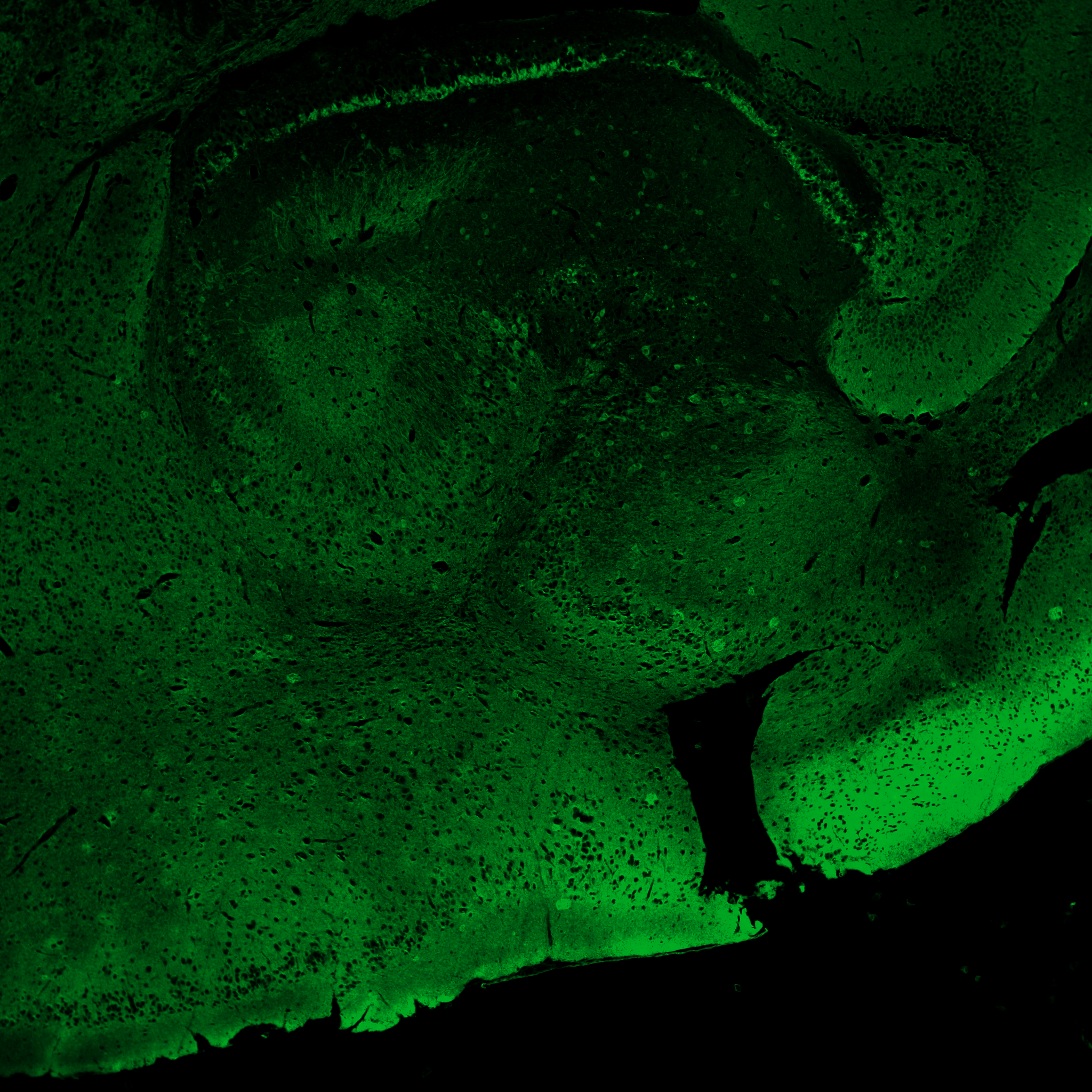

Supplement: Figure 4—source data 1. [file elife-86940-fig4-data1.zip › Figure 4-source data 1/36-CKO-RX CII FF-1M-SAGITAL-CB-55#-4-5X-vHPC-Image Export-32_AF488.tif]

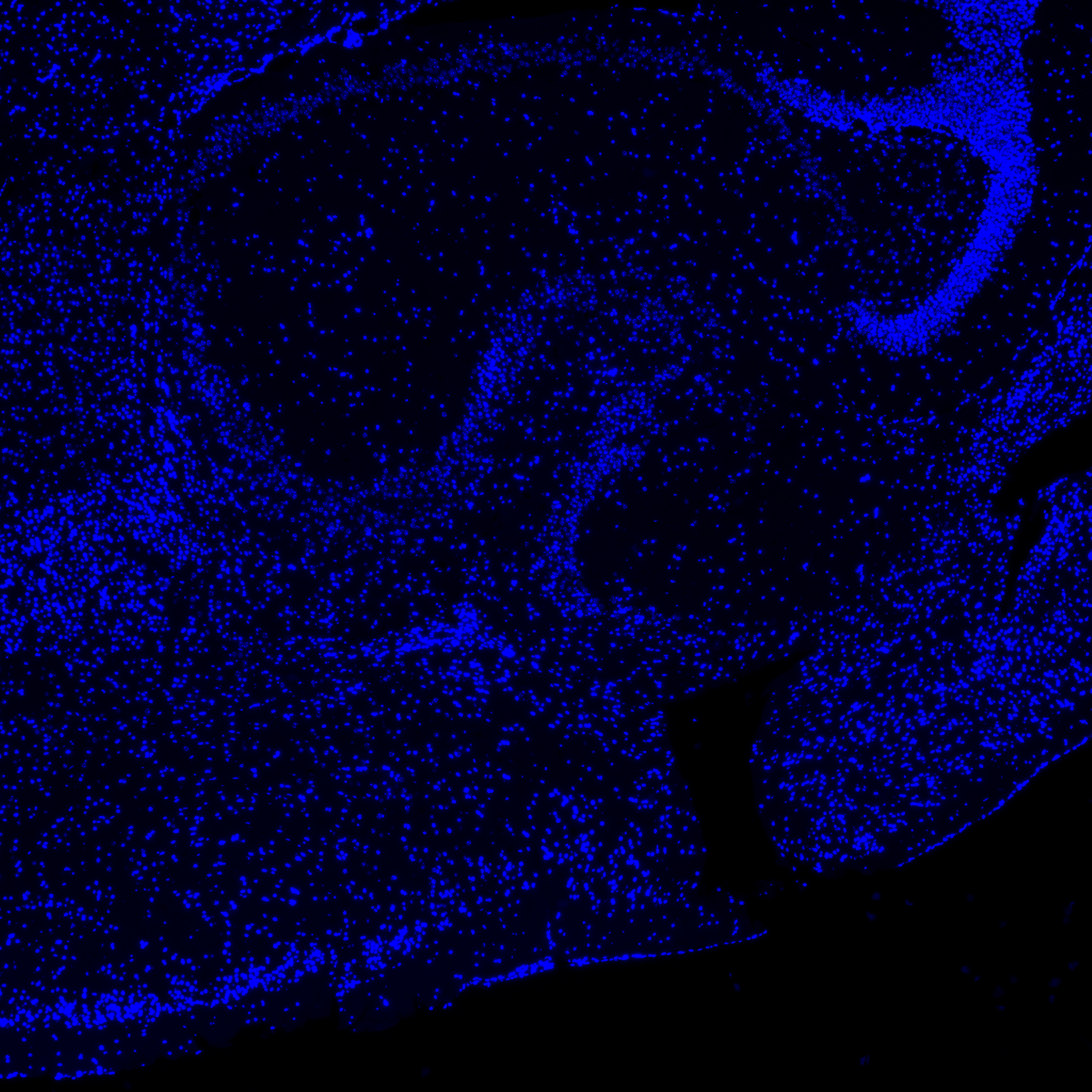

Supplement: Figure 4—source data 1. [file elife-86940-fig4-data1.zip › Figure 4-source data 1/36-CKO-RX CII FF-1M-SAGITAL-CB-55#-4-5X-vHPC-Image Export-32_DAPI.tif]

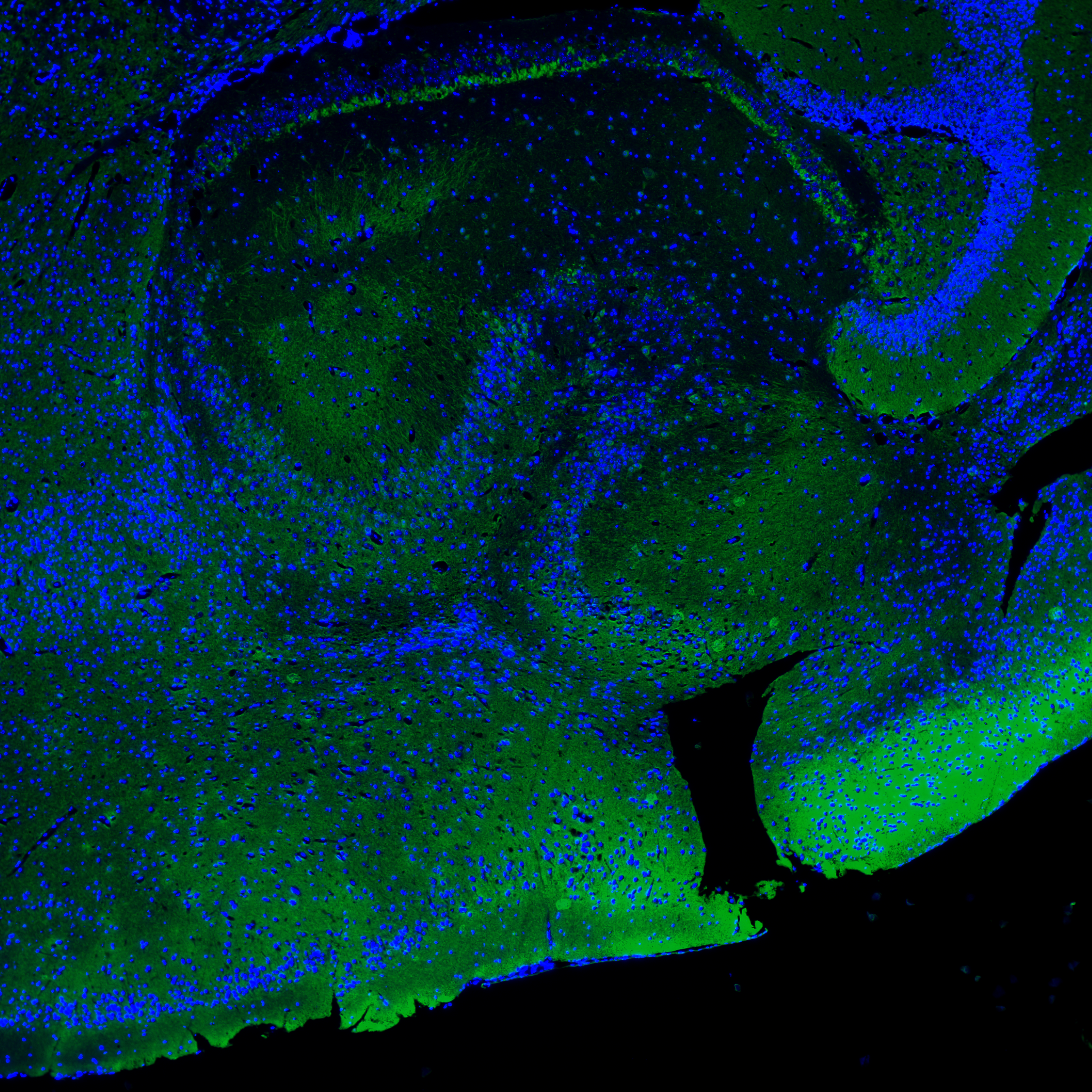

Supplement: Figure 4—source data 1. [file elife-86940-fig4-data1.zip › Figure 4-source data 1/36-CKO-RX CII FF-1M-SAGITAL-CB-55#-4-5X-vHPC-Image Export-32_G+D.tif]

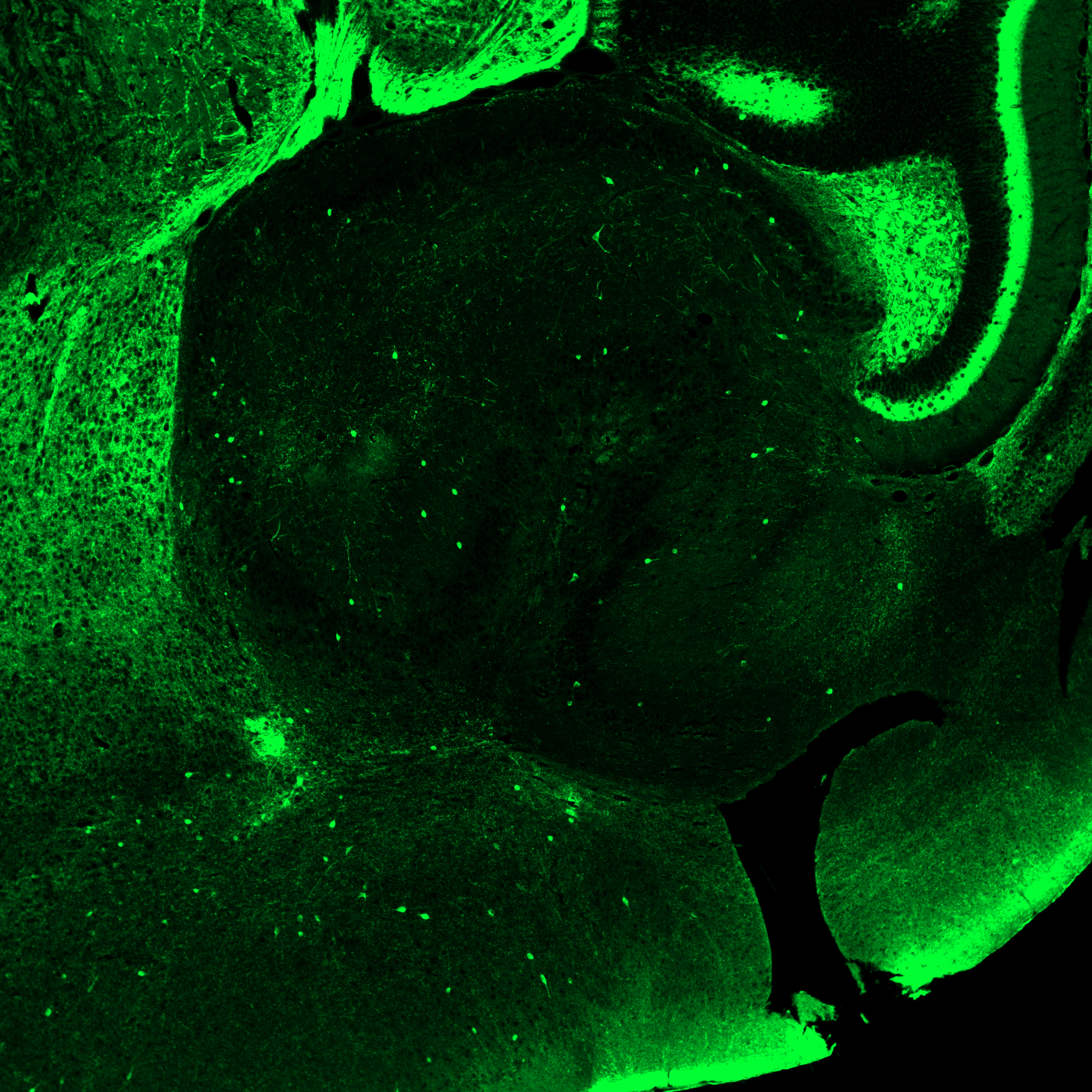

Supplement: Figure 4—source data 1. [file elife-86940-fig4-data1.zip › Figure 4-source data 1/36-CKO-RX CII FF-1M-SAGITAL-CR-55#-1-5X-vHPC-Image Export-20_AF488.tif]

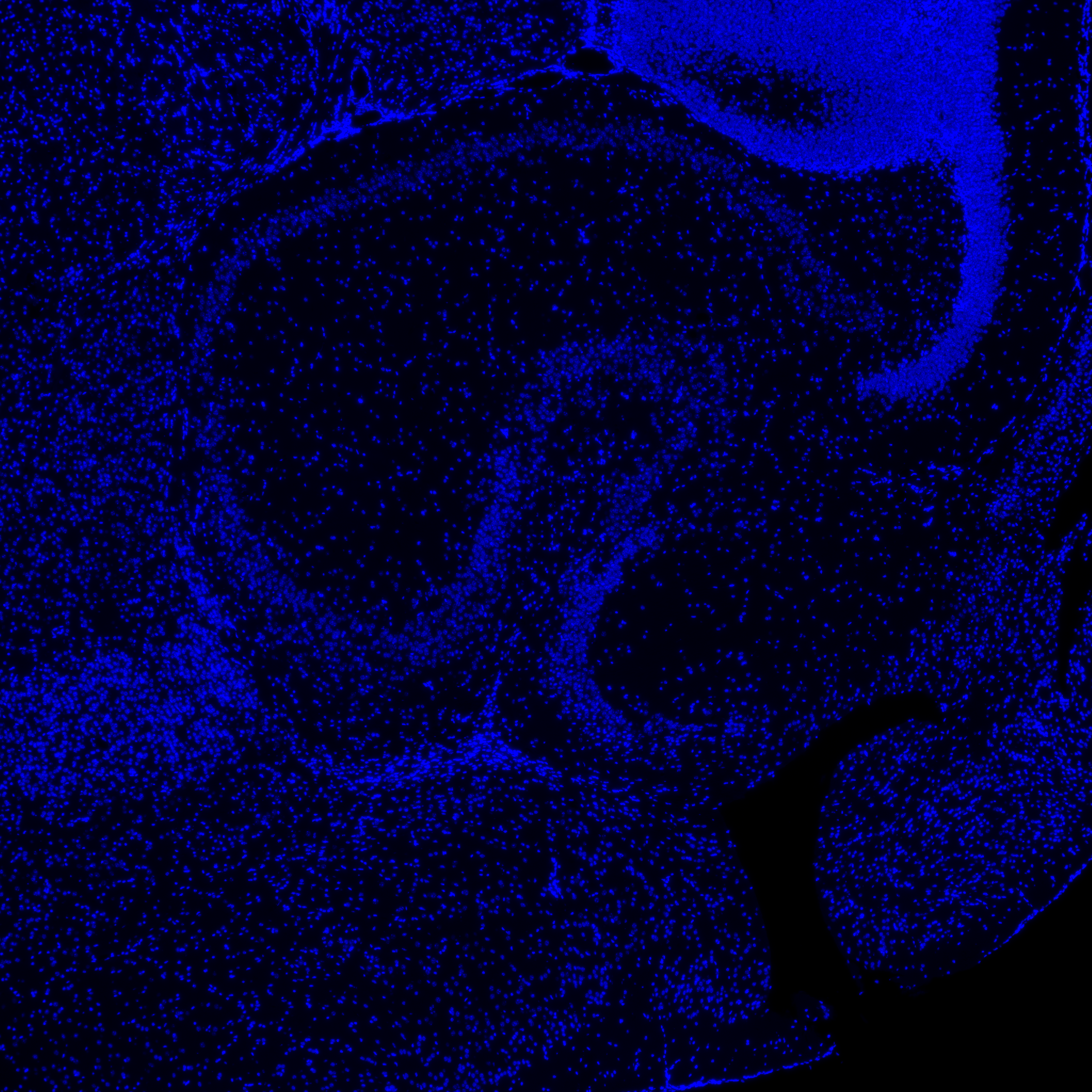

Supplement: Figure 4—source data 1. [file elife-86940-fig4-data1.zip › Figure 4-source data 1/36-CKO-RX CII FF-1M-SAGITAL-CR-55#-1-5X-vHPC-Image Export-20_DAPI.tif]

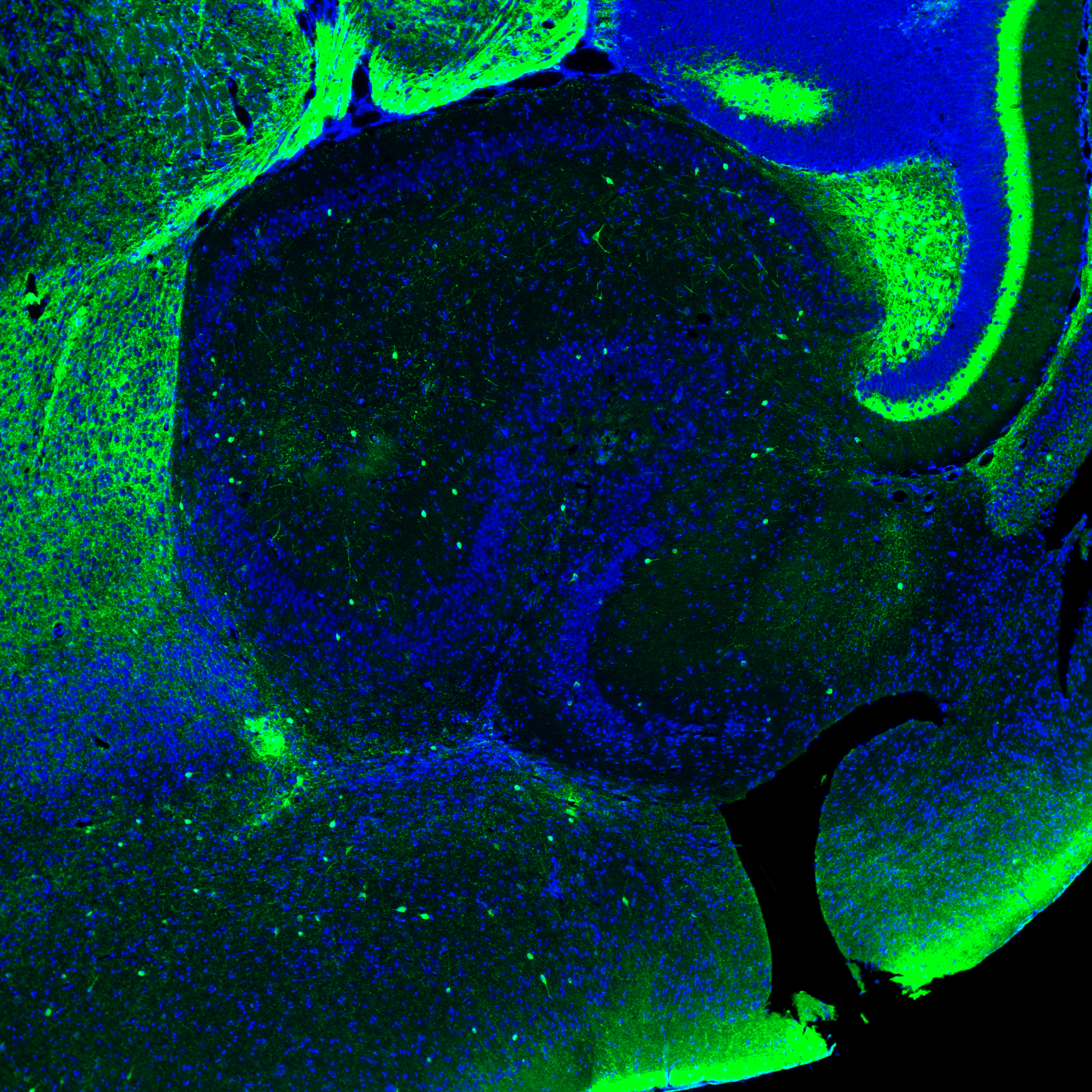

Supplement: Figure 4—source data 1. [file elife-86940-fig4-data1.zip › Figure 4-source data 1/36-CKO-RX CII FF-1M-SAGITAL-CR-55#-1-5X-vHPC-Image Export-20_G+D.tif]

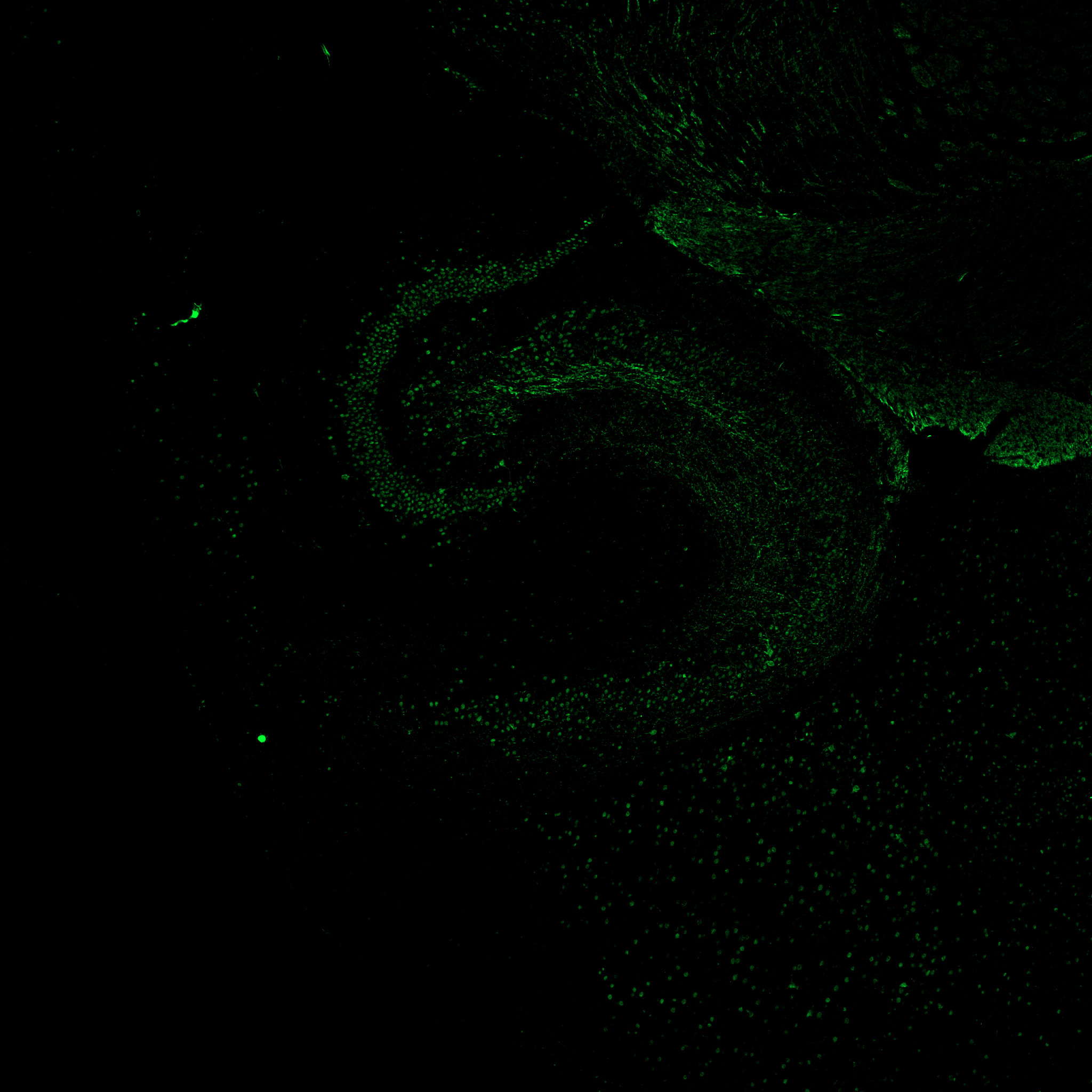

Supplement: Figure 4—source data 1. [file elife-86940-fig4-data1.zip › Figure 4-source data 1/2879-CON-CII FF-1M-5X-SMI312-2-vHPC-Image Export-18_AF488.tif]

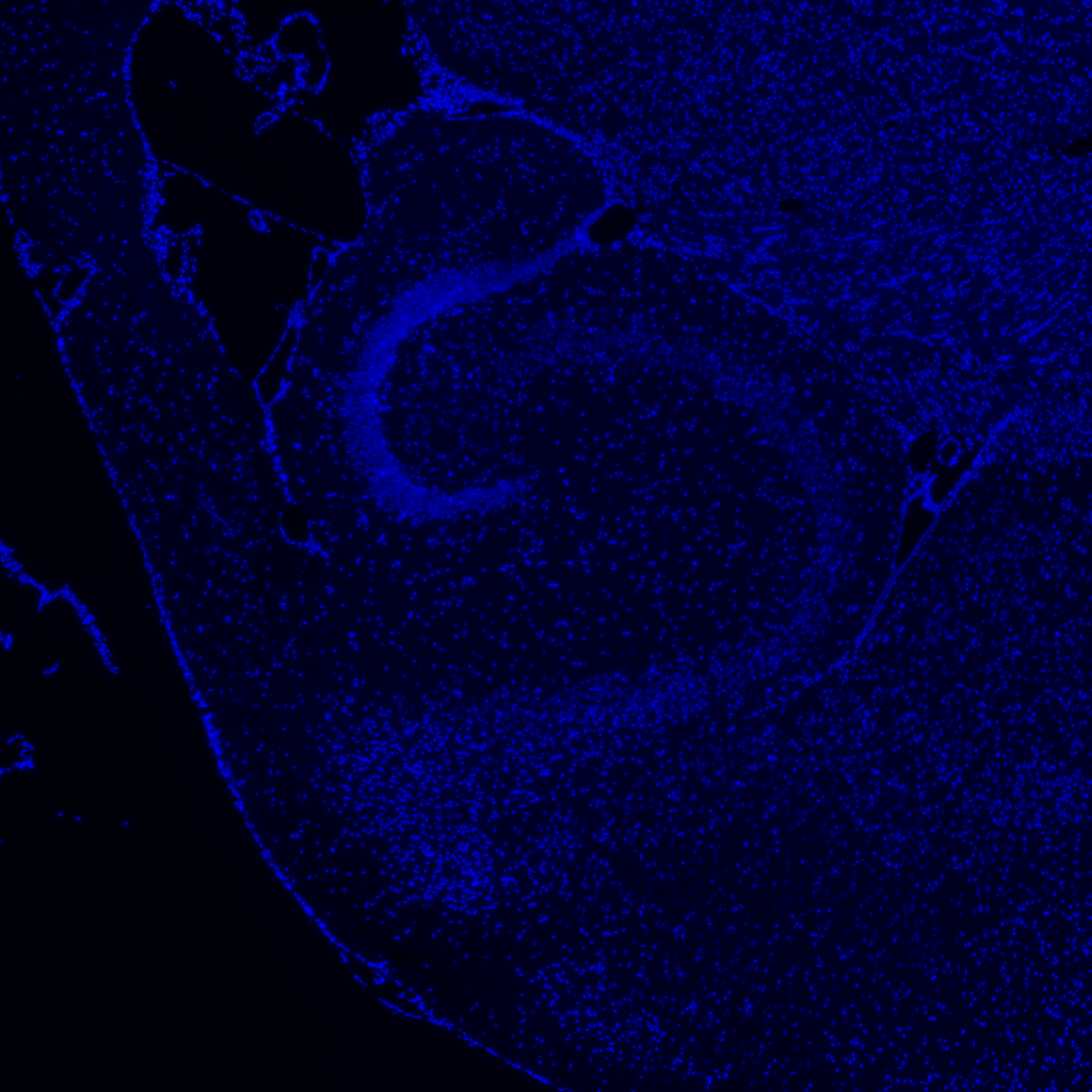

Supplement: Figure 4—source data 1. [file elife-86940-fig4-data1.zip › Figure 4-source data 1/2879-CON-CII FF-1M-5X-SMI312-2-vHPC-Image Export-18_DAPI.tif]

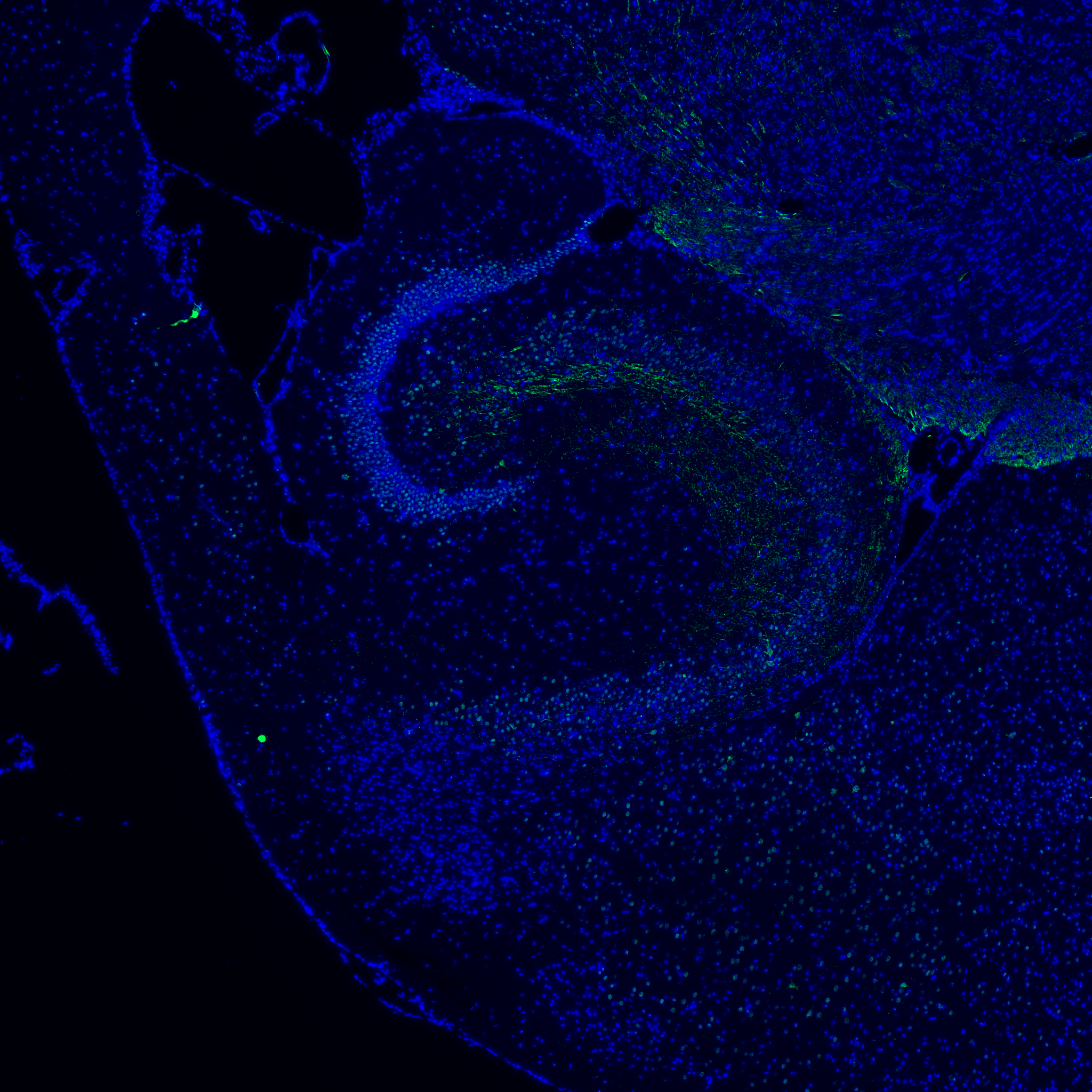

Supplement: Figure 4—source data 1. [file elife-86940-fig4-data1.zip › Figure 4-source data 1/2879-CON-CII FF-1M-5X-SMI312-2-vHPC-Image Export-18.tif]

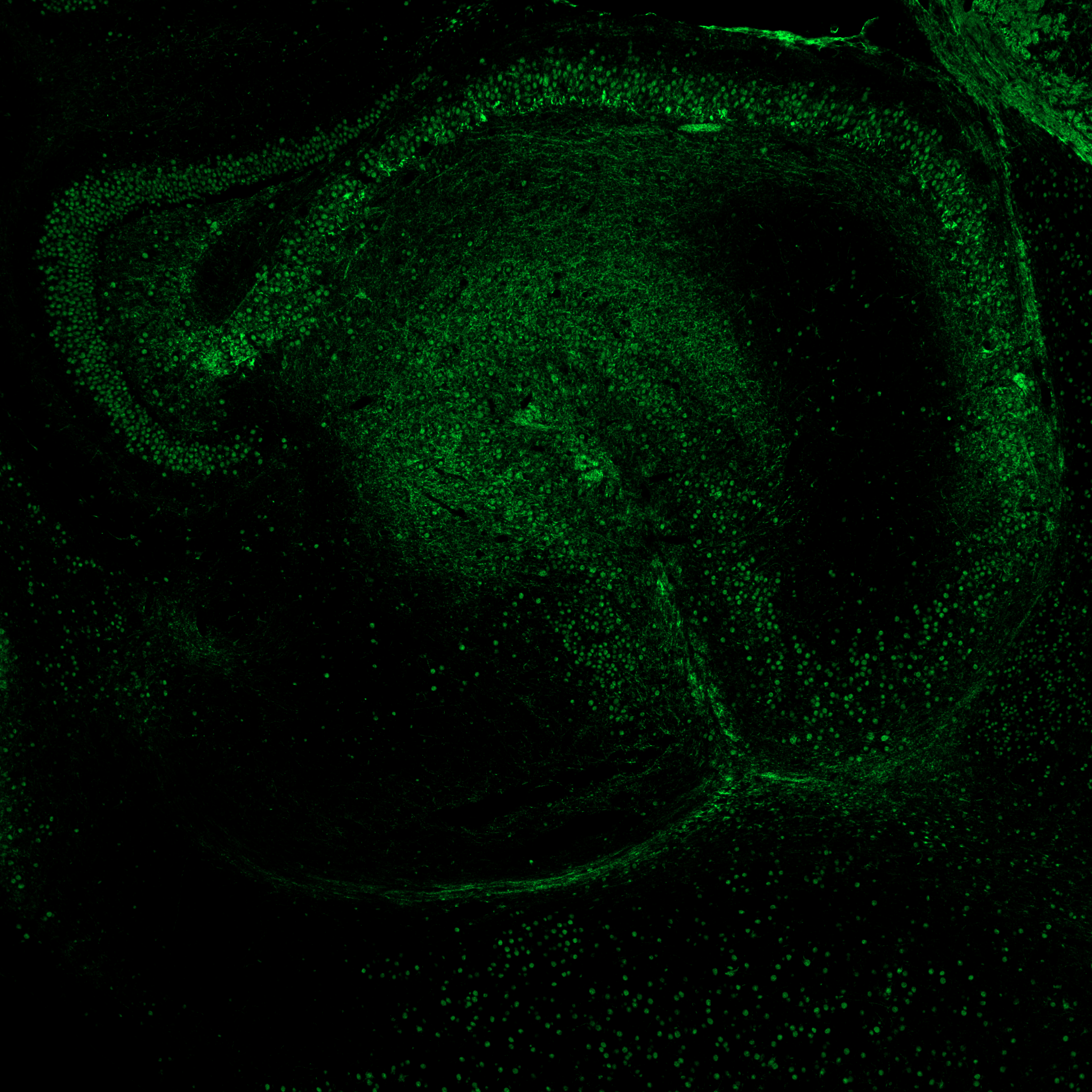

Supplement: Figure 4—source data 1. [file elife-86940-fig4-data1.zip › Figure 4-source data 1/2881-CII CKO-RX CII FF-1M-5X-SMI312-2-vHPC-Image Export-14_AF488.tif]

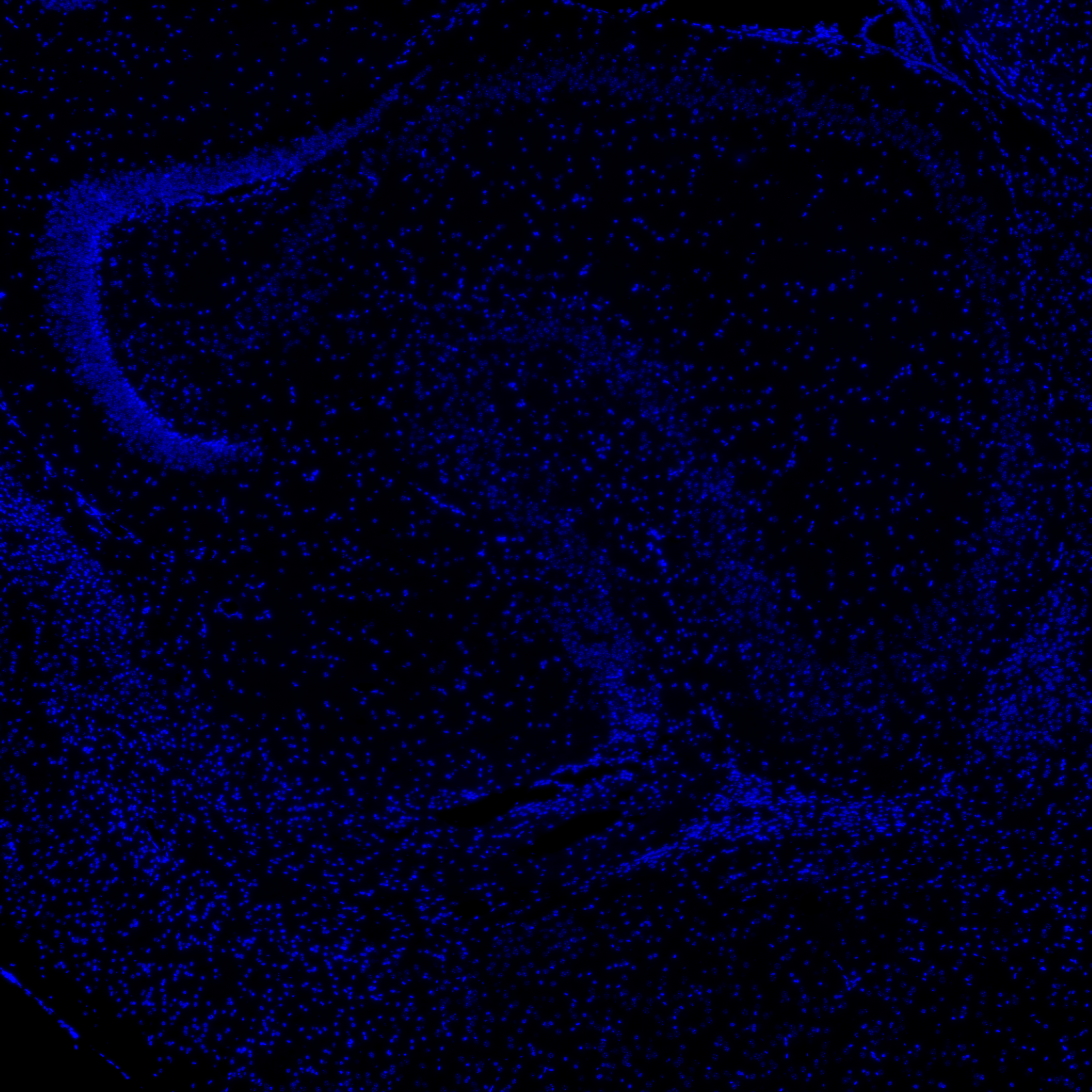

Supplement: Figure 4—source data 1. [file elife-86940-fig4-data1.zip › Figure 4-source data 1/2881-CII CKO-RX CII FF-1M-5X-SMI312-2-vHPC-Image Export-14_DAPI.tif]

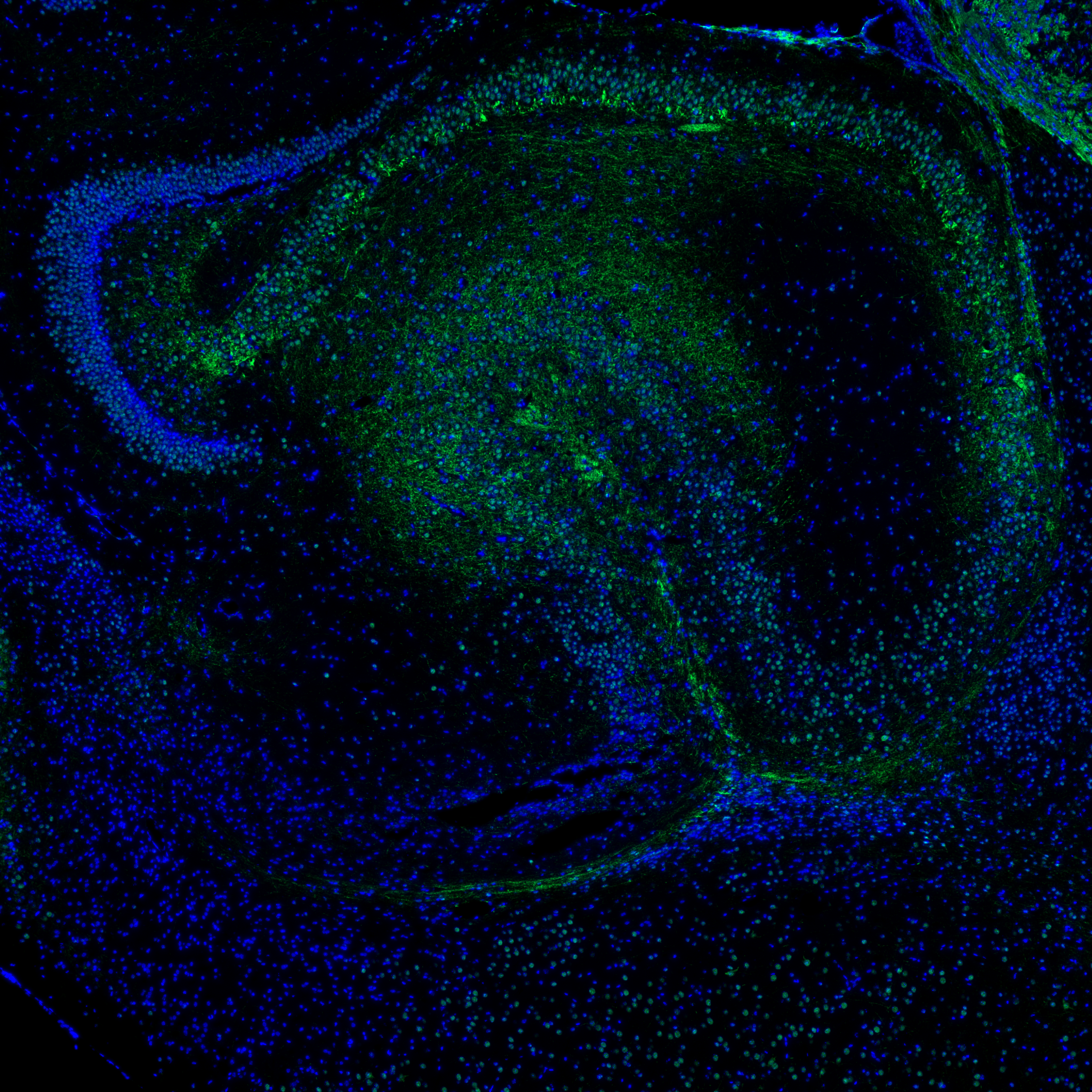

Supplement: Figure 4—source data 1. [file elife-86940-fig4-data1.zip › Figure 4-source data 1/2881-CII CKO-RX CII FF-1M-5X-SMI312-2-vHPC-Image Export-14.tif]

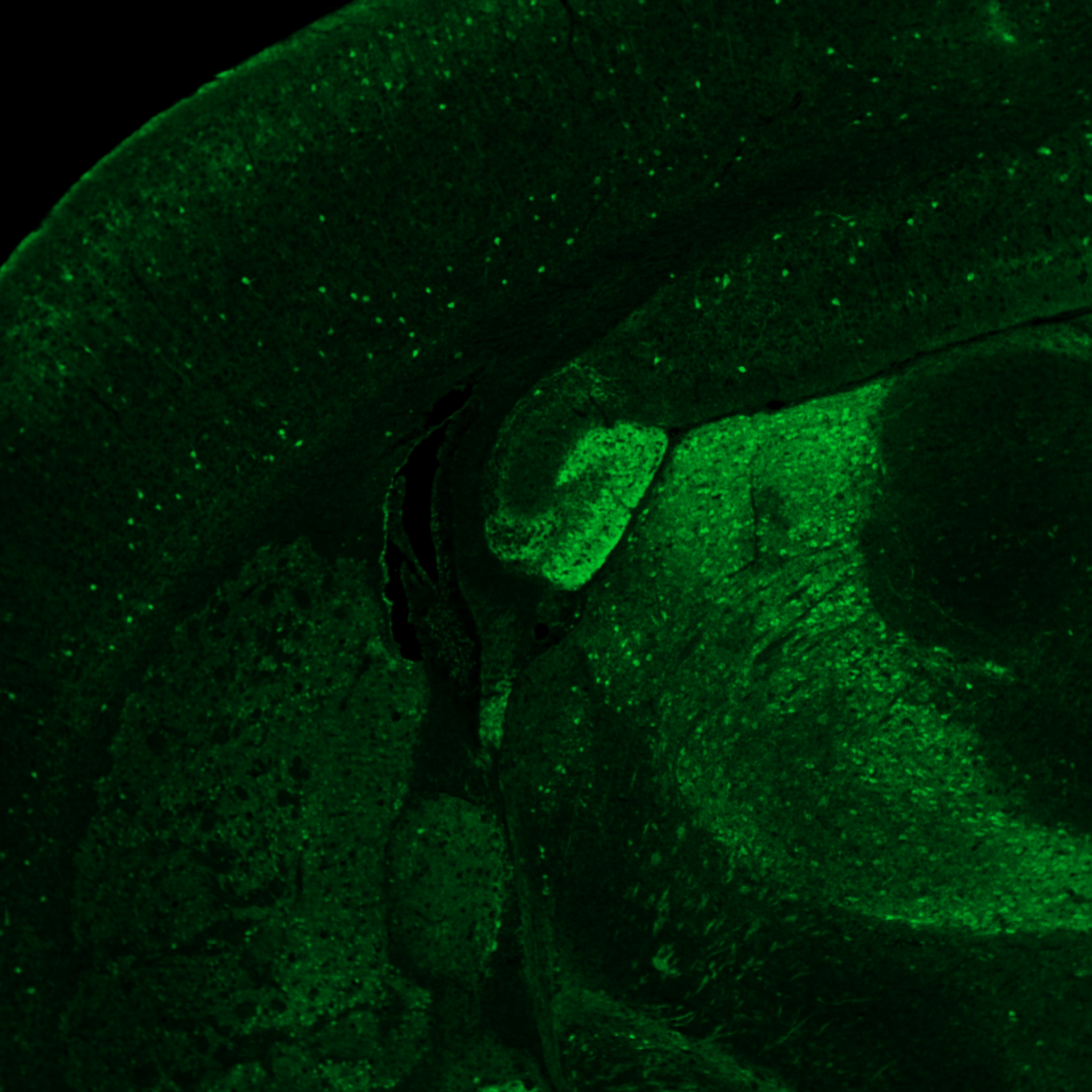

Supplement: Figure 4—source data 2. [file elife-86940-fig4-data2.zip › Figure 4-source data 2/F449-1-DKO-RX FF ff-P18-CB-125#-4-5X-left dHPC-Image Export-26_AF488.tif]

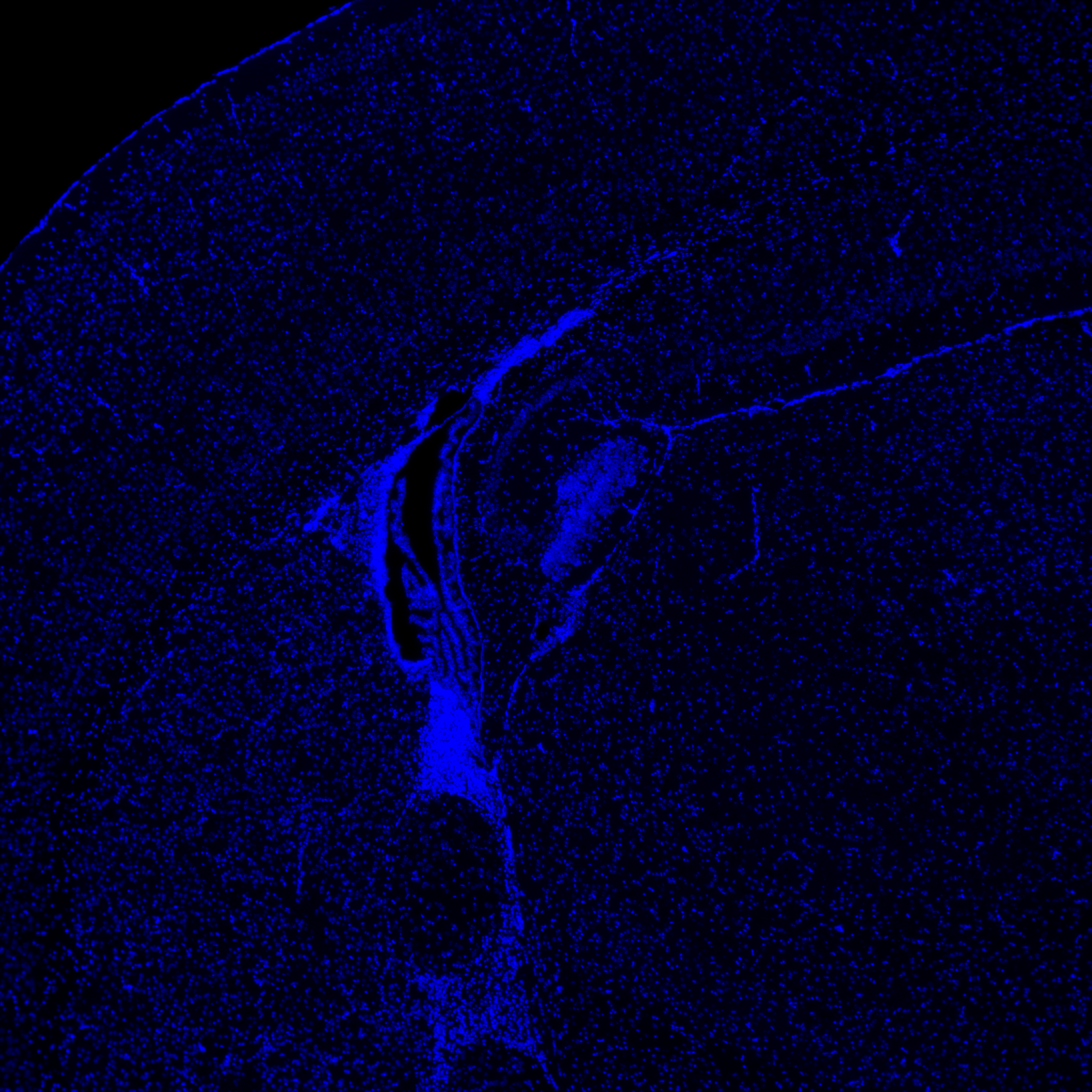

Supplement: Figure 4—source data 2. [file elife-86940-fig4-data2.zip › Figure 4-source data 2/F449-1-DKO-RX FF ff-P18-CB-125#-4-5X-left dHPC-Image Export-26_DAPI.tif]

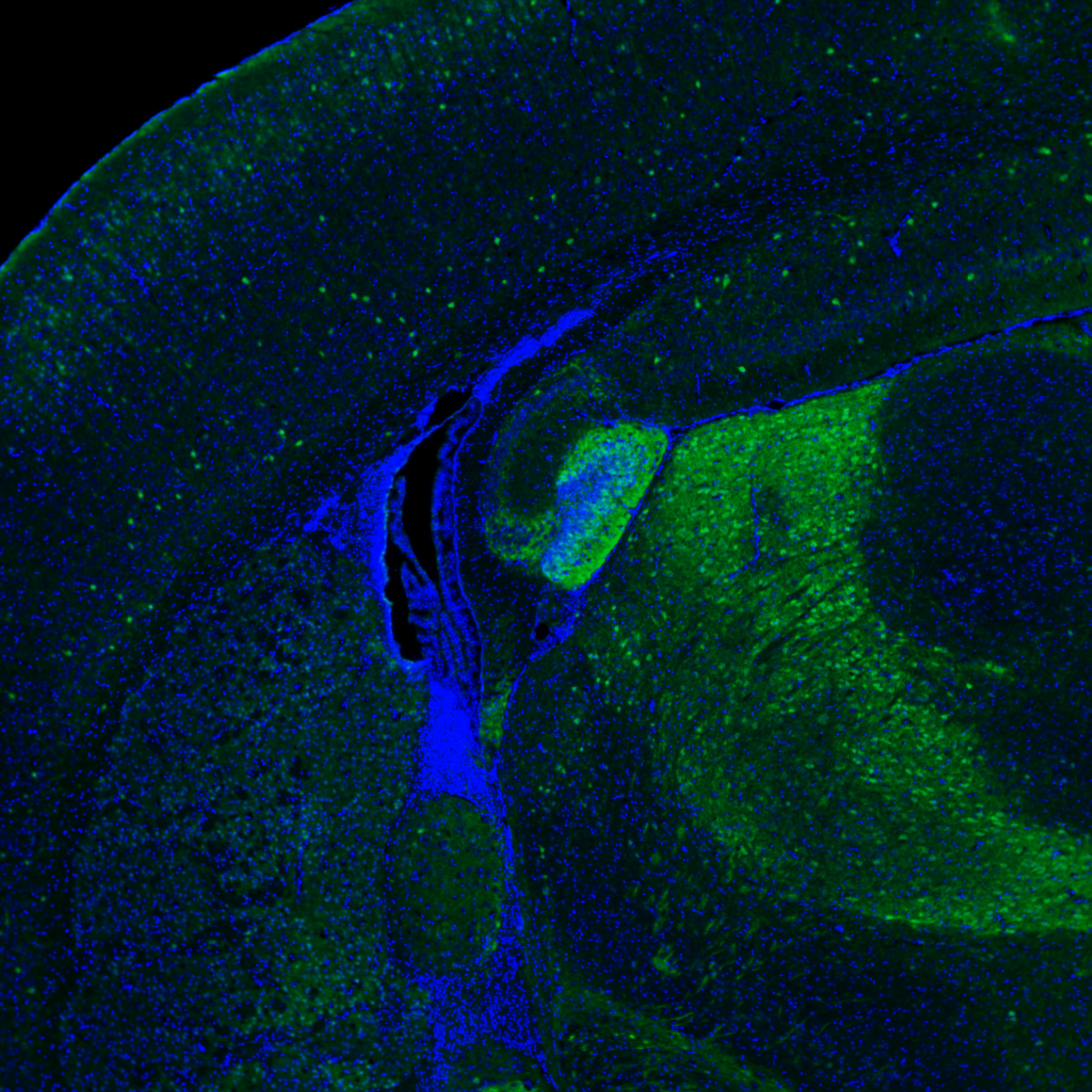

Supplement: Figure 4—source data 2. [file elife-86940-fig4-data2.zip › Figure 4-source data 2/F449-1-DKO-RX FF ff-P18-CB-125#-4-5X-left dHPC-Image Export-26_G+D.tif]

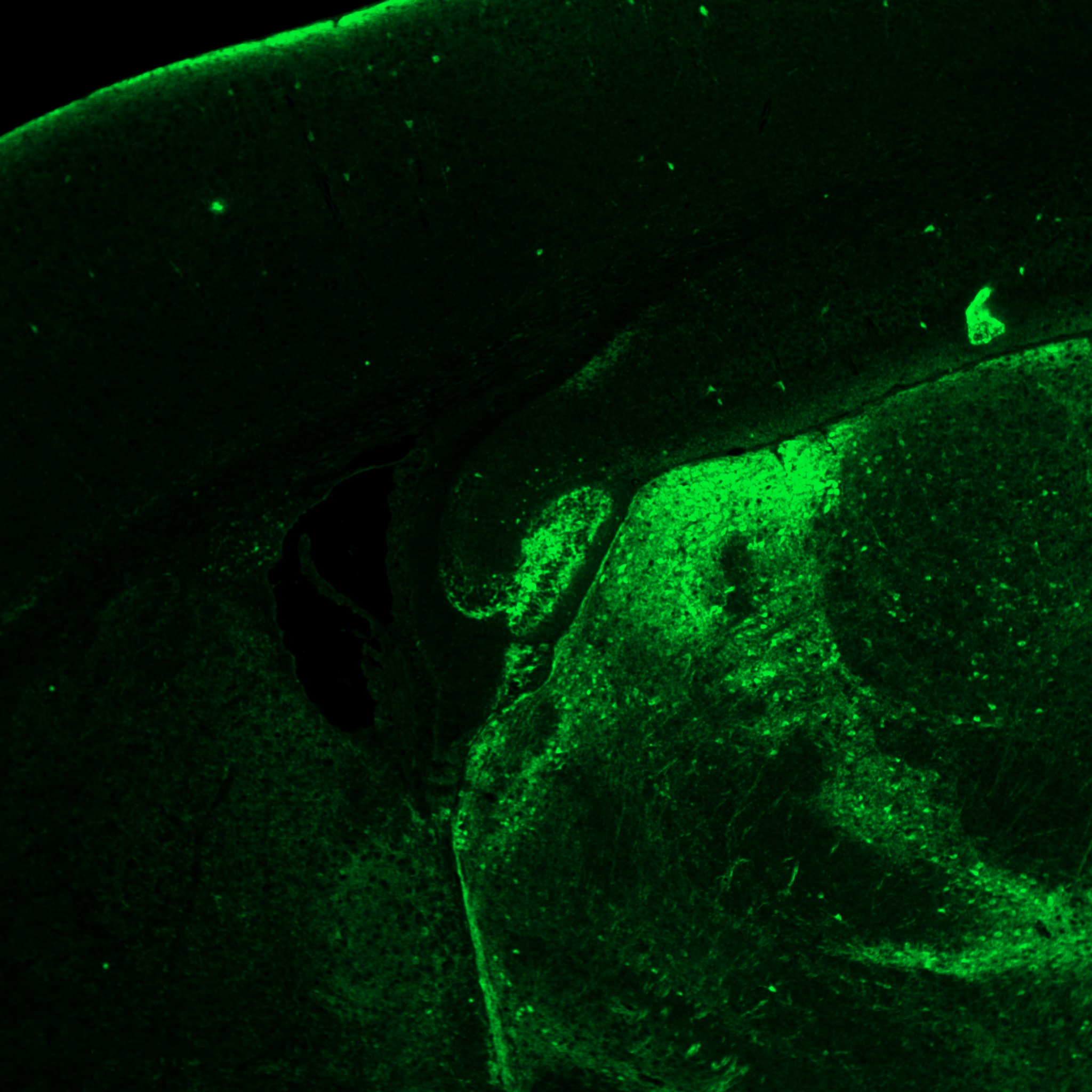

Supplement: Figure 4—source data 2. [file elife-86940-fig4-data2.zip › Figure 4-source data 2/F449-1-DKO-RX FF ff-P18-CR-125#-2-5X-left dHPC-Image Export-04_AF488.tif]

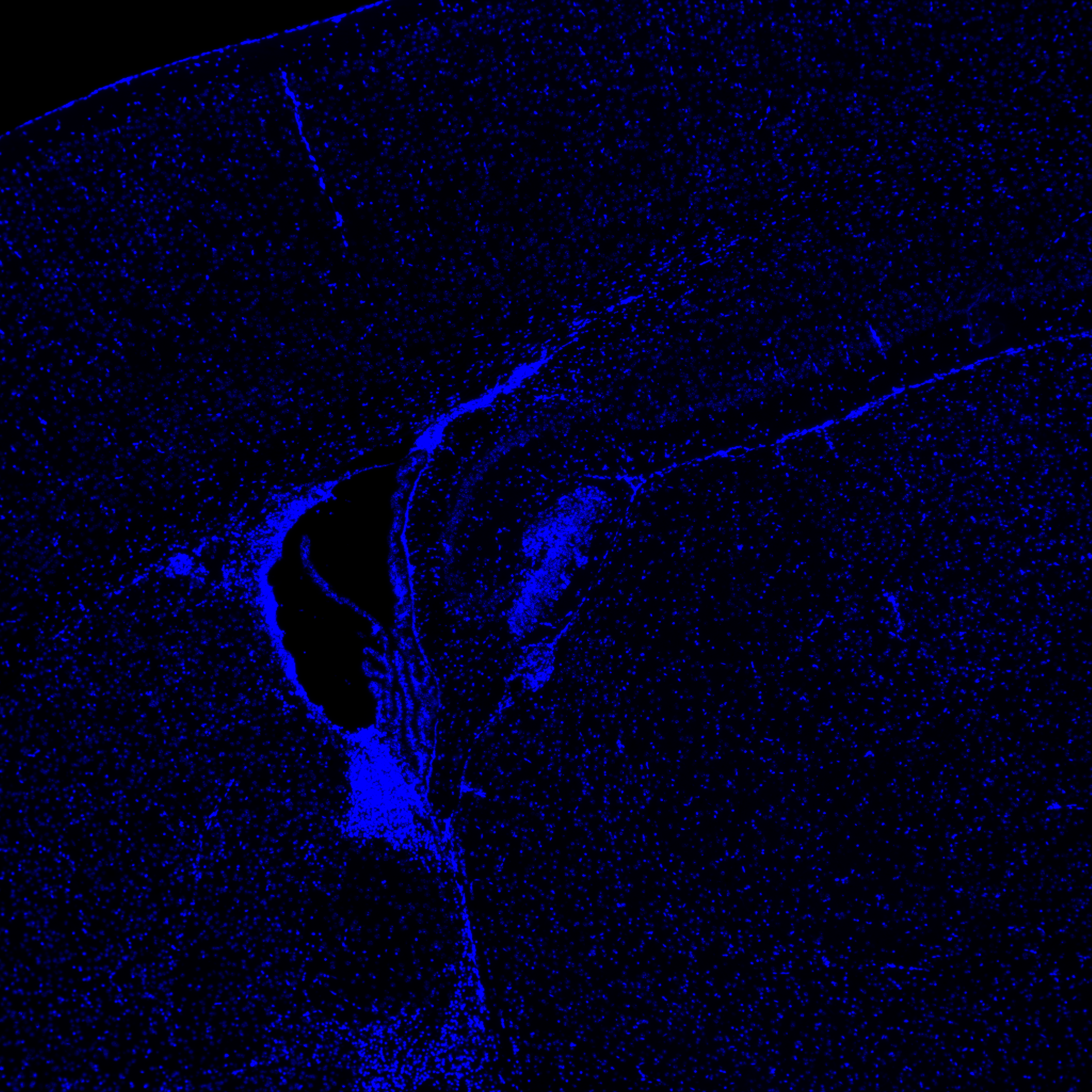

Supplement: Figure 4—source data 2. [file elife-86940-fig4-data2.zip › Figure 4-source data 2/F449-1-DKO-RX FF ff-P18-CR-125#-2-5X-left dHPC-Image Export-04_DAPI.tif]

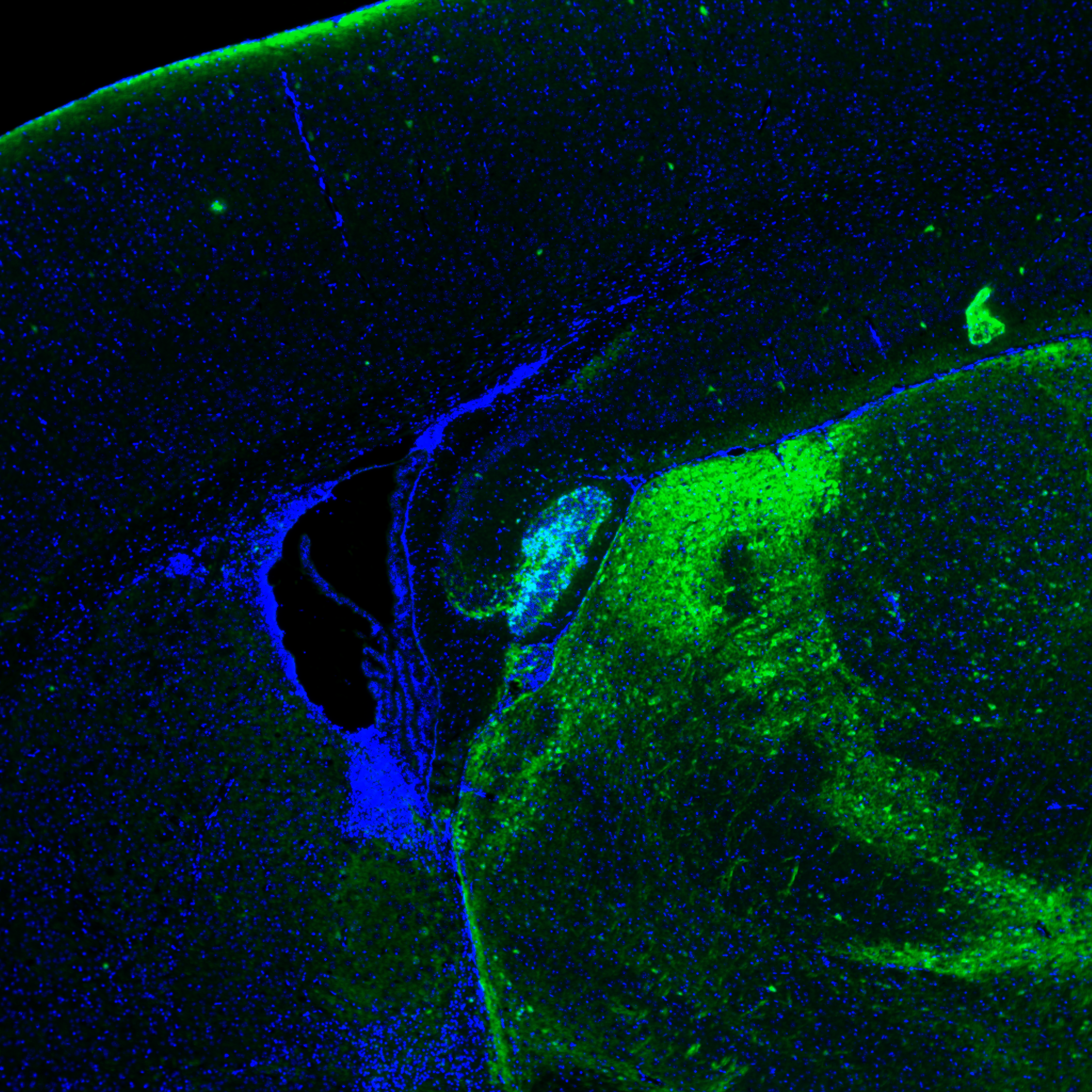

Supplement: Figure 4—source data 2. [file elife-86940-fig4-data2.zip › Figure 4-source data 2/F449-1-DKO-RX FF ff-P18-CR-125#-2-5X-left dHPC-Image Export-04_G+D.tif]

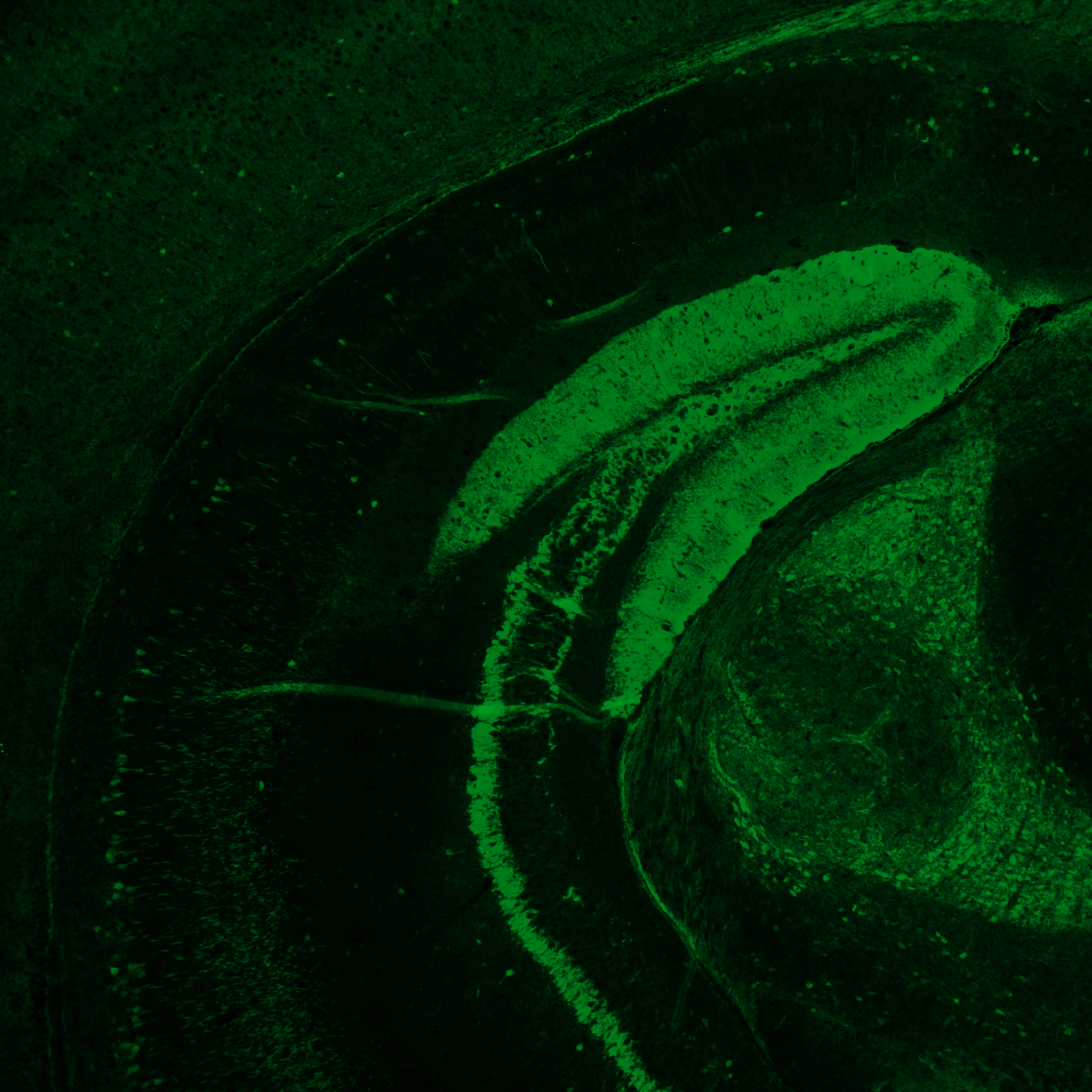

Supplement: Figure 4—source data 2. [file elife-86940-fig4-data2.zip › Figure 4-source data 2/F449-3-CON-F+ ff-P18-CB-151#-4-5X-left dHPC-Image Export-19_AF488.tif]

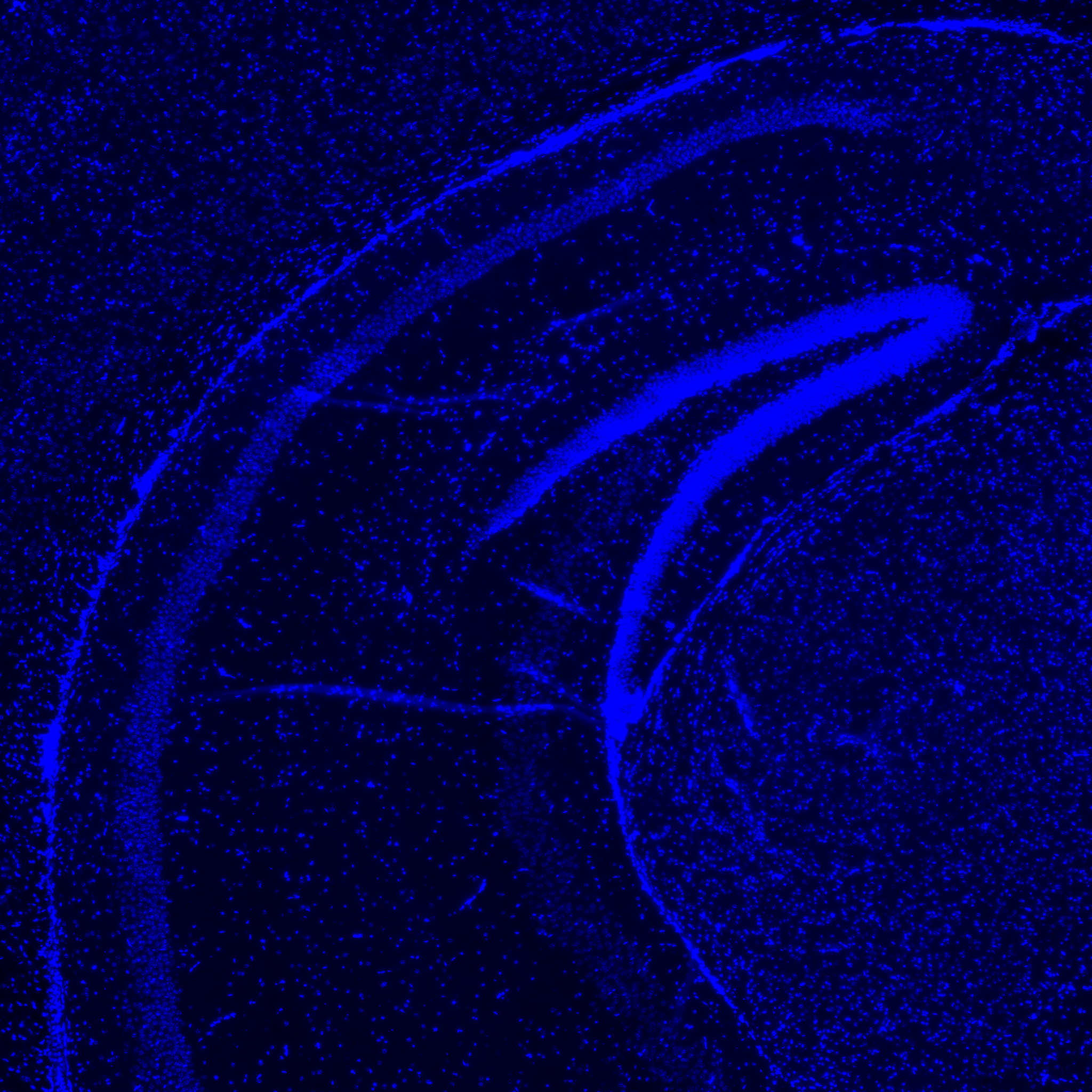

Supplement: Figure 4—source data 2. [file elife-86940-fig4-data2.zip › Figure 4-source data 2/F449-3-CON-F+ ff-P18-CB-151#-4-5X-left dHPC-Image Export-19_DAPI.tif]

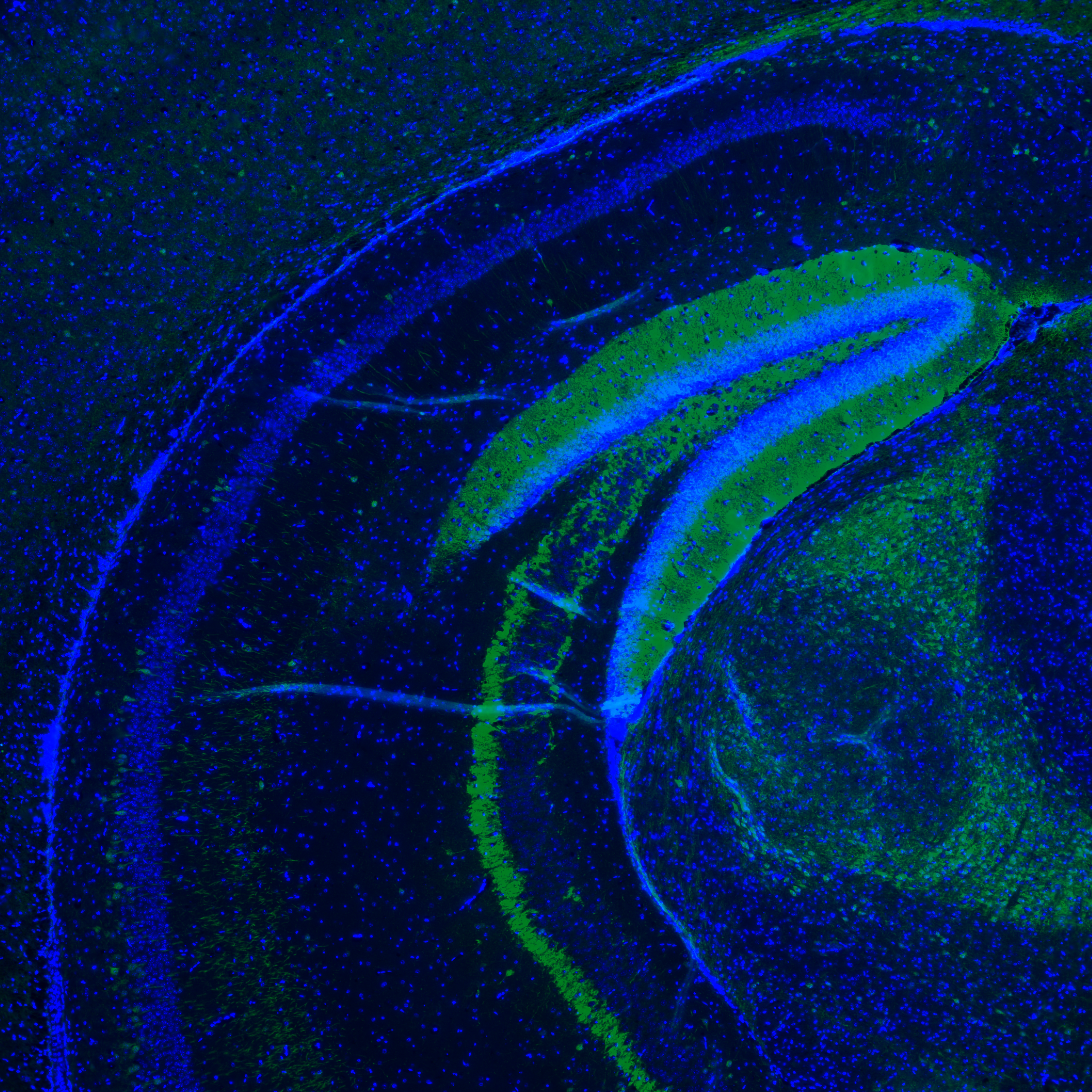

Supplement: Figure 4—source data 2. [file elife-86940-fig4-data2.zip › Figure 4-source data 2/F449-3-CON-F+ ff-P18-CB-151#-4-5X-left dHPC-Image Export-19_G+D.tif]

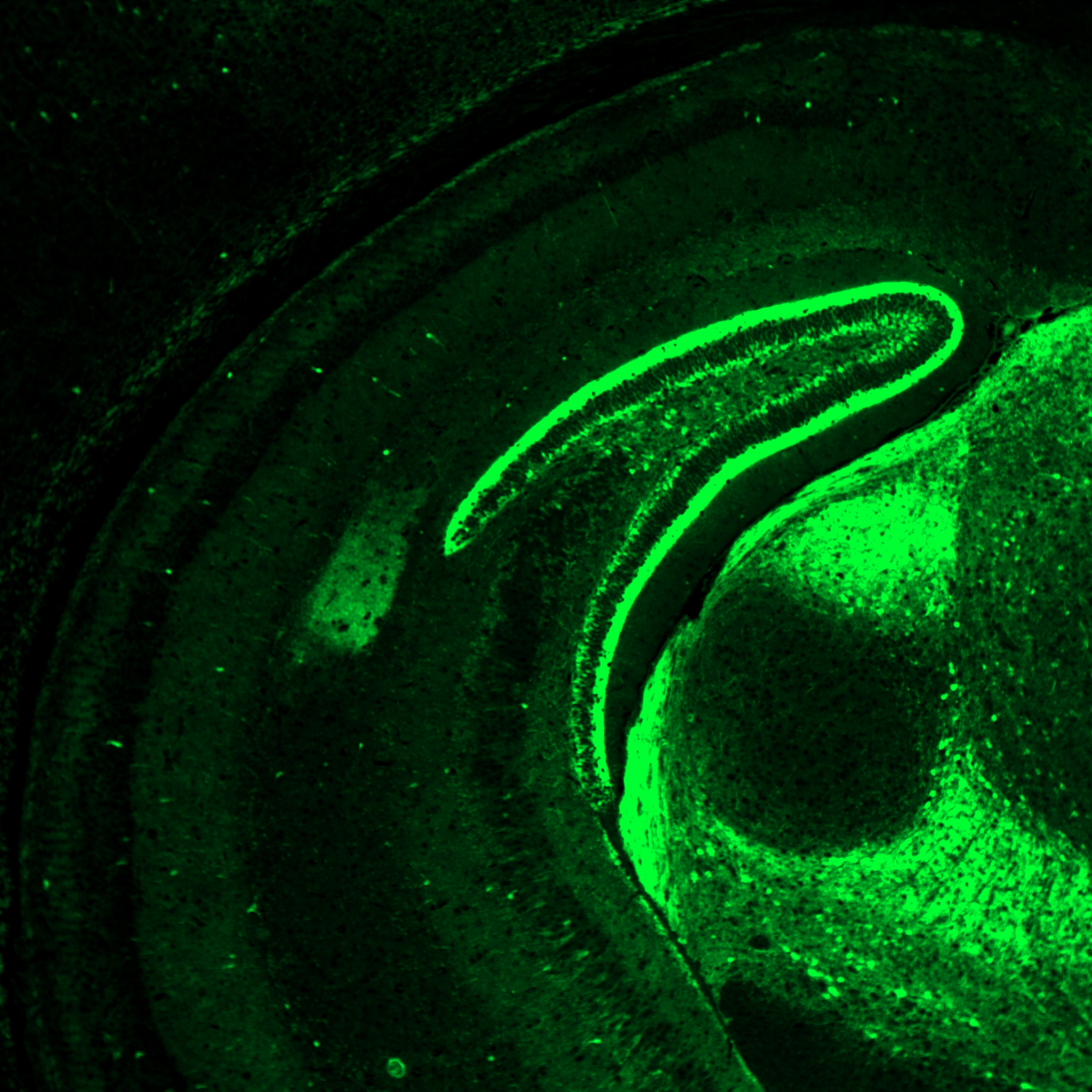

Supplement: Figure 4—source data 2. [file elife-86940-fig4-data2.zip › Figure 4-source data 2/F449-3-CON-F+ ff-P18-CR-151#-2-5X-left dHPC-Image Export-04_AF488.tif]

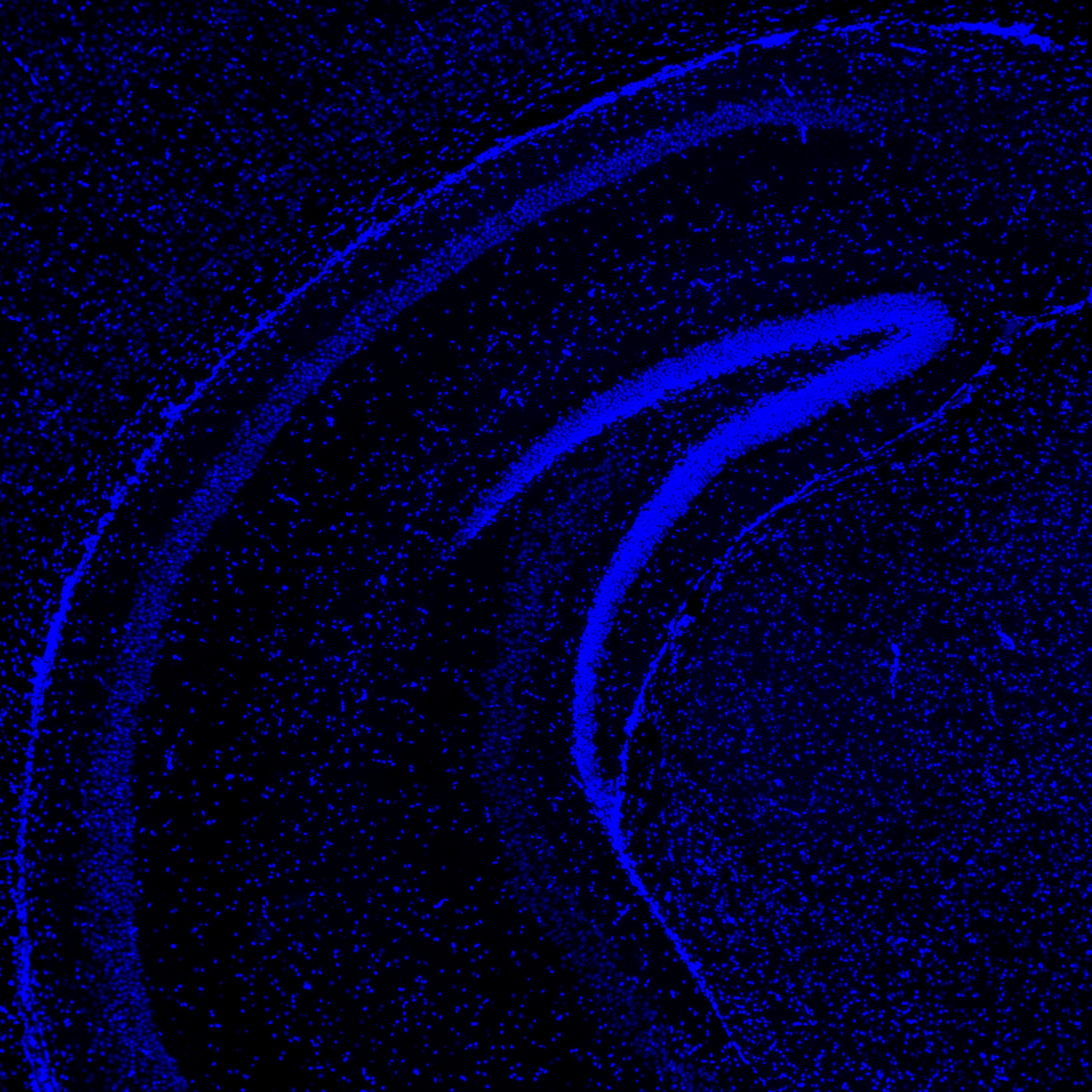

Supplement: Figure 4—source data 2. [file elife-86940-fig4-data2.zip › Figure 4-source data 2/F449-3-CON-F+ ff-P18-CR-151#-2-5X-left dHPC-Image Export-04_DAPI.tif]

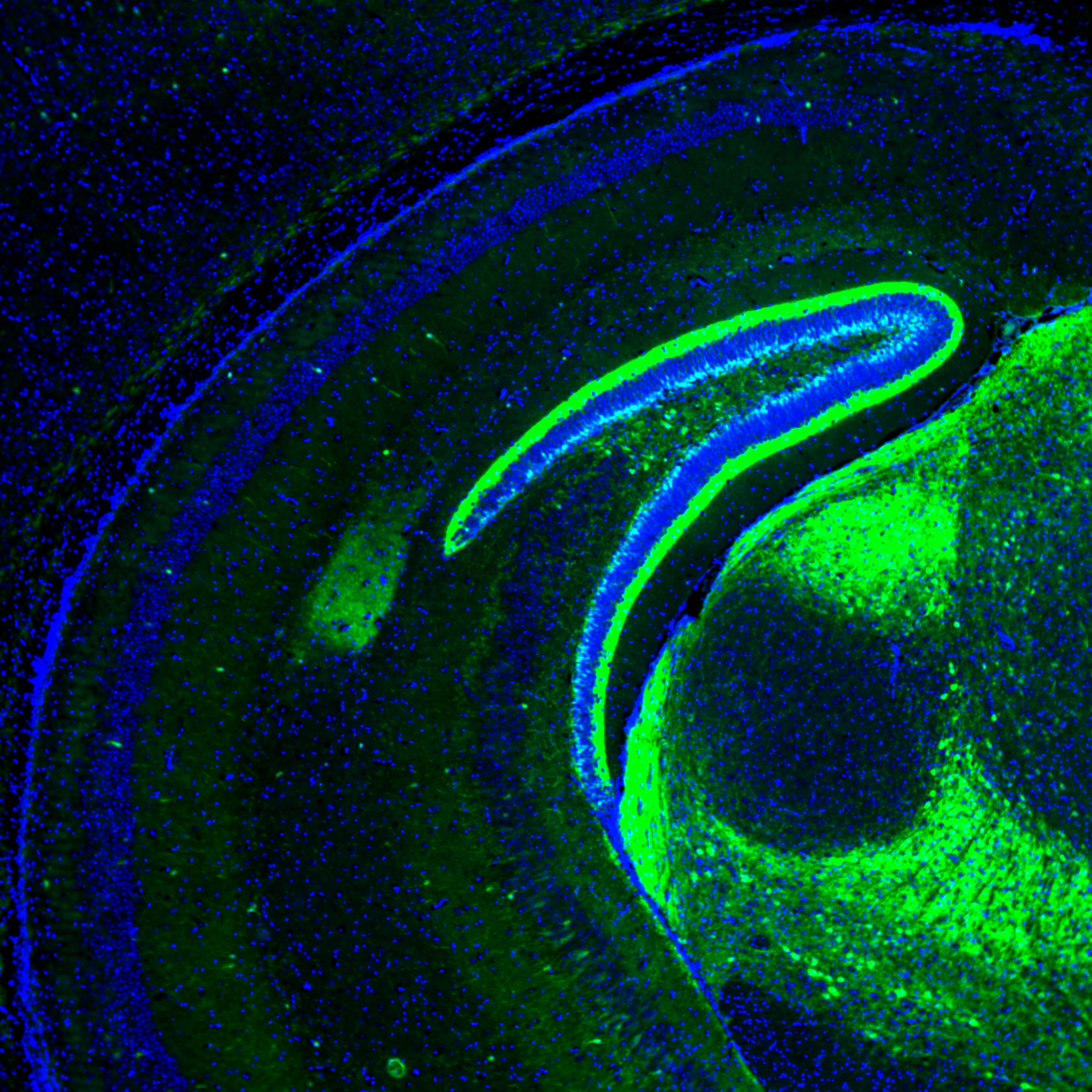

Supplement: Figure 4—source data 2. [file elife-86940-fig4-data2.zip › Figure 4-source data 2/F449-3-CON-F+ ff-P18-CR-151#-2-5X-left dHPC-Image Export-04_G+D.tif]

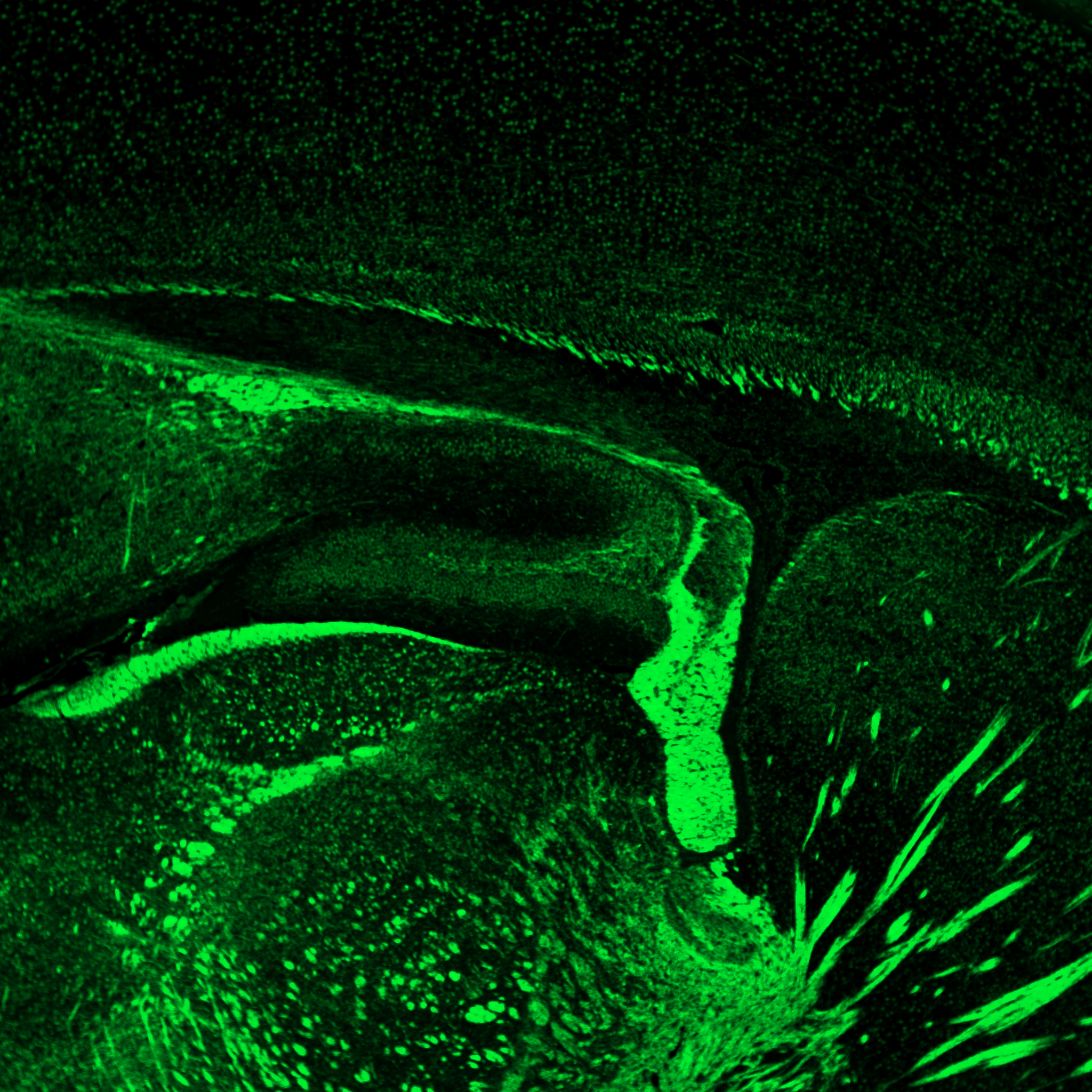

Supplement: Figure 4—source data 2. [file elife-86940-fig4-data2.zip › Figure 4-source data 2/F3084-3-CKO-1M-RX CI ff-5X-SAGITAL-SMI312-3-dHPC-Image Export-02_AF488.tif]

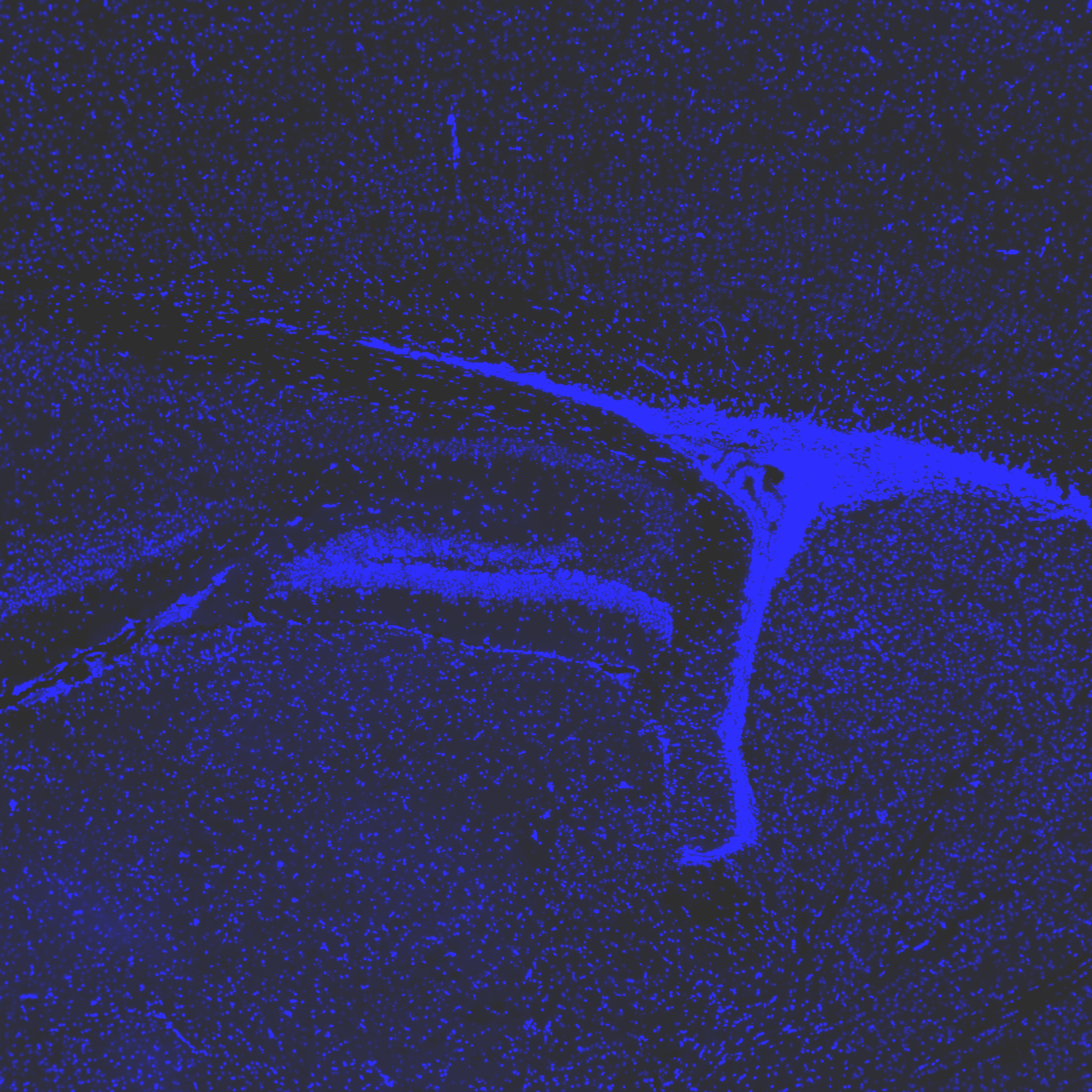

Supplement: Figure 4—source data 2. [file elife-86940-fig4-data2.zip › Figure 4-source data 2/F3084-3-CKO-1M-RX CI ff-5X-SAGITAL-SMI312-3-dHPC-Image Export-02_DAPI.tif]

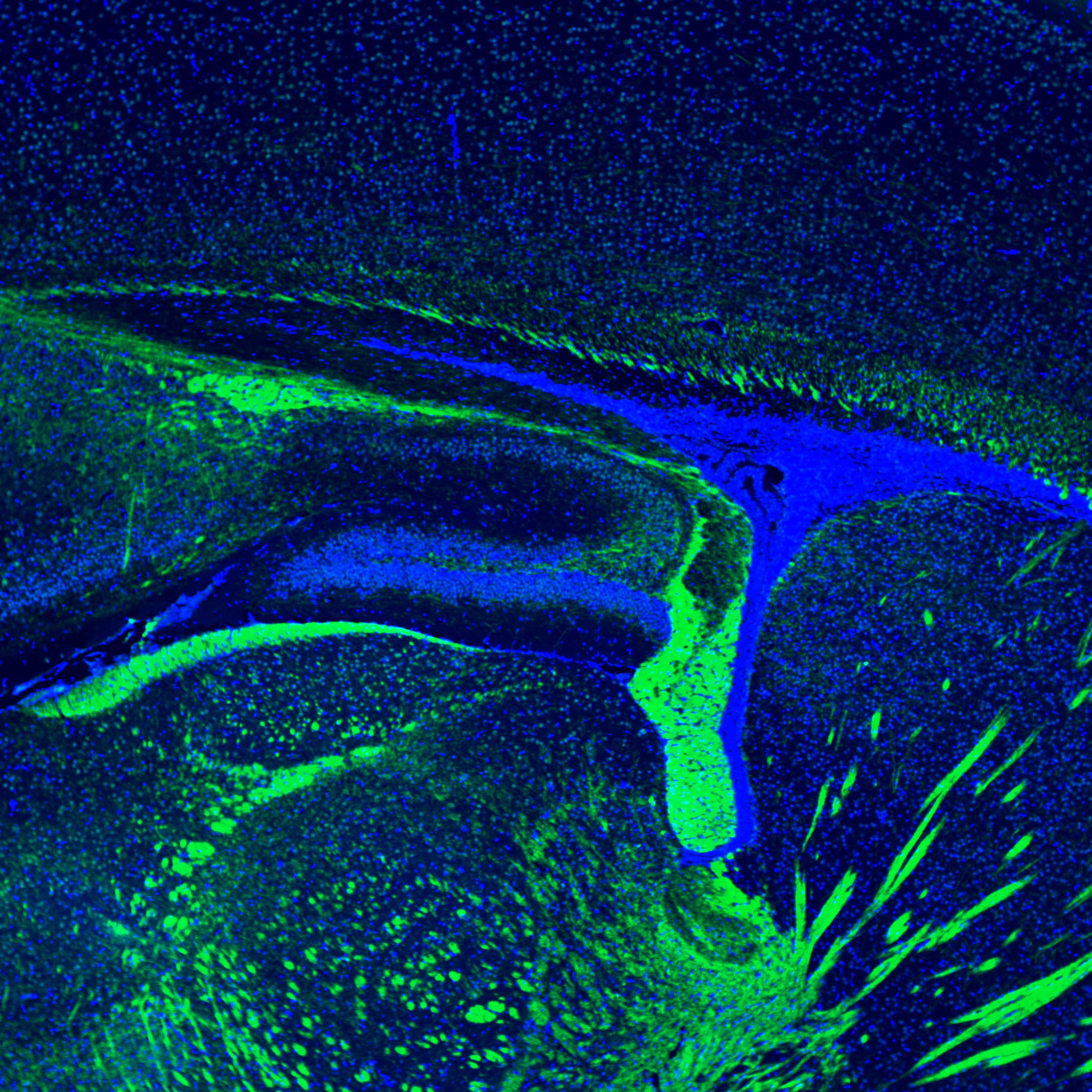

Supplement: Figure 4—source data 2. [file elife-86940-fig4-data2.zip › Figure 4-source data 2/F3084-3-CKO-1M-RX CI ff-5X-SAGITAL-SMI312-3-dHPC-Image Export-02_G+D.tif]

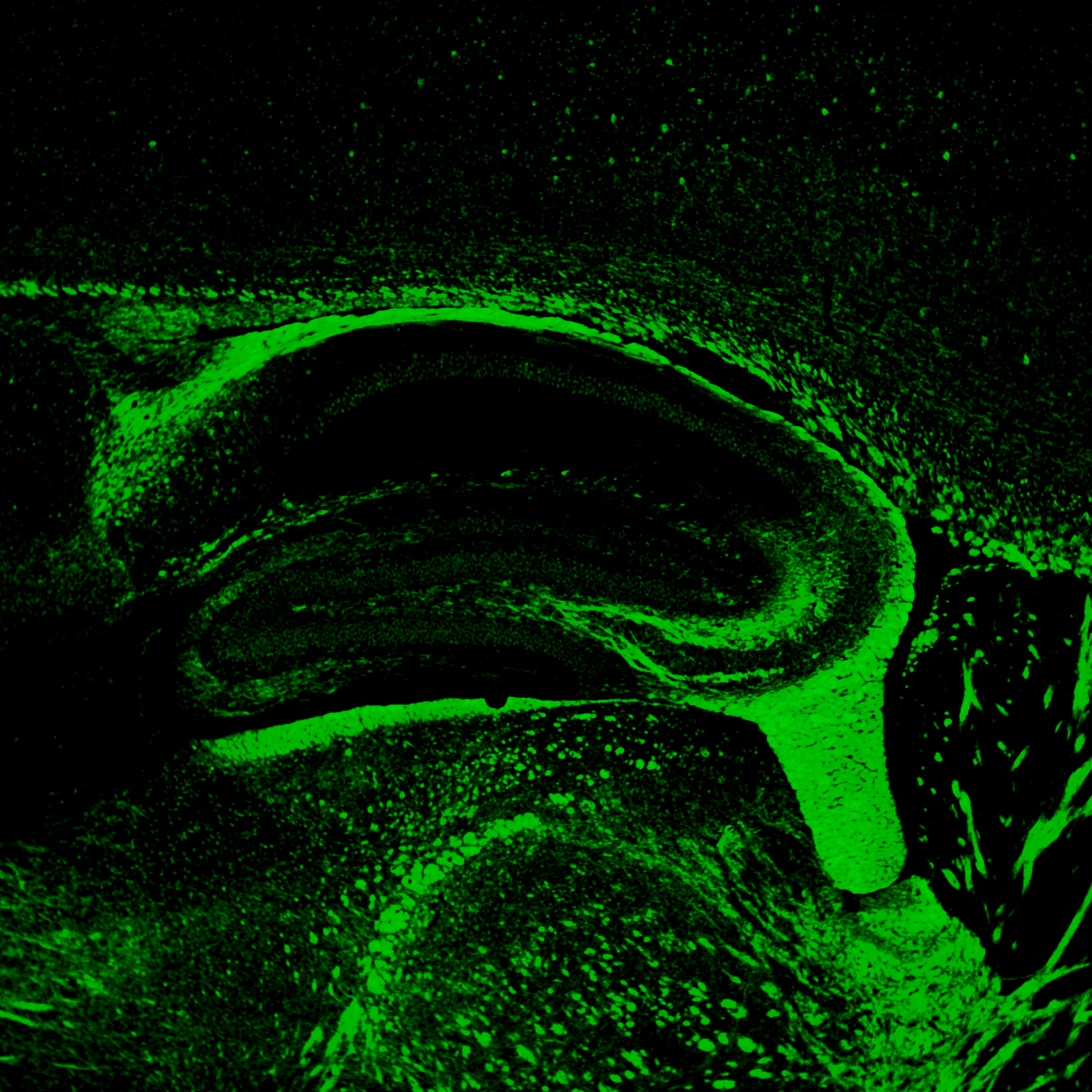

Supplement: Figure 4—source data 2. [file elife-86940-fig4-data2.zip › Figure 4-source data 2/F3094-2-CON-1M-RX CI f+-5X-SAGITAL-SMI312-3-dHPC-Image Export-02_AF488.tif]

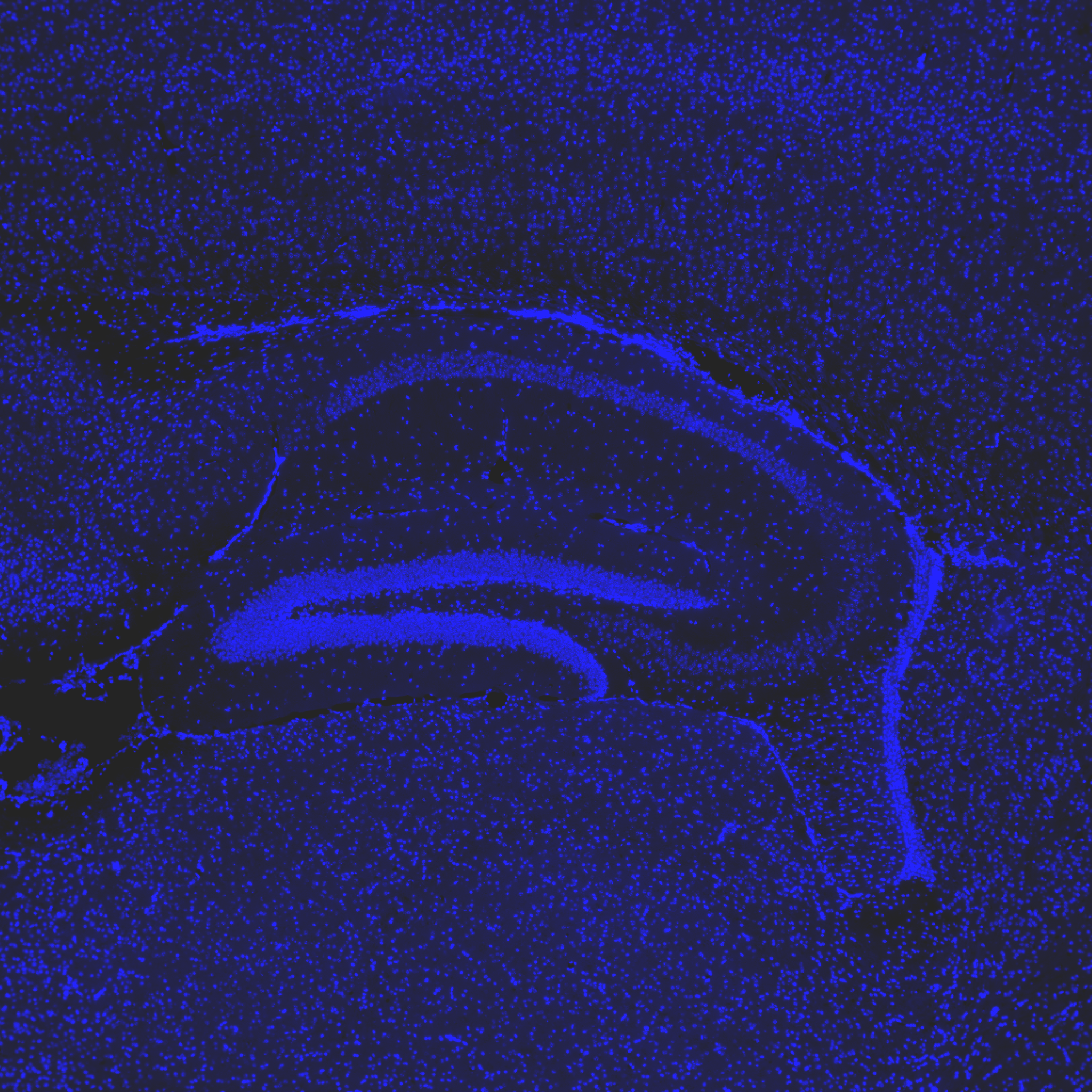

Supplement: Figure 4—source data 2. [file elife-86940-fig4-data2.zip › Figure 4-source data 2/F3094-2-CON-1M-RX CI f+-5X-SAGITAL-SMI312-3-dHPC-Image Export-02_DAPI.tif]

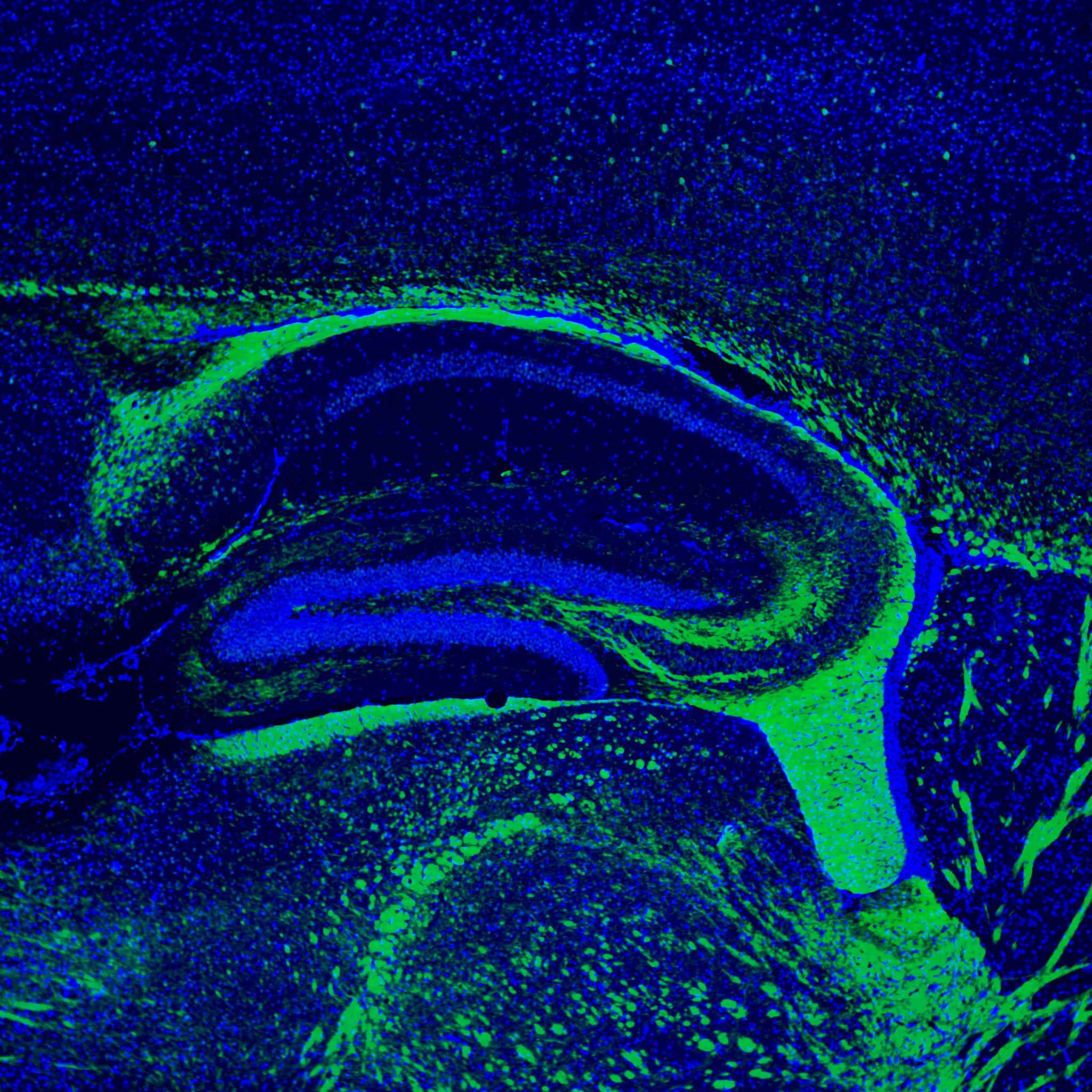

Supplement: Figure 4—source data 2. [file elife-86940-fig4-data2.zip › Figure 4-source data 2/F3094-2-CON-1M-RX CI f+-5X-SAGITAL-SMI312-3-dHPC-Image Export-02_G+D.tif]

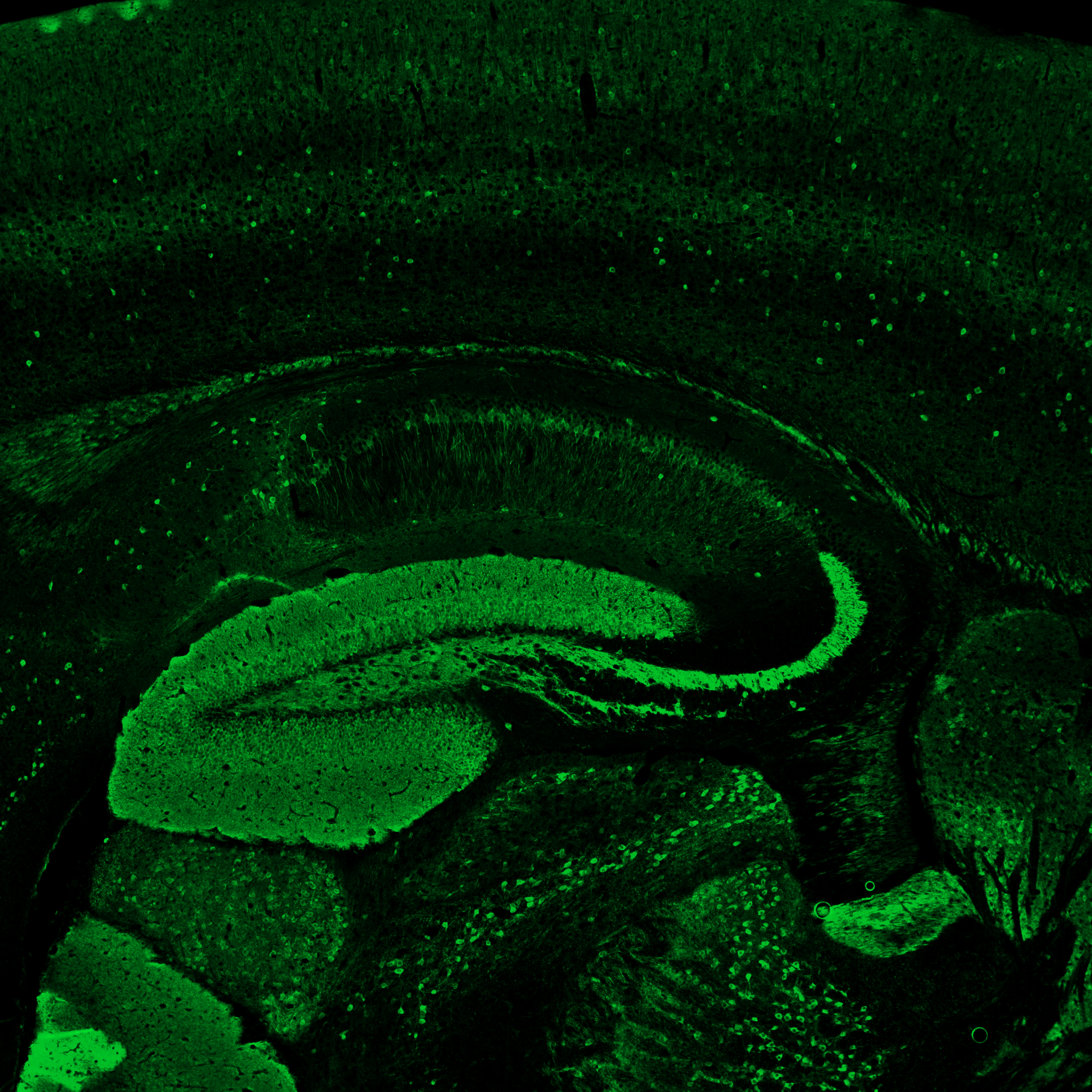

Supplement: Figure 4—source data 3. [file elife-86940-fig4-data3.zip › Figure 4-source data 3/F3094-2-CON-RX CI F+-1M-SAGITAL-CB-152#-4-5X-dHPC-Image Export-17_AF488.tif]

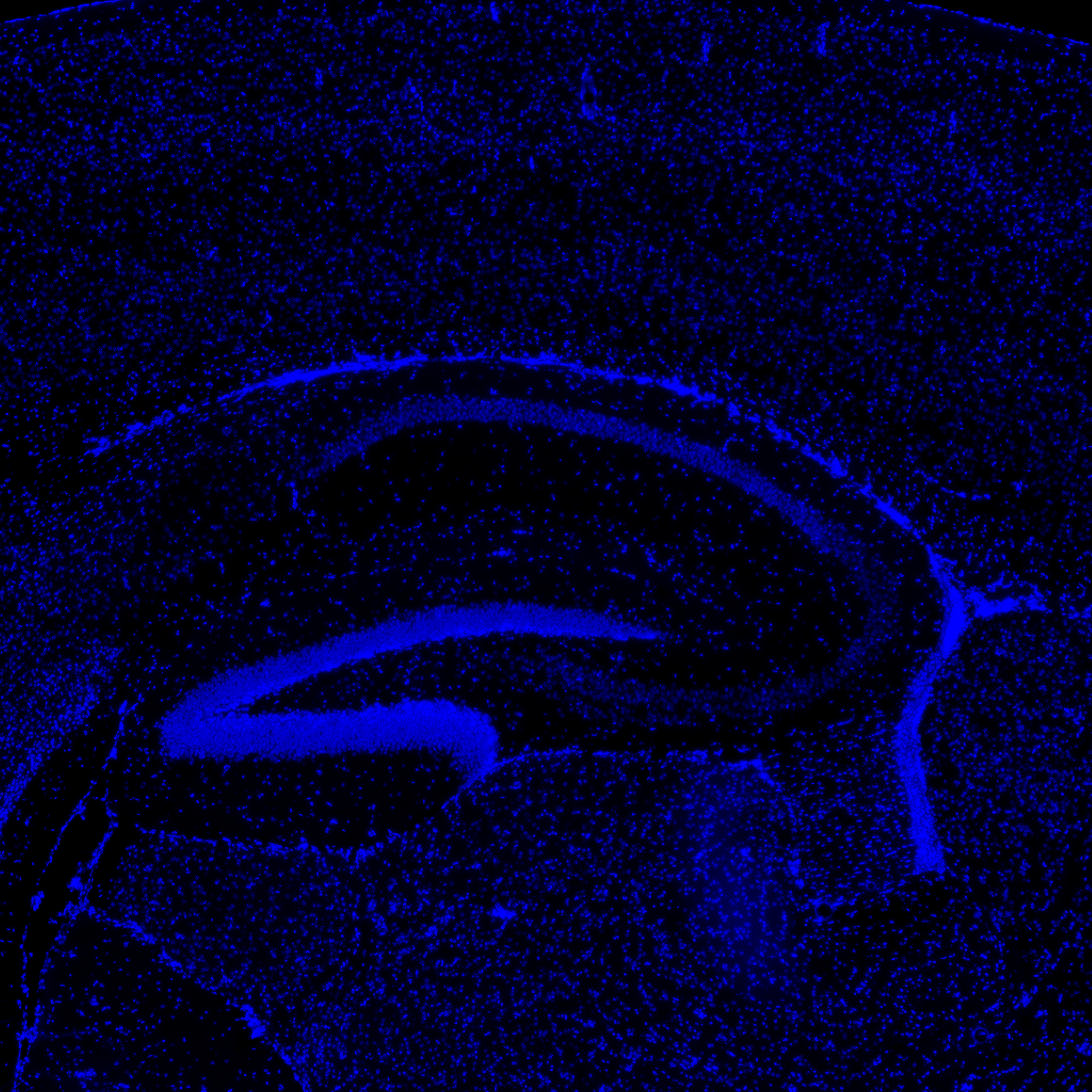

Supplement: Figure 4—source data 3. [file elife-86940-fig4-data3.zip › Figure 4-source data 3/F3094-2-CON-RX CI F+-1M-SAGITAL-CB-152#-4-5X-dHPC-Image Export-17_DAPI.tif]

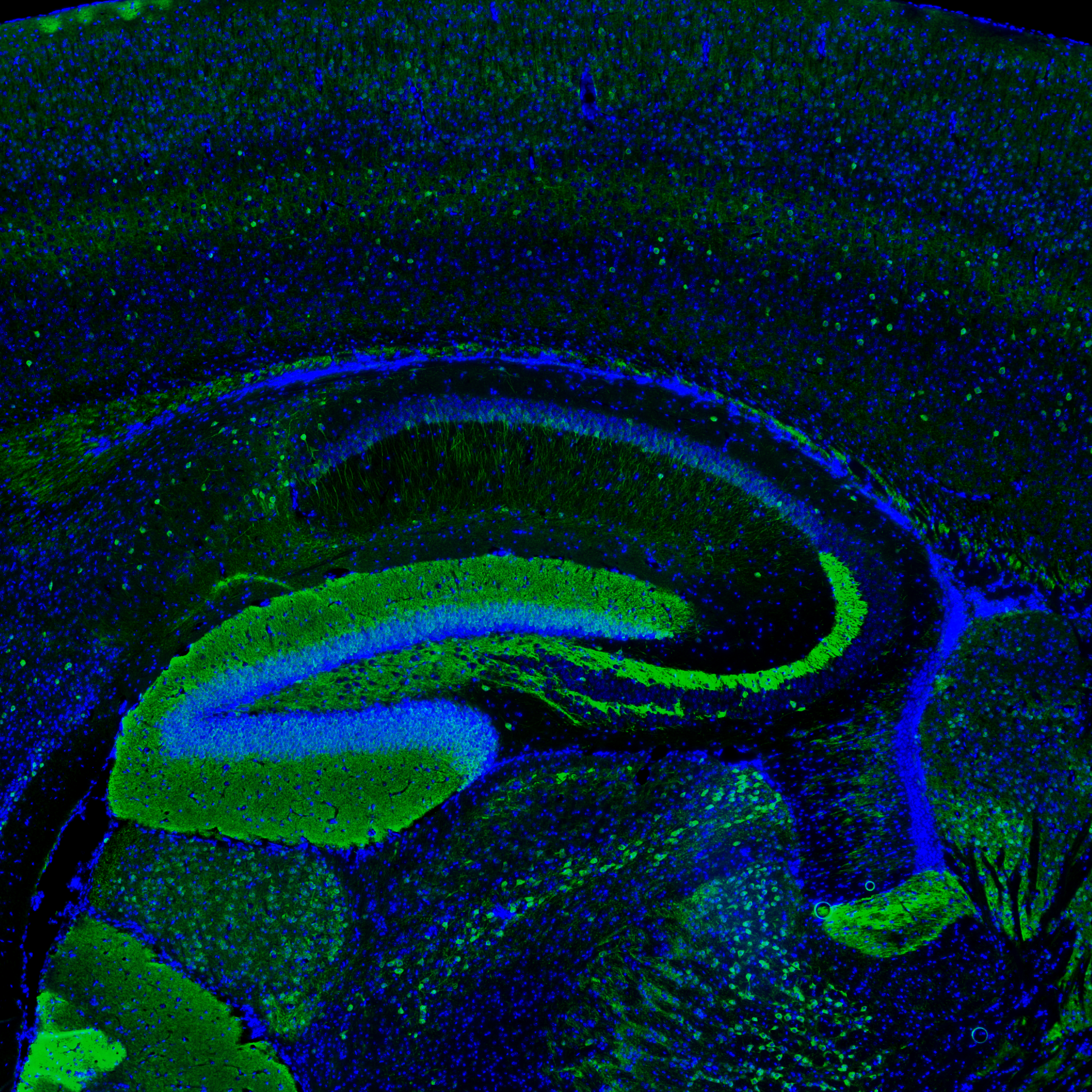

Supplement: Figure 4—source data 3. [file elife-86940-fig4-data3.zip › Figure 4-source data 3/F3094-2-CON-RX CI F+-1M-SAGITAL-CB-152#-4-5X-dHPC-Image Export-17_G+D.tif]

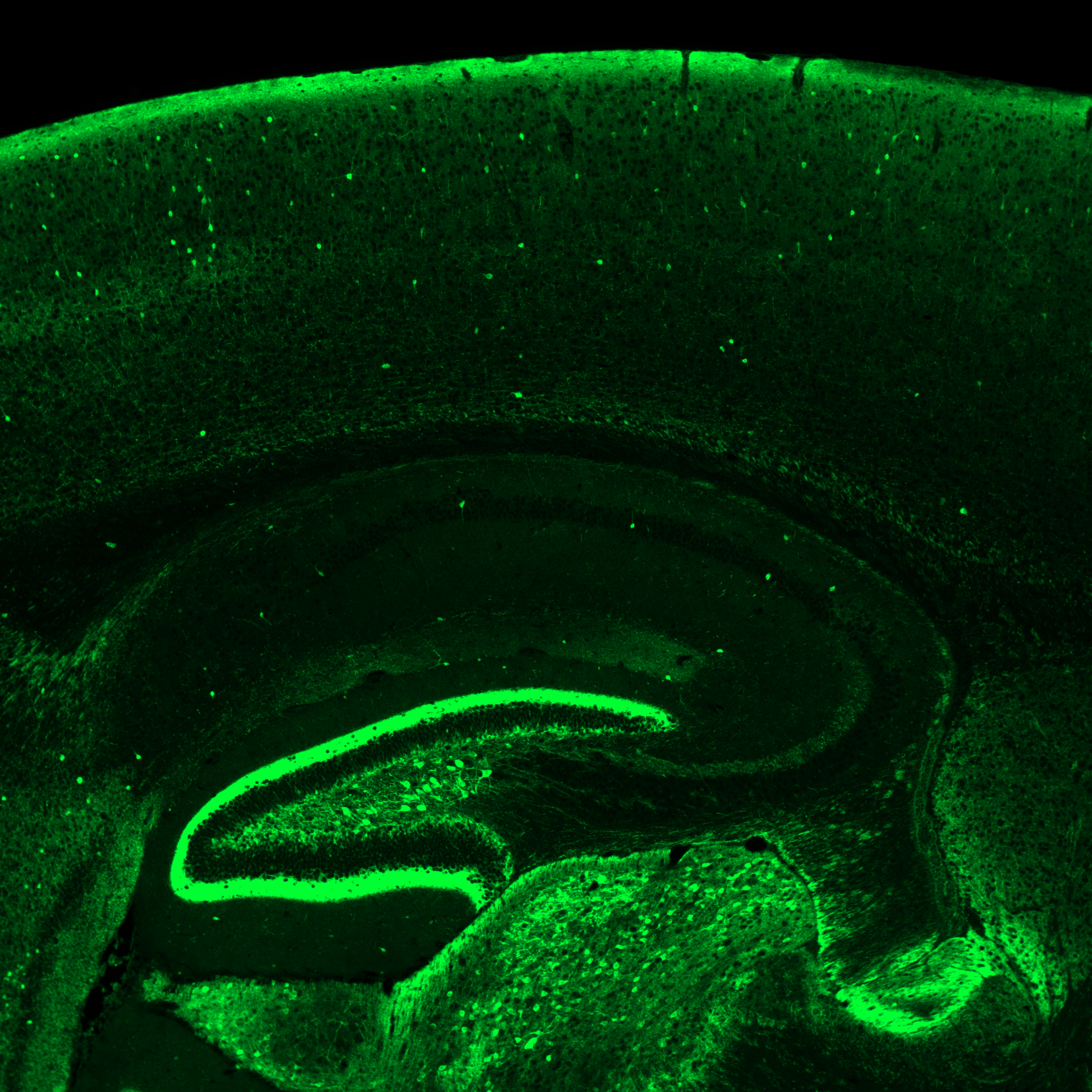

Supplement: Figure 4—source data 3. [file elife-86940-fig4-data3.zip › Figure 4-source data 3/F3094-2-CON-RX CI F+-1M-SAGITAL-CR-152#-1-5X-dHPC-Image Export-03_AF488.tif]

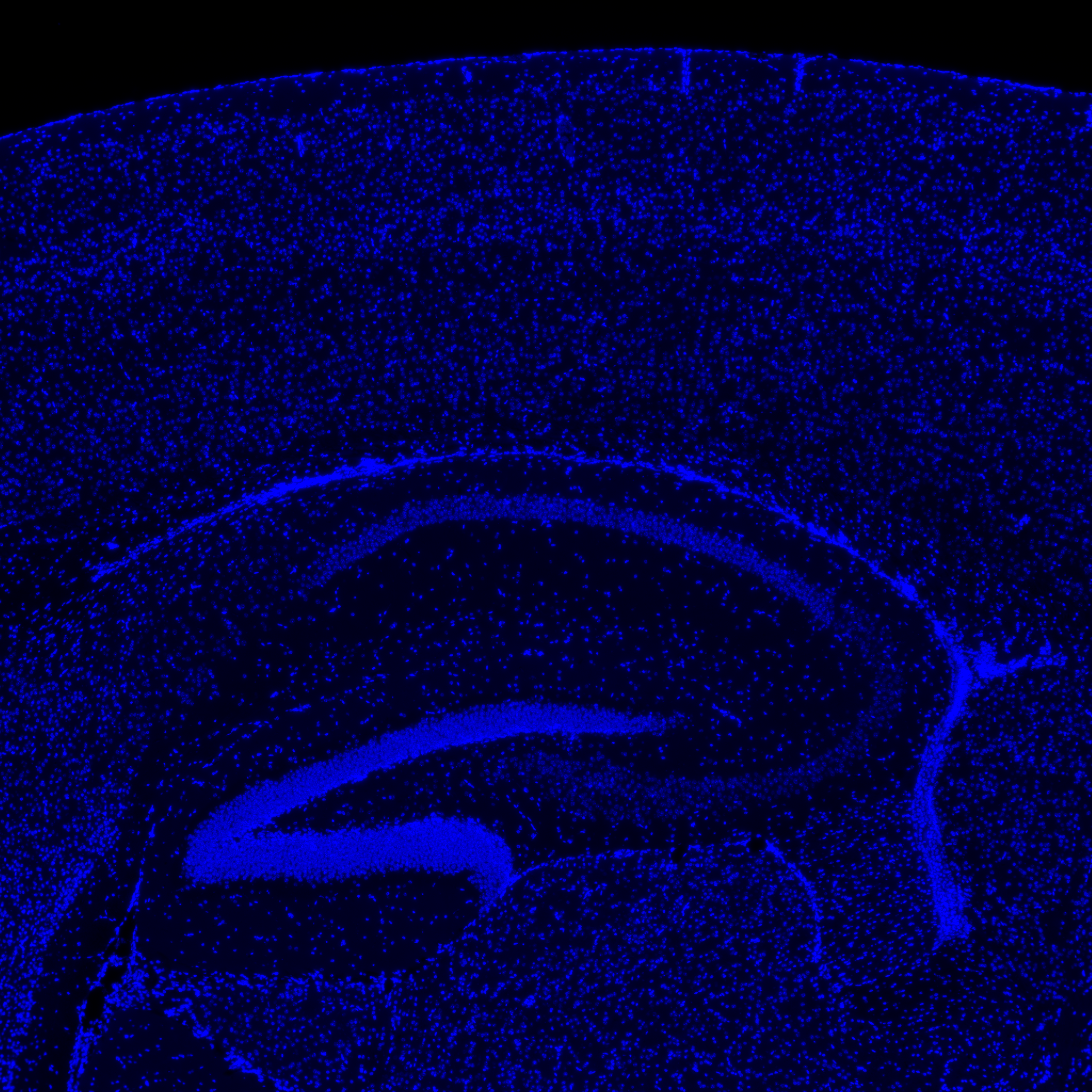

Supplement: Figure 4—source data 3. [file elife-86940-fig4-data3.zip › Figure 4-source data 3/F3094-2-CON-RX CI F+-1M-SAGITAL-CR-152#-1-5X-dHPC-Image Export-03_DAPI.tif]

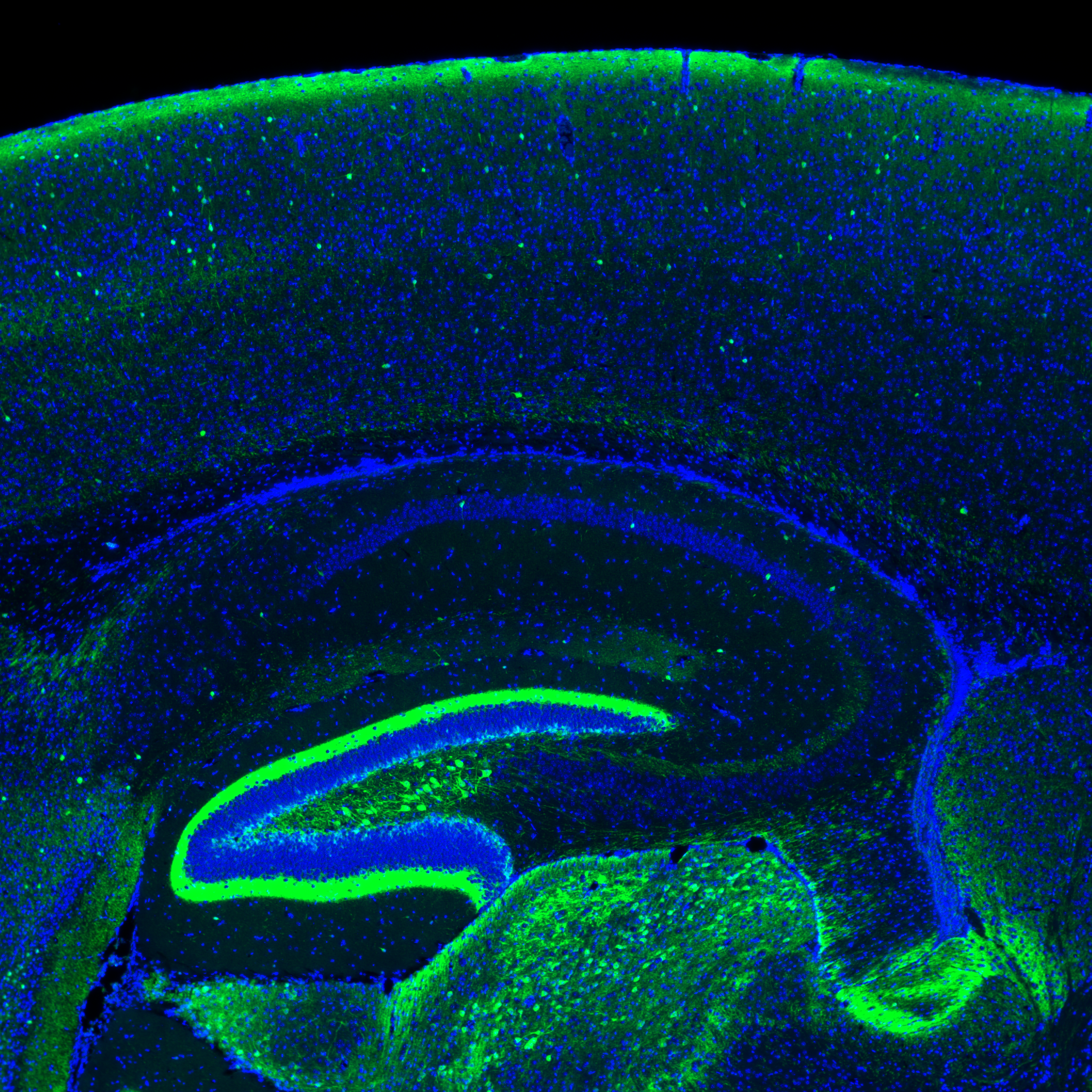

Supplement: Figure 4—source data 3. [file elife-86940-fig4-data3.zip › Figure 4-source data 3/F3094-2-CON-RX CI F+-1M-SAGITAL-CR-152#-1-5X-dHPC-Image Export-03_G+D.tif]

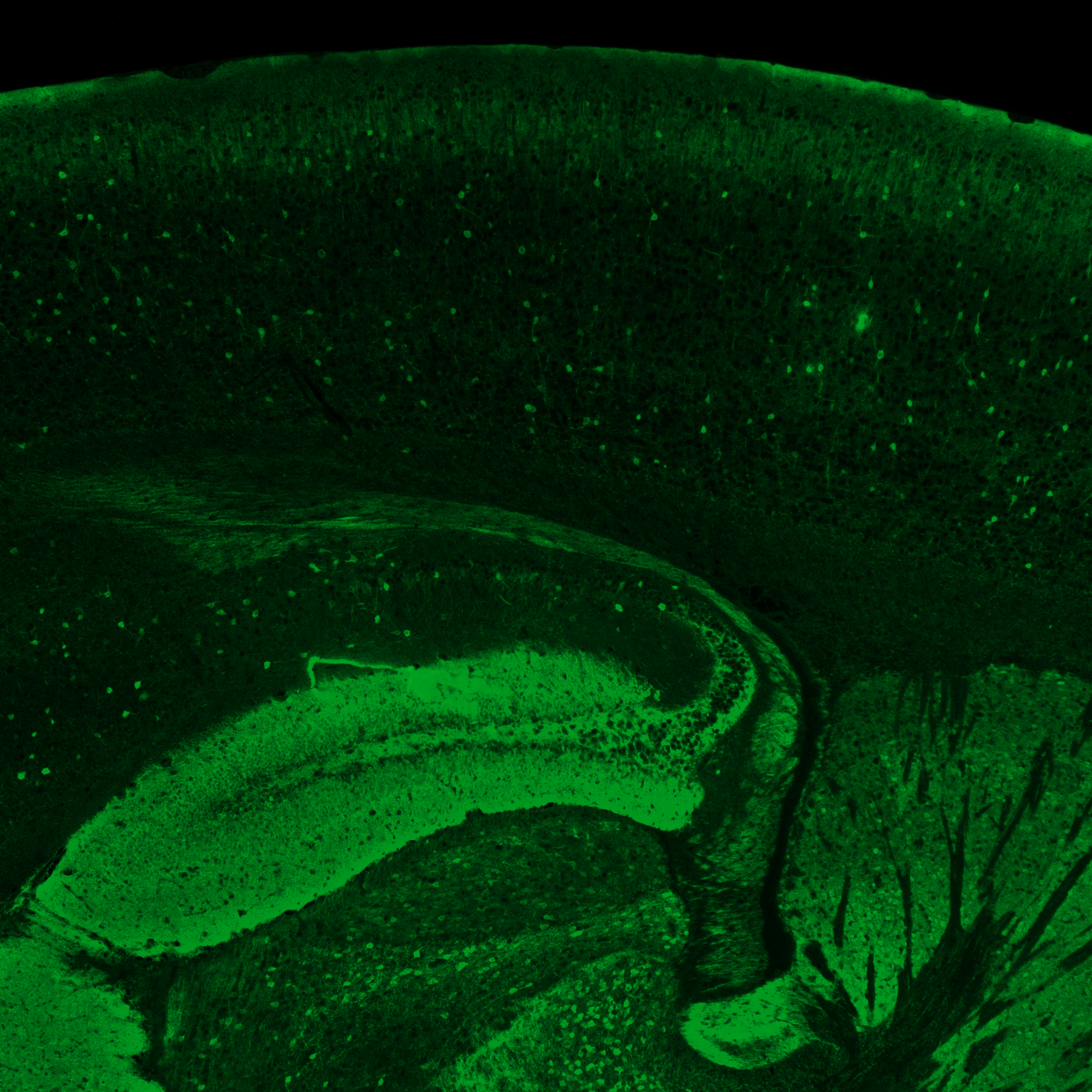

Supplement: Figure 4—source data 3. [file elife-86940-fig4-data3.zip › Figure 4-source data 3/F3094-3-CKO-RX CI FF-1M-SAGITAL-CB-24#-4-5X-dHPC-Image Export-15_AF488.tif]

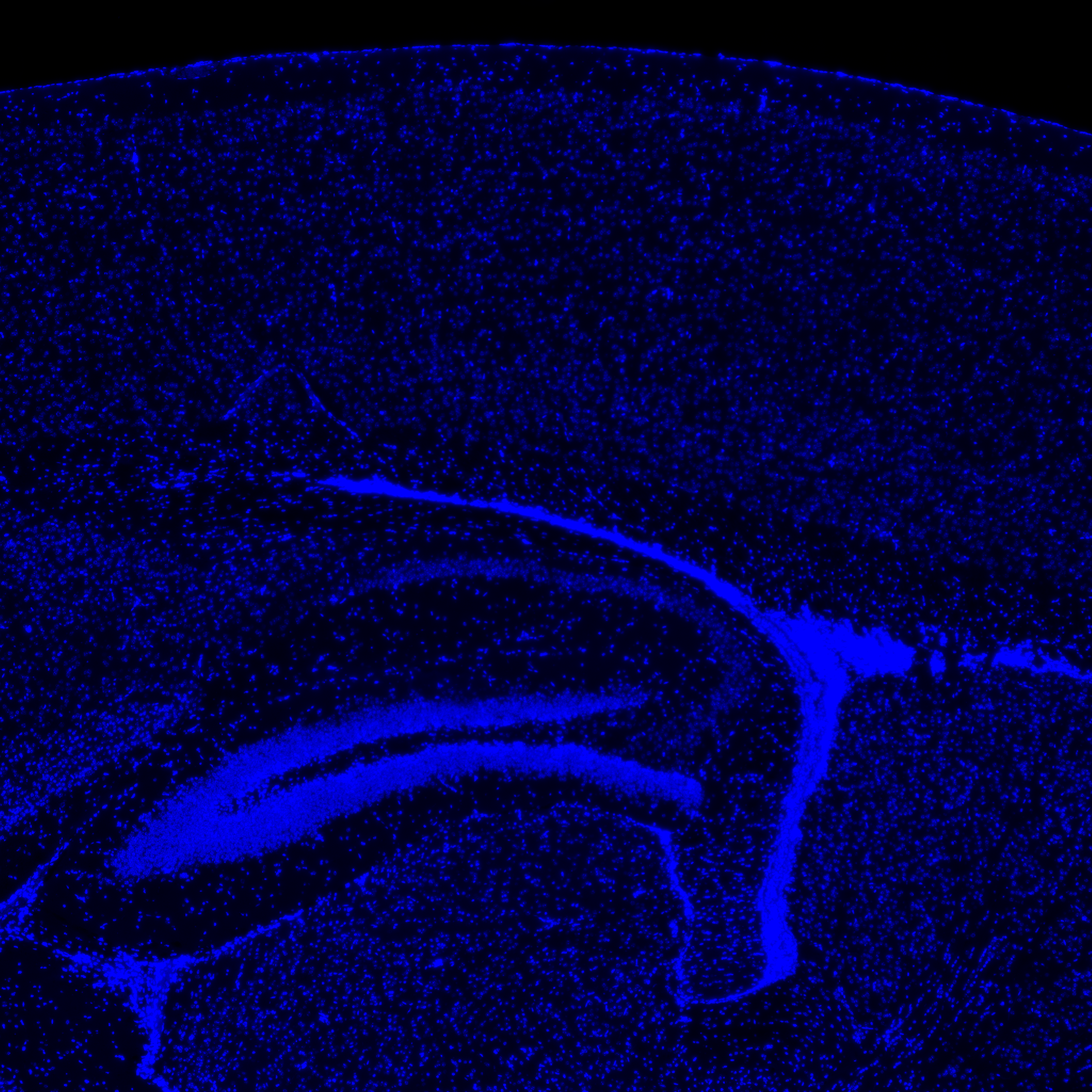

Supplement: Figure 4—source data 3. [file elife-86940-fig4-data3.zip › Figure 4-source data 3/F3094-3-CKO-RX CI FF-1M-SAGITAL-CB-24#-4-5X-dHPC-Image Export-15_DAPI.tif]

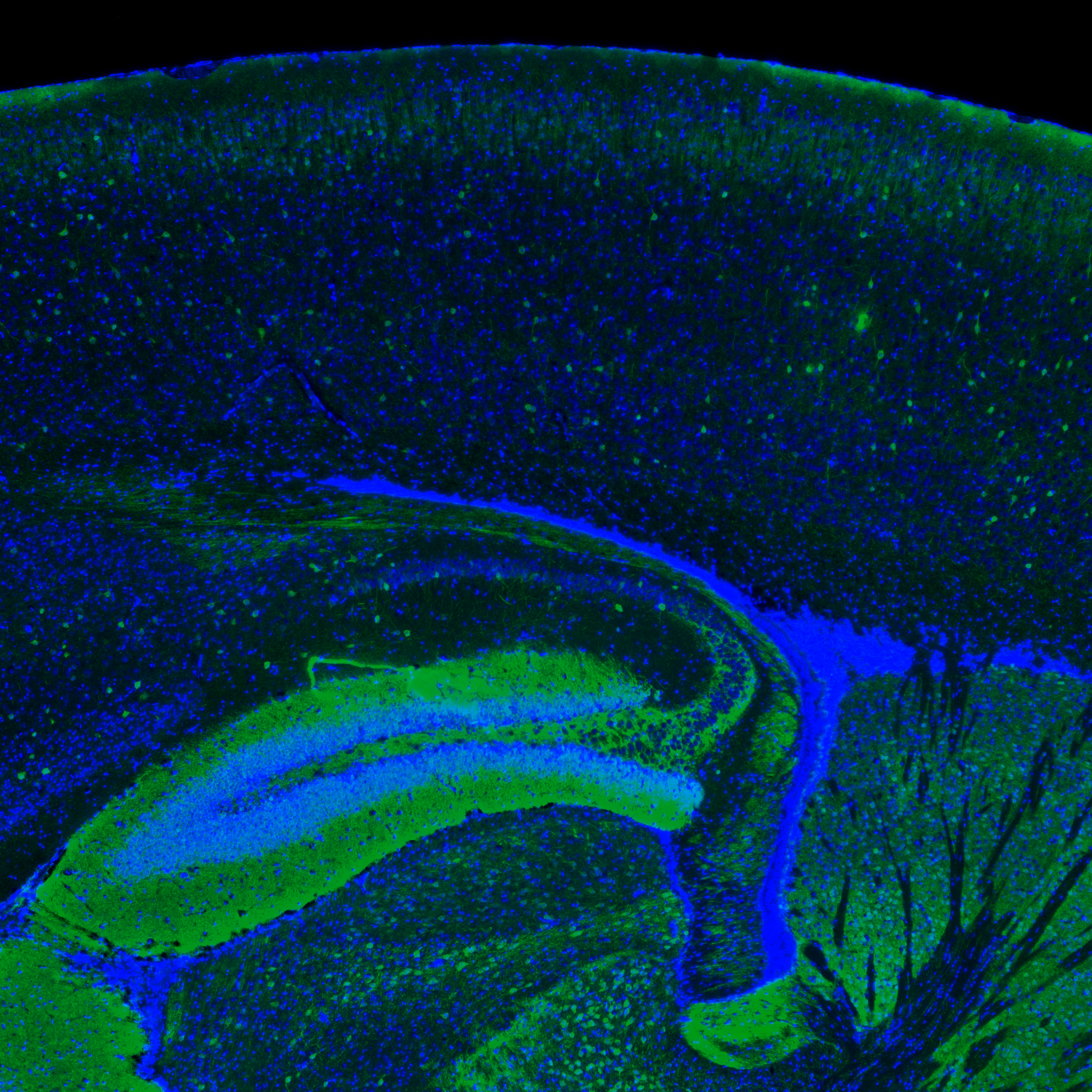

Supplement: Figure 4—source data 3. [file elife-86940-fig4-data3.zip › Figure 4-source data 3/F3094-3-CKO-RX CI FF-1M-SAGITAL-CB-24#-4-5X-dHPC-Image Export-15_G+D.tif]

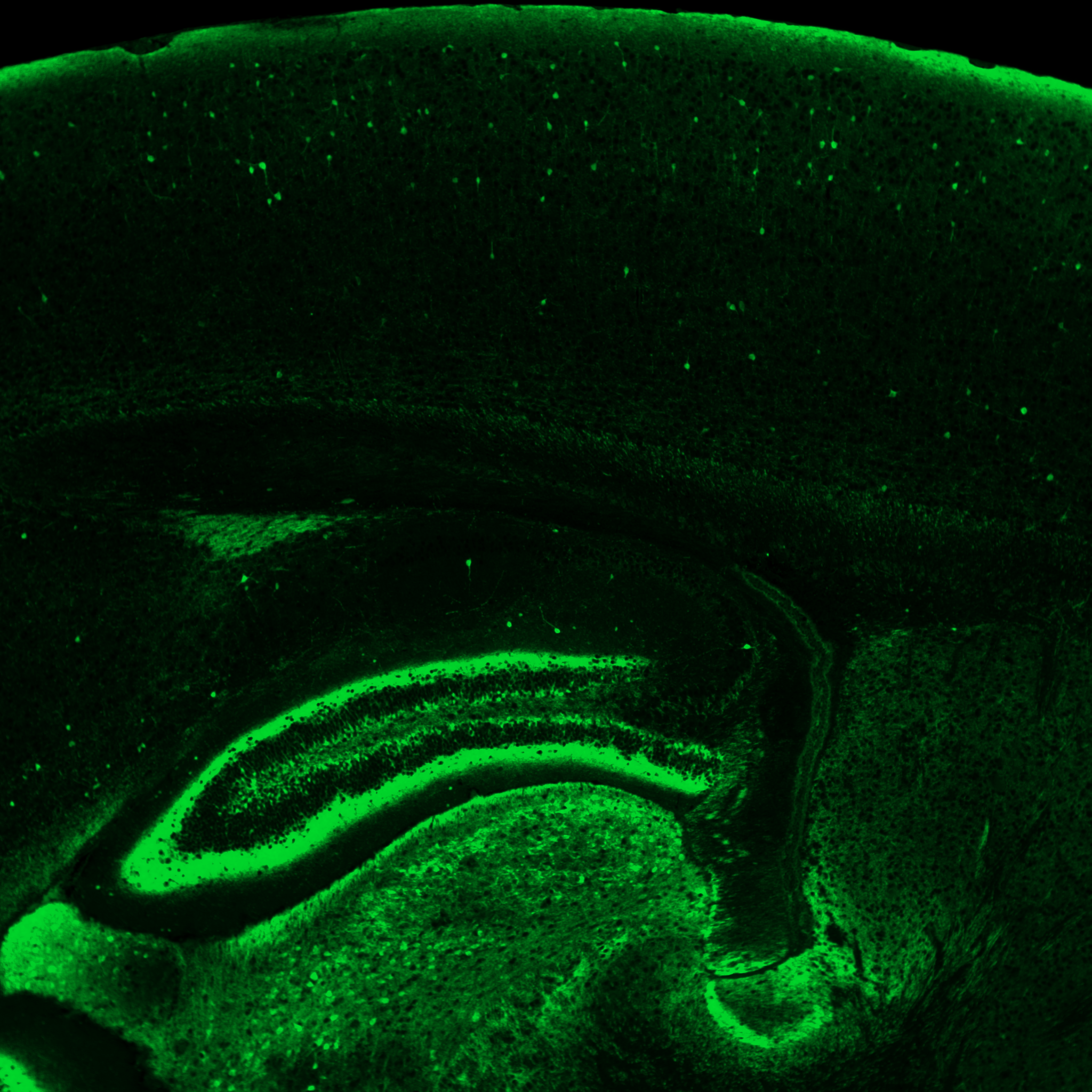

Supplement: Figure 4—source data 3. [file elife-86940-fig4-data3.zip › Figure 4-source data 3/F3094-3-CKO-RX CI FF-1M-SAGITAL-CR-24#-1-5X-dHPC-Image Export-03_AF488.tif]

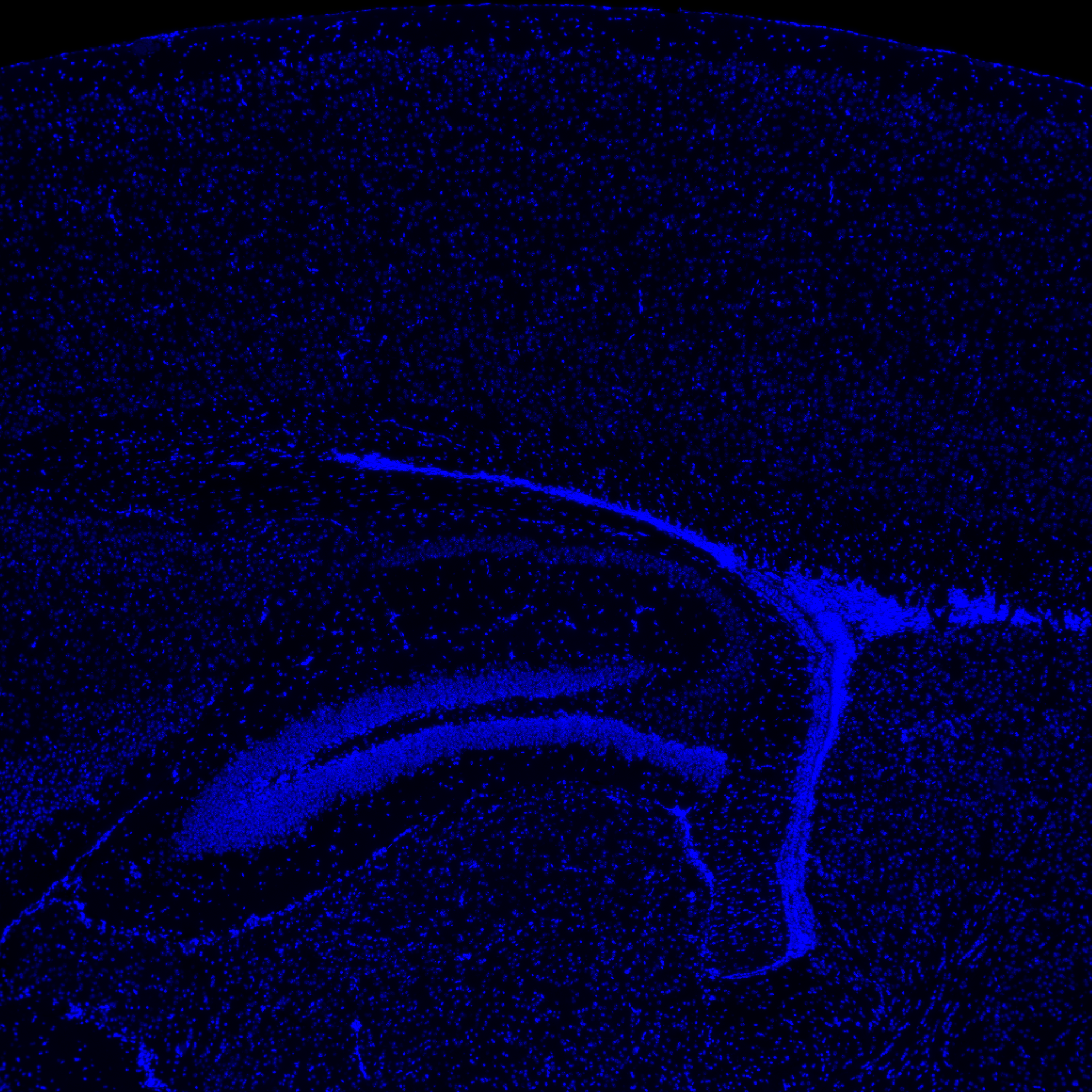

Supplement: Figure 4—source data 3. [file elife-86940-fig4-data3.zip › Figure 4-source data 3/F3094-3-CKO-RX CI FF-1M-SAGITAL-CR-24#-1-5X-dHPC-Image Export-03_DAPI.tif]

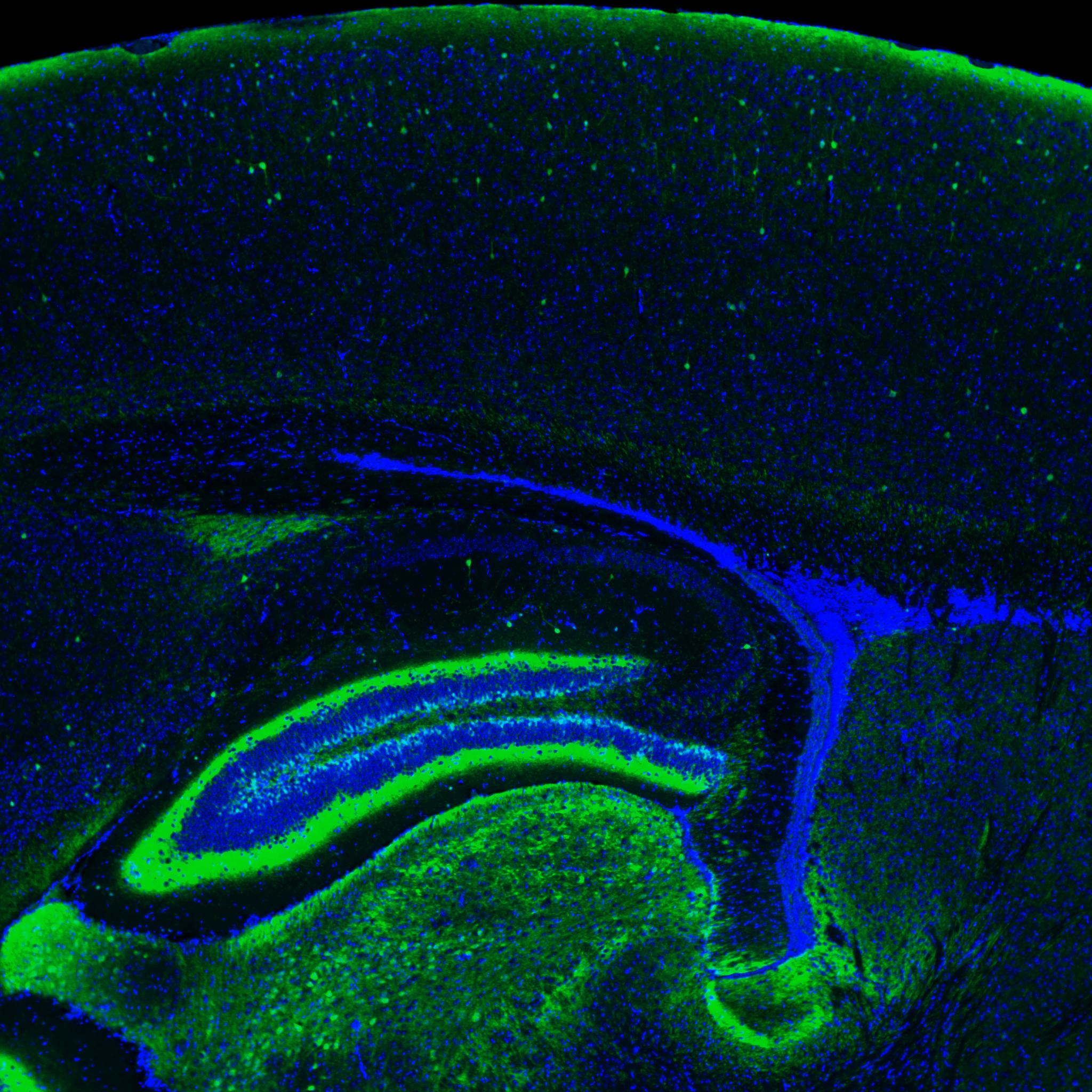

Supplement: Figure 4—source data 3. [file elife-86940-fig4-data3.zip › Figure 4-source data 3/F3094-3-CKO-RX CI FF-1M-SAGITAL-CR-24#-1-5X-dHPC-Image Export-03_G+D.tif]

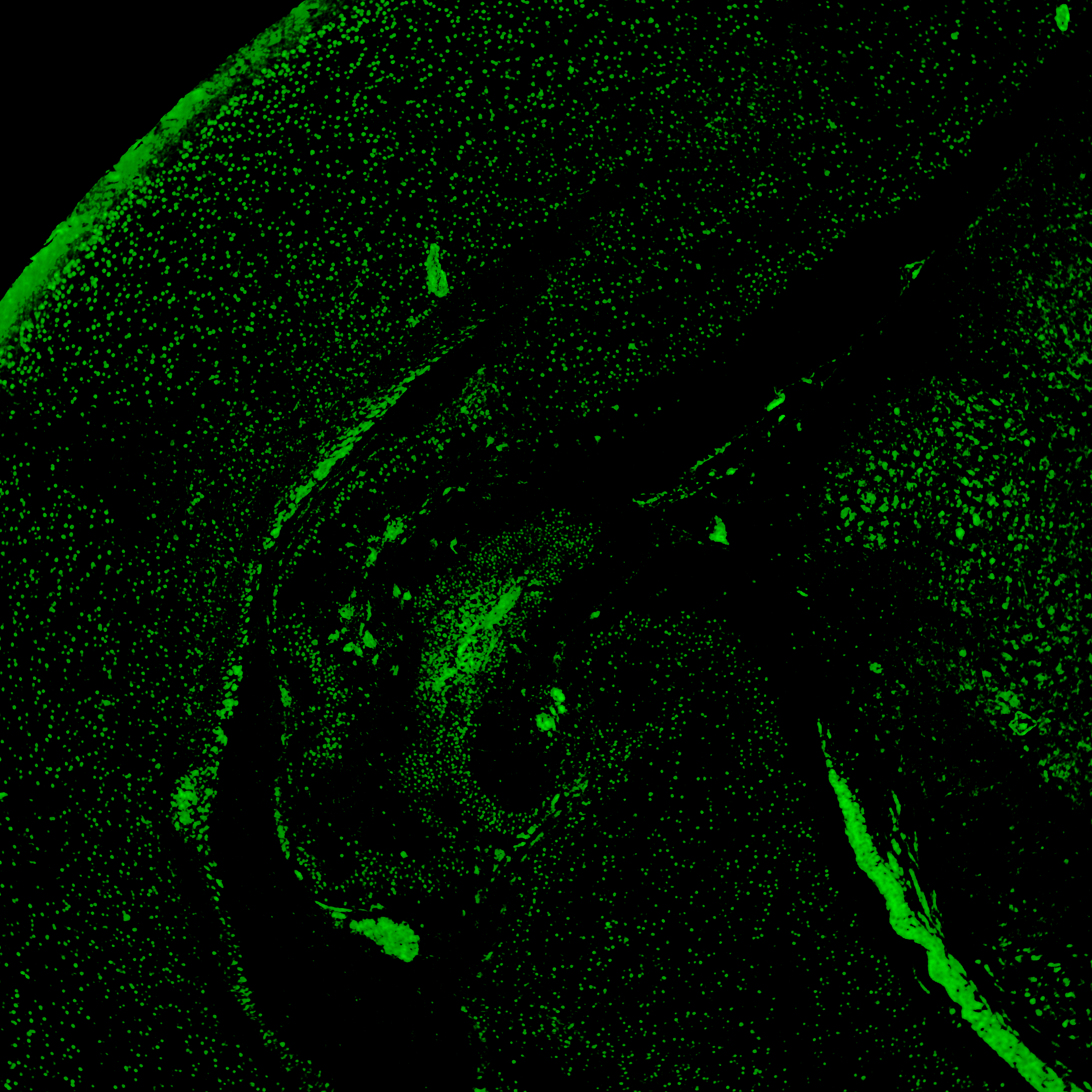

Supplement: Figure 4—source data 3. [file elife-86940-fig4-data3.zip › Figure 4-source data 3/F8099-1-DKO-RX FF ff-P20-5X-SMI312-#130-2-L-dHPC-Image Export-18_AF488.tif]

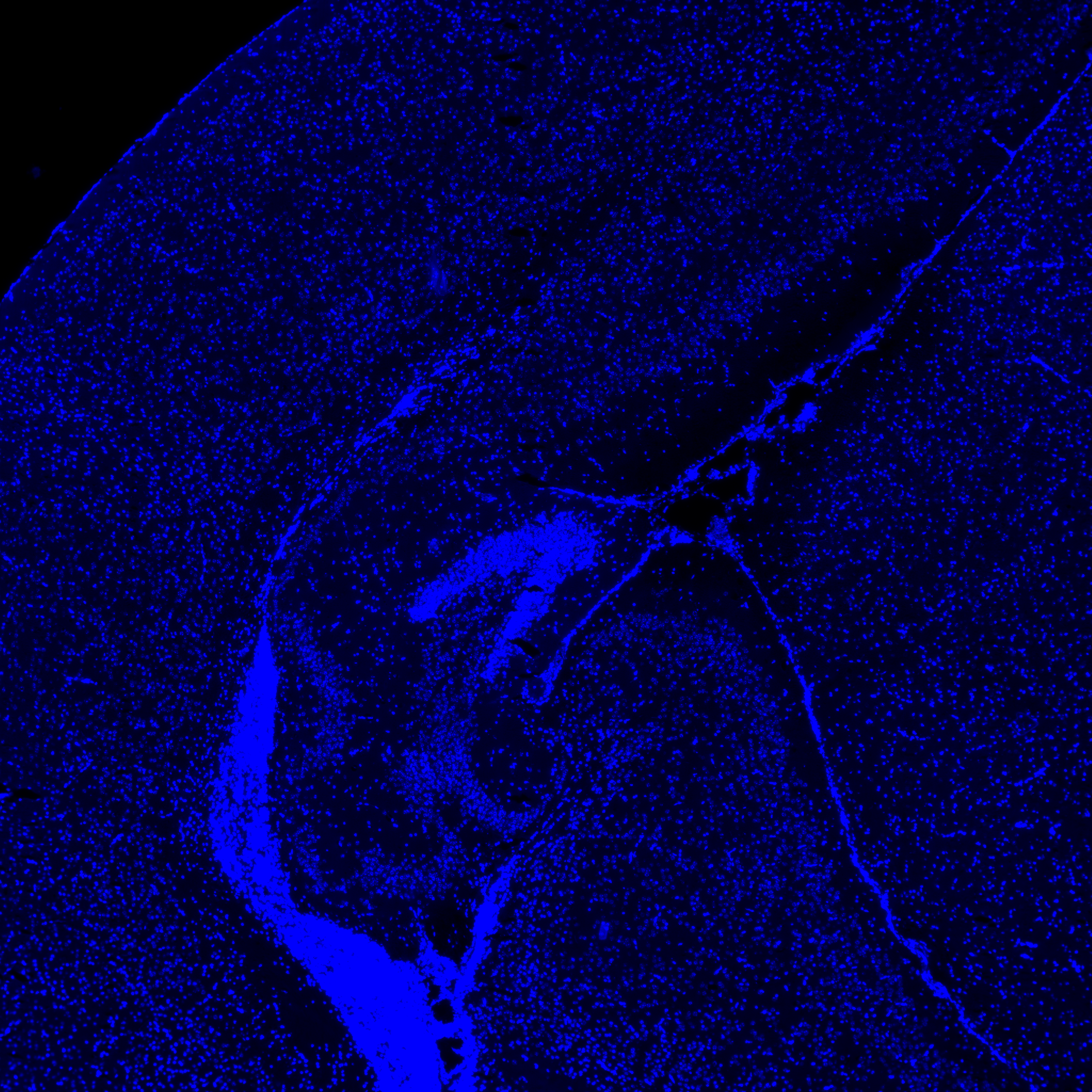

Supplement: Figure 4—source data 3. [file elife-86940-fig4-data3.zip › Figure 4-source data 3/F8099-1-DKO-RX FF ff-P20-5X-SMI312-#130-2-L-dHPC-Image Export-18_DAPI.tif]

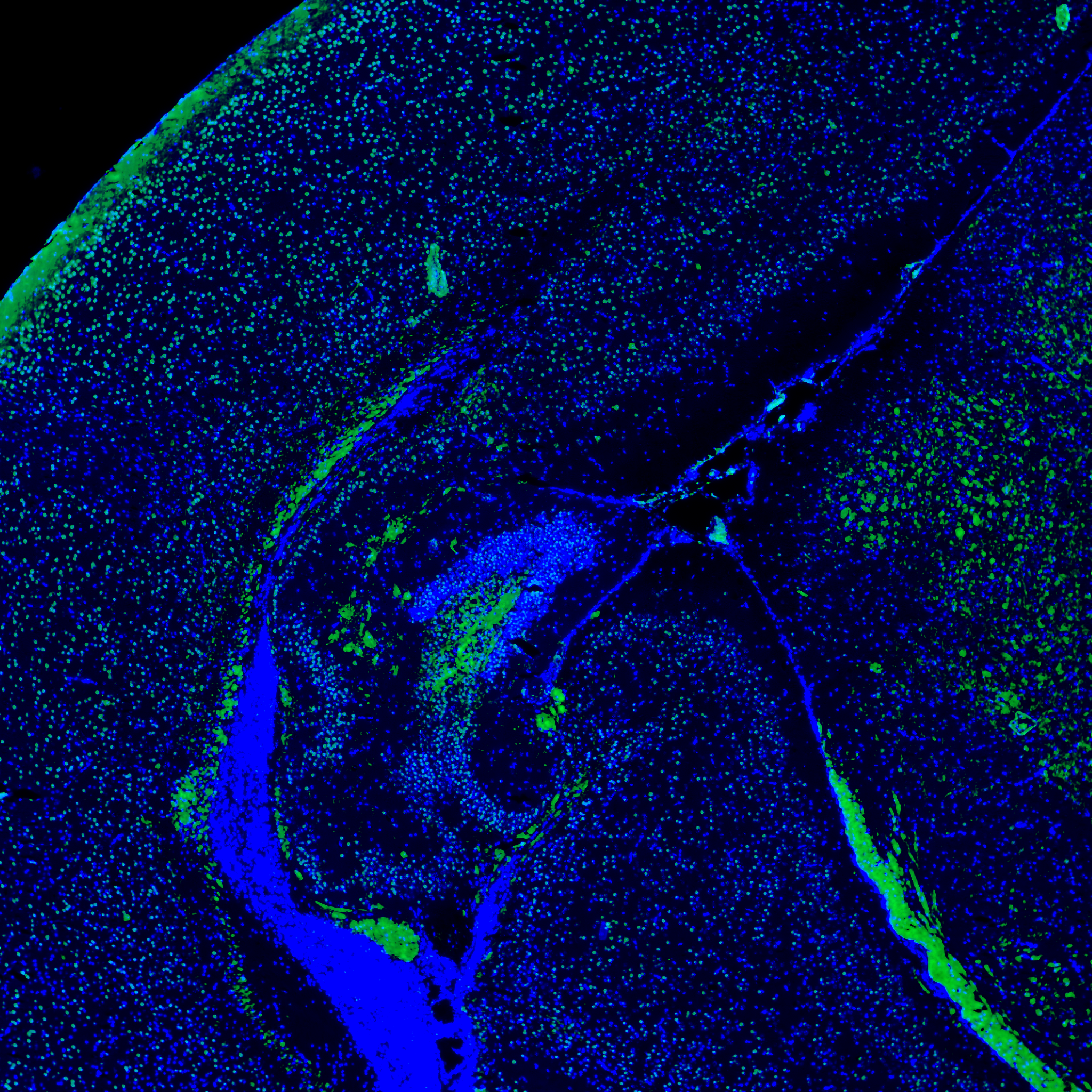

Supplement: Figure 4—source data 3. [file elife-86940-fig4-data3.zip › Figure 4-source data 3/F8099-1-DKO-RX FF ff-P20-5X-SMI312-#130-2-L-dHPC-Image Export-18_G+D.tif]
