# Supplementary figures and images for: The differentiation and integration of the hippocampal dorsoventral axis are controlled by two nuclear receptor genes (part 5 of 6)
Source: eLife. 2023 Sep 26;12:RP86940. doi: 10.7554/eLife.86940 (PMC10522401; doi:10.7554/eLife.86940)

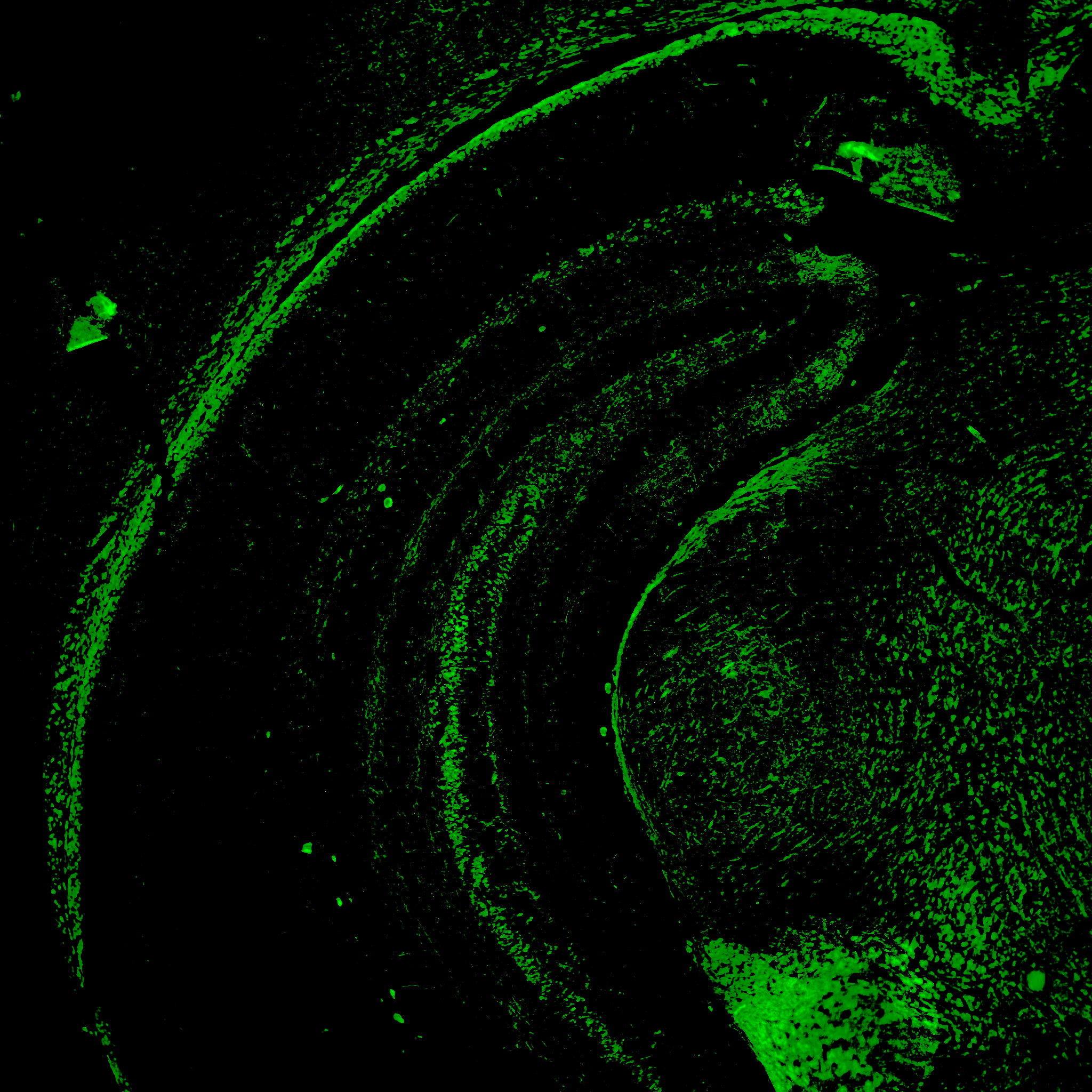

Supplement: Figure 4—source data 3. [file elife-86940-fig4-data3.zip › Figure 4-source data 3/F8099-3-CON-FF ff-P20-5X-SMI312-#141-2-L-dHPC-Image Export-11_AF488.tif]

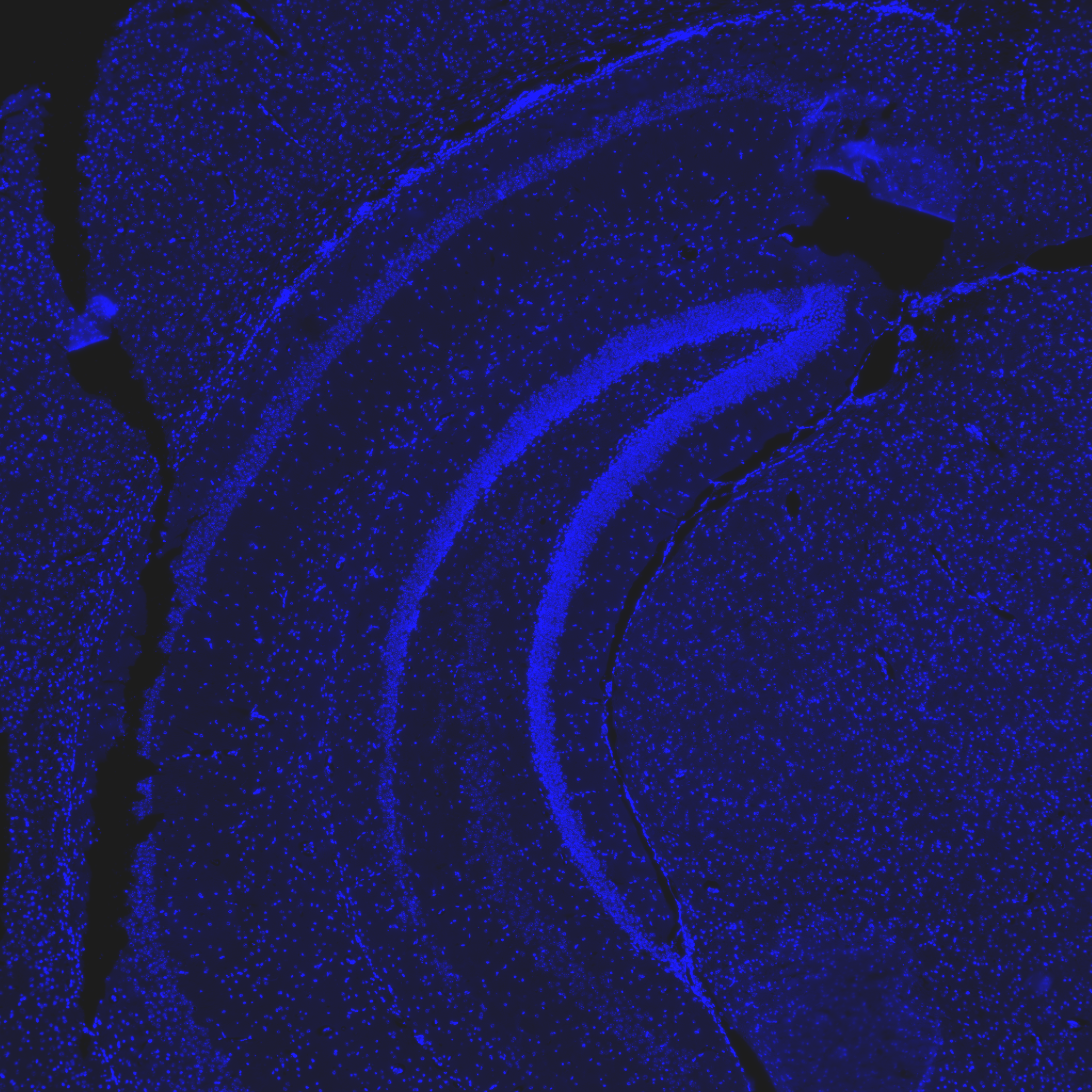

Supplement: Figure 4—source data 3. [file elife-86940-fig4-data3.zip › Figure 4-source data 3/F8099-3-CON-FF ff-P20-5X-SMI312-#141-2-L-dHPC-Image Export-11_DAPI.tif]

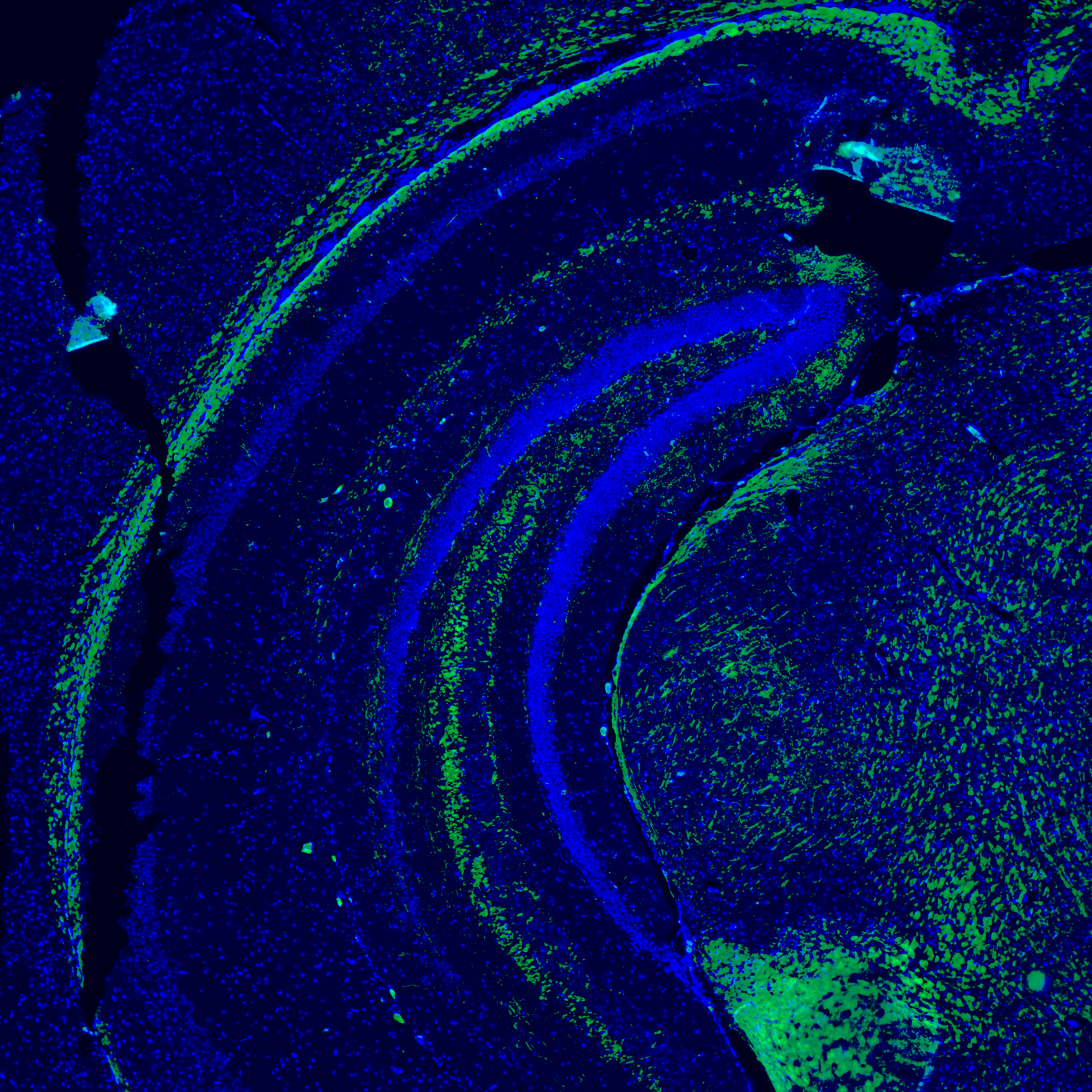

Supplement: Figure 4—source data 3. [file elife-86940-fig4-data3.zip › Figure 4-source data 3/F8099-3-CON-FF ff-P20-5X-SMI312-#141-2-L-dHPC-Image Export-11_G+D.tif]

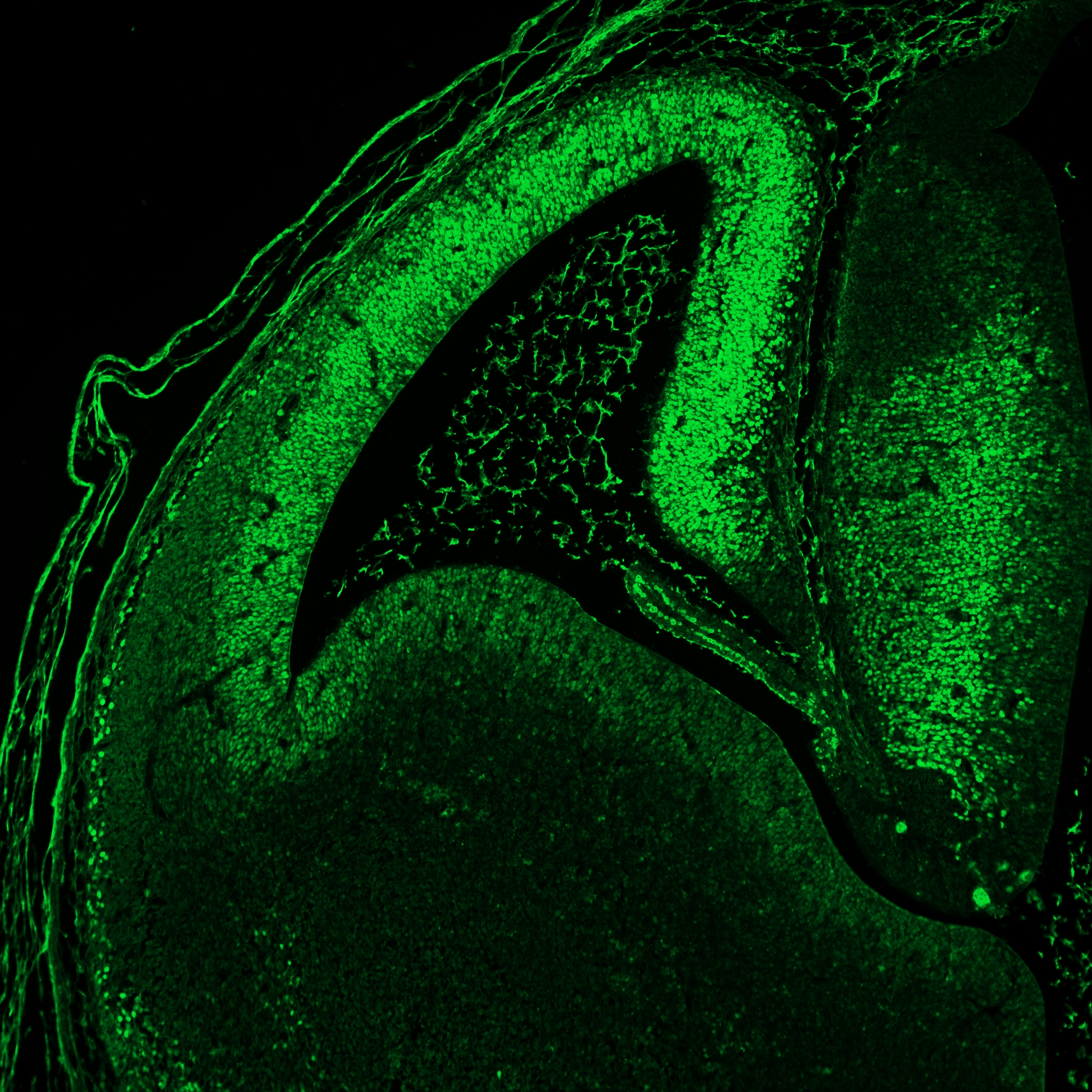

Supplement: Figure 5—source data 1. [file elife-86940-fig5-data1.zip › Figure 5-source data 1/F698-4-DKO-E14.5-RX FF ff-10X-gLhx2-25-2-L-Image Export-67_AF488.jpg]

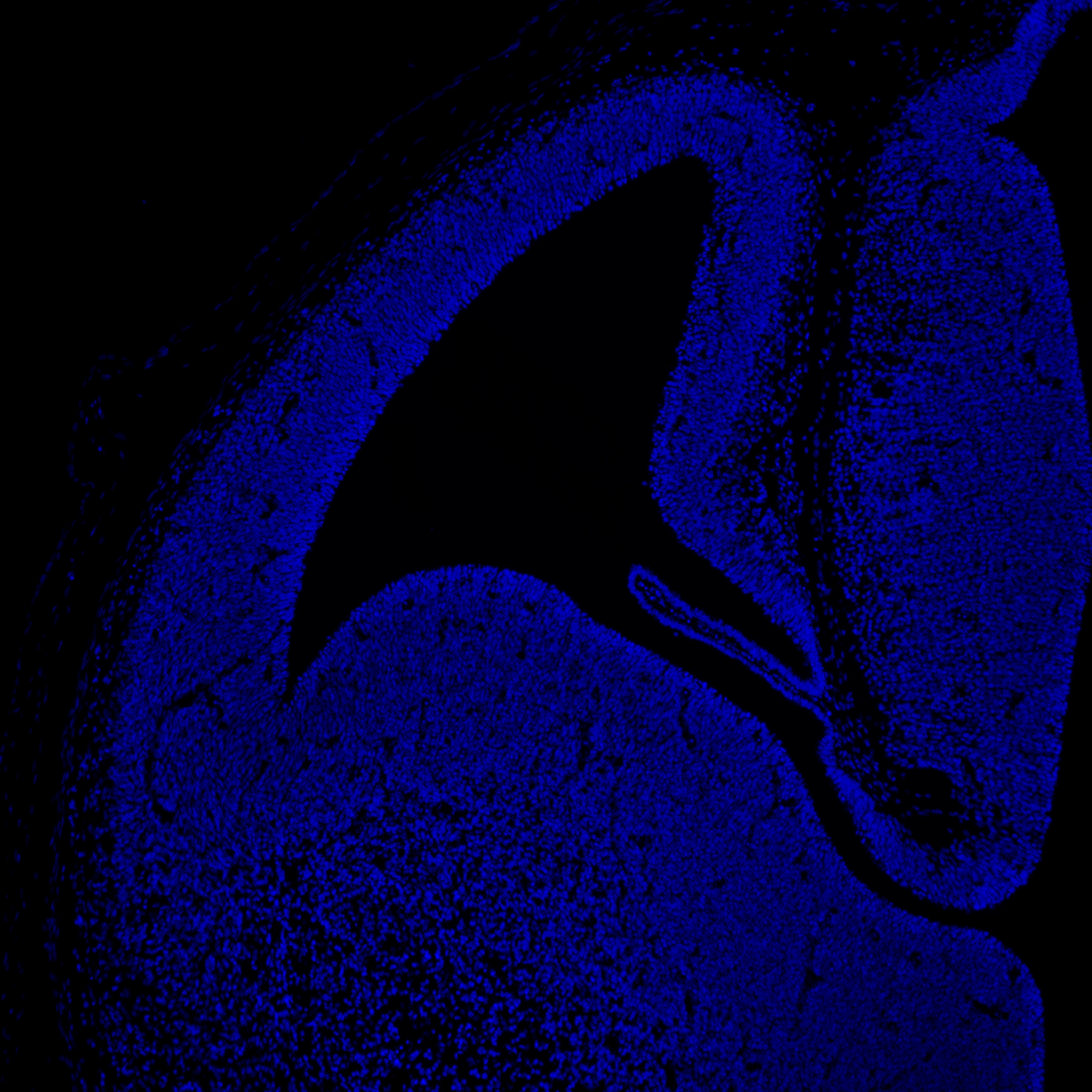

Supplement: Figure 5—source data 1. [file elife-86940-fig5-data1.zip › Figure 5-source data 1/F698-4-DKO-E14.5-RX FF ff-10X-gLhx2-25-2-L-Image Export-67_DAPI.jpg]

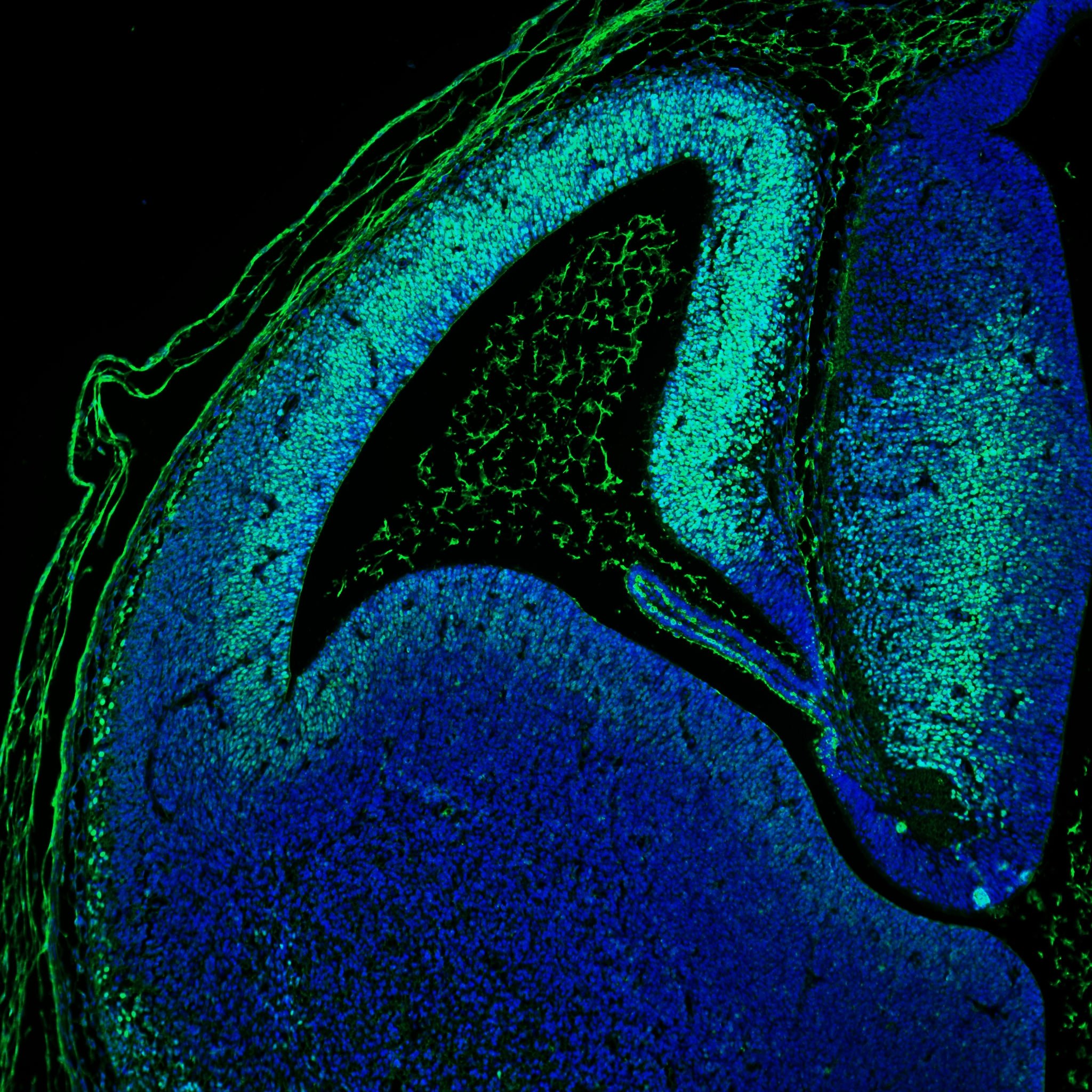

Supplement: Figure 5—source data 1. [file elife-86940-fig5-data1.zip › Figure 5-source data 1/F698-4-DKO-E14.5-RX FF ff-10X-gLhx2-25-2-L-Image Export-67.jpg]

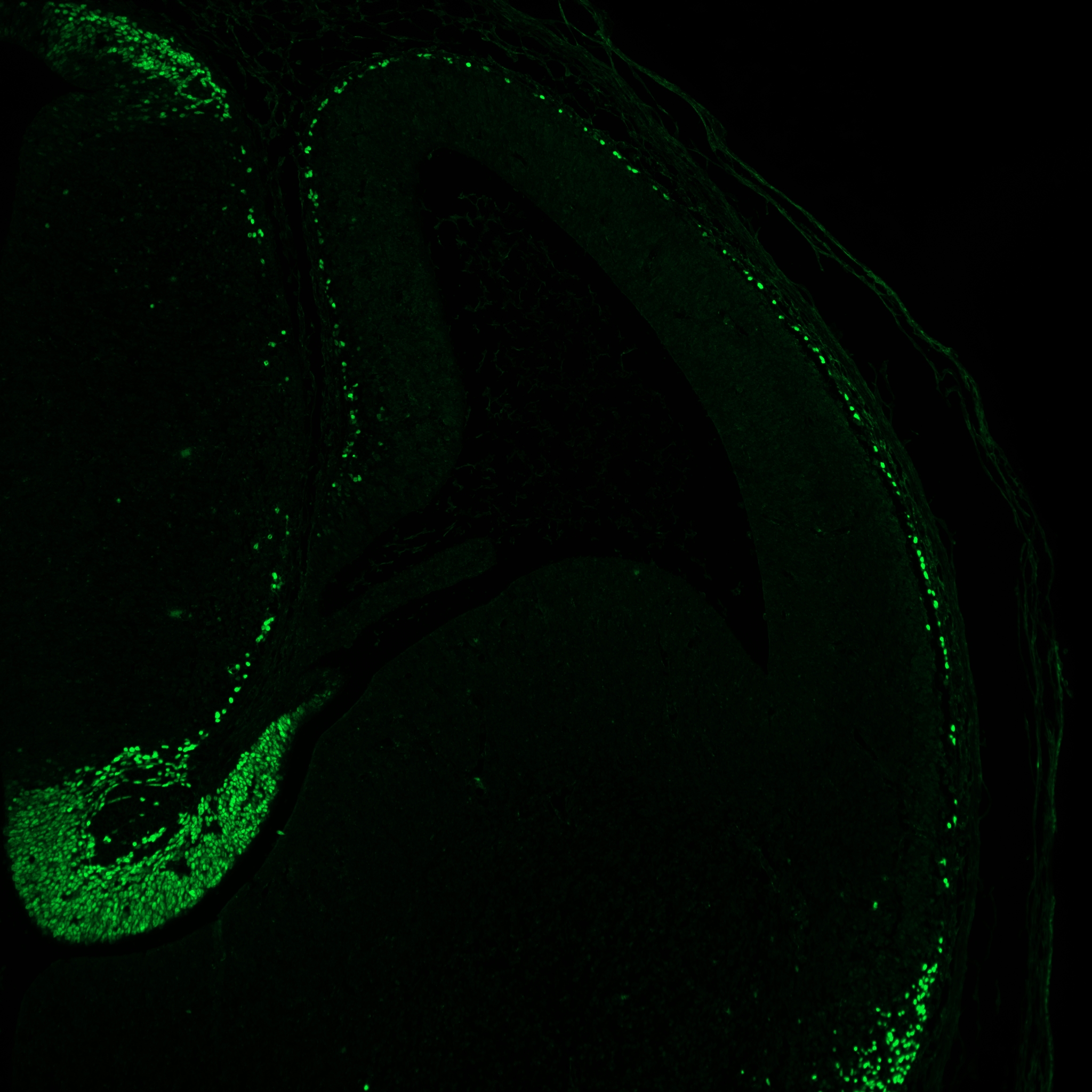

Supplement: Figure 5—source data 1. [file elife-86940-fig5-data1.zip › Figure 5-source data 1/F698-4-DKO-E14.5-RX FF ff-10X-gLhx5-27-3-R-Image Export-93_AF488.jpg]

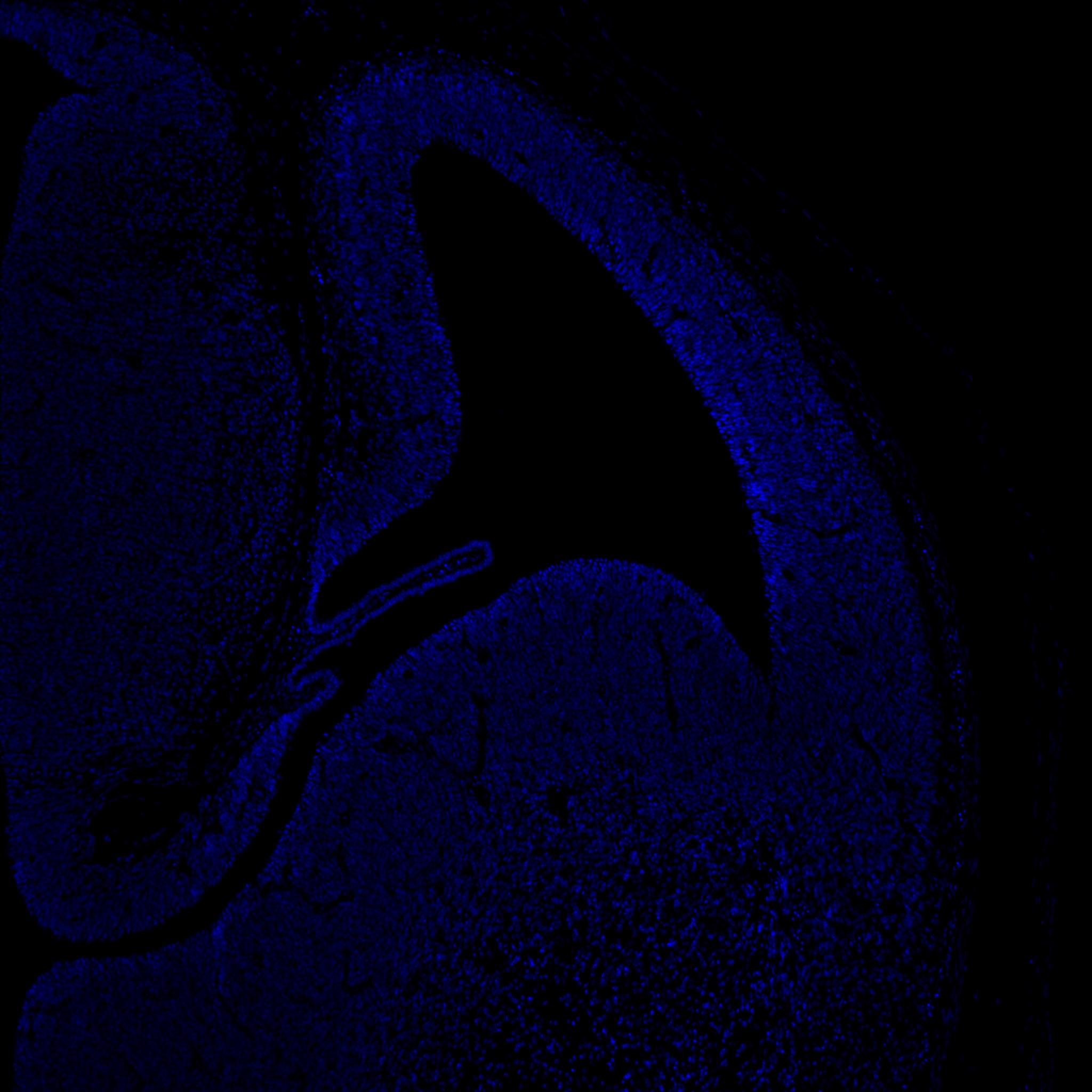

Supplement: Figure 5—source data 1. [file elife-86940-fig5-data1.zip › Figure 5-source data 1/F698-4-DKO-E14.5-RX FF ff-10X-gLhx5-27-3-R-Image Export-93_DAPI.jpg]

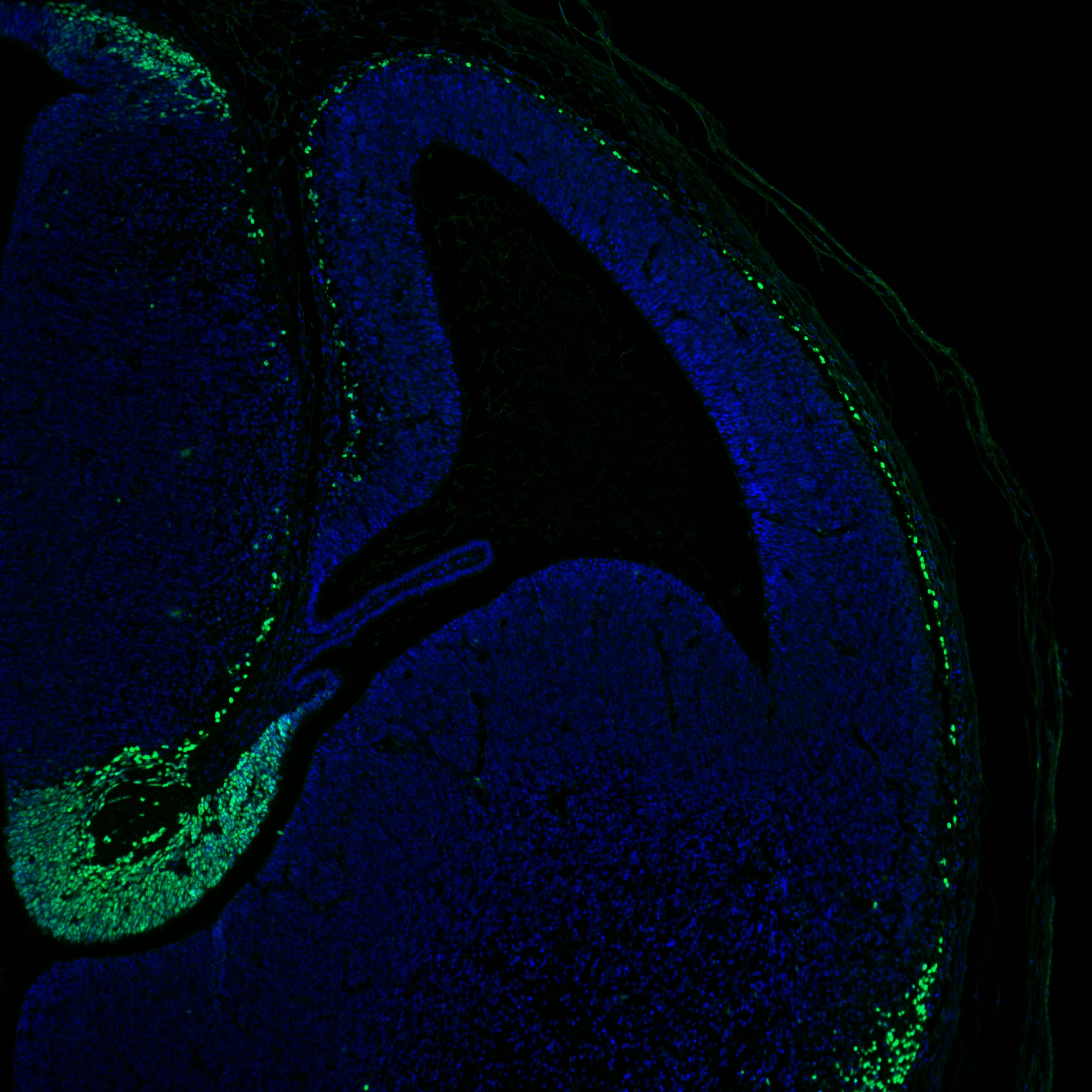

Supplement: Figure 5—source data 1. [file elife-86940-fig5-data1.zip › Figure 5-source data 1/F698-4-DKO-E14.5-RX FF ff-10X-gLhx5-27-3-R-Image Export-93.jpg]

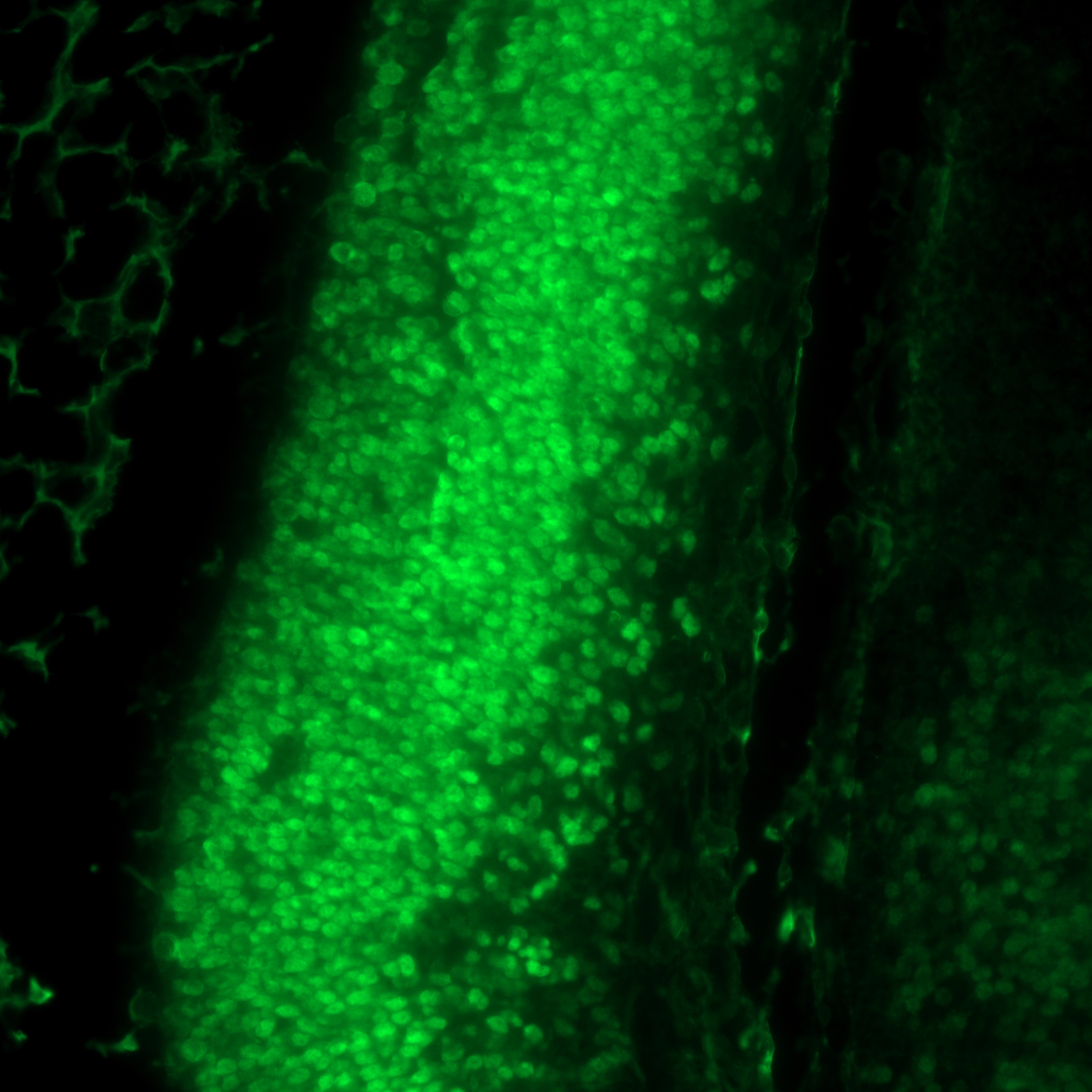

Supplement: Figure 5—source data 1. [file elife-86940-fig5-data1.zip › Figure 5-source data 1/F698-4-DKO-E14.5-RX FF ff-40X-gLhx2-25-2-L-MP-Image Export-69_AF488.jpg]

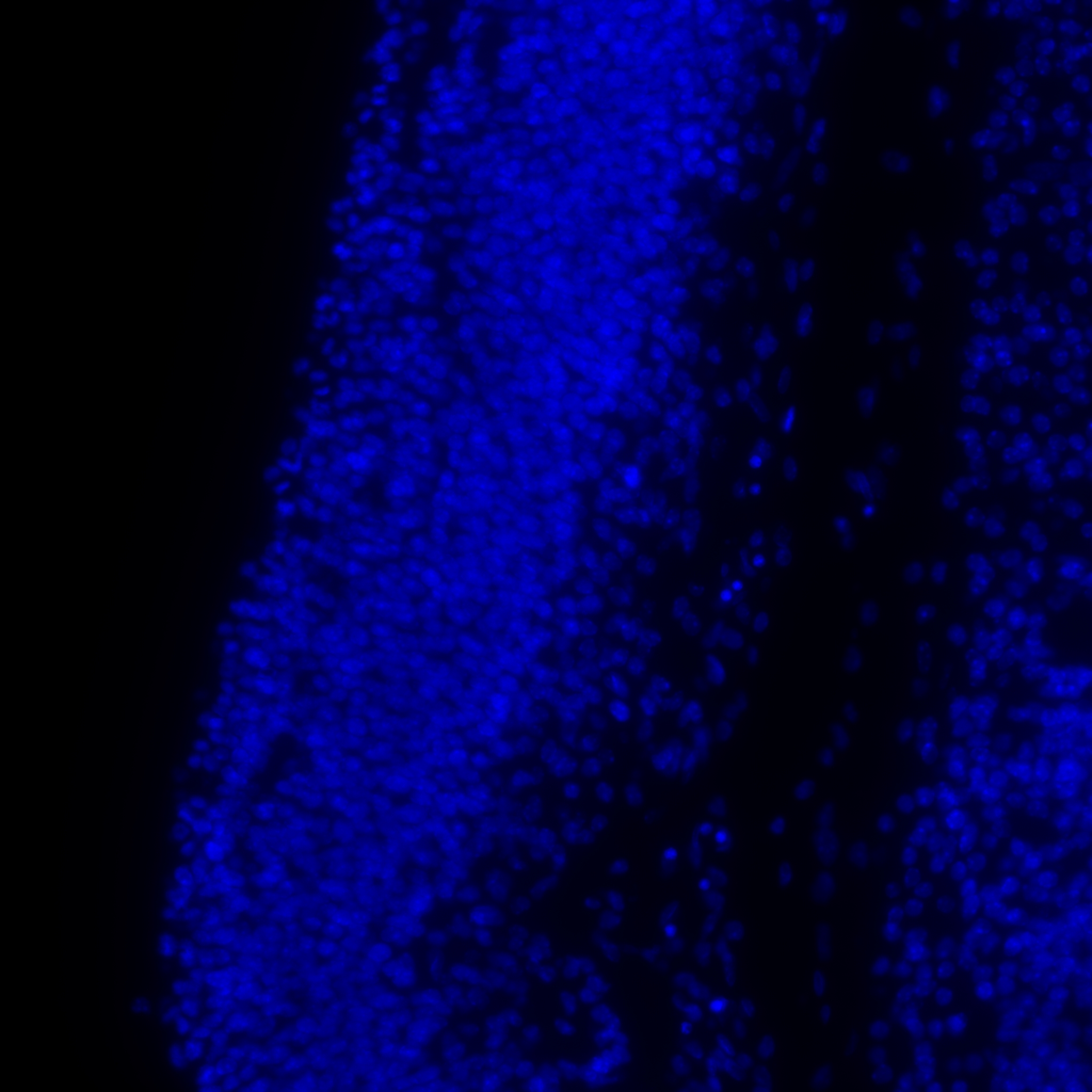

Supplement: Figure 5—source data 1. [file elife-86940-fig5-data1.zip › Figure 5-source data 1/F698-4-DKO-E14.5-RX FF ff-40X-gLhx2-25-2-L-MP-Image Export-69_DAPI.jpg]

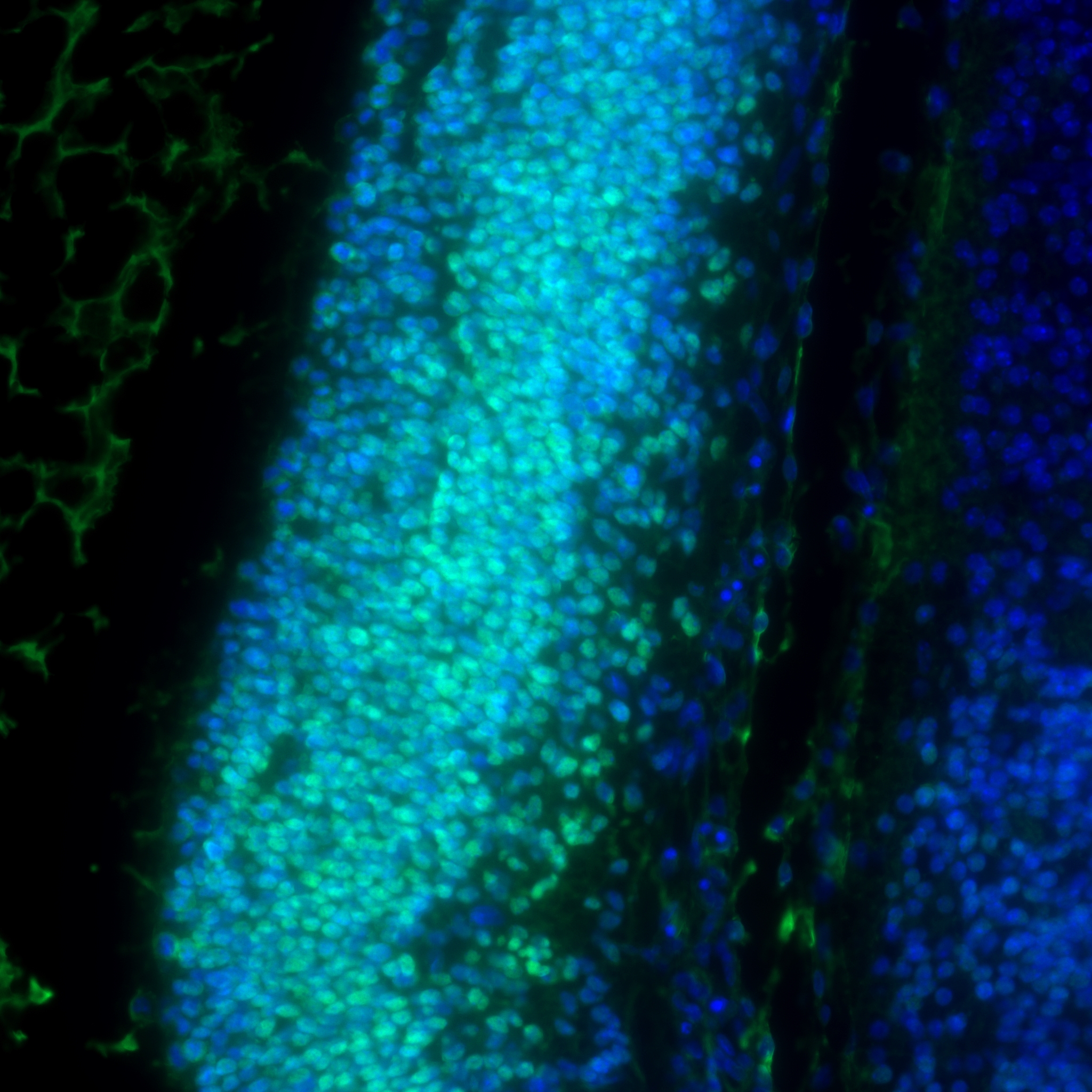

Supplement: Figure 5—source data 1. [file elife-86940-fig5-data1.zip › Figure 5-source data 1/F698-4-DKO-E14.5-RX FF ff-40X-gLhx2-25-2-L-MP-Image Export-69.jpg]

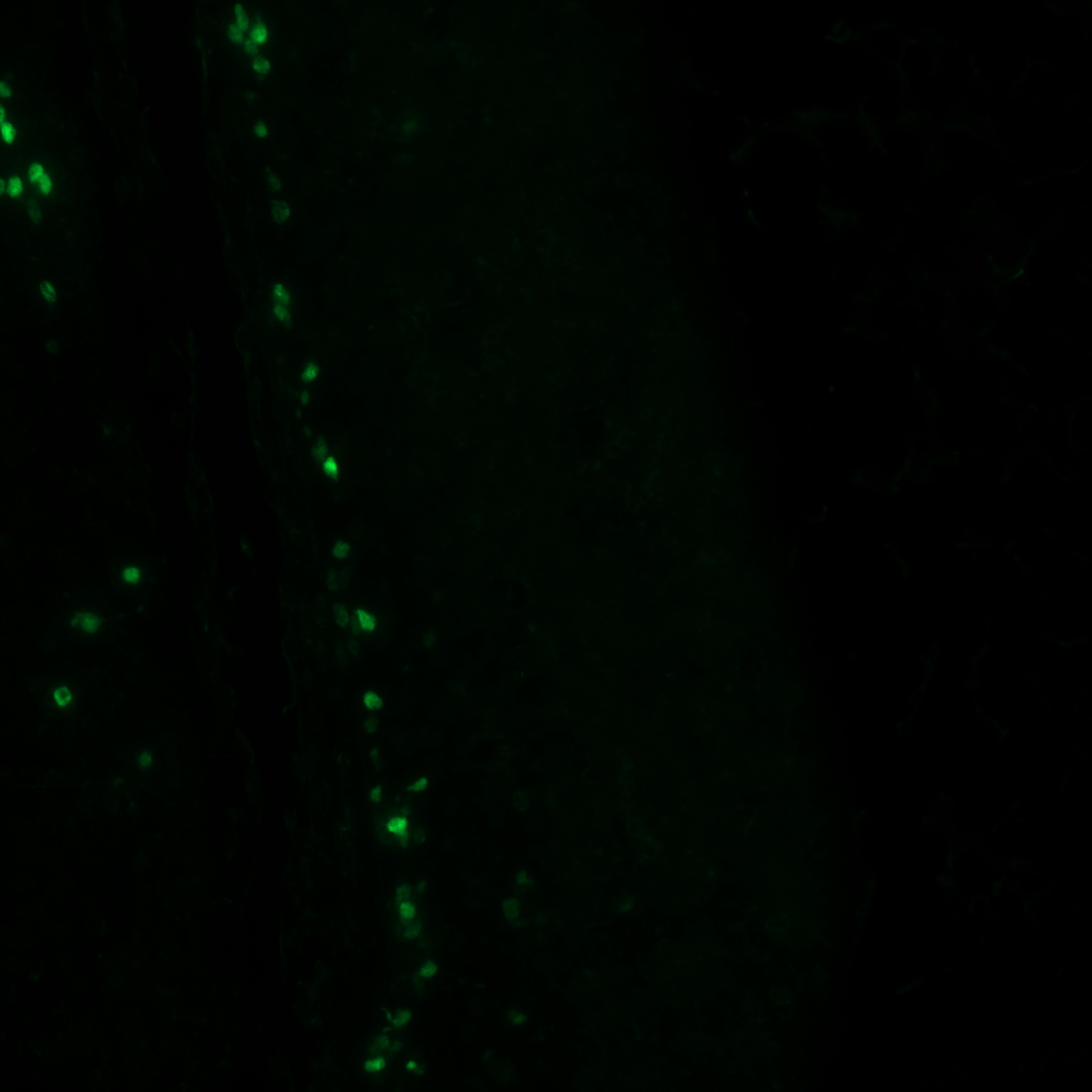

Supplement: Figure 5—source data 1. [file elife-86940-fig5-data1.zip › Figure 5-source data 1/F698-4-DKO-E14.5-RX FF ff-40X-gLhx5-27-3-R-MP-Image Export-95_AF488.jpg]

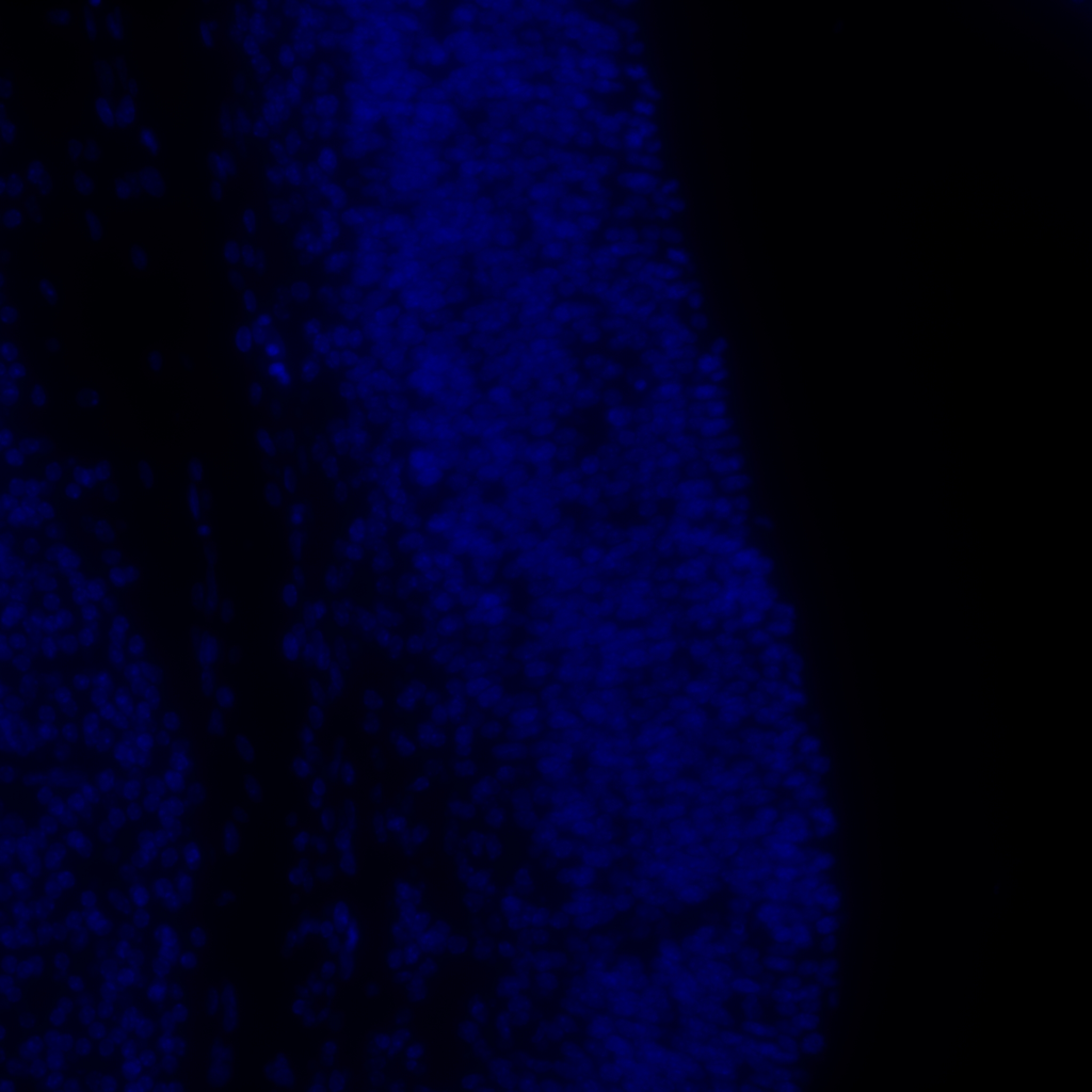

Supplement: Figure 5—source data 1. [file elife-86940-fig5-data1.zip › Figure 5-source data 1/F698-4-DKO-E14.5-RX FF ff-40X-gLhx5-27-3-R-MP-Image Export-95_DAPI.jpg]

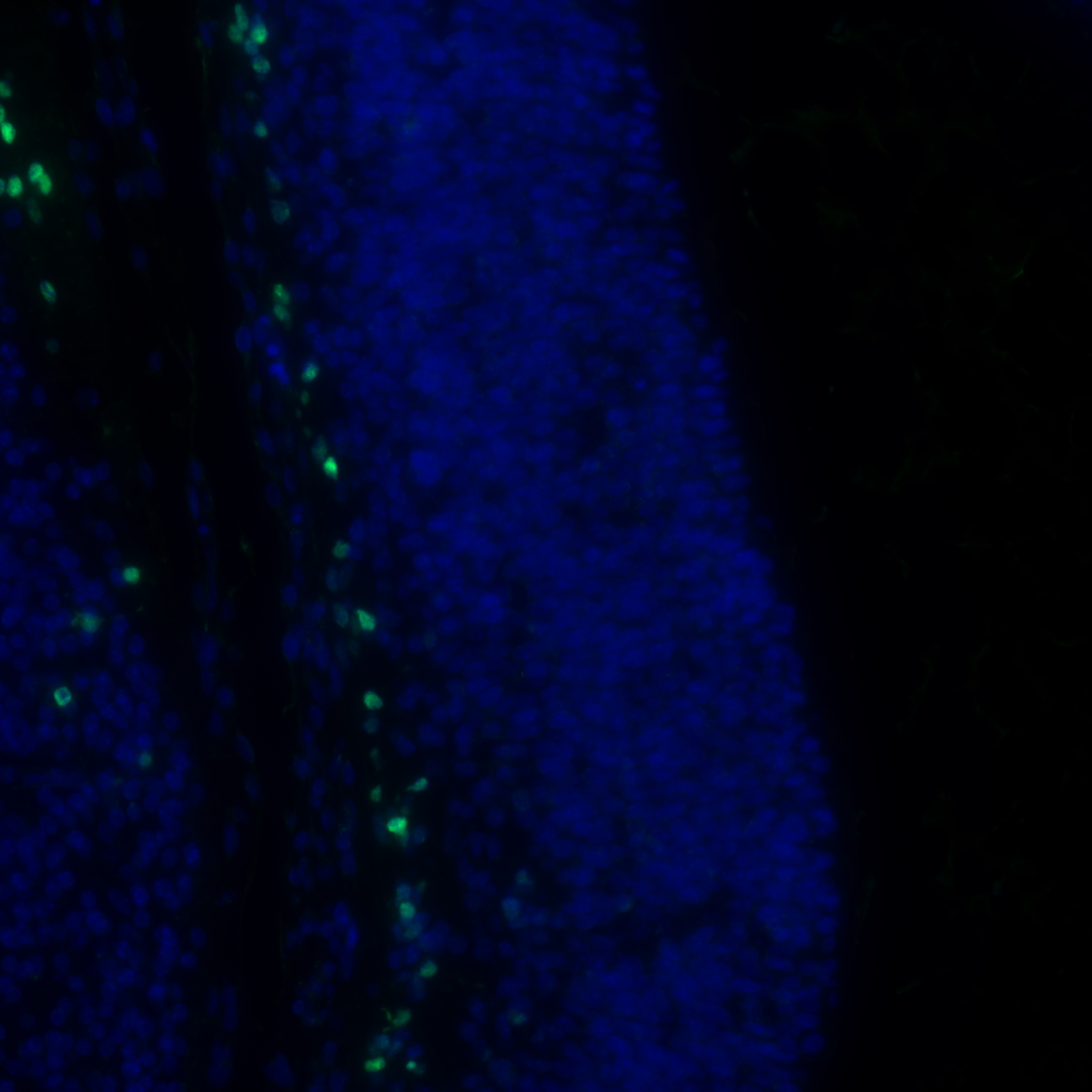

Supplement: Figure 5—source data 1. [file elife-86940-fig5-data1.zip › Figure 5-source data 1/F698-4-DKO-E14.5-RX FF ff-40X-gLhx5-27-3-R-MP-Image Export-95.jpg]

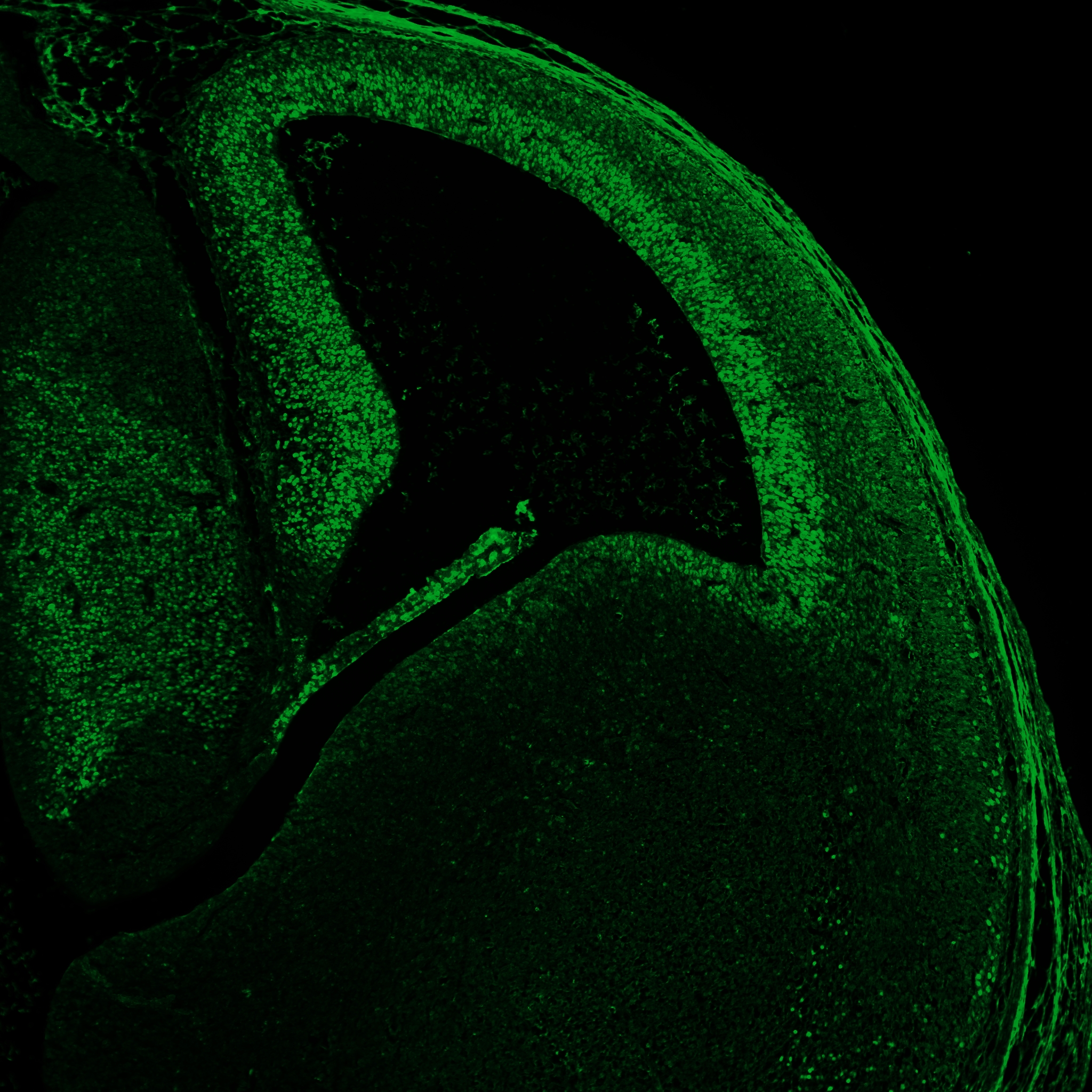

Supplement: Figure 5—source data 1. [file elife-86940-fig5-data1.zip › Figure 5-source data 1/F1189-4-CON-E14.5-RX F+ f+-10X-gLhx2-32-1-R-Image Export-43_AF488.jpg]

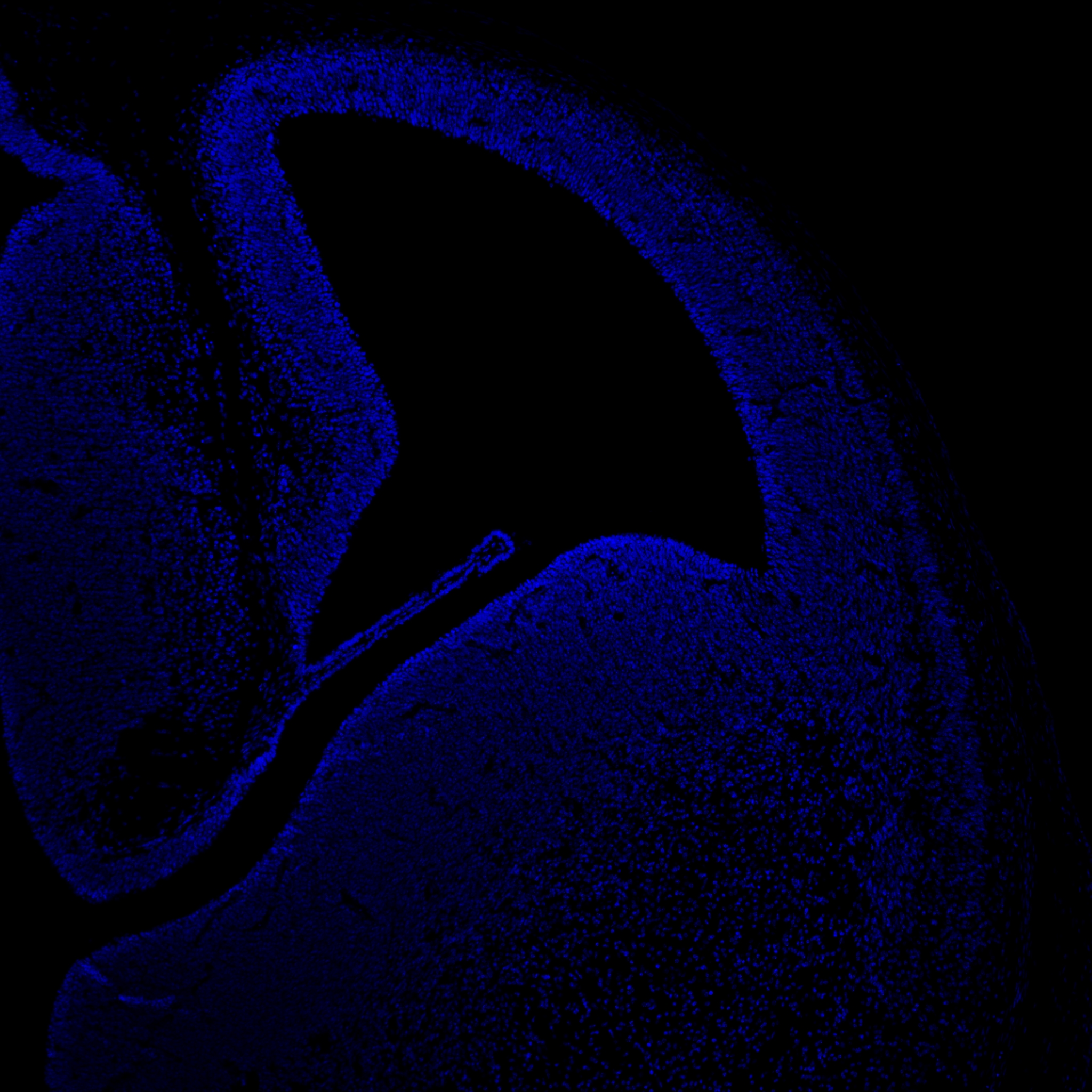

Supplement: Figure 5—source data 1. [file elife-86940-fig5-data1.zip › Figure 5-source data 1/F1189-4-CON-E14.5-RX F+ f+-10X-gLhx2-32-1-R-Image Export-43_DAPI.jpg]

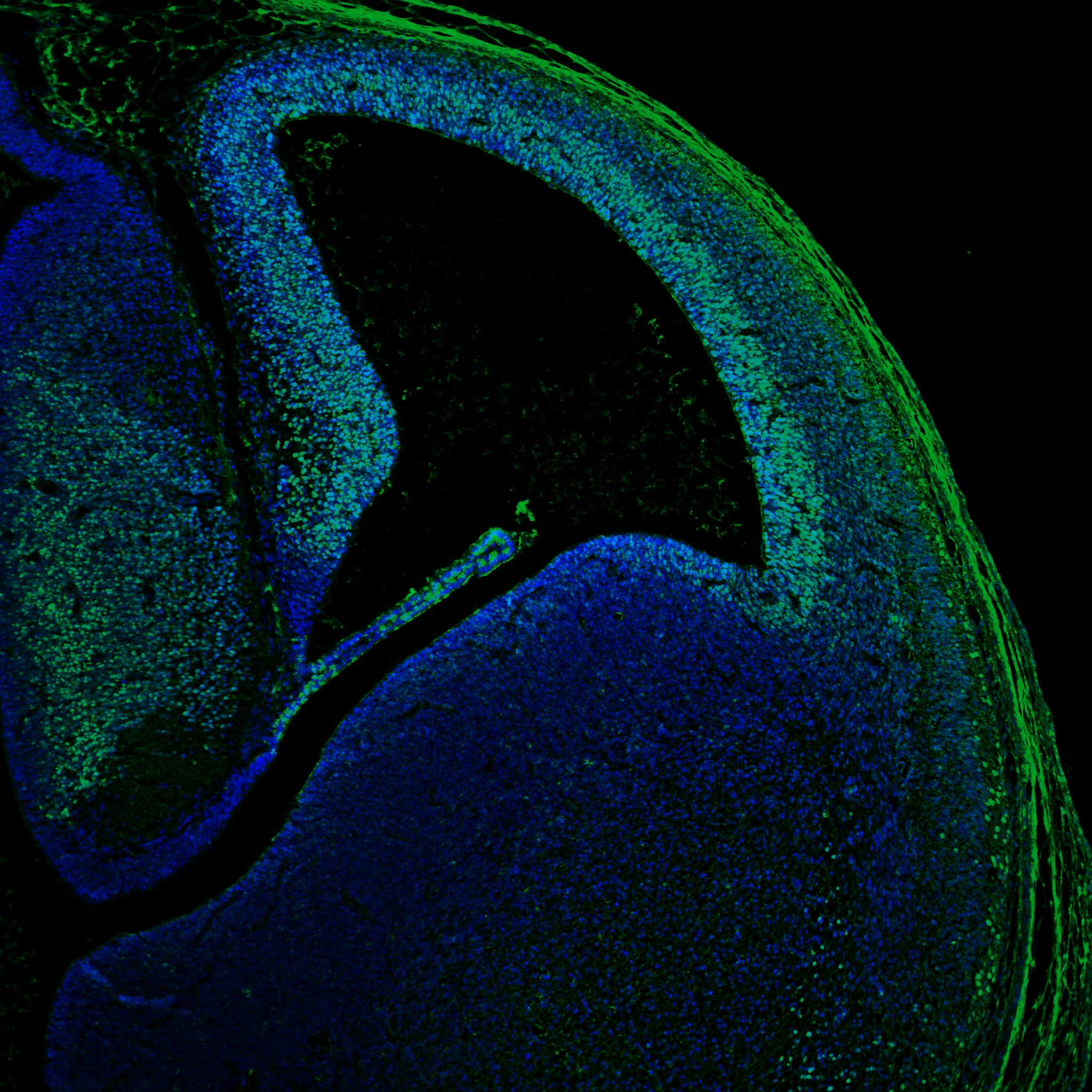

Supplement: Figure 5—source data 1. [file elife-86940-fig5-data1.zip › Figure 5-source data 1/F1189-4-CON-E14.5-RX F+ f+-10X-gLhx2-32-1-R-Image Export-43.jpg]

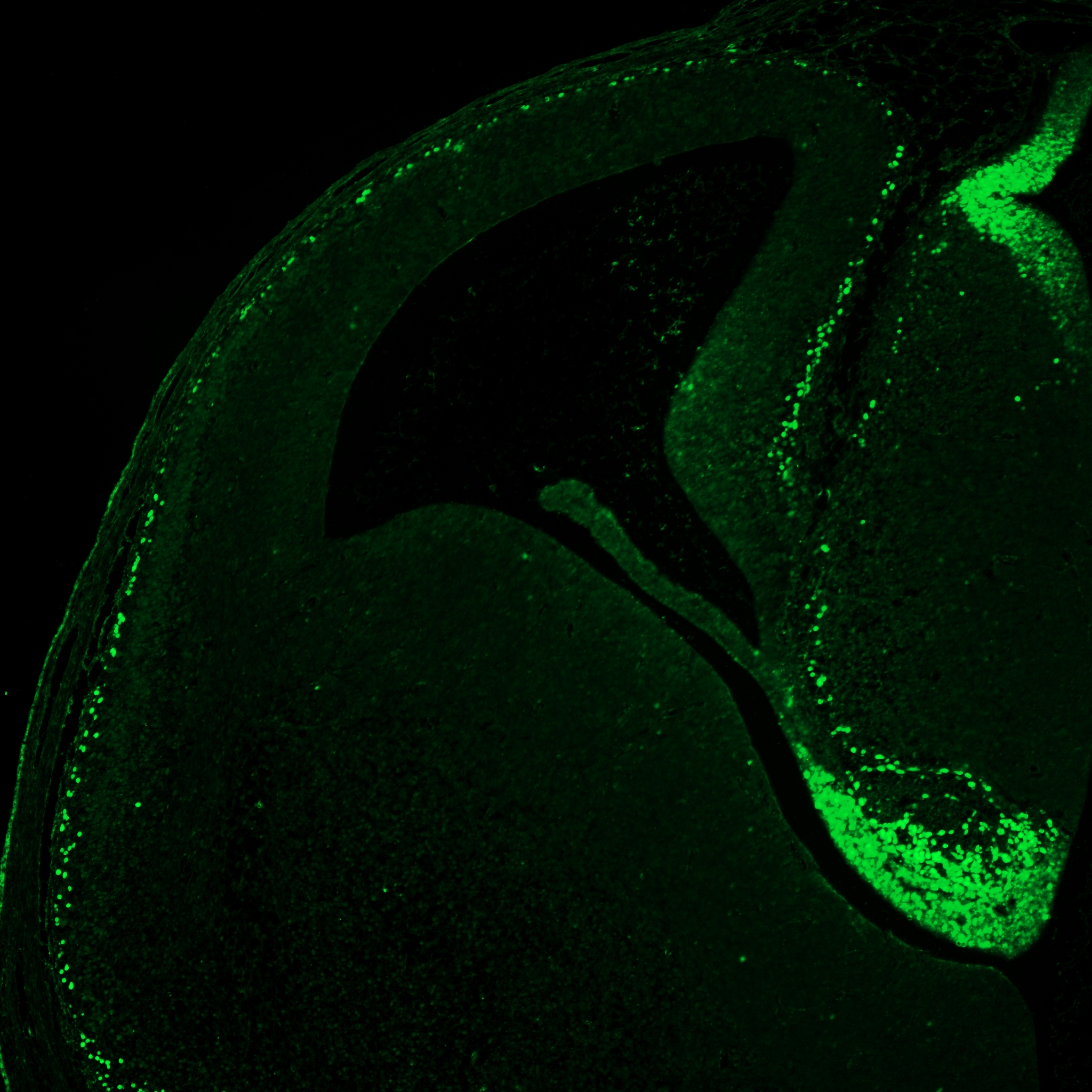

Supplement: Figure 5—source data 1. [file elife-86940-fig5-data1.zip › Figure 5-source data 1/F1189-4-CON-E14.5-RX F+ f+-10X-gLhx5-32-4-L-Image Export-57_AF488.jpg]

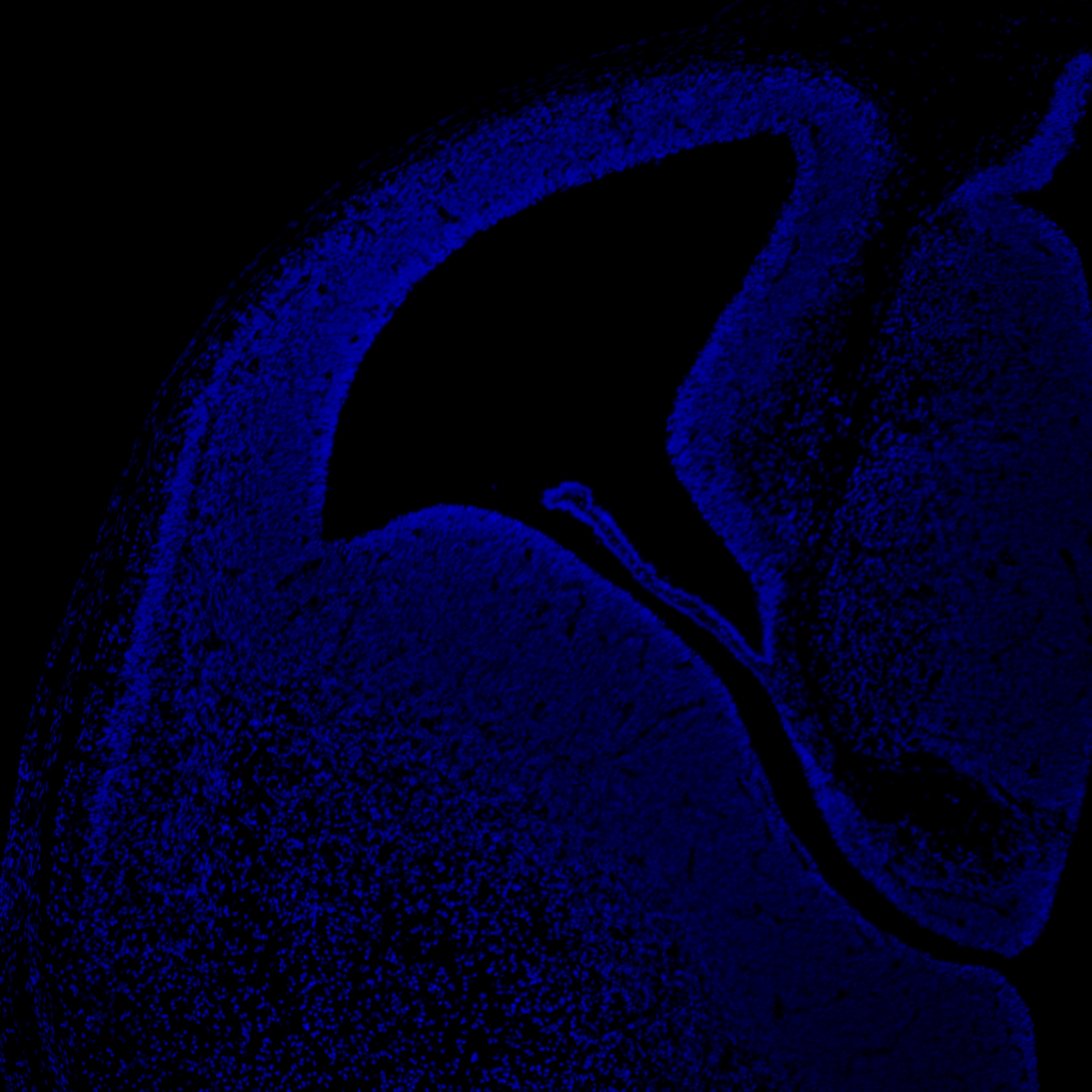

Supplement: Figure 5—source data 1. [file elife-86940-fig5-data1.zip › Figure 5-source data 1/F1189-4-CON-E14.5-RX F+ f+-10X-gLhx5-32-4-L-Image Export-57_DAPI.jpg]

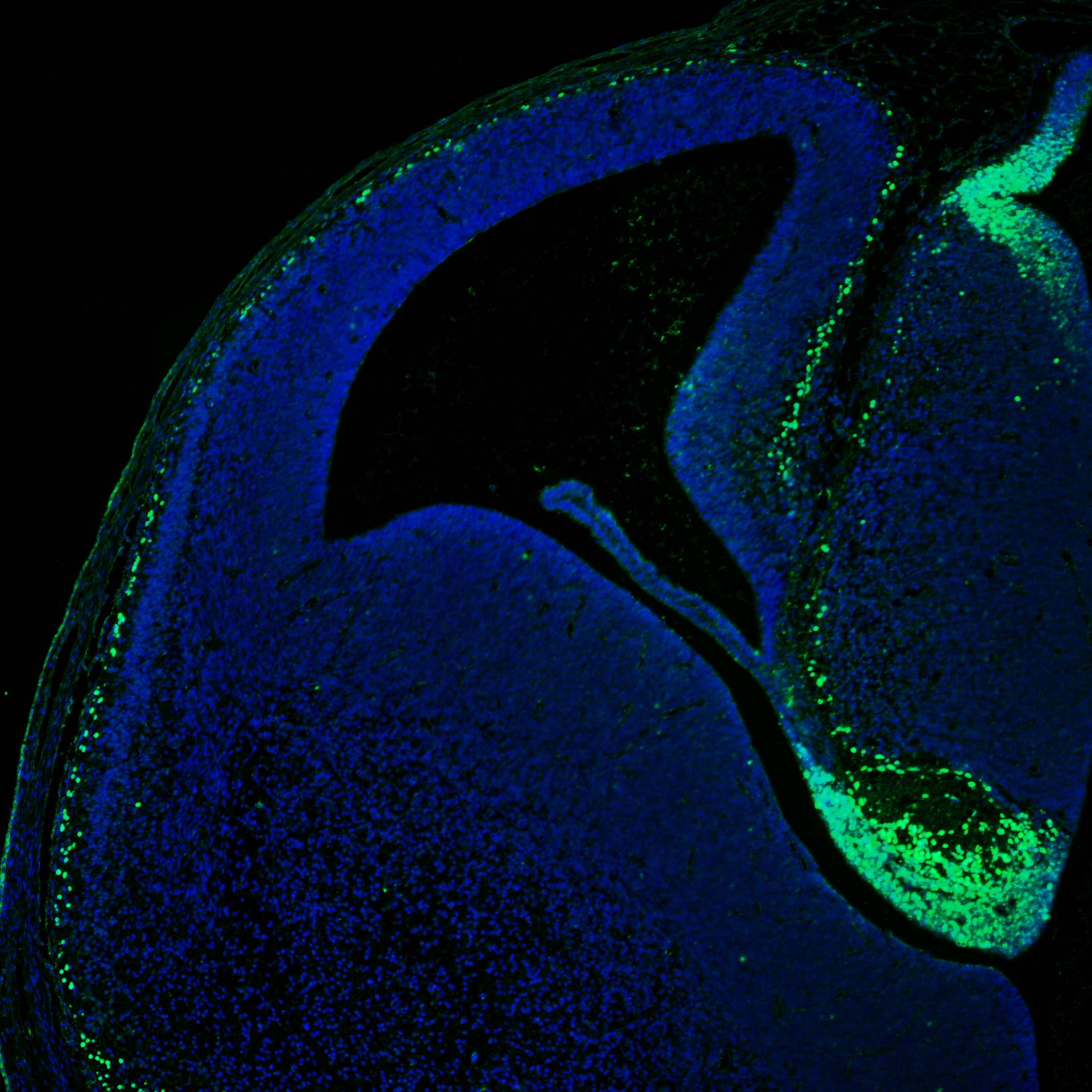

Supplement: Figure 5—source data 1. [file elife-86940-fig5-data1.zip › Figure 5-source data 1/F1189-4-CON-E14.5-RX F+ f+-10X-gLhx5-32-4-L-Image Export-57.jpg]

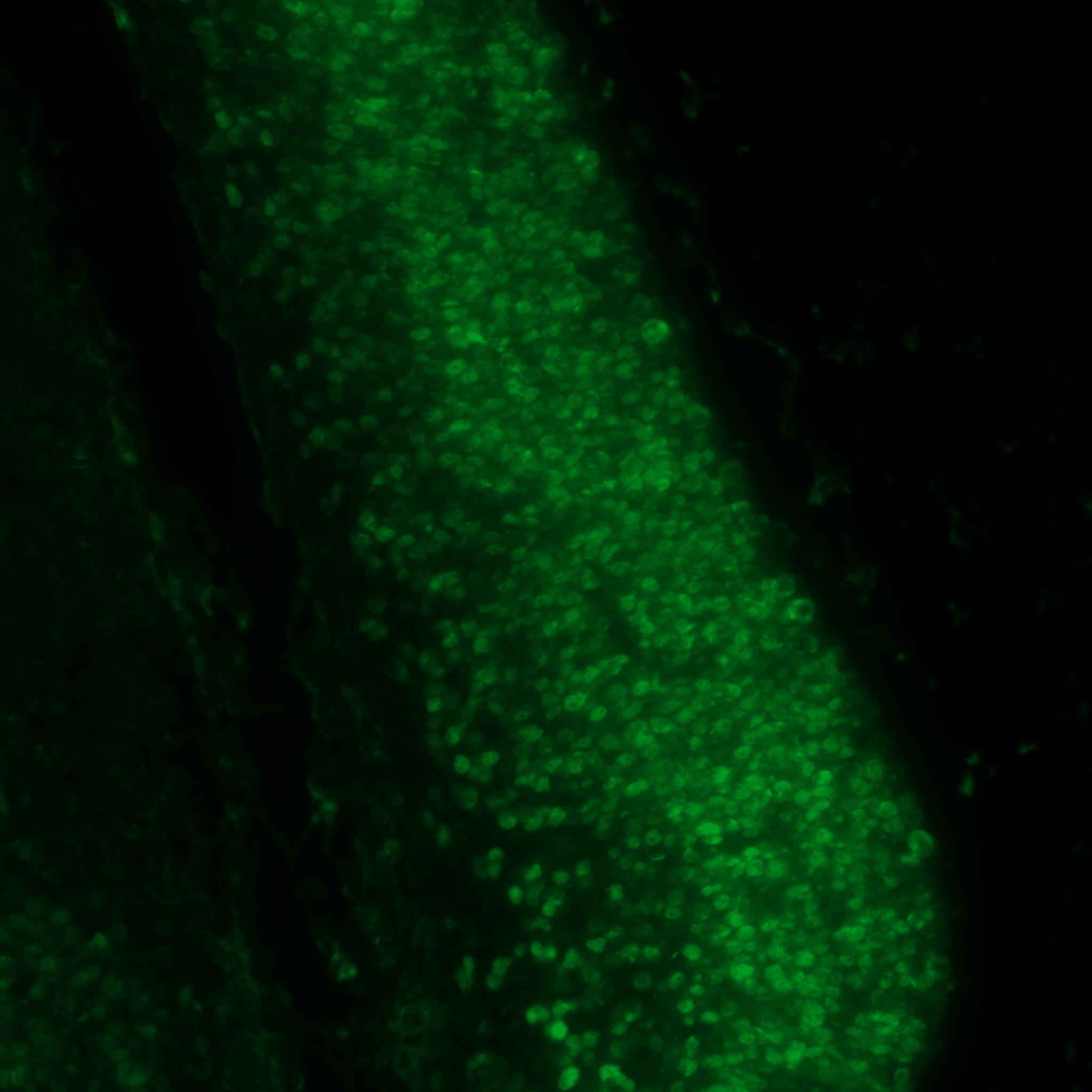

Supplement: Figure 5—source data 1. [file elife-86940-fig5-data1.zip › Figure 5-source data 1/F1189-4-CON-E14.5-RX F+ f+-40X-gLhx2-32-1-R-MP-Image Export-45_AF488.jpg]

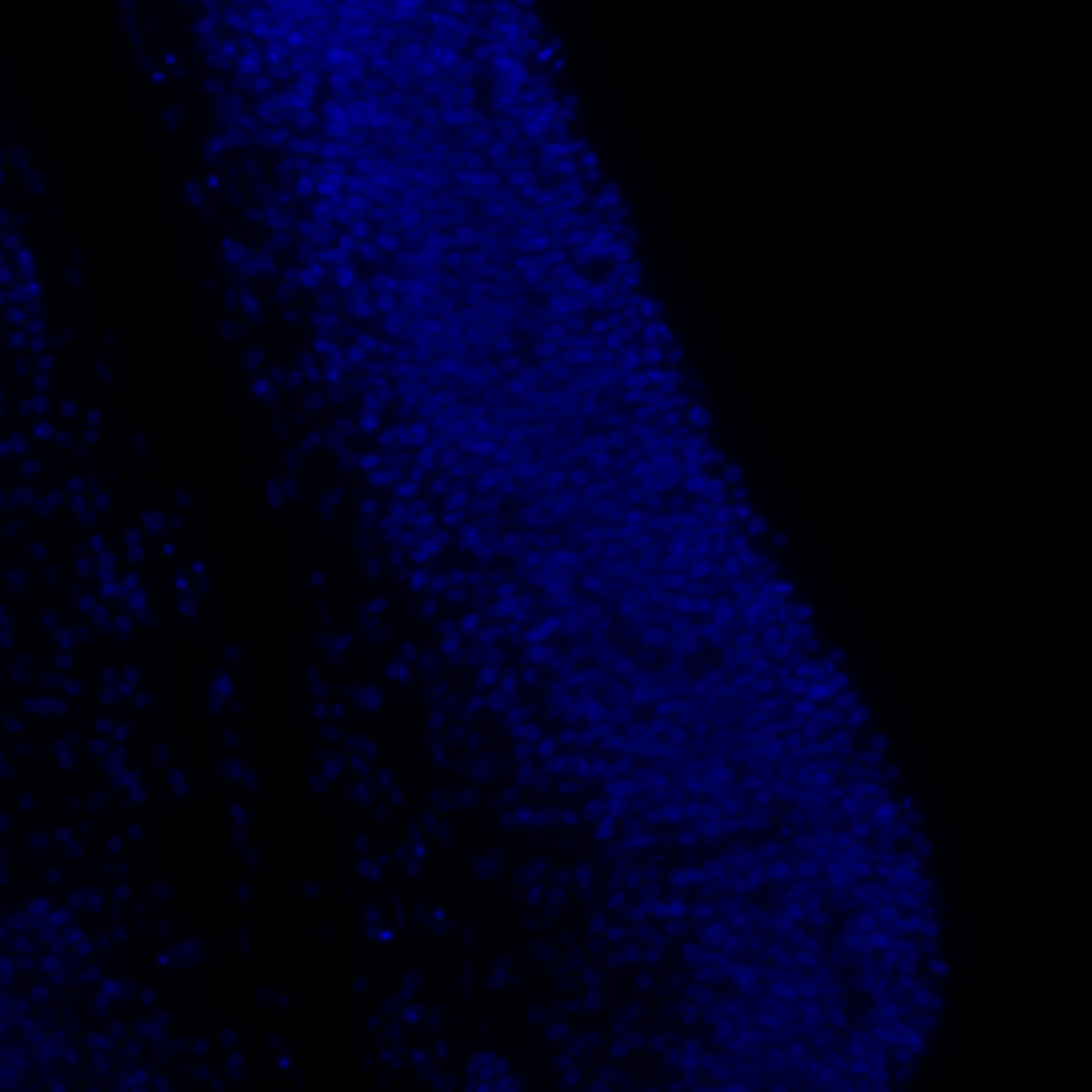

Supplement: Figure 5—source data 1. [file elife-86940-fig5-data1.zip › Figure 5-source data 1/F1189-4-CON-E14.5-RX F+ f+-40X-gLhx2-32-1-R-MP-Image Export-45_DAPI.jpg]

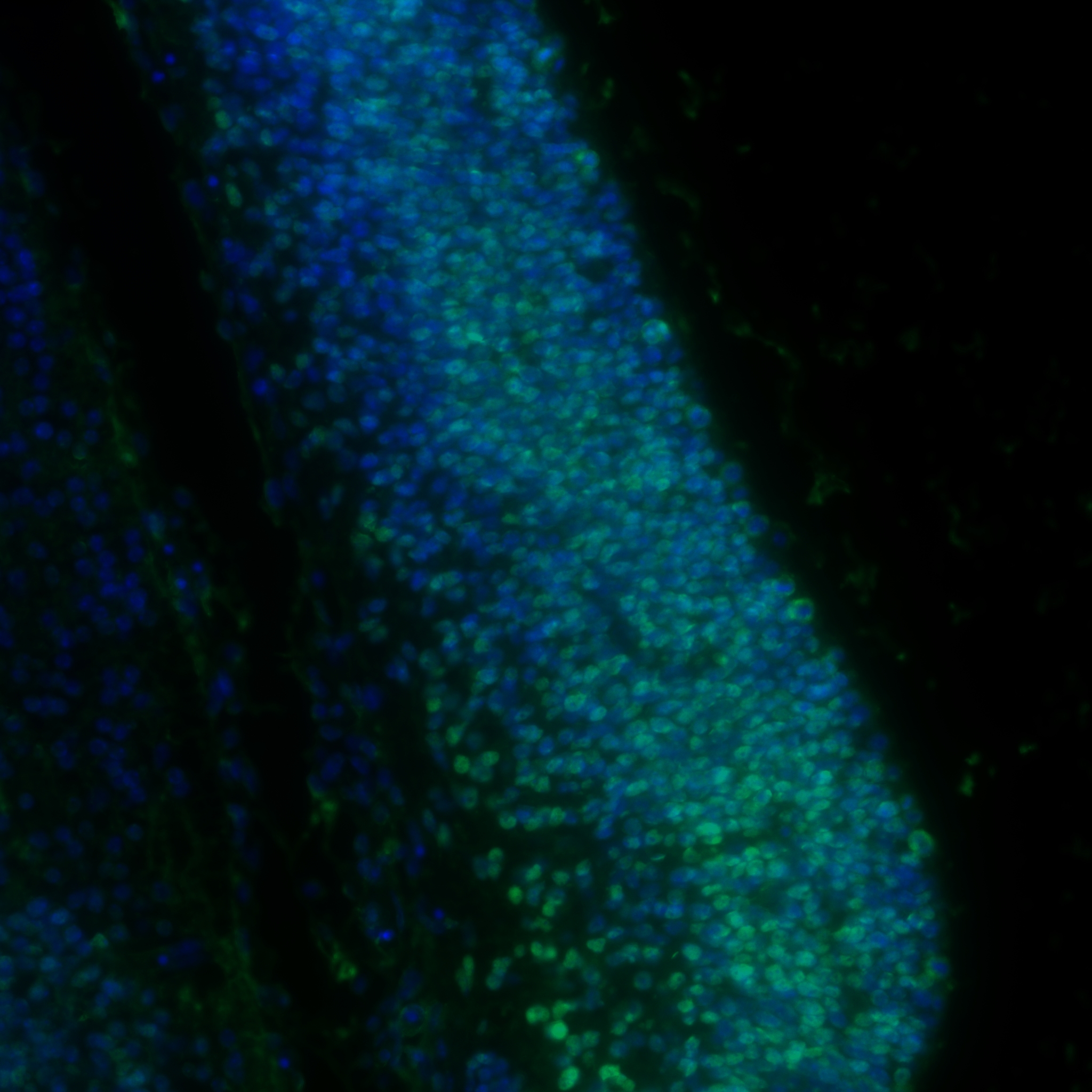

Supplement: Figure 5—source data 1. [file elife-86940-fig5-data1.zip › Figure 5-source data 1/F1189-4-CON-E14.5-RX F+ f+-40X-gLhx2-32-1-R-MP-Image Export-45.jpg]

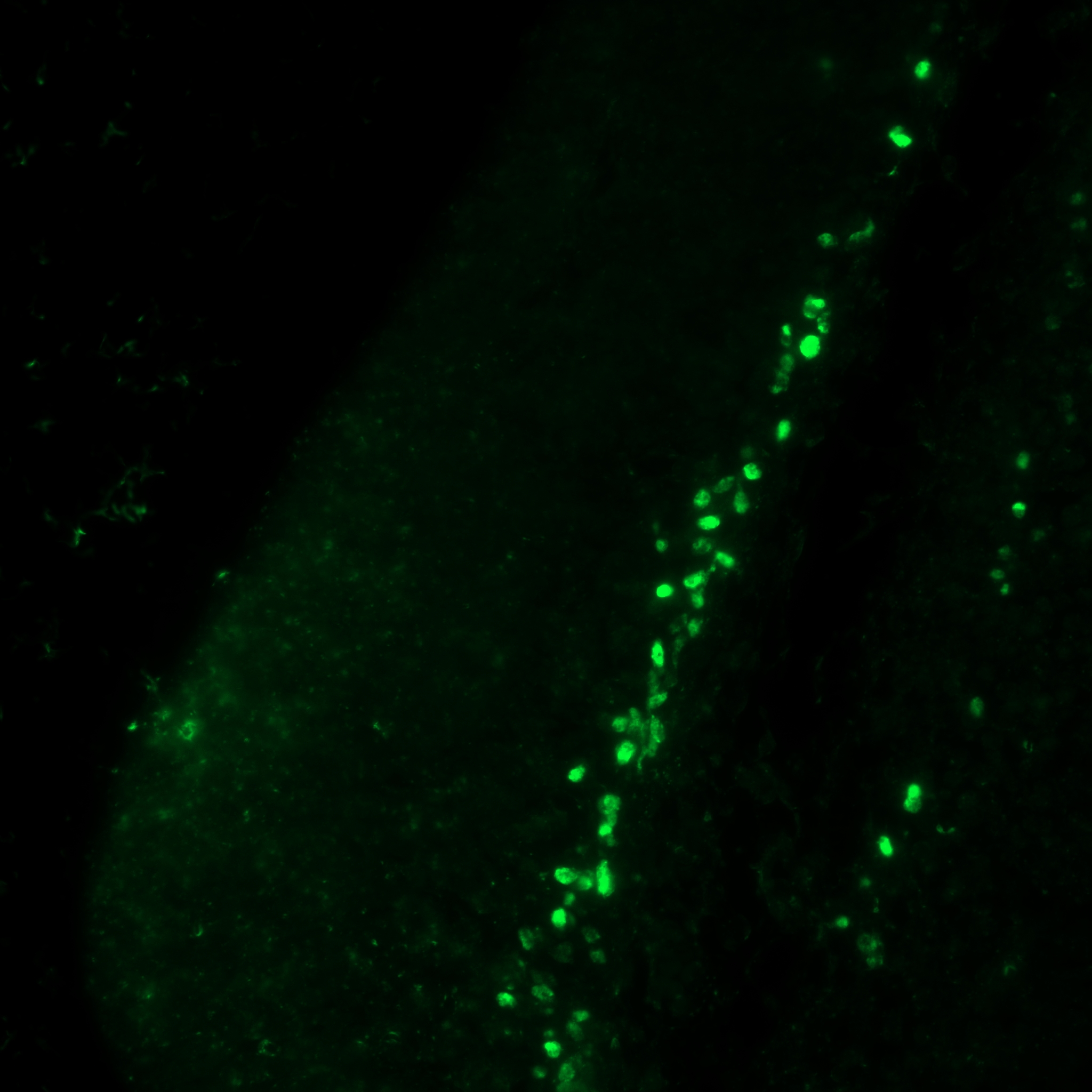

Supplement: Figure 5—source data 1. [file elife-86940-fig5-data1.zip › Figure 5-source data 1/F1189-4-CON-E14.5-RX F+ f+-40X-gLhx5-32-4-L-MP-Image Export-59_AF488.jpg]

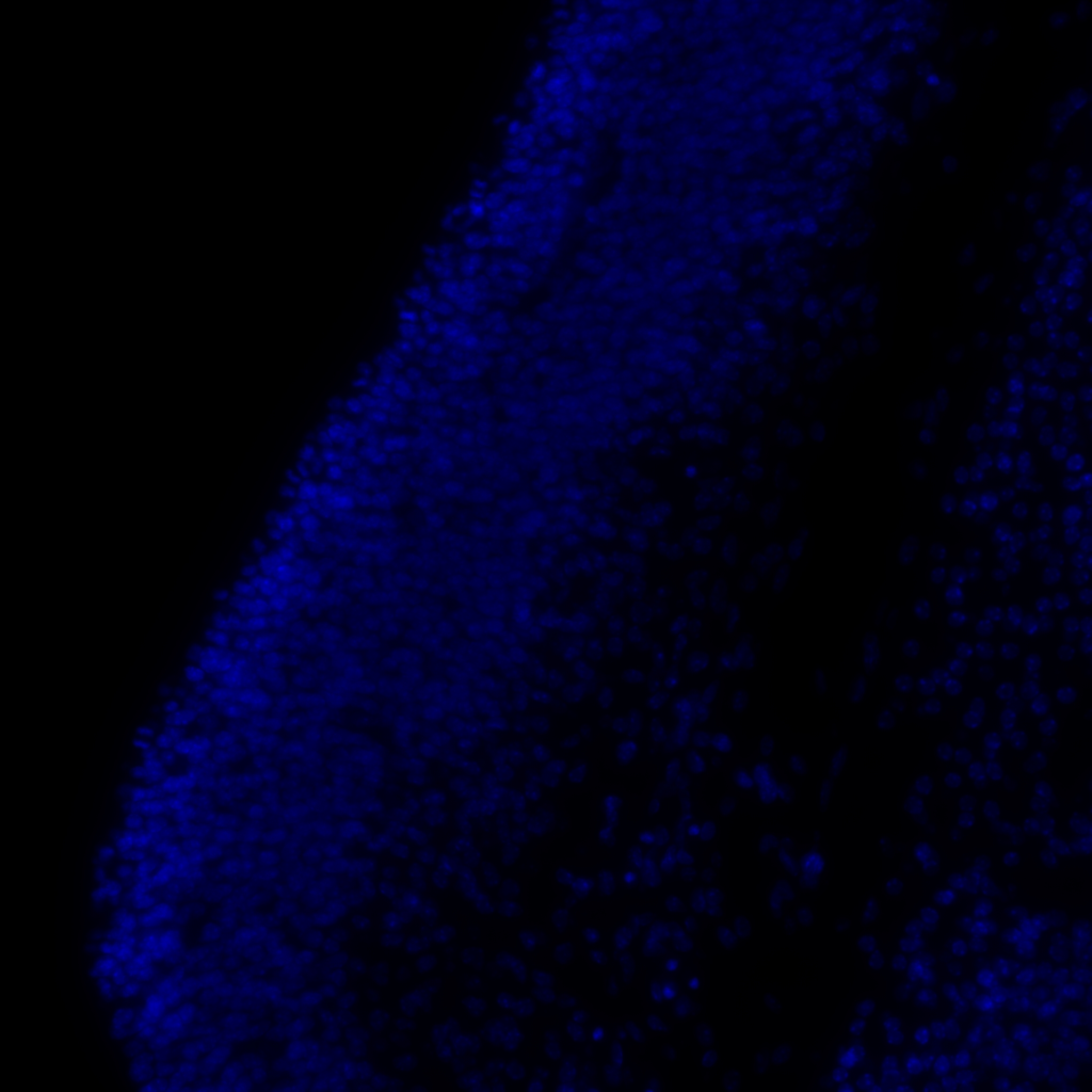

Supplement: Figure 5—source data 1. [file elife-86940-fig5-data1.zip › Figure 5-source data 1/F1189-4-CON-E14.5-RX F+ f+-40X-gLhx5-32-4-L-MP-Image Export-59_DAPI.jpg]

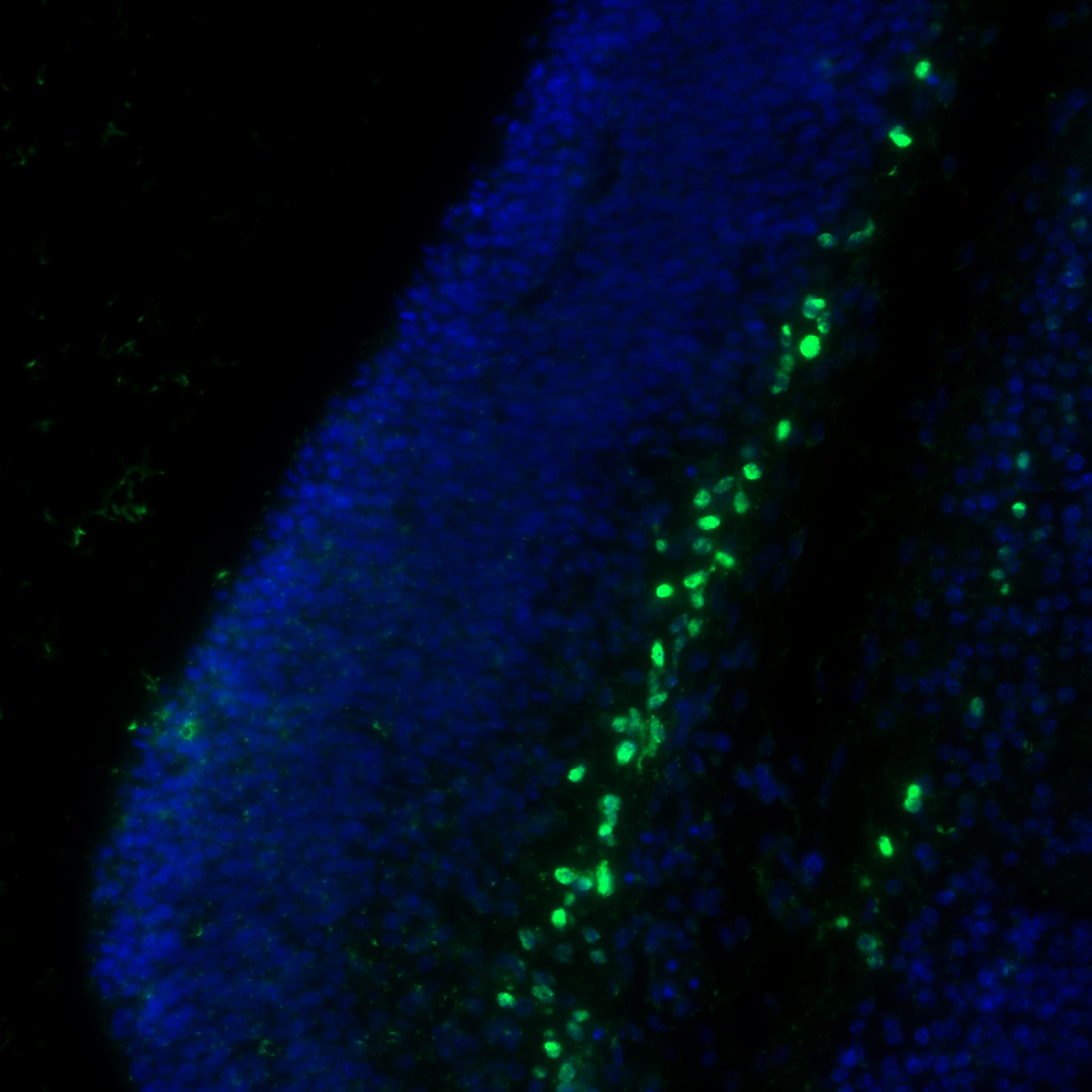

Supplement: Figure 5—source data 1. [file elife-86940-fig5-data1.zip › Figure 5-source data 1/F1189-4-CON-E14.5-RX F+ f+-40X-gLhx5-32-4-L-MP-Image Export-59.jpg]

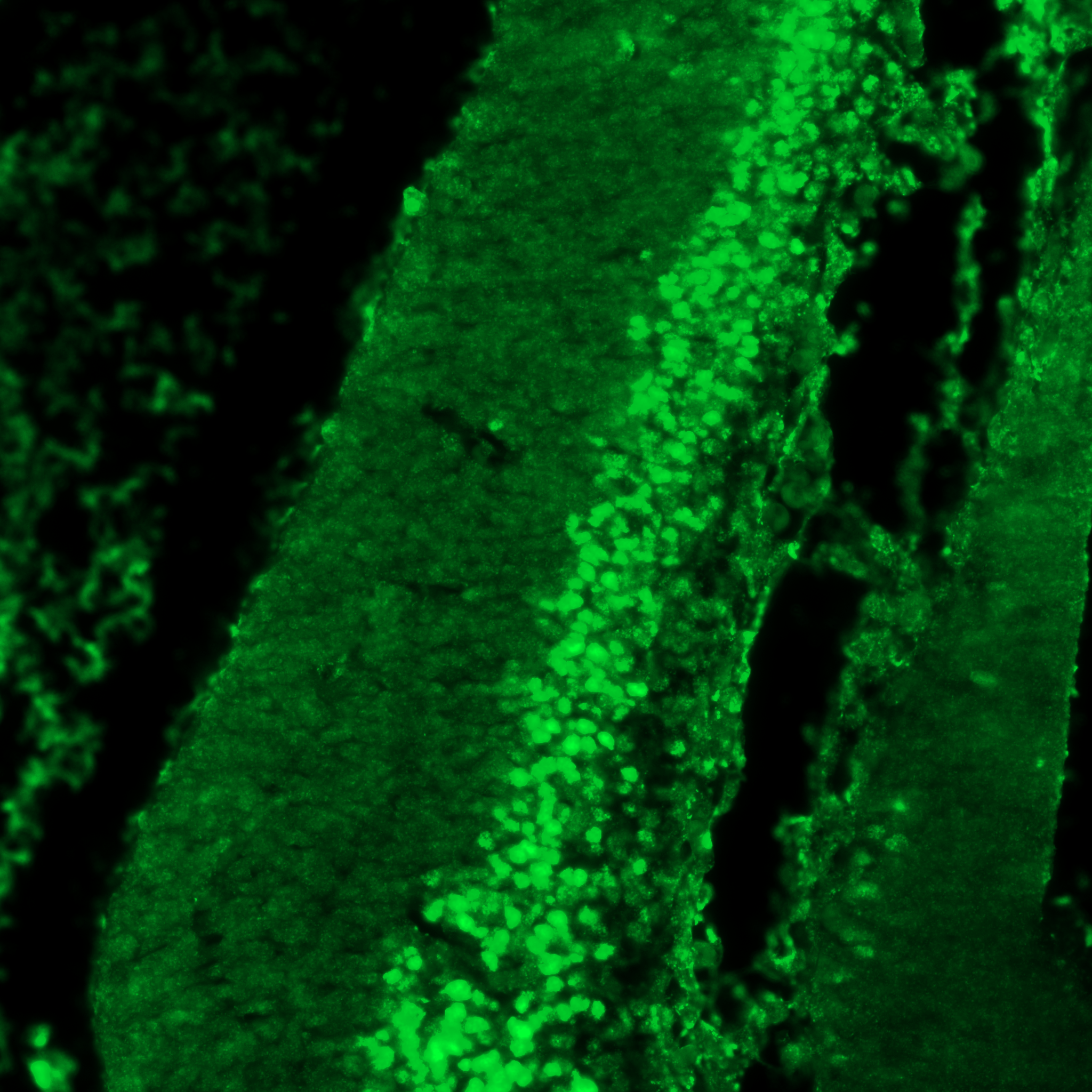

Supplement: Figure 5—source data 1. [file elife-86940-fig5-data1.zip › Figure 5-source data 1/F1189-4-E14.5-CON-f+ F+-38-2-40X-NEUROD1-L-Image Export-23_AF488.tif]

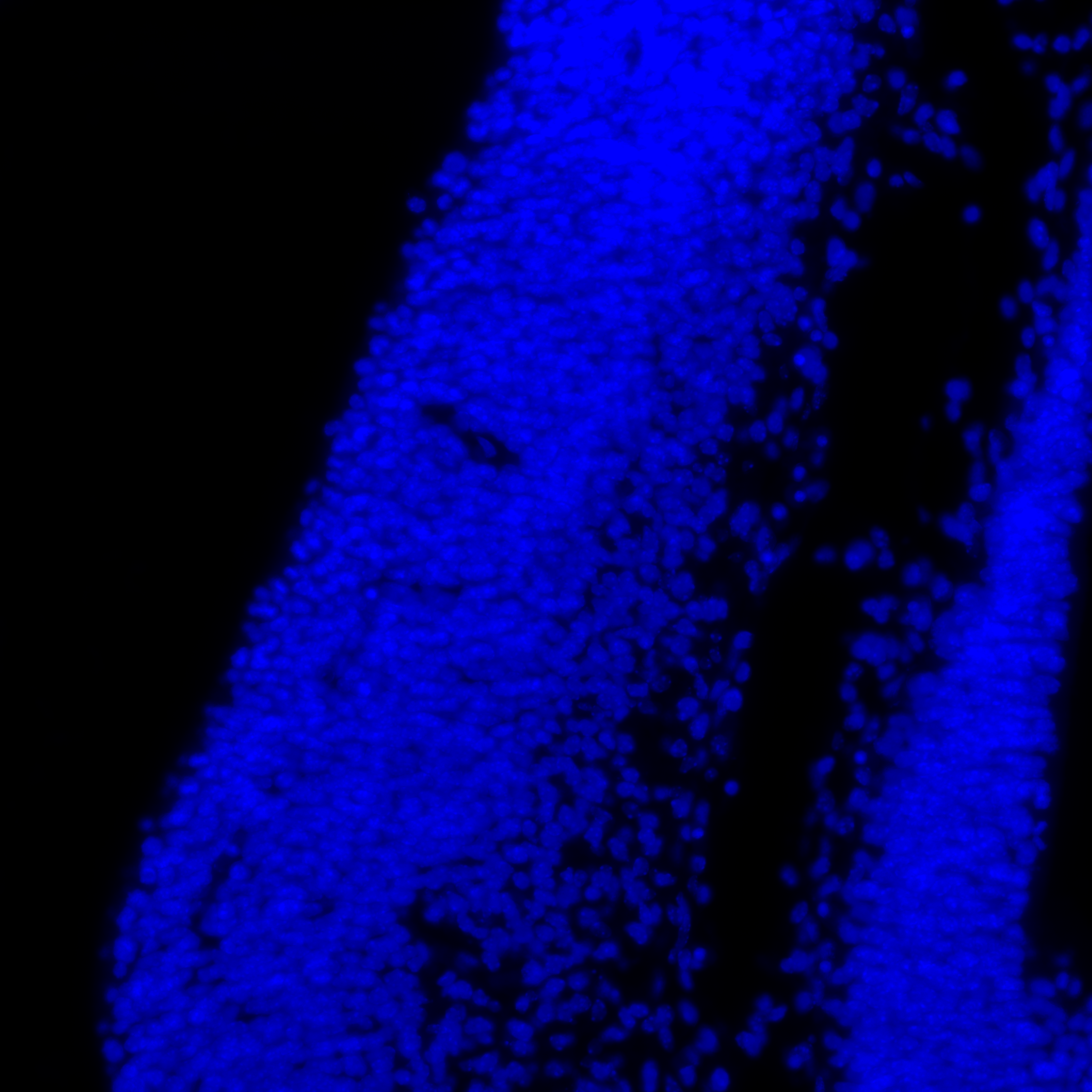

Supplement: Figure 5—source data 1. [file elife-86940-fig5-data1.zip › Figure 5-source data 1/F1189-4-E14.5-CON-f+ F+-38-2-40X-NEUROD1-L-Image Export-23_DAPI.tif]

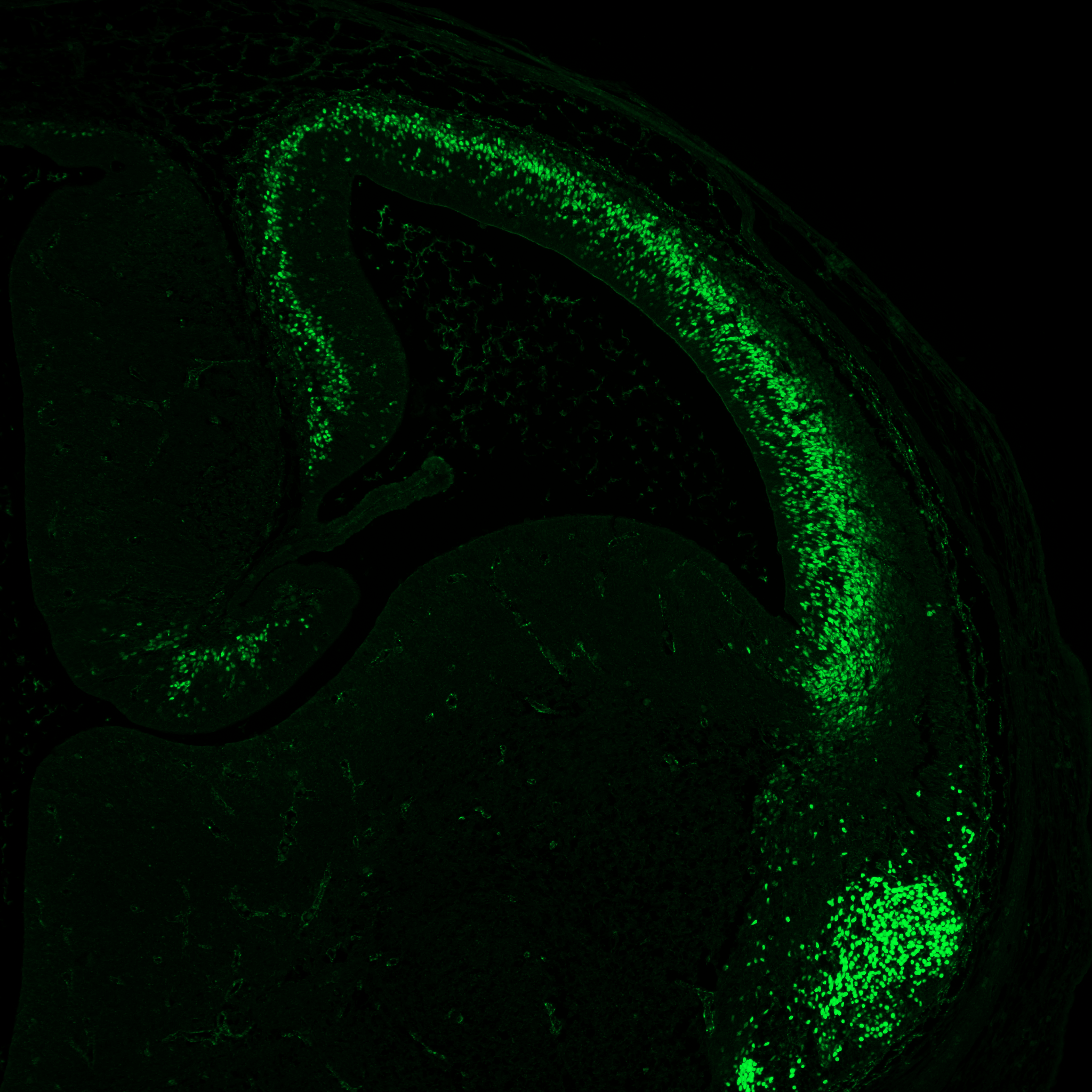

Supplement: Figure 5—source data 1. [file elife-86940-fig5-data1.zip › Figure 5-source data 1/F1189-7-E14.5-DKO-10X-RX FF ff-#30-TBR2-4-R-Image Export-17_AF488.tif]

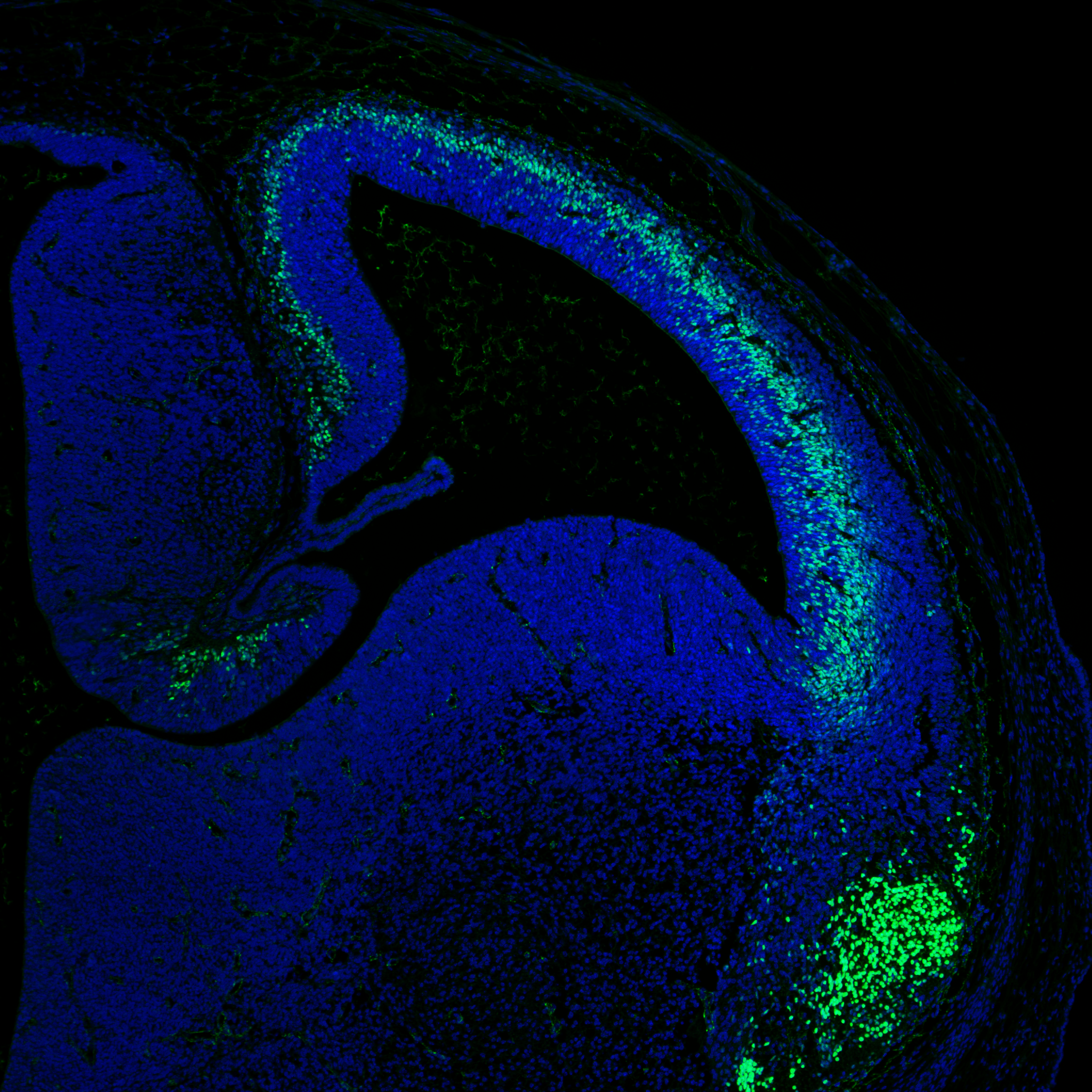

Supplement: Figure 5—source data 1. [file elife-86940-fig5-data1.zip › Figure 5-source data 1/F1189-7-E14.5-DKO-10X-RX FF ff-#30-TBR2-4-R-Image Export-17_G+D.tif]

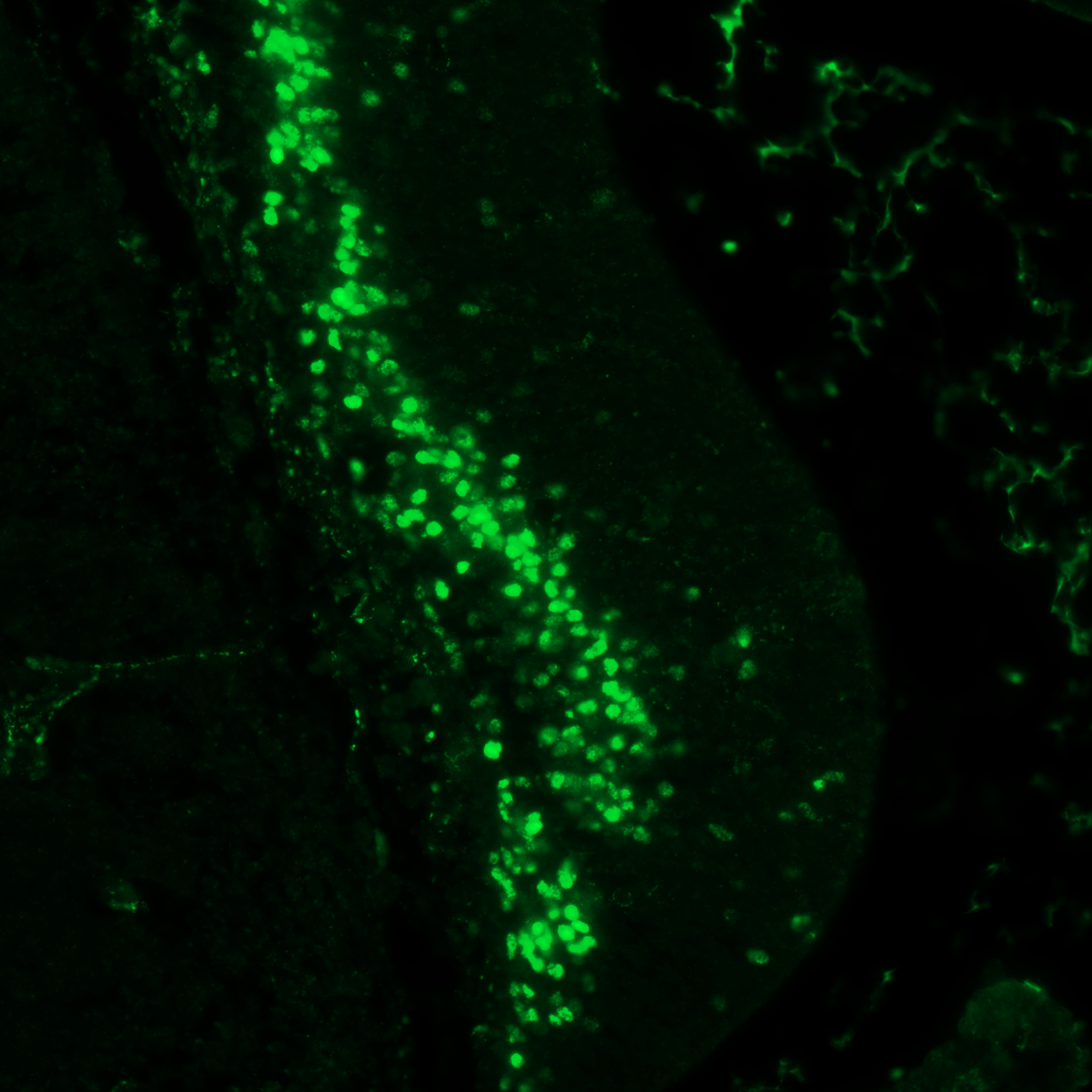

Supplement: Figure 5—source data 1. [file elife-86940-fig5-data1.zip › Figure 5-source data 1/F1189-7-E14.5-DKO-40X-RX FF ff-#30-TBR2-4-R-Image Export-20_AF488.tif]

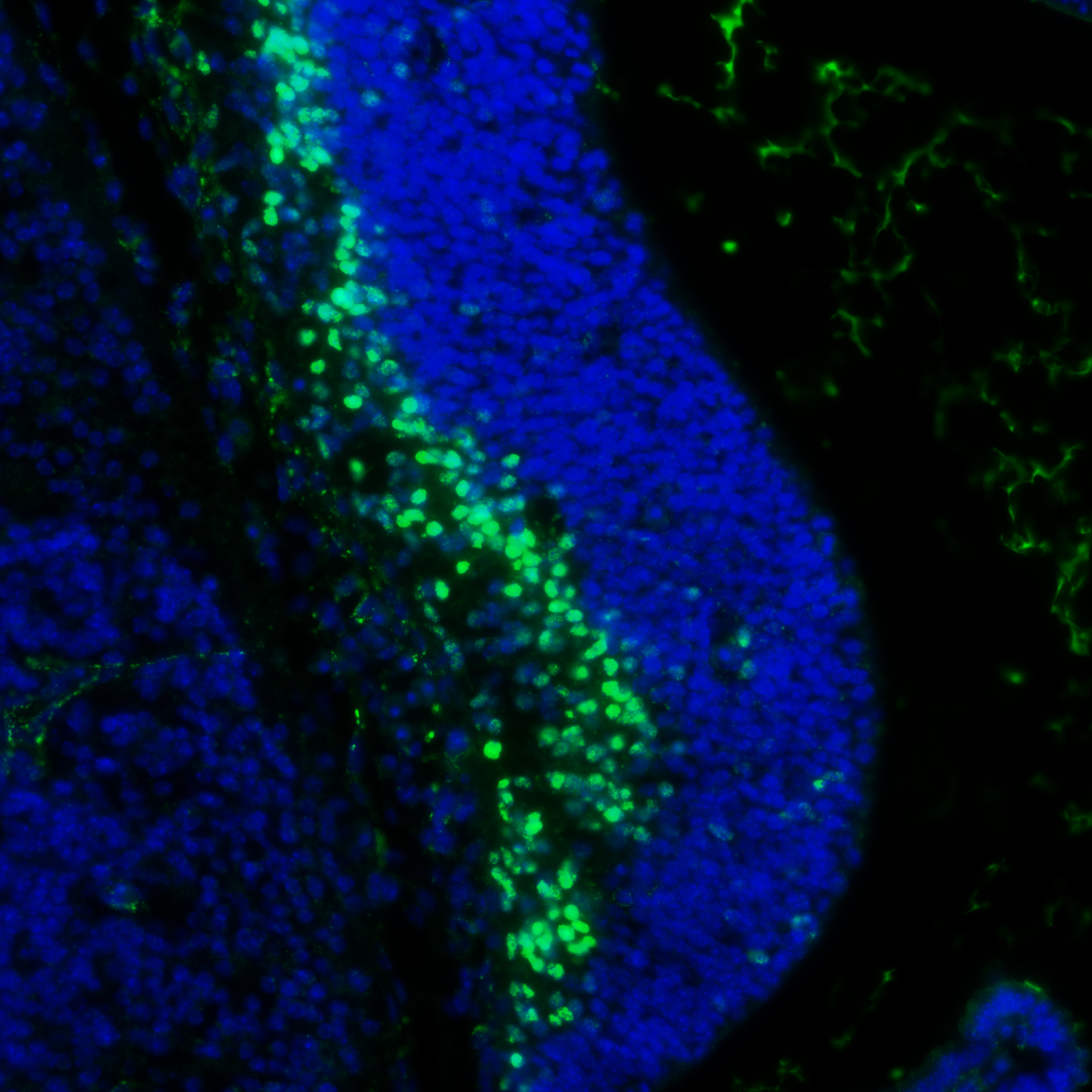

Supplement: Figure 5—source data 1. [file elife-86940-fig5-data1.zip › Figure 5-source data 1/F1189-7-E14.5-DKO-40X-RX FF ff-#30-TBR2-4-R-Image Export-20_G+D.tif]

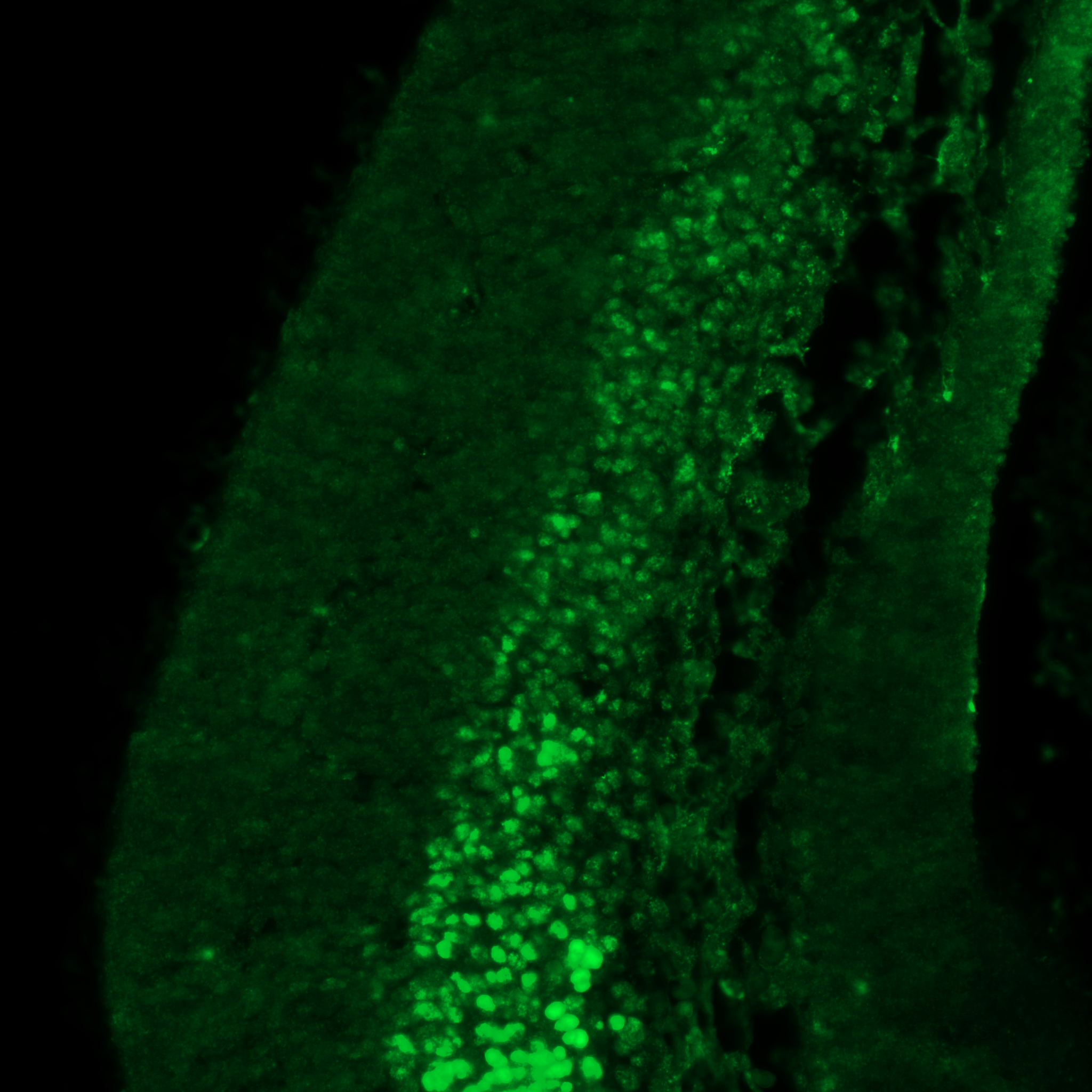

Supplement: Figure 5—source data 1. [file elife-86940-fig5-data1.zip › Figure 5-source data 1/F1189-7-E14.5-DKO-RX ff FF-36-1-40X-NEUROD1-L-Image Export-28_AF488.tif]

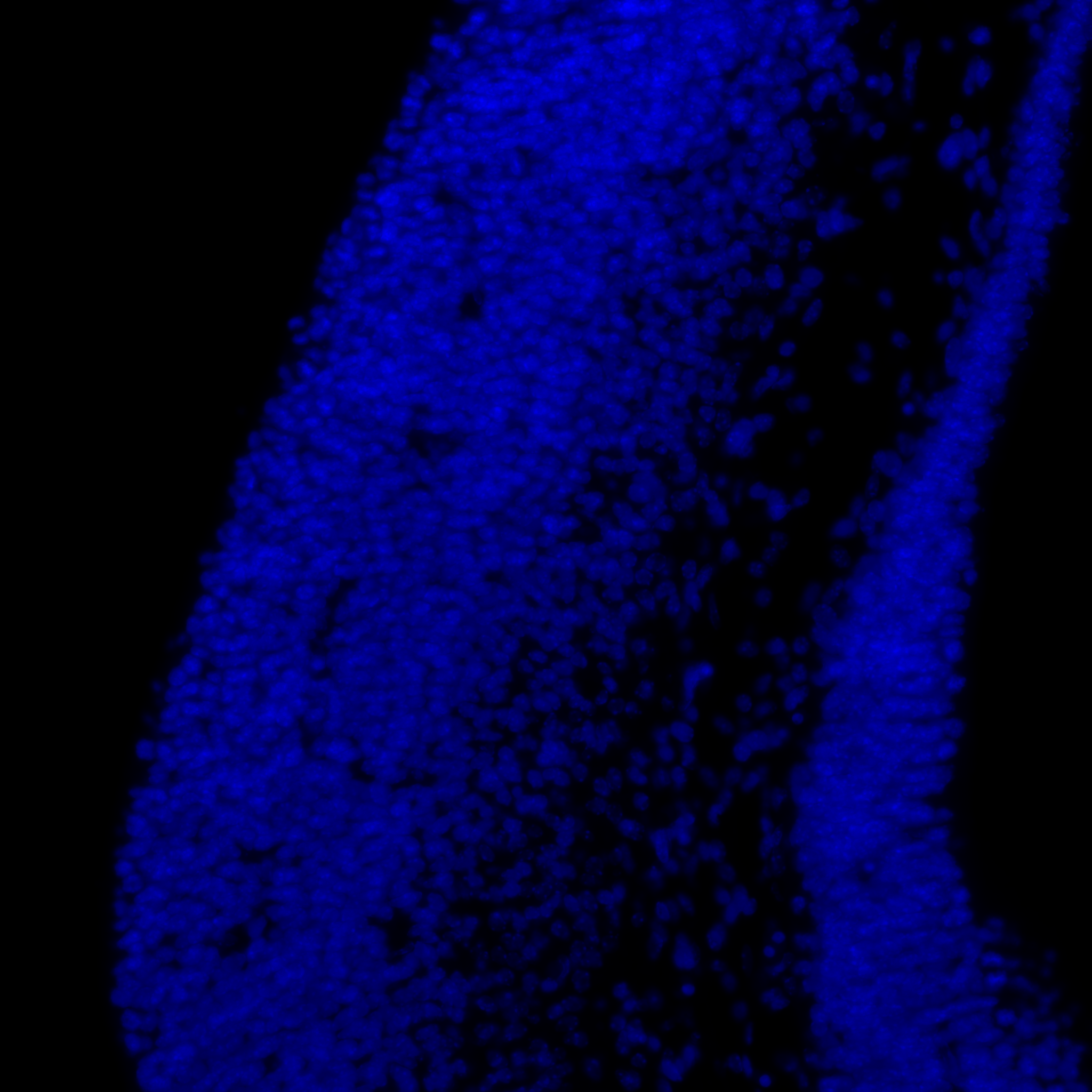

Supplement: Figure 5—source data 1. [file elife-86940-fig5-data1.zip › Figure 5-source data 1/F1189-7-E14.5-DKO-RX ff FF-36-1-40X-NEUROD1-L-Image Export-28_DAPI.tif]

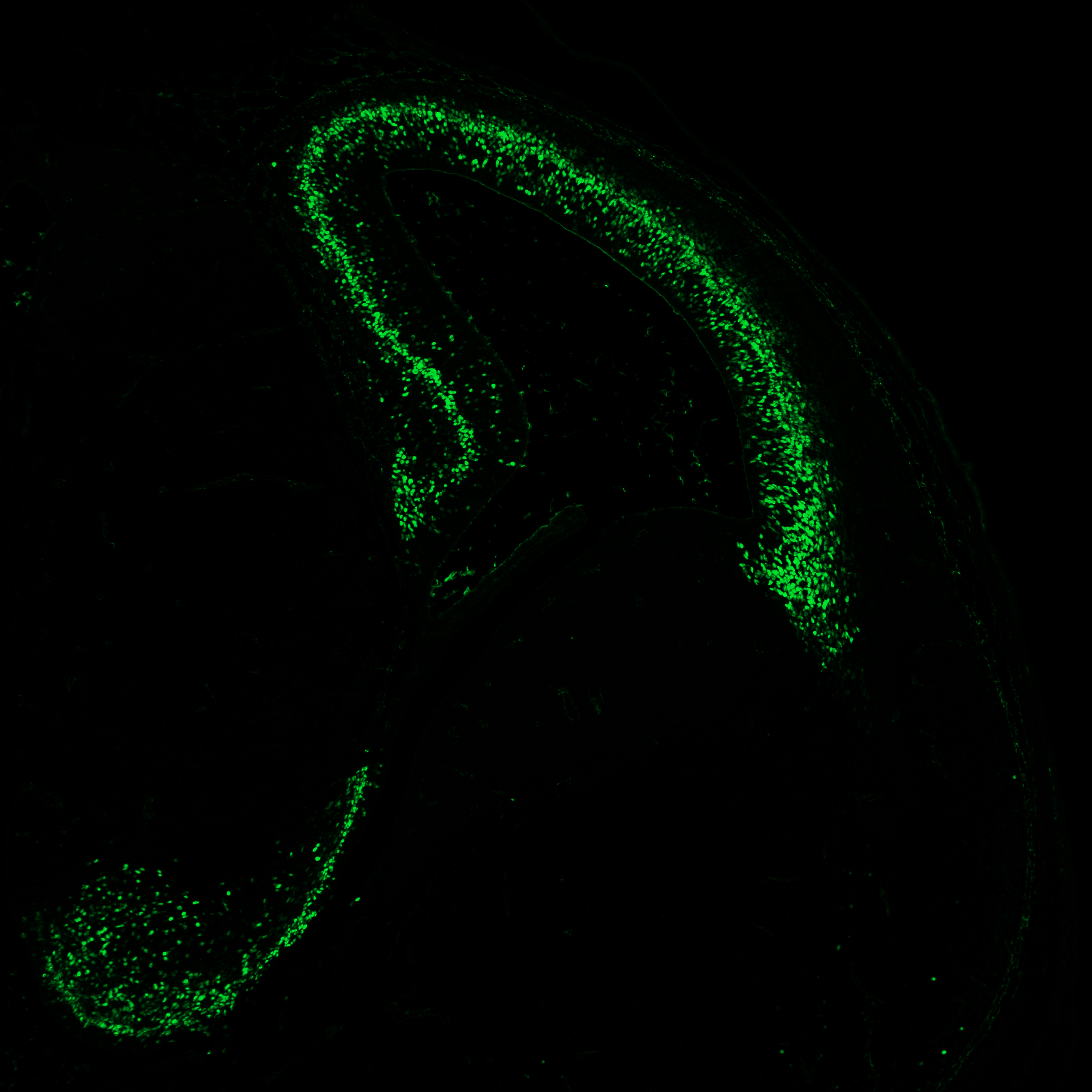

Supplement: Figure 5—source data 1. [file elife-86940-fig5-data1.zip › Figure 5-source data 1/F1189-8-E14.5-CON-10X-F+ ff-#32-TBR2-4-R-Image Export-03_AF488.tif]

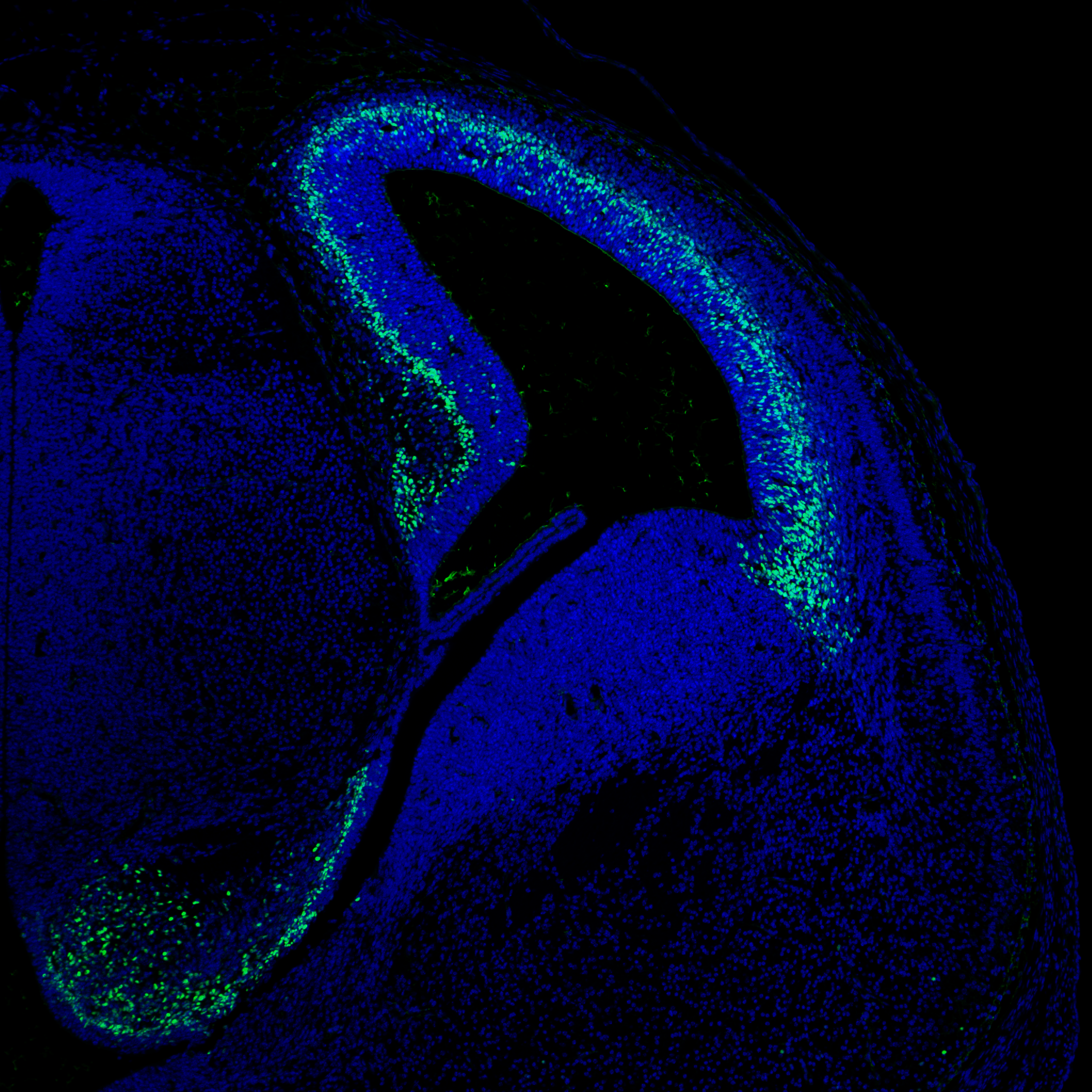

Supplement: Figure 5—source data 1. [file elife-86940-fig5-data1.zip › Figure 5-source data 1/F1189-8-E14.5-CON-10X-F+ ff-#32-TBR2-4-R-Image Export-03_G+D.tif]

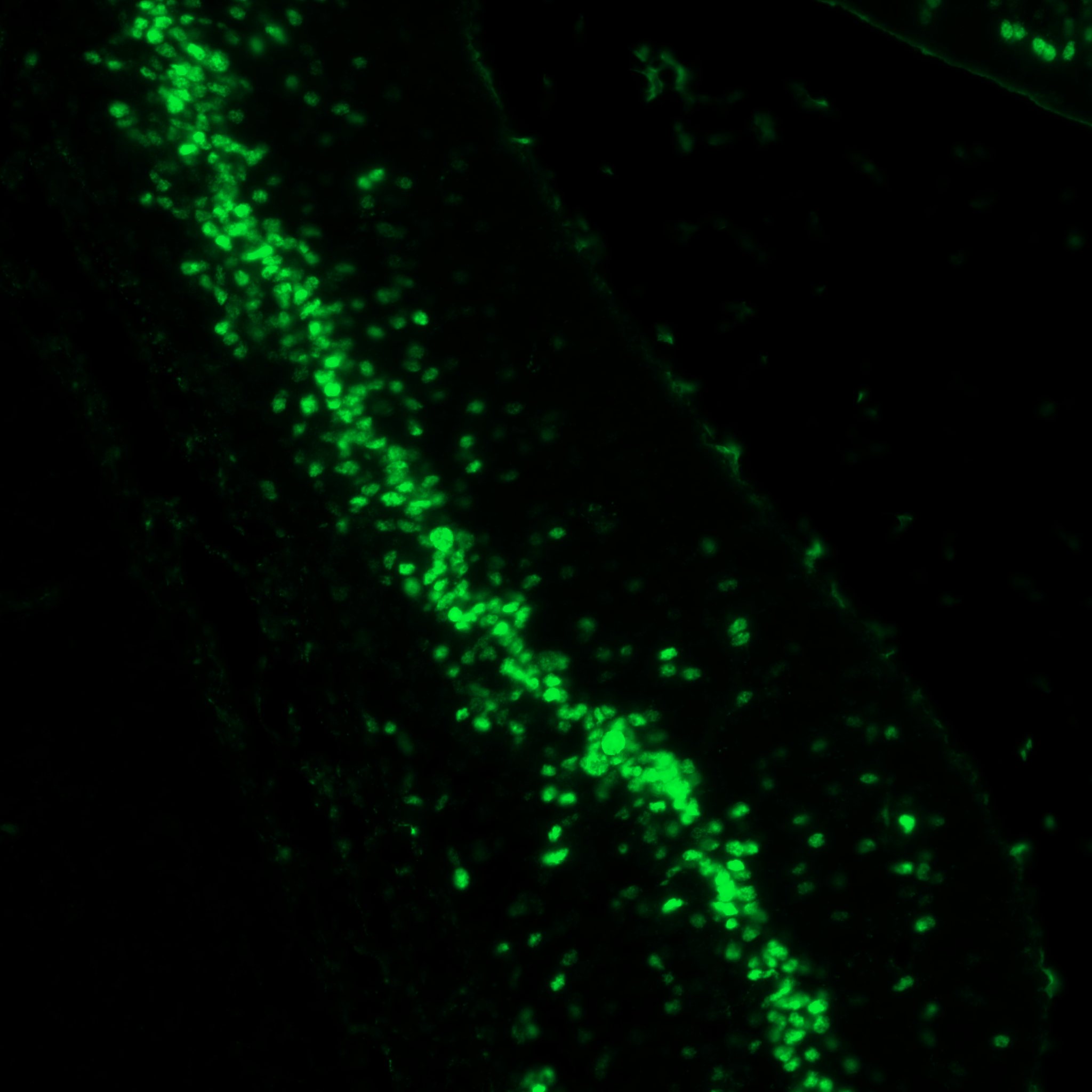

Supplement: Figure 5—source data 1. [file elife-86940-fig5-data1.zip › Figure 5-source data 1/F1189-8-E14.5-CON-40X-F+ ff-#32-TBR2-4-R-Image Export-07_AF488.tif]

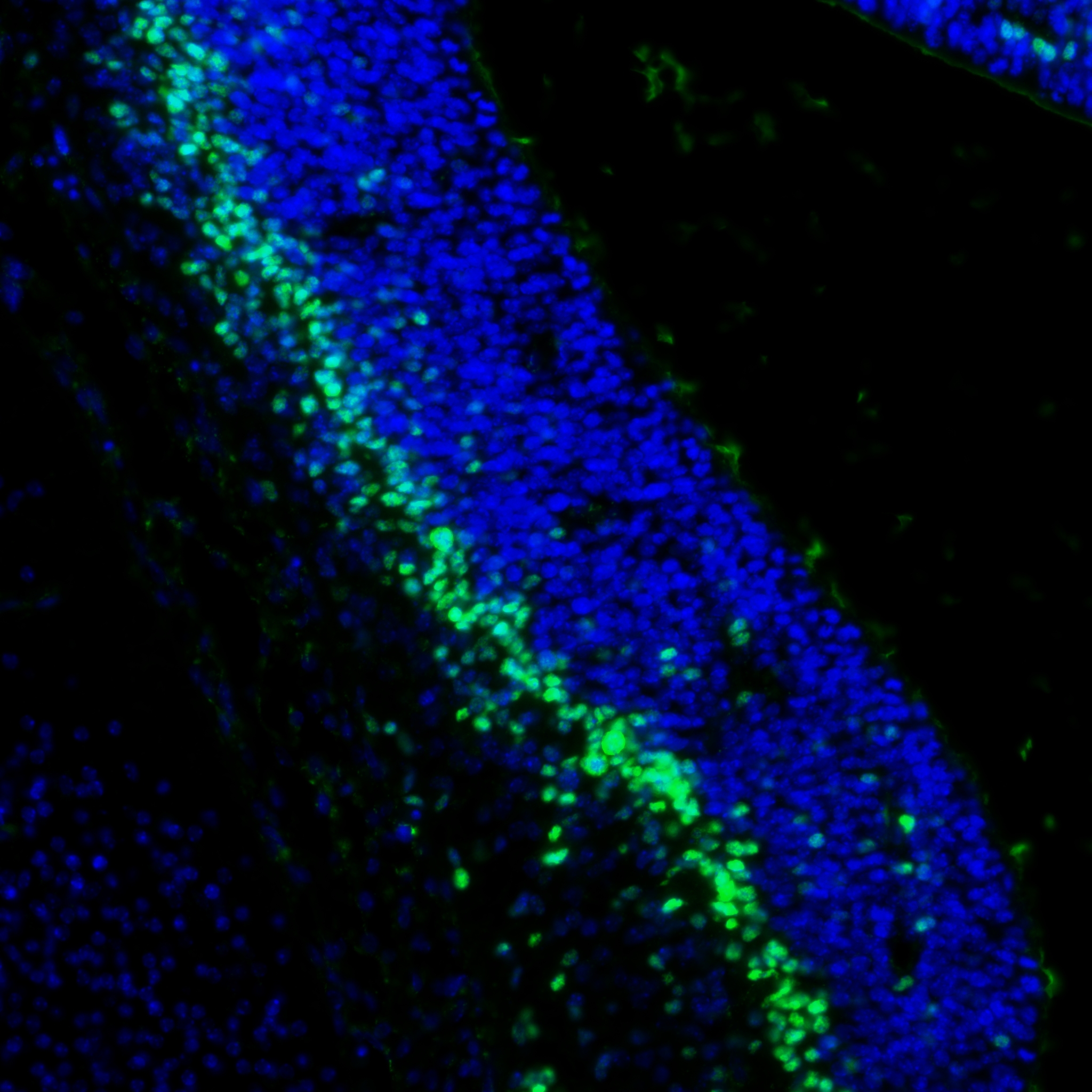

Supplement: Figure 5—source data 1. [file elife-86940-fig5-data1.zip › Figure 5-source data 1/F1189-8-E14.5-CON-40X-F+ ff-#32-TBR2-4-R-Image Export-07_G+D.tif]

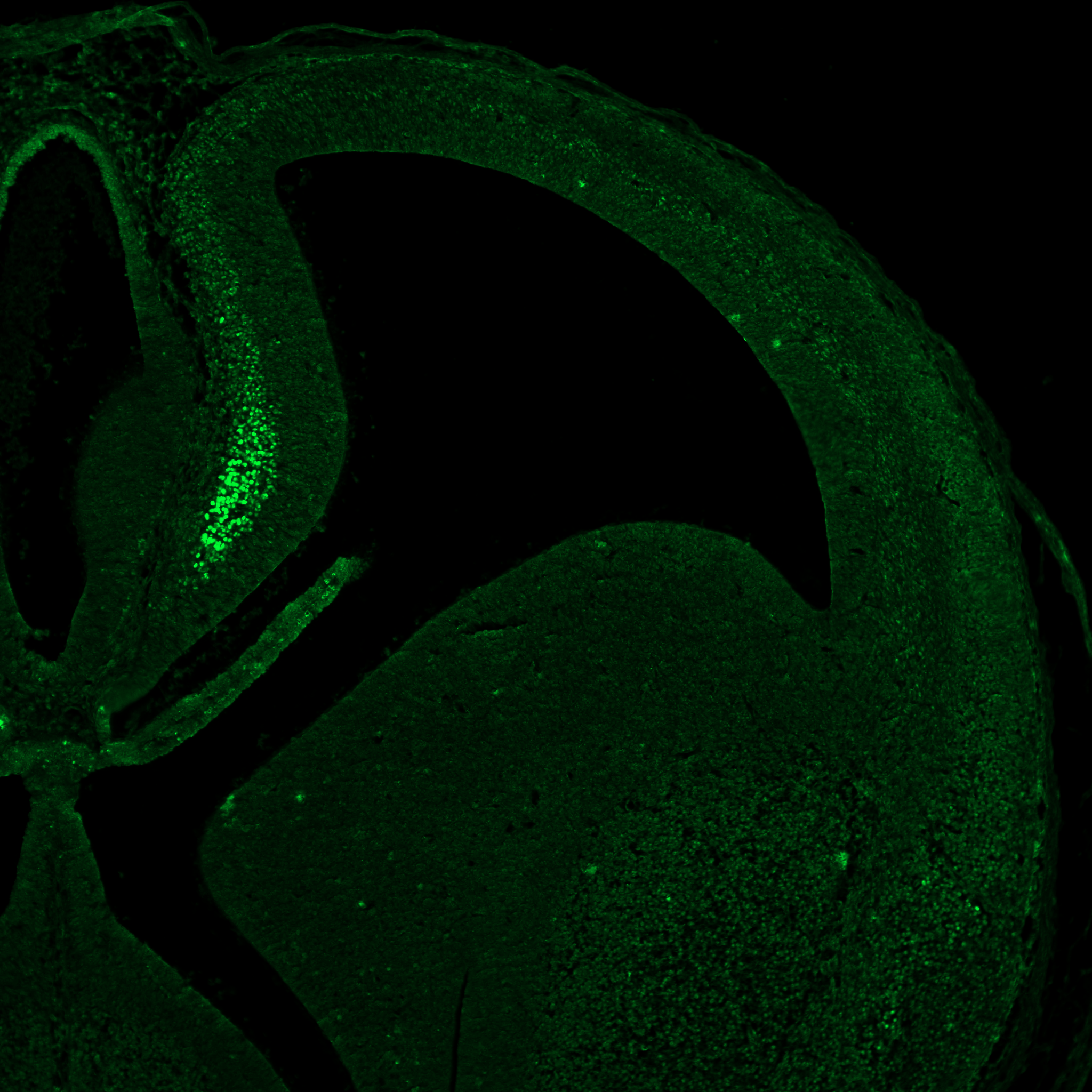

Supplement: Figure 5—source data 1. [file elife-86940-fig5-data1.zip › Figure 5-source data 1/F2116-1-E14.5-DKO-RX ff FF-18#-3-10X-NEUROD1-G-Image Export-38_AF488.tif]

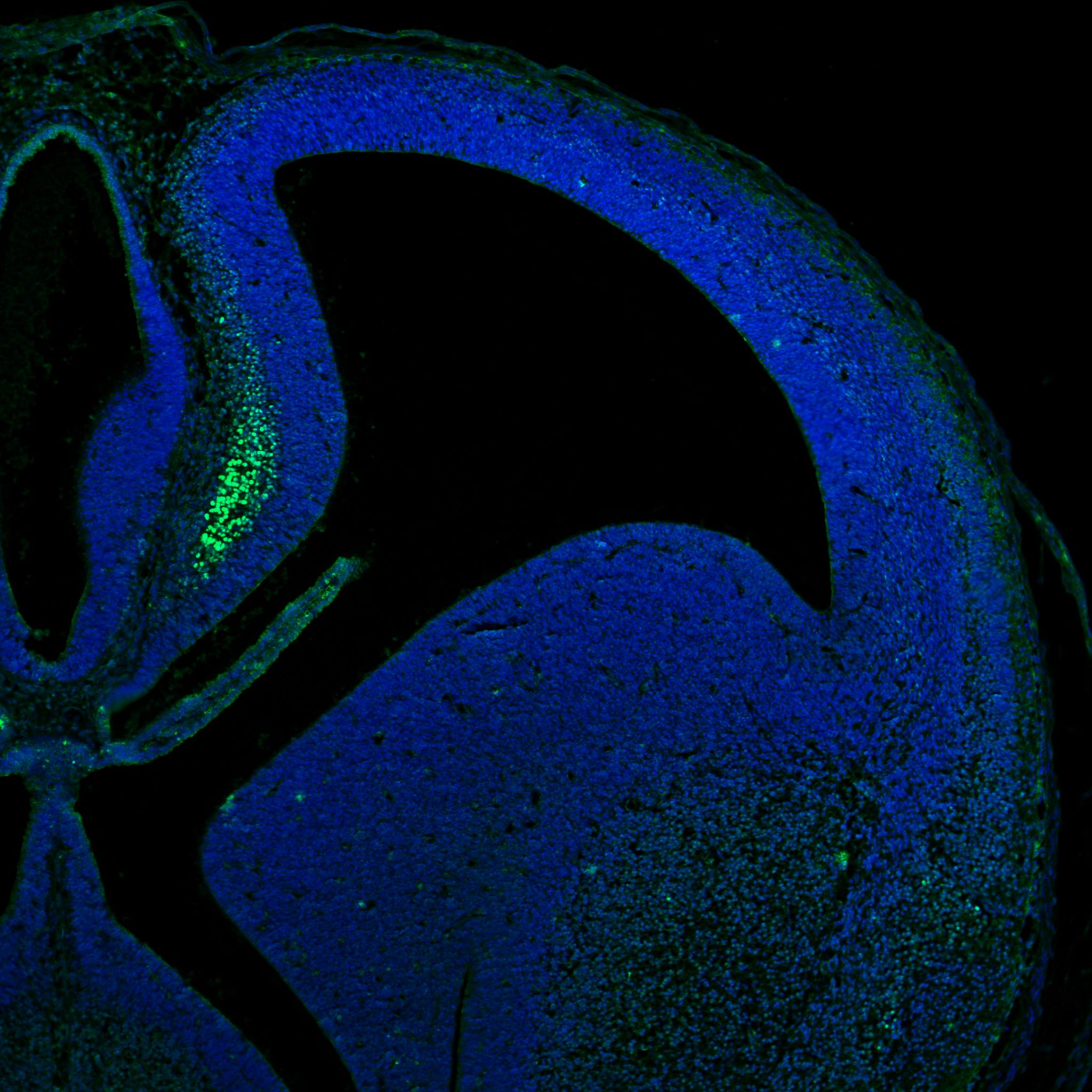

Supplement: Figure 5—source data 1. [file elife-86940-fig5-data1.zip › Figure 5-source data 1/F2116-1-E14.5-DKO-RX ff FF-18#-3-10X-NEUROD1-G+D-Image Export-38.tif]

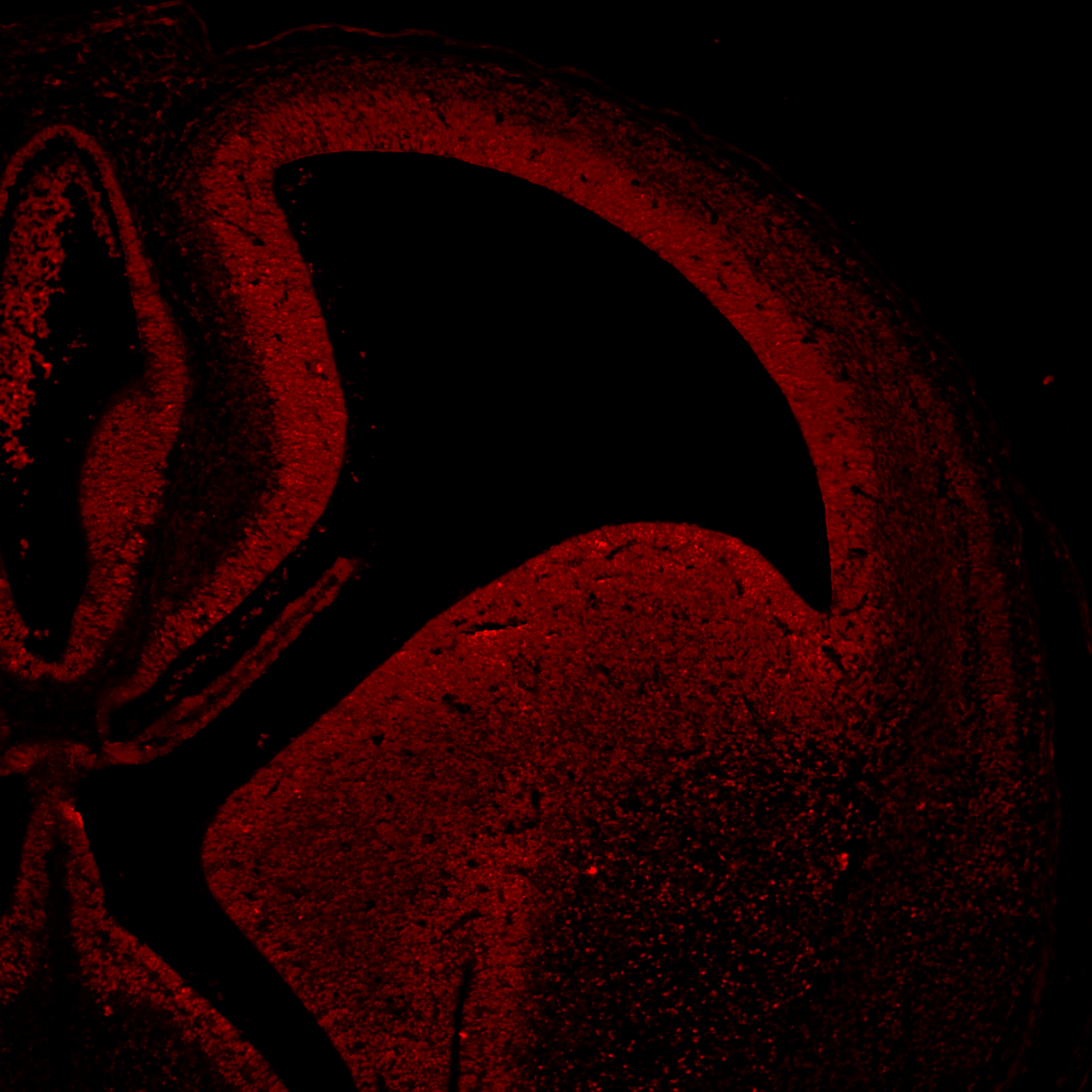

Supplement: Figure 5—source data 2. [file elife-86940-fig5-data2.zip › Figure 5-source data 2/F2116-1-E14.5-DKO-RX ff FF-18#-3-10X-SOX2-R-Image Export-39_AF594.tif]

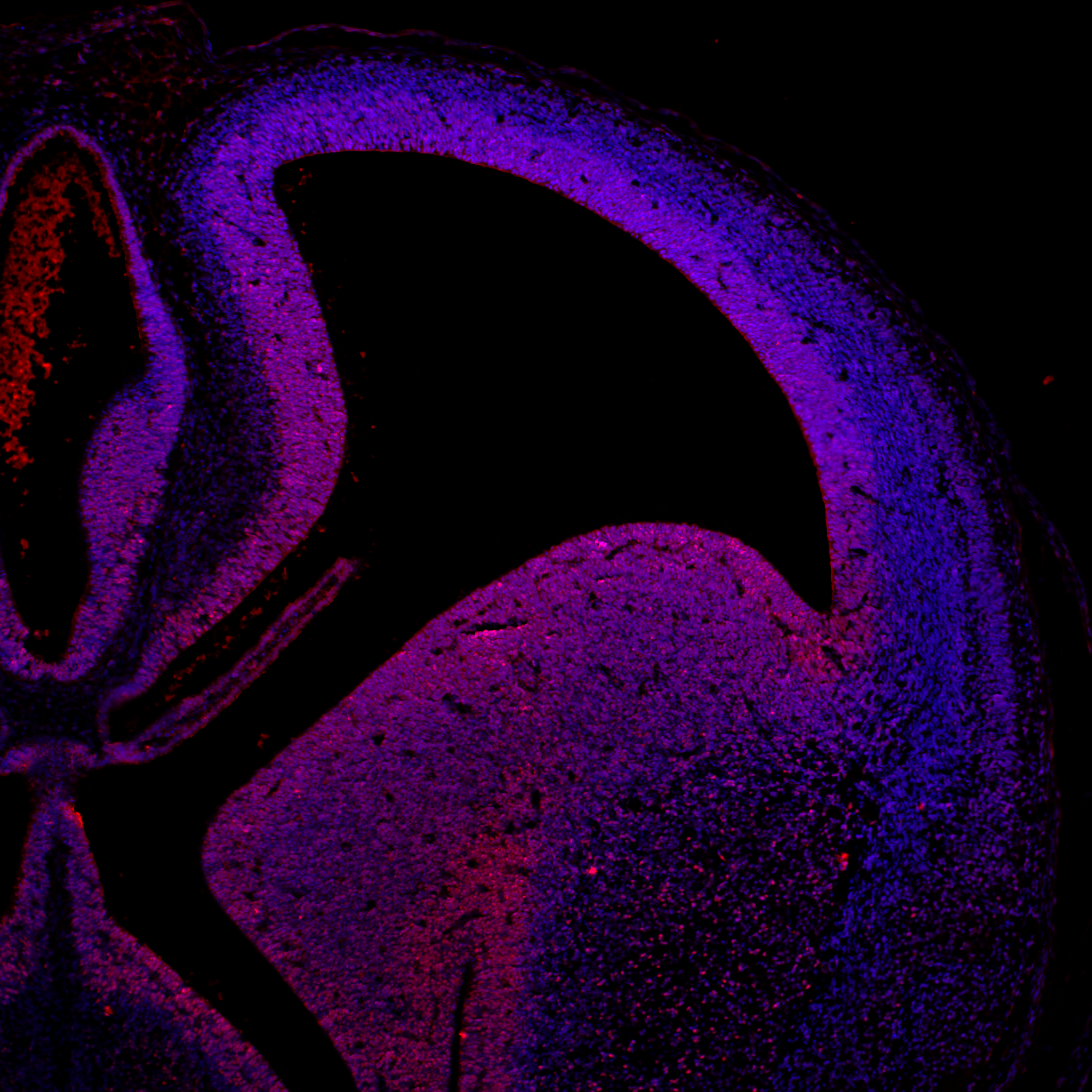

Supplement: Figure 5—source data 2. [file elife-86940-fig5-data2.zip › Figure 5-source data 2/F2116-1-E14.5-DKO-RX ff FF-18#-3-10X-SOX2-R+D-Image Export-39.tif]

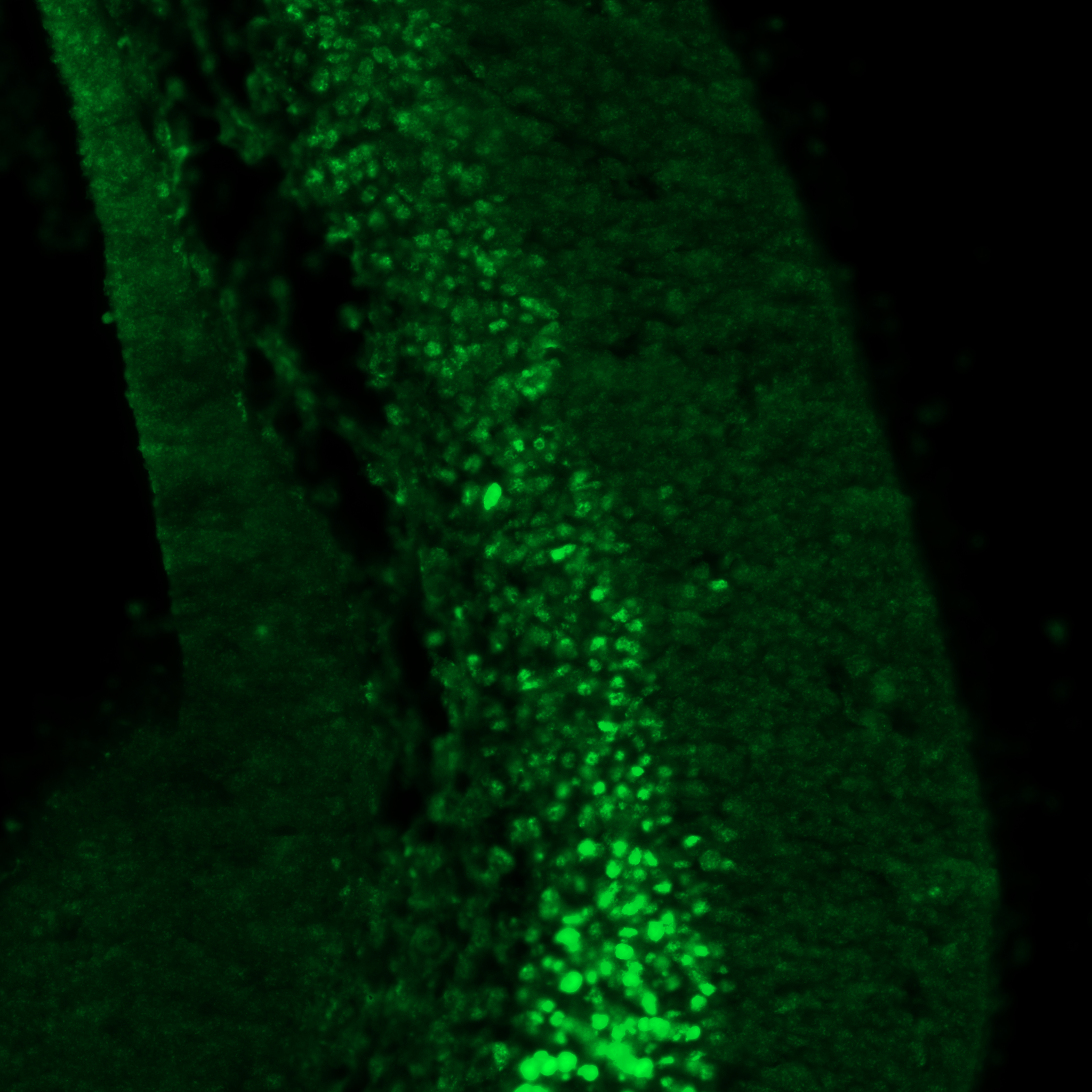

Supplement: Figure 5—source data 2. [file elife-86940-fig5-data2.zip › Figure 5-source data 2/F2116-1-E14.5-DKO-RX ff FF-18#-3-40X-NEUROD1-G-Image Export-44_AF488.tif]

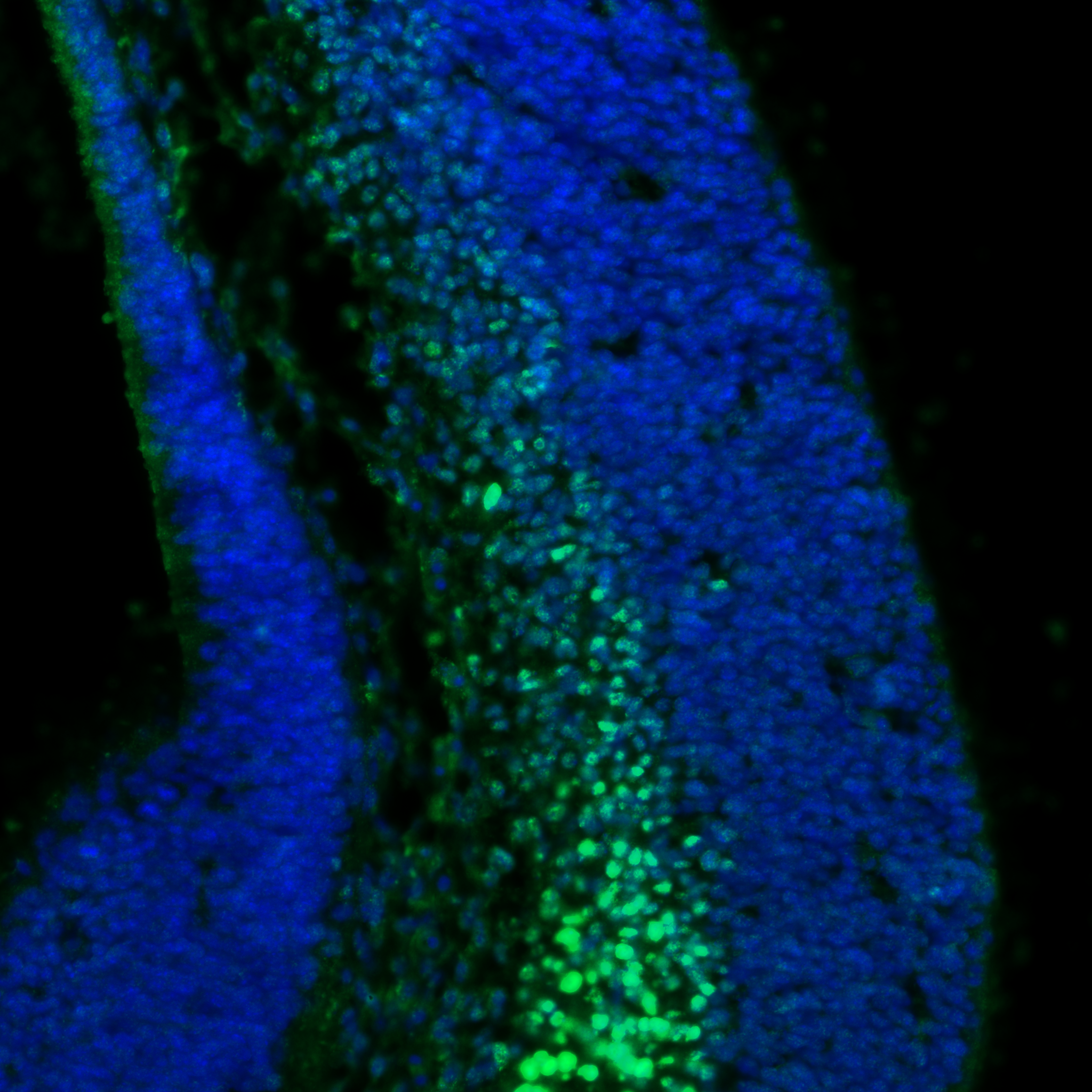

Supplement: Figure 5—source data 2. [file elife-86940-fig5-data2.zip › Figure 5-source data 2/F2116-1-E14.5-DKO-RX ff FF-18#-3-40X-NEUROD1-G+D-Image Export-44.tif]

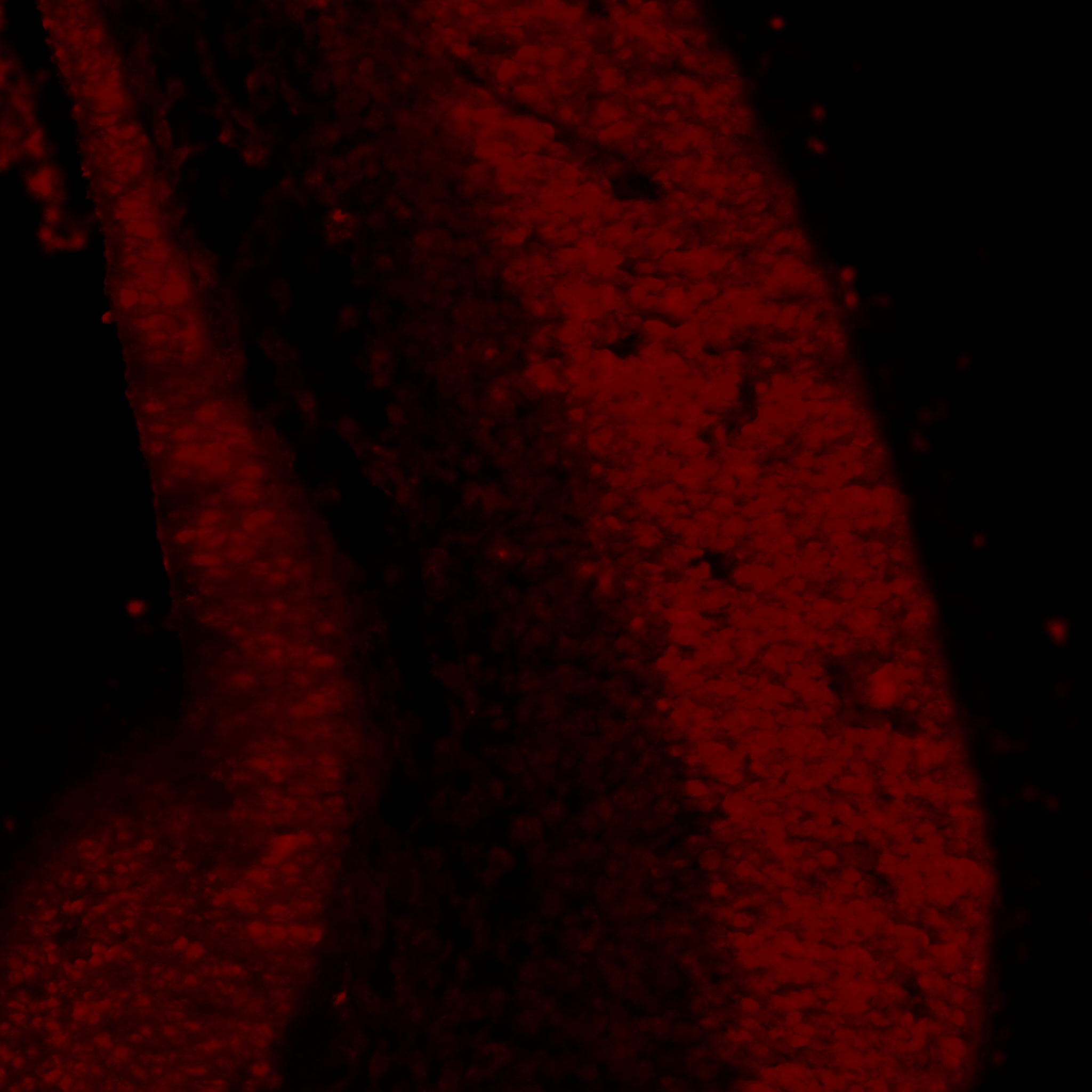

Supplement: Figure 5—source data 2. [file elife-86940-fig5-data2.zip › Figure 5-source data 2/F2116-1-E14.5-DKO-RX ff FF-18#-3-40X-SOX2-R-Image Export-45_AF594.tif]

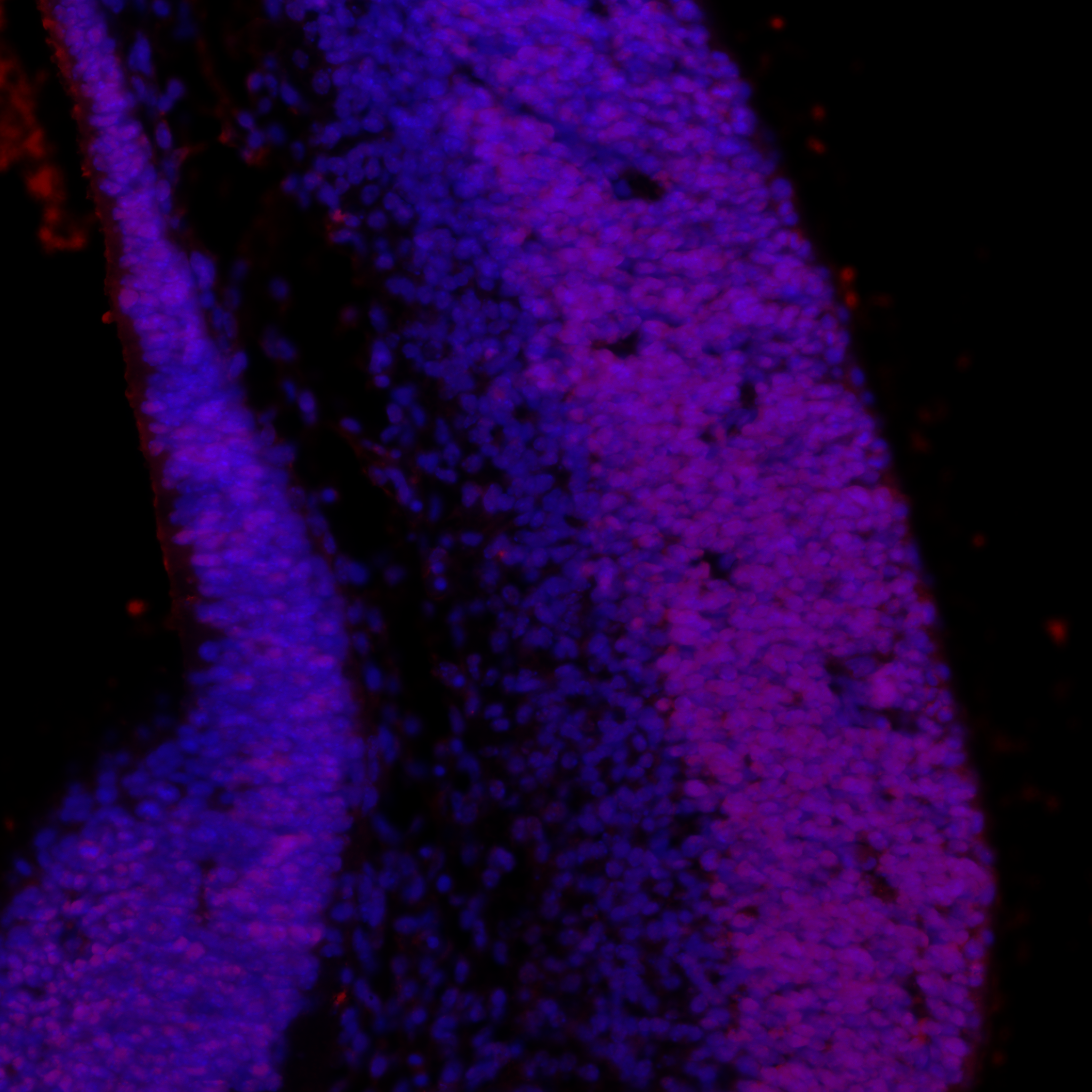

Supplement: Figure 5—source data 2. [file elife-86940-fig5-data2.zip › Figure 5-source data 2/F2116-1-E14.5-DKO-RX ff FF-18#-3-40X-SOX2-R+D-Image Export-45.tif]

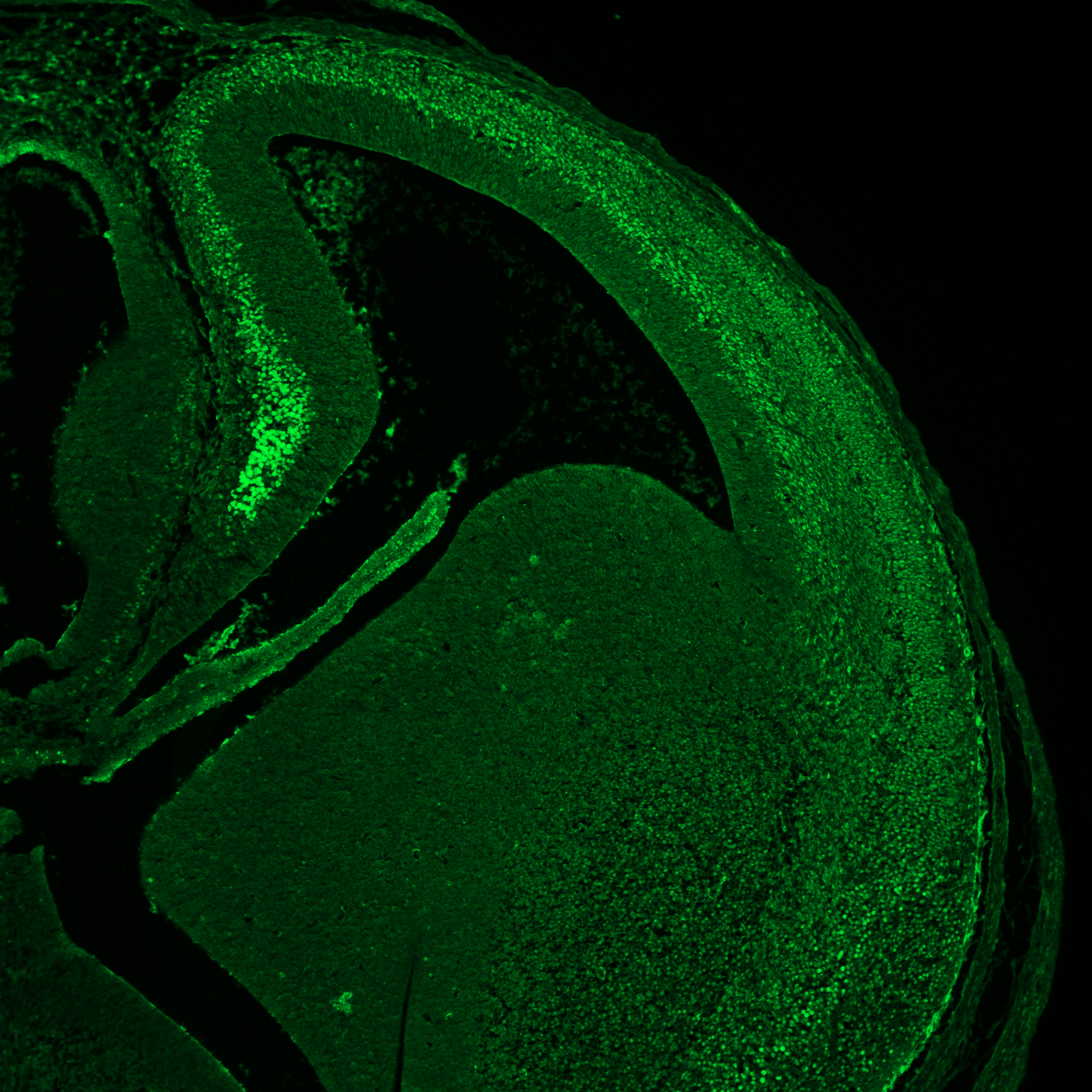

Supplement: Figure 5—source data 2. [file elife-86940-fig5-data2.zip › Figure 5-source data 2/F2116-6-E14.5-CON-RX f+ F+-115#-3-10X-NEUROD1-G-Image Export-38_AF488.tif]

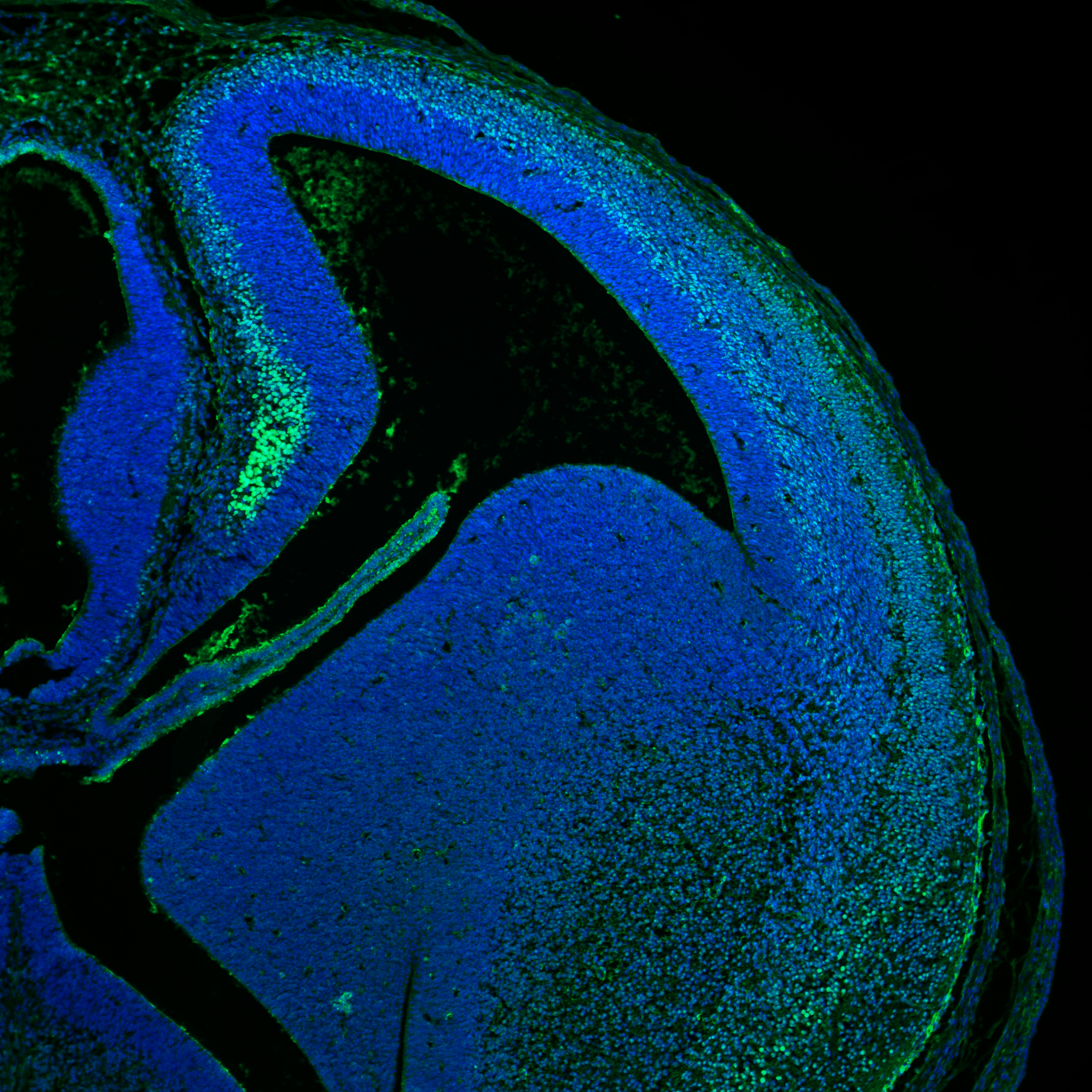

Supplement: Figure 5—source data 2. [file elife-86940-fig5-data2.zip › Figure 5-source data 2/F2116-6-E14.5-CON-RX f+ F+-115#-3-10X-NEUROD1-G+D-Image Export-38.tif]

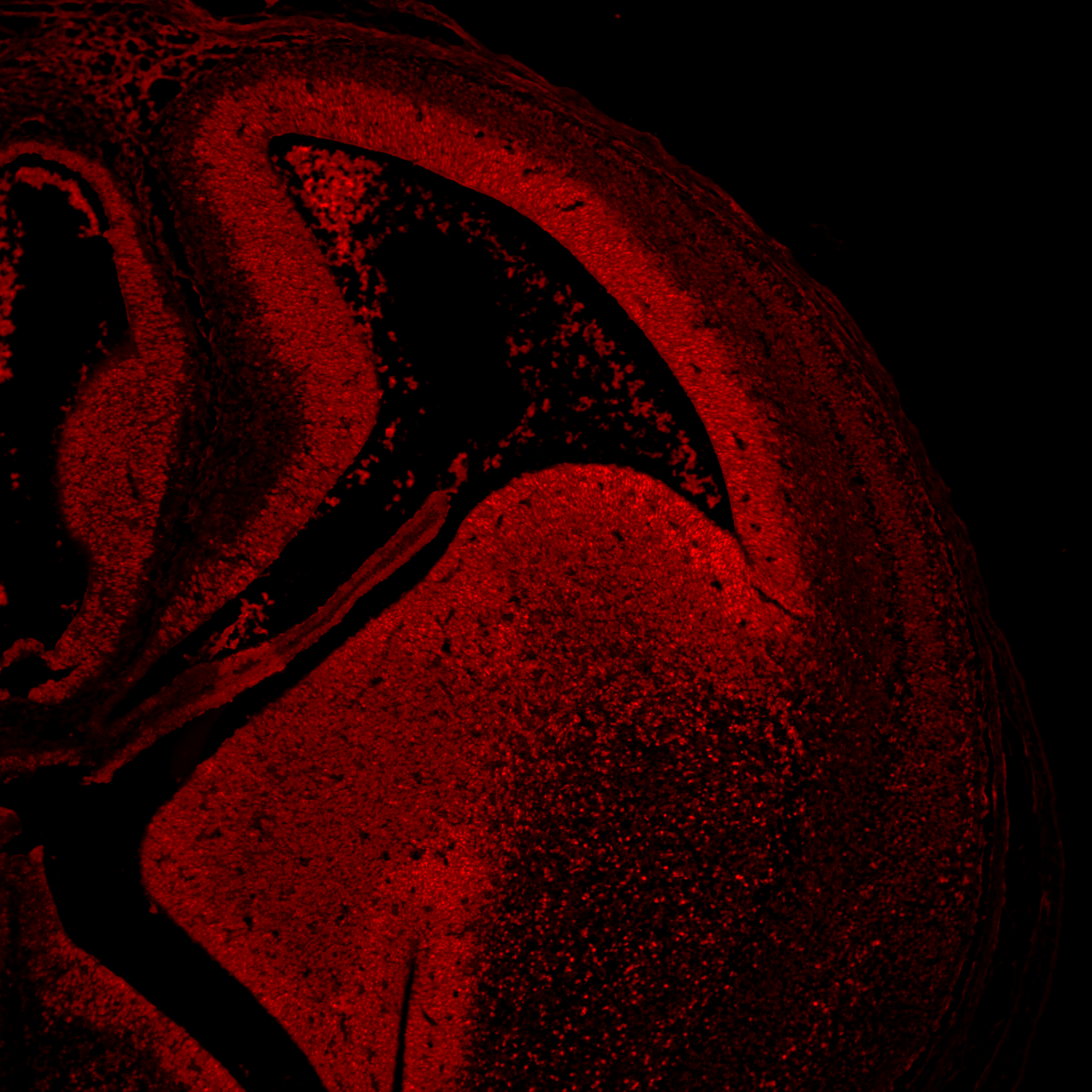

Supplement: Figure 5—source data 2. [file elife-86940-fig5-data2.zip › Figure 5-source data 2/F2116-6-E14.5-CON-RX f+ F+-115#-3-10X-SOX2-R-Image Export-39_AF594.tif]

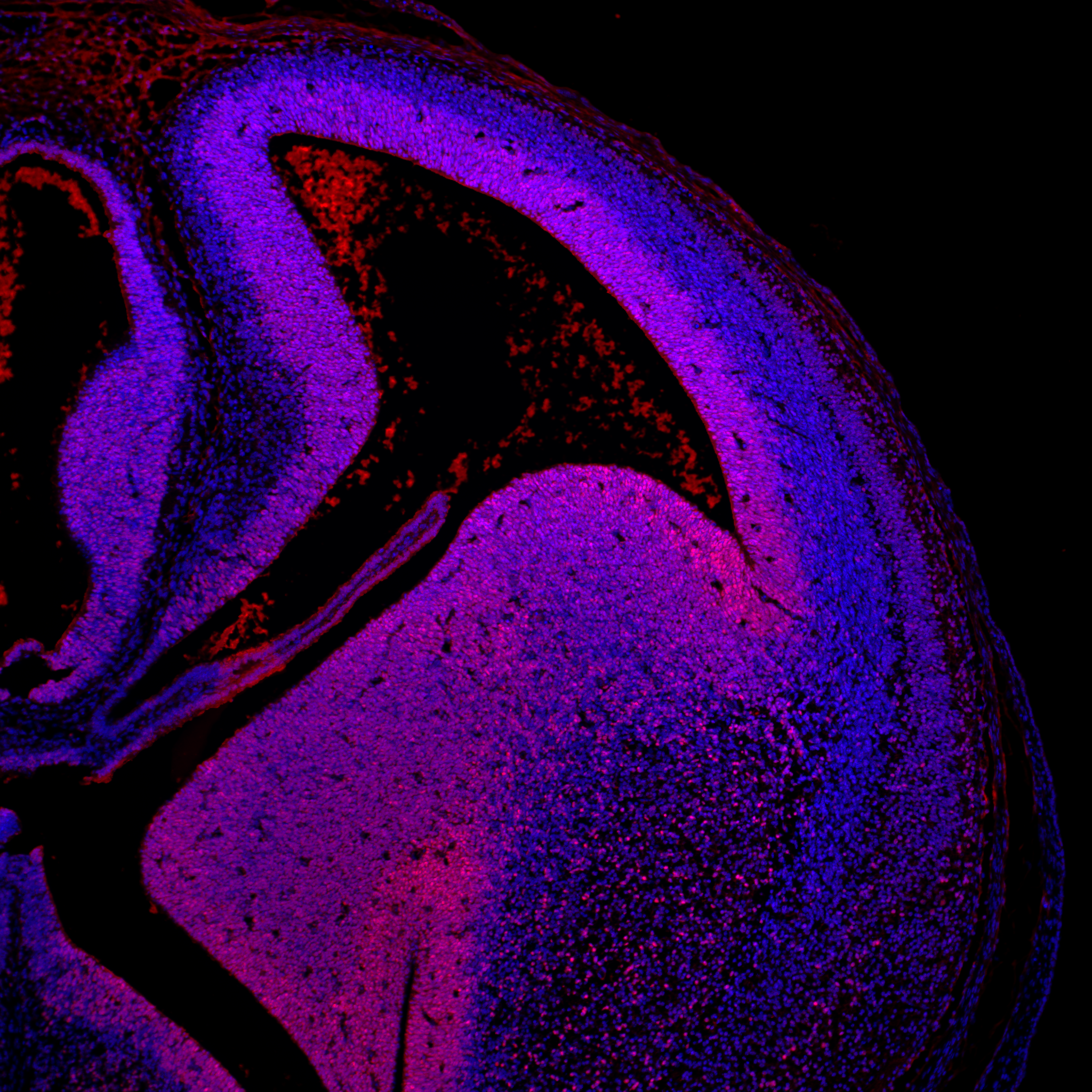

Supplement: Figure 5—source data 2. [file elife-86940-fig5-data2.zip › Figure 5-source data 2/F2116-6-E14.5-CON-RX f+ F+-115#-3-10X-SOX2-R+D-Image Export-39.tif]

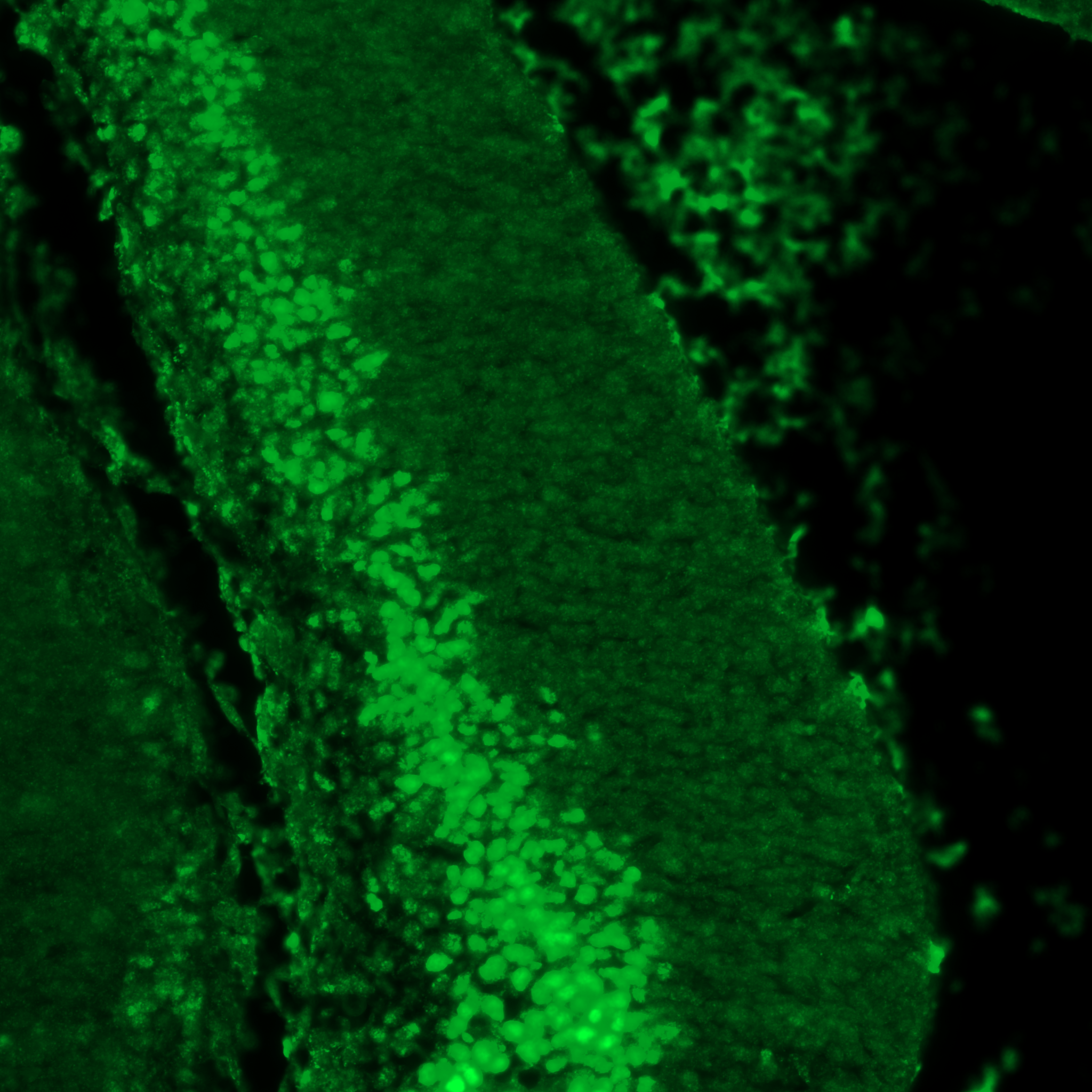

Supplement: Figure 5—source data 2. [file elife-86940-fig5-data2.zip › Figure 5-source data 2/F2116-6-E14.5-CON-RX f+ F+-115#-3-40X-NEUROD1-G-Image Export-44_AF488.tif]

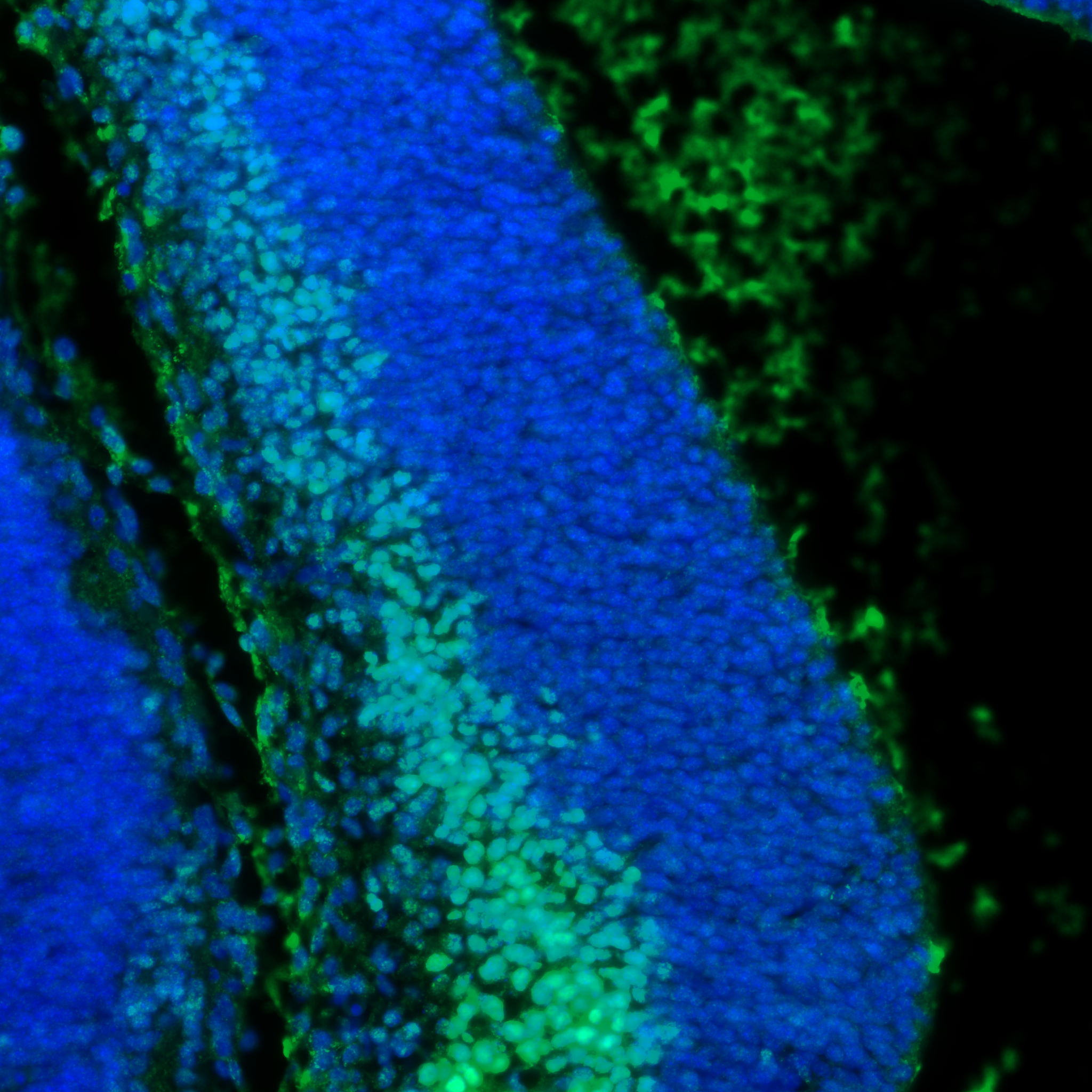

Supplement: Figure 5—source data 2. [file elife-86940-fig5-data2.zip › Figure 5-source data 2/F2116-6-E14.5-CON-RX f+ F+-115#-3-40X-NEUROD1-G+D-Image Export-44.tif]

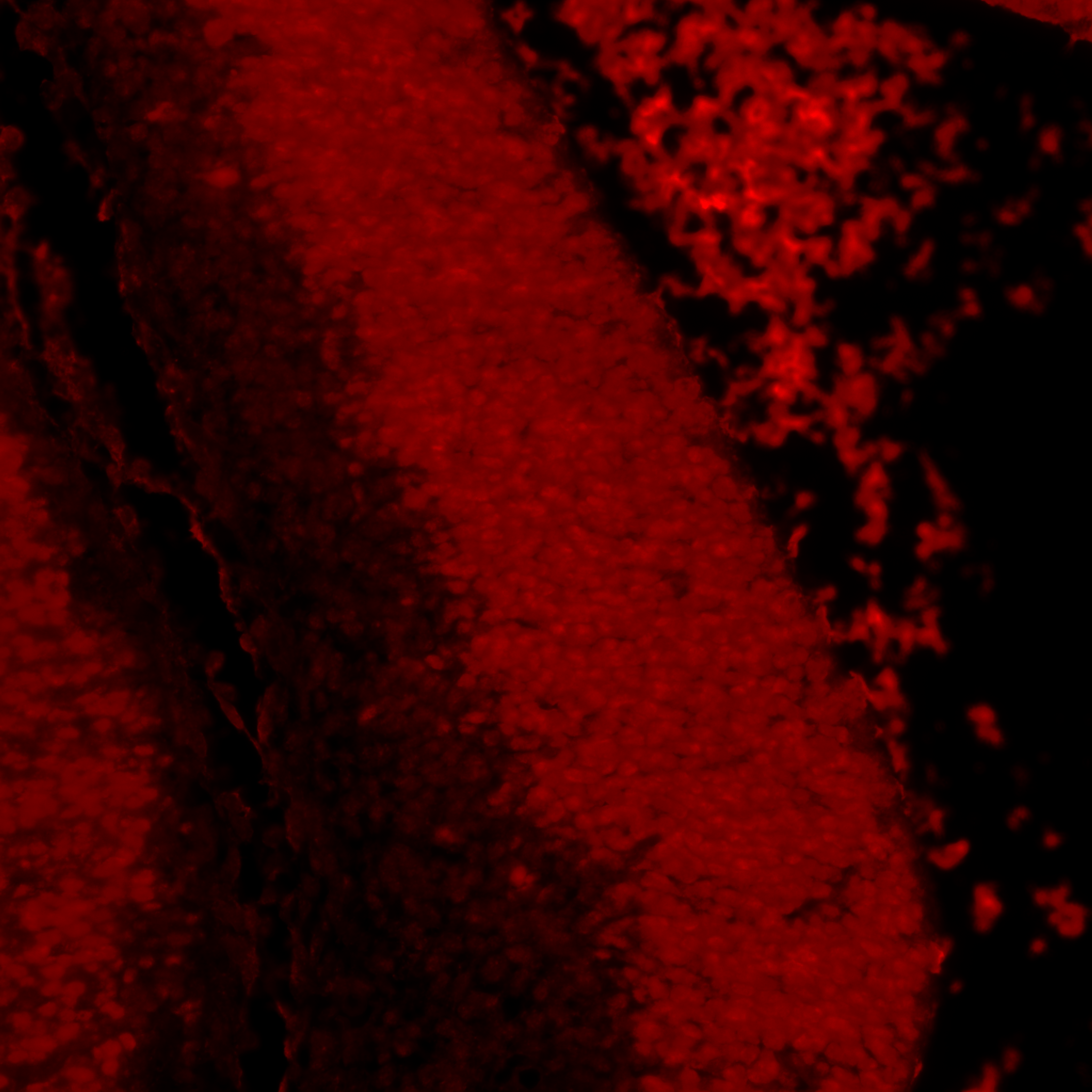

Supplement: Figure 5—source data 2. [file elife-86940-fig5-data2.zip › Figure 5-source data 2/F2116-6-E14.5-CON-RX f+ F+-115#-3-40X-SOX2-R-Image Export-45_AF594.tif]

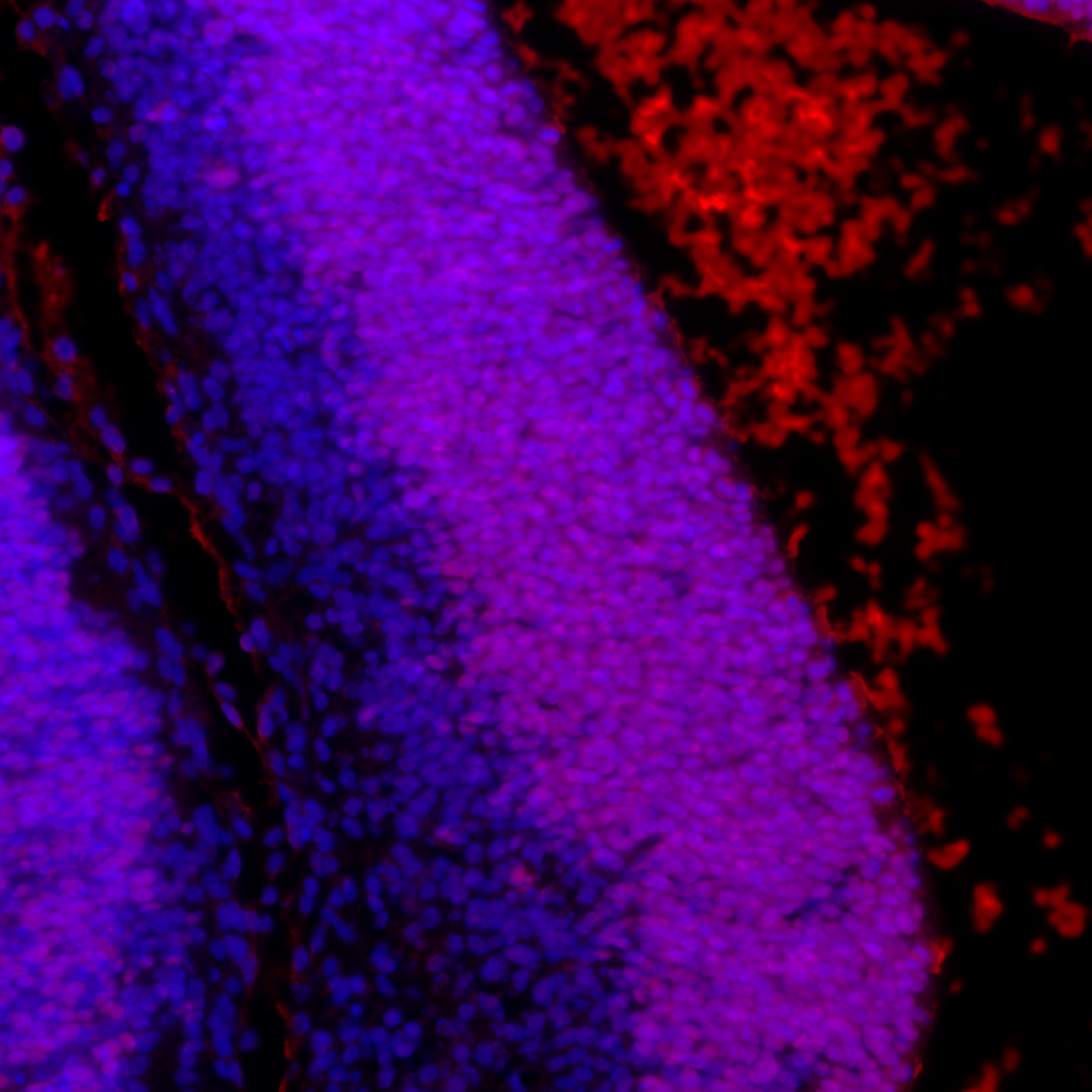

Supplement: Figure 5—source data 2. [file elife-86940-fig5-data2.zip › Figure 5-source data 2/F2116-6-E14.5-CON-RX f+ F+-115#-3-40X-SOX2-R+D-Image Export-45.tif]

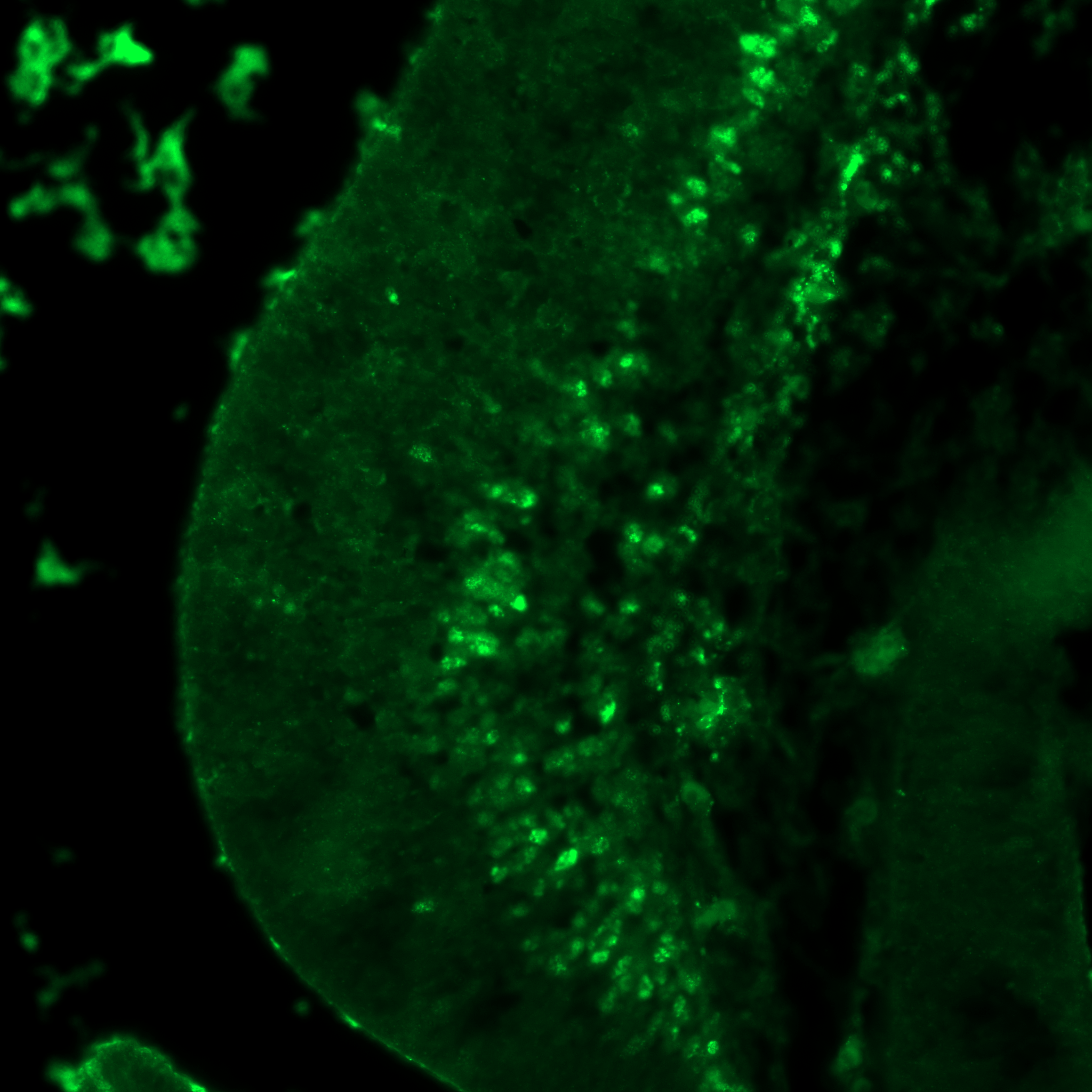

Supplement: Figure 5—source data 2. [file elife-86940-fig5-data2.zip › Figure 5-source data 2/F3204-3-E14.5-DKO-RX ff FF-40X-TBR2-20-1-L-Image Export-10_AF488.tif]

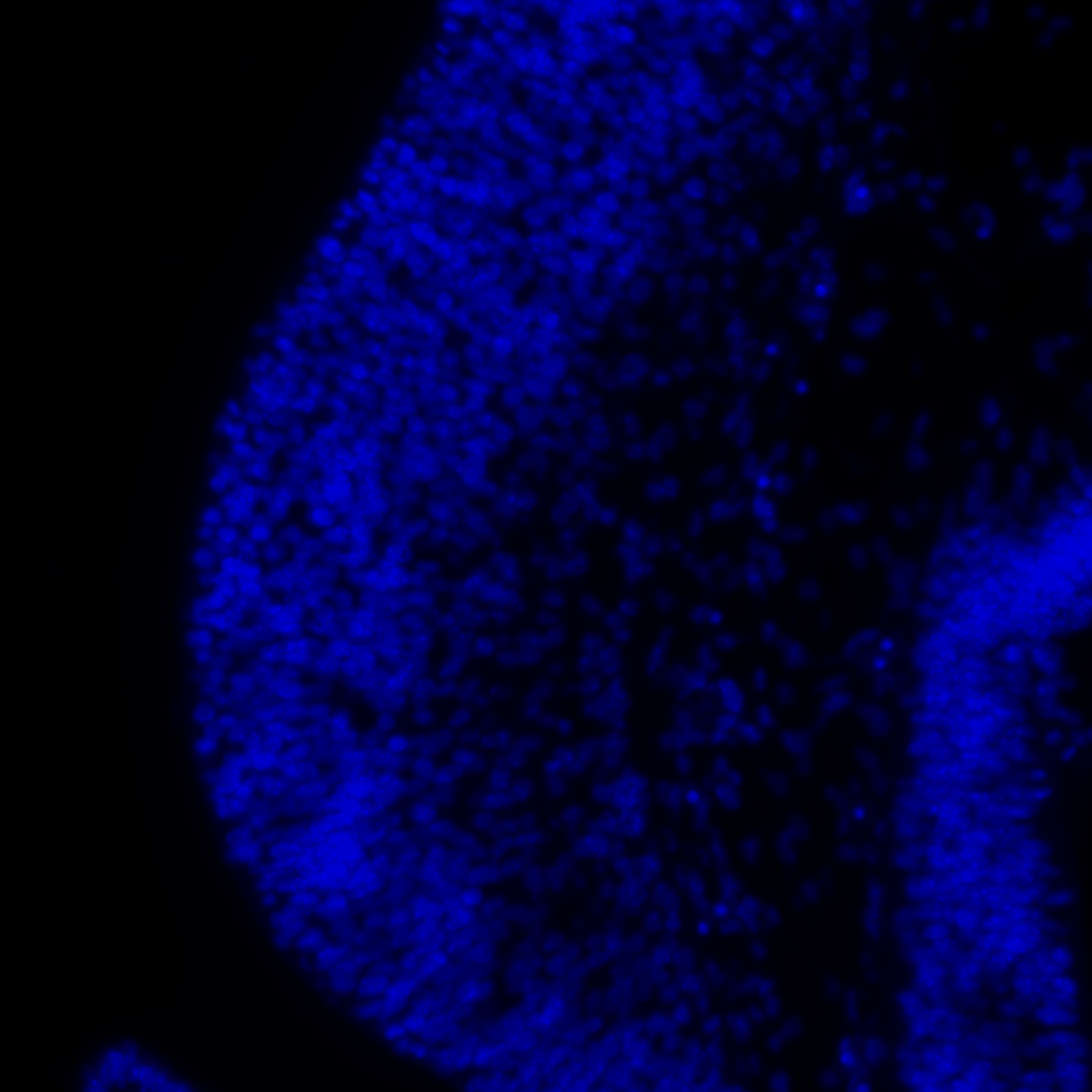

Supplement: Figure 5—source data 2. [file elife-86940-fig5-data2.zip › Figure 5-source data 2/F3204-3-E14.5-DKO-RX ff FF-40X-TBR2-20-1-L-Image Export-10_DAPI.tif]

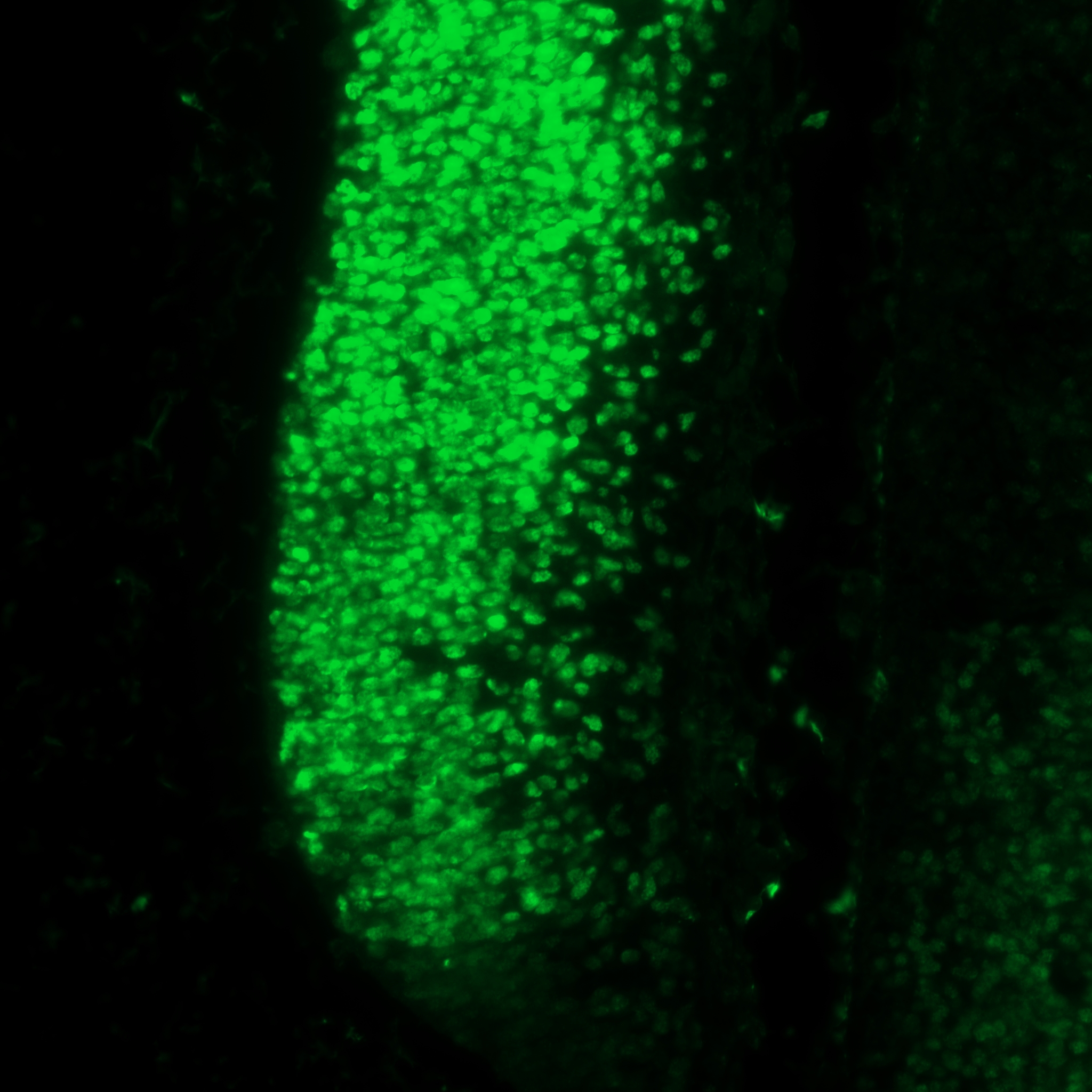

Supplement: Figure 5—source data 3. [file elife-86940-fig5-data3.zip › Figure 5-source data 3/F6091-8-DKO-E13.5-RX FF ff-40X-Lhx2-31-1-L-MP-Image Export-24_AF488.jpg]

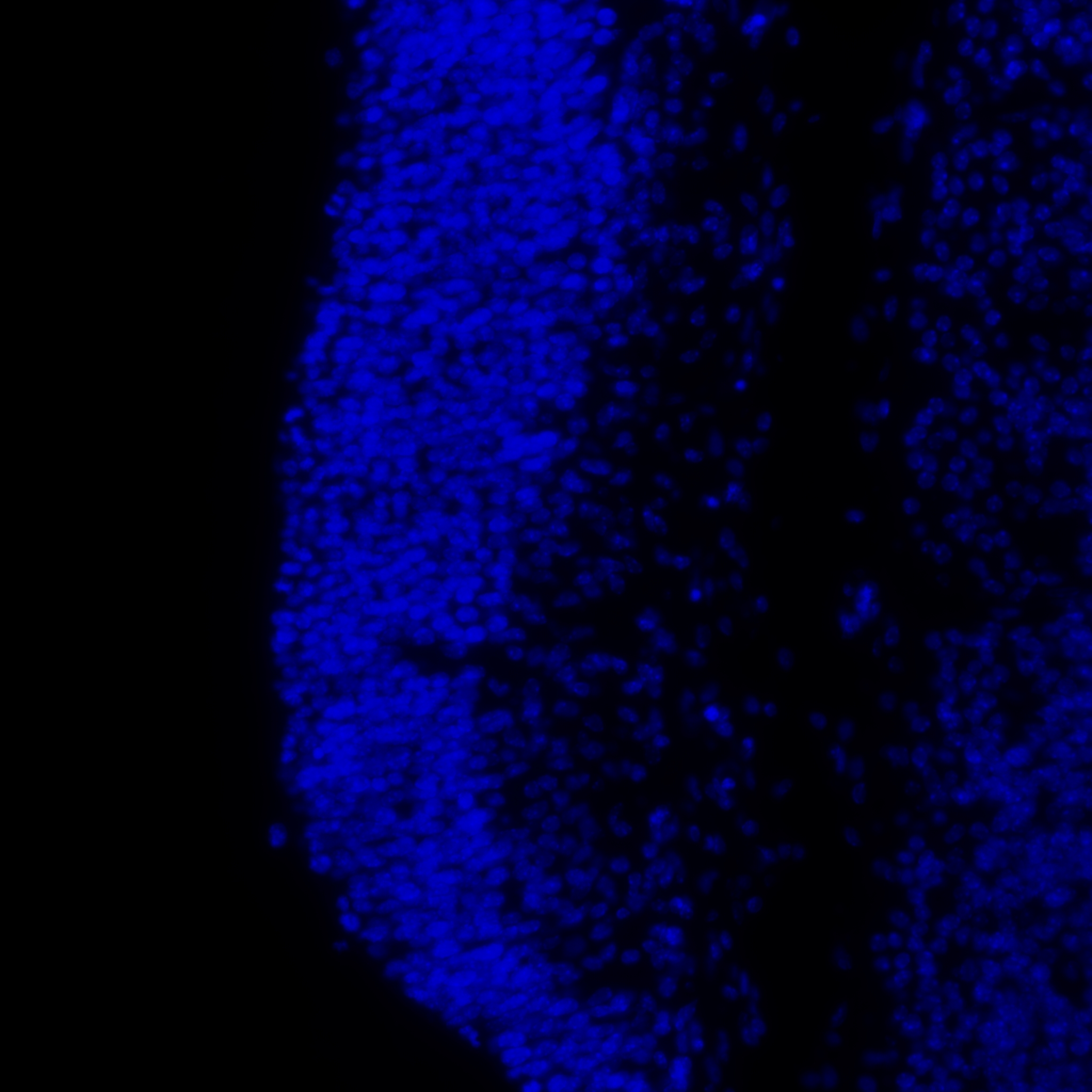

Supplement: Figure 5—source data 3. [file elife-86940-fig5-data3.zip › Figure 5-source data 3/F6091-8-DKO-E13.5-RX FF ff-40X-Lhx2-31-1-L-MP-Image Export-24_DAPI.jpg]

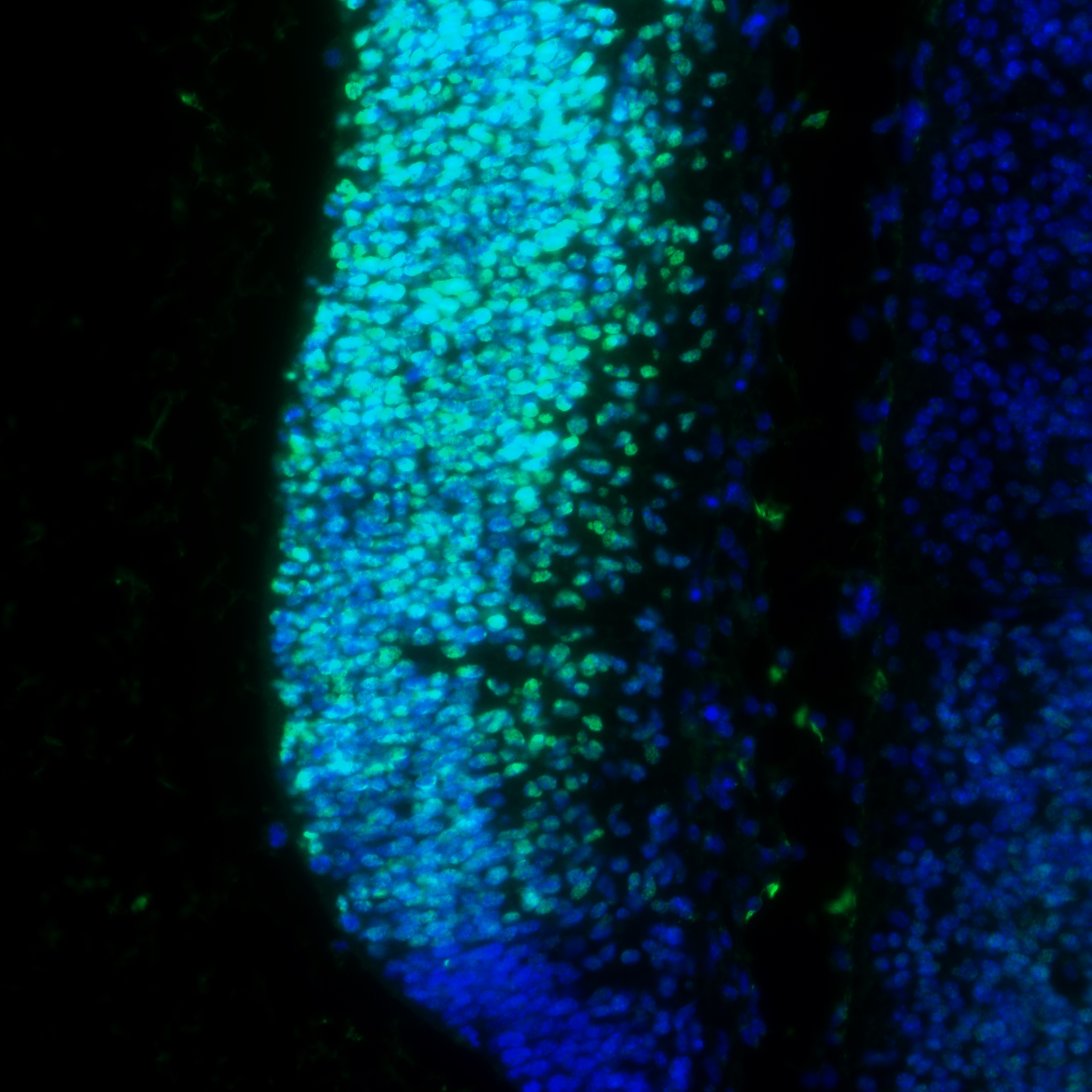

Supplement: Figure 5—source data 3. [file elife-86940-fig5-data3.zip › Figure 5-source data 3/F6091-8-DKO-E13.5-RX FF ff-40X-Lhx2-31-1-L-MP-Image Export-24.jpg]

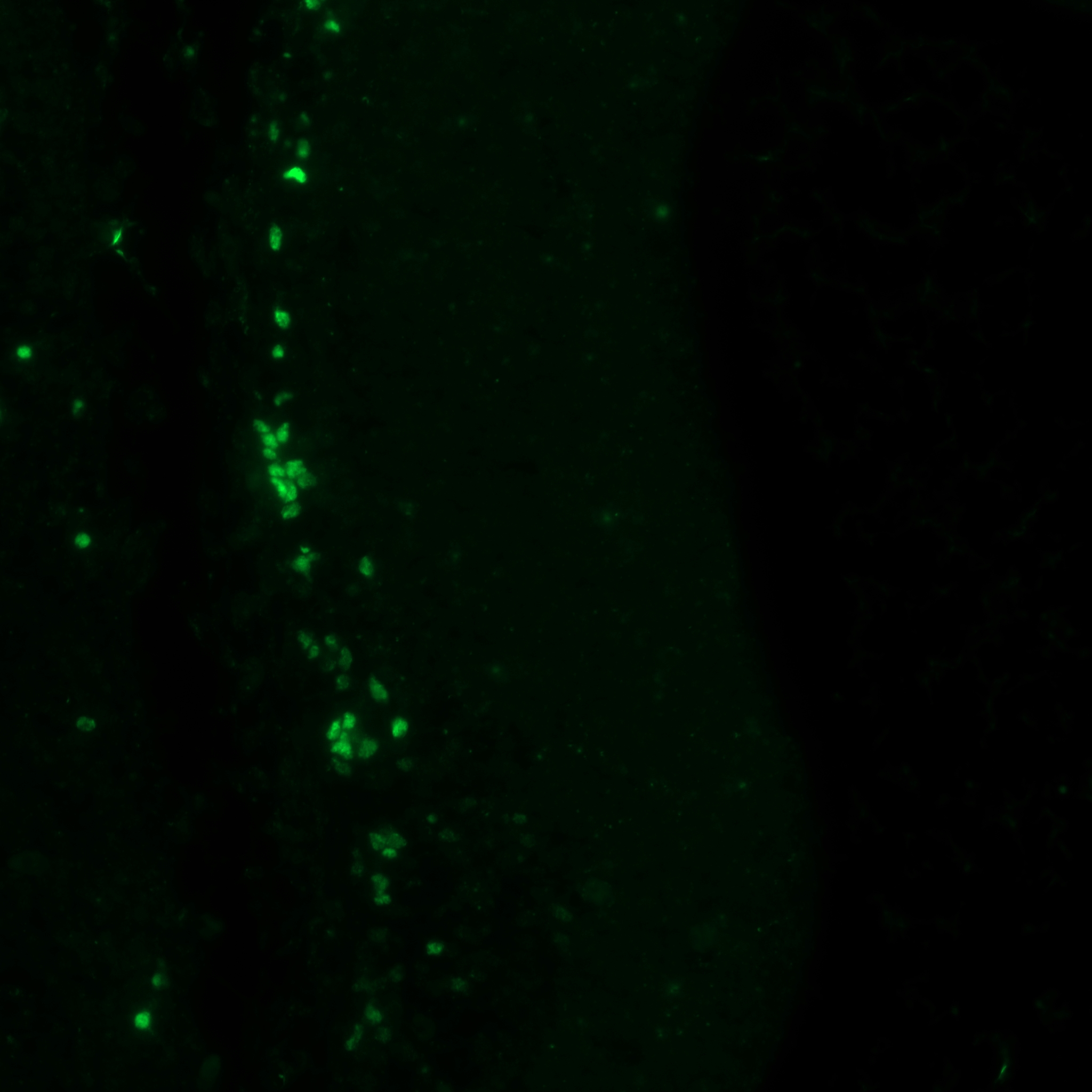

Supplement: Figure 5—source data 3. [file elife-86940-fig5-data3.zip › Figure 5-source data 3/F6091-8-DKO-E13.5-RX FF ff-40X-Lhx5-31-3-R-MP-Image Export-37_AF488.jpg]

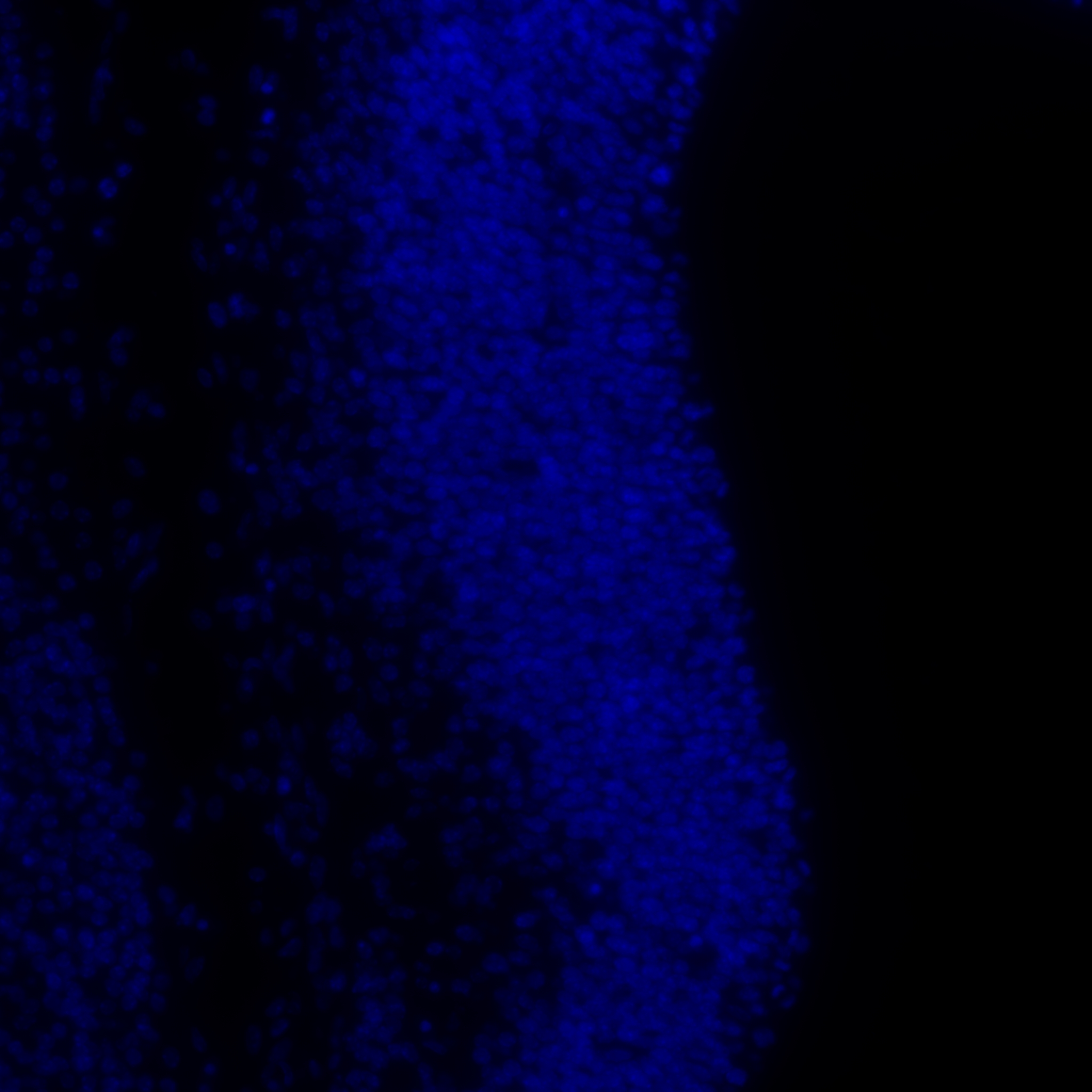

Supplement: Figure 5—source data 3. [file elife-86940-fig5-data3.zip › Figure 5-source data 3/F6091-8-DKO-E13.5-RX FF ff-40X-Lhx5-31-3-R-MP-Image Export-37_DAPI.jpg]

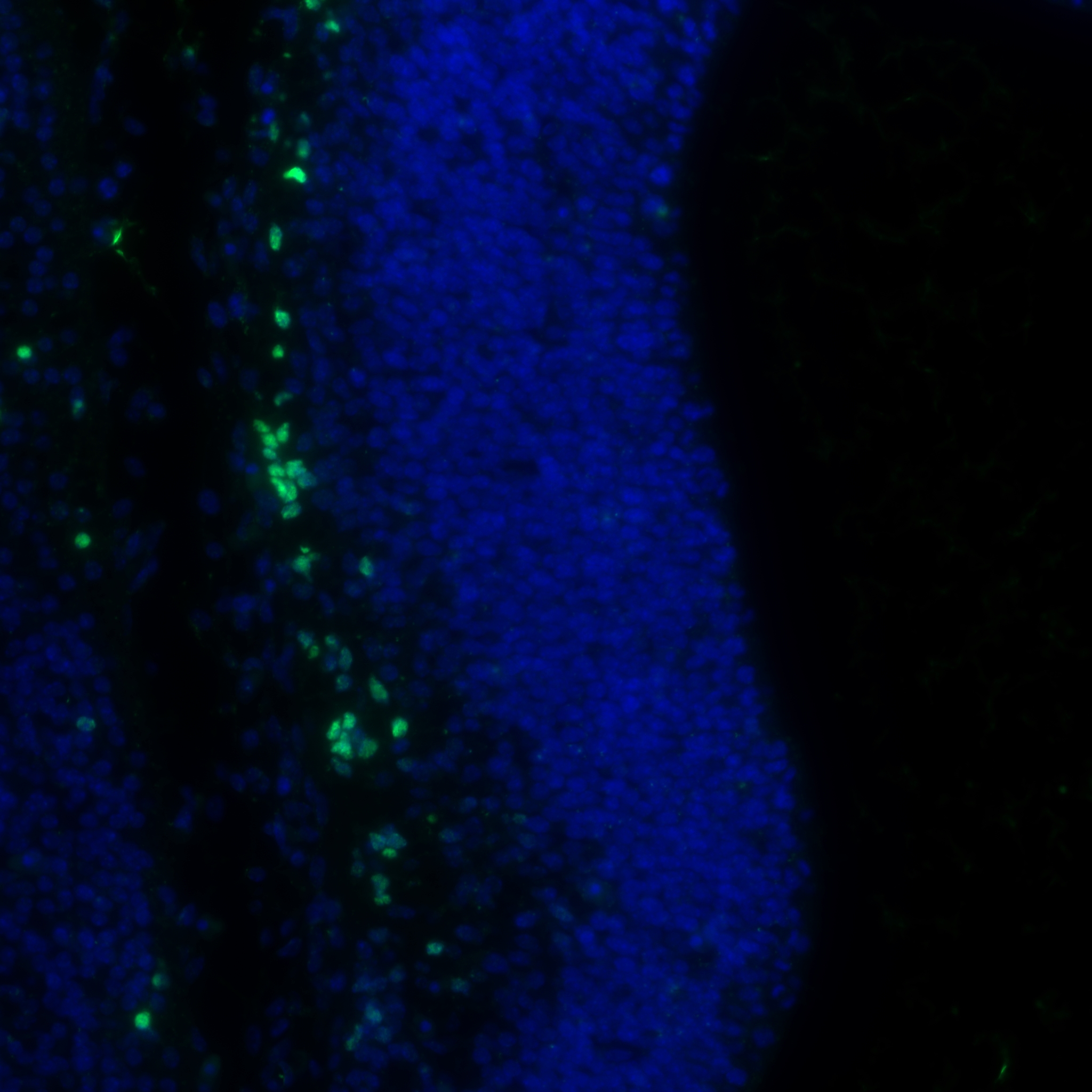

Supplement: Figure 5—source data 3. [file elife-86940-fig5-data3.zip › Figure 5-source data 3/F6091-8-DKO-E13.5-RX FF ff-40X-Lhx5-31-3-R-MP-Image Export-37.jpg]

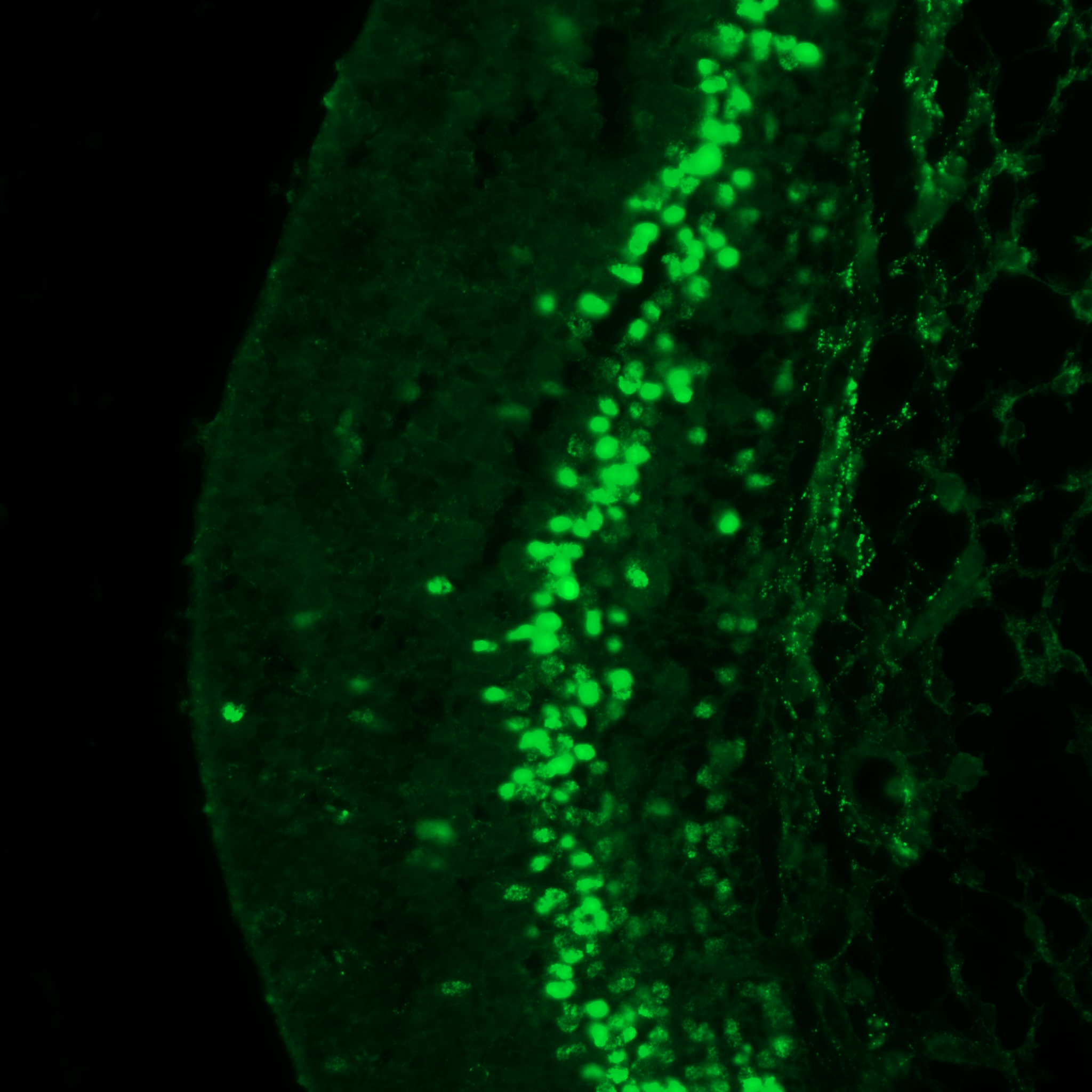

Supplement: Figure 5—source data 3. [file elife-86940-fig5-data3.zip › Figure 5-source data 3/F8273-2-CON-E14.5-f+ FF-40X-TBR2-25-2-L-Image Export-18_AF488.tif]

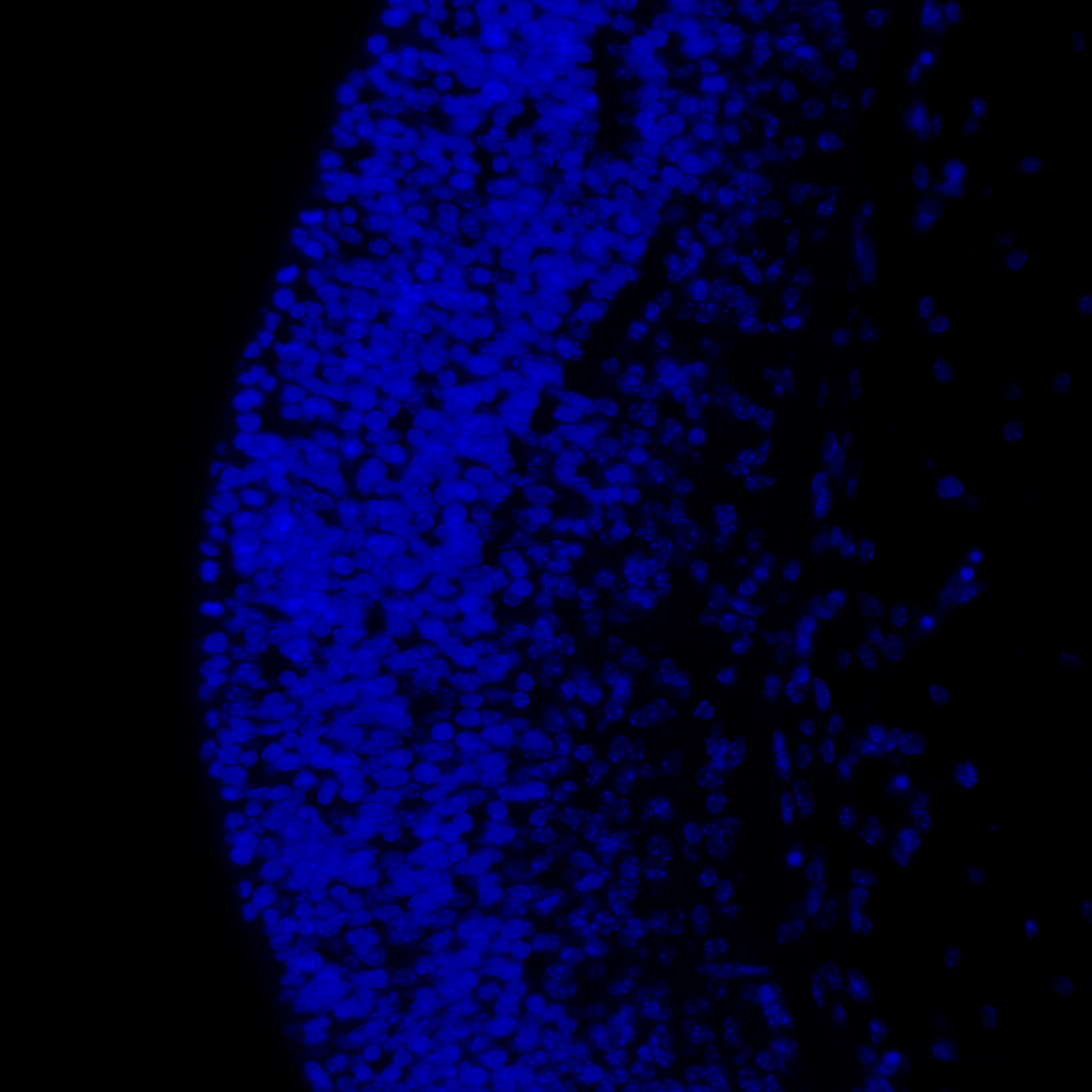

Supplement: Figure 5—source data 3. [file elife-86940-fig5-data3.zip › Figure 5-source data 3/F8273-2-CON-E14.5-f+ FF-40X-TBR2-25-2-L-Image Export-18_DAPI.tif]

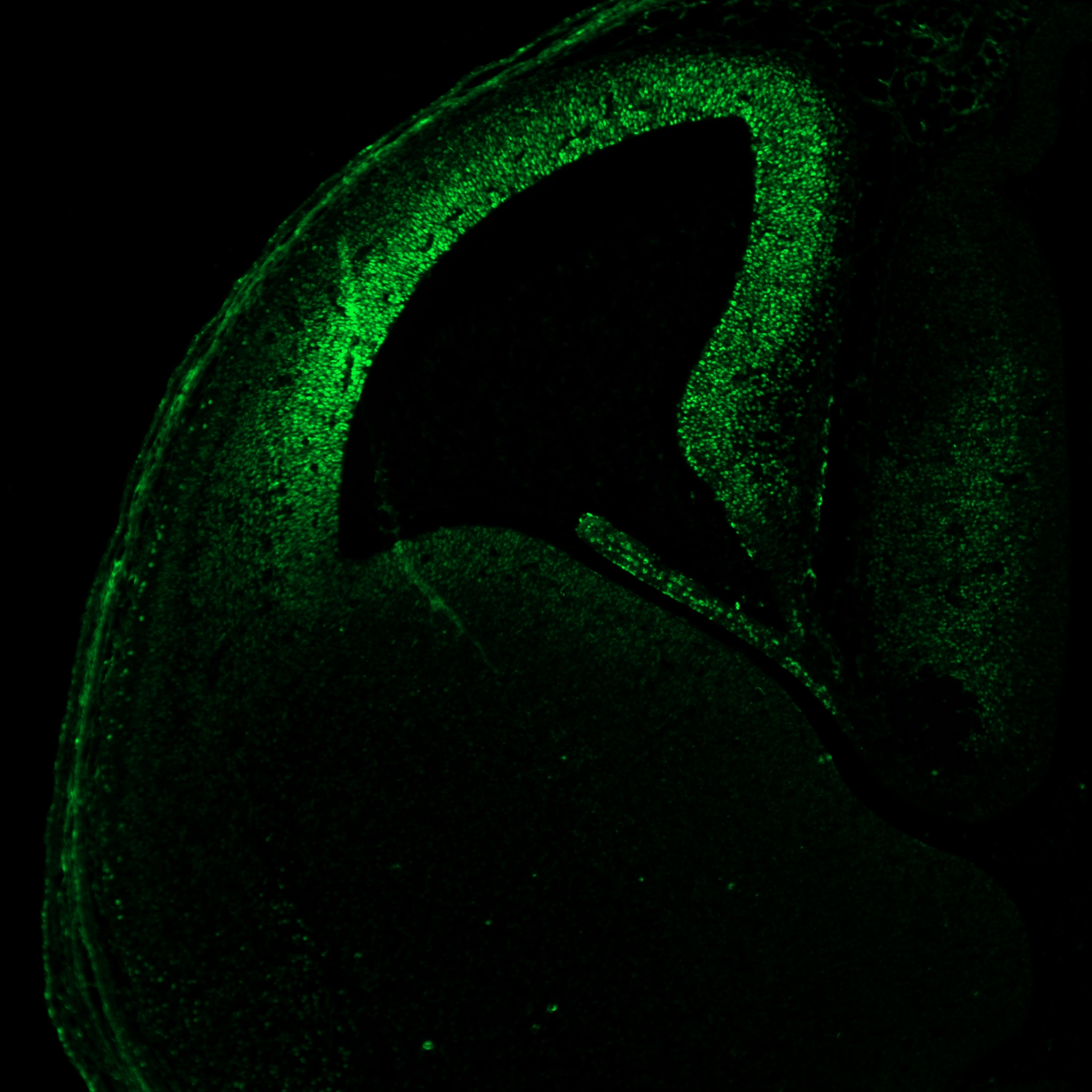

Supplement: Figure 5—source data 3. [file elife-86940-fig5-data3.zip › Figure 5-source data 3/F6091-5-CON-E13.5-FF f+-10X-Lhx2-30-1-L-Image Export-02_AF488.jpg]

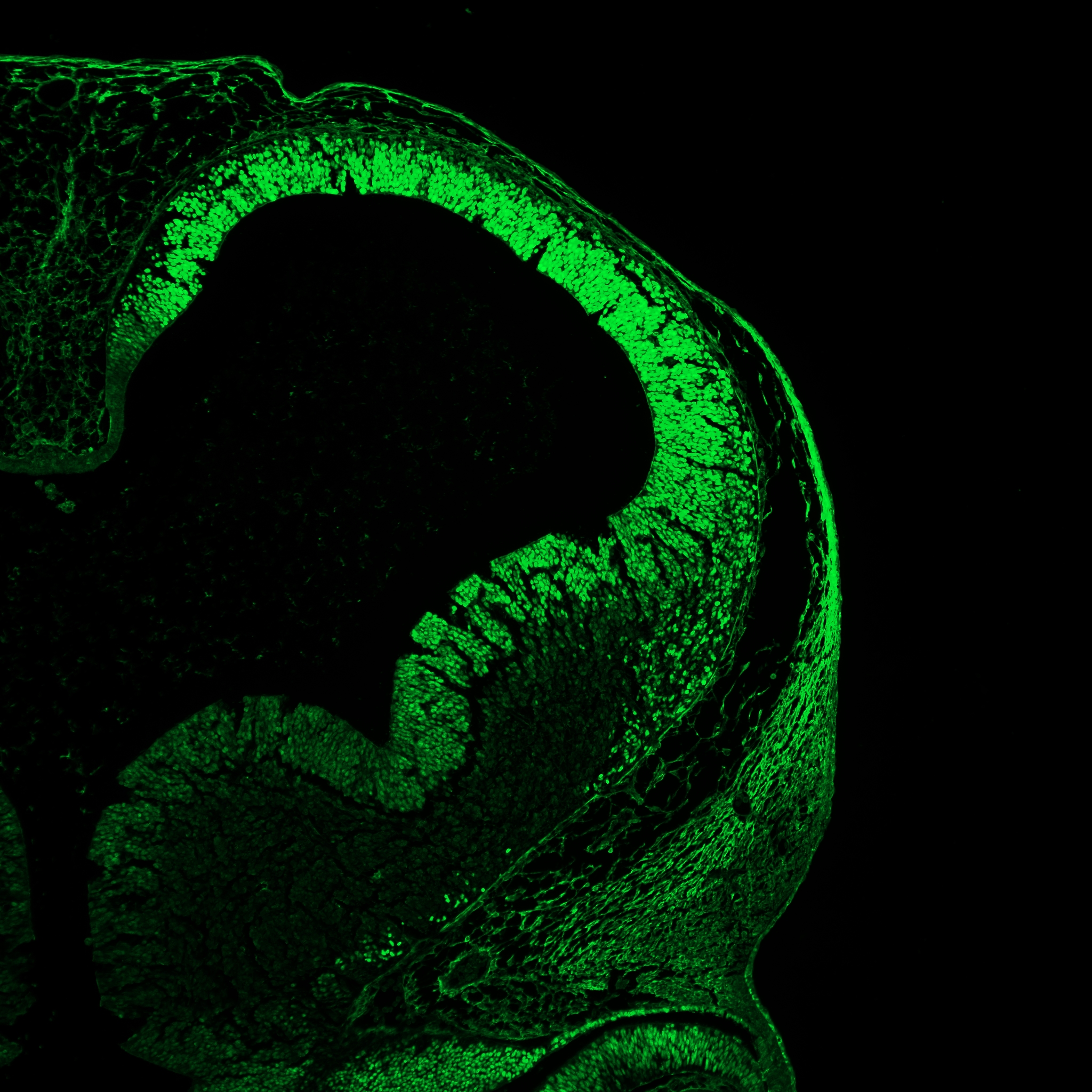

Supplement: Figure 5—source data 3. [file elife-86940-fig5-data3.zip › Figure 5-source data 3/F8871-1-DKO-E11.5-RX FF ff-10X-gLhx2-21-1-R-Image Export-61_AF488.jpg]

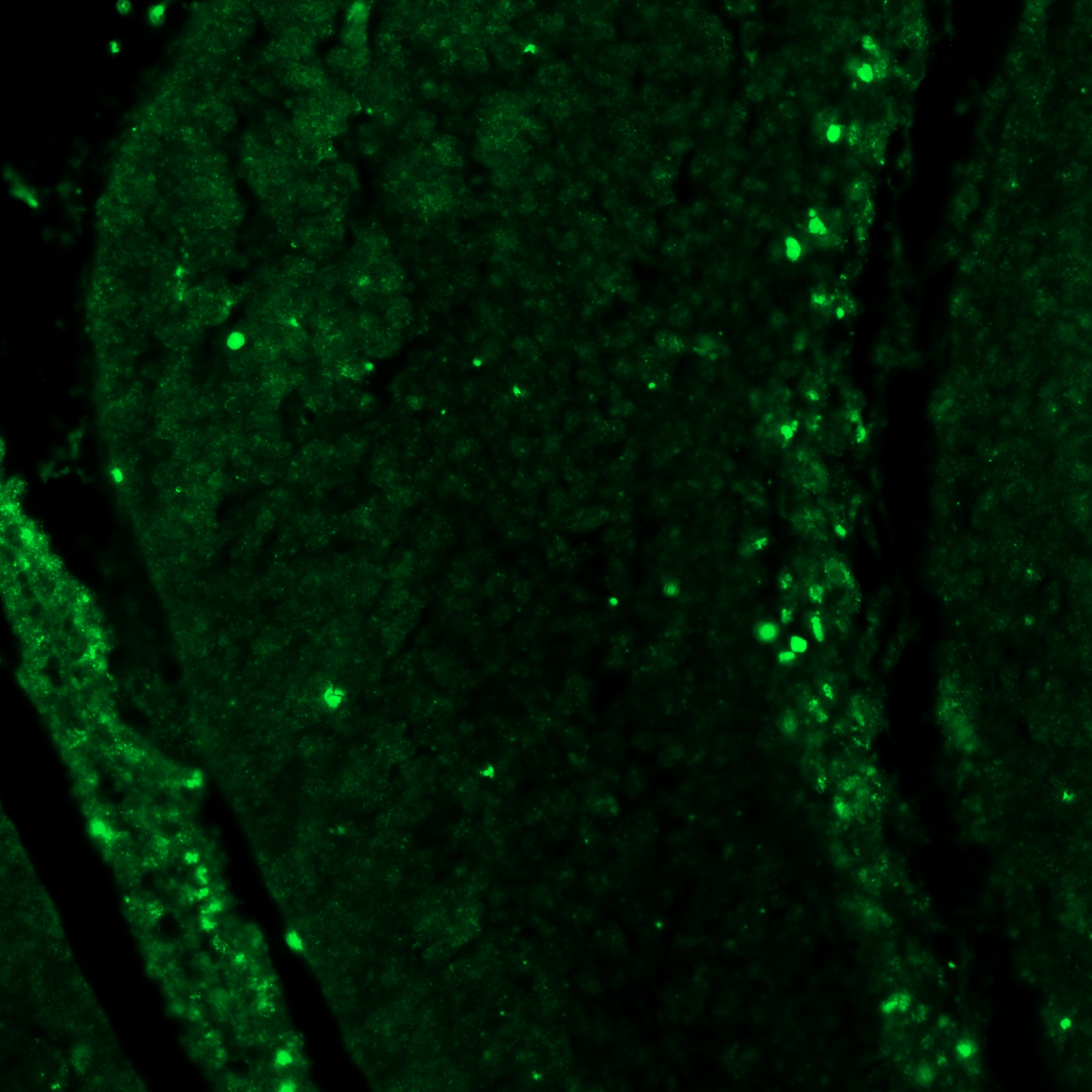

Supplement: Figure 5—source data 3. [file elife-86940-fig5-data3.zip › Figure 5-source data 3/F5734-3-DKO-RX FF ff-E14.5-40X-NEUROD1-24-1-L-Image Export-34_AF488.jpg]

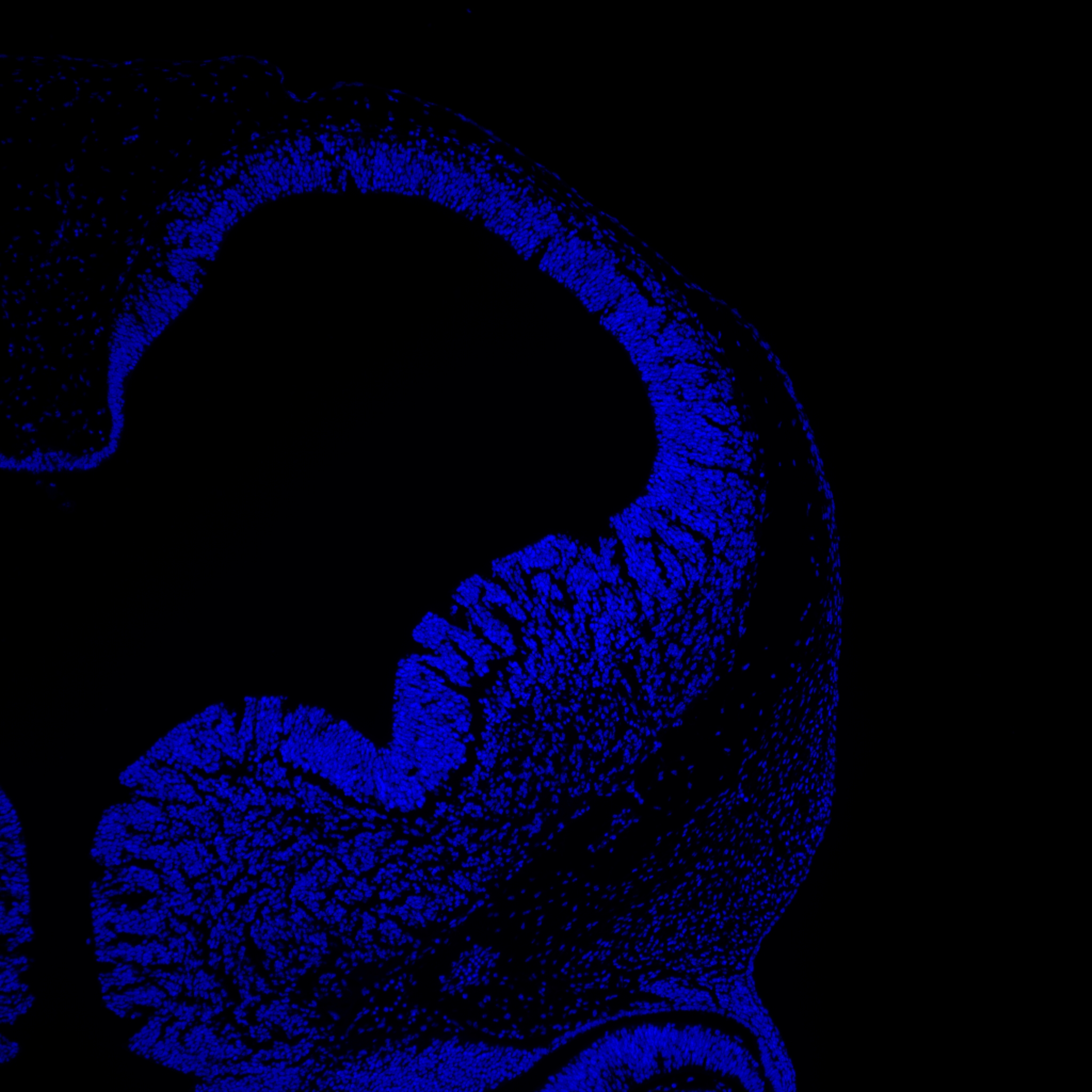

Supplement: Figure 5—source data 3. [file elife-86940-fig5-data3.zip › Figure 5-source data 3/F8871-1-DKO-E11.5-RX FF ff-10X-gLhx2-21-1-R-Image Export-61_DAPI.jpg]

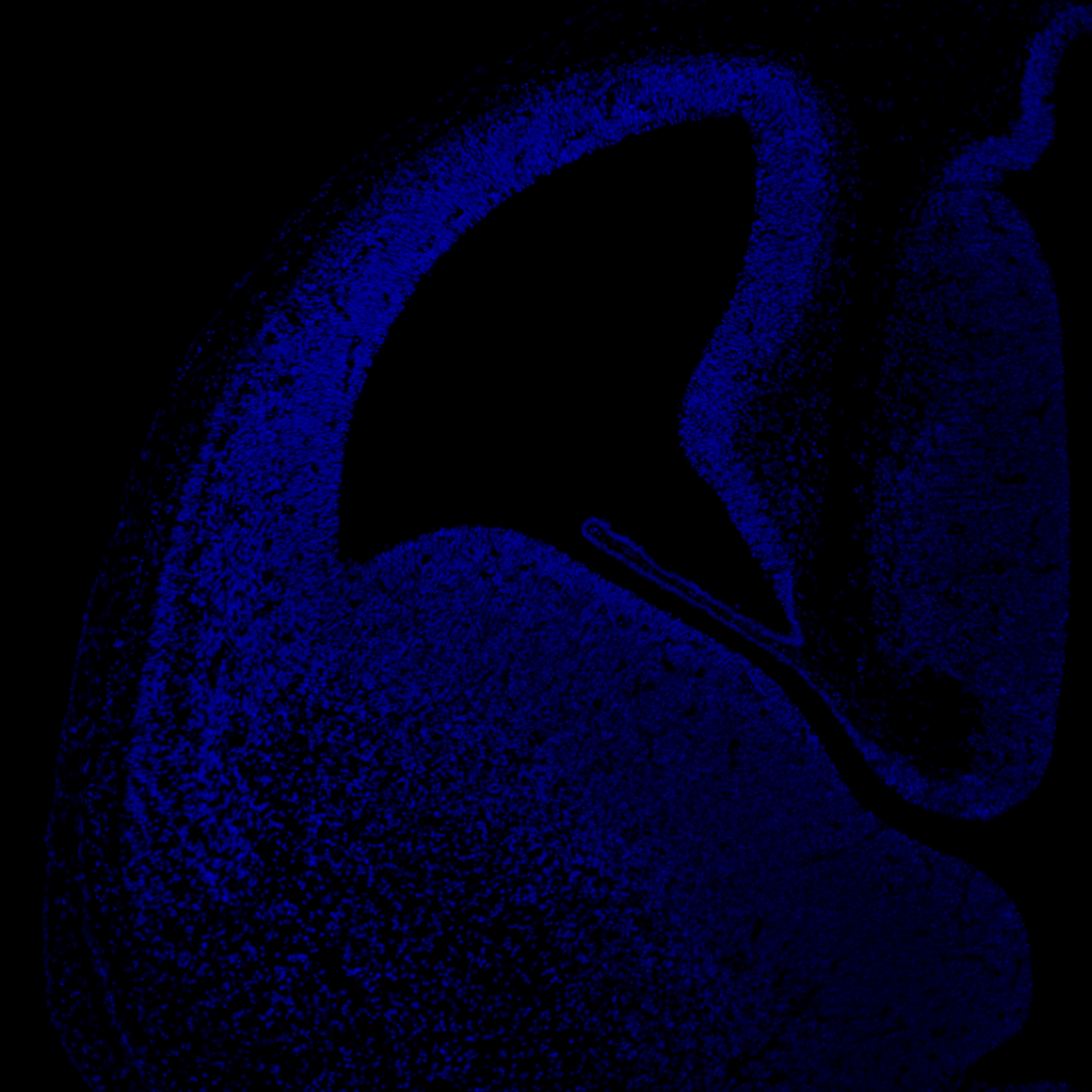

Supplement: Figure 5—source data 3. [file elife-86940-fig5-data3.zip › Figure 5-source data 3/F6091-5-CON-E13.5-FF f+-10X-Lhx2-30-1-L-Image Export-02_DAPI.jpg]

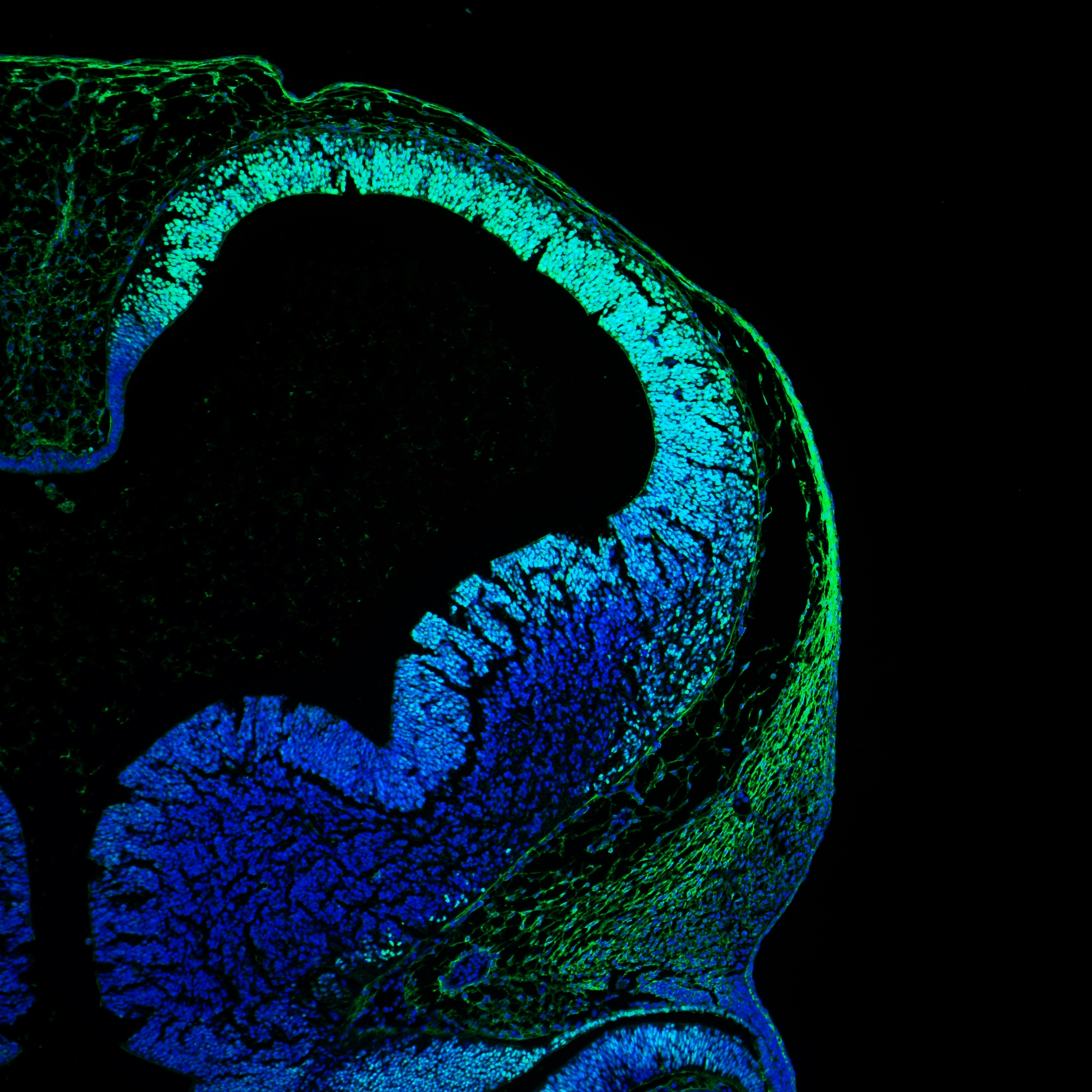

Supplement: Figure 5—source data 3. [file elife-86940-fig5-data3.zip › Figure 5-source data 3/F8871-1-DKO-E11.5-RX FF ff-10X-gLhx2-21-1-R-Image Export-61.jpg]

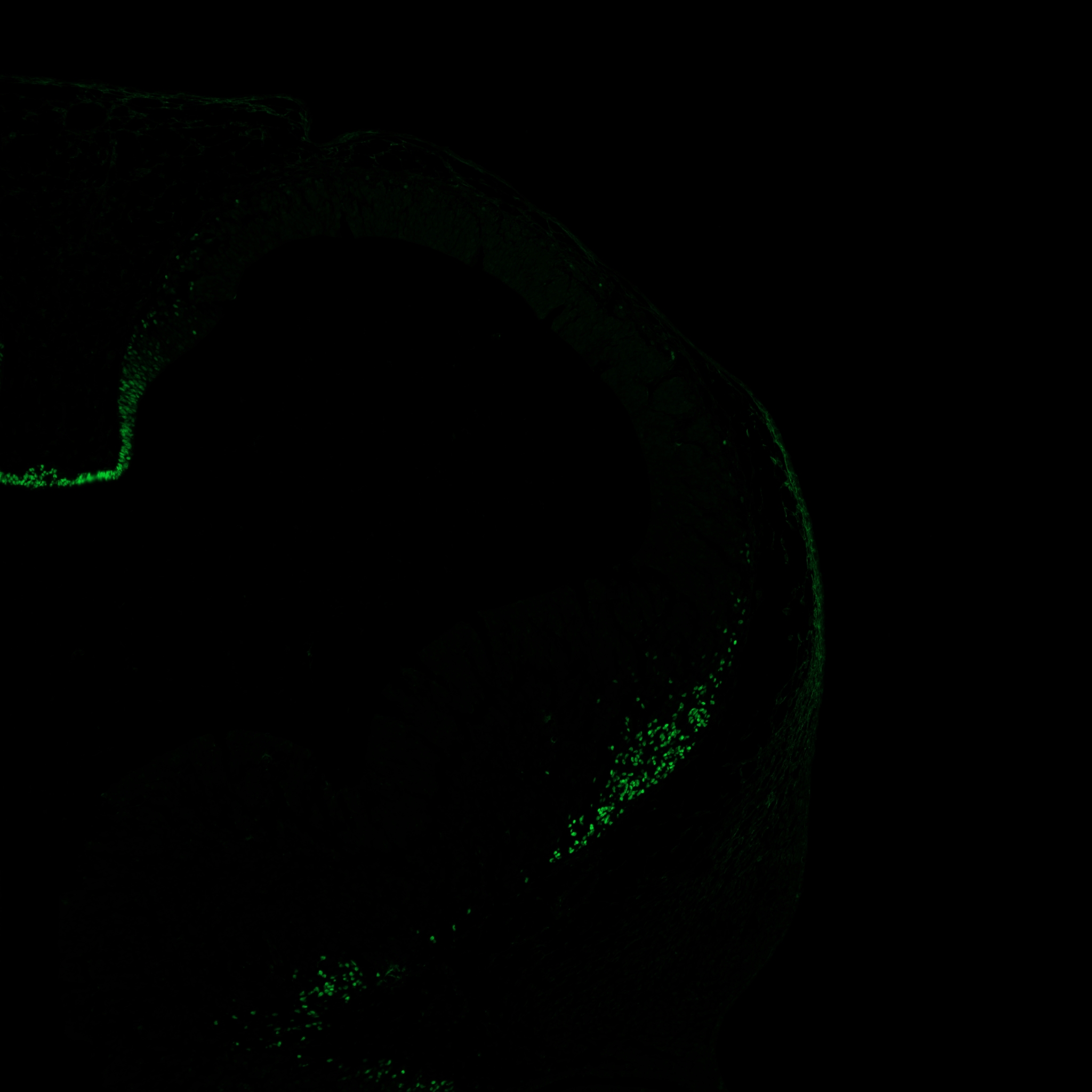

Supplement: Figure 5—source data 3. [file elife-86940-fig5-data3.zip › Figure 5-source data 3/F8871-1-DKO-E11.5-RX FF ff-10X-gLhx5-21-4-R-Image Export-67_AF488.jpg]

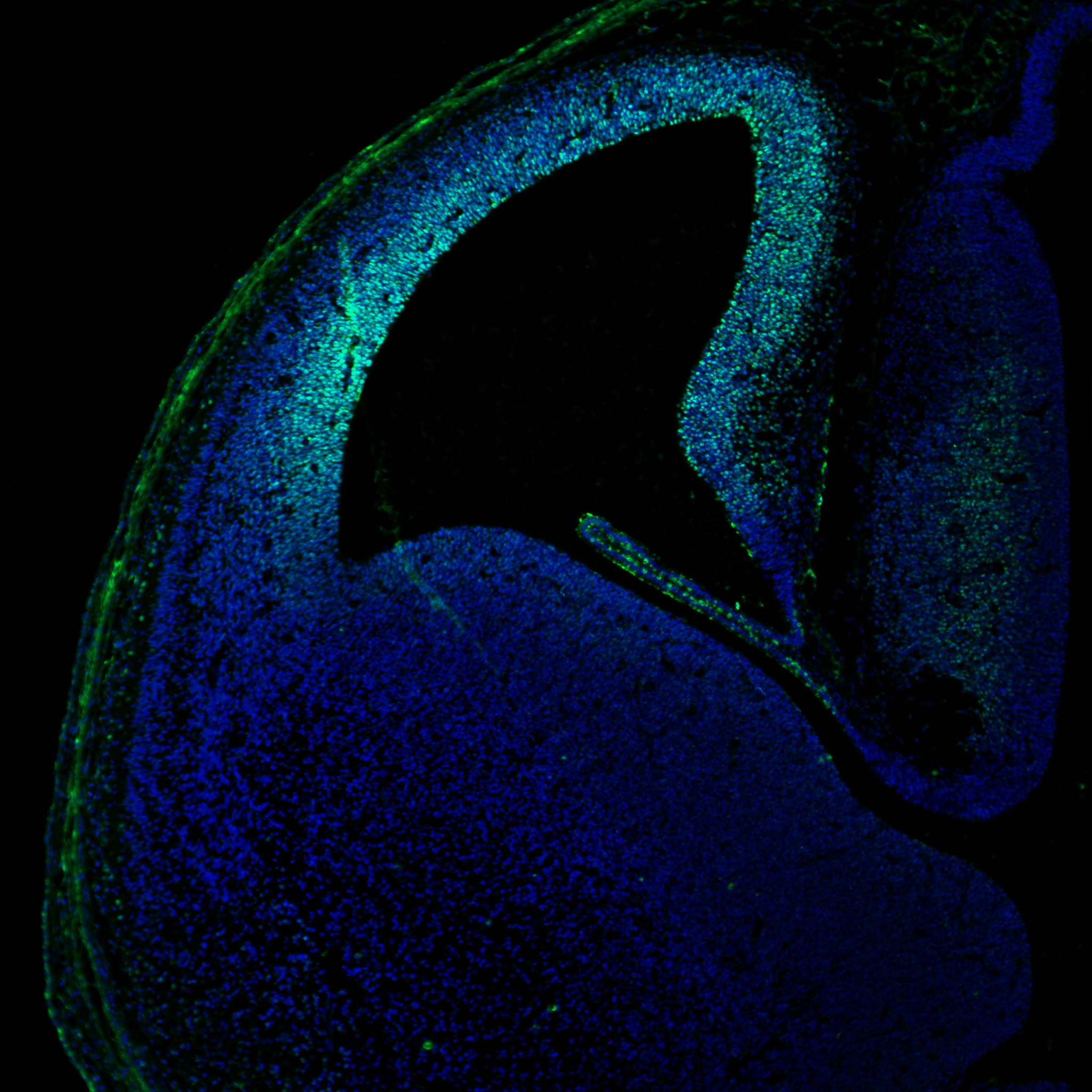

Supplement: Figure 5—source data 3. [file elife-86940-fig5-data3.zip › Figure 5-source data 3/F6091-5-CON-E13.5-FF f+-10X-Lhx2-30-1-L-Image Export-02.jpg]

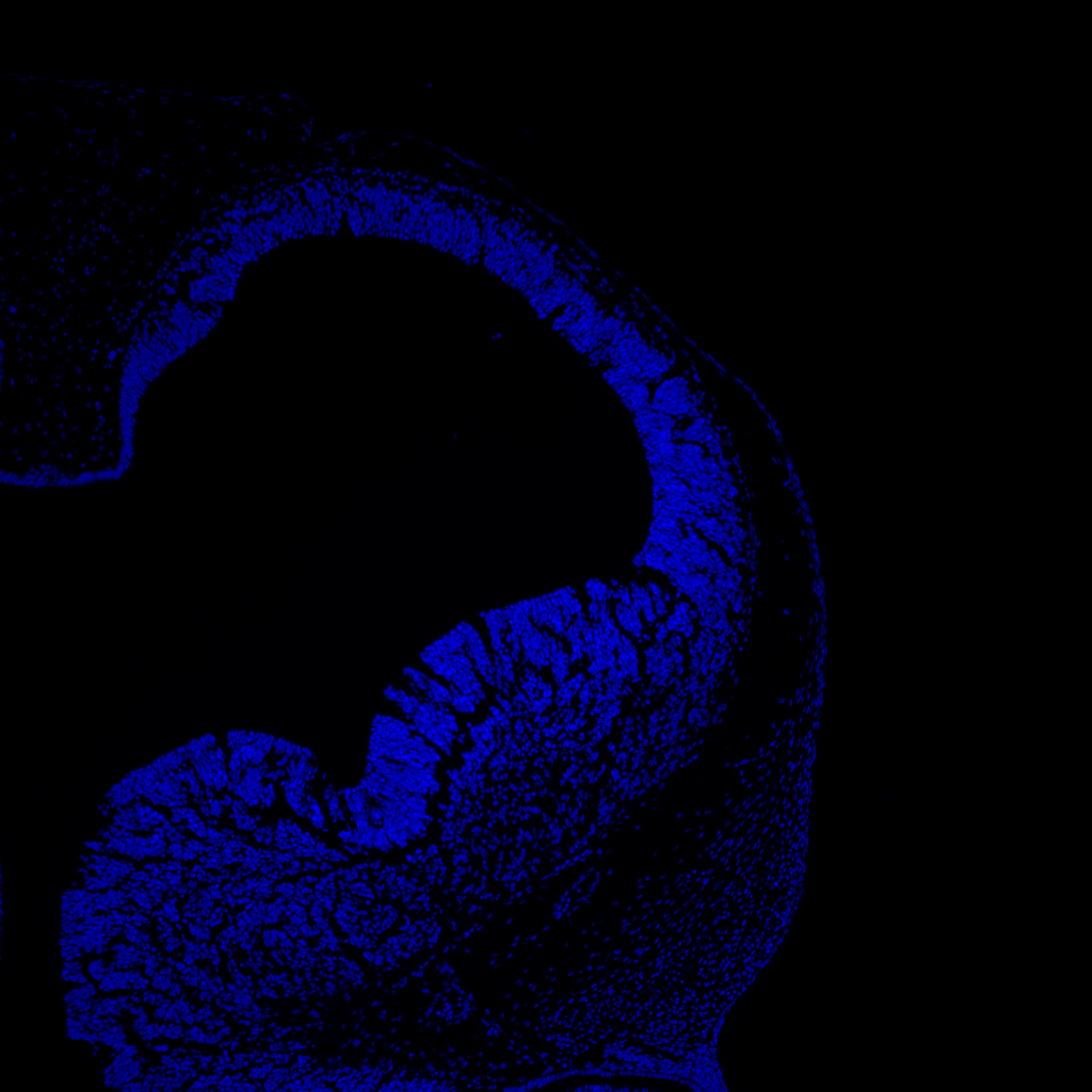

Supplement: Figure 5—source data 3. [file elife-86940-fig5-data3.zip › Figure 5-source data 3/F8871-1-DKO-E11.5-RX FF ff-10X-gLhx5-21-4-R-Image Export-67_DAPI.jpg]

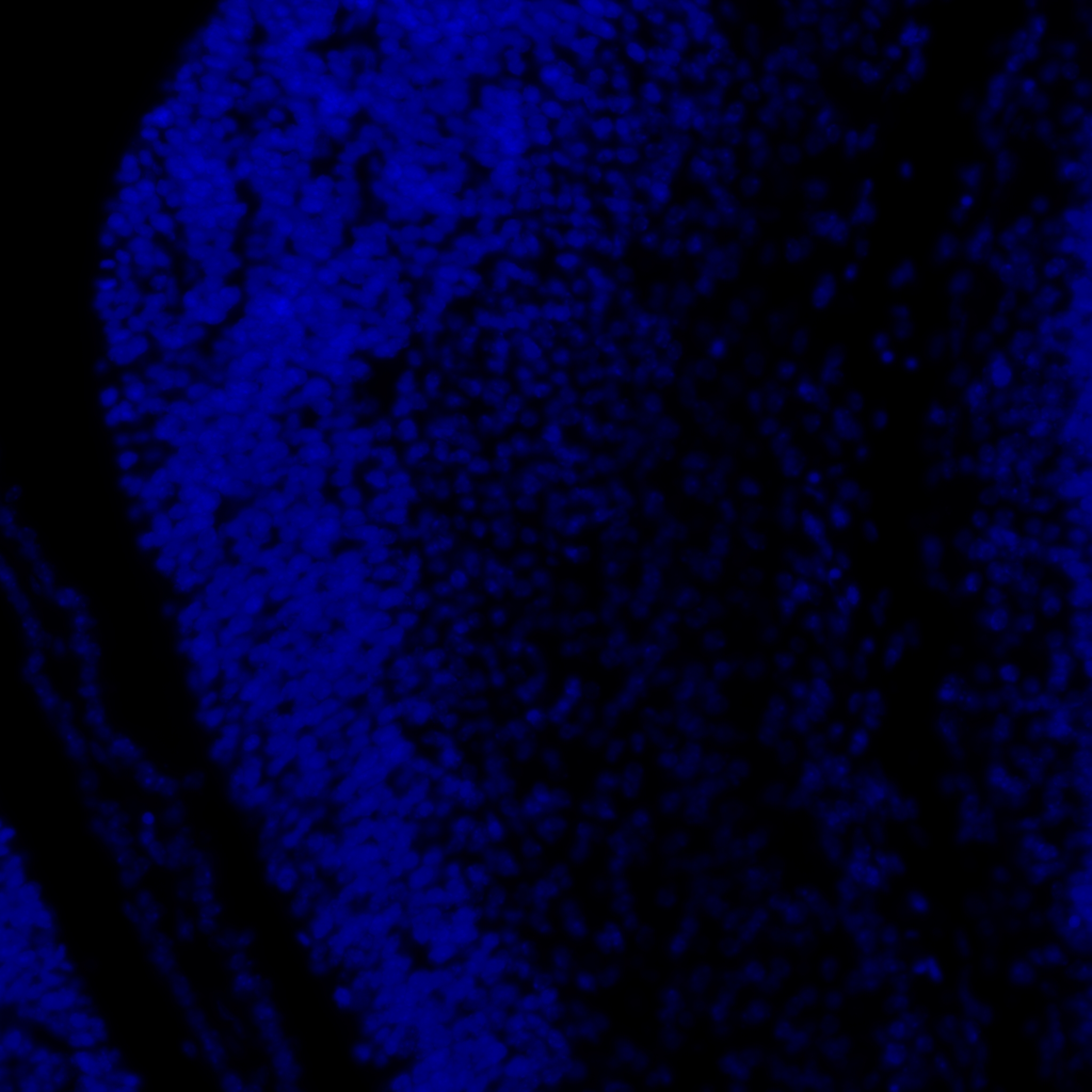

Supplement: Figure 5—source data 3. [file elife-86940-fig5-data3.zip › Figure 5-source data 3/F5734-3-DKO-RX FF ff-E14.5-40X-NEUROD1-24-1-L-Image Export-34_DAPI.jpg]

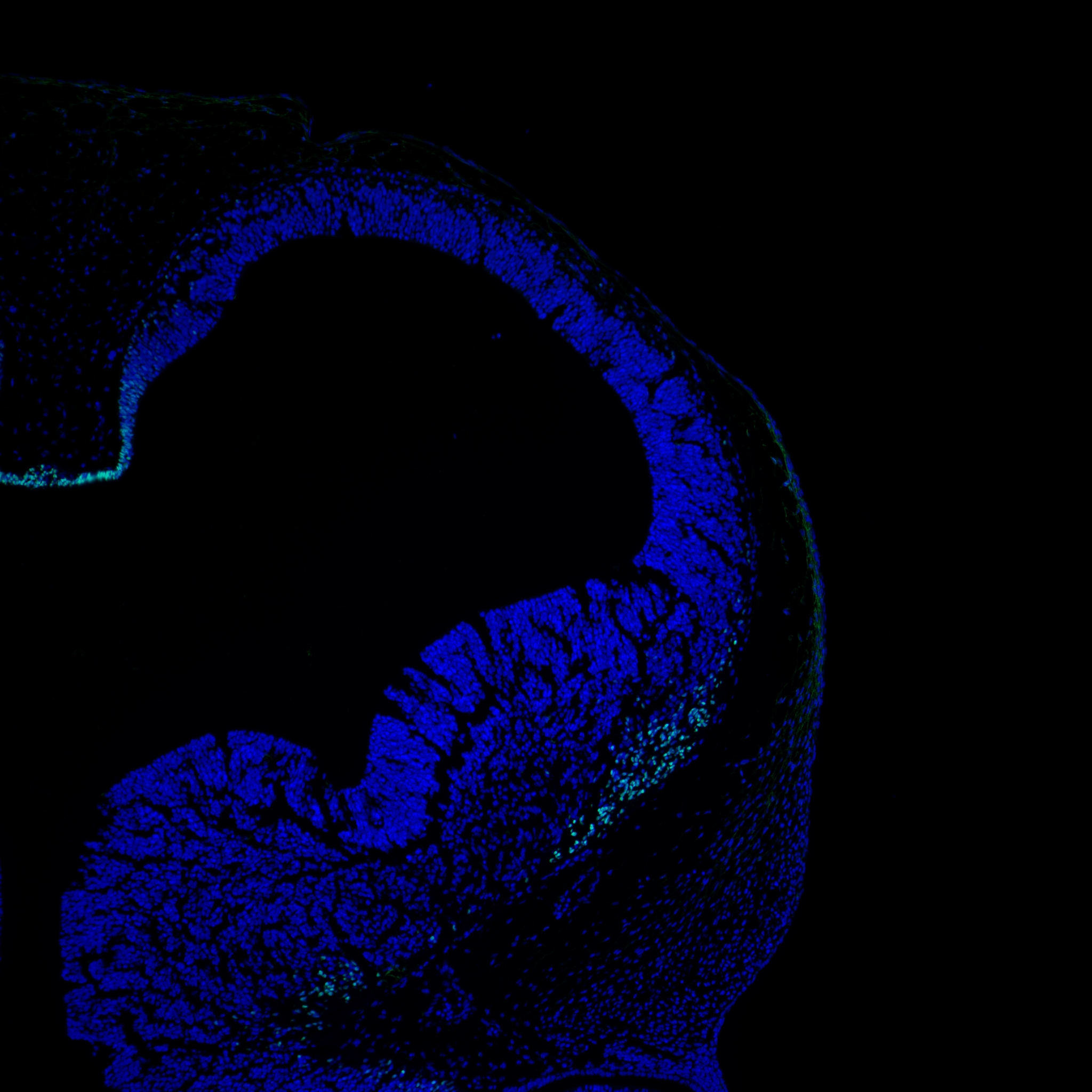

Supplement: Figure 5—source data 3. [file elife-86940-fig5-data3.zip › Figure 5-source data 3/F8871-1-DKO-E11.5-RX FF ff-10X-gLhx5-21-4-R-Image Export-67.jpg]

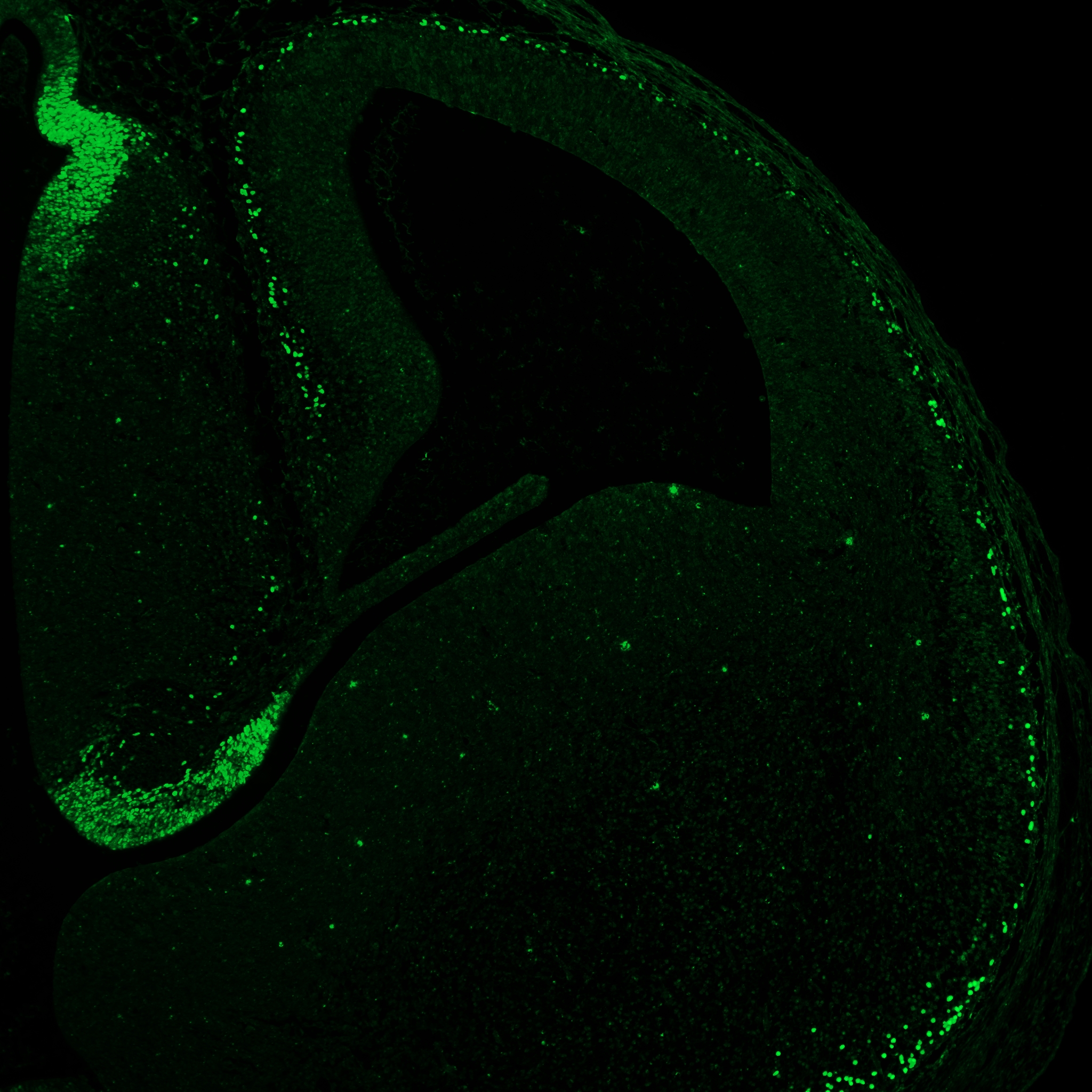

Supplement: Figure 5—source data 3. [file elife-86940-fig5-data3.zip › Figure 5-source data 3/F6091-5-CON-E13.5-FF f+-10X-Lhx5-30-4-R-Image Export-18_AF488.jpg]

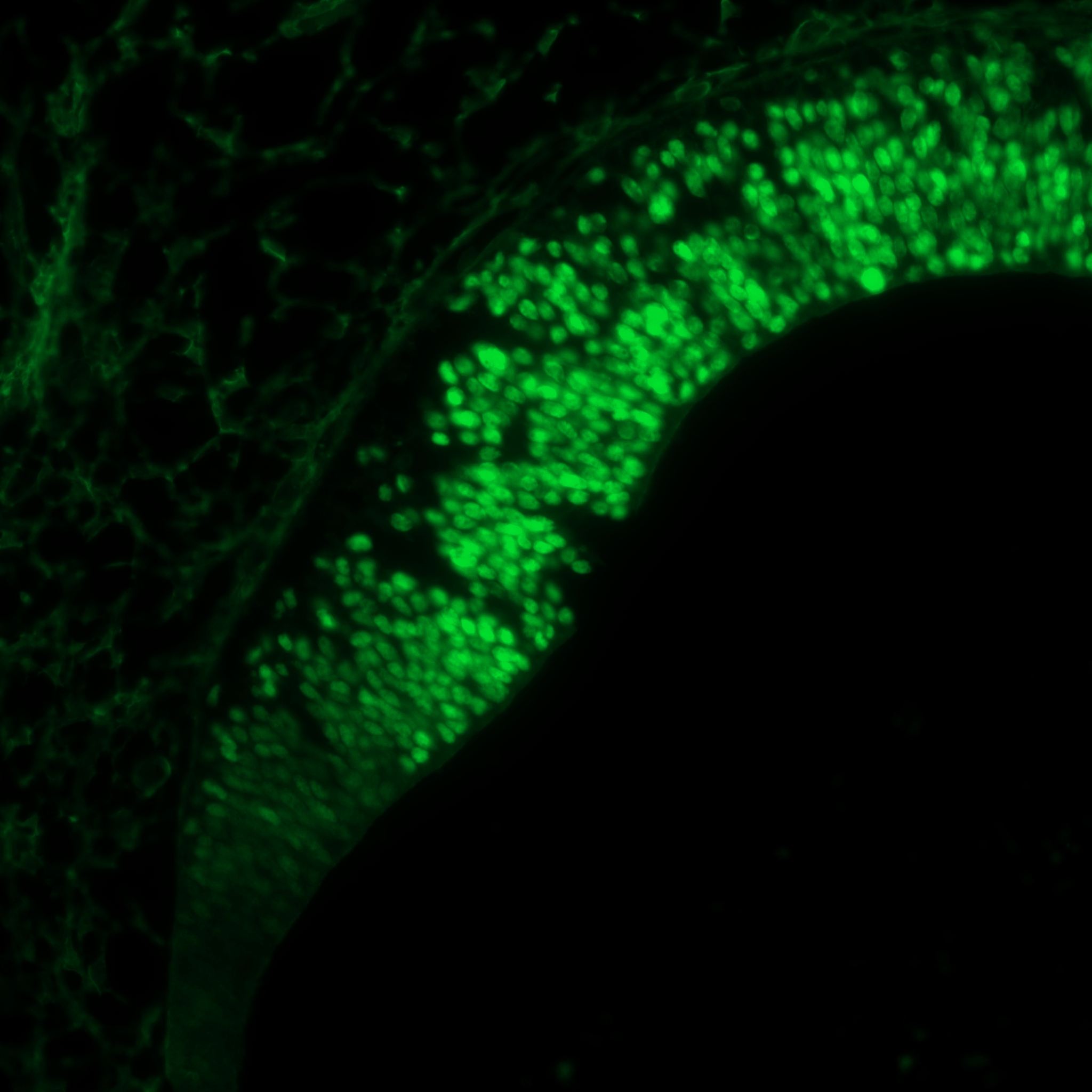

Supplement: Figure 5—source data 3. [file elife-86940-fig5-data3.zip › Figure 5-source data 3/F8871-1-DKO-E11.5-RX FF ff-40X-gLhx2-21-1-R-Image Export-69_AF488.jpg]

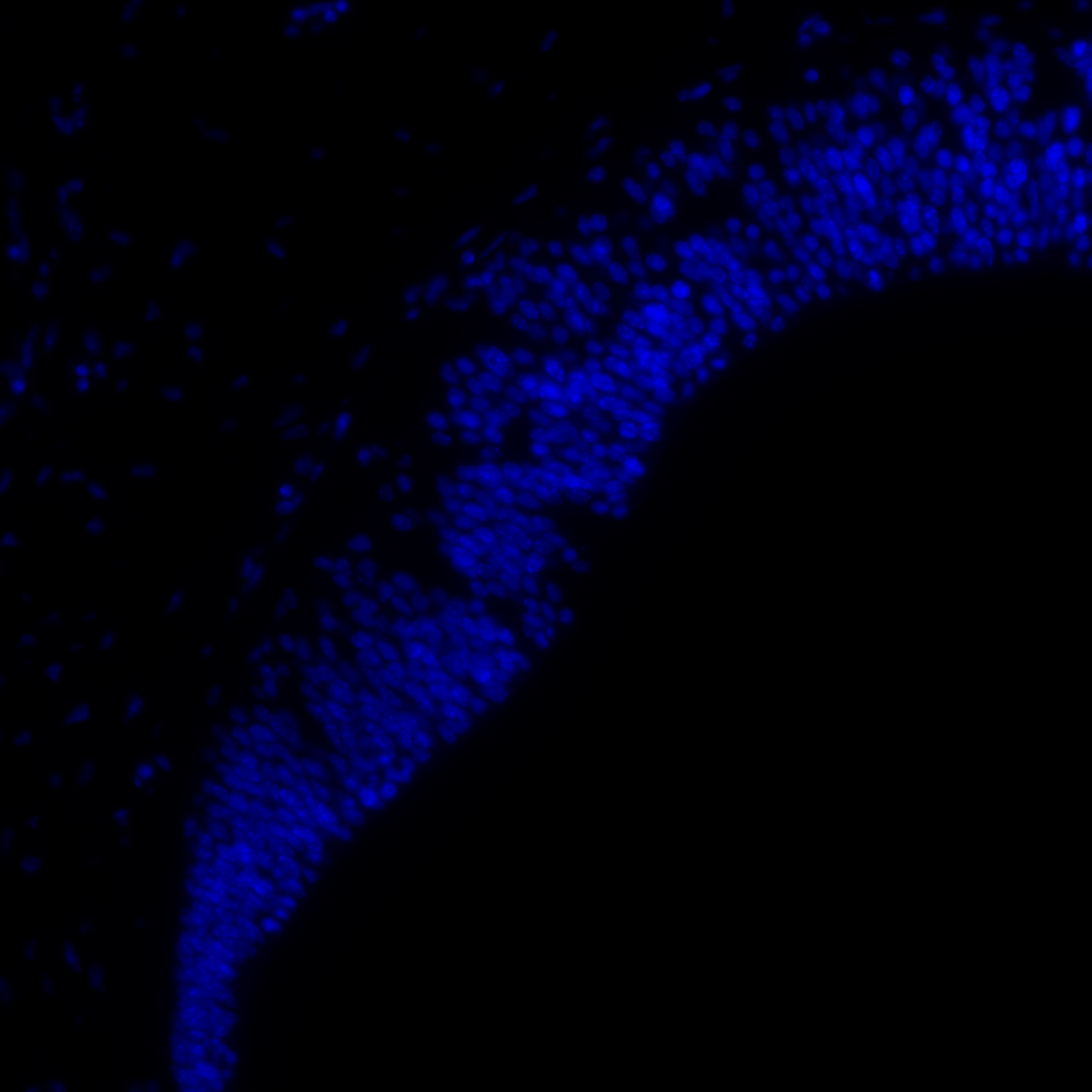

Supplement: Figure 5—source data 3. [file elife-86940-fig5-data3.zip › Figure 5-source data 3/F8871-1-DKO-E11.5-RX FF ff-40X-gLhx2-21-1-R-Image Export-69_DAPI.jpg]

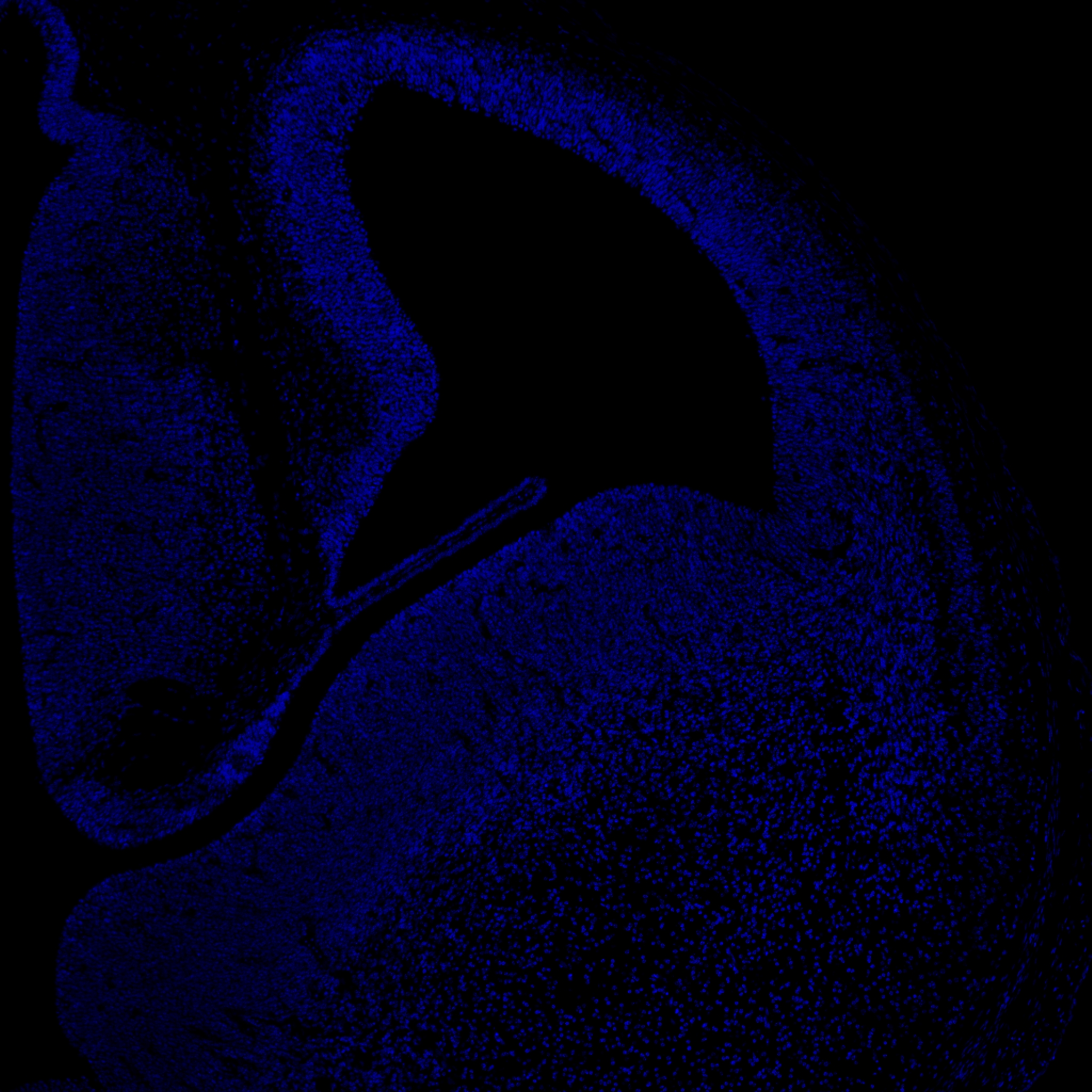

Supplement: Figure 5—source data 3. [file elife-86940-fig5-data3.zip › Figure 5-source data 3/F6091-5-CON-E13.5-FF f+-10X-Lhx5-30-4-R-Image Export-18_DAPI.jpg]

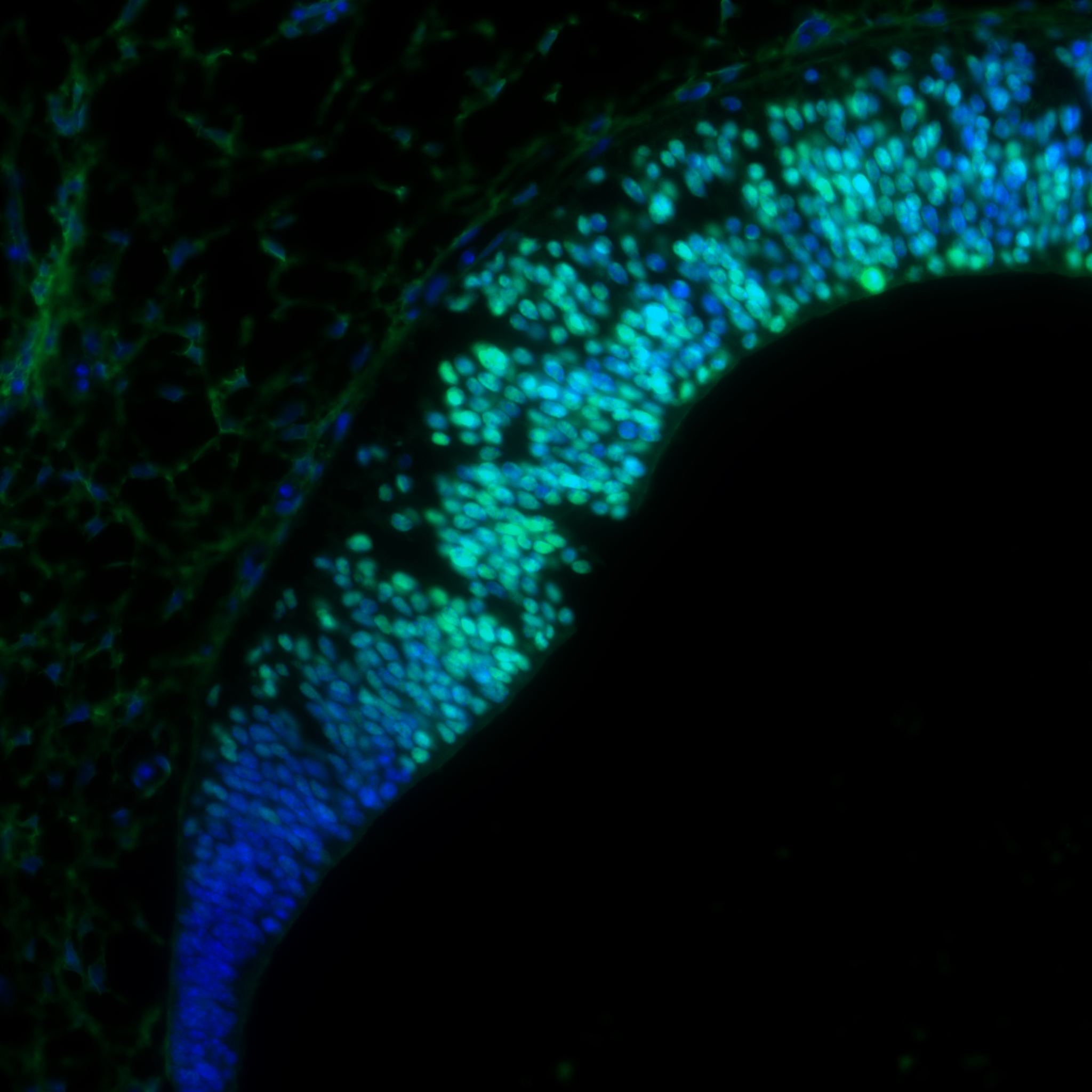

Supplement: Figure 5—source data 3. [file elife-86940-fig5-data3.zip › Figure 5-source data 3/F8871-1-DKO-E11.5-RX FF ff-40X-gLhx2-21-1-R-Image Export-69.jpg]

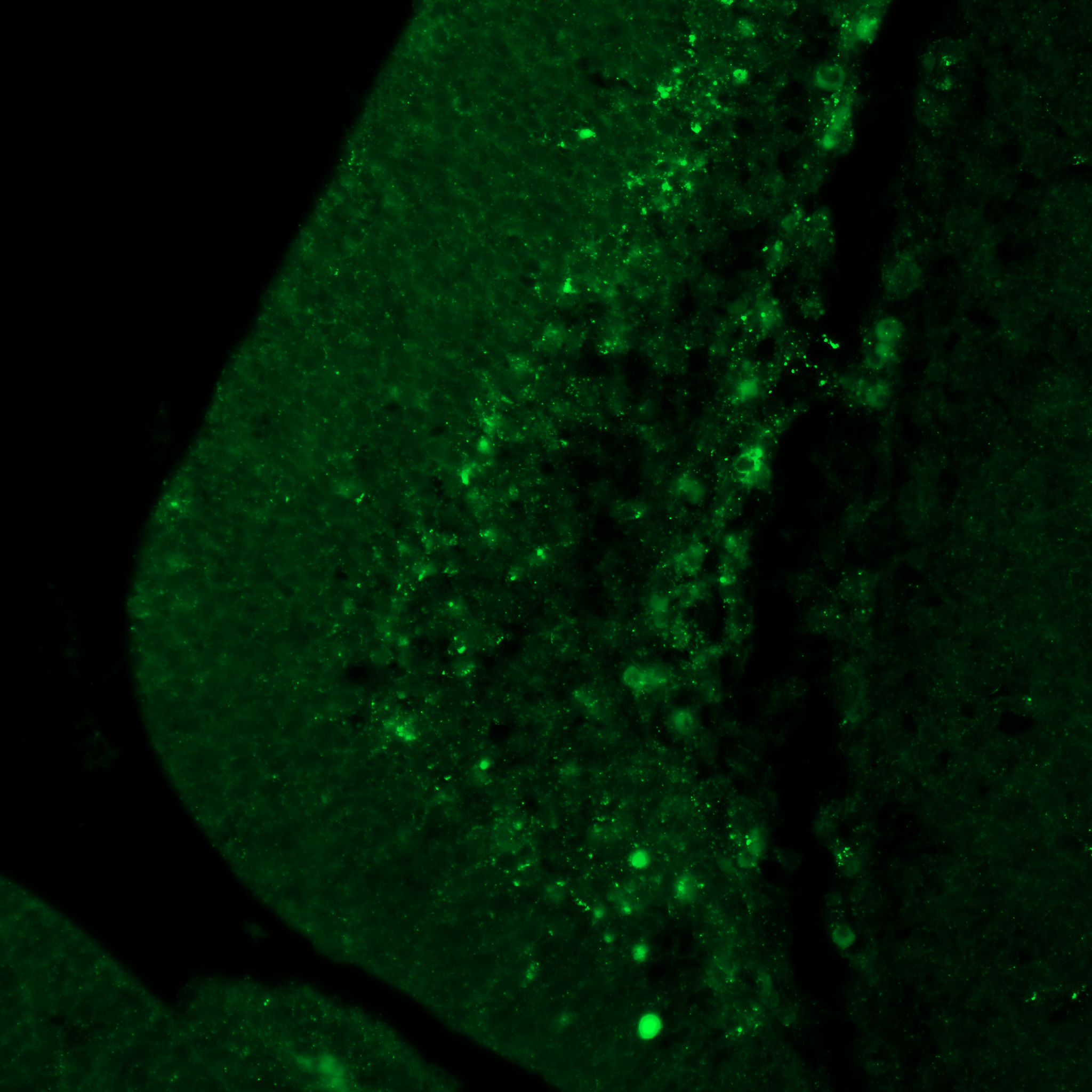

Supplement: Figure 5—source data 3. [file elife-86940-fig5-data3.zip › Figure 5-source data 3/F5734-3-E14.5-DKO-RX ff FF-40X-TBR2-30-1-L-Image Export-33_AF488.tif]

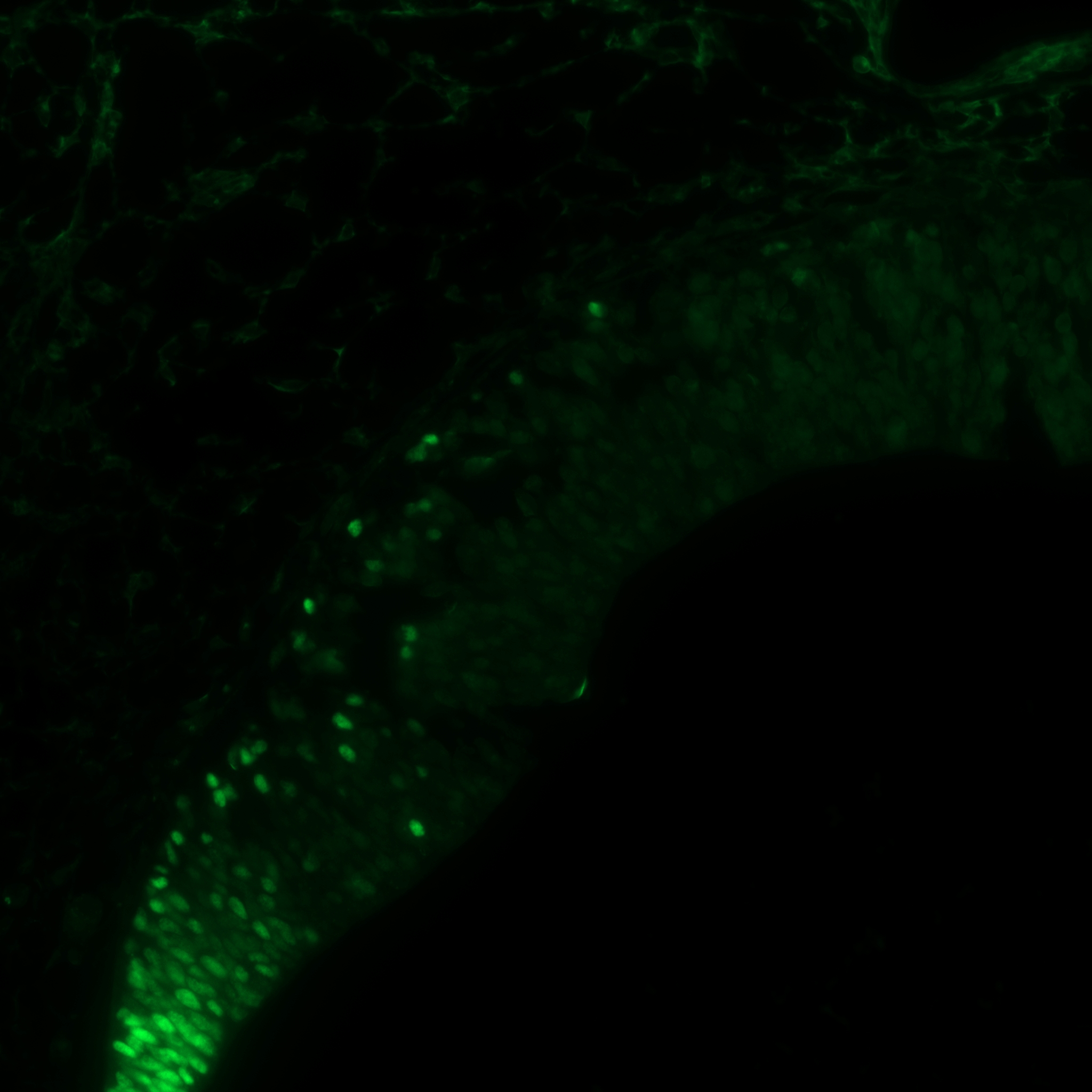

Supplement: Figure 5—source data 3. [file elife-86940-fig5-data3.zip › Figure 5-source data 3/F8871-1-DKO-E11.5-RX FF ff-40X-gLhx5-21-4-R-MP MIGRATION-Image Export-79_AF488.jpg]

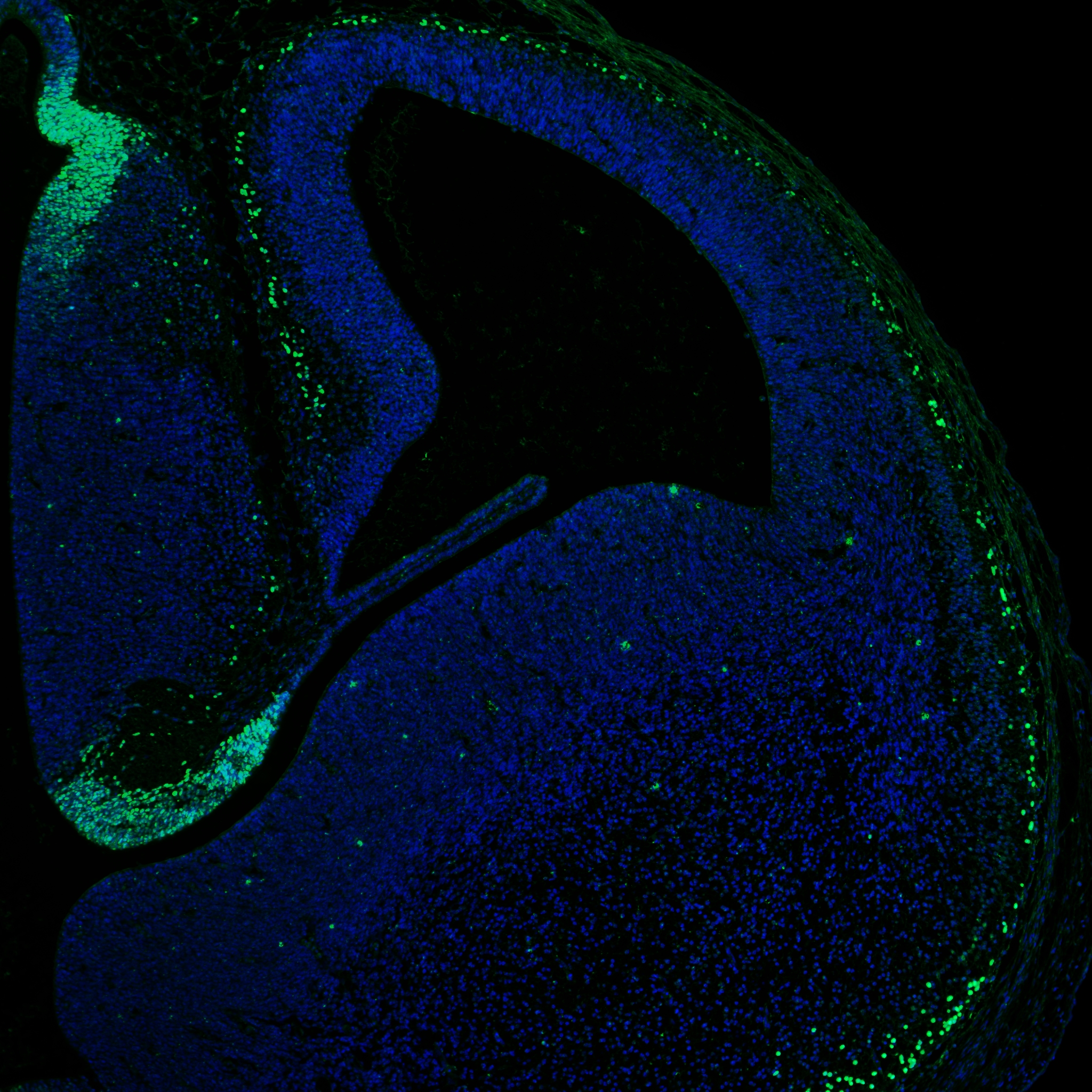

Supplement: Figure 5—source data 3. [file elife-86940-fig5-data3.zip › Figure 5-source data 3/F6091-5-CON-E13.5-FF f+-10X-Lhx5-30-4-R-Image Export-18.jpg]

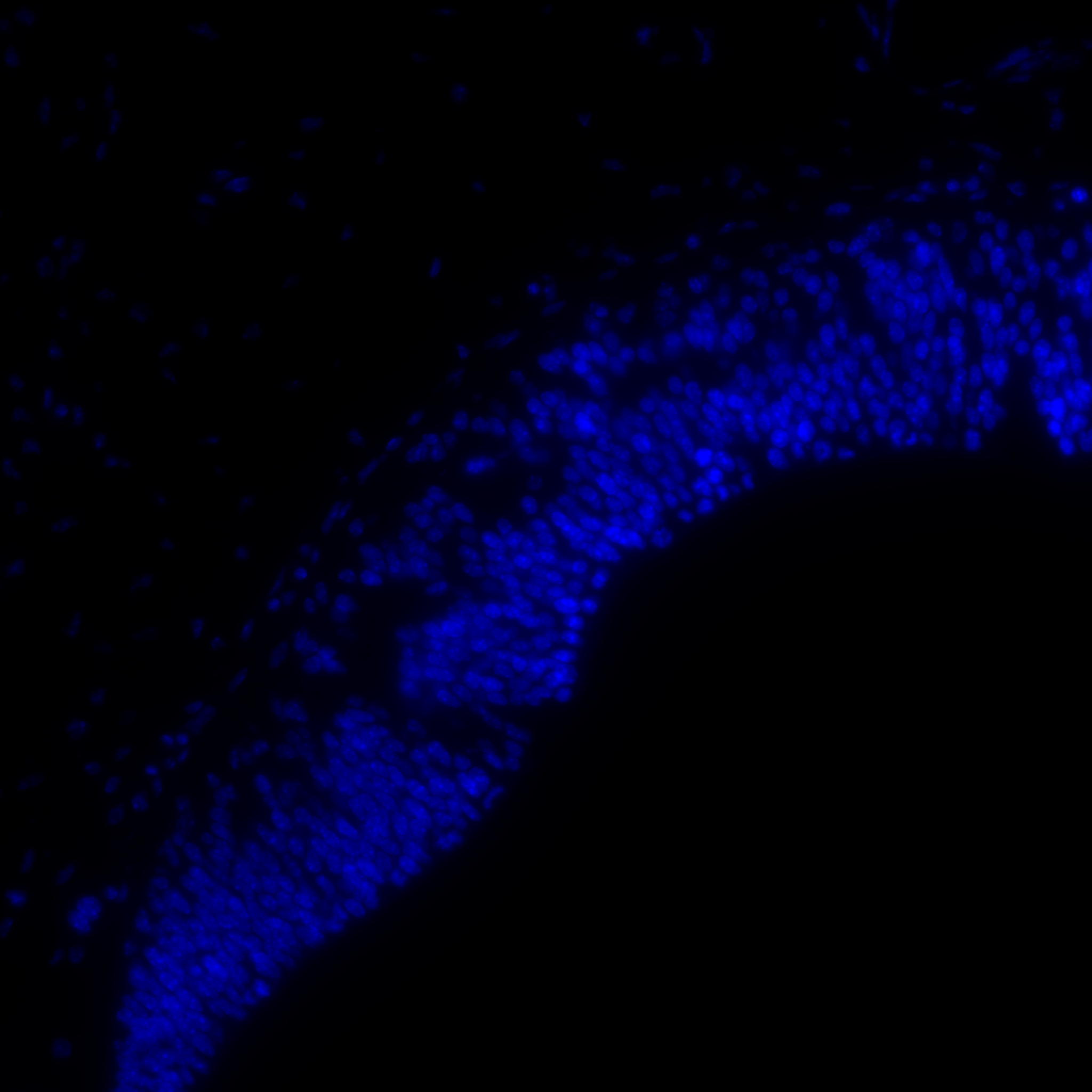

Supplement: Figure 5—source data 3. [file elife-86940-fig5-data3.zip › Figure 5-source data 3/F8871-1-DKO-E11.5-RX FF ff-40X-gLhx5-21-4-R-MP MIGRATION-Image Export-79_DAPI.jpg]

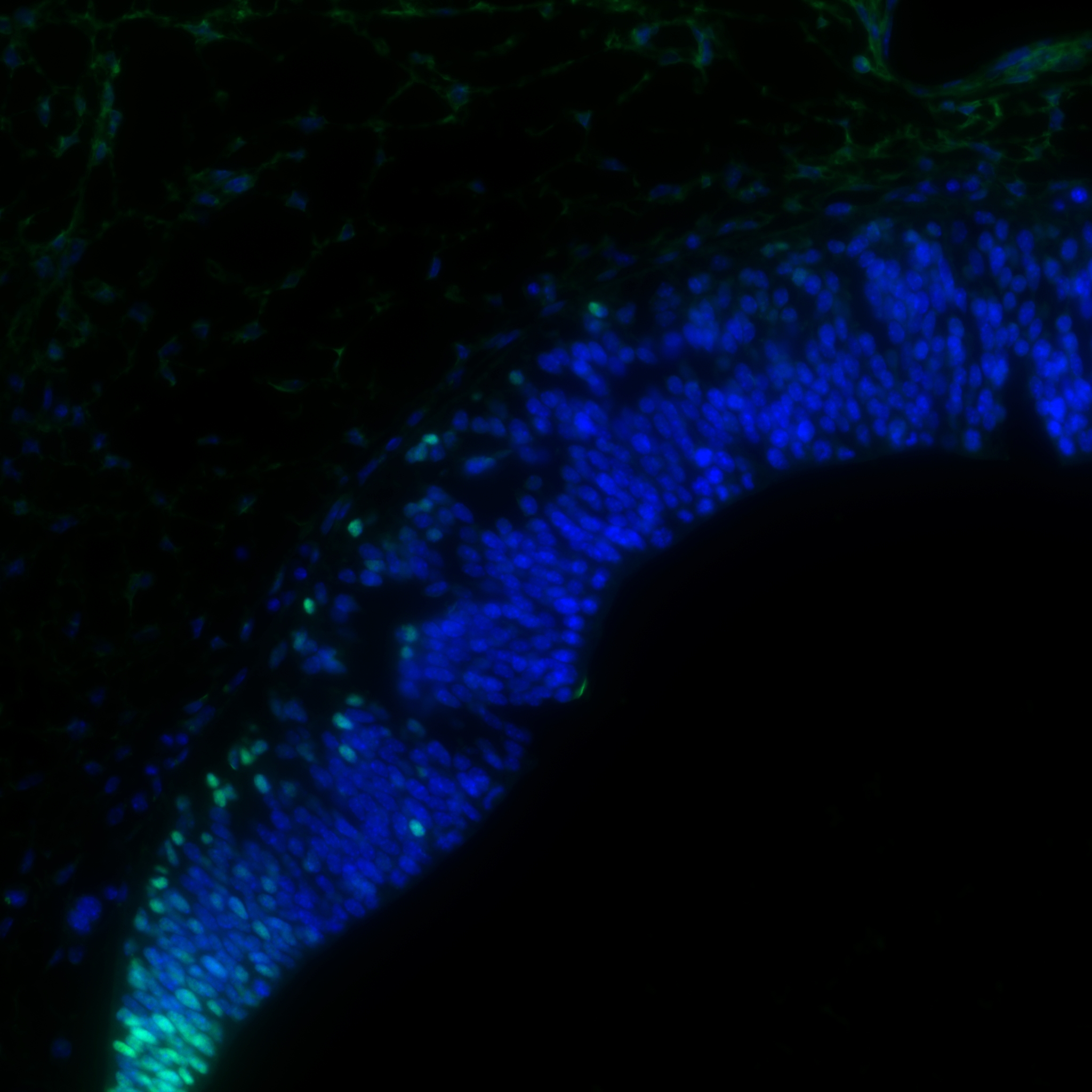

Supplement: Figure 5—source data 3. [file elife-86940-fig5-data3.zip › Figure 5-source data 3/F8871-1-DKO-E11.5-RX FF ff-40X-gLhx5-21-4-R-MP MIGRATION-Image Export-79.jpg]

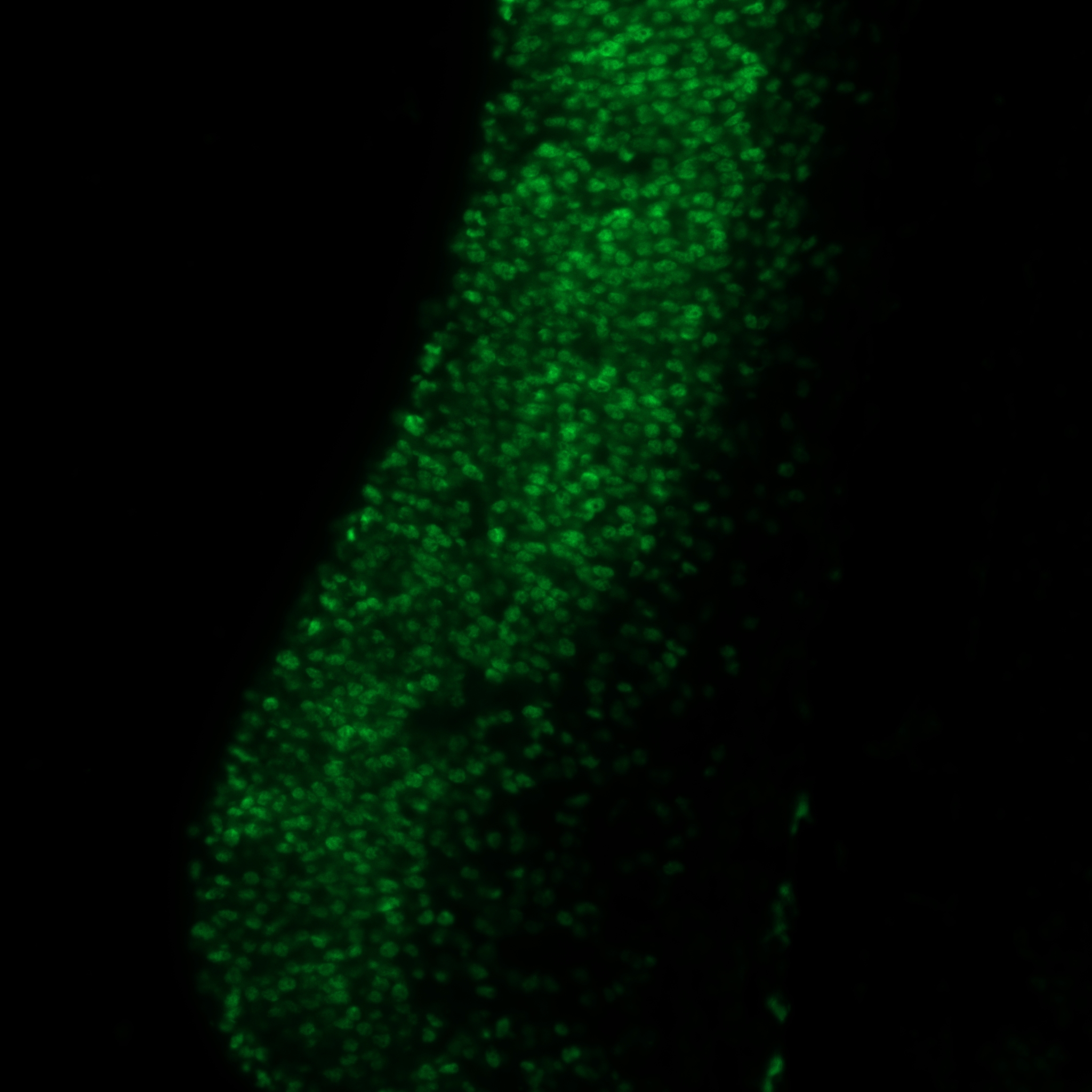

Supplement: Figure 5—source data 3. [file elife-86940-fig5-data3.zip › Figure 5-source data 3/F6091-5-CON-E13.5-FF f+-40X-Lhx2-30-1-L-MP-Image Export-04_AF488.jpg]

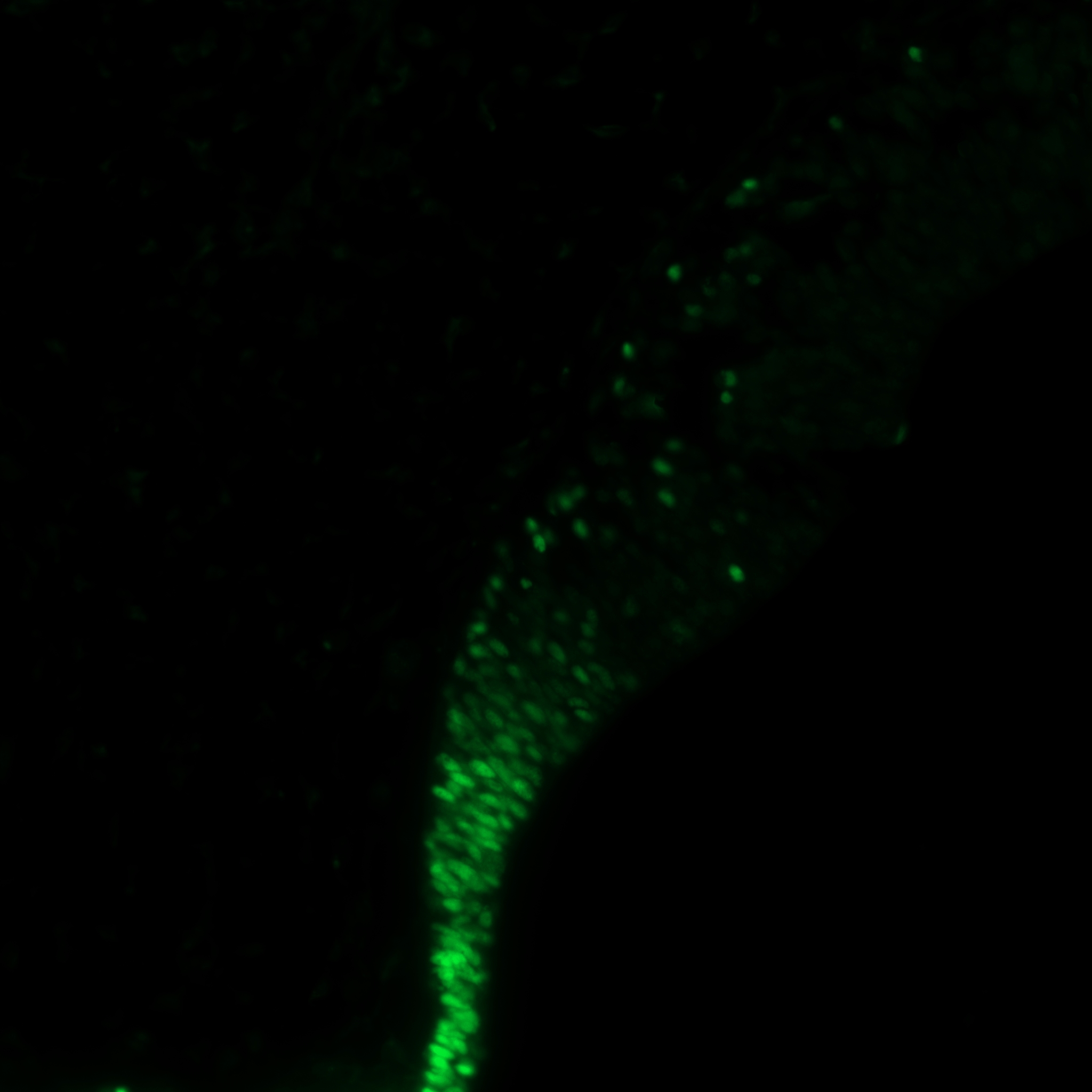

Supplement: Figure 5—source data 3. [file elife-86940-fig5-data3.zip › Figure 5-source data 3/F8871-1-DKO-E11.5-RX FF ff-40X-gLhx5-21-4-R-MP-Image Export-78_AF488.jpg]

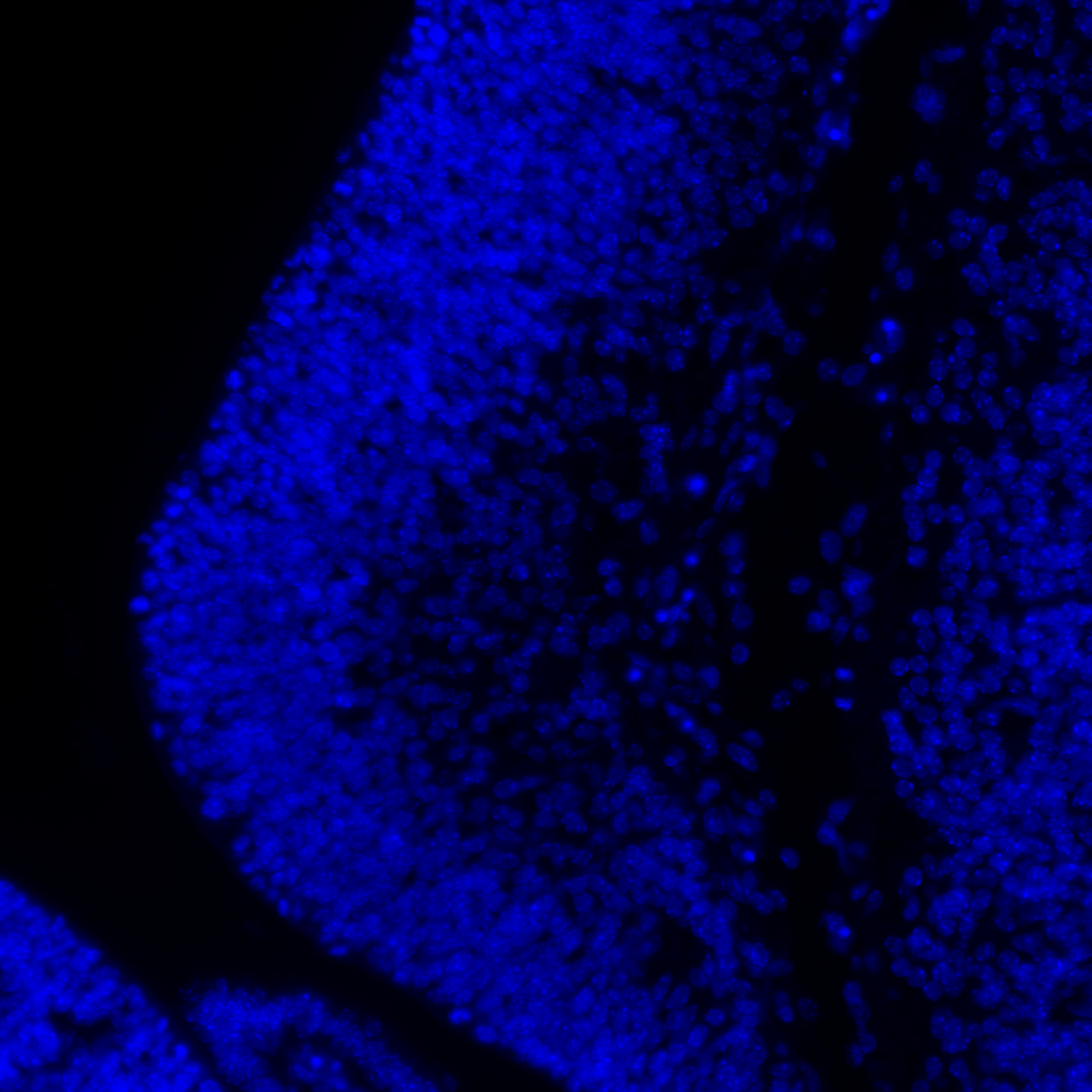

Supplement: Figure 5—source data 3. [file elife-86940-fig5-data3.zip › Figure 5-source data 3/F5734-3-E14.5-DKO-RX ff FF-40X-TBR2-30-1-L-Image Export-33_DAPI.tif]

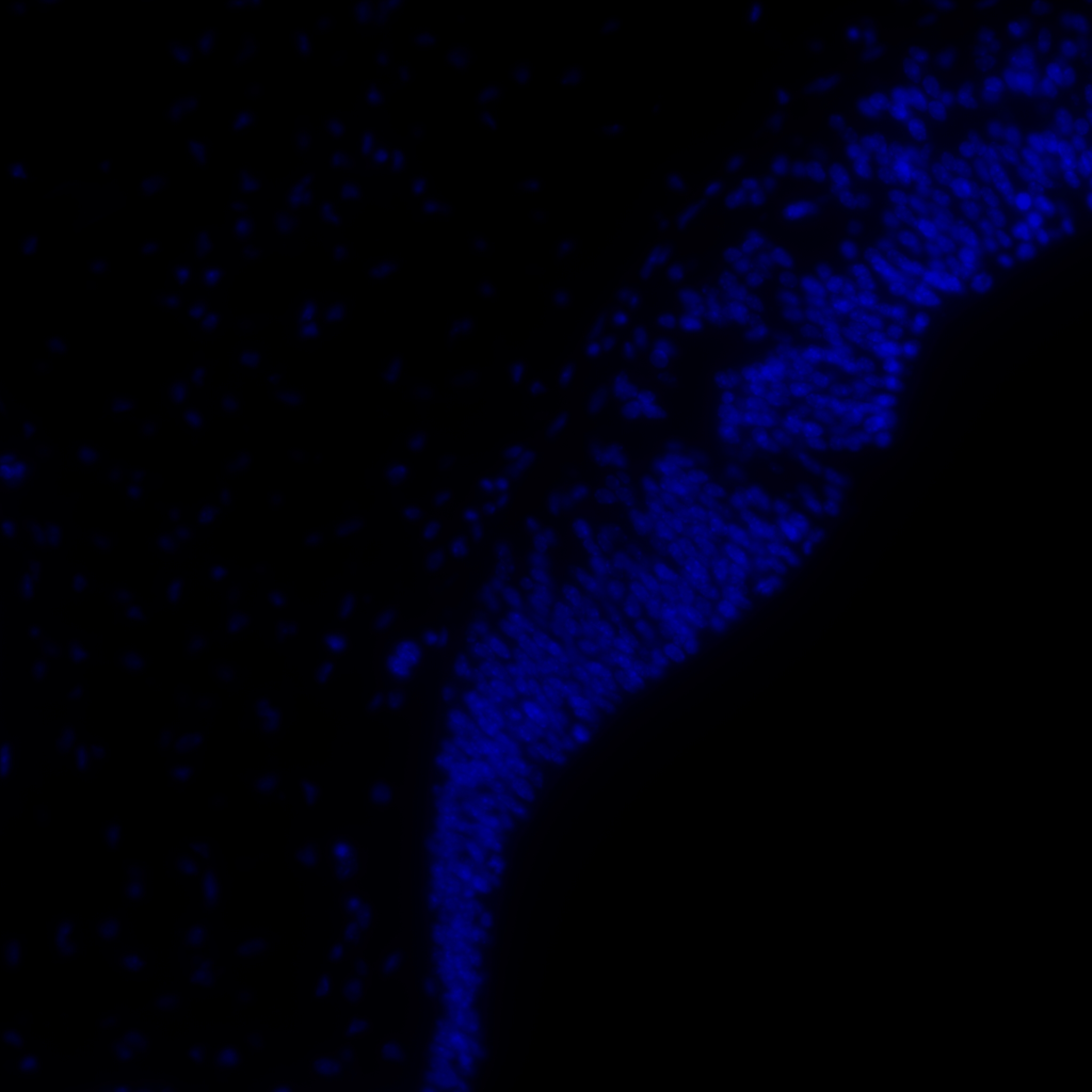

Supplement: Figure 5—source data 3. [file elife-86940-fig5-data3.zip › Figure 5-source data 3/F8871-1-DKO-E11.5-RX FF ff-40X-gLhx5-21-4-R-MP-Image Export-78_DAPI.jpg]

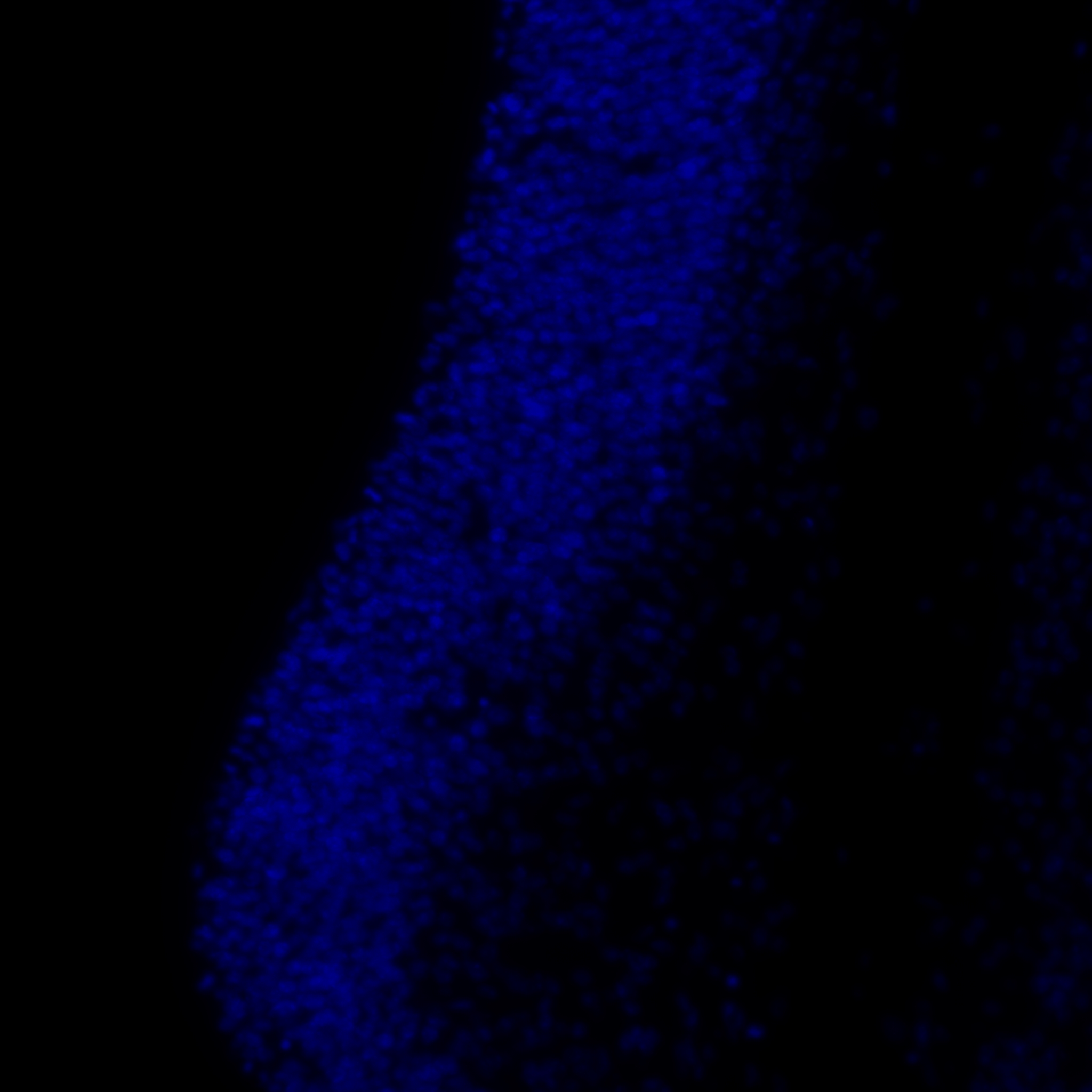

Supplement: Figure 5—source data 3. [file elife-86940-fig5-data3.zip › Figure 5-source data 3/F6091-5-CON-E13.5-FF f+-40X-Lhx2-30-1-L-MP-Image Export-04_DAPI.jpg]

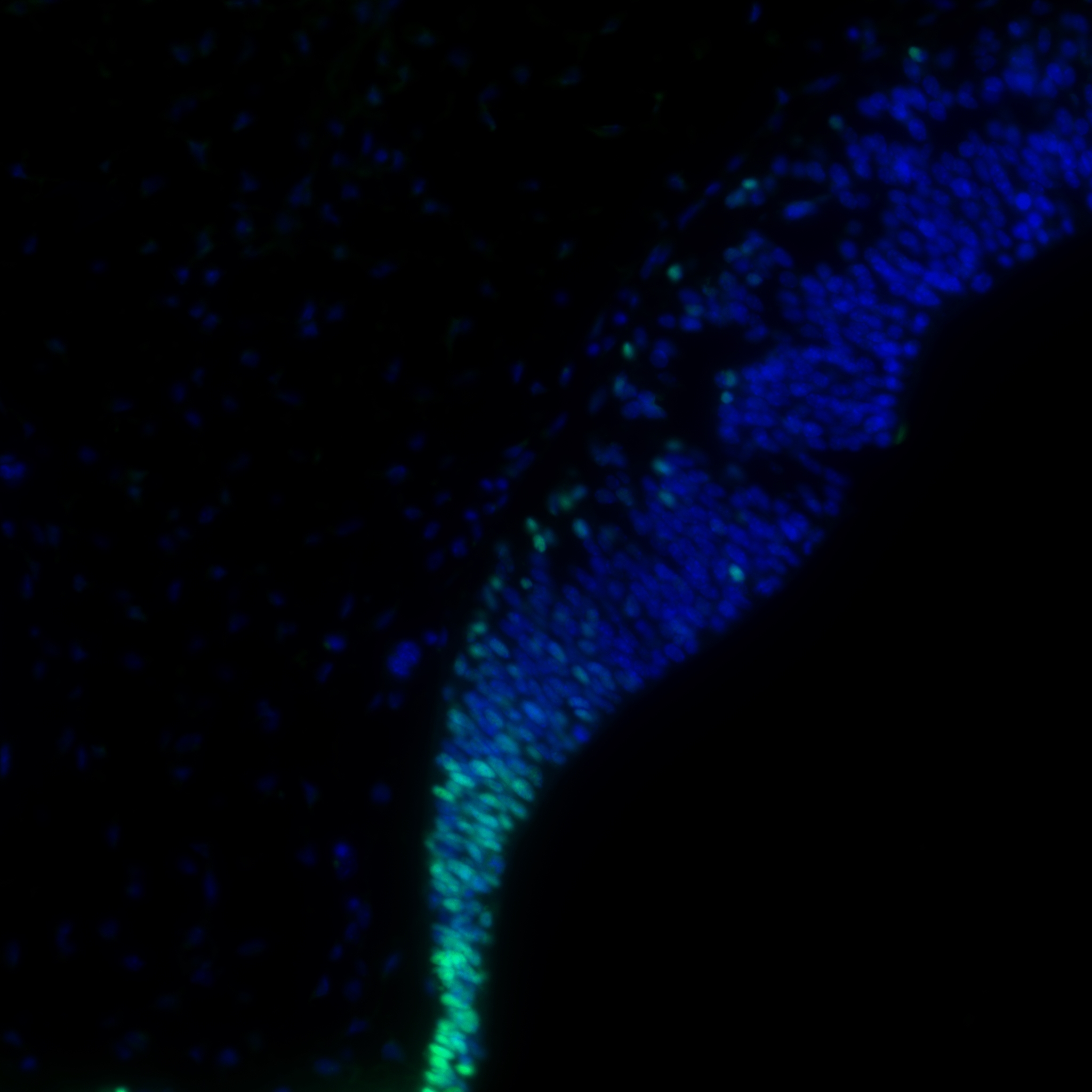

Supplement: Figure 5—source data 3. [file elife-86940-fig5-data3.zip › Figure 5-source data 3/F8871-1-DKO-E11.5-RX FF ff-40X-gLhx5-21-4-R-MP-Image Export-78.jpg]

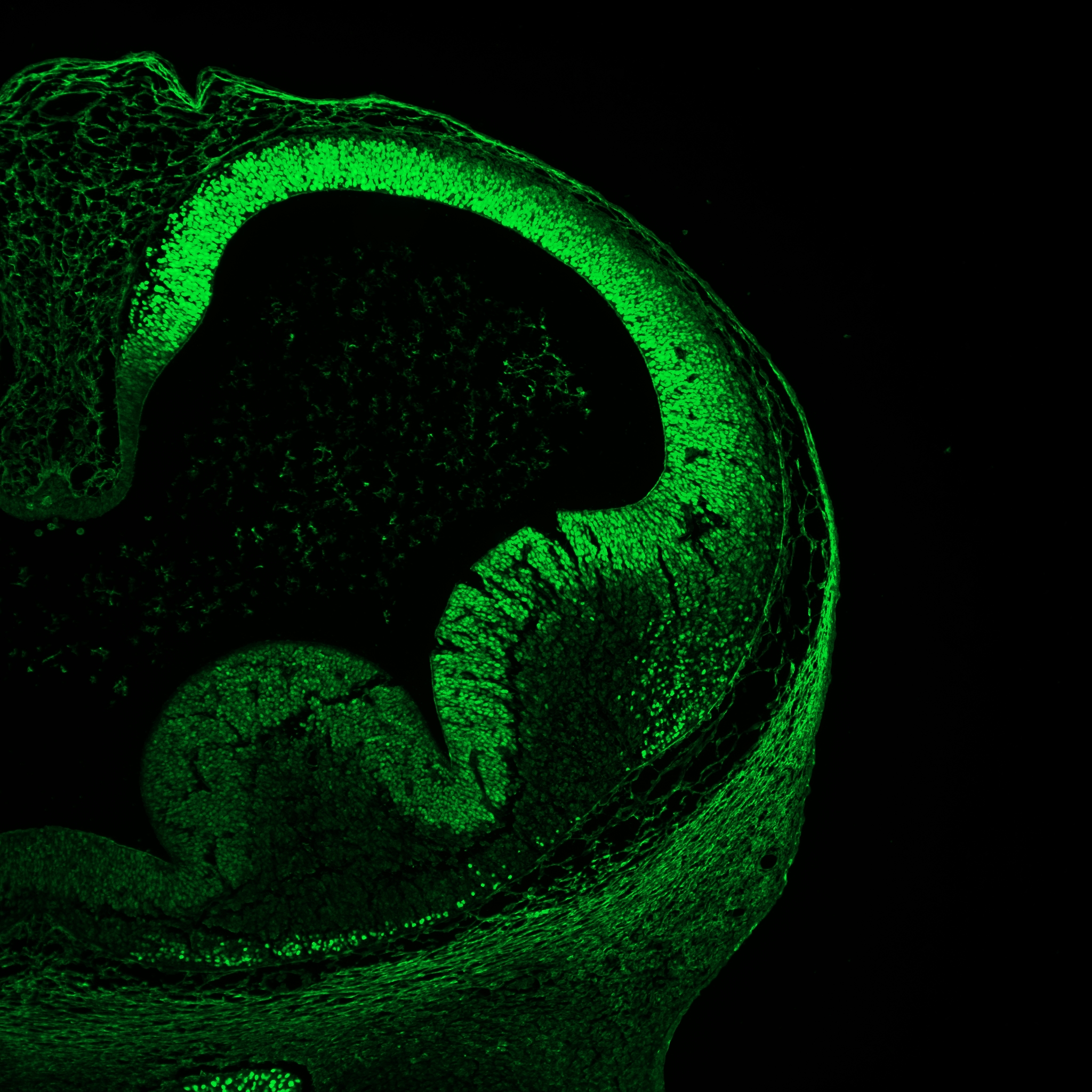

Supplement: Figure 5—source data 3. [file elife-86940-fig5-data3.zip › Figure 5-source data 3/F8871-2-CON-E11.5-F+ ff-10X-gLhx2-22-2-R-Image Export-42_AF488.jpg]

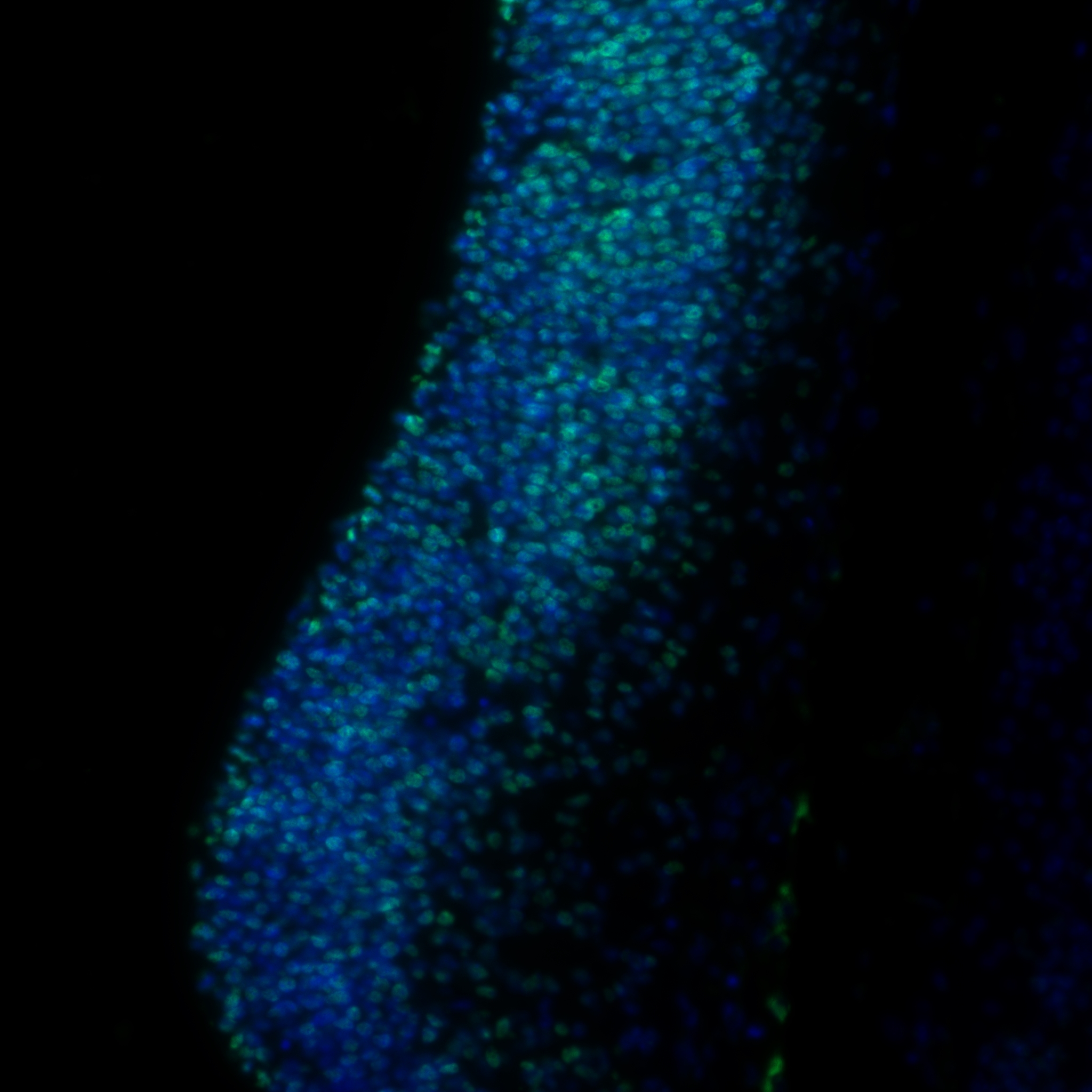

Supplement: Figure 5—source data 3. [file elife-86940-fig5-data3.zip › Figure 5-source data 3/F6091-5-CON-E13.5-FF f+-40X-Lhx2-30-1-L-MP-Image Export-04.jpg]

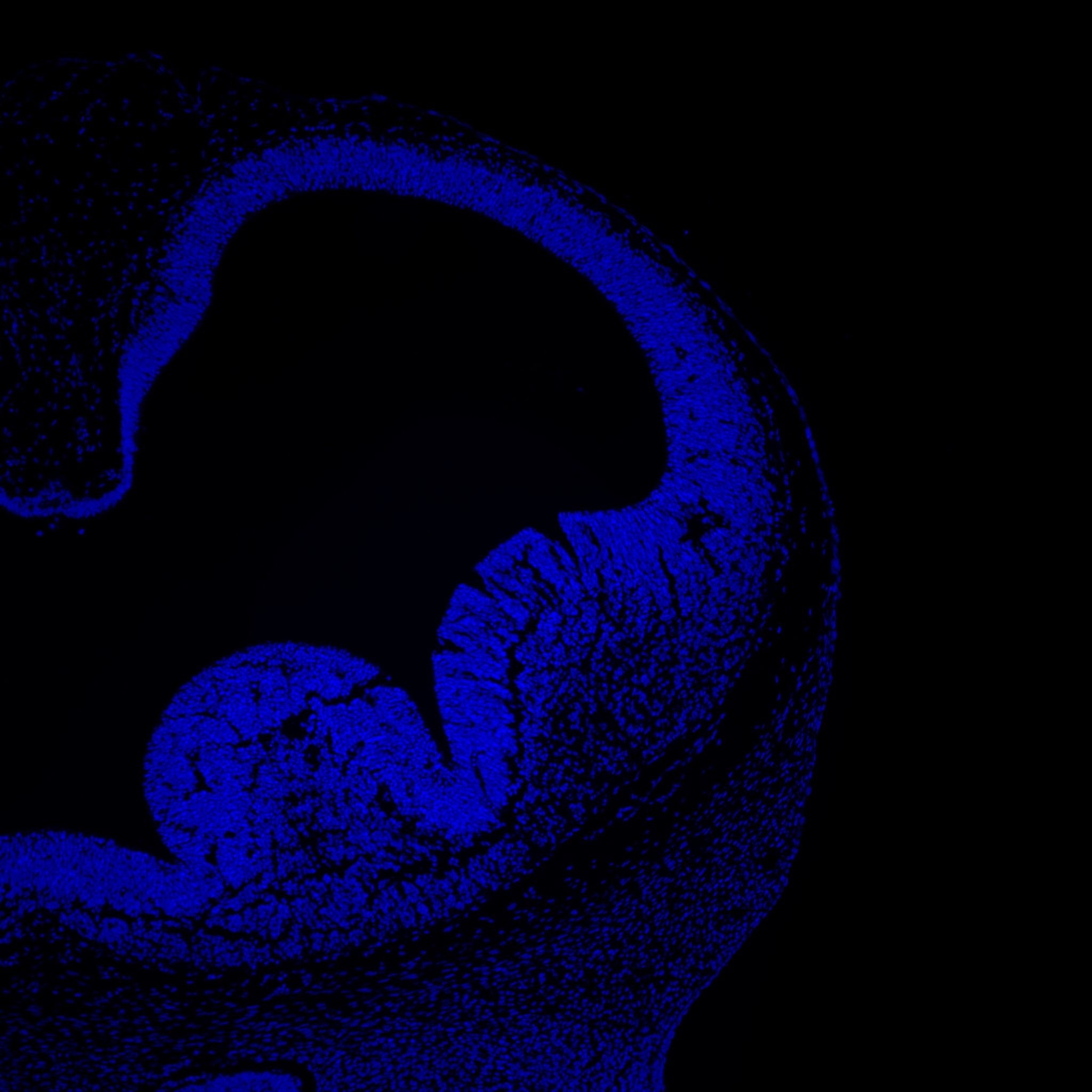

Supplement: Figure 5—source data 3. [file elife-86940-fig5-data3.zip › Figure 5-source data 3/F8871-2-CON-E11.5-F+ ff-10X-gLhx2-22-2-R-Image Export-42_DAPI.jpg]

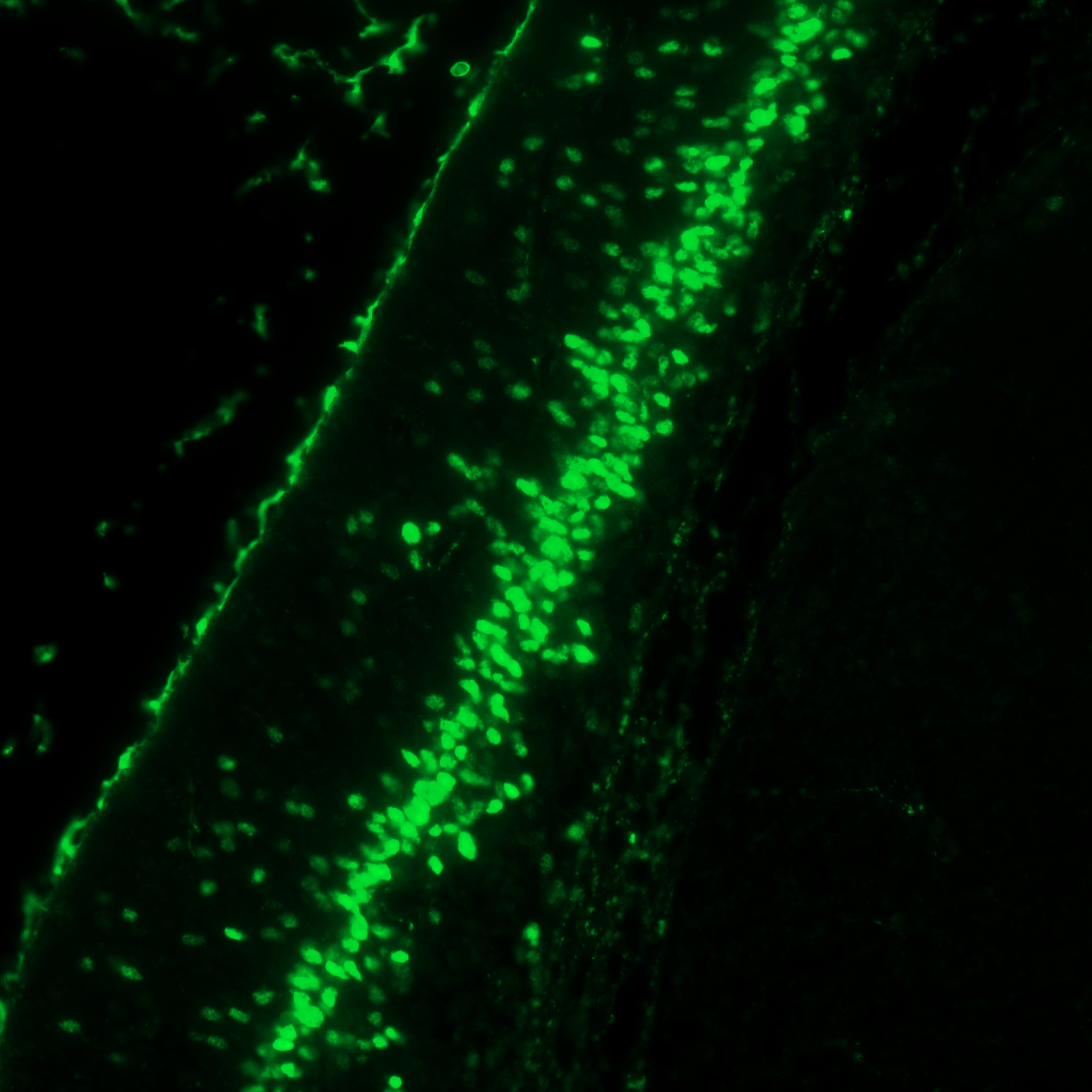

Supplement: Figure 5—source data 3. [file elife-86940-fig5-data3.zip › Figure 5-source data 3/F5734-5-CON-E14.5-RX f+ F+-40X-TBR2-22-3-L-Image Export-25_AF488.tif]

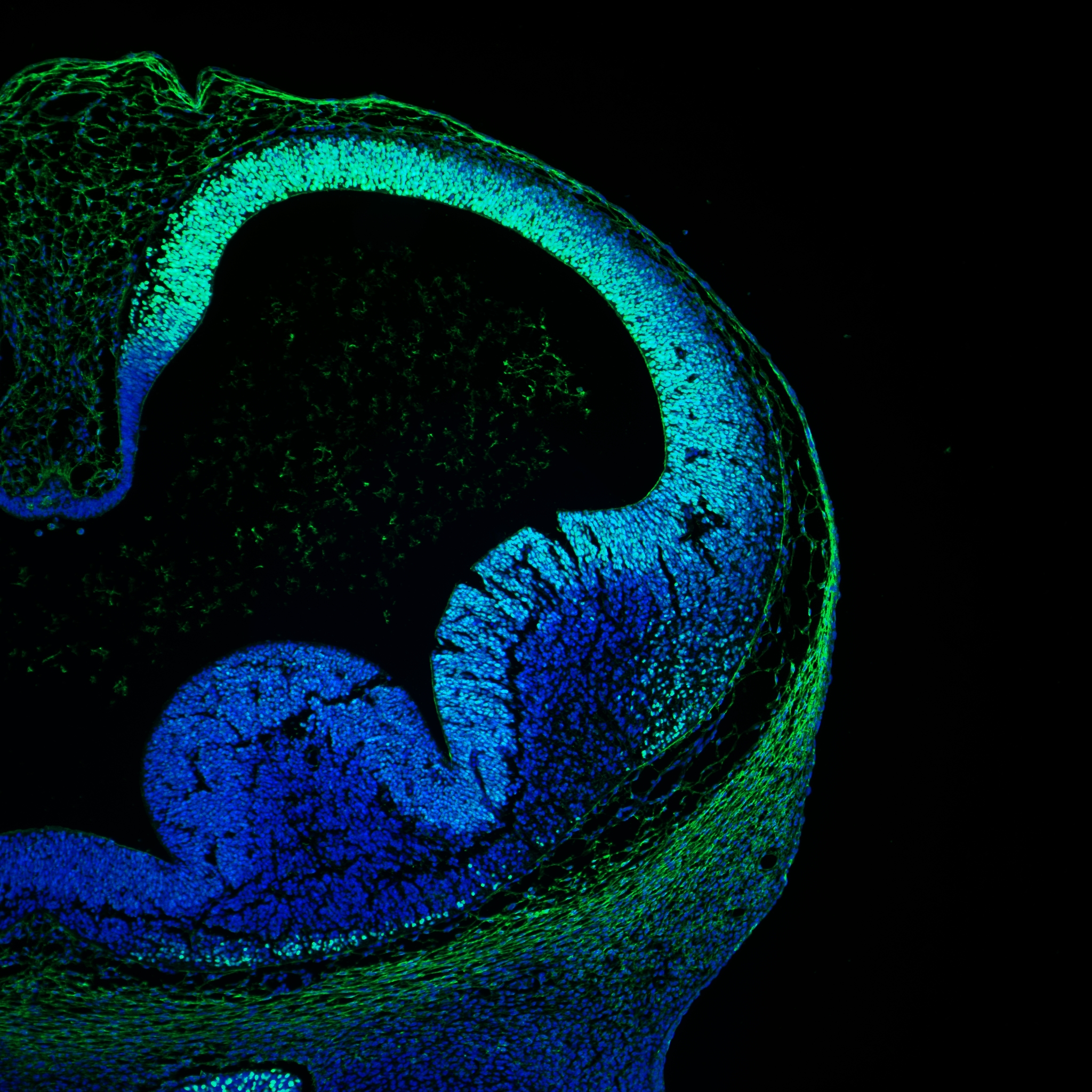

Supplement: Figure 5—source data 3. [file elife-86940-fig5-data3.zip › Figure 5-source data 3/F8871-2-CON-E11.5-F+ ff-10X-gLhx2-22-2-R-Image Export-42.jpg]

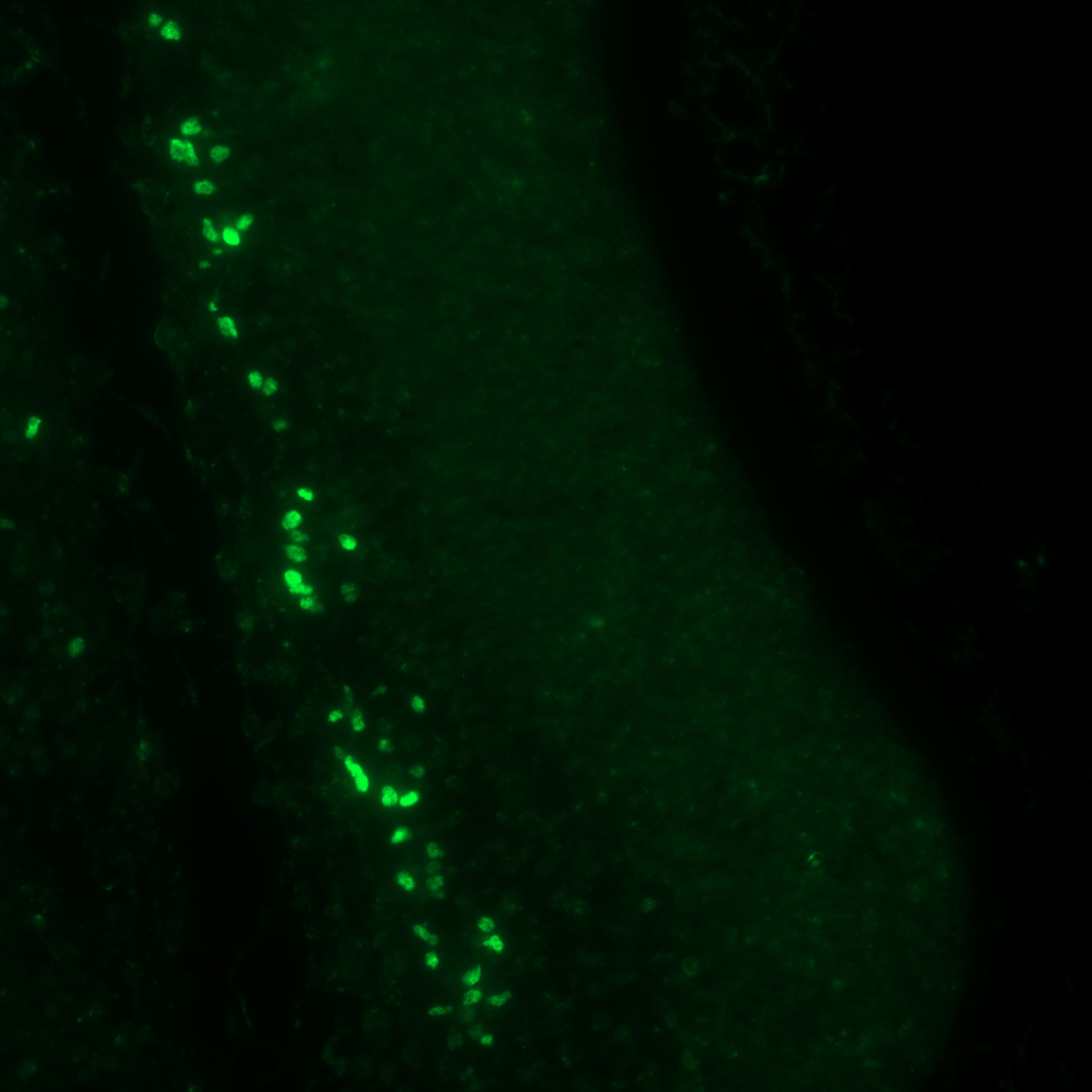

Supplement: Figure 5—source data 3. [file elife-86940-fig5-data3.zip › Figure 5-source data 3/F6091-5-CON-E13.5-FF f+-40X-Lhx5-30-4-R-MP-Image Export-20_AF488.jpg]

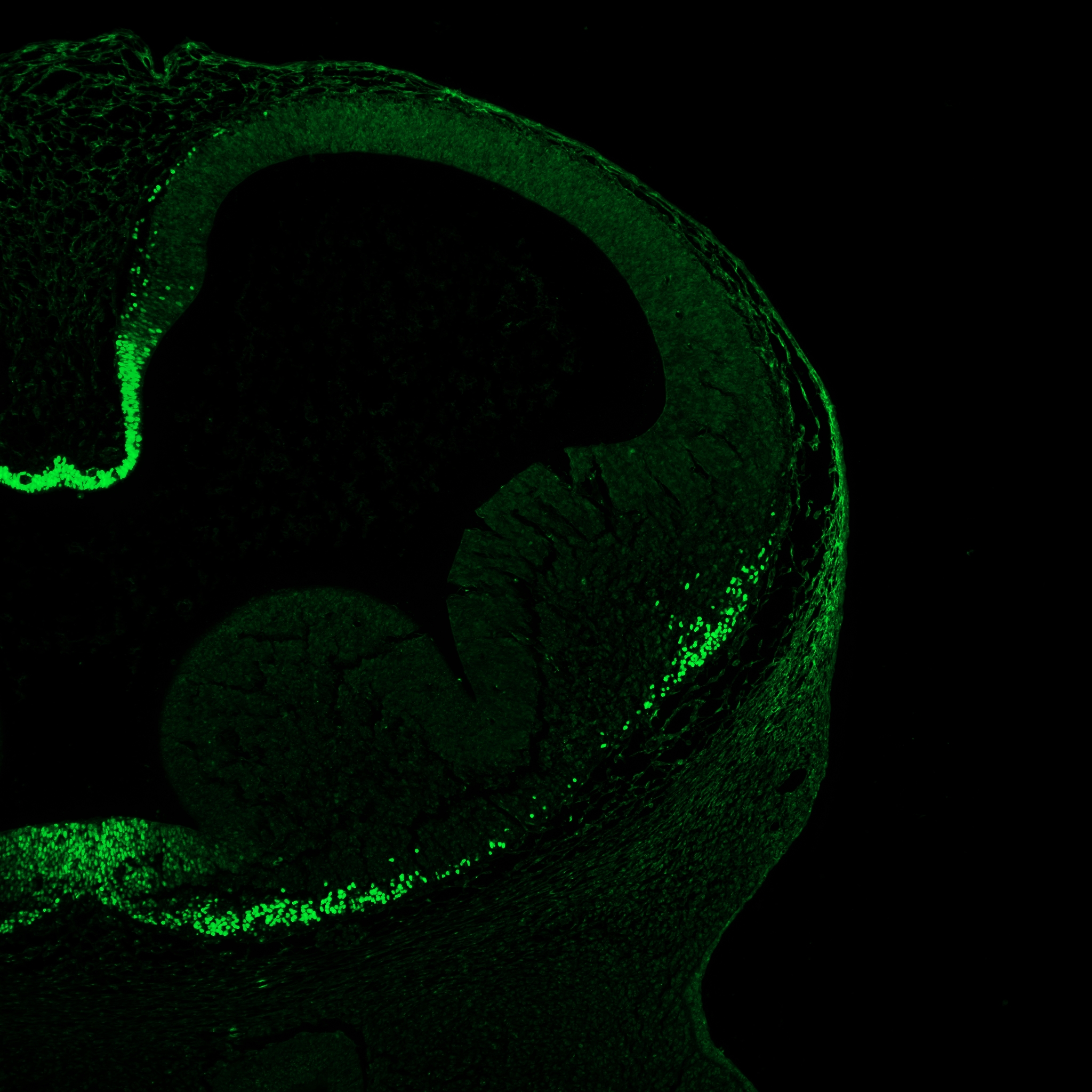

Supplement: Figure 5—source data 3. [file elife-86940-fig5-data3.zip › Figure 5-source data 3/F8871-2-CON-E11.5-F+ ff-10X-gLhx5-22-4-R-Image Export-46_AF488.jpg]

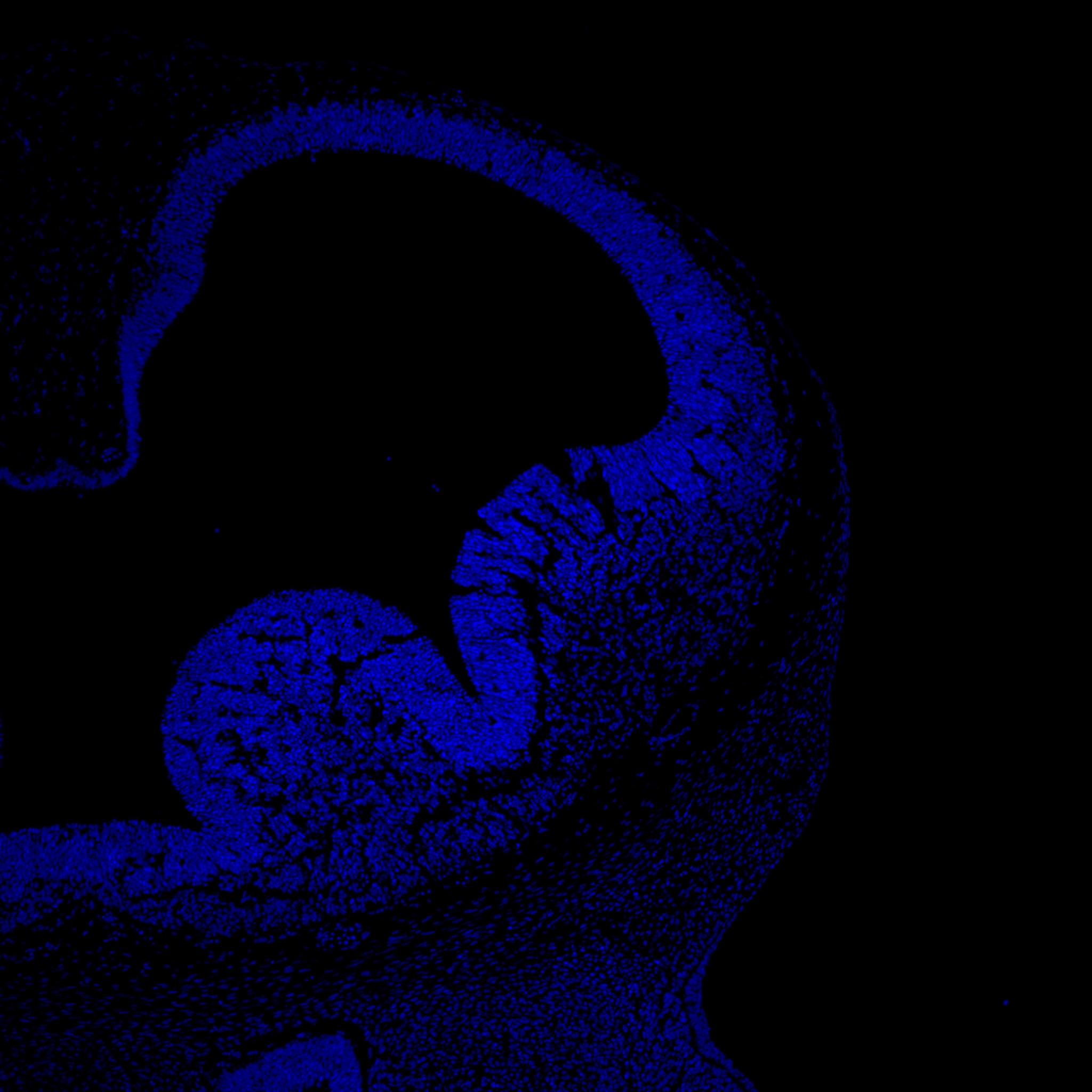

Supplement: Figure 5—source data 3. [file elife-86940-fig5-data3.zip › Figure 5-source data 3/F8871-2-CON-E11.5-F+ ff-10X-gLhx5-22-4-R-Image Export-46_DAPI.jpg]
