# Supplementary figures and images for: The differentiation and integration of the hippocampal dorsoventral axis are controlled by two nuclear receptor genes (part 6 of 6)
Source: eLife. 2023 Sep 26;12:RP86940. doi: 10.7554/eLife.86940 (PMC10522401; doi:10.7554/eLife.86940)

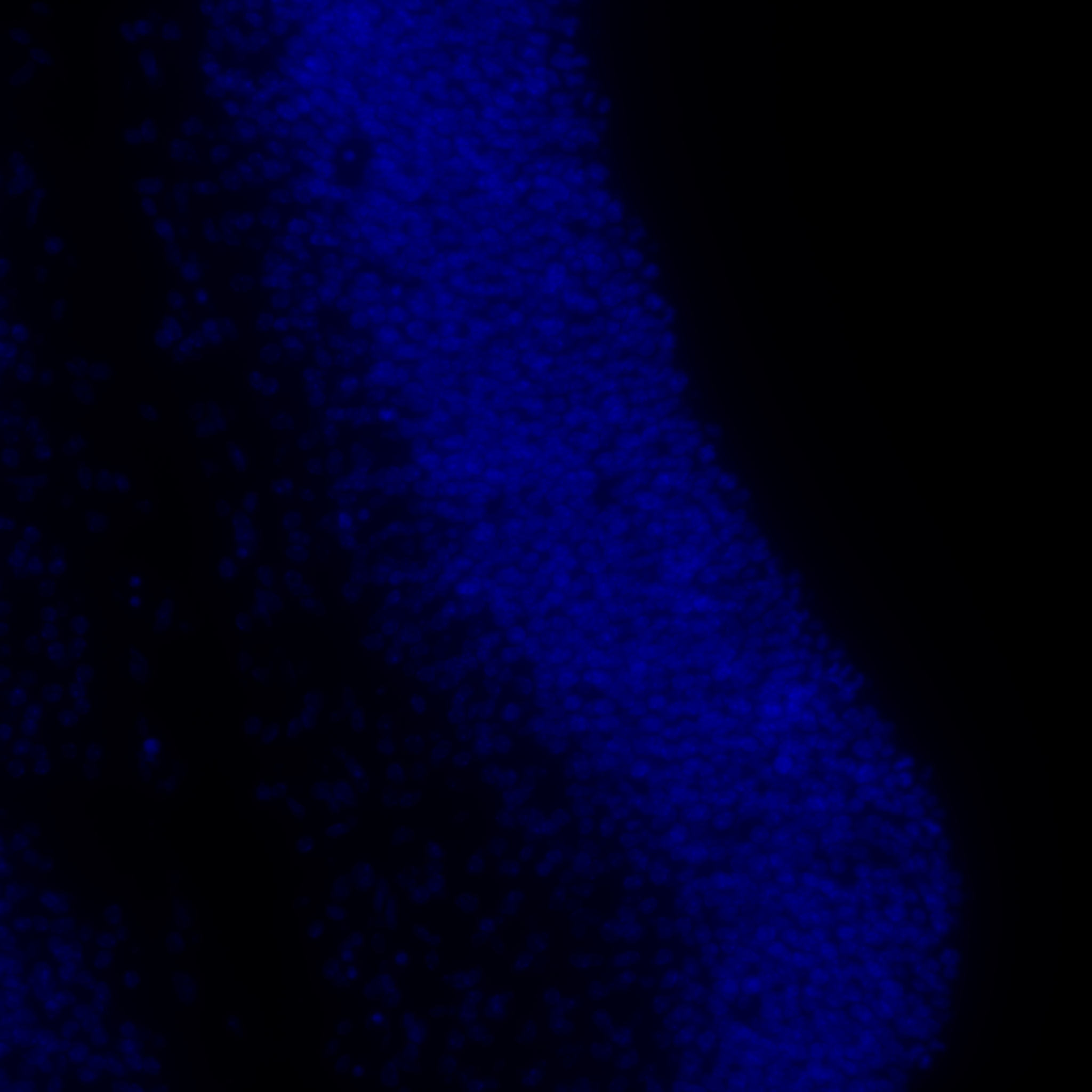

Supplement: Figure 5—source data 3. [file elife-86940-fig5-data3.zip › Figure 5-source data 3/F6091-5-CON-E13.5-FF f+-40X-Lhx5-30-4-R-MP-Image Export-20_DAPI.jpg]

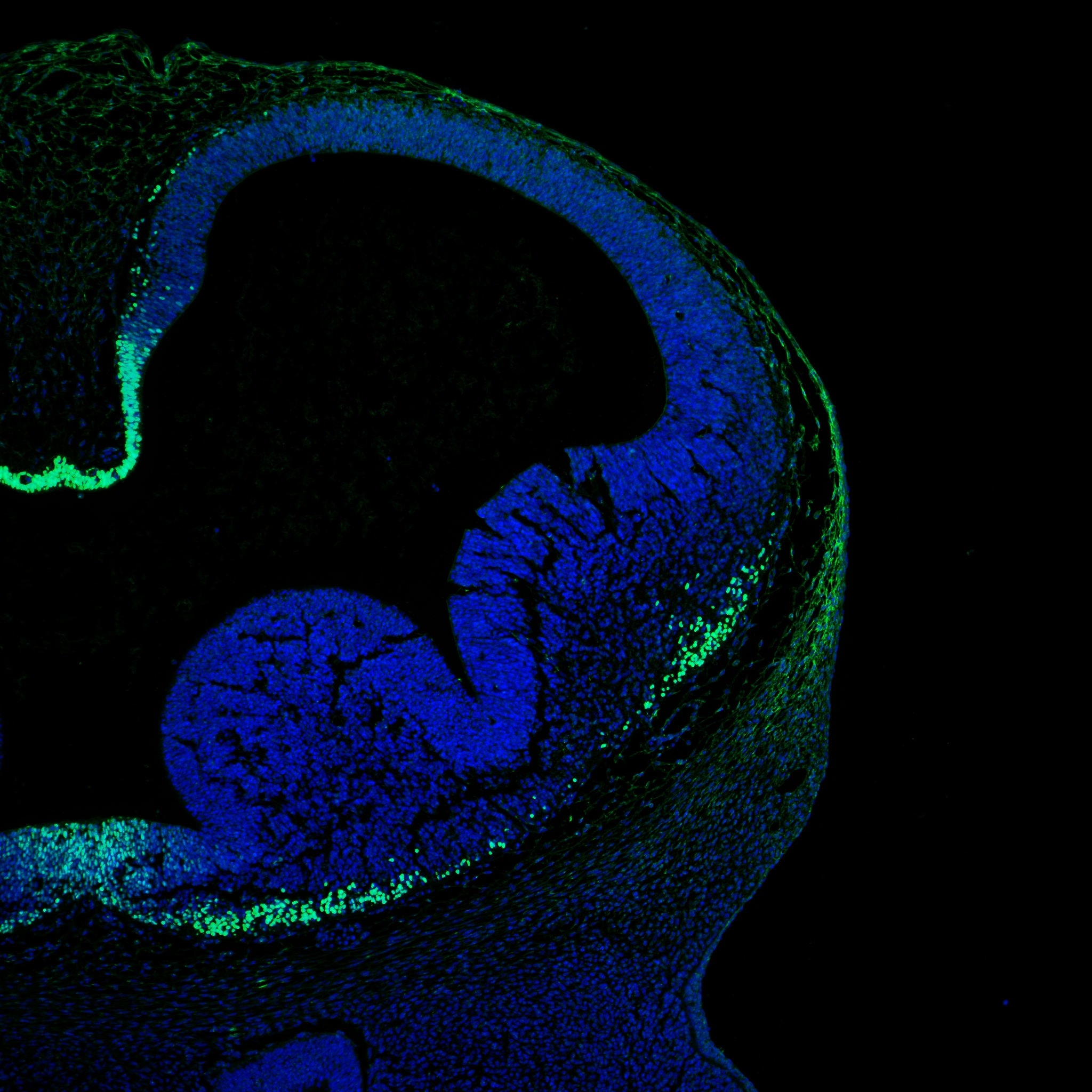

Supplement: Figure 5—source data 3. [file elife-86940-fig5-data3.zip › Figure 5-source data 3/F8871-2-CON-E11.5-F+ ff-10X-gLhx5-22-4-R-Image Export-46.jpg]

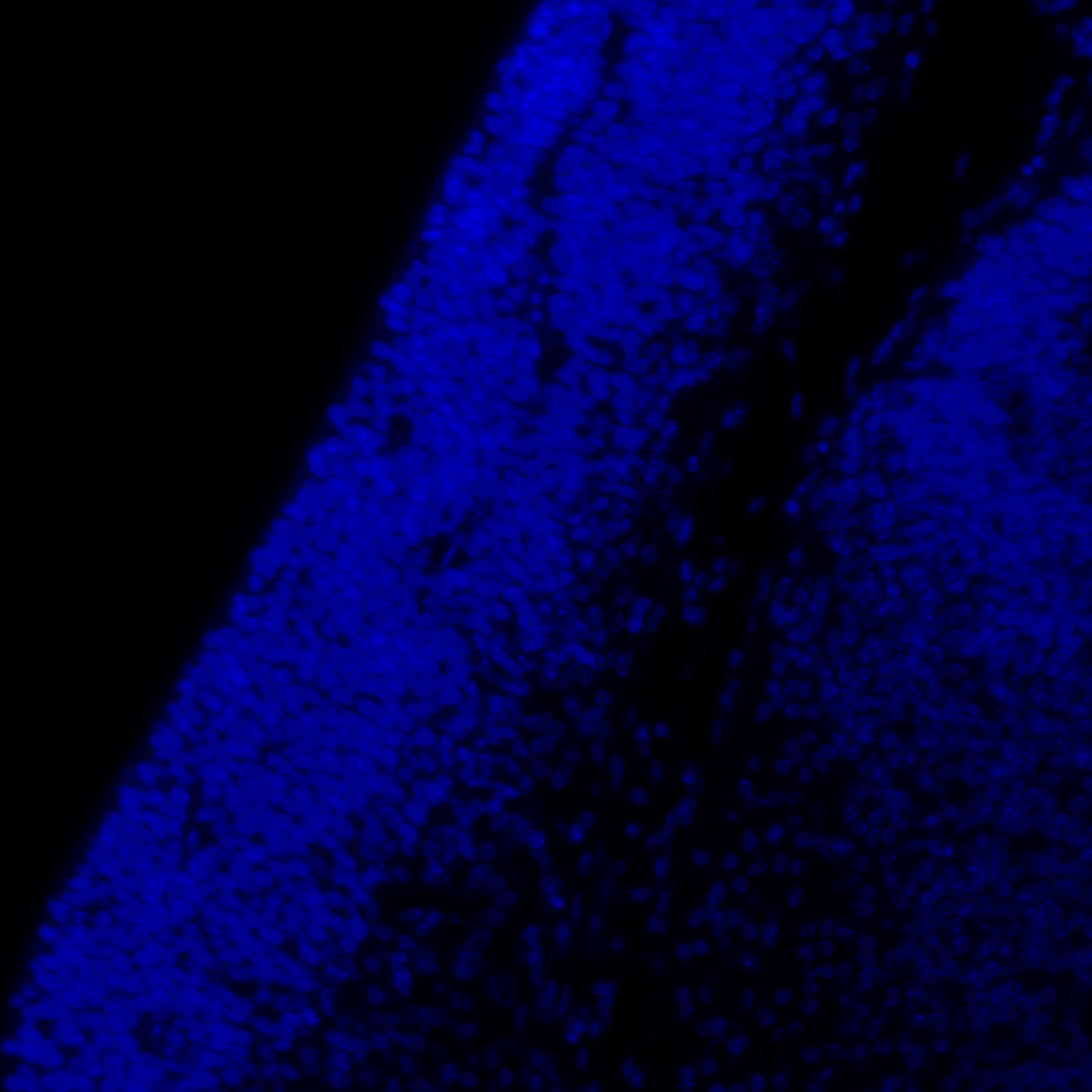

Supplement: Figure 5—source data 3. [file elife-86940-fig5-data3.zip › Figure 5-source data 3/F5734-5-CON-E14.5-RX f+ F+-40X-TBR2-22-3-L-Image Export-25_DAPI.tif]

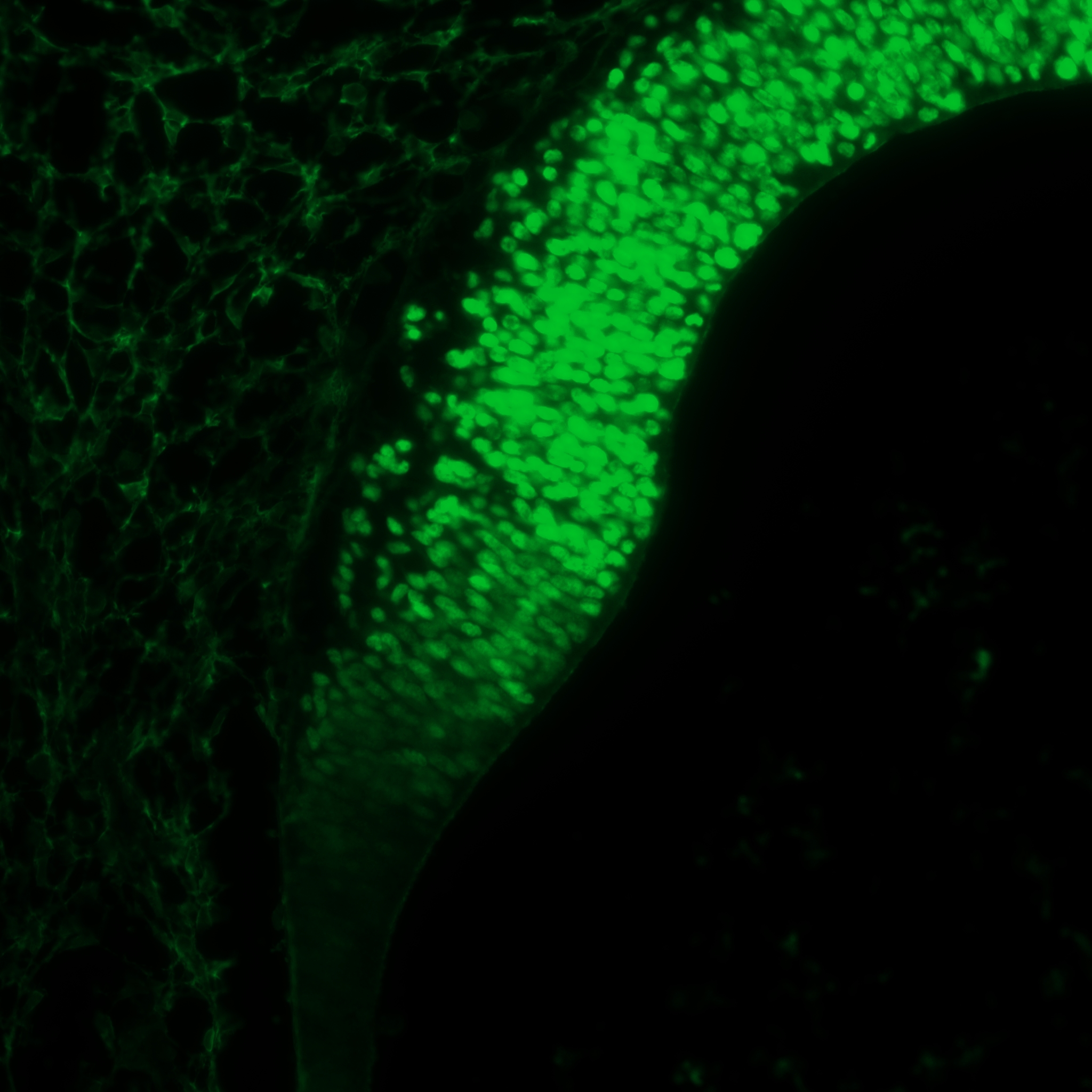

Supplement: Figure 5—source data 3. [file elife-86940-fig5-data3.zip › Figure 5-source data 3/F8871-2-CON-E11.5-F+ ff-40X-gLhx2-22-2-R-Image Export-51_AF488.jpg]

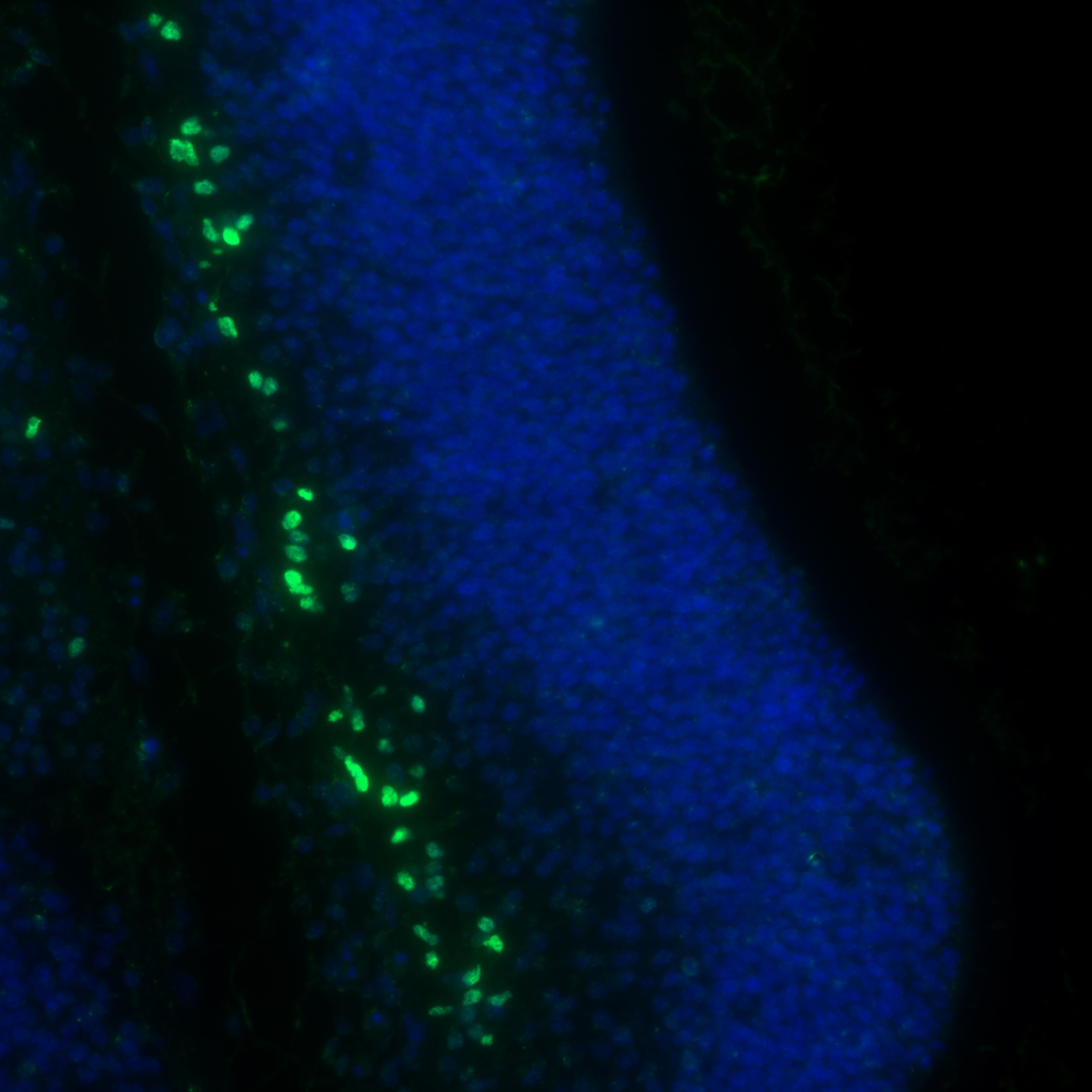

Supplement: Figure 5—source data 3. [file elife-86940-fig5-data3.zip › Figure 5-source data 3/F6091-5-CON-E13.5-FF f+-40X-Lhx5-30-4-R-MP-Image Export-20.jpg]

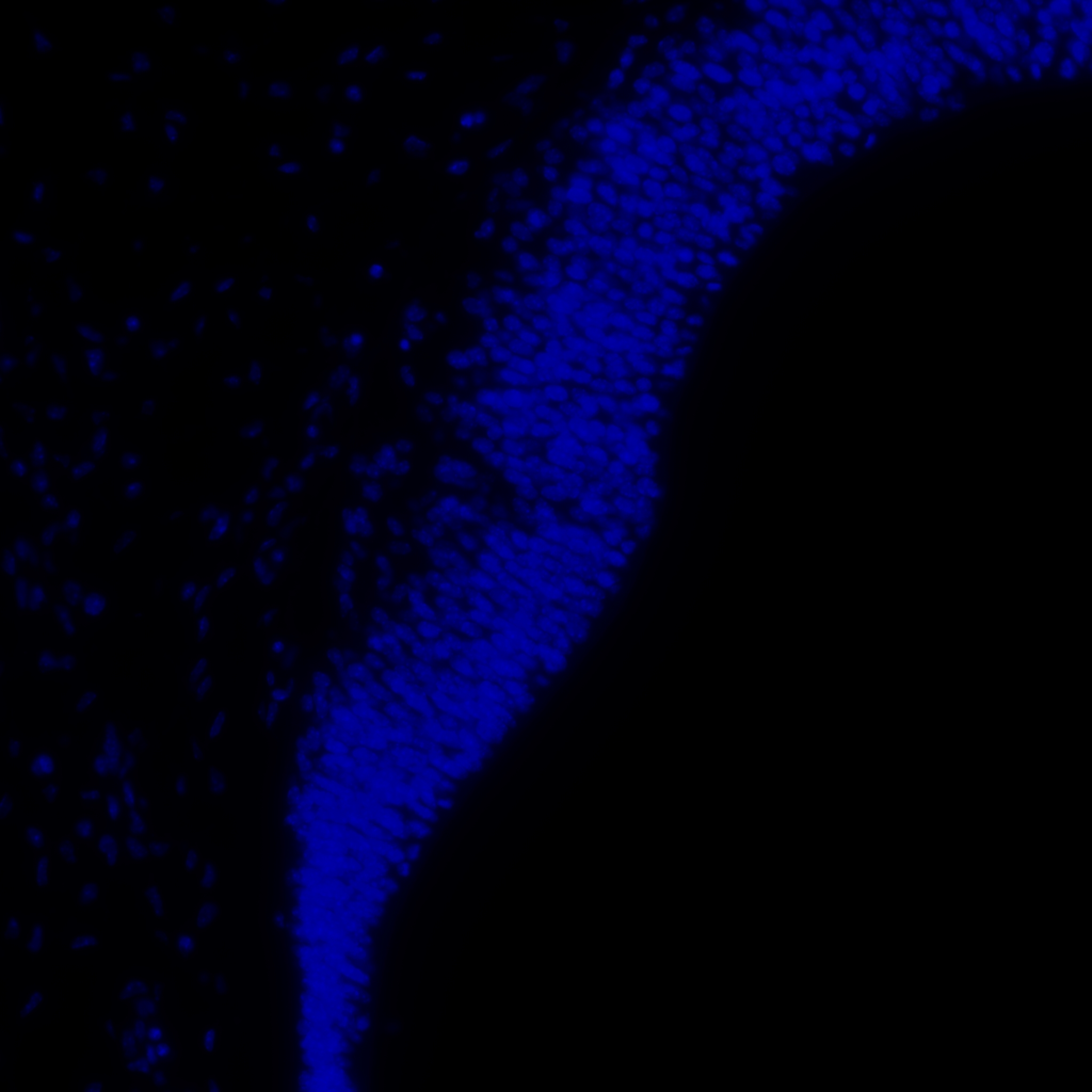

Supplement: Figure 5—source data 3. [file elife-86940-fig5-data3.zip › Figure 5-source data 3/F8871-2-CON-E11.5-F+ ff-40X-gLhx2-22-2-R-Image Export-51_DAPI.jpg]

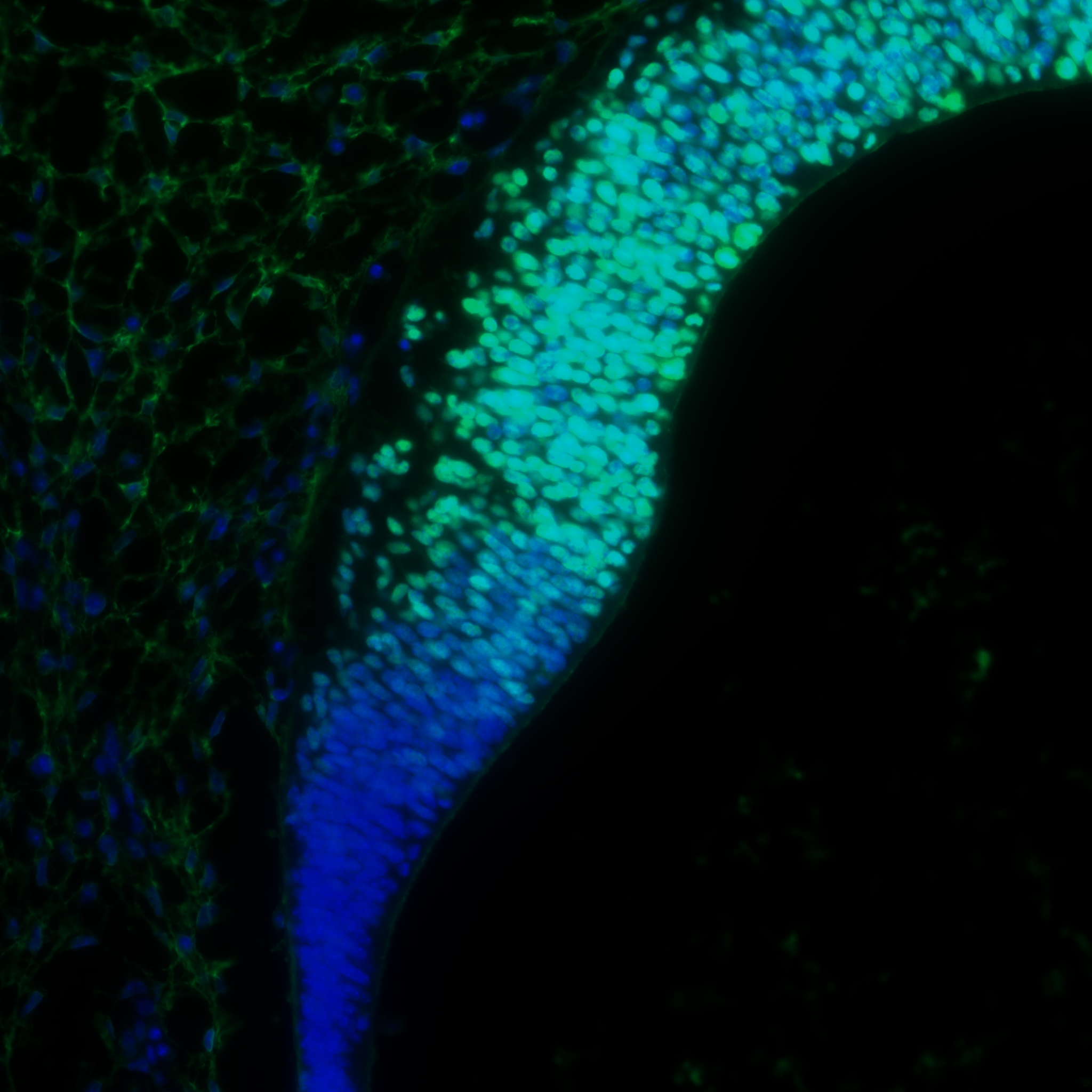

Supplement: Figure 5—source data 3. [file elife-86940-fig5-data3.zip › Figure 5-source data 3/F8871-2-CON-E11.5-F+ ff-40X-gLhx2-22-2-R-Image Export-51.jpg]

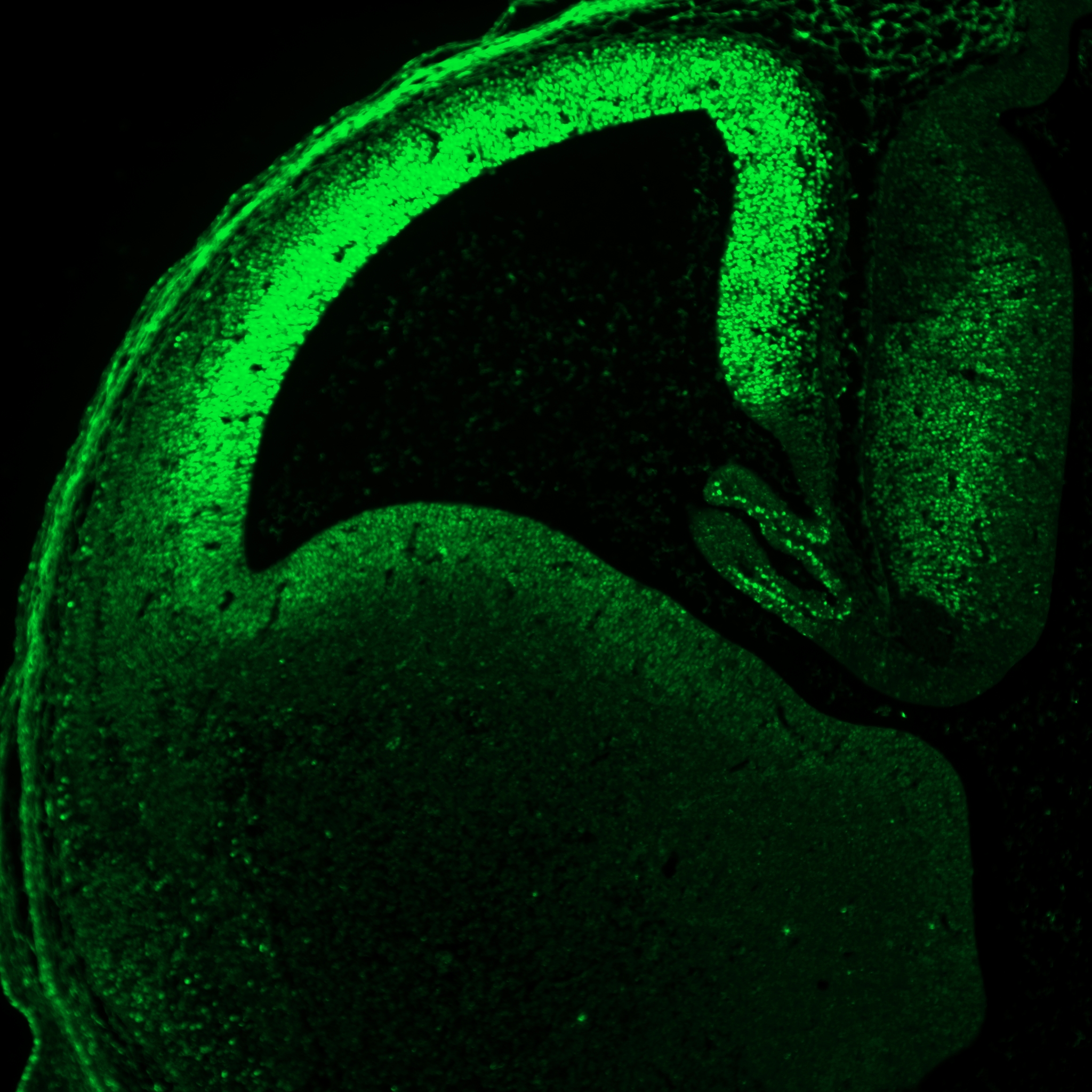

Supplement: Figure 5—source data 3. [file elife-86940-fig5-data3.zip › Figure 5-source data 3/F6091-8-DKO-E13.5-RX FF ff-10X-Lhx2-31-1-L-Image Export-22_AF488.jpg]

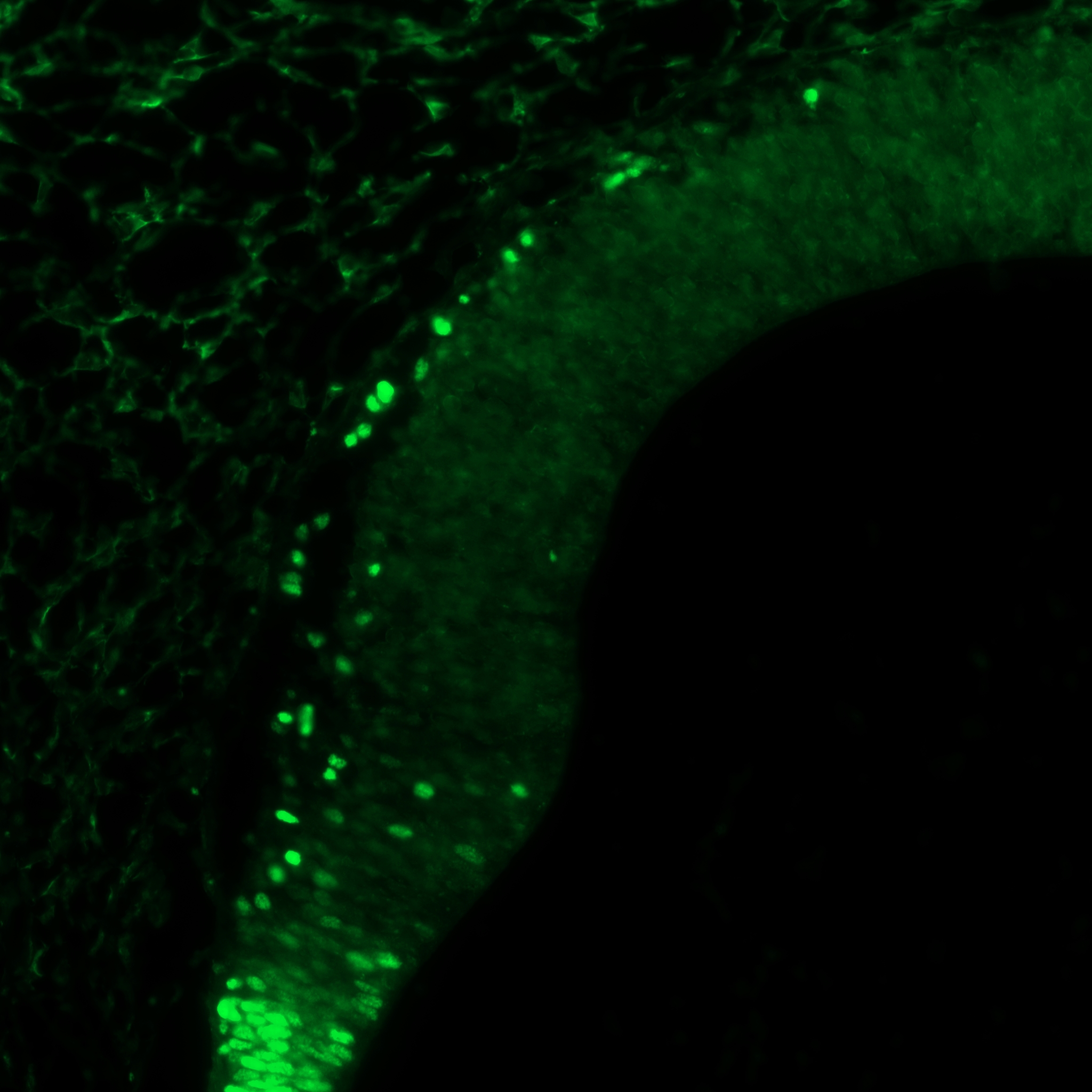

Supplement: Figure 5—source data 3. [file elife-86940-fig5-data3.zip › Figure 5-source data 3/F8871-2-CON-E11.5-F+ ff-40X-gLhx5-22-4-R-MP MIGRATION-Image Export-59_AF488.jpg]

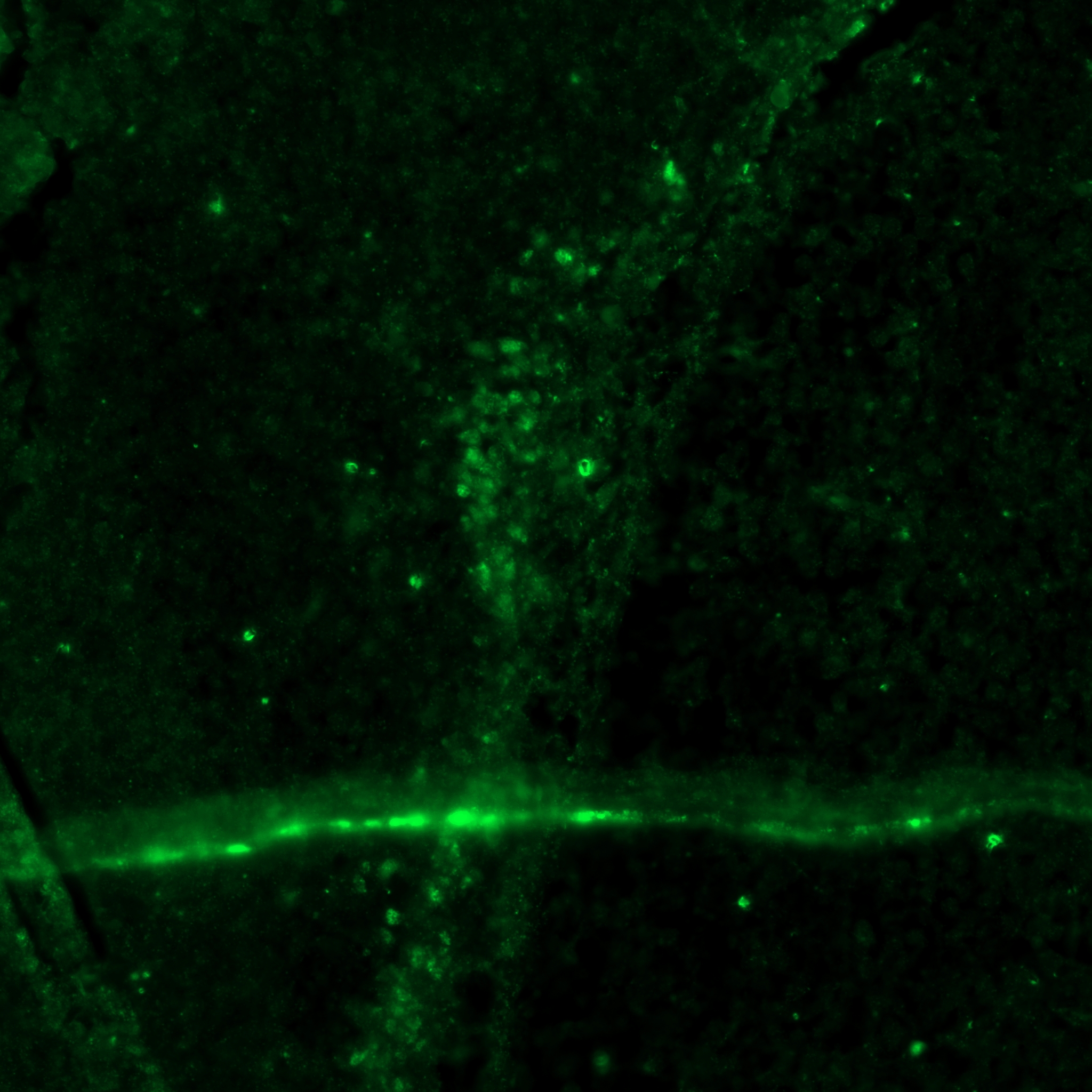

Supplement: Figure 5—source data 3. [file elife-86940-fig5-data3.zip › Figure 5-source data 3/F5734-5-CON-RX F+ f+-E14.5-40X-NEUROD1-17-1-L-Image Export-06_AF488.jpg]

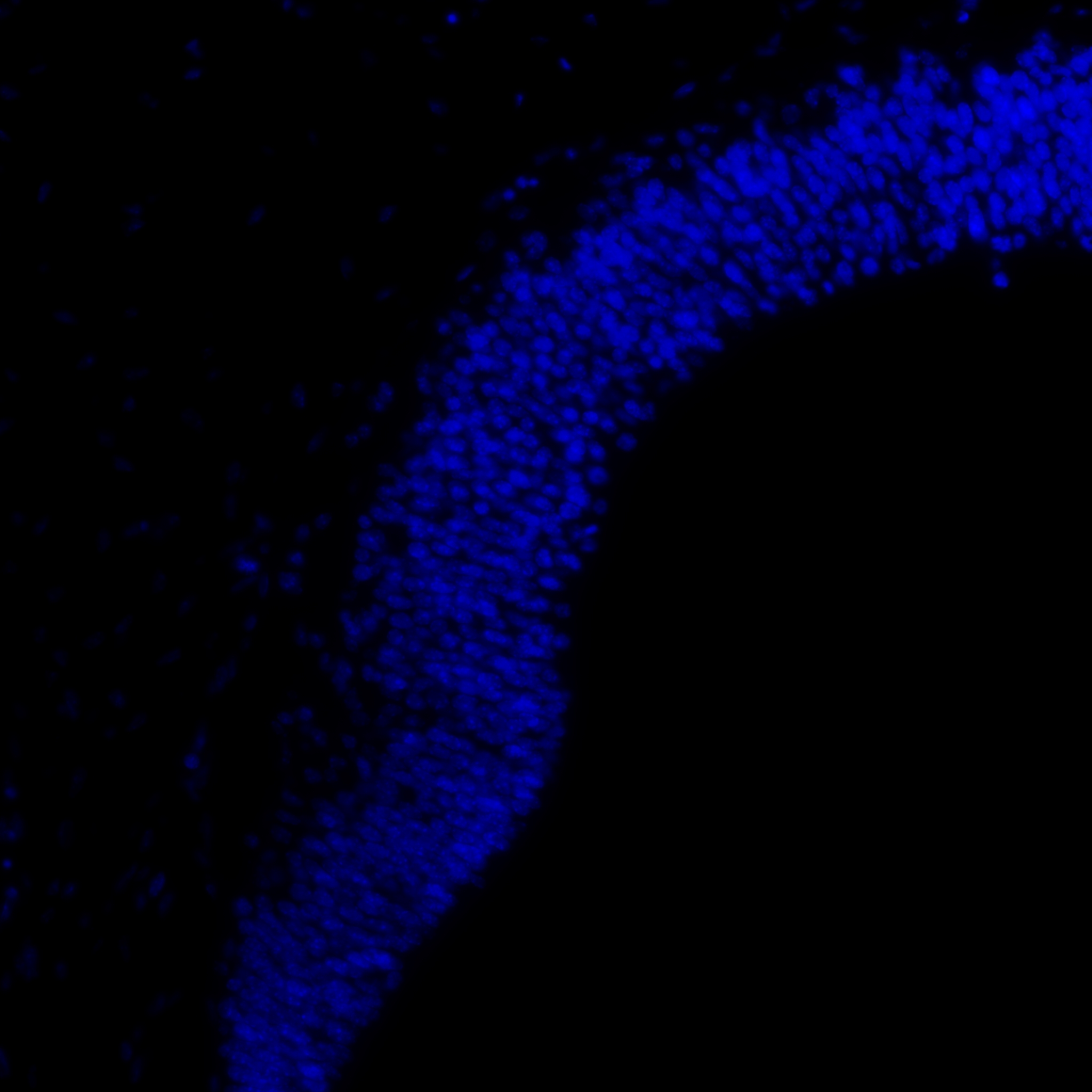

Supplement: Figure 5—source data 3. [file elife-86940-fig5-data3.zip › Figure 5-source data 3/F8871-2-CON-E11.5-F+ ff-40X-gLhx5-22-4-R-MP MIGRATION-Image Export-59_DAPI.jpg]

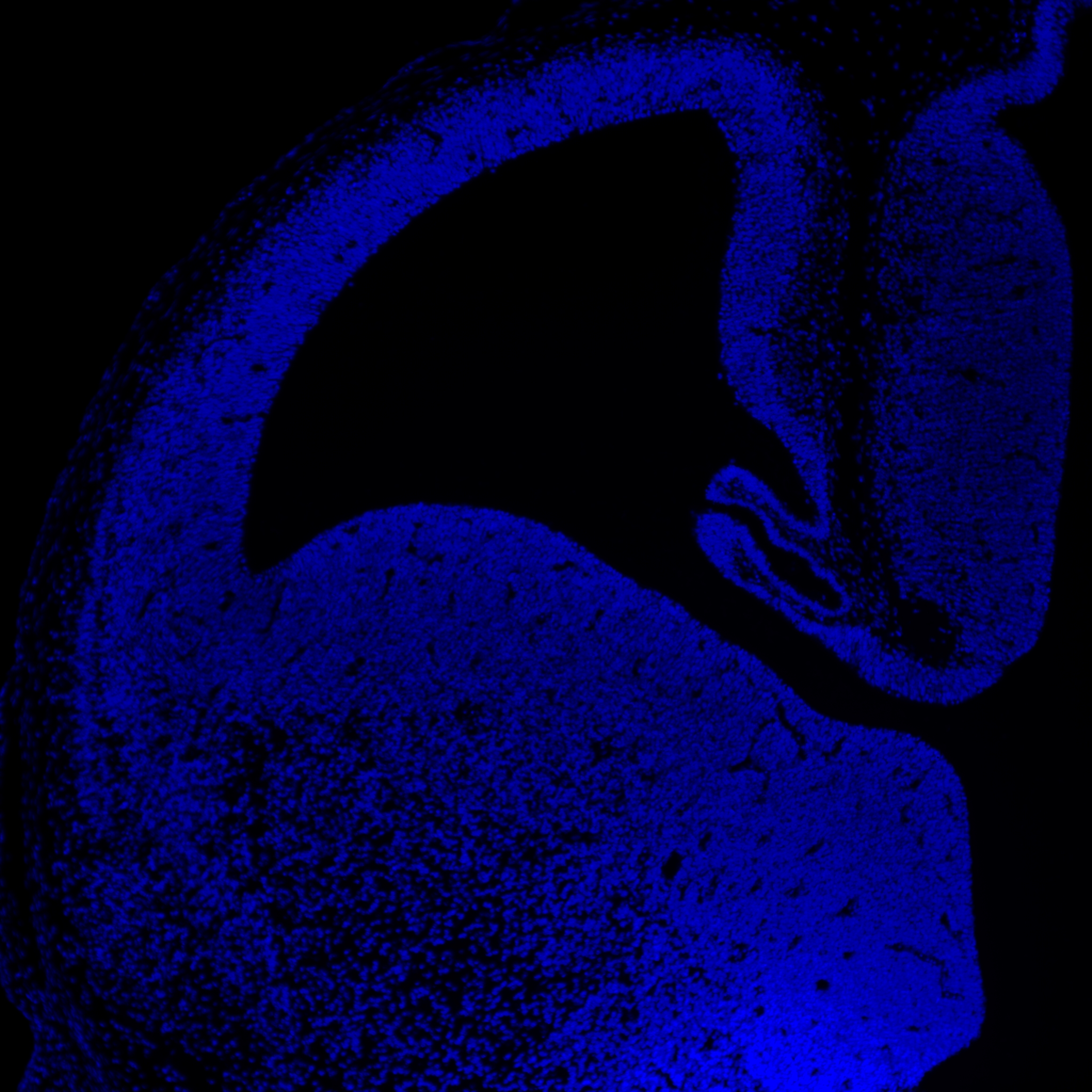

Supplement: Figure 5—source data 3. [file elife-86940-fig5-data3.zip › Figure 5-source data 3/F6091-8-DKO-E13.5-RX FF ff-10X-Lhx2-31-1-L-Image Export-22_DAPI.jpg]

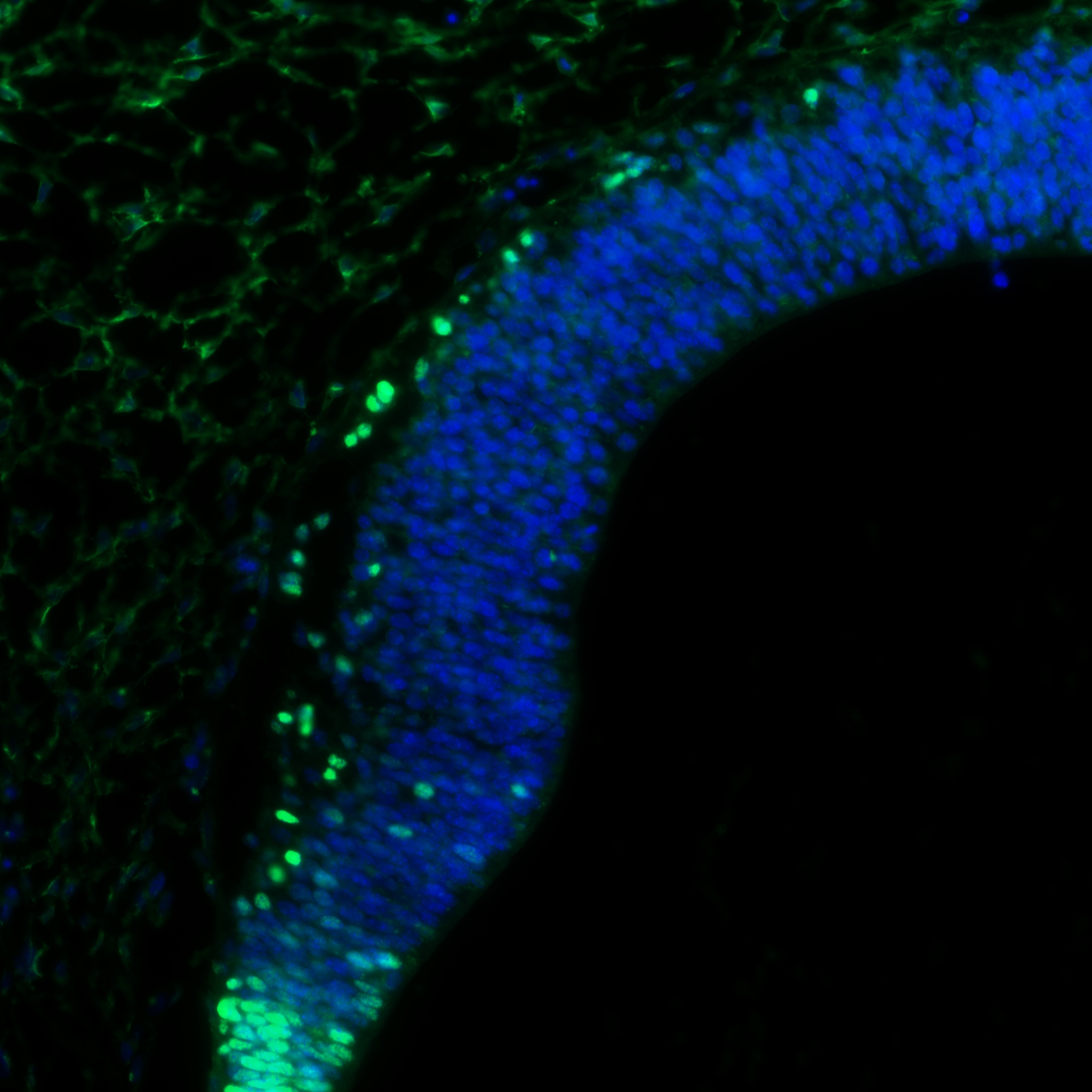

Supplement: Figure 5—source data 3. [file elife-86940-fig5-data3.zip › Figure 5-source data 3/F8871-2-CON-E11.5-F+ ff-40X-gLhx5-22-4-R-MP MIGRATION-Image Export-59.jpg]

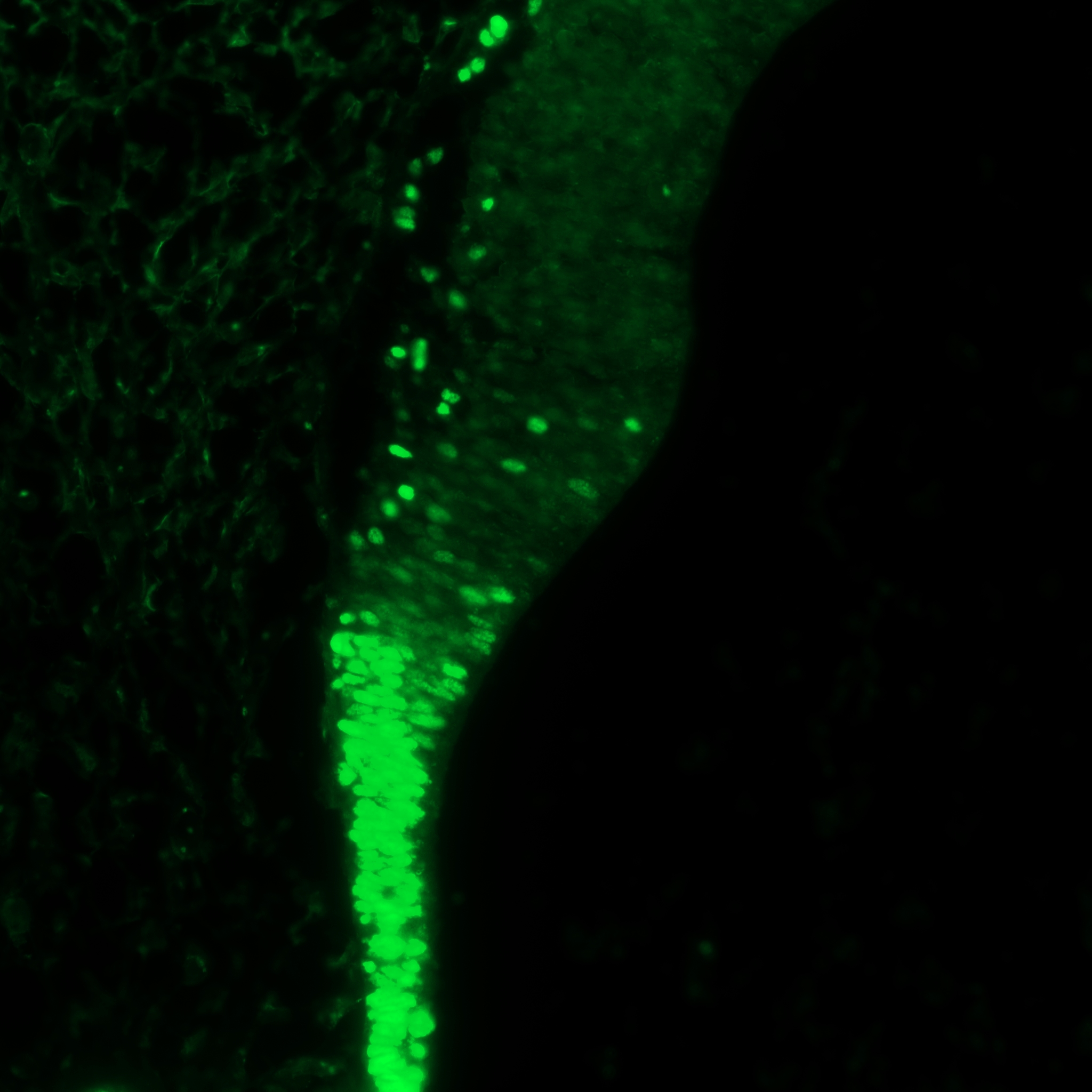

Supplement: Figure 5—source data 3. [file elife-86940-fig5-data3.zip › Figure 5-source data 3/F8871-2-CON-E11.5-F+ ff-40X-gLhx5-22-4-R-MP-Image Export-58_AF488.jpg]

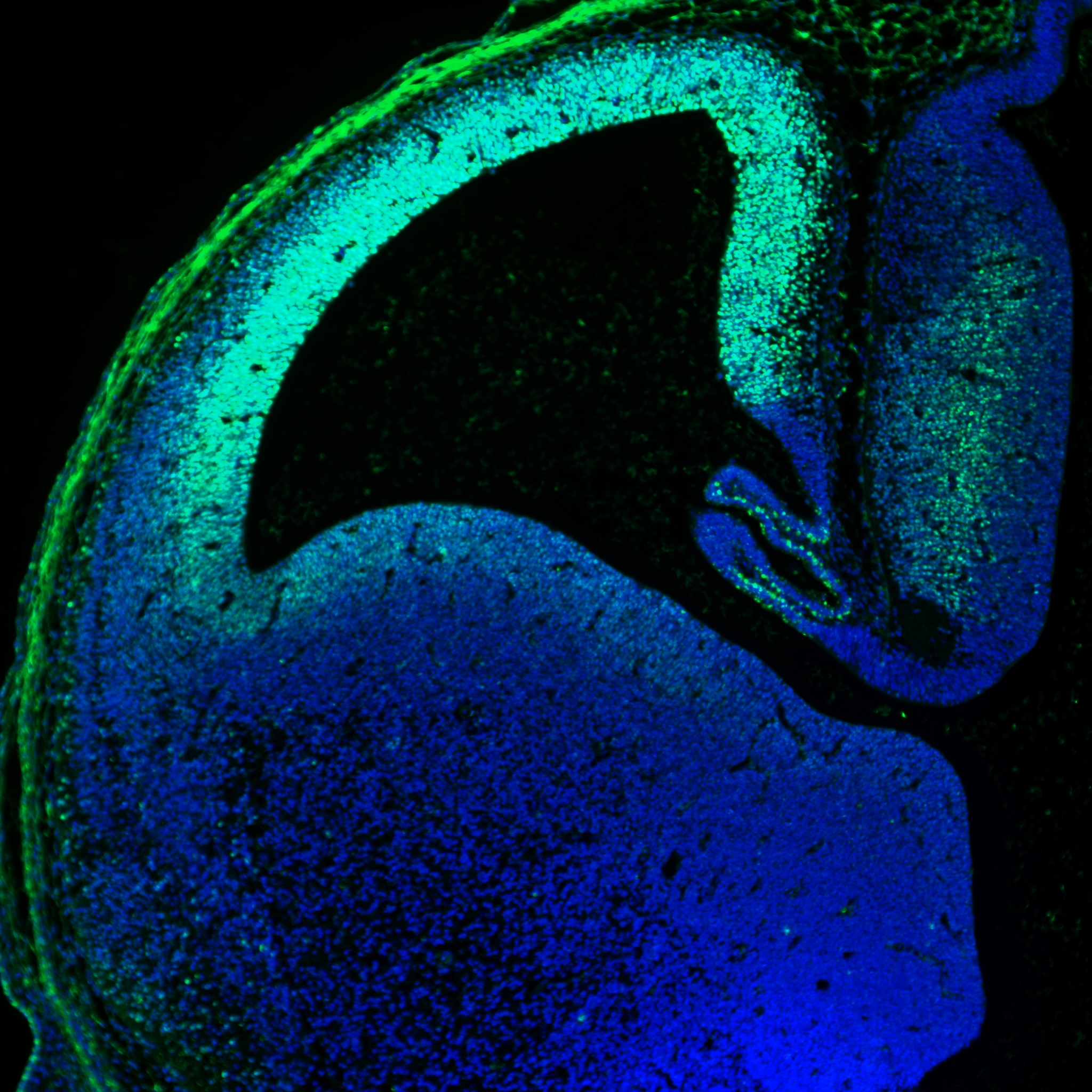

Supplement: Figure 5—source data 3. [file elife-86940-fig5-data3.zip › Figure 5-source data 3/F6091-8-DKO-E13.5-RX FF ff-10X-Lhx2-31-1-L-Image Export-22.jpg]

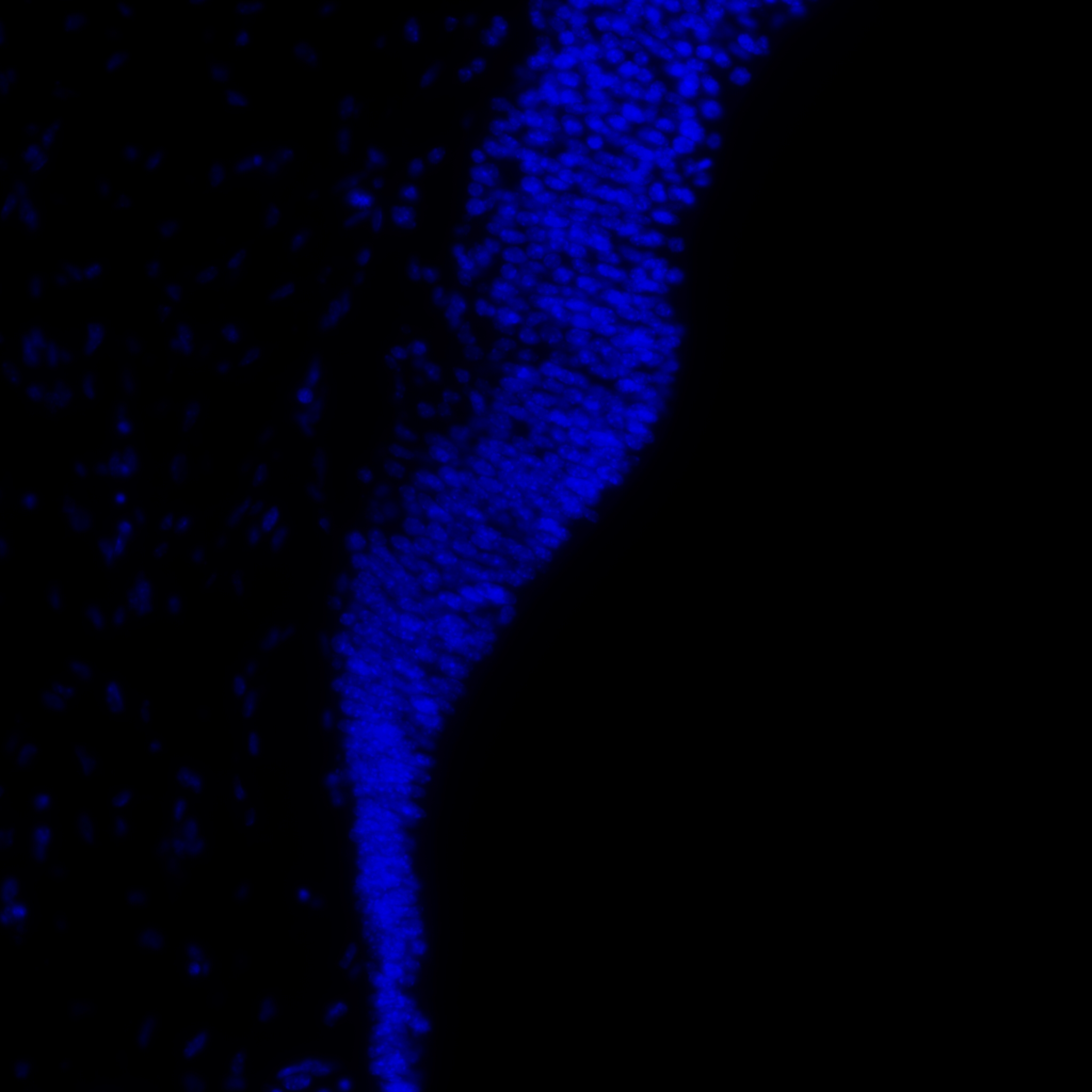

Supplement: Figure 5—source data 3. [file elife-86940-fig5-data3.zip › Figure 5-source data 3/F8871-2-CON-E11.5-F+ ff-40X-gLhx5-22-4-R-MP-Image Export-58_DAPI.jpg]

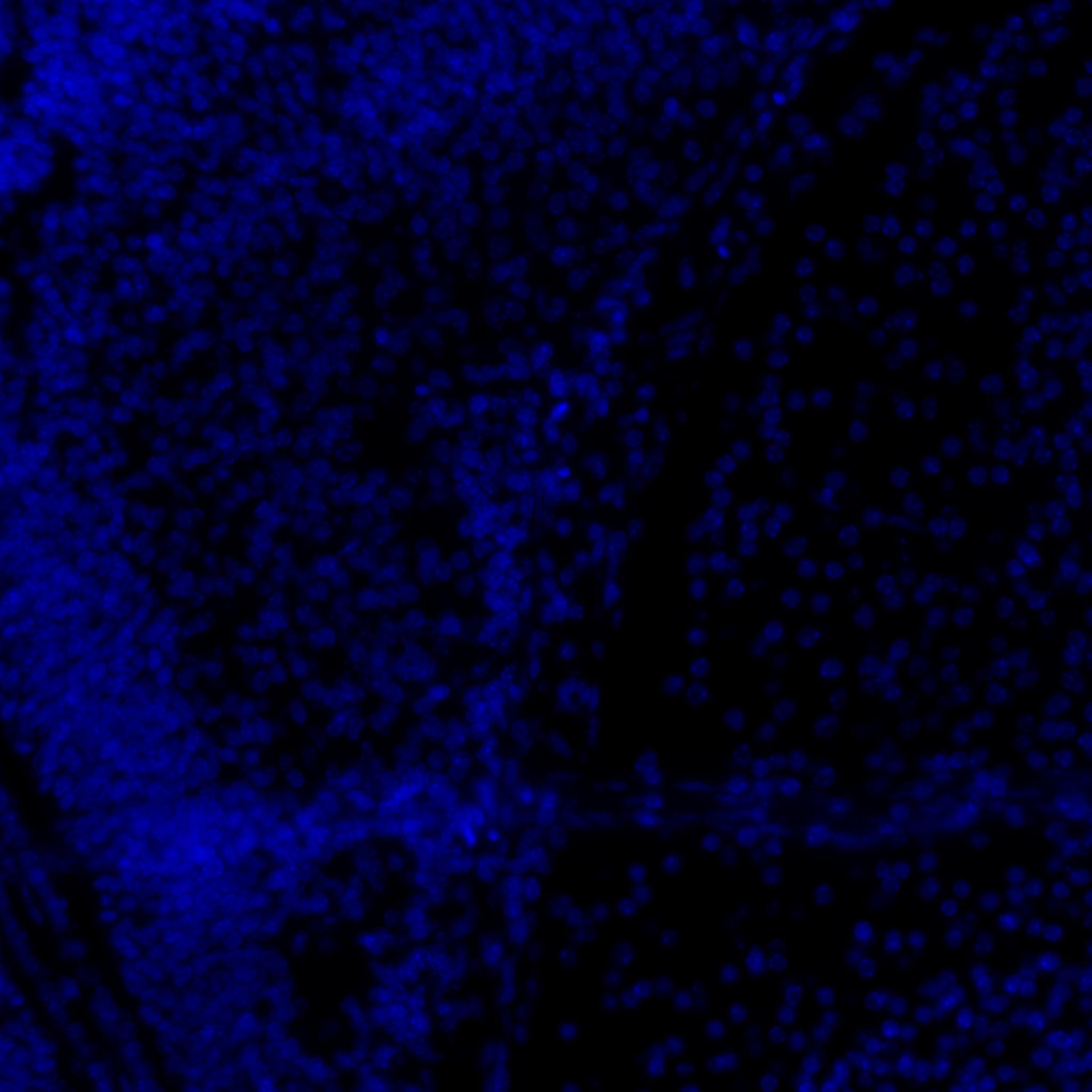

Supplement: Figure 5—source data 3. [file elife-86940-fig5-data3.zip › Figure 5-source data 3/F5734-5-CON-RX F+ f+-E14.5-40X-NEUROD1-17-1-L-Image Export-06_DAPI.jpg]

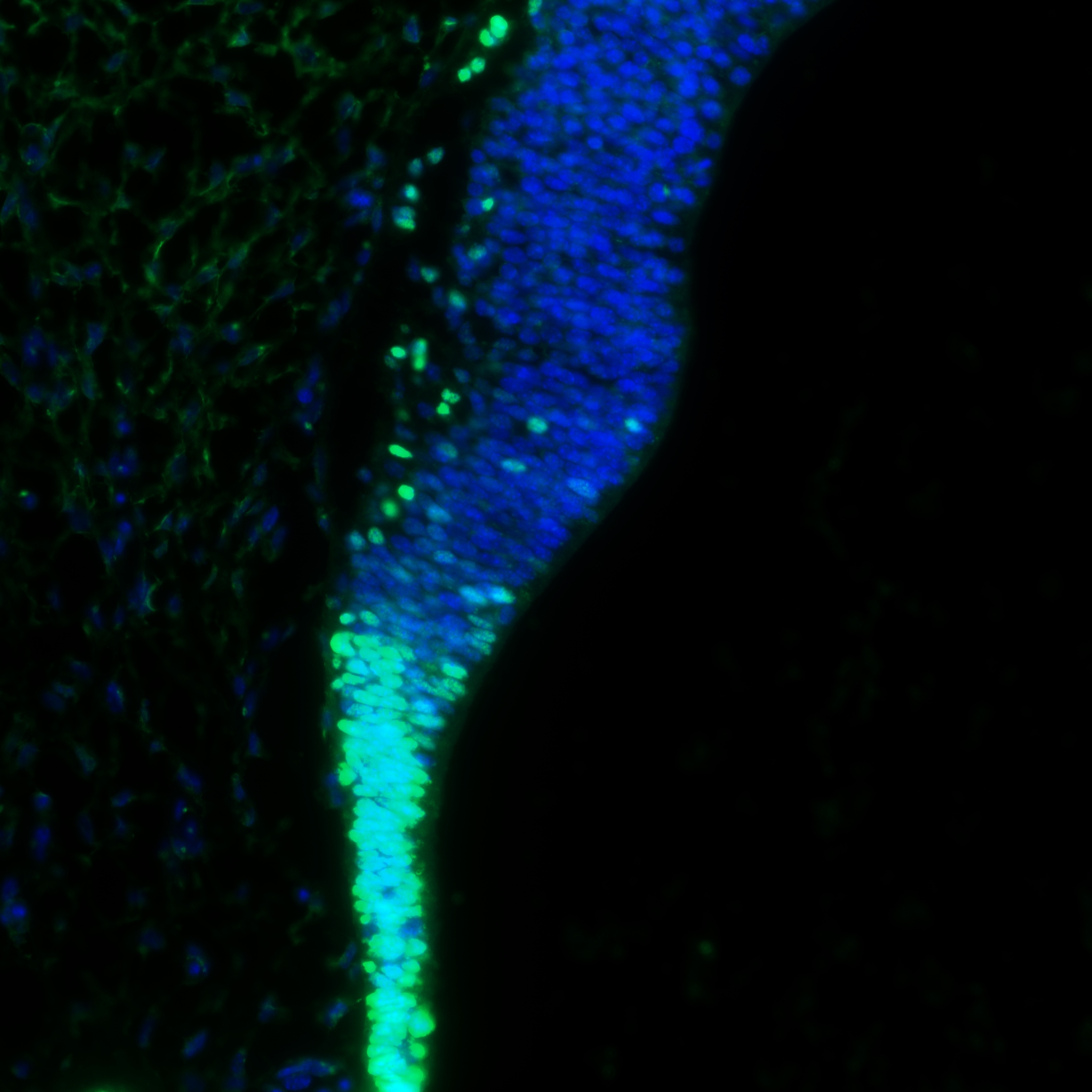

Supplement: Figure 5—source data 3. [file elife-86940-fig5-data3.zip › Figure 5-source data 3/F8871-2-CON-E11.5-F+ ff-40X-gLhx5-22-4-R-MP-Image Export-58.jpg]

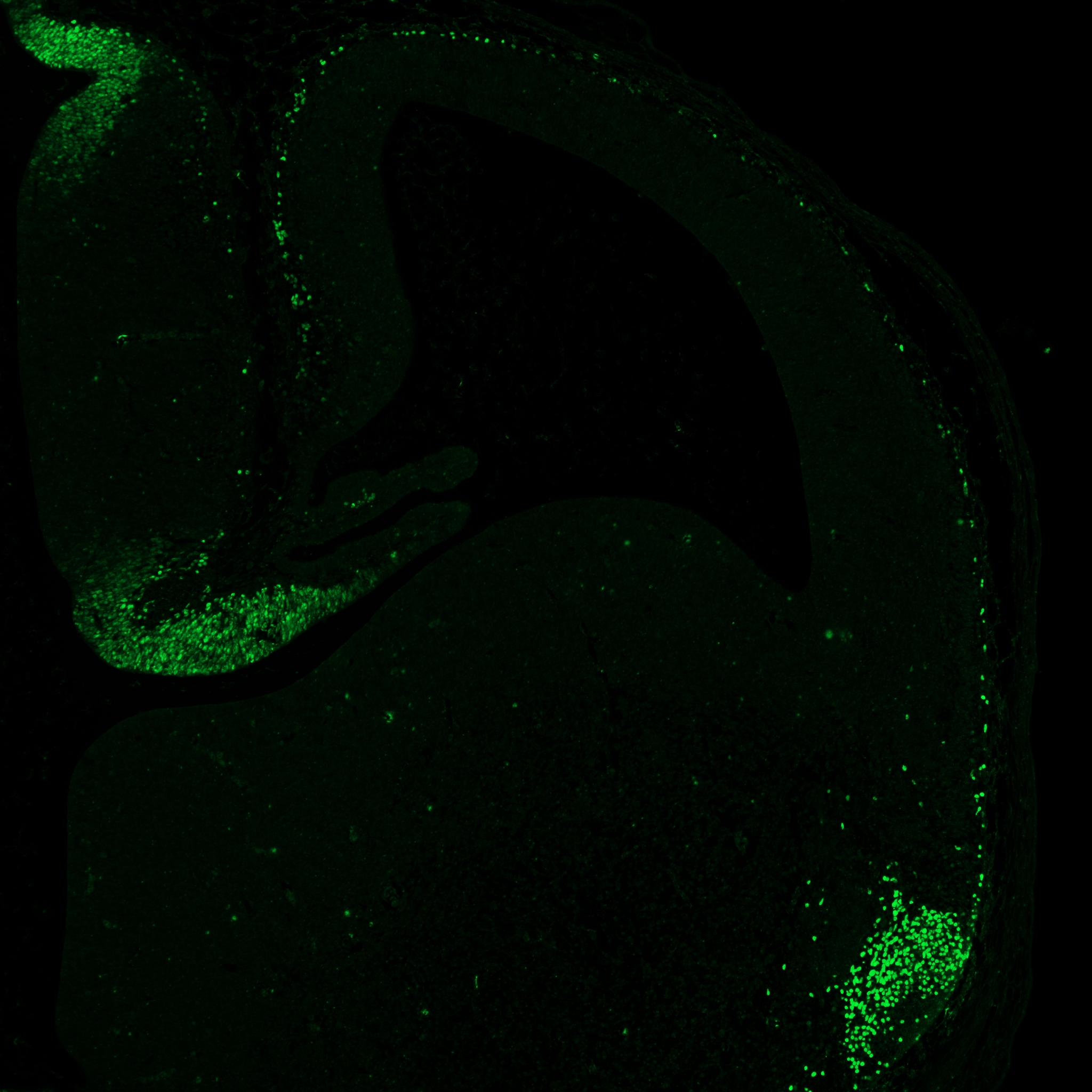

Supplement: Figure 5—source data 3. [file elife-86940-fig5-data3.zip › Figure 5-source data 3/F6091-8-DKO-E13.5-RX FF ff-10X-Lhx5-31-3-R-Image Export-35_AF488.jpg]

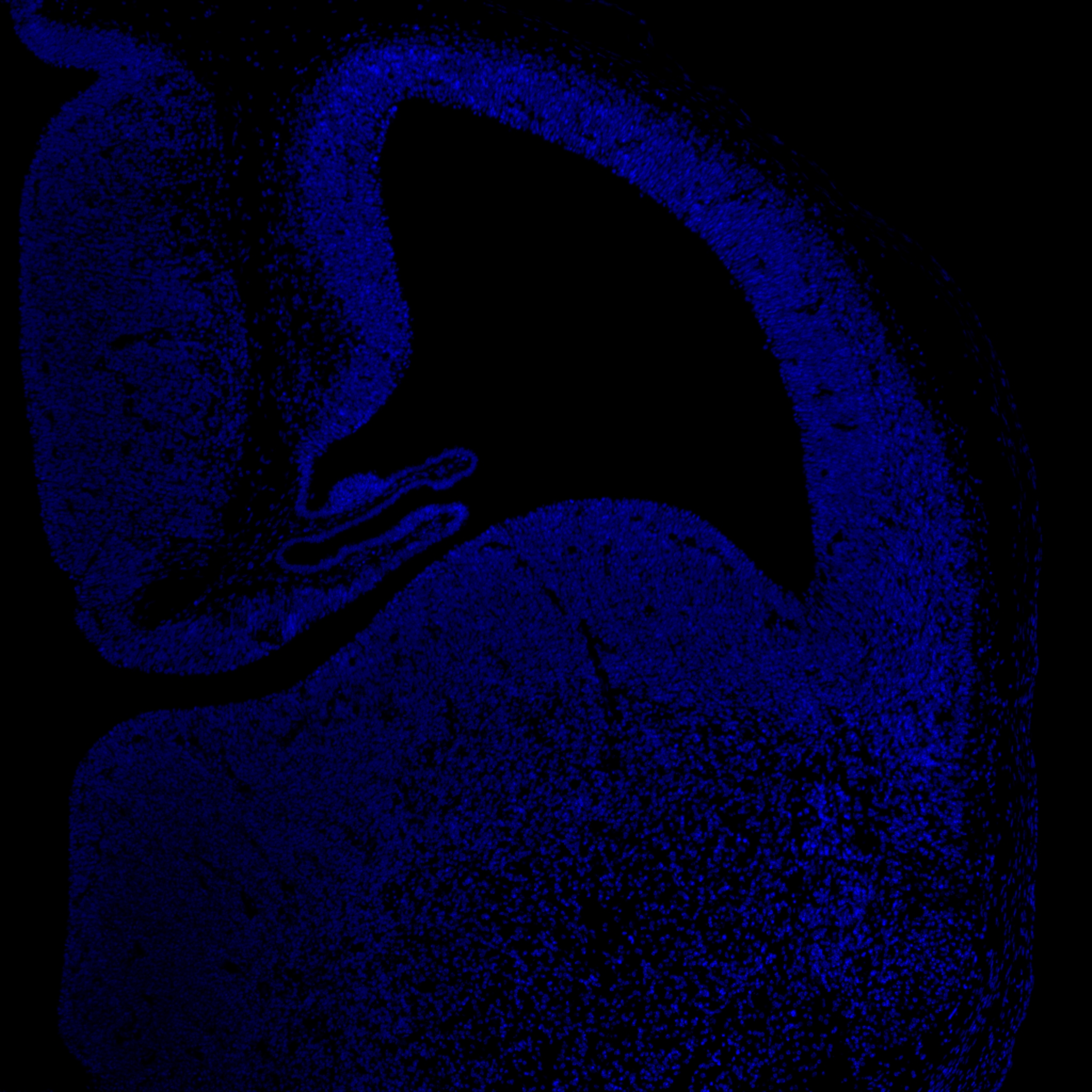

Supplement: Figure 5—source data 3. [file elife-86940-fig5-data3.zip › Figure 5-source data 3/F6091-8-DKO-E13.5-RX FF ff-10X-Lhx5-31-3-R-Image Export-35_DAPI.jpg]

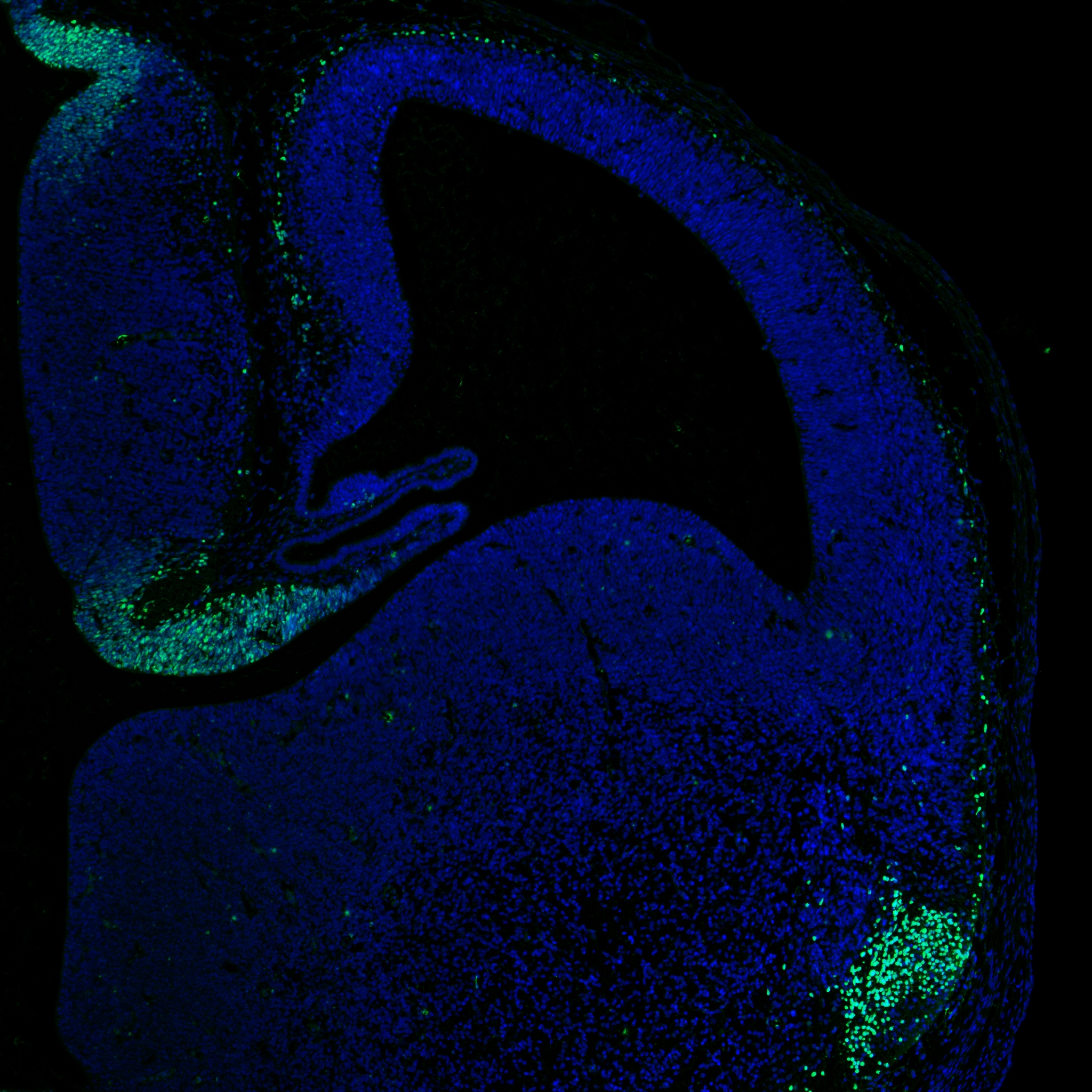

Supplement: Figure 5—source data 3. [file elife-86940-fig5-data3.zip › Figure 5-source data 3/F6091-8-DKO-E13.5-RX FF ff-10X-Lhx5-31-3-R-Image Export-35.jpg]

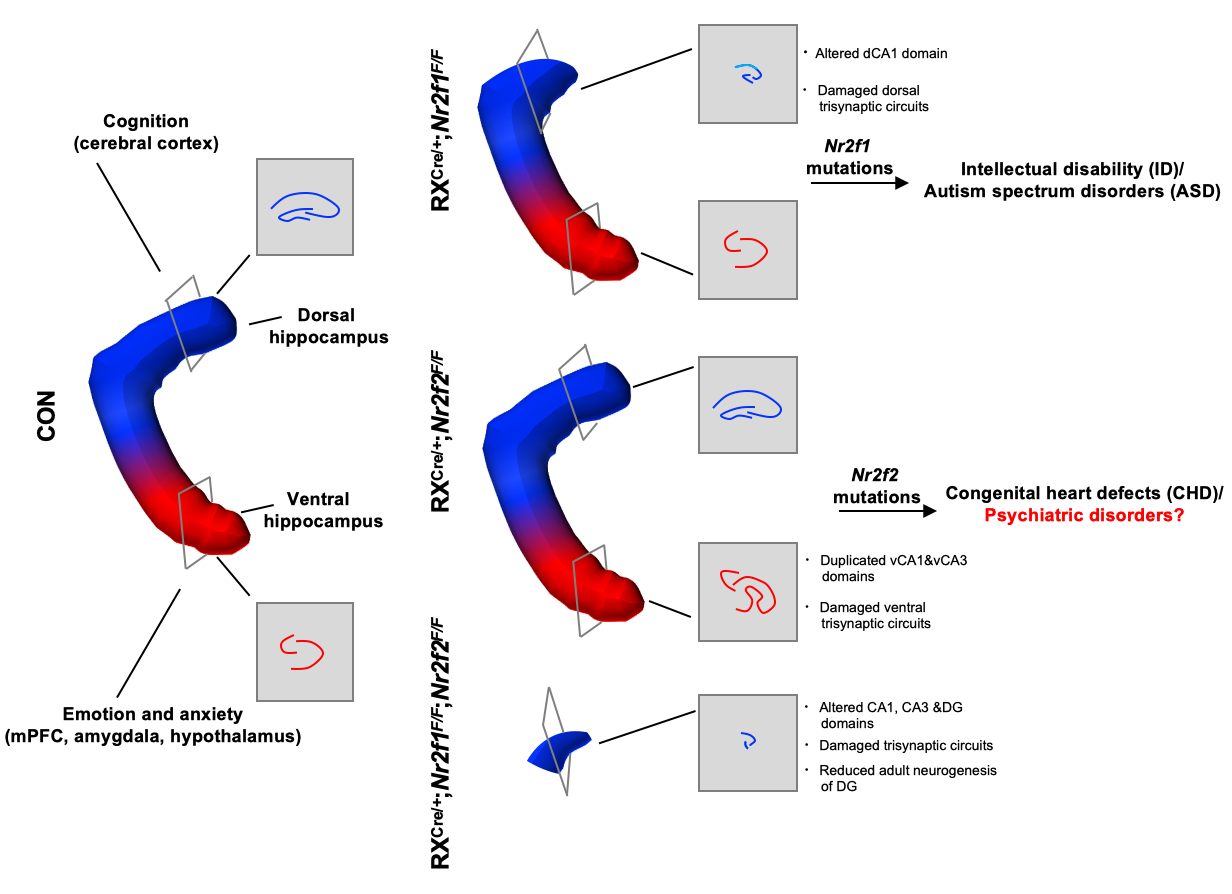

Supplement: Figure 5—figure supplement 1—source data 1. [file elife-86940-fig5-figsupp1-data1.zip › Figure 5-figure supplement 1-source data 1/Nr2f1 and Nr2f2 genes coordinate to control distinct characteristics of the hippocampus.png]
